# Proteomic Identification Reveals the Role of Ciliary Extracellular‐Like Vesicle in Cardiovascular Function

**DOI:** 10.1002/advs.201903140

**Published:** 2020-06-16

**Authors:** Ashraf M. Mohieldin, Rajasekharreddy Pala, Rinzhin T. Sherpa, Madhawi Alanazi, Ashwaq Alanazi, Kiumars Shamloo, Amir Ahsan, Wissam A. AbouAlaiwi, James J. Moresco, John R. Yates, Surya M. Nauli

**Affiliations:** ^1^ Department of Biomedical and Pharmaceutical Sciences Chapman University Irvine CA 92618 USA; ^2^ Department of Physics, Computer Science and Engineering Chapman University Orange CA 92866 USA; ^3^ Department of Pharmacology and Experimental Therapeutics University of Toledo Toledo OH 43614 USA; ^4^ Department of Molecular Medicine The Scripps Research Institute La Jolla CA 92037 USA; ^5^ Department of Medicine University of California Irvine Irvine CA 92868 USA

**Keywords:** aortic stenosis, arrythmia, cardiac edema, extracellular vesicles, fibrosis, hypotension, primary cilia

## Abstract

Primary cilia are shown to have membrane swelling, also known as ciliary bulbs. However, the role of these structures and their physiological relevance remains unknown. Here, it is reported that a ciliary bulb has extracellular vesicle (EV)‐like characteristics. The ciliary extracellular‐like vesicle (cELV) has a unique dynamic movement and can be released by mechanical fluid force. To better identify the cELV, differential multidimensional proteomic analyses are performed on the cELV. A database of 172 cELV proteins is generated, and all that examined are confirmed to be in the cELV. Repressing the expression of these proteins in vitro and in vivo inhibits cELV formation. In addition to the randomized heart looping, hydrocephalus, and cystic kidney in fish, compensated heart contractility is observed in both fish and mouse models. Specifically, low circulation of cELV results in hypotension with compensated heart function, left ventricular hypertrophy, cardiac fibrosis, and arrhythmogenic characteristics, which result in a high mortality rate in mice. Furthermore, the overall ejection fraction, stroke volume, and cardiac output are significantly decreased in mice lacking cELV. It is thus proposed that the cELV as a nanocompartment within a primary cilium plays an important role in cardiovascular functions.

## Graphical abstract

The ciliary extracellular‐like vesicle (cELV) acts as a nanocompartment within a cilium. The cELV increases the flexural rigidity of a cilium and can be released by mechanical fluid‐shear force. Proteomic identification of the cELV reveals its physiological roles in zebrafish and mouse models. In addition to the extracardiac phenotypes, the cELV is required for cardiovascular functions.

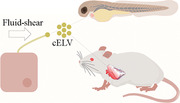

## 1 Introduction

Primary cilia exist on almost all mammalian cell types. Cilia have been hypothesized to act as mechanosensory and chemosensory organelles.^[^ ^1^, ^2^ ^]^ The ciliary membrane houses many receptors, ion channels, transporters, and other sensory proteins to support distinctive functions of cilia. The cilioplasm is also enriched with many signaling proteins. Any malfunctioning of ciliary signaling and protein transport to cilia, which are required to coordinate a key role in cilia assembly and function, can result in a wide range of diseases, termed ciliopathies. Since cilia are ubiquitous, ciliopathies include a wide spectrum of phenotypes as seen in Joubert syndrome,^[^ ^3^, ^4^, ^5^ ^]^ Bardet–Biedl syndrome,^[^ ^6^, ^7^ ^]^ polycystic kidney disease,^[^ ^8^, ^9^ ^]^ Meckel–Gruber syndrome,^[^ ^10^, ^11^ ^]^ and many others.

To better understand the roles of primary cilia, many studies have investigated the structure and the complexity of ciliary proteomes. To date, cilia proteome has revealed hundreds of crucial proteins relevant to cilia formation and ciliopathy.^[^ ^12^, ^13^, ^14^, ^15^ ^]^ However, these proteins have not been studied within the subdomain of ciliary membrane. Structurally, a primary cilium has a protruding vesicle‐like structure along the cilia, commonly referred to ciliary bulb. First observed in 1977,^[^ ^16^ ^]^ ciliary bulb has since been proposed to be an artifact resulting from changes in osmotic pressure during fixation.^[^ ^17^, ^18^, ^19^ ^]^

Many interesting studies have also emerged associating the shedding of ciliary proteins in membrane‐bound vesicles.^[^ ^20^, ^21^, ^22^ ^]^ While the release of these ciliary proteins regulates ciliary signaling for normal male mating behaviors in *Caenorhabditis elegans*,^[^ ^23^ ^]^ it also occurs when cells underwent flagellar resorption.^[^ ^24^ ^]^ It is recently proposed that ciliary membrane is also shredded in pathological contexts when a protein complex known as BBSome fails to retrieve from cilium to cell body.^[^ ^25^ ^]^ The complexity of the ciliary membrane shredding is further intrigued by a study showing that the release of ciliary membrane can occur freely as a normal process to regulate cilia length.^[^ ^26^ ^]^ Importantly, the impact of this shredding on the cilia function and ciliopathy is still unknown. Further, the proteome within this shredding is largely unidentified. At least in *C. elegans*, glutamylases and deglutamylases are required for ciliary shredding.^[^ ^27^, ^28^ ^]^ While ciliary glutamination may control the shredding in *C. elegans*, the initial environmental signals or the mechanisms that regulate activities of glutamination enzymes within a cilium are yet to be studied in the mammalian system.

Although ciliary membrane shredding has never been implicated in cardiovascular function, many extracellular membrane vesicles from the cell body have been thought to function as intracardiac communication for horizontal transfer of information within cardiovascular cells.^[^ ^29^ ^]^ Importantly, vascular endothelia and cardiomyocytes secrete microvesicles,^[^ ^30^, ^31^ ^]^ which have been proposed to indicate cardiovascular functions.^[^ ^32^ ^]^ The communication functions of these microvesicles have been eluded to play a role in angiogenesis, cardiac remodeling, and fibrosis with cardioprotective and atherosclerotic‐protective properties.^[^ ^30^, ^31^, ^33^ ^]^ For examples, high levels of circulating microvesicles with procoagulant potential are found in patients with acute coronary syndromes.^[^ ^34^ ^]^ While there is no clear consensus on different microenvironment that dictates communication via microvesicles versus autocrine, paracrine or endocrine routes, it is believed that microvesicles provide a better regulated and more protected cell–cell communication on complex different microenvironmental signals for cellular uptake within the cardiovascular system.

Although ciliary function or signaling has been implicated in human ciliopathies, not much has been studied with regards to microstructure of ciliary shaft. Further, to observe and study these ciliary vesicles in real time is very challenging if not impossible in monolayer setup. Most laboratories depend on the fluorescence imaging to see the shredding of primary cilia. Using a single‐cell–single‐cilium imaging technique allowed us to examine cilia structure without the need of fluorescence. We identified that the extracellular vesicle (EV) released from nonciliated cells is functionally different from ciliary extracellular‐like vesicle (cELV). We used unbiased differential proteomic approach to characterize the physiological relevance of this cELV. Our study showed that cELV is a specific microdomain of the primary cilium, and it may play an important physiological role in cardiovascular functions.

## 2 Results

### 2.1 The cELV Is a Subdomain of Ciliary Membrane and Mechanically Regulated by Fluid Shear Stress

Examining a single cell from the side, we studied the protruding structure (cELV) within the primary cilia (Figure S1a–c, Supporting Information). Because cilia are mechanosensory organelles,^[^ ^35^ ^]^ we used a low shear force (0.3 dyn cm^−2^) that would not significantly bend the cilium and a high shear force (1 dyn cm^−2^) that would bend the cilium over 45°. The perimeter and circularity of the cELV were greatly altered during cilium bending by high shear stress, suggesting a potential role for mechanical shear force on the cELV. The cELV moved up and down along the cilium during static conditions, but it failed to reach to the tip of the cilium (Movie S1, Supporting Information). In some cases, multiple cELV was clearly distinguished at the tip of the cilium during bending, due to shear flow (Movie S2, Supporting Information). Based on our mathematical modeling, the flexural rigidity of cilia was double in the present than in the absent of cELV (Supplement S1, Supporting Information), and the mass of the cELV directly correlated with the mass of primary cilia (Supplement S2, Supporting Information).

Because our initial studies indicated a potential role for mechanical fluid force on the cELV, we hypothesized that the cELV could be released from the cilia by fluid‐shear stress. First, we examined if the cELV had similar characteristics to the already known extracellular vesicle (EV). Like EV,^[^ ^36^, ^37^, ^38^ ^]^ the cELV formation could be inhibited by ceramide‐mediated vesicle blockers (Figure S1d–g, Supporting Information). These inhibitors did not affect cilia formation or cell division. In all our studies, cilia length was examined in three dimensions, utilizing the single‐cell–single‐cilium technique (Movie S3, Supporting Information). We also found that the cELV formation was depended on kinesin but not dynein.

**Figure 1:**
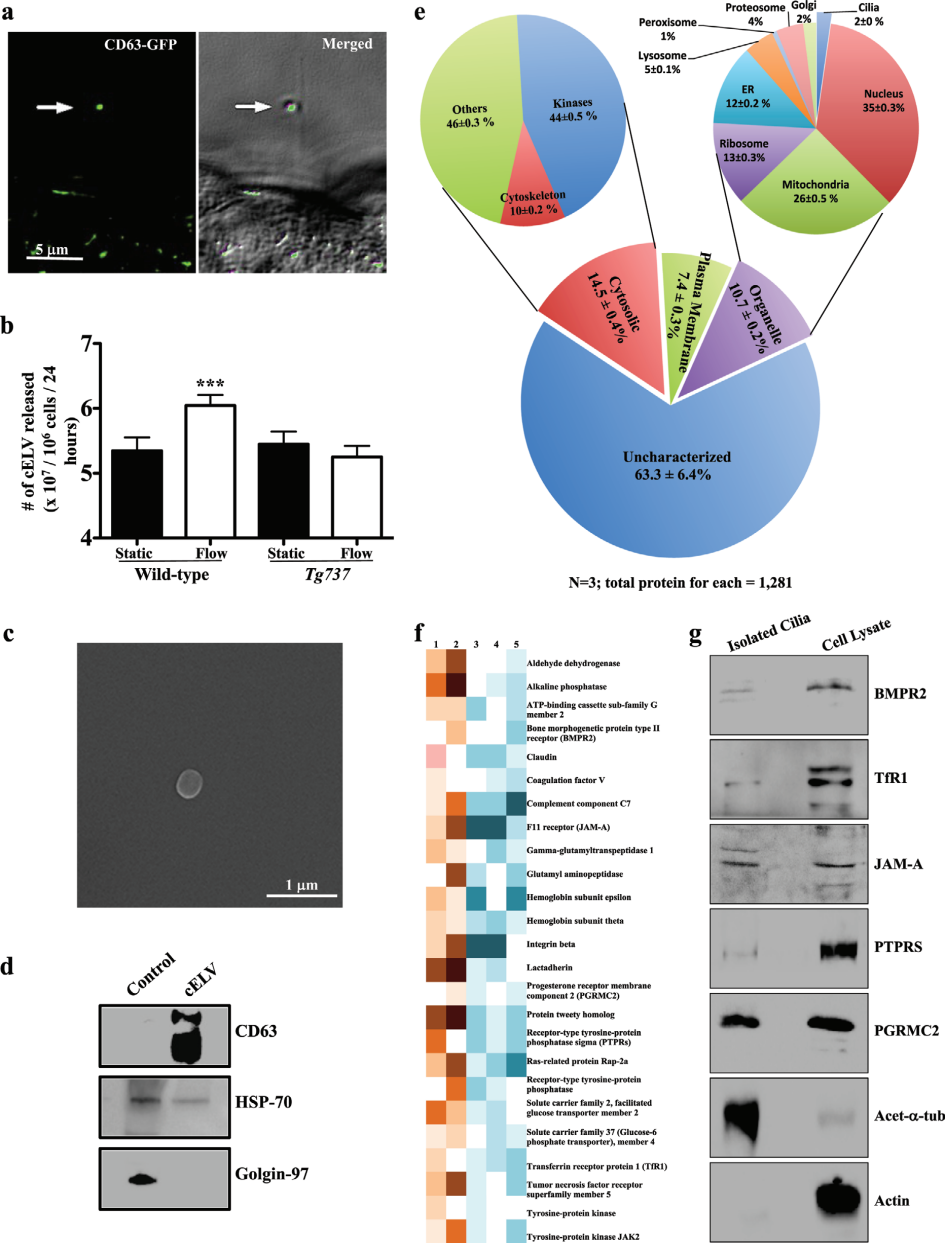
Proteomic identification of cELV proteins. a) CD63‐GFP was localized in the cELV (arrow; Movie S4, Supporting Information). The cELV can be seen in the differential interference contrast (DIC) image. CD63‐GFP localization to cELV at different time points is shown in Figure S2c (Supporting Information). b) Fluid shear stress (1 dyn cm^−2^ for 30 min) induced the release of cELV in cells with cilia (wild‐type) but not in those without cilia (*Tg737*). The release of the cELV from a cilium can be seen in Movie S4 (Supporting Information). c) After fluid flow, CD63‐GFP vesicles were isolated from the eluent. A scanning electron microscopy (SEM) image of a single CD63‐GFP vesicle is shown. Isolation and purification are shown in Figure S2 (Supporting Information). d) Purity of CD63‐GFP vesicles was further verified by immunoblotting for CD63 and HSP70 (positive controls) and Golgin‐97 (negative control). e) Based on the micro MudPIT separation, a comparative proteomic analysis between isolated primary cilia (with induced cELV appearance) and whole cell lysates, indicated that 2% of all organellar proteins were localized within the cilia (and cELV). About 18.5% of all these cELV proteins have been previously confirmed to be in the cilia (Table S2, Supporting Information). Proteomic analysis using a standard MudPIT separation was also performed (Figure S8f, Supporting Information). f) A representative heat‐map image shows the newly identified ciliary proteins that consistently appeared in all MudPIT samples. The relative abundance of each protein is indicated in orange (high abundance; samples 1 and 2) and blue (low abundance; samples 3, 4, and 5). g) A western blot analysis confirmed the expression of all five selected ciliary proteins. Acetylated *α*‐tubulin and actin were used as controls to indicate the purity of isolated cilia. *N* = 3 in each group. ***, *p* < 0.001.

Second, we examined the cELV formation with the EV marker CD63‐GFP^[^ ^39^ ^]^ (Figure S2a–c, Supporting Information). CD63‐GFP was expressed throughout the cell body and cilia in the first 24 h post‐transfection and specifically in the cELV at 72 h post‐transfection (**Figure** **1**a; Movie S4, Supporting Information). This further solidified the idea that cELV and EV shared similar characteristics.

Third, we examined the release of cELV in the absence and presence of fluid flow in ciliated wild‐type and nonciliated *Tg737* cells. Our results indicated that while cilia were not required to release basal level of vesicles, fluid flow might induce the release of cELV from primary cilia (Figure 1b). To confirm that cELVs were released from cilia, we performed several experiments in the absence and presence of fluid flow (Movie S4, Supporting Information). In all cases, fluid‐shear stress caused the release of cELV.

Forth, we examined the structure and function of cELV. After carefully standardized the cELV containing CD63‐GFP (Figure S1d–g, Supporting Information), we isolated and examined them with scanning electron microscopy (Figure 1c). The cELV had a round shape with a diameter of 237 ± 7 nm. We also verified the purity of the isolation method by immunoblotting for CD63 and HSP70 (positive controls) and Golgin‐97 (negative control) (Figure 1d), confirming the purity of the cELV isolation.^[^ ^40^, ^41^ ^]^ Using the same isolation method, we showed that exogenous cELV could trigger cellular signaling in the cilioplasm and cytoplasm (Figures S3–S7 and Movies S5–S7, Supporting Information).

### 2.2 A MudPIT Proteomic Analysis Reveals Novel cELV Proteins

To further analyze the functions of cELV in cell signaling, we used a comprehensive and unbiased strategy that included proteomic studies followed by in vivo validation. To evaluate the protein composition of cELV, we first isolated primary cilia containing ELV to distinguish cELV from other EV because the cell body releases EV into the extracellular media.^[^ ^25^, ^38^ ^]^ The cELV formation can be induced by mechanical fluid force;^[^ ^42^ ^]^ therefore, we isolated the primary cilia containing cELV by mechanical fluid force (see Methods and Movie S8, Supporting Information). Isolated cilia were thoroughly validated in multiple independent preparations (Figure S8, Supporting Information).

Second, the isolated cilia and whole‐cell lysates were then subjected to proteomic analysis using a sensitive multidimensional protein identification technology (MudPIT) technique.^[^ ^43^ ^]^ A standard MudPIT analysis (Figure S8, Supporting Information) and a more stringent MudPIT microanalysis (Figure 1e) were used and compared our proteomic data in order to increase our experimental rigor. Both MudPIT analyses consistently indicated that 2% of the total organellar protein fragments were localized in the cilia (Table S1, Supporting Information). However, the entire proteome was potential candidates of cELV‐derived proteins, including the organellar proteins that were previously known as ciliary proteins.

Third, we set our inclusion criterion based on the protein spectral count that depicted close‐to‐zero protein fragments in the whole cells but showed an abundance in the isolated cilia. We also included those protein fragments that were present at a two‐fold or higher ratio in cilia lysates than cell lysates (cilia‐to‐cell ratio ≥ 2). These criteria decreased the total number of potential candidates of cELV proteins to 211 (Table S2, Supporting Information); 172 of these proteins had never been reported in primary cilia. Of the 172 proteins, there were 25 proteins that consistently appeared in spectral counts of cilia lysates (Figure 1f). Further, 6.4% of the proteins were known to be EV biomarker proteins.

Forth, to validate the proteomic studies, we selected five membrane proteins (BMPR2, TfR1, JAM‐A, PTPRS, and PGRMC2) from these 25 proteins. Our selection was based on the fact that these proteins are membrane proteins, which may have a significant function in chemosensory functions of cilia. To examine the functions of these proteins, the expression of BMPR2, TfR1, JAM‐A, PTPRS, and PGRMC2 in isolated primary cilia were confirmed (Figure 1g).

### 2.3 Newly Identified Proteins Are Required for cELV Formation and Cellular Functions

**Figure 2:**
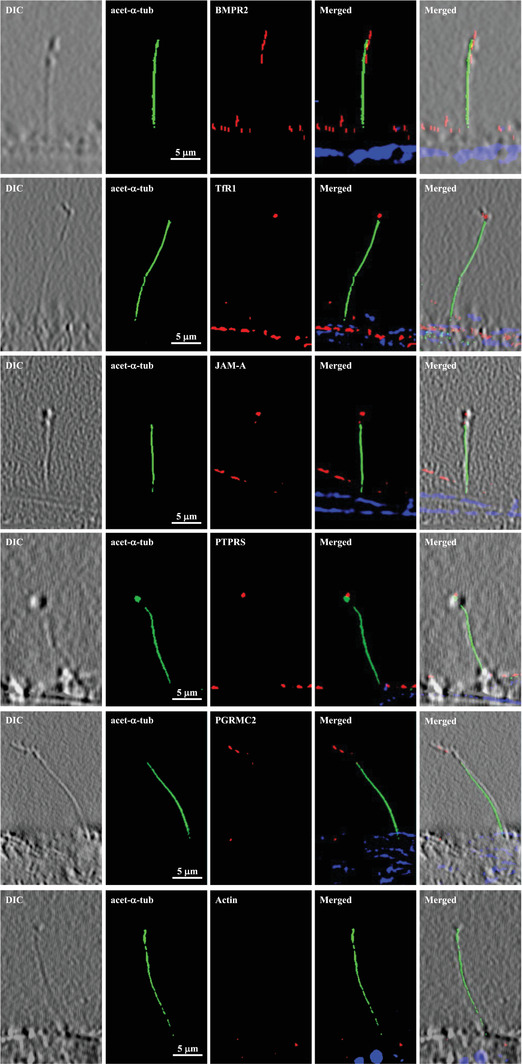
Selected cELV proteins are expressed in subdomain of primary cilia. Selected proteins (red) were localized to cELV. Acetylated *α*‐tubulin (green) and DAPI (blue) were used as ciliary and nuclear markers. High‐resolution differential interference contrast (DIC) images confirmed the presence of cilia. Actin was used as a negative control. Ciliary marker acetylated‐*α*‐tubulin = acet‐*α*‐tubulin.

We next took several approaches to investigate the functions of BMPR2, TfR1, JAM‐A, PTPRS, and PGRMC2. First, we confirmed that the subcellular localization of these proteins was observed in the cELV (**Figure** **2**). Because of the microenvironment sensitivity of cELV to shear stress and the conditions of the immunostaining technique requiring a series of washing steps followed by fixation, all cELV were consequentially seen at the tip of the cilium. The size of the cELV at the tip of the cilia was not reflective of the actual size of a single cELV, as in many cases cELV could aggregate together, especially during shear‐stress.

**Figure 3:**
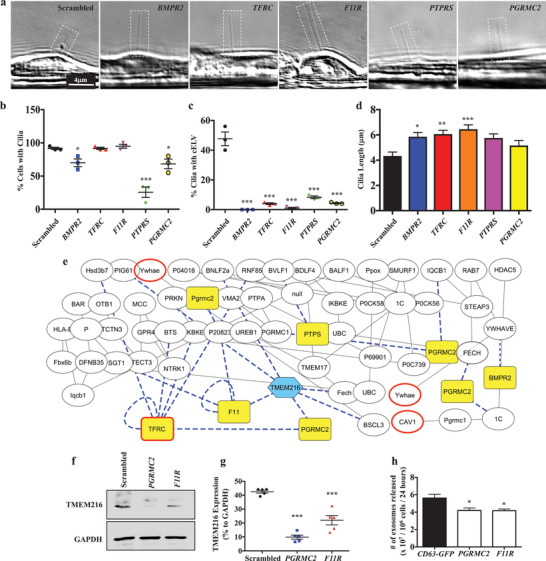
Selected cELV proteins are involved in cELV formation and suppression of *TMEM216* expression. a) A representative DIC images showing all corresponding knockdown genes impact on cilia and/or cELV formation. b,c) The effects of cELV proteins on cilia formation and cELV formation were quantified in *BMPR2*, *TFRC*, *F11R*, *PTPRS* and *PGRMC2* knockdown cells. d) The averaged cilium length is shown in the bar graph. The effect of the knockdown on ciliary length distributions is shown in the histogram (Figure S6d, Supporting Information). *N* = 3 with 40 measurements for each *N*. For the cELV measurements, total cELV from replicates are shown because most cilia did not have cELV. e) A network of protein–protein interactions analyzed from the proteomic studies shows the functional interface of the five selected cELV proteins (highlighted in yellow) with TMEM216 (highlighted in blue) and known EV marker proteins (bolded red boarder). Dotted blue lines represent an interaction with the selected ciliary proteins or TMEM216. f,g) A quantitative Western blot analysis of *TMEM216* expression was performed in *PGRMC2* or *F11R* knockdown cells. *N* = 5 for Western blot. h) The cELV secretion was quantified in *PGRMC2* or *F11R* knockdown cells. *N* = 3 for cELV quantification. *, *p* < 0.05; **, *p* < 0.01; ***, *p* < 0.001; compared with the scrambled control.

Second, we generated stable knockdown cells for each of the five corresponding genes (Figure S9, Supporting Information). For each knockdown, we used four potential sequences to select the sequence with the highest knockdown efficiency (see Methods in the Supporting Information). The formation of cilia and cELV were measured and quantified in these stable knockdown cells (**Figure** **3**a). BMPR2, PTPRS, and PGRMC2 were involved in cilia formation (Figure 3b). Even when cilia were present in these cells, the formation of cELV was significantly abolished in all five (*BMPR2*, *TFRC*, *F11R*, *PTPRS*, and *PGRMC2*) knockdown cells (Figure 3c). The *BMPR2* knockdown cells did not form cELV at all; *F11R* knockdown cells, when cELV did form, had much smaller cELV than the control cells. When the cilium length was tabulated, it showed that cilia were significantly longer in *BMPR2*, *TFRC*, and *F11R* knockdown cells than in the scrambled control cells (Figure 3d). Although *BMPR2* knockdown cells had significantly fewer cilia formation than the control cells, *BMPR2* knockdown cells had longer cilia when cilia were ever formed.

Third, we examined cellular proliferation and migration in stable knockdown cells (Figure S10, Supporting Information). Except for the *PTPRS* cells, which had a lower proliferation index, the proliferation indices for the rest of knockdown cells were increased when compared with the scrambled control cells. Compared with the control cells, all the knockdown cells exhibited a significant impairment in migration. Perhaps, the abnormal proliferation and migration, which were associated with cELV, were not surprising, given the roles of primary cilia in cell proliferation and migration.^[^ ^44^, ^45^ ^]^

Forth, to examine the relationship between the five‐selected proteins with each other and with other possible ciliopathy and EV proteins, we constructed a protein–protein interaction network from our proteomic database (Figure 3e). Interestingly, TFRC a known EV marker^[^ ^46^ ^]^ appeared in the network interaction. Likewise, other known EV markers (CAV1 and Ywhae) were also noted in the interaction network beside *TMEM216*.^[^ ^47^, ^48^ ^]^ Furthermore, while all five genes directly or indirectly interacted with each other and *TMEM216*, a gene responsible for Joubert syndrome and Meckel–Gruber syndrome ciliopathy disorders,^[^ ^3^, ^4^, ^5^ ^]^ *TMEM216* interacted directly with *F11R* and *PGRMC2* in the network interaction model. To validate this network model, we further investigated the interaction between these three proteins. *TMEM216* expression was significantly repressed in *F11R* and *PGRMC2* knockdown cells (Figure 3f,g).

Fifth, to identify the expression of *TMEM216*, *F11R*, and *PGRMC2* in cELV, we validated the expression of the exosomal marker CD63. There was a strong expression of *TMEM216* in the isolated cELV from the control cells but not from the knockdown cells. We also reverified our cELV isolation from CD63‐cELV. These results indicate that cELV release was impeded in the knockdown cells. Importantly, the total number of cELV collected from cell media was significantly decreased (Figure 3h).

### 2.4 Repressing cELV Protein Expression Results in Cystic Kidney, Hydrocephalus, Situs Inversus, and Cardiac Edema

To understand the in vivo relevance of these BMPR2, TfR1, JAM‐A, PTPRS, and PGRMC2 proteins, we first used zebrafish as a model to screen the phenotypes associated with abnormal cELV formation (Figure S12, Supporting Information). Because zebrafish has two isoforms (spice variants) of BMPR2, we examined the role of each variant (BMPR2A and BMPR2B). In these studies, scrambled and *Pkd2* knockdown were used as negative and positive controls, respectively. Although some variation and severity were observed, knockdown of the corresponding six genes (*BMPR2A*, *BMPR2B*, *TFRC*, *F11R*, *PTPRS*, and *PGRMC2*) resulted predominantly in ciliopathic disorders, including abnormal development, hydrocephalus, kidney cysts, and left–right asymmetry in cardiac looping (Movies S9 and S10, Supporting Information).

**Figure 4:**
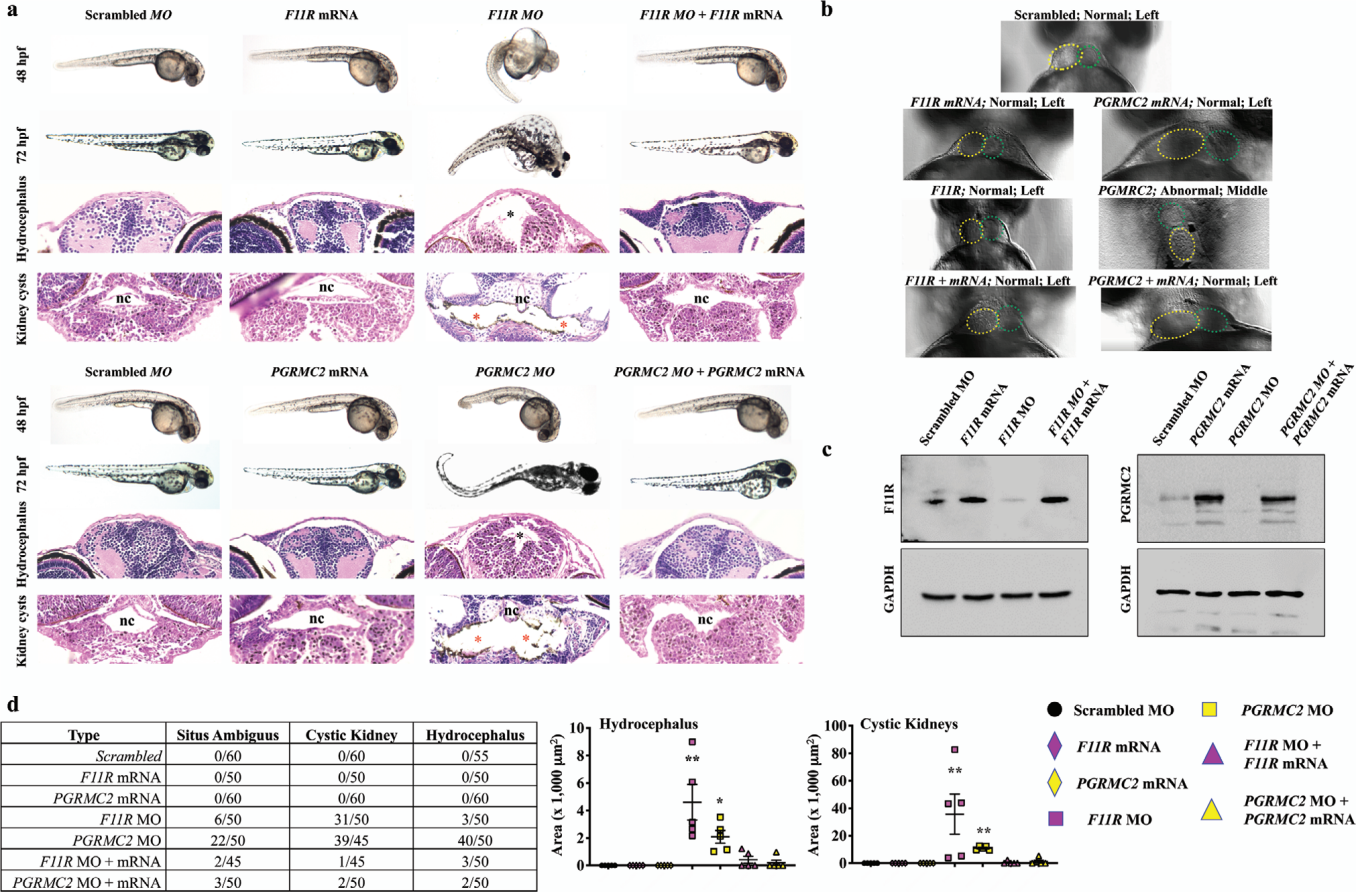
Repressed cELV protein expression results in ciliopathy phenotypes, pericardiac edema, and randomized heart position in zebrafish. a) Fish were injected with scrambled, specific *F11R* or *PGRMC2* mRNA, morpholino (MO), and both mRNA plus morpholino. Representative phase contrast images show zebrafish at 48 and 72 h postfertilization (hpf). The H&E images represent fish at 72‐hpf; black and red asterisks indicate hydrocephalus and renal cysts, respectively. nc = notochord. b) In addition to pericardiac edema (Figure S12, Supporting Information), randomized heart looping was observed in *PGRMC2* MO fish (Movie S9, Supporting Information) Yellow‐ and green‐dotted circles show ventricle and bulbus arteriosus, respectively. c) Confirmation of the rescue phenotype was confirmed with the corresponding protein expression via Western blot analysis. d) A quantitative analysis of asymmetry, kidney cysts and hydrocephalus depict the frequency and severity of the phenotypes. *N* = 40–60 fish per group. *, *p* < 0.05; **, *p* < 0.01; compared with the scrambled control.

Based on the protein interaction network interaction with *TMEM216*, *F11R*, and *PGRMC2* fish were selected for a more in‐depth analysis. The abnormal development of *F11R* and *PGRMC2* knockdown fish was indicated by either a strong dorsal axis curvature or incomplete development of the dorsal axis (**Figure** **4**a). Hydrocephalus and renal cysts were observed in the knockdown fish. The left–right asymmetry in cardiac looping was more apparent in *PGRMC2* than *F11R* fish (Figure 4b). The knockdown was rescued by introducing the corresponding human *PGRMC2* or human *F11R* into the fish. The expression of *PGRMC2* or *F11R* was confirmed (Figure 4c). The phenotypes from the corresponding knockdown fish were rescued by the expression of human *PGRMC2* or *F11R* (Figure 4d).

**Figure 5:**
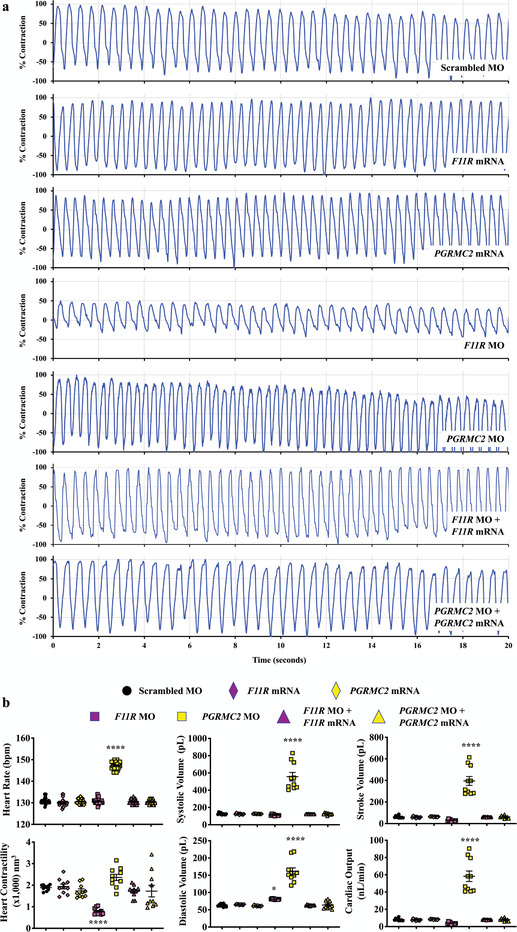
Repressing cELV protein expression affects the cardiac functions in zebrafish. Cardiac parameters were measured in all selected cELV proteins (Figure S12 and Movie S11, Supporting Information). a) cardiac contractility and heart rate were measured in fish injected with scrambled, specific *F11R* or *PGRMC2* mRNA, morpholino (MO), and both mRNA plus morpholino over a 20 s period (Movie S12, Supporting Information). b) The parameters of cardiac functions were quantified. *N* = 40–60 fish per group. *, *p* < 0.05; ****, *p* < 0.0001; compared with the scrambled control.

Pericardial edema was also observed in the majority of the knockdown fish, suggesting a potential cardiovascular abnormality. To investigate this possibility, we measured the main parameters of heart function (Movies S11 and S12, Supporting Information). We quantified heart rate and contractility in the knockdown and rescued fish (**Figure** **5**a). In *F11R* fish, cardiac contractility was decreased with an increased diastolic volume (Figure 5b). The data indicated that the *F11R* hearts were failing, and they were potentially compensated by an increase in preload (diastolic volume) to support normal stroke volume. In *PGRMC2* fish, heart rate was significantly increased, resulting in a marked increase in cardiac output. Interestingly, while the heart contractility was not significantly increased in *PGRMC2* fish, the systolic and diastolic volumes were markedly increased, resulting in a significant increase in stroke volume. The data indicated that the *PGRMC2* hearts were stimulated by the sympathetic innervation (increase heart rate), potentially to compensate for the weak cardiac contractility, i.e., after sympathetic stimulation, heart contractility was not changed while stroke volume increased through an increase in preload. Overall, the fish data indicated that from *F11R* and *PGRMC2* fish had failing hearts and were compensated by an increased in preload.

### 2.5 Mice Lacking cELV Are Characterized by Hypotension

**Figure 6:**
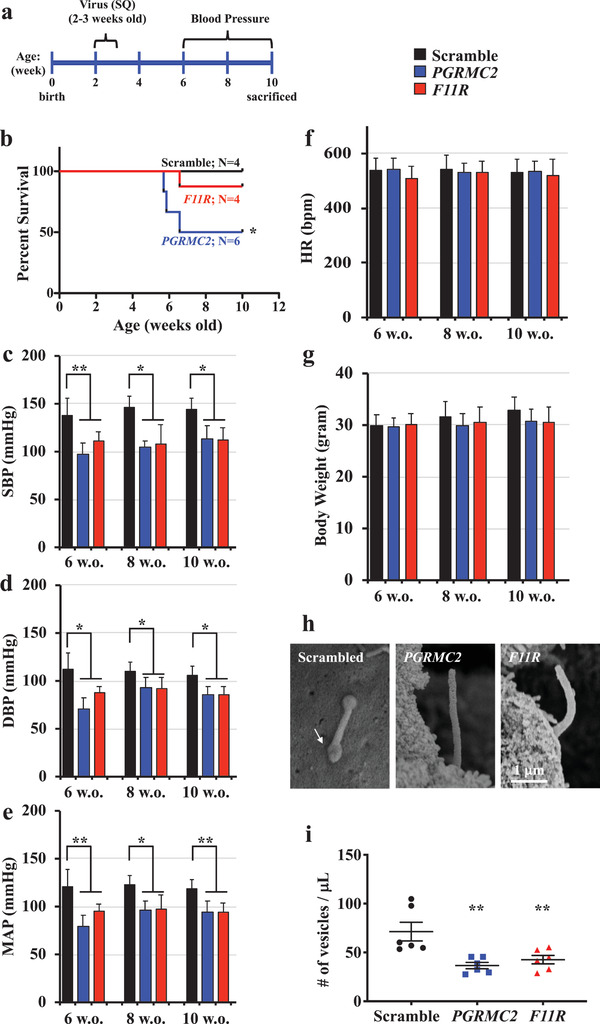
Mice lacking of cELV are hypotensive. a) Schematic timeline shows overall study approach in mice. Lentivirus was injected subcutaneously (SQ) into 2 week old mice. b) The survival curve shows that the *PGRMC2* knockdown mice had a significantly higher mortality rate. Systolic (SBP, c), diastolic (DBP, d) blood pressure, mean arterial pressure (MAP, e), heart rate (HR, f) and body weight (g) were recorded from 6 8, and 10 week old (w.o.) mice after 1 week of acclimation. h) Representative electron micrographs indicate *PGRMC2* and *F11R* knockdown mice lacked of cELV. Arrow indicates cELV in scrambled control mouse. i) Circulating cELVs were quantified by collecting serum of the mice at the end point of the study. *N* = 8–12 mice for the scrambled control, *PGRMC2* and *F11R* knockdown groups. *, *p* < 0.05; **, *p* < 0.01; compared with the scrambled control.

To validate zebrafish data, we used a lentivirus strategy to deliver *PGRMC2* or *F11R*‐targeted shRNA into 2 week old mice (**Figure** **6**a). The lentivirus infection was first verified using non‐GFP scrambled and GFP‐tagged *PGRMC2* or *F11R*‐targeted shRNA. We confirmed that shRNA was delivered to the heart (Figure S13, Supporting Information), in addition to other major visceral organs (Supplement S3, Supporting Information). We also verified the expressions of PGRMC2 and F11R. Based on the shRNA distribution, we designed our approach to include a complete evaluation of the cELV effect on these organs, especially to that of the cardiovascular function.

Four weeks after the *PGRMC2*‐targeted shRNA silencing, half of the mice died from cardiovascular collapse because of low blood pressure (Figure 6b). Compared with scrambled control mice, the systolic and diastolic blood pressures in *PGRMC2* and *F11R* mice were significantly lower (Figure 6c,d). The mean arterial pressure was also lower in *PGRMC2* and *F11R* mice compared with control mice but the heart rate was similar to that of control mice (Figure 6e,f). There were no apparent changes in body weight in both *PGRMC2* and *F11R* mice (Figure 6g). Further analyses confirmed that cELV was not present in *PGRMC2* or *F11R* mice (Figure 6h). Importantly, circulating vesicles (or cELV) collected from the blood serum were significantly repressed in *PGRMC2* or *F11R* mice (Figure 6i). Our in vivo data were consistent with our in vitro measurement showing decreased cELV and its release in *PGRMC2* or *F11R* cells. Extracardiac phenotypes were also observed (Supplement S3 and Movie S13, Supporting Information).

### 2.6 Mice Lacking cELV Have Arrhythmogenic Hearts Characterized with Compensated Left Ventricle Function and Cardiac Fibrosis

**Figure 7:**
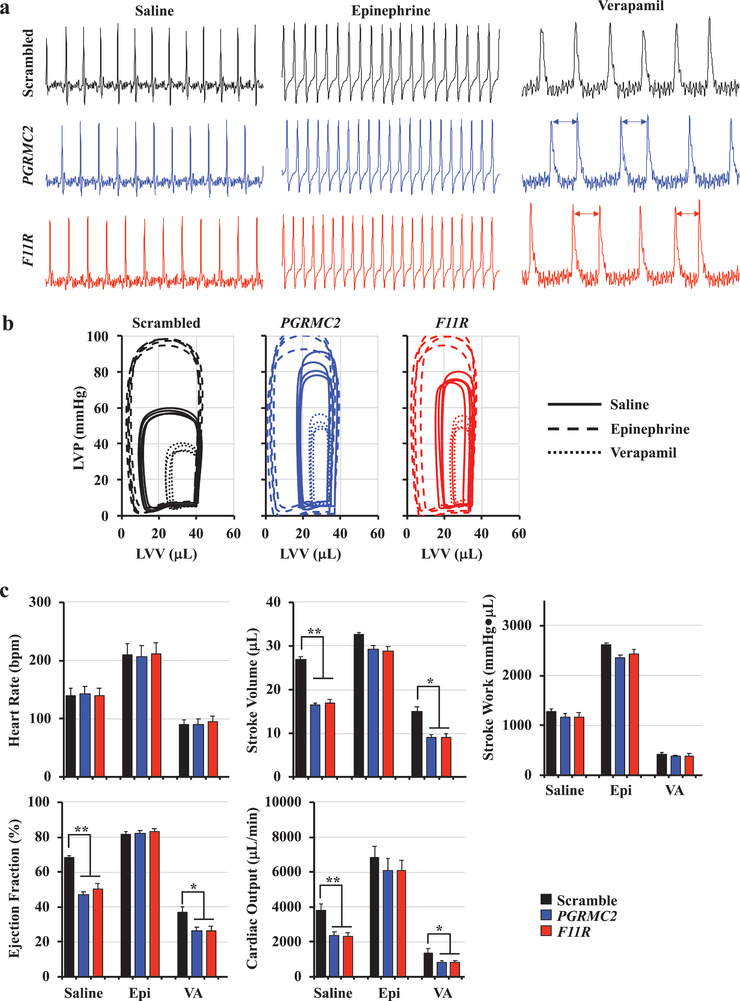
Mice lacking of cELV have arrhythmogenic heart with compensated left ventricle function. a) Measurements of heart electrical signal were performed using electrocardiograms in isolated hearts. Stress tests were performed with negative (verapamil; VA) or positive (epinephrine; Epi) chronotropic agents. Arrows indicate abnormal pacing. b) The relationship between the left ventricular pressure (LVP) and left ventricular volume (LVV) was analyzed as an index of heart function. c) Heart rate (bpm, beat per minute) and left ventricle functions were analyzed from the isolated working heart. A complete set of parameters for left ventricle function is shown in Table S3 (Supporting Information). *N* = 4 in each working heart analysis group. *, *p* < 0.05; **, *p* < 0.01; ***, *p* < 0.001; compared with the corresponding scrambled‐saline group.

To quantify cardiac performance, we measured cardiac parameters independent of the neuronal responses using the ex vivo isolated working heart system (Movie S14, Supporting Information). Abnormal rhythmic pacing was seen after the heart rate was slowed down with verapamil from 143 ± 12 to 91 ± 9 beats per minute (**Figure** **7**a), indicating that the hearts from *PGRMC2* and *F11R* mice could have been sustained with prolonged sympathetic stimulation. Further analyses of cardiac functions showed that the left ventricular volume was decreased while the left ventricular pressure increased in the hearts of *PGRMC2* and *F11R* knockdown mice (Figure 7b). When complete left ventricle parameters were measured (Table S3, Supporting Information), the overall left ventricle functions except stroke work were significantly lowered (Figure 7c). Because there was no change in heart rate, a decreased in cardiac output of *PGRMC2* and *F11R* hearts was primarily derived from a repressed in stroke volume. Decreased in ejection fraction was an indication of failing *PGRMC2* and *F11R* hearts. Consistent with this idea, hearts stimulation with epinephrine tended to correct ventricular functions comparable to the scrambled control.

**Figure 8:**
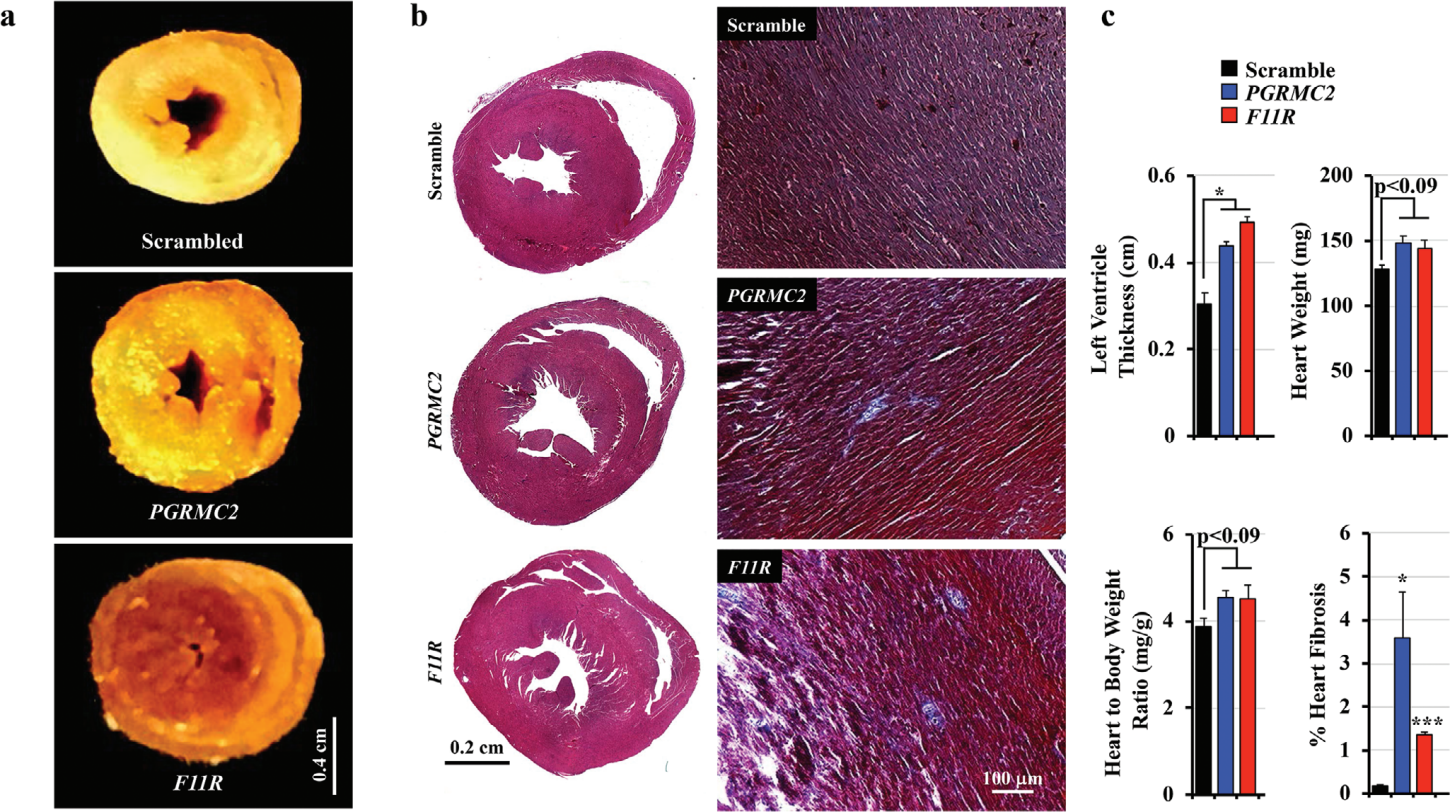
Mice lacking of cELVs have left ventricular hypertrophy and cardiac fibrosis. a) The thickness of the left ventricle was compared in transverse cut of whole mount hearts, showing left ventricular hypertrophy in *PGRMC2* and *F11R* mice. b) Representative images of H&E and Masson's Trichrome staining show left and right ventricles with significant fibrosis in the left ventricular endocardial regions of hearts from the *PGRMC2* and *F11R* knockdown mice. c) Left ventricular thickness, heart weight, heart‐to‐body weight ratio and percent of fibrosis in the heart were quantified. *N* = 3 mice in each scrambled control, *PGRMC2* and *F11R* knockdown groups. *, *p* < 0.05; ***, *p* < 0.001; compared with the corresponding scrambled control group.

The thickness of the left ventricle of *PGRMC2* and *F11R* hearts was compared in transverse cut of whole mount hearts (Figure 8a). Left ventricle hypertrophy was also apparent in these mice. Cardiac morphology was further analyzed with standard H&E and Masson's Trichrome staining (Figure 8b). The staining showed left and right ventricles with significant fibrosis in the left ventricular endocardial regions of hearts from the *PGRMC2* and *F11R* knockdown mice. While the hearts had significant hypertrophy, the mass of the heart and heart‐to‐body mass ratio were not significantly different in *F11R* or *PGRMC2* mice compared with scrambled control mice (Figure 8c). Together with left ventricular hypertrophy, such a cardiac phenotype potentially indicated aortic stenosis. As a result, the overall stroke volume and cardiac output are significantly decreased in mice lacking cELV.

## 3 Discussion

Our studies indicate that the ciliary extracellular‐like vesicle (cELV) and primary cilium influence the dynamic characteristics of each other. First, the cELV increases the flexural rigidity of cilia, likely due to an increase of protein transport from cell body to cilia. Consistently, blocking the cELV formation decreases ciliary mass and results in structurally less rigid primary cilia. Second, the bending of cilia changes the dynamic shape of the cELV (roundness, including circumference, and circularity) and allows the cELV to reposition to the tip of the cilium in response to fluid‐shear stress. This further indicates that cELV is not anchored to the ciliary axoneme and its movement along ciliary exoneme is regulated by the microenvironments surrounding the cell. Although tips of cilia can be excised through a process that is cell‐cycle dependent,^[^ ^26^ ^]^ surprisingly bending of a cilium by fluid‐shear stress is also sufficient to release the cELV. Third, the mass of the cELV is directly proportional to the mass of cilium.

To ensure that the cELV is not extracellular debris adhering onto the cilium, we have used different approaches to validate cELV is indeed generated from the cilia. First, unlike extracellular debris with random behaviors and patterns, the dynamic movements and characteristics of cELV along the cilia are mathematically not random. The cELV have predictable patterns (Movie S1 and Figure S1a–c, Supporting Information). Second, we have tagged the cEVL with known biomarker (CD63‐GFP) to selectively study and trace the movements of these vesicles every 12–24 h. CD63‐GFP is expressed throughout the cell body and cilia in the first 24 h post‐transfection and specifically in the cELV at 72 h post‐transfection (Figure 1a; Figure S2c, Supporting Information). Third, the cELV containing CD63‐GFP is uniquely localized to cilia. We never observe non‐GFP cELV in the cilia in cells expressing CD63‐GFP. The release of cELV from the primary cilium can also be observed (Movie S4, Supporting Information). Forth, when the cELV formation is inhibited, numbers of cilia with cELV are significantly decreased (Figure 3a,c). Fifth, the cELV formation can be induced by mechanical shear‐stress. Compared to static (no‐flow) condition where only 31% of cilia have cELV, shear‐flow induces cELV formation in over 85% of cilia (Movie S8, Supporting Information).^[^ ^42^ ^]^ Sixth, previous electron microscopy study shows that cELV is integrated within cilioplasm.^[^ ^49^ ^]^

Given that various proteins, RNAs, microRNAs, and even DNA materials have been found in common extracellular vesicle (EV),^[^ ^50^, ^51^, ^52^ ^]^ it opens the possibility that those contents could also be found in cELV. Further, this possibility could provide new functions of proteins or nucleotides within cilia that were once thought to be present only in the cytosol, as shown by this study that cytosolic EV proteins or markers can also be found in cELV. However, whether cELV can be assembled within the cilioplasm still remains unknown. Differentiating the origin of the cELV (cilia vs cytosolic) remains a challenge, because most known cytosolic EV markers are also found in the proteome of the cELV. However, we can differentiate cELV from other EV based on their release and signaling characteristics. To our advantage, since the cELV is induced by fluid‐shear stress^[^ ^42^ ^]^ and isolation of primary cilia is different from that of cytosolic EV, we isolated cilia containing cELV to avoid EV from the cell body. This enables us to distinguishably identify a list of a unique cELV protein composition by using differential proteomic analyses (Table S2, Supporting Information).

Validating five proteins (BMPR2, TfR1, JAM‐A, PTPRS, and PGRMC2) from the proteomic list show that these proteins are part of ciliary proteins, particularly localized in the cELV. It is very possible that not all proteins in our list localize in the cELV, as some proteins might be more specific to ciliary axonemes. However, due to the way in which we isolated the primary cilia, we hypothesize that the turnout of cELV proteins should be very high. It is also worth to note that many proteins in the list interact with each other and only few proteins interact with TMEM216, a protein involved in cilia formation.^[^ ^3^, ^4^, ^5^ ^]^ The biochemistry studies on the knockdown cells of all five genes show that some of them affected ciliogenesis, while all five knockdown cells show a significant impediment in cELV formation in addition to abnormal migration for wound closure. Importantly, knockdown of any of those five cELV protein significantly decreases release of cELV to the media. We thus speculate that the cell–cell communication process through primary cilia and cELV may be crucial in maintaining cellular function. The knockdown of cELV proteins can significantly interrupt the biogenesis of cELV and thus cellular function of many organs, including heart. In addition, the significant increase of cell proliferation index due to repressing the expression of cELV protein is indicative of the roles of cELV in cellular functions.

For the first time, we show that *PGRMC2* and *F11R* mice have cardiac arrhythmogenic defect characterized with a significant low ejection fraction (Table S3, Supporting Information). The left ventricular volume is decreased with augmented pressure in the hearts of mice lacking cELV. Both ejection fraction and cardiac output are decreased, and these characteristics are disappeared when challenged with epinephrine. Because arrhythmia is observed when the heart rate is decreased, the overall cardiac function could thus have been sustained via sympathetic stimulation. Together with left ventricular hypertrophy, such a cardiac phenotype indicates aortic stenosis, a cardiac abnormality commonly seen in patients with Joubert syndrome.^[^ ^53^ ^]^ Of note is that many cELV proteins directly or indirectly interacted with each other and *TMEM216*, a gene responsible for Joubert syndrome and Meckel–Gruber syndrome ciliopathy disorders.^[^ ^3^, ^4^, ^5^ ^]^ The aortic stenosis may thus explain the drop in blood pressure.^[^ ^54^, ^55^ ^]^ Overall, we here provide the first plausible explanation for primary cilia as antennae for cell–cell communication.

Repressing cELV protein expression in zebrafish animal model results in syndromic ciliopathy disorders and abnormal cardiovascular functions, including cystic kidney, hydrocephalus, randomized heart looping, pericardial edema, and abnormal cardiac functions. The regulation of *TMEM216* expression by F11R and PGRMC2 may partially explain the ciliopathy phenotypes seen in zebrafish model. However, we believe that the lacking of cELV has a predominant effect on the abnormal cardiac functions through the disruption of endocrine signaling mediators via cell–cell communication. Losing this signaling mechanism can prevent myocardial cells from transferring crucial proteins, miRNA and DNA from one cell to another. This cellular mechanism of cELVs is also observed in the mouse animal models, where low circulating cELVs affect the blood pressure, heart rhythm, left ventricle function, and myocardial morphology. While the role of cELVs is not known, microvesicles have been proposed to function as a secured and protected cell–cell communication device within the cardiovascular system.^[^ ^30^, ^31^, ^32^, ^33^ ^]^ In addition, cELVs are involved in Ca^2+^ and cAMP signaling (Figures S3–S7, Supporting Information). These second messengers are known to regulate contractility in the heart.^[^ ^56^, ^57^, ^58^ ^]^ All in all, we propose a cellular mechanism of primary cilia as follow: primary cilia (Figures 1 and 2) → cELVs (Figures 2 and 3) → endocrine signaling (Figures 4 and 5) → Ca2+ and cAMP signaling (Figures S3–S7, Supporting Information) → cardiovascular functions (Figures 6, 7, 8). Similar to the emerging evidence of extracellular vesicles as biomarkers,^[^ ^59^, ^60^, ^61^ ^]^ our results support that cELVs could potentially be used as a biomarker for cardiovascular and cilia‐related diseases.

In summary, we show that cELV is regulated and releasable by mechanical fluid force. We also report characteristics and impact of cELV on the cardiovascular system. Importantly, we have identified a novel protein composition of cELV by using differential proteomic analyses. Our results reveal that cELV plays a unique role in ciliary signaling, cellular functions, and maintaining cardiovascular homeostasis.

## Conflict of Interest

The authors declare no conflict of interest.

## Author Contributions

A.M.M. designed the experiments, analyzed data, drafted the manuscript, and oversaw the overall progress of the project. R.P. assisted with the zebrafish experiments and proteomic analyses (double‐blind). R.T.S. performed the cAMP signaling studies (double‐blind). M.A. and A.A. assisted in the proteomic analyses (Table S2, Supporting Information) and carried out other cell assays (double‐blind). A.A. provided all the calculations and statistical analyses. K.S. performed the working heart studies (double‐blind). W.A.A. performed some experiments in the early phase of the project. J.J.M. and J.R.Y. performed the proteomic studies and provided all relevant materials. S.M.N. designed experiments, finalized the manuscript, and oversaw the experimental progress of the project. All authors participated in reviewing the final draft of the manuscript.

## Supporting information

Supporting Informatio

Supplemental pdf 1

Supplemental pdf 2

Supplemental pdf 3

Supplemental Table 1

Supplemental Table 2

Supplemental Table 3

Supplemental Movie 1

Supplemental Movie 2

Supplemental Movie 3

Supplemental Movie 4

Supplemental Movie 5

Supplemental Movie 6

Supplemental Movie 7

Supplemental Movie 8

Supplemental Movie 9

Supplemental Movie 10

Supplemental Movie 11

Supplemental Movie 12

Supplemental Movie 13

Supplemental Movie 14

## References

[1] K. C. Corbit , P. Aanstad , V. Singla , A. R. Norman , D. Y. Stainier , J. F. Reiter , Nature 2005, 437, 1018. 16136078 10.1038/nature04117

[2] H. A. Praetorius , K. R. Spring , J. Membr. Biol. 2003, 191, 69. 12532278 10.1007/s00232-002-1042-4

[3] L. Huang , K. Szymanska , V. L. Jensen , A. R. Janecke , A. M. Innes , E. E. Davis , P. Frosk , C. Li , J. R. Willer , B. N. Chodirker , C. R. Greenberg , D. R. McLeod , F. P. Bernier , A. E. Chudley , T. Muller , M. Shboul , C. V. Logan , C. M. Loucks , C. L. Beaulieu , R. V. Bowie , S. M. Bell , J. Adkins , F. I. Zuniga , K. D. Ross , J. Wang , M. R. Ban , C. Becker , P. Nurnberg , S. Douglas , C. M. Craft , M. A. Akimenko , R. A. Hegele , C. Ober , G. Utermann , H. J. Bolz , D. E. Bulman , N. Katsanis , O. E. Blacque , D. Doherty , J. S. Parboosingh , M. R. Leroux , C. A. Johnson , K. M. Boycott , Am. J. Hum. Genet. 2011, 89, 713. 22152675 10.1016/j.ajhg.2011.11.005 PMC3234373

[4] F. R. Garcia‐Gonzalo , K. C. Corbit , M. S. Sirerol‐Piquer , G. Ramaswami , E. A. Otto , T. R. Noriega , A. D. Seol , J. F. Robinson , C. L. Bennett , D. J. Josifova , J. M. Garcia‐Verdugo , N. Katsanis , F. Hildebrandt , J. F. Reiter , Nat. Genet. 2011, 43, 776. 21725307 10.1038/ng.891 PMC3145011

[5] E. M. Valente , C. V. Logan , S. Mougou‐Zerelli , J. H. Lee , J. L. Silhavy , F. Brancati , M. Iannicelli , L. Travaglini , S. Romani , B. Illi , M. Adams , K. Szymanska , A. Mazzotta , J. E. Lee , J. C. Tolentino , D. Swistun , C. D. Salpietro , C. Fede , S. Gabriel , C. Russ , K. Cibulskis , C. Sougnez , F. Hildebrandt , E. A. Otto , S. Held , B. H. Diplas , E. E. Davis , M. Mikula , C. M. Strom , B. Ben‐Zeev , D. Lev , T. L. Sagie , M. Michelson , Y. Yaron , A. Krause , E. Boltshauser , N. Elkhartoufi , J. Roume , S. Shalev , A. Munnich , S. Saunier , C. Inglehearn , A. Saad , A. Alkindy , S. Thomas , M. Vekemans , B. Dallapiccola , N. Katsanis , C. A. Johnson , T. Attie‐Bitach , J. G. Gleeson , Nat. Genet. 2010, 42, 619. 20512146 10.1038/ng.594 PMC2894012

[6] C. C. Leitch , N. A. Zaghloul , E. E. Davis , C. Stoetzel , A. Diaz‐Font , S. Rix , M. Alfadhel , R. A. Lewis , W. Eyaid , E. Banin , H. Dollfus , P. L. Beales , J. L. Badano , N. Katsanis , Nat. Genet. 2008, 40, 443. 18327255 10.1038/ng.97

[7] S. J. Ansley , J. L. Badano , O. E. Blacque , J. Hill , B. E. Hoskins , C. C. Leitch , J. C. Kim , A. J. Ross , E. R. Eichers , T. M. Teslovich , A. K. Mah , R. C. Johnsen , J. C. Cavender , R. A. Lewis , M. R. Leroux , P. L. Beales , N. Katsanis , Nature 2003, 425, 628. 14520415 10.1038/nature02030

[8] M. Pema , L. Drusian , M. Chiaravalli , M. Castelli , Q. Yao , S. Ricciardi , S. Somlo , F. Qian , S. Biffo , A. Boletta , Nat. Commun. 2016, 7, 10786. 26931735 10.1038/ncomms10786 PMC4778067

[9] M. Ma , X. Tian , P. Igarashi , G. J. Pazour , S. Somlo , Nat. Genet. 2013, 45, 1004. 23892607 10.1038/ng.2715 PMC3758452

[10] N. J. Lambacher , A. L. Bruel , T. J. van Dam , K. Szymanska , G. G. Slaats , S. Kuhns , G. J. McManus , J. E. Kennedy , K. Gaff , K. M. Wu , R. van der Lee , L. Burglen , D. Doummar , J. B. Riviere , L. Faivre , T. Attie‐Bitach , S. Saunier , A. Curd , M. Peckham , R. H. Giles , C. A. Johnson , M. A. Huynen , C. Thauvin‐Robinet , O. E. Blacque , Nat. Cell Biol. 2016, 18, 122. 26595381 10.1038/ncb3273 PMC5580800

[11] A. C. Leightner , C. J. Hommerding , Y. Peng , J. L. Salisbury , V. G. Gainullin , P. G. Czarnecki , C. R. Sussman , P. C. Harris , Hum. Mol. Genet. 2013, 22, 2024. 23393159 10.1093/hmg/ddt054 PMC3695649

[12] D. U. Mick , R. B. Rodrigues , R. D. Leib , C. M. Adams , A. S. Chien , S. P. Gygi , M. V. Nachury , Dev. Cell. 2015, 35, 497. 26585297 10.1016/j.devcel.2015.10.015 PMC4662609

[13] H. Ishikawa , J. Thompson , J. R. Yates 3rd , W. F. Marshall , Curr. Biol. 2012, 22, 414. 22326026 10.1016/j.cub.2012.01.031 PMC3298568

[14] L. Sang , J. J. Miller , K. C. Corbit , R. H. Giles , M. J. Brauer , E. A. Otto , L. M. Baye , X. Wen , S. J. Scales , M. Kwong , E. G. Huntzicker , M. K. Sfakianos , W. Sandoval , J. F. Bazan , P. Kulkarni , F. R. Garcia‐Gonzalo , A. D. Seol , J. F. O'Toole , S. Held , H. M. Reutter , W. S. Lane , M. A. Rafiq , A. Noor , M. Ansar , A. R. Devi , V. C. Sheffield , D. C. Slusarski , J. B. Vincent , D. A. Doherty , F. Hildebrandt , J. F. Reiter , P. K. Jackson , Cell 2011, 145, 513. 21565611 10.1016/j.cell.2011.04.019 PMC3383065

[15] G. J. Pazour , N. Agrin , J. Leszyk , G. B. Witman , J. Cell Biol. 2005, 170, 103. 15998802 10.1083/jcb.200504008 PMC2171396

[16] P. N. Dilly , Cell Tissue Res. 1977, 180, 367. 872201 10.1007/BF00227602

[17] G. Short , S. L. Tamm , Biol. Bull. 1991, 180, 466. 29304655 10.2307/1542347

[18] K. E. Roth , C. L. Rieder , S. S. Bowser , J. Cell Sci. 1988, 89, 457. 3058727 10.1242/jcs.89.4.457

[19] P. N. Dilly , Cell Tissue Res. 1977, 185, 105. 589661 10.1007/BF00226672

[20] M. C. Hogan , L. Manganelli , J. R. Woollard , A. I. Masyuk , T. V. Masyuk , R. Tammachote , B. Q. Huang , A. A. Leontovich , T. G. Beito , B. J. Madden , M. C. Charlesworth , V. E. Torres , N. F. LaRusso , P. C. Harris , C. J. Ward , J. Am. Soc. Nephrol. 2009, 20, 278. 19158352 10.1681/ASN.2008060564 PMC2637052

[21] V. Dubreuil , A. M. Marzesco , D. Corbeil , W. B. Huttner , M. Wilsch‐Brauninger , J. Cell Biol. 2007, 176, 483. 17283184 10.1083/jcb.200608137 PMC2063983

[22] C. R. Wood , K. Huang , D. R. Diener , J. L. Rosenbaum , Curr. Biol. 2013, 23, 906. 23623554 10.1016/j.cub.2013.04.019 PMC3850760

[23] J. Wang , R. Kaletsky , M. Silva , A. Williams , L. A. Haas , R. J. Androwski , J. N. Landis , C. Patrick , A. Rashid , D. Santiago‐Martinez , M. Gravato‐Nobre , J. Hodgkin , D. H. Hall , C. T. Murphy , M. M. Barr , Curr. Biol. 2015, 25, 3232. 26687621 10.1016/j.cub.2015.10.057 PMC4698341

[24] H. Long , F. Zhang , N. Xu , G. Liu , D. R. Diener , J. L. Rosenbaum , K. Huang , Curr. Biol. 2016, 26, 3327. 27866888 10.1016/j.cub.2016.09.055 PMC5173405

[25] A. R. Nager , J. S. Goldstein , V. Herranz‐Perez , D. Portran , F. Ye , J. M. Garcia‐Verdugo , M. V. Nachury , Cell 2017, 168, 252. 28017328 10.1016/j.cell.2016.11.036 PMC5235987

[26] S. C. Phua , S. Chiba , M. Suzuki , E. Su , E. C. Roberson , G. V. Pusapati , M. Setou , R. Rohatgi , J. F. Reiter , K. Ikegami , T. Inoue , Cell 2017, 168, 264. 28086093 10.1016/j.cell.2016.12.032 PMC5660509

[27] R. O'Hagan , M. Silva , K. C. Q. Nguyen , W. Zhang , S. Bellotti , Y. H. Ramadan , D. H. Hall , M. M. Barr , Curr. Biol. 2017, 27, 3430. 29129530 10.1016/j.cub.2017.09.066 PMC5698134

[28] R. O'Hagan , B. P. Piasecki , M. Silva , P. Phirke , K. C. Nguyen , D. H. Hall , P. Swoboda , M. M. Barr , Curr. Biol. 2011, 21, 1685. 21982591 10.1016/j.cub.2011.08.049 PMC4680987

[29] J. P. Sluijter , V. Verhage , J. C. Deddens , F. van den Akker , P. A. Doevendans , Cardiovasc. Res. 2014, 102, 302. 24488559 10.1093/cvr/cvu022

[30] S. M. Davidson , J. A. Riquelme , Y. Zheng , J. M. Vicencio , S. Lavandero , D. M. Yellon , Sci. Rep. 2018, 8, 15885. 30367147 10.1038/s41598-018-34357-z PMC6203728

[31] T. M. Ribeiro‐Rodrigues , T. L. Laundos , R. Pereira‐Carvalho , D. Batista‐Almeida , R. Pereira , V. Coelho‐Santos , A. P. Silva , R. Fernandes , M. Zuzarte , F. J. Enguita , M. C. Costa , O. P. Pinto‐do , M. T. Pinto , P. Gouveia , L. Ferreira , J. C. Mason , P. Pereira , B. R. Kwak , D. S. Nascimento , H. Girao , Cardiovasc. Res. 2017, 113, 1338. 28859292 10.1093/cvr/cvx118

[32] T. Nozaki , S. Sugiyama , H. Koga , K. Sugamura , K. Ohba , Y. Matsuzawa , H. Sumida , K. Matsui , H. Jinnouchi , H. Ogawa , J. Am. Coll. Cardiol. 2009, 54, 601. 19660689 10.1016/j.jacc.2009.05.022

[33] E. Hergenreider , S. Heydt , K. Treguer , T. Boettger , A. J. Horrevoets , A. M. Zeiher , M. P. Scheffer , A. S. Frangakis , X. Yin , M. Mayr , T. Braun , C. Urbich , R. A. Boon , S. Dimmeler , Nat. Cell Biol. 2012, 14, 249. 22327366 10.1038/ncb2441

[34] Z. Mallat , H. Benamer , B. Hugel , J. Benessiano , P. G. Steg , J. M. Freyssinet , A. Tedgui , Circulation 2000, 101, 841. 10694520 10.1161/01.cir.101.8.841

[35] S. M. Nauli , F. J. Alenghat , Y. Luo , E. Williams , P. Vassilev , X. Li , A. E. Elia , W. Lu , E. M. Brown , S. J. Quinn , D. E. Ingber , J. Zhou , Nat. Genet. 2003, 33, 129. 12514735 10.1038/ng1076

[36] K. Essandoh , L. Yang , X. Wang , W. Huang , D. Qin , J. Hao , Y. Wang , B. Zingarelli , T. Peng , G. C. Fan , Biochim. Biophys. Acta, Mol. Basis Dis. 2015, 1852, 2362. 10.1016/j.bbadis.2015.08.010 PMC4581992 26300484

[37] J. Li , K. Liu , Y. Liu , Y. Xu , F. Zhang , H. Yang , J. Liu , T. Pan , J. Chen , M. Wu , X. Zhou , Z. Yuan , Nat. Immunol. 2013, 14, 793. 23832071 10.1038/ni.2647

[38] K. Trajkovic , C. Hsu , S. Chiantia , L. Rajendran , D. Wenzel , F. Wieland , P. Schwille , B. Brugger , M. Simons , Science 2008, 319, 1244. 18309083 10.1126/science.1153124

[39] M. Logozzi , A. De Milito , L. Lugini , M. Borghi , L. Calabro , M. Spada , M. Perdicchio , M. L. Marino , C. Federici , E. Iessi , D. Brambilla , G. Venturi , F. Lozupone , M. Santinami , V. Huber , M. Maio , L. Rivoltini , S. Fais , PLoS One 2009, 4, e5219. 19381331 10.1371/journal.pone.0005219 PMC2667632

[40] V. Pospichalova , J. Svoboda , Z. Dave , A. Kotrbova , K. Kaiser , D. Klemova , L. Ilkovics , A. Hampl , I. Crha , E. Jandakova , L. Minar , V. Weinberger , V. Bryja , J. Extracell. Vesicles 2015, 4, 25530. 25833224 10.3402/jev.v4.25530 PMC4382613

[41] J. Lotvall , A. F. Hill , F. Hochberg , E. I. Buzas , D. Di Vizio , C. Gardiner , Y. S. Gho , I. V. Kurochkin , S. Mathivanan , P. Quesenberry , S. Sahoo , H. Tahara , M. H. Wauben , K. W. Witwer , C. Thery , J. Extracell. Vesicles 2014, 3, 26913. 25536934 10.3402/jev.v3.26913 PMC4275645

[42] A. M. Mohieldin , H. S. Haymour , S. T. Lo , W. A. AbouAlaiwi , K. F. Atkinson , C. J. Ward , M. Gao , O. Wessely , S. M. Nauli , Cell. Mol. Life Sci. 2015, 72, 2415. 25650235 10.1007/s00018-015-1838-x PMC4503369

[43] J. R. Yates , C. I. Ruse , A. Nakorchevsky , Annu. Rev. Biomed. Eng. 2009, 11, 49. 19400705 10.1146/annurev-bioeng-061008-124934

[44] J. Munoz‐Estrada , R. J. Ferland , J. Cell Sci. 2019, 132, jcs230680. 31391239 10.1242/jcs.230680 PMC6771145

[45] J. Lee , S. Yi , J. Y. Chang , J. T. Kim , H. J. Sul , K. C. Park , X. Zhu , S. Y. Cheng , J. Kero , J. Kim , M. Shong , Mol. Cells 2019, 42, 113. 30622229 10.14348/molcells.2018.0430 PMC6399002

[46] A. Calzolari , C. Raggi , S. Deaglio , N. M. Sposi , M. Stafsnes , K. Fecchi , I. Parolini , F. Malavasi , C. Peschle , M. Sargiacomo , U. Testa , J. Cell Sci. 2006, 119, 4486. 17046995 10.1242/jcs.03228

[47] S. Keerthikumar , D. Chisanga , D. Ariyaratne , H. Al Saffar , S. Anand , K. Zhao , M. Samuel , M. Pathan , M. Jois , N. Chilamkurti , L. Gangoda , S. Mathivanan , J. Mol. Biol. 2016, 428, 688. 26434508 10.1016/j.jmb.2015.09.019 PMC4783248

[48] K. J. Svensson , H. C. Christianson , A. Wittrup , E. Bourseau‐Guilmain , E. Lindqvist , L. M. Svensson , M. Morgelin , M. Belting , J. Biol. Chem. 2013, 288, 17713. 23653359 10.1074/jbc.M112.445403 PMC3682571

[49] A. M. Mohieldin , W. A. AbouAlaiwi , M. Gao , S. M. Nauli , Sci. Rep. 2015, 5, 15982. 26521680 10.1038/srep15982 PMC4629161

[50] A. Takahashi , R. Okada , K. Nagao , Y. Kawamata , A. Hanyu , S. Yoshimoto , M. Takasugi , S. Watanabe , M. T. Kanemaki , C. Obuse , E. Hara , Nat. Commun. 2017, 8, 15287. 28508895 10.1038/ncomms15287 PMC5440838

[51] S. Mathivanan , R. J. Simpson , Proteomics 2009, 9, 4997. 19810033 10.1002/pmic.200900351

[52] D. D. Taylor , C. Gercel‐Taylor , Gynecol. Oncol. 2008, 110, 13. 18589210 10.1016/j.ygyno.2008.04.033

[53] N. Karp , L. Grosse‐Wortmann , S. Bowdin , Eur. J. Med. Genet. 2012, 55, 605. 22910529 10.1016/j.ejmg.2012.07.010

[54] A. Ozkan , S. Kapadia , M. Tuzcu , T. H. Marwick , Nat. Rev. Cardiol. 2011, 8, 494. 21670747 10.1038/nrcardio.2011.80

[55] U. N. Khot , G. M. Novaro , Z. B. Popovic , R. M. Mills , J. D. Thomas , E. M. Tuzcu , D. Hammer , S. E. Nissen , G. S. Francis , N. Engl. J. Med. 2003, 348, 1756. 12724481 10.1056/NEJMoa022021

[56] C. Vettel , M. Lindner , M. Dewenter , K. Lorenz , C. Schanbacher , M. Riedel , S. Lammle , S. Meinecke , F. E. Mason , S. Sossalla , A. Geerts , M. Hoffmann , F. Wunder , F. J. Brunner , T. Wieland , H. Mehel , S. Karam , P. Lechene , J. Leroy , G. Vandecasteele , M. Wagner , R. Fischmeister , A. El‐Armouche , Circ. Res. 2017, 120, 120. 27799254 10.1161/CIRCRESAHA.116.310069

[57] B. S. Muntean , C. M. Horvat , J. H. Behler , W. A. Aboualaiwi , A. M. Nauli , F. E. Williams , S. M. Nauli , Front. Pharmacol. 2010, 1, 139. 21833178 10.3389/fphar.2010.00139 PMC3153013

[58] D. M. Bers , Annu. Rev. Physiol. 2008, 70, 23. 17988210 10.1146/annurev.physiol.70.113006.100455

[59] L. Console , M. Scalise , C. Indiveri , Clin. Chim. Acta 2019, 488, 165. 30419221 10.1016/j.cca.2018.11.009

[60] M. Madeo , P. L. Colbert , D. W. Vermeer , C. T. Lucido , J. T. Cain , E. G. Vichaya , A. J. Grossberg , D. Muirhead , A. P. Rickel , Z. Hong , J. Zhao , J. M. Weimer , W. C. Spanos , J. H. Lee , R. Dantzer , P. D. Vermeer , Nat. Commun. 2018, 9, 4284. 30327461 10.1038/s41467-018-06640-0 PMC6191452

[61] G. Chen , A. C. Huang , W. Zhang , G. Zhang , M. Wu , W. Xu , Z. Yu , J. Yang , B. Wang , H. Sun , H. Xia , Q. Man , W. Zhong , L. F. Antelo , B. Wu , X. Xiong , X. Liu , L. Guan , T. Li , S. Liu , R. Yang , Y. Lu , L. Dong , S. McGettigan , R. Somasundaram , R. Radhakrishnan , G. Mills , Y. Lu , J. Kim , Y. H. Chen , H. Dong , Y. Zhao , G. C. Karakousis , T. C. Mitchell , L. M. Schuchter , M. Herlyn , E. J. Wherry , X. Xu , W. Guo , Nature 2018, 560, 382. 30089911 10.1038/s41586-018-0392-8 PMC6095740

