# Supplementary material for: Proteomic Identification Reveals the Role of Ciliary Extracellular‐Like Vesicle in Cardiovascular Function
Source: Adv Sci (Weinh). 2020 Jun 16;7(16):1903140. doi: 10.1002/advs.201903140 (PMC7435257; doi:10.1002/advs.201903140)
Supplement: Supplementary file 1 — Supporting Informatio [file ADVS-7-1903140-s001.pdf]

Supporting Information: Online Supplement Figures, Legends, Methods, and Discussion

Proteomic identification reveals the role of ciliary extracellular-like vesicle in cardiovascular function.

Ashraf M. Mohieldin[1], Rajasekharreddy Pala[1], Rinzhin T. Sherpa[1], Madhawi Alanazi[1], Ashwaq Alanazi[1], Kiumars Shamloo[1], Amir Ahsan[2], Wissam A. AbouAlaiwi[3], James J. Moresco[5], John R. Yates, III[5], Surya M. Nauli[1,6]

[1]Department of Biomedical & Pharmaceutical Sciences, Chapman University, Irvine, CA 92618, USA.
[2]Department of Physics, Computer Science & Engineering, Chapman University, Orange, CA 92866, USA
[3]Department of Pharmacology and Experimental Therapeutics, University of Toledo, Toledo, OH 43614, USA.
[5]Department of Molecular Medicine, The Scripps Research Institute, La Jolla, CA 92037, USA
[6]Department of Medicine, University of California Irvine, Irvine, CA 92868, USA

Corresponding author:
Surya Nauli
Chapman University
The University of California, Irvine
9401 Jeronimo Road.
Irvine, CA 92618-1908
Tel: 714-516-5480
Fax: 714-516-5481
Email: nauli@chapman.edu; snauli@uci.edu

Supplement Information:

1. **Supplement Figures and Legends** (pages 3-20)

2. **Supplement Movie Legends** (pages 21-23)

3. **Supplement Methods** (pages 24-36)

4. **Supplement Discussion** (37-40)

5. **Supplement References** (41-42)

1. Supplement Figures and Legends

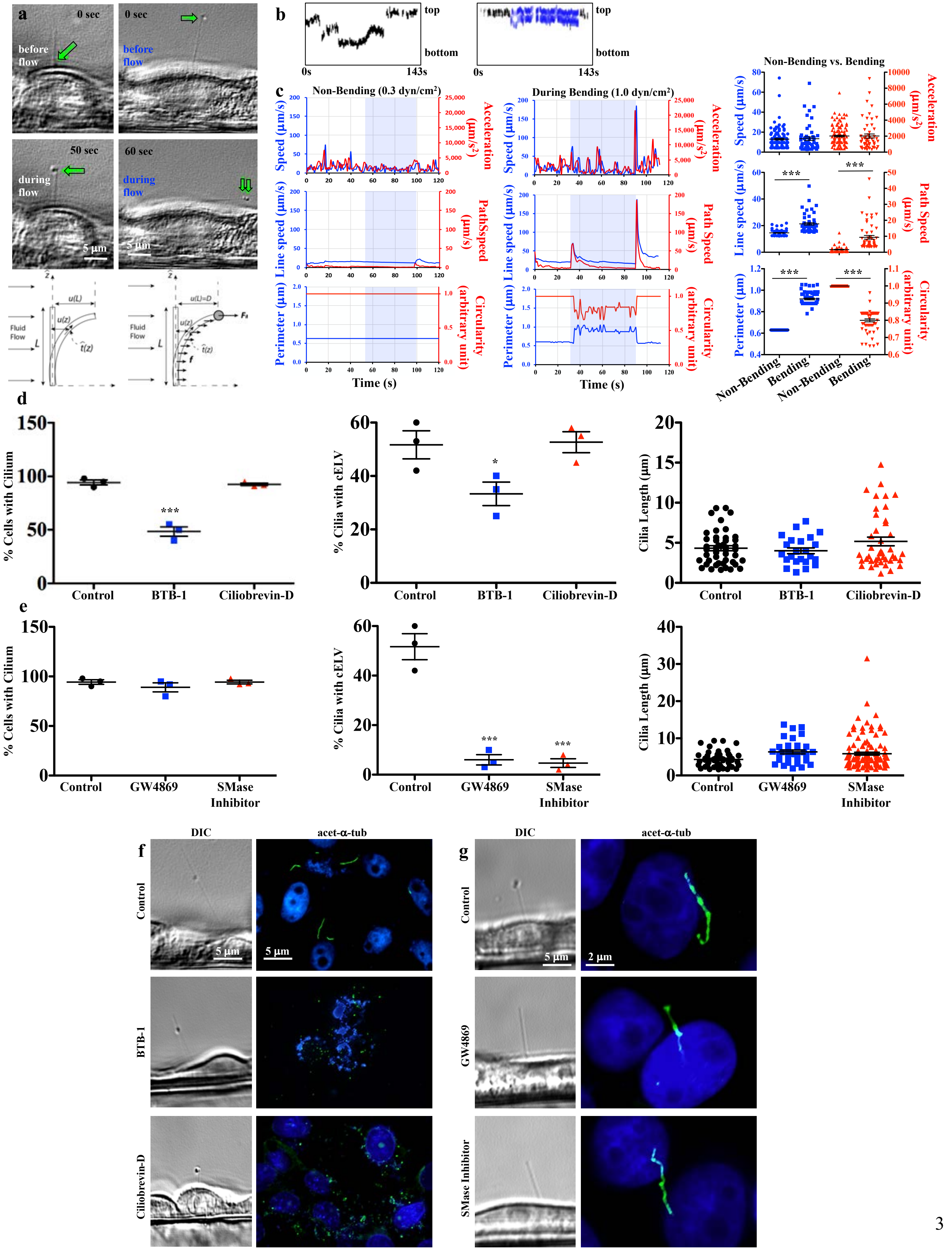

Figure S1. Dynamic cELV can be regulated mechanically.

a, Representative live images were taken from 2-minute videos depicting the dynamic movement of the cELV from non-bending and bending cilia induced by fluid shear stress (0.3 and 1 dyn/cm^2, respectively, **Movie S1 and S2**). A mathematical model explained the rigidity of the primary cilium in the absence and presence of cELV (**Supplement 1**). The presence of the cELV enhanced the reflection of the cilium, as described by the drag force on the cELV (F_B). **b,** A kymograph analysis demonstrates the dynamics of the movement of the cELV along the ciliary axoneme in seconds (s). The blue kymograph indicates a cilium during bending; the bottom and top depict the distal base and the proximal tip of a cilium. **c,** The presence of shear stress (0.3 or 1 dyn/cm^2) is shown in the blue shaded area on the graphs. The acceleration, speed and circularity of the cELV were quantified (**Movie S1 and S2**). The circularity of cELV was optically segmented to indicate a value of 1 for a perfect circle and a value of 0 for a rectangular. **d,** The kinesin inhibitor (BTB-1; 100 μM) but not the dynein inhibitor (ciliobrevin D; 100 μM) significantly decreased the number of cilia and cELV. Cilia length (N=23-46 replicates) were not altered by BTB-1 or ciliobrevin D. **e,** The inhibitors (20 μM GW4869 or 10 μM N-SMase Spiroepoxide inhibitor) did not alter the presence of cilia, but they decreased the number of cELV. GW4869 and SMase did not alter cilia length (N=35-120 replicates). **f,** Because the kinesin inhibitor (BTB-1; 100 μM) and the dynein inhibitor (ciliobrevin D; 100 μM) alter cell division, they were applied after the cells were fully differentiated, as evidenced from the presence of primary cilia (left panels). When applied prior to cell differentiation, both inhibitors prevented cell division or cilia formation (right panels). Primary cilia were stained with acetylated-α-tubulin (green) and the nucleus was stained with DAPI (blue). **g,** The effects of inhibitors of cELV formation (20 μM GW4869 or 10 μM N-SMase Spiroepoxide inhibitor) were analyzed in primary cilia stained with acetylated-α-tubulin (green) and with nuclei stained with DAPI (blue). N=154 for the kymograph analysis; unless otherwise indicated, N=3 in each group for the rest of the studies. *, $p<0.05$; ***, $p<0.001$.

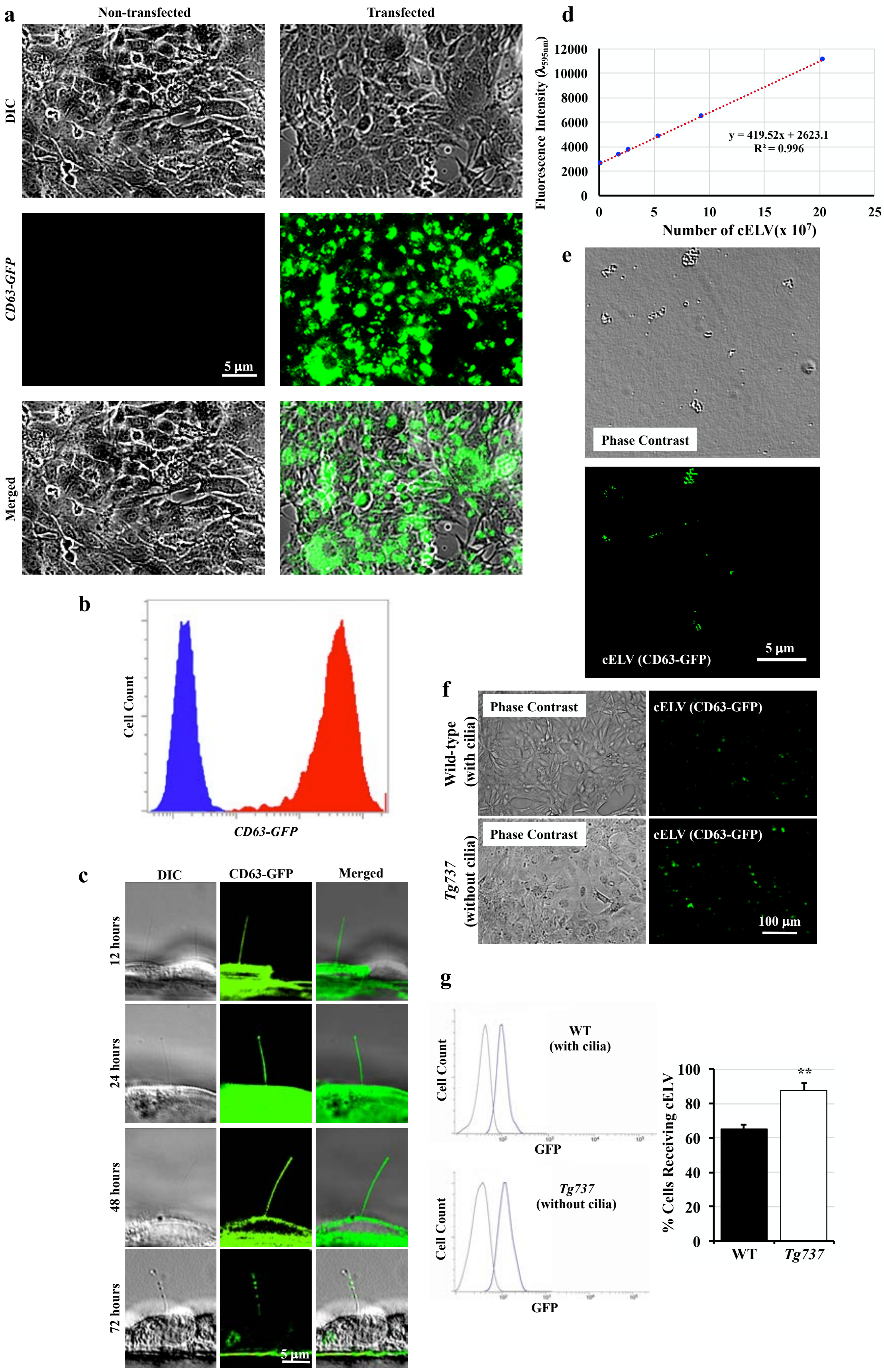

a
Non-transfected
Transfected
DIC
CD63-GFP
5 µm
Merged
b
Cell Count
CD63-GFP
c
DIC
CD63-GFP
Merged
12 hours
24 hours
48 hours
72 hours
5 µm
d
Fluorescence Intensity (λ595nm)
12000
10000
8000
6000
4000
2000
0
0
5
10
15
20
25
Number of cELV(x 10^7)
y = 419.52x + 2623.1
R² = 0.996
e
Phase Contrast
cELV (CD63-GFP)
5 µm
f
Wild-type (with cilia)
Tg737 (without cilia)
Phase Contrast
cELV (CD63-GFP)
100 µm
g
Cell Count
GFP
WT (with cilia)
Tg737 (without cilia)
% Cells Receiving cELV
100
80
60
40
20
0
WT
Tg737
**

Figure S2. Characterization of cELV.

a, The CD63-GFP construct was incorporated into the lentiviral vector (pCT-CD63-GFP). After puromycin selection, CD63-GFP expression was examined and compared with mock transduced cells. **b,** Flow cytometry indicated that the majority of cells expressed CD63-GFP after puromycin selection (red), compared with mock control transduced cells (blue). **c,** To understand the formation of the ciliary ELV, pCT-CD63-GFP was introduced into cells and CD63-GFP expression was observed every 12 to 24 hours. CD63-GFP expression was localized to the cELV 72 hours post-transfection. **d,** A standard curve was generated to quantify CD63-GFP-containing cELV release. **e,** Phase contrast and fluorescent images showed isolated cELV. **f,** Representative images of phase contrast and fluorescence microscopy show the uptake of extracellular vesicle containing CD63-GFP differently in ciliated and non-ciliated cells. **g,** Representative flow cytometry measurements show the uptake of cELV containing CD63-GFP in ciliated wild-type (WT) and non-ciliated (*Tg737*) cells. N=3 for each group. **, $p<0.01$

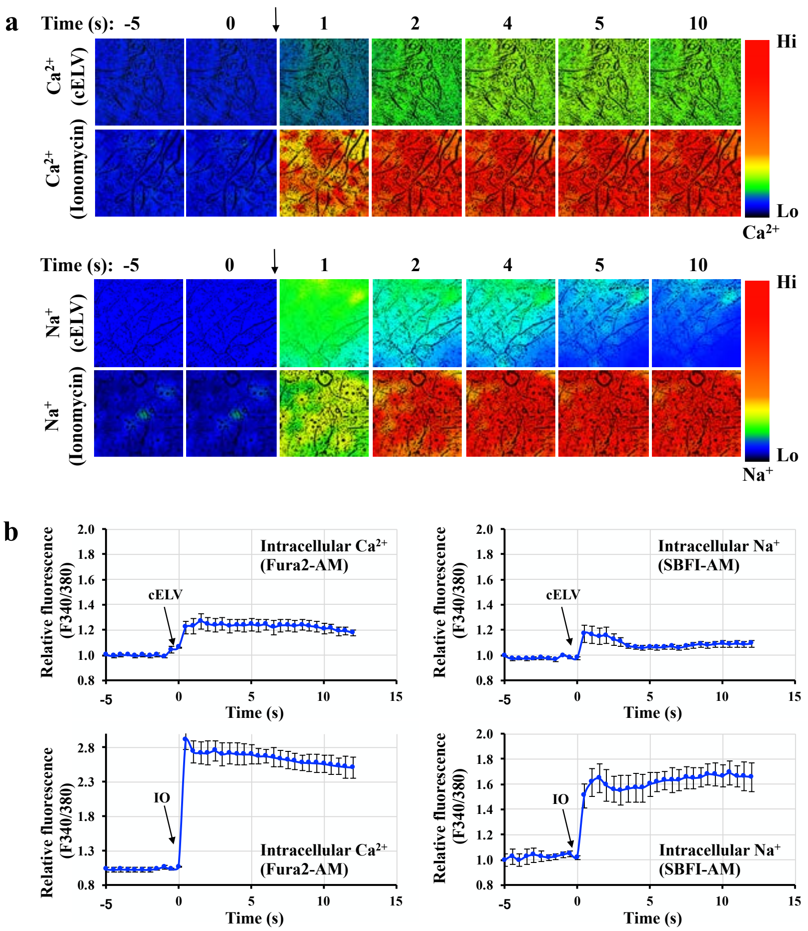

Figure S3. The cELV increases intracellular calcium and sodium levels in monolayer cells.

a, Intracellular calcium (Ca^{2+}) and sodium (Na^{+}) were imaged separately using Fura2-AM and SBFI-AM ratiometric indicators, respectively. Ratio images from 340 and 380 nm excitation wavelengths were captured at 32 fps. Numbers on the top of the representative images display time in seconds (s). The arrow indicates the addition of cELV ($5\times10^{6}/\mu L$) or ionomycin (positive control; 1 μM). Colored bars show the level of intracellular Na^{+} or Ca^{2+}. Complete Ca^{2+} and Na^{+} movies for cELV or ionomycin treatments are available in **Movie S6**. **b,** Line plots of the mean intracellular 340 nm and 380 nm fluorescence ratio (F340/380) traces in seconds (s). Arrows indicate the addition of cELV or ionomycin (IO). N=6 in each Ca^{2+} or Na^{+} study.

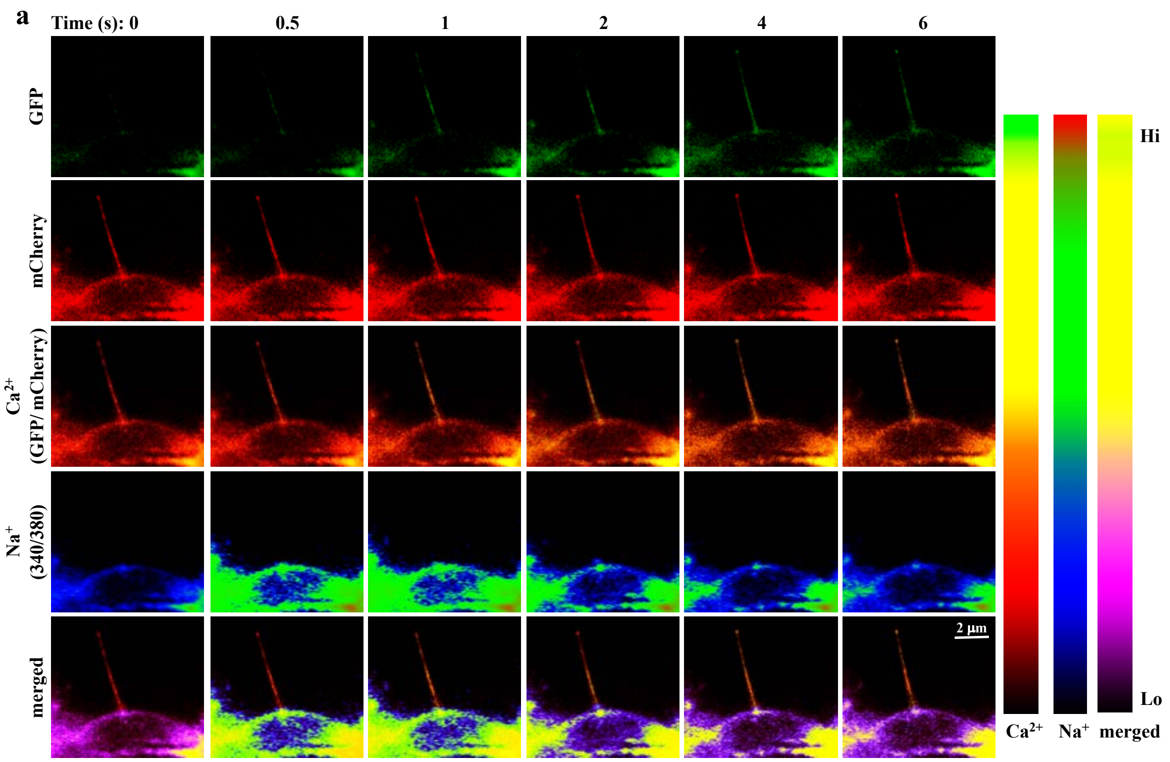
a
Time (s): 0
0.5
1
2
4
6
GFP
mCherry
Ca2+ (GFP/ mCherry)
Na+ (340/380)
merged
2 μm
Hi
Lo
Ca2+
Na+
merged

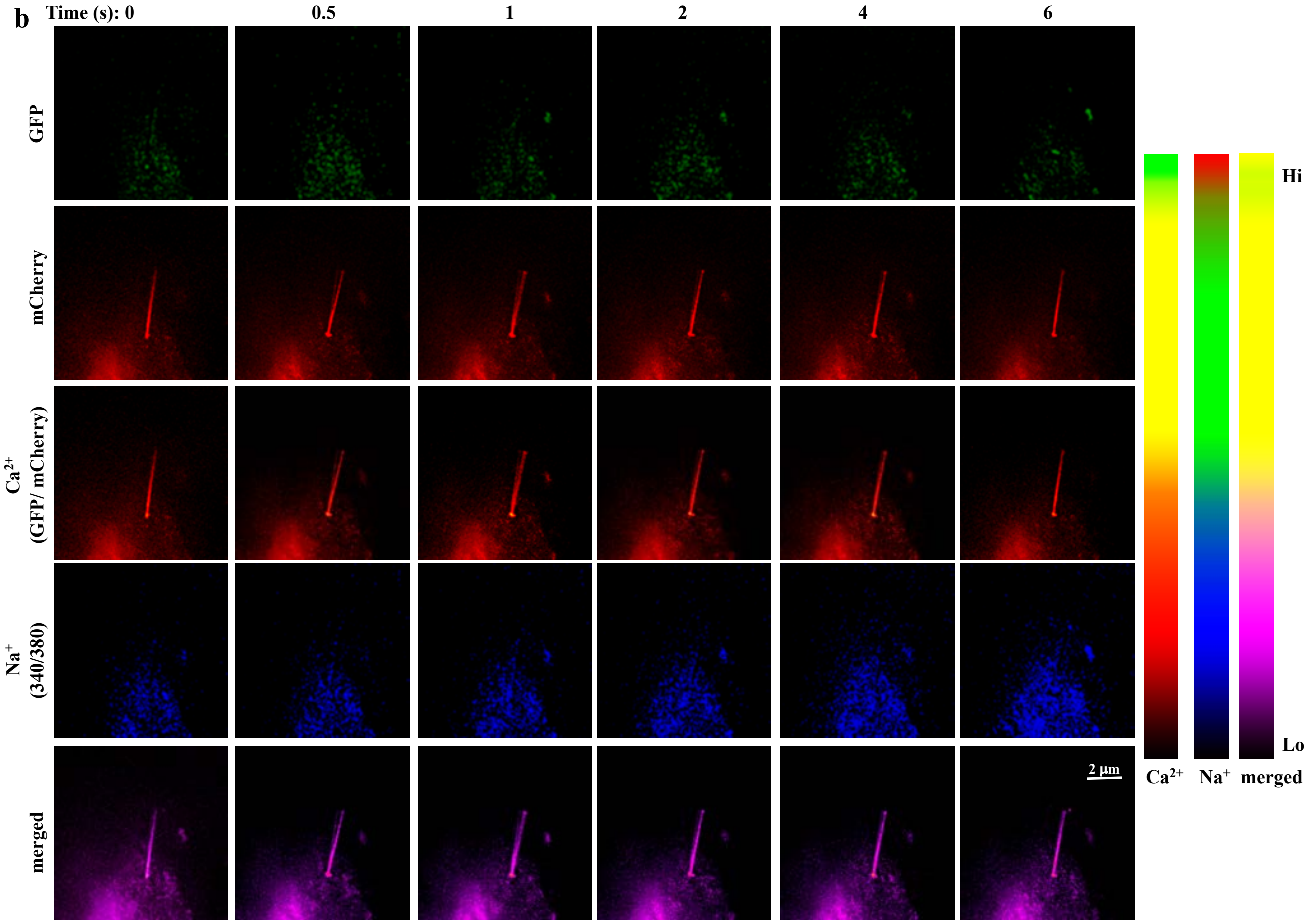
b
Time (s): 0
0.5
1
2
4
6
GFP
mCherry
Ca2+ (GFP/ mCherry)
Na+ (340/380)
merged
2 μm
Hi
Lo
Ca2+
Na+
merged

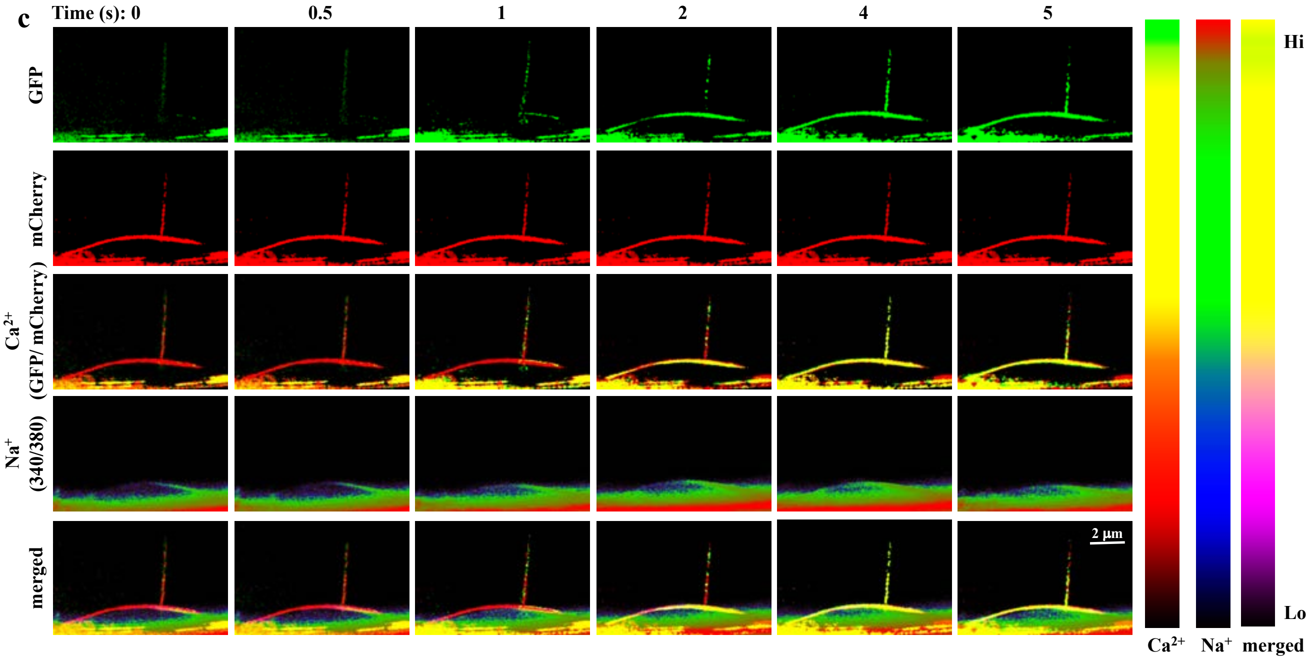

Figure S4. The cELV increases intracellular calcium and sodium levels.

Simultaneous measurement of intracellular calcium (Ca^{2+}) and sodium (Na^{+}) levels in a single cell with a cilium allowed differential analysis of cilioplasmic and cytoplasmic signals. A single cell transfected with a Ca^{2+} fluorescence reporter (5HT6-mCherry-G-GECO1.0) was loaded with ratiometric Na^{+} SBFI-AM dye. The GFP/mCherry ratio depicted the intracellular Ca^{2+} level, whereas the 340/380 ratio represented the Na^{+} level. Colored bars show the respective Ca^{2+}, Na^{+} and merged Ca^{2+} and Na^{+} levels. **a,** A cell was treated with cELV (5 X 10^{6}/µL). A set of GFP, mCherry, 340 nm and 380 nm images were captured at 32 fps. The Ca^{2+} and Na^{+} movies in response to cELV are available in **Movie S6**. **b,** A cell was treated with EV (5 X 10^{6}/µL). A set of GFP, mCherry, 340 nm and 380 nm images were captured at 32 fps. The Ca^{2+} and Na^{+} movies in response to EV are available in **Movie S6**. **c,** Ionomycin (1 µM) was used in a cell from a different preparation. A set of GFP, mCherry, 340 nm and 380 nm images were captured at 19 fps. The Na^{+} and Ca^{2+} movies in response to ionomycin are available in **Movie S6**. Time is shown in seconds (s).

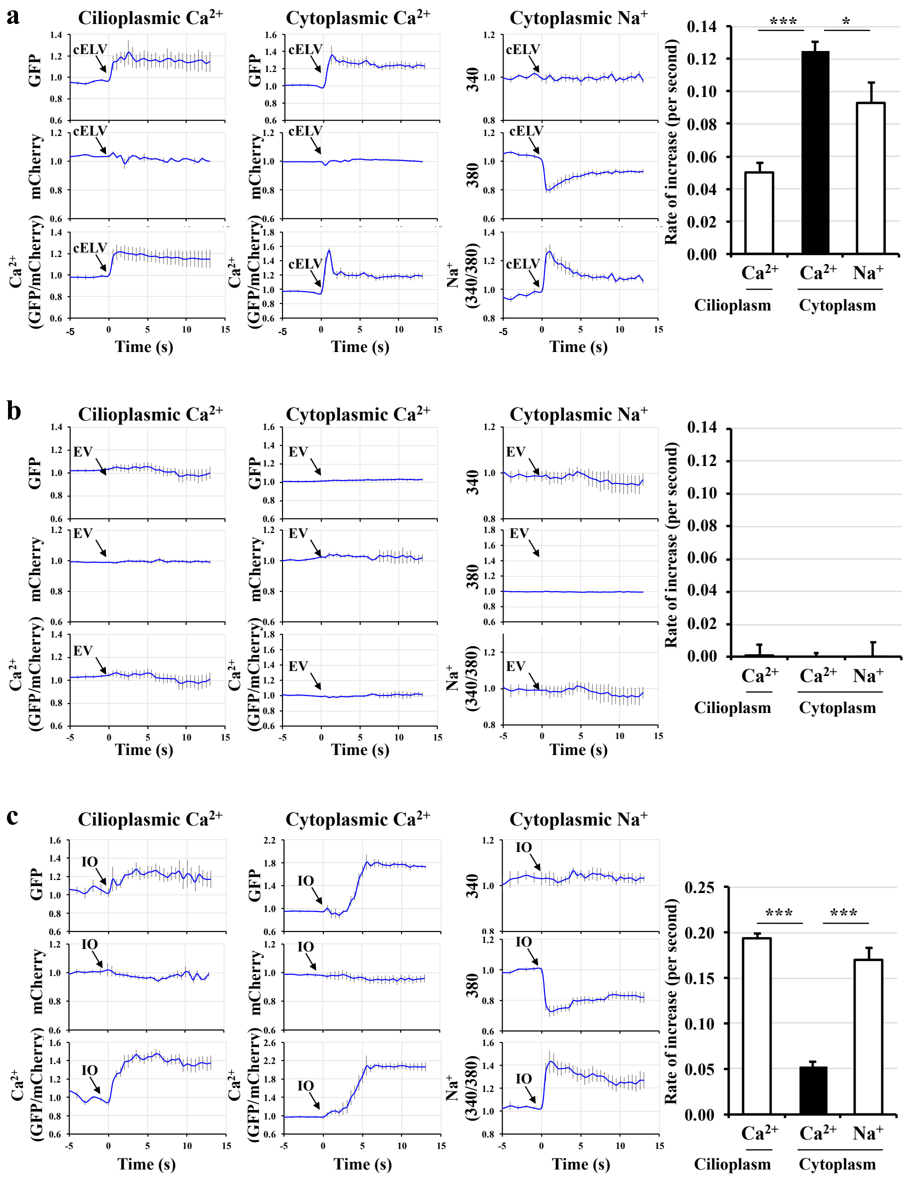

Figure S5. Isolated cELV triggers cellular sodium and calcium signaling in the cilioplasm and cytoplasm.

Simultaneous measurement of intracellular calcium (Ca^{2+}) and sodium (Na^{+}) levels in a single cell with a cilium allowed us to conduct a differential analysis of the cilioplasmic and cytoplasmic signals. A single cell transfected with a Ca^{2+} fluorescence reporter (5HT6-mCherry-G-GECO1.0) was loaded with ratiometric Na^{+} SBFI-AM dye. A set of GFP, mCherry, 340 nm and 380 nm images were captured at 19 fps. Complete Ca^{2+} and Na^{+} figures and movies for cELV (5×10^{6}/μL), EV (5×10^{6}/μL) or ionomycin (control; 1 μM) treatment are available in **Figure S4** and **Movie S6**. **a-c,** The averaged intracellular traces for each wavelength (490 nm for GFP, 590 nm for mCherry, 340 nm and 380 nm) and wavelength ratio (GFP/mCherry for Ca^{2+} and 340/380 for Na^{+}) are shown in response to cELV (ciliary extracellular-like vesicle, **a**), EV (extracellular vesicle, **b**) or IO (ionomycin, **c**). The rates of cilioplasmic and cytoplasmic Ca^{2+} or Na^{+} increase are shown in the bar graphs. N=6 in each group. Arrows indicate the addition of cELV, EV or ionomycin (IO). *, $p<0.05$; ***, $p<0.001$.

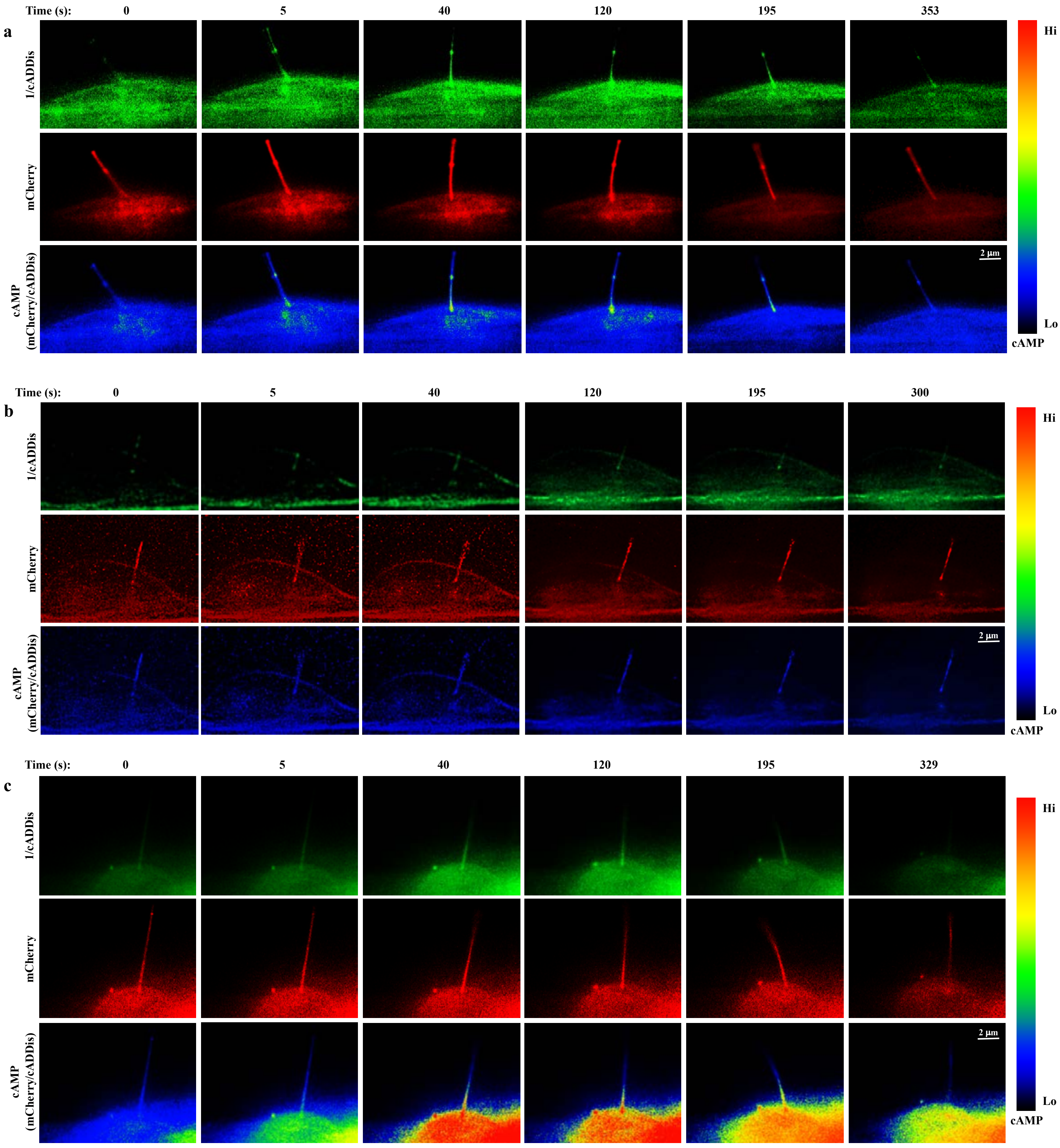

Time (s): 0 5 40 120 195 353
a
1/cADDis
mCherry
cAMP (mCherry/cADDis)
2 µm
Hi
Lo
cAMP
Time (s): 0 5 40 120 195 300
b
1/cADDis
mCherry
cAMP (mCherry/cADDis)
2 µm
Hi
Lo
cAMP
Time (s): 0 5 40 120 195 329
c
1/cADDis
mCherry
cAMP (mCherry/cADDis)
2 µm
Hi
Lo
cAMP

Figure S6. The cELV increases cilioplasmic and cytoplasmic cAMP levels.

A single cell transfected with a downward cAMP fluorescence reporter (5HT6-mCherry-cADDis) was used to allow for the differential analysis of cilioplasmic and cytoplasmic cAMP signals. A set of mCherry and cADDis images were captured at 48 frames per minute. The mCherry/cADDis ratio depicts the intracellular cAMP level. The colored bar shows the cAMP level. **a,** The response of a single cell after cELV treatment ($5\times10^6/\mu L$; **Movie S8**). **b,** The response of a single cell after EV treatment ($5\times10^6/\mu L$; **Movie S7**). **c,** The response of a single cell to forskolin (5 μM; **Movie S7**). Time is shown in seconds (s).

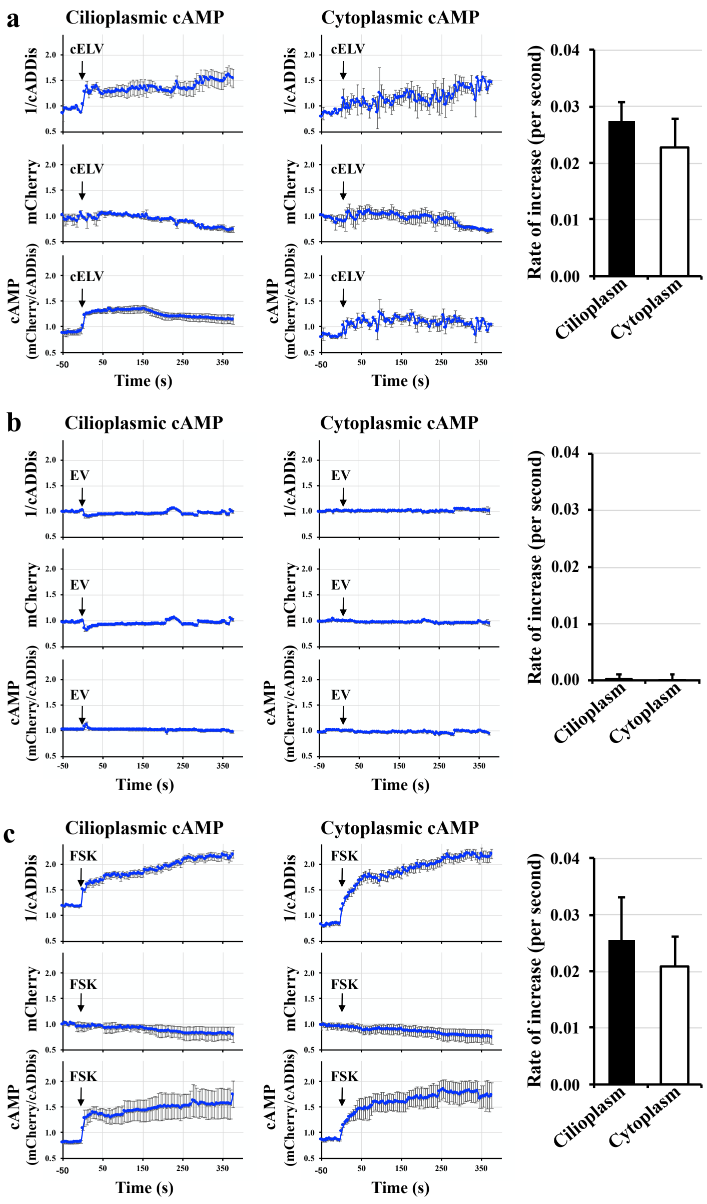

Figure S7. Isolated cELV triggers cAMP signaling in the cilioplasm and cytoplasm.

A single cell transfected with a downward cAMP fluorescence reporter (5HT6-mCherry-cADDis) was used to allow for the differential analysis of cAMP cilioplasmic and cytoplasmic signals. A set of mCherry and 1/cADDis images were captured at 48 frames per minute. The mCherry/cADDis ratio depicts the intracellular cAMP level. Complete cAMP figures and movies for cELV ($5\times10^6/\mu L$), EV ($5\times10^6/\mu L$) or forskolin (control; 5 μM) treatment are available in **Figure S6** and **Movie S7**. **a-c,** The averaged intracellular cAMP traces for each wavelength (590 nm for mCherry and 490 nm for cADDis) and wavelength ratio (mCherry/cADDis for intracellular cAMP) are shown in response to cELV (ciliary extracellular-like vesicle, **a**), EV (extracellular vesicle, **b**) or FSK (forskolin, **c**). Arrows indicate the addition of cELV, EV or FSK. The rates of cilioplasmic and cytoplasmic cAMP increase are shown in the bar graphs. N=3 in each group.

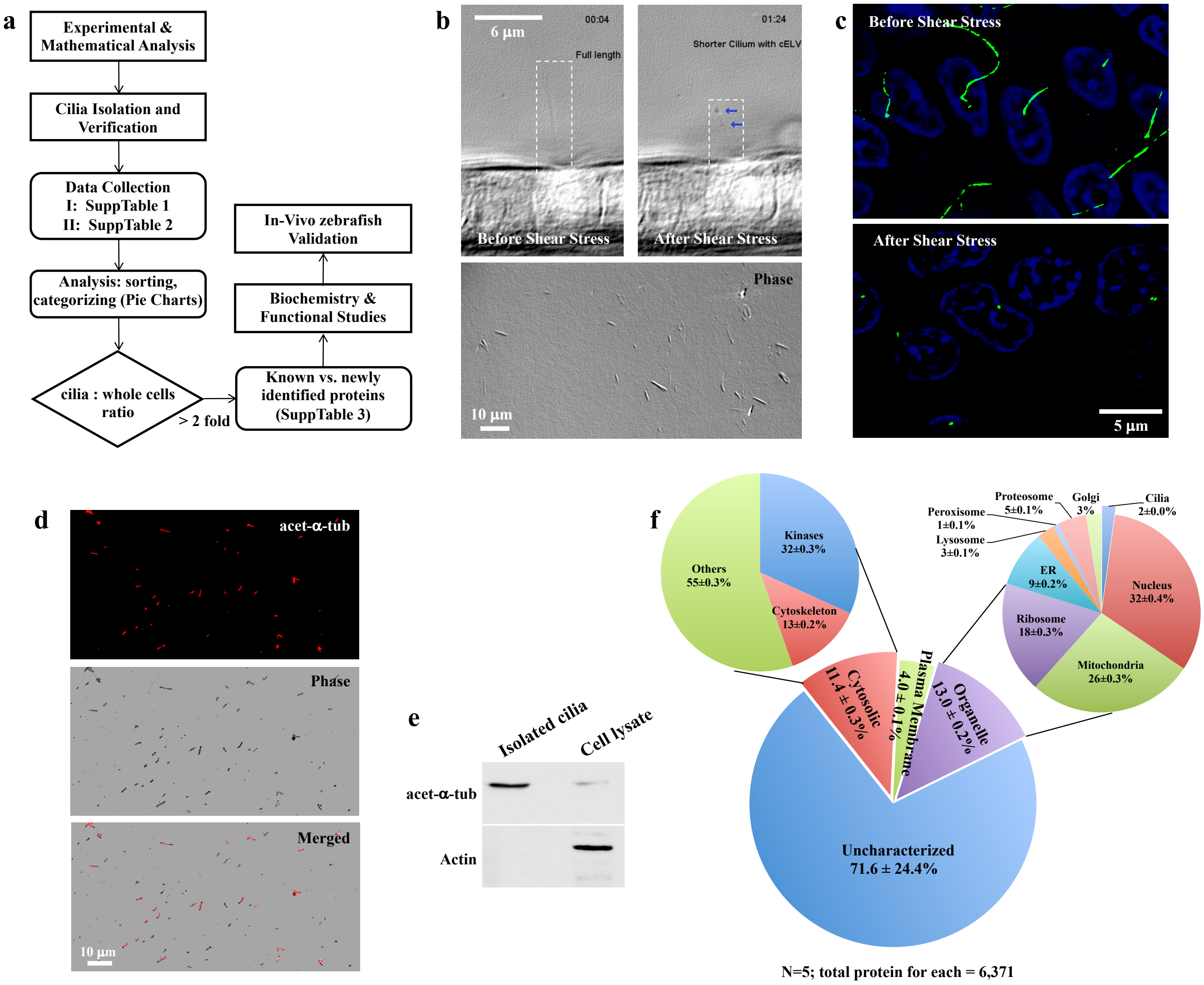

Figure S8. Isolation and identification of cELV proteins in cilia.

a, A schematic flowchart of the experimental design was used to deduce proteomic data for the *in vitro* and *in vivo* studies. **b,** Isolation of cilia containing cELV by mechanical force was observed in live cells without fluorescent-tagged protein (**Movie 8**). The lower panel shows lysate from the collected perfusate. **c,** The immunostaining depicts the length of the cilia before (upper panel) and after (lower panel) the application of mechanical force. **d,** Phase contrast and fluorescent, acetylated-α-tubulin (green) images show isolated primary cilia subject to mechanical force. Acetylated-α-tubulin (green) and DAPI (blue) were used as ciliary and nuclear markers, respectively. **e,** Cilia and cell lysates were immunoblotted with both negative (actin) and positive (acetylated-α-tubulin) controls to confirm the purity of the cilia fractions (N=6). **f,** Based on the standard approach for MudPIT separation, a pie chart summarizes the comparative proteomic analysis between isolated primary cilia (with an cELV-induced appearance) and whole cell lysates, indicating that 2% of the total organelle protein were localized within the cilia (and cELV; N=5).

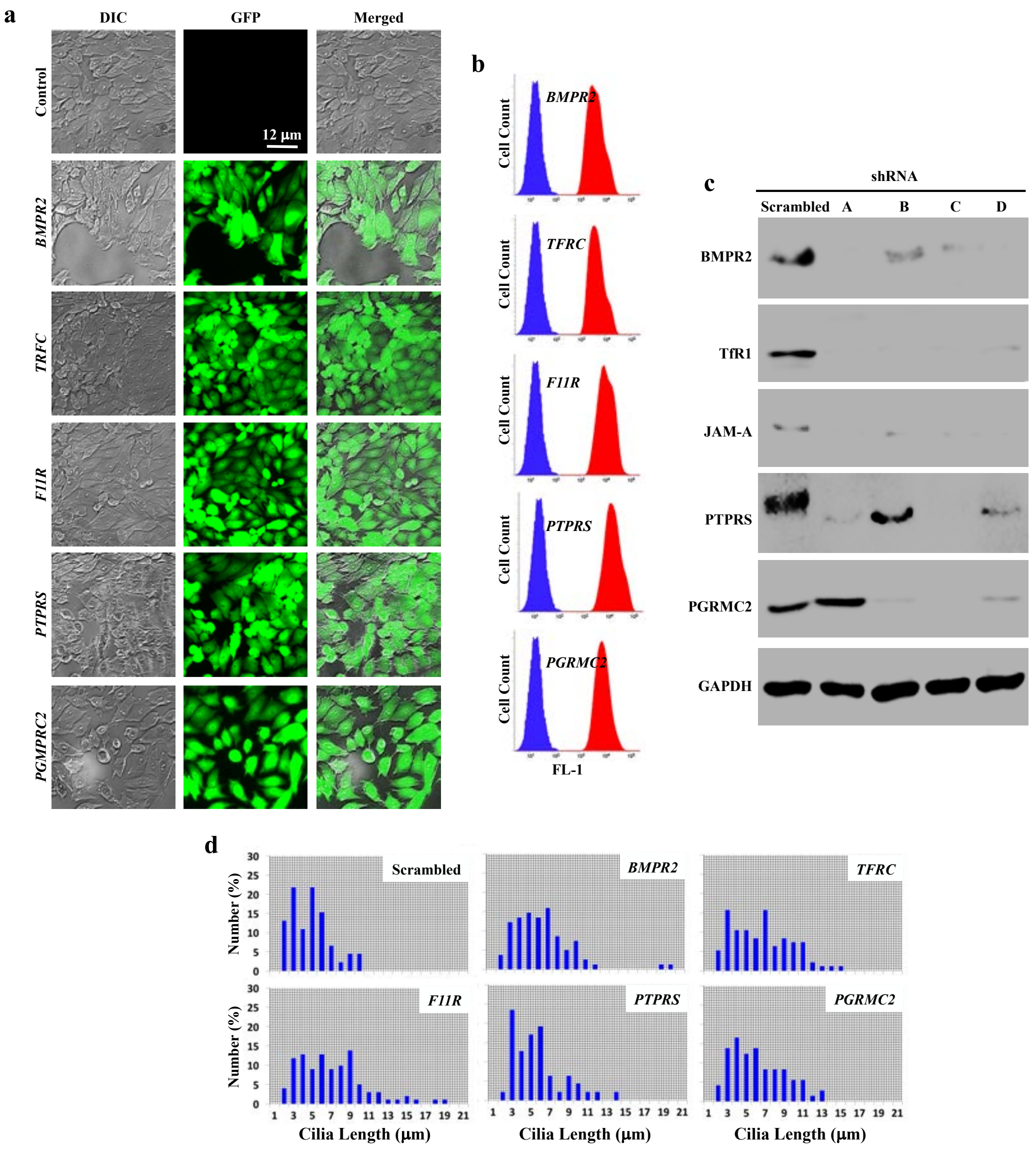

Figure S9. Establishing stable knockdown cells of selected cELV proteins.

a, Representative fluorescence images are shown for cells infected with lentivirus to knockdown a specific gene, as indicated in the figure. **b,** Infection efficiency was also verified by flow cytometry. Stable cells with a scrambled sequence that was not GFP-tagged was used as a control (blue), and stable cell lines with a specific sequence had a GFP marker (red). **c,** For each selected gene, four different sequences targeting shRNAs (A, B, C and D) were used to verify the knockdown efficiency by Western blot. GAPDH was used as a loading control. **d,** The effect of the cELV proteins on ciliary length distributions is shown in the histogram.

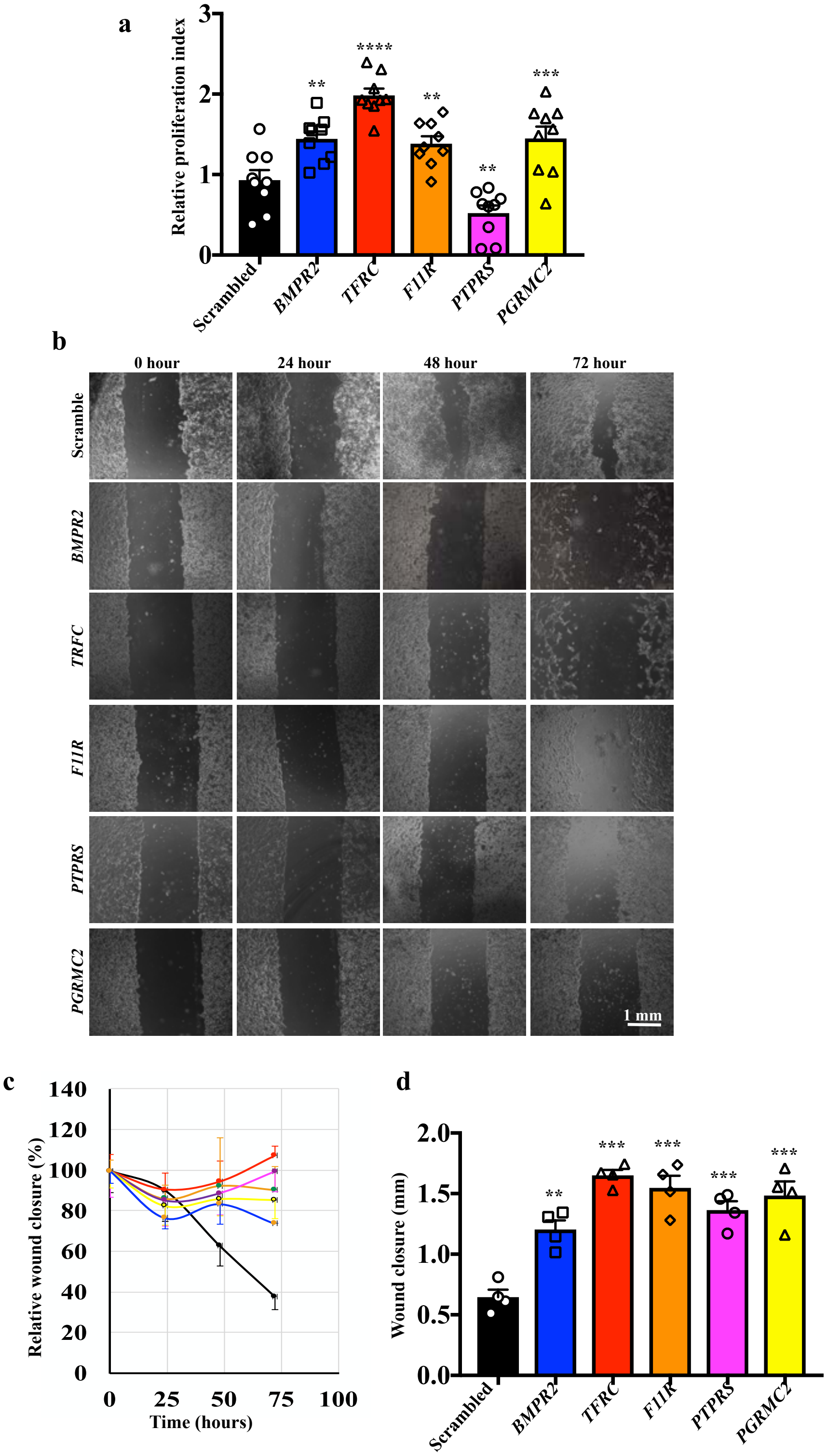

Figure S10. Selected cELV proteins are involved in cellular proliferation and cell migration.

a, The colorimetric MTT assay was used to measure proliferation in all stable knockdown cells. N=9 with triplicates. **b**, Representative phase contrast images taken every 24 hours show that the cELV played important roles in wound closure. Compared with the control cells, all the knockdown cells exhibited a significant impairment in migration. **c**, The cELV proteins played important roles in cell migration or wound closure over time. **d,** The average of cell migration in all stable knockdown cells were measured and quantified. N=4 with triplicates for the cell migration (wound healing) assay. **, p<0.01; ***, p<0.001; ****, p<0.0001 compared with the scrambled control.

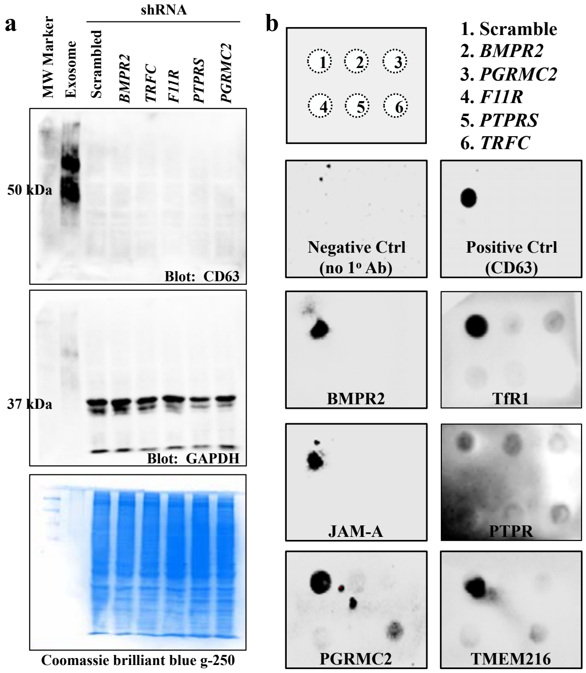

Figure S11. **Analyses of cELV marker expression.**

a, The expression of CD63 (cELV marker) was analyzed in the isolated control cELV and cell lysates from stable knockdown cell lines. CD63, at approximately 50 kDa, was observed only in the isolated cELV sample, indicating that CD63 from cell lysates might be too diluted for detection. A GAPDH blot and Coomassie blue staining were used to confirm equal loading and the presence of other proteins in the cell lysates. **b**, cELV from knockdown cell lines were collected and analyzed with dot blots. A CD63 blot and blot without primary antibody (1° Ab) were used as positive and negative controls (ctrl), respectively. This suggests the absence of cELV in knockdown cells.

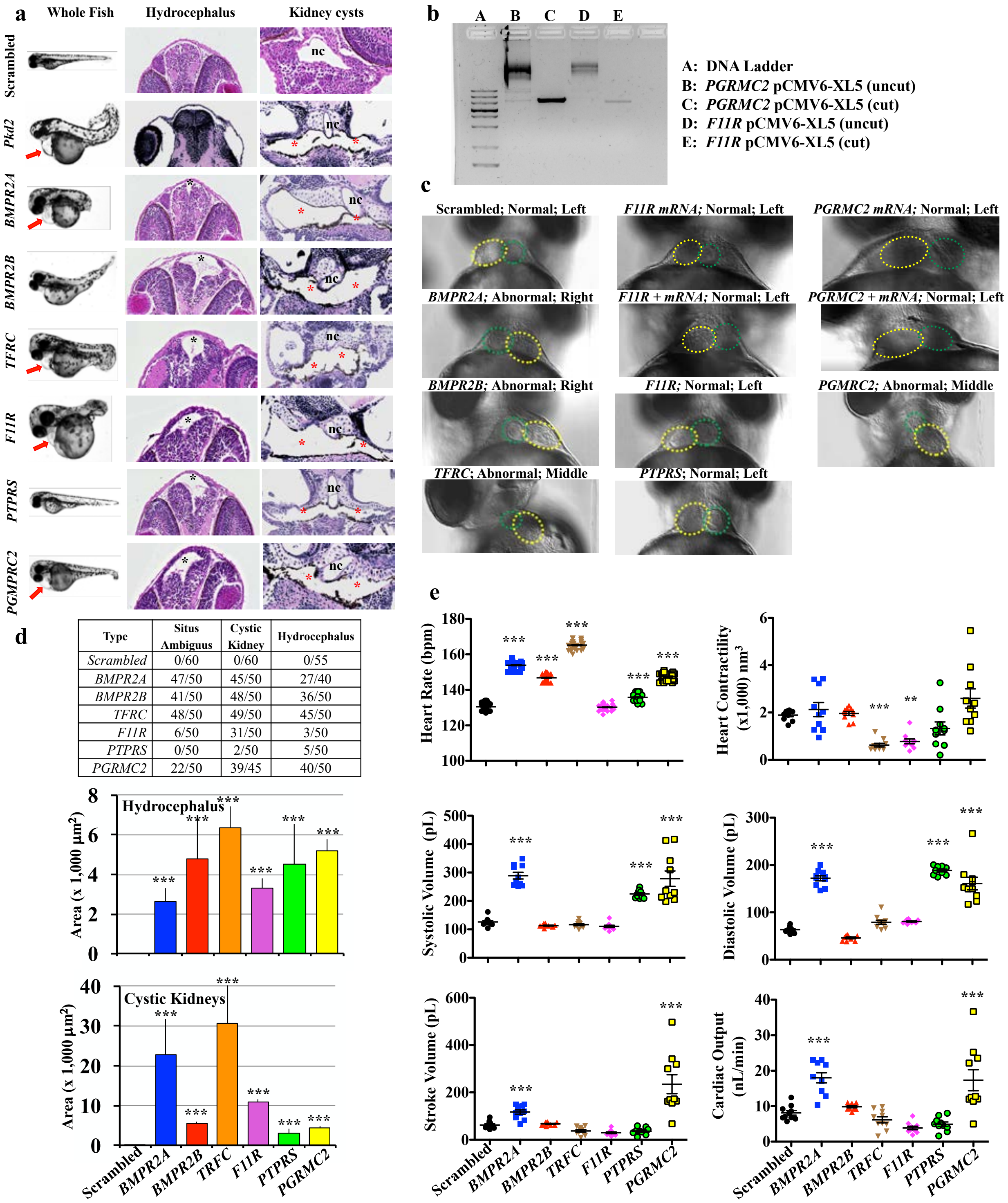

Type	Situs Ambiguus	Cystic Kidney	Hydrocephalus
Scrambled	0/60	0/60	0/55
BMPR2A	47/50	45/50	27/40
BMPR2B	41/50	48/50	36/50
TFRC	48/50	49/50	45/50
F11R	6/50	31/50	3/50
PTPRS	0/50	2/50	5/50
PGRMC2	22/50	39/45	40/50

Figure S12. Knockdown of cELV proteins results in syndromic ciliopathy phenotypes in zebrafish.

a, Representative phase contrast and H&E images showing zebrafish at 72 hours post-fertilization. Fish were injected with either scrambled or specific morpholinos. Red arrows indicate the pericardial edema. Black and red asterisks indicate hydrocephalus and renal cysts, respectively. nc=notochord. **b**, Plasmids were purchased from Origene and linearized with Sac1 restriction enzyme. PGRMC2 plasmid (SC111151: ~7.6 kb total; ~2.9 kb insert); F11R plasmid (SC114147: ~8.4 kb total; ~3.7 kb insert). **c,** Randomized heart looping was observed (**Movie S9**). Yellow- and green-dotted circles show ventricle and bulbus arteriosus, respectively. **d,** A quantitative analysis of asymmetry, kidney cysts and hydrocephalus depict the frequency and severity of the phenotypes. **e,** The parameters of heart function were quantified (**Movie S11**). N=40-60 fish per group. *, $p<0.05$; **, $p<0.01$; ***, $p<0.001$; ****, $p<0.0001$ compared with the scrambled control.

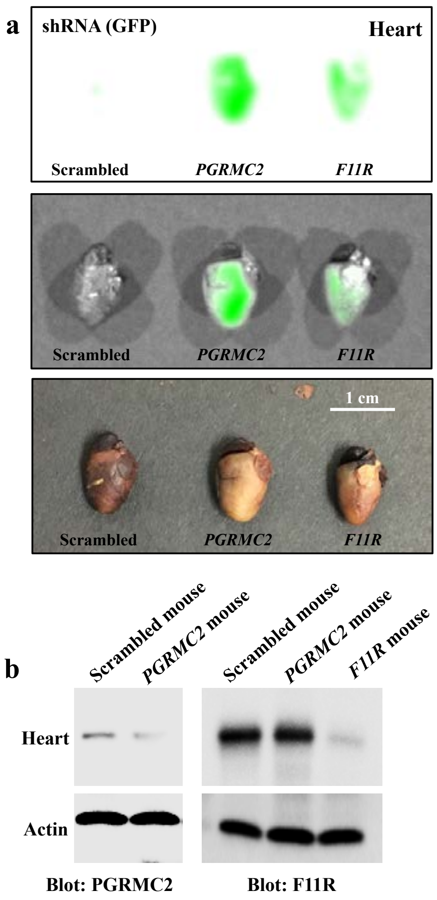

Figure S13. **Confirmation of *PGRMC2* and *F11R*-targeted lentivirus carrying GFP marker in mice.**

(**a**) Isolated heart organ confirmed the virus infection, as evidenced by the lentivirus-GFP marker, immediately after the mice were sacrificed. (**b**) Expressions of PGRMC2 and F11R in heart organ were analyzed.

Supplement Movie Legends

Movie S1. The cELV movement in a non-bending cilium.
A representative movie showing DIC live imaging of a single cell with a cELV (left panel), computer-masked of cELV movements (middle panel) and the use of the tracking system module to study the behavior of the cELV (right panel). The green arrow on the left panel indicates the movement of the cELV along a ciliary shaft. The black arrow on the left panel represents the direction of fluid flow at a magnitude of 0.3 dyn/cm^2 of shear stress from 0:55 to 1:47 (in minutes: seconds). The video was taken at 0.8 fps with video playback at 35X real time.

Movie S2. The cELV movement in a bending cilium.
A representative movie showing DIC live imaging of a single cell with the cELV (left panel), computer-masked of the cELV movement (middle panel) and the use of the tracking system module to study the behavior of the cELV (right panel). The green arrow on the left panel indicates the movement of the cELV along a ciliary shaft. The black arrow on the left panel represents the direction of fluid flow at a magnitude of 1.0 dyn/cm^2 of shear stress from 0:33 to 1:31 (in minutes: seconds). The video was taken at 0.8 fps with video playback at 37X real time.

Movie S3. A reconstructed 3D image is used to quantify cilia length.
Depicted is a cilium projecting from a single cell with a nucleus (DAPI; right), a cilium (green; middle) and merged cell organelles (blue/green; left). This side view imaging technique was used to more precisely quantify cilia length.

Movie S4. The cELV containing CD63-GFP is released from the primary cilium.
This video contains three independent movies (A, B and C). **Movie A** shows CD63-GFP (Part 1; fluorescence only) and its localization in the cELV (Part 2; fluorescence and phase contrast). Released CD63-GFP-tagged cELV can be observed in the background as indicated by the blue arrows in Part 1 and black arrows in Part 2. CD63-GFP on a cilium is indicated by a white arrow. **Movie B** shows that the release of three cELV (arrows) from a cilium was induced by fluid shear stress (5 dyn/cm^2). **Movie C** shows another example of the cELV (arrow) released from a cilium induced by shear stress (5 dyn/cm^2). In this case, the movie speed is slowed down to provide better visualization of the cELV release (9:52 to 9:57). Time is in minutes:seconds. The video was taken at 1 fps with playback at 29X real time and it was slowed down to 0.1X real time in some parts of Movie C to observe the release of the cELV from the cilium.

Movie S5. The cELV induces intracellular calcium and sodium signaling in a monolayer of cells.
This video contains two independent movies (A and B). **Movie A** shows the response of cells to cELV ($5\times10^6/\mu L$) and **Movie B** shows the response of cells to ionomycin (positive control; 1 μM). The cELV or ionomycin was added to the cells at 0 seconds. From two different preparations, the intracellular calcium (left panel) and sodium (right panel) were measured with Fura2-AM and SBFI-AM ratiometric dyes, respectively. Time is in seconds and the video was taken at 32 fps with playback at 1X real time.

Movie S6. The cELV induces intracellular calcium and sodium signaling in a single-ciliated cell.
This video contains three independent movies (A, B and C). **Movie A** shows the response of a cell to cELV ($5\times10^6/\mu L$), **Movie B** shows the response of a cell to EV ($5\times10^6/\mu L$) and **Movie C** shows the response of a cell to ionomycin (positive control; 1 μM). The cELV, EV or ionomycin was added at 0 seconds. From the same preparation, simultaneous measurement of intracellular Ca^{2+} and Na^{+} levels in a single cell with a cilium allowed for the differential analysis of cilioplasmic and cytoplasmic signals. A single cell transfected with a

Ca^{2+} fluorescence reporter (5HT6-mCherry-G-GECO1.0) was loaded with a ratiometric Na^+ SBFI-AM dye. Time is in seconds (sec) and the video was taken at 19 fps with playback at 1X real time.

Movie S7. The cELV induces intracellular cAMP signaling.
This video contains three independent movies (A, B and C). **Movie A** shows the response of a cell to cELV (5 X 10^6/μL), **Movie B** shows the response of a cell to EV (5×10^6/μL) and **Movie C** shows the response of a cell to forskolin (positive control; 5 μM). The cELV, EV or forskolin was added at 0 seconds. A single cell transfected with a cAMP fluorescence reporter (5HT6-mCherry-cADDis) was used to allow for the differential analysis of cAMP cilioplasmic and cytoplasmic signals. A set of mCherry, cADDis and ratio (mCherry/cADDis) movies is shown. The colored bar shows the cAMP level. Time is in seconds (sec) and the video was taken at 0.8 fps with playback at 36X real time.

Movie S8. The induction and release of cELV in response to shear force.
The movie shows the response of a cilium to shear stress. Multiple cELV were induced during the shear force of (1 dyn/cm^2), and part of the cilium with cELV was released into the perfusate for later analysis. The blue arrow indicates the induction and movements of multiple cELV along the ciliary shaft. The black arrow represents the direction of fluid flow at a magnitude of 1 dyn/cm^2 of shear stress from 00:05 to 1:03 (in minutes: seconds). The video was taken at 0.9 fps with playback at 49X real time.

Movie S9. The randomized heart position is seen in knockdown zebrafish.
A cartoon at the beginning of the video depicts the orientation of the heart ventricle (yellow) and bulbus arteriosus (green) as seen in the corresponding fish. Red arrows indicate the direction of blood flow. An abnormal heart position and direction of blood flow can be observed in all fish except for *F11R* and *PTPRS* knockdown fish. Time is in seconds (sec) and the video was taken at 36 fps with playback at 1X real time.

Movie S10. The randomized heart position is seen in knockdown and rescued zebrafish.
A cartoon at the beginning of the video depicts the orientation of the heart ventricle (yellow) and bulbus arteriosus (green) as seen in the corresponding fish. Red arrows indicate the direction of blood flow. An abnormal heart position and direction of blood flow can be observed in all fish except for *PGRMC2* knockdown fish, but not in rescued or mRNA or scrambled control fish. Time is in seconds (sec) and the video was taken at 36 fps with playback at 1X real time.

Movie S11. The effect of gene knockdown on heart rate is examined and quantified.
Heart rate was accurately measured using image scanning and segmentation to compute the overall heart beat (Y-axis) over a 21-second period (X-axis). The actual fish being measured is shown on the right side. The video corresponding to the heart beating was taken at 36 fps with playback at 1X real time.

Movie S12. The effect of gene knockdown and rescue on heart rate is examined and quantified.
Heart rate was accurately measured using image scanning and segmentation to compute the overall heart beat (Y-axis) over a 21-second period (X-axis). The actual fish being measured is shown on the right side. The video corresponding to the heart beating was taken at 36 fps with playback at 1X real time.

Movie S13. Kidney and cyst volumes are measured using constructed 3D CT scan images.
A renal parenchyma scan (top panel) and a renal surface scan (bottom panel) were used to evaluate the relative size of the cyst to the kidney and to analyze the renal cortical surface roughness, respectively, in 9-week-old mice. The kidney-specific contrast agent OptiPrep was intravenously injected into the tails of the mice. Mice were imaged with the IVIS Spectrum CT imaging system.

Movie S14. The working heart system is used to measure overall heart function.

This video was shot through a Plexiglas acrylic chamber in which an isolated heart was kept viable for as long as possible by maintaining a constant temperature via heat exchangers in and around the chamber. The temperature, pH and pO_2 were independently maintained in the perfusion buffer. The perfusion heads, electrocardiogram detector, transonic flow meter and pressure electrode are shown in the video.

Supplement Methods

Cell culture

The LL-CPK1 control and *Tg737* cells were cultured in Dulbecco's Modified Eagle Medium (DMEM) (*Corning Cellgro*), 10% fetal bovine serum (FBS) (*HyClone*) and 1% penicillin/streptomycin (*Corning Cellgro*) at 37°C in a 5% CO_2 incubator. Prior to all experiments, cells at 70-80% confluence were differentiated for 24-48 hours in serum-free and EV-free media so we could accurately quantify and study the effects of cELV in each experiment.

To examine the relationship between cELV and the intraflagellar transport system, cells were incubated with 50-100 μM BTB-1 (*Tocris*), a selective and ATP-competitive kinesin inhibitor, and 40-100 μM Ciliobrevin D (*Millipore*), a dynein inhibitor[1-3]. To inhibit cELV formation, cells were treated with 20 μM GW4869 (*Santa Cruz*) and 10 μM N-SMase Spiroepoxide inhibitor (*Santa Cruz*)[4-6]. In some cases, these inhibitors were applied to cells after differentiation. In other cases, the effects of these inhibitors were tested on dividing cells (prior to cell differentiation).

Isolation of primary cilia and cELV

The isolation of the primary cilia was induced by mechanical force, as previously described[7, 8]. After differentiation, the cells were rinsed briefly and gently with 10 ml of phosphate-buffered saline (PBS; pH 7.4). A 150-mm culture dish was placed on an orbital shaker (*Cole-Parmer*) for 4 minutes at 350 rotations per minute, resulting in a shear stress of 10 dyn/cm^2. The media containing the isolated cilia was carefully transferred to a 50 ml centrifuge tube and centrifuged for 30 minutes at 3,000×*g* at 4°C (*Eppendorf 5810R*). The supernatant containing the isolated cilia was then transferred to a polyallomer tube and centrifuged for 1 hour at 70,000×*g* at 4°C in an ultracentrifuge (*Beckman optima L-60*). The isolated primary cilia (pellet or cELV) were then re-suspended in radioimmunoprecipitation assay (RIPA) buffer for protein quantification followed by Western blot or proteomic analyses.

For isolation of vesicles (including cELV), media from cells grown in 150 mm culture dishes was collected for analyses. In some cases, the cells were perfused with a fluid shear stress of 1 dyn/cm^2 for 30 minutes. The perfusate media was then collected. The collected media was centrifuged at four different speeds: 300×*g* for 10 minutes, 2,000×*g* for 10 minutes, 10,000×*g* for 30 minutes and 100,000×*g* for 70 minutes. In the first three rounds of centrifugation, the supernatant was collected, in the last round of centrifugation, the pellet containing the vesicles was collected. A final wash of the vesicles pellet was done with centrifugation at 100,000×*g* for 70 minutes, as previously described[9]. The cELV were then suspended in either PBS or RIPA buffer. A quantitation kit was used to determine the concentration of the resuspended cELV solution (*System Bioscience*, FCET96-1).

Visualization of isolated primary cilia and cELV

A nitrogen smearing technique was used to visualize and verify the efficiency of isolated primary cilia and cELV. Right after the isolation process, isolated cilia were fixed in 10 µl of 2% paraformaldehyde, transferred, and spread equally on a glass slide. The coverslip was placed on top of the glass slide. The glass slide was then placed in liquid nitrogen for approximately 1-2 minutes. The coverslip was then peeled off from the glass slide using a razor blade and washed three times with PBS for 5 minutes each. The glass slide was air-dried and stored at -80°C. Images were taken using a Nikon TE2000.

Scanning electron microscopy

Isolated cELV were fixed in 2% glutaraldehyde for overnight at 4°C. Samples were then washed three times with PBS, dehydrated through a graded series of ethanol for 10 minutes each, and incubated in hexamethyldisilazane for 1 hour on ice. Samples were then mounted, air dried, sputter-coated, and examined with a Tescan GAIA3 microscope (Tescan).

Multidimensional protein identification technology (MudPIT)

Two approaches were used in this study. We refer to the first approach as the microanalysis MudPIT, which is more sensitive but fewer proteins would result from the analysis. Proteins were reduced with 5 mM tris(2-carboxyethyl)-phosphine hydrochloride (Sigma-Aldrich, St. Louis, MO, product C4706) and alkylated. Proteins were digested for 18 hours at 37°C in 2 M urea, 100 mM Tris pH 8.5, 1 mM $CaCl_2$ and 2 μg of trypsin (Promega, Madison, WI, product V5111). Analysis was performed using an Agilent 1100 quaternary pump and a Thermo LTQ Orbitrap Velos with an in-house built electrospray stage[10]. Protein and peptide identification and protein quantitation were done with the Integrated Proteomics Pipeline - IP2 (Integrated Proteomics Applications, Inc., San Diego, CA. http://www.integratedproteomics.com/). Tandem mass spectra were extracted from raw files using RawExtract 1.9.9[11] and were searched against Uniprot Sus scrofa database with reversed sequences using ProLuCID[12, 13]. The search space included all fully tryptic and half-tryptic peptide candidates for the tryptic digest with static modification of 57.02146 on cysteine. Peptide candidates were filtered using DTASelect, with -p 1 -y 1 --trypstat --pfp .01 --extra --pI -DM 10 --DB --dm –in as the parameters[11, 14].

The second approach is a more standard MudPIT analysis, which provided us with a bigger list of identified proteins. Proteins were reduced with 5 mM tris(2-carboxyethyl)-phosphine hydrochloride (Sigma-Aldrich, St. Louis, MO, product C4706) and alkylated. Proteins were digested for 18 hours at 37°C in 2 M urea, 100 mM Tris pH 8.5, 1 mM $CaCl_2$ and 2 μg of trypsin (Promega, Madison, WI, product V5111). Analysis was performed using an Agilent 1100 quaternary pump and a Thermo LTQ Orbitrap Velos with an in-house built electrospray stage[10]. Protein and peptide identification and protein quantitation were done with the Integrated Proteomics Pipeline - IP2 (Integrated Proteomics Applications, Inc., San Diego, CA. http://www.integratedproteomics.com/). Tandem mass spectra were extracted from raw files using RawExtract 1.9.9[11] and were searched against Uniprot Sus scrofa database with reversed sequences using ProLuCID[12, 13]. The search space included all fully tryptic and half-tryptic peptide candidates for the tryptic digest with static

modification of 57.02146 on cysteine. Peptide candidates were filtered using DTASelect, with -p 1 -y 1 --trypstat --pfp .01 --extra --pI --DB --dm –in as the parameters[11, 14].

Each separation was performed and clustered independently and two ciliary protein databases were generated by the MudPIT method. In addition, all proteins obtained from the different separations were categorized in terms of types and percentage of enrichment. In distinguishing between ciliary proteins and non-ciliary proteins, we analyzed protein abundance with the highest cilia-to-cell ratio. The cilia-to-cell ratio was determined by the spectral count for proteins that had a value equal to or greater than two.

Immunocytochemistry

Cells were immunostained using a standard protocol[8]. Briefly, cells were rinsed with sodium cacodylate buffer, fixed with 3% glutaraldehyde in 0.2 M sodium cacodylate buffer for 10 minutes, and permeabilized with 1% Triton-X in sodium cacodylate for 5 min. All primary antibodies were diluted in a 10% FBS solution where antibodies against bone morphogenetic protein type II receptor (BMPR2; *Biorbyt*), progesterone receptor membrane component 2 (PGRMC2; *Abnova*), junctional adhesion molecule-A (JAM-A; *Bioss*), receptor-type tyrosine-protein phosphatase sigma (PTPRs; *Santa Cruz*), transferrin receptor protein-1 (TfR1; *Abcam*), actin (*Sigma*), and acetylated-α-tubulin (*Sigma*) were used at dilutions of 1:200, 1:200, 1:200, 1:200, 1:200, 1:1,000 and 1:1,000, respectively. The FITC fluorescence secondary antibody (1:500; *Pierce*) and Texas Red fluorescence secondary antibody (1:500; *Pierce*) were also diluted in 10% FBS to decrease background fluorescence. Cells were then washed twice with cacodylate buffer and mounted with DAPI (*Vector laboratories*). Images were taken with Nikon Ti-E using Nikon Element software.

Immunoblotting

All cells were rinsed with PBS, incubated with RIPA buffer containing protease inhibitor (*Roche*), placed on ice, scraped from the culture dish, transferred to a microcentrifuge tube, vortexed for 30 minutes and centrifuged for 15 minutes at 10,000×*g* (accuSpin Micro 17, *Fisher scientific*). The lysate was transferred into

another microcentrifuge tube and stored at -80°C. Lysate samples were analyzed using standard 10% gradient sodium dodecyl sulfate-polyacrylamide gel electrophoresis (SDS-PAGE). In some cases, gels were stained with Coomassie blue dye (*Bio-Rad*, blue R-250) for 15-30 minutes to confirm the presence of proteins. Subsequently, gels were washed with de-staining solution (*Bio-Rad*) and imaged with the ChemiDoc XRS+ system (*Bio-Rad*). To detect CD63 expression in all genetically manipulated cell lines, dot blots were performed. Nitrocellulose membrane (*Thermo Fisher Scientific*) was cut into approximately 1 by 1 inch squares and 1-2 μl of each sample was placed onto the membrane to air dry. The membrane was then incubated in primary and secondary antibodies at room temperature for one hour. Transmembrane protein 216 (TMEM216 at dilution 1:500; *Antibody Verify*), glyceraldehyde 3-phosphate dehydrogenase (GAPDH; 1:1,000; *cell signaling*) and CD63 (1:1,000; *Santa Cruz*) were used in addition to the antibodies used for immunocytochemistry.

For protein extraction and immunoblotting analysis for zebrafish, embryos from scrambled MO, *F11R* mRNA, *PGRMC2* mRNA, *F11R* MO, *PGRMC2* MO, *F11R* MO plus *F11R* mRNA and *PGRMC2* MO plus *PGRMC2* mRNA were dechorionated and deyolked between 24-48 hpf and then lysed with RIPA buffer containing protease inhibitor (*Roche*). Purified proteins were then quantified and loaded into SDS-PAGE gels and transferred to nitrocellulose membrane. Primary antibodies were blotted for F11R (1:500; *ABGENT*) and PGRMC2 (1:500; *Cohesion*). GAPDH was used as the loading control (1:1,000; *Abcam*).

Cell migration (wound healing) and MTT assays

Both assays were performed double-blinded, from sample and data collection to data analysis and presentation. Cells were seeded in a six-well plate until they reached 60-80% confluence and then they were treated with PBS or cELV (5×10^6/μL). At 100% confluence, a wound was made in the cell layer and images were captured immediately with a Nikon Ti-E camera. The cells were incubated at 37 °C in a 5% CO_2 incubator and images were captured after 24, 48 and 72 hours. The migration rate was calculated based on the width of the wound over time.

Cell viability was determined by the MTT assay (*Beantown Chemical*). Cells were seeded at a density of 2×10^4 cells/well in 96 well plates. After 24 hours, 20 µl of MTT (5 mg/ml) was added to each well and the plate was incubated for 3-4 hours. Formazan dye was dissolved in 100 µl of dimethyl sulfoxide (DMSO) and added to each well. The reduction of MTT was quantified by measuring the absorbance at 570 nm (SpectraMax M5, *Molecular Devices*).

<u>RNAi knockdown</u>

HEK-293T cells were transfected with four shRNA lentiviral vectors (pGFP-C-shLenti; *Origene*) specific to scramble (pScramble-C-shLenti), *BMPR2* (pBMPR2-GFP-shLenti), *TFRC* (pTFRC-GFP-shLenti), *F11R* (pF11R-GFP-shLenti), *PTPRS* (pPTPRS-GFP-shLenti) and *PGRMC2* (pPGRMC2-GFP-shLenti). After 48 hours, the viral particles were collected and centrifuged at 4,000 rpm, and passed through a 0.45 µm filter. Viral particles containing 8 µg/ml polybrene were added to the cells, according to the manufacturer's guidelines (*Origene*). The cells were then centrifuged at 574×*g* (Allerga X-30R; *Beckman Coulter*) for 30 minutes at 30°C and cultured for up to 72 hours. For each gene, 4 targeting sequences were analyzed (see table below). The knockdown efficiency of each sequence for each gene was confirmed by Western blot and stable knockdown cell lines were obtained through cell sorting (*BD Bioscience*) and selective pressure using puromycin dihydrochloride (*Santa Cruz*). The RNAi sequences that were used in our studies are bolded in the table below.

**Gene**	**Sequence**
Scrambled	**5'-TGACCACCCTGACCTACGGCGTGCAGTGC-3'**
BMPR2	**5'-AGATCCATATCAGCAAGACCTTGGAATAG-3'**<br>5'-GGACTGGCTTATCTTCATACAGAATTACC-3'<br>5'-GACCACTATATCTGAGATGCCATACTCAG-3'<br>5'-ACTGAACCAATGGACTGTGAGGTCAACAA-3'
TFRC	**5'-GCTCGGCAGGTAGATGGTGATAACAGTCA-3'**<br>5'-GTCATCAGGATTGCCTAACATACCTGTGC-3'<br>5'-AGCCGAAGTGGCTGGTCATCTTGTGATTA-3'<br>5'-GTTGGCTCTAGCAACTTGGACTATTCAGG-3'
F11R	**5'-ATCACAGCTTCCTATGAGAACCGAGTTAC-3'**<br>5'-GGAGATTACACTTGTGAGGCACAGAATGG-3'<br>5'-GTCCTTGTCACACTCATTCTCCTCGGAGT-3'<br>5'-AGACAGACCTCATCATTCTTGGTGTGACC-3'
PTPRS	**5'-AACACCTTCTACAACTTCGTGCTGACCAA-3'**<br>5'-GAGATCACCTGGTTCAAGGACTTCTTACC-3'

	5'-AGCCTTCTGGAGGATGAGACATACACTGT-3'<br>5'-AGTTCAAGAACCGCCTGGTCAACATCATG-3'
PGRMC2	**5'-CAAGCAGCATTGAGACTGAAGTTGGAATA-3'**<br>5'-CCAGCCGCTTCTCTGCCTCGTATGAAGAA-3'<br>5'-ATTCAGAAGTCACTACAAGCAGCATTGAG-3'<br>5'-AGTCTGACTCTCACATCTCACTGATGTGG-3'

For rescue experiments, full length of the human *F11R* and *PGRMC2* genes without GFP (pCMV6-XL5 vectors; *Origene*) were linearized using SacI restriction enzyme (Anza, *Thermo Scientific*). Capped mRNAs were then synthesized *in vitro* with T7 RNA polymerase using the mMESSAGE mMACHINE T7 kit (AM 1344; *Thermo Scientific*), as described previously[15]. 1-2 nL of 50 ng/µL purified mRNA were either injected alone or co-injected with *F11R* or *PGRMC2* morpholinos into the 1-2 cell-stage embryos.

Single cell side view imaging

Briefly, a special flexible substratum (formvar; *Electron Microscopy Science*) was dissolved in ethylene dichloride to make a 2% formvar solution. An 18×18 glass slide was then coated with the formvar solution, the glass coverslip was placed in a six-well plate until dry and then coated with 50 µg/ml collagen (on top of the formvar layer). The six-well plate was then placed under ultraviolet light for 30 min to sterilize it. After sterilization, the formvar coverslip was briefly washed with PBS. Cells were then grown on this collagen-coated formvar flexible substratum (FFS). A pair of sharp forceps was used to peel the FFS off the slide and the FFS was gently folded to form a triangle shape where cilia were oriented upward for visualization. After folding, the FFS was placed on a special made glass-bottom plate and immobilized with a glass coverslip as previously described[8]. In some cases, live imaging was performed using calibrated shear stress between 0 and 5 dyn/cm^2. Based on our prior studies, a shear stress of 1 dyn/cm^2 was optimal. In other cases, cells were fixed for immunolocalization studies (please see below).

Calcium, sodium and cAMP imaging

For monolayer cell populations, intracellular measurements were obtained as previously described[16]. Briefly, monolayers of cells were incubated with either 5 µM Fura2-AM or SBFI-AM ratiometric dyes (*TEF Labs*) for

45 minutes at 37°C in a 5% CO_2 incubator. The cells were then washed and incubated in PBS for 20 minutes on the microscope stage to hydrolyze the ester group. To examine Ca^{2+} and Na^{+} signals, cELV ($5\times10^6/\mu L$), EV ($5\times10^6/\mu L$) or ionomycin (positive controls; 1 μM) were added to the cell population. The excitation/emission wavelengths of 340/510 nm and 380/510 nm were recorded for intracellular calcium (Fura2) or sodium (SBFI) measurements at 32 frames per seconds (fps).

In single cell studies, cells were grown on 2% formvar and transfected with the Ca^{2+} fluorescence reporter $5HT_6$-mCherry-G-GECO1.0 (*Addgene*) using the JetPrime transfection reagent (*Polyplus transfection*) as previously described and characterized[17]. For concurrent imaging, successfully transfected cells were incubated in 5 μM SBFI-AM for 45 minutes at 37°C in a 5% CO_2 incubator. The cELV, EV or ionomycin were resuspended in DPBS and pumped in at a rate of 10 μl/sec (InsTech P720) to prevent cilia deflection. Fluorescence signals were examined by focusing only on one cell or cilium. The excitation/emission wavelengths of 340/510 nm and 380/510 nm were recorded for intracellular sodium (SBFI); 590/610 nm and 490/510 nm were recorded for intracellular calcium ($5HT_6$-mCherry-G-GECO1.0). All images were captured at 19 fps.

In single cell studies, cells were grown on 2% formvar and transfected with the cAMP fluorescence reporter 5HT6-mCherry-cADDis (*Montana Molecular*) using the JetPrime transfection reagent. The cAMP reporter has been previously characterized[18]. A cell expressing the cAMP sensor in the cilia was used in our studies. cELV ($5\times10^6/\mu L$), EV ($5\times10^6/\mu L$) or forskolin (10 μM; *BioVision*) was slowly apllied to the cell to prevent cilia bending. Fluorescence readings were taken for mCherry and cADDis at excitation/emission wavelengths of 590/610 nm and 490/510 nm, respectively. A set of mCherry and 1/cADDis images at excitation/emission wavelengths of 590/610 nm and 490/510 nm were captured at 0.8 fps.

Animal models

All animal experiments were performed by two operators who were blinded to the experimental conditions. All animal procedures were performed according to Chapman University Animal Care and Use Committee Guidelines. Wild-type zebrafish AB strains were obtained from the Zebrafish International Resource Center. Embryos were injected with 1 mM morpholino oligos (GeneTools) at the 1-2 cell stage and cultured at 28.5°C in sterile egg water. Embryos were injected with morpholinos at 48 hours post-fertilization and fixed in agarose gel for observation under a microscope. The cardiac looping position was assessed by placing zebrafish on their dorsal axis to examine the relative locations of the ventricle and bulbus arteriosus and blood circulation. Histological examination was used to measure renal cyst formation and hydrocephalus at 3 days post-fertilization. Embryos were fixed in a PBS solution containing 4% paraformaldehyde overnight at 4°C, dehydrated through an ethanol gradient and embedded in wax. A microtome (HM-355S, *Thermo Scientific*) was used to cut 4 µm thick sections which were subsequently stained with standard hematoxylin and eosin (H&E). The morpholino sequences for each gene in zebrafish were interpolated from the knockdown efficiency from the *in vitro* cell studies. The morpholino sequences were used in our studies are bolded in the table below.

**Gene**	**Sequence**
Scrambled	**5'-CCTCTTACCTCAGTTACAATTTATA-3'**
PKD2	**5′-AGGACGAACGCGACTGGGCTCATC-3′**
*BMPR2*a	**5'-CTCTCCTTTTTCTTCAAATACAGTA-3'**<br>5'-GCATTCCAGGCAGAGGTGGGCAAT-3'<br>5'-GAGAACATAGCGCGCTTCCTAGAGA-3'<br>5'-GTACAGTGCGGTACATGGCTCCAGA-3'
*BMPR2*b	**5'-CACTCTCATCCTGCTGCCGCTTCTG-3'**<br>5'-GTGTGGCAGGTGACTGGCACATCAC-3'<br>5'-TTAGTCATTTCGGATTGTTTGCTCCG-3'<br>5'-ATGGAGTACTACCCTCATGGCTCGC-3'
TFRC	**5'-TGGACCATCCAGGGCTGTGCCAATG-3'**<br>5'-GGCGATAAAGATGACATCATCACAG-3'<br>5'-CACAGACATGATCAAGAATGATGGA-3'<br>5'-TGAGACGGCTCCGGTGAGACCAGAA-3'
F11R	**5'-AGCACACAAAGGCGAAGGTCAACAT-3'**<br>5'-AAGCCAACTGGTCAATATACAGGCC-3'<br>5'-GTCCCTCAGCGTGGTGATGAAGTGA-3'<br>5'-GCCAGCAAAAAGGGATATCTGCCAA-3'
PTPRS	**5'-ATGACGCAGATGACCTTTGACCTG-3'**<br>5'-CGTTTCCCATATAAATGCACGATCG-3'<br>5'-CCGCTGGAAACAAAAGACTTGATCA-3'<br>5'-CTGCCAGGACAGTAGCGATGGTGGA-3'

PGRMC2	**5'-TCGTCCGCCATACTGCAACGATAGC-3'**<br>5'-TGGTAACAATCCAAAAGCCAGTCAA-3'<br>5'-AGCAGAGATCAACACGTCTGTCTAA-3'<br>5'-GCAGCAGCTGCGCGACTACGACGGC-3'

To study cardiac function in the fish, video was taken at a minimum of 36 fps to monitor the ventricular heartbeat. The heart rate was measured using the image segmentation function available within the NIS-Elements software. The ventricular stroke volume was measured from the perpendicular long axis (l) and short axis (s) of ventricular diameters. The measurements were made at the end of systole (when the ventricle is most contracted) and diastole (when the ventricle is most relaxed) to calculate ventricle volumes at the end of systole (V_s) and diastole (V_d). A minimum of 10 V_s and V_d was averaged for each. The ventricular volume was calculated based on the formula volume = 0.5 X l X s^2. The stroke volume was calculated by subtracting V_d from V_s (stroke volume = V_s - V_d) and cardiac output was calculated by multiplying stroke volume by heart rate (cardiac output = stroke volume X heart rate).

The wild-type CD-1 mice used in this study were obtained from Charles River. Based on the protein-protein interaction network that showed *PGRMC2* and *F11R* interacting directly with *TMEM216*, the phenocopies of syndromic ciliopathy of *PGRMC2* and *F11R* genes were examined. Mice were injected with virus containing p-Scramble-C-shLenti, pPGRMC2-GFP-shLenti or pF11R-GFP-shLenti. The efficiency of knockdown for both genes was verified in the cell culture *in vitro*. Purified viral particles (10^{6-8} IFU/mL) were injected subcutaneously in two- and three-week–old mice. At 5 weeks old, the mice were introduced and acclimated to the blood pressure restrainers. Blood pressure was taken at 6 weeks old. with the non-invasive tail-cuff method using the CODA system (*Kent Scientific*). At 9 weeks, 150 μL of kidney-specific contrast agent OptiPrep (*Sigma*) was intravenously injected in the tail to analyze the kidney volume and cyst progression. Mice were imaged with an IVIS Spectrum CT imaging system (*PerkinElmer*). At 10 weeks, prior to euthanization, the mice were injected intraperitoneally with 100 IU of heparin (*Sigma*) to prevent coagulation of the blood in the coronary artery and heart. An overdose of 200 mg/kg ketamine (*ZoetisUS*) was injected intraperitoneally followed by isolation of the heart.

For systemic cELV analysis in mice, 0.5 mL of blood was collected from each mouse after euthanasia. Blood serum was diluted in PBS for centrifugation at four different speeds: 300×*g* for 10 minutes, 2,000×*g* for 10 minutes, 10,000×*g* for 30 minutes and 100,000×*g* for 70 minutes. A FluroCet Exosome Quantitation kit was used to determine the concentration of the resuspended exosome solution (*System Bioscience*, FCET96-1).

Heart function analyses

The isolated hearts were immediately used for functional study with the heart working system (Emka Technologies, France). The hearts were cannulated and perfused with Krebs-Ringer superfusion solution (125 mM NaCl, 2.5 mM KCl, 1.25 mM NaH_2PO_4, 2 mM $CaCl_2$, 1 mM $MgCl_2$, 25 mM $NHCO_3$ and 25 mM glucose). Carbogen (95% O_2 and 5% CO_2) was introduced to the warmed perfusate to reach a pH of 7.4 at 38.0 ºC. Cardiac function and electrocardiograms were then examined in the presence of saline, 0.08 μg/L verapamil (*Hospira*) or 4 μg/L epinephrine (*Vetone*). Isolated kidneys, livers, hearts, testes and spleens were imaged with the IVIS Spectrum and a high definition camera (Sony Exmor HD-CMOS; *Sony*) to validate the delivery of the pGFP-C-shLenti to the organs. All tissues obtained from the mice were further processed for staining (Excelsior, *Thermo Fisher*) with a standard H&E and Masson's Trichrome staining.

Data Analyses

Images taken with the Nikon A1R Confocal microscope were analyzed and reconstructed with Nikon NIS Element for Advanced Research software. Images taken with the Nikon TE2000 and Ti-E were analyzed with MetaMorph (*Molecular Devices*, version 7.8) and NIS-Elements (*Nikon;* version 4.30) software, respectively. The Nikon NIS-Element for Advanced Research was used for image capture and analysis, including 3D object reconstruction, image scanning and segmentation, optical flow, single-particle tracking and automatic object recognition. Whenever possible, a representative image is provided from digital images without empty magnification (stretching or enlarging the image). When impossible, videos are provided to depict the dynamic

image analysis, such as changes in particle size, shape, speed and acceleration. For the intracellular calcium, sodium and cAMP studies, the excitation wavelength changer for the DG4/DG5 system was used. For better focusing, the microscope was equipped with an XY-axis motorized flat top inverted stage, Nikon automatic focusing and a custom-designed vibration isolation platform. For a better controlled environment, the body of the microscope was enclosed inside a custom-built chamber to control CO_2 levels, humidity, heat and light. Videos and images taken from the Nikon microscopes were captured at the highest resolving power allowed by the imaging system. A Photometric CoolSNAP EZ CCD Monochrome Digital Camera was used with a 1392×1040 imaging array to resolve the fine details of the images. In other cases, both resonant and galvano scanners were used with a Nikon A1R confocal microscope for high-speed scanning with a resolution of 4096×4096. Scale bars are provided in all figures and videos to indicate the actual image reduction size. All videos and images were finalized on a 6-core Mac Pro 3.9 GHz to facilitate complete data extraction.

Potential genetic interactions were analyzed by *Cytoscape* (version 3.3.0) to study their protein-protein interaction networking. The list of the protein interactions was further simplified to only show proteins that interacted directly or indirectly with the selected five proteins, i.e., BMPR2, JAM-A/F11R, PGRMC2, PTPRS and TfR1. Proteins that interacted with TMEM216 were also analyzed. Proteins that interacted the most with TMEM216 were highlighted. Protein abundance of some of the newly identified proteins were characterized using Excel software to generate a heat map (version 15).

All quantifiable data are reported as the mean±standard error of the mean. The homogeneity of variance (homoscedasticity) was verified within each data set. When a data set was not normally distributed or heterogeneous variance was detected, the distributions were normalized via log transformation. This approach produced normally distributed data sets. Statistical analysis was performed using ANOVA (analysis of variance) followed by a Bonferroni post hoc test. Power analysis was determined from the coefficient variant. Most of our statistical analyses were performed with *GraphPad* Prism software (version 7.0). In some cases, Microsoft Excel v.15.4 was used for regression analyses. Linear regression was performed to obtain a standard

calibration curve and linear equation. In this case, the analysis was done with the ordinary least squares (OLS) regression of y on x. A non-linear logarithmic regression was used to fit the sigmoidal trend curve to show the dose-response relationship. Asterisks (*) denote statistically significant differences at various probability levels (*P*). The *P* values of the significant differences, numbers of experimental replicates and sample sizes are indicated in the figure legends.

Supplement Discussion

Characterizations of cELV

In our calculation, we used the deflection data of cilia with and without cELV by modeling the deflection in the small bending limit (**Supplement 1**). Our mathematical model showed that the impact of the cELV could alter the flexural rigidity of cilia, which was double in cilia with cELV (EI=6X10^{-23} Nm^2) compared to cilia without cELV (EI=3X10^{-23} Nm^2). Based on our calculations, the mass of the cELV directly correlated with the mass of primary cilia (**Supplement 2**). The mass of cELV was about 1.7±0.3% of the cilia mass.

Kinesis-binding proteins are known to modulate the activity of kinesin-8 (KIF18A), which is involved in vesicle transport[19, 20]. Since KIF18A is a motor protein that uses ATP to produce force and movement along microtubules[19, 20], KIF18A function was closely examined with regard to the movements and formation of cELV. We used a selective and ATP-competitive KIF18A inhibitor (BTB1)[1]. Because the trafficking of cargos into primary cilia depends on the intraflagellar transport (IFT) complex, which is composed of dynein motor[21], we also used dynein inhibitor (ciliobrevin D)[3] to evaluate if cELV is affected by the IFT system. Since kinesin and dynein are also involved in cell division and cilia formation[22-24], we applied the inhibitors only after the cilia had been formed in differentiated cells (**Figure S1f,g**). The inhibition of kinesin but not dynein induced cilia absorption (**Figure 1d**), confirming that kinesin is involved in cilia maintenance[25, 26]. In BTB1-treated cells with cilia lengths comparable with those of the control, inhibiting kinesin significantly reduced cELV formation but not movement, suggesting that the IFT complex had no effects on the dynamic movement of cELV. To examine if cELV was behaving just like commonly known extracellular vehicle (EV), we used inhibitors GW4869 and SMase to block EV generation. These inhibitors did not affect cilia formation or cell division but they significantly blocked cELV formation, indicating that cELV might have common properties of EV (**Figure S1d-g**).

We collected and concentrated the CD63-GFP vesicles (**Figure S1e**). Compared with ciliated cells, non-ciliated cells showed a significant binding of CD63-GFP vesicles to the cells (**Figure S1e-f**).

Exogenous cELV triggers cellular signaling in the cilioplasm and cytoplasm.

Using the isolated CD63-GFP containing vesicles, we looked at potential calcium and sodium signals that they might promote using respective Fura2-AM and SBFI-AM ratiometric indicators (**Figure S3a; Movie S5**). Relative to the positive control ionomycin (1 μM), CD63-GFP containing cELV ($5X10^6$/μL) caused a sustained increase in intracellular calcium and a spike increase in intracellular sodium (**Figure S3b**). Because primary cilia are sensory organelles that sense chemical signals[26, 27], we examined whether primary cilia could function as chemosensors to sense cELV. Together with a single-cell-single-cilium side view preparation to measure both cilioplasmic and cytoplasmic signals, we used the genetically encoded ratiometric reporter 5HT6-mCherry-G-GECO[26] and the ratiometric sodium indicator SBFI-AM[28] to measure calcium and sodium signals, respectively (**Figure S4; Movie S6**). Like Fura2-AM, SBFI-AM did not permeate and load into the cilia, preventing us from measuring intraciliary sodium levels. Data from the single-cell study also supported data from our cell population study that the cELV increased intracellular cytosolic calcium and sodium levels (**Figure S5a**). Furthermore, the cELV but not EV increased intraciliary calcium levels. CD63-GFP containing EV from non-ciliated *Tg737* cells did not increase intracellular cytosolic calcium and sodium levels, indicating that once released, cELV had a different property than EV (**Figure S5b**). To examine whether the increase in ciliary calcium occurred prior to the increase in cytosolic calcium, we calculated the rates of calcium increase in the cilia and cytosol. The increase in cytosolic calcium was approximately three times faster than the increase in ciliary calcium, indicating that a potential diffusion of calcium from the cytoplasm to the cilioplasm could not be excluded. However, when we measured ionomycin-induced calcium signals, the rate of ciliary calcium increase was approximately four-fold higher than the rate of cytosolic calcium increase (**Figure S5c**), suggesting that the cELV and ionomycin had different mechanisms for inducing intracellular calcium increases.

Since cAMP signaling may also be present in the primary cilia[18], we used the genetically-encoded ratiometric reporter 5HT6-mCherry-cADDis to study the potential role of cELV in cAMP signaling. Although cELV promoted an increase in cAMP in both cilioplasm and cytoplasm, the increase in cAMP was more prominent in the cilioplasm, particularly at the base of the cilia (**Figure S6; Movie S7**). The EV collected from non-ciliated *Tg737* cells did not increase intracellular cytosolic cAMP level, supporting the idea that cELV had a different property than EV. The cELV increased cytoplasmic and cilioplasmic cAMP levels (**Figure S7a**), but EV neither increased cytoplasmic nor cilioplasmic cAMP levels (**Figure S7b**). Because the rates of cAMP increase in the cilioplasm and cytoplasm were somewhat similar, it was likely that there was no cAMP diffusion into the ciliary compartment. Forskolin (5 μM), used as a positive control to activate adenylyl cyclase, caused intraciliary and cytosolic cAMP increases at a comparable rate, indicating that primary cilia could function as cAMP responsive compartments (**Figure S7c**).

While primary cilia release cELV, our data indicate that cELV also induces differential signaling between cilium and cell body. Even though cELV increases sodium, calcium and cAMP levels intracellularly, calcium and cAMP signaling can be observed and measured in both cilia and cell body. All these signals might be crucial for cELV signaling that differentiate its function from other EV. For instance, cAMP signal may be required for internalizing the unique cELV from other EV into the cells[29].

<u>Validation of cilia isolation.</u>

Isolated cilia were thoroughly validated in multiple independent preparations without (**Figure S8b**) and with (**Figure S8c,d**) immunofluorescence techniques. The purity of cilia lysate was confirmed with ciliary maker acetylated-α-tubulin and actin for positive and negative controls, respectively (**Figure S8e**). When the cilium length was measured in these stable knockdown cells, the results were tabulated to confirm normal distribution of cilia length (**Figure S9d**).

Stable knockdown cell lines.

For each knockdown, we confirmed frequency of transfection using a GFP reporter (**Figure S6a**). Transfected cells were isolated and cloned (**Figure S6b**). Because we used four potential shRNA sequences, we next selected the sequence with the highest knockdown efficiency (**Figure S6c**).

Abnormal cELV formation is associated with ciliopathy.

To understand the *in vivo* relevance of these BMPR2, TfR1, JAM-A, PTPRS and PGRMC2 proteins, we used zebrafish as a model to screen the phenotypes associated with abnormal cELV formation. Because zebrafish has two isoforms (spice variants) of BMPR2, we examined the role of each variant (BMPR2A and BMPR2B). In these studies, scrambled and *Pkd2* knockdown were used as negative and positive controls, respectively. Although some variation and severity were observed, knockdown of the corresponding six genes (*BMPR2A*, *BMPR2B*, *TFRC*, *F11R*, *PTPRS* and *PGRMC2*) resulted predominantly in ciliopathic disorders, including abnormal development, hydrocephalus, kidney cysts and left-right asymmetry in cardiac looping (**Figure S12**). The abnormal development was indicated by either a strong dorsal axis curvature or incomplete development of the dorsal axis. Although the *PTPRS* knockdown fish showed a relatively normal dorsal axis phenotype, ascites were consistently observed, albeit not as severe as in the other knockdown fish. Complementary to the left-right asymmetry in cardiac looping results, pericardial edema was also observed in the majority of the knockdown fish, suggesting a potential cardiovascular abnormality. To investigate this possibility, we measured the main parameters of heart rate and function (**Figure S9d**). The data indicated that the hearts were failing with an increased heart rate and/or lower heart contractility.

Characteristics of mice lacking cELV.

The cardiac phenotypes have been explained and discussed in the manuscript. The extra-cardiac phenotypes are discussed elsewhere (**Supplement 3**).

Supplement Reference

1 J. Locke, A. P. Joseph, A. Pena, M. M. Mockel, T. U. Mayer, M. Topf, and C. A. Moores, Structural basis of human kinesin-8 function and inhibition. *Proc Natl Acad Sci U S A*. 114, E9539-E9548 (**2017**)

2 M. Catarinella, T. Gruner, T. Strittmatter, A. Marx, and T. U. Mayer, BTB-1: a small molecule inhibitor of the mitotic motor protein Kif18A. *Angew Chem Int Ed Engl*. 48, 9072-6 (**2009**)

3 F. Ye, D. K. Breslow, E. F. Koslover, A. J. Spakowitz, W. J. Nelson, and M. V. Nachury, Single molecule imaging reveals a major role for diffusion in the exploration of ciliary space by signaling receptors. *Elife*. 2, e00654 (**2013**)

4 K. Essandoh, L. Yang, X. Wang, W. Huang, D. Qin, J. Hao, Y. Wang, B. Zingarelli, T. Peng, and G. C. Fan, Blockade of exosome generation with GW4869 dampens the sepsis-induced inflammation and cardiac dysfunction. *Biochim Biophys Acta*. 1852, 2362-71 (**2015**)

5 J. Li, K. Liu, Y. Liu, Y. Xu, F. Zhang, H. Yang, J. Liu, T. Pan, J. Chen, M. Wu, X. Zhou, and Z. Yuan, Exosomes mediate the cell-to-cell transmission of IFN-alpha-induced antiviral activity. *Nat Immunol*. 14, 793-803 (**2013**)

6 K. Trajkovic, C. Hsu, S. Chiantia, L. Rajendran, D. Wenzel, F. Wieland, P. Schwille, B. Brugger, and M. Simons, Ceramide triggers budding of exosome vesicles into multivesicular endosomes. *Science*. 319, 1244-7 (**2008**)

7 K. A. Mitchell, Isolation of primary cilia by shear force. *Curr Protoc Cell Biol*. Chapter 3, Unit 3 42 1-9 (**2013**)

8 A. M. Mohieldin, H. S. Haymour, S. T. Lo, W. A. AbouAlaiwi, K. F. Atkinson, C. J. Ward, M. Gao, O. Wessely, and S. M. Nauli, Protein composition and movements of membrane swellings associated with primary cilia. *Cell Mol Life Sci*. 72, 2415-29 (**2015**)

9 C. Thery, S. Amigorena, G. Raposo, and A. Clayton, Isolation and characterization of exosomes from cell culture supernatants and biological fluids. *Curr Protoc Cell Biol*. Chapter 3, Unit 3 22 (**2006**)

10 D. A. Wolters, M. P. Washburn, and J. R. Yates, 3rd, An automated multidimensional protein identification technology for shotgun proteomics. *Anal Chem*. 73, 5683-90 (**2001**)

11 W. H. McDonald, D. L. Tabb, R. G. Sadygov, M. J. MacCoss, J. Venable, J. Graumann, J. R. Johnson, D. Cociorva, and J. R. Yates, 3rd, MS1, MS2, and SQT-three unified, compact, and easily parsed file formats for the storage of shotgun proteomic spectra and identifications. *Rapid Commun Mass Spectrom*. 18, 2162-8 (**2004**)

12 T. Xu, S. K. Park, J. D. Venable, J. A. Wohlschlegel, J. K. Diedrich, D. Cociorva, B. Lu, L. Liao, J. Hewel, X. Han, C. C. L. Wong, B. Fonslow, C. Delahunty, Y. Gao, H. Shah, and J. R. Yates, 3rd, ProLuCID: An improved SEQUEST-like algorithm with enhanced sensitivity and specificity. *J Proteomics*. 129, 16-24 (**2015**)

13 J. Peng, J. E. Elias, C. C. Thoreen, L. J. Licklider, and S. P. Gygi, Evaluation of multidimensional chromatography coupled with tandem mass spectrometry (LC/LC-MS/MS) for large-scale protein analysis: the yeast proteome. *J Proteome Res*. 2, 43-50 (**2003**)

14 D. L. Tabb, W. H. McDonald, and J. R. Yates, 3rd, DTASelect and Contrast: tools for assembling and comparing protein identifications from shotgun proteomics. *J Proteome Res*. 1, 21-6 (**2002**)

15 W. A. Aboualaiwi, B. S. Muntean, S. Ratnam, B. Joe, L. Liu, R. L. Booth, I. Rodriguez, B. S. Herbert, R. L. Bacallao, M. Fruttiger, T. W. Mak, J. Zhou, and S. M. Nauli, Survivin-induced abnormal ploidy contributes to cystic kidney and aneurysm formation. *Circulation*. 129, 660-72 (**2014**)

16 S. M. Nauli, X. Jin, W. A. AbouAlaiwi, W. El-Jouni, X. Su, and J. Zhou, Non-motile primary cilia as fluid shear stress mechanosensors. *Methods Enzymol*. 525, 1-20 (**2013**)

17 S. Su, S. C. Phua, R. DeRose, S. Chiba, K. Narita, P. N. Kalugin, T. Katada, K. Kontani, S. Takeda, and T. Inoue, Genetically encoded calcium indicator illuminates calcium dynamics in primary cilia. *Nat Methods*. 10, 1105-7 (**2013**)

18 B. S. Moore, A. N. Stepanchick, P. H. Tewson, C. M. Hartle, J. Zhang, A. M. Quinn, T. E. Hughes, and T. Mirshahi, Cilia have high cAMP levels that are inhibited by Sonic Hedgehog-regulated calcium dynamics. *Proc Natl Acad Sci U S A*. 113, 13069-13074 (**2016**)

19 J. Pu, C. M. Guardia, T. Keren-Kaplan, and J. S. Bonifacino, Mechanisms and functions of lysosome positioning. *J Cell Sci*. 129, 4329-4339 (**2016**)

20 J. T. Kevenaar, S. Bianchi, M. van Spronsen, N. Olieric, J. Lipka, C. P. Frias, M. Mikhaylova, M. Harterink, N. Keijzer, P. S. Wulf, M. Hilbert, L. C. Kapitein, E. de Graaff, A. Ahkmanova, M. O. Steinmetz, and C. C. Hoogenraad, Kinesin-Binding Protein Controls Microtubule Dynamics and Cargo Trafficking by Regulating Kinesin Motor Activity. *Curr Biol*. 26, 849-61 (**2016**)

21 J. L. Rosenbaum, and G. B. Witman, Intraflagellar transport. *Nat Rev Mol Cell Biol*. 3, 813-25 (**2002**)

22 J. A. Jonassen, J. SanAgustin, S. P. Baker, and G. J. Pazour, Disruption of IFT complex A causes cystic kidneys without mitotic spindle misorientation. *J Am Soc Nephrol*. 23, 641-51 (**2012**)
23 Y. Hou, H. Qin, J. A. Follit, G. J. Pazour, J. L. Rosenbaum, and G. B. Witman, Functional analysis of an individual IFT protein: IFT46 is required for transport of outer dynein arms into flagella. *J Cell Biol*. 176, 653-65 (**2007**)
24 D. G. Cole, D. R. Diener, A. L. Himelblau, P. L. Beech, J. C. Fuster, and J. L. Rosenbaum, Chlamydomonas kinesin-II-dependent intraflagellar transport (IFT): IFT particles contain proteins required for ciliary assembly in Caenorhabditis elegans sensory neurons. *J Cell Biol*. 141, 993-1008 (**1998**)
25 M. He, R. Subramanian, F. Bangs, T. Omelchenko, K. F. Liem, Jr., T. M. Kapoor, and K. V. Anderson, The kinesin-4 protein Kif7 regulates mammalian Hedgehog signalling by organizing the cilium tip compartment. *Nat Cell Biol*. 16, 663-72 (**2014**)
26 S. Bhogaraju, L. Cajanek, C. Fort, T. Blisnick, K. Weber, M. Taschner, N. Mizuno, S. Lamla, P. Bastin, E. A. Nigg, and E. Lorentzen, Molecular basis of tubulin transport within the cilium by IFT74 and IFT81. *Science*. 341, 1009-12 (**2013**)
27 S. Abdul-Majeed, and S. M. Nauli, Dopamine receptor type 5 in the primary cilia has dual chemo- and mechano-sensory roles. *Hypertension*. 58, 325-31 (**2011**)
28 A. T. Harootunian, J. P. Kao, B. K. Eckert, and R. Y. Tsien, Fluorescence ratio imaging of cytosolic free Na+ in individual fibroblasts and lymphocytes. *J Biol Chem*. 264, 19458-67 (**1989**)
29 W. Oosthuyzen, K. M. Scullion, J. R. Ivy, E. E. Morrison, R. W. Hunter, P. J. Starkey Lewis, E. O'Duibhir, J. M. Street, A. Caporali, C. D. Gregory, S. J. Forbes, D. J. Webb, M. A. Bailey, and J. W. Dear, Vasopressin Regulates Extracellular Vesicle Uptake by Kidney Collecting Duct Cells. *J Am Soc Nephrol*. 27, 3345-3355 (**2016**)
