# Supplementary material for: Proteomic Identification Reveals the Role of Ciliary Extracellular‐Like Vesicle in Cardiovascular Function
Source: Adv Sci (Weinh). 2020 Jun 16;7(16):1903140. doi: 10.1002/advs.201903140 (PMC7435257; doi:10.1002/advs.201903140)
Supplement: Supplementary file 2 — Supplemental pdf 1 [file ADVS-7-1903140-s002.pdf]

Supplement S1

Mechanics of the deflection of primary cilia by fluid flow

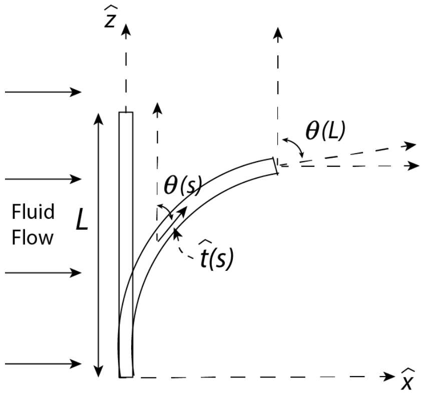

Figure 1. Bent elastic rod under fluid flow with rod fixed at s = 0 and free at s = L.

The impact of cELV on primary cilia is investigated using deflection data of cilia with and without cELV by modeling the deflection in the small bending limit and solving the resulting Euler-Bernoulli equation. The impact of the cELV is assumed to change the flexural rigidity and shown to nearly double the flexural rigidity relative to cELV-less cilia. This was calculated by using the force per unit length on cilia as a fitting parameter for the cELV-less cilia and then applied to the analysis of the rigidity of cilia with an cELV. The model does not strictly apply to the large deflection data and thus the analysis provides at best a qualitative impact of cELV on the bending of cilia in fluid flow.

I. Deflection of Cilia under fluid flow

Consider a model of cilia as a thin elastic cylindrical rod of length *L* with flexural stiffness *EI* (*E* is the Young's modulus of the rod and *I* is its second moment of inertia) attached to an infinite surface with shear flow. The energy *H* of the deflected elastic rod is a sum of the bending energy (adopting the Kratky-Porod or worm-like chain model) and the energy associated with the force *F(s)* exerted along the arc length of the rod due to shear flow:

$$H = \frac{1}{2} EI \int_0^L \left(\frac{d\hat{t}(s)}{ds} \right)^2 ds - \int_0^L \vec{F}(s) \cdot \hat{t}(s) ds \qquad (1)$$

The bending energy is taken to be the line integral of the square of the curvature parameterized in terms of the unit tangent vector at arc length s, $\hat{t}(s)$ with inextensibility assumption requiring $\left|\hat{t}(s)\right| = 1$. Assuming the rod lies along the z-axis with the fluid flow along the x –axis where $\vec{F}(s) = F(s)\hat{x}$. Parameterizing in

terms of the angle θ with respect to the z-axis where $\hat{t}(s)=\theta(s)$ (see figure 1) and $\vec{F}(s)\cdot\hat{t}(s)=F(s)\hat{x}\cdot\hat{t}(s)=F(s)\sin\theta(s)$, thus (1) becomes

$$H=\int_0^L ds\left\{\frac{1}{2}EI\left(\frac{d\theta(s)}{ds}\right)^2-F(s)\sin\theta(s)\right\} \tag{2}$$

Where the boundary conditions are that the elastic rod is fixed at $s=0$ thus $\theta(s=0)=0$ and is free at the other end s = L where the torque is zero, i.e. $d\theta(s)/ds\big|_{s=L}=0$.

Consider the *small deflection approximation* in order to develop an analytically tractable model, a reasonable approximation for moderate bending [1]. Thus in this limit we assume $ds\approx dz$, $\sin\theta(s)\approx\theta(s)$ where $\theta(s)\approx\left(du(z)/dz\right)$ with $u(z)$ the displacement of the elastic rod from the z-axis the energy (1.2) becomes

$$H=\int_0^L dz\left\{\frac{1}{2}EI\left(\frac{d^2u(z)}{dz^2}\right)^2-F(z)\left(\frac{du(z)}{dz}\right)\right\} \tag{3}$$

The extremum of the energy (1.3), given by the Euler-Lagrange equation, gives

$$\frac{d^3u(z)}{dz^3}=-\frac{F(z)}{EI} \tag{4}$$

with the boundary conditions:

$$\begin{aligned} &u(z=0)=0\\ &du(z)/dz\big|_{z=0}=0\\ &du^2(z)/dz^2\big|_{z=L}=0 \ \text{(zero moment at free end, M = 0)}\end{aligned} \tag{5}$$

Let's assume the force due to the fluid flow is constant $F(s)=F$. After the first integration of (4) we have

$$\frac{d^2u(z)}{dz^2}=-\frac{F}{EI}z+c_1 \tag{6}$$

where c_1 is a constant and can be determined from the boundary condition at s = L, (5), which gives $c_1=FL/EI$. Thus we have

$$\frac{d^2u(z)}{dz^2}=\frac{F}{EI}\left(L-z\right) \tag{7}$$

which is in the form $d^2u(z)/dz^2=M/EI$, where M is the bending moment. Integrating (1.7) twice further and determining the constants of integration from the boundary condition at z = 0, (1.5), the equilibrium displacement of the rod from its undeformed shape along the z-axis is

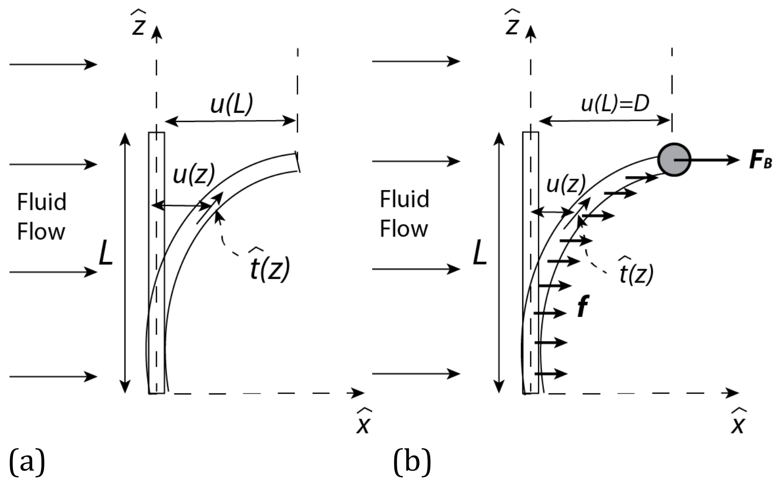

Figure 2. (a) Deformation of cilia in fluid flow parameterized in terms of the displacement *u(z)* from the *z*-axis. (b) with a massive cELV at the tip of cilia, located at maximum deflection u(L) = D where **f** is the force/length while **F** is the drag force on the cELV.

$$u(z)=\frac{Fz^2}{6EI}(3L-z) \tag{8}$$

with the maximum deflection D at the free end given by

$$D\equiv u(L)=\frac{FL^3}{3EI}\ , \tag{9}$$

equivalent to the deflection of a cantilever with spring constant $3EI/L^3$. Thus the characteristic shearing force F_c which bends cilia with $D \sim L$ is

$$F_c \sim \frac{3EI}{L^2}\ . \tag{10}$$

To relate the shearing force F to the wall shear stress σ generated in the fluid flow experiments consider the drag force due to shear flow on a cylinder, in the limit of low Reynolds number, given by Venier et al. [2] from slender body theory:

$$F \approx \frac{2\pi\sigma L^2}{\ln(L/a)} \tag{11}$$

where σ is the wall shear stress and a is the radius of cilia. Thus we can relate the characteristic shear force with its stress σ from (10) and (11) to find

$$\sigma \approx \frac{3EI\ln(L/a)}{2\pi L^4}\ . \tag{12}$$

If we take for the flexural rigidity of cilia to be $EI = 3\times10^{-23}\,Nm^2$ [3], the radius of primary cilia to be $a = 100nm$ with length $L = 5\mu m$, we find for the critical shear stress to be $\sigma \sim 0.09\ N/m^2 \sim 1\ dyne/cm^2$ which is on the order of magnitude of the stress that has been measured for bending of cilia.

II. Deflection of Cilia with cELV-mass on tip of cilia

Now consider an cELV of mass m_{ex} at the tip of cilia, see Figure 2(b), which produces a bending moment about the base of cilia resulting from the force on the cELV due to the relative velocity of the fluid and the cELV such that the equations determining the equilibrium deformation are given by the following equations:

$$M = EI\frac{d^2u(z)}{dz^2}$$
$$\frac{dM}{dz} = EI\frac{d^3u(z)}{dz^3} = F \text{ (shear force)} \tag{13}$$
$$\frac{d^2M}{dz^2} = EI\frac{d^4u(z)}{dz^4} = f \text{ (force per unit length)}$$

where the boundary conditions are given by (1.16) and (1.17), wherein the impact of the effects of the cELV are accounted for by the boundary condition at the free end:

(i) Clamped boundary condition at the base of cilia:

$$u(z=0)=0 \quad \left.\frac{du(z)}{dz}\right|_{z=0} = 0 \tag{14}$$

(ii) At the free end include the effect of the cELV mass where $F = -F_B$ and $M = 0$ at $z = L$, thus :

$$EI\left.\frac{d^3u(z)}{dz^3}\right|_{z=L} = -F_B \text{ and } \left.\frac{d^2u(z)}{dz^2}\right|_{z=L} = 0 \tag{15}$$

where F_B is the drag force on thecELV. The general solution of $EI\frac{d^4u(z)}{dz^4} = f$, the Euler-Bernoulli equation, with the boundary conditions given in (14) and (15) is thus given by:

$$u(z) = \frac{f}{24EI}z^4 - \frac{(F_B + fL)}{6EI}z^3 + \frac{L(F_B + fL)}{2EI}z^2 \tag{16}$$

The maximum deflection at $z = L$ is thus, $u(z=L) = D$:

$$D = \frac{L^3}{3EI}\left(\frac{3fL}{8} + F_B\right) \tag{17}$$

This is equivalent to the deflection of a clamped cantilever with spring constant $k = 3EI / L^3$ with an effective force of $3fL/8 + F_B$ applied at its free end. Assuming Stokes law for the drag force on the cELV, taken to be a sphere of radius r_B, then $F_B = 6\pi\mu r_B v$ where μ is the viscosity of the fluid, and v is the velocity of the fluid. Thus the maximum deflection (17) is

$$D = \frac{L^3}{3EI}\left(\frac{3fL}{8} + 6\pi\mu r_B v\right) . \tag{18}$$

Consider the maximum deflection data in Table 1 for two cilia under fluid flow with and without cELV in the same frame. We will assume that the flexural rigidity is not the same for the two cases due to the cELV. To characterize the impact of the cELV, the force per unit length *f* on cilia is calculated from the maximum deflection data of cilia without cELV using the flexural rigidity of cilia without cELV, $(EI)_0 = 3\times10^{-23} Nm^2$ [3], and then using the calculated force per unit length to determine the flexural rigidity *EI* with cELV as a fitting parameter that corresponds to the measured maximum deflection of 8.4 μm. Thus without the cELV, the maximum deflection is given by equation (17) by setting $F_B = 0$, thus $D_0 = fL_0^4 / 8EI$, where with $D_0 = 5.6\ \mu m$ and $L_0 = 6.8\ \mu$m gives for the force per unit length on cilia of $f = 8(EI)_0 D_0 / L_0^4$ equaling $f = 6.3\times10^{-7} N/m$. Thus, using this force per unit length on cilia, and taking the viscosity to be that of water of $\mu = 8.9\times10^{-4} Pa{\cdot}s$, and modeling the 4 cELV as one equivalent cELV of diameter $2r_B = 0.89\ \mu m$, the flexural rigidity *EI* of cilia with cELV is given by

$$EI = \left(EI\right)_0 \left(\frac{L}{L_0}\right)^4 \frac{D_0}{D} + \frac{F_B L^3}{3D} \tag{19}$$

$$where\ \ F_B = 6\pi\mu r_B v$$

and given the corresponding maximum deflection of $D = 8.4\ \mu$m, the rigidity is calculated to be $EI = 5.7\times10^{-23}\ Nm^2$. Thus, the impact of the cELV is to increase the flexural rigidity, approximately doubling it. The model assumed small bending which is not applicable to the deflection data and thus the analysis should only represent a qualitative picture of the impact of cELV on the bending cilia in fluid flow.

	Cilia	Cilia with cELV
Cilia length (μm)	L_0 = 6.8	L = 8.8
Cilia diameter (μm)	0.20	0.23
cELV diameter (μm)	-	4 cELV: 0.21; 0.20; 0.18; 0.15<br>= single cELV with diameter 2 r_B = 0.30 μm
Maximum deflection (μm)	D_0 = 5.6	D = 8.4
Fluid velocity (v) (μm/s)	14.84	14.84

Table 1. Maximum deflection data measured of cilia under fluid flow for cilia with and without cELV in the same frame.

[1] Pozrikidis, C. Shear flow over cylindrical rods attached to a substrate. Journal of Fluids and Structures 2010; 26 393-405.
[2] Venier P, Maggs AC, Carlier MF, Pantoloni D. Analysis of microtubule rigidity using hydrodynamic flow and thermal fluctuations. J Biol Chem. 1994; 269 (18): 13353-13360.
[3] Schwartz EA, Leonard ML, Bizios R, Bowser SS. Analysis and modeling of the primary cilium bending response to fluid shear. Am J Physiol. 1997;272(1 Pt 2):F132–138.
