# Supplementary material for: Proteomic Identification Reveals the Role of Ciliary Extracellular‐Like Vesicle in Cardiovascular Function
Source: Adv Sci (Weinh). 2020 Jun 16;7(16):1903140. doi: 10.1002/advs.201903140 (PMC7435257; doi:10.1002/advs.201903140)
Supplement: Supplementary file 3 — Supplemental pdf 2 [file ADVS-7-1903140-s003.pdf]

Supplement S2

Estimating the Mass of the Ciliary Extracellular like Vesicles (cELV)

Consider the density of the cELV to be equal to the density of cilium. To estimate the density of cilium consider its composition and obtain an average cilium density [1]; Cilium is composed of approximately 75% lipids/water with a density of 1.0 g/ cm^3, ~20% protein with a density of ~1.4 g/cm^3 and ~5% carbohydrates with a density of ~1.6 g/cm^3. Thus the average density of cilium is 1.11 g/cm^3. Let's assume the density of the cELV is equal to cilium density, $\rho_{bulb} \approx \rho_{cilium} \approx 1.11\, g/cm^3$, and from the measured size of the cELV, and assuming spherical geometry, the mass of the cELV can be estimated as

$$m_B = \rho_{bulb} V_{bulb} \approx \rho_{cilium} \left(\frac{4}{3} \pi r_B^3 \right) . \qquad (1)$$

Listed in table 1 is 14 different cases with a comparison to the mass of cilium, $m_c \simeq \rho_{cilium} \pi a^2 L$ (where $a = 100\ nm$), and its' length L. There is a statistically significant ($p < .01$) positive correlation between the mass or length of cilium and the ELV's mass (or diameter) with a correlation coefficient of $R = 0.75$ and a p-value of $p = 0.002$, see Figure 1a. Note that if the following two data points are neglected, {(.21, 3.2), (.23, 2.51)}, then the correlation coefficient is $R = 0.96$ and the correlation is even further positively enhanced, see Figure 1b. This positive, statistically significant, correlation suggests that the longer (and more massive) the cilium is then the larger and more massive is the associated cELV.

Cilia Length (μm)	cELV Diameter (μm)	Cilia Mass (pg)	cELV Mass (pg)
6.28	1.77	0.218987682	3.222764652
12.09	1.33	0.421586159	1.36729969
5.96	0.63	0.207829074	0.14532169
30.06	2.05	1.048211739	5.00691681
3.31	0.72	0.115421852	0.21692334
14.65	1.37	0.510855023	1.494412508
6.31	1.04	0.220033802	0.653745647
5.22	1.16	0.182024793	0.907157635
8.2	1.27	0.28593933	1.190474111
4.74	0.78	0.165286881	0.275798945
10.84	1.06	0.377997846	0.692191701
5.48	0.9	0.191091162	0.423678398
6.78	1.63	0.236423007	2.516932715
13.09	1.43	0.456456809	1.699483314

Table 1. Cilium and cELV characteristics.

a.

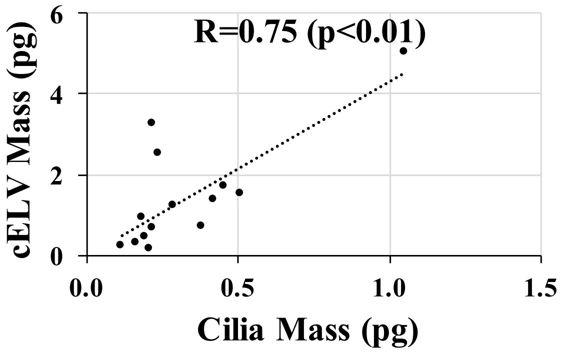

b.

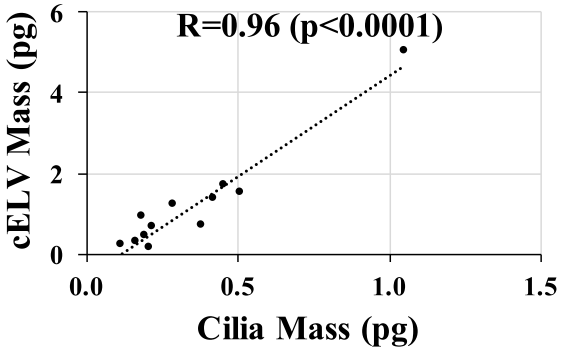

Figure 1. (a) Positive correlation between cELV and cilium mass with correlation coefficient R = 0.75 (p = 0.002) and even stronger positive correlation with the exclusion of the following two data points {(.21, 3.2), (.23, 2.51)} in (b).

cELV Mass Estimate from characteristic bending force without Fluid Flow

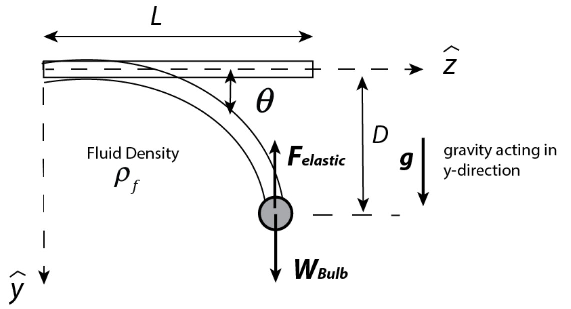

Figure 3. Equilibrium configuration of cilia without flow.

Consider the equilibrium configuration of cilia in the fluid medium of density ρ_f, figure 3, where the effective weight of the cELV and cilia are balanced by the elastic restoring force of bending cilia characterized by the (characteristic cantilever or beam) spring constant $k = 3EI/L^3$ and proportional to the maximum displacement of the tip D. Thus we have the equilibrium condition:

$$F_{elastic} \approx kD \approx W_{Bulb} + W_{cilia} = \left(m_B g - \rho_f V_B g\right) + \left(m_c g - \rho_f V_C g\right) \quad (2)$$

where W_{Bulb} and W_{cilia} is the effective weight of the cELV and cilia, respectively, where their masses are m_B and m_c and $V_B = \frac{4}{3}\pi r_B^3$ and $V_c = \pi a^2 L$ are their volumes. Letting $D \sim L\sin\theta$ then the mass of the cELV is given by

$$m_B = \frac{3EI}{L^2 g}\sin\theta + \rho_f (V_B + V_C) - \rho_c V_C \quad (3)$$

where ρ_c is the density of cilia, again taken to be $\rho_{cilium} \approx 1.11\, g/cm^3$ and the density of the fluid is $\rho_f = 1.005\, g/cm^3$. Equilibrium angular θ data is listed in table 2 with the corresponding cELV mass. The data is not reliable due to estimating the angle θ from the z-x-plane and not from the z-y plane, the relevant direction that θ is needed to obtain the ELV's mass with this approach. The cELV mass obtained is generally an order of magnitude greater than from its' size and density estimate.

Case	$L\ (\mu m)$	$\theta\ (deg)$	cELV diameter (μm)	cELV Mass (pg)
1	6.78	9.45	1.63	35.1
2	8.53	14.21	2.56	39.8
3	6.56	8.54	1.98	35.8

Table 2. Equilibrium angular data for 3 cases and the calculation of the cELV mass.

[1] Resnick, A., Hopfer, U. Force-response Considerations in Ciliary mechanosensation. Biophys. J. 2007. 93:1380-1390.
