# Supplementary material for: Proteomic Identification Reveals the Role of Ciliary Extracellular‐Like Vesicle in Cardiovascular Function
Source: Adv Sci (Weinh). 2020 Jun 16;7(16):1903140. doi: 10.1002/advs.201903140 (PMC7435257; doi:10.1002/advs.201903140)
Supplement: Supplementary file 4 — Supplemental pdf 3 [file ADVS-7-1903140-s004.pdf]

Supplement S3

Supplementary data on extra-cardiac phenotypes: kidney, liver, spleen, and testis

In addition to heart, we confirmed that shRNA was delivered to other major visceral organs, including the kidney, liver, testis, and spleen (**Figure 1**). Throughout our studies, no significant change in kidney volume was observed (**Movie S13**). While no polycystic kidney phenotype was observed in *F11R* and *PGRMC2* mice (**Figure 2**), mild isolated renal cysts including liver cysts were observed (**Figure 3**). A decrease in vascularization was apparent in the testis from the *PGRMC2* and *F11R* mice, as indicated by the lack of surface vasculature. Testicular vascularization in *F11R* or *PGRMC2* mice was significantly decreased by 15±3%, compared to control mice. Neither cysts nor abnormal spermatogenesis was observed in the testes. Fibrosis was detected in the kidney, liver, testis and spleen (**Figure 4**). Significant fibrosis was observed around the red pulps of the spleen in *PGRMC2* knockdown mice. The trabecular structures and periarteriolar lymphoid sheaths of the spleen were comparable between the *F11R* or *PGRMC2* and scrambled control mice.

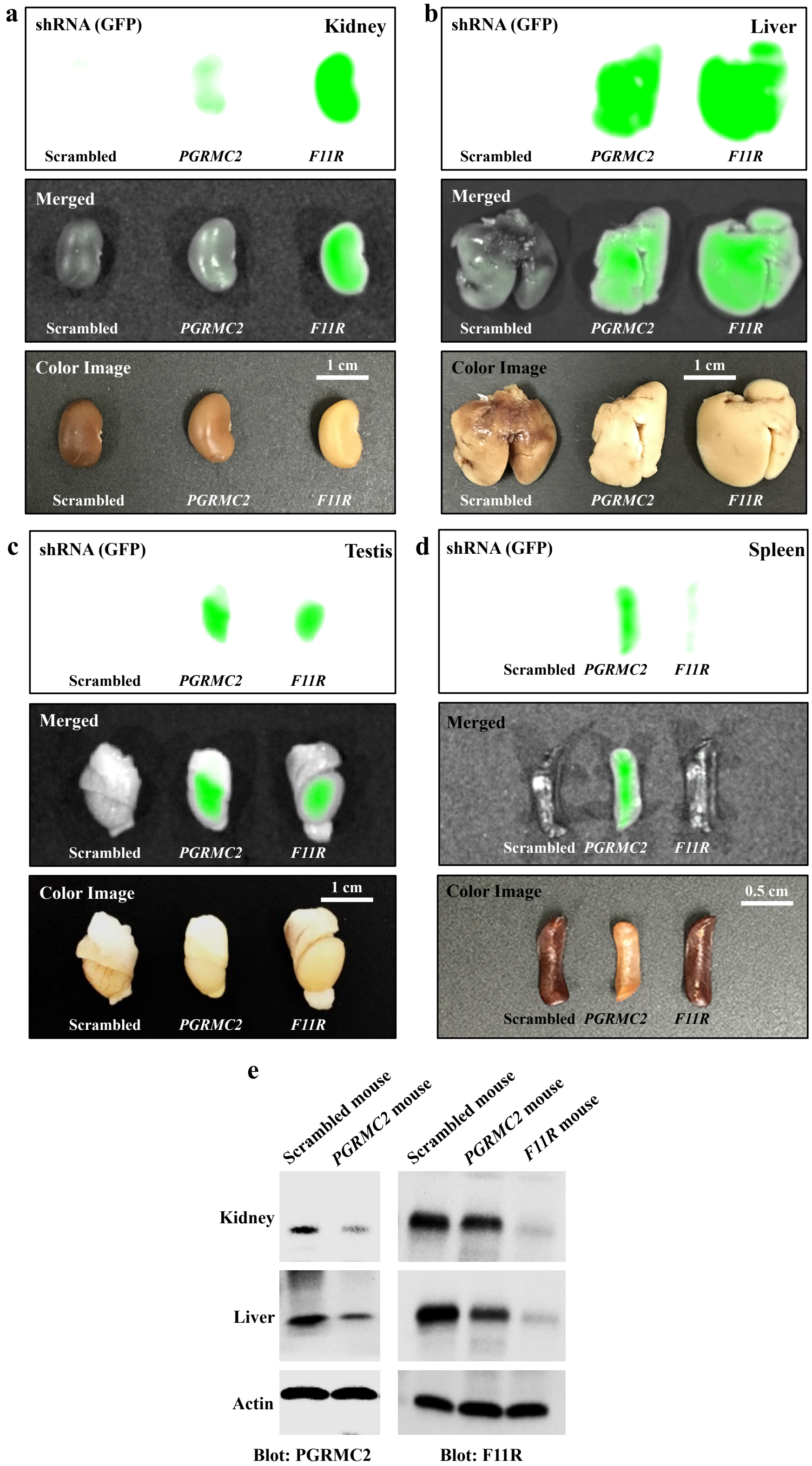

Figure 1. **Confirmation of *PGRMC2* and *F11R*-targeted lentivirus carrying GFP marker in mice.**

The kidney (**a**), liver (**b**), testis (**c**), and spleen (**d**) were confirmed for virus infection, as evidenced by the lentivirus-GFP marker, immediately after the mice were sacrificed. (**e**) Expressions of PGRMC2 and F11R in only kidney and liver organs were analyzed.

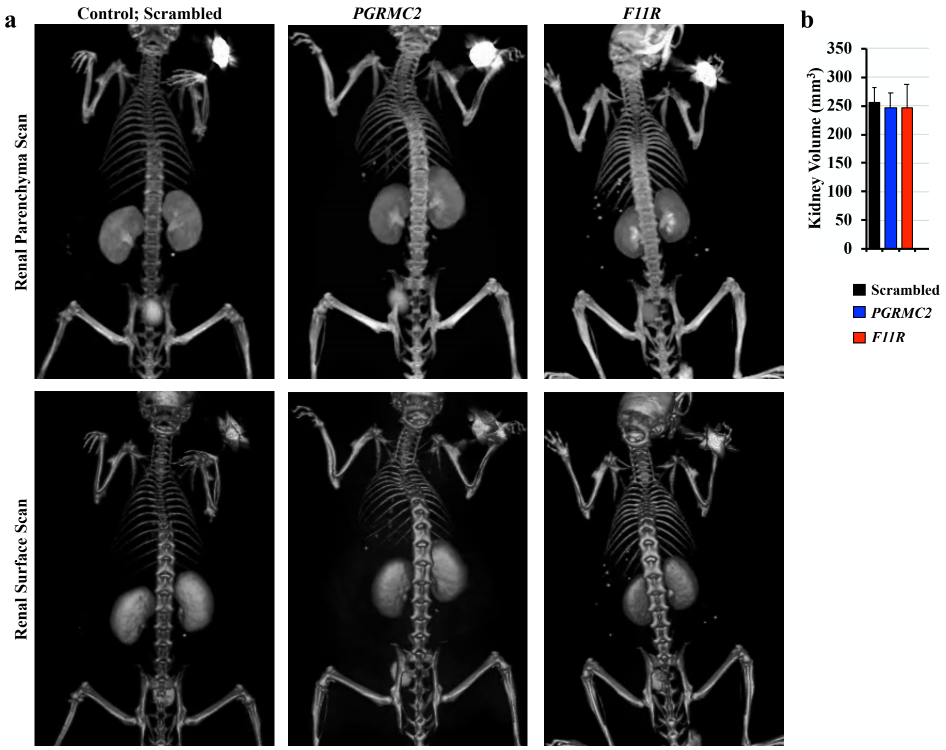

Figure 2. **No significant change in kidney volume from *PGRMC2* and *F11R* mice.**

a, The kidney-specific contrast agent OptiPrep was intravenously injected into the tail of the mice. Mice were imaged with the IVIS Spectrum CT imaging system. A computed tomography scan of the pair of kidneys was performed in 9-week-old live mice. The renal parenchyma scan (top panel) and renal surface scan (lower panel) were used to evaluate the volumes of the cyst and kidney, respectively (**Movie S13**). **b**, Total kidney volume shows that due to the small kidney cysts, there was no significant difference in renal volume between knockdown and scrambled control mice. N=3 for each group.

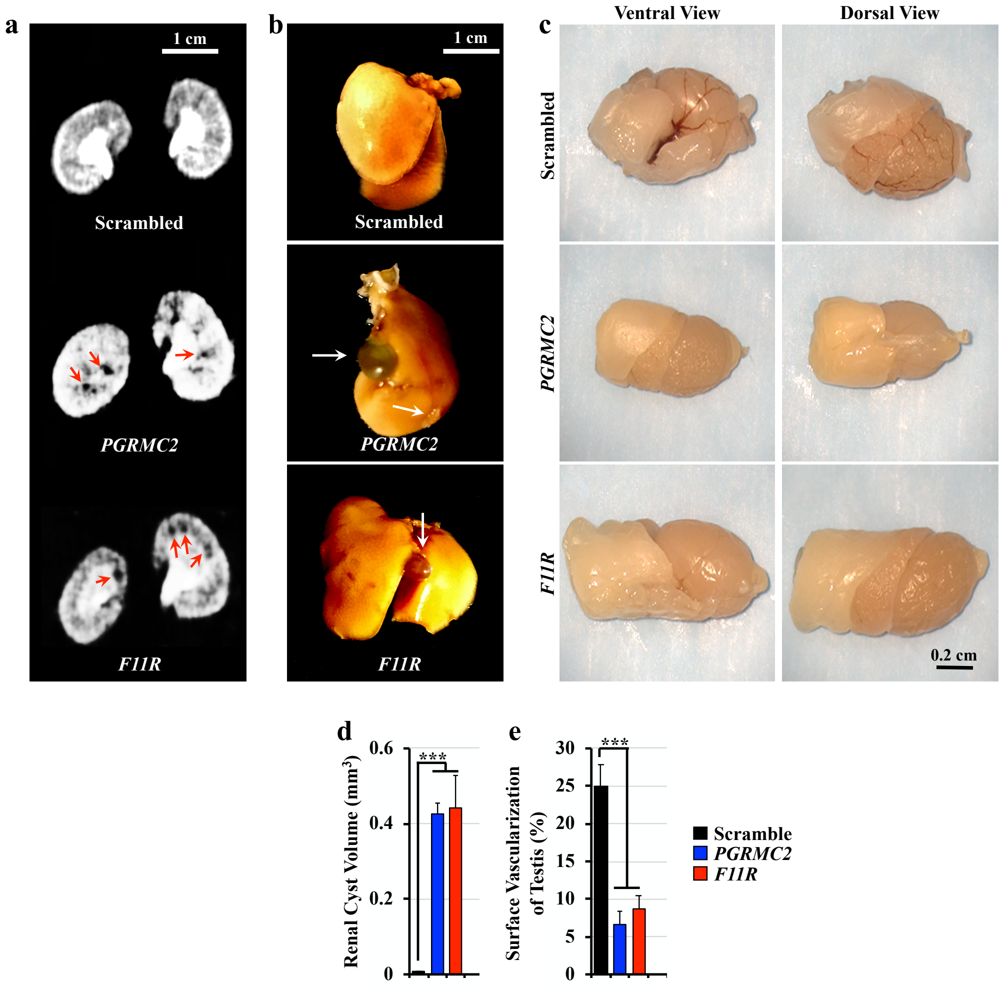

Figure 3. ***PGRMC2* and *F11R* knockdown induce kidney cyst, liver cyst, and testicular devascularization.**

a, A computed tomography scan was done on a pair of kidneys from a live 8-week-old mouse. Red arrows indicate mild isolated cysts. **b,** Isolated livers were immediately imaged after the mice were sacrificed; white arrows indicate isolated fluid-filled cysts. **c**, Representative images show the low vascular density around the testes in *PGRMC2* and *F11R* knockdown mice compared with scrambled control mice. Kidney cyst volume (**d**) and percentage of surface vascularization area in testis (**e**) were quantitated. N=3 mice in each scrambled control, *PGRMC2* and *F11R* knockdown groups. ***, $p<0.001$ compared with the corresponding scrambled control group.

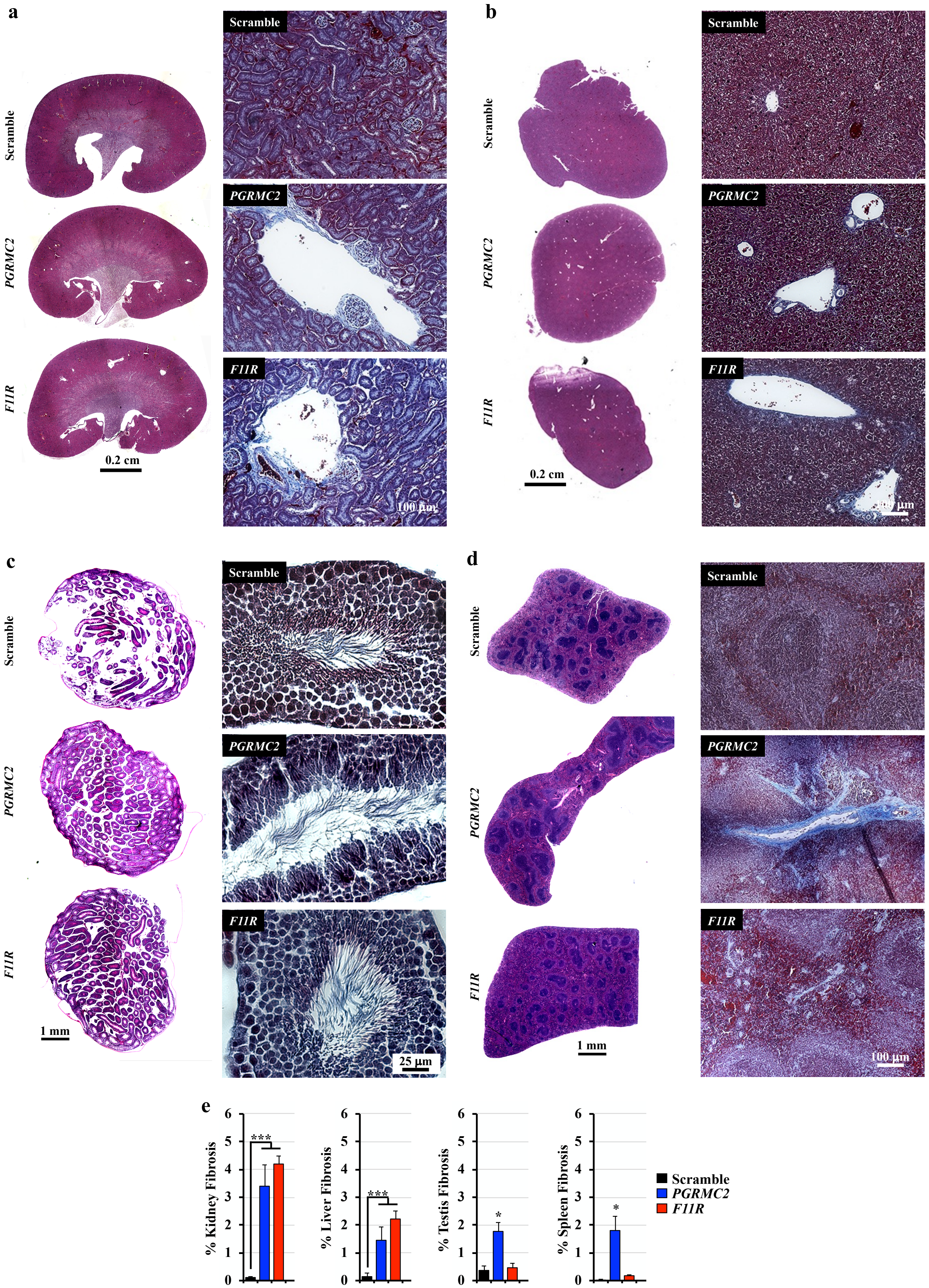
a
Scramble
PGRMC2
F11R
0.2 cm
100 µm
b
Scramble
PGRMC2
F11R
0.2 cm
100 µm
c
Scramble
PGRMC2
F11R
1 mm
25 µm
d
Scramble
PGRMC2
F11R
1 mm
100 µm
e
% Kidney Fibrosis
% Liver Fibrosis
% Testis Fibrosis
% Spleen Fibrosis
0
1
2
3
4
5
6
***
*
Scramble
PGRMC2
F11R

Figure 4. ***PGRMC2* and *F11R* knockdown result in kidney, liver, testis and spleen fibrosis.**

Hematoxylin and Eosin (H&E; left panels) and Masson's Trichrome (right panels) staining were performed for structural morphology analyses. **a,** Representative H&E staining shows isolated cysts around the cortex region of the kidneys from *PGRMC2* and *F11R* knockdown mice. Masson's Trichrome staining shows severe fibrosis around the cortex region. **b,** Representative H&E staining shows isolated cysts around the livers from the *PGRMC2* and *F11R* knockdown mice. Representative images of Masson's Trichrome staining shows fibrosis around the cyst area. **c,** Representative images show H&E staining of the testes and Masson's Trichrome staining shows fibrosis with potential testicular tubular ectasia with a decreased spermatogenesis in *PGRMC2* knockdown mice. **d,** Representative images of the H&E staining of the spleen and Masson's Trichrome staining show fibrosis in the spleens from *PGRMC2* knockdown mice. **e**, Percent of fibrosis in kidney, liver, testis and spleen were quantified. N=3 for each group. *, $p<0.05$; ***, $p<0.001$ compared with the corresponding scrambled control group.
