# Supplementary material for: Proteomic Identification Reveals the Role of Ciliary Extracellular‐Like Vesicle in Cardiovascular Function
Source: Adv Sci (Weinh). 2020 Jun 16;7(16):1903140. doi: 10.1002/advs.201903140 (PMC7435257; doi:10.1002/advs.201903140)
Supplement: Supplementary file 5 — Supplemental Table 1 [file ADVS-7-1903140-s005.pdf]

	Peptide Count								Spectral Count								Sequence Coverage							
	Whole Cell	Whole Cell	Whole Cell	Cilia Fraction	Cilia Fraction	Cilia Fraction	Cilia Fraction	Cilia Fraction	Whole Cell	Whole Cell	Whole Cell	Cilia Fraction	Cilia Fraction	Cilia Fraction	Cilia Fraction	Cilia Fraction	Whole Cell	Whole Cell	Whole Cell	Cilia Fraction	Cilia Fraction	Cilia Fraction	Cilia Fraction	Cilia Fraction
Sample#	1	2	6	4	3	5	7	8	1	2	6	4	3	5	7	8	1	2	6	4	3	5	7	8
**Accession**																								
tr|F1STN0|F1STN0	0	0	0	1	1	1	4	5	0	0	0	12	6	1	27	41	0.0%	0.0%	0.0%	2.6%	2.6%	2.6%	12.3%	16.7%
tr|F1SMI8|F1SMI8	0	0	0	1	5	0	0	0	0	0	0	2	11	0	0	0	0.0%	0.0%	0.0%	1.3%	5.7%	0.0%	0.0%	0.0%
Q9TUQ3	0	0	0	1	4	0	2	4	0	0	0	2	10	0	8	77	0.0%	0.0%	0.0%	1.2%	4.6%	0.0%	3.9%	3.6%
tr|F1RW52|F1RW52	0	0	1	2	1	0	4	4	0	0	7	6	3	0	7	16	0.0%	0.0%	3.5%	3.7%	1.8%	0.0%	8.7%	10.4%
Q8MIB3	0	0	1	2	1	0	4	4	0	0	7	6	3	0	7	16	0.0%	0.0%	3.5%	3.7%	1.8%	0.0%	8.7%	10.4%
tr|K7GKB4|K7GKB4	0	0	1	2	1	0	2	2	0	0	7	6	3	0	2	12	0.0%	0.0%	5.4%	5.6%	2.8%	0.0%	6.6%	7.7%
tr|F1SMJ1|F1SMJ1	0	0	0	1	2	0	1	1	0	0	0	2	7	0	1	70	0.0%	0.0%	0.0%	1.2%	2.3%	0.0%	1.5%	1.1%
tr|K7GQU9|K7GQU9	0	0	0	0	1	1	0	1	0	0	0	0	8	1	0	38	0.0%	0.0%	0.0%	0.0%	14.1%	14.1%	0.0%	14.1%
tr|F1S8A4|F1S8A4	0	0	0	1	1	0	0	1	0	0	0	8	1	0	0	12	0.0%	0.0%	0.0%	1.4%	1.4%	0.0%	0.0%	3.3%
tr|F1RW50|F1RW50	0	0	1	1	1	0	0	1	0	0	7	5	3	0	0	11	0.0%	0.0%	8.4%	4.4%	4.4%	0.0%	0.0%	8.4%
tr|F1RV50|F1RV50	0	0	0	2	1	0	0	1	0	0	0	6	2	0	0	6	0.0%	0.0%	0.0%	4.1%	4.1%	0.0%	0.0%	2.1%
tr|F1SDK4|F1SDK4	0	0	0	1	2	0	0	0	0	0	0	3	5	0	0	0	0.0%	0.0%	0.0%	2.3%	2.3%	0.0%	0.0%	0.0%
tr|F1S6D6|F1S6D6	0	0	1	3	2	1	2	5	0	0	1	4	3	1	3	29	0.0%	0.0%	1.9%	6.5%	5.1%	1.9%	4.4%	12.6%
tr|K7GN83|K7GN83	0	0	1	1	3	1	4	16	0	0	6	1	5	1	12	86	0.0%	0.0%	4.0%	3.4%	14.6%	4.0%	18.0%	37.2%
tr|F1S5D0|F1S5D0	0	0	1	1	3	1	3	15	0	0	6	1	5	1	11	84	0.0%	0.0%	5.1%	4.3%	18.5%	5.1%	16.9%	41.3%
tr|K7GKB6|K7GKB6	0	0	1	1	3	1	3	15	0	0	6	1	5	1	11	84	0.0%	0.0%	4.8%	4.1%	17.5%	4.8%	16.0%	39.0%
tr|I3L8B9|I3L8B9	0	0	1	1	3	1	3	13	0	0	6	1	5	1	11	70	0.0%	0.0%	5.0%	4.2%	18.1%	5.0%	16.6%	36.7%
tr|I3LSX8|I3LSX8	0	0	3	1	1	0	3	8	0	0	9	3	4	0	22	46	0.0%	0.0%	4.8%	2.6%	2.6%	0.0%	8.3%	16.8%
tr|I3LF11|I3LF11	0	0	1	1	0	0	0	0	0	0	3	7	0	0	0	0	0.0%	0.0%	15.2%	11.4%	0.0%	0.0%	0.0%	0.0%
tr|F1SNC8|F1SNC8	0	0	0	1	3	2	0	1	0	0	0	1	4	2	0	4	0.0%	0.0%	0.0%	3.6%	12.3%	12.7%	0.0%	2.4%
tr|I3LSC5|I3LSC5	0	0	0	1	3	0	1	5	0	0	0	2	4	0	1	28	0.0%	0.0%	0.0%	4.1%	9.1%	0.0%	4.9%	14.6%
tr|F1SME1|F1SME1	0	0	0	1	1	0	0	1	0	0	0	2	4	0	0	1	0.0%	0.0%	0.0%	0.4%	0.4%	0.0%	0.0%	0.4%
tr|I3L9E5|I3L9E5	0	0	2	1	2	1	1	5	0	0	10	2	2	1	65	14	0.0%	0.0%	6.3%	2.5%	5.4%	2.5%	2.5%	12.3%
tr|F1RH87|F1RH87	0	0	2	1	2	1	1	5	0	0	10	2	2	1	65	14	0.0%	0.0%	6.3%	2.5%	5.4%	2.5%	2.5%	12.3%
tr|I3LB86|I3LB86	0	0	0	2	2	0	4	8	0	0	0	3	2	0	19	140	0.0%	0.0%	0.0%	3.8%	10.5%	0.0%	12.9%	17.7%
tr|F1SRD0|F1SRD0	0	0	0	0	2	1	2	5	0	0	0	0	4	1	18	46	0.0%	0.0%	0.0%	0.0%	3.8%	1.4%	5.4%	9.3%
tr|F1SRC9|F1SRC9	0	0	0	0	2	1	0	3	0	0	0	0	4	1	0	9	0.0%	0.0%	0.0%	0.0%	9.6%	3.6%	0.0%	12.0%
tr|F1SDL2|F1SDL2	0	0	0	1	1	0	1	1	0	0	0	4	1	0	2	1	0.0%	0.0%	0.0%	1.5%	1.5%	0.0%	1.5%	1.9%
tr|F1RGC5|F1RGC5	0	0	0	1	2	0	1	1	0	0	0	3	2	0	1	1	0.0%	0.0%	0.0%	11.2%	25.2%	0.0%	11.2%	16.8%
tr|I3LGC0|I3LGC0	0	0	0	1	1	0	0	1	0	0	0	1	4	0	0	1	0.0%	0.0%	0.0%	4.7%	4.7%	0.0%	0.0%	3.8%
tr|I3L6U7|I3L6U7	0	0	0	0	0	1	0	0	0	0	0	0	0	5	0	0	0.0%	0.0%	0.0%	0.0%	0.0%	2.8%	0.0%	0.0%
tr|F1SLU8|F1SLU8	0	0	0	1	1	0	0	0	0	0	0	3	2	0	0	0	0.0%	0.0%	0.0%	2.9%	2.9%	0.0%	0.0%	0.0%
tr|F1SET6|F1SET6	0	0	0	1	1	0	0	0	0	0	0	3	2	0	0	0	0.0%	0.0%	0.0%	3.3%	3.3%	0.0%	0.0%	0.0%
tr|I3L855|I3L855	0	0	0	1	0	2	0	1	0	0	0	2	0	2	0	1	0.0%	0.0%	0.0%	4.6%	0.0%	4.6%	0.0%	5.1%
tr|Q2EN89|Q2EN89	0	0	0	0	0	3	0	0	0	0	0	0	0	4	0	0	0.0%	0.0%	0.0%	0.0%	0.0%	13.2%	0.0%	0.0%
tr|F1SHV9|F1SHV9	0	0	0	0	2	0	0	0	0	0	0	0	4	0	0	0	0.0%	0.0%	0.0%	0.0%	5.6%	0.0%	0.0%	0.0%
tr|F1RXG2|F1RXG2	0	0	0	1	1	0	0	0	0	0	0	2	2	0	0	0	0.0%	0.0%	0.0%	1.9%	1.5%	0.0%	0.0%	0.0%
Q29288	0	0	0	0	0	3	0	0	0	0	0	0	0	4	0	0	0.0%	0.0%	0.0%	0.0%	0.0%	24.0%	0.0%	0.0%
tr|K7GLL8|K7GLL8	0	0	0	0	0	3	0	0	0	0	0	0	0	4	0	0	0.0%	0.0%	0.0%	0.0%	0.0%	14.6%	0.0%	0.0%
tr|I3L5K9|I3L5K9	0	0	0	1	1	0	0	0	0	0	0	1	3	0	0	0	0.0%	0.0%	0.0%	12.0%	12.0%	0.0%	0.0%	0.0%
tr|I3LBX8|I3LBX8	0	0	2	1	1	0	2	3	0	0	8	2	1	0	45	24	0.0%	0.0%	6.2%	2.4%	2.4%	0.0%	14.8%	15.1%
tr|I3L8I4|I3L8I4	0	0	2	1	1	0	2	3	0	0	8	2	1	0	45	24	0.0%	0.0%	5.3%	2.1%	2.1%	0.0%	12.7%	12.9%
tr|F1SNF3|F1SNF3	0	0	2	1	1	0	2	3	0	0	8	2	1	0	45	24	0.0%	0.0%	5.8%	2.2%	2.2%	0.0%	13.9%	14.2%
Q29261	0	0	5	0	3	0	9	10	0	0	37	0	3	0	39	44	0.0%	0.0%	8.0%	0.0%	3.6%	0.0%	15.6%	21.6%
P00371	0	0	2	1	1	0	0	0	0	0	5	1	2	0	0	0	0.0%	0.0%	7.5%	3.7%	3.7%	0.0%	0.0%	0.0%
tr|F1SRY3|F1SRY3	0	0	6	0	3	0	10	10	0	0	170	0	3	0	40	44	0.0%	0.0%	10.3%	0.0%	3.6%	0.0%	17.2%	21.6%
Q06AU2	0	0	0	1	1	0	1	7	0	0	0	1	2	0	8	24	0.0%	0.0%	0.0%	8.2%	8.2%	0.0%	14.2%	41.0%
tr|F1SJM5|F1SJM5	0	0	0	1	1	0	1	3	0	0	0	1	2	0	8	8	0.0%	0.0%	0.0%	8.2%	8.2%	0.0%	14.3%	14.8%
tr|F1SAY0|F1SAY0	0	0	0	1	1	0	0	2	0	0	0	2	1	0	0	2	0.0%	0.0%	0.0%	0.9%	0.9%	0.0%	0.0%	3.9%
Reverse_tr|I3LCD2|I3LCD2	0	0	0	0	1	0	0	0	0	0	0	0	3	0	0	0	0.0%	0.0%	0.0%	0.0%	2.8%	0.0%	0.0%	0.0%
tr|Q8MHU0|Q8MHU0	0	0	0	1	1	0	0	0	0	0	0	1	2	0	0	0	0.0%	0.0%	0.0%	3.5%	3.5%	0.0%	0.0%	0.0%
tr|K7GNT1|K7GNT1	0	0	0	0	1	0	0	0	0	0	0	0	3	0	0	0	0.0%	0.0%	0.0%	0.0%	8.8%	0.0%	0.0%	0.0%
tr|F1RPE6|F1RPE6	0	0	0	0	1	0	0	0	0	0	0	0	3	0	0	0	0.0%	0.0%	0.0%	0.0%	2.8%	0.0%	0.0%	0.0%
tr|F1RZ55|F1RZ55	0	0	0	1	1	0	0	0	0	0	0	1	2	0	0	0	0.0%	0.0%	0.0%	6.5%	6.5%	0.0%	0.0%	0.0%
tr|F1RQU1|F1RQU1	0	0	0	1	1	0	0	0	0	0	0	2	1	0	0	0	0.0%	0.0%	0.0%	4.5%	4.5%	0.0%	0.0%	0.0%
tr|F1CNZ4|F1CNZ4	0	0	0	1	1	1	0	0	0	0	0	1	1	1	0	0	0.0%	0.0%	0.0%	2.8%	2.8%	2.8%	0.0%	0.0%
tr|I3LJ52|I3LJ52	0	0	0	0	2	0	0	0	0	0	0	0	3	0	0	0	0.0%	0.0%	0.0%	0.0%	5.7%	0.0%	0.0%	0.0%
tr|F1RTG7|F1RTG7	0	0	0	1	1	0	0	0	0	0	0	1	2	0	0	0	0.0%	0.0%	0.0%	6.1%	6.1%	0.0%	0.0%	0.0%
tr|I3LLJ2|I3LLJ2	0	0	0	0	1	0	0	0	0	0	0	0	3	0	0	0	0.0%	0.0%	0.0%	0.0%	4.6%	0.0%	0.0%	0.0%
P12069	0	0	0	0	1	0	0	0	0	0	0	0	3	0	0	0	0.0%	0.0%	0.0%	0.0%	8.1%	0.0%	0.0%	0.0%
P12068	0	0	0	0	1	0	0	0	0	0	0	0	3	0	0	0	0.0%	0.0%	0.0%	0.0%	8.2%	0.0%	0.0%	0.0%
P12067	0	0	0	0	1	0	0	0	0	0	0	0	3	0	0	0	0.0%	0.0%	0.0%	0.0%	9.4%	0.0%	0.0%	0.0%
tr|F1SDI6|F1SDI6	0	0	0	1	1	0	0	0	0	0	0	2	1	0	0	0	0.0%	0.0%	0.0%	2.9%	2.9%	0.0%	0.0%	0.0%
tr|I3L7K6|I3L7K6	0	0	0	0	2	1	0	0	0	0	0	0	2	1	0	0	0.0%	0.0%	0.0%	0.0%	3.0%	3.0%	0.0%	0.0%
Reverse_tr|I3LP18|I3LP18	0	0	0	0	0	1	0	0	0	0	0	0	0	3	0	0	0.0%	0.0%	0.0%	0.0%	0.0%	0.9%	0.0%	0.0%
tr|F1RTG6|F1RTG6	0	0	0	1	1	0	0	0	0	0	0	1	2	0	0	0	0.0%	0.0%	0.0%	3.7%	3.7%	0.0%	0.0%	0.0%
tr|F1SKA8|F1SKA8	0	0	0	1	0	0	0	0	0	0	0	3	0	0	0	0	0.0%	0.0%	0.0%	4.3%	0.0%	0.0%	0.0%	0.0%
tr|I3LDP5|I3LDP5	0	0	1	0	2	0	3	14	0	0	7	0	2	0	34	76	0.0%	0.0%	2.1%	0.0%	5.7%	0.0%	7.9%	23.7%
tr|I3LBB6|I3LBB6	0	0	0	1	0	0	5	12	0	0	0	2	0	0	43	76	0.0%	0.0%	0.0%	1.2%	0.0%	0.0%	8.3%	14.2%
tr|I6L6E1|I6L6E1	0	0	0	1	0	0	2	9	0	0	0	2	0	0	14	58	0.0%	0.0%	0.0%	2.7%	0.0%	0.0%	8.0%	31.7%
tr|K7GQA1|K7GQA1	0	0	0	0	1	0	1	3	0	0	0	0	2	0	1	14	0.0%	0.0%	0.0%	0.0%	4.0%	0.0%	4.0%	18.9%
tr|F1S3L9|F1S3L9	0	0	0	1	1	0	0	3	0	0	0	1	1	0	0	15	0.0%	0.0%	0.0%	1.3%	1.3%	0.0%	0.0%	6.6%
O19064	0	0	0	0	2	0	1	4	0	0	0	0	2	0	1	11	0.0%	0.0%	0.0%	0.0%	1.4%	0.0%	1.8%	7.3%
tr|J9JIK9|J9JIK9	0	0	0	0	2	0	0	3	0	0	0	0	2	0	0	7	0.0%	0.0%	0.0%	0.0%	1.5%	0.0%	0.0%	5.8%
tr|F1RG13|F1RG13	0	0	0	1	1	0	0	3	0	0	0	1	1	0	0	6	0.0%	0.0%	0.0%	0.6%	0.6%	0.0%	0.0%	3.4%
tr|F1SLG9|F1SLG9	0	0	0	1	1	0	1	0	0	0	0	1	1	0	6	0	0.0%	0.0%	0.0%	13.5%	13.5%	0.0%	13.5%	0.0%
tr|I3LKL3|I3LKL3	0	0	0	1	1	0	1	1	0	0	0	1	1	0	2	1	0.0%	0.0%	0.0%	3.8%	3.8%	0.0%	4.0%	4.0%
tr|F1RUN1|F1RUN1	0	0	0	1	1	0	1	0	0	0	0	1	1	0	2	0	0.0%	0.0%	0.0%	2.0%	2.0%	0.0%	2.0%	0.0%
tr|F1SGE0|F1SGE0	0	0	0	1	0	0	1	0	0	0	0	2	0	0	2	0	0.0%	0.0%	0.0%	5.2%	0.0%	0.0%	5.2%	0.0%
tr|F1S790|F1S790	0	0	0	0	2	0	0	1	0	0	0	0	2	0	0	1	0.0%	0.0%	0.0%	0.0%	6.7%	0.0%	0.0%	5.4%
tr|F1RPQ8|F1RPQ8	0	0	0	0	2	0	0	1	0	0	0	0	2	0	0	1	0.0%	0.0%	0.0%	0.0%	1.5%	0.0%	0.0%	1.5%

	Peptide Count								Spectral Count								Sequence Coverage							
	Whole Cell	Whole Cell	Whole Cell	Cilia Fraction	Cilia Fraction	Cilia Fraction	Cilia Fraction	Cilia Fraction	Whole Cell	Whole Cell	Whole Cell	Cilia Fraction	Cilia Fraction	Cilia Fraction	Cilia Fraction	Cilia Fraction	Whole Cell	Whole Cell	Whole Cell	Cilia Fraction	Cilia Fraction	Cilia Fraction	Cilia Fraction	Cilia Fraction
Sample#	1	2	6	4	3	5	7	8	1	2	6	4	3	5	7	8	1	2	6	4	3	5	7	8
**Accession**																								
tr|F1SPK3|F1SPK3	0	0	0	0	1	1	0	1	0	0	0	0	1	1	0	1	0.0%	0.0%	0.0%	0.0%	4.1%	4.1%	0.0%	4.1%
tr|F1SPA8|F1SPA8	0	0	0	1	0	0	0	0	0	0	0	2	0	0	0	0	0.0%	0.0%	0.0%	7.1%	0.0%	0.0%	0.0%	0.0%
tr|F1S0K4|F1S0K4	0	0	0	0	1	0	0	0	0	0	0	0	2	0	0	0	0.0%	0.0%	0.0%	0.0%	1.7%	0.0%	0.0%	0.0%
tr|F1RL47|F1RL47	0	0	0	0	1	1	0	0	0	0	0	0	1	1	0	0	0.0%	0.0%	0.0%	0.0%	3.7%	3.7%	0.0%	0.0%
Reverse_tr|I3LL04|I3LL04	0	0	0	1	0	0	0	0	0	0	0	2	0	0	0	0	0.0%	0.0%	0.0%	1.9%	0.0%	0.0%	0.0%	0.0%
tr|F1S0K7|F1S0K7	0	0	0	0	1	0	0	0	0	0	0	0	2	0	0	0	0.0%	0.0%	0.0%	0.0%	1.5%	0.0%	0.0%	0.0%
tr|F1SAK2|F1SAK2	0	0	0	1	0	0	0	0	0	0	0	2	0	0	0	0	0.0%	0.0%	0.0%	0.4%	0.0%	0.0%	0.0%	0.0%
tr|F1RGF2|F1RGF2	0	0	0	1	1	0	0	0	0	0	0	1	1	0	0	0	0.0%	0.0%	0.0%	9.7%	9.7%	0.0%	0.0%	0.0%
tr|K7GRK0|K7GRK0	0	0	0	1	0	0	0	0	0	0	0	2	0	0	0	0	0.0%	0.0%	0.0%	7.1%	0.0%	0.0%	0.0%	0.0%
tr|F1SRR8|F1SRR8	0	0	0	1	1	0	0	0	0	0	0	1	1	0	0	0	0.0%	0.0%	0.0%	0.7%	0.7%	0.0%	0.0%	0.0%
tr|F1SPK1|F1SPK1	0	0	0	1	1	0	0	0	0	0	0	1	1	0	0	0	0.0%	0.0%	0.0%	0.9%	0.4%	0.0%	0.0%	0.0%
tr|F1SQ40|F1SQ40	0	0	0	1	1	0	0	0	0	0	0	1	1	0	0	0	0.0%	0.0%	0.0%	3.8%	3.8%	0.0%	0.0%	0.0%
tr|F1S0K8|F1S0K8	0	0	0	0	1	0	0	0	0	0	0	0	2	0	0	0	0.0%	0.0%	0.0%	0.0%	2.1%	0.0%	0.0%	0.0%
tr|F1SC07|F1SC07	0	0	0	1	0	0	0	0	0	0	0	2	0	0	0	0	0.0%	0.0%	0.0%	0.2%	0.0%	0.0%	0.0%	0.0%
tr|I3LK92|I3LK92	0	0	0	1	1	0	0	0	0	0	0	1	1	0	0	0	0.0%	0.0%	0.0%	12.1%	12.1%	0.0%	0.0%	0.0%
tr|F1S8V8|F1S8V8	0	0	0	1	0	0	0	0	0	0	0	2	0	0	0	0	0.0%	0.0%	0.0%	0.8%	0.0%	0.0%	0.0%	0.0%
tr|F1RXF9|F1RXF9	0	0	0	1	0	0	0	0	0	0	0	2	0	0	0	0	0.0%	0.0%	0.0%	2.0%	0.0%	0.0%	0.0%	0.0%
tr|F1RXG1|F1RXG1	0	0	0	1	0	0	0	0	0	0	0	2	0	0	0	0	0.0%	0.0%	0.0%	2.0%	0.0%	0.0%	0.0%	0.0%
tr|I3LUM7|I3LUM7	0	0	0	0	1	0	0	0	0	0	0	0	2	0	0	0	0.0%	0.0%	0.0%	0.0%	1.7%	0.0%	0.0%	0.0%
tr|F1RMH5|F1RMH5	0	0	0	1	0	0	0	0	0	0	0	2	0	0	0	0	0.0%	0.0%	0.0%	1.9%	0.0%	0.0%	0.0%	0.0%
tr|K7GLK4|K7GLK4	0	0	0	1	0	0	0	0	0	0	0	2	0	0	0	0	0.0%	0.0%	0.0%	8.6%	0.0%	0.0%	0.0%	0.0%
tr|F1S0K6|F1S0K6	0	0	0	0	1	0	0	0	0	0	0	0	2	0	0	0	0.0%	0.0%	0.0%	0.0%	1.5%	0.0%	0.0%	0.0%
tr|I3LUJ7|I3LUJ7	0	0	0	0	1	0	0	0	0	0	0	0	2	0	0	0	0.0%	0.0%	0.0%	0.0%	1.7%	0.0%	0.0%	0.0%
tr|F1SLL2|F1SLL2	0	0	0	1	0	0	0	0	0	0	0	2	0	0	0	0	0.0%	0.0%	0.0%	2.3%	0.0%	0.0%	0.0%	0.0%
tr|I3LMY0|I3LMY0	0	0	1	0	1	0	2	10	0	0	4	0	1	0	14	55	0.0%	0.0%	2.5%	0.0%	1.1%	0.0%	4.8%	14.1%
tr|K7GKS4|K7GKS4	0	0	1	0	1	0	0	2	0	0	1	0	1	0	0	8	0.0%	0.0%	6.8%	0.0%	6.8%	0.0%	0.0%	14.1%
tr|F1S7D3|F1S7D3	0	0	1	1	0	0	1	0	0	0	1	1	0	0	6	0	0.0%	0.0%	4.9%	12.1%	0.0%	0.0%	5.8%	0.0%
P51781	0	0	1	1	0	0	1	0	0	0	1	1	0	0	6	0	0.0%	0.0%	5.0%	12.2%	0.0%	0.0%	5.9%	0.0%
tr|F1SBQ5|F1SBQ5	0	0	2	1	0	0	1	3	0	0	7	1	0	0	12	29	0.0%	0.0%	8.8%	2.0%	0.0%	0.0%	3.2%	7.4%
tr|F1RNK3|F1RNK3	0	0	1	0	1	0	1	2	0	0	1	0	1	0	1	4	0.0%	0.0%	7.1%	0.0%	7.1%	0.0%	7.1%	12.7%
tr|F1S3B4|F1S3B4	0	0	2	0	1	0	1	2	0	0	9	0	1	0	15	12	0.0%	0.0%	4.7%	0.0%	7.0%	0.0%	6.4%	24.5%
tr|K7GQU3|K7GQU3	0	0	1	1	0	0	2	1	0	0	7	1	0	0	14	1	0.0%	0.0%	13.6%	13.6%	0.0%	0.0%	24.7%	13.6%
tr|F1S7C9|F1S7C9	0	0	1	1	0	0	0	0	0	0	1	1	0	0	0	0	0.0%	0.0%	4.9%	12.1%	0.0%	0.0%	0.0%	0.0%
tr|I3LMD8|I3LMD8	0	0	1	0	1	0	0	1	0	0	4	0	1	0	0	1	0.0%	0.0%	0.9%	0.0%	1.0%	0.0%	0.0%	1.1%
tr|F1SE22|F1SE22	0	0	1	0	1	0	0	0	0	0	2	0	1	0	0	0	0.0%	0.0%	3.7%	0.0%	2.5%	0.0%	0.0%	0.0%
tr|F1RZ82|F1RZ82	0	0	1	1	0	0	0	0	0	0	2	1	0	0	0	0	0.0%	0.0%	1.1%	1.4%	0.0%	0.0%	0.0%	0.0%
tr|F1RRR7|F1RRR7	0	0	1	1	0	0	0	0	0	0	4	1	0	0	0	0	0.0%	0.0%	5.4%	2.9%	0.0%	0.0%	0.0%	0.0%
tr|F1SUH2|F1SUH2	0	0	2	0	1	0	0	0	0	0	5	0	1	0	0	0	0.0%	0.0%	8.5%	0.0%	4.9%	0.0%	0.0%	0.0%
tr|O77773|O77773	0	0	0	1	0	0	2	11	0	0	0	1	0	0	38	45	0.0%	0.0%	0.0%	1.0%	0.0%	0.0%	3.0%	16.2%
Q8SQ34	0	0	0	1	0	0	1	3	0	0	0	1	0	0	8	59	0.0%	0.0%	0.0%	3.6%	0.0%	0.0%	9.7%	35.6%
tr|K7GKQ2|K7GKQ2	0	0	0	1	0	0	1	3	0	0	0	1	0	0	8	59	0.0%	0.0%	0.0%	3.8%	0.0%	0.0%	10.3%	37.9%
tr|F1RRT6|F1RRT6	0	0	0	0	1	0	0	2	0	0	0	0	1	0	0	17	0.0%	0.0%	0.0%	0.0%	5.2%	0.0%	0.0%	18.9%
tr|F1S1J3|F1S1J3	0	0	0	1	0	0	0	4	0	0	0	1	0	0	0	13	0.0%	0.0%	0.0%	2.2%	0.0%	0.0%	0.0%	12.9%
tr|K7GS65|K7GS65	0	0	0	0	0	1	0	1	0	0	0	0	0	1	0	12	0.0%	0.0%	0.0%	0.0%	0.0%	15.7%	0.0%	14.3%
tr|F1S3A1|F1S3A1	0	0	0	1	0	0	3	2	0	0	0	1	0	0	5	5	0.0%	0.0%	0.0%	1.2%	0.0%	0.0%	3.2%	3.0%
tr|F1RLT0|F1RLT0	0	0	0	0	1	0	1	3	0	0	0	0	1	0	2	7	0.0%	0.0%	0.0%	0.0%	1.4%	0.0%	1.6%	4.5%
tr|F1SMP5|F1SMP5	0	0	0	0	1	0	1	1	0	0	0	0	1	0	4	3	0.0%	0.0%	0.0%	0.0%	2.0%	0.0%	1.4%	1.4%
tr|F1RHN2|F1RHN2	0	0	0	0	1	0	0	4	0	0	0	0	1	0	0	7	0.0%	0.0%	0.0%	0.0%	2.6%	0.0%	0.0%	7.2%
tr|F1RN32|F1RN32	0	0	0	1	0	0	1	1	0	0	0	1	0	0	4	2	0.0%	0.0%	0.0%	2.7%	0.0%	0.0%	2.4%	4.5%
tr|F1SAI9|F1SAI9	0	0	0	1	0	0	2	3	0	0	0	1	0	0	2	4	0.0%	0.0%	0.0%	7.2%	0.0%	0.0%	7.2%	9.6%
tr|I3LVB7|I3LVB7	0	0	0	1	0	0	2	3	0	0	0	1	0	0	2	4	0.0%	0.0%	0.0%	6.9%	0.0%	0.0%	6.9%	9.1%
tr|F2Z5G0|F2Z5G0	0	0	0	0	1	0	0	1	0	0	0	0	1	0	0	5	0.0%	0.0%	0.0%	0.0%	5.0%	0.0%	0.0%	5.7%
Q9GLP1	0	0	0	0	0	1	1	2	0	0	0	0	0	1	2	3	0.0%	0.0%	0.0%	0.0%	0.0%	0.6%	0.6%	1.2%
tr|F1S9Z2|F1S9Z2	0	0	0	0	1	0	1	1	0	0	0	0	1	0	2	3	0.0%	0.0%	0.0%	0.0%	2.9%	0.0%	2.2%	5.0%
tr|F1RPW2|F1RPW2	0	0	0	0	0	1	1	2	0	0	0	0	0	1	2	3	0.0%	0.0%	0.0%	0.0%	0.0%	0.6%	0.6%	1.2%
tr|F1STC7|F1STC7	0	0	0	1	0	0	0	2	0	0	0	1	0	0	0	5	0.0%	0.0%	0.0%	1.5%	0.0%	0.0%	0.0%	8.4%
tr|F1ST93|F1ST93	0	0	0	1	0	0	1	1	0	0	0	1	0	0	4	1	0.0%	0.0%	0.0%	2.5%	0.0%	0.0%	4.1%	4.1%
tr|K7GQB9|K7GQB9	0	0	0	1	0	0	1	0	0	0	0	1	0	0	4	0	0.0%	0.0%	0.0%	4.8%	0.0%	0.0%	4.2%	0.0%
tr|F1S5M5|F1S5M5	0	0	0	1	0	0	0	1	0	0	0	1	0	0	0	4	0.0%	0.0%	0.0%	4.6%	0.0%	0.0%	0.0%	4.1%
tr|F1RTL2|F1RTL2	0	0	0	1	0	0	0	2	0	0	0	1	0	0	0	3	0.0%	0.0%	0.0%	1.9%	0.0%	0.0%	0.0%	5.0%
tr|I3LFJ4|I3LFJ4	0	0	0	0	1	0	1	0	0	0	0	0	1	0	3	0	0.0%	0.0%	0.0%	0.0%	1.7%	0.0%	2.1%	0.0%
tr|I3LFV7|I3LFV7	0	0	0	0	1	0	1	0	0	0	0	0	1	0	3	0	0.0%	0.0%	0.0%	0.0%	2.2%	0.0%	2.8%	0.0%
tr|F1RPH3|F1RPH3	0	0	0	0	1	0	0	2	0	0	0	0	1	0	0	2	0.0%	0.0%	0.0%	0.0%	1.7%	0.0%	0.0%	3.2%
tr|K7GT44|K7GT44	0	0	0	0	1	0	0	2	0	0	0	0	1	0	0	2	0.0%	0.0%	0.0%	0.0%	1.7%	0.0%	0.0%	3.2%
tr|F1RWL1|F1RWL1	0	0	0	0	1	0	0	1	0	0	0	0	1	0	0	2	0.0%	0.0%	0.0%	0.0%	2.8%	0.0%	0.0%	4.1%
tr|F1RW53|F1RW53	0	0	0	1	0	0	1	1	0	0	0	1	0	0	1	1	0.0%	0.0%	0.0%	9.5%	0.0%	0.0%	9.5%	7.9%
tr|K7GRX8|K7GRX8	0	0	0	0	1	0	0	1	0	0	0	0	1	0	0	1	0.0%	0.0%	0.0%	0.0%	21.7%	0.0%	0.0%	26.1%
tr|F1S788|F1S788	0	0	0	0	1	0	1	0	0	0	0	0	1	0	1	0	0.0%	0.0%	0.0%	0.0%	2.7%	0.0%	2.7%	0.0%
tr|I3LPH3|I3LPH3	0	0	0	0	1	0	0	1	0	0	0	0	1	0	0	1	0.0%	0.0%	0.0%	0.0%	7.5%	0.0%	0.0%	7.5%
tr|F1SKC4|F1SKC4	0	0	0	1	0	0	1	0	0	0	0	1	0	0	1	0	0.0%	0.0%	0.0%	4.2%	0.0%	0.0%	6.0%	0.0%
Reverse_tr|I3LB53|I3LB53	0	0	0	0	0	1	0	0	0	0	0	0	0	1	0	0	0.0%	0.0%	0.0%	0.0%	0.0%	2.7%	0.0%	0.0%
tr|I3L9A8|I3L9A8	0	0	0	0	1	0	0	0	0	0	0	0	1	0	0	0	0.0%	0.0%	0.0%	0.0%	1.1%	0.0%	0.0%	0.0%
tr|A5GFR1|A5GFR1	0	0	0	1	0	0	0	0	0	0	0	1	0	0	0	0	0.0%	0.0%	0.0%	1.7%	0.0%	0.0%	0.0%	0.0%
tr|F1RL93|F1RL93	0	0	0	0	1	0	0	0	0	0	0	0	1	0	0	0	0.0%	0.0%	0.0%	0.0%	2.2%	0.0%	0.0%	0.0%
tr|F1RIQ8|F1RIQ8	0	0	0	1	0	0	0	0	0	0	0	1	0	0	0	0	0.0%	0.0%	0.0%	4.7%	0.0%	0.0%	0.0%	0.0%
Reverse_tr|F1S6Z8|F1S6Z8	0	0	0	1	0	0	0	0	0	0	0	1	0	0	0	0	0.0%	0.0%	0.0%	5.7%	0.0%	0.0%	0.0%	0.0%
tr|K7GNV3|K7GNV3	0	0	0	0	1	0	0	0	0	0	0	0	1	0	0	0	0.0%	0.0%	0.0%	0.0%	6.8%	0.0%	0.0%	0.0%
Reverse_tr|F1SP77|F1SP77	0	0	0	0	1	0	0	0	0	0	0	0	1	0	0	0	0.0%	0.0%	0.0%	0.0%	2.5%	0.0%	0.0%	0.0%
tr|F1SNW4|F1SNW4	0	0	0	0	1	0	0	0	0	0	0	0	1	0	0	0	0.0%	0.0%	0.0%	0.0%	6.6%	0.0%	0.0%	0.0%
tr|I3LAJ5|I3LAJ5	0	0	0	0	1	0	0	0	0	0	0	0	1	0	0	0	0.0%	0.0%	0.0%	0.0%	0.8%	0.0%	0.0%	0.0%
P37176	0	0	0	0	1	0	0	0	0	0	0	0	1	0	0	0	0.0%	0.0%	0.0%	0.0%	4.9%	0.0%	0.0%	0.0%

	Peptide Count								Spectral Count								Sequence Coverage							
	Whole Cell	Whole Cell	Whole Cell	Cilia Fraction	Cilia Fraction	Cilia Fraction	Cilia Fraction	Cilia Fraction	Whole Cell	Whole Cell	Whole Cell	Cilia Fraction	Cilia Fraction	Cilia Fraction	Cilia Fraction	Cilia Fraction	Whole Cell	Whole Cell	Whole Cell	Cilia Fraction	Cilia Fraction	Cilia Fraction	Cilia Fraction	Cilia Fraction
**Sample#**	**1**	**2**	**6**	**4**	**3**	**5**	**7**	**8**	**1**	**2**	**6**	**4**	**3**	**5**	**7**	**8**	**1**	**2**	**6**	**4**	**3**	**5**	**7**	**8**
**Accession**																								
tr|I3LUG6|I3LUG6	0	0	0	0	1	0	0	0	0	0	0	0	1	0	0	0	0.0%	0.0%	0.0%	0.0%	6.6%	0.0%	0.0%	0.0%
tr|F1SCG2|F1SCG2	0	0	0	0	1	0	0	0	0	0	0	0	1	0	0	0	0.0%	0.0%	0.0%	0.0%	2.3%	0.0%	0.0%	0.0%
Reverse_tr|F1SSN6|F1SSN6	0	0	0	0	1	0	0	0	0	0	0	0	1	0	0	0	0.0%	0.0%	0.0%	0.0%	2.9%	0.0%	0.0%	0.0%
tr|I3LDR6|I3LDR6	0	0	0	0	0	1	0	0	0	0	0	0	0	1	0	0	0.0%	0.0%	0.0%	0.0%	0.0%	2.0%	0.0%	0.0%
tr|F1S414|F1S414	0	0	0	0	1	0	0	0	0	0	0	0	1	0	0	0	0.0%	0.0%	0.0%	0.0%	1.3%	0.0%	0.0%	0.0%
Reverse_tr|F1S6B4|F1S6B4	0	0	0	1	0	0	0	0	0	0	0	1	0	0	0	0	0.0%	0.0%	0.0%	2.1%	0.0%	0.0%	0.0%	0.0%
tr|F1S6V0|F1S6V0	0	0	0	1	0	0	0	0	0	0	0	1	0	0	0	0	0.0%	0.0%	0.0%	3.4%	0.0%	0.0%	0.0%	0.0%
Reverse_tr|F1RZI9|F1RZI9	0	0	0	1	0	0	0	0	0	0	0	1	0	0	0	0	0.0%	0.0%	0.0%	1.1%	0.0%	0.0%	0.0%	0.0%
tr|C3VML0|C3VML0	0	0	0	0	1	0	0	0	0	0	0	0	1	0	0	0	0.0%	0.0%	0.0%	0.0%	5.7%	0.0%	0.0%	0.0%
Reverse_tr|K7GQG6|K7GQG6	0	0	0	0	1	0	0	0	0	0	0	0	1	0	0	0	0.0%	0.0%	0.0%	0.0%	4.3%	0.0%	0.0%	0.0%
tr|F1SEV7|F1SEV7	0	0	0	0	0	1	0	0	0	0	0	0	0	1	0	0	0.0%	0.0%	0.0%	0.0%	0.0%	5.5%	0.0%	0.0%
tr|B6DZ39|B6DZ39	0	0	0	0	1	0	0	0	0	0	0	0	1	0	0	0	0.0%	0.0%	0.0%	0.0%	3.9%	0.0%	0.0%	0.0%
tr|F1RQP7|F1RQP7	0	0	0	1	0	0	0	0	0	0	0	1	0	0	0	0	0.0%	0.0%	0.0%	1.5%	0.0%	0.0%	0.0%	0.0%
tr|F1SE88|F1SE88	0	0	0	0	1	0	0	0	0	0	0	0	1	0	0	0	0.0%	0.0%	0.0%	0.0%	1.1%	0.0%	0.0%	0.0%
Reverse_tr|F1SV69|F1SV69	0	0	0	0	1	0	0	0	0	0	0	0	1	0	0	0	0.0%	0.0%	0.0%	0.0%	3.2%	0.0%	0.0%	0.0%
Reverse_tr|F1RVD6|F1RVD6	0	0	0	0	0	1	0	0	0	0	0	0	0	1	0	0	0.0%	0.0%	0.0%	0.0%	0.0%	3.2%	0.0%	0.0%
Reverse_tr|I3LGC6|I3LGC6	0	0	0	0	1	0	0	0	0	0	0	0	1	0	0	0	0.0%	0.0%	0.0%	0.0%	0.6%	0.0%	0.0%	0.0%
Reverse_tr|F1SIP0|F1SIP0	0	0	0	0	1	0	0	0	0	0	0	0	1	0	0	0	0.0%	0.0%	0.0%	0.0%	5.5%	0.0%	0.0%	0.0%
Reverse_tr|F1S6P2|F1S6P2	0	0	0	1	0	0	0	0	0	0	0	1	0	0	0	0	0.0%	0.0%	0.0%	3.3%	0.0%	0.0%	0.0%	0.0%
tr|F1SRM1|F1SRM1	0	0	0	0	1	0	0	0	0	0	0	0	1	0	0	0	0.0%	0.0%	0.0%	0.0%	0.7%	0.0%	0.0%	0.0%
tr|F1SNV8|F1SNV8	0	0	0	1	0	0	0	0	0	0	0	1	0	0	0	0	0.0%	0.0%	0.0%	4.1%	0.0%	0.0%	0.0%	0.0%
tr|F1S012|F1S012	0	0	0	0	1	0	0	0	0	0	0	0	1	0	0	0	0.0%	0.0%	0.0%	0.0%	3.2%	0.0%	0.0%	0.0%
tr|F1RS03|F1RS03	0	0	0	0	1	0	0	0	0	0	0	0	1	0	0	0	0.0%	0.0%	0.0%	0.0%	4.9%	0.0%	0.0%	0.0%
tr|F1S887|F1S887	0	0	0	1	0	0	0	0	0	0	0	1	0	0	0	0	0.0%	0.0%	0.0%	3.3%	0.0%	0.0%	0.0%	0.0%
tr|F1RKK2|F1RKK2	0	0	0	0	1	0	0	0	0	0	0	0	1	0	0	0	0.0%	0.0%	0.0%	0.0%	0.7%	0.0%	0.0%	0.0%
tr|I3L905|I3L905	0	0	0	0	1	0	0	0	0	0	0	0	1	0	0	0	0.0%	0.0%	0.0%	0.0%	16.3%	0.0%	0.0%	0.0%
tr|K7GP73|K7GP73	0	0	0	1	0	0	0	0	0	0	0	1	0	0	0	0	0.0%	0.0%	0.0%	3.3%	0.0%	0.0%	0.0%	0.0%
tr|I3LNM9|I3LNM9	0	0	0	1	0	0	0	0	0	0	0	1	0	0	0	0	0.0%	0.0%	0.0%	2.7%	0.0%	0.0%	0.0%	0.0%
tr|K7GMH0|K7GMH0	0	0	0	0	1	0	0	0	0	0	0	0	1	0	0	0	0.0%	0.0%	0.0%	0.0%	0.9%	0.0%	0.0%	0.0%
tr|K7GNV8|K7GNV8	0	0	0	1	0	0	0	0	0	0	0	1	0	0	0	0	0.0%	0.0%	0.0%	6.0%	0.0%	0.0%	0.0%	0.0%
tr|I3LJU9|I3LJU9	0	0	0	0	0	1	0	0	0	0	0	0	0	1	0	0	0.0%	0.0%	0.0%	0.0%	0.0%	2.1%	0.0%	0.0%
tr|I3LCJ4|I3LCJ4	0	0	0	0	1	0	0	0	0	0	0	0	1	0	0	0	0.0%	0.0%	0.0%	0.0%	4.6%	0.0%	0.0%	0.0%
Reverse_tr|F1SSE6|F1SSE6	0	0	0	0	1	0	0	0	0	0	0	0	1	0	0	0	0.0%	0.0%	0.0%	0.0%	7.2%	0.0%	0.0%	0.0%
tr|F1S6V1|F1S6V1	0	0	0	0	1	0	0	0	0	0	0	0	1	0	0	0	0.0%	0.0%	0.0%	0.0%	1.0%	0.0%	0.0%	0.0%
tr|F1SJS4|F1SJS4	0	0	0	0	1	0	0	0	0	0	0	0	1	0	0	0	0.0%	0.0%	0.0%	0.0%	0.5%	0.0%	0.0%	0.0%
Reverse_sp|Q9MZS9|KMO	0	0	0	1	0	0	0	0	0	0	0	1	0	0	0	0	0.0%	0.0%	0.0%	3.8%	0.0%	0.0%	0.0%	0.0%
tr|F1SJB9|F1SJB9	0	0	0	1	0	0	0	0	0	0	0	1	0	0	0	0	0.0%	0.0%	0.0%	3.6%	0.0%	0.0%	0.0%	0.0%
tr|F1SDM5|F1SDM5	0	0	0	0	1	0	0	0	0	0	0	0	1	0	0	0	0.0%	0.0%	0.0%	0.0%	1.6%	0.0%	0.0%	0.0%
tr|F1RV90|F1RV90	0	0	0	0	1	0	0	0	0	0	0	0	1	0	0	0	0.0%	0.0%	0.0%	0.0%	2.7%	0.0%	0.0%	0.0%
tr|I3LP68|I3LP68	0	0	0	0	1	0	0	0	0	0	0	0	1	0	0	0	0.0%	0.0%	0.0%	0.0%	1.5%	0.0%	0.0%	0.0%
Reverse_tr|F1SBJ0|F1SBJ0	0	0	0	0	1	0	0	0	0	0	0	0	1	0	0	0	0.0%	0.0%	0.0%	0.0%	4.2%	0.0%	0.0%	0.0%
Reverse_tr|I3LU47|I3LU47	0	0	0	0	1	0	0	0	0	0	0	0	1	0	0	0	0.0%	0.0%	0.0%	0.0%	11.7%	0.0%	0.0%	0.0%
tr|K7GKW1|K7GKW1	0	0	0	0	1	0	0	0	0	0	0	0	1	0	0	0	0.0%	0.0%	0.0%	0.0%	6.3%	0.0%	0.0%	0.0%
tr|K7GQZ6|K7GQZ6	0	0	0	0	1	0	0	0	0	0	0	0	1	0	0	0	0.0%	0.0%	0.0%	0.0%	7.5%	0.0%	0.0%	0.0%
tr|F1RSK2|F1RSK2	0	0	0	0	1	0	0	0	0	0	0	0	1	0	0	0	0.0%	0.0%	0.0%	0.0%	1.2%	0.0%	0.0%	0.0%
tr|I3LPB2|I3LPB2	0	0	0	0	1	0	0	0	0	0	0	0	1	0	0	0	0.0%	0.0%	0.0%	0.0%	2.7%	0.0%	0.0%	0.0%
Reverse_tr|F1SIP7|F1SIP7	0	0	0	0	0	1	0	0	0	0	0	0	0	1	0	0	0.0%	0.0%	0.0%	0.0%	0.0%	5.1%	0.0%	0.0%
Q29043	0	0	0	1	0	0	0	0	0	0	0	1	0	0	0	0	0.0%	0.0%	0.0%	4.7%	0.0%	0.0%	0.0%	0.0%
Reverse_tr|F1RFL3|F1RFL3	0	0	0	0	0	1	0	0	0	0	0	0	0	1	0	0	0.0%	0.0%	0.0%	0.0%	0.0%	10.2%	0.0%	0.0%
tr|F1SHV8|F1SHV8	0	0	0	0	1	0	0	0	0	0	0	0	1	0	0	0	0.0%	0.0%	0.0%	0.0%	2.0%	0.0%	0.0%	0.0%
tr|F1SDH4|F1SDH4	0	0	0	1	0	0	0	0	0	0	0	1	0	0	0	0	0.0%	0.0%	0.0%	4.8%	0.0%	0.0%	0.0%	0.0%
tr|F1SLE9|F1SLE9	0	0	0	0	1	0	0	0	0	0	0	0	1	0	0	0	0.0%	0.0%	0.0%	0.0%	3.2%	0.0%	0.0%	0.0%
Reverse_tr|F1RU59|F1RU59	0	0	0	0	1	0	0	0	0	0	0	0	1	0	0	0	0.0%	0.0%	0.0%	0.0%	0.6%	0.0%	0.0%	0.0%
tr|K7GQF2|K7GQF2	0	0	0	0	1	0	0	0	0	0	0	0	1	0	0	0	0.0%	0.0%	0.0%	0.0%	5.2%	0.0%	0.0%	0.0%
tr|F1SIH2|F1SIH2	0	0	0	0	1	0	0	0	0	0	0	0	1	0	0	0	0.0%	0.0%	0.0%	0.0%	1.2%	0.0%	0.0%	0.0%
tr|I3L932|I3L932	0	0	0	1	0	0	0	0	0	0	0	1	0	0	0	0	0.0%	0.0%	0.0%	3.6%	0.0%	0.0%	0.0%	0.0%
tr|I3LKZ5|I3LKZ5	0	0	0	1	0	0	0	0	0	0	0	1	0	0	0	0	0.0%	0.0%	0.0%	0.2%	0.0%	0.0%	0.0%	0.0%
tr|F1RJQ6|F1RJQ6	0	0	0	1	0	0	0	0	0	0	0	1	0	0	0	0	0.0%	0.0%	0.0%	2.4%	0.0%	0.0%	0.0%	0.0%
Reverse_tr|I3LEH7|I3LEH7	0	0	0	0	0	1	0	0	0	0	0	0	0	1	0	0	0.0%	0.0%	0.0%	0.0%	0.0%	5.1%	0.0%	0.0%
tr|F1SQN2|F1SQN2	0	0	0	0	1	0	0	0	0	0	0	0	1	0	0	0	0.0%	0.0%	0.0%	0.0%	0.4%	0.0%	0.0%	0.0%
tr|F1REZ4|F1REZ4	0	0	0	0	1	0	0	0	0	0	0	0	1	0	0	0	0.0%	0.0%	0.0%	0.0%	4.1%	0.0%	0.0%	0.0%
tr|F1SID3|F1SID3	0	0	0	1	0	0	0	0	0	0	0	1	0	0	0	0	0.0%	0.0%	0.0%	5.8%	0.0%	0.0%	0.0%	0.0%
tr|I3LK96|I3LK96	0	0	0	0	1	0	0	0	0	0	0	0	1	0	0	0	0.0%	0.0%	0.0%	0.0%	1.3%	0.0%	0.0%	0.0%
tr|F1SMI5|F1SMI5	0	0	0	0	0	1	0	0	0	0	0	0	0	1	0	0	0.0%	0.0%	0.0%	0.0%	0.0%	1.8%	0.0%	0.0%
tr|F1RQ28|F1RQ28	0	0	0	0	1	0	0	0	0	0	0	0	1	0	0	0	0.0%	0.0%	0.0%	0.0%	2.8%	0.0%	0.0%	0.0%
Reverse_tr|I3L908|I3L908	0	0	0	0	1	0	0	0	0	0	0	0	1	0	0	0	0.0%	0.0%	0.0%	0.0%	2.3%	0.0%	0.0%	0.0%
tr|F1S0P6|F1S0P6	0	0	0	0	1	0	0	0	0	0	0	0	1	0	0	0	0.0%	0.0%	0.0%	0.0%	3.2%	0.0%	0.0%	0.0%
tr|F1RFH8|F1RFH8	0	0	0	1	0	0	0	0	0	0	0	1	0	0	0	0	0.0%	0.0%	0.0%	3.5%	0.0%	0.0%	0.0%	0.0%
tr|F1RXN8|F1RXN8	0	0	0	0	1	0	0	0	0	0	0	0	1	0	0	0	0.0%	0.0%	0.0%	0.0%	6.9%	0.0%	0.0%	0.0%
tr|F1SIE9|F1SIE9	0	0	0	1	0	0	0	0	0	0	0	1	0	0	0	0	0.0%	0.0%	0.0%	2.1%	0.0%	0.0%	0.0%	0.0%
tr|F1RY21|F1RY21	0	0	0	0	1	0	0	0	0	0	0	0	1	0	0	0	0.0%	0.0%	0.0%	0.0%	1.9%	0.0%	0.0%	0.0%
tr|K7GPF7|K7GPF7	0	0	0	0	1	0	0	0	0	0	0	0	1	0	0	0	0.0%	0.0%	0.0%	0.0%	11.8%	0.0%	0.0%	0.0%
tr|I3L7W7|I3L7W7	0	0	0	0	1	0	0	0	0	0	0	0	1	0	0	0	0.0%	0.0%	0.0%	0.0%	0.5%	0.0%	0.0%	0.0%
tr|I3LRZ5|I3LRZ5	0	0	0	0	1	0	0	0	0	0	0	0	1	0	0	0	0.0%	0.0%	0.0%	0.0%	12.6%	0.0%	0.0%	0.0%
tr|F1S367|F1S367	0	0	0	0	1	0	0	0	0	0	0	0	1	0	0	0	0.0%	0.0%	0.0%	0.0%	6.5%	0.0%	0.0%	0.0%
tr|K7GMB7|K7GMB7	0	0	0	0	1	0	0	0	0	0	0	0	1	0	0	0	0.0%	0.0%	0.0%	0.0%	3.7%	0.0%	0.0%	0.0%
tr|F1STH4|F1STH4	0	0	0	1	0	0	0	0	0	0	0	1	0	0	0	0	0.0%	0.0%	0.0%	2.1%	0.0%	0.0%	0.0%	0.0%
Reverse_tr|F1S959|F1S959	0	0	0	0	0	1	0	0	0	0	0	0	0	1	0	0	0.0%	0.0%	0.0%	0.0%	0.0%	1.3%	0.0%	0.0%
tr|K7GQJ9|K7GQJ9	0	0	1	0	1	0	3	16	0	0	4	0	1	0	63	148	0.0%	0.0%	6.1%	0.0%	9.5%	0.0%	13.8%	43.8%
tr|I3L9F1|I3L9F1	0	0	1	1	0	0	2	6	0	0	8	1	0	0	88	81	0.0%	0.0%	5.0%	5.0%	0.0%	0.0%	10.6%	17.9%
tr|F1S3J9|F1S3J9	0	1	2	3	2	2	3	8	0	1	7	20	4	4	40	38	0.0%	3.2%	5.8%	5.8%	5.8%	3.2%	6.3%	9.7%

	Peptide Count								Spectral Count								Sequence Coverage							
	Whole Cell	Whole Cell	Whole Cell	Cilia Fraction	Cilia Fraction	Cilia Fraction	Cilia Fraction	Cilia Fraction	Whole Cell	Whole Cell	Whole Cell	Cilia Fraction	Cilia Fraction	Cilia Fraction	Cilia Fraction	Cilia Fraction	Whole Cell	Whole Cell	Whole Cell	Cilia Fraction	Cilia Fraction	Cilia Fraction	Cilia Fraction	Cilia Fraction
Sample#	1	2	6	4	3	5	7	8	1	2	6	4	3	5	7	8	1	2	6	4	3	5	7	8
**Accession**																								
tr|I3LS30|I3LS30	3	0	1	3	4	3	7	12	3	0	2	13	21	3	81	145	4.3%	0.0%	3.0%	4.9%	8.2%	4.5%	15.4%	19.6%
tr|F1SK03|F1SK03	5	4	4	15	21	7	30	46	8	9	30	93	80	15	1312	884	11.6%	4.9%	6.6%	17.8%	23.6%	9.0%	36.9%	45.8%
tr|I3LD66|I3LD66	0	1	0	1	1	0	1	3	0	1	0	7	4	0	17	32	0.0%	6.2%	0.0%	6.2%	6.2%	0.0%	10.6%	14.4%
Reverse_tr|I3LVS7|I3LVS7	0	1	0	1	1	1	0	0	0	1	0	3	6	2	0	0	0.0%	8.8%	0.0%	8.8%	8.8%	8.8%	0.0%	0.0%
P15145	5	4	2	15	20	6	28	44	8	9	20	93	79	14	985	639	11.6%	4.9%	5.1%	17.8%	22.0%	7.5%	35.3%	44.2%
Q767L6	2	0	3	2	3	0	6	21	2	0	22	7	14	0	28	130	7.5%	0.0%	7.5%	4.4%	7.0%	0.0%	21.8%	43.1%
tr|K7GPJ9|K7GPJ9	2	0	3	2	3	0	3	13	2	0	22	7	14	0	22	104	10.5%	0.0%	10.5%	6.2%	9.8%	0.0%	15.1%	34.4%
tr|K7GPK7|K7GPK7	1	0	5	2	2	0	1	1	1	0	70	3	7	0	1	2	37.9%	0.0%	43.9%	33.3%	37.9%	0.0%	37.9%	37.9%
P22411	5	2	4	6	9	0	19	27	5	2	28	31	34	0	1116	614	6.0%	3.0%	4.3%	8.6%	12.7%	0.0%	24.8%	32.9%
tr|K7GMV8|K7GMV8	3	0	0	3	4	3	4	13	4	0	0	13	21	3	52	116	9.0%	0.0%	0.0%	9.2%	15.3%	8.5%	17.0%	38.0%
tr|K7GP66|K7GP66	2	0	1	2	2	1	6	24	2	0	1	11	4	2	40	108	1.4%	0.0%	0.9%	1.4%	1.4%	0.6%	6.1%	19.9%
tr|K7GPD4|K7GPD4	2	0	1	2	2	1	6	24	2	0	1	11	4	2	40	108	1.4%	0.0%	0.8%	1.4%	1.4%	0.6%	5.9%	19.3%
tr|F1RVY6|F1RVY6	2	0	1	2	2	1	6	24	2	0	1	11	4	2	40	108	1.4%	0.0%	0.8%	1.4%	1.4%	0.5%	5.9%	19.1%
tr|F1SQX4|F1SQX4	1	0	1	2	3	0	7	24	1	0	4	5	3	0	178	199	1.2%	0.0%	3.1%	2.6%	11.4%	0.0%	18.6%	38.9%
tr|I3LK11|I3LK11	1	0	0	1	1	0	1	0	1	0	0	3	5	0	7	0	10.6%	0.0%	0.0%	10.6%	10.6%	0.0%	10.6%	0.0%
tr|I3LTW0|I3LTW0	1	0	0	1	1	0	1	0	1	0	0	3	5	0	7	0	8.9%	0.0%	0.0%	8.9%	8.9%	0.0%	8.9%	0.0%
tr|Q7JFN4|Q7JFN4	1	0	2	1	1	0	0	0	1	0	14	7	1	0	0	0	3.7%	0.0%	7.1%	1.7%	5.8%	0.0%	0.0%	0.0%
tr|Q7JFN2|Q7JFN2	1	0	2	1	1	0	0	0	1	0	14	7	1	0	0	0	3.5%	0.0%	6.9%	1.7%	5.6%	0.0%	0.0%	0.0%
tr|Q7JFN3|Q7JFN3	1	0	2	1	1	0	0	0	1	0	14	7	1	0	0	0	3.8%	0.0%	7.5%	1.8%	6.1%	0.0%	0.0%	0.0%
tr|K7GM56|K7GM56	0	3	0	2	3	0	1	0	0	6	0	18	24	0	2	0	0.0%	20.4%	0.0%	12.0%	20.4%	0.0%	11.1%	0.0%
tr|F1RGX4|F1RGX4	0	3	0	2	3	0	1	0	0	6	0	18	24	0	2	0	0.0%	15.5%	0.0%	9.2%	15.5%	0.0%	8.5%	0.0%
tr|K7GQP8|K7GQP8	0	3	0	2	3	0	1	0	0	6	0	18	24	0	2	0	0.0%	18.0%	0.0%	10.7%	18.0%	0.0%	9.8%	0.0%
tr|K7GLP2|K7GLP2	0	3	0	2	3	0	1	0	0	6	0	18	24	0	2	0	0.0%	20.0%	0.0%	11.8%	20.0%	0.0%	10.9%	0.0%
P01965	0	3	0	2	3	0	1	0	0	6	0	18	24	0	2	0	0.0%	15.6%	0.0%	9.2%	15.6%	0.0%	8.5%	0.0%
tr|K7GKK8|K7GKK8	1	0	0	2	2	0	4	8	1	0	0	5	2	0	115	51	2.6%	0.0%	0.0%	5.5%	14.0%	0.0%	24.7%	35.2%
tr|F1RTK2|F1RTK2	1	0	0	1	1	0	1	1	1	0	0	3	4	0	6	1	2.9%	0.0%	0.0%	2.9%	2.9%	0.0%	2.9%	2.9%
tr|I3LQK6|I3LQK6	1	0	0	1	1	0	1	1	1	0	0	3	4	0	6	1	2.5%	0.0%	0.0%	2.5%	2.5%	0.0%	2.5%	2.5%
tr|F1RL74|F1RL74	1	0	1	1	0	0	0	0	1	0	3	7	0	0	0	0	4.0%	0.0%	2.5%	1.9%	0.0%	0.0%	0.0%	0.0%
tr|I3LSJ3|I3LSJ3	1	0	1	1	0	0	0	0	1	0	3	7	0	0	0	0	4.9%	0.0%	3.1%	2.3%	0.0%	0.0%	0.0%	0.0%
tr|I3LNG5|I3LNG5	1	0	1	1	0	0	0	0	1	0	3	7	0	0	0	0	5.0%	0.0%	3.2%	2.4%	0.0%	0.0%	0.0%	0.0%
tr|K7GT33|K7GT33	2	0	2	3	5	1	10	11	2	0	4	4	8	1	176	177	9.9%	0.0%	2.5%	8.7%	15.9%	1.9%	30.6%	31.3%
tr|F1SQ78|F1SQ78	8	1	6	9	8	4	15	26	14	1	38	48	35	9	356	321	10.3%	1.2%	6.4%	9.3%	7.7%	3.8%	18.6%	25.0%
tr|F1RP41|F1RP41	1	1	0	3	4	2	4	8	2	1	0	5	9	4	111	176	9.8%	4.9%	0.0%	12.9%	16.0%	8.7%	30.3%	32.4%
tr|F1RSY8|F1RSY8	1	0	0	1	1	0	0	3	2	0	0	8	4	0	0	4	3.7%	0.0%	0.0%	2.5%	2.5%	0.0%	0.0%	9.6%
tr|F1RII6|F1RII6	0	1	0	1	1	0	1	1	0	1	0	2	4	0	8	2	0.0%	6.8%	0.0%	6.8%	6.8%	0.0%	6.1%	6.1%
P02101	0	1	0	1	1	0	1	1	0	1	0	2	4	0	8	2	0.0%	6.8%	0.0%	6.8%	6.8%	0.0%	6.1%	6.1%
tr|F1RII5|F1RII5	0	1	0	1	1	0	1	1	0	1	0	2	4	0	8	2	0.0%	6.9%	0.0%	6.9%	6.9%	0.0%	6.2%	6.2%
P04246	0	1	0	1	1	0	1	1	0	1	0	2	4	0	8	2	0.0%	6.8%	0.0%	6.8%	6.8%	0.0%	6.2%	6.2%
tr|I3LPD9|I3LPD9	0	1	1	2	2	0	1	2	0	1	2	4	2	0	4	12	0.0%	6.8%	9.3%	6.8%	16.0%	0.0%	9.3%	19.1%
tr|F1SAX5|F1SAX5	1	0	1	1	1	0	2	1	1	0	5	5	1	0	12	10	8.1%	0.0%	8.1%	4.1%	4.1%	0.0%	21.7%	13.6%
tr|I3LDS3|I3LDS3	0	4	0	1	12	3	0	4	0	8	0	2	40	5	0	75	0.0%	7.9%	0.0%	1.6%	18.7%	3.1%	0.0%	12.0%
tr|F1SP55|F1SP55	3	1	3	3	6	3	5	9	3	1	13	7	9	7	17	58	2.9%	1.3%	4.6%	3.1%	4.5%	3.8%	6.9%	11.9%
tr|I3LEE5|I3LEE5	2	0	0	2	2	4	8	11	2	0	0	4	3	4	159	139	4.6%	0.0%	0.0%	2.9%	2.4%	6.0%	14.8%	16.7%
tr|F1RGC4|F1RGC4	1	1	1	3	4	0	2	2	1	1	1	5	5	0	135	49	20.0%	20.0%	20.0%	26.4%	32.8%	0.0%	32.8%	20.0%
tr|F1RGC3|F1RGC3	1	1	1	3	4	0	2	2	1	1	1	5	5	0	135	49	19.8%	19.8%	19.8%	26.2%	32.5%	0.0%	32.5%	19.8%
tr|F1S5Y5|F1S5Y5	1	0	0	1	2	0	1	8	1	0	0	1	4	0	2	39	5.1%	0.0%	0.0%	1.9%	7.9%	0.0%	2.2%	18.9%
tr|I3LNT8|I3LNT8	1	0	0	1	1	2	1	2	1	0	0	2	1	2	10	8	1.9%	0.0%	0.0%	2.5%	4.8%	4.8%	4.8%	6.7%
tr|F1SAG0|F1SAG0	1	0	1	1	1	0	2	6	1	0	5	3	2	0	10	33	1.4%	0.0%	1.3%	1.2%	1.4%	0.0%	2.5%	7.4%
Q9MZ16	2	2	8	1	5	8	5	7	3	3	211	1	8	19	38	28	7.1%	14.8%	27.2%	7.8%	25.4%	31.4%	26.1%	36.0%
tr|F1SHC0|F1SHC0	2	1	1	2	2	2	1	2	2	17	4	33	48	6	152	7	6.2%	2.8%	2.3%	5.4%	5.4%	3.1%	2.8%	3.1%
tr|K7GKQ4|K7GKQ4	2	0	0	1	2	1	2	3	4	0	0	7	8	3	19	77	47.3%	0.0%	0.0%	27.3%	47.3%	47.3%	47.3%	47.3%
P79385	0	1	0	3	1	5	7	12	0	6	0	10	1	13	64	85	0.0%	3.7%	0.0%	9.5%	3.7%	12.2%	28.4%	30.3%
tr|B2CZF8|B2CZF8	0	1	0	3	1	5	7	12	0	6	0	10	1	13	64	85	0.0%	3.5%	0.0%	9.0%	3.5%	11.6%	26.9%	28.8%
tr|K7GQE4|K7GQE4	1	0	1	0	2	1	1	4	3	0	6	0	10	2	36	46	31.2%	0.0%	27.3%	0.0%	31.2%	31.2%	27.3%	58.4%
tr|I3LUQ7|I3LUQ7	1	1	1	3	4	0	1	2	1	1	1	3	5	0	134	49	19.8%	19.8%	19.8%	26.2%	32.5%	0.0%	19.8%	19.8%
tr|F1S091|F1S091	1	1	0	1	1	0	1	1	1	1	0	4	4	0	35	3	7.6%	7.6%	0.0%	7.6%	7.6%	0.0%	7.6%	7.1%
tr|I3LH60|I3LH60	0	1	0	1	1	0	2	7	0	2	0	3	5	0	9	19	0.0%	8.2%	0.0%	8.2%	8.2%	0.0%	22.4%	43.2%
tr|F2Z519|F2Z519	0	1	0	1	1	0	2	4	0	2	0	3	5	0	9	14	0.0%	14.3%	0.0%	14.3%	14.3%	0.0%	39.0%	48.6%
tr|I3LFS8|I3LFS8	1	0	1	1	2	1	8	6	1	0	8	1	2	1	54	44	4.3%	0.0%	3.2%	3.2%	7.6%	5.7%	28.6%	16.0%
tr|F1RNA6|F1RNA6	1	0	1	1	2	1	8	6	1	0	8	1	2	1	54	44	4.6%	0.0%	3.4%	3.4%	8.0%	6.1%	30.3%	17.0%
tr|F1RQK5|F1RQK5	1	0	0	1	1	0	0	0	1	0	0	2	2	0	0	0	5.0%	0.0%	0.0%	5.0%	5.0%	0.0%	0.0%	0.0%
tr|I3LPW8|I3LPW8	6	2	4	5	9	3	4	17	9	2	14	18	22	3	39	395	18.2%	5.1%	11.1%	11.1%	15.2%	7.3%	11.7%	32.4%
tr|F1SRS2|F1SRS2	7	4	0	4	12	4	3	6	15	14	0	41	67	4	34	48	33.7%	26.4%	0.0%	21.5%	41.1%	16.3%	21.5%	39.8%
P00761	7	4	0	4	12	4	3	6	15	14	0	41	67	4	34	48	35.9%	28.1%	0.0%	22.9%	43.7%	17.3%	22.9%	42.4%
tr|F1STL6|F1STL6	0	1	1	2	1	0	0	4	0	4	1	9	6	0	0	11	0.0%	1.2%	2.2%	2.8%	1.2%	0.0%	0.0%	7.2%
tr|K7GLH2|K7GLH2	2	1	1	1	2	1	0	0	3	1	2	7	7	1	0	0	24.4%	24.4%	24.4%	24.4%	48.9%	24.4%	0.0%	0.0%
tr|K7GMZ6|K7GMZ6	2	1	1	1	2	1	0	0	3	1	2	7	7	1	0	0	18.6%	18.6%	18.6%	18.6%	37.3%	18.6%	0.0%	0.0%
tr|K7GR85|K7GR85	2	1	1	1	2	1	0	0	3	1	2	7	7	1	0	0	26.8%	26.8%	26.8%	26.8%	53.7%	26.8%	0.0%	0.0%
tr|B2ZI35|B2ZI35	2	2	0	2	3	2	2	4	4	6	0	27	7	3	4	44	5.0%	5.0%	0.0%	5.0%	8.4%	5.0%	8.4%	20.8%
tr|F1RJY0|F1RJY0	1	1	0	1	3	2	3	6	1	2	0	4	4	3	10	28	3.1%	1.1%	0.0%	1.1%	6.6%	5.2%	8.7%	16.5%
Q95334	6	0	3	4	10	2	14	22	8	0	10	5	21	3	244	255	10.8%	0.0%	3.7%	6.2%	16.3%	3.7%	24.7%	31.3%
tr|I3LHE9|I3LHE9	0	3	0	0	6	4	0	1	0	8	0	0	15	14	0	5	0.0%	10.9%	0.0%	0.0%	41.7%	10.9%	0.0%	6.6%
tr|I3L725|I3L725	2	1	1	1	2	1	0	0	3	2	2	8	9	1	0	0	12.5%	15.9%	12.5%	15.9%	28.4%	15.9%	0.0%	0.0%
tr|F1S138|F1S138	6	0	3	4	9	2	14	22	8	0	10	5	20	3	244	255	10.8%	0.0%	3.7%	6.2%	15.4%	3.7%	24.8%	31.3%
tr|I3LGI5|I3LGI5	1	1	0	1	1	0	1	4	1	1	0	3	4	0	6	5	4.6%	5.7%	0.0%	4.6%	4.6%	0.0%	4.6%	22.9%
tr|F1STN6|F1STN6	1	1	0	1	1	0	1	4	1	1	0	3	4	0	6	5	2.6%	3.2%	0.0%	2.6%	2.6%	0.0%	2.6%	12.9%
tr|A9QW81|A9QW81	1	0	1	1	3	1	1	3	2	0	2	3	3	1	1	23	21.0%	0.0%	21.0%	12.9%	33.9%	21.0%	12.9%	21.0%
tr|I3L7C2|I3L7C2	0	1	1	1	0	1	0	0	0	2	3	6	0	1	0	0	0.0%	3.8%	3.5%	3.8%	0.0%	3.8%	0.0%	0.0%
tr|F1S081|F1S081	3	2	0	4	4	6	10	13	3	8	0	17	6	12	199	240	5.7%	1.4%	0.0%	4.1%	3.7%	6.9%	15.1%	16.8%
tr|K7GT68|K7GT68	3	2	0	4	4	6	9	13	3	8	0	17	6	12	198	240	12.2%	3.1%	0.0%	8.9%	7.9%	14.9%	30.0%	36.2%
tr|F1SGY1|F1SGY1	0	1	2	2	2	0	2	4	0	6	7	15	4	0	17	35	0.0%	0.9%	4.0%	0.9%	0.9%	0.0%	4.0%	5.4%

	Peptide Count								Spectral Count								Sequence Coverage							
	Whole Cell	Whole Cell	Whole Cell	Cilia Fraction	Cilia Fraction	Cilia Fraction	Cilia Fraction	Cilia Fraction	Whole Cell	Whole Cell	Whole Cell	Cilia Fraction	Cilia Fraction	Cilia Fraction	Cilia Fraction	Cilia Fraction	Whole Cell	Whole Cell	Whole Cell	Cilia Fraction	Cilia Fraction	Cilia Fraction	Cilia Fraction	Cilia Fraction
Sample#	1	2	6	4	3	5	7	8	1	2	6	4	3	5	7	8	1	2	6	4	3	5	7	8
**Accession**																								
tr|F1SMF6|F1SMF6	1	1	0	1	1	1	1	6	1	6	0	12	7	2	2	32	1.1%	0.9%	0.0%	0.9%	0.9%	0.9%	0.9%	10.5%
tr|K7GS51|K7GS51	1	1	0	1	1	1	1	5	1	6	0	12	7	2	2	31	2.0%	1.7%	0.0%	1.7%	1.7%	1.7%	1.7%	18.2%
tr|F1SNG6|F1SNG6	3	0	1	1	2	0	2	3	4	0	2	6	6	0	6	8	19.9%	0.0%	2.7%	5.6%	12.0%	0.0%	5.6%	9.3%
tr|F2Z5I8|F2Z5I8	2	1	0	2	1	0	2	0	2	1	0	5	4	0	38	0	13.0%	7.2%	0.0%	14.0%	7.2%	0.0%	14.0%	0.0%
tr|F1SUW3|F1SUW3	0	1	0	1	1	0	1	1	0	1	0	2	1	0	16	12	0.0%	5.0%	0.0%	5.0%	5.0%	0.0%	6.7%	6.7%
tr|I3LHI0|I3LHI0	0	1	0	0	1	1	0	0	0	1	0	0	2	1	0	0	0.0%	2.2%	0.0%	0.0%	2.2%	2.2%	0.0%	0.0%
tr|I3L5F1|I3L5F1	0	1	0	1	1	0	0	0	0	1	0	1	2	0	0	0	0.0%	0.7%	0.0%	0.6%	0.6%	0.0%	0.0%	0.0%
tr|F1SUJ6|F1SUJ6	1	0	0	1	1	0	0	0	1	0	0	1	2	0	0	0	9.8%	0.0%	0.0%	9.8%	9.8%	0.0%	0.0%	0.0%
tr|I3L8L2|I3L8L2	1	0	0	1	1	0	0	0	1	0	0	2	1	0	0	0	5.2%	0.0%	0.0%	11.6%	11.6%	0.0%	0.0%	0.0%
tr|I3L9Z8|I3L9Z8	1	0	0	1	1	0	0	0	1	0	0	2	1	0	0	0	8.1%	0.0%	0.0%	10.0%	10.0%	0.0%	0.0%	0.0%
tr|F1RXG3|F1RXG3	0	1	0	0	1	1	0	0	0	1	0	0	2	1	0	0	0.0%	1.8%	0.0%	0.0%	1.8%	1.8%	0.0%	0.0%
Q6PQD5	0	1	0	1	1	0	0	0	0	1	0	1	2	0	0	0	0.0%	0.5%	0.0%	0.5%	0.5%	0.0%	0.0%	0.0%
tr|K7GQH2|K7GQH2	0	1	0	1	2	0	0	0	0	1	0	1	2	0	0	0	0.0%	6.4%	0.0%	5.9%	6.4%	0.0%	0.0%	0.0%
tr|I3LK01|I3LK01	0	1	0	1	1	0	1	1	0	21	0	11	51	0	14	13	0.0%	2.4%	0.0%	2.4%	2.4%	0.0%	2.4%	2.4%
tr|F1RR02|F1RR02	0	1	0	1	1	0	1	1	0	21	0	11	51	0	14	13	0.0%	2.6%	0.0%	2.6%	2.6%	0.0%	2.6%	2.6%
tr|Q06AS6|Q06AS6	10	6	8	10	15	4	15	24	26	56	28	112	108	9	465	429	43.4%	15.5%	21.7%	36.9%	42.8%	8.5%	40.6%	57.2%
tr|F1RYJ4|F1RYJ4	6	4	6	5	6	4	11	23	10	12	45	29	20	12	127	286	12.5%	2.5%	9.3%	4.1%	5.3%	3.7%	15.9%	36.6%
tr|F1SR53|F1SR53	3	3	4	6	6	3	7	11	6	7	20	19	12	5	83	115	2.2%	2.9%	4.2%	7.1%	4.9%	3.3%	13.2%	16.8%
tr|F1SM86|F1SM86	0	1	0	1	1	0	0	1	0	4	0	5	6	0	0	27	0.0%	1.0%	0.0%	1.0%	1.0%	0.0%	0.0%	1.5%
tr|F1SL62|F1SL62	0	1	0	1	1	1	1	1	0	4	0	4	6	1	2	1	0.0%	1.5%	0.0%	1.5%	1.5%	1.5%	1.5%	1.5%
tr|F1SL82|F1SL82	0	1	0	1	1	1	1	1	0	4	0	4	6	1	2	1	0.0%	1.2%	0.0%	1.2%	1.2%	1.2%	1.2%	1.2%
tr|K7GT59|K7GT59	6	4	6	5	6	3	11	21	10	12	45	29	20	11	127	270	13.5%	2.7%	10.1%	4.5%	5.7%	2.7%	17.2%	35.8%
tr|K7GMP6|K7GMP6	6	4	6	5	6	3	11	21	10	12	45	29	20	11	127	270	13.4%	2.7%	10.0%	4.4%	5.7%	2.7%	17.0%	35.6%
tr|K7GPM1|K7GPM1	6	4	6	5	6	3	11	21	10	12	45	29	20	11	127	270	13.6%	2.8%	10.1%	4.5%	5.8%	2.8%	17.3%	36.0%
Q2EN75	0	3	1	0	4	1	0	1	0	3	11	0	7	1	0	1	0.0%	34.4%	10.0%	0.0%	17.8%	16.7%	0.0%	17.8%
Q95266	2	0	3	1	1	0	0	1	3	0	20	7	1	0	0	1	8.8%	0.0%	10.6%	1.8%	6.0%	0.0%	0.0%	5.0%
tr|F1SGI2|F1SGI2	2	2	2	1	1	2	2	2	3	22	3	11	51	2	20	28	1.8%	4.1%	3.3%	2.3%	2.3%	3.7%	4.1%	4.1%
tr|F1RNT9|F1RNT9	2	2	2	1	1	1	1	1	2	2	2	5	4	1	3	1	4.3%	2.3%	3.6%	1.5%	1.5%	1.5%	1.5%	3.5%
tr|F1SGH0|F1SGH0	2	0	1	1	2	0	3	11	2	0	3	1	4	0	10	34	3.1%	0.0%	1.8%	1.2%	4.0%	0.0%	6.3%	13.5%
tr|F1RR07|F1RR07	0	1	0	2	3	0	0	2	0	2	0	2	3	0	0	8	0.0%	1.9%	0.0%	3.2%	5.1%	0.0%	0.0%	4.8%
tr|I3L8R7|I3L8R7	7	0	0	3	5	1	3	10	17	0	0	21	18	2	84	302	21.7%	0.0%	0.0%	8.7%	16.5%	6.2%	17.4%	24.5%
F1S584	0	1	0	1	1	0	1	3	0	5	0	8	4	0	9	9	0.0%	1.4%	0.0%	1.4%	1.4%	0.0%	3.0%	5.4%
tr|I3LCQ9|I3LCQ9	2	1	1	2	3	0	4	11	2	1	5	4	3	0	21	99	24.1%	5.1%	5.1%	5.1%	23.6%	0.0%	25.9%	40.3%
tr|I3LQN8|I3LQN8	3	3	1	1	1	0	2	2	4	23	2	11	51	0	20	28	4.1%	6.2%	1.3%	2.1%	2.1%	0.0%	3.7%	3.7%
tr|F2Z4Z8|F2Z4Z8	6	4	5	5	11	2	12	17	21	6	27	25	33	3	881	617	32.4%	24.9%	21.7%	22.7%	52.4%	13.6%	57.0%	57.0%
tr|F1SFV3|F1SFV3	5	0	3	1	2	1	4	8	8	0	70	7	8	3	129	142	63.5%	0.0%	33.7%	14.4%	25.0%	25.0%	44.2%	63.5%
tr|F1RV14|F1RV14	0	1	0	1	1	0	2	3	0	7	0	8	7	0	5	6	0.0%	2.6%	0.0%	2.6%	2.6%	0.0%	6.1%	10.0%
tr|F1RFC5|F1RFC5	0	1	0	1	1	0	1	1	0	7	0	8	7	0	1	4	0.0%	5.2%	0.0%	5.2%	5.2%	0.0%	5.2%	5.2%
Q9GLP0	14	4	3	9	11	2	15	24	46	14	25	73	41	13	620	585	23.3%	7.8%	4.6%	14.4%	19.3%	2.1%	19.7%	24.3%
tr|F1RX15|F1RX15	7	0	1	1	5	2	5	24	10	0	2	8	8	5	30	235	9.0%	0.0%	1.4%	2.5%	5.5%	4.1%	13.1%	38.2%
tr|K7GSB2|K7GSB2	7	0	2	1	5	2	5	27	10	0	5	8	8	5	30	242	7.9%	0.0%	2.8%	2.2%	4.8%	3.6%	11.6%	35.6%
tr|I3LSK5|I3LSK5	9	4	7	5	11	2	14	19	24	6	39	25	33	3	885	675	35.3%	22.6%	25.6%	20.6%	47.6%	12.4%	57.6%	57.6%
tr|F1RVE7|F1RVE7	15	4	3	9	11	2	15	28	49	14	25	73	41	13	620	636	27.8%	7.8%	4.6%	14.4%	19.3%	2.1%	19.7%	28.8%
tr|F1S6B3|F1S6B3	7	3	4	2	3	1	6	21	9	3	13	8	15	1	60	145	11.5%	7.5%	10.3%	6.5%	8.5%	2.5%	14.3%	36.5%
tr|I3LHZ2|I3LHZ2	2	1	0	0	1	0	1	1	2	1	0	0	6	0	2	3	21.9%	5.3%	0.0%	0.0%	15.2%	0.0%	9.9%	6.6%
tr|F1RKY1|F1RKY1	1	1	0	1	1	0	0	2	1	2	0	3	3	0	0	2	3.5%	4.3%	0.0%	4.3%	4.3%	0.0%	0.0%	16.3%
tr|I3LPF1|I3LPF1	2	0	3	1	1	0	0	0	3	0	18	1	5	0	0	0	20.5%	0.0%	24.7%	7.4%	7.4%	0.0%	0.0%	0.0%
tr|K7GQR8|K7GQR8	0	1	0	1	2	0	1	3	0	2	0	2	2	0	3	34	0.0%	7.5%	0.0%	7.5%	17.7%	0.0%	10.2%	20.4%
tr|I3LDD6|I3LDD6	0	2	0	1	1	0	0	0	0	2	0	3	1	0	0	0	0.0%	5.5%	0.0%	2.7%	2.7%	0.0%	0.0%	0.0%
tr|V9GZ89|V9GZ89	1	0	0	1	0	0	1	7	1	0	0	2	0	0	8	55	2.0%	0.0%	0.0%	2.2%	0.0%	0.0%	4.8%	19.3%
tr|F1RS11|F1RS11	0	1	0	0	1	0	1	9	0	1	0	0	2	0	5	37	0.0%	2.7%	0.0%	0.0%	2.7%	0.0%	5.5%	23.3%
tr|F1SAW7|F1SAW7	1	0	0	0	1	1	1	2	1	0	0	0	1	1	2	5	2.1%	0.0%	0.0%	0.0%	2.1%	2.1%	5.9%	6.9%
tr|F1RLJ0|F1RLJ0	0	1	0	0	1	0	1	1	0	1	0	0	2	0	1	6	0.0%	1.3%	0.0%	0.0%	1.3%	0.0%	1.3%	1.3%
tr|A5GFT0|A5GFT0	1	0	0	0	1	1	1	0	1	0	0	0	1	1	3	0	5.8%	0.0%	0.0%	0.0%	5.8%	5.8%	5.8%	0.0%
tr|A5GFT2|A5GFT2	1	0	0	0	1	1	1	0	1	0	0	0	1	1	3	0	5.6%	0.0%	0.0%	0.0%	5.6%	5.6%	5.6%	0.0%
tr|A5GFS9|A5GFS9	1	0	0	0	1	1	1	0	1	0	0	0	1	1	3	0	5.9%	0.0%	0.0%	0.0%	5.9%	5.9%	5.9%	0.0%
tr|A5GFT1|A5GFT1	1	0	0	0	1	1	1	0	1	0	0	0	1	1	3	0	5.5%	0.0%	0.0%	0.0%	5.5%	5.5%	5.5%	0.0%
tr|I3LHQ5|I3LHQ5	0	1	0	1	1	0	0	0	0	1	0	1	1	0	0	0	0.0%	11.6%	0.0%	11.6%	11.6%	0.0%	0.0%	0.0%
tr|I3LEA8|I3LEA8	1	0	0	1	0	1	0	0	1	0	0	1	0	1	0	0	2.6%	0.0%	0.0%	2.6%	0.0%	2.6%	0.0%	0.0%
tr|F1SP12|F1SP12	0	1	0	1	1	0	0	0	0	1	0	1	1	0	0	0	0.0%	1.0%	0.0%	1.0%	1.0%	0.0%	0.0%	0.0%
tr|F1SLS7|F1SLS7	1	0	0	1	0	1	0	0	1	0	0	1	0	1	0	0	4.1%	0.0%	0.0%	3.5%	0.0%	3.5%	0.0%	0.0%
tr|I3LGL1|I3LGL1	0	1	0	0	1	0	0	0	0	1	0	0	2	0	0	0	0.0%	2.1%	0.0%	0.0%	2.1%	0.0%	0.0%	0.0%
tr|I3LSA0|I3LSA0	1	0	0	0	1	0	0	0	1	0	0	0	2	0	0	0	5.5%	0.0%	0.0%	0.0%	5.5%	0.0%	0.0%	0.0%
tr|F1RGZ0|F1RGZ0	0	1	0	0	2	0	0	0	0	1	0	0	2	0	0	0	0.0%	4.7%	0.0%	0.0%	4.7%	0.0%	0.0%	0.0%
tr|F1RID1|F1RID1	1	0	0	0	1	0	0	0	1	0	0	0	2	0	0	0	3.4%	0.0%	0.0%	0.0%	3.4%	0.0%	0.0%	0.0%
tr|K7GQ60|K7GQ60	1	0	0	0	1	0	0	0	1	0	0	0	2	0	0	0	2.2%	0.0%	0.0%	0.0%	1.3%	0.0%	0.0%	0.0%
tr|F1RTL0|F1RTL0	1	0	0	0	1	0	0	0	1	0	0	0	2	0	0	0	2.2%	0.0%	0.0%	0.0%	1.3%	0.0%	0.0%	0.0%
tr|I3LR74|I3LR74	1	0	1	1	1	0	1	0	1	0	3	1	1	0	4	0	7.4%	0.0%	7.4%	7.4%	7.4%	0.0%	12.8%	0.0%
Q06AU6	1	0	1	1	1	0	1	0	1	0	3	1	1	0	4	0	5.1%	0.0%	5.1%	5.1%	5.1%	0.0%	8.8%	0.0%
tr|K7GKA5|K7GKA5	1	0	1	0	0	1	0	0	1	0	3	0	0	2	0	0	3.0%	0.0%	3.0%	0.0%	0.0%	4.5%	0.0%	0.0%
tr|F1RPH6|F1RPH6	1	0	1	0	0	1	0	0	1	0	3	0	0	2	0	0	2.7%	0.0%	2.7%	0.0%	0.0%	4.1%	0.0%	0.0%
tr|F1RVP5|F1RVP5	1	0	3	1	0	1	0	2	1	0	12	1	0	1	0	4	2.2%	0.0%	11.1%	2.2%	0.0%	2.2%	0.0%	8.3%
tr|F1RT98|F1RT98	1	0	2	1	1	0	0	0	1	0	5	1	1	0	0	0	1.9%	0.0%	8.2%	1.6%	1.6%	0.0%	0.0%	0.0%
tr|K7GQD2|K7GQD2	0	1	9	1	1	0	0	0	0	1	21	1	1	0	0	0	0.0%	1.6%	13.7%	1.6%	1.6%	0.0%	0.0%	0.0%
tr|K7GS94|K7GS94	14	4	3	8	10	2	15	28	46	14	25	66	40	13	620	636	26.5%	7.7%	4.6%	13.1%	18.0%	2.1%	19.6%	28.7%
tr|I3L5L1|I3L5L1	7	5	9	4	11	1	8	14	19	47	33	65	60	4	452	250	36.4%	33.7%	24.5%	33.7%	40.8%	8.2%	50.0%	57.6%
tr|I3LF84|I3LF84	6	4	3	3	9	1	2	5	18	39	7	58	47	4	387	170	29.2%	27.7%	21.9%	27.7%	37.2%	10.9%	19.7%	35.0%
tr|I3LEG0|I3LEG0	12	4	7	9	15	6	9	42	23	16	29	28	35	8	170	733	20.7%	5.3%	8.9%	11.2%	15.5%	7.5%	15.3%	41.0%
tr|J9JIM2|J9JIM2	6	1	2	3	4	1	9	13	10	1	16	7	12	1	243	238	11.6%	3.0%	5.1%	5.1%	11.8%	4.8%	16.4%	23.4%
tr|F1S0K2|F1S0K2	1	2	1	0	5	1	2	1	3	2	24	0	8	1	2	5	2.4%	4.3%	2.4%	0.0%	7.8%	2.0%	5.0%	2.4%
tr|I3LFN8|I3LFN8	1	3	1	1	1	1	0	6	1	3	1	3	3	1	0	32	1.4%	5.9%	1.8%	2.0%	2.0%	2.0%	0.0%	9.9%

	Peptide Count								Spectral Count								Sequence Coverage							
	Whole Cell	Whole Cell	Whole Cell	Cilia Fraction	Cilia Fraction	Cilia Fraction	Cilia Fraction	Cilia Fraction	Whole Cell	Whole Cell	Whole Cell	Cilia Fraction	Cilia Fraction	Cilia Fraction	Cilia Fraction	Cilia Fraction	Whole Cell	Whole Cell	Whole Cell	Cilia Fraction	Cilia Fraction	Cilia Fraction	Cilia Fraction	Cilia Fraction
Sample#	1	2	6	4	3	5	7	8	1	2	6	4	3	5	7	8	1	2	6	4	3	5	7	8
**Accession**																								
tr|K7GNJ3|K7GNJ3	2	0	2	2	2	1	0	6	4	0	10	2	3	2	0	20	5.7%	0.0%	2.9%	2.4%	2.4%	1.1%	0.0%	10.9%
tr|I3L6Q4|I3L6Q4	2	0	2	2	2	1	0	6	4	0	10	2	3	2	0	20	5.8%	0.0%	2.9%	2.4%	2.4%	1.2%	0.0%	11.0%
tr|F2Z4Z3|F2Z4Z3	0	2	0	1	1	0	0	0	0	4	0	4	3	0	0	0	0.0%	13.7%	0.0%	8.0%	8.0%	0.0%	0.0%	0.0%
tr|F1SK70|F1SK70	1	1	0	1	1	1	0	0	1	3	0	3	2	2	0	0	9.8%	9.8%	0.0%	9.8%	9.8%	9.8%	0.0%	0.0%
tr|I3LDA5|I3LDA5	0	2	1	4	5	1	2	1	0	28	1	21	25	3	8	2	0.0%	5.7%	4.9%	9.4%	12.1%	3.0%	5.7%	4.9%
tr|F1S0L1|F1S0L1	2	4	4	2	6	3	2	2	4	7	37	3	11	5	2	7	4.2%	8.0%	6.7%	3.8%	14.1%	3.8%	4.8%	6.1%
tr|F1SB33|F1SB33	4	1	0	1	2	1	0	2	7	3	0	7	8	2	0	17	18.9%	4.2%	0.0%	4.2%	8.1%	4.2%	0.0%	16.6%
Q58D68	13	3	2	13	14	4	12	24	28	48	4	61	48	19	604	903	37.7%	4.8%	10.0%	28.9%	32.7%	9.3%	34.8%	41.4%
tr|F1S3P8|F1S3P8	0	1	2	1	1	0	0	1	0	3	4	4	1	0	0	1	0.0%	5.4%	10.7%	5.4%	5.4%	0.0%	0.0%	5.4%
tr|Q06AT8|Q06AT8	2	2	0	2	2	2	3	4	14	71	0	70	58	11	13	10	16.8%	4.6%	0.0%	4.6%	4.6%	4.6%	25.5%	40.8%
tr|F1RZ59|F1RZ59	10	5	9	6	6	2	6	11	27	10	79	33	25	2	404	260	28.5%	20.2%	24.4%	22.7%	26.3%	6.9%	20.2%	31.3%
tr|K7GNL6|K7GNL6	5	6	1	5	9	5	5	9	19	41	4	22	49	26	166	94	14.1%	8.5%	2.2%	8.8%	13.5%	8.0%	8.8%	15.3%
tr|K7GKU7|K7GKU7	5	6	1	5	9	5	5	9	19	41	4	22	48	26	166	94	14.3%	8.7%	2.3%	8.9%	13.7%	8.2%	8.9%	15.5%
tr|F1S1N1|F1S1N1	10	5	8	5	6	2	6	10	27	10	78	32	25	2	404	244	28.5%	20.2%	21.9%	20.2%	26.3%	6.9%	20.2%	28.8%
tr|F1S1N0|F1S1N0	7	4	6	4	5	2	5	7	17	9	68	23	16	2	398	214	21.2%	14.2%	16.0%	14.2%	20.6%	7.3%	14.2%	21.8%
tr|I3LV59|I3LV59	1	1	0	1	1	0	1	1	3	4	0	3	8	0	115	26	8.3%	8.3%	0.0%	8.3%	8.3%	0.0%	8.3%	8.3%
tr|F2Z5R5|F2Z5R5	2	1	1	1	1	0	0	1	3	6	5	9	5	0	0	2	17.5%	4.7%	11.3%	4.7%	4.7%	0.0%	0.0%	11.3%
tr|F1SSB5|F1SSB5	4	2	8	1	1	0	5	7	6	11	154	19	7	0	40	99	9.3%	11.3%	38.5%	5.1%	5.1%	0.0%	31.1%	31.5%
Q52NJ1	8	4	3	4	4	0	1	6	16	12	13	16	26	0	3	34	42.1%	22.7%	14.4%	29.2%	21.8%	0.0%	6.9%	43.5%
tr|F1RWU4|F1RWU4	3	1	4	0	3	0	6	24	3	1	21	0	6	0	43	152	6.9%	2.3%	9.8%	0.0%	3.9%	0.0%	13.6%	34.2%
tr|K7GMS4|K7GMS4	2	0	0	1	2	0	3	6	2	0	0	1	2	0	32	19	10.1%	0.0%	0.0%	7.6%	26.1%	0.0%	32.8%	52.9%
tr|F1SI24|F1SI24	2	0	4	1	1	0	2	5	2	0	9	2	1	0	11	27	13.3%	0.0%	31.3%	8.1%	6.6%	0.0%	19.0%	39.3%
tr|F1RZ25|F1RZ25	1	1	1	1	2	0	0	2	1	1	4	1	2	0	0	11	2.6%	2.8%	6.0%	2.6%	2.8%	0.0%	0.0%	9.4%
tr|K7GP41|K7GP41	1	1	1	1	2	0	0	2	1	1	4	1	2	0	0	11	2.7%	2.9%	6.3%	2.7%	2.9%	0.0%	0.0%	9.9%
tr|I3LV82|I3LV82	2	0	0	0	2	0	0	0	2	0	0	0	3	0	0	0	5.5%	0.0%	0.0%	0.0%	5.5%	0.0%	0.0%	0.0%
Q03365	2	0	0	0	2	0	0	0	2	0	0	0	3	0	0	0	3.4%	0.0%	0.0%	0.0%	3.4%	0.0%	0.0%	0.0%
tr|F1RQM6|F1RQM6	1	1	0	2	1	0	0	0	1	1	0	2	1	0	0	0	1.4%	1.4%	0.0%	2.3%	0.9%	0.0%	0.0%	0.0%
tr|F1RWC9|F1RWC9	1	0	0	0	1	0	0	0	2	0	0	0	3	0	0	0	11.3%	0.0%	0.0%	0.0%	11.3%	0.0%	0.0%	0.0%
tr|I3L6G1|I3L6G1	2	0	0	1	1	0	0	0	2	0	0	2	1	0	0	0	5.8%	0.0%	0.0%	5.8%	5.8%	0.0%	0.0%	0.0%
tr|F1RL81|F1RL81	0	1	2	1	1	0	0	2	0	2	10	1	2	0	0	8	0.0%	5.6%	9.3%	5.6%	5.6%	0.0%	0.0%	14.1%
tr|F1RJP9|F1RJP9	1	1	3	1	1	0	0	1	1	1	8	1	2	0	0	3	0.6%	1.0%	4.5%	1.0%	1.4%	0.0%	0.0%	1.7%
tr|F1S9T2|F1S9T2	1	1	3	0	3	0	1	0	1	1	9	0	3	0	2	0	2.6%	7.9%	9.3%	0.0%	15.2%	0.0%	4.7%	0.0%
tr|K7GP25|K7GP25	12	5	5	12	15	5	16	23	32	62	35	59	57	24	107	116	15.4%	5.6%	5.5%	13.0%	13.6%	4.0%	19.1%	22.6%
tr|I3LDM6|I3LDM6	2	3	1	1	4	3	2	4	3	44	2	7	59	4	8	32	1.4%	3.3%	1.1%	1.7%	4.7%	4.5%	3.1%	4.7%
tr|I3LEY6|I3LEY6	1	1	0	1	1	1	1	3	1	18	0	17	9	2	1	9	2.9%	1.0%	0.0%	1.0%	1.0%	1.0%	2.0%	6.7%
tr|K7GKY0|K7GKY0	16	6	6	13	17	8	17	26	38	64	47	63	59	27	125	138	17.7%	6.1%	6.5%	13.1%	14.7%	5.9%	19.3%	23.2%
tr|F1SAG9|F1SAG9	3	1	1	2	2	2	2	13	6	3	5	3	3	7	34	79	8.9%	2.6%	5.1%	2.6%	6.1%	2.6%	7.6%	29.3%
tr|K7GP49|K7GP49	3	1	1	2	2	2	2	13	6	3	5	3	3	7	34	79	8.9%	2.7%	5.2%	2.7%	6.1%	2.7%	7.7%	29.6%
tr|F1SB90|F1SB90	2	4	3	4	5	2	4	5	5	48	18	33	37	6	204	173	7.2%	7.5%	8.7%	14.2%	11.9%	4.1%	15.4%	15.7%
tr|K7GNK3|K7GNK3	10	5	8	4	5	2	6	11	27	10	52	30	21	2	404	260	29.9%	21.2%	19.2%	17.4%	21.2%	7.3%	21.2%	32.8%
tr|F2Z518|F2Z518	0	2	0	2	2	1	1	0	0	14	0	15	4	1	1	0	0.0%	20.8%	0.0%	20.8%	20.8%	8.8%	12.0%	0.0%
Q29315	10	7	5	5	4	3	1	1	43	35	62	69	31	10	1	2	59.1%	43.5%	33.9%	16.5%	16.5%	16.5%	16.5%	16.5%
tr|F1SGG7|F1SGG7	2	3	1	1	2	1	2	3	3	44	2	7	57	2	8	25	1.7%	4.1%	1.4%	2.1%	2.3%	2.1%	3.9%	4.1%
tr|F1SGG4|F1SGG4	2	3	1	1	2	1	2	3	3	44	2	7	57	2	8	25	1.8%	4.2%	1.4%	2.2%	2.4%	2.2%	4.0%	4.2%
tr|F1SGG3|F1SGG3	5	8	1	3	9	3	2	6	6	51	2	11	65	4	8	69	4.1%	8.3%	1.1%	5.1%	12.1%	4.6%	3.2%	7.3%
tr|F1RQH9|F1RQH9	16	6	6	12	15	8	17	23	38	64	47	60	56	27	125	124	17.7%	6.1%	6.5%	11.4%	12.8%	5.9%	19.3%	21.3%
Q2MJK3	4	1	2	2	3	0	5	10	4	1	7	4	3	0	25	84	41.8%	5.8%	13.8%	5.8%	27.0%	0.0%	37.6%	41.3%
tr|I3LAT7|I3LAT7	0	1	0	1	0	1	0	0	0	5	0	1	0	6	0	0	0.0%	4.1%	0.0%	4.1%	0.0%	4.1%	0.0%	0.0%
tr|I3LUL5|I3LUL5	3	2	3	3	3	2	2	3	7	16	11	13	14	5	3	38	6.6%	3.4%	7.0%	5.6%	5.6%	3.4%	3.6%	5.0%
tr|I3LLY8|I3LLY8	4	5	1	1	6	1	2	5	6	48	2	7	66	2	8	41	5.8%	8.0%	1.3%	2.1%	8.0%	2.1%	3.7%	5.8%
tr|F1SGG6|F1SGG6	4	6	1	1	6	4	2	5	6	49	2	7	64	5	8	41	5.1%	8.8%	1.2%	1.8%	7.3%	6.4%	3.3%	5.1%
tr|F1S5D9|F1S5D9	0	2	0	1	3	1	0	0	0	8	0	1	5	5	0	0	0.0%	5.6%	0.0%	5.6%	13.3%	5.6%	0.0%	0.0%
tr|F1SGG9|F1SGG9	4	5	1	1	5	3	2	5	6	48	2	7	63	4	8	41	5.5%	7.7%	1.3%	2.0%	6.1%	5.2%	3.6%	5.5%
tr|F1RJS2|F1RJS2	6	2	8	2	5	2	4	11	9	2	32	3	10	2	77	62	15.7%	4.5%	22.5%	3.6%	15.7%	8.5%	11.2%	22.2%
tr|F1SGI7|F1SGI7	3	4	1	1	3	3	2	3	5	51	2	7	65	4	8	25	3.4%	5.4%	1.1%	1.8%	3.9%	4.8%	3.3%	3.4%
tr|F1RT62|F1RT62	13	9	1	6	10	8	5	17	32	48	4	26	51	30	166	171	17.9%	9.2%	1.8%	8.1%	11.9%	8.8%	7.0%	17.7%
tr|F1SNX6|F1SNX6	2	1	0	1	2	1	2	3	4	5	0	7	4	1	52	8	5.2%	2.5%	0.0%	2.5%	5.0%	2.5%	5.2%	6.5%
tr|F1SP53|F1SP53	1	1	0	1	2	0	1	5	1	2	0	2	2	0	3	36	2.0%	1.7%	0.0%	1.7%	4.0%	0.0%	2.3%	7.2%
tr|K7GL05|K7GL05	1	1	0	1	2	0	1	5	1	2	0	2	2	0	3	36	2.0%	1.7%	0.0%	1.7%	4.0%	0.0%	2.3%	7.2%
tr|K7GM61|K7GM61	1	1	0	1	2	0	1	5	1	2	0	2	2	0	3	36	2.0%	1.7%	0.0%	1.7%	4.0%	0.0%	2.3%	7.2%
tr|F1RXT2|F1RXT2	1	2	0	1	1	0	0	0	1	2	0	3	1	0	0	0	2.4%	5.5%	0.0%	2.7%	2.7%	0.0%	0.0%	0.0%
tr|I3LKB5|I3LKB5	1	2	0	1	1	0	0	0	1	2	0	3	1	0	0	0	2.6%	6.0%	0.0%	2.9%	2.9%	0.0%	0.0%	0.0%
Reverse_tr|F1RSG3|F1RSG3	1	1	0	1	0	0	0	0	2	1	0	4	0	0	0	0	1.2%	1.2%	0.0%	1.2%	0.0%	0.0%	0.0%	0.0%
tr|I3L961|I3L961	1	2	0	1	1	0	0	0	1	2	0	3	1	0	0	0	2.6%	6.1%	0.0%	3.0%	3.0%	0.0%	0.0%	0.0%
Reverse_tr|F1S9J8|F1S9J8	1	1	0	1	0	0	0	0	2	1	0	4	0	0	0	0	1.0%	1.0%	0.0%	1.0%	0.0%	0.0%	0.0%	0.0%
tr|I3LFZ8|I3LFZ8	2	0	2	2	2	0	1	1	3	0	6	2	2	0	3	1	10.9%	0.0%	10.9%	10.9%	10.9%	0.0%	8.3%	10.5%
tr|F1RYT3|F1RYT3	5	2	3	3	1	1	4	5	10	18	36	24	12	1	11	17	16.5%	4.2%	6.3%	6.9%	1.7%	1.7%	10.3%	11.1%
P08132	5	4	7	6	7	1	4	4	10	6	33	10	10	1	57	52	26.0%	15.7%	23.5%	28.5%	32.6%	11.0%	16.9%	20.4%
tr|F1SLC4|F1SLC4	5	4	7	6	7	1	4	4	10	6	33	10	10	1	57	52	26.0%	15.7%	23.5%	28.5%	32.6%	11.0%	16.9%	20.4%
tr|F1S485|F1S485	1	2	1	1	2	0	4	16	1	9	1	5	8	0	18	107	1.9%	2.3%	1.7%	0.9%	2.2%	0.0%	6.7%	20.8%
Q8SQ23	2	1	0	0	3	2	0	0	3	4	0	0	4	5	0	0	5.3%	3.6%	0.0%	0.0%	6.9%	6.9%	0.0%	0.0%
tr|F1SE20|F1SE20	2	1	0	0	3	2	0	0	3	4	0	0	4	5	0	0	5.3%	3.6%	0.0%	0.0%	6.9%	6.9%	0.0%	0.0%
tr|K7GM89|K7GM89	2	3	1	1	4	3	2	1	10	29	4	3	29	18	146	13	30.2%	22.4%	16.4%	13.8%	22.4%	22.4%	30.2%	13.8%
tr|B9P3U4|B9P3U4	2	4	1	2	2	1	1	0	4	14	4	12	10	1	3	0	22.2%	25.9%	7.4%	11.9%	11.9%	7.4%	11.9%	0.0%
tr|Q8WNW4|Q8WNW4	12	7	7	5	6	7	13	29	37	21	79	32	25	16	201	305	24.3%	13.3%	13.3%	7.6%	7.6%	13.7%	21.1%	40.6%
tr|F1S732|F1S732	2	2	0	0	2	0	0	3	2	2	0	0	5	0	0	12	7.6%	6.9%	0.0%	0.0%	6.6%	0.0%	0.0%	14.5%
Reverse_tr|F1SEQ1|F1SEQ1	1	1	0	1	1	0	0	0	1	3	0	3	2	0	0	0	2.4%	2.4%	0.0%	2.4%	2.4%	0.0%	0.0%	0.0%
tr|F1RX81|F1RX81	11	4	4	4	5	6	8	19	35	17	31	28	22	14	69	230	22.9%	5.5%	6.0%	5.8%	5.8%	7.7%	12.1%	23.7%
tr|F1S3M9|F1S3M9	6	2	3	3	3	2	4	9	10	16	12	13	14	5	12	88	7.7%	2.3%	5.7%	3.7%	3.7%	2.3%	8.2%	17.1%
tr|F1RKW9|F1RKW9	2	4	1	2	5	2	2	6	3	10	3	7	7	2	10	29	3.2%	3.8%	1.7%	1.6%	4.8%	1.4%	4.6%	10.6%
tr|F2Z5L0|F2Z5L0	3	2	3	3	2	4	2	1	21	13	46	22	11	8	227	1	20.6%	17.5%	28.6%	17.5%	11.9%	38.1%	20.6%	11.9%

	Peptide Count								Spectral Count								Sequence Coverage							
	Whole Cell	Whole Cell	Whole Cell	Cilia Fraction	Cilia Fraction	Cilia Fraction	Cilia Fraction	Cilia Fraction	Whole Cell	Whole Cell	Whole Cell	Cilia Fraction	Cilia Fraction	Cilia Fraction	Cilia Fraction	Cilia Fraction	Whole Cell	Whole Cell	Whole Cell	Cilia Fraction	Cilia Fraction	Cilia Fraction	Cilia Fraction	Cilia Fraction
Sample#	1	2	6	4	3	5	7	8	1	2	6	4	3	5	7	8	1	2	6	4	3	5	7	8
**Accession**																								
tr|I3LE39|I3LE39	3	2	3	3	2	4	2	1	21	13	46	22	11	8	227	1	20.6%	17.5%	28.6%	17.5%	11.9%	38.1%	20.6%	11.9%
tr|I3LEB6|I3LEB6	3	2	3	3	2	4	2	1	21	13	46	22	11	8	227	1	19.4%	16.4%	26.9%	16.4%	11.2%	35.8%	19.4%	11.2%
tr|F1SJY6|F1SJY6	16	6	13	5	14	3	12	37	41	36	198	40	44	7	63	393	26.2%	4.2%	19.1%	10.4%	12.4%	1.7%	17.9%	41.6%
tr|F1RF18|F1RF18	0	3	1	2	3	2	1	3	0	44	2	25	21	6	26	54	0.0%	4.0%	2.5%	6.2%	4.0%	4.0%	3.7%	8.5%
tr|F2Z584|F2Z584	3	2	4	3	2	3	2	1	21	13	55	22	11	7	227	1	20.6%	17.5%	28.6%	17.5%	11.9%	28.6%	20.6%	11.9%
tr|F2Z580|F2Z580	3	2	4	3	2	3	2	1	21	13	55	22	11	7	227	1	20.6%	17.5%	28.6%	17.5%	11.9%	28.6%	20.6%	11.9%
tr|F2Z578|F2Z578	3	2	4	3	2	3	2	1	21	13	55	22	11	7	227	1	20.6%	17.5%	28.6%	17.5%	11.9%	28.6%	20.6%	11.9%
tr|I3LAQ1|I3LAQ1	3	2	4	3	2	3	2	1	21	13	55	22	11	7	227	1	19.4%	16.4%	26.9%	16.4%	11.2%	26.9%	19.4%	11.2%
tr|F2Z579|F2Z579	3	2	4	3	2	3	2	1	21	13	55	22	11	7	227	1	20.6%	17.5%	28.6%	17.5%	11.9%	28.6%	20.6%	11.9%
tr|F2Z581|F2Z581	3	2	4	3	2	3	2	1	21	13	55	22	11	7	227	1	20.6%	17.5%	28.6%	17.5%	11.9%	28.6%	20.6%	11.9%
tr|Q06AS8|Q06AS8	6	6	7	7	10	5	16	22	11	88	44	51	54	11	522	434	23.2%	11.9%	17.2%	22.6%	32.2%	13.3%	48.0%	53.4%
tr|F1SCY2|F1SCY2	4	2	3	3	3	1	1	1	5	19	20	8	18	2	2	4	15.2%	3.9%	9.1%	3.9%	6.5%	1.8%	2.6%	2.6%
tr|F1RIT1|F1RIT1	5	1	1	0	2	2	5	17	5	1	1	0	3	4	30	90	7.2%	1.3%	1.5%	0.0%	3.1%	1.5%	8.6%	27.9%
tr|I3LF17|I3LF17	5	1	1	0	2	2	5	13	5	1	1	0	3	4	30	65	7.2%	1.3%	1.5%	0.0%	3.1%	1.5%	8.6%	19.6%
tr|F1RLQ9|F1RLQ9	4	3	1	2	3	2	0	6	5	27	4	22	12	3	0	21	6.5%	4.4%	1.4%	2.2%	3.4%	3.1%	0.0%	10.4%
tr|I3LAJ6|I3LAJ6	5	1	4	3	1	0	4	12	6	1	9	7	1	0	105	273	30.6%	5.3%	18.4%	15.5%	5.3%	0.0%	31.6%	40.3%
tr|F2Z5K4|F2Z5K4	11	10	3	3	9	7	6	10	52	104	16	74	79	24	41	82	57.5%	40.9%	12.4%	10.9%	40.9%	19.2%	38.3%	57.0%
Q29549	8	2	10	2	6	2	5	12	13	2	50	3	12	2	133	80	20.0%	4.5%	26.7%	3.6%	20.0%	8.5%	15.5%	26.5%
tr|F1RRP6|F1RRP6	13	10	17	6	12	7	13	29	36	25	121	33	26	10	363	385	53.7%	29.9%	57.9%	18.6%	42.1%	24.4%	51.1%	66.2%
tr|I3L8J8|I3L8J8	5	0	1	2	3	0	2	9	9	0	5	5	5	0	15	42	4.9%	0.0%	1.4%	2.3%	2.2%	0.0%	2.6%	11.3%
tr|F2Z5K3|F2Z5K3	6	5	7	3	7	0	5	11	18	16	28	15	22	0	277	190	28.2%	43.7%	23.9%	33.1%	34.5%	0.0%	35.9%	45.1%
tr|F2Z5J9|F2Z5J9	7	4	3	3	2	0	2	5	11	12	13	14	11	0	10	13	41.7%	22.5%	14.2%	15.6%	19.3%	0.0%	16.5%	28.4%
P05027	9	1	7	2	4	3	8	11	22	2	79	8	14	4	361	619	37.0%	7.3%	28.7%	8.3%	19.8%	12.5%	32.0%	32.7%
tr|I3LL41|I3LL41	3	2	3	3	2	3	2	1	21	13	46	22	11	3	227	1	20.2%	17.1%	27.9%	17.1%	11.6%	31.8%	20.2%	11.6%
tr|J9JIL3|J9JIL3	9	1	7	2	4	2	8	11	22	2	79	8	14	3	361	621	41.5%	8.1%	32.2%	9.3%	22.2%	9.3%	35.9%	36.7%
tr|F1SSP6|F1SSP6	2	2	1	2	2	2	1	1	8	21	1	16	9	5	9	2	2.7%	2.7%	2.7%	2.7%	2.7%	2.7%	2.7%	2.7%
tr|F1RYZ0|F1RYZ0	20	10	13	5	5	3	2	9	70	38	132	69	32	10	16	52	97.4%	89.6%	77.4%	16.5%	48.7%	16.5%	30.4%	63.5%
tr|F1S0K1|F1S0K1	1	2	2	0	5	0	1	1	3	4	30	0	7	0	1	5	2.4%	4.0%	4.0%	0.0%	8.4%	0.0%	2.4%	2.4%
tr|F1STG5|F1STG5	4	1	0	1	2	0	1	3	4	1	0	2	3	0	2	6	4.4%	0.9%	0.0%	0.6%	2.1%	0.0%	0.9%	2.6%
tr|F1S7W0|F1S7W0	3	1	12	1	1	0	4	8	3	2	30	4	1	0	18	26	1.9%	0.6%	17.2%	0.6%	0.6%	0.0%	5.2%	8.2%
tr|I3LA58|I3LA58	3	2	1	1	2	0	1	4	3	2	6	2	3	0	2	8	3.1%	2.2%	2.4%	0.7%	2.3%	0.0%	1.0%	6.0%
P79382	1	1	0	1	1	0	0	0	1	4	0	4	1	0	0	0	12.3%	7.7%	0.0%	7.7%	7.7%	0.0%	0.0%	0.0%
tr|F1RXD5|F1RXD5	11	5	1	4	6	1	8	16	24	15	1	20	18	1	59	78	21.6%	10.3%	2.6%	10.7%	12.6%	2.1%	22.3%	33.6%
tr|F1RUD0|F1RUD0	6	2	2	1	2	1	2	6	19	2	10	9	11	1	148	210	17.6%	9.5%	6.5%	6.5%	9.5%	3.0%	9.5%	17.6%
tr|K7GLN3|K7GLN3	6	2	2	1	2	1	2	6	19	2	10	9	11	1	148	210	23.5%	12.6%	8.7%	8.7%	12.6%	4.0%	12.6%	23.5%
tr|F1SCR1|F1SCR1	4	2	4	1	2	2	1	1	7	11	15	2	4	12	6	1	5.0%	1.9%	6.5%	1.2%	2.7%	2.7%	1.2%	1.9%
P01025	9	2	0	4	4	0	4	6	12	3	0	6	9	0	16	19	8.0%	2.3%	0.0%	3.2%	3.8%	0.0%	3.3%	6.9%
P20735	6	1	2	3	3	0	9	12	10	1	16	7	4	0	243	200	11.6%	3.0%	5.1%	5.1%	7.0%	0.0%	16.4%	18.7%
tr|I3LCJ5|I3LCJ5	3	2	0	3	3	2	1	2	8	3	0	6	3	2	8	2	24.5%	7.6%	0.0%	19.5%	19.5%	5.4%	4.3%	12.3%
tr|I3LQG7|I3LQG7	1	3	5	1	3	1	1	3	1	8	10	1	7	1	11	12	10.4%	40.0%	34.1%	8.1%	30.4%	12.6%	17.0%	31.1%
tr|F1RN68|F1RN68	3	1	3	1	1	0	0	3	4	5	7	6	3	0	0	5	2.9%	0.8%	3.9%	0.9%	0.9%	0.0%	0.0%	5.0%
tr|I3L8R1|I3L8R1	2	1	1	0	2	0	2	0	2	4	5	0	6	0	2	0	47.5%	21.3%	26.2%	0.0%	21.3%	0.0%	32.8%	0.0%
tr|I3LKU0|I3LKU0	2	1	3	1	2	0	1	1	3	1	13	2	2	0	3	2	5.7%	5.2%	17.2%	5.2%	10.9%	0.0%	6.2%	6.2%
tr|F1RT83|F1RT83	1	0	1	0	1	1	5	9	3	0	5	0	2	1	118	85	7.8%	0.0%	3.7%	0.0%	7.8%	7.8%	25.3%	32.8%
tr|I3LLN4|I3LLN4	1	0	3	0	0	1	2	6	3	0	5	0	0	3	4	25	2.8%	0.0%	4.1%	0.0%	0.0%	1.3%	2.3%	5.8%
P18648	2	1	2	1	1	0	4	3	2	1	19	2	1	0	46	14	11.7%	4.9%	6.8%	4.9%	4.9%	0.0%	17.7%	11.7%
tr|I3L955|I3L955	0	2	3	1	2	0	3	5	0	3	7	1	2	0	8	16	0.0%	2.5%	8.8%	2.5%	2.5%	0.0%	10.9%	13.5%
tr|I3LI92|I3LI92	1	2	0	1	1	0	0	1	1	2	0	1	2	0	0	5	9.9%	16.1%	0.0%	6.2%	6.2%	0.0%	0.0%	9.9%
tr|I3LI25|I3LI25	1	0	3	0	0	1	1	5	3	0	5	0	0	3	3	15	2.7%	0.0%	3.9%	0.0%	0.0%	1.2%	1.2%	4.6%
tr|I3LAA4|I3LAA4	2	0	0	1	1	0	0	2	3	0	0	2	1	0	0	2	8.1%	0.0%	0.0%	5.7%	5.7%	0.0%	0.0%	14.1%
tr|I3LFK1|I3LFK1	1	1	0	1	0	0	0	1	1	2	0	3	0	0	0	1	11.4%	5.9%	0.0%	5.9%	0.0%	0.0%	0.0%	9.5%
tr|F1SGG0|F1SGG0	0	1	0	1	1	0	0	0	0	3	0	1	2	0	0	0	0.0%	6.5%	0.0%	6.5%	6.5%	0.0%	0.0%	0.0%
tr|I3LBD8|I3LBD8	2	1	0	1	1	0	0	0	2	1	0	2	1	0	0	0	16.3%	4.2%	0.0%	4.2%	4.2%	0.0%	0.0%	0.0%
tr|F1RIV0|F1RIV0	1	1	0	0	1	1	0	0	1	2	0	0	2	1	0	0	5.7%	2.6%	0.0%	0.0%	2.6%	2.6%	0.0%	0.0%
tr|F1SAT6|F1SAT6	1	1	1	1	1	0	0	0	2	1	2	1	2	0	0	0	3.7%	3.7%	3.7%	3.7%	3.7%	0.0%	0.0%	0.0%
tr|I3L6B5|I3L6B5	1	1	1	0	2	0	0	0	1	2	6	0	3	0	0	0	1.8%	1.8%	1.8%	0.0%	3.7%	0.0%	0.0%	0.0%
tr|F1RXF8|F1RXF8	1	1	1	0	2	0	0	0	1	2	6	0	3	0	0	0	1.9%	1.9%	1.9%	0.0%	3.9%	0.0%	0.0%	0.0%
tr|F1ST88|F1ST88	1	0	0	0	2	0	0	4	2	0	0	0	2	0	0	36	14.3%	0.0%	0.0%	0.0%	22.7%	0.0%	0.0%	28.6%
tr|P79388|P79388	1	0	2	1	1	0	1	10	2	0	6	1	1	0	7	97	9.9%	0.0%	14.6%	4.3%	4.3%	0.0%	7.5%	53.0%
tr|F1SM37|F1SM37	2	0	0	0	1	1	2	4	2	0	0	0	1	1	5	7	3.5%	0.0%	0.0%	0.0%	2.2%	1.4%	3.9%	7.1%
tr|F1SM38|F1SM38	2	0	0	0	1	1	2	4	2	0	0	0	1	1	5	7	3.6%	0.0%	0.0%	0.0%	2.2%	1.4%	3.9%	7.2%
tr|F2Z5C9|F2Z5C9	2	0	1	1	1	0	2	1	2	0	1	1	1	0	13	5	12.3%	0.0%	5.7%	10.4%	10.4%	0.0%	16.1%	5.7%
tr|F1S3K9|F1S3K9	0	1	0	0	1	1	1	1	0	2	0	0	1	1	1	1	0.0%	3.2%	0.0%	0.0%	3.2%	3.2%	3.4%	3.4%
tr|F1RUC1|F1RUC1	2	0	0	1	1	0	2	0	2	0	0	1	1	0	2	0	4.6%	0.0%	0.0%	2.7%	1.9%	0.0%	5.7%	0.0%
tr|K7GPU8|K7GPU8	2	0	3	0	1	0	0	3	2	0	7	0	2	0	0	7	33.5%	0.0%	18.4%	0.0%	21.1%	0.0%	0.0%	16.2%
P02009	0	1	0	0	1	0	0	0	0	2	0	0	2	0	0	0	0.0%	6.4%	0.0%	0.0%	6.4%	0.0%	0.0%	0.0%
Q0MS45	0	1	0	0	1	1	0	0	0	2	0	0	1	1	0	0	0.0%	3.3%	0.0%	0.0%	3.3%	3.3%	0.0%	0.0%
tr|F1SDV2|F1SDV2	0	2	0	0	2	0	0	0	0	2	0	0	2	0	0	0	0.0%	2.9%	0.0%	0.0%	3.7%	0.0%	0.0%	0.0%
tr|F1SNV5|F1SNV5	2	0	0	1	1	0	0	0	2	0	0	1	1	0	0	0	8.5%	0.0%	0.0%	5.3%	5.3%	0.0%	0.0%	0.0%
tr|I3LKP2|I3LKP2	0	1	0	1	0	0	0	0	0	2	0	2	0	0	0	0	0.0%	12.7%	0.0%	12.7%	0.0%	0.0%	0.0%	0.0%
tr|F1RGX7|F1RGX7	0	1	0	0	1	0	0	0	0	2	0	0	2	0	0	0	0.0%	6.3%	0.0%	0.0%	6.3%	0.0%	0.0%	0.0%
tr|K7GP35|K7GP35	0	1	1	0	2	0	1	0	0	2	3	0	2	0	2	0	0.0%	21.7%	18.8%	0.0%	40.6%	0.0%	21.7%	0.0%
tr|F1RW43|F1RW43	0	1	1	0	2	0	1	0	0	2	3	0	2	0	2	0	0.0%	9.9%	8.6%	0.0%	18.4%	0.0%	9.9%	0.0%
tr|F1S1D6|F1S1D6	1	1	2	1	1	0	2	2	1	1	22	1	1	0	8	4	1.3%	1.2%	3.5%	1.2%	1.2%	0.0%	5.9%	2.3%
tr|F1S0R1|F1S0R1	1	1	2	1	1	0	2	0	1	1	22	1	1	0	8	0	1.3%	1.2%	3.5%	1.2%	1.2%	0.0%	5.8%	0.0%
tr|F1S089|F1S089	1	0	1	1	1	0	0	0	2	0	4	1	1	0	0	0	2.7%	0.0%	4.5%	2.7%	2.7%	0.0%	0.0%	0.0%
tr|F1RIR6|F1RIR6	1	1	1	1	1	0	0	0	1	1	6	1	1	0	0	0	8.6%	8.6%	8.0%	8.6%	8.6%	0.0%	0.0%	0.0%
tr|F1S2B3|F1S2B3	1	0	0	0	0	1	7	19	1	0	0	0	0	1	23	106	0.9%	0.0%	0.0%	0.0%	0.0%	1.4%	7.9%	22.6%
tr|K7GQP7|K7GQP7	1	0	0	0	0	1	7	19	1	0	0	0	0	1	23	106	0.9%	0.0%	0.0%	0.0%	0.0%	1.6%	8.6%	24.5%
tr|K7GRJ7|K7GRJ7	1	0	0	0	0	1	7	19	1	0	0	0	0	1	23	106	0.9%	0.0%	0.0%	0.0%	0.0%	1.5%	8.3%	23.5%
tr|K7GMU3|K7GMU3	1	0	0	0	0	1	4	18	1	0	0	0	0	1	12	66	1.0%	0.0%	0.0%	0.0%	0.0%	1.6%	6.0%	26.1%

	Peptide Count								Spectral Count								Sequence Coverage							
	Whole Cell	Whole Cell	Whole Cell	Cilia Fraction	Cilia Fraction	Cilia Fraction	Cilia Fraction	Cilia Fraction	Whole Cell	Whole Cell	Whole Cell	Cilia Fraction	Cilia Fraction	Cilia Fraction	Cilia Fraction	Cilia Fraction	Whole Cell	Whole Cell	Whole Cell	Cilia Fraction	Cilia Fraction	Cilia Fraction	Cilia Fraction	Cilia Fraction
Sample#	1	2	6	4	3	5	7	8	1	2	6	4	3	5	7	8	1	2	6	4	3	5	7	8
**Accession**																								
tr|F1RI78|F1RI78	1	0	0	1	0	0	2	2	1	0	0	1	0	0	13	30	2.7%	0.0%	0.0%	2.5%	0.0%	0.0%	8.4%	8.4%
tr|I3L5L9|I3L5L9	1	0	0	0	1	0	3	2	1	0	0	0	1	0	27	9	5.0%	0.0%	0.0%	0.0%	4.5%	0.0%	19.8%	14.0%
tr|F1RFW5|F1RFW5	1	0	0	0	1	0	3	2	1	0	0	0	1	0	27	9	5.9%	0.0%	0.0%	0.0%	5.4%	0.0%	23.6%	16.7%
tr|Q767M4|Q767M4	1	0	1	0	0	1	0	8	1	0	3	0	0	1	0	60	3.6%	0.0%	2.3%	0.0%	0.0%	1.2%	0.0%	18.1%
tr|F1S7A4|F1S7A4	1	0	3	0	1	0	4	9	1	0	15	0	1	0	19	116	3.1%	0.0%	12.1%	0.0%	5.0%	0.0%	18.3%	33.1%
tr|I3L876|I3L876	0	1	0	0	1	0	1	0	0	1	0	0	1	0	1	0	0.0%	20.2%	0.0%	0.0%	20.2%	0.0%	20.2%	0.0%
tr|I3LLW9|I3LLW9	1	0	0	0	1	0	0	1	1	0	0	0	1	0	0	1	13.1%	0.0%	0.0%	0.0%	13.1%	0.0%	0.0%	13.1%
Q29550	1	0	7	1	0	0	2	8	1	0	21	1	0	0	9	21	4.2%	0.0%	18.2%	1.2%	0.0%	0.0%	5.1%	20.7%
tr|F1RF15|F1RF15	1	0	6	1	0	0	1	7	1	0	18	1	0	0	2	19	5.0%	0.0%	18.3%	1.5%	0.0%	0.0%	3.1%	21.4%
tr|F1RF16|F1RF16	1	0	4	1	0	0	0	5	1	0	14	1	0	0	0	15	8.4%	0.0%	22.8%	2.5%	0.0%	0.0%	0.0%	30.2%
tr|F1SC85|F1SC85	1	0	0	1	0	0	0	0	1	0	0	1	0	0	0	0	2.2%	0.0%	0.0%	1.4%	0.0%	0.0%	0.0%	0.0%
tr|I3L5Z3|I3L5Z3	0	1	0	0	1	0	0	0	0	1	0	0	1	0	0	0	0.0%	0.8%	0.0%	0.0%	0.8%	0.0%	0.0%	0.0%
tr|F1S4E4|F1S4E4	0	1	0	1	0	0	0	0	0	1	0	1	0	0	0	0	0.0%	1.6%	0.0%	1.6%	0.0%	0.0%	0.0%	0.0%
tr|F1SAQ3|F1SAQ3	1	0	0	0	1	0	0	0	1	0	0	0	1	0	0	0	4.1%	0.0%	0.0%	0.0%	5.0%	0.0%	0.0%	0.0%
Reverse_tr|F1STN7|F1STN7	1	0	0	0	1	0	0	0	1	0	0	0	1	0	0	0	4.6%	0.0%	0.0%	0.0%	4.6%	0.0%	0.0%	0.0%
tr|I3L7Y5|I3L7Y5	0	1	0	0	1	0	0	0	0	1	0	0	1	0	0	0	0.0%	20.4%	0.0%	0.0%	20.4%	0.0%	0.0%	0.0%
tr|F1STR4|F1STR4	1	0	0	0	1	0	0	0	1	0	0	0	1	0	0	0	5.8%	0.0%	0.0%	0.0%	2.4%	0.0%	0.0%	0.0%
tr|F1RQM9|F1RQM9	0	1	0	1	0	0	0	0	0	1	0	1	0	0	0	0	0.0%	0.4%	0.0%	0.3%	0.0%	0.0%	0.0%	0.0%
tr|F1RFG6|F1RFG6	1	0	0	1	0	0	0	0	1	0	0	1	0	0	0	0	3.2%	0.0%	0.0%	1.2%	0.0%	0.0%	0.0%	0.0%
tr|F1RKY2|F1RKY2	0	1	0	0	1	0	0	0	0	1	0	0	1	0	0	0	0.0%	2.8%	0.0%	0.0%	2.8%	0.0%	0.0%	0.0%
tr|F1S9A6|F1S9A6	1	0	0	0	1	0	0	0	1	0	0	0	1	0	0	0	1.1%	0.0%	0.0%	0.0%	0.9%	0.0%	0.0%	0.0%
tr|I3LSQ5|I3LSQ5	1	0	0	0	0	1	0	0	1	0	0	0	0	1	0	0	11.6%	0.0%	0.0%	0.0%	0.0%	11.6%	0.0%	0.0%
tr|F1SSX4|F1SSX4	1	0	0	0	1	0	0	0	1	0	0	0	1	0	0	0	19.8%	0.0%	0.0%	0.0%	14.0%	0.0%	0.0%	0.0%
Reverse_tr|F1STM4|F1STM4	1	0	0	0	1	0	0	0	1	0	0	0	1	0	0	0	4.6%	0.0%	0.0%	0.0%	4.6%	0.0%	0.0%	0.0%
tr|I3L6R7|I3L6R7	1	0	0	0	0	1	0	0	1	0	0	0	0	1	0	0	4.3%	0.0%	0.0%	0.0%	0.0%	4.3%	0.0%	0.0%
Reverse_tr|F1SRK1|F1SRK1	1	0	0	0	1	0	0	0	1	0	0	0	1	0	0	0	1.3%	0.0%	0.0%	0.0%	1.3%	0.0%	0.0%	0.0%
tr|Q6YT39|Q6YT39	0	1	0	0	1	0	0	0	0	1	0	0	1	0	0	0	0.0%	1.3%	0.0%	0.0%	1.3%	0.0%	0.0%	0.0%
tr|F1SUK5|F1SUK5	0	1	0	0	1	0	0	0	0	1	0	0	1	0	0	0	0.0%	0.6%	0.0%	0.0%	1.2%	0.0%	0.0%	0.0%
tr|F1RQR3|F1RQR3	0	1	0	0	0	1	0	0	0	1	0	0	0	1	0	0	0.0%	1.0%	0.0%	0.0%	0.0%	1.0%	0.0%	0.0%
P14632	0	1	0	0	1	0	0	0	0	1	0	0	1	0	0	0	0.0%	1.3%	0.0%	0.0%	1.3%	0.0%	0.0%	0.0%
tr|F1S8X7|F1S8X7	1	0	1	0	1	0	0	0	1	0	1	0	1	0	0	0	1.7%	0.0%	2.7%	0.0%	4.0%	0.0%	0.0%	0.0%
tr|I3LI22|I3LI22	0	1	1	0	1	0	0	0	0	1	2	0	1	0	0	0	0.0%	0.4%	0.6%	0.0%	0.6%	0.0%	0.0%	0.0%
tr|F1SE95|F1SE95	0	1	1	0	1	0	0	0	0	1	2	0	1	0	0	0	0.0%	0.4%	0.5%	0.0%	0.6%	0.0%	0.0%	0.0%
tr|I3LSI9|I3LSI9	1	0	1	0	1	0	0	0	1	0	2	0	1	0	0	0	27.7%	0.0%	15.4%	0.0%	35.4%	0.0%	0.0%	0.0%
tr|I3LV95|I3LV95	1	0	1	0	0	1	1	0	1	0	11	0	0	1	2	0	8.2%	0.0%	8.2%	0.0%	0.0%	10.4%	8.2%	0.0%
tr|F1RFJ3|F1RFJ3	1	0	1	0	0	1	1	0	1	0	11	0	0	1	2	0	8.5%	0.0%	8.5%	0.0%	0.0%	10.8%	8.5%	0.0%
tr|F1RTQ6|F1RTQ6	1	0	1	0	0	1	1	0	1	0	11	0	0	1	2	0	8.7%	0.0%	8.7%	0.0%	0.0%	11.0%	8.7%	0.0%
tr|I3LPX9|I3LPX9	1	0	1	0	1	0	0	0	1	0	3	0	1	0	0	0	1.4%	0.0%	1.5%	0.0%	1.8%	0.0%	0.0%	0.0%
tr|D0G7F3|D0G7F3	1	0	1	0	1	0	0	0	1	0	3	0	1	0	0	0	3.3%	0.0%	4.1%	0.0%	6.8%	0.0%	0.0%	0.0%
tr|F1RJB1|F1RJB1	0	1	1	0	1	0	0	0	0	1	8	0	1	0	0	0	0.0%	1.8%	1.8%	0.0%	1.1%	0.0%	0.0%	0.0%
tr|F1RN44|F1RN44	1	1	0	1	1	1	0	2	6	33	0	24	7	7	0	37	2.4%	2.4%	0.0%	2.4%	2.4%	2.4%	0.0%	6.1%
tr|I3LCP0|I3LCP0	2	3	4	1	2	3	3	6	7	50	4	22	19	14	13	68	2.3%	4.1%	4.9%	1.3%	2.7%	2.6%	4.1%	7.4%
tr|F1RU49|F1RU49	2	3	4	1	2	3	3	6	7	50	4	22	19	14	13	68	2.3%	4.1%	5.0%	1.3%	2.8%	2.7%	4.1%	7.5%
P05024	26	11	21	14	14	2	23	44	71	25	142	53	37	2	1448	1134	27.6%	11.2%	23.4%	14.7%	15.3%	3.2%	25.7%	34.1%
tr|I3LS04|I3LS04	7	5	5	2	6	1	5	12	12	8	40	8	10	1	89	190	18.7%	12.8%	15.3%	5.5%	21.4%	2.5%	18.5%	29.6%
tr|F1RHL9|F1RHL9	3	3	5	1	2	3	5	11	8	50	13	22	19	14	28	141	3.5%	4.1%	6.3%	1.3%	2.8%	2.7%	7.0%	13.1%
tr|F1RTQ8|F1RTQ8	8	2	5	5	4	1	4	13	11	8	14	10	7	1	12	50	11.3%	1.8%	5.2%	5.6%	5.1%	1.0%	4.9%	13.8%
tr|I3LVS7|I3LVS7	12	10	3	3	9	7	6	11	79	101	20	71	75	24	74	115	54.9%	40.9%	12.4%	10.9%	40.9%	19.2%	38.3%	63.2%
Q29548	3	4	2	1	1	3	3	3	6	11	5	6	5	5	8	9	6.6%	8.7%	3.2%	1.9%	1.9%	6.2%	8.3%	6.6%
tr|D0G6X8|D0G6X8	3	4	2	1	1	3	3	3	6	11	5	6	5	5	8	9	6.5%	8.6%	3.2%	1.9%	1.9%	6.1%	8.2%	6.5%
tr|F1RS45|F1RS45	5	2	4	2	3	1	0	0	5	12	23	7	8	1	0	0	3.8%	1.6%	3.8%	1.7%	1.7%	0.7%	0.0%	0.0%
Q007T5	4	4	1	2	8	1	5	9	11	5	11	2	12	1	37	55	32.0%	29.7%	12.0%	12.0%	53.1%	11.4%	28.0%	37.1%
P02543	33	9	29	10	13	5	13	22	84	70	126	46	80	18	276	207	64.2%	20.0%	45.7%	23.2%	24.0%	9.0%	33.9%	47.4%
Q9MZ15	2	3	11	0	3	7	5	5	9	6	605	0	3	11	45	19	6.8%	10.2%	41.2%	0.0%	10.2%	17.3%	31.3%	31.3%
tr|F1S2F6|F1S2F6	2	3	8	0	3	7	4	4	9	6	592	0	3	11	19	13	6.8%	10.2%	30.5%	0.0%	10.2%	17.3%	23.4%	23.4%
tr|F1RLM1|F1RLM1	7	1	1	2	2	0	2	2	12	2	3	7	6	0	8	7	6.2%	1.2%	0.9%	1.8%	1.2%	0.0%	2.0%	2.8%
tr|F1RRE0|F1RRE0	6	3	0	2	2	1	0	2	8	6	0	7	5	1	0	3	20.0%	7.1%	0.0%	6.5%	6.5%	4.0%	0.0%	9.8%
tr|F1SUF2|F1SUF2	6	5	21	4	4	5	0	2	14	22	235	9	11	13	0	3	10.5%	6.5%	27.0%	4.9%	4.9%	5.1%	0.0%	6.4%
tr|K7GM45|K7GM45	7	2	0	3	4	1	8	9	9	12	0	7	9	3	45	50	17.0%	5.7%	0.0%	9.8%	11.3%	2.0%	20.5%	28.6%
tr|F1SEK8|F1SEK8	7	2	0	3	4	1	8	8	9	12	0	7	9	3	45	49	16.8%	5.7%	0.0%	9.7%	11.1%	2.0%	20.3%	20.1%
tr|F1S2J6|F1S2J6	3	1	0	0	1	3	0	1	6	4	0	0	1	8	0	19	5.8%	1.5%	0.0%	0.0%	1.5%	2.3%	0.0%	1.5%
tr|F1SG66|F1SG66	4	4	9	0	3	1	0	0	5	5	46	0	7	2	0	0	8.0%	6.8%	17.0%	0.0%	5.1%	2.0%	0.0%	0.0%
A7VK00	4	4	10	0	3	1	0	0	5	5	47	0	7	2	0	0	8.0%	6.8%	19.0%	0.0%	5.1%	2.0%	0.0%	0.0%
tr|I3LF24|I3LF24	2	2	1	3	2	2	2	1	4	45	3	17	12	15	218	109	32.5%	12.5%	26.2%	32.5%	12.5%	12.5%	38.8%	12.5%
tr|I3LR97|I3LR97	3	2	0	1	1	1	0	0	6	3	0	3	3	2	0	0	20.1%	14.6%	0.0%	6.5%	6.5%	6.5%	0.0%	0.0%
tr|F1SMX5|F1SMX5	3	2	0	1	1	1	0	0	6	3	0	3	3	2	0	0	11.7%	8.5%	0.0%	3.8%	3.8%	3.8%	0.0%	0.0%
tr|F1SAX3|F1SAX3	30	13	24	14	14	2	26	51	77	27	167	53	37	2	1480	1336	32.4%	15.8%	25.3%	14.7%	15.3%	3.2%	27.5%	38.7%
tr|F1RSZ8|F1RSZ8	10	3	1	6	6	4	8	15	18	48	3	21	18	19	462	769	22.0%	4.9%	4.9%	13.0%	13.9%	9.5%	24.8%	25.8%
tr|I3LVT1|I3LVT1	3	1	1	2	2	0	1	1	22	1	3	8	12	0	8	1	30.7%	18.8%	18.8%	18.8%	18.8%	0.0%	18.8%	18.8%
tr|F1RI39|F1RI39	12	7	13	6	9	6	14	22	33	97	69	51	35	26	92	230	16.5%	8.8%	19.1%	5.9%	10.1%	6.1%	23.3%	31.1%
tr|F1SBS4|F1SBS4	5	1	0	2	2	0	0	3	6	1	0	2	4	0	0	11	6.1%	1.2%	0.0%	2.2%	2.0%	0.0%	0.0%	3.9%
tr|F1SJF9|F1SJF9	4	1	0	1	2	0	1	0	5	1	0	2	3	0	2	0	21.1%	7.5%	0.0%	7.5%	7.5%	0.0%	7.5%	0.0%
tr|F1RLB6|F1RLB6	2	2	3	2	2	0	0	3	4	2	11	2	3	0	0	5	11.2%	11.2%	13.2%	8.8%	8.8%	0.0%	0.0%	12.2%
tr|F1RRD6|F1RRD6	17	3	7	5	6	0	11	14	42	9	23	22	20	0	141	76	30.4%	4.3%	10.4%	8.0%	9.5%	0.0%	22.8%	23.2%
tr|F1S2H7|F1S2H7	10	6	8	3	9	2	4	5	14	11	30	4	13	3	13	18	16.3%	8.9%	13.4%	4.9%	11.4%	2.5%	8.8%	12.1%
tr|K7GN58|K7GN58	2	1	1	2	1	0	4	3	4	1	12	3	1	0	40	52	11.2%	7.1%	7.1%	7.1%	7.1%	0.0%	19.6%	11.2%
tr|K7GQM9|K7GQM9	2	1	1	2	1	0	3	3	4	1	12	3	1	0	36	52	13.2%	8.3%	8.3%	8.3%	8.3%	0.0%	12.2%	13.2%
tr|F1S9G9|F1S9G9	5	0	1	1	2	0	1	2	5	0	2	1	3	0	6	6	18.2%	0.0%	4.8%	6.1%	7.6%	0.0%	7.6%	7.6%
tr|F1RQH5|F1RQH5	3	2	0	1	1	0	1	0	3	2	0	1	3	0	2	0	7.9%	3.9%	0.0%	2.3%	2.3%	0.0%	4.0%	0.0%
tr|I3L9S4|I3L9S4	3	0	0	0	1	1	0	1	5	0	0	0	2	2	0	1	25.4%	0.0%	0.0%	0.0%	9.4%	9.4%	0.0%	9.4%
tr|F2Z5G1|F2Z5G1	4	0	1	1	2	0	0	0	5	0	2	2	2	0	0	0	10.0%	0.0%	5.2%	4.7%	10.0%	0.0%	0.0%	0.0%

	Peptide Count								Spectral Count								Sequence Coverage							
	Whole Cell	Whole Cell	Whole Cell	Cilia Fraction	Cilia Fraction	Cilia Fraction	Cilia Fraction	Cilia Fraction	Whole Cell	Whole Cell	Whole Cell	Cilia Fraction	Cilia Fraction	Cilia Fraction	Cilia Fraction	Cilia Fraction	Whole Cell	Whole Cell	Whole Cell	Cilia Fraction	Cilia Fraction	Cilia Fraction	Cilia Fraction	Cilia Fraction
Sample#	1	2	6	4	3	5	7	8	1	2	6	4	3	5	7	8	1	2	6	4	3	5	7	8
**Accession**																								
tr|F2Z5N6|F2Z5N6	3	1	1	0	2	0	0	0	4	1	4	0	4	0	0	0	13.1%	8.9%	7.2%	0.0%	13.8%	0.0%	0.0%	0.0%
tr|I3LB80|I3LB80	9	3	4	3	2	1	6	15	16	29	7	21	8	6	97	177	18.3%	6.3%	13.0%	8.1%	5.6%	2.6%	23.6%	29.6%
tr|K7GPK0|K7GPK0	3	1	1	3	2	1	9	13	4	18	7	8	5	4	123	62	3.1%	1.2%	2.3%	3.5%	5.4%	1.2%	13.1%	17.2%
P80928	7	6	3	4	4	3	1	3	61	88	136	61	44	7	14	29	54.8%	57.4%	35.7%	35.7%	35.7%	27.8%	18.3%	36.5%
tr|F1SMF4|F1SMF4	4	1	1	3	3	1	10	19	6	18	7	8	6	4	180	163	5.0%	0.9%	1.8%	2.7%	6.2%	0.9%	12.8%	20.4%
tr|K7GP68|K7GP68	4	1	1	3	3	1	10	19	6	18	7	8	6	4	180	163	4.9%	0.9%	1.8%	2.7%	6.1%	0.9%	12.6%	20.1%
tr|F1S078|F1S078	5	1	3	1	1	0	0	0	8	4	10	4	5	0	0	0	24.1%	3.2%	13.5%	3.2%	3.2%	0.0%	0.0%	0.0%
tr|I3LV17|I3LV17	6	1	2	2	1	0	2	15	7	1	3	5	1	0	50	239	30.2%	5.2%	10.8%	8.5%	5.2%	0.0%	23.6%	51.4%
tr|F1SGQ0|F1SGQ0	3	3	1	1	2	0	0	0	3	5	5	2	4	0	0	0	12.5%	9.3%	6.1%	6.1%	6.1%	0.0%	0.0%	0.0%
tr|F2Z4Z0|F2Z4Z0	1	1	2	1	1	0	6	6	3	1	4	2	1	0	51	37	9.0%	7.5%	10.4%	7.5%	7.5%	0.0%	32.8%	32.8%
tr|F1CK17|F1CK17	4	0	1	2	1	0	2	10	4	0	2	2	1	0	11	50	4.5%	0.0%	2.0%	1.7%	1.7%	0.0%	2.7%	15.6%
tr|F1RP57|F1RP57	2	0	0	1	2	0	1	6	4	0	0	1	2	0	1	27	9.4%	0.0%	0.0%	3.0%	5.4%	0.0%	4.1%	17.7%
tr|F1S0L6|F1S0L6	2	2	2	1	1	0	1	1	2	2	7	1	2	0	2	5	13.8%	9.2%	8.1%	3.5%	3.5%	0.0%	5.7%	5.7%
tr|I3L7R0|I3L7R0	5	3	1	3	3	2	1	1	12	40	5	16	18	4	4	3	13.9%	5.2%	5.2%	5.0%	5.2%	5.2%	2.5%	7.3%
tr|A5GFU4|A5GFU4	5	1	1	1	4	0	6	11	8	3	2	1	7	0	39	175	30.2%	3.1%	2.5%	3.1%	19.5%	0.0%	22.0%	37.7%
tr|A5GFU3|A5GFU3	5	1	1	1	4	0	5	11	8	3	2	1	7	0	34	175	25.0%	2.6%	2.1%	2.6%	16.1%	0.0%	15.1%	31.2%
P62802	9	6	8	1	5	5	2	1	17	19	78	2	15	9	213	40	53.4%	42.7%	53.4%	11.7%	35.0%	36.9%	23.3%	11.7%
tr|F1RFV9|F1RFV9	5	2	0	1	2	0	1	3	5	2	0	2	3	0	2	8	4.5%	1.5%	0.0%	0.6%	1.3%	0.0%	0.9%	4.3%
tr|F2Z5W6|F2Z5W6	2	2	0	1	1	0	0	1	2	5	0	1	4	0	0	1	15.5%	15.5%	0.0%	8.1%	8.1%	0.0%	0.0%	9.9%
tr|F1RK90|F1RK90	2	1	1	1	1	1	0	2	2	5	4	3	1	1	0	3	2.6%	0.7%	1.4%	0.7%	0.7%	0.7%	0.0%	2.6%
tr|F2Z4Z4|F2Z4Z4	1	2	0	1	1	0	0	0	3	4	0	1	4	0	0	0	4.7%	7.1%	0.0%	4.2%	4.2%	0.0%	0.0%	0.0%
tr|I3LL16|I3LL16	2	1	1	1	1	0	0	0	2	5	5	3	2	0	0	0	7.7%	3.3%	5.9%	3.3%	3.3%	0.0%	0.0%	0.0%
tr|A7U5U2|A7U5U2	7	7	4	2	10	4	3	7	24	48	190	14	31	6	254	148	25.3%	24.9%	20.5%	10.9%	24.9%	24.9%	16.6%	25.8%
P63053	7	7	4	2	10	4	3	7	24	48	189	14	31	6	252	148	45.3%	44.5%	36.7%	19.5%	44.5%	44.5%	29.7%	46.1%
tr|F1SGG2|F1SGG2	42	20	34	3	21	5	10	10	95	86	203	12	108	7	58	62	67.9%	47.9%	46.0%	12.5%	40.5%	11.5%	20.4%	27.8%
tr|F1STW3|F1STW3	2	1	1	1	2	0	2	4	2	8	11	3	4	0	114	37	9.4%	4.1%	7.9%	5.3%	9.4%	0.0%	7.9%	21.8%
tr|F1SU04|F1SU04	1	3	0	2	2	2	2	7	1	12	0	4	3	2	14	21	2.3%	5.3%	0.0%	3.5%	3.5%	3.5%	4.4%	14.2%
tr|I3LG74|I3LG74	4	2	0	2	2	0	0	0	11	2	0	4	5	0	0	0	22.3%	17.3%	0.0%	9.5%	9.5%	0.0%	0.0%	0.0%
P0CG68	7	7	4	2	10	4	3	7	24	50	194	14	31	6	263	150	10.9%	10.7%	8.8%	4.7%	10.7%	10.7%	7.1%	11.1%
P02067	2	4	0	1	3	2	2	2	11	56	0	2	29	15	434	656	8.2%	15.0%	0.0%	6.8%	15.0%	8.2%	14.3%	14.3%
tr|F1RII7|F1RII7	2	4	0	1	3	2	2	2	11	56	0	2	29	15	434	656	8.2%	15.0%	0.0%	6.8%	15.0%	8.2%	14.3%	14.3%
Q07717	4	5	1	2	3	4	0	4	16	51	22	13	21	12	0	12	36.4%	38.1%	10.2%	18.6%	29.7%	38.1%	0.0%	45.8%
tr|F1STS9|F1STS9	5	3	3	4	2	0	0	1	9	6	14	6	4	0	0	17	15.2%	6.9%	12.5%	8.5%	4.5%	0.0%	0.0%	5.3%
tr|I3LIK6|I3LIK6	8	5	6	4	5	2	3	7	20	46	74	22	11	11	28	37	37.2%	20.9%	30.6%	10.9%	15.9%	7.0%	15.5%	42.6%
tr|K7GPM6|K7GPM6	2	1	1	1	1	0	0	2	6	6	9	3	5	0	0	3	11.9%	4.2%	4.0%	4.2%	4.2%	0.0%	0.0%	9.9%
tr|F1RUX1|F1RUX1	7	0	2	0	2	0	1	9	9	0	14	0	6	0	6	61	16.3%	0.0%	5.2%	0.0%	4.0%	0.0%	4.0%	17.7%
tr|F1SS81|F1SS81	2	1	0	0	2	1	2	1	6	3	0	0	4	2	4	2	19.6%	11.2%	0.0%	0.0%	19.6%	11.2%	8.4%	8.4%
tr|I3LD72|I3LD72	3	1	1	1	3	0	0	2	4	5	1	2	4	0	0	4	10.5%	2.0%	1.7%	2.0%	6.1%	0.0%	0.0%	3.9%
tr|I3LQU0|I3LQU0	4	3	0	1	1	0	0	0	6	3	0	5	1	0	0	0	29.2%	16.5%	0.0%	6.0%	6.0%	0.0%	0.0%	0.0%
tr|I3LBM1|I3LBM1	2	2	2	0	2	1	0	0	5	4	8	0	5	1	0	0	5.1%	6.0%	6.9%	0.0%	6.0%	3.6%	0.0%	0.0%
tr|F1S9L3|F1S9L3	2	2	2	0	2	1	0	0	5	4	8	0	5	1	0	0	5.1%	6.0%	6.9%	0.0%	6.0%	3.6%	0.0%	0.0%
tr|I3LVR9|I3LVR9	2	2	2	0	2	1	0	0	5	4	8	0	5	1	0	0	5.5%	6.4%	7.3%	0.0%	6.4%	3.8%	0.0%	0.0%
tr|I3LQT4|I3LQT4	2	1	2	0	1	0	1	1	5	1	10	0	4	0	1	3	8.1%	8.6%	17.1%	0.0%	8.6%	0.0%	8.1%	8.1%
tr|F1SFC9|F1SFC9	0	1	0	1	1	0	0	0	0	3	0	1	1	0	0	0	0.0%	0.8%	0.0%	0.8%	0.8%	0.0%	0.0%	0.0%
tr|F1SCF9|F1SCF9	1	1	0	1	1	0	0	0	1	2	0	1	1	0	0	0	7.3%	4.0%	0.0%	4.0%	4.0%	0.0%	0.0%	0.0%
tr|F1SB23|F1SB23	2	1	0	1	1	0	0	0	2	1	0	1	1	0	0	0	2.3%	1.0%	0.0%	1.0%	1.0%	0.0%	0.0%	0.0%
tr|I3LLQ0|I3LLQ0	0	1	0	1	1	0	0	0	0	3	0	1	1	0	0	0	0.0%	0.9%	0.0%	0.9%	0.9%	0.0%	0.0%	0.0%
tr|K7GPN9|K7GPN9	0	1	0	1	1	0	0	0	0	3	0	1	1	0	0	0	0.0%	0.9%	0.0%	0.9%	0.9%	0.0%	0.0%	0.0%
tr|F1REZ2|F1REZ2	0	1	0	1	1	0	0	0	0	3	0	1	1	0	0	0	0.0%	0.4%	0.0%	0.4%	0.4%	0.0%	0.0%	0.0%
P02540	2	0	2	1	0	0	1	0	3	0	8	2	0	0	2	0	5.3%	0.0%	4.7%	1.9%	0.0%	0.0%	3.2%	0.0%
tr|F1RTQ5|F1RTQ5	1	1	1	1	0	1	1	0	1	2	11	1	0	1	2	0	8.7%	5.5%	8.7%	5.5%	0.0%	11.0%	8.7%	0.0%
Q8WNW3	14	7	3	5	8	2	10	21	41	14	7	12	16	8	43	198	24.7%	13.2%	5.4%	10.5%	15.6%	2.7%	24.2%	41.5%
tr|F2Z5U0|F2Z5U0	11	7	8	2	10	4	3	7	30	48	235	14	31	6	252	148	58.3%	36.5%	50.6%	16.0%	36.5%	36.5%	24.4%	37.8%
tr|I3LS24|I3LS24	6	4	3	5	2	0	0	1	10	7	14	7	4	0	0	17	18.7%	9.9%	13.3%	11.6%	4.8%	0.0%	0.0%	5.6%
tr|F1S4G6|F1S4G6	2	3	0	2	2	1	0	0	4	13	0	5	5	1	0	0	11.0%	9.8%	0.0%	9.3%	9.3%	4.5%	0.0%	0.0%
tr|F1SES5|F1SES5	7	2	1	2	2	0	2	0	13	26	2	12	13	0	5	0	21.0%	7.3%	2.3%	7.3%	7.3%	0.0%	7.3%	0.0%
tr|I3L9F5|I3L9F5	3	2	0	2	2	1	2	1	3	19	0	7	6	1	9	7	8.6%	5.7%	0.0%	5.7%	5.7%	3.5%	9.2%	5.7%
tr|I3LRA0|I3LRA0	0	1	0	1	1	0	0	0	0	8	0	1	4	0	0	0	0.0%	4.2%	0.0%	4.2%	4.2%	0.0%	0.0%	0.0%
tr|I3L9I6|I3L9I6	3	1	2	1	1	0	0	4	7	6	12	3	5	0	0	11	8.4%	2.4%	7.6%	2.4%	2.4%	0.0%	0.0%	10.9%
tr|F1SCY1|F1SCY1	6	1	8	0	4	2	0	0	10	3	33	0	6	2	0	0	14.0%	2.7%	13.4%	0.0%	7.3%	5.5%	0.0%	0.0%
tr|K7GN56|K7GN56	6	1	8	0	4	2	0	0	10	3	33	0	6	2	0	0	14.0%	2.7%	13.4%	0.0%	7.3%	5.4%	0.0%	0.0%
tr|F1RQR4|F1RQR4	18	5	4	3	5	3	3	9	31	43	19	16	24	5	11	36	41.0%	8.6%	9.0%	3.9%	6.6%	6.1%	9.3%	25.1%
tr|I3LIL4|I3LIL4	30	10	11	6	15	5	7	23	73	54	108	20	46	11	119	219	32.8%	11.8%	14.5%	7.4%	18.6%	5.3%	11.1%	27.4%
tr|F1SE73|F1SE73	6	1	3	2	3	1	1	7	14	1	4	3	5	1	6	30	26.7%	3.3%	13.1%	6.0%	9.3%	5.8%	6.0%	21.4%
tr|F1RKY3|F1RKY3	0	1	0	1	2	1	4	7	0	10	0	2	3	1	17	13	0.0%	0.6%	0.0%	0.6%	1.5%	1.0%	4.1%	4.9%
Q8MJ76	0	1	0	1	0	0	1	0	0	5	0	3	0	0	26	0	0.0%	2.6%	0.0%	2.6%	0.0%	0.0%	2.6%	0.0%
tr|I3LLW3|I3LLW3	1	1	0	1	0	1	0	0	1	4	0	2	0	1	0	0	5.1%	2.8%	0.0%	2.8%	0.0%	2.8%	0.0%	0.0%
tr|F1RZA4|F1RZA4	2	2	2	0	2	0	0	0	3	2	12	0	3	0	0	0	2.1%	1.9%	3.3%	0.0%	2.7%	0.0%	0.0%	0.0%
tr|F1SP42|F1SP42	6	5	8	4	3	0	5	6	11	13	58	8	6	0	36	25	9.2%	6.4%	12.4%	4.8%	3.8%	0.0%	12.0%	14.4%
tr|F2Z560|F2Z560	2	2	4	3	2	0	2	4	9	3	12	5	2	0	32	8	11.6%	13.0%	24.1%	17.1%	11.6%	0.0%	15.3%	26.4%
tr|F1RQE9|F1RQE9	6	5	1	3	7	2	5	5	20	18	1	8	12	2	25	9	5.9%	5.1%	1.0%	3.2%	7.1%	1.2%	5.3%	6.4%
tr|K7GNC3|K7GNC3	7	3	0	1	1	3	0	6	12	7	0	4	3	4	0	60	39.6%	22.5%	0.0%	9.9%	9.9%	22.5%	0.0%	28.8%
tr|F1SNE9|F1SNE9	13	4	2	3	5	2	3	0	23	24	13	6	19	2	6	0	20.4%	6.2%	3.8%	4.1%	6.4%	3.7%	5.7%	0.0%
tr|F1SMJ4|F1SMJ4	1	2	3	1	1	0	2	3	2	12	17	4	4	0	5	15	1.6%	2.7%	8.6%	1.4%	1.4%	0.0%	4.0%	6.5%
tr|I3LU89|I3LU89	1	1	0	0	0	2	0	0	1	6	0	0	0	4	0	0	5.1%	5.1%	0.0%	0.0%	0.0%	5.1%	0.0%	0.0%
tr|F1SMU8|F1SMU8	4	1	0	1	2	0	0	0	4	3	0	1	3	0	0	0	4.1%	1.0%	0.0%	1.3%	2.3%	0.0%	0.0%	0.0%
tr|F1SSI2|F1SSI2	1	3	1	1	0	1	0	0	1	6	1	3	0	1	0	0	4.1%	9.7%	5.9%	2.9%	0.0%	6.8%	0.0%	0.0%
tr|F1SS51|F1SS51	4	1	1	0	1	1	0	0	5	2	4	0	2	2	0	0	13.0%	4.3%	3.2%	0.0%	4.3%	4.3%	0.0%	0.0%
tr|I3LT88|I3LT88	16	4	14	8	11	0	10	20	52	13	84	18	19	0	508	609	16.2%	4.2%	14.3%	9.5%	11.9%	0.0%	11.1%	16.9%
tr|F2Z5W2|F2Z5W2	4	3	2	2	5	3	1	4	10	64	6	12	26	4	229	124	18.3%	16.8%	11.5%	12.6%	22.5%	16.8%	6.8%	16.8%
P04178	10	6	1	2	4	4	4	12	64	35	20	11	20	25	144	140	52.3%	38.6%	7.8%	13.1%	15.0%	15.0%	34.6%	52.3%

	Peptide Count								Spectral Count								Sequence Coverage							
	Whole Cell	Whole Cell	Whole Cell	Cilia Fraction	Cilia Fraction	Cilia Fraction	Cilia Fraction	Cilia Fraction	Whole Cell	Whole Cell	Whole Cell	Cilia Fraction	Cilia Fraction	Cilia Fraction	Cilia Fraction	Cilia Fraction	Whole Cell	Whole Cell	Whole Cell	Cilia Fraction	Cilia Fraction	Cilia Fraction	Cilia Fraction	Cilia Fraction
Sample#	1	2	6	4	3	5	7	8	1	2	6	4	3	5	7	8	1	2	6	4	3	5	7	8
**Accession**																								
tr|F1RTA1|F1RTA1	15	5	11	4	7	3	14	37	26	17	59	8	13	3	254	627	17.4%	2.8%	10.4%	2.0%	7.0%	2.5%	11.8%	25.9%
tr|F1SLG5|F1SLG5	10	12	7	2	9	5	3	3	33	37	145	11	18	10	104	26	20.4%	26.4%	13.4%	8.2%	19.1%	10.9%	9.1%	14.6%
tr|F2Z5C1|F2Z5C1	20	12	9	7	11	8	7	5	88	145	135	48	57	24	50	8	58.2%	28.9%	33.0%	24.1%	43.2%	20.7%	32.0%	20.7%
tr|K7GSF0|K7GSF0	8	3	0	1	1	3	0	8	13	7	0	4	3	4	0	77	50.0%	18.1%	0.0%	8.0%	8.0%	18.1%	0.0%	41.3%
tr|F2Z5P1|F2Z5P1	1	3	0	2	2	3	0	2	24	51	0	8	6	27	0	9	22.8%	30.7%	0.0%	22.8%	29.9%	25.2%	0.0%	22.8%
tr|I3LPU8|I3LPU8	7	1	7	1	1	1	2	2	16	6	85	3	7	2	24	5	29.8%	1.6%	40.5%	6.3%	6.3%	6.3%	19.0%	6.8%
tr|I3L973|I3L973	6	1	2	1	1	0	1	1	9	2	7	3	3	0	3	20	22.5%	4.1%	12.0%	4.1%	4.1%	0.0%	4.1%	4.1%
tr|F1SC82|F1SC82	63	26	40	10	22	11	52	77	159	140	294	73	64	25	1373	1135	35.6%	13.6%	21.4%	5.7%	13.0%	5.6%	32.5%	41.5%
tr|J9JIL5|J9JIL5	7	8	1	3	8	5	2	3	79	54	32	19	45	8	46	54	73.5%	73.5%	11.2%	30.6%	73.5%	66.3%	30.6%	26.5%
P31950	7	8	1	3	8	5	2	3	79	54	32	19	45	8	46	54	72.7%	72.7%	11.1%	30.3%	72.7%	65.7%	30.3%	26.3%
tr|I3LP17|I3LP17	6	2	2	2	1	1	3	7	11	28	3	11	4	6	74	60	15.5%	3.2%	9.1%	6.2%	3.2%	3.2%	13.0%	14.6%
tr|I3LCB1|I3LCB1	6	2	2	2	1	1	3	7	11	28	3	11	4	6	74	60	14.1%	2.9%	8.3%	5.6%	2.9%	2.9%	11.8%	13.3%
tr|A5GFU6|A5GFU6	7	1	4	1	4	0	6	14	12	3	6	1	7	0	39	245	32.2%	2.6%	11.6%	2.6%	16.4%	0.0%	18.5%	38.5%
tr|A5GFU2|A5GFU2	7	1	4	1	4	0	6	14	12	3	6	1	7	0	39	245	19.3%	1.6%	7.0%	1.6%	9.8%	0.0%	11.1%	23.1%
P29797	7	1	4	1	4	0	5	14	12	3	6	1	7	0	34	245	30.7%	2.5%	11.1%	2.5%	15.6%	0.0%	14.6%	36.8%
tr|A5GFU7|A5GFU7	7	1	5	1	4	0	6	16	12	3	9	1	7	0	39	254	30.9%	2.5%	16.2%	2.5%	15.7%	0.0%	17.7%	42.0%
tr|A5GFU0|A5GFU0	7	1	5	1	4	0	6	16	12	3	9	1	7	0	39	254	12.1%	1.0%	6.4%	1.0%	6.2%	0.0%	7.0%	16.5%
tr|A5GFU5|A5GFU5	7	1	5	1	4	0	6	16	12	3	9	1	7	0	39	254	31.0%	2.5%	16.2%	2.5%	15.7%	0.0%	17.8%	42.1%
tr|A5GFT9|A5GFT9	7	1	5	1	4	0	6	15	12	3	9	1	7	0	39	252	11.7%	1.0%	6.1%	1.0%	5.9%	0.0%	6.7%	14.4%
tr|F1ST01|F1ST01	12	6	5	4	1	3	2	9	21	32	43	17	6	5	22	49	33.3%	14.4%	12.1%	10.0%	2.8%	11.7%	7.8%	31.6%
tr|F1RWH7|F1RWH7	4	3	1	1	1	1	0	1	5	16	4	5	1	5	0	2	35.2%	18.8%	8.5%	8.9%	8.9%	9.9%	0.0%	10.3%
tr|F1RKU4|F1RKU4	4	3	1	1	2	1	1	1	15	8	12	3	8	1	4	5	23.0%	11.8%	10.1%	7.0%	7.0%	7.0%	7.0%	10.1%
P62844	7	4	2	2	3	2	4	4	58	23	32	22	16	4	52	25	55.9%	37.2%	21.4%	15.2%	15.2%	15.2%	36.6%	36.6%
Q007T2	9	3	4	3	6	3	3	5	23	64	29	14	27	4	272	132	32.5%	16.8%	20.4%	17.8%	27.7%	16.8%	15.7%	25.7%
tr|K7GQ84|K7GQ84	2	5	2	0	4	1	1	0	6	25	15	0	15	1	1	0	23.7%	65.6%	23.7%	0.0%	61.3%	11.8%	11.8%	0.0%
tr|F1SFV8|F1SFV8	2	5	2	0	4	1	1	0	6	25	15	0	15	1	1	0	19.6%	54.5%	19.6%	0.0%	50.9%	9.8%	9.8%	0.0%
tr|F2Z5D2|F2Z5D2	12	7	12	4	4	3	6	9	42	41	155	21	15	6	55	49	48.3%	23.0%	40.7%	11.0%	11.0%	8.1%	26.8%	43.1%
tr|I3LR85|I3LR85	2	1	0	1	1	1	1	2	2	8	0	1	3	1	3	2	5.9%	1.2%	0.0%	1.2%	1.2%	1.2%	2.2%	4.4%
tr|I3LSZ5|I3LSZ5	2	1	0	1	1	0	0	0	2	8	0	1	4	0	0	0	17.5%	3.6%	0.0%	3.6%	3.6%	0.0%	0.0%	0.0%
tr|K7GMI1|K7GMI1	1	2	8	1	1	0	0	4	2	8	37	3	2	0	0	10	1.0%	3.7%	13.8%	1.3%	1.3%	0.0%	0.0%	7.4%
tr|K7GNH3|K7GNH3	1	2	8	1	1	0	0	4	2	8	37	3	2	0	0	10	0.9%	3.4%	12.6%	1.2%	1.2%	0.0%	0.0%	6.8%
tr|F1SJ30|F1SJ30	5	1	4	1	1	0	1	2	9	1	43	2	3	0	1	4	20.1%	3.5%	17.5%	3.5%	3.5%	0.0%	5.0%	6.4%
tr|K7GPL5|K7GPL5	2	2	3	1	2	2	1	1	20	40	126	7	19	4	30	2	15.1%	15.1%	15.1%	15.1%	15.1%	15.1%	15.1%	15.1%
tr|F1RRA6|F1RRA6	4	1	1	1	1	2	0	0	12	10	67	5	4	2	0	0	6.8%	3.7%	3.7%	3.7%	3.7%	4.9%	0.0%	0.0%
tr|F1SKQ9|F1SKQ9	4	1	0	1	1	0	0	3	6	2	0	2	2	0	0	6	28.8%	7.8%	0.0%	7.8%	7.8%	0.0%	0.0%	36.6%
tr|F1SQR1|F1SQR1	2	3	0	1	1	0	0	0	2	6	0	2	2	0	0	0	14.0%	4.5%	0.0%	4.2%	4.2%	0.0%	0.0%	0.0%
tr|F1SG50|F1SG50	2	1	1	0	2	1	0	0	7	1	1	0	3	1	0	0	14.9%	14.9%	7.0%	0.0%	14.9%	14.9%	0.0%	0.0%
tr|I3LNT6|I3LNT6	3	2	1	0	1	0	1	1	4	2	2	0	3	0	6	15	3.6%	3.6%	1.2%	0.0%	2.1%	0.0%	1.6%	1.6%
tr|F1SNQ1|F1SNQ1	5	0	3	0	1	1	0	1	6	0	8	0	1	2	0	1	8.5%	0.0%	3.1%	0.0%	1.8%	1.8%	0.0%	1.4%
tr|I3LGH9|I3LGH9	0	1	2	1	1	0	0	0	0	6	7	2	1	0	0	0	0.0%	5.6%	16.8%	5.6%	5.6%	0.0%	0.0%	0.0%
tr|F1RNS2|F1RNS2	0	1	2	1	1	0	0	0	0	6	7	2	1	0	0	0	0.0%	4.2%	12.7%	4.2%	4.2%	0.0%	0.0%	0.0%
tr|I3LPR3|I3LPR3	1	0	0	0	1	0	0	3	4	0	0	0	2	0	0	37	11.5%	0.0%	0.0%	0.0%	11.5%	0.0%	0.0%	18.6%
tr|K7GMI9|K7GMI9	1	0	0	0	1	0	0	3	4	0	0	0	2	0	0	37	18.8%	0.0%	0.0%	0.0%	18.8%	0.0%	0.0%	30.4%
tr|F1SL24|F1SL24	1	0	0	0	1	0	0	3	4	0	0	0	2	0	0	37	12.1%	0.0%	0.0%	0.0%	12.1%	0.0%	0.0%	19.5%
tr|F1SGI5|F1SGI5	2	1	1	0	0	2	1	1	3	1	2	0	0	2	6	15	4.9%	4.9%	3.8%	0.0%	0.0%	9.8%	4.9%	4.9%
tr|F1RPT5|F1RPT5	2	0	1	1	1	0	1	3	4	0	3	1	1	0	2	17	5.0%	0.0%	1.3%	1.1%	1.1%	0.0%	1.1%	6.4%
tr|I3L819|I3L819	0	1	0	1	1	0	1	0	0	4	0	1	1	0	1	0	0.0%	4.2%	0.0%	4.2%	4.2%	0.0%	4.2%	0.0%
tr|K7GMY8|K7GMY8	1	2	0	1	1	0	0	0	1	3	0	1	1	0	0	0	5.3%	6.8%	0.0%	3.9%	3.9%	0.0%	0.0%	0.0%
tr|F1RQ09|F1RQ09	0	1	0	1	1	0	0	0	0	4	0	1	1	0	0	0	0.0%	4.5%	0.0%	4.5%	4.5%	0.0%	0.0%	0.0%
tr|F1S8C6|F1S8C6	1	2	0	1	1	0	0	0	1	3	0	1	1	0	0	0	3.8%	5.0%	0.0%	2.9%	2.9%	0.0%	0.0%	0.0%
tr|I3LRI1|I3LRI1	0	2	2	1	1	0	0	1	0	4	9	1	1	0	0	2	0.0%	7.6%	6.1%	3.4%	3.4%	0.0%	0.0%	6.1%
tr|I3LD59|I3LD59	0	2	2	1	1	0	0	1	0	4	9	1	1	0	0	2	0.0%	7.2%	5.8%	3.2%	3.2%	0.0%	0.0%	5.8%
tr|F1S0J9|F1S0J9	1	1	1	0	2	0	1	1	3	1	24	0	2	0	1	5	11.8%	11.8%	11.8%	0.0%	35.5%	0.0%	11.8%	11.8%
tr|F1SE08|F1SE08	4	0	2	0	0	2	0	0	4	0	3	0	0	2	0	0	12.4%	0.0%	8.7%	0.0%	0.0%	8.7%	0.0%	0.0%
tr|F1SDZ8|F1SDZ8	2	1	1	0	2	0	0	0	3	1	3	0	2	0	0	0	12.0%	5.0%	3.8%	0.0%	5.0%	0.0%	0.0%	0.0%
tr|F1RF93|F1RF93	0	2	3	1	1	0	0	1	0	4	11	1	1	0	0	2	0.0%	7.5%	12.1%	3.4%	3.4%	0.0%	0.0%	6.0%
tr|F1SMD7|F1SMD7	1	0	0	0	1	0	2	6	2	0	0	0	1	0	8	22	7.0%	0.0%	0.0%	0.0%	8.4%	0.0%	15.4%	34.4%
tr|F1RZL4|F1RZL4	1	0	0	0	0	1	4	2	2	0	0	0	0	1	10	16	2.8%	0.0%	0.0%	0.0%	0.0%	2.2%	8.7%	2.8%
tr|K7GKE2|K7GKE2	2	0	1	0	1	0	1	10	2	0	5	0	1	0	7	75	9.4%	0.0%	7.8%	0.0%	5.2%	0.0%	7.5%	33.2%
tr|K7GSW5|K7GSW5	2	0	4	0	1	0	2	4	2	0	13	0	1	0	20	22	7.8%	0.0%	18.7%	0.0%	7.8%	0.0%	7.8%	14.0%
tr|F1S8R0|F1S8R0	1	1	0	0	1	0	0	1	1	1	0	0	1	0	0	4	3.5%	3.0%	0.0%	0.0%	3.0%	0.0%	0.0%	6.5%
tr|I3L695|I3L695	1	1	0	0	1	0	0	1	1	1	0	0	1	0	0	4	3.5%	3.0%	0.0%	0.0%	3.0%	0.0%	0.0%	6.5%
tr|F1RZL1|F1RZL1	1	1	0	1	0	0	0	2	1	1	0	1	0	0	0	3	11.4%	7.0%	0.0%	4.1%	0.0%	0.0%	0.0%	21.9%
tr|F1RSD1|F1RSD1	0	1	0	0	1	0	1	0	0	2	0	0	1	0	3	0	0.0%	2.4%	0.0%	0.0%	2.4%	0.0%	4.0%	0.0%
tr|K7GRM0|K7GRM0	0	1	0	0	1	0	1	0	0	2	0	0	1	0	2	0	0.0%	15.2%	0.0%	0.0%	15.2%	0.0%	15.2%	0.0%
tr|K7GMG3|K7GMG3	0	1	0	0	1	0	1	0	0	2	0	0	1	0	2	0	0.0%	13.3%	0.0%	0.0%	13.3%	0.0%	13.3%	0.0%
tr|K7GRJ5|K7GRJ5	0	1	0	0	1	0	1	0	0	2	0	0	1	0	2	0	0.0%	42.9%	0.0%	0.0%	42.9%	0.0%	42.9%	0.0%
P35463	0	1	0	1	0	0	1	0	0	2	0	1	0	0	1	0	0.0%	3.4%	0.0%	3.4%	0.0%	0.0%	3.4%	0.0%
Q2HY40	2	0	3	0	1	0	1	2	2	0	15	0	1	0	1	10	9.5%	0.0%	10.7%	0.0%	3.1%	0.0%	3.6%	11.5%
tr|F1S5K2|F1S5K2	0	1	0	1	0	0	0	0	0	2	0	1	0	0	0	0	0.0%	2.1%	0.0%	2.1%	0.0%	0.0%	0.0%	0.0%
tr|I3LSV4|I3LSV4	1	1	0	0	1	0	0	0	1	1	0	0	1	0	0	0	2.9%	2.9%	0.0%	0.0%	2.9%	0.0%	0.0%	0.0%
tr|I3LMM7|I3LMM7	1	1	0	0	1	0	0	0	1	1	0	0	1	0	0	0	3.1%	2.5%	0.0%	0.0%	2.5%	0.0%	0.0%	0.0%
tr|I3LQY9|I3LQY9	0	1	0	0	0	1	0	0	0	2	0	0	0	1	0	0	0.0%	4.0%	0.0%	0.0%	0.0%	4.0%	0.0%	0.0%
tr|F1SIQ2|F1SIQ2	1	1	0	0	1	0	0	0	1	1	0	0	1	0	0	0	4.6%	7.1%	0.0%	0.0%	7.1%	0.0%	0.0%	0.0%
tr|F1RFF1|F1RFF1	1	0	0	0	1	0	0	0	2	0	0	0	1	0	0	0	4.5%	0.0%	0.0%	0.0%	4.5%	0.0%	0.0%	0.0%
tr|I3LVF9|I3LVF9	2	0	0	0	1	0	0	0	2	0	0	0	1	0	0	0	25.8%	0.0%	0.0%	0.0%	11.0%	0.0%	0.0%	0.0%
Reverse_tr|I3LUX5|I3LUX5	1	0	0	0	0	1	0	0	2	0	0	0	0	1	0	0	3.1%	0.0%	0.0%	0.0%	0.0%	3.1%	0.0%	0.0%
tr|F1RJU3|F1RJU3	1	0	0	1	0	0	0	0	2	0	0	1	0	0	0	0	0.8%	0.0%	0.0%	0.8%	0.0%	0.0%	0.0%	0.0%
tr|F1RMP0|F1RMP0	0	1	0	0	1	0	0	0	0	2	0	0	1	0	0	0	0.0%	14.6%	0.0%	0.0%	14.6%	0.0%	0.0%	0.0%
Reverse_tr|F1S5N9|F1S5N9	1	0	0	0	0	1	0	0	2	0	0	0	0	1	0	0	1.0%	0.0%	0.0%	0.0%	0.0%	1.0%	0.0%	0.0%
tr|F1SNS3|F1SNS3	0	1	0	0	0	1	0	0	0	2	0	0	0	1	0	0	0.0%	1.3%	0.0%	0.0%	0.0%	1.3%	0.0%	0.0%

	Peptide Count								Spectral Count								Sequence Coverage							
	Whole Cell	Whole Cell	Whole Cell	Cilia Fraction	Cilia Fraction	Cilia Fraction	Cilia Fraction	Cilia Fraction	Whole Cell	Whole Cell	Whole Cell	Cilia Fraction	Cilia Fraction	Cilia Fraction	Cilia Fraction	Cilia Fraction	Whole Cell	Whole Cell	Whole Cell	Cilia Fraction	Cilia Fraction	Cilia Fraction	Cilia Fraction	Cilia Fraction
Sample#	1	2	6	4	3	5	7	8	1	2	6	4	3	5	7	8	1	2	6	4	3	5	7	8
**Accession**																								
O79881	2	0	0	0	0	1	0	0	2	0	0	0	0	1	0	0	7.6%	0.0%	0.0%	0.0%	0.0%	3.5%	0.0%	0.0%
tr|I3LFH3|I3LFH3	1	1	0	0	1	0	0	0	1	1	0	0	1	0	0	0	1.9%	1.3%	0.0%	0.0%	1.3%	0.0%	0.0%	0.0%
tr|I3LCJ1|I3LCJ1	1	1	0	0	1	0	0	0	1	1	0	0	1	0	0	0	1.9%	1.3%	0.0%	0.0%	1.3%	0.0%	0.0%	0.0%
tr|I3L9K8|I3L9K8	1	1	0	0	1	0	0	0	1	1	0	0	1	0	0	0	13.0%	6.2%	0.0%	0.0%	9.9%	0.0%	0.0%	0.0%
tr|I3L8G9|I3L8G9	1	1	0	0	1	0	0	0	1	1	0	0	1	0	0	0	4.6%	7.1%	0.0%	0.0%	7.1%	0.0%	0.0%	0.0%
tr|F1S762|F1S762	0	1	0	0	1	0	0	0	0	2	0	0	1	0	0	0	0.0%	3.1%	0.0%	0.0%	2.8%	0.0%	0.0%	0.0%
tr|F2Z5C5|F2Z5C5	2	0	0	1	0	0	0	0	2	0	0	1	0	0	0	0	45.0%	0.0%	0.0%	32.5%	0.0%	0.0%	0.0%	0.0%
tr|I3LDR1|I3LDR1	0	1	0	0	1	0	0	0	0	2	0	0	1	0	0	0	0.0%	1.9%	0.0%	0.0%	3.1%	0.0%	0.0%	0.0%
tr|I3LSF4|I3LSF4	0	2	0	0	0	1	0	0	0	2	0	0	0	1	0	0	0.0%	13.7%	0.0%	0.0%	0.0%	13.7%	0.0%	0.0%
tr|F1RPN4|F1RPN4	0	1	0	0	1	0	0	0	0	2	0	0	1	0	0	0	0.0%	2.8%	0.0%	0.0%	2.8%	0.0%	0.0%	0.0%
tr|F2Z5I4|F2Z5I4	0	1	0	0	1	0	0	0	0	2	0	0	1	0	0	0	0.0%	5.6%	0.0%	0.0%	7.2%	0.0%	0.0%	0.0%
tr|F1RUY4|F1RUY4	1	1	0	0	1	0	0	0	1	1	0	0	1	0	0	0	1.8%	1.8%	0.0%	0.0%	1.8%	0.0%	0.0%	0.0%
tr|F1SMA3|F1SMA3	1	1	0	0	1	0	0	0	1	1	0	0	1	0	0	0	3.1%	2.4%	0.0%	0.0%	2.3%	0.0%	0.0%	0.0%
tr|F1S5Y7|F1S5Y7	0	1	0	0	1	0	0	0	0	2	0	0	1	0	0	0	0.0%	2.2%	0.0%	0.0%	2.2%	0.0%	0.0%	0.0%
tr|F1RY51|F1RY51	1	1	0	0	1	0	0	0	1	1	0	0	1	0	0	0	1.7%	0.7%	0.0%	0.0%	0.7%	0.0%	0.0%	0.0%
tr|I3LGD6|I3LGD6	1	0	0	0	1	0	0	0	2	0	0	0	1	0	0	0	7.6%	0.0%	0.0%	0.0%	7.6%	0.0%	0.0%	0.0%
tr|F1SDY0|F1SDY0	0	1	0	0	1	0	0	0	0	2	0	0	1	0	0	0	0.0%	2.0%	0.0%	0.0%	3.1%	0.0%	0.0%	0.0%
tr|F1RY62|F1RY62	1	1	0	1	0	0	0	0	1	1	0	1	0	0	0	0	3.0%	3.0%	0.0%	3.0%	0.0%	0.0%	0.0%	0.0%
tr|I3LTE2|I3LTE2	0	1	0	1	0	0	0	0	0	2	0	1	0	0	0	0	0.0%	6.6%	0.0%	6.6%	0.0%	0.0%	0.0%	0.0%
tr|F1S1U4|F1S1U4	0	1	0	0	1	0	0	0	0	2	0	0	1	0	0	0	0.0%	1.5%	0.0%	0.0%	1.5%	0.0%	0.0%	0.0%
tr|F1RZ01|F1RZ01	1	0	0	1	0	0	0	0	2	0	0	1	0	0	0	0	2.3%	0.0%	0.0%	4.1%	0.0%	0.0%	0.0%	0.0%
tr|F1SB77|F1SB77	2	0	3	0	0	1	0	2	2	0	9	0	0	1	0	4	4.5%	0.0%	4.5%	0.0%	0.0%	2.5%	0.0%	8.6%
tr|F2Z5V3|F2Z5V3	1	1	3	0	1	0	0	1	1	1	10	0	1	0	0	3	9.4%	12.5%	19.5%	0.0%	21.9%	0.0%	0.0%	12.5%
tr|F1S3V5|F1S3V5	1	0	1	1	0	0	0	0	2	0	1	1	0	0	0	0	5.8%	0.0%	5.8%	5.8%	0.0%	0.0%	0.0%	0.0%
tr|F1SN58|F1SN58	0	1	1	0	0	1	0	0	0	2	2	0	0	1	0	0	0.0%	3.6%	3.1%	0.0%	0.0%	3.1%	0.0%	0.0%
tr|F1SN59|F1SN59	0	1	1	0	0	1	0	0	0	2	2	0	0	1	0	0	0.0%	5.4%	4.7%	0.0%	0.0%	4.7%	0.0%	0.0%
tr|F1SN99|F1SN99	1	1	1	0	1	0	0	0	1	1	3	0	1	0	0	0	0.6%	0.4%	0.7%	0.0%	0.6%	0.0%	0.0%	0.0%
tr|F1RTB6|F1RTB6	1	0	1	0	1	0	0	0	2	0	4	0	1	0	0	0	7.4%	0.0%	3.7%	0.0%	5.3%	0.0%	0.0%	0.0%
tr|F1SIR9|F1SIR9	2	0	1	0	1	0	0	0	2	0	5	0	1	0	0	0	3.3%	0.0%	1.8%	0.0%	1.2%	0.0%	0.0%	0.0%
tr|F1SJ68|F1SJ68	1	1	1	0	1	0	0	0	1	1	5	0	1	0	0	0	3.1%	0.9%	1.8%	0.0%	0.9%	0.0%	0.0%	0.0%
tr|F1RWZ4|F1RWZ4	1	1	2	0	1	0	0	0	1	1	6	0	1	0	0	0	4.8%	4.8%	7.6%	0.0%	4.1%	0.0%	0.0%	0.0%
tr|F1SSB4|F1SSB4	2	0	2	0	1	0	0	0	2	0	11	0	1	0	0	0	36.5%	0.0%	20.9%	0.0%	23.5%	0.0%	0.0%	0.0%
tr|F1SIF2|F1SIF2	9	4	5	1	3	1	0	1	16	9	8	6	5	1	0	1	28.0%	9.5%	15.5%	4.2%	10.6%	4.2%	0.0%	5.7%
tr|F1S9K3|F1S9K3	2	3	3	2	1	1	1	1	3	18	20	6	3	1	2	1	9.4%	11.4%	14.3%	7.5%	3.9%	3.9%	3.9%	3.9%
tr|F1SM28|F1SM28	4	4	0	1	2	1	0	0	9	8	0	3	4	1	0	0	11.9%	17.7%	0.0%	5.5%	5.5%	5.5%	0.0%	0.0%
tr|C7AI81|C7AI81	19	21	13	5	14	7	5	9	90	169	244	46	57	18	131	255	33.7%	35.8%	21.8%	12.7%	29.4%	15.4%	12.2%	28.4%
tr|I3LS62|I3LS62	0	3	0	0	0	2	0	0	0	26	0	0	0	12	0	0	0.0%	20.7%	0.0%	0.0%	0.0%	13.2%	0.0%	0.0%
tr|F1RM08|F1RM08	5	2	3	2	4	0	5	14	11	2	48	2	4	0	21	198	11.3%	5.1%	9.3%	6.8%	7.7%	0.0%	11.7%	29.2%
tr|F2Z501|F2Z501	3	2	1	1	1	1	2	3	13	46	6	14	11	2	64	14	24.9%	12.4%	4.5%	6.5%	6.5%	6.5%	12.4%	20.4%
Q35915	1	2	0	2	1	1	1	0	8	14	0	3	2	5	2	0	6.2%	6.2%	0.0%	6.2%	6.2%	6.2%	6.2%	0.0%
tr|F1RP54|F1RP54	1	2	0	0	2	1	0	1	1	10	0	0	3	2	0	7	1.0%	1.0%	0.0%	0.0%	1.0%	1.0%	0.0%	1.0%
tr|B6VNT8|B6VNT8	22	23	13	5	15	7	5	9	100	174	244	46	58	18	131	255	36.6%	38.7%	21.8%	12.7%	32.4%	15.4%	12.2%	28.4%
tr|I3LFI3|I3LFI3	3	1	2	0	2	2	3	3	7	2	8	0	2	2	29	19	12.0%	4.5%	10.6%	0.0%	10.4%	12.0%	12.3%	12.3%
tr|F1SH45|F1SH45	0	2	0	1	1	0	0	0	0	9	0	2	2	0	0	0	0.0%	2.5%	0.0%	2.5%	2.5%	0.0%	0.0%	0.0%
tr|B7NZV3|B7NZV3	3	1	1	0	2	1	0	0	8	1	1	0	3	1	0	0	31.4%	10.7%	5.0%	0.0%	10.7%	10.7%	0.0%	0.0%
P28491	33	18	40	1	6	9	7	21	212	981	426	278	173	78	276	323	70.7%	63.3%	59.5%	2.6%	23.7%	15.1%	26.6%	59.0%
P68137	22	23	13	5	15	6	5	9	100	174	244	46	58	17	131	255	36.6%	38.7%	21.8%	12.7%	32.4%	10.9%	12.2%	28.4%
tr|I3LDA8|I3LDA8	32	18	22	4	10	3	17	24	106	51	169	21	44	4	136	140	31.9%	19.0%	19.1%	3.5%	11.9%	2.7%	21.0%	32.3%
tr|I3LMI4|I3LMI4	3	5	1	2	2	1	0	1	17	24	1	6	10	2	0	3	35.0%	23.1%	7.7%	12.6%	7.7%	7.7%	0.0%	19.6%
tr|Q52NJ5|Q52NJ5	5	6	1	1	4	0	1	2	17	22	4	8	9	0	11	9	23.2%	27.6%	12.2%	5.0%	35.9%	0.0%	8.8%	18.2%
tr|F1RR89|F1RR89	8	3	3	1	1	0	0	1	14	14	13	7	5	0	0	2	29.9%	8.1%	10.1%	4.2%	4.2%	0.0%	0.0%	3.6%
tr|A5A780|A5A780	3	2	0	0	1	1	1	1	3	4	0	0	1	2	1	2	8.7%	3.0%	0.0%	0.0%	5.0%	3.0%	5.0%	5.0%
tr|F1RR05|F1RR05	0	1	0	1	1	0	0	0	0	7	0	2	1	0	0	0	0.0%	6.1%	0.0%	6.1%	9.5%	0.0%	0.0%	0.0%
tr|F1SFV7|F1SFV7	17	13	9	2	11	4	2	5	47	80	267	11	37	6	46	22	83.2%	50.5%	59.4%	19.8%	77.2%	27.7%	19.8%	58.4%
tr|I3LGD4|I3LGD4	43	26	18	8	18	9	13	27	151	179	126	50	71	18	121	163	58.0%	35.0%	17.8%	13.4%	29.8%	14.4%	27.7%	47.7%
Q95276	6	7	3	2	3	3	3	3	27	35	213	9	10	7	57	22	39.2%	39.2%	25.6%	14.4%	22.4%	25.6%	28.0%	28.0%
tr|F2Z5Q2|F2Z5Q2	5	3	1	2	2	1	0	0	16	15	3	6	6	1	0	0	23.6%	19.1%	5.1%	10.8%	10.8%	5.1%	0.0%	0.0%
tr|F1SFV5|F1SFV5	12	6	10	0	8	1	1	5	34	14	91	0	19	1	4	64	58.2%	57.1%	17.3%	0.0%	39.8%	22.4%	16.3%	26.5%
tr|K7GQ95|K7GQ95	12	6	10	0	8	1	1	5	34	14	91	0	19	1	4	64	58.8%	57.7%	17.5%	0.0%	40.2%	22.7%	16.5%	26.8%
tr|K7GLV7|K7GLV7	12	6	10	0	8	1	1	5	34	14	91	0	19	1	4	64	46.0%	45.2%	13.7%	0.0%	31.5%	17.7%	12.9%	21.0%
tr|K7GSS3|K7GSS3	2	3	8	1	1	0	0	4	3	9	37	3	2	0	0	10	1.1%	1.9%	5.3%	0.5%	0.5%	0.0%	0.0%	2.9%
tr|K7GLA8|K7GLA8	2	3	8	1	1	0	0	4	3	9	37	3	2	0	0	10	1.6%	2.6%	7.5%	0.7%	0.7%	0.0%	0.0%	4.0%
tr|F1RG02|F1RG02	2	3	8	1	1	0	0	4	3	9	37	3	2	0	0	10	0.7%	1.1%	3.2%	0.3%	0.3%	0.0%	0.0%	1.7%
tr|K7GNQ7|K7GNQ7	2	3	8	1	1	0	0	4	3	9	37	3	2	0	0	10	0.7%	1.1%	3.2%	0.3%	0.3%	0.0%	0.0%	1.7%
tr|F1SNW8|F1SNW8	6	4	5	1	2	1	0	0	6	6	12	2	2	1	0	0	11.5%	6.2%	6.3%	1.3%	3.6%	1.3%	0.0%	0.0%
tr|F1RGF9|F1RGF9	10	4	13	3	6	0	13	18	29	12	113	5	12	0	828	487	13.3%	5.5%	14.3%	3.5%	6.0%	0.0%	13.1%	15.2%
tr|F1RGJ2|F1RGJ2	31	6	8	7	16	4	21	39	100	16	51	16	22	10	183	420	43.0%	12.4%	13.1%	10.4%	27.2%	5.3%	32.9%	47.4%
tr|F1RHJ9|F1RHJ9	8	2	5	1	1	0	0	3	11	6	24	3	4	0	0	9	22.2%	7.7%	14.9%	2.4%	2.4%	0.0%	0.0%	11.1%
tr|F1RZA6|F1RZA6	11	3	6	0	4	0	1	0	14	3	15	0	7	0	1	0	4.7%	1.4%	2.9%	0.0%	1.9%	0.0%	0.6%	0.0%
tr|F1RRS4|F1RRS4	5	7	1	3	7	0	4	4	32	24	12	5	18	0	43	11	46.6%	52.4%	10.7%	44.7%	52.4%	0.0%	44.7%	22.3%
tr|F1ST43|F1ST43	2	3	3	1	1	2	0	0	7	20	7	2	3	6	0	0	5.7%	7.1%	10.1%	3.2%	3.2%	4.8%	0.0%	0.0%
tr|F1RZU8|F1RZU8	17	5	10	3	3	2	1	7	24	35	24	11	11	2	1	13	4.2%	0.8%	2.0%	0.4%	0.4%	0.3%	0.2%	1.4%
tr|F1S0V3|F1S0V3	41	14	26	4	8	6	18	27	175	113	230	55	43	19	383	337	66.4%	26.8%	42.5%	11.3%	20.8%	12.3%	45.0%	51.0%
D2WKD8	12	3	12	3	6	0	14	17	31	11	91	5	12	0	578	400	15.7%	3.9%	12.6%	3.4%	6.0%	0.0%	13.9%	16.0%
tr|F2Z5M4|F2Z5M4	6	2	4	0	2	2	4	8	24	78	24	0	7	34	36	128	45.6%	21.4%	27.2%	0.0%	21.4%	21.4%	44.7%	49.5%
tr|K7GQD4|K7GQD4	6	2	4	0	2	2	4	8	24	78	24	0	7	34	36	128	48.5%	22.7%	28.9%	0.0%	22.7%	22.7%	47.4%	52.6%
tr|I3LS96|I3LS96	3	5	2	2	2	2	1	2	24	36	205	9	9	6	17	18	10.8%	10.8%	10.5%	6.3%	6.3%	6.3%	6.3%	10.5%
tr|F1S8J6|F1S8J6	7	7	2	2	3	4	1	4	17	23	15	2	9	5	9	24	30.6%	25.0%	10.6%	13.4%	13.4%	19.0%	5.1%	18.1%
tr|K7GQZ0|K7GQZ0	0	1	0	1	2	0	0	0	0	10	0	1	3	0	0	0	0.0%	0.7%	0.0%	0.7%	2.3%	0.0%	0.0%	0.0%
tr|F1SG21|F1SG21	0	1	0	1	2	0	0	0	0	10	0	1	3	0	0	0	0.0%	0.7%	0.0%	0.7%	2.4%	0.0%	0.0%	0.0%
tr|F1SMX7|F1SMX7	1	2	0	1	1	0	0	0	1	9	0	2	2	0	0	0	2.9%	2.4%	0.0%	2.4%	2.4%	0.0%	0.0%	0.0%

	Peptide Count								Spectral Count								Sequence Coverage							
	Whole Cell	Whole Cell	Whole Cell	Cilia Fraction	Cilia Fraction	Cilia Fraction	Cilia Fraction	Cilia Fraction	Whole Cell	Whole Cell	Whole Cell	Cilia Fraction	Cilia Fraction	Cilia Fraction	Cilia Fraction	Cilia Fraction	Whole Cell	Whole Cell	Whole Cell	Cilia Fraction	Cilia Fraction	Cilia Fraction	Cilia Fraction	Cilia Fraction
Sample#	1	2	6	4	3	5	7	8	1	2	6	4	3	5	7	8	1	2	6	4	3	5	7	8
Accession																								
tr|F1RP39|F1RP39	3	3	1	1	2	0	0	0	5	5	4	1	3	0	0	0	3.1%	1.9%	0.5%	0.5%	1.2%	0.0%	0.0%	0.0%
tr|K7GNZ0|K7GNZ0	2	0	0	1	1	0	1	2	5	0	0	1	1	0	1	12	9.9%	0.0%	0.0%	6.2%	6.2%	0.0%	13.6%	9.9%
tr|I3LFQ9|I3LFQ9	1	1	1	1	1	0	1	2	1	4	2	1	1	0	1	4	5.0%	5.4%	4.1%	5.4%	5.4%	0.0%	5.4%	7.2%
tr|F1SVC7|F1SVC7	1	1	1	1	1	0	1	0	1	4	2	1	1	0	1	0	5.1%	5.5%	4.1%	5.5%	5.5%	0.0%	5.5%	0.0%
tr|F1RND0|F1RND0	1	1	1	0	1	1	0	0	1	4	1	0	1	1	0	0	3.7%	5.8%	9.9%	0.0%	5.8%	5.8%	0.0%	0.0%
tr|I3LG94|I3LG94	3	2	1	1	0	0	0	0	3	2	1	2	0	0	0	0	4.9%	1.8%	1.4%	0.9%	0.0%	0.0%	0.0%	0.0%
tr|I3LHI4|I3LHI4	3	0	1	0	1	0	0	0	5	0	1	0	2	0	0	0	6.6%	0.0%	1.2%	0.0%	1.6%	0.0%	0.0%	0.0%
tr|I3LRH3|I3LRH3	1	1	1	0	1	1	0	0	1	4	1	0	1	1	0	0	3.8%	5.8%	10.0%	0.0%	5.8%	5.8%	0.0%	0.0%
tr|F1RFP8|F1RFP8	3	0	1	1	1	0	0	0	5	0	3	1	1	0	0	0	6.5%	0.0%	2.2%	2.2%	4.8%	0.0%	0.0%	0.0%
tr|F1RGE2|F1RGE2	2	2	8	1	1	0	0	0	3	2	17	1	1	0	0	0	2.6%	3.0%	5.7%	1.0%	1.0%	0.0%	0.0%	0.0%
tr|I3LJY8|I3LJY8	7	10	2	2	3	3	1	0	13	30	9	3	6	8	4	0	47.9%	47.5%	12.6%	10.5%	15.5%	11.3%	10.5%	0.0%
tr|I3LE12|I3LE12	7	10	2	2	3	3	1	0	13	30	9	3	6	8	4	0	47.7%	47.3%	12.6%	10.5%	15.5%	11.3%	10.5%	0.0%
tr|F1RPL2|F1RPL2	3	0	1	0	2	0	0	1	18	0	10	0	7	0	0	8	31.6%	0.0%	8.1%	0.0%	23.5%	0.0%	0.0%	23.5%
tr|F1S182|F1S182	25	9	13	2	7	2	3	8	68	74	44	17	32	6	20	22	40.4%	11.8%	17.3%	1.3%	13.3%	4.8%	5.5%	19.5%
tr|F1RFY1|F1RFY1	13	10	7	5	4	1	6	6	150	892	472	196	176	30	282	37	67.9%	69.3%	52.1%	47.9%	42.1%	12.1%	48.6%	42.9%
tr|F1SPG9|F1SPG9	20	11	7	3	7	2	4	6	44	26	32	8	16	3	15	22	27.6%	13.4%	7.7%	2.6%	10.0%	3.9%	6.7%	9.1%
tr|I3L9V2|I3L9V2	9	9	4	2	5	3	6	7	13	52	27	6	13	6	56	38	37.0%	26.5%	14.2%	7.2%	17.5%	11.2%	24.5%	27.0%
tr|F1SBL1|F1SBL1	6	0	6	2	2	0	5	17	13	0	20	3	2	0	28	45	9.3%	0.0%	7.3%	1.9%	3.5%	0.0%	10.6%	24.1%
Q1W674	6	4	5	1	2	1	0	0	7	6	12	2	2	1	0	0	8.2%	5.8%	5.9%	1.2%	3.4%	1.2%	0.0%	0.0%
tr|F1S9Y5|F1S9Y5	5	0	2	1	1	0	0	0	13	0	12	1	4	0	0	0	15.1%	0.0%	3.2%	3.0%	3.0%	0.0%	0.0%	0.0%
tr|I3LM80|I3LM80	8	7	1	0	4	4	1	1	23	24	3	0	6	12	5	7	20.3%	15.7%	2.9%	0.0%	9.7%	7.9%	3.7%	3.7%
tr|I3L7Z6|I3L7Z6	4	6	3	1	3	3	1	2	23	61	126	7	20	5	30	4	26.7%	26.7%	12.2%	12.2%	16.7%	23.3%	12.2%	22.2%
tr|K7GQK6|K7GQK6	2	1	3	0	2	2	0	3	2	19	7	0	3	5	0	7	26.4%	3.8%	14.5%	0.0%	20.4%	3.8%	0.0%	12.8%
P67872	2	1	3	0	2	2	0	3	2	19	7	0	3	5	0	7	28.8%	4.2%	15.8%	0.0%	22.3%	4.2%	0.0%	14.0%
tr|I3L6Y6|I3L6Y6	30	11	14	3	10	2	5	10	80	83	34	19	37	6	22	32	42.8%	14.4%	16.7%	2.4%	15.0%	4.2%	8.5%	20.8%
tr|F2Z5D4|F2Z5D4	21	7	11	1	1	1	1	4	43	29	35	15	10	2	3	10	37.0%	16.9%	22.2%	1.6%	1.6%	1.6%	2.1%	15.2%
tr|F1S2E2|F1S2E2	7	2	11	1	4	0	8	11	14	2	214	1	5	0	81	213	16.7%	5.2%	21.7%	2.0%	8.4%	0.0%	17.5%	17.1%
tr|K7GLW0|K7GLW0	1	3	1	0	1	0	0	0	1	7	1	0	3	0	0	0	3.9%	8.1%	2.6%	0.0%	5.1%	0.0%	0.0%	0.0%
tr|F1S7Z8|F1S7Z8	3	2	2	0	1	0	0	1	4	4	10	0	3	0	0	1	6.1%	4.5%	5.2%	0.0%	3.2%	0.0%	0.0%	3.9%
tr|I3LEF8|I3LEF8	8	8	7	4	4	1	2	1	10	25	114	8	4	1	7	2	28.6%	24.8%	18.4%	12.2%	16.7%	3.6%	8.8%	3.6%
tr|F1S0V2|F1S0V2	8	3	3	1	3	0	1	5	37	17	26	11	9	0	5	66	57.1%	27.6%	21.0%	10.5%	29.5%	0.0%	10.5%	45.7%
tr|I3L9F7|I3L9F7	6	3	0	1	1	2	2	0	14	13	0	3	4	3	10	0	21.4%	8.5%	0.0%	3.1%	3.1%	3.1%	7.0%	0.0%
tr|F1SCY0|F1SCY0	6	3	4	1	3	1	1	1	26	12	24	5	8	1	1	1	15.6%	9.8%	7.7%	2.4%	5.6%	2.4%	2.4%	4.3%
tr|F1S6G2|F1S6G2	7	2	2	1	1	0	0	0	12	7	4	3	4	0	0	0	9.1%	2.6%	2.8%	1.4%	1.4%	0.0%	0.0%	0.0%
tr|B5APV0|B5APV0	5	2	3	2	1	0	0	0	12	7	9	4	3	0	0	0	33.8%	16.6%	30.5%	16.6%	7.9%	0.0%	0.0%	0.0%
tr|F1SKG6|F1SKG6	3	1	2	0	1	1	2	10	3	8	13	0	3	1	114	86	32.9%	4.5%	13.2%	0.0%	4.5%	3.7%	8.6%	46.1%
tr|F1SU12|F1SU12	10	6	3	4	3	0	2	3	25	22	19	11	6	0	2	6	37.8%	17.8%	6.8%	10.9%	8.4%	0.0%	5.7%	15.9%
Q99028	10	8	1	1	2	2	4	3	19	20	4	3	8	3	26	5	60.8%	49.5%	8.1%	14.0%	14.0%	7.0%	33.3%	19.9%
tr|F1S3E0|F1S3E0	4	1	2	2	1	1	2	5	6	22	12	5	3	2	8	18	20.6%	4.0%	10.5%	4.0%	4.0%	4.0%	12.9%	24.2%
tr|F1RTU5|F1RTU5	5	3	0	1	2	2	1	0	8	23	0	3	5	3	9	0	14.5%	6.5%	0.0%	2.6%	6.5%	2.6%	2.6%	0.0%
tr|F1RNU9|F1RNU9	22	15	15	4	8	4	10	12	161	90	90	39	43	7	60	146	43.5%	26.2%	26.2%	8.6%	14.5%	5.6%	20.1%	29.7%
P08059	23	15	15	4	8	4	10	12	162	90	90	39	43	7	60	146	48.9%	26.2%	26.2%	8.6%	14.5%	5.6%	20.1%	29.7%
tr|F1RP05|F1RP05	6	4	3	1	2	2	2	9	28	23	6	3	7	8	8	18	24.8%	13.6%	11.4%	3.6%	10.9%	10.9%	6.1%	34.5%
tr|F1RZH4|F1RZH4	2	0	0	1	0	0	0	1	17	0	0	6	0	0	0	3	11.3%	0.0%	0.0%	4.8%	0.0%	0.0%	0.0%	11.3%
tr|I3LCB2|I3LCB2	0	1	0	1	1	0	1	0	0	17	0	2	4	0	1	0	0.0%	8.7%	0.0%	8.7%	8.7%	0.0%	18.0%	0.0%
tr|I3LJY5|I3LJY5	4	2	0	1	1	1	0	0	6	14	0	2	4	1	0	0	13.8%	4.7%	0.0%	4.7%	4.7%	4.7%	0.0%	0.0%
tr|K7GLU6|K7GLU6	5	4	2	2	2	0	0	1	9	11	25	3	4	0	0	2	9.2%	5.1%	3.5%	2.9%	2.9%	0.0%	0.0%	1.3%
tr|F1RNU1|F1RNU1	5	4	2	2	2	0	0	1	9	11	25	3	4	0	0	2	8.3%	4.6%	3.2%	2.6%	2.6%	0.0%	0.0%	1.2%
tr|F1RJ33|F1RJ33	7	5	5	3	6	3	2	4	15	91	27	13	15	9	40	34	25.4%	15.5%	15.8%	9.3%	18.9%	9.3%	9.0%	16.1%
P81693	2	2	2	1	1	1	1	0	7	16	3	2	5	1	3	0	21.5%	15.8%	13.9%	7.6%	7.6%	7.6%	7.6%	0.0%
tr|F1SPG2|F1SPG2	26	14	16	3	10	4	6	10	89	61	142	13	30	9	47	61	43.8%	30.9%	24.5%	10.7%	18.9%	11.2%	18.6%	26.3%
Q9GMB0	26	14	16	3	10	4	6	10	89	61	142	13	30	9	47	61	43.8%	30.9%	24.5%	10.7%	18.9%	11.2%	18.6%	26.3%
tr|F1S0J8|F1S0J8	30	23	27	3	15	5	7	12	190	141	219	26	75	12	170	100	61.9%	65.1%	51.0%	6.2%	42.6%	10.9%	22.5%	33.9%
tr|F1SGM3|F1SGM3	12	3	9	2	3	2	3	5	67	46	62	13	19	6	39	24	52.8%	11.6%	34.0%	6.4%	15.2%	6.4%	19.6%	30.0%
Q863Z0	12	3	9	2	3	2	3	5	67	46	62	13	19	6	39	24	55.2%	12.1%	35.6%	6.7%	15.9%	6.7%	20.5%	31.4%
tr|K7GRW3|K7GRW3	13	12	7	1	7	4	2	4	35	78	262	7	25	6	46	21	68.9%	68.9%	59.0%	18.0%	59.0%	45.9%	32.8%	62.3%
tr|F1S8P1|F1S8P1	6	2	2	2	1	0	0	2	16	5	4	5	2	0	0	7	25.9%	5.8%	7.2%	12.6%	5.8%	0.0%	0.0%	6.8%
tr|I3LB08|I3LB08	2	1	1	1	1	0	1	0	2	19	1	5	2	0	4	0	18.9%	8.4%	11.9%	8.4%	8.4%	0.0%	8.4%	0.0%
tr|F1SS24|F1SS24	11	7	0	1	4	1	1	2	13	8	0	1	4	2	1	2	6.4%	2.8%	0.0%	0.6%	3.1%	0.5%	0.9%	1.1%
tr|F1RX99|F1RX99	7	3	8	1	1	0	2	5	11	10	105	6	1	0	3	6	40.8%	20.1%	67.4%	7.6%	7.6%	0.0%	27.7%	50.5%
tr|K7GQ59|K7GQ59	6	2	9	1	3	0	5	9	13	2	179	1	4	0	60	173	17.0%	5.9%	21.1%	2.3%	7.5%	0.0%	12.9%	15.9%
tr|Q2QC87|Q2QC87	9	8	2	3	5	1	2	5	16	50	19	9	12	1	4	19	22.4%	17.0%	4.6%	5.5%	11.8%	1.9%	4.6%	12.1%
tr|I3LR15|I3LR15	14	3	5	3	4	1	9	11	39	15	16	8	8	2	184	204	44.4%	12.4%	15.2%	12.4%	18.7%	3.2%	33.0%	33.7%
tr|I3LMT8|I3LMT8	7	2	2	1	1	0	0	0	25	2	16	5	4	0	0	0	35.3%	16.8%	12.2%	4.2%	4.2%	0.0%	0.0%	0.0%
tr|I3L8L8|I3L8L8	4	1	5	1	4	0	6	8	13	5	36	1	5	0	508	350	6.9%	1.0%	4.7%	0.9%	3.1%	0.0%	6.0%	7.0%
tr|I3LL84|I3LL84	2	4	0	0	2	0	1	2	5	13	0	0	6	0	4	6	14.9%	22.1%	0.0%	0.0%	8.9%	0.0%	9.8%	19.8%
tr|F1S8Y8|F1S8Y8	8	2	2	0	2	1	0	0	13	5	3	0	4	2	0	0	10.4%	1.4%	3.7%	0.0%	3.2%	1.4%	0.0%	0.0%
tr|F1SU03|F1SU03	7	3	3	0	1	3	0	3	9	3	7	0	1	3	0	4	8.4%	1.7%	2.4%	0.0%	0.9%	1.7%	0.0%	3.3%
tr|I3LKI5|I3LKI5	1	1	0	1	1	1	0	0	1	11	0	1	2	1	0	0	2.4%	2.4%	0.0%	2.4%	2.4%	2.4%	0.0%	0.0%
tr|F1S8H8|F1S8H8	2	2	3	1	0	1	0	0	5	7	12	3	0	1	0	0	10.4%	6.2%	15.6%	3.2%	0.0%	6.2%	0.0%	0.0%
tr|F1SLX0|F1SLX0	3	0	1	0	1	1	0	2	6	0	1	0	1	1	0	9	19.7%	0.0%	5.0%	0.0%	9.7%	3.6%	0.0%	8.6%
tr|K7GM88|K7GM88	4	1	1	0	1	1	1	2	5	1	4	0	1	1	3	3	14.9%	6.8%	11.3%	0.0%	6.8%	8.1%	6.8%	5.9%
tr|F1RSP0|F1RSP0	4	1	1	0	1	1	1	2	5	1	4	0	1	1	3	3	14.5%	6.6%	11.0%	0.0%	6.6%	7.9%	6.6%	5.7%
tr|F1SMY1|F1SMY1	1	1	0	1	0	1	0	0	2	4	0	1	0	1	0	0	3.1%	2.9%	0.0%	2.9%	0.0%	3.3%	0.0%	0.0%
tr|F1SU87|F1SU87	2	2	0	1	1	0	0	0	4	2	0	1	1	0	0	0	6.7%	6.7%	0.0%	3.1%	3.1%	0.0%	0.0%	0.0%
tr|I3LI89|I3LI89	1	1	0	1	1	0	0	0	1	5	0	1	1	0	0	0	8.3%	3.6%	0.0%	3.6%	3.6%	0.0%	0.0%	0.0%
tr|I3LMY9|I3LMY9	3	1	1	0	1	1	0	0	5	1	1	0	1	1	0	0	2.4%	0.5%	0.4%	0.0%	0.4%	1.2%	0.0%	0.0%
tr|Q95JH1|Q95JH1	3	0	2	0	1	0	8	16	3	0	5	0	1	0	127	117	7.7%	0.0%	3.6%	0.0%	4.1%	0.0%	17.0%	20.8%
tr|F1S2H8|F1S2H8	2	0	1	0	1	0	1	2	3	0	3	0	1	0	6	12	17.8%	0.0%	3.3%	0.0%	9.5%	0.0%	6.9%	18.4%
tr|I3L889|I3L889	1	1	0	1	0	0	1	0	1	2	0	1	0	0	3	0	10.6%	13.5%	0.0%	13.5%	0.0%	0.0%	10.6%	0.0%
tr|I3LLF3|I3LLF3	3	0	0	1	0	0	0	0	3	0	0	1	0	0	0	0	12.9%	0.0%	0.0%	5.2%	0.0%	0.0%	0.0%	0.0%

	Peptide Count								Spectral Count								Sequence Coverage							
	Whole Cell	Whole Cell	Whole Cell	Cilia Fraction	Cilia Fraction	Cilia Fraction	Cilia Fraction	Cilia Fraction	Whole Cell	Whole Cell	Whole Cell	Cilia Fraction	Cilia Fraction	Cilia Fraction	Cilia Fraction	Cilia Fraction	Whole Cell	Whole Cell	Whole Cell	Cilia Fraction	Cilia Fraction	Cilia Fraction	Cilia Fraction	Cilia Fraction
Sample#	1	2	6	4	3	5	7	8	1	2	6	4	3	5	7	8	1	2	6	4	3	5	7	8
**Accession**																								
tr|F1SD85|F1SD85	1	2	0	0	1	0	0	0	1	2	0	0	1	0	0	0	3.4%	7.3%	0.0%	0.0%	3.4%	0.0%	0.0%	0.0%
tr|F1RQQ6|F1RQQ6	0	3	0	1	0	0	0	0	0	3	0	1	0	0	0	0	0.0%	8.6%	0.0%	1.9%	0.0%	0.0%	0.0%	0.0%
tr|I3LA49|I3LA49	1	2	0	1	0	0	0	0	1	2	0	1	0	0	0	0	3.6%	6.3%	0.0%	3.3%	0.0%	0.0%	0.0%	0.0%
tr|C6L245|C6L245	0	1	0	0	1	0	0	0	0	3	0	0	1	0	0	0	0.0%	3.2%	0.0%	0.0%	6.1%	0.0%	0.0%	0.0%
tr|F1SBL4|F1SBL4	2	1	0	0	1	0	0	0	2	1	0	0	1	0	0	0	4.6%	2.8%	0.0%	0.0%	1.8%	0.0%	0.0%	0.0%
tr|I3L8X1|I3L8X1	3	0	0	1	0	0	0	0	3	0	0	1	0	0	0	0	11.3%	0.0%	0.0%	4.5%	0.0%	0.0%	0.0%	0.0%
tr|F1RX97|F1RX97	3	0	0	0	1	0	0	0	3	0	0	0	1	0	0	0	32.3%	0.0%	0.0%	0.0%	16.2%	0.0%	0.0%	0.0%
tr|F1RIR7|F1RIR7	1	0	0	0	0	1	0	0	3	0	0	0	0	1	0	0	4.1%	0.0%	0.0%	0.0%	0.0%	4.1%	0.0%	0.0%
tr|F1RKE9|F1RKE9	0	1	0	0	1	0	0	0	0	3	0	0	1	0	0	0	0.0%	2.6%	0.0%	0.0%	4.6%	0.0%	0.0%	0.0%
tr|I3L5J2|I3L5J2	0	1	0	0	1	0	0	0	0	3	0	0	1	0	0	0	0.0%	2.2%	0.0%	0.0%	2.2%	0.0%	0.0%	0.0%
tr|F1S010|F1S010	0	1	0	0	1	0	0	0	0	3	0	0	1	0	0	0	0.0%	5.3%	0.0%	0.0%	5.3%	0.0%	0.0%	0.0%
tr|F1SD35|F1SD35	1	2	0	0	0	1	0	0	1	2	0	0	0	1	0	0	4.0%	4.0%	0.0%	0.0%	0.0%	2.6%	0.0%	0.0%
tr|I3LEE6|I3LEE6	0	1	0	1	0	0	0	0	0	3	0	1	0	0	0	0	0.0%	1.9%	0.0%	1.9%	0.0%	0.0%	0.0%	0.0%
tr|F1RSE6|F1RSE6	0	1	1	0	1	0	0	0	0	3	1	0	1	0	0	0	0.0%	0.3%	0.4%	0.0%	0.4%	0.0%	0.0%	0.0%
tr|F1RJN4|F1RJN4	2	1	1	0	1	0	0	0	2	1	1	0	1	0	0	0	8.4%	2.9%	2.7%	0.0%	5.4%	0.0%	0.0%	0.0%
tr|F1RSX1|F1RSX1	2	0	1	0	1	0	0	0	3	0	2	0	1	0	0	0	5.8%	0.0%	5.8%	0.0%	5.8%	0.0%	0.0%	0.0%
tr|F1S0R3|F1S0R3	3	0	1	0	1	0	0	0	3	0	3	0	1	0	0	0	16.0%	0.0%	5.4%	0.0%	3.7%	0.0%	0.0%	0.0%
tr|F1SAS3|F1SAS3	2	1	1	0	0	1	0	0	2	1	7	0	0	1	0	0	14.0%	3.0%	4.5%	0.0%	0.0%	3.0%	0.0%	0.0%
tr|F1RSR8|F1RSR8	3	0	2	0	1	0	0	0	3	0	9	0	1	0	0	0	11.2%	0.0%	3.3%	0.0%	2.9%	0.0%	0.0%	0.0%
tr|I3L9S5|I3L9S5	1	1	1	0	1	0	0	0	1	2	9	0	1	0	0	0	20.5%	7.4%	20.5%	0.0%	7.4%	0.0%	0.0%	0.0%
tr|I3LV02|I3LV02	20	10	10	4	5	2	2	4	58	112	91	20	30	6	18	14	28.8%	11.3%	13.7%	3.7%	8.9%	4.1%	2.5%	8.9%
tr|F1RFI2|F1RFI2	20	10	10	4	5	2	2	4	58	112	91	20	30	6	18	14	28.8%	11.3%	13.7%	3.7%	8.9%	4.1%	2.5%	8.9%
tr|F1SV93|F1SV93	7	4	0	1	3	4	2	1	16	33	0	3	6	7	13	2	17.1%	10.8%	0.0%	2.9%	12.7%	6.5%	6.5%	3.7%
Q29214	22	19	8	5	6	5	3	4	88	112	132	27	28	10	20	13	69.2%	62.6%	31.1%	24.2%	32.4%	21.7%	20.8%	28.3%
P19620	50	33	34	14	29	8	13	29	535	139	356	47	127	44	706	486	75.2%	67.3%	57.2%	44.0%	65.8%	23.9%	46.0%	65.5%
tr|F1S073|F1S073	50	33	34	14	29	8	13	29	535	139	356	47	127	44	706	486	75.2%	67.3%	57.2%	44.0%	65.8%	23.9%	46.0%	65.5%
tr|I3LAW4|I3LAW4	3	3	0	1	3	1	1	3	14	17	0	4	5	1	4	11	38.6%	27.6%	0.0%	12.6%	27.6%	12.6%	12.6%	38.6%
tr|D2JYW4|D2JYW4	11	5	11	2	3	1	3	8	30	57	69	17	9	2	33	80	46.6%	20.2%	38.6%	9.7%	13.4%	4.7%	13.7%	31.4%
A0PFK7	11	5	11	2	3	1	3	8	30	57	69	17	9	2	33	80	46.6%	20.2%	38.6%	9.7%	13.4%	4.7%	13.7%	31.4%
tr|A9XFX6|A9XFX6	12	5	12	2	3	1	3	10	31	57	71	17	9	2	33	82	52.9%	20.6%	44.5%	9.9%	13.6%	4.8%	14.0%	37.5%
tr|F2Z576|F2Z576	4	1	1	0	2	0	0	1	19	3	10	0	7	0	0	8	38.2%	6.6%	8.1%	0.0%	23.5%	0.0%	0.0%	23.5%
tr|F1RZ83|F1RZ83	5	4	3	1	3	0	1	0	8	14	9	1	6	0	1	0	13.1%	11.4%	7.8%	4.6%	12.7%	0.0%	4.6%	0.0%
tr|F1SN33|F1SN33	5	4	0	1	2	1	1	5	9	32	0	3	5	5	23	15	6.0%	6.0%	0.0%	1.2%	2.8%	1.5%	3.1%	7.9%
tr|F2Z587|F2Z587	3	2	0	0	2	1	1	1	16	3	0	0	4	2	12	1	14.6%	20.0%	0.0%	0.0%	21.5%	14.6%	14.6%	14.6%
tr|F1SHR2|F1SHR2	8	1	2	1	1	0	0	1	12	7	18	3	3	0	0	6	26.1%	3.0%	7.8%	3.0%	3.0%	0.0%	0.0%	5.8%
tr|I3LCA2|I3LCA2	25	13	15	2	5	3	3	8	70	73	135	16	20	9	4	36	45.6%	29.9%	28.6%	6.0%	15.5%	7.3%	10.9%	21.7%
tr|F1RHN3|F1RHN3	13	9	2	1	3	2	4	5	25	23	8	3	9	3	26	16	55.2%	44.4%	8.5%	9.6%	20.0%	4.8%	23.0%	24.1%
tr|F2Z5F2|F2Z5F2	4	3	1	0	3	0	1	5	7	9	2	0	5	0	2	14	23.5%	22.5%	6.0%	0.0%	22.0%	0.0%	5.5%	35.5%
tr|F1RTV5|F1RTV5	11	8	6	2	3	2	2	5	30	28	19	3	8	7	10	23	33.4%	16.6%	15.4%	7.5%	7.5%	4.9%	4.9%	12.6%
Q6QRN9	8	14	3	4	5	9	1	2	28	144	74	17	18	18	10	26	23.2%	43.0%	9.7%	17.4%	20.8%	34.6%	9.1%	12.4%
Q5G6V9	10	2	5	2	2	0	2	3	41	11	49	6	10	0	25	15	54.2%	15.1%	33.7%	15.1%	15.1%	0.0%	20.5%	19.3%
tr|F1RVM6|F1RVM6	1	2	2	0	2	1	1	1	6	7	8	0	3	1	3	1	7.7%	12.9%	15.8%	0.0%	12.9%	5.3%	7.7%	7.7%
tr|I3L7B9|I3L7B9	1	2	2	0	2	1	1	1	6	7	8	0	3	1	3	1	7.6%	12.9%	15.7%	0.0%	12.9%	5.2%	7.6%	7.6%
tr|F1SP93|F1SP93	16	6	15	1	2	3	8	17	35	11	102	6	4	4	52	67	29.0%	17.0%	26.2%	2.6%	5.3%	6.6%	20.9%	33.2%
tr|I3LIX1|I3LIX1	4	6	2	1	3	1	1	2	10	13	11	3	3	1	1	6	17.8%	37.8%	14.8%	5.9%	20.7%	5.9%	8.1%	18.5%
tr|F1RZ28|F1RZ28	10	5	3	2	2	4	2	5	44	36	24	13	5	6	6	34	39.4%	23.6%	14.5%	14.5%	14.5%	24.2%	9.1%	27.3%
Q45FY6	8	2	2	1	1	0	0	1	28	2	16	5	4	0	0	7	52.3%	18.3%	13.3%	4.6%	4.6%	0.0%	0.0%	13.8%
tr|F1RPL1|F1RPL1	4	2	2	0	2	1	1	1	17	3	10	0	4	2	12	1	23.1%	20.0%	8.5%	0.0%	21.5%	14.6%	14.6%	14.6%
tr|F2Z575|F2Z575	4	2	2	0	2	1	1	1	17	3	10	0	4	2	12	1	23.1%	20.0%	8.5%	0.0%	21.5%	14.6%	14.6%	14.6%
tr|F1RPL3|F1RPL3	4	2	2	0	2	1	1	1	17	3	10	0	4	2	12	1	22.7%	19.7%	8.3%	0.0%	21.2%	14.4%	14.4%	14.4%
tr|F2Z5L5|F2Z5L5	4	2	2	0	2	1	1	1	17	3	10	0	4	2	12	1	23.3%	20.2%	8.5%	0.0%	21.7%	14.7%	14.7%	14.7%
tr|F1RPK0|F1RPK0	4	2	2	0	2	1	1	1	17	3	10	0	4	2	12	1	23.1%	20.0%	8.5%	0.0%	21.5%	14.6%	14.6%	14.6%
tr|F2Z5L2|F2Z5L2	4	2	2	0	2	1	1	1	17	3	10	0	4	2	12	1	23.1%	20.0%	8.5%	0.0%	21.5%	14.6%	14.6%	14.6%
tr|J9JIL1|J9JIL1	3	1	0	1	1	0	2	5	8	2	0	1	2	0	13	9	11.0%	9.9%	0.0%	9.9%	9.9%	0.0%	19.9%	20.9%
tr|F1SK55|F1SK55	2	2	0	0	0	2	1	5	3	7	0	0	0	3	4	18	10.1%	3.6%	0.0%	0.0%	0.0%	3.6%	6.6%	26.8%
tr|I3L5Y4|I3L5Y4	2	2	0	1	1	1	0	0	2	8	0	1	1	1	0	0	4.3%	1.3%	0.0%	2.1%	1.1%	1.1%	0.0%	0.0%
tr|K7GRB9|K7GRB9	2	3	1	0	1	0	0	0	3	7	1	0	3	0	0	0	7.5%	6.8%	2.1%	0.0%	4.3%	0.0%	0.0%	0.0%
tr|F1RX17|F1RX17	5	2	1	0	2	0	0	0	7	3	1	0	3	0	0	0	4.7%	1.4%	0.6%	0.0%	1.4%	0.0%	0.0%	0.0%
tr|F1SPV0|F1SPV0	2	3	1	0	1	0	0	0	3	7	1	0	3	0	0	0	8.0%	7.3%	2.3%	0.0%	4.6%	0.0%	0.0%	0.0%
tr|K7GME3|K7GME3	4	2	2	1	1	0	0	0	5	5	5	1	2	0	0	0	19.5%	9.3%	16.7%	4.7%	4.7%	0.0%	0.0%	0.0%
tr|F1RMJ4|F1RMJ4	31	14	14	4	8	4	7	14	76	55	136	16	18	5	46	54	39.5%	18.6%	18.0%	4.2%	9.6%	7.1%	16.0%	25.7%
tr|I3LDJ6|I3LDJ6	8	6	2	3	3	5	1	1	27	84	71	12	9	12	10	1	37.1%	33.5%	10.8%	20.1%	20.1%	30.9%	13.9%	13.9%
P26044	27	15	12	9	13	3	10	22	73	141	110	25	29	9	229	226	42.5%	24.2%	24.7%	20.9%	23.5%	4.5%	21.8%	36.4%
tr|F1RIF3|F1RIF3	8	3	3	1	2	0	1	4	13	4	14	2	3	0	19	16	23.4%	11.0%	7.9%	3.1%	6.9%	0.0%	3.8%	14.1%
tr|F1SBN7|F1SBN7	9	6	3	0	1	3	4	3	29	29	23	0	4	13	18	17	26.2%	19.1%	12.9%	0.0%	5.9%	11.3%	18.4%	15.6%
Q2XSV9	7	5	2	1	7	2	6	10	25	47	2	3	10	8	41	74	21.2%	13.9%	11.7%	5.3%	27.0%	4.2%	22.3%	34.0%
tr|I3L6U9|I3L6U9	7	5	2	1	7	2	6	10	25	47	2	3	10	8	41	74	21.2%	13.9%	11.7%	5.3%	27.0%	4.2%	22.3%	34.0%
tr|I3LJ20|I3LJ20	4	3	0	2	2	0	2	3	10	14	0	2	5	0	7	9	32.8%	25.3%	0.0%	12.6%	12.6%	0.0%	19.7%	13.1%
tr|F1RRV6|F1RRV6	10	10	3	2	5	5	2	8	68	56	53	17	11	8	57	80	60.5%	43.3%	13.5%	4.7%	24.9%	15.2%	13.7%	35.7%
tr|F1SU44|F1SU44	12	5	0	2	4	0	0	1	15	16	0	2	7	0	0	1	23.7%	5.2%	0.0%	3.3%	5.2%	0.0%	0.0%	2.3%
tr|I3LLE3|I3LLE3	7	4	5	1	1	2	0	0	17	21	16	2	3	6	0	0	13.3%	6.8%	10.3%	2.4%	2.4%	3.6%	0.0%	0.0%
tr|F1SV14|F1SV14	29	16	13	9	13	3	10	22	76	142	113	25	29	9	229	226	44.8%	26.4%	26.9%	20.9%	23.5%	4.5%	21.8%	36.4%
tr|I3LFI0|I3LFI0	5	5	2	2	4	2	0	3	8	37	10	3	4	6	0	9	13.9%	36.7%	10.8%	14.5%	15.1%	9.0%	0.0%	13.9%
tr|F2Z512|F2Z512	5	7	1	2	2	2	0	1	19	51	1	6	10	4	0	3	42.7%	31.5%	7.7%	12.6%	7.7%	16.1%	0.0%	19.6%
Q6SA96	5	7	1	2	2	2	0	1	19	51	1	6	10	4	0	3	42.7%	31.5%	7.7%	12.6%	7.7%	16.1%	0.0%	19.6%
Q9MYT8	4	4	1	1	1	1	1	0	8	62	5	10	3	7	1	0	50.7%	45.1%	16.9%	18.3%	18.3%	18.3%	18.3%	0.0%
tr|F2Z5K9|F2Z5K9	4	1	1	0	2	0	0	1	18	3	10	0	6	0	0	11	38.2%	6.6%	8.1%	0.0%	23.5%	0.0%	0.0%	23.5%
tr|I3LG73|I3LG73	2	3	2	0	1	2	0	0	2	19	8	0	3	3	0	0	4.9%	7.4%	9.0%	0.0%	2.7%	5.1%	0.0%	0.0%
tr|F2Z5D0|F2Z5D0	4	1	1	0	0	1	0	0	13	1	2	0	0	4	0	0	9.7%	5.3%	2.3%	0.0%	0.0%	5.3%	0.0%	0.0%
Q9TSX5	1	1	1	1	1	1	0	0	4	10	17	2	1	1	0	0	3.6%	3.6%	3.6%	3.6%	3.6%	3.6%	0.0%	0.0%
tr|F1SFX0|F1SFX0	1	1	1	1	1	1	0	0	4	10	17	2	1	1	0	0	4.1%	4.1%	4.1%	4.1%	4.1%	4.1%	0.0%	0.0%

	Peptide Count								Spectral Count								Sequence Coverage							
	Whole Cell	Whole Cell	Whole Cell	Cilia Fraction	Cilia Fraction	Cilia Fraction	Cilia Fraction	Cilia Fraction	Whole Cell	Whole Cell	Whole Cell	Cilia Fraction	Cilia Fraction	Cilia Fraction	Cilia Fraction	Cilia Fraction	Whole Cell	Whole Cell	Whole Cell	Cilia Fraction	Cilia Fraction	Cilia Fraction	Cilia Fraction	Cilia Fraction
Sample#	1	2	6	4	3	5	7	8	1	2	6	4	3	5	7	8	1	2	6	4	3	5	7	8
**Accession**																								
tr|K7GSP7|K7GSP7	4	1	4	0	2	0	3	19	5	2	9	0	2	0	38	204	11.7%	2.9%	11.8%	0.0%	4.7%	0.0%	8.3%	32.2%
tr|K7GN86|K7GN86	4	1	4	0	2	0	3	19	5	2	9	0	2	0	38	204	12.0%	3.0%	12.1%	0.0%	4.8%	0.0%	8.5%	33.1%
tr|F1SQX7|F1SQX7	4	1	4	0	2	0	3	18	5	2	9	0	2	0	38	203	12.2%	3.1%	12.3%	0.0%	4.9%	0.0%	8.6%	31.4%
P19156	2	0	3	1	1	0	2	2	7	0	20	1	1	0	266	117	2.7%	0.0%	2.7%	1.5%	1.3%	0.0%	1.3%	1.3%
tr|F1RM59|F1RM59	2	0	3	1	1	0	2	2	7	0	20	1	1	0	266	117	3.2%	0.0%	3.2%	1.7%	1.5%	0.0%	1.5%	1.5%
tr|K7GQM7|K7GQM7	0	2	0	1	1	0	2	2	0	7	0	1	1	0	37	14	0.0%	9.0%	0.0%	3.0%	3.0%	0.0%	9.0%	9.0%
tr|I3LLG9|I3LLG9	0	2	0	1	1	0	2	2	0	7	0	1	1	0	37	14	0.0%	6.6%	0.0%	2.2%	2.2%	0.0%	6.6%	6.6%
tr|K7GRD9|K7GRD9	0	2	0	1	1	0	2	2	0	7	0	1	1	0	37	14	0.0%	11.6%	0.0%	3.9%	3.9%	0.0%	11.6%	11.6%
tr|F1RUC0|F1RUC0	0	2	0	1	1	0	2	2	0	7	0	1	1	0	37	14	0.0%	6.6%	0.0%	2.2%	2.2%	0.0%	6.6%	6.6%
tr|F1S535|F1S535	2	2	3	0	2	0	0	2	4	3	7	0	2	0	0	2	5.1%	2.6%	5.9%	0.0%	3.4%	0.0%	0.0%	5.1%
tr|F1SH97|F1SH97	2	4	0	0	2	0	0	0	2	5	0	0	2	0	0	0	6.8%	8.2%	0.0%	0.0%	3.5%	0.0%	0.0%	0.0%
tr|F1RZW9|F1RZW9	1	2	0	0	0	2	0	0	3	4	0	0	0	2	0	0	5.5%	5.5%	0.0%	0.0%	0.0%	5.5%	0.0%	0.0%
tr|F1RPT3|F1RPT3	2	1	0	1	1	0	0	0	2	5	0	1	1	0	0	0	2.8%	0.5%	0.0%	0.5%	0.5%	0.0%	0.0%	0.0%
tr|F1SJ99|F1SJ99	1	1	0	0	1	0	0	0	2	5	0	0	2	0	0	0	13.0%	7.4%	0.0%	0.0%	7.4%	0.0%	0.0%	0.0%
tr|F1RRJ3|F1RRJ3	0	2	0	0	1	1	0	0	0	7	0	0	1	1	0	0	0.0%	6.6%	0.0%	0.0%	6.6%	6.6%	0.0%	0.0%
tr|F1S8H0|F1S8H0	3	1	0	0	1	0	0	0	4	3	0	0	2	0	0	0	30.1%	24.3%	0.0%	0.0%	24.3%	0.0%	0.0%	0.0%
tr|F1RVJ2|F1RVJ2	2	2	3	1	1	0	0	0	2	5	7	1	1	0	0	0	3.4%	1.7%	4.1%	0.8%	0.8%	0.0%	0.0%	0.0%
tr|F1SCS1|F1SCS1	11	9	6	1	5	1	1	1	19	27	40	3	8	2	1	2	21.8%	11.8%	8.6%	3.8%	9.4%	3.8%	1.5%	3.2%
tr|I3LMJ8|I3LMJ8	2	3	1	1	2	1	0	1	3	29	1	1	5	3	0	4	6.1%	12.2%	6.6%	2.9%	5.6%	2.9%	0.0%	6.6%
A1XQU5	5	7	4	1	3	1	1	3	11	14	26	3	3	1	1	8	33.1%	55.1%	30.1%	5.9%	20.6%	5.9%	8.1%	33.8%
tr|F1RYQ0|F1RYQ0	8	5	2	1	1	1	0	0	12	31	14	6	5	1	0	0	31.2%	16.8%	7.9%	2.3%	2.3%	2.3%	0.0%	0.0%
tr|I3LFN7|I3LFN7	5	3	3	1	2	0	0	0	11	7	21	2	3	0	0	0	27.0%	18.5%	16.3%	6.7%	12.9%	0.0%	0.0%	0.0%
tr|I3LG46|I3LG46	8	5	4	1	2	3	0	1	28	19	31	4	5	4	0	3	24.4%	17.4%	19.0%	3.7%	5.4%	5.4%	0.0%	11.2%
tr|F1RZQ6|F1RZQ6	8	13	3	4	5	7	1	2	25	138	69	14	15	16	10	26	27.2%	36.9%	12.8%	17.4%	20.8%	24.5%	9.1%	12.4%
tr|F1S758|F1S758	1	1	0	1	1	1	0	0	2	27	0	1	4	3	0	0	2.9%	2.9%	0.0%	2.9%	2.9%	2.9%	0.0%	0.0%
tr|F1RVF0|F1RVF0	1	1	0	1	1	1	0	0	2	27	0	1	4	3	0	0	2.4%	2.4%	0.0%	2.4%	2.4%	2.4%	0.0%	0.0%
tr|F1SPL8|F1SPL8	0	4	0	1	1	3	0	0	0	62	0	5	1	11	0	0	0.0%	13.3%	0.0%	10.9%	12.5%	13.3%	0.0%	0.0%
tr|I3LV37|I3LV37	0	4	0	1	1	3	0	0	0	62	0	5	1	11	0	0	0.0%	16.0%	0.0%	13.2%	15.1%	16.0%	0.0%	0.0%
Q29381	8	5	2	3	2	0	1	1	22	11	18	5	4	0	1	1	28.7%	15.3%	4.3%	8.4%	5.9%	0.0%	3.2%	9.3%
tr|F1RPY1|F1RPY1	10	2	3	1	3	0	2	3	18	4	12	3	3	0	4	4	27.2%	5.7%	13.3%	4.0%	5.7%	0.0%	3.4%	9.1%
tr|I3L8T2|I3L8T2	4	2	4	0	1	1	0	0	6	5	33	0	2	1	0	0	16.8%	5.0%	10.9%	0.0%	5.7%	2.8%	0.0%	0.0%
tr|F1SP18|F1SP18	14	8	4	2	4	1	2	7	33	45	16	8	11	2	6	16	23.7%	16.9%	11.5%	3.9%	6.5%	2.1%	4.2%	12.1%
tr|F1SPG0|F1SPG0	6	2	2	1	1	1	4	9	17	9	50	4	2	1	37	75	50.0%	15.8%	20.4%	6.6%	6.6%	6.6%	31.6%	49.3%
P54612	28	13	14	4	5	4	6	8	66	61	226	14	14	6	21	38	49.2%	20.2%	22.8%	7.0%	7.1%	4.9%	17.0%	22.6%
P23687	9	6	5	1	1	1	1	0	13	32	29	6	5	1	1	0	23.1%	13.0%	13.0%	1.5%	1.5%	1.5%	1.8%	0.0%
Q3ZDR4	5	0	1	0	0	2	0	4	19	0	39	0	0	5	0	20	25.3%	0.0%	7.4%	0.0%	0.0%	10.5%	0.0%	17.9%
tr|F1RMJ9|F1RMJ9	2	2	0	1	1	0	1	1	5	14	0	1	4	0	1	1	3.5%	3.6%	0.0%	1.6%	1.6%	0.0%	2.0%	2.0%
tr|F1S227|F1S227	3	1	1	1	1	0	0	0	3	20	6	4	2	0	0	0	27.4%	7.8%	8.4%	7.8%	7.8%	0.0%	0.0%	0.0%
tr|K7GMF7|K7GMF7	11	5	5	2	2	0	1	0	18	9	78	2	5	0	3	0	66.4%	38.9%	26.5%	10.6%	10.6%	0.0%	11.9%	0.0%
tr|I3LSY1|I3LSY1	3	1	1	1	1	1	0	0	29	2	4	2	1	5	0	0	29.8%	29.8%	16.7%	28.6%	29.8%	29.8%	0.0%	0.0%
tr|F1SL07|F1SL07	5	4	2	0	2	3	0	0	7	24	13	0	2	6	0	0	22.3%	10.6%	6.9%	0.0%	5.0%	8.2%	0.0%	0.0%
tr|I3LC00|I3LC00	5	3	1	1	2	1	0	1	6	29	1	1	5	3	0	4	13.6%	9.6%	5.2%	2.3%	4.4%	2.3%	0.0%	5.2%
tr|F1SQE6|F1SQE6	8	3	2	1	4	0	1	3	25	15	7	1	9	0	6	8	48.5%	18.0%	13.2%	11.4%	23.4%	0.0%	11.4%	16.8%
Q4FAT7	5	3	5	1	2	0	2	2	13	7	37	2	3	0	5	4	12.1%	5.8%	11.0%	1.5%	4.4%	0.0%	4.9%	4.9%
tr|F1SEQ7|F1SEQ7	19	17	14	8	9	6	4	9	119	145	276	28	29	9	37	100	54.6%	59.4%	41.9%	27.9%	42.8%	24.5%	23.6%	31.9%
tr|F1S533|F1S533	6	2	2	1	3	1	1	4	20	12	26	2	5	1	8	14	41.2%	18.1%	12.4%	8.0%	20.4%	10.2%	10.2%	24.3%
tr|F2Z5L6|F2Z5L6	5	2	0	0	2	1	1	4	22	2	0	0	4	2	12	21	23.1%	17.7%	0.0%	0.0%	21.5%	14.6%	14.6%	23.1%
Q29293	3	4	1	0	0	1	0	1	4	12	11	0	0	4	0	4	16.9%	17.7%	8.5%	0.0%	0.0%	9.2%	0.0%	8.5%
tr|F1S610|F1S610	5	3	2	0	2	0	2	8	5	7	7	0	3	0	8	24	16.8%	9.5%	4.5%	0.0%	10.7%	0.0%	9.3%	27.3%
P21999	0	1	0	0	1	1	0	0	0	12	0	0	2	1	0	0	0.0%	2.4%	0.0%	0.0%	2.4%	2.4%	0.0%	0.0%
P14057	0	1	0	0	1	1	0	0	0	12	0	0	2	1	0	0	0.0%	5.2%	0.0%	0.0%	5.2%	5.2%	0.0%	0.0%
tr|F1SCI9|F1SCI9	7	0	2	0	2	0	4	20	8	0	8	0	2	0	33	165	16.2%	0.0%	4.7%	0.0%	3.4%	0.0%	9.9%	30.5%
tr|K7GM14|K7GM14	4	0	3	0	2	0	4	5	8	0	10	0	2	0	38	62	8.3%	0.0%	9.4%	0.0%	6.7%	0.0%	9.6%	16.3%
tr|K7GLE2|K7GLE2	4	0	3	0	2	0	4	5	8	0	10	0	2	0	38	62	14.2%	0.0%	16.1%	0.0%	11.5%	0.0%	16.5%	28.0%
tr|K7GL11|K7GL11	4	0	3	0	2	0	4	5	8	0	10	0	2	0	38	62	6.3%	0.0%	7.1%	0.0%	5.1%	0.0%	7.3%	12.3%
tr|F1SGT4|F1SGT4	4	0	3	0	2	0	4	5	8	0	10	0	2	0	38	62	3.9%	0.0%	4.4%	0.0%	3.2%	0.0%	4.6%	7.7%
tr|K7GN90|K7GN90	4	0	3	0	2	0	4	5	8	0	10	0	2	0	38	62	4.2%	0.0%	4.7%	0.0%	3.4%	0.0%	4.8%	8.2%
tr|K7GND9|K7GND9	4	0	3	0	2	0	4	5	8	0	10	0	2	0	38	62	4.4%	0.0%	5.0%	0.0%	3.6%	0.0%	5.1%	8.7%
tr|K7GLL1|K7GLL1	4	0	3	0	2	0	4	5	8	0	10	0	2	0	38	62	7.2%	0.0%	8.1%	0.0%	5.8%	0.0%	8.4%	14.2%
tr|I3LI00|I3LI00	4	0	0	1	1	0	0	1	8	0	0	1	1	0	0	2	11.9%	0.0%	0.0%	3.1%	3.1%	0.0%	0.0%	3.1%
tr|F1SP04|F1SP04	6	1	3	0	1	0	0	1	7	1	6	0	2	0	0	4	8.5%	2.1%	4.6%	0.0%	2.1%	0.0%	0.0%	1.2%
tr|I3LJB6|I3LJB6	5	1	0	1	1	0	0	1	6	2	0	1	1	0	0	1	12.1%	2.4%	0.0%	2.6%	1.7%	0.0%	0.0%	2.4%
tr|F1S0U8|F1S0U8	5	1	0	1	1	0	0	1	6	2	0	1	1	0	0	1	16.7%	3.3%	0.0%	3.5%	2.3%	0.0%	0.0%	3.3%
tr|I3L5E2|I3L5E2	0	2	0	0	1	0	0	1	0	8	0	0	2	0	0	1	0.0%	3.6%	0.0%	0.0%	3.6%	0.0%	0.0%	3.6%
tr|F1RVS8|F1RVS8	1	1	0	0	0	1	0	0	4	4	0	0	0	2	0	0	10.0%	3.3%	0.0%	0.0%	0.0%	3.3%	0.0%	0.0%
tr|F1RVX9|F1RVX9	2	1	0	1	1	0	0	0	2	6	0	1	1	0	0	0	4.0%	1.7%	0.0%	1.7%	1.7%	0.0%	0.0%	0.0%
tr|I3L8F3|I3L8F3	3	2	1	0	2	0	0	0	5	3	1	0	2	0	0	0	3.0%	2.0%	1.3%	0.0%	3.5%	0.0%	0.0%	0.0%
tr|F1SHY2|F1SHY2	3	2	1	0	2	0	0	0	5	3	1	0	2	0	0	0	3.0%	1.9%	1.3%	0.0%	3.5%	0.0%	0.0%	0.0%
tr|K7GKQ6|K7GKQ6	7	1	1	0	1	0	0	0	7	1	4	0	2	0	0	0	10.8%	1.1%	1.4%	0.0%	1.9%	0.0%	0.0%	0.0%
Q06AU4	2	1	0	0	1	0	1	1	2	2	0	0	1	0	4	1	14.3%	5.4%	0.0%	0.0%	6.2%	0.0%	7.7%	6.2%
tr|F1SB19|F1SB19	1	0	0	1	0	0	0	1	4	0	0	1	0	0	0	3	4.6%	0.0%	0.0%	3.4%	0.0%	0.0%	0.0%	4.6%
tr|F1SI09|F1SI09	3	1	1	0	1	0	1	0	3	1	6	0	1	0	5	0	10.8%	5.0%	5.3%	0.0%	6.1%	0.0%	5.0%	0.0%
tr|F1RXP8|F1RXP8	3	1	1	0	1	0	1	2	3	1	5	0	1	0	1	3	3.4%	1.4%	1.3%	0.0%	1.7%	0.0%	1.3%	2.7%
P09571	1	1	0	0	1	0	1	0	1	3	0	0	1	0	1	0	1.7%	1.3%	0.0%	0.0%	1.3%	0.0%	2.6%	0.0%
P84024	2	1	0	0	0	1	0	1	2	2	0	0	0	1	0	1	8.0%	2.6%	0.0%	0.0%	0.0%	6.4%	0.0%	6.4%
tr|F1RPR3|F1RPR3	2	1	0	0	0	1	0	1	2	2	0	0	0	1	0	1	7.7%	2.5%	0.0%	0.0%	0.0%	6.2%	0.0%	6.2%
tr|B3CL06|B3CL06	1	1	0	0	1	0	1	0	1	3	0	0	1	0	1	0	1.7%	1.3%	0.0%	0.0%	1.3%	0.0%	2.5%	0.0%
tr|F1SJJ0|F1SJJ0	2	1	0	0	0	1	0	1	2	2	0	0	0	1	0	1	9.5%	3.1%	0.0%	0.0%	0.0%	7.6%	0.0%	7.6%
tr|F1RXG4|F1RXG4	1	1	1	0	1	0	1	1	3	1	24	0	1	0	1	5	2.5%	2.5%	2.5%	0.0%	2.5%	0.0%	2.5%	2.5%
tr|F1RMN9|F1RMN9	2	1	0	1	0	0	0	0	3	1	0	1	0	0	0	0	12.0%	6.5%	0.0%	6.5%	0.0%	0.0%	0.0%	0.0%
tr|I3LQW7|I3LQW7	1	2	0	0	1	0	0	0	1	3	0	0	1	0	0	0	3.8%	4.1%	0.0%	0.0%	4.3%	0.0%	0.0%	0.0%

	Peptide Count								Spectral Count								Sequence Coverage							
	Whole Cell	Whole Cell	Whole Cell	Cilia Fraction	Cilia Fraction	Cilia Fraction	Cilia Fraction	Cilia Fraction	Whole Cell	Whole Cell	Whole Cell	Cilia Fraction	Cilia Fraction	Cilia Fraction	Cilia Fraction	Cilia Fraction	Whole Cell	Whole Cell	Whole Cell	Cilia Fraction	Cilia Fraction	Cilia Fraction	Cilia Fraction	Cilia Fraction
Sample#	1	2	6	4	3	5	7	8	1	2	6	4	3	5	7	8	1	2	6	4	3	5	7	8
**Accession**																								
tr|I3LQA5|I3LQA5	1	3	0	0	1	0	0	0	1	3	0	0	1	0	0	0	0.9%	2.3%	0.0%	0.0%	0.9%	0.0%	0.0%	0.0%
tr|I3L591|I3L591	0	1	0	0	1	0	0	0	0	4	0	0	1	0	0	0	0.0%	8.0%	0.0%	0.0%	8.0%	0.0%	0.0%	0.0%
tr|F1SB37|F1SB37	3	0	0	0	1	0	0	0	4	0	0	0	1	0	0	0	6.7%	0.0%	0.0%	0.0%	3.4%	0.0%	0.0%	0.0%
tr|I3L8E1|I3L8E1	2	1	0	1	0	0	0	0	2	2	0	1	0	0	0	0	4.3%	4.3%	0.0%	4.3%	0.0%	0.0%	0.0%	0.0%
tr|K7GNW3|K7GNW3	2	1	0	0	1	0	0	0	2	2	0	0	1	0	0	0	6.6%	2.0%	0.0%	0.0%	2.0%	0.0%	0.0%	0.0%
tr|F1SKP7|F1SKP7	1	2	0	0	1	0	0	0	1	3	0	0	1	0	0	0	3.1%	3.4%	0.0%	0.0%	3.6%	0.0%	0.0%	0.0%
Q29116	1	1	0	0	0	1	0	0	1	3	0	0	0	1	0	0	0.9%	0.9%	0.0%	0.0%	0.0%	1.6%	0.0%	0.0%
tr|I3LP46|I3LP46	1	2	0	0	1	0	0	0	1	3	0	0	1	0	0	0	4.4%	4.7%	0.0%	0.0%	5.1%	0.0%	0.0%	0.0%
tr|I3LRK0|I3LRK0	1	2	0	0	1	0	0	0	1	3	0	0	1	0	0	0	3.1%	4.6%	0.0%	0.0%	2.9%	0.0%	0.0%	0.0%
tr|I3LVF4|I3LVF4	1	2	0	0	1	0	0	0	1	3	0	0	1	0	0	0	3.7%	4.0%	0.0%	0.0%	4.2%	0.0%	0.0%	0.0%
tr|I3LH17|I3LH17	1	1	0	0	1	0	0	0	1	3	0	0	1	0	0	0	6.0%	2.3%	0.0%	0.0%	2.3%	0.0%	0.0%	0.0%
tr|F1SI45|F1SI45	1	3	0	0	1	0	0	0	1	3	0	0	1	0	0	0	0.9%	2.3%	0.0%	0.0%	0.9%	0.0%	0.0%	0.0%
tr|I3L7A9|I3L7A9	0	1	0	0	1	0	0	0	0	4	0	0	1	0	0	0	0.0%	9.3%	0.0%	0.0%	9.3%	0.0%	0.0%	0.0%
tr|F1SU27|F1SU27	4	0	0	0	0	1	0	0	4	0	0	0	0	1	0	0	4.3%	0.0%	0.0%	0.0%	0.0%	1.5%	0.0%	0.0%
tr|F1SFL0|F1SFL0	1	1	0	1	0	0	0	0	3	1	0	1	0	0	0	0	3.8%	3.8%	0.0%	3.8%	0.0%	0.0%	0.0%	0.0%
tr|F1RLV7|F1RLV7	1	1	0	0	1	0	0	0	1	3	0	0	1	0	0	0	5.9%	2.2%	0.0%	0.0%	2.2%	0.0%	0.0%	0.0%
tr|F1SUT0|F1SUT0	3	0	2	1	0	0	0	1	4	0	14	1	0	0	0	3	13.5%	0.0%	12.0%	5.3%	0.0%	0.0%	0.0%	7.2%
tr|I3LHS8|I3LHS8	3	0	1	0	1	0	0	0	4	0	2	0	1	0	0	0	8.1%	0.0%	5.0%	0.0%	3.3%	0.0%	0.0%	0.0%
tr|F1SS96|F1SS96	2	1	1	0	1	0	0	0	3	1	3	0	1	0	0	0	6.8%	3.4%	4.2%	0.0%	3.4%	0.0%	0.0%	0.0%
tr|I3LN50|I3LN50	2	1	1	0	1	0	0	0	2	2	4	0	1	0	0	0	7.6%	3.1%	4.7%	0.0%	6.8%	0.0%	0.0%	0.0%
tr|F1RTN3|F1RTN3	38	15	26	9	15	3	16	32	112	153	437	28	28	9	657	408	57.4%	25.8%	35.9%	23.1%	25.8%	4.5%	35.0%	55.1%
tr|F1RWC5|F1RWC5	27	3	6	1	3	0	0	5	45	8	28	4	9	0	0	18	30.2%	4.3%	7.6%	2.3%	4.5%	0.0%	0.0%	8.8%
tr|I3LVD5|I3LVD5	46	35	29	13	27	17	16	37	1244	346	1111	116	242	31	2487	1223	67.5%	70.4%	56.5%	32.3%	62.1%	49.1%	39.5%	56.5%
Q6QAQ1	46	35	29	13	27	17	16	37	1244	346	1111	116	242	31	2487	1223	67.5%	70.4%	56.5%	32.3%	62.1%	49.1%	39.5%	56.5%
tr|F1SB42|F1SB42	17	11	12	5	9	3	1	13	59	105	123	13	18	9	46	225	32.6%	26.6%	26.3%	16.7%	16.7%	7.4%	3.1%	31.7%
tr|F2Z565|F2Z565	16	15	4	4	5	8	1	2	41	148	86	14	15	17	10	26	46.6%	48.0%	15.8%	17.4%	20.8%	30.2%	9.1%	12.4%
tr|F1S3D5|F1S3D5	8	2	6	1	1	1	3	7	13	12	24	1	4	1	67	119	31.5%	13.1%	22.0%	7.2%	7.2%	7.2%	18.4%	23.4%
P27485	8	5	4	1	2	3	2	1	18	24	24	2	4	4	3	1	35.8%	19.9%	19.4%	5.0%	6.0%	6.0%	19.9%	11.4%
tr|F1SC80|F1SC80	8	5	4	1	2	3	2	1	18	24	24	2	4	4	3	1	35.8%	19.9%	19.4%	5.0%	6.0%	6.0%	19.9%	11.4%
tr|F1RVA0|F1RVA0	4	1	1	0	2	0	0	1	18	3	10	0	5	0	0	9	38.2%	6.6%	8.1%	0.0%	23.5%	0.0%	0.0%	23.5%
Q71LE2	4	1	1	0	2	0	0	1	18	3	10	0	5	0	0	9	38.2%	6.6%	8.1%	0.0%	23.5%	0.0%	0.0%	23.5%
tr|I3LBK9|I3LBK9	9	2	1	1	2	0	0	1	18	3	15	2	3	0	0	1	24.2%	5.6%	3.8%	6.0%	9.5%	0.0%	0.0%	6.0%
tr|I3LI44|I3LI44	3	4	4	2	1	2	2	1	18	37	12	4	4	5	17	5	13.1%	21.9%	17.5%	11.7%	11.7%	11.7%	20.4%	11.7%
A5A8V7	14	8	7	2	4	0	1	4	28	23	52	6	6	0	3	6	22.5%	17.3%	10.6%	4.5%	10.1%	0.0%	3.6%	10.0%
tr|I3LG31|I3LG31	7	5	4	0	3	2	0	1	13	17	22	0	4	3	0	2	16.2%	9.8%	11.0%	0.0%	4.5%	2.7%	0.0%	2.7%
P34930	24	7	16	2	5	1	4	10	43	13	100	3	9	1	21	22	31.2%	15.4%	20.0%	4.8%	13.3%	3.4%	9.5%	20.0%
tr|F1RKI9|F1RKI9	6	5	2	2	2	3	1	1	11	54	6	6	5	4	1	1	21.1%	25.1%	7.4%	8.0%	8.0%	16.8%	4.3%	4.3%
tr|I3L7T6|I3L7T6	5	3	0	0	2	1	1	4	22	4	0	0	4	2	12	21	21.0%	21.0%	0.0%	0.0%	19.6%	13.3%	13.3%	21.0%
tr|F1RTQ7|F1RTQ7	5	3	0	0	2	1	1	4	22	4	0	0	4	2	12	21	22.9%	22.9%	0.0%	0.0%	21.4%	14.5%	14.5%	22.9%
tr|F1RR06|F1RR06	7	1	2	0	2	1	0	1	23	3	7	0	4	2	0	1	13.3%	5.2%	6.0%	0.0%	8.5%	5.2%	0.0%	2.2%
tr|I3LPP8|I3LPP8	1	3	0	1	2	0	0	0	2	11	0	1	2	0	0	0	3.8%	6.1%	0.0%	2.4%	6.1%	0.0%	0.0%	0.0%
tr|F1S530|F1S530	16	16	11	2	3	3	5	6	56	57	266	9	10	7	89	55	44.1%	42.1%	34.0%	6.1%	9.4%	10.8%	19.9%	23.9%
tr|I3LK51|I3LK51	8	4	2	1	4	1	0	1	31	17	15	4	6	1	0	6	30.1%	27.0%	14.1%	13.5%	28.2%	13.5%	0.0%	13.5%
tr|F1STD5|F1STD5	8	9	0	2	4	3	0	4	28	99	0	9	12	8	0	18	25.8%	13.1%	0.0%	4.4%	12.8%	9.7%	0.0%	17.8%
P80031	13	5	8	1	4	1	2	6	56	54	154	6	13	6	4	26	63.3%	28.0%	32.9%	5.3%	24.6%	5.3%	13.0%	32.9%
tr|Q2QC86|Q2QC86	2	1	0	1	1	1	0	0	4	18	0	2	2	1	0	0	32.8%	10.2%	0.0%	10.2%	10.2%	10.2%	0.0%	0.0%
tr|I3LIZ8|I3LIZ8	6	4	3	1	2	0	0	0	14	8	21	2	3	0	0	0	25.8%	18.8%	13.6%	5.6%	10.8%	0.0%	0.0%	0.0%
tr|F1SK45|F1SK45	3	2	5	1	3	1	0	0	6	16	25	1	3	1	0	0	17.2%	7.9%	26.0%	7.9%	14.1%	7.9%	0.0%	0.0%
tr|K7GS49|K7GS49	12	6	6	2	2	0	1	0	19	12	79	2	5	0	3	0	41.0%	25.1%	22.1%	6.2%	6.2%	0.0%	6.9%	0.0%
tr|F1SLR8|F1SLR8	18	19	10	5	7	5	7	9	404	359	1183	27	126	19	853	686	89.0%	89.0%	43.1%	47.7%	47.7%	34.9%	71.6%	82.6%
tr|F1S5Q1|F1S5Q1	6	3	2	1	1	1	2	3	25	55	20	3	9	6	4	10	13.6%	3.9%	3.2%	2.3%	2.3%	2.3%	5.5%	5.8%
Q19PY3	10	4	12	0	3	1	1	5	30	10	63	0	8	1	3	16	25.9%	13.5%	24.2%	0.0%	4.0%	2.4%	2.4%	18.0%
tr|F2Z5V0|F2Z5V0	10	4	12	0	3	1	1	5	30	10	63	0	8	1	3	16	25.9%	13.5%	24.2%	0.0%	4.0%	2.4%	2.4%	18.0%
P26042	36	14	23	8	14	3	14	29	107	147	404	22	26	9	573	363	50.6%	23.1%	32.4%	20.3%	23.1%	4.5%	26.9%	45.9%
tr|F1RM74|F1RM74	5	5	4	2	2	0	0	1	27	31	471	6	7	0	0	1	10.5%	10.5%	10.5%	8.6%	8.6%	0.0%	0.0%	3.9%
tr|F1SMC0|F1SMC0	18	8	5	2	2	0	1	3	45	45	37	8	12	0	8	6	47.3%	26.9%	18.1%	5.1%	5.1%	0.0%	5.1%	13.0%
tr|I3LCZ7|I3LCZ7	3	4	0	1	1	0	0	1	6	12	0	1	3	0	0	1	4.2%	4.4%	0.0%	2.4%	2.4%	0.0%	0.0%	1.8%
tr|I3LDQ9|I3LDQ9	1	3	3	0	0	1	0	0	4	14	23	0	0	4	0	0	6.7%	7.2%	18.9%	0.0%	0.0%	6.7%	0.0%	0.0%
tr|F1RK12|F1RK12	1	2	0	1	1	0	0	5	1	8	0	1	1	0	0	10	7.5%	7.9%	0.0%	3.8%	3.8%	0.0%	0.0%	27.1%
Q52N47	1	2	0	1	1	0	0	5	1	8	0	1	1	0	0	10	7.5%	7.9%	0.0%	3.8%	3.8%	0.0%	0.0%	27.1%
tr|F1RJI1|F1RJI1	2	2	1	0	1	0	1	1	6	3	3	0	2	0	4	3	5.5%	10.0%	4.2%	0.0%	4.5%	0.0%	8.0%	8.0%
tr|F1ST47|F1ST47	0	1	0	1	1	0	0	0	0	9	0	1	1	0	0	0	0.0%	6.3%	0.0%	6.3%	6.3%	0.0%	0.0%	0.0%
tr|Q56P20|Q56P20	14	11	2	3	9	1	8	9	125	47	20	7	30	1	62	86	52.2%	58.9%	18.3%	33.3%	47.8%	16.1%	56.1%	42.2%
D2SW95	23	12	6	2	6	2	5	7	52	57	38	7	15	2	22	19	30.6%	13.2%	7.5%	2.9%	7.2%	2.8%	7.7%	13.1%
tr|F2Z5S3|F2Z5S3	7	2	2	0	2	2	0	0	20	12	3	0	2	5	0	0	18.9%	10.1%	4.4%	0.0%	7.1%	10.1%	0.0%	0.0%
tr|I3LD13|I3LD13	7	2	2	0	2	2	0	0	20	12	3	0	2	5	0	0	18.7%	10.0%	4.4%	0.0%	7.1%	10.0%	0.0%	0.0%
tr|F2Z4Z1|F2Z4Z1	15	6	9	1	6	2	6	8	67	20	116	3	14	2	50	30	53.4%	30.8%	27.5%	4.0%	29.1%	5.7%	19.4%	36.8%
tr|F1SAK6|F1SAK6	6	6	0	2	2	3	0	2	24	100	0	4	4	19	0	7	58.3%	35.0%	0.0%	14.6%	14.6%	19.4%	0.0%	14.6%
tr|B9V4F0|B9V4F0	9	9	1	3	8	0	3	8	86	29	12	5	20	0	42	82	40.0%	38.9%	6.1%	23.3%	47.8%	0.0%	17.8%	23.9%
Q2PKF4	5	5	2	1	4	2	7	11	22	47	4	1	6	8	56	83	14.5%	13.9%	12.5%	5.3%	12.0%	4.2%	27.3%	32.3%
tr|F1RYL3|F1RYL3	10	4	4	1	3	0	2	5	15	8	21	2	3	0	13	12	13.2%	5.1%	3.9%	1.2%	4.1%	0.0%	4.4%	7.7%
tr|F1SJA1|F1SJA1	3	3	1	1	1	2	0	1	30	67	4	2	17	2	0	2	15.6%	10.2%	5.3%	7.0%	7.0%	15.6%	0.0%	7.0%
tr|F1RPU0|F1RPU0	8	4	8	1	1	3	1	1	14	23	39	3	1	4	1	6	15.5%	7.0%	16.7%	1.5%	1.5%	5.3%	4.4%	4.4%
tr|I3L6B9|I3L6B9	11	3	1	1	2	1	0	0	25	12	7	2	4	2	0	0	47.2%	12.7%	4.0%	3.4%	8.0%	3.7%	0.0%	0.0%
tr|I3LP78|I3LP78	12	12	3	0	5	4	3	2	52	50	26	0	17	5	11	11	48.4%	52.6%	21.4%	0.0%	22.9%	15.1%	17.2%	14.6%
tr|F1RQC8|F1RQC8	9	4	2	2	2	0	1	1	13	38	13	5	6	0	6	7	8.1%	2.8%	3.6%	1.5%	1.3%	0.0%	1.0%	1.0%
tr|F1SNC6|F1SNC6	17	8	3	1	4	2	1	0	28	23	6	1	6	4	1	0	14.0%	6.1%	3.0%	0.8%	3.3%	2.0%	1.6%	0.0%
P26234	34	16	36	3	8	1	8	12	63	30	365	4	15	1	55	59	35.9%	19.0%	34.6%	3.8%	8.9%	1.5%	10.8%	17.7%
tr|F1SBP9|F1SBP9	19	5	1	1	1	1	0	2	29	27	7	5	6	1	0	2	23.8%	6.1%	1.9%	1.1%	1.1%	1.1%	0.0%	4.1%
tr|F1RV20|F1RV20	2	1	4	0	2	0	6	13	4	10	19	0	3	0	75	171	9.8%	3.4%	12.4%	0.0%	9.2%	0.0%	22.2%	34.6%
tr|I3LFZ2|I3LFZ2	3	1	1	0	1	0	2	1	4	10	8	0	3	0	11	8	44.6%	9.9%	15.7%	0.0%	9.9%	0.0%	34.7%	19.0%

	Peptide Count								Spectral Count								Sequence Coverage							
	Whole Cell	Whole Cell	Whole Cell	Cilia Fraction	Cilia Fraction	Cilia Fraction	Cilia Fraction	Cilia Fraction	Whole Cell	Whole Cell	Whole Cell	Cilia Fraction	Cilia Fraction	Cilia Fraction	Cilia Fraction	Cilia Fraction	Whole Cell	Whole Cell	Whole Cell	Cilia Fraction	Cilia Fraction	Cilia Fraction	Cilia Fraction	Cilia Fraction
Sample#	1	2	6	4	3	5	7	8	1	2	6	4	3	5	7	8	1	2	6	4	3	5	7	8
**Accession**																								
tr|F1S3K5|F1S3K5	4	3	5	1	2	0	0	0	7	7	22	1	2	0	0	0	9.1%	3.6%	8.1%	2.1%	2.1%	0.0%	0.0%	0.0%
tr|I3L566|I3L566	4	3	5	1	2	0	0	0	7	7	22	1	2	0	0	0	9.5%	3.8%	8.4%	2.2%	2.2%	0.0%	0.0%	0.0%
tr|F1SQT3|F1SQT3	12	8	3	2	3	4	1	0	40	73	26	8	7	9	5	0	31.7%	16.4%	9.4%	7.5%	10.8%	8.9%	3.1%	0.0%
tr|F1SKE2|F1SKE2	8	7	0	1	1	3	0	0	14	19	0	2	1	4	0	0	9.6%	10.6%	0.0%	2.5%	2.6%	5.1%	0.0%	0.0%
P62901	5	3	3	1	1	1	0	0	20	18	6	3	4	1	0	0	20.8%	26.4%	25.6%	11.2%	11.2%	11.2%	0.0%	0.0%
tr|F1STY9|F1STY9	1	2	0	1	1	0	0	0	10	9	0	2	2	0	0	0	10.3%	10.3%	0.0%	10.3%	10.3%	0.0%	0.0%	0.0%
tr|F1RIU3|F1RIU3	31	8	15	1	5	3	8	12	166	181	147	42	27	4	53	56	55.4%	28.4%	28.1%	2.4%	18.3%	7.7%	25.8%	29.4%
Q6QAT0	2	9	1	1	1	3	0	0	4	77	11	5	1	11	0	0	17.0%	46.7%	9.6%	10.4%	11.9%	12.6%	0.0%	0.0%
tr|F1RGM3|F1RGM3	4	2	2	1	1	0	1	1	8	16	12	2	3	0	4	1	43.9%	17.1%	20.3%	8.1%	8.1%	0.0%	15.4%	15.4%
tr|A4L2Z4|A4L2Z4	3	2	0	0	3	2	3	3	18	40	0	0	4	8	34	28	10.1%	4.2%	0.0%	0.0%	10.1%	4.2%	9.6%	10.1%
tr|F1SNB6|F1SNB6	1	2	0	1	1	1	1	1	33	103	0	10	10	8	18	1	9.1%	9.1%	0.0%	9.1%	9.1%	9.1%	9.1%	9.1%
P17741	12	5	5	1	1	1	0	1	32	32	33	9	3	1	0	1	31.4%	25.7%	20.0%	5.7%	5.7%	5.7%	0.0%	6.7%
tr|I3LK33|I3LK33	6	10	1	0	4	4	0	2	28	106	10	0	5	22	0	6	19.1%	31.9%	6.9%	0.0%	13.7%	14.7%	0.0%	5.9%
P79324	6	10	1	0	4	4	0	2	28	106	10	0	5	22	0	6	25.5%	42.5%	9.2%	0.0%	18.3%	19.6%	0.0%	7.8%
tr|F1S232|F1S232	15	16	17	3	3	2	9	7	34	56	127	7	7	4	62	24	33.4%	36.1%	19.2%	7.4%	10.5%	3.7%	23.6%	14.0%
tr|F1RGC0|F1RGC0	9	5	2	1	4	1	0	1	33	22	15	4	6	1	0	6	41.2%	37.8%	15.5%	14.9%	31.1%	14.9%	0.0%	14.9%
tr|F1SIH5|F1SIH5	10	1	4	2	1	0	0	0	17	3	169	3	1	0	0	0	49.2%	8.1%	23.9%	8.4%	8.4%	0.0%	0.0%	0.0%
tr|I3LK71|I3LK71	4	5	5	1	1	0	2	2	7	8	34	1	2	0	7	5	16.6%	16.3%	13.4%	3.9%	3.9%	0.0%	9.1%	15.3%
tr|F1RQS8|F1RQS8	0	1	0	1	1	0	0	0	0	15	0	1	2	0	0	0	0.0%	6.5%	0.0%	6.5%	6.5%	0.0%	0.0%	0.0%
tr|F1RJI7|F1RJI7	2	0	0	0	0	2	1	1	10	0	0	0	0	2	2	7	19.3%	0.0%	0.0%	0.0%	0.0%	19.3%	19.3%	19.3%
tr|F1S6Z3|F1S6Z3	4	1	4	1	0	0	3	2	8	2	36	2	0	0	13	12	49.4%	16.0%	27.2%	16.0%	0.0%	0.0%	45.7%	29.6%
tr|I3LU51|I3LU51	5	1	5	0	2	0	1	4	6	4	16	0	2	0	4	6	10.9%	2.2%	6.8%	0.0%	2.5%	0.0%	2.2%	10.4%
tr|Q6Q781|Q6Q781	5	0	3	1	0	0	1	3	10	0	18	2	0	0	1	4	16.0%	0.0%	9.3%	3.1%	0.0%	0.0%	4.2%	10.6%
tr|I3LC53|I3LC53	0	1	0	1	1	0	0	0	0	10	0	1	1	0	0	0	0.0%	3.6%	0.0%	3.6%	3.6%	0.0%	0.0%	0.0%
tr|I3LSG2|I3LSG2	0	1	0	1	1	0	0	0	0	10	0	1	1	0	0	0	0.0%	4.7%	0.0%	4.7%	4.7%	0.0%	0.0%	0.0%
tr|F1RGM8|F1RGM8	6	2	0	0	2	0	0	0	8	2	0	0	2	0	0	0	5.7%	2.8%	0.0%	0.0%	1.3%	0.0%	0.0%	0.0%
tr|F1RFG9|F1RFG9	1	1	0	0	2	0	0	0	2	8	0	0	2	0	0	0	4.4%	1.3%	0.0%	0.0%	5.8%	0.0%	0.0%	0.0%
tr|F1S4E0|F1S4E0	1	2	0	0	1	1	0	0	1	9	0	0	1	1	0	0	2.6%	2.6%	0.0%	0.0%	2.6%	2.6%	0.0%	0.0%
Q27HV0	9	1	1	0	1	0	0	0	9	1	4	0	2	0	0	0	12.0%	1.1%	1.3%	0.0%	1.9%	0.0%	0.0%	0.0%
tr|F1RSV2|F1RSV2	9	1	1	0	1	0	0	0	9	1	4	0	2	0	0	0	12.0%	1.1%	1.3%	0.0%	1.9%	0.0%	0.0%	0.0%
tr|F1SV89|F1SV89	4	2	3	0	1	0	0	0	7	3	14	0	2	0	0	0	13.8%	6.6%	10.7%	0.0%	2.3%	0.0%	0.0%	0.0%
tr|K7GRT9|K7GRT9	3	0	0	0	1	0	1	5	5	0	0	0	1	0	1	57	11.0%	0.0%	0.0%	0.0%	3.9%	0.0%	5.7%	23.7%
tr|F1RYZ1|F1RYZ1	3	0	0	0	1	0	1	5	5	0	0	0	1	0	1	57	9.9%	0.0%	0.0%	0.0%	3.6%	0.0%	5.1%	21.3%
tr|K7GR53|K7GR53	3	0	0	0	1	0	0	3	5	0	0	0	1	0	0	52	16.3%	0.0%	0.0%	0.0%	5.9%	0.0%	0.0%	16.3%
tr|K7GRB8|K7GRB8	3	0	0	0	1	0	0	3	5	0	0	0	1	0	0	52	12.3%	0.0%	0.0%	0.0%	4.4%	0.0%	0.0%	12.3%
tr|F1RRE1|F1RRE1	1	2	2	0	1	0	1	2	1	4	4	0	1	0	24	7	8.7%	6.0%	17.9%	0.0%	5.4%	0.0%	9.2%	14.7%
tr|I3LI77|I3LI77	3	0	2	1	0	0	2	4	5	0	31	1	0	0	48	45	8.9%	0.0%	4.2%	1.9%	0.0%	0.0%	10.4%	13.2%
tr|I3LFK8|I3LFK8	3	0	3	0	0	1	0	5	5	0	7	0	0	1	0	25	12.3%	0.0%	11.9%	0.0%	0.0%	2.3%	0.0%	20.9%
tr|F1SMN6|F1SMN6	3	0	0	0	1	0	1	2	5	0	0	0	1	0	3	6	26.9%	0.0%	0.0%	0.0%	14.3%	0.0%	14.3%	16.0%
tr|K7GNE9|K7GNE9	0	1	0	1	0	0	0	4	0	5	0	1	0	0	0	8	0.0%	1.4%	0.0%	1.4%	0.0%	0.0%	0.0%	17.4%
tr|F1RN78|F1RN78	0	1	0	1	0	0	0	4	0	5	0	1	0	0	0	8	0.0%	1.2%	0.0%	1.2%	0.0%	0.0%	0.0%	15.1%
tr|K7GQD0|K7GQD0	1	1	2	0	1	0	1	1	2	3	23	0	1	0	36	6	43.2%	43.2%	43.2%	0.0%	43.2%	0.0%	43.2%	43.2%
tr|F1S7N7|F1S7N7	2	1	0	0	1	0	1	0	4	1	0	0	1	0	6	0	38.7%	18.5%	0.0%	0.0%	18.5%	0.0%	18.5%	0.0%
tr|I3LVK2|I3LVK2	3	0	2	0	1	0	0	1	5	0	7	0	1	0	0	4	10.2%	0.0%	6.4%	0.0%	6.1%	0.0%	0.0%	4.1%
Q5FY69	2	0	1	1	0	0	0	1	5	0	8	1	0	0	0	3	5.4%	0.0%	2.8%	1.8%	0.0%	0.0%	0.0%	2.5%
tr|F1S432|F1S432	4	1	0	0	1	0	0	0	4	1	0	0	1	0	0	0	11.2%	2.1%	0.0%	0.0%	2.1%	0.0%	0.0%	0.0%
tr|I3LTA9|I3LTA9	0	2	0	0	1	0	0	0	0	5	0	0	1	0	0	0	0.0%	4.2%	0.0%	0.0%	1.5%	0.0%	0.0%	0.0%
tr|F1SR18|F1SR18	0	1	0	0	1	0	0	0	0	5	0	0	1	0	0	0	0.0%	1.6%	0.0%	0.0%	1.6%	0.0%	0.0%	0.0%
tr|F1SSS0|F1SSS0	1	2	0	0	0	1	0	0	1	4	0	0	0	1	0	0	1.9%	1.9%	0.0%	0.0%	0.0%	1.0%	0.0%	0.0%
tr|F1SFE4|F1SFE4	0	1	0	0	1	0	0	0	0	5	0	0	1	0	0	0	0.0%	6.0%	0.0%	0.0%	6.0%	0.0%	0.0%	0.0%
tr|F1SAY6|F1SAY6	2	1	0	0	1	0	0	0	4	1	0	0	1	0	0	0	6.3%	2.9%	0.0%	0.0%	3.3%	0.0%	0.0%	0.0%
tr|F2Z5P0|F2Z5P0	1	1	0	0	0	1	0	0	2	3	0	0	0	1	0	0	15.6%	15.6%	0.0%	0.0%	0.0%	15.6%	0.0%	0.0%
tr|F1RQW2|F1RQW2	2	1	0	1	0	0	0	0	2	3	0	1	0	0	0	0	2.4%	0.5%	0.0%	0.5%	0.0%	0.0%	0.0%	0.0%
tr|F1RSN2|F1RSN2	0	1	0	1	0	0	0	0	0	5	0	1	0	0	0	0	0.0%	2.1%	0.0%	2.1%	0.0%	0.0%	0.0%	0.0%
tr|F1RZD2|F1RZD2	3	2	0	0	1	0	0	0	3	2	0	0	1	0	0	0	2.5%	0.8%	0.0%	0.0%	0.8%	0.0%	0.0%	0.0%
tr|F1REY1|F1REY1	1	2	0	0	0	1	0	0	1	4	0	0	0	1	0	0	8.8%	13.2%	0.0%	0.0%	0.0%	4.4%	0.0%	0.0%
tr|I3LCH3|I3LCH3	1	2	0	0	1	0	0	0	1	4	0	0	1	0	0	0	9.1%	15.6%	0.0%	0.0%	9.1%	0.0%	0.0%	0.0%
tr|F1RK18|F1RK18	4	0	1	1	0	0	0	0	5	0	1	1	0	0	0	0	10.1%	0.0%	5.0%	3.0%	0.0%	0.0%	0.0%	0.0%
P19619	35	35	20	11	17	11	14	16	567	1330	1305	192	126	61	452	514	58.1%	61.8%	40.8%	34.7%	48.0%	27.2%	35.3%	37.9%
Q29092	61	22	40	7	11	11	7	16	246	115	312	26	26	20	64	101	52.1%	26.5%	47.1%	9.6%	14.9%	10.8%	15.0%	19.4%
tr|F1SA70|F1SA70	16	9	15	3	5	0	2	3	49	27	109	7	8	0	32	10	15.3%	16.0%	14.3%	6.0%	10.1%	0.0%	1.9%	3.9%
tr|F2Z5K5|F2Z5K5	42	33	21	10	19	10	13	27	614	614	828	78	130	33	319	537	75.0%	65.1%	61.3%	29.3%	50.5%	23.9%	43.9%	51.1%
tr|F1RTY6|F1RTY6	7	5	2	1	2	0	2	4	13	38	5	3	7	0	3	19	27.3%	13.5%	13.2%	3.2%	7.6%	0.0%	7.3%	15.1%
Q6S4N2	30	9	23	3	6	1	6	14	66	21	188	6	10	1	43	56	42.4%	19.7%	35.1%	6.9%	15.3%	3.4%	16.2%	31.4%
Q767L7	58	40	24	12	26	13	19	36	968	896	2141	95	216	52	809	910	79.5%	77.3%	61.5%	32.0%	63.5%	23.9%	50.0%	59.2%
tr|I3L7S2|I3L7S2	15	11	3	2	3	2	4	3	35	99	20	12	11	3	11	5	26.8%	13.8%	5.5%	3.6%	3.7%	1.9%	9.6%	7.2%
P54613	15	11	3	2	3	2	4	3	35	99	20	12	11	3	11	5	30.9%	15.9%	6.3%	4.2%	4.3%	2.2%	11.1%	8.3%
tr|F1STR9|F1STR9	10	6	4	2	2	1	1	1	35	22	90	5	5	1	1	9	46.0%	23.8%	17.7%	10.5%	10.5%	6.5%	5.2%	5.2%
tr|F2Z5H2|F2Z5H2	2	2	4	1	1	0	0	0	6	20	20	3	2	0	0	0	9.6%	6.3%	15.1%	3.0%	3.0%	0.0%	0.0%	0.0%
P36887	6	3	2	1	2	0	0	3	13	8	11	1	3	0	0	6	22.2%	13.4%	8.3%	3.7%	7.1%	0.0%	0.0%	15.4%
tr|F1SD45|F1SD45	6	3	2	1	2	0	0	3	13	8	11	1	3	0	0	6	22.2%	13.4%	8.3%	3.7%	7.1%	0.0%	0.0%	15.4%
tr|F1S420|F1S420	3	2	4	2	0	0	1	0	7	14	29	4	0	0	3	0	7.7%	4.3%	12.0%	6.7%	0.0%	0.0%	4.3%	0.0%
tr|F1S4K7|F1S4K7	6	5	11	1	1	1	0	2	10	11	37	1	2	1	0	2	22.0%	9.9%	17.5%	1.8%	3.3%	2.1%	0.0%	8.6%
tr|C4MXZ1|C4MXZ1	18	12	3	6	12	0	7	17	200	76	26	17	35	0	63	220	80.1%	61.3%	19.9%	45.9%	64.6%	0.0%	50.3%	60.8%
tr|F1S0X8|F1S0X8	2	2	4	2	1	1	0	2	3	151	13	19	9	1	0	6	3.3%	1.6%	12.0%	1.6%	1.6%	1.6%	0.0%	7.8%
tr|F2Z5G5|F2Z5G5	15	10	3	2	4	4	1	5	44	121	84	9	12	10	28	23	44.9%	14.9%	10.6%	4.3%	12.2%	11.7%	5.9%	16.8%
tr|F2Z571|F2Z571	55	38	25	12	23	12	17	32	824	696	1576	87	154	44	632	863	79.3%	74.4%	65.2%	31.9%	53.9%	23.8%	46.5%	53.0%
tr|F1RW08|F1RW08	11	6	4	0	2	1	0	0	23	9	15	0	3	3	0	0	19.2%	10.1%	10.4%	0.0%	5.9%	3.5%	0.0%	0.0%
tr|F1RQM2|F1RQM2	10	2	1	1	1	0	1	3	13	3	2	2	1	0	2	7	20.7%	3.3%	2.4%	1.8%	1.8%	0.0%	3.1%	8.7%
tr|I3LRS3|I3LRS3	1	2	0	0	1	0	0	0	7	9	0	0	3	0	0	0	1.3%	0.9%	0.0%	0.0%	0.8%	0.0%	0.0%	0.0%
tr|F1RI97|F1RI97	1	2	0	0	1	0	0	0	7	9	0	0	3	0	0	0	1.2%	0.8%	0.0%	0.0%	0.7%	0.0%	0.0%	0.0%

	Peptide Count								Spectral Count								Sequence Coverage							
	Whole Cell	Whole Cell	Whole Cell	Cilia Fraction	Cilia Fraction	Cilia Fraction	Cilia Fraction	Cilia Fraction	Whole Cell	Whole Cell	Whole Cell	Cilia Fraction	Cilia Fraction	Cilia Fraction	Cilia Fraction	Cilia Fraction	Whole Cell	Whole Cell	Whole Cell	Cilia Fraction	Cilia Fraction	Cilia Fraction	Cilia Fraction	Cilia Fraction
Sample#	1	2	6	4	3	5	7	8	1	2	6	4	3	5	7	8	1	2	6	4	3	5	7	8
**Accession**																								
tr|K7GPV5|K7GPV5	7	3	4	1	1	0	0	0	10	6	27	1	2	0	0	0	23.7%	11.5%	16.2%	3.1%	3.1%	0.0%	0.0%	0.0%
tr|F1S982|F1S982	18	9	6	1	3	1	3	3	34	41	36	3	10	1	17	6	30.5%	16.3%	12.5%	2.0%	5.8%	1.9%	5.6%	8.3%
Q6QAP7	5	5	6	3	3	2	4	3	11	32	40	3	3	2	66	9	51.9%	31.1%	43.7%	23.7%	23.7%	23.7%	40.7%	40.7%
tr|I3LGC6|I3LGC6	8	3	0	1	3	0	0	0	10	17	0	1	4	0	0	0	5.4%	1.5%	0.0%	0.5%	1.9%	0.0%	0.0%	0.0%
P62272	10	8	8	1	6	1	1	3	25	67	96	4	12	1	1	16	48.7%	30.3%	42.8%	5.9%	31.6%	6.6%	5.3%	16.4%
tr|F1S441|F1S441	23	9	33	2	2	6	3	14	83	43	159	8	5	10	23	55	48.4%	18.5%	41.5%	3.5%	4.9%	13.8%	5.2%	25.6%
tr|F1SM21|F1SM21	11	6	2	1	2	1	0	0	22	22	14	3	4	1	0	0	23.8%	14.0%	3.6%	3.0%	3.0%	3.0%	0.0%	0.0%
tr|K7GQ61|K7GQ61	6	3	4	1	1	1	1	1	13	9	62	2	1	1	17	19	41.4%	17.9%	22.8%	8.6%	8.6%	8.6%	9.3%	9.3%
tr|F1RRW8|F1RRW8	4	4	0	1	1	0	0	2	10	12	0	1	3	0	0	4	4.3%	3.1%	0.0%	1.7%	1.7%	0.0%	0.0%	2.6%
tr|F1SMG8|F1SMG8	4	3	0	1	1	0	0	0	7	15	0	2	2	0	0	0	16.1%	5.5%	0.0%	3.3%	3.3%	0.0%	0.0%	0.0%
Q7YS91	2	5	0	2	1	1	0	0	2	20	0	2	1	1	0	0	5.2%	9.7%	0.0%	3.8%	1.6%	2.2%	0.0%	0.0%
tr|F1ST64|F1ST64	2	5	0	2	1	1	0	0	2	20	0	2	1	1	0	0	5.2%	9.7%	0.0%	3.8%	1.6%	2.2%	0.0%	0.0%
tr|F1S9B3|F1S9B3	2	3	0	1	1	0	0	0	2	20	0	1	3	0	0	0	7.8%	9.1%	0.0%	3.4%	3.4%	0.0%	0.0%	0.0%
tr|I3L9S9|I3L9S9	4	5	0	0	2	1	0	0	5	17	0	0	3	1	0	0	4.6%	4.0%	0.0%	0.0%	1.7%	0.9%	0.0%	0.0%
tr|F2Z5G3|F2Z5G3	3	1	1	0	2	0	2	5	10	1	2	0	2	0	19	22	36.2%	10.7%	11.4%	0.0%	10.7%	0.0%	24.8%	51.0%
tr|I3L7I0|I3L7I0	4	2	1	0	1	0	0	1	9	2	1	0	2	0	0	1	18.1%	2.8%	7.7%	0.0%	6.8%	0.0%	0.0%	7.7%
tr|F1RYS7|F1RYS7	1	1	0	1	1	0	0	0	1	10	0	1	1	0	0	0	1.3%	0.6%	0.0%	0.6%	0.6%	0.0%	0.0%	0.0%
tr|F1SRR5|F1SRR5	2	2	3	0	1	1	0	0	2	9	8	0	1	1	0	0	0.6%	0.4%	1.0%	0.0%	0.3%	0.2%	0.0%	0.0%
tr|I3LTM2|I3LTM2	2	2	3	0	1	1	0	0	2	9	8	0	1	1	0	0	0.6%	0.4%	1.0%	0.0%	0.3%	0.2%	0.0%	0.0%
tr|I3LEW5|I3LEW5	26	13	10	1	5	2	3	6	59	47	43	1	14	4	17	27	42.7%	20.6%	14.9%	2.2%	6.2%	2.2%	6.2%	7.0%
tr|F1SAD7|F1SAD7	5	3	0	1	1	0	0	0	5	23	0	3	2	0	0	0	9.5%	4.1%	0.0%	1.2%	1.2%	0.0%	0.0%	0.0%
tr|F1RJN6|F1RJN6	8	6	5	1	3	2	0	1	14	155	39	2	19	9	0	2	21.5%	14.5%	13.4%	2.5%	8.1%	3.1%	0.0%	3.6%
tr|F1S0P4|F1S0P4	2	3	3	1	2	0	0	0	2	32	10	2	4	0	0	0	4.7%	6.9%	8.5%	2.0%	6.9%	0.0%	0.0%	0.0%
tr|F1RFX9|F1RFX9	8	8	3	0	3	3	0	0	15	19	23	0	3	3	0	0	36.2%	19.9%	13.7%	0.0%	12.7%	10.7%	0.0%	0.0%
tr|F1RKM1|F1RKM1	4	1	2	1	1	0	1	2	13	4	13	1	2	0	1	6	15.8%	3.2%	9.4%	3.2%	3.2%	0.0%	3.2%	3.9%
tr|F1RGQ6|F1RGQ6	4	4	0	0	2	1	0	1	4	13	0	0	2	1	0	1	5.3%	4.4%	0.0%	0.0%	3.9%	2.0%	0.0%	2.0%
tr|I3LLT4|I3LLT4	1	1	0	1	0	1	0	0	2	15	0	2	0	1	0	0	8.1%	8.1%	0.0%	8.1%	0.0%	8.1%	0.0%	0.0%
P05383	5	2	2	1	1	0	0	1	10	7	11	1	2	0	0	1	17.4%	10.0%	8.3%	3.7%	3.7%	0.0%	0.0%	3.7%
Q29599	8	7	8	1	3	2	1	1	14	157	42	2	19	9	6	2	25.5%	21.5%	24.4%	2.6%	8.3%	3.2%	6.6%	3.7%
tr|F1SB63|F1SB63	29	23	16	3	8	2	11	15	82	90	77	8	19	3	131	65	58.8%	47.1%	34.9%	5.8%	23.4%	7.4%	37.1%	36.5%
tr|F1S935|F1S935	8	12	3	1	6	2	1	2	24	108	6	2	19	2	1	6	22.8%	34.8%	10.7%	4.0%	17.0%	8.5%	6.2%	10.7%
tr|I3L7B0|I3L7B0	2	2	0	0	1	2	0	2	19	27	0	0	1	7	0	6	6.9%	7.5%	0.0%	0.0%	6.9%	6.9%	0.0%	11.6%
tr|I3L5H6|I3L5H6	4	1	3	0	2	1	0	0	6	17	8	0	2	2	0	0	6.2%	1.5%	5.2%	0.0%	3.4%	1.9%	0.0%	0.0%
Q29205	13	10	2	3	5	3	1	1	26	223	15	10	28	5	5	12	50.0%	32.6%	11.8%	25.8%	30.9%	9.6%	4.5%	4.5%
tr|Q2EN77|Q2EN77	1	3	5	1	2	1	0	1	3	26	33	2	2	1	0	7	15.9%	18.5%	15.9%	8.6%	18.5%	8.6%	0.0%	15.9%
tr|Q3IA98|Q3IA98	4	5	1	2	4	3	0	2	106	312	7	4	37	31	0	49	49.0%	61.2%	20.4%	49.0%	61.2%	40.8%	0.0%	28.6%
P03974	38	22	30	3	3	6	7	11	127	94	240	16	11	11	82	66	53.6%	26.1%	34.5%	4.7%	1.7%	2.0%	13.9%	19.7%
tr|I3LPL7|I3LPL7	21	5	14	1	3	3	3	8	41	41	92	5	6	3	6	26	19.6%	4.5%	15.4%	0.8%	2.0%	3.1%	7.2%	10.3%
tr|I3LK07|I3LK07	6	5	1	1	2	1	1	1	50	120	169	10	11	8	18	1	30.0%	30.0%	11.5%	9.2%	20.8%	9.2%	9.2%	9.2%
tr|F1S2E5|F1S2E5	6	5	1	1	2	1	1	1	50	120	169	10	11	8	18	1	23.9%	23.9%	9.2%	7.4%	16.6%	7.4%	7.4%	7.4%
tr|I3LJP6|I3LJP6	6	5	1	1	2	1	1	1	50	120	169	10	11	8	18	1	29.5%	29.5%	11.4%	9.1%	20.5%	9.1%	9.1%	9.1%
P00355	43	43	19	11	22	12	12	20	947	614	1982	44	187	35	1328	819	76.6%	87.4%	39.6%	36.3%	59.8%	27.3%	40.2%	54.1%
Q29024	13	9	6	2	3	3	4	3	33	61	39	6	7	3	27	10	31.1%	16.6%	15.9%	4.9%	7.7%	5.1%	10.3%	9.8%
tr|I3LD74|I3LD74	14	11	3	3	5	3	1	1	28	226	23	10	28	5	5	12	55.1%	37.6%	16.9%	25.8%	30.9%	9.6%	4.5%	4.5%
tr|F1RU33|F1RU33	4	3	5	1	1	1	1	2	31	117	298	4	14	7	50	21	53.9%	14.6%	48.3%	13.5%	13.5%	13.5%	13.5%	40.4%
P02554	53	37	23	12	23	11	16	32	798	662	1487	62	143	40	513	728	80.2%	74.4%	66.1%	31.9%	51.2%	23.8%	46.5%	56.4%
P79403	26	13	11	4	5	4	5	13	74	105	70	11	11	8	21	43	27.9%	14.4%	16.3%	5.9%	6.8%	5.5%	11.1%	15.7%
tr|F1SIH8|F1SIH8	40	22	34	3	3	6	7	13	133	94	260	16	11	11	82	91	55.7%	26.0%	36.6%	4.7%	1.7%	2.0%	13.9%	21.9%
tr|I3LNH3|I3LNH3	27	13	11	4	5	4	5	13	75	105	70	11	11	8	21	42	30.6%	14.4%	16.3%	5.9%	6.8%	5.5%	11.1%	15.7%
Q9GLV6	18	12	5	1	2	3	1	0	33	33	15	2	6	3	1	0	24.7%	14.0%	6.3%	1.6%	4.3%	3.0%	1.6%	0.0%
tr|I3LRU5|I3LRU5	8	3	1	1	1	1	2	1	13	35	1	3	3	2	4	5	25.6%	8.4%	5.0%	2.3%	2.3%	2.3%	5.0%	6.1%
tr|I3L945|I3L945	8	3	3	1	1	1	2	1	13	35	5	3	3	2	4	5	25.0%	8.2%	11.9%	2.3%	2.3%	2.3%	4.9%	5.9%
tr|F2Z5B1|F2Z5B1	8	2	2	1	1	0	1	4	15	9	8	2	2	0	6	7	29.6%	2.6%	5.8%	2.6%	2.6%	0.0%	5.3%	17.5%
P80229	8	2	2	1	1	0	1	4	15	9	8	2	2	0	6	7	29.6%	2.6%	5.8%	2.6%	2.6%	0.0%	5.3%	17.5%
tr|F1RHW4|F1RHW4	7	1	1	0	2	1	3	13	17	1	2	0	2	1	36	112	41.7%	6.9%	7.3%	0.0%	18.8%	6.9%	20.2%	60.6%
tr|F8S319|F8S319	5	1	0	0	1	2	1	4	17	1	0	0	1	2	4	12	20.4%	3.1%	0.0%	0.0%	6.1%	3.2%	2.3%	8.2%
tr|F1SMM0|F1SMM0	7	4	2	0	1	2	3	3	11	7	9	0	1	2	10	5	8.3%	4.5%	2.8%	0.0%	1.8%	3.4%	3.8%	3.2%
tr|I3LGJ8|I3LGJ8	1	3	0	1	1	1	0	0	2	16	0	1	1	1	0	0	3.2%	7.4%	0.0%	4.6%	4.6%	2.8%	0.0%	0.0%
tr|I3L7T9|I3L7T9	5	5	3	0	0	1	0	0	8	10	9	0	0	3	0	0	25.1%	19.4%	4.5%	0.0%	0.0%	8.2%	0.0%	0.0%
tr|I3LKZ4|I3LKZ4	8	4	5	1	1	0	0	0	12	6	22	1	2	0	0	0	18.9%	6.8%	9.0%	1.7%	1.7%	0.0%	0.0%	0.0%
tr|I3LVL9|I3LVL9	3	3	3	1	1	0	1	2	6	6	29	1	1	0	3	7	7.4%	16.3%	11.1%	6.9%	6.9%	0.0%	6.9%	12.3%
tr|F1SH51|F1SH51	6	2	0	0	1	0	0	0	9	3	0	0	2	0	0	0	4.5%	2.2%	0.0%	0.0%	1.1%	0.0%	0.0%	0.0%
tr|F1RYJ5|F1RYJ5	3	2	1	0	1	1	0	0	3	9	2	0	1	1	0	0	8.0%	6.8%	2.3%	0.0%	2.3%	2.3%	0.0%	0.0%
tr|F1RYA1|F1RYA1	4	1	1	0	1	0	0	0	7	5	5	0	2	0	0	0	19.2%	4.2%	2.5%	0.0%	4.2%	0.0%	0.0%	0.0%
tr|K7GKE4|K7GKE4	2	0	0	0	1	0	2	2	6	0	0	0	1	0	25	37	13.6%	0.0%	0.0%	0.0%	13.6%	0.0%	13.6%	13.6%
Q9TVC1	3	1	0	1	0	0	1	2	4	2	0	1	0	0	8	6	11.4%	2.3%	0.0%	2.7%	0.0%	0.0%	4.8%	7.0%
tr|F1RSG6|F1RSG6	3	1	0	1	0	0	1	2	4	2	0	1	0	0	8	6	11.4%	2.3%	0.0%	2.7%	0.0%	0.0%	4.8%	7.0%
tr|I3LQB3|I3LQB3	3	1	0	1	0	0	1	2	4	2	0	1	0	0	8	6	11.8%	2.4%	0.0%	2.8%	0.0%	0.0%	4.9%	7.3%
tr|F1S8V9|F1S8V9	4	1	0	0	0	1	1	1	4	2	0	0	0	1	1	1	3.6%	0.8%	0.0%	0.0%	0.0%	0.8%	1.4%	1.2%
tr|F1S7C8|F1S7C8	4	1	0	0	1	0	0	1	4	2	0	0	1	0	0	2	26.0%	10.8%	0.0%	0.0%	10.8%	0.0%	0.0%	9.4%
tr|F1RW98|F1RW98	3	1	1	0	1	0	0	1	4	2	6	0	1	0	0	4	44.2%	30.1%	15.9%	0.0%	30.1%	0.0%	0.0%	30.1%
tr|I3L8S3|I3L8S3	1	1	0	0	1	0	0	0	1	5	0	0	1	0	0	0	13.0%	7.2%	0.0%	0.0%	7.2%	0.0%	0.0%	0.0%
tr|I3L6K8|I3L6K8	1	1	0	0	1	0	0	0	1	5	0	0	1	0	0	0	1.8%	2.2%	0.0%	0.0%	2.2%	0.0%	0.0%	0.0%
tr|I3LRK9|I3LRK9	4	0	0	1	0	0	0	0	6	0	0	1	0	0	0	0	14.8%	0.0%	0.0%	3.8%	0.0%	0.0%	0.0%	0.0%
tr|F1RU11|F1RU11	3	0	0	0	1	0	0	0	6	0	0	0	1	0	0	0	6.1%	0.0%	0.0%	0.0%	1.9%	0.0%	0.0%	0.0%
Q8MJ49	4	1	0	0	1	0	0	0	5	1	0	0	1	0	0	0	14.0%	5.6%	0.0%	0.0%	5.6%	0.0%	0.0%	0.0%
tr|I3LB61|I3LB61	1	1	0	0	1	0	0	0	1	5	0	0	1	0	0	0	1.5%	1.5%	0.0%	0.0%	1.2%	0.0%	0.0%	0.0%
tr|F1RUS9|F1RUS9	4	1	0	0	1	0	0	0	5	1	0	0	1	0	0	0	22.7%	6.7%	0.0%	0.0%	6.7%	0.0%	0.0%	0.0%
tr|F1RIS8|F1RIS8	0	1	0	1	0	0	0	0	0	6	0	1	0	0	0	0	0.0%	2.5%	0.0%	2.5%	0.0%	0.0%	0.0%	0.0%
tr|K7GLL5|K7GLL5	3	0	0	0	1	0	0	0	6	0	0	0	1	0	0	0	16.3%	0.0%	0.0%	0.0%	7.1%	0.0%	0.0%	0.0%
tr|F1S6Z2|F1S6Z2	3	2	2	0	1	0	0	0	4	2	3	0	1	0	0	0	3.5%	2.0%	4.4%	0.0%	2.0%	0.0%	0.0%	0.0%

	Peptide Count								Spectral Count								Sequence Coverage							
	Whole Cell	Whole Cell	Whole Cell	Cilia Fraction	Cilia Fraction	Cilia Fraction	Cilia Fraction	Cilia Fraction	Whole Cell	Whole Cell	Whole Cell	Cilia Fraction	Cilia Fraction	Cilia Fraction	Cilia Fraction	Cilia Fraction	Whole Cell	Whole Cell	Whole Cell	Cilia Fraction	Cilia Fraction	Cilia Fraction	Cilia Fraction	Cilia Fraction
Sample#	1	2	6	4	3	5	7	8	1	2	6	4	3	5	7	8	1	2	6	4	3	5	7	8
**Accession**																								
tr|F1SSU6|F1SSU6	1	3	1	0	1	0	0	0	1	5	6	0	1	0	0	0	2.1%	5.4%	1.2%	0.0%	1.7%	0.0%	0.0%	0.0%
Q0PIT9	4	0	2	0	0	1	1	1	6	0	105	0	0	1	4	1	28.8%	0.0%	14.2%	0.0%	0.0%	9.0%	8.0%	8.0%
tr|A1XQS3|A1XQS3	0	2	3	0	1	0	0	0	0	6	22	0	1	0	0	0	0.0%	26.8%	25.6%	0.0%	12.2%	0.0%	0.0%	0.0%
tr|F1RX49|F1RX49	3	2	2	0	1	0	0	0	4	2	25	0	1	0	0	0	20.2%	16.7%	16.7%	0.0%	12.7%	0.0%	0.0%	0.0%
tr|I3LEC2|I3LEC2	15	8	7	3	3	1	4	6	52	51	42	8	7	2	28	15	72.2%	31.7%	37.6%	9.6%	9.6%	5.9%	16.3%	35.7%
tr|F1RR78|F1RR78	37	6	56	1	9	0	16	27	63	10	320	1	11	0	95	84	22.3%	2.2%	25.2%	0.4%	5.6%	0.0%	11.1%	18.6%
tr|F1RWF5|F1RWF5	9	6	5	0	2	3	0	0	23	44	36	0	3	8	0	0	40.9%	20.2%	24.5%	0.0%	4.7%	12.5%	0.0%	0.0%
tr|F1SN52|F1SN52	2	2	1	2	1	1	1	0	5	44	1	3	1	4	1	0	28.8%	7.9%	20.9%	29.5%	21.6%	7.9%	21.6%	0.0%
tr|F6Q4L6|F6Q4L6	10	8	7	0	2	2	0	0	18	31	37	0	3	5	0	0	26.2%	24.8%	21.2%	0.0%	5.8%	5.8%	0.0%	0.0%
tr|F1RX16|F1RX16	24	7	9	2	5	2	1	3	60	26	42	3	9	2	7	13	35.9%	14.7%	15.6%	3.6%	9.1%	5.0%	3.3%	6.8%
Q29073	7	3	1	1	1	0	3	1	17	20	10	5	1	0	5	4	37.1%	7.0%	4.3%	3.6%	3.6%	0.0%	12.2%	4.3%
tr|F2Z543|F2Z543	10	6	6	0	2	3	0	0	24	44	45	0	3	8	0	0	45.6%	19.0%	30.3%	0.0%	4.4%	11.7%	0.0%	0.0%
tr|F1SJQ6|F1SJQ6	7	6	4	1	4	1	1	2	33	29	27	2	7	1	4	12	33.8%	30.0%	19.2%	5.6%	22.1%	5.6%	5.6%	11.3%
tr|F1RKC9|F1RKC9	6	2	0	2	2	0	0	2	26	5	0	2	3	0	0	3	29.8%	14.0%	0.0%	6.4%	6.4%	0.0%	0.0%	11.9%
tr|F1SHF4|F1SHF4	8	2	2	1	2	0	1	0	19	12	11	1	4	0	1	0	19.6%	7.3%	5.8%	3.1%	3.1%	0.0%	3.1%	0.0%
tr|I3LRS5|I3LRS5	21	8	9	4	6	2	1	6	104	76	147	11	15	3	144	32	55.5%	40.2%	34.4%	18.2%	30.6%	12.9%	7.2%	36.4%
tr|F1S9S5|F1S9S5	13	3	12	2	1	1	3	7	28	152	85	19	9	1	21	24	24.0%	3.3%	22.6%	1.4%	1.4%	1.4%	7.9%	16.1%
tr|F2Z5F5|F2Z5F5	9	10	7	0	2	3	1	1	32	55	52	0	4	10	1	2	43.3%	44.7%	33.7%	0.0%	12.0%	21.2%	7.2%	7.2%
tr|F1SJB5|F1SJB5	41	37	26	11	19	15	17	18	595	296	1389	57	56	30	417	432	67.1%	71.4%	51.7%	41.3%	54.6%	35.5%	42.2%	44.2%
tr|F1S9S9|F1S9S9	14	3	10	2	1	1	3	5	29	152	77	19	9	1	21	21	26.0%	3.0%	16.7%	1.3%	1.3%	1.3%	7.2%	7.2%
tr|I3LI20|I3LI20	10	4	2	1	1	1	0	0	12	13	7	2	1	1	0	0	18.0%	5.5%	6.2%	1.8%	1.8%	1.8%	0.0%	0.0%
O02772	11	14	9	3	5	4	3	6	135	334	64	5	38	32	14	82	70.7%	76.7%	33.8%	24.8%	32.3%	26.3%	27.1%	37.6%
tr|F2Z5Q6|F2Z5Q6	16	8	6	2	1	2	1	1	56	82	40	3	17	2	4	2	43.3%	29.6%	22.7%	11.7%	6.9%	15.4%	8.1%	6.9%
A1XQU3	8	6	4	1	4	1	1	2	34	29	27	2	7	1	4	12	37.1%	30.0%	19.2%	5.6%	22.1%	5.6%	5.6%	11.3%
P62279	12	6	4	0	3	2	0	0	34	99	16	0	11	10	0	0	48.6%	46.7%	33.6%	0.0%	21.5%	12.1%	0.0%	0.0%
tr|F1STV2|F1STV2	2	2	2	2	1	0	0	0	8	11	17	2	1	0	0	0	14.3%	14.3%	14.3%	14.3%	7.5%	0.0%	0.0%	0.0%
tr|I3LTW5|I3LTW5	2	2	2	2	1	0	0	0	8	11	17	2	1	0	0	0	14.3%	14.3%	14.3%	14.3%	7.5%	0.0%	0.0%	0.0%
Q4PLW0	7	4	7	0	0	1	0	0	10	9	28	0	0	3	0	0	29.8%	16.6%	12.9%	0.0%	0.0%	8.3%	0.0%	0.0%
tr|I3LGW3|I3LGW3	20	12	5	1	2	3	1	0	37	33	15	2	6	3	1	0	25.6%	13.8%	6.1%	1.6%	4.2%	2.9%	1.6%	0.0%
tr|F2Z4Y1|F2Z4Y1	18	6	7	1	4	2	2	3	61	16	106	3	7	2	14	9	50.8%	22.8%	29.7%	4.1%	20.3%	5.7%	17.9%	17.5%
tr|F6Q5P0|F6Q5P0	12	7	4	0	3	2	0	0	34	101	16	0	11	10	0	0	34.0%	37.9%	23.5%	0.0%	15.0%	8.5%	0.0%	0.0%
tr|F1SUM3|F1SUM3	15	9	6	1	4	2	1	7	59	57	58	5	10	3	1	39	43.3%	58.1%	28.6%	5.2%	29.0%	9.5%	5.2%	22.9%
Q49I35	10	5	10	2	5	0	5	3	94	74	225	2	24	0	279	65	54.1%	48.1%	55.6%	25.2%	48.1%	0.0%	39.3%	20.0%
tr|E1CAJ5|E1CAJ5	48	26	33	3	11	8	7	19	168	233	525	6	35	21	83	256	68.7%	50.3%	56.6%	7.3%	22.4%	15.2%	20.6%	35.6%
tr|F1SSA6|F1SSA6	50	17	54	1	6	6	14	15	101	48	707	2	11	10	93	78	25.3%	8.5%	28.2%	0.8%	4.6%	3.4%	10.0%	11.2%
tr|F1SJS8|F1SJS8	10	7	5	2	2	0	1	3	20	58	20	9	3	0	1	24	46.8%	40.8%	25.9%	10.0%	10.0%	0.0%	4.0%	14.4%
tr|F1RTJ9|F1RTJ9	6	7	3	1	2	3	2	3	24	28	15	1	4	3	6	15	27.5%	33.1%	20.6%	9.4%	9.4%	11.2%	9.4%	16.2%
tr|I3LUC8|I3LUC8	9	5	7	1	1	0	2	4	15	11	45	3	1	0	36	64	23.9%	27.4%	23.9%	4.0%	6.5%	0.0%	6.0%	12.9%
tr|F1RJM2|F1RJM2	9	3	3	1	2	1	0	2	21	5	32	1	2	1	0	19	34.7%	15.3%	6.1%	3.8%	11.5%	5.7%	0.0%	21.4%
tr|F1RHI8|F1RHI8	7	3	3	1	1	0	0	0	9	17	12	1	3	0	0	0	35.5%	17.4%	11.6%	6.5%	6.5%	0.0%	0.0%	0.0%
tr|F2Z5E2|F2Z5E2	1	3	0	1	1	0	3	1	1	12	0	1	1	0	14	1	2.4%	7.1%	0.0%	4.5%	4.5%	0.0%	9.3%	4.5%
tr|F1SUQ5|F1SUQ5	5	3	1	0	2	0	1	0	6	7	5	0	2	0	4	0	5.9%	2.8%	1.9%	0.0%	1.8%	0.0%	1.2%	0.0%
tr|F1SU18|F1SU18	1	1	1	0	1	0	0	0	1	12	1	0	2	0	0	0	3.8%	3.8%	9.8%	0.0%	3.8%	0.0%	0.0%	0.0%
P12682	13	4	5	1	1	1	0	1	44	41	22	9	3	1	0	1	48.4%	19.1%	19.5%	5.6%	5.6%	5.6%	0.0%	6.5%
B6CVD7	11	5	3	0	1	2	0	2	25	47	7	0	3	8	0	3	23.7%	7.9%	12.8%	0.0%	2.6%	5.6%	0.0%	10.3%
tr|F1S538|F1S538	12	4	3	0	4	2	0	3	31	28	6	0	6	3	0	11	58.8%	16.1%	16.1%	0.0%	27.4%	5.8%	0.0%	11.3%
tr|F1SGG1|F1SGG1	17	4	6	0	3	0	6	8	64	15	49	0	12	0	25	57	44.0%	17.5%	22.2%	0.0%	10.9%	0.0%	24.9%	23.8%
tr|F1SV22|F1SV22	14	7	10	1	3	0	1	2	21	12	25	2	3	0	2	3	4.0%	1.1%	2.4%	0.1%	0.9%	0.0%	0.3%	0.6%
tr|I3LQN4|I3LQN4	11	7	4	3	4	2	0	3	42	91	17	10	4	6	0	4	41.8%	26.1%	13.7%	11.4%	14.7%	8.7%	0.0%	16.4%
tr|F1RVD4|F1RVD4	9	7	2	1	3	3	1	2	24	56	3	1	5	6	5	9	8.5%	6.4%	1.7%	1.2%	4.3%	2.6%	1.6%	2.6%
tr|F1RSG9|F1RSG9	9	1	4	1	1	1	1	0	31	9	26	3	1	2	1	0	43.5%	6.5%	19.4%	6.5%	6.5%	6.5%	6.5%	0.0%
tr|I3LJS6|I3LJS6	2	2	1	1	1	1	0	0	2	18	2	1	1	1	0	0	10.8%	10.8%	4.6%	4.2%	4.2%	6.7%	0.0%	0.0%
tr|I3LL80|I3LL80	21	18	7	8	12	4	2	3	238	463	64	35	61	9	15	28	56.9%	46.7%	24.6%	20.1%	27.5%	12.3%	10.5%	17.1%
tr|F1SDR7|F1SDR7	22	8	9	1	5	2	8	8	95	32	129	3	14	2	102	66	62.6%	24.8%	31.7%	4.1%	22.8%	5.7%	35.8%	46.3%
tr|F1SUM7|F1SUM7	7	7	4	1	3	0	1	3	16	31	33	2	5	0	3	14	39.5%	42.6%	25.1%	7.2%	20.0%	0.0%	7.2%	19.5%
tr|F1S408|F1S408	6	5	0	0	4	0	0	2	14	13	0	0	4	0	0	4	14.8%	10.6%	0.0%	0.0%	7.5%	0.0%	0.0%	5.6%
tr|A5GFX6|A5GFX6	5	6	3	1	2	3	2	5	32	131	115	2	12	10	6	37	4.7%	4.7%	6.2%	2.4%	2.4%	2.4%	4.5%	4.7%
tr|F1SFF4|F1SFF4	13	3	3	1	3	0	1	5	25	9	12	1	4	0	22	8	43.0%	12.3%	12.3%	5.7%	12.7%	0.0%	6.6%	31.1%
tr|F1RF77|F1RF77	12	6	3	1	1	2	0	1	13	21	8	1	1	3	0	5	21.0%	8.5%	4.2%	2.1%	2.1%	1.3%	0.0%	2.9%
Q9TSX9	17	10	15	3	6	3	4	9	44	664	141	50	44	10	22	73	65.2%	41.1%	51.8%	10.3%	25.0%	11.2%	33.0%	44.6%
tr|K7GLE1|K7GLE1	34	33	23	9	16	13	14	15	524	240	1369	40	48	23	381	374	54.7%	60.1%	47.0%	31.1%	44.7%	29.9%	32.0%	40.5%
tr|I3LL55|I3LL55	8	4	1	1	1	4	2	0	20	42	1	2	1	6	3	0	21.4%	9.7%	3.0%	2.6%	2.6%	7.3%	5.0%	0.0%
tr|F1S1V1|F1S1V1	21	4	9	1	2	0	1	2	54	29	59	2	10	0	8	3	45.0%	15.7%	17.2%	3.5%	10.2%	0.0%	3.5%	8.5%
tr|F1SPM7|F1SPM7	28	9	4	2	3	2	4	6	75	78	41	4	11	7	15	14	46.3%	15.3%	8.4%	4.6%	7.0%	4.6%	9.8%	11.6%
tr|F1SPM6|F1SPM6	28	9	4	2	3	2	4	6	75	78	41	4	11	7	15	14	45.1%	14.9%	8.2%	4.4%	6.9%	4.4%	9.6%	11.3%
tr|I3LBY0|I3LBY0	15	13	4	2	3	1	1	2	22	48	9	5	4	1	1	2	32.9%	29.4%	9.0%	3.7%	6.1%	1.8%	2.9%	6.1%
tr|F2Z5I2|F2Z5I2	6	3	1	1	2	0	0	1	10	11	5	1	2	0	0	1	22.7%	5.0%	2.9%	1.9%	5.0%	0.0%	0.0%	3.0%
tr|F2Z536|F2Z536	4	2	0	0	1	0	0	1	9	5	0	0	2	0	0	25	51.7%	13.5%	0.0%	0.0%	12.4%	0.0%	0.0%	12.4%
tr|F1SAM8|F1SAM8	2	2	1	0	0	2	0	2	2	12	1	0	0	2	0	4	1.8%	0.8%	0.8%	0.0%	0.0%	0.8%	0.0%	1.7%
tr|I3LV69|I3LV69	5	1	1	0	2	0	0	1	12	2	4	0	2	0	0	1	29.2%	4.6%	7.4%	0.0%	4.6%	0.0%	0.0%	5.3%
tr|F1RKQ8|F1RKQ8	5	0	0	0	1	0	2	2	7	0	0	0	1	0	6	4	11.5%	0.0%	0.0%	0.0%	5.4%	0.0%	5.4%	5.4%
tr|F1RY91|F1RY91	0	1	0	1	0	0	0	1	0	7	0	1	0	0	0	4	0.0%	2.7%	0.0%	2.7%	0.0%	0.0%	0.0%	2.7%
tr|I3L939|I3L939	0	1	0	1	0	0	0	1	0	7	0	1	0	0	0	4	0.0%	3.3%	0.0%	3.3%	0.0%	0.0%	0.0%	3.3%
tr|F1S057|F1S057	5	1	1	0	1	0	0	3	6	1	1	0	1	0	0	4	9.3%	1.2%	1.5%	0.0%	1.8%	0.0%	0.0%	7.1%
tr|I3LDD5|I3LDD5	2	1	0	0	1	0	0	1	6	1	0	0	1	0	0	2	56.6%	39.5%	0.0%	0.0%	39.5%	0.0%	0.0%	17.1%
tr|F1RT87|F1RT87	3	0	4	0	1	0	1	2	7	0	25	0	1	0	3	6	44.7%	0.0%	57.4%	0.0%	14.9%	0.0%	12.8%	25.5%
tr|F1RW09|F1RW09	3	2	2	0	1	0	0	2	4	3	8	0	1	0	0	2	7.5%	3.7%	5.9%	0.0%	1.7%	0.0%	0.0%	5.9%
tr|F1SLN0|F1SLN0	4	2	0	0	1	0	0	0	4	3	0	0	1	0	0	0	4.6%	1.8%	0.0%	0.0%	0.7%	0.0%	0.0%	0.0%
tr|I3LUV7|I3LUV7	3	1	0	0	1	0	0	0	4	3	0	0	1	0	0	0	22.0%	5.2%	0.0%	0.0%	5.2%	0.0%	0.0%	0.0%
tr|I3LQ47|I3LQ47	4	2	0	0	1	0	0	0	4	3	0	0	1	0	0	0	4.6%	1.8%	0.0%	0.0%	0.7%	0.0%	0.0%	0.0%
Q29238	2	2	0	0	1	0	0	0	4	3	0	0	1	0	0	0	19.1%	18.2%	0.0%	0.0%	10.9%	0.0%	0.0%	0.0%

	Peptide Count								Spectral Count								Sequence Coverage							
	Whole Cell	Whole Cell	Whole Cell	Cilia Fraction	Cilia Fraction	Cilia Fraction	Cilia Fraction	Cilia Fraction	Whole Cell	Whole Cell	Whole Cell	Cilia Fraction	Cilia Fraction	Cilia Fraction	Cilia Fraction	Cilia Fraction	Whole Cell	Whole Cell	Whole Cell	Cilia Fraction	Cilia Fraction	Cilia Fraction	Cilia Fraction	Cilia Fraction
Sample#	1	2	6	4	3	5	7	8	1	2	6	4	3	5	7	8	1	2	6	4	3	5	7	8
**Accession**																								
tr|F1RGA7|F1RGA7	3	2	0	0	1	0	0	0	3	4	0	0	1	0	0	0	4.5%	3.5%	0.0%	0.0%	2.6%	0.0%	0.0%	0.0%
tr|F1RGT0|F1RGT0	3	3	0	0	1	0	0	0	3	4	0	0	1	0	0	0	4.1%	5.6%	0.0%	0.0%	1.9%	0.0%	0.0%	0.0%
P56941	3	0	0	0	0	1	0	0	7	0	0	0	0	1	0	0	4.6%	0.0%	0.0%	0.0%	0.0%	1.9%	0.0%	0.0%
tr|F1SUV1|F1SUV1	1	3	0	0	1	0	0	0	2	5	0	0	1	0	0	0	5.8%	16.2%	0.0%	0.0%	5.5%	0.0%	0.0%	0.0%
tr|F1SCZ6|F1SCZ6	2	1	0	1	0	0	0	0	2	5	0	1	0	0	0	0	7.5%	2.2%	0.0%	2.2%	0.0%	0.0%	0.0%	0.0%
tr|I3LG95|I3LG95	4	2	0	0	1	0	0	0	4	3	0	0	1	0	0	0	4.6%	1.8%	0.0%	0.0%	0.7%	0.0%	0.0%	0.0%
tr|F1SBB5|F1SBB5	3	0	0	0	0	1	0	0	7	0	0	0	0	1	0	0	4.6%	0.0%	0.0%	0.0%	0.0%	1.9%	0.0%	0.0%
tr|I3LRT6|I3LRT6	2	1	1	0	1	0	0	0	3	4	1	0	1	0	0	0	10.2%	3.7%	2.0%	0.0%	3.7%	0.0%	0.0%	0.0%
tr|F1RXF3|F1RXF3	3	2	2	0	1	0	0	0	4	3	4	0	1	0	0	0	15.2%	4.3%	6.1%	0.0%	8.5%	0.0%	0.0%	0.0%
tr|F1S129|F1S129	3	1	1	0	1	0	0	0	5	2	5	0	1	0	0	0	4.1%	0.9%	1.8%	0.0%	1.6%	0.0%	0.0%	0.0%
tr|F1SUP1|F1SUP1	4	1	4	0	1	0	0	2	6	1	57	0	1	0	0	4	12.7%	3.0%	17.6%	0.0%	4.1%	0.0%	0.0%	5.4%
tr|F1SIE0|F1SIE0	4	1	3	0	0	1	0	1	6	1	31	0	0	1	0	1	23.4%	4.3%	9.6%	0.0%	0.0%	5.3%	0.0%	6.9%
tr|F1SB57|F1SB57	5	1	4	1	0	0	0	0	5	2	14	1	0	0	0	0	36.5%	6.2%	12.4%	6.2%	0.0%	0.0%	0.0%	0.0%
tr|F1SB68|F1SB68	5	1	4	1	0	0	0	0	5	2	14	1	0	0	0	0	32.9%	5.6%	11.2%	5.6%	0.0%	0.0%	0.0%	0.0%
tr|F2Z5B2|F2Z5B2	51	37	23	12	22	11	16	30	774	541	1616	46	101	40	514	725	78.4%	77.1%	64.0%	31.9%	48.5%	23.8%	46.5%	53.9%
tr|F1SMZ6|F1SMZ6	10	6	8	1	2	2	2	4	67	130	275	16	9	3	14	17	54.9%	45.1%	65.7%	13.7%	25.5%	21.6%	25.5%	37.3%
tr|I3LDR2|I3LDR2	22	19	5	5	7	6	5	18	466	1263	65	57	115	72	742	476	29.1%	29.3%	12.8%	12.0%	18.3%	11.8%	12.3%	29.1%
P00336	30	20	12	8	12	4	2	4	279	467	86	35	61	9	15	34	66.8%	57.2%	35.0%	20.1%	27.5%	12.3%	10.5%	22.2%
tr|F1SV06|F1SV06	22	8	7	2	3	0	2	2	81	47	29	6	12	0	14	5	30.1%	15.4%	14.7%	2.5%	4.0%	0.0%	6.3%	3.8%
P10668	19	12	8	2	3	3	7	14	187	77	227	16	15	6	356	277	69.9%	63.3%	47.0%	15.1%	21.7%	20.5%	38.6%	57.8%
tr|F1RP44|F1RP44	33	14	5	2	7	1	1	2	65	78	26	5	14	1	3	2	38.5%	21.3%	6.8%	0.8%	11.6%	0.8%	1.3%	3.6%
Q95ND5	9	5	1	1	1	0	3	3	17	26	3	3	3	0	19	3	39.4%	27.8%	4.0%	5.1%	5.1%	0.0%	18.1%	16.2%
P27594	19	6	16	0	3	2	0	1	35	8	85	0	4	2	0	2	40.6%	13.1%	24.1%	0.0%	7.2%	4.2%	0.0%	3.2%
P00339	28	24	9	8	12	6	6	8	325	473	50	36	51	24	34	182	55.7%	59.9%	25.0%	26.8%	35.5%	14.5%	24.4%	29.8%
tr|I3LBB2|I3LBB2	19	10	4	2	5	2	0	1	40	32	17	2	6	2	0	1	29.1%	22.5%	6.4%	4.4%	14.3%	4.5%	0.0%	2.0%
tr|F1SR05|F1SR05	26	17	10	6	11	4	2	4	272	457	69	32	60	9	15	34	58.5%	50.4%	28.5%	16.6%	24.0%	12.2%	10.4%	22.0%
tr|F1RXA7|F1RXA7	10	4	4	1	1	4	2	0	23	42	8	2	1	6	3	0	25.2%	9.1%	10.1%	2.4%	2.4%	6.9%	4.7%	0.0%
tr|F1RPS8|F1RPS8	30	27	26	5	5	8	8	8	186	753	362	63	29	38	105	34	52.7%	51.4%	32.3%	14.6%	14.6%	16.7%	22.3%	27.8%
P80021	30	27	26	5	5	8	8	8	186	753	362	63	29	38	105	34	50.8%	49.5%	31.1%	14.1%	14.1%	16.1%	21.5%	26.8%
tr|F1SLA7|F1SLA7	3	6	1	1	2	1	0	1	6	23	10	1	2	1	0	2	17.8%	22.3%	6.9%	8.4%	9.9%	8.4%	0.0%	7.9%
tr|A6M930|A6M930	17	13	12	1	8	0	3	9	77	83	64	2	20	0	96	58	48.2%	32.4%	21.6%	4.7%	26.5%	0.0%	8.6%	29.2%
tr|F1RMR6|F1RMR6	8	5	3	1	2	2	1	0	25	26	8	1	2	4	1	0	31.7%	11.2%	13.2%	4.8%	7.6%	4.8%	3.4%	0.0%
tr|F1RQJ4|F1RQJ4	15	6	5	0	2	1	3	4	47	26	42	0	4	6	14	14	16.2%	4.7%	6.5%	0.0%	1.9%	0.9%	2.9%	5.2%
tr|F1SHQ8|F1SHQ8	27	22	7	8	10	7	8	21	575	1369	641	67	126	73	1007	503	40.8%	33.9%	19.2%	18.5%	24.1%	14.3%	18.7%	33.6%
tr|D0G0B6|D0G0B6	8	5	7	0	0	1	0	0	12	10	28	0	0	3	0	0	32.9%	19.6%	12.9%	0.0%	0.0%	8.3%	0.0%	0.0%
A6M931	8	6	7	1	4	0	3	2	22	37	24	1	7	0	70	35	17.0%	15.3%	11.7%	4.6%	16.5%	0.0%	8.5%	3.9%
tr|F2Z594|F2Z594	16	5	12	1	1	1	0	3	50	46	59	9	3	1	0	11	57.7%	27.9%	28.8%	5.6%	5.6%	5.6%	0.0%	15.3%
tr|K7GSU7|K7GSU7	15	5	3	2	2	2	4	7	64	25	33	5	2	5	64	41	62.7%	28.5%	25.4%	10.9%	10.9%	10.9%	27.5%	47.2%
Q2XQV4	13	6	11	1	2	1	1	1	38	14	54	1	3	3	1	1	42.4%	22.6%	24.4%	2.7%	12.9%	6.1%	2.7%	1.7%
tr|I3LN32|I3LN32	7	6	7	1	1	1	0	0	12	33	46	2	3	1	0	0	24.7%	15.9%	25.9%	3.2%	3.2%	3.6%	0.0%	0.0%
tr|K7GKN2|K7GKN2	20	6	17	0	3	2	0	1	37	8	99	0	4	2	0	2	41.0%	12.7%	25.5%	0.0%	7.0%	4.1%	0.0%	3.1%
tr|F1RS15|F1RS15	9	1	7	1	2	1	1	8	17	13	24	1	2	1	2	31	16.2%	1.7%	18.7%	1.7%	5.1%	1.7%	1.7%	16.8%
tr|F1RHZ9|F1RHZ9	2	2	0	1	1	1	0	1	2	28	0	2	1	1	0	1	4.7%	1.6%	0.0%	1.5%	1.5%	1.5%	0.0%	2.6%
tr|F1RM47|F1RM47	3	3	0	0	1	0	0	0	11	19	0	0	4	0	0	0	5.0%	5.4%	0.0%	0.0%	2.3%	0.0%	0.0%	0.0%
tr|F1SHR3|F1SHR3	5	2	6	0	1	1	2	5	8	7	52	0	1	1	5	55	26.1%	14.2%	24.3%	0.0%	4.4%	8.8%	15.9%	30.1%
Q52NJ3	4	3	0	0	1	0	2	0	8	7	0	0	2	0	5	0	35.9%	11.6%	0.0%	0.0%	5.6%	0.0%	17.2%	0.0%
tr|A5D9J7|A5D9J7	8	3	3	0	1	1	1	2	11	4	7	0	1	1	1	2	18.5%	4.8%	6.3%	0.0%	2.3%	2.3%	2.3%	3.2%
tr|F1RQ21|F1RQ21	8	3	3	0	1	1	1	2	11	4	7	0	1	1	1	2	18.5%	4.8%	6.3%	0.0%	2.3%	2.3%	2.3%	3.2%
tr|I3LLU0|I3LLU0	4	4	1	0	1	1	1	0	5	10	12	0	1	1	1	0	12.8%	17.1%	3.4%	0.0%	4.3%	3.1%	4.0%	0.0%
tr|B1NI70|B1NI70	9	4	2	1	1	1	0	0	18	35	15	3	2	2	0	0	61.0%	18.8%	10.8%	5.4%	5.4%	5.4%	0.0%	0.0%
tr|I3LBK8|I3LBK8	9	4	2	1	1	1	0	0	18	35	15	3	2	2	0	0	77.7%	24.0%	13.7%	6.9%	6.9%	6.9%	0.0%	0.0%
tr|I3L7U0|I3L7U0	28	8	22	2	3	0	6	12	62	14	183	3	7	0	43	51	43.2%	18.9%	35.4%	5.2%	10.5%	0.0%	17.4%	29.9%
tr|I3LLT2|I3LLT2	9	5	0	0	2	1	0	2	16	22	0	0	4	1	0	4	8.5%	4.0%	0.0%	0.0%	2.4%	0.6%	0.0%	2.4%
tr|F1SL54|F1SL54	15	6	16	1	2	2	5	5	22	16	77	1	2	2	13	8	27.7%	7.4%	19.2%	1.4%	3.6%	2.2%	8.6%	9.7%
P41367	13	5	3	1	2	2	2	0	51	71	16	5	5	6	2	0	33.7%	13.1%	8.1%	2.1%	5.7%	5.7%	11.6%	0.0%
tr|K7GST6|K7GST6	5	6	2	1	3	0	0	1	15	54	10	3	6	0	0	3	23.0%	29.1%	17.6%	7.4%	18.2%	0.0%	0.0%	5.4%
tr|K7GQ66|K7GQ66	5	6	2	1	3	0	0	1	15	54	10	3	6	0	0	3	23.1%	29.3%	17.7%	7.5%	18.4%	0.0%	0.0%	5.4%
tr|K7GMK5|K7GMK5	10	4	5	1	1	0	0	0	16	7	33	1	2	0	0	0	32.3%	15.5%	19.6%	2.7%	2.7%	0.0%	0.0%	0.0%
tr|F1RJX8|F1RJX8	44	16	7	2	6	1	3	13	108	46	41	6	12	2	9	33	40.2%	15.4%	7.5%	2.0%	6.8%	1.1%	4.0%	17.1%
P53027	6	4	3	1	2	0	0	2	14	140	44	3	17	0	0	11	23.6%	24.2%	20.6%	7.3%	7.9%	0.0%	0.0%	8.5%
tr|I3LJ44|I3LJ44	8	3	16	0	1	1	4	2	19	12	479	0	2	2	27	2	15.9%	3.0%	23.4%	0.0%	1.8%	1.8%	8.0%	5.3%
tr|F1SEN1|F1SEN1	36	20	14	4	5	3	9	8	170	211	136	14	14	21	148	37	77.4%	56.3%	39.8%	10.1%	15.0%	7.6%	38.8%	28.1%
tr|F6PV15|F6PV15	54	19	20	5	17	6	14	24	247	103	320	7	32	6	152	183	59.2%	29.4%	25.1%	6.9%	29.6%	7.5%	21.8%	30.1%
tr|K7GQL8|K7GQL8	54	19	20	5	17	6	14	24	247	103	320	7	32	6	152	183	59.7%	29.6%	25.4%	6.9%	29.9%	7.6%	22.0%	30.4%
tr|F1RQF6|F1RQF6	15	3	8	0	2	1	1	3	29	10	31	0	4	1	3	7	30.1%	4.4%	11.5%	0.0%	2.8%	2.8%	2.8%	12.5%
Q29201	7	13	3	1	2	2	2	4	22	103	73	6	6	4	2	13	35.6%	64.4%	21.9%	7.5%	15.8%	9.6%	15.8%	24.0%
P14332	6	4	1	1	3	1	3	3	33	53	1	3	6	2	10	18	27.6%	24.4%	3.2%	4.8%	14.0%	9.2%	14.0%	14.4%
Q0Z8U2	19	14	9	1	7	2	3	7	53	57	117	2	10	2	42	73	62.6%	61.7%	42.0%	5.8%	39.9%	12.8%	20.2%	33.7%
tr|I3LQ51|I3LQ51	15	6	8	2	5	2	4	4	66	123	61	9	10	5	53	33	59.8%	23.7%	35.7%	13.3%	21.2%	7.5%	20.7%	15.4%
tr|F1SML4|F1SML4	16	13	12	1	4	4	5	3	73	132	84	6	12	8	21	7	37.4%	29.3%	23.1%	1.9%	8.1%	6.6%	12.1%	7.4%
tr|F1SKJ1|F1SKJ1	102	30	89	2	10	8	18	32	231	378	1147	12	30	35	134	135	42.8%	13.5%	34.8%	0.7%	5.0%	2.5%	12.8%	21.6%
tr|K7GRF2|K7GRF2	14	8	3	1	4	0	1	3	41	46	26	3	8	0	4	28	56.8%	32.4%	19.9%	6.2%	22.7%	0.0%	4.5%	31.2%
tr|K7GNY4|K7GNY4	12	9	3	1	5	0	1	3	33	63	16	3	9	0	4	30	41.5%	27.1%	14.4%	4.7%	20.3%	0.0%	3.4%	19.5%
tr|F1SDJ8|F1SDJ8	17	4	7	2	2	0	1	5	32	16	25	3	3	0	2	6	57.5%	21.9%	22.5%	6.3%	6.3%	0.0%	3.9%	16.8%
tr|F1SB67|F1SB67	13	4	8	1	0	2	0	14	17	7	25	1	0	2	0	29	9.9%	1.7%	4.4%	0.8%	0.0%	1.3%	0.0%	13.2%
tr|F1RJV1|F1RJV1	1	3	0	1	1	0	1	1	1	23	0	1	2	0	3	5	2.6%	3.7%	0.0%	2.6%	2.6%	0.0%	2.6%	2.6%
tr|I3LDH5|I3LDH5	7	2	0	0	1	0	1	1	10	14	0	0	3	0	2	1	9.9%	1.6%	0.0%	0.0%	0.8%	0.0%	1.9%	0.8%
tr|F1SGB6|F1SGB6	6	3	0	0	1	1	0	0	8	16	0	0	2	1	0	0	12.1%	4.7%	0.0%	0.0%	1.1%	1.1%	0.0%	0.0%
tr|F1S0P3|F1S0P3	3	1	4	0	1	0	3	6	5	11	16	0	2	0	18	87	11.4%	3.1%	17.4%	0.0%	3.1%	0.0%	14.8%	26.9%
O46560	6	2	2	0	2	0	0	1	13	3	7	0	2	0	0	1	29.5%	7.8%	10.2%	0.0%	4.0%	0.0%	0.0%	4.7%
tr|F1ST63|F1ST63	1	4	0	1	1	0	0	0	1	15	0	1	1	0	0	0	3.7%	9.1%	0.0%	1.9%	1.9%	0.0%	0.0%	0.0%

	Peptide Count								Spectral Count								Sequence Coverage							
	Whole Cell	Whole Cell	Whole Cell	Cilia Fraction	Cilia Fraction	Cilia Fraction	Cilia Fraction	Cilia Fraction	Whole Cell	Whole Cell	Whole Cell	Cilia Fraction	Cilia Fraction	Cilia Fraction	Cilia Fraction	Cilia Fraction	Whole Cell	Whole Cell	Whole Cell	Cilia Fraction	Cilia Fraction	Cilia Fraction	Cilia Fraction	Cilia Fraction
Sample#	1	2	6	4	3	5	7	8	1	2	6	4	3	5	7	8	1	2	6	4	3	5	7	8
**Accession**																								
tr|I3LV91|I3LV91	2	2	4	1	1	0	0	1	3	13	14	1	1	0	0	1	9.4%	8.3%	12.0%	4.3%	4.3%	0.0%	0.0%	5.8%
tr|K7GPC9|K7GPC9	2	2	0	0	1	0	0	2	5	3	0	0	1	0	0	12	13.1%	13.1%	0.0%	0.0%	10.0%	0.0%	0.0%	13.8%
tr|K7GKQ1|K7GKQ1	2	2	0	0	1	0	0	2	5	3	0	0	1	0	0	12	11.9%	11.9%	0.0%	0.0%	9.0%	0.0%	0.0%	12.4%
Q09138	4	2	0	0	1	0	1	0	4	4	0	0	1	0	2	0	24.5%	3.6%	0.0%	0.0%	3.3%	0.0%	6.1%	0.0%
tr|F1RR91|F1RR91	1	1	0	0	1	0	0	1	5	3	0	0	1	0	0	1	3.0%	3.0%	0.0%	0.0%	3.0%	0.0%	0.0%	3.0%
tr|F1SBC8|F1SBC8	3	0	0	0	1	0	0	1	8	0	0	0	1	0	0	1	6.0%	0.0%	0.0%	0.0%	2.3%	0.0%	0.0%	1.0%
tr|E1U8C5|E1U8C5	3	1	0	0	1	0	0	0	3	5	0	0	1	0	0	0	7.9%	1.9%	0.0%	0.0%	1.9%	0.0%	0.0%	0.0%
tr|F1S210|F1S210	1	2	0	0	1	0	0	0	1	7	0	0	1	0	0	0	2.5%	4.4%	0.0%	0.0%	2.5%	0.0%	0.0%	0.0%
Q9GKE7	2	2	0	0	0	1	0	0	3	5	0	0	0	1	0	0	4.2%	3.4%	0.0%	0.0%	0.0%	3.4%	0.0%	0.0%
tr|I3L7N1|I3L7N1	0	1	0	1	0	0	0	0	0	8	0	1	0	0	0	0	0.0%	3.2%	0.0%	3.2%	0.0%	0.0%	0.0%	0.0%
P28839	1	2	1	0	1	0	0	0	1	7	1	0	1	0	0	0	25.0%	25.0%	50.0%	0.0%	25.0%	0.0%	0.0%	0.0%
tr|F1RHL7|F1RHL7	2	3	1	0	1	0	0	0	2	6	2	0	1	0	0	0	1.9%	1.8%	0.9%	0.0%	0.9%	0.0%	0.0%	0.0%
tr|F1SEY1|F1SEY1	4	1	1	0	1	0	0	0	4	4	3	0	1	0	0	0	7.7%	1.1%	1.3%	0.0%	1.1%	0.0%	0.0%	0.0%
tr|F1SMJ9|F1SMJ9	4	1	3	1	0	0	1	1	7	1	51	1	0	0	1	2	65.6%	10.0%	36.7%	10.0%	0.0%	0.0%	24.4%	26.7%
tr|K7GLP8|K7GLP8	4	0	1	0	1	0	0	0	8	0	7	0	1	0	0	0	11.7%	0.0%	2.3%	0.0%	3.3%	0.0%	0.0%	0.0%
tr|F1SV03|F1SV03	2	1	1	0	1	0	0	0	5	3	8	0	1	0	0	0	11.3%	3.0%	3.0%	0.0%	3.0%	0.0%	0.0%	0.0%
tr|F1RP34|F1RP34	5	1	2	0	0	1	1	1	7	1	105	0	0	1	4	1	33.0%	4.2%	14.2%	0.0%	0.0%	9.0%	8.0%	8.0%
tr|I3LK68|I3LK68	4	3	4	0	1	0	0	0	5	3	28	0	1	0	0	0	12.3%	9.0%	14.6%	0.0%	5.2%	0.0%	0.0%	0.0%
tr|F2Z5S8|F2Z5S8	39	27	14	9	11	7	12	30	797	1418	1236	69	130	73	1403	868	70.5%	50.0%	38.2%	20.5%	26.1%	14.3%	31.9%	59.2%
tr|F1SPR4|F1SPR4	7	6	0	2	2	1	2	3	15	34	0	3	2	1	3	5	32.2%	19.7%	0.0%	7.2%	6.9%	3.9%	10.3%	15.6%
P0C5I2	7	6	0	2	2	1	2	3	15	34	0	3	2	1	3	5	32.2%	19.7%	0.0%	7.2%	6.9%	3.9%	10.3%	15.6%
Q2XVP4	46	33	16	10	16	7	12	33	878	1450	1503	70	141	73	1421	891	78.9%	71.8%	47.2%	22.6%	44.3%	14.2%	31.7%	67.8%
tr|F1RWJ5|F1RWJ5	38	16	9	2	6	4	7	7	117	170	58	2	15	18	19	22	51.9%	25.5%	12.9%	5.9%	9.9%	4.0%	14.8%	11.3%
tr|I3L929|I3L929	11	5	5	1	1	0	2	3	30	11	33	4	1	0	52	14	30.7%	17.8%	14.4%	5.5%	3.1%	0.0%	7.7%	15.6%
tr|F2Z5N5|F2Z5N5	4	5	3	0	2	2	0	2	10	31	14	0	3	2	0	3	28.3%	26.9%	12.4%	0.0%	11.0%	15.9%	0.0%	13.8%
tr|F1RJK5|F1RJK5	29	12	3	1	5	1	2	1	63	102	14	4	15	1	5	3	25.4%	12.9%	3.8%	1.2%	3.6%	1.2%	3.7%	1.6%
tr|F1SJJ5|F1SJJ5	29	16	15	2	5	4	2	3	87	95	91	5	10	7	7	13	43.9%	35.0%	28.6%	6.8%	10.6%	8.2%	9.6%	12.0%
tr|F1SMN5|F1SMN5	26	10	26	1	2	1	12	22	49	42	142	2	8	1	39	48	14.0%	3.3%	13.0%	0.4%	1.0%	0.4%	6.4%	13.8%
tr|F1RIF8|F1RIF8	14	7	8	1	4	1	3	3	59	57	43	3	9	2	10	18	39.1%	23.3%	22.2%	2.5%	12.1%	4.8%	7.3%	7.5%
tr|F1SCH3|F1SCH3	16	8	5	2	3	2	2	3	34	49	25	3	5	2	22	10	32.2%	14.9%	10.3%	4.4%	6.9%	2.3%	5.1%	8.8%
tr|I3LDV0|I3LDV0	21	6	6	2	2	1	0	3	51	24	33	3	5	1	0	13	30.1%	12.4%	9.6%	3.6%	3.6%	1.6%	0.0%	6.7%
tr|F1S393|F1S393	6	1	9	0	2	1	2	3	22	28	85	0	3	3	47	60	38.9%	6.2%	35.4%	0.0%	15.3%	6.2%	12.5%	18.8%
tr|I3LAC0|I3LAC0	11	5	5	1	1	0	1	0	17	8	33	1	2	0	3	0	37.2%	21.4%	18.4%	2.6%	2.6%	0.0%	6.9%	0.0%
tr|F1S197|F1S197	2	4	3	1	1	1	0	0	2	23	15	1	1	1	0	0	7.5%	12.4%	7.5%	2.5%	4.2%	2.5%	0.0%	0.0%
tr|I3LIM9|I3LIM9	2	4	3	1	1	1	0	0	2	23	15	1	1	1	0	0	8.0%	13.2%	8.0%	2.7%	4.5%	2.7%	0.0%	0.0%
tr|I3L7Y1|I3L7Y1	4	6	3	0	2	2	0	2	10	32	14	0	3	2	0	3	28.3%	33.1%	12.4%	0.0%	11.0%	15.9%	0.0%	13.8%
tr|F1RZK8|F1RZK8	9	7	3	0	2	2	0	1	17	25	13	0	2	3	0	2	19.1%	14.2%	9.8%	0.0%	2.4%	2.4%	0.0%	2.4%
tr|F1SA98|F1SA98	17	8	12	1	2	1	5	4	86	41	129	3	6	6	72	47	41.8%	30.1%	35.2%	3.9%	13.3%	9.4%	19.5%	24.2%
tr|F1SFE3|F1SFE3	15	2	4	1	1	0	2	8	28	6	14	2	2	0	13	33	47.9%	6.1%	12.9%	3.8%	3.8%	0.0%	7.5%	31.1%
tr|I3L7J2|I3L7J2	4	3	1	1	1	0	1	1	7	27	9	1	3	0	3	7	51.4%	25.2%	24.3%	10.3%	10.3%	0.0%	10.3%	24.3%
tr|F1SS01|F1SS01	3	4	4	0	2	0	2	5	5	12	29	0	2	0	3	10	24.6%	18.0%	20.4%	0.0%	9.5%	0.0%	14.7%	36.5%
tr|F1S0C0|F1S0C0	4	1	0	0	0	2	0	0	12	5	0	0	0	2	0	0	15.8%	5.7%	0.0%	0.0%	0.0%	5.7%	0.0%	0.0%
tr|F1SRK6|F1SRK6	53	18	32	5	10	6	5	14	212	46	248	9	15	6	46	97	43.9%	25.4%	37.2%	7.7%	16.9%	8.8%	10.2%	17.4%
tr|F1S2G1|F1S2G1	11	5	0	0	2	1	2	4	20	23	0	0	4	1	3	5	10.3%	5.1%	0.0%	0.0%	2.1%	2.0%	2.7%	4.3%
Q9GL01	18	5	7	1	5	1	2	5	63	6	33	1	6	1	14	29	34.2%	15.7%	14.6%	3.5%	16.2%	4.9%	4.8%	16.5%
tr|F1SLU6|F1SLU6	16	8	7	0	2	2	3	4	37	32	87	0	6	2	32	10	29.0%	8.3%	12.6%	0.0%	2.8%	2.8%	4.2%	8.8%
Q06AU7	5	4	4	0	1	2	2	3	13	13	23	0	1	2	35	43	30.3%	23.9%	20.4%	0.0%	8.5%	15.9%	17.9%	16.9%
Q9GJT2	10	2	4	0	2	0	5	4	19	7	31	0	3	0	36	45	56.7%	20.6%	14.9%	0.0%	14.5%	0.0%	28.4%	20.9%
Q29048	11	4	13	0	0	2	5	13	21	5	95	0	0	3	28	53	17.5%	8.3%	21.4%	0.0%	0.0%	4.1%	13.5%	27.1%
tr|F1S5J8|F1S5J8	13	5	4	2	1	1	1	4	39	22	9	3	3	1	1	9	24.2%	7.5%	7.9%	1.5%	1.5%	1.5%	3.1%	11.1%
tr|F1SI10|F1SI10	6	6	5	0	4	2	1	3	23	47	232	0	6	2	11	22	22.9%	26.6%	17.3%	0.0%	26.6%	12.1%	12.6%	13.6%
tr|F1SNL8|F1SNL8	8	2	2	0	2	1	0	1	17	53	9	0	4	4	0	1	22.3%	4.9%	4.0%	0.0%	8.7%	2.8%	0.0%	3.6%
tr|F1SN37|F1SN37	27	10	16	0	4	3	4	10	70	45	312	0	10	3	52	36	45.6%	14.5%	24.4%	0.0%	6.3%	5.4%	9.4%	18.5%
tr|I3LCL6|I3LCL6	14	5	4	2	1	1	1	4	40	22	9	3	3	1	1	9	24.2%	7.5%	7.9%	1.5%	1.5%	1.5%	3.1%	11.1%
tr|K7GND3|K7GND3	9	6	2	0	2	1	1	0	29	33	88	0	2	5	3	0	34.1%	17.6%	7.4%	0.0%	9.0%	5.0%	5.0%	0.0%
tr|F1S666|F1S666	15	7	6	0	3	1	1	1	36	35	100	0	3	5	3	3	39.0%	16.3%	15.4%	0.0%	9.5%	3.9%	3.9%	2.7%
Q764M5	28	10	16	0	4	3	5	11	71	45	312	0	10	3	55	41	49.8%	14.5%	24.4%	0.0%	6.3%	5.4%	13.6%	22.7%
tr|B5KJG2|B5KJG2	3	5	2	0	2	3	1	4	17	73	13	0	6	4	20	84	8.3%	13.0%	8.3%	0.0%	4.7%	4.7%	4.0%	10.7%
tr|F2Z567|F2Z567	16	10	4	1	5	0	1	4	44	64	29	3	9	0	4	31	44.7%	31.1%	19.5%	4.3%	18.7%	0.0%	3.1%	24.5%
tr|F8S326|F8S326	10	1	0	0	1	2	2	5	26	1	0	0	1	2	7	23	32.6%	3.1%	0.0%	0.0%	6.1%	3.2%	7.6%	11.8%
tr|I3LFM9|I3LFM9	10	1	0	0	1	2	2	5	26	1	0	0	1	2	7	23	32.6%	3.1%	0.0%	0.0%	6.1%	3.2%	7.6%	11.8%
tr|I3LB18|I3LB18	10	1	0	0	1	2	2	5	26	1	0	0	1	2	7	23	32.6%	3.1%	0.0%	0.0%	6.1%	3.2%	7.6%	11.8%
tr|I3LAE0|I3LAE0	10	1	0	0	1	2	2	5	26	1	0	0	1	2	7	23	32.6%	3.1%	0.0%	0.0%	6.1%	3.2%	7.6%	11.8%
tr|I3LQ83|I3LQ83	5	3	2	0	1	0	2	3	11	7	5	0	2	0	18	29	8.4%	5.1%	4.2%	0.0%	1.0%	0.0%	3.7%	8.2%
tr|F1SCY4|F1SCY4	6	2	0	0	2	0	0	1	16	2	0	0	2	0	0	1	25.8%	9.3%	0.0%	0.0%	12.0%	0.0%	0.0%	6.3%
tr|I3LQ91|I3LQ91	2	3	0	0	1	1	0	0	6	12	0	0	1	1	0	0	4.2%	8.7%	0.0%	0.0%	4.5%	4.2%	0.0%	0.0%
tr|F1RIW4|F1RIW4	2	4	0	0	2	0	0	0	4	14	0	0	2	0	0	0	1.6%	3.0%	0.0%	0.0%	1.6%	0.0%	0.0%	0.0%
tr|I3LLW7|I3LLW7	5	4	0	1	0	1	0	0	9	9	0	1	0	1	0	0	12.4%	5.7%	0.0%	2.5%	0.0%	2.5%	0.0%	0.0%
tr|F1SV40|F1SV40	7	2	4	1	1	0	0	1	16	2	26	1	1	0	0	1	19.6%	9.2%	7.8%	3.0%	3.0%	0.0%	0.0%	2.6%
tr|I3LKL5|I3LKL5	3	1	0	0	0	1	1	4	8	1	0	0	0	1	4	12	7.7%	3.1%	0.0%	0.0%	0.0%	3.1%	2.3%	8.3%
tr|F1ST81|F1ST81	4	1	2	0	1	0	2	1	8	1	22	0	1	0	8	5	15.1%	3.4%	8.3%	0.0%	3.4%	0.0%	8.0%	2.3%
tr|I3LDG9|I3LDG9	4	0	2	0	1	0	1	1	9	0	12	0	1	0	7	1	9.5%	0.0%	5.2%	0.0%	3.4%	0.0%	3.6%	3.6%
tr|F1RZZ1|F1RZZ1	4	2	2	0	1	0	1	0	6	3	11	0	1	0	5	0	3.8%	2.7%	3.5%	0.0%	1.7%	0.0%	2.9%	0.0%
tr|F1RRF2|F1RRF2	6	0	1	0	1	0	0	1	9	0	2	0	1	0	0	1	7.2%	0.0%	2.8%	0.0%	1.7%	0.0%	0.0%	1.7%
tr|I3LAZ9|I3LAZ9	2	3	0	0	1	0	0	0	2	7	0	0	1	0	0	0	4.3%	3.7%	0.0%	0.0%	1.0%	0.0%	0.0%	0.0%
tr|B7UCT2|B7UCT2	4	0	0	0	1	0	0	0	9	0	0	0	1	0	0	0	9.2%	0.0%	0.0%	0.0%	2.3%	0.0%	0.0%	0.0%
tr|F1SQ36|F1SQ36	3	2	0	0	1	0	0	0	5	4	0	0	1	0	0	0	3.7%	1.6%	0.0%	0.0%	0.8%	0.0%	0.0%	0.0%
tr|F1RSL1|F1RSL1	2	3	0	0	1	0	0	0	2	7	0	0	1	0	0	0	4.1%	3.5%	0.0%	0.0%	0.9%	0.0%	0.0%	0.0%
tr|I3LLA3|I3LLA3	4	2	0	0	0	1	0	0	5	4	0	0	0	1	0	0	5.3%	5.3%	0.0%	0.0%	0.0%	2.6%	0.0%	0.0%
tr|I3LJD8|I3LJD8	2	1	1	0	0	1	0	0	2	7	3	0	0	1	0	0	10.8%	3.0%	2.8%	0.0%	0.0%	3.0%	0.0%	0.0%
tr|I3LHC8|I3LHC8	2	1	1	0	1	0	0	0	5	4	5	0	1	0	0	0	11.8%	5.3%	6.9%	0.0%	5.3%	0.0%	0.0%	0.0%

	Peptide Count								Spectral Count								Sequence Coverage							
	Whole Cell	Whole Cell	Whole Cell	Cilia Fraction	Cilia Fraction	Cilia Fraction	Cilia Fraction	Cilia Fraction	Whole Cell	Whole Cell	Whole Cell	Cilia Fraction	Cilia Fraction	Cilia Fraction	Cilia Fraction	Cilia Fraction	Whole Cell	Whole Cell	Whole Cell	Cilia Fraction	Cilia Fraction	Cilia Fraction	Cilia Fraction	Cilia Fraction
Sample#	1	2	6	4	3	5	7	8	1	2	6	4	3	5	7	8	1	2	6	4	3	5	7	8
**Accession**																								
tr|F1S4D8|F1S4D8	5	0	1	0	1	0	0	0	9	0	7	0	1	0	0	0	12.4%	0.0%	2.0%	0.0%	2.9%	0.0%	0.0%	0.0%
tr|F1SV18|F1SV18	3	1	2	1	0	0	0	0	5	4	18	1	0	0	0	0	11.1%	1.8%	7.4%	3.9%	0.0%	0.0%	0.0%	0.0%
tr|F1S086|F1S086	3	2	4	0	0	1	0	0	4	5	33	0	0	1	0	0	11.4%	5.1%	11.1%	0.0%	0.0%	2.9%	0.0%	0.0%
tr|I3LJ87|I3LJ87	10	10	3	2	2	2	0	2	36	55	52	2	5	3	0	7	42.4%	47.8%	12.5%	15.2%	15.2%	14.7%	0.0%	17.9%
tr|F1S0A2|F1S0A2	11	7	4	1	1	1	1	3	25	21	21	3	1	1	1	31	34.7%	36.4%	22.7%	4.5%	7.4%	9.1%	8.0%	15.9%
tr|I3LS54|I3LS54	11	6	16	1	1	2	1	3	43	31	92	1	3	4	11	13	25.8%	15.1%	19.4%	2.2%	2.2%	3.5%	2.2%	9.4%
tr|F1RG16|F1RG16	12	7	5	0	5	1	7	12	37	28	44	0	6	1	141	104	38.4%	22.9%	18.1%	0.0%	15.2%	4.1%	26.3%	42.3%
tr|F2Z554|F2Z554	8	9	1	1	2	2	0	2	28	37	24	1	3	3	0	23	51.3%	60.9%	10.4%	10.4%	24.3%	11.3%	0.0%	27.8%
tr|F1S0M2|F1S0M2	6	5	6	1	2	0	2	3	11	17	89	1	2	0	19	29	56.2%	47.4%	56.2%	19.7%	15.3%	0.0%	28.5%	48.2%
P82460	9	7	5	2	3	2	2	1	151	1336	23	65	51	43	172	101	78.1%	57.1%	40.0%	26.7%	35.2%	25.7%	24.8%	14.3%
tr|F1SEM2|F1SEM2	10	2	3	1	3	0	1	5	45	2	20	1	4	0	12	29	50.2%	22.2%	15.4%	10.0%	34.4%	0.0%	5.4%	44.8%
tr|I3L630|I3L630	8	3	2	1	2	0	0	0	21	26	37	1	4	0	0	0	84.8%	33.3%	19.0%	11.4%	21.9%	0.0%	0.0%	0.0%
tr|I3LKF3|I3LKF3	27	18	19	2	6	3	11	15	133	253	213	16	22	3	282	223	51.0%	44.4%	31.0%	7.3%	20.2%	9.2%	35.2%	42.7%
tr|F1RFN9|F1RFN9	27	18	19	2	6	3	11	15	133	253	213	16	22	3	282	223	49.7%	43.2%	30.2%	7.1%	19.7%	8.9%	34.3%	41.6%
O19069	9	7	2	1	2	1	1	2	29	37	4	2	2	3	2	6	24.0%	26.6%	6.9%	8.1%	8.1%	8.1%	4.6%	8.1%
tr|I3LB44|I3LB44	21	13	9	2	3	1	1	2	94	104	221	8	11	2	20	3	63.8%	56.7%	32.8%	8.4%	14.2%	5.9%	5.9%	6.8%
tr|F1S3H7|F1S3H7	10	5	5	1	3	1	3	3	28	29	20	1	4	1	15	28	34.9%	21.6%	21.9%	4.0%	15.9%	7.7%	15.6%	15.6%
tr|F1SQX2|F1SQX2	14	1	3	0	3	0	1	5	36	2	10	0	4	0	35	40	24.4%	1.3%	6.4%	0.0%	2.5%	0.0%	1.9%	12.3%
tr|F1SGC5|F1SGC5	2	3	0	0	1	1	0	1	4	15	0	0	1	1	0	1	2.5%	2.5%	0.0%	0.0%	2.5%	2.5%	0.0%	2.5%
tr|K7GPM3|K7GPM3	1	3	0	0	1	0	0	0	5	14	0	0	2	0	0	0	5.9%	13.2%	0.0%	0.0%	5.9%	0.0%	0.0%	0.0%
tr|I3LIJ5|I3LIJ5	5	2	3	0	0	2	0	0	14	5	13	0	0	2	0	0	35.2%	18.4%	22.4%	0.0%	0.0%	10.4%	0.0%	0.0%
tr|F1SR72|F1SR72	8	4	2	1	0	0	0	0	12	7	15	2	0	0	0	0	15.4%	8.2%	4.6%	1.4%	0.0%	0.0%	0.0%	0.0%
tr|F1SP56|F1SP56	5	3	2	0	0	1	0	0	11	8	16	0	0	2	0	0	32.4%	16.2%	13.4%	0.0%	0.0%	5.7%	0.0%	0.0%
P11607	13	9	9	1	2	1	4	3	22	26	40	1	2	2	7	4	17.8%	8.6%	12.7%	1.5%	2.8%	1.3%	8.4%	5.7%
Q29195	10	7	7	0	4	2	1	4	29	48	234	0	6	2	11	24	33.6%	32.7%	26.6%	0.0%	26.6%	12.1%	12.6%	18.2%
tr|A8U4R4|A8U4R4	31	19	21	4	3	1	12	22	175	114	329	7	17	6	268	208	54.3%	40.9%	28.9%	8.3%	7.9%	2.2%	26.5%	38.2%
tr|I3LL07|I3LL07	6	3	3	0	1	1	1	3	13	16	29	0	2	1	1	10	20.2%	5.1%	9.2%	0.0%	2.5%	2.5%	3.7%	8.7%
tr|I3LS12|I3LS12	13	1	7	0	1	0	0	2	28	1	34	0	3	0	0	4	31.1%	2.7%	17.3%	0.0%	4.8%	0.0%	0.0%	7.5%
tr|I3LBV1|I3LBV1	28	20	8	5	10	4	6	8	283	206	228	8	31	11	75	107	63.9%	44.3%	22.2%	14.8%	32.6%	6.8%	24.1%	20.4%
tr|F1S0N2|F1S0N2	12	6	4	1	2	0	1	0	23	75	12	2	8	0	8	0	29.4%	13.2%	9.5%	2.7%	4.8%	0.0%	2.7%	0.0%
Q04967	12	5	10	1	2	0	3	3	28	21	70	3	2	0	21	5	15.4%	9.6%	15.4%	2.0%	5.8%	0.0%	5.6%	5.6%
tr|F2Z557|F2Z557	24	16	9	2	5	1	3	8	99	107	52	6	14	1	15	33	35.5%	28.1%	18.4%	2.5%	4.7%	2.2%	8.0%	15.7%
tr|I3LS00|I3LS00	24	16	9	2	5	1	3	8	99	107	52	6	14	1	15	33	34.9%	27.7%	18.1%	2.5%	4.6%	2.2%	7.9%	15.5%
tr|F1S2G5|F1S2G5	10	8	1	0	2	1	3	1	21	38	4	0	3	3	13	2	38.1%	12.7%	3.3%	0.0%	7.7%	4.4%	16.3%	3.3%
tr|F1RS36|F1RS36	54	34	42	8	15	9	10	20	328	322	534	20	29	17	139	199	59.3%	46.1%	43.2%	17.2%	27.8%	13.7%	19.6%	34.4%
tr|F1S8Y5|F1S8Y5	27	19	18	0	7	6	6	17	198	277	184	0	15	33	152	207	78.3%	57.8%	41.9%	0.0%	31.4%	19.0%	37.2%	62.0%
Q29387	19	12	8	1	3	3	3	4	64	36	106	1	6	3	71	22	54.6%	36.1%	19.9%	3.0%	10.6%	6.5%	13.7%	17.1%
tr|F1RPW9|F1RPW9	19	12	8	1	3	3	3	4	64	36	106	1	6	3	71	22	53.8%	35.5%	19.6%	3.0%	10.5%	6.4%	13.4%	16.9%
tr|F2Z5K2|F2Z5K2	12	6	3	1	1	0	2	11	41	49	26	4	5	0	19	27	52.3%	32.4%	13.3%	5.0%	5.0%	0.0%	12.9%	40.2%
tr|F2Z5F7|F2Z5F7	5	2	0	0	2	2	2	1	41	29	0	0	2	5	13	9	47.5%	20.2%	0.0%	0.0%	20.2%	20.2%	20.2%	20.2%
tr|F1RJH2|F1RJH2	7	3	2	1	1	2	0	0	30	30	3	1	1	4	0	0	31.1%	6.3%	11.7%	5.3%	6.1%	6.3%	0.0%	0.0%
tr|F1S4M2|F1S4M2	4	3	3	0	2	0	1	2	9	21	28	0	3	0	1	13	23.0%	15.1%	18.4%	0.0%	13.2%	0.0%	5.3%	9.9%
tr|F1RM03|F1RM03	8	2	5	1	1	0	2	1	15	15	42	1	2	0	5	4	34.1%	11.3%	24.9%	3.8%	3.8%	0.0%	13.0%	6.4%
tr|I3LL97|I3LL97	8	4	7	0	1	0	4	3	14	6	49	0	2	0	35	14	50.3%	28.0%	34.2%	0.0%	7.8%	0.0%	36.8%	25.4%
tr|F1RY04|F1RY04	9	2	3	0	1	0	0	0	18	2	4	0	2	0	0	0	17.2%	3.7%	4.9%	0.0%	2.5%	0.0%	0.0%	0.0%
tr|F1SQM6|F1SQM6	7	1	3	0	2	0	1	0	18	2	35	0	2	0	1	0	61.9%	12.4%	41.9%	0.0%	32.4%	0.0%	11.4%	0.0%
tr|K7GLZ1|K7GLZ1	3	0	3	0	0	1	4	11	10	0	15	0	0	1	32	102	14.1%	0.0%	18.9%	0.0%	0.0%	8.6%	27.6%	44.3%
tr|F1SL63|F1SL63	2	2	2	0	1	0	0	6	3	7	7	0	1	0	0	12	2.0%	0.8%	2.3%	0.0%	0.8%	0.0%	0.0%	6.5%
tr|K7GS03|K7GS03	2	2	3	0	1	0	0	5	3	7	8	0	1	0	0	11	2.2%	0.9%	3.9%	0.0%	0.9%	0.0%	0.0%	6.6%
tr|F1SME6|F1SME6	7	0	3	0	1	0	3	0	10	0	10	0	1	0	10	0	26.2%	0.0%	10.1%	0.0%	6.7%	0.0%	15.7%	0.0%
tr|I3LG92|I3LG92	2	2	3	0	1	0	0	3	3	7	8	0	1	0	0	8	4.4%	1.7%	7.8%	0.0%	1.7%	0.0%	0.0%	4.8%
tr|I3LTF9|I3LTF9	2	3	0	1	0	0	0	1	2	8	0	1	0	0	0	3	8.9%	7.2%	0.0%	2.8%	0.0%	0.0%	0.0%	4.7%
tr|F1SKH6|F1SKH6	2	3	0	1	0	0	0	1	2	8	0	1	0	0	0	3	12.7%	10.4%	0.0%	4.0%	0.0%	0.0%	0.0%	6.8%
tr|I3LBK6|I3LBK6	5	0	2	0	1	0	1	2	10	0	12	0	1	0	1	5	34.2%	0.0%	13.8%	0.0%	13.3%	0.0%	7.1%	13.3%
tr|F1S590|F1S590	6	0	0	0	1	0	0	1	10	0	0	0	1	0	0	2	15.6%	0.0%	0.0%	0.0%	3.6%	0.0%	0.0%	3.0%
tr|F1RSY4|F1RSY4	4	2	0	0	1	0	0	0	6	4	0	0	1	0	0	0	13.7%	6.4%	0.0%	0.0%	3.3%	0.0%	0.0%	0.0%
tr|F1SIF8|F1SIF8	5	1	0	0	1	0	0	0	7	3	0	0	1	0	0	0	6.0%	0.8%	0.0%	0.0%	0.8%	0.0%	0.0%	0.0%
tr|I3LKF2|I3LKF2	5	2	0	0	0	1	0	0	6	4	0	0	0	1	0	0	2.0%	1.3%	0.0%	0.0%	0.0%	0.6%	0.0%	0.0%
tr|I3L667|I3L667	1	1	0	0	1	0	0	0	2	8	0	0	1	0	0	0	3.5%	2.0%	0.0%	0.0%	3.0%	0.0%	0.0%	0.0%
tr|K7GP62|K7GP62	2	1	2	0	1	0	0	0	5	5	24	0	1	0	0	0	6.3%	6.3%	12.7%	0.0%	6.3%	0.0%	0.0%	0.0%
tr|F1RLG5|F1RLG5	4	3	3	0	1	0	0	0	6	4	38	0	1	0	0	0	14.3%	10.6%	13.8%	0.0%	6.9%	0.0%	0.0%	0.0%
tr|F1RUN2|F1RUN2	9	5	9	1	2	2	4	7	52	29	71	1	5	2	254	131	9.4%	5.8%	11.2%	2.1%	4.1%	4.1%	8.1%	10.0%
P08835	9	5	9	1	2	2	4	7	52	29	71	1	5	2	254	131	9.4%	5.8%	11.2%	2.1%	4.1%	4.1%	8.1%	10.0%
tr|K7GSL7|K7GSL7	12	6	4	1	2	0	1	4	36	45	29	3	5	0	4	31	42.6%	25.2%	24.8%	5.4%	9.4%	0.0%	4.0%	31.2%
Q64L94	21	10	12	1	3	2	5	8	69	73	65	4	6	4	34	35	64.7%	40.6%	42.6%	4.8%	16.9%	15.7%	22.5%	37.3%
tr|F1SGM1|F1SGM1	21	10	12	1	3	2	5	8	69	73	65	4	6	4	34	35	64.7%	40.6%	42.6%	4.8%	16.9%	15.7%	22.5%	37.3%
tr|F1S9Z1|F1S9Z1	22	10	17	4	3	0	1	8	132	41	498	9	8	0	1	127	41.3%	40.1%	48.0%	19.0%	12.7%	0.0%	5.6%	31.7%
tr|F1S6M7|F1S6M7	33	25	14	6	13	4	9	18	337	264	1017	7	40	12	269	354	47.1%	53.3%	44.7%	18.0%	30.4%	4.9%	27.8%	37.1%
tr|K7GLA0|K7GLA0	12	7	3	1	3	0	0	3	37	55	23	3	6	0	0	29	37.1%	26.7%	19.0%	5.0%	12.2%	0.0%	0.0%	24.9%
tr|F1ST73|F1ST73	8	2	0	1	1	0	0	4	20	11	0	2	1	0	0	10	36.2%	7.3%	0.0%	2.7%	2.7%	0.0%	0.0%	29.2%
tr|D0G0C8|D0G0C8	36	21	18	1	3	1	8	13	158	101	258	8	13	4	64	80	63.7%	45.6%	36.3%	2.8%	9.2%	2.8%	22.2%	37.6%
tr|Q2EHH8|Q2EHH8	19	7	4	0	2	1	5	10	50	33	32	0	7	1	83	39	43.9%	19.4%	16.4%	0.0%	7.6%	4.2%	22.4%	29.1%
tr|F1SHC1|F1SHC1	43	31	13	8	15	7	11	31	664	458	947	31	62	15	965	548	79.3%	66.8%	37.4%	22.7%	44.5%	14.3%	31.8%	63.5%
tr|I3LUR5|I3LUR5	5	5	1	1	1	1	0	0	6	46	3	2	1	2	0	0	13.0%	13.7%	3.0%	3.9%	3.9%	3.9%	0.0%	0.0%
P62936	24	15	13	2	9	4	6	7	253	143	391	9	23	6	241	124	78.7%	75.0%	47.0%	19.5%	54.9%	35.4%	39.0%	39.6%
tr|F1SUF6|F1SUF6	15	7	7	1	3	0	1	1	38	25	21	1	5	0	1	1	25.3%	6.8%	9.3%	1.4%	5.2%	0.0%	1.9%	2.0%
tr|F2Z5N8|F2Z5N8	14	2	2	1	1	1	2	6	32	10	6	2	1	1	12	26	58.6%	14.4%	13.5%	7.0%	4.7%	7.0%	14.4%	32.6%
Q52NJ6	14	2	2	1	1	1	2	6	32	10	6	2	1	1	12	26	58.6%	14.4%	13.5%	7.0%	4.7%	7.0%	14.4%	32.6%
tr|I3L5C7|I3L5C7	10	2	6	0	1	0	1	4	18	3	15	0	2	0	4	56	20.2%	6.5%	17.8%	0.0%	1.8%	0.0%	2.5%	14.3%
tr|D9U8D1|D9U8D1	9	2	0	0	1	0	0	2	16	5	0	0	2	0	0	28	51.7%	13.5%	0.0%	0.0%	12.4%	0.0%	0.0%	21.3%
tr|I3LNE5|I3LNE5	2	1	2	0	0	1	2	1	2	19	11	0	0	2	3	2	4.6%	2.2%	3.5%	0.0%	0.0%	2.2%	5.8%	2.2%

	Peptide Count								Spectral Count								Sequence Coverage							
	Whole Cell	Whole Cell	Whole Cell	Cilia Fraction	Cilia Fraction	Cilia Fraction	Cilia Fraction	Cilia Fraction	Whole Cell	Whole Cell	Whole Cell	Cilia Fraction	Cilia Fraction	Cilia Fraction	Cilia Fraction	Cilia Fraction	Whole Cell	Whole Cell	Whole Cell	Cilia Fraction	Cilia Fraction	Cilia Fraction	Cilia Fraction	Cilia Fraction
Sample#	1	2	6	4	3	5	7	8	1	2	6	4	3	5	7	8	1	2	6	4	3	5	7	8
**Accession**																								
tr|I3LC48|I3LC48	2	3	2	0	1	1	0	0	5	16	23	0	1	1	0	0	7.8%	7.8%	7.8%	0.0%	5.1%	5.1%	0.0%	0.0%
A5GFY8	18	11	14	2	5	2	7	9	65	51	98	2	7	2	111	102	49.3%	28.7%	28.5%	1.7%	7.5%	3.8%	19.5%	26.8%
tr|I3LQ79|I3LQ79	19	3	13	1	0	1	7	18	47	6	431	1	0	4	31	131	28.2%	4.9%	20.0%	1.7%	0.0%	2.8%	13.8%	33.6%
tr|F1SUZ5|F1SUZ5	16	10	3	0	5	0	1	0	43	42	12	0	8	0	1	0	21.2%	18.1%	9.6%	0.0%	8.5%	0.0%	2.7%	0.0%
tr|F1SAD9|F1SAD9	26	7	19	0	1	2	4	8	56	61	385	0	1	10	32	38	36.2%	7.7%	28.8%	0.0%	1.4%	3.1%	11.9%	24.0%
tr|F2Z5G8|F2Z5G8	6	6	3	0	1	1	1	1	18	14	23	0	1	2	7	10	24.0%	25.6%	24.0%	0.0%	7.2%	10.4%	8.8%	8.8%
tr|F1RQ91|F1RQ91	13	12	7	1	3	2	1	1	30	56	63	1	4	3	1	1	41.1%	46.4%	24.3%	3.4%	15.6%	14.4%	4.9%	4.9%
tr|F1SS99|F1SS99	5	2	1	0	2	2	1	0	11	43	9	0	2	3	2	0	20.5%	3.8%	4.6%	0.0%	7.3%	3.8%	3.8%	0.0%
tr|F1S431|F1S431	19	5	7	0	5	2	7	3	45	53	32	0	7	2	34	7	30.0%	6.7%	11.7%	0.0%	4.8%	1.6%	10.9%	7.5%
tr|D0G7F6|D0G7F6	31	22	17	5	10	1	4	14	162	143	710	11	16	1	202	165	85.5%	83.9%	73.0%	35.1%	53.2%	5.2%	30.6%	69.8%
P00346	28	15	15	2	3	1	1	3	114	115	282	8	11	2	20	5	72.8%	62.7%	43.2%	8.0%	13.6%	5.6%	5.6%	10.1%
tr|I3LP41|I3LP41	28	15	15	2	3	1	1	3	114	115	282	8	11	2	20	5	72.8%	62.7%	43.2%	8.0%	13.6%	5.6%	5.6%	10.1%
tr|F1S9Y8|F1S9Y8	23	7	3	0	3	1	0	3	41	25	14	0	4	2	0	6	18.3%	6.5%	2.8%	0.0%	2.4%	1.1%	0.0%	4.9%
tr|F2Z5F1|F2Z5F1	1	1	0	1	1	0	0	0	1	43	0	1	3	0	0	0	9.6%	8.8%	0.0%	8.8%	8.8%	0.0%	0.0%	0.0%
tr|I3LU04|I3LU04	7	2	4	1	1	0	1	1	17	5	25	1	1	0	24	12	32.0%	7.7%	16.0%	3.7%	3.7%	0.0%	7.7%	7.7%
tr|F1S0C1|F1S0C1	8	3	3	1	1	0	0	6	14	8	21	1	1	0	0	11	34.0%	11.8%	8.6%	6.4%	6.4%	0.0%	0.0%	27.0%
A1XQU1	6	5	2	0	0	2	0	0	7	15	9	0	0	2	0	0	38.3%	5.4%	9.0%	0.0%	0.0%	5.1%	0.0%	0.0%
tr|F1RN21|F1RN21	4	1	5	0	1	0	5	5	10	1	38	0	1	0	298	124	6.2%	3.5%	6.2%	0.0%	2.4%	0.0%	6.2%	6.2%
tr|I3LN55|I3LN55	4	1	5	0	1	0	5	5	10	1	38	0	1	0	298	124	3.0%	1.7%	3.0%	0.0%	1.2%	0.0%	3.0%	3.0%
tr|I3L5F7|I3L5F7	1	1	0	0	1	0	0	5	1	10	0	0	1	0	0	21	3.1%	1.2%	0.0%	0.0%	1.2%	0.0%	0.0%	8.8%
tr|F1SG93|F1SG93	1	1	1	0	1	0	0	5	1	10	2	0	1	0	0	21	3.0%	1.2%	1.5%	0.0%	1.2%	0.0%	0.0%	8.6%
tr|I3LRZ4|I3LRZ4	1	1	1	0	1	0	0	5	1	10	2	0	1	0	0	21	3.2%	1.2%	1.6%	0.0%	1.2%	0.0%	0.0%	9.0%
tr|F1SRI1|F1SRI1	5	2	0	0	1	0	0	2	8	3	0	0	1	0	0	12	20.9%	10.4%	0.0%	0.0%	8.0%	0.0%	0.0%	10.9%
tr|I3L916|I3L916	3	3	3	0	1	0	0	4	3	8	19	0	1	0	0	20	4.8%	6.2%	7.7%	0.0%	2.7%	0.0%	0.0%	10.4%
tr|F1SKY2|F1SKY2	3	2	4	0	0	1	1	1	4	7	22	0	0	1	5	2	18.4%	7.8%	26.9%	0.0%	0.0%	4.2%	12.4%	4.2%
tr|I3LSD1|I3LSD1	6	1	1	1	0	0	0	1	10	1	11	1	0	0	0	3	9.7%	1.7%	3.1%	1.7%	0.0%	0.0%	0.0%	4.9%
tr|I3LR20|I3LR20	1	5	1	0	0	1	1	0	3	8	6	0	0	1	1	0	1.0%	4.4%	1.6%	0.0%	0.0%	1.0%	1.0%	0.0%
tr|F1RLP9|F1RLP9	7	1	1	0	1	0	0	1	10	1	8	0	1	0	0	1	16.3%	1.7%	1.7%	0.0%	1.0%	0.0%	0.0%	2.5%
tr|F1SKD4|F1SKD4	2	1	0	0	1	0	0	0	2	9	0	0	1	0	0	0	2.1%	0.6%	0.0%	0.0%	0.6%	0.0%	0.0%	0.0%
tr|I3LLT3|I3LLT3	4	0	0	0	1	0	0	0	11	0	0	0	1	0	0	0	19.8%	0.0%	0.0%	0.0%	7.3%	0.0%	0.0%	0.0%
tr|F1RPJ8|F1RPJ8	2	2	0	0	1	0	0	0	2	9	0	0	1	0	0	0	6.2%	3.5%	0.0%	0.0%	1.9%	0.0%	0.0%	0.0%
tr|K7GM11|K7GM11	2	2	0	0	1	0	0	0	2	9	0	0	1	0	0	0	6.7%	3.7%	0.0%	0.0%	2.0%	0.0%	0.0%	0.0%
tr|F1S0B2|F1S0B2	1	1	0	0	0	1	0	0	4	7	0	0	0	1	0	0	5.1%	5.1%	0.0%	0.0%	0.0%	5.1%	0.0%	0.0%
tr|F1SMJ3|F1SMJ3	4	1	1	0	0	1	0	0	4	7	3	0	0	1	0	0	13.9%	2.1%	1.9%	0.0%	0.0%	2.1%	0.0%	0.0%
tr|F1S7C4|F1S7C4	3	1	2	0	1	0	0	0	6	5	3	0	1	0	0	0	4.8%	1.0%	2.2%	0.0%	1.0%	0.0%	0.0%	0.0%
tr|F2Z5Q7|F2Z5Q7	5	1	1	0	0	1	0	0	5	6	4	0	0	1	0	0	30.2%	7.7%	7.1%	0.0%	0.0%	7.7%	0.0%	0.0%
tr|F1S1B9|F1S1B9	4	2	1	1	0	0	0	0	5	6	5	1	0	0	0	0	30.6%	12.2%	6.7%	6.7%	0.0%	0.0%	0.0%	0.0%
tr|F1SBI1|F1SBI1	6	1	2	0	1	0	0	0	7	4	9	0	1	0	0	0	25.4%	3.4%	6.3%	0.0%	3.4%	0.0%	0.0%	0.0%
tr|F1S8H5|F1S8H5	5	0	5	0	1	0	1	0	11	0	56	0	1	0	2	0	27.8%	0.0%	21.5%	0.0%	5.7%	0.0%	4.7%	0.0%
tr|K7GQ06|K7GQ06	4	1	4	1	0	0	0	0	6	5	13	1	0	0	0	0	23.3%	4.9%	19.9%	4.9%	0.0%	0.0%	0.0%	0.0%
tr|I3LII3|I3LII3	73	48	26	6	18	11	17	26	635	605	214	17	68	27	233	315	67.1%	50.1%	27.9%	10.1%	23.3%	11.5%	27.2%	39.2%
tr|F1RYI3|F1RYI3	27	13	9	3	3	1	3	8	61	72	74	6	5	1	18	18	32.0%	15.4%	8.1%	3.1%	3.0%	1.1%	5.7%	9.9%
tr|F1SQN1|F1SQN1	24	10	11	1	2	1	6	10	60	63	260	1	6	4	23	24	51.8%	27.1%	26.0%	3.5%	5.8%	2.2%	17.8%	28.8%
tr|F1SL99|F1SL99	7	4	5	0	1	0	1	3	23	22	12	0	4	0	1	24	27.5%	23.1%	26.2%	0.0%	7.5%	0.0%	7.5%	27.5%
tr|F1RUV5|F1RUV5	18	1	3	0	1	1	0	0	33	1	29	0	2	1	0	0	36.0%	1.8%	5.7%	0.0%	2.1%	2.2%	0.0%	0.0%
tr|F1SGR0|F1SGR0	9	5	0	0	3	0	0	3	33	24	0	0	5	0	0	6	30.7%	14.2%	0.0%	0.0%	8.6%	0.0%	0.0%	13.1%
tr|F1RSE7|F1RSE7	8	9	1	0	1	1	0	0	11	46	11	0	4	1	0	0	13.9%	8.6%	2.9%	0.0%	1.6%	1.6%	0.0%	0.0%
tr|F1RX74|F1RX74	12	5	5	1	2	1	1	6	56	24	37	2	4	1	50	26	25.8%	13.0%	10.8%	4.5%	8.8%	4.5%	4.5%	15.9%
Q7M2W6	22	9	13	3	6	2	4	13	435	148	342	13	34	4	481	513	71.4%	46.3%	56.6%	23.4%	38.9%	23.4%	25.7%	47.4%
tr|I3LJE2|I3LJE2	24	13	18	1	3	2	8	17	50	76	153	1	6	4	53	70	45.5%	26.6%	28.8%	2.4%	5.8%	1.5%	16.5%	36.9%
tr|F1RRK9|F1RRK9	31	20	17	2	8	3	3	4	107	260	84	4	22	6	5	9	32.9%	14.6%	17.0%	2.1%	10.7%	2.1%	3.5%	8.6%
tr|B8XH67|B8XH67	10	5	8	0	1	0	3	16	11	12	122	0	2	0	68	174	40.0%	19.2%	30.4%	0.0%	7.4%	0.0%	20.0%	52.3%
tr|I3LPP4|I3LPP4	8	3	2	0	2	0	2	0	16	7	14	0	2	0	6	0	21.9%	6.4%	5.6%	0.0%	4.4%	0.0%	4.6%	0.0%
tr|I3L5C0|I3L5C0	1	5	0	0	0	2	0	0	3	20	0	0	0	2	0	0	11.4%	21.0%	0.0%	0.0%	0.0%	9.7%	0.0%	0.0%
tr|I3LGQ9|I3LGQ9	0	2	3	0	1	1	0	0	0	23	10	0	1	1	0	0	0.0%	5.8%	13.7%	0.0%	3.0%	3.0%	0.0%	0.0%
Q29371	25	18	14	3	8	1	3	9	140	125	505	9	13	1	186	105	62.5%	60.9%	50.0%	16.9%	41.9%	5.2%	19.0%	46.4%
tr|I3L5B3|I3L5B3	33	8	42	0	2	3	12	11	58	23	652	0	3	4	91	51	24.2%	5.7%	31.8%	0.0%	2.3%	2.6%	12.2%	11.2%
tr|F1RVY7|F1RVY7	13	2	6	1	1	0	0	3	27	8	12	1	2	0	0	6	38.1%	5.0%	16.4%	2.6%	2.6%	0.0%	0.0%	6.9%
Q29197	6	8	7	0	2	1	0	1	12	23	31	0	2	1	0	4	26.9%	38.5%	35.4%	0.0%	13.1%	7.7%	0.0%	7.7%
tr|I3L6D0|I3L6D0	17	3	7	1	1	0	0	0	26	9	60	1	2	0	0	0	19.9%	2.7%	6.2%	1.5%	1.4%	0.0%	0.0%	0.0%
tr|F1SEF7|F1SEF7	20	4	7	1	1	0	5	15	51	31	55	1	6	0	70	50	46.8%	12.5%	20.5%	4.2%	4.2%	0.0%	23.7%	54.5%
P61292	20	4	7	1	1	0	5	15	51	31	55	1	6	0	70	50	44.6%	11.9%	19.6%	4.0%	4.0%	0.0%	22.6%	52.0%
tr|I3LCN1|I3LCN1	7	9	1	2	4	2	3	4	345	1977	40	81	54	63	408	334	13.1%	13.1%	4.1%	3.5%	8.5%	3.9%	12.7%	12.7%
Q2YGT9	22	8	11	2	3	2	3	5	82	59	79	2	7	3	40	30	48.2%	25.0%	22.9%	8.8%	15.8%	9.9%	13.7%	20.8%
Q0QF01	14	8	20	1	1	2	3	6	59	35	163	1	3	4	19	29	34.0%	23.2%	28.6%	2.1%	2.1%	3.3%	11.6%	22.9%
P50578	10	6	6	0	3	0	0	0	34	13	34	0	4	0	0	0	22.2%	19.4%	18.2%	0.0%	10.2%	0.0%	0.0%	0.0%
tr|I3LSD3|I3LSD3	10	9	3	0	1	2	1	1	24	82	9	0	2	7	1	2	30.8%	29.9%	14.7%	0.0%	5.2%	10.4%	6.2%	5.2%
Q6Q7J2	29	13	13	1	6	3	7	14	148	42	96	1	12	3	66	59	46.5%	36.0%	35.3%	5.4%	19.1%	10.3%	21.1%	37.1%
tr|F2Z5R6|F2Z5R6	28	13	9	3	3	1	4	8	71	72	74	6	5	1	26	18	33.6%	15.4%	8.1%	3.1%	3.0%	1.1%	7.3%	9.9%
Q1KYT0	14	14	6	3	6	3	7	8	396	2049	118	83	58	64	668	381	26.0%	26.0%	13.6%	6.9%	15.2%	7.1%	22.6%	25.6%
tr|F1RFY2|F1RFY2	14	14	6	3	6	3	7	8	397	2049	118	83	58	64	668	381	26.0%	26.0%	13.6%	6.9%	15.2%	7.1%	22.6%	25.6%
tr|I3LAB6|I3LAB6	8	5	3	1	1	1	3	8	38	22	31	3	1	1	17	42	39.3%	30.3%	13.7%	6.0%	6.0%	6.0%	18.4%	34.2%
tr|I3LM93|I3LM93	15	7	5	0	4	0	2	2	30	66	23	0	8	0	3	3	24.2%	9.2%	8.0%	0.0%	5.3%	0.0%	5.2%	3.8%
tr|K7GLQ2|K7GLQ2	1	3	0	0	1	1	0	0	2	22	0	0	1	1	0	0	0.9%	2.6%	0.0%	0.0%	0.8%	0.8%	0.0%	0.0%
tr|K7GMM0|K7GMM0	1	3	0	0	1	1	0	0	2	22	0	0	1	1	0	0	0.9%	2.6%	0.0%	0.0%	0.9%	0.9%	0.0%	0.0%
tr|I3LTT5|I3LTT5	1	3	0	0	1	1	0	0	2	22	0	0	1	1	0	0	1.3%	3.6%	0.0%	0.0%	1.2%	1.2%	0.0%	0.0%
tr|F1RTI9|F1RTI9	1	3	0	0	1	1	0	0	2	22	0	0	1	1	0	0	0.9%	2.6%	0.0%	0.0%	0.8%	0.8%	0.0%	0.0%
tr|K7GNV1|K7GNV1	1	3	0	0	1	1	0	0	2	22	0	0	1	1	0	0	0.9%	2.6%	0.0%	0.0%	0.8%	0.8%	0.0%	0.0%
I3L5V6	1	2	3	0	1	1	0	0	1	23	10	0	1	1	0	0	3.6%	2.9%	6.9%	0.0%	1.5%	1.5%	0.0%	0.0%
Q9GKE8	3	0	0	0	0	1	1	6	12	0	0	0	0	1	10	77	12.5%	0.0%	0.0%	0.0%	0.0%	4.6%	4.6%	11.9%
tr|F1SL37|F1SL37	3	0	0	0	0	1	1	6	12	0	0	0	0	1	10	77	13.2%	0.0%	0.0%	0.0%	0.0%	4.9%	4.9%	12.5%

	Peptide Count								Spectral Count								Sequence Coverage							
	Whole Cell	Whole Cell	Whole Cell	Cilia Fraction	Cilia Fraction	Cilia Fraction	Cilia Fraction	Cilia Fraction	Whole Cell	Whole Cell	Whole Cell	Cilia Fraction	Cilia Fraction	Cilia Fraction	Cilia Fraction	Cilia Fraction	Whole Cell	Whole Cell	Whole Cell	Cilia Fraction	Cilia Fraction	Cilia Fraction	Cilia Fraction	Cilia Fraction
Sample#	1	2	6	4	3	5	7	8	1	2	6	4	3	5	7	8	1	2	6	4	3	5	7	8
**Accession**																								
tr|F1RWI8|F1RWI8	4	1	2	0	1	0	0	2	9	3	3	0	1	0	0	5	19.8%	3.3%	8.0%	0.0%	3.3%	0.0%	0.0%	4.2%
tr|F1RG35|F1RG35	8	1	1	0	1	0	0	1	10	2	1	0	1	0	0	4	34.7%	3.6%	5.0%	0.0%	3.6%	0.0%	0.0%	3.0%
tr|F1SIS9|F1SIS9	5	1	2	0	1	0	0	1	11	1	4	0	1	0	0	2	17.1%	2.5%	6.7%	0.0%	6.2%	0.0%	0.0%	7.0%
tr|F1S3I3|F1S3I3	6	1	6	0	0	1	1	3	8	4	30	0	0	1	2	3	27.3%	2.8%	22.4%	0.0%	0.0%	2.8%	4.0%	11.8%
tr|F1SR01|F1SR01	5	2	0	0	1	0	0	0	6	6	0	0	1	0	0	0	8.9%	4.0%	0.0%	0.0%	2.8%	0.0%	0.0%	0.0%
tr|K7GMQ4|K7GMQ4	1	1	0	0	1	0	0	0	2	10	0	0	1	0	0	0	11.1%	11.1%	0.0%	0.0%	11.1%	0.0%	0.0%	0.0%
tr|I3LBL5|I3LBL5	2	3	0	0	1	0	0	0	8	4	0	0	1	0	0	0	34.1%	61.2%	0.0%	0.0%	34.1%	0.0%	0.0%	0.0%
tr|K7GL68|K7GL68	1	1	0	0	1	0	0	0	2	10	0	0	1	0	0	0	21.1%	21.1%	0.0%	0.0%	21.1%	0.0%	0.0%	0.0%
tr|F1SEX4|F1SEX4	0	1	0	0	1	0	0	0	0	12	0	0	1	0	0	0	0.0%	7.3%	0.0%	0.0%	7.3%	0.0%	0.0%	0.0%
Q6Q2C2	3	3	2	1	0	0	0	0	3	9	2	1	0	0	0	0	9.7%	6.3%	5.9%	3.8%	0.0%	0.0%	0.0%	0.0%
tr|I3LDY2|I3LDY2	5	3	1	0	1	0	0	0	7	5	3	0	1	0	0	0	30.3%	11.5%	7.0%	0.0%	4.2%	0.0%	0.0%	0.0%
tr|I3LC09|I3LC09	7	1	3	0	1	0	0	0	9	3	19	0	1	0	0	0	16.3%	2.2%	7.4%	0.0%	2.2%	0.0%	0.0%	0.0%
tr|F1SLX5|F1SLX5	1	2	6	0	1	0	0	0	1	11	23	0	1	0	0	0	2.7%	4.3%	6.9%	0.0%	1.8%	0.0%	0.0%	0.0%
tr|F1RQU2|F1RQU2	74	43	47	6	11	4	14	26	570	432	472	21	54	8	195	335	62.3%	52.1%	44.1%	10.2%	21.1%	8.7%	26.5%	37.4%
tr|I3LK24|I3LK24	19	6	8	1	2	1	4	7	28	33	31	1	3	1	34	25	27.3%	7.2%	9.9%	1.0%	3.7%	1.5%	9.7%	14.1%
tr|F1RH60|F1RH60	19	6	8	1	2	1	4	7	28	33	31	1	3	1	34	25	26.7%	7.1%	9.7%	1.0%	3.6%	1.4%	9.5%	13.8%
tr|F1RWI5|F1RWI5	15	12	7	1	5	1	2	3	91	80	80	2	10	2	8	12	33.9%	30.0%	24.1%	5.4%	21.8%	4.3%	11.7%	11.7%
tr|E1CAJ6|E1CAJ6	19	6	6	1	3	1	3	6	56	30	84	1	4	2	18	53	47.3%	18.6%	16.1%	3.4%	6.1%	2.5%	5.7%	27.3%
tr|F1RUK8|F1RUK8	33	13	19	1	6	3	7	17	155	42	136	1	12	3	66	74	58.2%	36.0%	44.3%	5.4%	19.1%	10.3%	21.1%	44.0%
tr|F1SGD7|F1SGD7	15	9	10	2	4	1	1	4	80	107	63	7	7	1	2	11	40.4%	35.8%	26.6%	10.2%	20.7%	2.8%	4.9%	19.7%
tr|F1RQI7|F1RQI7	34	20	24	1	7	3	10	12	74	101	138	1	8	5	78	59	25.7%	17.1%	20.9%	1.4%	9.0%	2.2%	13.2%	11.7%
tr|F1SF87|F1SF87	15	10	6	1	1	4	1	5	41	34	56	1	1	4	1	7	30.6%	21.1%	9.6%	3.2%	1.5%	9.9%	5.5%	13.4%
tr|I3LT81|I3LT81	8	2	3	0	0	2	1	2	21	4	15	0	0	2	2	7	37.7%	6.0%	8.2%	0.0%	0.0%	6.0%	8.7%	18.6%
tr|I3LI24|I3LI24	19	11	12	1	3	0	4	4	71	42	63	3	6	0	21	16	51.4%	48.6%	36.0%	2.8%	14.5%	0.0%	13.9%	11.4%
tr|F1SNY2|F1SNY2	24	17	8	0	6	5	2	7	100	114	372	0	7	10	18	100	42.2%	36.0%	22.1%	0.0%	11.7%	14.4%	8.4%	23.8%
Q29122	31	18	22	1	6	3	8	11	71	93	121	1	7	5	44	53	23.9%	15.8%	20.0%	1.4%	7.7%	2.2%	10.7%	10.8%
tr|I3L5B2|I3L5B2	12	5	5	0	1	2	2	9	70	31	82	0	1	7	7	58	53.6%	41.2%	17.5%	0.0%	6.2%	6.2%	19.1%	47.4%
P42639	19	3	24	1	1	0	2	7	34	4	232	2	1	0	19	31	38.4%	7.7%	37.0%	3.5%	3.5%	0.0%	4.6%	31.3%
tr|F1S2J4|F1S2J4	18	3	9	1	1	0	0	0	29	9	69	1	2	0	0	0	21.4%	2.9%	8.3%	1.6%	1.6%	0.0%	0.0%	0.0%
tr|I3LK59|I3LK59	47	23	33	4	5	2	10	17	533	2018	597	83	55	63	671	417	67.5%	46.9%	39.0%	10.7%	16.1%	4.8%	38.4%	47.2%
tr|I3LQS0|I3LQS0	23	13	18	1	6	1	0	7	79	137	358	1	15	1	0	56	45.3%	28.6%	35.6%	5.2%	14.6%	3.4%	0.0%	26.6%
Q19S50	18	3	7	0	2	0	3	4	35	16	22	0	4	0	32	23	34.5%	3.1%	14.8%	0.0%	2.3%	0.0%	7.9%	11.6%
tr|I3LLF2|I3LLF2	12	4	5	1	3	0	2	4	46	5	27	1	3	0	14	15	23.6%	12.2%	12.2%	3.9%	10.6%	0.0%	5.3%	12.8%
tr|F1RYY0|F1RYY0	12	8	6	0	2	0	3	4	26	25	54	0	4	0	25	9	28.9%	24.1%	12.7%	0.0%	9.9%	0.0%	11.0%	11.0%
tr|F1SLA0|F1SLA0	41	22	24	4	6	4	8	22	401	111	904	13	16	11	58	190	72.0%	53.8%	44.1%	9.7%	17.8%	10.0%	15.9%	47.7%
tr|F1S9Q1|F1S9Q1	47	26	27	3	13	4	8	19	308	166	686	5	26	6	266	244	47.1%	45.1%	35.3%	5.1%	32.2%	5.1%	13.8%	23.8%
tr|F1SHD7|F1SHD7	13	4	9	0	1	2	0	4	27	50	52	0	3	3	0	6	26.1%	7.7%	14.0%	0.0%	1.8%	6.5%	0.0%	8.9%
tr|F2Z558|F2Z558	36	18	25	4	10	3	8	16	348	65	433	12	15	5	126	221	75.8%	66.5%	67.3%	21.8%	41.5%	15.3%	26.2%	41.5%
tr|I3LFQ5|I3LFQ5	23	11	7	0	3	0	6	16	56	35	70	0	7	0	123	39	33.1%	16.3%	11.6%	0.0%	5.7%	0.0%	14.3%	29.6%
P10775	23	7	25	0	3	0	11	9	47	18	359	0	5	0	159	67	62.5%	22.8%	62.5%	0.0%	6.1%	0.0%	32.2%	25.2%
tr|F1RVN0|F1RVN0	13	4	7	0	3	0	1	6	54	11	100	0	5	0	3	26	61.7%	24.0%	29.1%	0.0%	20.4%	0.0%	8.2%	34.7%
P12026	9	8	8	0	3	1	2	7	44	34	83	0	4	2	36	64	73.6%	66.7%	63.2%	0.0%	32.2%	11.5%	20.7%	51.7%
tr|F1RYU9|F1RYU9	18	2	11	0	2	0	2	1	31	8	35	0	3	0	3	4	29.1%	2.6%	16.0%	0.0%	3.4%	0.0%	4.6%	3.2%
tr|I3LQE2|I3LQE2	19	3	7	1	1	0	0	0	30	9	60	1	2	0	0	0	22.8%	2.9%	6.7%	1.6%	1.6%	0.0%	0.0%	0.0%
tr|F1S3C7|F1S3C7	11	5	1	0	2	0	2	5	18	8	6	0	2	0	9	25	19.3%	7.8%	3.3%	0.0%	4.3%	0.0%	3.7%	8.3%
P79381	15	0	5	0	0	2	1	8	26	0	86	0	0	2	1	61	33.5%	0.0%	12.3%	0.0%	0.0%	7.0%	2.2%	19.8%
tr|F1SRG2|F1SRG2	2	2	0	0	1	0	0	1	4	22	0	0	2	0	0	1	12.5%	11.7%	0.0%	0.0%	11.7%	0.0%	0.0%	11.7%
tr|F2Z553|F2Z553	4	1	2	0	1	0	1	1	9	4	6	0	1	0	17	7	16.8%	11.5%	35.4%	0.0%	11.5%	0.0%	21.2%	21.2%
tr|F1SSF5|F1SSF5	4	2	0	0	1	0	1	1	7	6	0	0	1	0	2	2	21.3%	7.8%	0.0%	0.0%	3.4%	0.0%	5.7%	4.4%
tr|K7GNM9|K7GNM9	2	1	1	0	1	0	1	1	3	10	6	0	1	0	4	2	17.5%	7.0%	7.0%	0.0%	7.0%	0.0%	7.0%	7.0%
tr|I3L9T4|I3L9T4	4	0	0	0	1	0	0	3	13	0	0	0	1	0	0	3	18.1%	0.0%	0.0%	0.0%	6.1%	0.0%	0.0%	5.9%
tr|F1ST14|F1ST14	6	3	1	0	1	0	0	1	7	6	1	0	1	0	0	3	18.3%	3.9%	2.4%	0.0%	2.0%	0.0%	0.0%	6.3%
tr|K7N7E7|K7N7E7	5	2	2	1	0	0	0	1	6	7	9	1	0	0	0	2	9.8%	5.0%	3.3%	3.2%	0.0%	0.0%	0.0%	1.8%
tr|F1ST12|F1ST12	3	2	0	0	1	0	0	0	10	3	0	0	1	0	0	0	2.6%	1.2%	0.0%	0.0%	0.8%	0.0%	0.0%	0.0%
tr|I3LQQ6|I3LQQ6	1	2	0	0	0	1	0	0	1	12	0	0	0	1	0	0	2.8%	6.4%	0.0%	0.0%	0.0%	2.8%	0.0%	0.0%
tr|F2Z5B7|F2Z5B7	5	1	3	0	1	0	0	0	10	3	17	0	1	0	0	0	32.1%	15.5%	38.1%	0.0%	15.5%	0.0%	0.0%	0.0%
tr|F2Z514|F2Z514	5	1	3	0	1	0	0	0	10	3	17	0	1	0	0	0	32.9%	15.9%	39.0%	0.0%	15.9%	0.0%	0.0%	0.0%
tr|F1S5N0|F1S5N0	6	1	8	0	1	0	0	0	6	7	43	0	1	0	0	0	15.2%	2.3%	18.6%	0.0%	2.3%	0.0%	0.0%	0.0%
tr|I3LMG2|I3LMG2	3	2	3	0	1	0	0	0	5	8	84	0	1	0	0	0	8.9%	5.5%	8.9%	0.0%	2.7%	0.0%	0.0%	0.0%
P00506	27	20	14	0	2	9	0	1	91	118	285	0	2	14	0	7	64.0%	43.3%	28.1%	0.0%	8.4%	23.5%	0.0%	4.0%
tr|F1SFZ8|F1SFZ8	72	18	45	2	3	1	11	27	183	55	266	9	7	2	64	120	41.1%	11.0%	23.9%	1.7%	1.5%	0.8%	8.0%	17.5%
tr|I3LLS7|I3LLS7	17	14	5	0	5	0	4	0	35	71	37	0	8	0	11	0	26.3%	15.3%	6.3%	0.0%	9.1%	0.0%	7.7%	0.0%
P63246	21	12	12	1	4	1	4	5	100	46	63	3	7	1	21	30	59.0%	56.2%	36.0%	2.8%	22.1%	7.6%	13.9%	18.9%
tr|I3LNB4|I3LNB4	12	4	11	0	2	0	2	0	33	7	90	0	3	0	3	0	32.0%	11.3%	28.5%	0.0%	3.5%	0.0%	8.2%	0.0%
P02550	38	31	16	9	15	6	11	31	738	1596	1483	32	76	67	1340	939	68.1%	61.2%	47.2%	18.6%	40.4%	14.2%	27.7%	63.9%
tr|F1SBF8|F1SBF8	21	4	18	0	3	0	3	6	49	18	96	0	5	0	9	13	31.3%	5.0%	24.4%	0.0%	4.1%	0.0%	5.8%	12.9%
tr|I3LHZ6|I3LHZ6	42	19	14	0	6	1	1	3	98	63	131	0	7	5	3	5	39.1%	14.3%	13.3%	0.0%	6.2%	1.3%	1.3%	3.1%
tr|F1RZ02|F1RZ02	10	5	7	2	0	0	2	4	16	11	17	2	0	0	10	13	14.4%	5.0%	9.8%	3.3%	0.0%	0.0%	4.7%	7.5%
tr|C0SW08|C0SW08	11	3	1	0	1	0	1	0	22	5	1	0	2	0	3	0	37.0%	15.5%	5.4%	0.0%	6.7%	0.0%	6.7%	0.0%
tr|F1RY68|F1RY68	5	5	1	0	1	0	0	0	11	16	21	0	2	0	0	0	7.0%	7.4%	1.6%	0.0%	2.2%	0.0%	0.0%	0.0%
Q7SIB7	32	19	24	3	4	2	8	22	136	68	190	6	4	5	119	153	73.9%	56.6%	48.7%	8.9%	10.8%	5.0%	29.0%	61.6%
tr|F1SA74|F1SA74	21	6	6	1	2	1	1	3	46	22	32	1	3	1	3	5	35.8%	10.1%	8.4%	1.2%	2.7%	1.5%	1.7%	6.3%
O02705	75	37	47	5	9	2	9	29	648	402	697	23	49	5	165	402	55.9%	50.1%	43.4%	8.0%	13.6%	3.3%	13.0%	37.9%
tr|F1RPH0|F1RPH0	34	18	20	3	4	2	7	22	138	67	169	6	4	5	90	150	73.1%	54.1%	43.7%	9.1%	11.1%	5.2%	25.7%	60.5%
tr|K7GKJ8|K7GKJ8	34	18	20	3	4	2	7	22	138	67	169	6	4	5	90	150	78.1%	57.8%	46.7%	9.8%	11.9%	5.5%	27.4%	64.6%
tr|I3LQH7|I3LQH7	9	3	1	0	2	0	1	2	23	18	4	0	3	0	1	10	75.4%	22.2%	4.8%	0.0%	10.1%	0.0%	7.2%	16.9%
tr|F1S833|F1S833	12	5	1	0	3	0	0	1	19	22	1	0	3	0	0	1	28.2%	7.1%	2.6%	0.0%	5.4%	0.0%	0.0%	5.1%
Q4GWZ2	14	11	8	2	3	2	1	5	73	133	95	4	8	3	21	44	48.8%	42.7%	34.6%	12.2%	17.3%	12.9%	9.2%	26.8%
tr|F1RKU0|F1RKU0	12	6	6	0	0	2	1	1	36	33	37	0	0	5	2	1	31.7%	14.8%	20.8%	0.0%	0.0%	4.6%	4.6%	4.6%
tr|Q6QAS5|Q6QAS5	12	6	8	1	4	0	1	5	52	31	59	2	4	0	2	17	50.8%	39.3%	38.2%	8.4%	29.3%	0.0%	4.7%	30.9%
tr|F1SIU3|F1SIU3	22	13	10	1	7	2	3	4	84	235	47	2	17	4	5	9	31.5%	14.4%	12.2%	1.6%	12.9%	1.6%	4.5%	11.3%

	Peptide Count								Spectral Count								Sequence Coverage							
	Whole Cell	Whole Cell	Whole Cell	Cilia Fraction	Cilia Fraction	Cilia Fraction	Cilia Fraction	Cilia Fraction	Whole Cell	Whole Cell	Whole Cell	Cilia Fraction	Cilia Fraction	Cilia Fraction	Cilia Fraction	Cilia Fraction	Whole Cell	Whole Cell	Whole Cell	Cilia Fraction	Cilia Fraction	Cilia Fraction	Cilia Fraction	Cilia Fraction
Sample#	1	2	6	4	3	5	7	8	1	2	6	4	3	5	7	8	1	2	6	4	3	5	7	8
**Accession**																								
tr|F1S3U9|F1S3U9	16	10	13	0	4	4	4	3	57	96	73	0	6	5	39	18	49.7%	34.5%	31.5%	0.0%	23.4%	15.2%	23.4%	23.4%
tr|I3L6A8|I3L6A8	22	14	11	1	7	2	3	4	84	236	48	2	17	4	5	9	31.5%	15.8%	13.6%	1.6%	12.9%	1.6%	4.5%	11.3%
A1XQV4	14	3	19	1	1	0	1	8	38	4	193	2	1	0	33	76	28.5%	7.7%	34.9%	3.5%	3.5%	0.0%	3.9%	27.1%
tr|I3LIC6|I3LIC6	10	5	1	0	2	0	0	0	17	11	1	0	2	0	0	0	6.3%	2.5%	0.6%	0.0%	0.9%	0.0%	0.0%	0.0%
tr|F1S8S9|F1S8S9	11	3	4	0	2	0	0	0	17	11	14	0	2	0	0	0	11.4%	2.0%	3.4%	0.0%	1.2%	0.0%	0.0%	0.0%
tr|F1SSD2|F1SSD2	8	1	1	1	0	0	1	7	13	1	2	1	0	0	5	29	43.6%	2.0%	7.9%	7.7%	0.0%	0.0%	7.7%	26.3%
tr|K7GPS0|K7GPS0	3	2	0	0	1	0	1	0	6	8	0	0	1	0	6	0	15.4%	8.9%	0.0%	0.0%	6.1%	0.0%	6.1%	0.0%
tr|I3L5D4|I3L5D4	3	2	2	0	1	0	1	0	6	8	3	0	1	0	6	0	13.8%	8.0%	3.2%	0.0%	5.5%	0.0%	5.5%	0.0%
tr|F1SSJ8|F1SSJ8	0	3	0	0	0	1	0	2	0	14	0	0	0	1	0	3	0.0%	3.3%	0.0%	0.0%	0.0%	1.5%	0.0%	4.9%
tr|F1RN05|F1RN05	3	2	3	0	1	0	0	1	6	8	8	0	1	0	0	1	11.5%	5.9%	6.3%	0.0%	2.0%	0.0%	0.0%	4.3%
tr|A5D9J1|A5D9J1	7	2	0	1	0	0	0	0	11	3	0	1	0	0	0	0	53.7%	15.4%	0.0%	6.9%	0.0%	0.0%	0.0%	0.0%
tr|F1RM55|F1RM55	7	2	0	1	0	0	0	0	9	5	0	1	0	0	0	0	10.8%	2.5%	0.0%	1.3%	0.0%	0.0%	0.0%	0.0%
P79335	2	2	0	0	0	1	0	0	2	12	0	0	0	1	0	0	7.0%	6.2%	0.0%	0.0%	0.0%	2.7%	0.0%	0.0%
tr|I3L7H0|I3L7H0	2	2	1	0	1	0	0	0	2	12	5	0	1	0	0	0	8.5%	5.5%	6.6%	0.0%	5.0%	0.0%	0.0%	0.0%
tr|I3LEQ0|I3LEQ0	5	3	2	0	1	0	0	0	9	5	7	0	1	0	0	0	7.6%	4.5%	2.2%	0.0%	1.4%	0.0%	0.0%	0.0%
tr|I3LE01|I3LE01	6	0	1	0	1	0	0	0	14	0	7	0	1	0	0	0	13.5%	0.0%	2.0%	0.0%	2.9%	0.0%	0.0%	0.0%
tr|F2Z5T5|F2Z5T5	45	33	16	10	16	6	12	32	864	1621	1502	33	77	67	1342	947	78.9%	71.8%	47.2%	22.6%	44.3%	14.2%	31.7%	67.8%
tr|F1SHL9|F1SHL9	65	38	35	12	19	4	24	41	887	460	1713	30	57	8	593	607	72.0%	66.1%	50.7%	21.7%	39.4%	8.5%	38.7%	53.7%
tr|F1RK98|F1RK98	37	29	12	9	14	6	11	26	787	1601	1198	31	70	67	1322	891	68.0%	59.6%	32.9%	20.7%	39.1%	14.2%	28.4%	51.8%
tr|F1RS49|F1RS49	14	3	3	0	2	0	2	1	25	32	17	0	4	0	8	2	30.2%	6.2%	3.8%	0.0%	3.3%	0.0%	4.2%	3.3%
tr|F2Z5C7|F2Z5C7	18	13	13	1	1	2	5	5	53	47	91	2	3	2	53	37	49.6%	40.5%	39.4%	3.0%	3.0%	7.6%	17.0%	17.0%
tr|F1SR80|F1SR80	36	28	13	8	12	6	11	28	829	1618	1438	31	73	67	1340	930	56.9%	50.0%	36.3%	16.4%	29.6%	14.2%	27.7%	50.7%
Q29375	10	5	4	0	1	2	0	2	24	19	24	0	1	2	0	5	52.3%	34.1%	25.8%	0.0%	9.8%	10.6%	0.0%	23.5%
tr|Q0PY11|Q0PY11	43	24	24	0	9	6	10	26	842	382	787	0	73	12	1485	645	56.7%	49.1%	34.8%	0.0%	21.2%	17.3%	29.7%	43.1%
tr|F1SC47|F1SC47	20	7	7	1	1	0	1	0	54	33	225	4	2	0	9	0	29.8%	12.5%	11.4%	1.5%	1.5%	0.0%	3.0%	0.0%
P79303	6	3	3	1	1	0	1	2	8	21	13	1	1	0	6	8	12.0%	6.9%	6.3%	3.0%	3.9%	0.0%	2.8%	6.7%
Q29561	12	6	7	2	2	1	2	1	27	105	55	3	4	2	14	2	36.7%	18.9%	34.7%	12.2%	12.2%	6.1%	12.8%	6.1%
P67937	21	5	25	1	1	0	1	6	37	7	163	2	1	0	5	24	59.7%	16.9%	67.7%	4.0%	4.0%	0.0%	8.1%	33.9%
tr|F1SGF1|F1SGF1	4	3	3	0	1	0	1	1	7	37	8	0	3	0	3	1	11.3%	11.3%	12.7%	0.0%	3.1%	0.0%	4.5%	5.2%
tr|F1SJR1|F1SJR1	9	6	6	1	2	1	1	3	31	28	77	1	2	1	21	35	43.6%	35.1%	35.1%	6.2%	13.3%	7.1%	12.8%	26.5%
tr|I3LSU1|I3LSU1	12	3	6	0	2	0	2	4	54	20	26	0	5	0	13	24	29.1%	7.9%	16.1%	0.0%	7.2%	0.0%	4.9%	14.2%
tr|K7GNZ3|K7GNZ3	9	7	2	1	1	1	1	2	42	33	8	1	2	2	1	8	28.8%	27.4%	13.5%	6.0%	6.0%	6.0%	7.0%	13.0%
tr|F1RPQ3|F1RPQ3	7	3	3	0	1	1	2	1	22	23	9	0	2	1	8	1	31.6%	12.3%	13.5%	0.0%	3.2%	3.2%	7.1%	4.5%
tr|D7RK08|D7RK08	7	5	0	1	2	0	0	1	9	36	0	1	2	0	0	4	9.9%	6.8%	0.0%	1.7%	2.9%	0.0%	0.0%	2.7%
tr|K7GST8|K7GST8	14	5	7	0	1	1	2	7	33	12	252	0	2	1	7	29	60.7%	21.4%	31.7%	0.0%	5.3%	5.3%	14.5%	36.6%
tr|I3LI61|I3LI61	14	5	7	0	1	1	2	7	33	12	252	0	2	1	7	29	60.7%	21.4%	31.7%	0.0%	5.3%	5.3%	14.5%	36.6%
tr|F1RGK9|F1RGK9	11	3	6	0	1	0	2	5	23	7	15	0	2	0	18	58	12.5%	4.6%	9.3%	0.0%	0.9%	0.0%	3.4%	9.5%
tr|F1S3I9|F1S3I9	8	4	2	1	1	0	0	1	21	9	9	1	1	0	0	3	22.7%	15.1%	5.0%	2.2%	3.1%	0.0%	0.0%	3.1%
tr|F1SIX3|F1SIX3	7	3	2	1	1	0	0	1	9	21	7	1	1	0	0	1	20.1%	8.3%	4.3%	3.5%	4.7%	0.0%	0.0%	4.7%
tr|I3L7E5|I3L7E5	16	4	0	0	1	0	0	0	20	10	0	0	2	0	0	0	28.2%	4.5%	0.0%	0.0%	1.4%	0.0%	0.0%	0.0%
tr|F1RFU3|F1RFU3	11	5	1	0	2	0	0	0	19	11	1	0	2	0	0	0	6.9%	2.5%	0.6%	0.0%	0.9%	0.0%	0.0%	0.0%
tr|F2Z562|F2Z562	7	3	0	0	1	0	0	4	10	5	0	0	1	0	0	7	22.6%	11.1%	0.0%	0.0%	7.1%	0.0%	0.0%	22.2%
tr|F1SAE9|F1SAE9	5	1	2	0	1	0	0	2	7	8	4	0	1	0	0	5	3.1%	0.6%	2.2%	0.0%	1.4%	0.0%	0.0%	2.8%
tr|K7GRA7|K7GRA7	8	2	0	0	1	0	0	0	11	4	0	0	1	0	0	0	49.7%	19.1%	0.0%	0.0%	11.0%	0.0%	0.0%	0.0%
tr|F1SGZ8|F1SGZ8	6	2	7	0	1	0	2	4	11	4	184	0	1	0	2	8	33.4%	10.9%	25.4%	0.0%	8.9%	0.0%	6.6%	14.9%
tr|I3LN23|I3LN23	2	4	1	0	0	1	0	0	8	7	5	0	0	1	0	0	9.7%	20.3%	3.9%	0.0%	0.0%	6.3%	0.0%	0.0%
tr|I3LEB7|I3LEB7	4	1	2	0	1	0	0	0	5	10	11	0	1	0	0	0	6.1%	1.4%	4.5%	0.0%	1.4%	0.0%	0.0%	0.0%
tr|F1S9Q3|F1S9Q3	63	35	36	4	13	4	12	22	370	202	886	6	26	6	307	257	58.9%	56.7%	42.8%	7.4%	32.1%	5.1%	18.0%	29.8%
tr|F1RFU5|F1RFU5	15	14	11	0	2	5	0	0	41	80	187	0	2	6	0	0	63.3%	65.1%	41.4%	0.0%	16.7%	20.5%	0.0%	0.0%
tr|F1RYA4|F1RYA4	11	8	9	0	2	1	0	0	47	44	63	0	2	4	0	0	29.6%	20.3%	27.5%	0.0%	2.9%	2.9%	0.0%	0.0%
tr|F1RJ93|F1RJ93	23	18	15	3	7	1	7	6	213	92	390	5	14	1	124	86	74.0%	71.6%	46.6%	21.6%	37.5%	5.3%	45.7%	38.9%
tr|F6PVJ7|F6PVJ7	12	5	7	1	1	0	3	5	38	23	31	2	2	0	116	57	61.2%	30.9%	37.5%	5.9%	5.9%	0.0%	17.1%	34.9%
tr|F1SQ01|F1SQ01	13	4	5	0	2	1	2	10	37	9	34	0	2	1	29	72	46.7%	22.4%	17.3%	0.0%	12.1%	4.4%	9.6%	51.1%
tr|F1RZT0|F1RZT0	8	4	0	1	1	0	1	4	28	18	0	1	2	0	9	20	27.6%	11.0%	0.0%	4.1%	4.1%	0.0%	4.1%	15.8%
tr|F1SGT2|F1SGT2	9	5	3	1	1	0	1	2	15	31	43	2	1	0	2	9	41.7%	24.0%	18.6%	5.0%	5.0%	0.0%	5.8%	15.3%
Q8HZV3	7	5	0	1	2	0	0	1	9	37	0	1	2	0	0	4	9.9%	6.8%	0.0%	1.7%	2.9%	0.0%	0.0%	2.7%
tr|F1SAI8|F1SAI8	37	14	14	1	4	2	3	2	154	46	65	1	8	4	4	5	39.0%	16.2%	21.8%	1.9%	5.4%	3.5%	5.3%	3.1%
tr|F1SKM9|F1SKM9	12	6	10	2	5	0	7	5	322	79	225	2	24	0	415	209	65.9%	60.0%	55.6%	25.2%	48.1%	0.0%	51.1%	31.9%
tr|F1SN05|F1SN05	5	3	8	1	1	0	1	3	11	20	19	1	1	0	3	5	21.6%	13.6%	36.7%	4.9%	4.9%	0.0%	4.9%	13.6%
tr|F1RKW8|F1RKW8	13	6	3	0	1	1	1	0	24	7	16	0	1	1	7	0	33.4%	10.7%	10.2%	0.0%	2.8%	3.1%	3.1%	0.0%
tr|F1RSM2|F1RSM2	5	3	4	0	1	1	3	0	16	15	46	0	1	1	5	0	14.5%	9.6%	7.2%	0.0%	2.4%	2.4%	10.5%	0.0%
tr|A3EX84|A3EX84	8	7	4	1	1	3	3	7	36	167	32	5	1	7	63	54	25.8%	26.2%	11.2%	6.5%	6.5%	10.4%	10.8%	21.2%
tr|F2Z5B6|F2Z5B6	26	3	31	1	1	0	2	7	43	4	321	2	1	0	19	31	52.5%	7.7%	51.4%	3.5%	3.5%	0.0%	4.6%	31.3%
tr|F2Z5K6|F2Z5K6	4	2	1	0	2	2	0	0	22	89	9	0	2	5	0	0	21.9%	12.4%	9.5%	0.0%	21.9%	12.4%	0.0%	0.0%
tr|I3LKK0|I3LKK0	4	4	3	0	2	0	1	1	18	14	6	0	2	0	1	1	13.4%	8.5%	8.9%	0.0%	10.2%	0.0%	4.9%	5.2%
tr|F1RRA7|F1RRA7	13	7	4	1	1	0	0	0	18	14	32	1	1	0	0	0	25.3%	10.6%	6.4%	1.4%	1.8%	0.0%	0.0%	0.0%
tr|F1RUF5|F1RUF5	7	0	7	0	0	1	6	19	16	0	26	0	0	1	44	168	16.7%	0.0%	18.1%	0.0%	0.0%	3.4%	17.1%	40.1%
tr|F1SA39|F1SA39	2	2	0	0	1	0	2	5	2	14	0	0	1	0	8	18	11.9%	5.7%	0.0%	0.0%	5.7%	0.0%	12.2%	24.9%
tr|F1RPY3|F1RPY3	4	2	4	0	1	0	1	2	13	3	31	0	1	0	14	5	11.9%	11.0%	11.0%	0.0%	4.7%	0.0%	4.7%	5.5%
tr|F1RGS2|F1RGS2	3	3	3	0	1	0	1	1	12	4	6	0	1	0	3	1	7.5%	9.5%	5.4%	0.0%	3.0%	0.0%	3.2%	4.1%
Q70KH2	3	3	3	0	1	0	1	1	12	4	6	0	1	0	3	1	7.5%	9.5%	5.4%	0.0%	3.0%	0.0%	3.2%	4.1%
tr|F1S754|F1S754	4	5	1	0	1	0	1	0	9	7	5	0	1	0	3	0	10.8%	13.2%	1.9%	0.0%	4.4%	0.0%	4.4%	0.0%
Q29576	3	3	1	1	0	0	1	0	6	10	8	1	0	0	1	0	36.5%	45.2%	13.5%	14.4%	0.0%	0.0%	23.1%	0.0%
tr|F1RSJ9|F1RSJ9	2	2	0	0	1	0	0	0	2	14	0	0	1	0	0	0	6.8%	5.8%	0.0%	0.0%	3.6%	0.0%	0.0%	0.0%
tr|F1RUH5|F1RUH5	1	2	0	0	0	1	0	0	1	15	0	0	0	1	0	0	3.3%	6.2%	0.0%	0.0%	0.0%	3.5%	0.0%	0.0%
tr|F1SGS7|F1SGS7	5	3	1	0	1	0	0	0	5	11	2	0	1	0	0	0	6.8%	2.5%	2.5%	0.0%	1.5%	0.0%	0.0%	0.0%
P83884	3	4	1	0	0	1	0	0	6	10	3	0	0	1	0	0	11.3%	32.1%	7.5%	0.0%	0.0%	13.2%	0.0%	0.0%
tr|F1RJS3|F1RJS3	5	3	3	1	0	0	0	0	7	9	5	1	0	0	0	0	13.5%	6.3%	9.0%	3.8%	0.0%	0.0%	0.0%	0.0%
tr|F1S4P6|F1S4P6	30	15	16	2	2	1	3	2	56	89	71	4	4	1	3	3	24.1%	11.6%	11.7%	2.5%	2.2%	1.2%	4.1%	3.0%
tr|F1RIP4|F1RIP4	11	5	8	1	3	0	2	6	18	79	45	3	3	0	8	12	31.3%	11.4%	22.5%	3.0%	11.2%	0.0%	5.4%	18.1%
tr|I3LR32|I3LR32	18	7	11	1	1	2	2	8	98	32	68	3	1	4	15	65	39.7%	19.6%	18.2%	3.4%	2.4%	5.7%	6.1%	26.1%

	Peptide Count								Spectral Count								Sequence Coverage							
	Whole Cell	Whole Cell	Whole Cell	Cilia Fraction	Cilia Fraction	Cilia Fraction	Cilia Fraction	Cilia Fraction	Whole Cell	Whole Cell	Whole Cell	Cilia Fraction	Cilia Fraction	Cilia Fraction	Cilia Fraction	Cilia Fraction	Whole Cell	Whole Cell	Whole Cell	Cilia Fraction	Cilia Fraction	Cilia Fraction	Cilia Fraction	Cilia Fraction
Sample#	1	2	6	4	3	5	7	8	1	2	6	4	3	5	7	8	1	2	6	4	3	5	7	8
**Accession**																								
tr|K7GPB0|K7GPB0	5	4	2	1	1	1	0	0	19	46	13	2	1	1	0	0	46.4%	20.8%	12.0%	7.7%	7.7%	7.7%	0.0%	0.0%
tr|F1SK86|F1SK86	19	7	8	0	3	2	1	9	55	43	41	0	3	3	1	33	43.4%	16.0%	18.6%	0.0%	5.9%	2.9%	3.9%	19.7%
tr|F1SN27|F1SN27	6	8	4	0	0	1	1	3	46	52	35	0	0	6	5	18	31.2%	27.2%	17.4%	0.0%	0.0%	5.1%	5.1%	11.8%
tr|F1RHH8|F1RHH8	17	5	2	1	1	0	2	0	27	22	22	1	2	0	3	0	10.8%	2.4%	2.1%	0.6%	0.9%	0.0%	2.0%	0.0%
tr|F1SFQ4|F1SFQ4	11	8	7	0	4	0	3	2	41	41	73	0	5	0	3	7	51.8%	30.3%	37.1%	0.0%	13.6%	0.0%	16.1%	19.0%
tr|F1RX79|F1RX79	2	2	0	0	1	1	0	0	8	74	0	0	1	4	0	0	7.1%	7.1%	0.0%	0.0%	7.1%	7.1%	0.0%	0.0%
tr|F1RS60|F1RS60	2	2	0	0	1	1	0	0	8	74	0	0	1	4	0	0	5.5%	5.5%	0.0%	0.0%	5.5%	5.5%	0.0%	0.0%
tr|F1RGK5|F1RGK5	25	4	29	2	1	0	4	11	60	6	295	3	1	0	63	105	55.2%	14.9%	56.9%	9.7%	4.0%	0.0%	15.7%	43.5%
tr|K7GQL0|K7GQL0	5	3	1	1	1	0	0	3	10	23	1	1	1	0	0	9	29.7%	17.6%	7.7%	6.6%	6.6%	0.0%	0.0%	13.2%
tr|K7GQS5|K7GQS5	5	3	1	1	1	0	0	3	10	23	1	1	1	0	0	9	19.0%	11.3%	4.9%	4.2%	4.2%	0.0%	0.0%	8.5%
tr|F1RRG9|F1RRG9	6	4	4	0	1	1	0	0	7	26	11	0	1	1	0	0	7.0%	5.0%	7.6%	0.0%	0.9%	0.9%	0.0%	0.0%
tr|F1SDN2|F1SDN2	15	8	8	1	1	0	1	2	35	48	60	2	3	0	1	2	41.3%	27.8%	22.5%	1.9%	1.9%	0.0%	5.7%	11.2%
tr|F1RFQ7|F1RFQ7	17	9	4	0	3	3	0	5	64	88	26	0	4	5	0	20	60.2%	30.1%	16.7%	0.0%	12.5%	12.0%	0.0%	17.6%
tr|D0G0C6|D0G0C6	21	7	7	0	3	0	3	4	49	19	57	0	4	0	13	4	49.7%	13.0%	14.3%	0.0%	9.8%	0.0%	8.0%	11.2%
tr|F1RPX1|F1RPX1	10	5	4	0	1	1	0	1	18	33	14	0	1	2	0	1	23.8%	8.8%	7.5%	0.0%	1.5%	1.9%	0.0%	1.9%
tr|F1RHU6|F1RHU6	10	3	7	0	1	0	3	5	27	7	39	0	2	0	15	22	32.9%	11.5%	21.1%	0.0%	5.1%	0.0%	13.2%	19.9%
tr|F1SLY7|F1SLY7	8	1	3	0	0	1	4	2	12	5	10	0	0	1	8	4	44.5%	4.9%	24.2%	0.0%	0.0%	4.9%	26.4%	18.7%
tr|F1S519|F1S519	0	2	0	0	0	1	1	2	0	17	0	0	0	1	4	3	0.0%	3.7%	0.0%	0.0%	0.0%	2.5%	2.5%	4.2%
A1XQR9	2	1	0	0	1	0	1	0	15	2	0	0	1	0	3	0	39.1%	12.0%	0.0%	0.0%	12.0%	0.0%	17.4%	0.0%
tr|I3LDW9|I3LDW9	2	1	0	0	1	0	1	0	15	2	0	0	1	0	3	0	39.1%	12.0%	0.0%	0.0%	12.0%	0.0%	17.4%	0.0%
tr|A5D9J5|A5D9J5	4	3	2	1	0	0	1	1	7	10	11	1	0	0	1	4	20.6%	18.7%	11.1%	6.0%	0.0%	0.0%	9.5%	4.8%
tr|A5D9J4|A5D9J4	4	3	2	1	0	0	1	1	7	10	11	1	0	0	1	4	18.8%	17.0%	10.1%	5.4%	0.0%	0.0%	8.7%	4.3%
tr|A5D9J6|A5D9J6	4	3	2	1	0	0	1	1	7	10	11	1	0	0	1	4	25.0%	22.6%	13.5%	7.2%	0.0%	0.0%	11.5%	5.8%
tr|I3LD63|I3LD63	6	3	2	1	0	0	1	0	8	9	14	1	0	0	3	0	14.8%	8.0%	4.8%	2.4%	0.0%	0.0%	4.6%	0.0%
tr|F1SBY1|F1SBY1	1	1	0	0	1	0	0	0	1	16	0	0	1	0	0	0	1.2%	1.2%	0.0%	0.0%	1.2%	0.0%	0.0%	0.0%
tr|K7GSN4|K7GSN4	5	2	0	0	0	1	0	0	15	2	0	0	0	1	0	0	30.2%	23.3%	0.0%	0.0%	0.0%	10.7%	0.0%	0.0%
tr|F1S1R0|F1S1R0	1	1	0	0	1	0	0	0	1	16	0	0	1	0	0	0	1.8%	1.3%	0.0%	0.0%	1.3%	0.0%	0.0%	0.0%
tr|I3L5T3|I3L5T3	3	2	4	0	1	0	0	1	13	4	19	0	1	0	0	1	18.2%	8.5%	12.8%	0.0%	3.1%	0.0%	0.0%	3.1%
tr|F1S4H0|F1S4H0	8	1	5	0	1	0	0	1	14	3	58	0	1	0	0	3	26.4%	1.7%	12.7%	0.0%	1.7%	0.0%	0.0%	4.7%
tr|F1SSR4|F1SSR4	7	4	6	0	1	0	0	1	11	6	53	0	1	0	0	2	33.1%	16.4%	24.9%	0.0%	5.2%	0.0%	0.0%	4.7%
tr|I3LQ01|I3LQ01	5	0	4	0	0	1	0	0	17	0	8	0	0	1	0	0	11.1%	0.0%	13.9%	0.0%	0.0%	2.0%	0.0%	0.0%
tr|I3LSM7|I3LSM7	10	2	14	0	1	0	0	0	14	3	61	0	1	0	0	0	22.3%	4.5%	21.4%	0.0%	4.8%	0.0%	0.0%	0.0%
tr|I3LDM3|I3LDM3	21	11	3	0	3	3	0	1	54	66	9	0	4	3	0	1	19.1%	15.2%	5.1%	0.0%	5.1%	3.0%	0.0%	2.0%
tr|F2Z5E6|F2Z5E6	9	8	4	1	1	1	2	3	39	64	14	3	2	1	7	13	37.7%	28.4%	23.0%	6.4%	6.4%	6.4%	13.7%	14.7%
Q29308	9	5	4	1	2	0	1	7	34	35	21	1	3	0	13	96	37.5%	24.3%	21.3%	5.1%	12.5%	0.0%	8.1%	50.7%
tr|F1STM4|F1STM4	12	12	6	0	3	3	2	5	452	245	294	0	34	6	101	90	22.8%	20.2%	11.3%	0.0%	10.8%	9.5%	6.3%	12.6%
tr|F1STN7|F1STN7	13	12	6	0	3	3	2	5	453	245	294	0	34	6	101	90	26.5%	20.2%	11.3%	0.0%	10.8%	9.5%	6.3%	12.6%
tr|F1S6D8|F1S6D8	10	5	6	1	1	1	2	1	21	84	41	2	2	2	14	2	34.9%	14.5%	32.6%	7.0%	7.0%	7.0%	14.5%	7.0%
tr|F1S8K5|F1S8K5	9	5	1	1	0	1	0	1	10	25	5	1	0	1	0	3	11.0%	6.0%	1.6%	1.5%	0.0%	1.8%	0.0%	2.5%
tr|F1SG00|F1SG00	19	5	21	1	1	0	2	5	46	7	122	2	1	0	19	32	52.1%	12.3%	40.5%	3.5%	3.5%	0.0%	11.6%	31.3%
tr|F1SJR7|F1SJR7	29	10	9	2	3	1	1	8	75	67	31	2	5	1	3	21	59.4%	15.3%	16.9%	3.1%	5.1%	1.8%	1.8%	22.4%
tr|I3LCW1|I3LCW1	17	8	4	0	1	3	3	3	61	28	151	0	1	4	13	10	48.0%	23.1%	11.7%	0.0%	3.5%	13.5%	9.3%	9.4%
tr|I3LLD5|I3LLD5	33	20	16	1	9	0	6	8	147	405	103	2	29	0	104	82	72.3%	47.4%	31.2%	4.7%	28.2%	0.0%	23.7%	21.9%
tr|Q6QA25|Q6QA25	29	5	31	2	1	0	4	12	65	7	307	3	1	0	63	120	63.7%	19.0%	60.9%	9.7%	4.0%	0.0%	15.7%	48.0%
tr|I3LEX0|I3LEX0	8	12	7	0	1	2	0	3	23	49	20	0	1	3	0	8	25.4%	43.0%	23.3%	0.0%	4.7%	11.4%	0.0%	14.0%
Q9N0F1	7	8	6	0	0	2	0	0	36	18	29	0	0	3	0	0	22.9%	22.0%	17.8%	0.0%	0.0%	8.8%	0.0%	0.0%
tr|F1S6V4|F1S6V4	12	6	4	0	2	0	1	3	17	19	25	0	2	0	1	9	19.8%	10.6%	6.3%	0.0%	2.2%	0.0%	1.7%	5.6%
tr|I3L813|I3L813	10	6	3	1	1	0	0	1	15	21	16	1	1	0	0	3	50.2%	47.1%	16.9%	5.0%	5.0%	0.0%	0.0%	11.1%
tr|I3LGP0|I3LGP0	11	4	5	0	2	0	1	3	29	7	47	0	2	0	1	4	24.8%	7.1%	11.1%	0.0%	3.1%	0.0%	1.9%	7.6%
tr|K7GLN4|K7GLN4	2	2	0	0	1	0	0	3	12	6	0	0	1	0	0	15	21.2%	19.0%	0.0%	0.0%	14.0%	0.0%	0.0%	21.2%
tr|F1SPF6|F1SPF6	7	3	1	0	1	0	1	5	10	8	6	0	1	0	9	10	20.1%	13.4%	6.3%	0.0%	5.2%	0.0%	5.2%	29.4%
tr|F1SBA5|F1SBA5	5	5	5	0	1	0	1	3	12	6	27	0	1	0	4	22	19.2%	24.8%	18.8%	0.0%	9.2%	0.0%	8.8%	9.2%
tr|I3LS28|I3LS28	1	4	3	0	1	0	0	2	4	14	12	0	1	0	0	11	2.9%	11.1%	14.2%	0.0%	3.2%	0.0%	0.0%	11.3%
tr|I3LHP2|I3LHP2	8	1	5	0	1	0	1	2	17	1	39	0	1	0	6	5	38.4%	9.3%	24.6%	0.0%	9.3%	0.0%	6.4%	6.4%
tr|F1SDR6|F1SDR6	5	4	1	0	1	0	1	1	10	8	2	0	1	0	1	1	6.1%	7.2%	2.8%	0.0%	1.5%	0.0%	1.8%	1.8%
tr|A5GFQ9|A5GFQ9	7	0	0	1	0	0	0	1	18	0	0	1	0	0	0	1	14.5%	0.0%	0.0%	2.5%	0.0%	0.0%	0.0%	2.0%
tr|F1RYS5|F1RYS5	9	5	7	0	1	0	1	2	11	7	21	0	1	0	1	2	25.4%	13.9%	22.2%	0.0%	4.6%	0.0%	3.2%	10.4%
tr|F1S429|F1S429	4	2	1	0	1	0	0	0	8	10	4	0	1	0	0	0	8.1%	2.9%	3.1%	0.0%	1.4%	0.0%	0.0%	0.0%
tr|I3LB90|I3LB90	5	2	2	0	1	0	0	0	15	3	26	0	1	0	0	0	24.7%	11.7%	18.7%	0.0%	3.7%	0.0%	0.0%	0.0%
tr|F1RSZ3|F1RSZ3	7	3	6	0	1	0	0	0	10	8	241	0	1	0	0	0	14.2%	4.6%	7.7%	0.0%	2.6%	0.0%	0.0%	0.0%
tr|F1SB62|F1SB62	13	8	7	0	2	1	1	7	46	27	35	0	2	2	9	25	43.8%	32.0%	27.8%	0.0%	8.6%	2.7%	3.9%	32.8%
tr|F1SB60|F1SB60	8	6	4	1	1	1	1	1	24	50	62	2	1	1	17	19	47.7%	23.9%	16.7%	6.3%	6.3%	6.3%	6.8%	6.8%
tr|I3LE53|I3LE53	12	7	9	1	2	1	3	3	46	28	212	1	2	1	45	31	33.7%	30.4%	30.4%	7.7%	12.7%	7.2%	23.2%	14.4%
tr|I3LCU9|I3LCU9	5	2	1	0	2	0	1	4	29	8	1	0	2	0	2	20	41.1%	10.1%	7.0%	0.0%	10.1%	0.0%	10.1%	32.9%
tr|A1X898|A1X898	20	2	8	1	1	0	2	9	46	10	42	2	1	0	12	60	47.4%	6.6%	17.0%	2.6%	3.6%	0.0%	4.3%	23.8%
tr|A6M928|A6M928	34	20	16	1	9	0	7	9	177	405	103	2	29	0	108	83	71.4%	46.8%	30.8%	4.7%	27.8%	0.0%	30.8%	30.8%
tr|F1RYZ5|F1RYZ5	10	7	4	0	1	3	0	4	41	91	39	0	1	6	0	16	39.2%	40.9%	23.3%	0.0%	6.8%	16.5%	0.0%	15.9%
P00795	10	4	7	1	1	0	2	2	26	12	54	1	1	0	3	3	31.9%	10.4%	23.8%	4.1%	4.1%	0.0%	9.3%	9.3%
tr|F1S8M3|F1S8M3	13	3	3	0	1	1	0	1	18	20	10	0	1	1	0	1	34.3%	10.7%	12.4%	0.0%	3.0%	3.0%	0.0%	5.6%
tr|F1S564|F1S564	5	4	2	0	1	0	1	0	12	7	3	0	1	0	3	0	21.9%	13.7%	11.0%	0.0%	3.8%	0.0%	7.4%	0.0%
tr|I3LMF1|I3LMF1	6	3	0	0	1	0	0	1	14	5	0	0	1	0	0	1	28.6%	27.9%	0.0%	0.0%	12.1%	0.0%	0.0%	6.4%
tr|K7GRY4|K7GRY4	6	3	0	0	1	0	0	1	14	5	0	0	1	0	0	1	25.6%	25.0%	0.0%	0.0%	10.9%	0.0%	0.0%	5.8%
tr|I3LB95|I3LB95	6	3	0	0	1	0	0	1	14	5	0	0	1	0	0	1	28.8%	28.1%	0.0%	0.0%	12.2%	0.0%	0.0%	6.5%
tr|K7GQ62|K7GQ62	6	3	0	0	1	0	0	1	14	5	0	0	1	0	0	1	75.5%	73.6%	0.0%	0.0%	32.1%	0.0%	0.0%	17.0%
tr|F1STS3|F1STS3	3	1	0	0	1	0	0	0	3	16	0	0	1	0	0	0	4.9%	1.2%	0.0%	0.0%	1.2%	0.0%	0.0%	0.0%
tr|F1RP17|F1RP17	34	12	17	0	4	1	2	10	82	33	154	0	5	1	19	60	50.8%	25.8%	29.8%	0.0%	9.0%	2.2%	5.1%	22.3%
tr|I3LFL4|I3LFL4	10	9	3	1	3	0	1	2	27	69	16	1	4	0	1	5	35.5%	38.5%	21.3%	6.5%	20.1%	0.0%	7.7%	13.6%
tr|I3L6F1|I3L6F1	10	9	3	1	3	0	1	2	27	69	16	1	4	0	1	5	31.9%	34.6%	19.1%	5.9%	18.1%	0.0%	6.9%	12.2%
tr|F1SDP3|F1SDP3	12	3	2	1	1	1	0	0	50	46	13	2	1	2	0	0	39.9%	4.8%	6.8%	2.5%	2.5%	2.5%	0.0%	0.0%
tr|I3L5T8|I3L5T8	10	4	9	0	1	1	0	3	22	36	41	0	1	2	0	9	22.7%	10.8%	18.4%	0.0%	2.3%	2.3%	0.0%	9.8%
P34935	13	5	12	1	2	1	2	5	75	81	81	3	3	2	11	25	43.5%	23.3%	30.9%	6.5%	13.4%	6.9%	6.1%	18.3%

	Peptide Count								Spectral Count								Sequence Coverage							
	Whole Cell	Whole Cell	Whole Cell	Cilia Fraction	Cilia Fraction	Cilia Fraction	Cilia Fraction	Cilia Fraction	Whole Cell	Whole Cell	Whole Cell	Cilia Fraction	Cilia Fraction	Cilia Fraction	Cilia Fraction	Cilia Fraction	Whole Cell	Whole Cell	Whole Cell	Cilia Fraction	Cilia Fraction	Cilia Fraction	Cilia Fraction	Cilia Fraction
Sample#	1	2	6	4	3	5	7	8	1	2	6	4	3	5	7	8	1	2	6	4	3	5	7	8
**Accession**																								
tr|K7GP05|K7GP05	3	5	0	0	1	1	0	0	5	34	0	0	1	1	0	0	18.6%	22.7%	0.0%	0.0%	7.3%	11.4%	0.0%	0.0%
tr|F1S1Q7|F1S1Q7	3	5	2	0	1	1	0	0	5	34	7	0	1	1	0	0	15.3%	18.7%	6.7%	0.0%	6.0%	9.3%	0.0%	0.0%
tr|K7GNC6|K7GNC6	3	5	2	0	1	1	0	0	5	34	7	0	1	1	0	0	15.3%	18.7%	6.7%	0.0%	6.0%	9.3%	0.0%	0.0%
tr|I3LJW4|I3LJW4	46	25	20	1	6	4	6	11	178	222	167	1	13	6	219	42	52.3%	31.2%	28.7%	1.0%	10.2%	6.1%	12.4%	17.6%
tr|F1RQ06|F1RQ06	4	1	1	1	1	1	0	2	16	44	2	1	1	1	0	10	30.0%	10.7%	10.7%	10.7%	10.7%	10.7%	0.0%	20.0%
tr|F1SJ34|F1SJ34	12	3	4	0	1	1	1	4	34	6	16	0	1	1	3	35	56.6%	27.6%	27.0%	0.0%	11.2%	11.2%	6.6%	30.9%
tr|F2Z522|F2Z522	8	4	5	0	1	1	2	2	33	7	31	0	1	1	5	7	50.0%	23.1%	30.1%	0.0%	8.3%	7.1%	16.0%	7.7%
tr|F1SE82|F1SE82	0	2	0	1	0	1	0	0	0	40	0	1	0	1	0	0	0.0%	8.4%	0.0%	8.4%	0.0%	8.4%	0.0%	0.0%
B0KYV5	3	4	5	0	1	0	0	3	6	14	26	0	1	0	0	13	5.6%	5.6%	12.3%	0.0%	1.6%	0.0%	0.0%	8.1%
tr|I3LI05|I3LI05	6	1	5	0	1	0	1	1	16	4	12	0	1	0	4	4	11.3%	1.2%	7.4%	0.0%	3.0%	0.0%	1.4%	1.4%
tr|F1RHF0|F1RHF0	7	1	2	0	1	0	0	2	13	7	13	0	1	0	0	3	49.5%	6.6%	9.4%	0.0%	6.6%	0.0%	0.0%	14.6%
tr|I3LTE8|I3LTE8	9	1	2	0	1	0	0	1	14	6	7	0	1	0	0	1	17.4%	1.3%	2.5%	0.0%	1.3%	0.0%	0.0%	1.3%
tr|I3L5Y1|I3L5Y1	3	2	0	0	1	0	0	0	6	14	0	0	1	0	0	0	43.9%	17.1%	0.0%	0.0%	17.1%	0.0%	0.0%	0.0%
Q95307	5	5	0	0	0	1	0	0	13	7	0	0	0	1	0	0	19.0%	39.4%	0.0%	0.0%	0.0%	9.5%	0.0%	0.0%
tr|K7GQW4|K7GQW4	4	1	1	0	1	0	0	0	4	16	2	0	1	0	0	0	6.9%	1.3%	1.8%	0.0%	1.3%	0.0%	0.0%	0.0%
tr|F1SQN4|F1SQN4	4	1	1	0	1	0	0	0	4	16	2	0	1	0	0	0	6.9%	1.3%	1.8%	0.0%	1.3%	0.0%	0.0%	0.0%
tr|K7GLT8|K7GLT8	26	15	16	1	3	2	6	18	297	56	690	4	10	3	55	164	57.7%	42.1%	32.6%	2.8%	12.1%	6.3%	13.0%	37.8%
tr|I3LVI8|I3LVI8	12	11	5	0	1	2	0	4	29	34	29	0	1	2	0	14	29.7%	29.3%	16.9%	0.0%	4.9%	5.3%	0.0%	19.2%
tr|F1RFM7|F1RFM7	8	3	2	1	1	0	2	2	15	27	44	1	1	0	18	11	32.3%	8.5%	10.0%	2.5%	2.5%	0.0%	14.4%	14.4%
tr|F1S1U5|F1S1U5	4	4	3	1	1	0	0	2	16	26	21	1	1	0	0	2	18.6%	10.8%	7.5%	4.6%	3.8%	0.0%	0.0%	8.2%
tr|F2Z505|F2Z505	9	7	2	1	1	0	0	0	17	25	4	1	1	0	0	0	21.5%	25.4%	5.5%	3.0%	3.0%	0.0%	0.0%	0.0%
tr|I3L687|I3L687	5	2	3	0	1	0	2	2	15	6	25	0	1	0	147	63	12.8%	5.3%	7.6%	0.0%	2.3%	0.0%	8.0%	5.9%
tr|I3LJJ8|I3LJJ8	10	3	11	0	1	0	5	10	14	7	87	0	1	0	75	148	31.9%	16.7%	43.3%	0.0%	6.1%	0.0%	32.7%	37.6%
tr|I3LSM3|I3LSM3	11	1	4	0	1	0	2	5	20	1	17	0	1	0	6	12	31.7%	1.7%	14.8%	0.0%	1.0%	0.0%	10.7%	15.8%
tr|F1SR76|F1SR76	9	2	6	1	0	0	0	4	17	4	20	1	0	0	0	15	27.5%	5.7%	16.5%	2.3%	0.0%	0.0%	0.0%	12.5%
tr|Q2PYM7|Q2PYM7	9	2	0	1	0	0	0	2	18	3	0	1	0	0	0	5	62.1%	12.3%	0.0%	5.5%	0.0%	0.0%	0.0%	20.1%
tr|F1SCZ4|F1SCZ4	11	2	0	0	1	0	0	0	17	4	0	0	1	0	0	0	26.5%	5.4%	0.0%	0.0%	3.1%	0.0%	0.0%	0.0%
tr|F1SEQ3|F1SEQ3	3	3	0	0	1	0	0	0	5	16	0	0	1	0	0	0	9.3%	9.0%	0.0%	0.0%	5.8%	0.0%	0.0%	0.0%
tr|F1S2X3|F1S2X3	3	2	1	0	1	0	0	0	7	14	2	0	1	0	0	0	14.7%	8.8%	5.9%	0.0%	3.9%	0.0%	0.0%	0.0%
tr|F1SEN2|F1SEN2	38	26	23	1	2	5	3	10	176	100	349	4	2	7	39	42	62.0%	51.3%	36.2%	3.6%	3.8%	8.6%	7.5%	19.7%
tr|I3LPZ8|I3LPZ8	4	2	1	1	1	0	0	0	109	19	24	2	4	0	0	0	8.9%	8.9%	3.2%	3.2%	3.2%	0.0%	0.0%	0.0%
tr|F1RQC5|F1RQC5	15	8	3	0	2	0	2	1	23	41	5	0	3	0	3	2	17.0%	5.7%	5.0%	0.0%	1.7%	0.0%	4.1%	2.0%
tr|F1S8L9|F1S8L9	46	16	21	1	5	2	7	15	137	164	250	3	9	2	131	104	47.3%	21.0%	26.5%	1.1%	6.0%	1.3%	15.3%	27.5%
tr|F1RGG1|F1RGG1	10	7	4	1	2	0	1	7	39	47	21	1	3	0	13	96	42.8%	31.0%	20.0%	4.8%	11.7%	0.0%	7.6%	47.6%
tr|F1S593|F1S593	13	5	3	0	2	0	0	3	28	15	11	0	2	0	0	8	18.7%	5.8%	9.0%	0.0%	1.9%	0.0%	0.0%	5.0%
tr|F1SLR5|F1SLR5	14	5	12	0	2	0	0	0	26	17	29	0	2	0	0	0	12.5%	3.1%	10.5%	0.0%	1.6%	0.0%	0.0%	0.0%
tr|F1SUE3|F1SUE3	11	3	5	0	2	1	1	2	39	26	18	0	2	1	10	25	50.9%	10.4%	37.0%	0.0%	4.8%	4.8%	5.5%	10.4%
tr|I3LFQ4|I3LFQ4	6	4	2	0	3	0	1	2	39	26	14	0	3	0	2	4	32.9%	24.0%	18.6%	0.0%	24.0%	0.0%	5.4%	14.4%
tr|F1RT96|F1RT96	16	5	17	1	2	0	0	3	48	17	180	1	2	0	0	6	33.5%	12.8%	33.3%	1.8%	4.3%	0.0%	0.0%	7.4%
tr|F1SP32|F1SP32	9	5	4	1	2	1	4	7	37	50	16	1	2	1	25	24	33.6%	9.8%	11.0%	4.4%	7.1%	2.7%	21.8%	33.1%
tr|I3L893|I3L893	20	8	14	0	3	0	5	6	91	19	75	0	5	0	93	41	42.5%	26.0%	39.8%	0.0%	8.7%	0.0%	11.6%	20.1%
tr|Q2TJA5|Q2TJA5	10	3	5	0	1	0	1	3	19	3	20	0	1	0	1	13	44.1%	10.2%	19.6%	0.0%	5.0%	0.0%	6.2%	10.6%
tr|F1RRS3|F1RRS3	11	1	2	0	1	0	1	2	16	6	7	0	1	0	2	3	23.3%	1.3%	2.5%	0.0%	1.3%	0.0%	4.3%	5.6%
tr|I3LSB8|I3LSB8	6	5	1	0	1	0	1	1	11	11	3	0	1	0	1	2	3.7%	2.6%	0.8%	0.0%	0.6%	0.0%	0.6%	1.1%
tr|I3LDN4|I3LDN4	6	5	1	0	1	0	1	1	11	11	3	0	1	0	1	2	3.6%	2.6%	0.8%	0.0%	0.6%	0.0%	0.6%	1.1%
tr|F1RQC1|F1RQC1	9	3	2	1	0	0	0	1	18	4	10	1	0	0	0	3	22.2%	6.4%	8.2%	4.6%	0.0%	0.0%	0.0%	1.8%
tr|I3L703|I3L703	9	3	2	1	0	0	0	1	18	4	10	1	0	0	0	3	22.0%	6.3%	8.1%	4.6%	0.0%	0.0%	0.0%	1.8%
tr|F1SVB0|F1SVB0	9	3	9	0	1	0	1	2	19	3	94	0	1	0	1	8	38.4%	19.2%	26.9%	0.0%	8.0%	0.0%	8.0%	7.4%
tr|F2Z5Q8|F2Z5Q8	2	2	1	0	0	1	0	0	8	14	5	0	0	1	0	0	20.0%	18.2%	9.1%	0.0%	0.0%	12.7%	0.0%	0.0%
tr|I3LT97|I3LT97	1	2	1	0	0	1	0	0	2	20	12	0	0	1	0	0	5.2%	9.9%	5.2%	0.0%	0.0%	4.7%	0.0%	0.0%
tr|I3LRY4|I3LRY4	10	3	3	0	1	0	0	0	14	8	14	0	1	0	0	0	10.5%	2.6%	2.6%	0.0%	1.6%	0.0%	0.0%	0.0%
tr|F1SM00|F1SM00	10	3	3	0	1	0	0	0	14	8	14	0	1	0	0	0	10.9%	2.7%	2.7%	0.0%	1.6%	0.0%	0.0%	0.0%
tr|F1SIQ0|F1SIQ0	1	1	5	0	1	0	0	0	1	21	29	0	1	0	0	0	1.8%	1.5%	7.1%	0.0%	1.5%	0.0%	0.0%	0.0%
P06348	7	3	3	0	1	0	0	0	17	5	43	0	1	0	0	0	7.6%	7.6%	7.6%	0.0%	5.2%	0.0%	0.0%	0.0%
tr|F1RGP1|F1RGP1	51	20	14	2	3	1	3	0	169	118	64	5	6	2	11	0	41.2%	17.7%	14.9%	1.1%	3.8%	1.1%	2.9%	0.0%
tr|I3LNG8|I3LNG8	21	12	11	1	2	0	4	7	41	48	59	1	3	0	20	48	44.3%	25.1%	31.1%	2.2%	5.5%	0.0%	13.7%	18.8%
Q6RI85	9	6	9	1	1	0	2	7	24	21	64	1	1	0	3	23	19.9%	17.3%	15.6%	3.8%	3.8%	0.0%	7.7%	17.3%
tr|I3LHE7|I3LHE7	11	5	0	0	0	2	0	1	19	26	0	0	0	2	0	1	26.0%	6.8%	0.0%	0.0%	0.0%	3.9%	0.0%	3.7%
tr|I3LRU1|I3LRU1	11	5	0	0	0	2	0	1	19	26	0	0	0	2	0	1	25.5%	6.7%	0.0%	0.0%	0.0%	3.8%	0.0%	3.7%
tr|I3LC73|I3LC73	33	24	16	0	4	1	4	14	88	71	75	0	5	2	27	49	39.1%	23.7%	18.0%	0.0%	5.5%	1.0%	7.9%	21.3%
tr|F2Z5L7|F2Z5L7	9	4	6	0	0	2	0	4	14	32	47	0	0	2	0	17	32.7%	14.8%	24.0%	0.0%	0.0%	4.6%	0.0%	14.4%
tr|F1RY92|F1RY92	6	2	7	1	1	0	0	3	11	35	64	1	1	0	0	8	25.0%	11.6%	34.8%	4.3%	5.5%	0.0%	0.0%	19.5%
tr|I3LAE9|I3LAE9	6	2	7	1	1	0	0	3	11	35	64	1	1	0	0	8	25.0%	11.6%	34.8%	4.3%	5.5%	0.0%	0.0%	19.5%
tr|F1SI88|F1SI88	14	4	1	1	1	0	0	2	42	4	1	1	1	0	0	2	28.4%	8.3%	1.9%	2.1%	2.1%	0.0%	0.0%	5.7%
Q2EN81	9	4	3	1	0	1	0	0	36	10	16	1	0	1	0	0	43.2%	24.9%	14.6%	8.0%	0.0%	8.0%	0.0%	0.0%
tr|I3LPB8|I3LPB8	7	4	1	0	1	0	0	4	12	11	4	0	1	0	0	6	24.8%	18.5%	7.0%	0.0%	4.3%	0.0%	0.0%	16.9%
tr|F1SHH7|F1SHH7	4	3	2	1	0	0	1	1	4	19	12	1	0	0	1	1	16.6%	11.3%	5.0%	5.5%	0.0%	0.0%	2.9%	5.5%
tr|F1S9J9|F1S9J9	3	3	1	0	1	0	0	0	3	20	1	0	1	0	0	0	4.6%	1.9%	1.5%	0.0%	1.2%	0.0%	0.0%	0.0%
tr|F1S1G1|F1S1G1	2	2	2	0	0	1	0	0	6	17	8	0	0	1	0	0	5.7%	5.7%	6.4%	0.0%	0.0%	5.7%	0.0%	0.0%
Q5S1U1	34	18	19	3	7	1	4	11	265	134	222	5	11	1	108	239	92.3%	80.7%	62.8%	20.3%	41.1%	4.8%	27.1%	59.9%
tr|I3L5D5|I3L5D5	32	7	35	0	4	0	12	63	67	27	170	0	4	0	101	434	26.6%	6.4%	26.0%	0.0%	4.8%	0.0%	13.3%	43.0%
A4Z6H1	13	6	3	0	1	1	0	1	18	29	10	0	1	1	0	1	25.4%	14.3%	9.2%	0.0%	2.2%	2.2%	0.0%	4.2%
tr|I3L7T4|I3L7T4	15	6	5	1	1	0	0	0	31	16	34	1	1	0	0	0	25.3%	8.0%	5.0%	1.4%	1.4%	0.0%	0.0%	0.0%
Q95342	7	7	2	1	2	0	1	2	16	55	5	1	2	0	1	5	34.6%	39.4%	24.0%	10.6%	22.1%	0.0%	12.5%	22.1%
tr|I3LDC7|I3LDC7	17	7	8	0	1	2	1	6	37	59	54	0	1	3	6	9	47.0%	21.7%	23.6%	0.0%	4.0%	3.0%	5.4%	16.1%
tr|F1SIK9|F1SIK9	5	4	9	1	0	0	1	8	18	6	41	1	0	0	2	32	18.0%	20.6%	22.8%	4.1%	0.0%	0.0%	8.6%	30.5%
tr|I3L742|I3L742	10	4	4	0	1	0	1	7	15	9	207	0	1	0	9	34	34.6%	11.6%	12.9%	0.0%	3.1%	0.0%	3.1%	19.9%
tr|I3LTR9|I3LTR9	7	2	2	0	1	0	0	2	22	2	14	0	1	0	0	6	52.4%	25.8%	21.8%	0.0%	6.5%	0.0%	0.0%	23.4%
tr|F1RIT0|F1RIT0	2	2	0	1	0	0	0	0	3	21	0	1	0	0	0	0	25.4%	11.0%	0.0%	5.7%	0.0%	0.0%	0.0%	0.0%
P68266	2	2	0	1	0	0	0	0	3	21	0	1	0	0	0	0	25.4%	11.0%	0.0%	5.7%	0.0%	0.0%	0.0%	0.0%
tr|F1SG10|F1SG10	7	4	2	0	0	1	0	0	19	5	8	0	0	1	0	0	25.5%	14.8%	6.9%	0.0%	0.0%	5.3%	0.0%	0.0%

	Peptide Count								Spectral Count								Sequence Coverage							
	Whole Cell	Whole Cell	Whole Cell	Cilia Fraction	Cilia Fraction	Cilia Fraction	Cilia Fraction	Cilia Fraction	Whole Cell	Whole Cell	Whole Cell	Cilia Fraction	Cilia Fraction	Cilia Fraction	Cilia Fraction	Cilia Fraction	Whole Cell	Whole Cell	Whole Cell	Cilia Fraction	Cilia Fraction	Cilia Fraction	Cilia Fraction	Cilia Fraction
Sample#	1	2	6	4	3	5	7	8	1	2	6	4	3	5	7	8	1	2	6	4	3	5	7	8
**Accession**																								
tr|F1RTH3|F1RTH3	6	2	6	1	0	0	0	0	10	14	21	1	0	0	0	0	15.5%	5.9%	14.4%	2.6%	0.0%	0.0%	0.0%	0.0%
tr|K7GP58|K7GP58	6	2	6	1	0	0	0	0	10	14	21	1	0	0	0	0	15.6%	5.9%	14.4%	2.6%	0.0%	0.0%	0.0%	0.0%
tr|F1S543|F1S543	11	12	2	0	2	1	1	1	32	41	20	0	2	1	23	5	47.7%	45.6%	9.1%	0.0%	12.5%	4.0%	6.1%	6.1%
tr|F1SVA0|F1SVA0	2	2	0	1	0	0	2	2	2	47	0	2	0	0	28	35	17.0%	5.5%	0.0%	5.5%	0.0%	0.0%	10.0%	10.0%
tr|F1RI15|F1RI15	41	17	22	0	3	1	3	7	85	40	296	0	4	1	3	34	44.5%	20.7%	36.3%	0.0%	6.0%	1.9%	6.5%	14.8%
tr|F1SBU7|F1SBU7	18	6	13	1	1	0	0	0	34	16	70	1	1	0	0	0	27.3%	7.5%	11.4%	1.4%	1.4%	0.0%	0.0%	0.0%
tr|F1RRC0|F1RRC0	11	1	16	0	1	0	11	18	24	1	260	0	1	0	87	103	18.7%	1.5%	25.7%	0.0%	1.8%	0.0%	19.0%	27.7%
tr|I3LEZ7|I3LEZ7	9	4	0	0	0	1	2	3	18	7	0	0	0	1	4	8	12.3%	4.2%	0.0%	0.0%	0.0%	2.1%	4.6%	4.6%
tr|I3LEQ7|I3LEQ7	8	3	4	0	1	0	1	2	11	14	23	0	1	0	1	2	30.0%	11.4%	16.3%	0.0%	4.0%	0.0%	5.1%	12.0%
Q06AB3	8	2	3	0	1	0	0	2	16	9	23	0	1	0	0	3	52.6%	13.0%	15.7%	0.0%	6.1%	0.0%	0.0%	13.5%
tr|I3LNC4|I3LNC4	8	3	4	0	1	0	1	2	11	14	23	0	1	0	1	2	28.9%	11.0%	15.7%	0.0%	3.9%	0.0%	5.0%	11.6%
tr|I3L769|I3L769	5	2	0	0	0	1	0	1	18	7	0	0	0	1	0	1	13.6%	2.2%	0.0%	0.0%	0.0%	3.5%	0.0%	2.0%
tr|F1SKL2|F1SKL2	1	3	3	0	0	1	0	1	1	24	9	0	0	1	0	1	4.7%	12.5%	15.5%	0.0%	0.0%	4.4%	0.0%	14.5%
tr|F2Z5M2|F2Z5M2	8	2	7	0	1	1	3	7	42	9	50	0	1	1	197	127	59.8%	27.8%	40.2%	0.0%	10.3%	10.3%	27.8%	40.2%
tr|K7GMM3|K7GMM3	8	2	7	0	1	1	3	7	42	9	50	0	1	1	197	127	70.7%	32.9%	47.6%	0.0%	12.2%	12.2%	32.9%	47.6%
P04163	8	2	7	0	1	1	3	7	42	9	50	0	1	1	196	127	60.4%	28.1%	40.6%	0.0%	10.4%	10.4%	28.1%	40.6%
tr|F1S0D9|F1S0D9	12	5	6	0	2	0	2	6	21	30	29	0	2	0	2	25	21.8%	13.4%	15.6%	0.0%	5.8%	0.0%	7.4%	13.6%
tr|F1SF78|F1SF78	10	4	3	1	1	0	0	0	37	14	5	1	1	0	0	0	27.9%	8.0%	5.6%	2.4%	2.4%	0.0%	0.0%	0.0%
tr|I3LRP1|I3LRP1	15	3	11	0	1	0	1	3	62	15	19	0	3	0	5	7	25.3%	4.9%	27.6%	0.0%	3.8%	0.0%	3.2%	6.5%
tr|K7GMY3|K7GMY3	2	1	2	1	1	1	0	0	2	127	10	3	1	1	0	0	14.3%	16.7%	33.3%	16.7%	16.7%	16.7%	0.0%	0.0%
tr|F1RX64|F1RX64	4	1	0	0	0	1	1	4	19	7	0	0	0	1	14	14	20.9%	4.7%	0.0%	0.0%	0.0%	4.7%	9.8%	16.3%
tr|F1SGJ3|F1SGJ3	13	3	9	1	0	0	6	15	22	4	84	1	0	0	44	60	26.9%	7.2%	17.5%	1.8%	0.0%	0.0%	19.7%	34.4%
tr|F1S409|F1S409	9	0	3	0	1	0	2	2	26	0	7	0	1	0	9	3	15.2%	0.0%	8.4%	0.0%	3.0%	0.0%	5.5%	7.8%
tr|F1RGD9|F1RGD9	8	3	2	0	1	0	1	3	16	10	35	0	1	0	1	6	26.1%	7.1%	5.7%	0.0%	2.8%	0.0%	2.8%	13.0%
tr|F1RZ96|F1RZ96	3	4	0	0	1	0	0	0	8	18	0	0	1	0	0	0	3.0%	5.0%	0.0%	0.0%	2.0%	0.0%	0.0%	0.0%
tr|F1S3A3|F1S3A3	1	1	0	0	1	0	0	0	6	20	0	0	1	0	0	0	2.8%	3.8%	0.0%	0.0%	2.8%	0.0%	0.0%	0.0%
tr|F1SUQ8|F1SUQ8	8	5	2	0	1	0	0	0	9	17	7	0	1	0	0	0	20.5%	15.3%	8.4%	0.0%	2.6%	0.0%	0.0%	0.0%
tr|K7GQI9|K7GQI9	3	2	3	1	1	1	0	0	3	128	15	3	1	1	0	0	25.2%	27.1%	40.2%	13.1%	13.1%	13.1%	0.0%	0.0%
tr|F1RYY5|F1RYY5	16	5	4	1	0	1	3	10	36	43	49	2	0	1	13	58	25.1%	7.9%	6.2%	2.0%	0.0%	1.1%	7.4%	15.6%
tr|F1S764|F1S764	15	7	8	1	0	1	0	0	33	46	46	1	0	2	0	0	27.4%	9.0%	11.9%	2.3%	0.0%	2.3%	0.0%	0.0%
tr|F1SDD5|F1SDD5	15	3	21	0	1	0	9	21	20	7	164	0	1	0	149	283	33.7%	8.5%	44.8%	0.0%	3.1%	0.0%	31.2%	50.4%
tr|F1S1A2|F1S1A2	1	2	0	0	1	0	1	1	3	24	0	0	1	0	2	2	5.7%	5.7%	0.0%	0.0%	5.7%	0.0%	5.7%	5.7%
tr|F1S8D4|F1S8D4	8	2	0	0	1	0	0	1	20	7	0	0	1	0	0	2	30.1%	6.1%	0.0%	0.0%	2.9%	0.0%	0.0%	4.4%
A5GFZ5	6	2	0	0	0	1	1	0	12	15	0	0	0	1	2	0	28.2%	9.7%	0.0%	0.0%	0.0%	8.5%	4.6%	0.0%
tr|I3L5Y0|I3L5Y0	7	2	0	0	0	1	0	1	20	7	0	0	0	1	0	1	16.4%	2.3%	0.0%	0.0%	0.0%	3.6%	0.0%	2.1%
tr|F1SDN6|F1SDN6	4	6	3	0	1	0	0	1	10	17	7	0	1	0	0	1	7.7%	11.1%	5.7%	0.0%	2.2%	0.0%	0.0%	4.0%
tr|F1STE8|F1STE8	27	9	2	0	4	0	0	6	79	58	12	0	5	0	0	11	29.9%	8.9%	3.4%	0.0%	6.5%	0.0%	0.0%	7.5%
tr|I3L8K4|I3L8K4	27	9	2	0	4	0	0	6	79	58	12	0	5	0	0	11	29.9%	8.9%	3.4%	0.0%	6.5%	0.0%	0.0%	7.5%
tr|I3LBT8|I3LBT8	4	2	3	1	0	0	2	2	10	18	42	1	0	0	15	14	22.9%	3.8%	12.1%	3.3%	0.0%	0.0%	8.8%	8.8%
tr|F1S891|F1S891	4	2	3	1	0	0	2	2	10	18	42	1	0	0	15	14	23.1%	3.8%	12.2%	3.4%	0.0%	0.0%	8.8%	8.8%
tr|F1SSG5|F1SSG5	7	6	1	0	0	1	1	0	19	9	1	0	0	1	1	0	29.2%	12.5%	3.3%	0.0%	0.0%	5.0%	2.5%	0.0%
tr|F1RK74|F1RK74	7	3	1	1	0	0	0	1	23	5	4	1	0	0	0	1	17.9%	8.8%	3.6%	2.6%	0.0%	0.0%	0.0%	3.9%
tr|F1RRD3|F1RRD3	9	6	6	0	0	1	0	1	20	8	15	0	0	1	0	1	15.1%	10.4%	7.7%	0.0%	0.0%	3.2%	0.0%	3.2%
A5A761	8	3	3	0	1	0	0	1	25	3	34	0	1	0	0	1	33.2%	18.3%	13.0%	0.0%	10.6%	0.0%	0.0%	10.6%
Q29554	26	5	13	0	1	2	0	3	74	11	114	0	1	2	0	5	47.1%	13.2%	20.3%	0.0%	3.4%	5.1%	0.0%	3.7%
tr|I3LLI8|I3LLI8	22	10	16	0	4	1	4	12	123	21	102	0	4	1	214	236	44.8%	46.1%	28.4%	0.0%	19.8%	8.2%	25.4%	31.9%
tr|F1S6R7|F1S6R7	18	13	10	1	2	1	1	8	127	133	61	1	4	4	17	43	47.0%	36.4%	26.0%	3.2%	3.2%	3.2%	4.1%	21.5%
tr|K7GQY9|K7GQY9	4	2	3	1	1	0	4	4	49	9	26	1	1	0	59	101	41.5%	41.5%	41.5%	11.7%	11.7%	0.0%	38.3%	38.3%
tr|I3LUJ5|I3LUJ5	10	6	6	0	2	0	2	3	27	31	23	0	2	0	26	36	39.8%	28.5%	34.0%	0.0%	8.1%	0.0%	15.9%	14.9%
A1E295	8	4	6	0	1	1	2	3	32	26	41	0	1	1	6	20	34.6%	20.9%	22.1%	0.0%	3.3%	3.3%	14.3%	10.7%
tr|F1SLI3|F1SLI3	7	6	2	0	0	1	1	4	16	13	7	0	0	1	1	24	7.4%	6.1%	4.0%	0.0%	0.0%	1.9%	2.1%	6.0%
tr|F1RSJ7|F1RSJ7	3	5	2	0	1	0	0	2	3	26	5	0	1	0	0	5	3.0%	5.1%	4.1%	0.0%	1.3%	0.0%	0.0%	4.5%
tr|F2Z572|F2Z572	9	3	3	0	0	1	0	0	23	6	10	0	0	1	0	0	32.7%	8.5%	11.0%	0.0%	0.0%	5.3%	0.0%	0.0%
tr|F1RT92|F1RT92	12	1	5	0	1	0	0	1	28	1	88	0	1	0	0	1	43.6%	5.1%	24.6%	0.0%	5.1%	0.0%	0.0%	5.5%
tr|I3L9M7|I3L9M7	12	2	6	0	1	0	0	0	26	3	90	0	1	0	0	0	32.4%	7.9%	21.3%	0.0%	3.5%	0.0%	0.0%	0.0%
tr|I3L995|I3L995	12	2	6	0	1	0	0	0	26	3	90	0	1	0	0	0	36.4%	8.9%	23.9%	0.0%	3.9%	0.0%	0.0%	0.0%
tr|I3L9J4|I3L9J4	17	7	7	0	3	2	5	9	82	93	80	0	3	3	44	43	49.7%	37.9%	21.2%	0.0%	17.3%	7.7%	23.6%	33.5%
P00889	18	10	3	1	3	3	1	1	89	271	22	2	3	7	5	1	37.7%	26.3%	8.8%	2.6%	10.3%	9.9%	6.5%	6.5%
tr|Q53DY5|Q53DY5	12	9	5	0	2	1	0	1	32	88	69	0	2	2	0	7	17.2%	27.6%	16.7%	0.0%	10.9%	5.9%	0.0%	5.9%
tr|I3LSI3|I3LSI3	13	6	5	0	2	0	2	8	49	11	22	0	2	0	6	24	57.1%	30.4%	17.8%	0.0%	14.2%	0.0%	12.1%	36.8%
tr|F1S8Y2|F1S8Y2	15	8	0	0	2	0	2	3	29	31	0	0	2	0	5	5	22.8%	8.9%	0.0%	0.0%	4.5%	0.0%	4.0%	6.5%
tr|F1S1F4|F1S1F4	5	3	0	0	0	1	1	1	14	16	0	0	0	1	3	20	2.4%	1.4%	0.0%	0.0%	0.0%	0.9%	0.9%	0.9%
tr|I3LAE8|I3LAE8	8	2	1	1	0	0	1	1	22	8	2	1	0	0	3	3	23.3%	5.9%	3.3%	3.7%	0.0%	0.0%	3.9%	3.9%
tr|F2Z5V4|F2Z5V4	7	1	0	0	0	1	0	1	26	4	0	0	0	1	0	3	48.0%	3.9%	0.0%	0.0%	0.0%	3.9%	0.0%	8.3%
tr|F1S4D7|F1S4D7	14	0	2	0	1	0	0	0	30	0	9	0	1	0	0	0	25.8%	0.0%	3.7%	0.0%	2.9%	0.0%	0.0%	0.0%
tr|F1SPY2|F1SPY2	8	3	5	0	1	0	0	0	18	12	38	0	1	0	0	0	27.7%	7.7%	12.3%	0.0%	4.1%	0.0%	0.0%	0.0%
tr|K7GND4|K7GND4	7	5	4	1	1	1	0	0	20	132	19	3	1	1	0	0	41.6%	35.7%	29.7%	7.6%	7.6%	7.6%	0.0%	0.0%
tr|K7GNA5|K7GNA5	6	5	4	0	1	0	2	3	36	26	31	0	2	0	34	36	26.0%	26.0%	20.1%	0.0%	4.5%	0.0%	11.8%	15.9%
tr|I3LD43|I3LD43	8	4	12	0	1	0	2	3	15	16	80	0	1	0	22	21	46.4%	14.5%	38.3%	0.0%	3.8%	0.0%	22.1%	27.2%
tr|F1RMH7|F1RMH7	7	5	3	0	1	0	2	1	11	20	18	0	1	0	4	2	11.8%	5.7%	5.9%	0.0%	1.6%	0.0%	3.4%	2.1%
tr|F2Z5J4|F2Z5J4	12	2	3	0	1	0	1	1	28	3	8	0	1	0	1	2	55.2%	5.5%	7.5%	0.0%	5.5%	0.0%	6.3%	5.5%
tr|F1S0B9|F1S0B9	5	1	2	0	1	0	0	0	11	20	9	0	1	0	0	0	47.9%	3.2%	12.9%	0.0%	3.2%	0.0%	0.0%	0.0%
tr|F1RI01|F1RI01	7	7	2	0	0	1	0	0	21	10	12	0	0	1	0	0	22.1%	35.8%	9.3%	0.0%	0.0%	6.4%	0.0%	0.0%
tr|F1RYM4|F1RYM4	13	3	6	0	0	1	0	0	22	9	23	0	0	1	0	0	26.6%	5.3%	10.5%	0.0%	0.0%	1.9%	0.0%	0.0%
tr|K7GNR6|K7GNR6	6	4	8	1	1	1	1	1	8	148	60	3	1	1	1	1	43.2%	29.0%	52.7%	8.3%	8.3%	8.3%	13.6%	13.6%
tr|F1SKF1|F1SKF1	11	5	6	1	2	1	1	6	20	105	37	1	2	1	9	31	26.0%	14.5%	17.8%	2.3%	8.0%	2.3%	4.1%	16.2%
P33198	27	13	26	0	1	2	4	3	72	85	335	0	2	3	18	14	54.4%	43.2%	40.6%	0.0%	7.4%	6.2%	15.0%	12.8%
tr|F1SK00|F1SK00	27	13	26	0	1	2	4	3	72	85	335	0	2	3	18	14	55.4%	44.1%	41.4%	0.0%	7.5%	6.3%	15.3%	13.1%
tr|F1SMZ7|F1SMZ7	65	36	27	4	7	7	16	14	1011	561	310	5	17	28	75	62	77.0%	61.4%	44.2%	7.1%	11.8%	9.7%	37.2%	37.0%
Q08094	11	7	5	0	2	0	2	3	31	32	22	0	2	0	26	36	54.1%	42.2%	23.0%	0.0%	8.4%	0.0%	16.6%	15.5%
tr|K7GK75|K7GK75	8	5	1	1	1	0	0	2	23	40	13	1	1	0	0	43	75.4%	42.1%	19.3%	21.1%	19.3%	0.0%	0.0%	21.1%

	Peptide Count								Spectral Count								Sequence Coverage							
	Whole Cell	Whole Cell	Whole Cell	Cilia Fraction	Cilia Fraction	Cilia Fraction	Cilia Fraction	Cilia Fraction	Whole Cell	Whole Cell	Whole Cell	Cilia Fraction	Cilia Fraction	Cilia Fraction	Cilia Fraction	Cilia Fraction	Whole Cell	Whole Cell	Whole Cell	Cilia Fraction	Cilia Fraction	Cilia Fraction	Cilia Fraction	Cilia Fraction
Sample#	1	2	6	4	3	5	7	8	1	2	6	4	3	5	7	8	1	2	6	4	3	5	7	8
**Accession**																								
tr|F1SPF9|F1SPF9	23	5	6	0	1	1	5	2	53	10	25	0	1	1	25	3	59.5%	15.8%	13.9%	0.0%	2.4%	2.4%	23.3%	9.2%
tr|I3LIM2|I3LIM2	18	7	3	0	2	0	0	4	32	31	13	0	2	0	0	10	41.5%	14.0%	10.1%	0.0%	4.5%	0.0%	0.0%	18.0%
tr|I3L918|I3L918	6	4	2	0	1	1	1	0	9	86	7	0	2	1	1	0	40.8%	16.5%	13.1%	0.0%	5.8%	5.8%	5.8%	0.0%
tr|F2Z568|F2Z568	7	4	0	0	2	0	0	2	36	28	0	0	2	0	0	20	35.7%	52.9%	0.0%	0.0%	18.6%	0.0%	0.0%	18.6%
tr|F2Z4Y8|F2Z4Y8	10	4	5	0	1	0	1	2	24	8	35	0	1	0	16	22	44.3%	24.1%	35.4%	0.0%	6.3%	0.0%	11.4%	18.4%
tr|F1SKQ0|F1SKQ0	14	4	14	0	1	0	5	3	24	8	85	0	1	0	14	26	21.7%	6.0%	21.9%	0.0%	1.6%	0.0%	7.7%	4.3%
tr|I3LGH6|I3LGH6	7	5	3	1	0	0	2	1	21	11	16	1	0	0	7	1	21.3%	12.1%	8.7%	2.4%	0.0%	0.0%	4.4%	3.7%
tr|F1RWJ6|F1RWJ6	12	5	12	0	1	0	0	5	24	8	59	0	1	0	0	16	22.8%	7.7%	19.9%	0.0%	3.0%	0.0%	0.0%	15.9%
tr|F1SGD1|F1SGD1	16	1	3	0	1	0	2	1	31	1	11	0	1	0	3	2	37.3%	2.1%	6.9%	0.0%	2.3%	0.0%	6.1%	2.7%
tr|I3LR66|I3LR66	16	1	3	0	1	0	2	1	31	1	11	0	1	0	3	2	36.7%	2.1%	6.8%	0.0%	2.2%	0.0%	6.0%	2.7%
P60982	7	2	1	0	0	1	0	1	26	6	3	0	0	1	0	2	24.8%	7.3%	6.7%	0.0%	0.0%	7.3%	0.0%	5.5%
tr|F2Z5I9|F2Z5I9	7	2	1	0	0	1	0	1	26	6	3	0	0	1	0	2	25.0%	7.3%	6.7%	0.0%	0.0%	7.3%	0.0%	5.5%
tr|I3LLF6|I3LLF6	1	3	2	0	1	0	0	0	1	31	7	0	1	0	0	0	6.2%	9.6%	6.9%	0.0%	6.2%	0.0%	0.0%	0.0%
tr|I3LG52|I3LG52	1	3	2	0	1	0	0	0	1	31	7	0	1	0	0	0	5.9%	9.2%	6.6%	0.0%	5.9%	0.0%	0.0%	0.0%
tr|F1SA50|F1SA50	30	15	18	0	2	0	4	3	81	80	111	0	5	0	37	6	38.9%	22.6%	25.7%	0.0%	3.0%	0.0%	8.1%	7.0%
tr|F1RYY6|F1RYY6	13	7	8	1	3	0	2	2	47	114	77	1	4	0	7	14	30.9%	19.0%	18.7%	3.3%	18.4%	0.0%	8.0%	14.8%
Q2EN76	15	10	4	1	3	0	4	8	146	82	18	2	5	0	123	70	65.8%	61.2%	17.1%	5.9%	18.4%	0.0%	29.6%	48.7%
tr|F1SSL6|F1SSL6	7	5	3	0	0	1	0	2	17	16	23	0	0	1	0	16	22.7%	19.2%	8.6%	0.0%	0.0%	3.5%	0.0%	10.2%
tr|F1SA35|F1SA35	8	2	5	0	1	0	0	3	29	4	51	0	1	0	0	10	15.8%	7.2%	8.6%	0.0%	2.9%	0.0%	0.0%	5.7%
P04175	12	4	2	1	0	0	1	0	23	10	14	1	0	0	3	0	18.3%	6.8%	3.2%	1.6%	0.0%	0.0%	3.1%	0.0%
tr|F1SLZ4|F1SLZ4	7	6	2	1	2	2	0	0	23	249	10	2	2	4	0	0	35.2%	28.9%	14.5%	7.5%	15.7%	15.7%	0.0%	0.0%
tr|I3LBB3|I3LBB3	7	6	2	1	2	2	0	0	23	249	10	2	2	4	0	0	26.7%	21.9%	11.0%	5.7%	11.9%	11.9%	0.0%	0.0%
tr|F1SNL7|F1SNL7	19	4	14	0	1	0	6	15	57	12	77	0	2	0	44	123	41.1%	20.0%	28.2%	0.0%	6.1%	0.0%	27.4%	51.6%
tr|K7GRY0|K7GRY0	42	18	18	0	6	1	13	18	175	103	127	0	7	1	98	71	44.5%	21.8%	19.8%	0.0%	10.5%	1.6%	21.8%	26.2%
tr|F1RWX8|F1RWX8	41	19	17	0	6	1	13	18	174	105	117	0	7	1	99	69	38.6%	22.0%	17.1%	0.0%	9.6%	1.5%	19.8%	23.8%
tr|I3LIC2|I3LIC2	46	11	21	0	4	2	1	1	129	116	142	0	4	3	6	1	38.9%	11.7%	19.5%	0.0%	4.4%	1.9%	2.2%	1.5%
tr|F1SV90|F1SV90	8	6	4	0	1	0	3	3	23	12	19	0	1	0	13	3	37.8%	30.2%	17.5%	0.0%	7.4%	0.0%	13.5%	20.3%
tr|F1RU52|F1RU52	9	5	3	1	0	0	2	1	24	11	16	1	0	0	7	1	25.9%	11.1%	8.0%	2.2%	0.0%	0.0%	4.1%	3.4%
tr|F1S5J1|F1S5J1	47	11	21	0	4	2	1	1	130	116	142	0	4	3	6	1	39.8%	11.7%	19.5%	0.0%	4.5%	1.9%	2.2%	1.5%
tr|K7GLL2|K7GLL2	10	7	9	1	1	1	1	1	25	152	64	3	1	1	1	1	48.8%	34.1%	40.1%	5.6%	5.6%	5.6%	9.1%	9.1%
tr|F1S956|F1S956	4	3	5	1	1	0	1	2	9	62	30	1	1	0	2	2	23.7%	10.3%	22.1%	6.1%	6.1%	0.0%	6.1%	14.1%
tr|F1SPY4|F1SPY4	9	3	4	0	0	1	1	4	16	20	13	0	0	1	1	13	26.1%	9.7%	8.3%	0.0%	0.0%	1.9%	3.0%	12.5%
tr|I3LUM9|I3LUM9	9	3	4	0	0	1	1	4	16	20	13	0	0	1	1	13	26.1%	9.7%	8.3%	0.0%	0.0%	1.9%	3.0%	12.5%
tr|F1SBF9|F1SBF9	9	4	7	1	0	0	0	3	20	16	22	1	0	0	0	9	18.5%	5.2%	6.8%	1.7%	0.0%	0.0%	0.0%	7.6%
tr|K7GP28|K7GP28	13	7	9	1	1	1	1	1	29	152	64	3	1	1	1	1	49.5%	29.4%	34.5%	4.8%	4.8%	4.8%	7.8%	7.8%
tr|F1RUI1|F1RUI1	13	7	9	1	1	1	1	1	29	152	64	3	1	1	1	1	55.6%	33.0%	38.7%	5.4%	5.4%	5.4%	8.8%	8.8%
tr|I3LP37|I3LP37	28	5	4	0	2	0	1	5	57	16	17	0	2	0	1	14	36.2%	7.8%	7.4%	0.0%	4.2%	0.0%	1.3%	8.3%
tr|I3LH11|I3LH11	28	5	4	0	2	0	1	5	57	16	17	0	2	0	1	14	37.4%	8.1%	7.7%	0.0%	4.3%	0.0%	1.4%	8.6%
tr|F1SKN5|F1SKN5	10	3	6	0	1	0	1	1	15	22	40	0	1	0	1	8	27.3%	4.1%	11.7%	0.0%	2.3%	0.0%	2.8%	2.1%
P52552	6	5	4	0	1	0	0	0	27	10	56	0	1	0	0	0	36.2%	37.0%	29.9%	0.0%	8.7%	0.0%	0.0%	0.0%
tr|K7GNI9|K7GNI9	22	10	9	1	2	0	2	12	76	36	72	1	2	0	9	44	71.2%	42.0%	31.4%	7.1%	10.6%	0.0%	13.3%	53.5%
tr|F1SED0|F1SED0	8	6	6	0	1	0	1	0	26	12	36	0	1	0	5	0	38.2%	20.8%	19.9%	0.0%	5.1%	0.0%	5.1%	0.0%
Q4JHS0	10	3	1	0	0	1	0	0	24	14	2	0	0	1	0	0	54.7%	15.6%	13.0%	0.0%	0.0%	9.9%	0.0%	0.0%
tr|F1SAC1|F1SAC1	12	4	3	0	2	0	3	3	73	44	11	0	3	0	18	11	46.7%	22.8%	8.0%	0.0%	14.5%	0.0%	15.9%	14.5%
tr|F1S814|F1S814	14	2	7	0	1	0	0	2	32	7	34	0	1	0	0	4	40.6%	6.2%	12.5%	0.0%	4.4%	0.0%	0.0%	10.7%
tr|F1RZR0|F1RZR0	14	7	4	0	1	0	0	0	23	16	17	0	1	0	0	0	7.0%	2.4%	3.2%	0.0%	0.4%	0.0%	0.0%	0.0%
tr|F1SII4|F1SII4	32	18	10	0	3	1	10	8	101	95	62	0	3	2	81	26	40.7%	23.2%	16.6%	0.0%	7.1%	1.8%	18.1%	17.4%
tr|I3L9N6|I3L9N6	56	31	23	4	5	6	14	12	894	423	276	5	9	19	73	60	71.0%	56.0%	40.3%	7.9%	10.2%	7.9%	36.5%	36.3%
tr|K7GRJ0|K7GRJ0	23	11	11	1	2	0	3	13	82	38	82	1	2	0	10	45	75.2%	47.5%	38.7%	6.7%	10.1%	0.0%	20.2%	58.4%
tr|I3LMH4|I3LMH4	25	12	15	1	2	0	0	7	69	94	49	1	3	0	0	16	19.2%	8.3%	12.9%	1.4%	2.3%	0.0%	0.0%	7.9%
tr|F1SGS6|F1SGS6	12	1	2	0	0	1	3	5	40	1	2	0	0	1	24	29	23.7%	1.6%	5.5%	0.0%	0.0%	4.2%	11.1%	15.5%
tr|I3L704|I3L704	5	7	1	0	1	0	2	4	13	28	3	0	1	0	20	11	20.8%	29.7%	5.8%	0.0%	6.7%	0.0%	13.7%	18.8%
Q29384	6	4	2	1	0	0	2	2	14	27	19	1	0	0	10	14	55.2%	40.9%	14.3%	13.0%	0.0%	0.0%	27.3%	27.3%
P37111	13	4	2	1	0	0	0	5	19	22	11	1	0	0	0	19	42.5%	14.5%	7.1%	5.4%	0.0%	0.0%	0.0%	18.9%
tr|F1SK12|F1SK12	38	11	35	0	1	2	2	9	60	64	212	0	1	2	3	30	22.5%	4.8%	21.6%	0.0%	0.4%	1.2%	2.1%	8.5%
P35750	8	2	2	0	1	0	0	4	15	27	7	0	1	0	0	11	14.4%	2.9%	2.2%	0.0%	4.1%	0.0%	0.0%	12.9%
Q29558	15	4	2	1	0	0	2	5	36	6	21	1	0	0	3	7	39.5%	10.6%	6.1%	5.0%	0.0%	0.0%	7.0%	14.2%
tr|F1RQM4|F1RQM4	16	3	2	1	0	0	2	5	37	5	21	1	0	0	3	7	42.7%	9.3%	6.0%	4.9%	0.0%	0.0%	6.9%	13.9%
tr|F1SP46|F1SP46	3	5	1	0	1	0	1	3	9	34	29	0	1	0	1	12	15.1%	23.3%	6.8%	0.0%	8.2%	0.0%	8.2%	16.4%
tr|F2Z5V1|F2Z5V1	1	4	0	0	1	0	0	1	1	42	0	0	1	0	0	4	20.3%	21.9%	0.0%	0.0%	15.6%	0.0%	0.0%	15.6%
tr|F1SUG7|F1SUG7	22	11	4	0	2	0	0	0	46	41	14	0	2	0	0	0	26.9%	14.0%	5.6%	0.0%	4.3%	0.0%	0.0%	0.0%
tr|K7GLK5|K7GLK5	3	8	2	0	1	0	1	1	6	38	45	0	1	0	1	3	22.5%	52.7%	16.3%	0.0%	9.3%	0.0%	9.3%	9.3%
tr|F1SLF6|F1SLF6	23	10	14	0	2	0	7	9	165	15	79	0	4	0	34	51	57.8%	30.4%	28.5%	0.0%	2.9%	0.0%	16.8%	31.7%
P00503	15	3	4	0	1	0	2	4	27	18	20	0	1	0	5	18	52.5%	10.9%	10.2%	0.0%	4.8%	0.0%	9.9%	15.3%
tr|F1S5A8|F1S5A8	17	8	5	0	2	0	2	0	42	49	21	0	2	0	3	0	29.9%	9.1%	6.3%	0.0%	6.0%	0.0%	5.9%	0.0%
tr|I3L816|I3L816	9	7	4	0	1	0	3	4	33	13	31	0	1	0	151	101	25.3%	23.6%	11.8%	0.0%	2.4%	0.0%	12.3%	10.6%
tr|K7GPC4|K7GPC4	10	7	3	2	3	0	1	4	27	250	8	3	3	0	1	12	22.9%	12.6%	6.8%	4.0%	7.3%	0.0%	4.3%	6.3%
tr|K7GMS2|K7GMS2	11	7	3	2	3	0	1	4	28	250	8	3	3	0	1	12	24.1%	12.1%	6.5%	3.8%	7.0%	0.0%	4.1%	6.0%
tr|F1RWT2|F1RWT2	11	7	3	2	3	0	1	4	28	250	8	3	3	0	1	12	24.1%	12.0%	6.5%	3.8%	7.0%	0.0%	4.1%	6.0%
tr|F1S418|F1S418	16	7	7	0	1	0	1	1	64	29	215	0	2	0	1	9	52.1%	21.1%	24.1%	0.0%	5.4%	0.0%	5.4%	4.2%
tr|F2Z5H9|F2Z5H9	9	8	4	0	1	0	4	5	33	14	31	0	1	0	152	102	23.8%	26.7%	11.1%	0.0%	2.2%	0.0%	16.0%	14.5%
tr|F1RPN9|F1RPN9	10	4	6	0	0	1	0	1	20	27	23	0	0	1	0	1	31.1%	8.2%	16.0%	0.0%	0.0%	1.5%	0.0%	6.0%
tr|F1RK02|F1RK02	25	11	5	1	3	1	3	6	74	256	12	2	3	2	6	25	45.1%	13.7%	11.1%	1.9%	6.9%	1.9%	6.3%	13.8%
tr|I3LEA6|I3LEA6	4	2	0	0	0	1	0	1	7	41	0	0	0	1	0	1	27.4%	9.7%	0.0%	0.0%	0.0%	9.7%	0.0%	9.7%
tr|F2Z5J8|F2Z5J8	11	4	5	0	0	1	1	1	19	30	36	0	0	1	2	3	40.3%	4.1%	24.4%	0.0%	0.0%	4.1%	4.1%	6.3%
tr|I3L770|I3L770	30	7	5	0	2	1	1	2	67	85	25	0	2	1	1	5	12.8%	2.3%	2.2%	0.0%	0.5%	0.3%	0.3%	1.1%
tr|F1RUH0|F1RUH0	30	7	5	0	2	1	1	2	67	85	25	0	2	1	1	5	12.8%	2.3%	2.2%	0.0%	0.5%	0.3%	0.3%	1.1%
tr|K7GNJ7|K7GNJ7	30	7	5	0	2	1	1	2	67	85	25	0	2	1	1	5	12.8%	2.3%	2.2%	0.0%	0.5%	0.3%	0.3%	1.1%
tr|K7GRM2|K7GRM2	4	4	1	0	1	0	0	2	15	37	13	0	1	0	0	43	36.5%	19.0%	17.5%	0.0%	17.5%	0.0%	0.0%	19.0%
tr|F1SEG5|F1SEG5	5	9	2	0	1	0	1	3	13	39	45	0	1	0	1	12	28.1%	54.8%	14.4%	0.0%	8.2%	0.0%	8.2%	16.4%
tr|K7GSD9|K7GSD9	5	9	2	0	1	0	1	3	13	39	45	0	1	0	1	12	28.3%	55.2%	14.5%	0.0%	8.3%	0.0%	8.3%	16.6%

	Peptide Count								Spectral Count								Sequence Coverage							
	Whole Cell	Whole Cell	Whole Cell	Cilia Fraction	Cilia Fraction	Cilia Fraction	Cilia Fraction	Cilia Fraction	Whole Cell	Whole Cell	Whole Cell	Cilia Fraction	Cilia Fraction	Cilia Fraction	Cilia Fraction	Cilia Fraction	Whole Cell	Whole Cell	Whole Cell	Cilia Fraction	Cilia Fraction	Cilia Fraction	Cilia Fraction	Cilia Fraction
Sample#	1	2	6	4	3	5	7	8	1	2	6	4	3	5	7	8	1	2	6	4	3	5	7	8
**Accession**																								
tr|F1ST02|F1ST02	8	5	3	1	0	0	3	3	21	32	28	1	0	0	32	16	45.1%	33.0%	14.0%	7.6%	0.0%	0.0%	25.0%	25.0%
tr|I3LBH4|I3LBH4	3	2	0	0	1	0	1	2	30	24	0	0	1	0	2	4	12.6%	4.7%	0.0%	0.0%	4.7%	0.0%	4.7%	12.6%
tr|F2Z5M3|F2Z5M3	18	10	11	0	3	1	3	15	217	56	123	0	4	1	21	75	66.2%	42.9%	22.7%	0.0%	23.4%	7.8%	26.0%	46.1%
tr|F1SA66|F1SA66	8	7	1	0	0	1	0	0	14	45	4	0	0	1	0	0	16.7%	11.2%	2.6%	0.0%	0.0%	3.5%	0.0%	0.0%
tr|F1SQR8|F1SQR8	22	6	8	0	1	0	2	7	49	12	52	0	1	0	3	31	44.7%	11.9%	17.2%	0.0%	4.9%	0.0%	5.6%	15.7%
tr|F1RQB6|F1RQB6	15	4	8	0	1	0	1	2	34	27	69	0	1	0	8	3	32.1%	11.4%	19.8%	0.0%	2.2%	0.0%	3.7%	8.2%
tr|F1SMW9|F1SMW9	11	5	6	0	1	0	1	0	38	23	23	0	1	0	2	0	38.8%	10.4%	12.9%	0.0%	2.0%	0.0%	4.2%	0.0%
tr|F1SGH5|F1SGH5	10	2	7	0	1	0	0	0	33	28	79	0	1	0	0	0	35.8%	10.8%	24.2%	0.0%	4.4%	0.0%	0.0%	0.0%
tr|F1S9K5|F1S9K5	50	15	32	0	2	1	6	16	149	96	170	0	3	1	79	94	36.1%	13.7%	21.7%	0.0%	2.7%	1.3%	8.7%	19.0%
P00348	9	4	7	0	1	0	2	2	43	19	18	0	1	0	12	5	51.0%	18.2%	21.0%	0.0%	8.3%	0.0%	16.6%	17.2%
tr|F1RLQ2|F1RLQ2	22	6	28	0	1	0	6	3	48	15	169	0	1	0	61	47	36.3%	9.6%	43.4%	0.0%	2.3%	0.0%	10.5%	7.2%
Q3ZD69	22	6	29	0	1	0	6	3	48	15	172	0	1	0	61	47	36.3%	9.6%	44.7%	0.0%	2.3%	0.0%	10.5%	7.2%
tr|F1SS26|F1SS26	14	4	9	0	1	0	0	3	23	40	48	0	1	0	0	10	16.6%	3.6%	9.4%	0.0%	0.8%	0.0%	0.0%	5.1%
tr|F1SKI5|F1SKI5	22	6	8	0	1	0	3	8	51	13	23	0	1	0	19	28	45.7%	23.0%	17.1%	0.0%	5.3%	0.0%	8.9%	28.2%
P62831	7	6	3	1	0	0	2	6	42	25	53	1	0	0	28	16	45.0%	40.7%	30.7%	14.3%	0.0%	0.0%	25.0%	19.3%
tr|I3LRH2|I3LRH2	34	26	17	2	3	0	8	11	225	144	132	2	3	0	50	34	34.0%	29.3%	23.1%	2.9%	5.9%	0.0%	14.9%	18.4%
tr|I3LCC2|I3LCC2	14	3	1	1	1	0	2	3	32	120	1	1	1	0	5	3	26.5%	4.7%	1.6%	1.2%	2.6%	0.0%	5.4%	8.7%
tr|D0G6X4|D0G6X4	13	5	5	0	0	1	1	2	35	43	17	0	0	1	5	8	25.2%	23.3%	7.6%	0.0%	0.0%	5.5%	4.3%	7.6%
tr|F1RGJ3|F1RGJ3	34	16	28	1	0	1	2	2	90	67	173	1	0	1	2	2	43.3%	27.5%	32.7%	2.1%	0.0%	2.1%	5.7%	5.2%
tr|F1S8T1|F1S8T1	5	9	2	0	1	0	0	0	18	66	6	0	1	0	0	0	8.8%	10.0%	3.5%	0.0%	3.4%	0.0%	0.0%	0.0%
P46405	11	8	6	0	0	1	2	3	37	50	63	0	0	1	26	24	58.3%	48.5%	39.4%	0.0%	0.0%	8.3%	18.9%	26.5%
tr|Q06A94|Q06A94	32	12	7	0	1	1	4	11	190	76	44	0	2	1	86	42	58.8%	36.6%	29.4%	0.0%	5.0%	5.3%	20.9%	38.1%
tr|F1SJX1|F1SJX1	20	5	8	0	1	0	0	2	71	18	45	0	1	0	0	5	57.7%	28.5%	26.1%	0.0%	8.1%	0.0%	0.0%	13.8%
tr|F1SQS2|F1SQS2	9	4	4	0	1	0	0	1	25	74	8	0	1	0	0	1	56.8%	14.2%	32.6%	0.0%	7.4%	0.0%	0.0%	13.7%
tr|F1RGI7|F1RGI7	22	8	15	0	1	0	1	1	66	37	76	0	1	0	1	1	34.6%	9.8%	17.9%	0.0%	2.9%	0.0%	2.1%	2.1%
tr|F1SDX9|F1SDX9	9	6	4	0	1	0	0	5	99	17	56	0	1	0	0	16	58.1%	38.9%	19.2%	0.0%	5.6%	0.0%	0.0%	34.8%
tr|I3LA63|I3LA63	3	1	0	1	0	0	0	0	5	115	0	1	0	0	0	0	10.6%	3.8%	0.0%	3.8%	0.0%	0.0%	0.0%	0.0%
tr|F1SMV6|F1SMV6	25	11	12	0	1	0	7	10	185	42	58	0	1	0	45	28	51.2%	39.7%	24.9%	0.0%	6.0%	0.0%	23.8%	30.1%
tr|I3LTH8|I3LTH8	19	14	19	0	1	0	4	6	259	70	113	0	1	0	140	49	54.4%	38.9%	48.1%	0.0%	7.4%	0.0%	17.0%	29.7%
tr|F1RRY2|F1RRY2	19	14	19	0	1	0	4	6	259	70	113	0	1	0	140	49	52.4%	37.4%	46.3%	0.0%	7.1%	0.0%	16.3%	28.6%
tr|I3LUP6|I3LUP6	19	14	20	0	1	0	4	6	259	70	115	0	1	0	140	49	50.5%	36.1%	45.2%	0.0%	6.9%	0.0%	15.7%	27.5%
P11708	15	10	7	0	0	0	1	6	60	93	121	0	0	0	5	37	45.5%	34.1%	28.4%	0.0%	0.0%	0.0%	4.5%	18.0%
tr|F1S827|F1S827	18	18	5	0	0	0	0	1	54	97	14	0	0	0	0	1	35.4%	36.9%	22.1%	0.0%	0.0%	0.0%	0.0%	3.7%
Q29099	16	11	10	0	0	0	1	8	113	29	61	0	0	0	17	43	43.8%	33.2%	26.0%	0.0%	0.0%	0.0%	4.1%	21.5%
tr|F1SIJ9|F1SIJ9	15	8	5	0	0	0	5	11	79	27	49	0	0	0	13	79	48.9%	27.0%	18.0%	0.0%	0.0%	0.0%	22.8%	43.9%
P16276	30	8	23	0	0	0	1	2	70	21	579	0	0	0	2	4	43.5%	14.6%	27.3%	0.0%	0.0%	0.0%	2.8%	4.7%
tr|F1SRC5|F1SRC5	30	8	24	0	0	0	1	2	70	21	589	0	0	0	2	4	44.2%	14.8%	28.7%	0.0%	0.0%	0.0%	2.9%	4.8%
tr|F2Z546|F2Z546	1	6	4	0	0	0	0	0	2	84	27	0	0	0	0	0	8.7%	26.5%	14.3%	0.0%	0.0%	0.0%	0.0%	0.0%
tr|I3LBY9|I3LBY9	13	8	7	0	0	0	1	6	52	33	122	0	0	0	5	37	46.5%	32.5%	35.1%	0.0%	0.0%	0.0%	5.5%	22.1%
tr|I3LSG1|I3LSG1	13	6	4	0	0	0	0	0	28	57	10	0	0	0	0	0	16.6%	6.2%	5.2%	0.0%	0.0%	0.0%	0.0%	0.0%
tr|I3LPB7|I3LPB7	13	6	4	0	0	0	0	0	28	57	10	0	0	0	0	0	16.3%	6.1%	5.1%	0.0%	0.0%	0.0%	0.0%	0.0%
tr|F1RJL2|F1RJL2	13	6	4	0	0	0	0	0	28	57	10	0	0	0	0	0	16.3%	6.1%	5.1%	0.0%	0.0%	0.0%	0.0%	0.0%
tr|I3LDH3|I3LDH3	22	7	13	0	0	0	1	1	39	45	59	0	0	0	1	1	29.0%	8.6%	14.8%	0.0%	0.0%	0.0%	1.3%	1.4%
tr|F1SIX1|F1SIX1	3	4	1	0	0	0	0	0	9	70	2	0	0	0	0	0	22.7%	23.9%	6.7%	0.0%	0.0%	0.0%	0.0%	0.0%
tr|F1SHD6|F1SHD6	15	3	2	0	0	0	2	3	49	29	8	0	0	0	10	29	60.9%	15.6%	12.4%	0.0%	0.0%	0.0%	26.7%	31.6%
tr|I3LPP1|I3LPP1	12	5	10	0	0	0	1	3	68	10	105	0	0	0	5	13	37.8%	15.9%	23.6%	0.0%	0.0%	0.0%	4.5%	15.0%
tr|I3L6Q5|I3L6Q5	7	6	4	0	0	0	0	1	20	58	30	0	0	0	0	3	18.7%	17.2%	13.8%	0.0%	0.0%	0.0%	0.0%	6.4%
tr|F1RMZ8|F1RMZ8	19	2	5	0	0	0	12	19	70	2	23	0	0	0	47	69	39.9%	6.3%	11.7%	0.0%	0.0%	0.0%	37.8%	47.6%
tr|F1SEV8|F1SEV8	12	7	9	0	0	0	6	6	34	36	26	0	0	0	142	61	22.4%	15.9%	10.0%	0.0%	0.0%	0.0%	16.2%	11.8%
P61288	10	7	2	0	0	0	2	5	30	40	6	0	0	0	5	36	61.0%	52.9%	27.9%	0.0%	0.0%	0.0%	8.7%	47.7%
Q710C4	19	10	15	0	0	0	3	7	41	29	110	0	0	0	15	47	38.7%	23.1%	31.0%	0.0%	0.0%	0.0%	10.9%	24.1%
tr|I3LT08|I3LT08	11	7	9	0	0	0	6	6	33	36	26	0	0	0	142	61	17.9%	15.7%	9.9%	0.0%	0.0%	0.0%	16.0%	11.7%
tr|F1S4Y7|F1S4Y7	18	9	14	0	0	0	3	7	40	26	104	0	0	0	15	47	36.9%	21.0%	29.1%	0.0%	0.0%	0.0%	11.1%	24.6%
tr|Q2VTP6|Q2VTP6	7	3	1	0	0	0	1	3	44	20	25	0	0	0	1	6	77.8%	29.6%	13.0%	0.0%	0.0%	0.0%	13.0%	45.4%
P10173	11	5	9	0	0	0	0	2	53	10	81	0	0	0	0	10	37.8%	15.9%	23.6%	0.0%	0.0%	0.0%	0.0%	10.5%
tr|F1STR6|F1STR6	12	3	5	0	0	0	1	1	22	38	35	0	0	0	1	2	44.4%	9.1%	16.0%	0.0%	0.0%	0.0%	3.6%	3.6%
tr|K7GQE0|K7GQE0	1	1	0	0	0	0	0	0	1	58	0	0	0	0	0	0	5.4%	2.5%	0.0%	0.0%	0.0%	0.0%	0.0%	0.0%
tr|F1RKG8|F1RKG8	14	5	9	0	0	0	4	4	48	9	104	0	0	0	41	75	71.1%	38.0%	52.9%	0.0%	0.0%	0.0%	32.1%	27.3%
tr|B5APU3|B5APU3	18	3	9	0	0	0	2	1	52	3	51	0	0	0	77	9	48.5%	9.6%	24.6%	0.0%	0.0%	0.0%	7.4%	2.8%
tr|F1SIU2|F1SIU2	12	7	9	0	0	0	1	3	40	15	56	0	0	0	7	17	41.4%	34.6%	22.8%	0.0%	0.0%	0.0%	8.0%	9.5%
tr|F1SIV3|F1SIV3	12	7	10	0	0	0	1	3	40	15	61	0	0	0	7	17	44.1%	36.8%	26.7%	0.0%	0.0%	0.0%	8.5%	10.1%
tr|F1SAP4|F1SAP4	16	2	4	0	0	0	0	1	43	12	18	0	0	0	0	2	37.1%	4.6%	6.6%	0.0%	0.0%	0.0%	0.0%	3.3%
tr|F1RFI1|F1RFI1	16	10	15	0	0	0	0	2	25	30	102	0	0	0	0	4	45.8%	25.4%	37.2%	0.0%	0.0%	0.0%	0.0%	8.6%
tr|F1SGN4|F1SGN4	15	4	0	0	0	0	0	0	24	31	0	0	0	0	0	0	23.4%	4.1%	0.0%	0.0%	0.0%	0.0%	0.0%	0.0%
tr|F1RNL4|F1RNL4	14	2	4	0	0	0	3	8	39	14	32	0	0	0	32	26	30.2%	7.5%	16.8%	0.0%	0.0%	0.0%	12.1%	22.4%
P28768	5	5	2	0	0	0	1	1	8	45	49	0	0	0	17	19	38.9%	27.1%	10.4%	0.0%	0.0%	0.0%	10.4%	10.4%
tr|F1RR69|F1RR69	5	4	5	0	0	0	2	0	16	37	29	0	0	0	2	0	29.1%	11.1%	24.2%	0.0%	0.0%	0.0%	8.3%	0.0%
tr|F1RST0|F1RST0	25	5	9	0	0	0	0	0	39	14	41	0	0	0	0	0	30.4%	6.4%	20.5%	0.0%	0.0%	0.0%	0.0%	0.0%
tr|F1SID4|F1SID4	15	3	6	0	0	0	1	3	39	13	46	0	0	0	9	8	41.0%	9.2%	18.2%	0.0%	0.0%	0.0%	2.7%	7.8%
tr|K7GSM4|K7GSM4	12	5	2	0	0	0	0	0	21	31	6	0	0	0	0	0	9.1%	2.4%	1.3%	0.0%	0.0%	0.0%	0.0%	0.0%
tr|F1RU02|F1RU02	12	5	2	0	0	0	0	0	21	31	6	0	0	0	0	0	9.2%	2.4%	1.3%	0.0%	0.0%	0.0%	0.0%	0.0%
tr|F1S1B5|F1S1B5	21	4	7	0	0	0	0	0	48	4	26	0	0	0	0	0	16.8%	2.7%	4.5%	0.0%	0.0%	0.0%	0.0%	0.0%
Q6DUB7	11	1	1	0	0	0	0	1	49	2	4	0	0	0	0	4	38.9%	8.7%	6.0%	0.0%	0.0%	0.0%	0.0%	9.4%
tr|F1SAF0|F1SAF0	8	4	4	0	0	0	0	2	32	19	20	0	0	0	0	3	25.1%	10.6%	7.1%	0.0%	0.0%	0.0%	0.0%	8.6%
tr|I3LPB5|I3LPB5	9	2	3	0	0	0	0	0	48	3	15	0	0	0	0	0	53.8%	18.5%	19.6%	0.0%	0.0%	0.0%	0.0%	0.0%
tr|F1SSH8|F1SSH8	14	4	6	0	0	0	0	0	26	25	17	0	0	0	0	0	24.0%	3.9%	8.1%	0.0%	0.0%	0.0%	0.0%	0.0%
tr|F2Z5H8|F2Z5H8	9	3	1	0	0	0	1	1	40	10	6	0	0	0	1	7	51.4%	19.5%	4.8%	0.0%	0.0%	0.0%	10.5%	13.3%
P09623	7	4	4	0	0	0	0	2	31	19	20	0	0	0	0	3	20.2%	10.6%	7.1%	0.0%	0.0%	0.0%	0.0%	8.6%
tr|F1SSN5|F1SSN5	26	0	12	0	0	0	0	2	50	0	49	0	0	0	0	4	22.4%	0.0%	12.7%	0.0%	0.0%	0.0%	0.0%	2.8%
tr|G8ENL4|G8ENL4	9	4	7	0	0	0	2	2	31	18	120	0	0	0	49	37	22.4%	16.6%	12.2%	0.0%	0.0%	0.0%	9.3%	9.3%
tr|F1SLE5|F1SLE5	10	2	4	0	0	0	3	4	47	2	21	0	0	0	10	13	32.0%	6.9%	10.4%	0.0%	0.0%	0.0%	9.7%	18.0%

	Peptide Count								Spectral Count								Sequence Coverage							
	Whole Cell	Whole Cell	Whole Cell	Cilia Fraction	Cilia Fraction	Cilia Fraction	Cilia Fraction	Cilia Fraction	Whole Cell	Whole Cell	Whole Cell	Cilia Fraction	Cilia Fraction	Cilia Fraction	Cilia Fraction	Cilia Fraction	Whole Cell	Whole Cell	Whole Cell	Cilia Fraction	Cilia Fraction	Cilia Fraction	Cilia Fraction	Cilia Fraction
Sample#	1	2	6	4	3	5	7	8	1	2	6	4	3	5	7	8	1	2	6	4	3	5	7	8
Accession																								
tr|F1SGS9|F1SGS9	23	5	10	0	0	0	2	4	41	8	50	0	0	0	10	19	56.9%	10.2%	26.6%	0.0%	0.0%	0.0%	9.5%	16.7%
tr|F1SIA4|F1SIA4	6	6	6	0	0	0	1	4	8	40	31	0	0	0	2	10	17.2%	13.8%	12.7%	0.0%	0.0%	0.0%	3.9%	11.4%
tr|F1RM15|F1RM15	16	3	7	0	0	0	1	5	39	9	69	0	0	0	3	8	17.3%	2.8%	8.3%	0.0%	0.0%	0.0%	1.4%	8.1%
tr|F1RIE2|F1RIE2	14	6	5	0	0	0	2	12	37	10	137	0	0	0	6	27	14.0%	5.6%	7.9%	0.0%	0.0%	0.0%	4.2%	14.9%
tr|I3L7P7|I3L7P7	5	5	2	0	0	0	0	2	23	24	39	0	0	0	0	13	47.1%	47.1%	26.5%	0.0%	0.0%	0.0%	0.0%	20.6%
P60662	8	4	5	0	0	0	1	3	40	5	46	0	0	0	109	86	42.4%	32.5%	31.1%	0.0%	0.0%	0.0%	6.0%	16.6%
tr|F1RII4|F1RII4	8	3	10	0	0	0	2	3	29	16	64	0	0	0	122	43	71.7%	22.5%	55.1%	0.0%	0.0%	0.0%	11.6%	33.3%
tr|I3LNA4|I3LNA4	7	3	4	0	0	0	0	2	36	9	24	0	0	0	0	12	28.6%	11.1%	8.7%	0.0%	0.0%	0.0%	0.0%	5.8%
tr|F2Z5J1|F2Z5J1	14	4	5	0	0	0	1	2	30	15	30	0	0	0	6	4	35.7%	7.5%	14.1%	0.0%	0.0%	0.0%	3.4%	7.5%
tr|I3LVK3|I3LVK3	15	3	7	0	0	0	1	4	36	9	69	0	0	0	3	6	16.2%	2.9%	8.5%	0.0%	0.0%	0.0%	1.4%	6.9%
tr|F1SSG6|F1SSG6	10	4	5	0	0	0	0	0	20	25	45	0	0	0	0	0	40.0%	22.7%	15.4%	0.0%	0.0%	0.0%	0.0%	0.0%
P20305	13	9	13	0	0	0	6	15	27	17	68	0	0	0	102	104	33.8%	19.6%	18.7%	0.0%	0.0%	0.0%	16.6%	32.4%
tr|F1RQR0|F1RQR0	10	3	0	0	0	0	4	5	32	12	0	0	0	0	17	26	18.6%	7.0%	0.0%	0.0%	0.0%	0.0%	7.8%	12.2%
P12309	11	3	1	0	0	0	2	3	26	18	5	0	0	0	11	12	69.8%	23.6%	13.2%	0.0%	0.0%	0.0%	27.4%	38.7%
tr|I3LFV4|I3LFV4	11	5	6	0	0	0	2	4	27	17	45	0	0	0	10	17	39.5%	26.5%	29.0%	0.0%	0.0%	0.0%	11.1%	24.7%
tr|F1RI41|F1RI41	5	2	1	0	0	0	0	1	18	26	5	0	0	0	0	2	29.4%	9.2%	6.9%	0.0%	0.0%	0.0%	0.0%	6.4%
tr|F1RSC3|F1RSC3	3	2	1	0	0	0	1	0	10	33	13	0	0	0	4	0	7.5%	4.4%	3.3%	0.0%	0.0%	0.0%	3.1%	0.0%
tr|F1RYA3|F1RYA3	12	3	4	0	0	0	1	0	34	9	17	0	0	0	2	0	17.4%	3.5%	5.7%	0.0%	0.0%	0.0%	1.8%	0.0%
tr|F1S1G5|F1S1G5	3	4	1	0	0	0	0	1	12	31	2	0	0	0	0	1	8.2%	8.2%	2.4%	0.0%	0.0%	0.0%	0.0%	4.7%
Q52NJ4	16	3	7	0	0	0	6	9	35	7	32	0	0	0	17	65	60.4%	25.8%	43.4%	0.0%	0.0%	0.0%	46.2%	63.2%
tr|F1RJ25|F1RJ25	10	3	6	0	0	0	1	4	28	14	89	0	0	0	24	36	31.0%	16.8%	14.8%	0.0%	0.0%	0.0%	7.1%	14.8%
tr|I3LFC4|I3LFC4	9	1	4	0	0	0	0	4	18	24	16	0	0	0	0	24	27.0%	3.8%	17.2%	0.0%	0.0%	0.0%	0.0%	15.0%
O62839	20	5	8	0	0	0	2	3	34	8	36	0	0	0	10	16	49.7%	10.2%	22.8%	0.0%	0.0%	0.0%	9.5%	14.0%
tr|F1SIX2|F1SIX2	6	3	3	0	0	0	1	5	26	15	10	0	0	0	5	32	36.2%	19.7%	25.7%	0.0%	0.0%	0.0%	9.9%	29.6%
tr|F1SPH1|F1SPH1	14	1	3	0	0	0	0	2	37	4	51	0	0	0	0	6	43.3%	5.7%	16.7%	0.0%	0.0%	0.0%	0.0%	11.0%
tr|I3LP11|I3LP11	7	3	7	0	0	0	1	1	18	23	52	0	0	0	3	1	14.2%	4.7%	10.6%	0.0%	0.0%	0.0%	3.2%	4.5%
tr|I3L6H2|I3L6H2	5	2	0	0	0	0	1	1	30	10	0	0	0	0	5	1	40.7%	22.9%	0.0%	0.0%	0.0%	0.0%	21.4%	21.4%
tr|K7GSE0|K7GSE0	17	1	11	0	0	0	1	1	39	1	195	0	0	0	3	1	24.2%	3.2%	22.4%	0.0%	0.0%	0.0%	3.2%	4.3%
tr|F1S9F3|F1S9F3	17	1	11	0	0	0	1	1	39	1	195	0	0	0	3	1	26.2%	3.5%	24.3%	0.0%	0.0%	0.0%	3.5%	4.7%
tr|I3LAI1|I3LAI1	4	2	3	0	0	0	1	0	31	8	11	0	0	0	8	0	11.5%	9.2%	8.8%	0.0%	0.0%	0.0%	6.5%	0.0%
tr|K7GKK5|K7GKK5	5	3	6	0	0	0	1	3	22	17	197	0	0	0	2	12	38.0%	33.8%	31.0%	0.0%	0.0%	0.0%	19.7%	27.5%
tr|F1SQ14|F1SQ14	5	3	6	0	0	0	1	3	22	17	197	0	0	0	2	12	35.5%	31.6%	28.9%	0.0%	0.0%	0.0%	18.4%	25.7%
tr|F1SBH2|F1SBH2	16	5	4	0	0	0	0	2	33	6	14	0	0	0	0	2	26.1%	9.0%	6.4%	0.0%	0.0%	0.0%	0.0%	2.2%
P62197	14	2	5	0	0	0	0	1	35	3	20	0	0	0	0	1	40.4%	4.4%	17.0%	0.0%	0.0%	0.0%	0.0%	4.2%
P62895	7	5	4	0	0	0	0	0	18	20	8	0	0	0	0	0	56.2%	61.0%	32.4%	0.0%	0.0%	0.0%	0.0%	0.0%
tr|F1SIT7|F1SIT7	5	2	1	0	0	0	2	1	20	17	14	0	0	0	15	4	66.7%	28.9%	14.0%	0.0%	0.0%	0.0%	51.8%	37.7%
tr|F1SS25|F1SS25	14	1	7	0	0	0	1	3	36	1	33	0	0	0	1	5	40.9%	2.5%	19.3%	0.0%	0.0%	0.0%	5.8%	14.5%
tr|I3LCI0|I3LCI0	10	4	5	0	0	0	1	4	32	4	43	0	0	0	2	21	35.4%	22.5%	17.5%	0.0%	0.0%	0.0%	5.3%	18.2%
tr|F1S5Z3|F1S5Z3	21	4	6	0	0	0	2	3	32	4	21	0	0	0	4	12	45.7%	10.9%	9.1%	0.0%	0.0%	0.0%	5.4%	9.9%
tr|F2Z5B4|F2Z5B4	16	2	14	0	0	0	1	3	34	2	106	0	0	0	1	17	34.6%	8.4%	28.0%	0.0%	0.0%	0.0%	4.2%	14.2%
tr|F1S1X3|F1S1X3	16	6	6	0	0	0	0	3	23	13	25	0	0	0	0	5	32.7%	12.9%	17.7%	0.0%	0.0%	0.0%	0.0%	9.1%
tr|K7GM70|K7GM70	11	7	15	0	0	0	1	1	20	16	282	0	0	0	24	1	25.2%	9.8%	28.1%	0.0%	0.0%	0.0%	1.7%	1.3%
tr|K7GL83|K7GL83	11	7	15	0	0	0	1	1	20	16	282	0	0	0	24	1	24.6%	9.5%	27.5%	0.0%	0.0%	0.0%	1.7%	1.3%
tr|K7GP99|K7GP99	11	7	15	0	0	0	1	1	20	16	282	0	0	0	24	1	21.5%	8.3%	24.0%	0.0%	0.0%	0.0%	1.5%	1.1%
tr|I3L5S8|I3L5S8	9	5	3	0	0	0	0	2	26	10	15	0	0	0	0	2	46.5%	18.8%	20.0%	0.0%	0.0%	0.0%	0.0%	11.4%
tr|C1PIG4|C1PIG4	12	4	9	0	0	0	6	5	28	7	45	0	0	0	51	44	29.4%	13.5%	26.9%	0.0%	0.0%	0.0%	23.4%	19.5%
P67985	5	3	3	0	0	0	1	1	27	8	17	0	0	0	16	24	41.4%	39.1%	19.5%	0.0%	0.0%	0.0%	20.3%	20.3%
tr|F1SIP0|F1SIP0	12	3	10	0	0	0	2	7	31	4	76	0	0	0	3	34	46.0%	9.1%	32.4%	0.0%	0.0%	0.0%	8.3%	27.1%
tr|F1SFF8|F1SFF8	10	4	4	0	0	0	1	1	13	22	15	0	0	0	1	5	16.6%	5.2%	5.6%	0.0%	0.0%	0.0%	1.6%	4.2%
tr|F1S1J9|F1S1J9	12	3	2	0	0	0	1	1	31	4	3	0	0	0	1	1	32.1%	15.0%	8.3%	0.0%	0.0%	0.0%	5.5%	5.5%
tr|F1S0R7|F1S0R7	16	3	7	0	0	0	1	0	32	3	64	0	0	0	1	0	34.3%	5.4%	11.2%	0.0%	0.0%	0.0%	2.6%	0.0%
P29804	17	4	12	0	0	0	0	1	25	10	80	0	0	0	0	1	53.0%	14.1%	39.1%	0.0%	0.0%	0.0%	0.0%	8.0%
tr|I3LCI2|I3LCI2	17	4	12	0	0	0	0	1	25	10	80	0	0	0	0	1	52.8%	14.1%	39.0%	0.0%	0.0%	0.0%	0.0%	7.9%
tr|F1SDG7|F1SDG7	5	3	1	0	0	0	0	0	18	17	8	0	0	0	0	0	36.6%	20.9%	8.2%	0.0%	0.0%	0.0%	0.0%	0.0%
tr|F1SN47|F1SN47	10	3	2	0	0	0	0	0	22	13	8	0	0	0	0	0	28.5%	5.7%	6.0%	0.0%	0.0%	0.0%	0.0%	0.0%
tr|F1RK45|F1RK45	20	2	5	0	0	0	0	0	33	2	27	0	0	0	0	0	35.7%	4.3%	9.3%	0.0%	0.0%	0.0%	0.0%	0.0%
tr|F1S1G0|F1S1G0	9	2	3	0	0	0	2	2	31	3	14	0	0	0	7	4	27.1%	6.1%	7.2%	0.0%	0.0%	0.0%	8.5%	4.5%
tr|F1RUH7|F1RUH7	12	3	2	0	0	0	0	2	25	9	12	0	0	0	0	9	51.8%	16.7%	16.3%	0.0%	0.0%	0.0%	0.0%	12.2%
tr|F1SMB2|F1SMB2	8	4	3	0	0	0	1	1	27	7	79	0	0	0	4	2	14.4%	8.3%	7.9%	0.0%	0.0%	0.0%	2.6%	2.6%
tr|F1RVN1|F1RVN1	10	6	1	0	0	0	0	0	12	22	7	0	0	0	0	0	23.5%	10.3%	3.7%	0.0%	0.0%	0.0%	0.0%	0.0%
Q29361	6	6	3	0	0	0	0	0	15	19	13	0	0	0	0	0	22.8%	27.6%	22.0%	0.0%	0.0%	0.0%	0.0%	0.0%
tr|I3LNK5|I3LNK5	12	3	6	0	0	0	1	5	30	3	20	0	0	0	1	13	30.2%	5.5%	15.7%	0.0%	0.0%	0.0%	3.4%	13.4%
tr|F1SNT8|F1SNT8	18	1	5	0	0	0	0	3	28	5	13	0	0	0	0	8	22.9%	1.2%	5.3%	0.0%	0.0%	0.0%	0.0%	6.5%
tr|I3LQD3|I3LQD3	12	3	3	0	0	0	0	4	30	3	18	0	0	0	0	5	29.9%	11.8%	6.2%	0.0%	0.0%	0.0%	0.0%	18.6%
tr|F1SI06|F1SI06	14	5	8	0	0	0	0	4	24	9	49	0	0	0	0	8	15.5%	6.7%	10.0%	0.0%	0.0%	0.0%	0.0%	6.9%
tr|F1SE06|F1SE06	7	3	4	0	0	0	0	2	13	20	26	0	0	0	0	4	33.1%	11.2%	16.3%	0.0%	0.0%	0.0%	0.0%	16.3%
tr|F2Z5R7|F2Z5R7	3	5	1	0	0	0	0	1	4	29	2	0	0	0	0	1	39.3%	60.7%	14.3%	0.0%	0.0%	0.0%	0.0%	14.3%
tr|F1RVZ1|F1RVZ1	9	4	4	0	0	0	0	0	17	16	15	0	0	0	0	0	13.7%	10.0%	13.0%	0.0%	0.0%	0.0%	0.0%	0.0%
tr|F1RKI3|F1RKI3	5	2	1	0	0	0	1	1	25	7	2	0	0	0	80	27	52.4%	11.1%	11.1%	0.0%	0.0%	0.0%	11.1%	11.1%
tr|F1SI55|F1SI55	8	3	0	0	0	0	2	0	21	11	0	0	0	0	4	0	15.0%	13.8%	0.0%	0.0%	0.0%	0.0%	12.6%	0.0%
P29412	5	2	1	0	0	0	1	0	11	21	2	0	0	0	1	0	25.0%	9.8%	6.7%	0.0%	0.0%	0.0%	12.5%	0.0%
Q6QAP6	3	4	1	0	0	0	0	1	4	28	2	0	0	0	0	1	39.3%	41.1%	14.3%	0.0%	0.0%	0.0%	0.0%	14.3%
tr|F1STL7|F1STL7	1	2	0	0	0	0	0	0	2	30	0	0	0	0	0	0	2.9%	4.9%	0.0%	0.0%	0.0%	0.0%	0.0%	0.0%
tr|F1RR45|F1RR45	7	5	0	0	0	0	0	0	14	18	0	0	0	0	0	0	14.5%	12.8%	0.0%	0.0%	0.0%	0.0%	0.0%	0.0%
P04574	11	1	2	0	0	0	5	1	30	1	11	0	0	0	22	14	66.2%	7.1%	10.9%	0.0%	0.0%	0.0%	24.8%	9.0%
tr|I3LBE4|I3LBE4	8	3	2	0	0	0	1	3	28	3	6	0	0	0	2	15	23.2%	14.6%	5.3%	0.0%	0.0%	0.0%	5.3%	10.3%
tr|F1RPH1|F1RPH1	12	2	2	0	0	0	1	4	27	4	20	0	0	0	8	9	65.4%	13.4%	25.2%	0.0%	0.0%	0.0%	11.8%	41.7%
tr|F1S2Q3|F1S2Q3	11	1	11	0	0	0	3	4	30	1	83	0	0	0	5	19	42.5%	5.0%	37.0%	0.0%	0.0%	0.0%	17.4%	23.7%
tr|C5H0C6|C5H0C6	8	1	5	0	0	0	1	3	30	1	34	0	0	0	1	9	27.7%	3.0%	24.7%	0.0%	0.0%	0.0%	3.7%	10.7%
tr|C3RZ98|C3RZ98	8	4	4	0	0	0	1	1	16	15	10	0	0	0	3	1	17.3%	6.6%	7.4%	0.0%	0.0%	0.0%	4.9%	4.9%
tr|F1SAJ5|F1SAJ5	9	7	4	0	0	0	0	2	18	13	10	0	0	0	0	2	28.0%	19.0%	10.8%	0.0%	0.0%	0.0%	0.0%	8.1%

	Peptide Count								Spectral Count								Sequence Coverage							
	Whole Cell	Whole Cell	Whole Cell	Cilia Fraction	Cilia Fraction	Cilia Fraction	Cilia Fraction	Cilia Fraction	Whole Cell	Whole Cell	Whole Cell	Cilia Fraction	Cilia Fraction	Cilia Fraction	Cilia Fraction	Cilia Fraction	Whole Cell	Whole Cell	Whole Cell	Cilia Fraction	Cilia Fraction	Cilia Fraction	Cilia Fraction	Cilia Fraction
Sample#	1	2	6	4	3	5	7	8	1	2	6	4	3	5	7	8	1	2	6	4	3	5	7	8
**Accession**																								
tr|F1S5C5|F1S5C5	5	5	1	0	0	0	0	0	7	24	1	0	0	0	0	0	11.4%	9.5%	1.4%	0.0%	0.0%	0.0%	0.0%	0.0%
tr|I3LUE8|I3LUE8	6	3	4	0	0	0	0	0	13	18	11	0	0	0	0	0	15.6%	6.9%	9.9%	0.0%	0.0%	0.0%	0.0%	0.0%
tr|F1SMQ0|F1SMQ0	12	1	4	0	0	0	0	0	23	8	12	0	0	0	0	0	23.5%	1.4%	6.3%	0.0%	0.0%	0.0%	0.0%	0.0%
Q6UAQ8	8	2	8	0	0	0	0	0	27	4	66	0	0	0	0	0	30.6%	19.2%	37.3%	0.0%	0.0%	0.0%	0.0%	0.0%
A1XQU9	9	3	3	0	0	0	3	5	24	6	21	0	0	0	67	74	23.5%	36.1%	16.0%	0.0%	0.0%	0.0%	19.3%	22.7%
tr|F2Z539|F2Z539	9	3	3	0	0	0	3	5	24	6	21	0	0	0	67	74	23.5%	36.1%	16.0%	0.0%	0.0%	0.0%	19.3%	22.7%
tr|F2Z5N0|F2Z5N0	6	11	4	0	0	0	1	5	12	18	19	0	0	0	1	13	33.3%	43.5%	21.5%	0.0%	0.0%	0.0%	6.9%	26.0%
tr|J9JIM9|J9JIM9	13	3	7	0	0	0	1	4	26	4	79	0	0	0	8	8	25.8%	11.2%	18.4%	0.0%	0.0%	0.0%	5.3%	9.1%
Q2QLE3	13	3	7	0	0	0	1	4	26	4	79	0	0	0	8	8	25.7%	11.2%	18.3%	0.0%	0.0%	0.0%	5.2%	9.0%
A5GFS8	7	1	11	0	0	0	0	2	28	2	74	0	0	0	0	7	32.9%	5.8%	37.4%	0.0%	0.0%	0.0%	0.0%	13.6%
tr|I3LJY0|I3LJY0	2	3	1	0	0	0	0	1	3	27	2	0	0	0	0	1	25.0%	26.8%	14.3%	0.0%	0.0%	0.0%	0.0%	14.3%
tr|F1SLL6|F1SLL6	8	2	1	0	0	0	0	1	14	16	6	0	0	0	0	1	10.5%	2.4%	2.7%	0.0%	0.0%	0.0%	0.0%	2.7%
tr|F1RN65|F1RN65	2	1	1	0	0	0	1	0	14	16	14	0	0	0	1	0	17.6%	17.6%	17.6%	0.0%	0.0%	0.0%	17.6%	0.0%
tr|I3LKJ6|I3LKJ6	7	1	0	0	0	0	0	0	9	21	0	0	0	0	0	0	9.7%	1.9%	0.0%	0.0%	0.0%	0.0%	0.0%	0.0%
tr|F1ST49|F1ST49	8	8	3	0	0	0	0	0	9	21	13	0	0	0	0	0	14.7%	14.4%	4.9%	0.0%	0.0%	0.0%	0.0%	0.0%
tr|I3LHF0|I3LHF0	6	3	8	0	0	0	3	3	22	7	24	0	0	0	121	41	42.9%	34.7%	40.1%	0.0%	0.0%	0.0%	26.5%	26.5%
Q9GL51	5	6	5	0	0	0	4	4	6	23	35	0	0	0	27	18	14.9%	13.9%	11.7%	0.0%	0.0%	0.0%	14.6%	11.5%
tr|F2Z521|F2Z521	5	6	5	0	0	0	4	4	6	23	35	0	0	0	27	18	14.9%	13.9%	11.7%	0.0%	0.0%	0.0%	14.6%	11.5%
tr|I3LKT8|I3LKT8	10	1	1	0	0	0	0	2	28	1	6	0	0	0	0	5	45.9%	2.3%	1.8%	0.0%	0.0%	0.0%	0.0%	15.6%
tr|I3LS70|I3LS70	8	0	0	0	0	0	1	2	29	0	0	0	0	0	2	2	31.2%	0.0%	0.0%	0.0%	0.0%	0.0%	7.2%	7.2%
tr|I3L5X7|I3L5X7	3	2	0	0	0	0	0	1	25	4	0	0	0	0	0	4	32.1%	28.2%	0.0%	0.0%	0.0%	0.0%	0.0%	20.5%
tr|F1SB53|F1SB53	12	2	7	0	0	0	0	3	26	3	26	0	0	0	0	7	33.7%	5.1%	24.8%	0.0%	0.0%	0.0%	0.0%	11.6%
tr|F1S710|F1S710	11	3	5	0	0	0	0	2	23	6	14	0	0	0	0	4	47.0%	23.9%	17.4%	0.0%	0.0%	0.0%	0.0%	19.6%
tr|F1RHM8|F1RHM8	7	3	4	0	0	0	0	1	25	4	21	0	0	0	0	4	48.5%	21.8%	21.8%	0.0%	0.0%	0.0%	0.0%	11.2%
tr|D3K5N7|D3K5N7	11	1	3	0	0	0	0	1	27	2	32	0	0	0	0	3	33.7%	4.3%	18.1%	0.0%	0.0%	0.0%	0.0%	4.3%
tr|K7GRM6|K7GRM6	6	3	7	0	0	0	1	1	8	21	54	0	0	0	1	1	48.3%	23.2%	47.7%	0.0%	0.0%	0.0%	15.2%	15.2%
tr|F1SMZ9|F1SMZ9	14	2	12	0	0	0	1	1	27	2	69	0	0	0	1	1	14.0%	1.9%	12.2%	0.0%	0.0%	0.0%	1.5%	1.8%
tr|F1SPE9|F1SPE9	13	6	4	0	0	0	1	0	18	11	22	0	0	0	1	0	8.2%	4.8%	2.9%	0.0%	0.0%	0.0%	1.2%	0.0%
tr|K7GMN8|K7GMN8	15	3	11	0	0	0	0	1	22	7	65	0	0	0	0	1	50.7%	8.6%	35.7%	0.0%	0.0%	0.0%	0.0%	8.6%
tr|F1S8R2|F1S8R2	4	3	2	0	0	0	0	0	6	23	9	0	0	0	0	0	16.2%	10.3%	9.7%	0.0%	0.0%	0.0%	0.0%	0.0%
tr|I3LQK3|I3LQK3	4	1	2	0	0	0	1	3	27	1	8	0	0	0	13	65	17.9%	10.3%	7.6%	0.0%	0.0%	0.0%	7.6%	17.9%
Q9N1F5	8	2	9	0	0	0	3	5	20	8	33	0	0	0	29	25	40.7%	12.4%	33.6%	0.0%	0.0%	0.0%	17.0%	34.4%
tr|F1SL01|F1SL01	2	3	6	0	0	0	2	3	5	23	39	0	0	0	3	14	5.3%	10.0%	25.4%	0.0%	0.0%	0.0%	14.5%	25.7%
tr|I3LNI2|I3LNI2	2	3	6	0	0	0	2	3	5	23	39	0	0	0	3	14	5.3%	10.0%	25.3%	0.0%	0.0%	0.0%	14.4%	25.6%
tr|F1SHL3|F1SHL3	13	3	3	0	0	0	3	2	25	3	14	0	0	0	5	5	23.1%	4.4%	5.1%	0.0%	0.0%	0.0%	5.2%	3.5%
tr|I3LJ30|I3LJ30	11	2	6	0	0	0	0	2	25	3	21	0	0	0	0	2	27.9%	5.5%	20.0%	0.0%	0.0%	0.0%	0.0%	5.5%
tr|F1RVE8|F1RVE8	16	5	0	0	0	0	1	0	22	6	0	0	0	0	1	0	19.6%	6.2%	0.0%	0.0%	0.0%	0.0%	1.9%	0.0%
tr|F1SC98|F1SC98	11	4	5	0	0	0	0	1	24	4	13	0	0	0	0	1	11.1%	3.0%	10.8%	0.0%	0.0%	0.0%	0.0%	3.9%
tr|F1RL66|F1RL66	9	3	2	0	0	0	0	0	20	8	4	0	0	0	0	0	8.2%	2.6%	1.5%	0.0%	0.0%	0.0%	0.0%	0.0%
tr|F1RMT5|F1RMT5	8	6	2	0	0	0	0	0	8	20	9	0	0	0	0	0	8.8%	5.9%	2.8%	0.0%	0.0%	0.0%	0.0%	0.0%
tr|I3LUK9|I3LUK9	4	2	0	0	0	0	1	2	21	6	0	0	0	0	5	5	36.5%	15.7%	0.0%	0.0%	0.0%	0.0%	20.9%	15.7%
tr|F1S105|F1S105	4	2	0	0	0	0	1	2	21	6	0	0	0	0	5	5	28.6%	12.2%	0.0%	0.0%	0.0%	0.0%	16.3%	12.2%
Q06AA9	4	2	0	0	0	0	1	2	21	6	0	0	0	0	5	5	28.6%	12.2%	0.0%	0.0%	0.0%	0.0%	16.3%	12.2%
P63221	5	2	6	0	0	0	1	1	24	3	85	0	0	0	1	7	45.8%	32.5%	45.8%	0.0%	0.0%	0.0%	21.7%	21.7%
tr|K7GPJ3|K7GPJ3	12	3	7	0	0	0	0	1	18	9	41	0	0	0	0	3	14.9%	3.0%	8.0%	0.0%	0.0%	0.0%	0.0%	1.3%
tr|I3LKV0|I3LKV0	5	2	0	0	0	0	0	0	8	19	0	0	0	0	0	0	51.0%	22.5%	0.0%	0.0%	0.0%	0.0%	0.0%	0.0%
P62863	3	3	1	0	0	0	0	0	6	21	1	0	0	0	0	0	20.3%	20.3%	16.9%	0.0%	0.0%	0.0%	0.0%	0.0%
tr|F1RQU9|F1RQU9	3	3	1	0	0	0	0	0	6	21	1	0	0	0	0	0	9.0%	9.0%	7.5%	0.0%	0.0%	0.0%	0.0%	0.0%
tr|I3LMT3|I3LMT3	5	4	1	0	0	0	0	0	7	20	3	0	0	0	0	0	18.8%	9.6%	1.7%	0.0%	0.0%	0.0%	0.0%	0.0%
tr|I3LD01|I3LD01	0	2	1	0	0	0	0	0	0	27	3	0	0	0	0	0	0.0%	14.5%	7.8%	0.0%	0.0%	0.0%	0.0%	0.0%
tr|F1SM17|F1SM17	7	2	2	0	0	0	0	0	13	14	14	0	0	0	0	0	17.0%	4.1%	3.6%	0.0%	0.0%	0.0%	0.0%	0.0%
tr|F1SRB9|F1SRB9	9	4	6	0	0	0	1	7	21	5	27	0	0	0	1	41	20.9%	12.5%	11.1%	0.0%	0.0%	0.0%	3.0%	11.1%
tr|F2Z5G9|F2Z5G9	4	3	1	0	0	0	2	2	21	5	6	0	0	0	11	9	27.7%	27.7%	10.9%	0.0%	0.0%	0.0%	16.8%	27.7%
tr|F1RVC9|F1RVC9	4	3	2	0	0	0	0	4	7	19	6	0	0	0	0	11	18.8%	19.3%	16.3%	0.0%	0.0%	0.0%	0.0%	17.8%
tr|I3LAT6|I3LAT6	2	3	0	0	0	0	0	1	4	22	0	0	0	0	0	6	6.6%	8.0%	0.0%	0.0%	0.0%	0.0%	0.0%	3.6%
tr|F1S7E0|F1S7E0	13	0	1	0	0	0	2	2	26	0	7	0	0	0	2	4	54.9%	0.0%	3.9%	0.0%	0.0%	0.0%	14.9%	6.5%
tr|F1RMC3|F1RMC3	7	4	4	0	0	0	3	0	9	17	13	0	0	0	7	0	12.9%	4.6%	4.8%	0.0%	0.0%	0.0%	7.0%	0.0%
tr|I3LJC4|I3LJC4	7	4	4	0	0	0	3	0	9	17	13	0	0	0	7	0	12.9%	4.6%	4.8%	0.0%	0.0%	0.0%	7.0%	0.0%
tr|F1S848|F1S848	10	3	0	0	0	0	0	1	21	5	0	0	0	0	0	2	29.0%	8.4%	0.0%	0.0%	0.0%	0.0%	0.0%	5.7%
tr|F2Z528|F2Z528	11	3	6	0	0	0	1	1	23	3	30	0	0	0	2	1	56.3%	16.1%	28.7%	0.0%	0.0%	0.0%	9.2%	4.2%
Q29187	2	2	0	0	0	0	0	1	6	20	0	0	0	0	0	1	11.9%	11.9%	0.0%	0.0%	0.0%	0.0%	0.0%	10.9%
tr|F1S585|F1S585	8	6	8	0	0	0	0	1	14	12	209	0	0	0	0	1	16.5%	7.6%	14.8%	0.0%	0.0%	0.0%	0.0%	1.2%
tr|F1RTA3|F1RTA3	5	1	0	0	0	0	0	0	23	3	0	0	0	0	0	0	20.8%	3.1%	0.0%	0.0%	0.0%	0.0%	0.0%	0.0%
tr|F1RQW4|F1RQW4	6	2	1	0	0	0	0	0	7	19	3	0	0	0	0	0	7.9%	1.8%	1.6%	0.0%	0.0%	0.0%	0.0%	0.0%
tr|F1S7V9|F1S7V9	6	2	2	0	0	0	0	0	15	11	5	0	0	0	0	0	28.6%	6.2%	11.2%	0.0%	0.0%	0.0%	0.0%	0.0%
tr|F1RHF7|F1RHF7	6	3	3	0	0	0	0	0	8	18	9	0	0	0	0	0	13.4%	3.9%	4.3%	0.0%	0.0%	0.0%	0.0%	0.0%
tr|F1SUX4|F1SUX4	12	2	7	0	0	0	0	0	23	3	23	0	0	0	0	0	8.9%	1.8%	6.2%	0.0%	0.0%	0.0%	0.0%	0.0%
tr|I3L6D2|I3L6D2	6	2	3	0	0	0	2	6	20	5	17	0	0	0	71	37	36.8%	16.4%	22.4%	0.0%	0.0%	0.0%	17.9%	40.8%
tr|F2Z5U4|F2Z5U4	6	2	3	0	0	0	2	6	20	5	17	0	0	0	71	37	36.6%	16.3%	22.3%	0.0%	0.0%	0.0%	17.8%	40.6%
Q52NJ2	6	2	3	0	0	0	2	4	20	5	17	0	0	0	71	26	36.1%	16.1%	22.0%	0.0%	0.0%	0.0%	17.6%	29.8%
tr|I3LEY2|I3LEY2	8	2	8	0	0	0	3	5	23	2	62	0	0	0	82	58	26.9%	5.1%	15.8%	0.0%	0.0%	0.0%	12.4%	18.2%
tr|F1SU59|F1SU59	8	2	8	0	0	0	3	5	23	2	62	0	0	0	82	58	27.2%	5.2%	16.0%	0.0%	0.0%	0.0%	12.5%	18.4%
tr|I3LSS1|I3LSS1	3	1	2	0	0	0	1	3	22	3	4	0	0	0	5	29	16.7%	9.5%	13.8%	0.0%	0.0%	0.0%	7.1%	16.7%
tr|F1S458|F1S458	12	1	6	0	0	0	0	4	23	2	30	0	0	0	0	21	28.8%	2.1%	10.9%	0.0%	0.0%	0.0%	0.0%	10.2%
tr|I3LU39|I3LU39	2	2	4	0	0	0	1	3	17	8	34	0	0	0	14	6	19.3%	9.6%	25.3%	0.0%	0.0%	0.0%	9.6%	46.4%
tr|F1SNZ7|F1SNZ7	6	4	2	0	0	0	1	2	16	9	4	0	0	0	2	6	15.0%	18.5%	6.9%	0.0%	0.0%	0.0%	4.6%	8.1%
tr|F2Z5P3|F2Z5P3	2	2	0	0	0	0	0	2	19	6	0	0	0	0	0	5	12.2%	12.2%	0.0%	0.0%	0.0%	0.0%	0.0%	12.2%
tr|K7GQ68|K7GQ68	7	4	11	0	0	0	1	1	15	10	263	0	0	0	24	1	26.2%	10.3%	38.1%	0.0%	0.0%	0.0%	3.2%	2.4%
tr|F1RQQ7|F1RQQ7	4	2	0	0	0	0	0	0	7	18	0	0	0	0	0	0	8.8%	3.0%	0.0%	0.0%	0.0%	0.0%	0.0%	0.0%
O46374	5	4	1	0	0	0	0	0	7	18	2	0	0	0	0	0	4.6%	2.5%	1.0%	0.0%	0.0%	0.0%	0.0%	0.0%
tr|K7GPJ0|K7GPJ0	7	3	1	0	0	0	0	0	21	4	4	0	0	0	0	0	57.1%	29.4%	9.5%	0.0%	0.0%	0.0%	0.0%	0.0%

	Peptide Count								Spectral Count								Sequence Coverage							
	Whole Cell	Whole Cell	Whole Cell	Cilia Fraction	Cilia Fraction	Cilia Fraction	Cilia Fraction	Cilia Fraction	Whole Cell	Whole Cell	Whole Cell	Cilia Fraction	Cilia Fraction	Cilia Fraction	Cilia Fraction	Cilia Fraction	Whole Cell	Whole Cell	Whole Cell	Cilia Fraction	Cilia Fraction	Cilia Fraction	Cilia Fraction	Cilia Fraction
Sample#	1	2	6	4	3	5	7	8	1	2	6	4	3	5	7	8	1	2	6	4	3	5	7	8
**Accession**																								
tr|F1SU06|F1SU06	10	0	1	0	0	0	0	0	25	0	29	0	0	0	0	0	25.9%	0.0%	2.0%	0.0%	0.0%	0.0%	0.0%	0.0%
tr|F1S179|F1S179	5	3	1	0	0	0	0	3	12	12	1	0	0	0	0	51	54.2%	11.5%	9.2%	0.0%	0.0%	0.0%	0.0%	54.2%
tr|B8Y648|B8Y648	9	0	2	0	0	0	0	6	24	0	12	0	0	0	0	26	48.1%	0.0%	20.1%	0.0%	0.0%	0.0%	0.0%	41.6%
B2DCZ9	13	0	3	0	0	0	0	13	24	0	15	0	0	0	0	28	19.7%	0.0%	5.4%	0.0%	0.0%	0.0%	0.0%	23.6%
tr|F1RLP5|F1RLP5	13	0	3	0	0	0	0	13	24	0	15	0	0	0	0	28	19.7%	0.0%	5.4%	0.0%	0.0%	0.0%	0.0%	23.6%
tr|F1SM78|F1SM78	8	3	4	0	0	0	3	5	20	4	37	0	0	0	22	18	55.8%	29.7%	27.9%	0.0%	0.0%	0.0%	23.3%	57.0%
tr|F1RZ71|F1RZ71	8	4	4	0	0	0	2	1	13	11	60	0	0	0	19	8	18.6%	7.0%	15.2%	0.0%	0.0%	0.0%	7.2%	4.1%
P49666	3	4	0	0	0	0	0	1	13	11	0	0	0	0	0	5	17.7%	32.3%	0.0%	0.0%	0.0%	0.0%	0.0%	17.7%
tr|F1SC51|F1SC51	10	2	7	0	0	0	0	2	22	2	29	0	0	0	0	9	37.3%	9.7%	20.3%	0.0%	0.0%	0.0%	0.0%	10.9%
tr|F1RQZ9|F1RQZ9	8	4	9	0	0	0	3	2	15	9	48	0	0	0	7	4	15.0%	3.9%	14.9%	0.0%	0.0%	0.0%	5.1%	4.3%
tr|F1SKJ5|F1SKJ5	9	3	6	0	0	0	0	1	14	10	81	0	0	0	0	7	20.4%	7.7%	16.6%	0.0%	0.0%	0.0%	0.0%	4.6%
tr|F1S062|F1S062	8	4	5	0	0	0	1	0	17	7	28	0	0	0	3	0	36.1%	28.6%	21.8%	0.0%	0.0%	0.0%	5.5%	0.0%
tr|K7GLA7|K7GLA7	10	3	7	0	0	0	0	1	15	9	68	0	0	0	0	1	44.7%	13.3%	30.8%	0.0%	0.0%	0.0%	0.0%	9.4%
tr|I3L9E0|I3L9E0	0	2	0	0	0	0	0	0	0	24	0	0	0	0	0	0	0.0%	6.6%	0.0%	0.0%	0.0%	0.0%	0.0%	0.0%
tr|F1SLI6|F1SLI6	5	2	0	0	0	0	0	0	9	15	0	0	0	0	0	0	8.5%	1.9%	0.0%	0.0%	0.0%	0.0%	0.0%	0.0%
tr|I3LEJ2|I3LEJ2	0	2	0	0	0	0	0	0	0	24	0	0	0	0	0	0	0.0%	6.3%	0.0%	0.0%	0.0%	0.0%	0.0%	0.0%
tr|F1SFR0|F1SFR0	4	1	1	0	0	0	0	0	13	11	5	0	0	0	0	0	8.6%	2.1%	2.9%	0.0%	0.0%	0.0%	0.0%	0.0%
Q95281	3	2	1	0	0	0	0	0	13	11	87	0	0	0	0	0	16.2%	16.2%	11.2%	0.0%	0.0%	0.0%	0.0%	0.0%
tr|F2Z5V6|F2Z5V6	8	2	4	0	0	0	3	4	20	3	37	0	0	0	22	15	55.8%	12.2%	27.9%	0.0%	0.0%	0.0%	23.3%	39.5%
Q29221	5	2	1	0	0	0	1	3	20	3	8	0	0	0	1	13	19.2%	5.9%	3.5%	0.0%	0.0%	0.0%	5.6%	26.6%
Q6SEG5	10	1	4	0	0	0	0	6	22	1	11	0	0	0	0	13	73.5%	3.6%	15.7%	0.0%	0.0%	0.0%	0.0%	30.5%
tr|F1SLF0|F1SLF0	10	2	3	0	0	0	1	3	21	2	20	0	0	0	8	8	43.8%	9.4%	19.5%	0.0%	0.0%	0.0%	7.1%	18.7%
tr|F1SFE6|F1SFE6	11	4	4	0	0	0	1	1	16	7	37	0	0	0	5	6	33.7%	12.4%	12.9%	0.0%	0.0%	0.0%	7.4%	7.4%
P43367	8	3	1	0	0	0	0	4	20	3	7	0	0	0	0	5	31.2%	16.4%	3.1%	0.0%	0.0%	0.0%	0.0%	25.9%
tr|I3LKW2|I3LKW2	8	1	2	0	0	0	0	2	22	1	2	0	0	0	0	3	21.0%	3.3%	6.6%	0.0%	0.0%	0.0%	0.0%	4.4%
tr|I3LM03|I3LM03	8	3	4	0	0	0	2	4	17	6	165	0	0	0	7	13	29.5%	15.3%	18.1%	0.0%	0.0%	0.0%	11.0%	14.6%
tr|F1RGG2|F1RGG2	13	3	1	0	0	0	0	1	18	5	1	0	0	0	0	2	18.5%	2.6%	1.3%	0.0%	0.0%	0.0%	0.0%	1.3%
tr|I3L677|I3L677	9	4	1	0	0	0	0	1	14	9	1	0	0	0	0	2	25.8%	7.1%	2.1%	0.0%	0.0%	0.0%	0.0%	3.9%
F1RKQ4	11	2	4	0	0	0	1	1	19	4	36	0	0	0	3	1	31.3%	3.6%	11.4%	0.0%	0.0%	0.0%	2.9%	2.9%
tr|F1RSB0|F1RSB0	5	2	2	0	0	0	0	2	8	15	12	0	0	0	0	2	19.2%	6.4%	11.4%	0.0%	0.0%	0.0%	0.0%	12.8%
tr|F1SF95|F1SF95	1	3	0	0	0	0	0	1	2	21	0	0	0	0	0	1	15.3%	14.7%	0.0%	0.0%	0.0%	0.0%	0.0%	15.3%
tr|F1SFH5|F1SFH5	6	1	0	0	0	0	0	0	13	10	0	0	0	0	0	0	23.7%	2.6%	0.0%	0.0%	0.0%	0.0%	0.0%	0.0%
tr|I3LDT2|I3LDT2	1	2	0	0	0	0	0	0	2	21	0	0	0	0	0	0	0.4%	0.5%	0.0%	0.0%	0.0%	0.0%	0.0%	0.0%
tr|F1RVX6|F1RVX6	3	5	0	0	0	0	0	0	3	20	0	0	0	0	0	0	8.7%	10.5%	0.0%	0.0%	0.0%	0.0%	0.0%	0.0%
tr|F1SI65|F1SI65	1	2	0	0	0	0	0	0	2	21	0	0	0	0	0	0	0.4%	0.5%	0.0%	0.0%	0.0%	0.0%	0.0%	0.0%
tr|I3LEJ9|I3LEJ9	1	1	1	0	0	0	0	0	7	16	6	0	0	0	0	0	19.4%	19.4%	8.9%	0.0%	0.0%	0.0%	0.0%	0.0%
tr|I3LU95|I3LU95	9	7	1	0	0	0	0	0	13	10	8	0	0	0	0	0	12.2%	6.0%	1.5%	0.0%	0.0%	0.0%	0.0%	0.0%
tr|F1RLH7|F1RLH7	5	1	8	0	0	0	0	0	14	9	27	0	0	0	0	0	55.8%	14.0%	67.4%	0.0%	0.0%	0.0%	0.0%	0.0%
tr|I3LP02|I3LP02	11	2	5	0	0	0	0	0	19	4	32	0	0	0	0	0	39.7%	6.8%	20.1%	0.0%	0.0%	0.0%	0.0%	0.0%
tr|F1SRV9|F1SRV9	5	1	3	0	0	0	0	5	12	10	14	0	0	0	0	22	11.1%	1.8%	7.7%	0.0%	0.0%	0.0%	0.0%	13.0%
P80276	10	1	1	0	0	0	1	6	20	2	14	0	0	0	4	13	51.3%	4.4%	2.8%	0.0%	0.0%	0.0%	11.1%	43.4%
Q5S3G4	6	2	3	0	0	0	0	4	18	4	20	0	0	0	0	16	30.2%	24.0%	28.7%	0.0%	0.0%	0.0%	0.0%	30.2%
tr|I3L650|I3L650	8	5	13	0	0	0	3	2	8	14	57	0	0	0	11	13	13.1%	8.4%	21.1%	0.0%	0.0%	0.0%	8.9%	5.6%
tr|F1SQR0|F1SQR0	7	1	2	0	0	0	0	2	21	1	8	0	0	0	0	5	44.2%	2.1%	6.1%	0.0%	0.0%	0.0%	0.0%	9.2%
tr|I3LV46|I3LV46	7	1	2	0	0	0	0	2	21	1	8	0	0	0	0	5	40.1%	1.9%	5.5%	0.0%	0.0%	0.0%	0.0%	8.3%
tr|F1RQX8|F1RQX8	8	2	0	0	0	0	0	1	17	5	0	0	0	0	0	2	9.2%	2.1%	0.0%	0.0%	0.0%	0.0%	0.0%	0.8%
A5D9M6	8	2	0	0	0	0	0	1	17	5	0	0	0	0	0	2	8.8%	2.0%	0.0%	0.0%	0.0%	0.0%	0.0%	0.8%
tr|F1SBS1|F1SBS1	8	5	5	0	0	0	0	2	13	9	12	0	0	0	0	2	9.6%	7.1%	6.7%	0.0%	0.0%	0.0%	0.0%	1.9%
tr|F1RZR6|F1RZR6	3	2	1	0	0	0	1	1	11	11	113	0	0	0	3	4	33.3%	10.2%	7.9%	0.0%	0.0%	0.0%	10.2%	10.2%
tr|I3LJP0|I3LJP0	3	2	0	0	0	0	0	0	6	16	0	0	0	0	0	0	22.6%	9.7%	0.0%	0.0%	0.0%	0.0%	0.0%	0.0%
tr|K7GSJ1|K7GSJ1	2	1	0	0	0	0	0	0	21	1	0	0	0	0	0	0	68.0%	68.0%	0.0%	0.0%	0.0%	0.0%	0.0%	0.0%
tr|F1SN95|F1SN95	11	3	3	0	0	0	0	0	17	5	11	0	0	0	0	0	11.5%	4.2%	3.2%	0.0%	0.0%	0.0%	0.0%	0.0%
tr|F1S9K1|F1S9K1	5	2	5	0	0	0	0	0	5	17	14	0	0	0	0	0	12.6%	3.6%	9.7%	0.0%	0.0%	0.0%	0.0%	0.0%
tr|F1ST98|F1ST98	6	2	3	0	0	0	3	2	17	4	15	0	0	0	80	2	39.3%	15.3%	15.3%	0.0%	0.0%	0.0%	31.1%	15.3%
tr|F1RKM0|F1RKM0	11	1	9	0	0	0	2	7	20	1	24	0	0	0	16	27	23.7%	1.9%	16.0%	0.0%	0.0%	0.0%	6.8%	17.1%
tr|I3LBF7|I3LBF7	6	2	3	0	0	0	3	4	18	3	33	0	0	0	22	15	43.8%	11.8%	16.9%	0.0%	0.0%	0.0%	22.5%	38.2%
tr|F1SQW0|F1SQW0	8	2	4	0	0	0	1	2	17	4	30	0	0	0	13	7	26.3%	6.2%	14.2%	0.0%	0.0%	0.0%	2.5%	9.6%
tr|F1SNH3|F1SNH3	7	5	13	0	0	0	3	2	7	14	57	0	0	0	11	13	15.6%	11.9%	29.9%	0.0%	0.0%	0.0%	12.7%	7.9%
tr|F1S880|F1S880	9	2	3	0	0	0	0	1	17	4	17	0	0	0	0	10	32.7%	12.4%	15.1%	0.0%	0.0%	0.0%	0.0%	7.3%
tr|I3L656|I3L656	7	1	0	0	0	0	0	2	20	1	0	0	0	0	0	5	41.1%	7.3%	0.0%	0.0%	0.0%	0.0%	0.0%	21.0%
tr|K7GRD3|K7GRD3	6	1	2	0	0	0	0	2	20	1	8	0	0	0	0	5	37.7%	2.2%	6.2%	0.0%	0.0%	0.0%	0.0%	9.4%
tr|I3LN31|I3LN31	12	3	6	0	0	0	1	4	18	3	33	0	0	0	2	7	49.3%	10.9%	18.2%	0.0%	0.0%	0.0%	7.0%	25.6%
tr|I3L7S4|I3L7S4	7	5	21	0	0	0	3	2	7	14	132	0	0	0	11	13	16.4%	12.5%	38.0%	0.0%	0.0%	0.0%	13.3%	8.3%
tr|I3LNF5|I3LNF5	12	3	6	0	0	0	1	3	18	3	33	0	0	0	2	4	48.5%	10.8%	17.9%	0.0%	0.0%	0.0%	6.9%	14.5%
tr|F1SNR0|F1SNR0	5	1	0	0	0	0	0	1	20	1	0	0	0	0	0	2	34.7%	7.4%	0.0%	0.0%	0.0%	0.0%	0.0%	10.8%
tr|F1S4G7|F1S4G7	3	3	13	0	0	0	0	2	9	12	41	0	0	0	0	4	4.8%	3.4%	20.1%	0.0%	0.0%	0.0%	0.0%	2.6%
tr|I3LTN6|I3LTN6	8	1	7	0	0	0	1	1	18	3	54	0	0	0	3	1	26.1%	2.6%	15.0%	0.0%	0.0%	0.0%	2.6%	5.5%
tr|A9YTX9|A9YTX9	3	2	0	0	0	0	0	0	17	4	0	0	0	0	0	0	22.0%	7.9%	0.0%	0.0%	0.0%	0.0%	0.0%	0.0%
tr|F1SSH4|F1SSH4	2	1	0	0	0	0	0	0	12	9	0	0	0	0	0	0	4.7%	2.2%	0.0%	0.0%	0.0%	0.0%	0.0%	0.0%
tr|F1SI64|F1SI64	0	2	0	0	0	0	0	0	0	21	0	0	0	0	0	0	0.0%	2.0%	0.0%	0.0%	0.0%	0.0%	0.0%	0.0%
tr|F1SRP0|F1SRP0	7	2	1	0	0	0	0	0	12	9	1	0	0	0	0	0	11.8%	1.7%	1.3%	0.0%	0.0%	0.0%	0.0%	0.0%
tr|F1SU78|F1SU78	6	2	3	0	0	0	0	0	16	5	10	0	0	0	0	0	21.1%	12.1%	9.7%	0.0%	0.0%	0.0%	0.0%	0.0%
tr|I3L594|I3L594	7	1	2	0	0	0	2	3	14	6	12	0	0	0	13	6	41.7%	12.7%	10.5%	0.0%	0.0%	0.0%	12.7%	28.1%
tr|I3LRD5|I3LRD5	7	1	2	0	0	0	2	3	14	6	12	0	0	0	13	6	38.3%	11.7%	9.7%	0.0%	0.0%	0.0%	11.7%	25.8%
tr|F1RVD1|F1RVD1	5	1	0	0	0	0	0	3	19	1	0	0	0	0	0	7	27.3%	7.4%	0.0%	0.0%	0.0%	0.0%	0.0%	23.6%
tr|F1RX66|F1RX66	7	6	7	0	0	0	1	4	10	10	23	0	0	0	1	11	28.8%	14.8%	21.5%	0.0%	0.0%	0.0%	4.3%	17.0%
Q29236	4	0	3	0	0	0	1	3	20	0	36	0	0	0	2	13	32.7%	0.0%	22.1%	0.0%	0.0%	0.0%	12.5%	32.7%
tr|I3LUB8|I3LUB8	8	2	0	0	0	0	0	1	15	5	0	0	0	0	0	5	8.2%	2.0%	0.0%	0.0%	0.0%	0.0%	0.0%	1.4%
tr|F1RSZ5|F1RSZ5	6	3	1	0	0	0	1	1	9	11	3	0	0	0	4	1	26.9%	17.8%	4.5%	0.0%	0.0%	0.0%	6.2%	5.0%
tr|F1SQ46|F1SQ46	9	0	2	0	0	0	1	3	20	0	25	0	0	0	1	6	33.0%	0.0%	4.8%	0.0%	0.0%	0.0%	5.4%	25.0%
tr|F1SL58|F1SL58	11	3	6	0	0	0	1	3	17	3	33	0	0	0	2	4	52.6%	12.9%	21.5%	0.0%	0.0%	0.0%	8.2%	17.4%

	Peptide Count								Spectral Count								Sequence Coverage							
	Whole Cell	Whole Cell	Whole Cell	Cilia Fraction	Cilia Fraction	Cilia Fraction	Cilia Fraction	Cilia Fraction	Whole Cell	Whole Cell	Whole Cell	Cilia Fraction	Cilia Fraction	Cilia Fraction	Cilia Fraction	Cilia Fraction	Whole Cell	Whole Cell	Whole Cell	Cilia Fraction	Cilia Fraction	Cilia Fraction	Cilia Fraction	Cilia Fraction
Sample#	1	2	6	4	3	5	7	8	1	2	6	4	3	5	7	8	1	2	6	4	3	5	7	8
**Accession**																								
tr|F1SKF9|F1SKF9	11	3	6	0	0	0	1	3	17	3	33	0	0	0	2	4	54.1%	13.3%	22.1%	0.0%	0.0%	0.0%	8.5%	17.8%
tr|F1SD96|F1SD96	2	3	0	0	0	0	0	1	5	15	0	0	0	0	0	2	12.6%	7.1%	0.0%	0.0%	0.0%	0.0%	0.0%	7.9%
tr|I3LHB2|I3LHB2	9	6	2	0	0	0	1	0	10	10	7	0	0	0	2	0	20.3%	17.3%	3.0%	0.0%	0.0%	0.0%	4.3%	0.0%
tr|F1RHH4|F1RHH4	9	6	2	0	0	0	1	0	10	10	7	0	0	0	2	0	20.6%	17.5%	3.1%	0.0%	0.0%	0.0%	4.4%	0.0%
tr|D0G777|D0G777	5	2	5	0	0	0	1	1	12	8	36	0	0	0	3	1	15.7%	8.6%	17.0%	0.0%	0.0%	0.0%	3.3%	4.1%
tr|F1RNZ1|F1RNZ1	3	4	5	0	0	0	0	1	9	11	22	0	0	0	0	2	26.6%	17.2%	14.2%	0.0%	0.0%	0.0%	0.0%	8.8%
tr|Q2XVP5|Q2XVP5	9	0	6	0	0	0	1	0	20	0	22	0	0	0	1	0	43.7%	0.0%	17.5%	0.0%	0.0%	0.0%	4.5%	0.0%
tr|F1SJ65|F1SJ65	1	1	0	0	0	0	0	0	1	19	0	0	0	0	0	0	10.1%	7.5%	0.0%	0.0%	0.0%	0.0%	0.0%	0.0%
tr|I3LV00|I3LV00	3	4	0	0	0	0	0	0	3	17	0	0	0	0	0	0	1.2%	1.7%	0.0%	0.0%	0.0%	0.0%	0.0%	0.0%
Q764M6	2	2	0	0	0	0	0	0	16	4	0	0	0	0	0	0	11.5%	7.9%	0.0%	0.0%	0.0%	0.0%	0.0%	0.0%
tr|F1RJ58|F1RJ58	3	4	0	0	0	0	0	0	3	17	0	0	0	0	0	0	1.2%	1.7%	0.0%	0.0%	0.0%	0.0%	0.0%	0.0%
tr|F1RG40|F1RG40	6	4	1	0	0	0	0	0	7	13	1	0	0	0	0	0	8.7%	3.4%	1.9%	0.0%	0.0%	0.0%	0.0%	0.0%
tr|I3LA48|I3LA48	1	3	1	0	0	0	0	0	1	19	5	0	0	0	0	0	1.0%	2.0%	1.5%	0.0%	0.0%	0.0%	0.0%	0.0%
tr|I3LMJ9|I3LMJ9	1	3	1	0	0	0	0	0	1	19	5	0	0	0	0	0	1.0%	2.0%	1.5%	0.0%	0.0%	0.0%	0.0%	0.0%
tr|F1SEP0|F1SEP0	5	2	2	0	0	0	0	0	9	11	5	0	0	0	0	0	8.4%	2.9%	2.9%	0.0%	0.0%	0.0%	0.0%	0.0%
tr|F1S0L8|F1S0L8	8	1	7	0	0	0	0	0	12	8	31	0	0	0	0	0	19.8%	1.9%	13.4%	0.0%	0.0%	0.0%	0.0%	0.0%
tr|F2Z5I5|F2Z5I5	10	0	3	0	0	0	2	10	19	0	9	0	0	0	12	56	25.1%	0.0%	7.2%	0.0%	0.0%	0.0%	11.0%	29.4%
tr|I3LU12|I3LU12	6	2	1	0	0	0	2	5	13	6	5	0	0	0	3	22	33.3%	10.8%	20.6%	0.0%	0.0%	0.0%	32.4%	33.3%
P67776	10	1	3	0	0	0	1	5	18	1	14	0	0	0	4	29	54.4%	8.1%	14.9%	0.0%	0.0%	0.0%	8.1%	19.7%
P11493	10	1	3	0	0	0	1	5	18	1	14	0	0	0	4	29	57.3%	8.5%	15.7%	0.0%	0.0%	0.0%	8.5%	20.8%
tr|F1RX68|F1RX68	10	1	3	0	0	0	1	5	18	1	14	0	0	0	4	29	60.2%	9.0%	16.5%	0.0%	0.0%	0.0%	9.0%	21.9%
Q29068	3	1	2	0	0	0	2	2	18	1	28	0	0	0	17	10	36.6%	24.4%	36.6%	0.0%	0.0%	0.0%	36.6%	36.6%
tr|F1SBT6|F1SBT6	8	3	9	0	0	0	4	3	16	3	84	0	0	0	9	35	17.0%	4.4%	16.7%	0.0%	0.0%	0.0%	10.0%	8.2%
tr|I3LP80|I3LP80	4	1	0	0	0	0	0	3	18	1	0	0	0	0	0	7	22.1%	6.9%	0.0%	0.0%	0.0%	0.0%	0.0%	22.1%
tr|F1RUP7|F1RUP7	5	1	0	0	0	0	0	2	18	1	0	0	0	0	0	5	34.3%	7.4%	0.0%	0.0%	0.0%	0.0%	0.0%	21.3%
tr|K7GKI1|K7GKI1	5	3	7	0	0	0	1	0	11	8	73	0	0	0	24	0	27.9%	9.7%	28.5%	0.0%	0.0%	0.0%	4.0%	0.0%
tr|F1SNZ6|F1SNZ6	5	4	2	0	0	0	0	1	10	9	4	0	0	0	0	5	9.9%	17.6%	6.6%	0.0%	0.0%	0.0%	0.0%	3.3%
tr|F1RQP0|F1RQP0	7	2	7	0	0	0	1	2	15	4	48	0	0	0	1	5	42.0%	10.5%	42.6%	0.0%	0.0%	0.0%	9.9%	18.5%
tr|F1SJN5|F1SJN5	6	2	2	0	0	0	0	1	12	7	3	0	0	0	0	1	19.1%	4.5%	6.1%	0.0%	0.0%	0.0%	0.0%	6.1%
tr|F1RR49|F1RR49	3	3	0	0	0	0	0	0	3	16	0	0	0	0	0	0	3.5%	2.1%	0.0%	0.0%	0.0%	0.0%	0.0%	0.0%
tr|F1RS55|F1RS55	2	2	0	0	0	0	0	0	5	14	0	0	0	0	0	0	12.3%	12.3%	0.0%	0.0%	0.0%	0.0%	0.0%	0.0%
tr|F1RQQ8|F1RQQ8	1	2	0	0	0	0	0	0	1	18	0	0	0	0	0	0	1.0%	2.6%	0.0%	0.0%	0.0%	0.0%	0.0%	0.0%
tr|F1SS78|F1SS78	0	1	1	0	0	0	0	0	0	19	2	0	0	0	0	0	0.0%	8.6%	13.8%	0.0%	0.0%	0.0%	0.0%	0.0%
A7Y521	9	0	1	0	0	0	0	0	19	0	3	0	0	0	0	0	29.1%	0.0%	3.0%	0.0%	0.0%	0.0%	0.0%	0.0%
tr|F1SP81|F1SP81	4	2	2	0	0	0	0	0	8	11	3	0	0	0	0	0	9.1%	4.2%	3.0%	0.0%	0.0%	0.0%	0.0%	0.0%
tr|I3LVD6|I3LVD6	8	0	1	0	0	0	0	0	19	0	6	0	0	0	0	0	16.2%	0.0%	3.5%	0.0%	0.0%	0.0%	0.0%	0.0%
tr|F1SQ55|F1SQ55	8	0	1	0	0	0	0	0	19	0	6	0	0	0	0	0	16.3%	0.0%	3.5%	0.0%	0.0%	0.0%	0.0%	0.0%
tr|F1RU62|F1RU62	1	6	1	0	0	0	0	0	1	18	7	0	0	0	0	0	2.2%	6.1%	1.6%	0.0%	0.0%	0.0%	0.0%	0.0%
tr|F1RUV6|F1RUV6	6	0	3	0	0	0	0	0	19	0	15	0	0	0	0	0	23.2%	0.0%	12.0%	0.0%	0.0%	0.0%	0.0%	0.0%
tr|K7GPJ4|K7GPJ4	8	3	4	0	0	0	0	0	12	7	19	0	0	0	0	0	24.0%	8.1%	9.9%	0.0%	0.0%	0.0%	0.0%	0.0%
tr|F1SFR6|F1SFR6	10	3	13	0	0	0	0	0	16	3	147	0	0	0	0	0	26.6%	10.4%	35.9%	0.0%	0.0%	0.0%	0.0%	0.0%
P53590	10	3	13	0	0	0	0	0	16	3	147	0	0	0	0	0	26.6%	10.4%	35.8%	0.0%	0.0%	0.0%	0.0%	0.0%
tr|F1SCF0|F1SCF0	2	3	0	0	0	0	0	2	7	11	0	0	0	0	0	24	3.1%	4.5%	0.0%	0.0%	0.0%	0.0%	0.0%	3.1%
tr|F1SM07|F1SM07	5	2	0	0	0	0	0	3	6	12	0	0	0	0	0	21	48.3%	12.4%	0.0%	0.0%	0.0%	0.0%	0.0%	28.7%
tr|F1S8S4|F1S8S4	8	1	2	0	0	0	1	4	17	1	4	0	0	0	4	20	10.8%	2.1%	3.6%	0.0%	0.0%	0.0%	3.1%	8.9%
tr|F1RI11|F1RI11	9	1	3	0	0	0	1	5	17	1	14	0	0	0	4	29	61.6%	9.8%	18.0%	0.0%	0.0%	0.0%	9.8%	23.9%
tr|F1S8T7|F1S8T7	3	0	2	0	0	0	2	1	18	0	16	0	0	0	15	6	23.9%	0.0%	19.3%	0.0%	0.0%	0.0%	19.3%	11.9%
Q95250	4	3	3	0	0	0	0	3	12	6	9	0	0	0	0	13	28.4%	7.7%	30.4%	0.0%	0.0%	0.0%	0.0%	28.4%
tr|F1S6K5|F1S6K5	4	3	1	0	0	0	1	1	14	4	8	0	0	0	7	4	26.7%	10.0%	7.2%	0.0%	0.0%	0.0%	8.9%	11.1%
tr|I3LJS0|I3LJS0	5	3	1	0	0	0	0	1	11	7	3	0	0	0	0	6	23.8%	7.5%	4.2%	0.0%	0.0%	0.0%	0.0%	6.9%
tr|F1RKW7|F1RKW7	0	4	2	0	0	0	2	2	0	18	7	0	0	0	3	4	0.0%	5.0%	2.8%	0.0%	0.0%	0.0%	7.3%	7.3%
tr|F1SFH7|F1SFH7	10	1	5	0	0	0	1	3	16	2	31	0	0	0	2	9	29.0%	3.1%	27.0%	0.0%	0.0%	0.0%	6.9%	14.3%
P83686	7	2	2	0	0	0	0	3	14	4	10	0	0	0	0	6	48.2%	11.0%	6.2%	0.0%	0.0%	0.0%	0.0%	14.3%
tr|F1SJQ5|F1SJQ5	7	2	2	0	0	0	0	3	14	4	10	0	0	0	0	6	42.7%	9.8%	5.5%	0.0%	0.0%	0.0%	0.0%	12.7%
tr|F1SS16|F1SS16	6	2	2	0	0	0	1	1	15	3	16	0	0	0	3	4	23.4%	8.2%	5.8%	0.0%	0.0%	0.0%	5.4%	2.8%
tr|F1SKD5|F1SKD5	8	3	1	0	0	0	0	1	11	7	8	0	0	0	0	5	14.3%	3.2%	2.1%	0.0%	0.0%	0.0%	0.0%	3.1%
tr|F1SUG4|F1SUG4	5	1	4	0	0	0	1	1	10	8	10	0	0	0	1	3	39.8%	10.2%	46.6%	0.0%	0.0%	0.0%	10.2%	32.2%
tr|F1S193|F1S193	8	2	6	0	0	0	0	1	15	3	17	0	0	0	0	4	28.2%	9.3%	27.2%	0.0%	0.0%	0.0%	0.0%	7.7%
tr|F1S194|F1S194	8	2	6	0	0	0	0	1	15	3	17	0	0	0	0	4	26.5%	8.7%	25.6%	0.0%	0.0%	0.0%	0.0%	7.2%
tr|I3LQF8|I3LQF8	9	0	2	0	0	0	1	2	18	0	25	0	0	0	1	3	27.4%	0.0%	3.9%	0.0%	0.0%	0.0%	4.5%	14.2%
tr|I3LJ73|I3LJ73	6	4	5	0	0	0	1	1	11	7	18	0	0	0	2	1	23.9%	14.4%	12.8%	0.0%	0.0%	0.0%	4.5%	5.9%
tr|F1SLR1|F1SLR1	6	0	2	0	0	0	0	1	18	0	18	0	0	0	0	1	40.7%	0.0%	10.5%	0.0%	0.0%	0.0%	0.0%	9.9%
tr|I3L7B7|I3L7B7	2	3	0	0	0	0	0	0	5	13	0	0	0	0	0	0	4.9%	6.6%	0.0%	0.0%	0.0%	0.0%	0.0%	0.0%
tr|F1RIY8|F1RIY8	0	2	0	0	0	0	0	0	0	18	0	0	0	0	0	0	0.0%	3.0%	0.0%	0.0%	0.0%	0.0%	0.0%	0.0%
tr|F1RIK3|F1RIK3	4	3	0	0	0	0	0	0	8	10	0	0	0	0	0	0	11.8%	10.1%	0.0%	0.0%	0.0%	0.0%	0.0%	0.0%
tr|F1RY13|F1RY13	2	3	0	0	0	0	0	0	5	13	0	0	0	0	0	0	5.1%	6.9%	0.0%	0.0%	0.0%	0.0%	0.0%	0.0%
tr|I3LQ36|I3LQ36	2	1	0	0	0	0	0	0	17	1	0	0	0	0	0	0	11.2%	7.5%	0.0%	0.0%	0.0%	0.0%	0.0%	0.0%
tr|F1SE83|F1SE83	1	1	0	0	0	0	0	0	1	17	0	0	0	0	0	0	12.4%	6.7%	0.0%	0.0%	0.0%	0.0%	0.0%	0.0%
tr|I3L840|I3L840	2	3	0	0	0	0	0	0	5	13	0	0	0	0	0	0	5.1%	6.9%	0.0%	0.0%	0.0%	0.0%	0.0%	0.0%
tr|F1SAR7|F1SAR7	0	1	2	0	0	0	0	0	0	18	4	0	0	0	0	0	0.0%	1.2%	7.0%	0.0%	0.0%	0.0%	0.0%	0.0%
P61291	5	1	2	0	0	0	0	0	10	8	6	0	0	0	0	0	25.2%	3.5%	9.8%	0.0%	0.0%	0.0%	0.0%	0.0%
tr|F1SUZ2|F1SUZ2	6	3	4	0	0	0	0	0	9	9	13	0	0	0	0	0	4.9%	2.6%	2.8%	0.0%	0.0%	0.0%	0.0%	0.0%
tr|K7GP45|K7GP45	5	1	2	0	0	0	0	0	11	7	14	0	0	0	0	0	31.4%	8.1%	15.1%	0.0%	0.0%	0.0%	0.0%	0.0%
tr|F1SU29|F1SU29	13	2	4	0	0	0	0	0	16	2	22	0	0	0	0	0	5.9%	1.4%	2.2%	0.0%	0.0%	0.0%	0.0%	0.0%
tr|F1SL74|F1SL74	8	2	5	0	0	0	0	0	15	3	29	0	0	0	0	0	22.6%	6.2%	11.7%	0.0%	0.0%	0.0%	0.0%	0.0%
P50447	2	2	0	0	0	0	0	2	7	10	0	0	0	0	0	24	3.1%	3.1%	0.0%	0.0%	0.0%	0.0%	0.0%	3.1%
tr|F1S5N8|F1S5N8	2	2	3	0	0	0	0	3	9	8	14	0	0	0	0	16	22.8%	15.5%	14.0%	0.0%	0.0%	0.0%	0.0%	29.5%
tr|Q69GF7|Q69GF7	4	0	1	0	0	0	0	1	17	0	13	0	0	0	0	15	20.1%	0.0%	4.4%	0.0%	0.0%	0.0%	0.0%	8.7%
P50667	4	0	1	0	0	0	0	1	17	0	13	0	0	0	0	15	20.2%	0.0%	4.4%	0.0%	0.0%	0.0%	0.0%	8.8%
tr|F1S9Y6|F1S9Y6	2	1	2	0	0	0	1	0	5	12	16	0	0	0	16	0	28.8%	28.8%	28.8%	0.0%	0.0%	0.0%	28.8%	0.0%
tr|F1SEX0|F1SEX0	6	0	4	0	0	0	1	4	17	0	310	0	0	0	2	90	50.5%	0.0%	35.5%	0.0%	0.0%	0.0%	11.8%	30.6%

	Peptide Count								Spectral Count								Sequence Coverage							
	Whole Cell	Whole Cell	Whole Cell	Cilia Fraction	Cilia Fraction	Cilia Fraction	Cilia Fraction	Cilia Fraction	Whole Cell	Whole Cell	Whole Cell	Cilia Fraction	Cilia Fraction	Cilia Fraction	Cilia Fraction	Cilia Fraction	Whole Cell	Whole Cell	Whole Cell	Cilia Fraction	Cilia Fraction	Cilia Fraction	Cilia Fraction	Cilia Fraction
Sample#	1	2	6	4	3	5	7	8	1	2	6	4	3	5	7	8	1	2	6	4	3	5	7	8
**Accession**																								
tr|F1SG38|F1SG38	7	3	4	0	0	0	1	2	13	4	15	0	0	0	2	6	25.9%	6.0%	11.0%	0.0%	0.0%	0.0%	4.8%	12.0%
tr|F2Z5M9|F2Z5M9	6	0	1	0	0	0	0	1	17	0	4	0	0	0	0	5	23.2%	0.0%	4.4%	0.0%	0.0%	0.0%	0.0%	3.8%
tr|I3LLE6|I3LLE6	5	2	2	0	0	0	1	1	14	3	16	0	0	0	3	4	24.6%	10.6%	7.6%	0.0%	0.0%	0.0%	7.0%	3.6%
tr|F1SU52|F1SU52	8	2	5	0	0	0	2	3	13	4	60	0	0	0	8	6	35.8%	10.2%	22.4%	0.0%	0.0%	0.0%	11.5%	16.3%
Q95274	7	3	11	0	0	0	2	4	14	3	181	0	0	0	8	26	61.4%	45.5%	79.5%	0.0%	0.0%	0.0%	43.2%	45.5%
tr|F1RYY8|F1RYY8	8	2	2	0	0	0	0	1	15	2	13	0	0	0	0	2	31.4%	10.9%	11.2%	0.0%	0.0%	0.0%	0.0%	8.1%
tr|F1SUD6|F1SUD6	7	0	5	0	0	0	0	1	17	0	8	0	0	0	0	1	14.0%	0.0%	8.4%	0.0%	0.0%	0.0%	0.0%	2.7%
tr|F1SDC7|F1SDC7	8	3	4	0	0	0	0	1	12	5	23	0	0	0	0	1	21.0%	11.5%	9.2%	0.0%	0.0%	0.0%	0.0%	3.1%
tr|I3LGE3|I3LGE3	2	1	0	0	0	0	0	0	3	14	0	0	0	0	0	0	34.3%	11.1%	0.0%	0.0%	0.0%	0.0%	0.0%	0.0%
tr|I3LAL7|I3LAL7	3	3	0	0	0	0	0	0	6	11	0	0	0	0	0	0	10.3%	8.9%	0.0%	0.0%	0.0%	0.0%	0.0%	0.0%
tr|I3LF70|I3LF70	10	1	0	0	0	0	0	0	15	2	0	0	0	0	0	0	5.1%	0.5%	0.0%	0.0%	0.0%	0.0%	0.0%	0.0%
tr|F1RN43|F1RN43	2	6	0	0	0	0	0	0	2	15	0	0	0	0	0	0	3.8%	7.1%	0.0%	0.0%	0.0%	0.0%	0.0%	0.0%
tr|F1SP73|F1SP73	6	2	1	0	0	0	0	0	8	9	1	0	0	0	0	0	10.4%	1.7%	1.3%	0.0%	0.0%	0.0%	0.0%	0.0%
P68211	3	3	1	0	0	0	0	0	4	13	1	0	0	0	0	0	27.3%	14.9%	9.1%	0.0%	0.0%	0.0%	0.0%	0.0%
tr|F1RSH0|F1RSH0	6	1	1	0	0	0	0	0	10	7	1	0	0	0	0	0	25.0%	4.4%	4.4%	0.0%	0.0%	0.0%	0.0%	0.0%
tr|F1SDG5|F1SDG5	5	1	1	0	0	0	0	0	5	12	2	0	0	0	0	0	8.9%	1.9%	4.7%	0.0%	0.0%	0.0%	0.0%	0.0%
tr|F1SSZ2|F1SSZ2	2	3	2	0	0	0	0	0	6	11	6	0	0	0	0	0	2.1%	2.0%	1.1%	0.0%	0.0%	0.0%	0.0%	0.0%
tr|F1RIM3|F1RIM3	2	3	2	0	0	0	0	0	6	11	6	0	0	0	0	0	2.0%	1.9%	1.1%	0.0%	0.0%	0.0%	0.0%	0.0%
tr|F1SG42|F1SG42	5	3	1	0	0	0	0	0	7	10	6	0	0	0	0	0	12.6%	4.1%	3.6%	0.0%	0.0%	0.0%	0.0%	0.0%
tr|F1SG41|F1SG41	5	3	1	0	0	0	0	0	7	10	6	0	0	0	0	0	12.6%	4.1%	3.6%	0.0%	0.0%	0.0%	0.0%	0.0%
tr|F1SS77|F1SS77	3	3	2	0	0	0	0	0	4	13	6	0	0	0	0	0	29.2%	15.9%	20.4%	0.0%	0.0%	0.0%	0.0%	0.0%
tr|I3LJ05|I3LJ05	7	0	3	0	0	0	0	0	17	0	7	0	0	0	0	0	32.0%	0.0%	11.6%	0.0%	0.0%	0.0%	0.0%	0.0%
Q6QAT1	6	1	2	0	0	0	0	0	14	3	7	0	0	0	0	0	40.6%	17.4%	23.2%	0.0%	0.0%	0.0%	0.0%	0.0%
tr|F1SEX5|F1SEX5	5	3	3	0	0	0	0	0	6	11	10	0	0	0	0	0	9.0%	3.8%	5.5%	0.0%	0.0%	0.0%	0.0%	0.0%
P79274	12	0	5	0	0	0	0	0	17	0	19	0	0	0	0	0	26.3%	0.0%	15.6%	0.0%	0.0%	0.0%	0.0%	0.0%
tr|I3L7S7|I3L7S7	5	1	4	0	0	0	0	0	16	1	24	0	0	0	0	0	34.2%	4.4%	13.1%	0.0%	0.0%	0.0%	0.0%	0.0%
tr|F1SMS8|F1SMS8	6	0	2	0	0	0	1	6	16	0	11	0	0	0	63	83	24.6%	0.0%	8.5%	0.0%	0.0%	0.0%	6.2%	18.4%
tr|F1SMQ1|F1SMQ1	7	2	3	0	0	0	3	3	12	4	21	0	0	0	67	48	20.2%	19.3%	16.0%	0.0%	0.0%	0.0%	19.3%	19.3%
tr|I3LQ28|I3LQ28	4	1	5	0	0	0	1	1	14	2	17	0	0	0	46	1	26.8%	6.3%	28.4%	0.0%	0.0%	0.0%	6.3%	6.3%
tr|F2Z5T3|F2Z5T3	4	1	5	0	0	0	1	1	14	2	17	0	0	0	46	1	26.7%	6.3%	28.3%	0.0%	0.0%	0.0%	6.3%	6.3%
tr|I3LCR4|I3LCR4	2	2	3	0	0	0	0	2	4	12	11	0	0	0	0	27	42.9%	27.3%	27.3%	0.0%	0.0%	0.0%	0.0%	27.3%
Q29290	2	2	4	0	0	0	0	2	4	12	14	0	0	0	0	27	33.7%	21.4%	40.8%	0.0%	0.0%	0.0%	0.0%	21.4%
tr|I3LGJ5|I3LGJ5	5	2	8	0	0	0	1	8	9	7	25	0	0	0	1	18	7.6%	2.1%	7.7%	0.0%	0.0%	0.0%	0.9%	12.0%
Q8MIK9	6	0	1	0	0	0	1	3	16	0	4	0	0	0	3	6	42.2%	0.0%	16.3%	0.0%	0.0%	0.0%	8.8%	32.0%
tr|I3LB68|I3LB68	6	3	1	0	0	0	3	1	11	5	24	0	0	0	12	2	12.3%	12.8%	3.9%	0.0%	0.0%	0.0%	10.3%	3.9%
tr|I3LVJ7|I3LVJ7	7	4	4	0	0	0	1	2	11	5	30	0	0	0	2	10	33.3%	23.1%	16.7%	0.0%	0.0%	0.0%	6.9%	14.8%
tr|F1SU97|F1SU97	7	0	2	0	0	0	0	2	16	0	7	0	0	0	0	6	26.3%	0.0%	4.0%	0.0%	0.0%	0.0%	0.0%	8.4%
tr|I3L7T8|I3L7T8	4	1	2	0	0	0	1	2	12	4	17	0	0	0	2	5	40.2%	13.4%	19.6%	0.0%	0.0%	0.0%	13.4%	17.5%
tr|F1RPD2|F1RPD2	10	1	6	0	0	0	0	4	15	1	30	0	0	0	0	9	36.6%	3.3%	17.9%	0.0%	0.0%	0.0%	0.0%	15.3%
tr|F1SU53|F1SU53	8	0	4	0	0	0	1	4	16	0	26	0	0	0	1	6	11.4%	0.0%	4.7%	0.0%	0.0%	0.0%	1.7%	6.4%
tr|F1STM9|F1STM9	6	0	1	0	0	0	0	1	16	0	3	0	0	0	0	3	35.9%	0.0%	7.4%	0.0%	0.0%	0.0%	0.0%	5.2%
tr|K7GSX0|K7GSX0	5	4	2	0	0	0	1	1	11	5	12	0	0	0	1	3	5.2%	3.0%	2.5%	0.0%	0.0%	0.0%	0.8%	1.3%
tr|F1RZU3|F1RZU3	5	4	2	0	0	0	1	1	11	5	12	0	0	0	1	3	5.1%	2.9%	2.5%	0.0%	0.0%	0.0%	0.8%	1.3%
P14287	11	0	6	0	0	0	2	0	16	0	40	0	0	0	7	0	43.6%	0.0%	25.7%	0.0%	0.0%	0.0%	5.3%	0.0%
tr|F1STK8|F1STK8	7	1	0	0	0	0	0	2	15	1	0	0	0	0	0	2	32.2%	4.5%	0.0%	0.0%	0.0%	0.0%	0.0%	17.4%
tr|K7GNY9|K7GNY9	6	0	0	0	0	0	1	0	16	0	0	0	0	0	2	0	44.7%	0.0%	0.0%	0.0%	0.0%	0.0%	12.7%	0.0%
tr|F1RHJ2|F1RHJ2	6	2	17	0	0	0	1	3	13	3	102	0	0	0	2	11	28.8%	11.1%	63.0%	0.0%	0.0%	0.0%	4.5%	28.8%
P00571	5	1	4	0	0	0	1	2	12	4	39	0	0	0	1	5	35.6%	6.2%	24.7%	0.0%	0.0%	0.0%	7.2%	17.5%
tr|F1SMG3|F1SMG3	4	1	2	0	0	0	0	1	6	10	11	0	0	0	0	2	7.5%	1.0%	3.6%	0.0%	0.0%	0.0%	0.0%	3.5%
tr|F1RMA3|F1RMA3	10	2	5	0	0	0	0	1	12	4	31	0	0	0	0	3	16.7%	2.2%	10.5%	0.0%	0.0%	0.0%	0.0%	3.4%
tr|F1RNZ2|F1RNZ2	4	2	1	0	0	0	0	1	11	5	4	0	0	0	0	1	33.9%	25.2%	10.4%	0.0%	0.0%	0.0%	0.0%	19.1%
tr|F1SPE5|F1SPE5	0	2	0	0	0	0	0	0	0	16	0	0	0	0	0	0	0.0%	14.5%	0.0%	0.0%	0.0%	0.0%	0.0%	0.0%
tr|I3LNN3|I3LNN3	9	1	0	0	0	0	0	0	14	2	0	0	0	0	0	0	4.6%	0.5%	0.0%	0.0%	0.0%	0.0%	0.0%	0.0%
tr|F1SU36|F1SU36	7	2	0	0	0	0	0	0	11	5	0	0	0	0	0	0	14.5%	4.3%	0.0%	0.0%	0.0%	0.0%	0.0%	0.0%
tr|F1SV27|F1SV27	1	1	0	0	0	0	0	0	1	15	0	0	0	0	0	0	3.6%	3.6%	0.0%	0.0%	0.0%	0.0%	0.0%	0.0%
tr|D7RA28|D7RA28	0	2	0	0	0	0	0	0	0	16	0	0	0	0	0	0	0.0%	14.5%	0.0%	0.0%	0.0%	0.0%	0.0%	0.0%
tr|F1SI34|F1SI34	8	1	1	0	0	0	0	0	14	2	2	0	0	0	0	0	21.1%	1.8%	2.5%	0.0%	0.0%	0.0%	0.0%	0.0%
tr|F1RX50|F1RX50	8	2	3	0	0	0	0	0	13	3	4	0	0	0	0	0	11.3%	5.9%	8.8%	0.0%	0.0%	0.0%	0.0%	0.0%
tr|F1SPF3|F1SPF3	8	3	3	0	0	0	0	0	12	4	5	0	0	0	0	0	11.4%	3.5%	5.2%	0.0%	0.0%	0.0%	0.0%	0.0%
tr|I3LVP1|I3LVP1	4	3	4	0	0	0	0	0	9	7	8	0	0	0	0	0	33.3%	39.0%	32.4%	0.0%	0.0%	0.0%	0.0%	0.0%
tr|F1S297|F1S297	9	1	1	0	0	0	0	0	15	1	9	0	0	0	0	0	25.5%	3.3%	7.9%	0.0%	0.0%	0.0%	0.0%	0.0%
tr|F1S325|F1S325	5	2	1	0	0	0	0	0	6	10	11	0	0	0	0	0	35.4%	6.5%	9.0%	0.0%	0.0%	0.0%	0.0%	0.0%
tr|F1RNS7|F1RNS7	7	2	6	0	0	0	2	4	12	3	51	0	0	0	57	33	45.1%	16.1%	22.4%	0.0%	0.0%	0.0%	12.2%	21.2%
tr|J9JIM3|J9JIM3	3	2	3	0	0	0	2	2	7	8	12	0	0	0	21	9	31.5%	32.6%	29.3%	0.0%	0.0%	0.0%	29.3%	37.0%
tr|F1SGN8|F1SGN8	7	3	2	0	0	0	2	3	11	4	10	0	0	0	7	8	25.0%	14.1%	8.3%	0.0%	0.0%	0.0%	15.8%	23.9%
tr|I3LUD1|I3LUD1	4	1	0	0	0	0	0	2	14	1	0	0	0	0	0	5	27.2%	4.9%	0.0%	0.0%	0.0%	0.0%	0.0%	14.8%
tr|F1S146|F1S146	11	1	2	0	0	0	0	3	14	1	2	0	0	0	0	4	4.4%	0.3%	0.7%	0.0%	0.0%	0.0%	0.0%	2.1%
tr|F1RFA5|F1RFA5	4	4	0	0	0	0	1	0	5	10	0	0	0	0	3	0	12.8%	18.9%	0.0%	0.0%	0.0%	0.0%	6.4%	0.0%
tr|F1S6E6|F1S6E6	2	2	0	0	0	0	0	1	5	10	0	0	0	0	0	3	7.5%	5.9%	0.0%	0.0%	0.0%	0.0%	0.0%	2.0%
tr|K7GLR2|K7GLR2	2	2	0	0	0	0	0	1	5	10	0	0	0	0	0	3	6.6%	5.2%	0.0%	0.0%	0.0%	0.0%	0.0%	1.7%
tr|A5D9J3|A5D9J3	6	2	2	0	0	0	0	2	13	2	7	0	0	0	0	4	16.2%	2.7%	4.7%	0.0%	0.0%	0.0%	0.0%	4.2%
tr|F1RK53|F1RK53	7	1	4	0	0	0	2	0	12	3	15	0	0	0	5	0	14.5%	1.0%	7.9%	0.0%	0.0%	0.0%	3.7%	0.0%
tr|I3L6T2|I3L6T2	5	0	4	0	0	0	1	2	15	0	36	0	0	0	2	5	28.5%	0.0%	28.5%	0.0%	0.0%	0.0%	6.9%	28.5%
tr|F1RU38|F1RU38	10	1	9	0	0	0	1	3	14	1	46	0	0	0	1	4	12.4%	1.1%	12.6%	0.0%	0.0%	0.0%	1.1%	4.7%
tr|I3LQI3|I3LQI3	4	3	4	0	0	0	0	1	8	7	24	0	0	0	0	3	18.7%	26.1%	30.6%	0.0%	0.0%	0.0%	0.0%	17.9%
tr|F1SPP8|F1SPP8	9	0	4	0	0	0	0	3	15	0	26	0	0	0	0	3	19.3%	0.0%	9.5%	0.0%	0.0%	0.0%	0.0%	10.9%
tr|F1RHC7|F1RHC7	5	1	0	0	0	0	0	1	11	4	0	0	0	0	0	1	8.6%	0.8%	0.0%	0.0%	0.0%	0.0%	0.0%	3.1%
tr|F1RS44|F1RS44	4	2	0	0	0	0	0	1	13	2	0	0	0	0	0	1	9.2%	3.2%	0.0%	0.0%	0.0%	0.0%	0.0%	4.7%
tr|F1S1X9|F1S1X9	7	2	6	0	0	0	0	1	12	3	110	0	0	0	0	2	27.7%	11.1%	20.8%	0.0%	0.0%	0.0%	0.0%	7.3%
tr|F1S4Y4|F1S4Y4	2	1	0	0	0	0	0	0	4	11	0	0	0	0	0	0	8.0%	4.1%	0.0%	0.0%	0.0%	0.0%	0.0%	0.0%
tr|F1RT47|F1RT47	3	4	0	0	0	0	0	0	5	10	0	0	0	0	0	0	16.9%	15.9%	0.0%	0.0%	0.0%	0.0%	0.0%	0.0%

	Peptide Count								Spectral Count								Sequence Coverage							
	Whole Cell	Whole Cell	Whole Cell	Cilia Fraction	Cilia Fraction	Cilia Fraction	Cilia Fraction	Cilia Fraction	Whole Cell	Whole Cell	Whole Cell	Cilia Fraction	Cilia Fraction	Cilia Fraction	Cilia Fraction	Cilia Fraction	Whole Cell	Whole Cell	Whole Cell	Cilia Fraction	Cilia Fraction	Cilia Fraction	Cilia Fraction	Cilia Fraction
Sample#	1	2	6	4	3	5	7	8	1	2	6	4	3	5	7	8	1	2	6	4	3	5	7	8
**Accession**																								
P81045	5	1	0	0	0	0	0	0	9	6	0	0	0	0	0	0	64.5%	25.8%	0.0%	0.0%	0.0%	0.0%	0.0%	0.0%
Reverse_tr|F1SRM1|F1SRM1	0	1	0	0	0	0	0	0	0	15	0	0	0	0	0	0	0.0%	0.3%	0.0%	0.0%	0.0%	0.0%	0.0%	0.0%
tr|I3LP43|I3LP43	7	3	0	0	0	0	0	0	9	6	0	0	0	0	0	0	12.6%	3.9%	0.0%	0.0%	0.0%	0.0%	0.0%	0.0%
tr|I3LFW7|I3LFW7	3	4	0	0	0	0	0	0	5	10	0	0	0	0	0	0	18.5%	17.4%	0.0%	0.0%	0.0%	0.0%	0.0%	0.0%
tr|F1RJM3|F1RJM3	7	3	0	0	0	0	0	0	9	6	0	0	0	0	0	0	12.6%	3.9%	0.0%	0.0%	0.0%	0.0%	0.0%	0.0%
tr|D2XUP7|D2XUP7	5	1	0	0	0	0	0	0	9	6	0	0	0	0	0	0	63.5%	25.4%	0.0%	0.0%	0.0%	0.0%	0.0%	0.0%
tr|I3LCW3|I3LCW3	10	1	2	0	0	0	0	0	14	1	3	0	0	0	0	0	4.8%	0.5%	1.2%	0.0%	0.0%	0.0%	0.0%	0.0%
tr|K7GKS8|K7GKS8	5	1	1	0	0	0	0	0	11	4	8	0	0	0	0	0	10.3%	1.9%	2.3%	0.0%	0.0%	0.0%	0.0%	0.0%
tr|F1SVC4|F1SVC4	5	1	1	0	0	0	0	0	11	4	8	0	0	0	0	0	10.3%	1.9%	2.3%	0.0%	0.0%	0.0%	0.0%	0.0%
tr|F1SI13|F1SI13	7	2	1	0	0	0	0	0	8	7	12	0	0	0	0	0	51.2%	7.4%	10.7%	0.0%	0.0%	0.0%	0.0%	0.0%
tr|F1SU05|F1SU05	5	1	3	0	0	0	0	0	8	7	14	0	0	0	0	0	8.8%	1.3%	4.7%	0.0%	0.0%	0.0%	0.0%	0.0%
tr|I3LSP1|I3LSP1	6	2	2	0	0	0	0	0	9	6	28	0	0	0	0	0	11.7%	4.4%	8.2%	0.0%	0.0%	0.0%	0.0%	0.0%
tr|F1S479|F1S479	3	1	0	0	0	0	1	1	4	10	0	0	0	0	6	3	16.2%	5.9%	0.0%	0.0%	0.0%	0.0%	5.5%	5.5%
tr|B2CCY7|B2CCY7	8	3	6	0	0	0	1	3	11	3	42	0	0	0	8	21	13.9%	5.6%	14.3%	0.0%	0.0%	0.0%	6.3%	22.6%
tr|F1RR44|F1RR44	7	0	1	0	0	0	0	2	14	0	3	0	0	0	0	8	27.7%	0.0%	3.6%	0.0%	0.0%	0.0%	0.0%	14.3%
tr|F2Z509|F2Z509	7	1	1	0	0	0	0	2	13	1	2	0	0	0	0	6	25.7%	2.5%	2.5%	0.0%	0.0%	0.0%	0.0%	9.8%
tr|F1SNK3|F1SNK3	7	1	3	0	0	0	1	2	10	4	13	0	0	0	3	7	38.4%	3.6%	12.5%	0.0%	0.0%	0.0%	6.0%	6.0%
tr|I3LS26|I3LS26	6	0	2	0	0	0	0	2	14	0	7	0	0	0	0	6	22.4%	0.0%	4.0%	0.0%	0.0%	0.0%	0.0%	8.3%
tr|F1S0W9|F1S0W9	6	1	2	0	0	0	0	2	6	8	8	0	0	0	0	3	4.7%	0.9%	1.7%	0.0%	0.0%	0.0%	0.0%	2.8%
tr|F1S2G3|F1S2G3	9	0	2	0	0	0	1	1	14	0	9	0	0	0	2	1	50.0%	0.0%	12.5%	0.0%	0.0%	0.0%	12.5%	26.9%
tr|I3LTV1|I3LTV1	2	2	2	0	0	0	0	2	6	8	16	0	0	0	0	3	4.2%	4.2%	2.2%	0.0%	0.0%	0.0%	0.0%	4.2%
tr|F1SR94|F1SR94	2	2	2	0	0	0	0	2	6	8	16	0	0	0	0	3	7.3%	7.3%	3.9%	0.0%	0.0%	0.0%	0.0%	7.3%
tr|F1RGC9|F1RGC9	7	2	5	0	0	0	0	2	12	2	43	0	0	0	0	4	26.1%	5.6%	15.7%	0.0%	0.0%	0.0%	0.0%	7.2%
Q29090	7	1	1	0	0	0	0	1	13	1	2	0	0	0	0	1	27.0%	2.6%	2.6%	0.0%	0.0%	0.0%	0.0%	3.3%
tr|F1S296|F1S296	4	3	3	0	0	0	0	2	6	8	213	0	0	0	0	7	25.0%	19.8%	18.6%	0.0%	0.0%	0.0%	0.0%	11.0%
tr|K7GM72|K7GM72	4	3	3	0	0	0	0	2	6	8	213	0	0	0	0	7	26.4%	20.9%	19.6%	0.0%	0.0%	0.0%	0.0%	11.7%
tr|K7GK90|K7GK90	4	3	3	0	0	0	0	2	6	8	213	0	0	0	0	7	24.9%	19.7%	18.5%	0.0%	0.0%	0.0%	0.0%	11.0%
tr|F1S084|F1S084	5	2	0	0	0	0	0	0	9	5	0	0	0	0	0	0	22.7%	5.5%	0.0%	0.0%	0.0%	0.0%	0.0%	0.0%
tr|F1SAK7|F1SAK7	4	3	0	0	0	0	0	0	7	7	0	0	0	0	0	0	7.2%	2.4%	0.0%	0.0%	0.0%	0.0%	0.0%	0.0%
tr|K7GT08|K7GT08	1	2	0	0	0	0	0	0	1	13	0	0	0	0	0	0	28.6%	28.6%	0.0%	0.0%	0.0%	0.0%	0.0%	0.0%
tr|F1RKQ9|F1RKQ9	4	1	0	0	0	0	0	0	9	5	0	0	0	0	0	0	28.9%	8.4%	0.0%	0.0%	0.0%	0.0%	0.0%	0.0%
tr|F1S403|F1S403	1	2	0	0	0	0	0	0	1	13	0	0	0	0	0	0	3.7%	5.0%	0.0%	0.0%	0.0%	0.0%	0.0%	0.0%
tr|K7GN10|K7GN10	1	2	0	0	0	0	0	0	1	13	0	0	0	0	0	0	16.7%	16.7%	0.0%	0.0%	0.0%	0.0%	0.0%	0.0%
tr|F1S8S1|F1S8S1	0	1	0	0	0	0	0	0	0	14	0	0	0	0	0	0	0.0%	2.4%	0.0%	0.0%	0.0%	0.0%	0.0%	0.0%
tr|I3LS69|I3LS69	2	2	0	0	0	0	0	0	2	12	0	0	0	0	0	0	26.8%	26.8%	0.0%	0.0%	0.0%	0.0%	0.0%	0.0%
tr|K7GP63|K7GP63	1	2	0	0	0	0	0	0	1	13	0	0	0	0	0	0	19.6%	19.6%	0.0%	0.0%	0.0%	0.0%	0.0%	0.0%
tr|F1SHL4|F1SHL4	2	1	0	0	0	0	0	0	11	3	0	0	0	0	0	0	5.4%	2.9%	0.0%	0.0%	0.0%	0.0%	0.0%	0.0%
tr|F1SQW1|F1SQW1	4	1	1	0	0	0	0	0	11	3	1	0	0	0	0	0	53.1%	19.8%	11.1%	0.0%	0.0%	0.0%	0.0%	0.0%
tr|F1RNI3|F1RNI3	3	1	1	0	0	0	0	0	3	11	1	0	0	0	0	0	8.6%	3.8%	5.2%	0.0%	0.0%	0.0%	0.0%	0.0%
tr|F1RPB7|F1RPB7	5	3	2	0	0	0	0	0	5	9	6	0	0	0	0	0	19.0%	8.9%	6.4%	0.0%	0.0%	0.0%	0.0%	0.0%
tr|I3LJH3|I3LJH3	4	1	2	0	0	0	0	0	7	7	6	0	0	0	0	0	32.8%	8.6%	24.2%	0.0%	0.0%	0.0%	0.0%	0.0%
B3SP85	8	0	1	0	0	0	0	0	14	0	6	0	0	0	0	0	41.5%	0.0%	5.3%	0.0%	0.0%	0.0%	0.0%	0.0%
tr|I3LNL7|I3LNL7	7	0	3	0	0	0	0	0	14	0	12	0	0	0	0	0	10.0%	0.0%	8.7%	0.0%	0.0%	0.0%	0.0%	0.0%
tr|F1SQ00|F1SQ00	6	3	4	0	0	0	0	0	7	7	19	0	0	0	0	0	21.0%	8.0%	9.8%	0.0%	0.0%	0.0%	0.0%	0.0%
tr|F1RWB7|F1RWB7	6	0	2	0	0	0	0	0	14	0	25	0	0	0	0	0	7.9%	0.0%	5.0%	0.0%	0.0%	0.0%	0.0%	0.0%
tr|F1SND2|F1SND2	7	3	4	0	0	0	0	0	11	3	28	0	0	0	0	0	22.5%	6.2%	12.2%	0.0%	0.0%	0.0%	0.0%	0.0%
tr|I3LHM3|I3LHM3	7	2	7	0	0	0	0	0	11	3	29	0	0	0	0	0	12.7%	2.8%	8.7%	0.0%	0.0%	0.0%	0.0%	0.0%
tr|F1SDV7|F1SDV7	7	2	7	0	0	0	0	0	11	3	29	0	0	0	0	0	13.5%	3.0%	9.2%	0.0%	0.0%	0.0%	0.0%	0.0%
tr|F1SK95|F1SK95	7	3	8	0	0	0	0	0	10	4	44	0	0	0	0	0	22.4%	10.4%	22.2%	0.0%	0.0%	0.0%	0.0%	0.0%
tr|F1S854|F1S854	3	2	2	0	0	0	2	2	7	6	7	0	0	0	11	31	42.5%	45.0%	27.5%	0.0%	0.0%	0.0%	57.5%	60.0%
tr|F1S2K3|F1S2K3	3	2	0	0	0	0	1	2	6	7	0	0	0	0	7	14	44.9%	23.6%	0.0%	0.0%	0.0%	0.0%	18.1%	12.6%
tr|F1SH60|F1SH60	3	0	1	0	0	0	2	2	13	0	1	0	0	0	8	12	19.7%	0.0%	7.7%	0.0%	0.0%	0.0%	12.0%	12.0%
tr|F1SIG7|F1SIG7	5	0	2	0	0	0	3	3	13	0	8	0	0	0	15	8	12.3%	0.0%	5.9%	0.0%	0.0%	0.0%	10.4%	13.0%
tr|I3L700|I3L700	3	2	0	0	0	0	0	2	6	7	0	0	0	0	0	14	44.5%	23.4%	0.0%	0.0%	0.0%	0.0%	0.0%	12.5%
tr|I3LR51|I3LR51	3	0	0	0	0	0	0	2	13	0	0	0	0	0	0	13	26.6%	0.0%	0.0%	0.0%	0.0%	0.0%	0.0%	12.1%
tr|K7GMF2|K7GMF2	8	0	1	0	0	0	1	2	13	0	7	0	0	0	8	7	54.3%	0.0%	16.3%	0.0%	0.0%	0.0%	16.3%	21.7%
tr|F1S4U9|F1S4U9	8	1	1	0	0	0	1	2	11	2	3	0	0	0	6	6	9.9%	1.2%	1.9%	0.0%	0.0%	0.0%	2.4%	2.6%
tr|F1RRT3|F1RRT3	8	0	2	0	0	0	1	3	13	0	8	0	0	0	7	7	26.5%	0.0%	12.7%	0.0%	0.0%	0.0%	4.3%	11.4%
tr|F1SIX7|F1SIX7	6	2	3	0	0	0	2	1	9	4	18	0	0	0	2	12	24.6%	6.3%	10.3%	0.0%	0.0%	0.0%	9.5%	4.6%
tr|F1SMN1|F1SMN1	9	0	12	0	0	0	1	4	13	0	91	0	0	0	17	23	39.9%	0.0%	33.0%	0.0%	0.0%	0.0%	4.1%	21.7%
Q28960	5	0	1	0	0	0	0	3	13	0	11	0	0	0	0	9	15.2%	0.0%	4.2%	0.0%	0.0%	0.0%	0.0%	15.9%
tr|F2Z523|F2Z523	3	0	1	0	0	0	0	1	13	0	2	0	0	0	0	5	19.2%	0.0%	6.6%	0.0%	0.0%	0.0%	0.0%	7.9%
tr|F1RFV5|F1RFV5	5	0	0	0	0	0	0	1	13	0	0	0	0	0	0	4	43.6%	0.0%	0.0%	0.0%	0.0%	0.0%	0.0%	16.2%
tr|I3L9C8|I3L9C8	6	1	2	0	0	0	0	2	11	2	4	0	0	0	0	5	17.3%	2.0%	4.6%	0.0%	0.0%	0.0%	0.0%	5.8%
tr|F1RUJ9|F1RUJ9	6	2	5	0	0	0	1	3	11	2	15	0	0	0	1	7	9.5%	4.1%	9.2%	0.0%	0.0%	0.0%	2.6%	6.4%
tr|K7GK99|K7GK99	6	2	5	0	0	0	1	3	11	2	15	0	0	0	1	7	10.6%	4.6%	10.3%	0.0%	0.0%	0.0%	2.9%	7.2%
tr|F1SFR2|F1SFR2	6	1	4	0	0	0	1	1	12	1	22	0	0	0	8	1	48.1%	9.4%	30.6%	0.0%	0.0%	0.0%	11.2%	11.2%
tr|F1RL02|F1RL02	7	0	6	0	0	0	0	4	13	0	29	0	0	0	0	10	40.6%	0.0%	20.9%	0.0%	0.0%	0.0%	0.0%	24.3%
tr|K7GKC0|K7GKC0	3	3	1	0	0	0	0	2	9	4	29	0	0	0	0	9	22.7%	32.0%	10.3%	0.0%	0.0%	0.0%	0.0%	12.4%
tr|F1SGC8|F1SGC8	7	1	0	0	0	0	0	1	12	1	0	0	0	0	0	2	19.8%	3.0%	0.0%	0.0%	0.0%	0.0%	0.0%	7.0%
tr|F1S365|F1S365	6	0	0	0	0	0	0	1	13	0	0	0	0	0	0	2	30.5%	0.0%	0.0%	0.0%	0.0%	0.0%	0.0%	9.5%
tr|I3L6M8|I3L6M8	8	1	8	0	0	0	3	1	12	1	196	0	0	0	22	6	44.4%	6.4%	49.1%	0.0%	0.0%	0.0%	25.7%	6.4%
tr|F1SLA8|F1SLA8	2	1	6	0	0	0	0	1	11	2	38	0	0	0	0	3	12.0%	8.9%	27.7%	0.0%	0.0%	0.0%	0.0%	12.0%
Q863I2	4	4	1	0	0	0	0	1	4	9	23	0	0	0	0	2	9.8%	7.2%	3.4%	0.0%	0.0%	0.0%	0.0%	4.7%
tr|F1RX51|F1RX51	6	0	1	0	0	0	1	0	13	0	9	0	0	0	1	0	49.2%	0.0%	9.4%	0.0%	0.0%	0.0%	10.5%	0.0%
tr|I3LKS6|I3LKS6	5	5	2	0	0	0	0	1	5	8	14	0	0	0	0	1	30.5%	22.6%	16.5%	0.0%	0.0%	0.0%	0.0%	5.3%
Q8MJ30	5	5	2	0	0	0	0	1	5	8	14	0	0	0	0	1	30.5%	22.6%	16.5%	0.0%	0.0%	0.0%	0.0%	5.3%
tr|I3L976|I3L976	2	1	0	0	0	0	0	0	4	9	0	0	0	0	0	0	3.7%	2.2%	0.0%	0.0%	0.0%	0.0%	0.0%	0.0%
tr|F1SJN9|F1SJN9	2	2	0	0	0	0	0	0	3	10	0	0	0	0	0	0	22.1%	5.5%	0.0%	0.0%	0.0%	0.0%	0.0%	0.0%
tr|F1SBE8|F1SBE8	4	2	0	0	0	0	0	0	7	6	0	0	0	0	0	0	2.6%	1.3%	0.0%	0.0%	0.0%	0.0%	0.0%	0.0%
tr|F1SVC3|F1SVC3	1	3	0	0	0	0	0	0	1	12	0	0	0	0	0	0	0.8%	2.2%	0.0%	0.0%	0.0%	0.0%	0.0%	0.0%

	Peptide Count								Spectral Count								Sequence Coverage							
	Whole Cell	Whole Cell	Whole Cell	Cilia Fraction	Cilia Fraction	Cilia Fraction	Cilia Fraction	Cilia Fraction	Whole Cell	Whole Cell	Whole Cell	Cilia Fraction	Cilia Fraction	Cilia Fraction	Cilia Fraction	Cilia Fraction	Whole Cell	Whole Cell	Whole Cell	Cilia Fraction	Cilia Fraction	Cilia Fraction	Cilia Fraction	Cilia Fraction
Sample#	1	2	6	4	3	5	7	8	1	2	6	4	3	5	7	8	1	2	6	4	3	5	7	8
**Accession**																								
tr|I3LLU6|I3LLU6	0	3	0	0	0	0	0	0	0	13	0	0	0	0	0	0	0.0%	1.2%	0.0%	0.0%	0.0%	0.0%	0.0%	0.0%
tr|I3LBW5|I3LBW5	3	0	0	0	0	0	0	0	13	0	0	0	0	0	0	0	31.3%	0.0%	0.0%	0.0%	0.0%	0.0%	0.0%	0.0%
tr|I3LM15|I3LM15	5	2	0	0	0	0	0	0	10	3	0	0	0	0	0	0	14.1%	3.4%	0.0%	0.0%	0.0%	0.0%	0.0%	0.0%
tr|I3LI86|I3LI86	3	1	0	0	0	0	0	0	3	10	0	0	0	0	0	0	4.6%	1.3%	0.0%	0.0%	0.0%	0.0%	0.0%	0.0%
tr|F1SQM5|F1SQM5	0	3	0	0	0	0	0	0	0	13	0	0	0	0	0	0	0.0%	1.2%	0.0%	0.0%	0.0%	0.0%	0.0%	0.0%
tr|I3L9M5|I3L9M5	0	1	0	0	0	0	0	0	0	13	0	0	0	0	0	0	0.0%	14.8%	0.0%	0.0%	0.0%	0.0%	0.0%	0.0%
tr|F1SLI4|F1SLI4	8	2	0	0	0	0	0	0	9	4	0	0	0	0	0	0	9.7%	2.0%	0.0%	0.0%	0.0%	0.0%	0.0%	0.0%
tr|F1STX7|F1STX7	5	1	0	0	0	0	0	0	6	7	0	0	0	0	0	0	7.6%	1.3%	0.0%	0.0%	0.0%	0.0%	0.0%	0.0%
tr|F1RKG3|F1RKG3	3	3	0	0	0	0	0	0	5	8	0	0	0	0	0	0	13.6%	10.7%	0.0%	0.0%	0.0%	0.0%	0.0%	0.0%
tr|I3LK09|I3LK09	1	4	0	0	0	0	0	0	1	12	0	0	0	0	0	0	3.1%	13.6%	0.0%	0.0%	0.0%	0.0%	0.0%	0.0%
tr|F1SB49|F1SB49	0	1	0	0	0	0	0	0	0	13	0	0	0	0	0	0	0.0%	3.1%	0.0%	0.0%	0.0%	0.0%	0.0%	0.0%
tr|F1STX8|F1STX8	5	1	0	0	0	0	0	0	6	7	0	0	0	0	0	0	7.4%	1.3%	0.0%	0.0%	0.0%	0.0%	0.0%	0.0%
tr|F1RTB0|F1RTB0	4	3	1	0	0	0	0	0	7	6	2	0	0	0	0	0	23.1%	10.3%	14.2%	0.0%	0.0%	0.0%	0.0%	0.0%
tr|I3LHS1|I3LHS1	4	1	1	0	0	0	0	0	10	3	2	0	0	0	0	0	16.0%	3.9%	3.9%	0.0%	0.0%	0.0%	0.0%	0.0%
tr|I3LT20|I3LT20	4	3	1	0	0	0	0	0	5	8	2	0	0	0	0	0	29.7%	9.6%	4.5%	0.0%	0.0%	0.0%	0.0%	0.0%
tr|F1SB55|F1SB55	4	1	1	0	0	0	0	0	10	3	2	0	0	0	0	0	16.0%	3.9%	3.9%	0.0%	0.0%	0.0%	0.0%	0.0%
tr|F1S665|F1S665	6	2	2	0	0	0	0	0	10	3	4	0	0	0	0	0	54.0%	11.2%	15.5%	0.0%	0.0%	0.0%	0.0%	0.0%
tr|F1S2E3|F1S2E3	4	2	2	0	0	0	0	0	6	7	9	0	0	0	0	0	38.6%	12.1%	16.4%	0.0%	0.0%	0.0%	0.0%	0.0%
tr|I3LHU9|I3LHU9	4	2	1	0	0	0	0	0	8	5	12	0	0	0	0	0	7.5%	3.1%	1.5%	0.0%	0.0%	0.0%	0.0%	0.0%
tr|F1SUQ3|F1SUQ3	4	2	1	0	0	0	0	0	8	5	12	0	0	0	0	0	7.5%	3.1%	1.5%	0.0%	0.0%	0.0%	0.0%	0.0%
tr|F1RHN4|F1RHN4	5	0	2	0	0	0	0	0	13	0	12	0	0	0	0	0	12.5%	0.0%	5.7%	0.0%	0.0%	0.0%	0.0%	0.0%
tr|F1SFL7|F1SFL7	2	1	5	0	0	0	0	0	3	10	15	0	0	0	0	0	4.1%	2.3%	18.0%	0.0%	0.0%	0.0%	0.0%	0.0%
tr|K7GKD3|K7GKD3	3	2	5	0	0	0	0	0	9	4	23	0	0	0	0	0	9.4%	5.8%	12.9%	0.0%	0.0%	0.0%	0.0%	0.0%
tr|K7GQ87|K7GQ87	3	2	5	0	0	0	0	0	9	4	23	0	0	0	0	0	9.8%	6.0%	13.5%	0.0%	0.0%	0.0%	0.0%	0.0%
tr|K7GQW0|K7GQW0	3	2	5	0	0	0	0	0	9	4	23	0	0	0	0	0	10.3%	6.3%	14.0%	0.0%	0.0%	0.0%	0.0%	0.0%
tr|F1SFK0|F1SFK0	3	2	5	0	0	0	0	0	9	4	23	0	0	0	0	0	9.4%	5.7%	12.8%	0.0%	0.0%	0.0%	0.0%	0.0%
tr|F1S4Y8|F1S4Y8	7	2	9	0	0	0	0	0	9	4	33	0	0	0	0	0	25.6%	6.7%	29.9%	0.0%	0.0%	0.0%	0.0%	0.0%
tr|F1RHG1|F1RHG1	8	1	7	0	0	0	0	0	11	2	38	0	0	0	0	0	10.2%	1.5%	11.8%	0.0%	0.0%	0.0%	0.0%	0.0%
Q95339	3	2	2	0	0	0	0	0	9	4	47	0	0	0	0	0	27.3%	27.3%	27.3%	0.0%	0.0%	0.0%	0.0%	0.0%
tr|F1REX4|F1REX4	3	2	2	0	0	0	0	0	9	4	47	0	0	0	0	0	27.6%	27.6%	27.6%	0.0%	0.0%	0.0%	0.0%	0.0%
tr|F1SDG0|F1SDG0	3	2	1	0	0	0	1	1	7	5	3	0	0	0	30	1	15.1%	8.7%	3.3%	0.0%	0.0%	0.0%	4.7%	5.7%
tr|I3L6T0|I3L6T0	4	1	2	0	0	0	3	4	11	1	9	0	0	0	21	9	15.6%	6.7%	6.0%	0.0%	0.0%	0.0%	10.5%	19.0%
tr|F1REX9|F1REX9	4	2	1	0	0	0	1	2	9	3	1	0	0	0	5	12	18.9%	10.8%	4.6%	0.0%	0.0%	0.0%	4.6%	6.2%
tr|F6Q4Y6|F6Q4Y6	6	2	3	0	0	0	2	4	8	4	38	0	0	0	29	12	39.7%	10.3%	23.8%	0.0%	0.0%	0.0%	19.2%	36.4%
tr|F1SBE9|F1SBE9	2	2	0	0	0	0	1	0	9	3	0	0	0	0	6	0	22.8%	22.8%	0.0%	0.0%	0.0%	0.0%	22.8%	0.0%
tr|F1RSG8|F1RSG8	8	1	3	0	0	0	2	2	11	1	19	0	0	0	8	6	32.3%	4.0%	9.3%	0.0%	0.0%	0.0%	9.3%	14.3%
tr|F1RWD5|F1RWD5	7	0	6	0	0	0	2	1	12	0	55	0	0	0	19	5	18.2%	0.0%	18.2%	0.0%	0.0%	0.0%	5.6%	5.6%
tr|I3LGT3|I3LGT3	8	2	11	0	0	0	2	3	9	3	78	0	0	0	6	17	21.4%	4.0%	32.3%	0.0%	0.0%	0.0%	5.3%	9.1%
tr|F1S4X9|F1S4X9	5	2	2	0	0	0	2	0	9	3	19	0	0	0	7	0	19.8%	6.3%	6.1%	0.0%	0.0%	0.0%	7.4%	0.0%
tr|F1RN28|F1RN28	7	1	5	0	0	0	1	3	9	3	35	0	0	0	4	4	23.0%	2.6%	18.7%	0.0%	0.0%	0.0%	5.0%	16.1%
tr|F1S7T1|F1S7T1	8	2	2	0	0	0	0	1	10	2	6	0	0	0	0	3	3.8%	0.8%	1.7%	0.0%	0.0%	0.0%	0.0%	0.6%
tr|I3L851|I3L851	4	1	0	0	0	0	1	1	8	4	0	0	0	0	1	1	26.5%	5.6%	0.0%	0.0%	0.0%	0.0%	14.3%	14.3%
tr|F1RSN3|F1RSN3	2	1	1	0	0	0	0	1	2	10	1	0	0	0	0	2	5.8%	1.5%	1.6%	0.0%	0.0%	0.0%	0.0%	3.3%
P47788	6	2	5	0	0	0	1	0	10	2	11	0	0	0	3	0	16.0%	1.6%	8.6%	0.0%	0.0%	0.0%	3.2%	0.0%
tr|F1S8G5|F1S8G5	6	2	5	0	0	0	1	0	10	2	11	0	0	0	3	0	16.0%	1.6%	8.6%	0.0%	0.0%	0.0%	3.2%	0.0%
tr|Q684M6|Q684M6	7	2	11	0	0	0	1	2	10	2	65	0	0	0	1	8	24.2%	8.4%	27.6%	0.0%	0.0%	0.0%	4.2%	9.7%
tr|I3L8I5|I3L8I5	5	0	3	0	0	0	0	1	12	0	14	0	0	0	0	3	47.9%	0.0%	18.0%	0.0%	0.0%	0.0%	0.0%	11.8%
tr|F1RUZ6|F1RUZ6	9	0	3	0	0	0	0	1	12	0	9	0	0	0	0	2	26.7%	0.0%	15.5%	0.0%	0.0%	0.0%	0.0%	3.0%
tr|F1SS19|F1SS19	8	1	3	0	0	0	1	1	10	2	11	0	0	0	1	1	15.7%	1.2%	6.8%	0.0%	0.0%	0.0%	3.8%	3.8%
tr|F4ZS20|F4ZS20	4	0	1	0	0	0	0	1	12	0	1	0	0	0	0	1	44.4%	0.0%	7.2%	0.0%	0.0%	0.0%	0.0%	12.4%
tr|Q6IM70|Q6IM70	3	0	1	0	0	0	0	1	12	0	1	0	0	0	0	1	14.1%	0.0%	7.0%	0.0%	0.0%	0.0%	0.0%	8.0%
tr|I3LDC3|I3LDC3	4	1	3	0	0	0	0	1	11	1	4	0	0	0	0	1	30.7%	11.4%	20.5%	0.0%	0.0%	0.0%	0.0%	8.0%
tr|F1RX84|F1RX84	6	0	2	0	0	0	1	0	12	0	5	0	0	0	1	0	24.4%	0.0%	10.9%	0.0%	0.0%	0.0%	6.2%	0.0%
tr|I3LIA3|I3LIA3	5	0	7	0	0	0	0	1	12	0	47	0	0	0	0	3	10.1%	0.0%	11.2%	0.0%	0.0%	0.0%	0.0%	2.4%
tr|I3LIL5|I3LIL5	3	2	2	0	0	0	1	0	9	3	14	0	0	0	1	0	11.7%	11.4%	4.0%	0.0%	0.0%	0.0%	4.3%	0.0%
tr|I3LUR1|I3LUR1	8	0	9	0	0	0	0	1	12	0	40	0	0	0	0	1	12.7%	0.0%	15.7%	0.0%	0.0%	0.0%	0.0%	3.2%
tr|F1RH32|F1RH32	3	4	0	0	0	0	0	0	7	5	0	0	0	0	0	0	6.3%	3.8%	0.0%	0.0%	0.0%	0.0%	0.0%	0.0%
tr|I3LDF6|I3LDF6	3	2	0	0	0	0	0	0	4	8	0	0	0	0	0	0	7.1%	2.9%	0.0%	0.0%	0.0%	0.0%	0.0%	0.0%
tr|F1SAH5|F1SAH5	0	1	0	0	0	0	0	0	0	12	0	0	0	0	0	0	0.0%	2.7%	0.0%	0.0%	0.0%	0.0%	0.0%	0.0%
tr|I3LKZ3|I3LKZ3	2	1	0	0	0	0	0	0	2	10	0	0	0	0	0	0	17.3%	8.0%	0.0%	0.0%	0.0%	0.0%	0.0%	0.0%
tr|F1S534|F1S534	5	0	0	0	0	0	0	0	12	0	0	0	0	0	0	0	31.8%	0.0%	0.0%	0.0%	0.0%	0.0%	0.0%	0.0%
tr|F2Z5U3|F2Z5U3	5	2	0	0	0	0	0	0	6	6	0	0	0	0	0	0	15.7%	8.3%	0.0%	0.0%	0.0%	0.0%	0.0%	0.0%
tr|F1RZQ7|F1RZQ7	4	0	0	0	0	0	0	0	12	0	0	0	0	0	0	0	32.0%	0.0%	0.0%	0.0%	0.0%	0.0%	0.0%	0.0%
tr|F1RU40|F1RU40	7	1	0	0	0	0	0	0	8	4	0	0	0	0	0	0	18.5%	2.2%	0.0%	0.0%	0.0%	0.0%	0.0%	0.0%
tr|F1SK25|F1SK25	0	3	0	0	0	0	0	0	0	12	0	0	0	0	0	0	0.0%	3.5%	0.0%	0.0%	0.0%	0.0%	0.0%	0.0%
tr|F1S3J8|F1S3J8	5	1	0	0	0	0	0	0	10	2	0	0	0	0	0	0	26.9%	4.1%	0.0%	0.0%	0.0%	0.0%	0.0%	0.0%
tr|F1SJZ7|F1SJZ7	1	1	0	0	0	0	0	0	1	11	0	0	0	0	0	0	12.5%	12.5%	0.0%	0.0%	0.0%	0.0%	0.0%	0.0%
tr|I3LBK3|I3LBK3	3	1	1	0	0	0	0	0	9	3	2	0	0	0	0	0	10.8%	3.9%	3.9%	0.0%	0.0%	0.0%	0.0%	0.0%
tr|F1SF21|F1SF21	8	0	1	0	0	0	0	0	12	0	2	0	0	0	0	0	18.1%	0.0%	1.9%	0.0%	0.0%	0.0%	0.0%	0.0%
P00377	5	2	2	0	0	0	0	0	7	5	3	0	0	0	0	0	33.3%	9.7%	18.3%	0.0%	0.0%	0.0%	0.0%	0.0%
tr|F1RF10|F1RF10	5	2	2	0	0	0	0	0	7	5	3	0	0	0	0	0	33.3%	9.7%	18.3%	0.0%	0.0%	0.0%	0.0%	0.0%
tr|F1SID9|F1SID9	3	2	1	0	0	0	0	0	6	6	3	0	0	0	0	0	15.7%	6.1%	5.2%	0.0%	0.0%	0.0%	0.0%	0.0%
tr|I3LA84|I3LA84	3	1	2	0	0	0	0	0	11	1	3	0	0	0	0	0	6.1%	2.3%	4.3%	0.0%	0.0%	0.0%	0.0%	0.0%
tr|F1S155|F1S155	7	0	1	0	0	0	0	0	12	0	3	0	0	0	0	0	13.2%	0.0%	2.2%	0.0%	0.0%	0.0%	0.0%	0.0%
tr|I3LFF1|I3LFF1	3	2	1	0	0	0	0	0	5	7	3	0	0	0	0	0	25.4%	12.0%	10.0%	0.0%	0.0%	0.0%	0.0%	0.0%
tr|F1RFD3|F1RFD3	6	0	4	0	0	0	0	0	12	0	7	0	0	0	0	0	9.2%	0.0%	5.5%	0.0%	0.0%	0.0%	0.0%	0.0%
tr|D0G0B3|D0G0B3	8	0	2	0	0	0	0	0	12	0	7	0	0	0	0	0	29.2%	0.0%	9.6%	0.0%	0.0%	0.0%	0.0%	0.0%
tr|K7GKT5|K7GKT5	2	3	1	0	0	0	0	0	4	8	7	0	0	0	0	0	15.9%	22.5%	8.6%	0.0%	0.0%	0.0%	0.0%	0.0%
tr|I3LQZ7|I3LQZ7	6	0	4	0	0	0	0	0	12	0	7	0	0	0	0	0	9.6%	0.0%	5.8%	0.0%	0.0%	0.0%	0.0%	0.0%
tr|F1S897|F1S897	6	1	1	0	0	0	0	0	9	3	8	0	0	0	0	0	24.3%	2.3%	5.0%	0.0%	0.0%	0.0%	0.0%	0.0%
tr|F1SNA8|F1SNA8	6	3	2	0	0	0	0	0	8	4	8	0	0	0	0	0	18.9%	12.0%	5.0%	0.0%	0.0%	0.0%	0.0%	0.0%

	Peptide Count								Spectral Count								Sequence Coverage							
	Whole Cell	Whole Cell	Whole Cell	Cilia Fraction	Cilia Fraction	Cilia Fraction	Cilia Fraction	Cilia Fraction	Whole Cell	Whole Cell	Whole Cell	Cilia Fraction	Cilia Fraction	Cilia Fraction	Cilia Fraction	Cilia Fraction	Whole Cell	Whole Cell	Whole Cell	Cilia Fraction	Cilia Fraction	Cilia Fraction	Cilia Fraction	Cilia Fraction
Sample#	1	2	6	4	3	5	7	8	1	2	6	4	3	5	7	8	1	2	6	4	3	5	7	8
**Accession**																								
tr|F1S300|F1S300	7	1	5	0	0	0	0	0	9	3	12	0	0	0	0	0	4.6%	0.8%	3.1%	0.0%	0.0%	0.0%	0.0%	0.0%
tr|F1RHM9|F1RHM9	5	1	1	0	0	0	0	0	7	5	12	0	0	0	0	0	12.4%	1.6%	5.5%	0.0%	0.0%	0.0%	0.0%	0.0%
tr|I3LKF1|I3LKF1	6	2	5	0	0	0	0	0	7	5	33	0	0	0	0	0	20.1%	16.0%	24.9%	0.0%	0.0%	0.0%	0.0%	0.0%
tr|F1RJ01|F1RJ01	6	2	5	0	0	0	0	0	7	5	33	0	0	0	0	0	16.3%	13.0%	20.2%	0.0%	0.0%	0.0%	0.0%	0.0%
tr|K7GSE9|K7GSE9	6	0	6	0	0	0	0	0	12	0	58	0	0	0	0	0	25.9%	0.0%	29.4%	0.0%	0.0%	0.0%	0.0%	0.0%
tr|I3L638|I3L638	3	2	0	0	0	0	0	1	4	7	0	0	0	0	0	10	7.2%	3.9%	0.0%	0.0%	0.0%	0.0%	0.0%	1.7%
tr|I3L9F8|I3L9F8	3	2	0	0	0	0	0	1	4	7	0	0	0	0	0	10	8.5%	4.7%	0.0%	0.0%	0.0%	0.0%	0.0%	2.1%
P48819	3	2	0	0	0	0	0	1	4	7	0	0	0	0	0	10	7.2%	3.9%	0.0%	0.0%	0.0%	0.0%	0.0%	1.7%
tr|I3LP50|I3LP50	3	2	0	0	0	0	0	1	4	7	0	0	0	0	0	10	7.2%	3.9%	0.0%	0.0%	0.0%	0.0%	0.0%	1.7%
tr|I3L998|I3L998	3	2	0	0	0	0	0	1	4	7	0	0	0	0	0	10	8.5%	4.7%	0.0%	0.0%	0.0%	0.0%	0.0%	2.1%
tr|F1RWW4|F1RWW4	4	4	1	0	0	0	0	2	6	5	1	0	0	0	0	9	6.5%	7.0%	2.0%	0.0%	0.0%	0.0%	0.0%	5.6%
tr|F1S0N6|F1S0N6	4	0	0	0	0	0	1	2	11	0	0	0	0	0	1	5	12.6%	0.0%	0.0%	0.0%	0.0%	0.0%	3.6%	6.9%
tr|I3L5V7|I3L5V7	4	0	3	0	0	0	0	2	11	0	8	0	0	0	0	9	43.0%	0.0%	18.3%	0.0%	0.0%	0.0%	0.0%	35.2%
tr|F1SKH3|F1SKH3	7	0	0	0	0	0	0	1	11	0	0	0	0	0	0	5	6.2%	0.0%	0.0%	0.0%	0.0%	0.0%	0.0%	1.4%
tr|B5APU8|B5APU8	4	0	3	0	0	0	3	2	11	0	14	0	0	0	8	2	30.9%	0.0%	19.1%	0.0%	0.0%	0.0%	19.7%	23.6%
tr|F2Z540|F2Z540	4	2	0	0	0	0	1	0	7	4	0	0	0	0	4	0	9.0%	7.8%	0.0%	0.0%	0.0%	0.0%	5.7%	0.0%
tr|F1SB18|F1SB18	6	1	1	0	0	0	0	3	10	1	9	0	0	0	0	6	14.9%	4.3%	3.8%	0.0%	0.0%	0.0%	0.0%	14.3%
tr|I3LFW6|I3LFW6	3	0	3	0	0	0	2	2	11	0	21	0	0	0	3	5	12.9%	0.0%	17.0%	0.0%	0.0%	0.0%	12.9%	27.9%
tr|I3LC26|I3LC26	2	2	1	0	0	0	0	1	4	7	7	0	0	0	0	4	6.1%	9.2%	5.4%	0.0%	0.0%	0.0%	0.0%	13.8%
tr|F2Z545|F2Z545	2	3	3	0	0	0	1	1	3	8	21	0	0	0	5	2	29.5%	44.6%	41.1%	0.0%	0.0%	0.0%	17.9%	11.6%
A7TX81	5	1	2	0	0	0	0	2	8	3	10	0	0	0	0	4	27.6%	3.4%	9.6%	0.0%	0.0%	0.0%	0.0%	13.0%
tr|F1SEY8|F1SEY8	5	3	4	0	0	0	0	1	8	3	11	0	0	0	0	2	10.0%	5.2%	10.3%	0.0%	0.0%	0.0%	0.0%	3.8%
tr|I7GF95|I7GF95	4	2	0	0	0	0	0	1	5	6	0	0	0	0	0	1	10.7%	3.8%	0.0%	0.0%	0.0%	0.0%	0.0%	3.0%
tr|F1RS34|F1RS34	5	1	2	0	0	0	0	1	10	1	19	0	0	0	0	2	4.8%	0.9%	2.4%	0.0%	0.0%	0.0%	0.0%	1.3%
tr|F1SN63|F1SN63	2	2	2	0	0	0	1	0	5	6	6	0	0	0	1	0	4.3%	4.7%	5.8%	0.0%	0.0%	0.0%	2.4%	0.0%
tr|F1RJ74|F1RJ74	7	1	6	0	0	0	1	1	10	1	24	0	0	0	1	1	22.7%	2.4%	22.7%	0.0%	0.0%	0.0%	5.9%	8.0%
tr|F1SGB0|F1SGB0	4	0	2	0	0	0	1	0	11	0	26	0	0	0	2	0	6.9%	0.0%	5.7%	0.0%	0.0%	0.0%	2.6%	0.0%
tr|I3LAK5|I3LAK5	4	0	3	0	0	0	1	0	11	0	31	0	0	0	2	0	5.9%	0.0%	7.4%	0.0%	0.0%	0.0%	2.3%	0.0%
tr|I3LH62|I3LH62	4	0	3	0	0	0	1	0	11	0	31	0	0	0	2	0	5.5%	0.0%	6.8%	0.0%	0.0%	0.0%	2.1%	0.0%
tr|F1SIY3|F1SIY3	4	2	2	0	0	0	1	0	7	4	14	0	0	0	1	0	25.6%	13.1%	11.3%	0.0%	0.0%	0.0%	11.3%	0.0%
tr|F1STY1|F1STY1	3	2	2	0	0	0	1	0	6	5	15	0	0	0	1	0	29.5%	24.6%	16.4%	0.0%	0.0%	0.0%	9.0%	0.0%
tr|F1RPV1|F1RPV1	5	0	0	0	0	0	0	0	11	0	0	0	0	0	0	0	22.7%	0.0%	0.0%	0.0%	0.0%	0.0%	0.0%	0.0%
tr|F1RT28|F1RT28	9	1	0	0	0	0	0	0	10	1	0	0	0	0	0	0	15.7%	1.4%	0.0%	0.0%	0.0%	0.0%	0.0%	0.0%
tr|F1RK23|F1RK23	0	2	0	0	0	0	0	0	0	11	0	0	0	0	0	0	0.0%	12.4%	0.0%	0.0%	0.0%	0.0%	0.0%	0.0%
tr|F1SSM7|F1SSM7	6	2	0	0	0	0	0	0	7	4	0	0	0	0	0	0	10.5%	4.3%	0.0%	0.0%	0.0%	0.0%	0.0%	0.0%
tr|F1SG88|F1SG88	7	1	0	0	0	0	0	0	10	1	0	0	0	0	0	0	15.5%	4.5%	0.0%	0.0%	0.0%	0.0%	0.0%	0.0%
tr|F1S9W8|F1S9W8	7	1	0	0	0	0	0	0	7	4	0	0	0	0	0	0	19.6%	2.5%	0.0%	0.0%	0.0%	0.0%	0.0%	0.0%
tr|F2Z5M0|F2Z5M0	0	1	0	0	0	0	0	0	0	11	0	0	0	0	0	0	0.0%	6.9%	0.0%	0.0%	0.0%	0.0%	0.0%	0.0%
tr|I3LEF1|I3LEF1	7	1	0	0	0	0	0	0	10	1	0	0	0	0	0	0	15.5%	4.5%	0.0%	0.0%	0.0%	0.0%	0.0%	0.0%
tr|F1S221|F1S221	1	3	0	0	0	0	0	0	1	10	0	0	0	0	0	0	2.5%	3.7%	0.0%	0.0%	0.0%	0.0%	0.0%	0.0%
tr|I3LRC8|I3LRC8	2	2	0	0	0	0	0	0	7	4	0	0	0	0	0	0	17.6%	11.3%	0.0%	0.0%	0.0%	0.0%	0.0%	0.0%
tr|F1SIV9|F1SIV9	4	1	0	0	0	0	0	0	9	2	0	0	0	0	0	0	10.7%	2.5%	0.0%	0.0%	0.0%	0.0%	0.0%	0.0%
tr|F1SSL4|F1SSL4	5	0	0	0	0	0	0	0	11	0	0	0	0	0	0	0	11.0%	0.0%	0.0%	0.0%	0.0%	0.0%	0.0%	0.0%
tr|F1SIB5|F1SIB5	5	2	0	0	0	0	0	0	7	4	0	0	0	0	0	0	5.1%	3.0%	0.0%	0.0%	0.0%	0.0%	0.0%	0.0%
tr|F1RHR8|F1RHR8	6	3	0	0	0	0	0	0	8	3	0	0	0	0	0	0	4.5%	1.9%	0.0%	0.0%	0.0%	0.0%	0.0%	0.0%
tr|Q2TJZ7|Q2TJZ7	1	2	0	0	0	0	0	0	1	10	0	0	0	0	0	0	15.7%	15.7%	0.0%	0.0%	0.0%	0.0%	0.0%	0.0%
tr|F1SCV1|F1SCV1	2	2	0	0	0	0	0	0	2	9	0	0	0	0	0	0	1.7%	4.7%	0.0%	0.0%	0.0%	0.0%	0.0%	0.0%
tr|I3LHZ9|I3LHZ9	10	0	1	0	0	0	0	0	11	0	1	0	0	0	0	0	11.2%	0.0%	0.9%	0.0%	0.0%	0.0%	0.0%	0.0%
tr|F1SL55|F1SL55	8	0	1	0	0	0	0	0	11	0	1	0	0	0	0	0	32.5%	0.0%	4.2%	0.0%	0.0%	0.0%	0.0%	0.0%
tr|F1SRE0|F1SRE0	4	2	1	0	0	0	0	0	5	6	2	0	0	0	0	0	8.6%	4.3%	2.3%	0.0%	0.0%	0.0%	0.0%	0.0%
tr|F1SMR6|F1SMR6	3	1	1	0	0	0	0	0	9	2	2	0	0	0	0	0	14.6%	3.4%	5.9%	0.0%	0.0%	0.0%	0.0%	0.0%
tr|I3LTG5|I3LTG5	3	1	1	0	0	0	0	0	9	2	2	0	0	0	0	0	14.2%	3.3%	5.8%	0.0%	0.0%	0.0%	0.0%	0.0%
tr|K7GL02|K7GL02	5	3	3	0	0	0	0	0	6	5	3	0	0	0	0	0	9.0%	3.7%	6.5%	0.0%	0.0%	0.0%	0.0%	0.0%
Q767L0	5	3	3	0	0	0	0	0	6	5	3	0	0	0	0	0	9.4%	3.8%	6.8%	0.0%	0.0%	0.0%	0.0%	0.0%
tr|F1SH24|F1SH24	2	1	1	0	0	0	0	0	2	9	5	0	0	0	0	0	6.1%	1.6%	3.0%	0.0%	0.0%	0.0%	0.0%	0.0%
tr|I3L6A9|I3L6A9	5	1	1	0	0	0	0	0	10	1	5	0	0	0	0	0	42.0%	5.7%	8.3%	0.0%	0.0%	0.0%	0.0%	0.0%
tr|F1S912|F1S912	4	3	2	0	0	0	0	0	6	5	7	0	0	0	0	0	14.7%	10.9%	3.4%	0.0%	0.0%	0.0%	0.0%	0.0%
tr|Q8HXL4|Q8HXL4	5	3	2	0	0	0	0	0	6	5	7	0	0	0	0	0	29.1%	14.3%	16.8%	0.0%	0.0%	0.0%	0.0%	0.0%
tr|I3LSQ7|I3LSQ7	5	0	2	0	0	0	0	0	11	0	9	0	0	0	0	0	14.7%	0.0%	5.1%	0.0%	0.0%	0.0%	0.0%	0.0%
tr|F1SLH0|F1SLH0	6	0	1	0	0	0	0	0	11	0	11	0	0	0	0	0	33.5%	0.0%	10.0%	0.0%	0.0%	0.0%	0.0%	0.0%
tr|I3LGP4|I3LGP4	7	3	4	0	0	0	0	0	7	4	11	0	0	0	0	0	8.0%	3.5%	6.9%	0.0%	0.0%	0.0%	0.0%	0.0%
tr|F1SB16|F1SB16	6	1	3	0	0	0	0	0	9	2	17	0	0	0	0	0	27.4%	3.1%	11.3%	0.0%	0.0%	0.0%	0.0%	0.0%
tr|F1S684|F1S684	4	3	6	0	0	0	0	0	6	5	18	0	0	0	0	0	5.5%	2.9%	8.9%	0.0%	0.0%	0.0%	0.0%	0.0%
tr|I3LQS6|I3LQS6	1	2	5	0	0	0	0	0	2	9	19	0	0	0	0	0	7.7%	18.8%	22.2%	0.0%	0.0%	0.0%	0.0%	0.0%
tr|F1RW89|F1RW89	5	0	4	0	0	0	0	0	11	0	25	0	0	0	0	0	13.0%	0.0%	11.4%	0.0%	0.0%	0.0%	0.0%	0.0%
tr|F1SPG4|F1SPG4	10	1	4	0	0	0	0	0	10	1	27	0	0	0	0	0	10.9%	0.5%	3.5%	0.0%	0.0%	0.0%	0.0%	0.0%
tr|F2Z5S7|F2Z5S7	4	0	2	0	0	0	0	0	11	0	30	0	0	0	0	0	40.1%	0.0%	13.0%	0.0%	0.0%	0.0%	0.0%	0.0%
tr|I3LMV4|I3LMV4	4	0	1	0	0	0	2	3	10	0	1	0	0	0	3	15	50.9%	0.0%	17.5%	0.0%	0.0%	0.0%	33.3%	33.3%
tr|I3L8K2|I3L8K2	2	1	5	0	0	0	2	2	7	3	18	0	0	0	7	33	9.5%	9.1%	20.2%	0.0%	0.0%	0.0%	13.7%	9.5%
tr|F1SDG9|F1SDG9	4	0	0	0	0	0	1	1	10	0	0	0	0	0	7	1	22.9%	0.0%	0.0%	0.0%	0.0%	0.0%	5.7%	6.9%
tr|F1S9A4|F1S9A4	8	0	4	0	0	0	0	2	10	0	14	0	0	0	0	17	34.8%	0.0%	15.0%	0.0%	0.0%	0.0%	0.0%	6.0%
P50390	2	2	0	0	0	0	0	2	8	2	0	0	0	0	0	6	15.3%	15.3%	0.0%	0.0%	0.0%	0.0%	0.0%	15.3%
tr|I3L9N5|I3L9N5	8	0	1	0	0	0	1	1	10	0	3	0	0	0	4	2	73.3%	0.0%	9.3%	0.0%	0.0%	0.0%	9.3%	9.9%
tr|K7GPN4|K7GPN4	4	1	0	0	0	0	0	2	9	1	0	0	0	0	0	4	24.4%	3.7%	0.0%	0.0%	0.0%	0.0%	0.0%	8.1%
tr|F1RNX2|F1RNX2	6	1	0	0	0	0	0	1	9	1	0	0	0	0	0	4	37.6%	8.8%	0.0%	0.0%	0.0%	0.0%	0.0%	8.8%
tr|I3L5D0|I3L5D0	6	1	0	0	0	0	0	1	9	1	0	0	0	0	0	4	35.3%	8.3%	0.0%	0.0%	0.0%	0.0%	0.0%	8.3%
tr|K7GKX8|K7GKX8	3	0	1	0	0	0	0	1	10	0	2	0	0	0	0	3	33.1%	0.0%	7.5%	0.0%	0.0%	0.0%	0.0%	4.7%
tr|F1SKM0|F1SKM0	6	1	9	0	0	0	1	4	8	2	146	0	0	0	7	30	22.7%	2.5%	29.6%	0.0%	0.0%	0.0%	6.0%	14.6%
tr|I3LUM8|I3LUM8	4	1	0	0	0	0	0	1	8	2	0	0	0	0	0	2	28.7%	14.8%	0.0%	0.0%	0.0%	0.0%	0.0%	9.0%
tr|I3L9W8|I3L9W8	7	0	0	0	0	0	0	1	10	0	0	0	0	0	0	2	27.4%	0.0%	0.0%	0.0%	0.0%	0.0%	0.0%	5.2%
tr|F2Z5F8|F2Z5F8	4	1	0	0	0	0	0	1	8	2	0	0	0	0	0	2	22.2%	10.4%	0.0%	0.0%	0.0%	0.0%	0.0%	10.4%

	Peptide Count								Spectral Count								Sequence Coverage							
	Whole Cell	Whole Cell	Whole Cell	Cilia Fraction	Cilia Fraction	Cilia Fraction	Cilia Fraction	Cilia Fraction	Whole Cell	Whole Cell	Whole Cell	Cilia Fraction	Cilia Fraction	Cilia Fraction	Cilia Fraction	Cilia Fraction	Whole Cell	Whole Cell	Whole Cell	Cilia Fraction	Cilia Fraction	Cilia Fraction	Cilia Fraction	Cilia Fraction
Sample#	1	2	6	4	3	5	7	8	1	2	6	4	3	5	7	8	1	2	6	4	3	5	7	8
**Accession**																								
tr|F1S4Y6|F1S4Y6	5	1	2	0	0	0	1	1	7	3	27	0	0	0	3	4	59.4%	16.7%	21.9%	0.0%	0.0%	0.0%	16.7%	22.9%
tr|F1RRB7|F1RRB7	7	0	2	0	0	0	1	0	10	0	8	0	0	0	3	0	19.0%	0.0%	7.5%	0.0%	0.0%	0.0%	4.7%	0.0%
tr|I3LPH5|I3LPH5	6	1	3	0	0	0	1	0	8	2	20	0	0	0	4	0	22.5%	2.2%	8.7%	0.0%	0.0%	0.0%	3.8%	0.0%
Q5PYH3	6	1	1	0	0	0	0	2	9	1	5	0	0	0	0	2	29.8%	5.6%	5.6%	0.0%	0.0%	0.0%	0.0%	19.7%
tr|F1S6G4|F1S6G4	4	2	0	0	0	0	0	1	7	3	0	0	0	0	0	1	25.3%	16.7%	0.0%	0.0%	0.0%	0.0%	0.0%	22.4%
tr|K7GSG6|K7GSG6	3	1	0	0	0	0	0	1	9	1	0	0	0	0	0	1	9.3%	1.5%	0.0%	0.0%	0.0%	0.0%	0.0%	2.7%
tr|F1SI69|F1SI69	3	1	0	0	0	0	0	1	9	1	0	0	0	0	0	1	7.7%	4.2%	0.0%	0.0%	0.0%	0.0%	0.0%	3.5%
tr|F1S5H9|F1S5H9	2	2	0	0	0	0	0	1	3	7	0	0	0	0	0	1	10.8%	3.5%	0.0%	0.0%	0.0%	0.0%	0.0%	5.2%
tr|F1RRR5|F1RRR5	3	1	0	0	0	0	0	1	9	1	0	0	0	0	0	1	5.8%	0.9%	0.0%	0.0%	0.0%	0.0%	0.0%	1.7%
tr|F1RGP8|F1RGP8	6	0	4	0	0	0	0	1	10	0	13	0	0	0	0	2	22.2%	0.0%	14.4%	0.0%	0.0%	0.0%	0.0%	5.3%
tr|F1SR61|F1SR61	2	2	1	0	0	0	0	1	2	8	2	0	0	0	0	1	6.5%	5.3%	4.2%	0.0%	0.0%	0.0%	0.0%	2.4%
Q8MIZ3	5	0	1	0	0	0	0	1	10	0	4	0	0	0	0	1	21.4%	0.0%	5.6%	0.0%	0.0%	0.0%	0.0%	5.9%
tr|I3LC35|I3LC35	4	2	1	0	0	0	0	1	5	5	4	0	0	0	0	1	16.2%	5.0%	4.0%	0.0%	0.0%	0.0%	0.0%	1.9%
tr|F1S4D5|F1S4D5	3	3	1	0	0	0	0	1	7	3	6	0	0	0	0	1	12.1%	12.8%	2.8%	0.0%	0.0%	0.0%	0.0%	2.1%
tr|F1S087|F1S087	6	0	9	0	0	0	0	2	10	0	48	0	0	0	0	2	19.9%	0.0%	21.4%	0.0%	0.0%	0.0%	0.0%	8.8%
tr|I3LB79|I3LB79	7	1	3	0	0	0	1	0	9	1	22	0	0	0	1	0	43.6%	5.5%	25.7%	0.0%	0.0%	0.0%	5.5%	0.0%
tr|F1SB09|F1SB09	7	1	3	0	0	0	1	0	9	1	22	0	0	0	1	0	44.2%	5.6%	26.0%	0.0%	0.0%	0.0%	5.6%	0.0%
tr|I3LQS2|I3LQS2	5	1	0	0	0	0	0	0	9	1	0	0	0	0	0	0	27.8%	7.1%	0.0%	0.0%	0.0%	0.0%	0.0%	0.0%
tr|K7GQ86|K7GQ86	1	4	0	0	0	0	0	0	1	9	0	0	0	0	0	0	3.8%	7.6%	0.0%	0.0%	0.0%	0.0%	0.0%	0.0%
tr|I3LQD7|I3LQD7	6	1	0	0	0	0	0	0	9	1	0	0	0	0	0	0	8.6%	2.0%	0.0%	0.0%	0.0%	0.0%	0.0%	0.0%
Q28832	0	1	0	0	0	0	0	0	0	10	0	0	0	0	0	0	0.0%	1.6%	0.0%	0.0%	0.0%	0.0%	0.0%	0.0%
tr|F1S940|F1S940	2	1	0	0	0	0	0	0	4	6	0	0	0	0	0	0	4.7%	1.3%	0.0%	0.0%	0.0%	0.0%	0.0%	0.0%
tr|F1RQT7|F1RQT7	3	0	0	0	0	0	0	0	10	0	0	0	0	0	0	0	8.1%	0.0%	0.0%	0.0%	0.0%	0.0%	0.0%	0.0%
tr|F1SFA7|F1SFA7	3	3	0	0	0	0	0	0	5	5	0	0	0	0	0	0	3.2%	2.3%	0.0%	0.0%	0.0%	0.0%	0.0%	0.0%
tr|F1S5G4|F1S5G4	7	1	0	0	0	0	0	0	9	1	0	0	0	0	0	0	23.3%	4.1%	0.0%	0.0%	0.0%	0.0%	0.0%	0.0%
Q29318	0	2	0	0	0	0	0	0	0	10	0	0	0	0	0	0	0.0%	13.4%	0.0%	0.0%	0.0%	0.0%	0.0%	0.0%
tr|F1S9P9|F1S9P9	2	2	0	0	0	0	0	0	3	7	0	0	0	0	0	0	5.6%	5.6%	0.0%	0.0%	0.0%	0.0%	0.0%	0.0%
tr|F1RIG2|F1RIG2	6	0	0	0	0	0	0	0	10	0	0	0	0	0	0	0	8.0%	0.0%	0.0%	0.0%	0.0%	0.0%	0.0%	0.0%
tr|I3LT48|I3LT48	7	1	0	0	0	0	0	0	9	1	0	0	0	0	0	0	50.9%	9.6%	0.0%	0.0%	0.0%	0.0%	0.0%	0.0%
tr|I3LKN9|I3LKN9	2	2	0	0	0	0	0	0	3	7	0	0	0	0	0	0	5.5%	5.5%	0.0%	0.0%	0.0%	0.0%	0.0%	0.0%
tr|K7GL74|K7GL74	3	0	0	0	0	0	0	0	10	0	0	0	0	0	0	0	70.5%	0.0%	0.0%	0.0%	0.0%	0.0%	0.0%	0.0%
tr|K7GQ13|K7GQ13	1	4	0	0	0	0	0	0	1	9	0	0	0	0	0	0	3.6%	7.2%	0.0%	0.0%	0.0%	0.0%	0.0%	0.0%
tr|I3LNS4|I3LNS4	4	1	0	0	0	0	0	0	7	3	0	0	0	0	0	0	8.4%	7.5%	0.0%	0.0%	0.0%	0.0%	0.0%	0.0%
tr|F1SJE8|F1SJE8	2	1	1	0	0	0	0	0	2	8	1	0	0	0	0	0	8.6%	3.5%	3.5%	0.0%	0.0%	0.0%	0.0%	0.0%
tr|F1RVX4|F1RVX4	7	0	1	0	0	0	0	0	10	0	2	0	0	0	0	0	34.3%	0.0%	5.4%	0.0%	0.0%	0.0%	0.0%	0.0%
tr|F1RJR2|F1RJR2	5	0	1	0	0	0	0	0	10	0	3	0	0	0	0	0	16.2%	0.0%	6.6%	0.0%	0.0%	0.0%	0.0%	0.0%
tr|I3L712|I3L712	6	2	2	0	0	0	0	0	6	4	3	0	0	0	0	0	9.6%	2.2%	4.0%	0.0%	0.0%	0.0%	0.0%	0.0%
P40125	2	1	1	0	0	0	0	0	3	7	4	0	0	0	0	0	8.6%	5.2%	4.7%	0.0%	0.0%	0.0%	0.0%	0.0%
tr|I3LVG4|I3LVG4	5	1	3	0	0	0	0	0	8	2	5	0	0	0	0	0	31.8%	5.1%	12.9%	0.0%	0.0%	0.0%	0.0%	0.0%
tr|I3LDD2|I3LDD2	6	0	2	0	0	0	0	0	10	0	5	0	0	0	0	0	26.2%	0.0%	6.0%	0.0%	0.0%	0.0%	0.0%	0.0%
tr|F1SP49|F1SP49	3	0	1	0	0	0	0	0	10	0	6	0	0	0	0	0	7.4%	0.0%	6.8%	0.0%	0.0%	0.0%	0.0%	0.0%
tr|F1SND4|F1SND4	4	1	1	0	0	0	0	0	9	1	7	0	0	0	0	0	23.5%	3.1%	6.2%	0.0%	0.0%	0.0%	0.0%	0.0%
tr|K7GRE2|K7GRE2	1	2	1	0	0	0	0	0	3	7	7	0	0	0	0	0	12.9%	22.8%	12.9%	0.0%	0.0%	0.0%	0.0%	0.0%
tr|I3LDX3|I3LDX3	2	1	4	0	0	0	0	0	9	1	15	0	0	0	0	0	20.0%	4.3%	11.3%	0.0%	0.0%	0.0%	0.0%	0.0%
tr|K7GMV2|K7GMV2	5	2	4	0	0	0	0	0	5	5	19	0	0	0	0	0	29.8%	8.1%	17.3%	0.0%	0.0%	0.0%	0.0%	0.0%
P55931	4	0	3	0	0	0	0	0	10	0	19	0	0	0	0	0	10.2%	0.0%	7.9%	0.0%	0.0%	0.0%	0.0%	0.0%
tr|F1S3W0|F1S3W0	3	0	3	0	0	0	0	0	10	0	254	0	0	0	0	0	48.4%	0.0%	48.4%	0.0%	0.0%	0.0%	0.0%	0.0%
tr|F1RY38|F1RY38	4	0	4	0	0	0	2	6	9	0	24	0	0	0	23	112	24.3%	0.0%	27.2%	0.0%	0.0%	0.0%	23.3%	28.2%
tr|K7GT57|K7GT57	3	1	0	0	0	0	1	3	7	2	0	0	0	0	21	10	41.5%	9.2%	0.0%	0.0%	0.0%	0.0%	7.7%	32.3%
tr|K7GMI5|K7GMI5	2	0	0	0	0	0	0	4	9	0	0	0	0	0	0	31	7.5%	0.0%	0.0%	0.0%	0.0%	0.0%	0.0%	7.5%
tr|F1RTG8|F1RTG8	2	0	0	0	0	0	0	4	9	0	0	0	0	0	0	31	7.7%	0.0%	0.0%	0.0%	0.0%	0.0%	0.0%	7.7%
tr|F1RGA9|F1RGA9	5	2	3	0	0	0	3	3	5	4	12	0	0	0	24	19	18.4%	5.5%	6.1%	0.0%	0.0%	0.0%	13.3%	14.0%
P24853	3	0	0	0	0	0	1	2	9	0	0	0	0	0	11	6	17.4%	0.0%	0.0%	0.0%	0.0%	0.0%	13.3%	20.6%
Q8MJ14	4	0	0	0	0	0	1	4	9	0	0	0	0	0	3	11	18.9%	0.0%	0.0%	0.0%	0.0%	0.0%	10.7%	26.7%
tr|I3LDF4|I3LDF4	8	0	3	0	0	0	3	5	9	0	6	0	0	0	7	11	32.4%	0.0%	4.9%	0.0%	0.0%	0.0%	18.7%	36.8%
tr|F1RGU1|F1RGU1	3	0	1	0	0	0	1	1	9	0	2	0	0	0	4	8	12.8%	0.0%	7.6%	0.0%	0.0%	0.0%	6.6%	6.6%
P20112	5	1	4	0	0	0	1	5	6	3	39	0	0	0	9	33	26.7%	3.3%	9.7%	0.0%	0.0%	0.0%	4.3%	20.3%
tr|F2Z5W0|F2Z5W0	4	0	2	0	0	0	0	2	9	0	11	0	0	0	0	15	35.9%	0.0%	31.1%	0.0%	0.0%	0.0%	0.0%	19.4%
tr|F1RKX9|F1RKX9	4	1	1	0	0	0	0	2	8	1	3	0	0	0	0	8	18.8%	4.3%	5.6%	0.0%	0.0%	0.0%	0.0%	7.9%
tr|F1SSS1|F1SSS1	5	1	1	0	0	0	0	2	6	3	1	0	0	0	0	4	20.6%	3.0%	4.8%	0.0%	0.0%	0.0%	0.0%	9.3%
tr|F1RN71|F1RN71	3	2	1	0	0	0	0	2	4	5	1	0	0	0	0	4	21.4%	17.7%	6.8%	0.0%	0.0%	0.0%	0.0%	15.1%
tr|I3LGJ4|I3LGJ4	3	2	4	0	0	0	0	2	7	2	17	0	0	0	0	9	13.6%	3.3%	20.9%	0.0%	0.0%	0.0%	0.0%	9.2%
tr|I3L852|I3L852	5	1	0	0	0	0	0	1	8	1	0	0	0	0	0	3	20.5%	7.3%	0.0%	0.0%	0.0%	0.0%	0.0%	7.3%
tr|F1RM51|F1RM51	6	1	0	0	0	0	0	1	8	1	0	0	0	0	0	3	32.9%	3.8%	0.0%	0.0%	0.0%	0.0%	0.0%	11.1%
tr|Q06AB2|Q06AB2	4	3	2	0	0	0	1	1	4	5	11	0	0	0	1	5	32.0%	23.0%	11.5%	0.0%	0.0%	0.0%	13.5%	13.5%
tr|I3LRX7|I3LRX7	6	0	1	0	0	0	0	1	9	0	8	0	0	0	0	5	17.2%	0.0%	4.3%	0.0%	0.0%	0.0%	0.0%	6.1%
tr|F1RNU6|F1RNU6	5	3	3	0	0	0	1	1	6	3	15	0	0	0	5	2	37.7%	22.5%	15.2%	0.0%	0.0%	0.0%	6.5%	6.5%
tr|F1RG66|F1RG66	3	2	6	0	0	0	0	2	7	2	24	0	0	0	0	9	13.6%	3.3%	26.1%	0.0%	0.0%	0.0%	0.0%	9.2%
tr|I3LF32|I3LF32	2	0	3	0	0	0	1	1	9	0	37	0	0	0	1	10	2.8%	0.0%	2.8%	0.0%	0.0%	0.0%	1.2%	2.8%
tr|F1RQS2|F1RQS2	2	0	3	0	0	0	1	1	9	0	37	0	0	0	1	10	7.1%	0.0%	7.1%	0.0%	0.0%	0.0%	3.2%	7.1%
tr|F1SD97|F1SD97	4	1	0	0	0	0	0	1	8	1	0	0	0	0	0	2	19.8%	2.4%	0.0%	0.0%	0.0%	0.0%	0.0%	5.9%
tr|I3LSJ9|I3LSJ9	4	2	0	0	0	0	1	0	5	4	0	0	0	0	2	0	12.9%	8.7%	0.0%	0.0%	0.0%	0.0%	4.9%	0.0%
tr|K7GML1|K7GML1	2	0	1	0	0	0	0	1	9	0	6	0	0	0	0	3	34.3%	0.0%	4.0%	0.0%	0.0%	0.0%	0.0%	6.9%
tr|Q3ZEL3|Q3ZEL3	4	0	3	0	0	0	1	3	9	0	18	0	0	0	1	4	14.6%	0.0%	10.6%	0.0%	0.0%	0.0%	4.8%	12.0%
P12675	4	0	3	0	0	0	1	3	9	0	18	0	0	0	1	4	14.2%	0.0%	10.2%	0.0%	0.0%	0.0%	4.6%	11.6%
tr|I3LL27|I3LL27	3	1	2	0	0	0	2	2	5	4	41	0	0	0	5	3	7.8%	2.7%	6.6%	0.0%	0.0%	0.0%	5.9%	6.6%
P19133	6	0	3	0	0	0	0	2	9	0	31	0	0	0	0	6	52.6%	0.0%	35.9%	0.0%	0.0%	0.0%	0.0%	20.5%
tr|F1RIP3|F1RIP3	6	0	4	0	0	0	0	2	9	0	39	0	0	0	0	6	23.4%	0.0%	25.1%	0.0%	0.0%	0.0%	0.0%	9.1%
tr|F1RMZ0|F1RMZ0	4	1	2	0	0	0	0	2	4	5	7	0	0	0	0	2	4.4%	0.9%	1.5%	0.0%	0.0%	0.0%	0.0%	2.3%
tr|I3LKN7|I3LKN7	4	1	5	0	0	0	1	1	8	1	19	0	0	0	2	1	21.0%	4.0%	16.1%	0.0%	0.0%	0.0%	3.5%	7.5%
tr|F1SEZ3|F1SEZ3	4	2	4	0	0	0	0	1	7	2	11	0	0	0	0	2	8.7%	4.1%	12.4%	0.0%	0.0%	0.0%	0.0%	4.6%

<table>
<tr><th></th><th colspan="8">Peptide Count</th><th colspan="8">Spectral Count</th><th colspan="8">Sequence Coverage</th></tr>
<tr><th></th><th>Whole Cell</th><th>Whole Cell</th><th>Whole Cell</th><th>Cilia Fraction</th><th>Cilia Fraction</th><th>Cilia Fraction</th><th>Cilia Fraction</th><th>Cilia Fraction</th><th>Whole Cell</th><th>Whole Cell</th><th>Whole Cell</th><th>Cilia Fraction</th><th>Cilia Fraction</th><th>Cilia Fraction</th><th>Cilia Fraction</th><th>Cilia Fraction</th><th>Whole Cell</th><th>Whole Cell</th><th>Whole Cell</th><th>Cilia Fraction</th><th>Cilia Fraction</th><th>Cilia Fraction</th><th>Cilia Fraction</th><th>Cilia Fraction</th></tr>
<tr><th>Sample#</th><th>1</th><th>2</th><th>6</th><th>4</th><th>3</th><th>5</th><th>7</th><th>8</th><th>1</th><th>2</th><th>6</th><th>4</th><th>3</th><th>5</th><th>7</th><th>8</th><th>1</th><th>2</th><th>6</th><th>4</th><th>3</th><th>5</th><th>7</th><th>8</th></tr>
<tr><td>Accession</td><td></td><td></td><td></td><td></td><td></td><td></td><td></td><td></td><td></td><td></td><td></td><td></td><td></td><td></td><td></td><td></td><td></td><td></td><td></td><td></td><td></td><td></td><td></td><td></td></tr>
<tr><td>tr|I3L7L5|I3L7L5</td><td>2</td><td>3</td><td>2</td><td>0</td><td>0</td><td>0</td><td>2</td><td>2</td><td>3</td><td>6</td><td>150</td><td>0</td><td>0</td><td>0</td><td>7</td><td>6</td><td>16.3%</td><td>25.0%</td><td>15.7%</td><td>0.0%</td><td>0.0%</td><td>0.0%</td><td>18.0%</td><td>15.1%</td></tr>
<tr><td>tr|F1SUG3|F1SUG3</td><td>4</td><td>1</td><td>2</td><td>0</td><td>0</td><td>0</td><td>1</td><td>0</td><td>5</td><td>4</td><td>21</td><td>0</td><td>0</td><td>0</td><td>2</td><td>0</td><td>21.0%</td><td>4.0%</td><td>5.2%</td><td>0.0%</td><td>0.0%</td><td>0.0%</td><td>5.2%</td><td>0.0%</td></tr>
<tr><td>tr|I3LT85|I3LT85</td><td>2</td><td>2</td><td>3</td><td>0</td><td>0</td><td>0</td><td>0</td><td>1</td><td>4</td><td>5</td><td>8</td><td>0</td><td>0</td><td>0</td><td>0</td><td>1</td><td>7.6%</td><td>5.5%</td><td>10.9%</td><td>0.0%</td><td>0.0%</td><td>0.0%</td><td>0.0%</td><td>7.1%</td></tr>
<tr><td>tr|I3LB64|I3LB64</td><td>3</td><td>2</td><td>4</td><td>0</td><td>0</td><td>0</td><td>0</td><td>1</td><td>7</td><td>2</td><td>20</td><td>0</td><td>0</td><td>0</td><td>0</td><td>1</td><td>9.7%</td><td>4.2%</td><td>11.7%</td><td>0.0%</td><td>0.0%</td><td>0.0%</td><td>0.0%</td><td>2.2%</td></tr>
<tr><td>tr|F2Z5A8|F2Z5A8</td><td>8</td><td>1</td><td>4</td><td>0</td><td>0</td><td>0</td><td>0</td><td>1</td><td>8</td><td>1</td><td>20</td><td>0</td><td>0</td><td>0</td><td>0</td><td>1</td><td>7.5%</td><td>0.7%</td><td>7.0%</td><td>0.0%</td><td>0.0%</td><td>0.0%</td><td>0.0%</td><td>2.5%</td></tr>
<tr><td>tr|F1S895|F1S895</td><td>3</td><td>2</td><td>4</td><td>0</td><td>0</td><td>0</td><td>0</td><td>1</td><td>7</td><td>2</td><td>20</td><td>0</td><td>0</td><td>0</td><td>0</td><td>1</td><td>9.7%</td><td>4.2%</td><td>11.7%</td><td>0.0%</td><td>0.0%</td><td>0.0%</td><td>0.0%</td><td>2.2%</td></tr>
<tr><td>tr|I3LMC6|I3LMC6</td><td>8</td><td>1</td><td>4</td><td>0</td><td>0</td><td>0</td><td>0</td><td>1</td><td>8</td><td>1</td><td>20</td><td>0</td><td>0</td><td>0</td><td>0</td><td>1</td><td>7.4%</td><td>0.7%</td><td>7.0%</td><td>0.0%</td><td>0.0%</td><td>0.0%</td><td>0.0%</td><td>2.5%</td></tr>
<tr><td>tr|K7GNZ6|K7GNZ6</td><td>3</td><td>2</td><td>2</td><td>0</td><td>0</td><td>0</td><td>0</td><td>2</td><td>3</td><td>6</td><td>206</td><td>0</td><td>0</td><td>0</td><td>0</td><td>7</td><td>27.5%</td><td>19.3%</td><td>17.4%</td><td>0.0%</td><td>0.0%</td><td>0.0%</td><td>0.0%</td><td>17.4%</td></tr>
<tr><td>tr|F1RL38|F1RL38</td><td>4</td><td>2</td><td>3</td><td>0</td><td>0</td><td>0</td><td>1</td><td>0</td><td>5</td><td>4</td><td>25</td><td>0</td><td>0</td><td>0</td><td>1</td><td>0</td><td>28.0%</td><td>19.5%</td><td>22.0%</td><td>0.0%</td><td>0.0%</td><td>0.0%</td><td>9.3%</td><td>0.0%</td></tr>
<tr><td>tr|E0YLM4|E0YLM4</td><td>2</td><td>3</td><td>7</td><td>0</td><td>0</td><td>0</td><td>1</td><td>0</td><td>3</td><td>6</td><td>28</td><td>0</td><td>0</td><td>0</td><td>1</td><td>0</td><td>14.0%</td><td>21.8%</td><td>30.7%</td><td>0.0%</td><td>0.0%</td><td>0.0%</td><td>7.8%</td><td>0.0%</td></tr>
<tr><td>tr|I3L759|I3L759</td><td>5</td><td>2</td><td>9</td><td>0</td><td>0</td><td>0</td><td>0</td><td>1</td><td>7</td><td>2</td><td>34</td><td>0</td><td>0</td><td>0</td><td>0</td><td>1</td><td>20.8%</td><td>5.0%</td><td>16.5%</td><td>0.0%</td><td>0.0%</td><td>0.0%</td><td>0.0%</td><td>5.9%</td></tr>
<tr><td>tr|K7GPB5|K7GPB5</td><td>3</td><td>0</td><td>0</td><td>0</td><td>0</td><td>0</td><td>0</td><td>0</td><td>9</td><td>0</td><td>0</td><td>0</td><td>0</td><td>0</td><td>0</td><td>0</td><td>14.0%</td><td>0.0%</td><td>0.0%</td><td>0.0%</td><td>0.0%</td><td>0.0%</td><td>0.0%</td><td>0.0%</td></tr>
<tr><td>tr|F1RWB0|F1RWB0</td><td>4</td><td>3</td><td>0</td><td>0</td><td>0</td><td>0</td><td>0</td><td>0</td><td>4</td><td>5</td><td>0</td><td>0</td><td>0</td><td>0</td><td>0</td><td>0</td><td>15.0%</td><td>7.4%</td><td>0.0%</td><td>0.0%</td><td>0.0%</td><td>0.0%</td><td>0.0%</td><td>0.0%</td></tr>
<tr><td>tr|F1SD69|F1SD69</td><td>3</td><td>0</td><td>0</td><td>0</td><td>0</td><td>0</td><td>0</td><td>0</td><td>9</td><td>0</td><td>0</td><td>0</td><td>0</td><td>0</td><td>0</td><td>0</td><td>11.9%</td><td>0.0%</td><td>0.0%</td><td>0.0%</td><td>0.0%</td><td>0.0%</td><td>0.0%</td><td>0.0%</td></tr>
<tr><td>tr|K7GMZ5|K7GMZ5</td><td>0</td><td>1</td><td>0</td><td>0</td><td>0</td><td>0</td><td>0</td><td>0</td><td>0</td><td>9</td><td>0</td><td>0</td><td>0</td><td>0</td><td>0</td><td>0</td><td>0.0%</td><td>5.7%</td><td>0.0%</td><td>0.0%</td><td>0.0%</td><td>0.0%</td><td>0.0%</td><td>0.0%</td></tr>
<tr><td>tr|I3LSP4|I3LSP4</td><td>6</td><td>0</td><td>0</td><td>0</td><td>0</td><td>0</td><td>0</td><td>0</td><td>9</td><td>0</td><td>0</td><td>0</td><td>0</td><td>0</td><td>0</td><td>0</td><td>7.8%</td><td>0.0%</td><td>0.0%</td><td>0.0%</td><td>0.0%</td><td>0.0%</td><td>0.0%</td><td>0.0%</td></tr>
<tr><td>tr|K7GNH2|K7GNH2</td><td>0</td><td>1</td><td>0</td><td>0</td><td>0</td><td>0</td><td>0</td><td>0</td><td>0</td><td>9</td><td>0</td><td>0</td><td>0</td><td>0</td><td>0</td><td>0</td><td>0.0%</td><td>5.8%</td><td>0.0%</td><td>0.0%</td><td>0.0%</td><td>0.0%</td><td>0.0%</td><td>0.0%</td></tr>
<tr><td>tr|F6Q2D2|F6Q2D2</td><td>4</td><td>1</td><td>0</td><td>0</td><td>0</td><td>0</td><td>0</td><td>0</td><td>7</td><td>2</td><td>0</td><td>0</td><td>0</td><td>0</td><td>0</td><td>0</td><td>10.3%</td><td>5.3%</td><td>0.0%</td><td>0.0%</td><td>0.0%</td><td>0.0%</td><td>0.0%</td><td>0.0%</td></tr>
<tr><td>A4Z6H0</td><td>1</td><td>2</td><td>0</td><td>0</td><td>0</td><td>0</td><td>0</td><td>0</td><td>1</td><td>8</td><td>0</td><td>0</td><td>0</td><td>0</td><td>0</td><td>0</td><td>3.3%</td><td>3.5%</td><td>0.0%</td><td>0.0%</td><td>0.0%</td><td>0.0%</td><td>0.0%</td><td>0.0%</td></tr>
<tr><td>tr|I3LAW0|I3LAW0</td><td>3</td><td>3</td><td>0</td><td>0</td><td>0</td><td>0</td><td>0</td><td>0</td><td>4</td><td>5</td><td>0</td><td>0</td><td>0</td><td>0</td><td>0</td><td>0</td><td>12.9%</td><td>11.4%</td><td>0.0%</td><td>0.0%</td><td>0.0%</td><td>0.0%</td><td>0.0%</td><td>0.0%</td></tr>
<tr><td>tr|F1RKB2|F1RKB2</td><td>3</td><td>2</td><td>0</td><td>0</td><td>0</td><td>0</td><td>0</td><td>0</td><td>7</td><td>2</td><td>0</td><td>0</td><td>0</td><td>0</td><td>0</td><td>0</td><td>9.2%</td><td>5.0%</td><td>0.0%</td><td>0.0%</td><td>0.0%</td><td>0.0%</td><td>0.0%</td><td>0.0%</td></tr>
<tr><td>tr|I3LU38|I3LU38</td><td>0</td><td>1</td><td>0</td><td>0</td><td>0</td><td>0</td><td>0</td><td>0</td><td>0</td><td>9</td><td>0</td><td>0</td><td>0</td><td>0</td><td>0</td><td>0</td><td>0.0%</td><td>0.5%</td><td>0.0%</td><td>0.0%</td><td>0.0%</td><td>0.0%</td><td>0.0%</td><td>0.0%</td></tr>
<tr><td>tr|I3LKM9|I3LKM9</td><td>3</td><td>0</td><td>0</td><td>0</td><td>0</td><td>0</td><td>0</td><td>0</td><td>9</td><td>0</td><td>0</td><td>0</td><td>0</td><td>0</td><td>0</td><td>0</td><td>10.9%</td><td>0.0%</td><td>0.0%</td><td>0.0%</td><td>0.0%</td><td>0.0%</td><td>0.0%</td><td>0.0%</td></tr>
<tr><td>tr|K7GRT1|K7GRT1</td><td>3</td><td>0</td><td>0</td><td>0</td><td>0</td><td>0</td><td>0</td><td>0</td><td>9</td><td>0</td><td>0</td><td>0</td><td>0</td><td>0</td><td>0</td><td>0</td><td>18.0%</td><td>0.0%</td><td>0.0%</td><td>0.0%</td><td>0.0%</td><td>0.0%</td><td>0.0%</td><td>0.0%</td></tr>
<tr><td>tr|I3L8C5|I3L8C5</td><td>0</td><td>1</td><td>0</td><td>0</td><td>0</td><td>0</td><td>0</td><td>0</td><td>0</td><td>9</td><td>0</td><td>0</td><td>0</td><td>0</td><td>0</td><td>0</td><td>0.0%</td><td>1.9%</td><td>0.0%</td><td>0.0%</td><td>0.0%</td><td>0.0%</td><td>0.0%</td><td>0.0%</td></tr>
<tr><td>tr|F1SDP2|F1SDP2</td><td>1</td><td>0</td><td>0</td><td>0</td><td>0</td><td>0</td><td>0</td><td>0</td><td>9</td><td>0</td><td>0</td><td>0</td><td>0</td><td>0</td><td>0</td><td>0</td><td>7.8%</td><td>0.0%</td><td>0.0%</td><td>0.0%</td><td>0.0%</td><td>0.0%</td><td>0.0%</td><td>0.0%</td></tr>
<tr><td>tr|F1S4Z5|F1S4Z5</td><td>0</td><td>2</td><td>0</td><td>0</td><td>0</td><td>0</td><td>0</td><td>0</td><td>0</td><td>9</td><td>0</td><td>0</td><td>0</td><td>0</td><td>0</td><td>0</td><td>0.0%</td><td>15.7%</td><td>0.0%</td><td>0.0%</td><td>0.0%</td><td>0.0%</td><td>0.0%</td><td>0.0%</td></tr>
<tr><td>tr|I3LEI8|I3LEI8</td><td>4</td><td>1</td><td>0</td><td>0</td><td>0</td><td>0</td><td>0</td><td>0</td><td>7</td><td>2</td><td>0</td><td>0</td><td>0</td><td>0</td><td>0</td><td>0</td><td>30.2%</td><td>4.5%</td><td>0.0%</td><td>0.0%</td><td>0.0%</td><td>0.0%</td><td>0.0%</td><td>0.0%</td></tr>
<tr><td>tr|F1RUA5|F1RUA5</td><td>2</td><td>2</td><td>0</td><td>0</td><td>0</td><td>0</td><td>0</td><td>0</td><td>5</td><td>4</td><td>0</td><td>0</td><td>0</td><td>0</td><td>0</td><td>0</td><td>19.7%</td><td>19.7%</td><td>0.0%</td><td>0.0%</td><td>0.0%</td><td>0.0%</td><td>0.0%</td><td>0.0%</td></tr>
<tr><td>tr|F1RWX6|F1RWX6</td><td>4</td><td>0</td><td>0</td><td>0</td><td>0</td><td>0</td><td>0</td><td>0</td><td>9</td><td>0</td><td>0</td><td>0</td><td>0</td><td>0</td><td>0</td><td>0</td><td>8.4%</td><td>0.0%</td><td>0.0%</td><td>0.0%</td><td>0.0%</td><td>0.0%</td><td>0.0%</td><td>0.0%</td></tr>
<tr><td>tr|K7GR43|K7GR43</td><td>2</td><td>2</td><td>0</td><td>0</td><td>0</td><td>0</td><td>0</td><td>0</td><td>5</td><td>4</td><td>0</td><td>0</td><td>0</td><td>0</td><td>0</td><td>0</td><td>18.6%</td><td>18.6%</td><td>0.0%</td><td>0.0%</td><td>0.0%</td><td>0.0%</td><td>0.0%</td><td>0.0%</td></tr>
<tr><td>tr|F1SR64|F1SR64</td><td>3</td><td>2</td><td>0</td><td>0</td><td>0</td><td>0</td><td>0</td><td>0</td><td>3</td><td>6</td><td>0</td><td>0</td><td>0</td><td>0</td><td>0</td><td>0</td><td>11.7%</td><td>8.1%</td><td>0.0%</td><td>0.0%</td><td>0.0%</td><td>0.0%</td><td>0.0%</td><td>0.0%</td></tr>
<tr><td>tr|F1SLS9|F1SLS9</td><td>5</td><td>2</td><td>0</td><td>0</td><td>0</td><td>0</td><td>0</td><td>0</td><td>5</td><td>4</td><td>0</td><td>0</td><td>0</td><td>0</td><td>0</td><td>0</td><td>6.9%</td><td>2.6%</td><td>0.0%</td><td>0.0%</td><td>0.0%</td><td>0.0%</td><td>0.0%</td><td>0.0%</td></tr>
<tr><td>tr|F1SKI2|F1SKI2</td><td>2</td><td>2</td><td>0</td><td>0</td><td>0</td><td>0</td><td>0</td><td>0</td><td>2</td><td>7</td><td>0</td><td>0</td><td>0</td><td>0</td><td>0</td><td>0</td><td>2.1%</td><td>1.3%</td><td>0.0%</td><td>0.0%</td><td>0.0%</td><td>0.0%</td><td>0.0%</td><td>0.0%</td></tr>
<tr><td>tr|F1RRA0|F1RRA0</td><td>6</td><td>0</td><td>0</td><td>0</td><td>0</td><td>0</td><td>0</td><td>0</td><td>9</td><td>0</td><td>0</td><td>0</td><td>0</td><td>0</td><td>0</td><td>0</td><td>7.6%</td><td>0.0%</td><td>0.0%</td><td>0.0%</td><td>0.0%</td><td>0.0%</td><td>0.0%</td><td>0.0%</td></tr>
<tr><td>tr|F1RTY7|F1RTY7</td><td>6</td><td>1</td><td>0</td><td>0</td><td>0</td><td>0</td><td>0</td><td>0</td><td>7</td><td>2</td><td>0</td><td>0</td><td>0</td><td>0</td><td>0</td><td>0</td><td>17.9%</td><td>3.7%</td><td>0.0%</td><td>0.0%</td><td>0.0%</td><td>0.0%</td><td>0.0%</td><td>0.0%</td></tr>
<tr><td>tr|F1RG44|F1RG44</td><td>6</td><td>1</td><td>0</td><td>0</td><td>0</td><td>0</td><td>0</td><td>0</td><td>8</td><td>1</td><td>0</td><td>0</td><td>0</td><td>0</td><td>0</td><td>0</td><td>14.4%</td><td>1.8%</td><td>0.0%</td><td>0.0%</td><td>0.0%</td><td>0.0%</td><td>0.0%</td><td>0.0%</td></tr>
<tr><td>tr|I3LEI5|I3LEI5</td><td>3</td><td>0</td><td>0</td><td>0</td><td>0</td><td>0</td><td>0</td><td>0</td><td>9</td><td>0</td><td>0</td><td>0</td><td>0</td><td>0</td><td>0</td><td>0</td><td>6.5%</td><td>0.0%</td><td>0.0%</td><td>0.0%</td><td>0.0%</td><td>0.0%</td><td>0.0%</td><td>0.0%</td></tr>
<tr><td>tr|I3LUG7|I3LUG7</td><td>8</td><td>0</td><td>0</td><td>0</td><td>0</td><td>0</td><td>0</td><td>0</td><td>9</td><td>0</td><td>0</td><td>0</td><td>0</td><td>0</td><td>0</td><td>0</td><td>10.7%</td><td>0.0%</td><td>0.0%</td><td>0.0%</td><td>0.0%</td><td>0.0%</td><td>0.0%</td><td>0.0%</td></tr>
<tr><td>tr|F1SNS1|F1SNS1</td><td>7</td><td>2</td><td>0</td><td>0</td><td>0</td><td>0</td><td>0</td><td>0</td><td>7</td><td>2</td><td>0</td><td>0</td><td>0</td><td>0</td><td>0</td><td>0</td><td>16.4%</td><td>4.1%</td><td>0.0%</td><td>0.0%</td><td>0.0%</td><td>0.0%</td><td>0.0%</td><td>0.0%</td></tr>
<tr><td>tr|F1S279|F1S279</td><td>2</td><td>1</td><td>0</td><td>0</td><td>0</td><td>0</td><td>0</td><td>0</td><td>4</td><td>5</td><td>0</td><td>0</td><td>0</td><td>0</td><td>0</td><td>0</td><td>6.4%</td><td>2.8%</td><td>0.0%</td><td>0.0%</td><td>0.0%</td><td>0.0%</td><td>0.0%</td><td>0.0%</td></tr>
<tr><td>tr|K7GRW0|K7GRW0</td><td>0</td><td>1</td><td>0</td><td>0</td><td>0</td><td>0</td><td>0</td><td>0</td><td>0</td><td>9</td><td>0</td><td>0</td><td>0</td><td>0</td><td>0</td><td>0</td><td>0.0%</td><td>5.9%</td><td>0.0%</td><td>0.0%</td><td>0.0%</td><td>0.0%</td><td>0.0%</td><td>0.0%</td></tr>
<tr><td>tr|F1RT23|F1RT23</td><td>0</td><td>1</td><td>0</td><td>0</td><td>0</td><td>0</td><td>0</td><td>0</td><td>0</td><td>9</td><td>0</td><td>0</td><td>0</td><td>0</td><td>0</td><td>0</td><td>0.0%</td><td>1.8%</td><td>0.0%</td><td>0.0%</td><td>0.0%</td><td>0.0%</td><td>0.0%</td><td>0.0%</td></tr>
<tr><td>tr|F1S4S5|F1S4S5</td><td>0</td><td>1</td><td>0</td><td>0</td><td>0</td><td>0</td><td>0</td><td>0</td><td>0</td><td>9</td><td>0</td><td>0</td><td>0</td><td>0</td><td>0</td><td>0</td><td>0.0%</td><td>1.9%</td><td>0.0%</td><td>0.0%</td><td>0.0%</td><td>0.0%</td><td>0.0%</td><td>0.0%</td></tr>
<tr><td>tr|F1RIU1|F1RIU1</td><td>1</td><td>1</td><td>0</td><td>0</td><td>0</td><td>0</td><td>0</td><td>0</td><td>1</td><td>8</td><td>0</td><td>0</td><td>0</td><td>0</td><td>0</td><td>0</td><td>5.3%</td><td>5.3%</td><td>0.0%</td><td>0.0%</td><td>0.0%</td><td>0.0%</td><td>0.0%</td><td>0.0%</td></tr>
<tr><td>tr|F1S6T0|F1S6T0</td><td>0</td><td>1</td><td>0</td><td>0</td><td>0</td><td>0</td><td>0</td><td>0</td><td>0</td><td>9</td><td>0</td><td>0</td><td>0</td><td>0</td><td>0</td><td>0</td><td>0.0%</td><td>1.3%</td><td>0.0%</td><td>0.0%</td><td>0.0%</td><td>0.0%</td><td>0.0%</td><td>0.0%</td></tr>
<tr><td>tr|F1S390|F1S390</td><td>1</td><td>2</td><td>0</td><td>0</td><td>0</td><td>0</td><td>0</td><td>0</td><td>3</td><td>6</td><td>0</td><td>0</td><td>0</td><td>0</td><td>0</td><td>0</td><td>1.7%</td><td>4.2%</td><td>0.0%</td><td>0.0%</td><td>0.0%</td><td>0.0%</td><td>0.0%</td><td>0.0%</td></tr>
<tr><td>tr|I3LTC4|I3LTC4</td><td>0</td><td>1</td><td>0</td><td>0</td><td>0</td><td>0</td><td>0</td><td>0</td><td>0</td><td>9</td><td>0</td><td>0</td><td>0</td><td>0</td><td>0</td><td>0</td><td>0.0%</td><td>4.8%</td><td>0.0%</td><td>0.0%</td><td>0.0%</td><td>0.0%</td><td>0.0%</td><td>0.0%</td></tr>
<tr><td>tr|I3LG16|I3LG16</td><td>3</td><td>2</td><td>1</td><td>0</td><td>0</td><td>0</td><td>0</td><td>0</td><td>4</td><td>5</td><td>1</td><td>0</td><td>0</td><td>0</td><td>0</td><td>0</td><td>10.4%</td><td>4.8%</td><td>2.2%</td><td>0.0%</td><td>0.0%</td><td>0.0%</td><td>0.0%</td><td>0.0%</td></tr>
<tr><td>tr|F1RW03|F1RW03</td><td>4</td><td>1</td><td>1</td><td>0</td><td>0</td><td>0</td><td>0</td><td>0</td><td>4</td><td>5</td><td>1</td><td>0</td><td>0</td><td>0</td><td>0</td><td>0</td><td>9.7%</td><td>1.8%</td><td>4.7%</td><td>0.0%</td><td>0.0%</td><td>0.0%</td><td>0.0%</td><td>0.0%</td></tr>
<tr><td>tr|F1S5H7|F1S5H7</td><td>4</td><td>2</td><td>1</td><td>0</td><td>0</td><td>0</td><td>0</td><td>0</td><td>5</td><td>4</td><td>1</td><td>0</td><td>0</td><td>0</td><td>0</td><td>0</td><td>10.5%</td><td>3.6%</td><td>2.1%</td><td>0.0%</td><td>0.0%</td><td>0.0%</td><td>0.0%</td><td>0.0%</td></tr>
<tr><td>tr|I3L8B8|I3L8B8</td><td>3</td><td>2</td><td>1</td><td>0</td><td>0</td><td>0</td><td>0</td><td>0</td><td>4</td><td>5</td><td>1</td><td>0</td><td>0</td><td>0</td><td>0</td><td>0</td><td>11.5%</td><td>5.3%</td><td>2.5%</td><td>0.0%</td><td>0.0%</td><td>0.0%</td><td>0.0%</td><td>0.0%</td></tr>
<tr><td>tr|D2KPI8|D2KPI8</td><td>5</td><td>0</td><td>1</td><td>0</td><td>0</td><td>0</td><td>0</td><td>0</td><td>9</td><td>0</td><td>1</td><td>0</td><td>0</td><td>0</td><td>0</td><td>0</td><td>17.1%</td><td>0.0%</td><td>3.7%</td><td>0.0%</td><td>0.0%</td><td>0.0%</td><td>0.0%</td><td>0.0%</td></tr>
<tr><td>tr|I3LGP6|I3LGP6</td><td>2</td><td>2</td><td>1</td><td>0</td><td>0</td><td>0</td><td>0</td><td>0</td><td>5</td><td>4</td><td>1</td><td>0</td><td>0</td><td>0</td><td>0</td><td>0</td><td>7.2%</td><td>3.5%</td><td>3.5%</td><td>0.0%</td><td>0.0%</td><td>0.0%</td><td>0.0%</td><td>0.0%</td></tr>
<tr><td>tr|F1SKL8|F1SKL8</td><td>4</td><td>1</td><td>1</td><td>0</td><td>0</td><td>0</td><td>0</td><td>0</td><td>7</td><td>2</td><td>1</td><td>0</td><td>0</td><td>0</td><td>0</td><td>0</td><td>17.9%</td><td>3.3%</td><td>2.4%</td><td>0.0%</td><td>0.0%</td><td>0.0%</td><td>0.0%</td><td>0.0%</td></tr>
<tr><td>tr|K7GMX6|K7GMX6</td><td>2</td><td>2</td><td>1</td><td>0</td><td>0</td><td>0</td><td>0</td><td>0</td><td>3</td><td>6</td><td>1</td><td>0</td><td>0</td><td>0</td><td>0</td><td>0</td><td>7.6%</td><td>4.0%</td><td>2.2%</td><td>0.0%</td><td>0.0%</td><td>0.0%</td><td>0.0%</td><td>0.0%</td></tr>
<tr><td>tr|I3LUL6|I3LUL6</td><td>3</td><td>0</td><td>1</td><td>0</td><td>0</td><td>0</td><td>0</td><td>0</td><td>9</td><td>0</td><td>2</td><td>0</td><td>0</td><td>0</td><td>0</td><td>0</td><td>21.8%</td><td>0.0%</td><td>9.0%</td><td>0.0%</td><td>0.0%</td><td>0.0%</td><td>0.0%</td><td>0.0%</td></tr>
<tr><td>tr|F1SDY8|F1SDY8</td><td>2</td><td>2</td><td>1</td><td>0</td><td>0</td><td>0</td><td>0</td><td>0</td><td>2</td><td>7</td><td>2</td><td>0</td><td>0</td><td>0</td><td>0</td><td>0</td><td>3.8%</td><td>3.3%</td><td>2.3%</td><td>0.0%</td><td>0.0%</td><td>0.0%</td><td>0.0%</td><td>0.0%</td></tr>
<tr><td>tr|C5I4T6|C5I4T6</td><td>6</td><td>1</td><td>2</td><td>0</td><td>0</td><td>0</td><td>0</td><td>0</td><td>8</td><td>1</td><td>2</td><td>0</td><td>0</td><td>0</td><td>0</td><td>0</td><td>18.6%</td><td>1.4%</td><td>5.6%</td><td>0.0%</td><td>0.0%</td><td>0.0%</td><td>0.0%</td><td>0.0%</td></tr>
<tr><td>tr|F1RT21|F1RT21</td><td>3</td><td>1</td><td>1</td><td>0</td><td>0</td><td>0</td><td>0</td><td>0</td><td>6</td><td>3</td><td>3</td><td>0</td><td>0</td><td>0</td><td>0</td><td>0</td><td>11.3%</td><td>9.4%</td><td>7.5%</td><td>0.0%</td><td>0.0%</td><td>0.0%</td><td>0.0%</td><td>0.0%</td></tr>
<tr><td>tr|F1STS4|F1STS4</td><td>6</td><td>0</td><td>1</td><td>0</td><td>0</td><td>0</td><td>0</td><td>0</td><td>9</td><td>0</td><td>3</td><td>0</td><td>0</td><td>0</td><td>0</td><td>0</td><td>24.6%</td><td>0.0%</td><td>3.3%</td><td>0.0%</td><td>0.0%</td><td>0.0%</td><td>0.0%</td><td>0.0%</td></tr>
<tr><td>tr|I3LUD5|I3LUD5</td><td>2</td><td>0</td><td>1</td><td>0</td><td>0</td><td>0</td><td>0</td><td>0</td><td>9</td><td>0</td><td>3</td><td>0</td><td>0</td><td>0</td><td>0</td><td>0</td><td>17.8%</td><td>0.0%</td><td>9.8%</td><td>0.0%</td><td>0.0%</td><td>0.0%</td><td>0.0%</td><td>0.0%</td></tr>
<tr><td>tr|F1RKJ9|F1RKJ9</td><td>6</td><td>0</td><td>1</td><td>0</td><td>0</td><td>0</td><td>0</td><td>0</td><td>9</td><td>0</td><td>5</td><td>0</td><td>0</td><td>0</td><td>0</td><td>0</td><td>21.5%</td><td>0.0%</td><td>4.7%</td><td>0.0%</td><td>0.0%</td><td>0.0%</td><td>0.0%</td><td>0.0%</td></tr>
<tr><td>tr|F1SP98|F1SP98</td><td>5</td><td>0</td><td>1</td><td>0</td><td>0</td><td>0</td><td>0</td><td>0</td><td>9</td><td>0</td><td>7</td><td>0</td><td>0</td><td>0</td><td>0</td><td>0</td><td>15.9%</td><td>0.0%</td><td>3.9%</td><td>0.0%</td><td>0.0%</td><td>0.0%</td><td>0.0%</td><td>0.0%</td></tr>
<tr><td>tr|I3LLD7|I3LLD7</td><td>3</td><td>1</td><td>1</td><td>0</td><td>0</td><td>0</td><td>0</td><td>0</td><td>8</td><td>1</td><td>7</td><td>0</td><td>0</td><td>0</td><td>0</td><td>0</td><td>28.3%</td><td>9.0%</td><td>11.7%</td><td>0.0%</td><td>0.0%</td><td>0.0%</td><td>0.0%</td><td>0.0%</td></tr>
<tr><td>tr|K7GNL4|K7GNL4</td><td>4</td><td>2</td><td>2</td><td>0</td><td>0</td><td>0</td><td>0</td><td>0</td><td>6</td><td>3</td><td>8</td><td>0</td><td>0</td><td>0</td><td>0</td><td>0</td><td>16.1%</td><td>12.4%</td><td>7.0%</td><td>0.0%</td><td>0.0%</td><td>0.0%</td><td>0.0%</td><td>0.0%</td></tr>
<tr><td>tr|I3L8X0|I3L8X0</td><td>4</td><td>1</td><td>1</td><td>0</td><td>0</td><td>0</td><td>0</td><td>0</td><td>6</td><td>3</td><td>8</td><td>0</td><td>0</td><td>0</td><td>0</td><td>0</td><td>19.9%</td><td>2.9%</td><td>6.1%</td><td>0.0%</td><td>0.0%</td><td>0.0%</td><td>0.0%</td><td>0.0%</td></tr>
<tr><td>tr|K7GL17|K7GL17</td><td>2</td><td>2</td><td>2</td><td>0</td><td>0</td><td>0</td><td>0</td><td>0</td><td>7</td><td>2</td><td>9</td><td>0</td><td>0</td><td>0</td><td>0</td><td>0</td><td>37.1%</td><td>37.1%</td><td>65.7%</td><td>0.0%</td><td>0.0%</td><td>0.0%</td><td>0.0%</td><td>0.0%</td></tr>
<tr><td>tr|I3LIY7|I3LIY7</td><td>2</td><td>2</td><td>2</td><td>0</td><td>0</td><td>0</td><td>0</td><td>0</td><td>7</td><td>2</td><td>9</td><td>0</td><td>0</td><td>0</td><td>0</td><td>0</td><td>32.5%</td><td>32.5%</td><td>57.5%</td><td>0.0%</td><td>0.0%</td><td>0.0%</td><td>0.0%</td><td>0.0%</td></tr>
<tr><td>tr|F1SM67|F1SM67</td><td>5</td><td>1</td><td>1</td><td>0</td><td>0</td><td>0</td><td>0</td><td>0</td><td>8</td><td>1</td><td>12</td><td>0</td><td>0</td><td>0</td><td>0</td><td>0</td><td>11.2%</td><td>1.3%</td><td>3.7%</td><td>0.0%</td><td>0.0%</td><td>0.0%</td><td>0.0%</td><td>0.0%</td></tr>
<tr><td>tr|F1SQ11|F1SQ11</td><td>7</td><td>1</td><td>7</td><td>0</td><td>0</td><td>0</td><td>0</td><td>0</td><td>7</td><td>2</td><td>14</td><td>0</td><td>0</td><td>0</td><td>0</td><td>0</td><td>8.9%</td><td>1.3%</td><td>6.3%</td><td>0.0%</td><td>0.0%</td><td>0.0%</td><td>0.0%</td><td>0.0%</td></tr>
<tr><td>tr|I3LHX5|I3LHX5</td><td>4</td><td>1</td><td>1</td><td>0</td><td>0</td><td>0</td><td>0</td><td>0</td><td>5</td><td>4</td><td>14</td><td>0</td><td>0</td><td>0</td><td>0</td><td>0</td><td>18.4%</td><td>9.4%</td><td>6.1%</td><td>0.0%</td><td>0.0%</td><td>0.0%</td><td>0.0%</td><td>0.0%</td></tr>
<tr><td>tr|F1SHP6|F1SHP6</td><td>4</td><td>1</td><td>1</td><td>0</td><td>0</td><td>0</td><td>0</td><td>0</td><td>5</td><td>4</td><td>14</td><td>0</td><td>0</td><td>0</td><td>0</td><td>0</td><td>13.4%</td><td>6.8%</td><td>4.5%</td><td>0.0%</td><td>0.0%</td><td>0.0%</td><td>0.0%</td><td>0.0%</td></tr>
<tr><td>tr|F1S9I9|F1S9I9</td><td>3</td><td>2</td><td>3</td><td>0</td><td>0</td><td>0</td><td>0</td><td>0</td><td>6</td><td>3</td><td>15</td><td>0</td><td>0</td><td>0</td><td>0</td><td>0</td><td>14.0%</td><td>8.3%</td><td>11.6%</td><td>0.0%</td><td>0.0%</td><td>0.0%</td><td>0.0%</td><td>0.0%</td></tr>
<tr><td>tr|K7GQX0|K7GQX0</td><td>2</td><td>3</td><td>3</td><td>0</td><td>0</td><td>0</td><td>0</td><td>0</td><td>3</td><td>6</td><td>18</td><td>0</td><td>0</td><td>0</td><td>0</td><td>0</td><td>17.1%</td><td>10.3%</td><td>13.2%</td><td>0.0%</td><td>0.0%</td><td>0.0%</td><td>0.0%</td><td>0.0%</td></tr>
<tr><td>Q52I78</td><td>6</td><td>0</td><td>5</td><td>0</td><td>0</td><td>0</td><td>0</td><td>0</td><td>9</td><td>0</td><td>19</td><td>0</td><td>0</td><td>0</td><td>0</td><td>0</td><td>28.7%</td><td>0.0%</td><td>11.4%</td><td>0.0%</td><td>0.0%</td><td>0.0%</td><td>0.0%</td><td>0.0%</td></tr>
<tr><td>A1XQT2</td><td>1</td><td>1</td><td>5</td><td>0</td><td>0</td><td>0</td><td>0</td><td>0</td><td>1</td><td>8</td><td>41</td><td>0</td><td>0</td><td>0</td><td>0</td><td>0</td><td>9.2%</td><td>11.8%</td><td>26.3%</td><td>0.0%</td><td>0.0%</td><td>0.0%</td><td>0.0%</td><td>0.0%</td></tr>
<tr><td>tr|F1S0P0|F1S0P0</td><td>1</td><td>1</td><td>5</td><td>0</td><td>0</td><td>0</td><td>0</td><td>0</td><td>1</td><td>8</td><td>41</td><td>0</td><td>0</td><td>0</td><td>0</td><td>0</td><td>9.3%</td><td>12.0%</td><td>26.7%</td><td>0.0%</td><td>0.0%</td><td>0.0%</td><td>0.0%</td><td>0.0%</td></tr>
<tr><td>Q3YLA6</td><td>4</td><td>0</td><td>6</td><td>0</td><td>0</td><td>0</td><td>0</td><td>0</td><td>9</td><td>0</td><td>45</td><td>0</td><td>0</td><td>0</td><td>0</td><td>0</td><td>14.1%</td><td>0.0%</td><td>24.6%</td><td>0.0%</td><td>0.0%</td><td>0.0%</td><td>0.0%</td><td>0.0%</td></tr>
<tr><td>tr|I3LER5|I3LER5</td><td>4</td><td>1</td><td>6</td><td>0</td><td>0</td><td>0</td><td>0</td><td>0</td><td>8</td><td>1</td><td>49</td><td>0</td><td>0</td><td>0</td><td>0</td><td>0</td><td>20.1%</td><td>5.9%</td><td>33.1%</td><td>0.0%</td><td>0.0%</td><td>0.0%</td><td>0.0%</td><td>0.0%</td></tr>
<tr><td>tr|F1RLE5|F1RLE5</td><td>2</td><td>4</td><td>1</td><td>0</td><td>0</td><td>0</td><td>3</td><td>2</td><td>3</td><td>5</td><td>2</td><td>0</td><td>0</td><td>0</td><td>41</td><td>4</td><td>5.8%</td><td>20.0%</td><td>4.4%</td><td>0.0%</td><td>0.0%</td><td>0.0%</td><td>23.1%</td><td>17.3%</td></tr>
</table>

	Peptide Count								Spectral Count								Sequence Coverage							
	Whole Cell	Whole Cell	Whole Cell	Cilia Fraction	Cilia Fraction	Cilia Fraction	Cilia Fraction	Cilia Fraction	Whole Cell	Whole Cell	Whole Cell	Cilia Fraction	Cilia Fraction	Cilia Fraction	Cilia Fraction	Cilia Fraction	Whole Cell	Whole Cell	Whole Cell	Cilia Fraction	Cilia Fraction	Cilia Fraction	Cilia Fraction	Cilia Fraction
Sample#	1	2	6	4	3	5	7	8	1	2	6	4	3	5	7	8	1	2	6	4	3	5	7	8
**Accession**																								
tr|K7GLS2|K7GLS2	1	1	0	0	0	0	1	0	4	4	0	0	0	0	24	0	15.7%	9.0%	0.0%	0.0%	0.0%	0.0%	9.0%	0.0%
tr|I3L920|I3L920	4	2	3	0	0	0	3	3	4	4	12	0	0	0	24	19	26.2%	8.8%	9.8%	0.0%	0.0%	0.0%	21.3%	22.6%
tr|F1RM62|F1RM62	4	1	1	0	0	0	3	2	7	1	6	0	0	0	13	14	36.8%	8.6%	6.1%	0.0%	0.0%	0.0%	20.9%	12.9%
tr|F1SQI6|F1SQI6	3	1	1	0	0	0	1	2	3	5	2	0	0	0	1	17	5.5%	1.1%	3.1%	0.0%	0.0%	0.0%	2.6%	5.3%
tr|F1S733|F1S733	7	0	2	0	0	0	0	8	8	0	5	0	0	0	0	21	15.0%	0.0%	6.5%	0.0%	0.0%	0.0%	0.0%	14.2%
tr|K7GRX5|K7GRX5	2	1	0	0	0	0	0	2	7	1	0	0	0	0	0	9	19.4%	19.4%	0.0%	0.0%	0.0%	0.0%	0.0%	19.4%
tr|K7GQD9|K7GQD9	2	1	0	0	0	0	0	2	7	1	0	0	0	0	0	9	16.9%	16.9%	0.0%	0.0%	0.0%	0.0%	0.0%	16.9%
tr|K7GMA5|K7GMA5	2	1	0	0	0	0	0	2	7	1	0	0	0	0	0	9	17.4%	17.4%	0.0%	0.0%	0.0%	0.0%	0.0%	17.4%
tr|F1RLJ4|F1RLJ4	4	1	1	0	0	0	1	2	6	2	9	0	0	0	8	11	18.7%	5.8%	3.7%	0.0%	0.0%	0.0%	3.7%	15.9%
tr|F1S8P9|F1S8P9	2	0	3	0	0	0	0	6	8	0	11	0	0	0	0	21	7.0%	0.0%	7.3%	0.0%	0.0%	0.0%	0.0%	16.8%
tr|F1RQ14|F1RQ14	6	0	3	0	0	0	1	3	8	0	15	0	0	0	9	16	29.6%	0.0%	11.7%	0.0%	0.0%	0.0%	5.3%	12.6%
tr|K7GMZ2|K7GMZ2	3	1	0	0	0	0	0	2	7	1	0	0	0	0	0	8	27.3%	27.3%	0.0%	0.0%	0.0%	0.0%	0.0%	27.3%
tr|I3LDM5|I3LDM5	5	0	1	0	0	0	1	1	8	0	2	0	0	0	5	2	9.9%	0.0%	2.4%	0.0%	0.0%	0.0%	2.4%	2.7%
tr|I3LC63|I3LC63	5	0	1	0	0	0	1	1	8	0	2	0	0	0	5	2	10.0%	0.0%	2.5%	0.0%	0.0%	0.0%	2.5%	2.7%
tr|I3LR43|I3LR43	4	0	1	0	0	0	1	2	8	0	2	0	0	0	1	6	26.5%	0.0%	11.4%	0.0%	0.0%	0.0%	11.4%	9.6%
P34934	3	0	2	0	0	0	1	1	8	0	20	0	0	0	16	3	5.5%	0.0%	5.5%	0.0%	0.0%	0.0%	3.2%	3.2%
tr|F1SE87|F1SE87	3	0	1	0	0	0	2	2	8	0	16	0	0	0	7	9	26.8%	0.0%	8.9%	0.0%	0.0%	0.0%	15.1%	15.1%
Q56P28	4	0	1	0	0	0	1	0	8	0	14	0	0	0	14	0	10.6%	0.0%	10.1%	0.0%	0.0%	0.0%	10.1%	0.0%
tr|A5D9J0|A5D9J0	3	0	0	0	0	0	0	2	8	0	0	0	0	0	0	5	54.0%	0.0%	0.0%	0.0%	0.0%	0.0%	0.0%	44.0%
tr|I3LAI3|I3LAI3	3	1	1	0	0	0	1	0	4	4	2	0	0	0	5	0	13.6%	11.4%	11.4%	0.0%	0.0%	0.0%	13.6%	0.0%
tr|F1SIU8|F1SIU8	4	1	0	0	0	0	0	1	7	1	0	0	0	0	0	3	8.9%	3.6%	0.0%	0.0%	0.0%	0.0%	0.0%	3.1%
tr|F1SJH8|F1SJH8	4	1	0	0	0	0	0	1	7	1	0	0	0	0	0	3	11.9%	4.8%	0.0%	0.0%	0.0%	0.0%	0.0%	4.1%
tr|K7GPB8|K7GPB8	2	2	1	0	0	0	1	0	4	4	1	0	0	0	3	0	6.6%	7.7%	4.6%	0.0%	0.0%	0.0%	4.0%	0.0%
tr|F1RZK5|F1RZK5	2	2	1	0	0	0	1	0	4	4	1	0	0	0	3	0	6.6%	7.7%	4.6%	0.0%	0.0%	0.0%	4.0%	0.0%
tr|F1RTV1|F1RTV1	5	1	1	0	0	0	1	1	7	1	5	0	0	0	3	1	13.2%	2.4%	6.1%	0.0%	0.0%	0.0%	2.0%	2.0%
tr|F1RTV4|F1RTV4	5	1	1	0	0	0	1	1	7	1	5	0	0	0	3	1	13.7%	2.5%	6.4%	0.0%	0.0%	0.0%	2.1%	2.1%
tr|K7GLU1|K7GLU1	2	2	0	0	0	0	0	1	3	5	0	0	0	0	0	2	3.0%	3.1%	0.0%	0.0%	0.0%	0.0%	0.0%	1.2%
tr|I3LNV3|I3LNV3	2	0	4	0	0	0	0	1	8	0	5	0	0	0	0	3	6.4%	0.0%	11.2%	0.0%	0.0%	0.0%	0.0%	1.8%
tr|F1S3G5|F1S3G5	4	1	2	0	0	0	1	1	7	1	6	0	0	0	1	2	36.2%	3.7%	9.2%	0.0%	0.0%	0.0%	11.7%	11.7%
tr|I3LN21|I3LN21	6	1	1	0	0	0	1	1	7	1	2	0	0	0	1	1	22.5%	2.9%	6.1%	0.0%	0.0%	0.0%	6.1%	7.2%
tr|I3LSU8|I3LSU8	4	0	3	0	0	0	0	1	8	0	8	0	0	0	0	3	8.3%	0.0%	10.7%	0.0%	0.0%	0.0%	0.0%	7.4%
tr|F1SVB2|F1SVB2	4	0	1	0	0	0	0	1	8	0	3	0	0	0	0	2	11.1%	0.0%	1.5%	0.0%	0.0%	0.0%	0.0%	4.9%
tr|I3LDQ1|I3LDQ1	3	1	1	0	0	0	0	1	6	2	6	0	0	0	0	2	39.9%	9.8%	9.8%	0.0%	0.0%	0.0%	0.0%	17.5%
tr|K7GSP4|K7GSP4	1	0	0	0	0	0	0	1	8	0	0	0	0	0	0	1	24.3%	0.0%	0.0%	0.0%	0.0%	0.0%	0.0%	24.3%
tr|K7GRD4|K7GRD4	1	0	0	0	0	0	0	1	8	0	0	0	0	0	0	1	21.5%	0.0%	0.0%	0.0%	0.0%	0.0%	0.0%	21.5%
Q6QA76	1	0	0	0	0	0	0	1	8	0	0	0	0	0	0	1	18.6%	0.0%	0.0%	0.0%	0.0%	0.0%	0.0%	18.6%
tr|K7GNX1|K7GNX1	1	0	0	0	0	0	0	1	8	0	0	0	0	0	0	1	19.1%	0.0%	0.0%	0.0%	0.0%	0.0%	0.0%	19.1%
tr|F1SQK5|F1SQK5	2	0	0	0	0	0	0	1	8	0	0	0	0	0	0	1	27.5%	0.0%	0.0%	0.0%	0.0%	0.0%	0.0%	14.0%
tr|I3LRX9|I3LRX9	6	1	3	0	0	0	1	0	7	1	17	0	0	0	2	0	71.1%	40.8%	71.1%	0.0%	0.0%	0.0%	40.8%	0.0%
tr|F1RJ35|F1RJ35	1	1	0	0	0	0	0	0	1	7	0	0	0	0	0	0	3.5%	3.5%	0.0%	0.0%	0.0%	0.0%	0.0%	0.0%
tr|F1S396|F1S396	4	0	0	0	0	0	0	0	8	0	0	0	0	0	0	0	9.3%	0.0%	0.0%	0.0%	0.0%	0.0%	0.0%	0.0%
tr|F1RY52|F1RY52	3	0	0	0	0	0	0	0	8	0	0	0	0	0	0	0	7.5%	0.0%	0.0%	0.0%	0.0%	0.0%	0.0%	0.0%
tr|F2Z591|F2Z591	4	1	0	0	0	0	0	0	6	2	0	0	0	0	0	0	20.8%	4.4%	0.0%	0.0%	0.0%	0.0%	0.0%	0.0%
tr|F1SPS7|F1SPS7	0	1	0	0	0	0	0	0	0	8	0	0	0	0	0	0	0.0%	1.5%	0.0%	0.0%	0.0%	0.0%	0.0%	0.0%
tr|F1SAB2|F1SAB2	3	0	0	0	0	0	0	0	8	0	0	0	0	0	0	0	9.3%	0.0%	0.0%	0.0%	0.0%	0.0%	0.0%	0.0%
tr|F1SSE6|F1SSE6	4	2	0	0	0	0	0	0	5	3	0	0	0	0	0	0	20.4%	4.6%	0.0%	0.0%	0.0%	0.0%	0.0%	0.0%
tr|F1RYU6|F1RYU6	1	2	0	0	0	0	0	0	2	6	0	0	0	0	0	0	2.6%	4.5%	0.0%	0.0%	0.0%	0.0%	0.0%	0.0%
tr|K7GLU3|K7GLU3	0	1	0	0	0	0	0	0	0	8	0	0	0	0	0	0	0.0%	3.1%	0.0%	0.0%	0.0%	0.0%	0.0%	0.0%
tr|F1RFF5|F1RFF5	4	0	0	0	0	0	0	0	8	0	0	0	0	0	0	0	18.0%	0.0%	0.0%	0.0%	0.0%	0.0%	0.0%	0.0%
tr|F1S0H7|F1S0H7	2	2	0	0	0	0	0	0	2	6	0	0	0	0	0	0	3.7%	6.4%	0.0%	0.0%	0.0%	0.0%	0.0%	0.0%
tr|I3LDE2|I3LDE2	0	2	0	0	0	0	0	0	0	8	0	0	0	0	0	0	0.0%	13.1%	0.0%	0.0%	0.0%	0.0%	0.0%	0.0%
tr|A7YX22|A7YX22	3	2	0	0	0	0	0	0	3	5	0	0	0	0	0	0	11.7%	10.5%	0.0%	0.0%	0.0%	0.0%	0.0%	0.0%
tr|F1SNB7|F1SNB7	4	1	0	0	0	0	0	0	7	1	0	0	0	0	0	0	14.1%	2.8%	0.0%	0.0%	0.0%	0.0%	0.0%	0.0%
Q9TUZ0	3	2	0	0	0	0	0	0	4	4	0	0	0	0	0	0	6.6%	2.7%	0.0%	0.0%	0.0%	0.0%	0.0%	0.0%
tr|F1SQH6|F1SQH6	3	2	0	0	0	0	0	0	5	3	0	0	0	0	0	0	7.7%	3.9%	0.0%	0.0%	0.0%	0.0%	0.0%	0.0%
tr|F1STU5|F1STU5	1	2	0	0	0	0	0	0	1	7	0	0	0	0	0	0	8.8%	8.8%	0.0%	0.0%	0.0%	0.0%	0.0%	0.0%
P19205	0	1	0	0	0	0	0	0	0	8	0	0	0	0	0	0	0.0%	1.5%	0.0%	0.0%	0.0%	0.0%	0.0%	0.0%
tr|F1SML7|F1SML7	6	0	0	0	0	0	0	0	8	0	0	0	0	0	0	0	18.9%	0.0%	0.0%	0.0%	0.0%	0.0%	0.0%	0.0%
tr|F1RNB0|F1RNB0	3	2	0	0	0	0	0	0	3	5	0	0	0	0	0	0	4.3%	1.2%	0.0%	0.0%	0.0%	0.0%	0.0%	0.0%
tr|I3LFX8|I3LFX8	0	1	0	0	0	0	0	0	0	8	0	0	0	0	0	0	0.0%	1.6%	0.0%	0.0%	0.0%	0.0%	0.0%	0.0%
tr|F1RK86|F1RK86	2	0	0	0	0	0	0	0	8	0	0	0	0	0	0	0	6.5%	0.0%	0.0%	0.0%	0.0%	0.0%	0.0%	0.0%
tr|I3LKD4|I3LKD4	3	0	0	0	0	0	0	0	8	0	0	0	0	0	0	0	11.7%	0.0%	0.0%	0.0%	0.0%	0.0%	0.0%	0.0%
tr|F1SRK2|F1SRK2	3	1	0	0	0	0	0	0	3	5	0	0	0	0	0	0	13.1%	2.4%	0.0%	0.0%	0.0%	0.0%	0.0%	0.0%
tr|F1S034|F1S034	0	2	0	0	0	0	0	0	0	8	0	0	0	0	0	0	0.0%	7.2%	0.0%	0.0%	0.0%	0.0%	0.0%	0.0%
tr|F1SDW6|F1SDW6	0	2	0	0	0	0	0	0	0	8	0	0	0	0	0	0	0.0%	2.0%	0.0%	0.0%	0.0%	0.0%	0.0%	0.0%
tr|I3LL64|I3LL64	3	1	0	0	0	0	0	0	7	1	0	0	0	0	0	0	24.3%	8.9%	0.0%	0.0%	0.0%	0.0%	0.0%	0.0%
tr|I3LMX3|I3LMX3	3	1	0	0	0	0	0	0	4	4	0	0	0	0	0	0	6.5%	3.2%	0.0%	0.0%	0.0%	0.0%	0.0%	0.0%
tr|I3LA56|I3LA56	3	0	0	0	0	0	0	0	8	0	0	0	0	0	0	0	12.6%	0.0%	0.0%	0.0%	0.0%	0.0%	0.0%	0.0%
tr|F1SFW4|F1SFW4	6	0	0	0	0	0	0	0	8	0	0	0	0	0	0	0	8.4%	0.0%	0.0%	0.0%	0.0%	0.0%	0.0%	0.0%
tr|F1RL04|F1RL04	3	0	0	0	0	0	0	0	8	0	0	0	0	0	0	0	14.2%	0.0%	0.0%	0.0%	0.0%	0.0%	0.0%	0.0%
tr|F1SKB5|F1SKB5	3	1	0	0	0	0	0	0	4	4	0	0	0	0	0	0	4.1%	2.6%	0.0%	0.0%	0.0%	0.0%	0.0%	0.0%
tr|F1RLM2|F1RLM2	3	0	0	0	0	0	0	0	8	0	0	0	0	0	0	0	11.6%	0.0%	0.0%	0.0%	0.0%	0.0%	0.0%	0.0%
tr|F1RZP9|F1RZP9	1	2	0	0	0	0	0	0	1	7	0	0	0	0	0	0	2.3%	2.3%	0.0%	0.0%	0.0%	0.0%	0.0%	0.0%
tr|I3LMN3|I3LMN3	2	0	0	0	0	0	0	0	8	0	0	0	0	0	0	0	6.7%	0.0%	0.0%	0.0%	0.0%	0.0%	0.0%	0.0%
tr|K7GT21|K7GT21	0	1	0	0	0	0	0	0	0	8	0	0	0	0	0	0	0.0%	5.7%	0.0%	0.0%	0.0%	0.0%	0.0%	0.0%
tr|A1YPR8|A1YPR8	0	1	0	0	0	0	0	0	0	8	0	0	0	0	0	0	0.0%	2.6%	0.0%	0.0%	0.0%	0.0%	0.0%	0.0%
tr|I3L9H8|I3L9H8	1	1	0	0	0	0	0	0	1	7	0	0	0	0	0	0	3.4%	2.9%	0.0%	0.0%	0.0%	0.0%	0.0%	0.0%
tr|F1RX98|F1RX98	3	0	0	0	0	0	0	0	8	0	0	0	0	0	0	0	13.8%	0.0%	0.0%	0.0%	0.0%	0.0%	0.0%	0.0%
tr|F1RL99|F1RL99	5	2	0	0	0	0	0	0	6	2	0	0	0	0	0	0	9.8%	2.2%	0.0%	0.0%	0.0%	0.0%	0.0%	0.0%
tr|I3LEU6|I3LEU6	0	1	0	0	0	0	0	0	0	8	0	0	0	0	0	0	0.0%	1.5%	0.0%	0.0%	0.0%	0.0%	0.0%	0.0%
tr|I3LRI0|I3LRI0	6	0	0	0	0	0	0	0	8	0	0	0	0	0	0	0	20.5%	0.0%	0.0%	0.0%	0.0%	0.0%	0.0%	0.0%

	Peptide Count								Spectral Count								Sequence Coverage							
	Whole Cell	Whole Cell	Whole Cell	Cilia Fraction	Cilia Fraction	Cilia Fraction	Cilia Fraction	Cilia Fraction	Whole Cell	Whole Cell	Whole Cell	Cilia Fraction	Cilia Fraction	Cilia Fraction	Cilia Fraction	Cilia Fraction	Whole Cell	Whole Cell	Whole Cell	Cilia Fraction	Cilia Fraction	Cilia Fraction	Cilia Fraction	Cilia Fraction
Sample#	1	2	6	4	3	5	7	8	1	2	6	4	3	5	7	8	1	2	6	4	3	5	7	8
**Accession**																								
tr|F1S0M9|F1S0M9	4	1	0	0	0	0	0	0	7	1	0	0	0	0	0	0	12.9%	3.9%	0.0%	0.0%	0.0%	0.0%	0.0%	0.0%
tr|F1RRB4|F1RRB4	1	3	1	0	0	0	0	0	1	7	1	0	0	0	0	0	1.5%	5.5%	2.8%	0.0%	0.0%	0.0%	0.0%	0.0%
tr|I3LFK0|I3LFK0	2	3	1	0	0	0	0	0	3	5	1	0	0	0	0	0	7.2%	8.8%	3.8%	0.0%	0.0%	0.0%	0.0%	0.0%
tr|F1SER1|F1SER1	3	1	1	0	0	0	0	0	7	1	1	0	0	0	0	0	9.1%	5.3%	3.8%	0.0%	0.0%	0.0%	0.0%	0.0%
tr|K7GKC7|K7GKC7	3	1	1	0	0	0	0	0	7	1	2	0	0	0	0	0	20.1%	8.7%	4.4%	0.0%	0.0%	0.0%	0.0%	0.0%
tr|F1STV0|F1STV0	2	0	1	0	0	0	0	0	8	0	2	0	0	0	0	0	3.9%	0.0%	2.8%	0.0%	0.0%	0.0%	0.0%	0.0%
tr|K7GSK7|K7GSK7	3	1	1	0	0	0	0	0	7	1	2	0	0	0	0	0	23.0%	10.0%	5.0%	0.0%	0.0%	0.0%	0.0%	0.0%
tr|F1RKS3|F1RKS3	6	0	1	0	0	0	0	0	8	0	3	0	0	0	0	0	27.6%	0.0%	5.3%	0.0%	0.0%	0.0%	0.0%	0.0%
tr|F1SB38|F1SB38	3	1	2	0	0	0	0	0	7	1	3	0	0	0	0	0	7.3%	2.7%	3.9%	0.0%	0.0%	0.0%	0.0%	0.0%
tr|F1S402|F1S402	2	2	2	0	0	0	0	0	3	5	4	0	0	0	0	0	1.1%	1.0%	0.9%	0.0%	0.0%	0.0%	0.0%	0.0%
tr|F1RQ19|F1RQ19	2	1	1	0	0	0	0	0	4	4	4	0	0	0	0	0	15.8%	5.9%	6.9%	0.0%	0.0%	0.0%	0.0%	0.0%
tr|F1S8E9|F1S8E9	2	1	1	0	0	0	0	0	7	1	4	0	0	0	0	0	6.4%	2.1%	2.5%	0.0%	0.0%	0.0%	0.0%	0.0%
tr|I3LEL9|I3LEL9	2	1	3	0	0	0	0	0	7	1	5	0	0	0	0	0	6.6%	6.6%	11.6%	0.0%	0.0%	0.0%	0.0%	0.0%
tr|F1S2D0|F1S2D0	4	0	2	0	0	0	0	0	8	0	5	0	0	0	0	0	16.9%	0.0%	8.9%	0.0%	0.0%	0.0%	0.0%	0.0%
tr|I3LRW8|I3LRW8	2	1	1	0	0	0	0	0	5	3	6	0	0	0	0	0	13.3%	4.8%	4.3%	0.0%	0.0%	0.0%	0.0%	0.0%
Q06A98	2	1	1	0	0	0	0	0	5	3	6	0	0	0	0	0	11.3%	4.1%	3.6%	0.0%	0.0%	0.0%	0.0%	0.0%
tr|F1RXG7|F1RXG7	4	0	1	0	0	0	0	0	8	0	7	0	0	0	0	0	34.0%	0.0%	4.7%	0.0%	0.0%	0.0%	0.0%	0.0%
tr|F1RZU5|F1RZU5	3	1	4	0	0	0	0	0	7	1	8	0	0	0	0	0	19.6%	8.5%	12.3%	0.0%	0.0%	0.0%	0.0%	0.0%
tr|I3LV33|I3LV33	3	0	1	0	0	0	0	0	8	0	8	0	0	0	0	0	22.8%	0.0%	15.2%	0.0%	0.0%	0.0%	0.0%	0.0%
tr|I3LBE6|I3LBE6	3	2	1	0	0	0	0	0	3	5	8	0	0	0	0	0	9.7%	4.9%	1.3%	0.0%	0.0%	0.0%	0.0%	0.0%
tr|K7GN50|K7GN50	4	2	1	0	0	0	0	0	6	2	21	0	0	0	0	0	19.5%	9.7%	6.5%	0.0%	0.0%	0.0%	0.0%	0.0%
tr|F1S9I0|F1S9I0	6	0	7	0	0	0	0	0	8	0	23	0	0	0	0	0	4.8%	0.0%	5.7%	0.0%	0.0%	0.0%	0.0%	0.0%
tr|F1STB2|F1STB2	4	0	2	0	0	0	0	0	8	0	31	0	0	0	0	0	29.4%	0.0%	12.3%	0.0%	0.0%	0.0%	0.0%	0.0%
tr|F1RFI8|F1RFI8	4	1	2	0	0	0	0	0	4	4	32	0	0	0	0	0	16.5%	2.5%	4.8%	0.0%	0.0%	0.0%	0.0%	0.0%
P21753	2	1	3	0	0	0	0	0	7	1	49	0	0	0	0	0	31.0%	31.0%	45.2%	0.0%	0.0%	0.0%	0.0%	0.0%
tr|I3LJ48|I3LJ48	5	0	2	0	0	0	0	0	8	0	50	0	0	0	0	0	56.8%	0.0%	13.0%	0.0%	0.0%	0.0%	0.0%	0.0%
tr|F1RZN4|F1RZN4	5	1	1	0	0	0	3	2	6	1	4	0	0	0	18	10	8.2%	1.2%	2.1%	0.0%	0.0%	0.0%	6.9%	2.3%
tr|A0ENH4|A0ENH4	5	1	1	0	0	0	3	2	6	1	4	0	0	0	18	10	8.2%	1.2%	2.1%	0.0%	0.0%	0.0%	6.9%	2.3%
tr|F2Z5F3|F2Z5F3	3	1	0	0	0	0	1	1	5	2	0	0	0	0	11	6	20.4%	5.8%	0.0%	0.0%	0.0%	0.0%	5.8%	6.1%
tr|F1SIC0|F1SIC0	4	0	0	0	0	0	2	4	7	0	0	0	0	0	2	14	10.4%	0.0%	0.0%	0.0%	0.0%	0.0%	6.6%	14.4%
tr|I3LVD1|I3LVD1	4	0	0	0	0	0	1	4	7	0	0	0	0	0	1	14	10.7%	0.0%	0.0%	0.0%	0.0%	0.0%	3.9%	14.8%
tr|F1SIM3|F1SIM3	3	1	2	0	0	0	1	4	4	3	2	0	0	0	4	12	12.5%	2.5%	5.1%	0.0%	0.0%	0.0%	3.2%	13.6%
tr|K7GNH0|K7GNH0	3	1	2	0	0	0	1	4	4	3	2	0	0	0	4	12	12.3%	2.4%	5.0%	0.0%	0.0%	0.0%	3.1%	13.4%
tr|F1S520|F1S520	6	0	3	0	0	0	1	4	7	0	8	0	0	0	11	14	11.4%	0.0%	4.4%	0.0%	0.0%	0.0%	3.3%	9.0%
tr|I3L548|I3L548	3	0	0	0	0	0	1	1	7	0	0	0	0	0	8	3	5.9%	0.0%	0.0%	0.0%	0.0%	0.0%	2.1%	2.1%
F1SPM8	3	0	0	0	0	0	1	1	7	0	0	0	0	0	8	3	5.9%	0.0%	0.0%	0.0%	0.0%	0.0%	2.1%	2.1%
tr|F1RQZ0|F1RQZ0	5	0	1	0	0	0	1	2	7	0	1	0	0	0	2	5	8.9%	0.0%	2.9%	0.0%	0.0%	0.0%	5.8%	8.4%
tr|F1RWN4|F1RWN4	2	1	0	0	0	0	0	1	5	2	0	0	0	0	0	6	21.4%	9.5%	0.0%	0.0%	0.0%	0.0%	0.0%	11.9%
tr|B6E241|B6E241	5	0	1	0	0	0	1	2	7	0	4	0	0	0	3	6	21.2%	0.0%	4.1%	0.0%	0.0%	0.0%	13.4%	9.7%
tr|F1SSH7|F1SSH7	5	0	1	0	0	0	0	3	7	0	6	0	0	0	0	10	27.8%	0.0%	7.1%	0.0%	0.0%	0.0%	0.0%	17.4%
tr|F1SH28|F1SH28	2	1	0	0	0	0	1	1	2	5	0	0	0	0	2	3	5.7%	3.1%	0.0%	0.0%	0.0%	0.0%	5.2%	4.6%
tr|I3LUE6|I3LUE6	2	1	0	0	0	0	0	2	6	1	0	0	0	0	0	4	13.8%	13.8%	0.0%	0.0%	0.0%	0.0%	0.0%	13.8%
tr|F1SNT0|F1SNT0	3	0	1	0	0	0	2	0	7	0	6	0	0	0	7	0	21.0%	0.0%	8.2%	0.0%	0.0%	0.0%	16.4%	0.0%
tr|F1RJU9|F1RJU9	4	1	2	0	0	0	0	3	6	1	2	0	0	0	0	4	11.8%	4.6%	6.2%	0.0%	0.0%	0.0%	0.0%	13.0%
tr|F1S861|F1S861	4	0	0	0	0	0	1	0	7	0	0	0	0	0	3	0	6.6%	0.0%	0.0%	0.0%	0.0%	0.0%	2.7%	0.0%
tr|F2Z5Q3|F2Z5Q3	2	2	0	0	0	0	1	1	2	5	0	0	0	0	1	2	10.9%	11.6%	0.0%	0.0%	0.0%	0.0%	5.1%	11.3%
tr|K7GPP9|K7GPP9	2	0	0	0	0	0	0	1	7	0	0	0	0	0	0	3	24.5%	0.0%	0.0%	0.0%	0.0%	0.0%	0.0%	24.5%
tr|F1SEH2|F1SEH2	2	0	0	0	0	0	0	1	7	0	0	0	0	0	0	3	19.0%	0.0%	0.0%	0.0%	0.0%	0.0%	0.0%	19.0%
tr|F1SRU2|F1SRU2	2	0	2	0	0	0	0	3	7	0	8	0	0	0	0	5	11.1%	0.0%	10.1%	0.0%	0.0%	0.0%	0.0%	21.2%
P29269	4	0	2	0	0	0	0	1	7	0	10	0	0	0	0	5	26.7%	0.0%	15.7%	0.0%	0.0%	0.0%	0.0%	16.3%
tr|A9LM01|A9LM01	2	1	0	0	0	0	0	2	5	2	0	0	0	0	0	2	20.2%	6.3%	0.0%	0.0%	0.0%	0.0%	0.0%	15.2%
tr|I3L643|I3L643	3	1	0	0	0	0	0	1	6	1	0	0	0	0	0	2	14.4%	7.0%	0.0%	0.0%	0.0%	0.0%	0.0%	7.6%
O46427	2	2	2	0	0	0	0	1	4	3	14	0	0	0	0	5	17.9%	7.8%	5.4%	0.0%	0.0%	0.0%	0.0%	10.1%
tr|I3LQI9|I3LQI9	4	0	3	0	0	0	1	0	7	0	7	0	0	0	3	0	10.0%	0.0%	10.2%	0.0%	0.0%	0.0%	3.9%	0.0%
tr|F1SJM2|F1SJM2	3	0	4	0	0	0	1	1	7	0	12	0	0	0	3	1	41.0%	0.0%	30.8%	0.0%	0.0%	0.0%	13.3%	12.3%
tr|F1SPI1|F1SPI1	4	3	1	0	0	0	0	2	4	3	13	0	0	0	0	4	16.0%	7.2%	2.5%	0.0%	0.0%	0.0%	0.0%	6.8%
tr|I3LPY7|I3LPY7	5	0	2	0	0	0	1	0	7	0	8	0	0	0	3	0	20.4%	0.0%	8.5%	0.0%	0.0%	0.0%	5.3%	0.0%
tr|F1RPD4|F1RPD4	3	1	1	0	0	0	0	1	6	1	4	0	0	0	0	2	44.9%	7.0%	7.5%	0.0%	0.0%	0.0%	0.0%	17.3%
tr|K7GLK3|K7GLK3	5	0	6	0	0	0	0	3	7	0	29	0	0	0	0	6	36.9%	0.0%	25.9%	0.0%	0.0%	0.0%	0.0%	25.5%
tr|F1SQD9|F1SQD9	4	1	1	0	0	0	0	1	6	1	6	0	0	0	0	2	18.8%	2.9%	4.0%	0.0%	0.0%	0.0%	0.0%	8.3%
tr|F1RQB3|F1RQB3	3	1	2	0	0	0	0	1	4	3	20	0	0	0	0	4	26.5%	6.5%	10.3%	0.0%	0.0%	0.0%	0.0%	6.5%
tr|A3RKG5|A3RKG5	2	1	0	0	0	0	0	1	5	2	0	0	0	0	0	1	27.3%	8.5%	0.0%	0.0%	0.0%	0.0%	0.0%	8.5%
tr|I3L545|I3L545	3	1	0	0	0	0	0	1	3	4	0	0	0	0	0	1	9.1%	2.3%	0.0%	0.0%	0.0%	0.0%	0.0%	3.2%
tr|F1SR60|F1SR60	0	1	0	0	0	0	0	1	0	7	0	0	0	0	0	1	0.0%	4.2%	0.0%	0.0%	0.0%	0.0%	0.0%	4.2%
tr|F1SA44|F1SA44	7	0	4	0	0	0	0	1	7	0	9	0	0	0	0	2	19.1%	0.0%	11.8%	0.0%	0.0%	0.0%	0.0%	4.4%
tr|F1SCF5|F1SCF5	5	1	1	0	0	0	0	1	5	2	2	0	0	0	0	1	20.6%	3.5%	2.9%	0.0%	0.0%	0.0%	0.0%	4.1%
tr|I3LEF2|I3LEF2	5	0	1	0	0	0	1	0	7	0	4	0	0	0	1	0	27.7%	0.0%	4.9%	0.0%	0.0%	0.0%	6.7%	0.0%
tr|F1SGK9|F1SGK9	5	2	3	0	0	0	1	1	5	2	18	0	0	0	1	1	9.2%	4.1%	11.2%	0.0%	0.0%	0.0%	5.6%	4.4%
tr|I3LK43|I3LK43	1	1	1	0	0	0	0	1	1	6	7	0	0	0	0	1	4.2%	11.6%	4.6%	0.0%	0.0%	0.0%	0.0%	11.6%
tr|I3LHL4|I3LHL4	1	1	1	0	0	0	0	1	1	6	7	0	0	0	0	1	4.1%	11.5%	4.6%	0.0%	0.0%	0.0%	0.0%	11.5%
tr|F1RRN1|F1RRN1	6	0	3	0	0	0	0	1	7	0	9	0	0	0	0	1	11.7%	0.0%	4.2%	0.0%	0.0%	0.0%	0.0%	2.3%
tr|F1RXD4|F1RXD4	3	2	2	0	0	0	0	1	4	3	10	0	0	0	0	1	18.3%	2.8%	4.4%	0.0%	0.0%	0.0%	0.0%	6.3%
tr|I3LGI9|I3LGI9	2	1	3	0	0	0	0	1	4	3	26	0	0	0	0	1	10.0%	4.8%	13.1%	0.0%	0.0%	0.0%	0.0%	4.8%
tr|F1SL47|F1SL47	2	2	0	0	0	0	0	0	2	5	0	0	0	0	0	0	6.3%	4.2%	0.0%	0.0%	0.0%	0.0%	0.0%	0.0%
tr|K7GS54|K7GS54	3	1	0	0	0	0	0	0	6	1	0	0	0	0	0	0	21.1%	5.6%	0.0%	0.0%	0.0%	0.0%	0.0%	0.0%
tr|F1S1K1|F1S1K1	3	1	0	0	0	0	0	0	6	1	0	0	0	0	0	0	4.9%	1.8%	0.0%	0.0%	0.0%	0.0%	0.0%	0.0%
tr|F1S131|F1S131	2	3	0	0	0	0	0	0	2	5	0	0	0	0	0	0	10.3%	11.6%	0.0%	0.0%	0.0%	0.0%	0.0%	0.0%
tr|F1RGD8|F1RGD8	2	2	0	0	0	0	0	0	2	5	0	0	0	0	0	0	9.9%	4.5%	0.0%	0.0%	0.0%	0.0%	0.0%	0.0%
tr|K7GL03|K7GL03	3	3	0	0	0	0	0	0	3	4	0	0	0	0	0	0	20.3%	17.1%	0.0%	0.0%	0.0%	0.0%	0.0%	0.0%
tr|F1RTM1|F1RTM1	3	0	0	0	0	0	0	0	7	0	0	0	0	0	0	0	18.9%	0.0%	0.0%	0.0%	0.0%	0.0%	0.0%	0.0%
tr|F1SQV8|F1SQV8	5	0	0	0	0	0	0	0	7	0	0	0	0	0	0	0	13.6%	0.0%	0.0%	0.0%	0.0%	0.0%	0.0%	0.0%

	Peptide Count								Spectral Count								Sequence Coverage							
	Whole Cell	Whole Cell	Whole Cell	Cilia Fraction	Cilia Fraction	Cilia Fraction	Cilia Fraction	Cilia Fraction	Whole Cell	Whole Cell	Whole Cell	Cilia Fraction	Cilia Fraction	Cilia Fraction	Cilia Fraction	Cilia Fraction	Whole Cell	Whole Cell	Whole Cell	Cilia Fraction	Cilia Fraction	Cilia Fraction	Cilia Fraction	Cilia Fraction
Sample#	1	2	6	4	3	5	7	8	1	2	6	4	3	5	7	8	1	2	6	4	3	5	7	8
**Accession**																								
tr|F1RS87|F1RS87	0	2	0	0	0	0	0	0	0	7	0	0	0	0	0	0	0.0%	14.0%	0.0%	0.0%	0.0%	0.0%	0.0%	0.0%
tr|F1RH94|F1RH94	2	1	0	0	0	0	0	0	6	1	0	0	0	0	0	0	11.7%	5.1%	0.0%	0.0%	0.0%	0.0%	0.0%	0.0%
tr|F1STH9|F1STH9	1	2	0	0	0	0	0	0	3	4	0	0	0	0	0	0	3.8%	2.5%	0.0%	0.0%	0.0%	0.0%	0.0%	0.0%
tr|K7GRA1|K7GRA1	4	2	0	0	0	0	0	0	5	2	0	0	0	0	0	0	6.9%	4.6%	0.0%	0.0%	0.0%	0.0%	0.0%	0.0%
tr|F1RU15|F1RU15	1	1	0	0	0	0	0	0	4	3	0	0	0	0	0	0	6.9%	6.9%	0.0%	0.0%	0.0%	0.0%	0.0%	0.0%
tr|I3LGG1|I3LGG1	0	2	0	0	0	0	0	0	0	7	0	0	0	0	0	0	0.0%	13.5%	0.0%	0.0%	0.0%	0.0%	0.0%	0.0%
tr|F1RJR9|F1RJR9	3	2	0	0	0	0	0	0	5	2	0	0	0	0	0	0	25.9%	6.6%	0.0%	0.0%	0.0%	0.0%	0.0%	0.0%
tr|I3LL38|I3LL38	4	0	0	0	0	0	0	0	7	0	0	0	0	0	0	0	44.7%	0.0%	0.0%	0.0%	0.0%	0.0%	0.0%	0.0%
tr|K7GNI4|K7GNI4	0	1	0	0	0	0	0	0	0	7	0	0	0	0	0	0	0.0%	6.4%	0.0%	0.0%	0.0%	0.0%	0.0%	0.0%
tr|K7GT54|K7GT54	0	1	0	0	0	0	0	0	0	7	0	0	0	0	0	0	0.0%	5.1%	0.0%	0.0%	0.0%	0.0%	0.0%	0.0%
tr|I3LUT2|I3LUT2	0	1	0	0	0	0	0	0	0	7	0	0	0	0	0	0	0.0%	8.1%	0.0%	0.0%	0.0%	0.0%	0.0%	0.0%
tr|I3LIP8|I3LIP8	1	1	0	0	0	0	0	0	1	6	0	0	0	0	0	0	7.4%	7.0%	0.0%	0.0%	0.0%	0.0%	0.0%	0.0%
tr|F1SFF6|F1SFF6	2	1	0	0	0	0	0	0	5	2	0	0	0	0	0	0	9.0%	2.9%	0.0%	0.0%	0.0%	0.0%	0.0%	0.0%
tr|F1SS29|F1SS29	0	1	0	0	0	0	0	0	0	7	0	0	0	0	0	0	0.0%	9.1%	0.0%	0.0%	0.0%	0.0%	0.0%	0.0%
tr|F1RGD0|F1RGD0	0	2	0	0	0	0	0	0	0	7	0	0	0	0	0	0	0.0%	4.0%	0.0%	0.0%	0.0%	0.0%	0.0%	0.0%
tr|I3LM77|I3LM77	1	1	0	0	0	0	0	0	2	5	0	0	0	0	0	0	2.8%	4.3%	0.0%	0.0%	0.0%	0.0%	0.0%	0.0%
tr|F1RWL6|F1RWL6	1	1	0	0	0	0	0	0	2	5	0	0	0	0	0	0	2.6%	4.0%	0.0%	0.0%	0.0%	0.0%	0.0%	0.0%
tr|F1SLH1|F1SLH1	1	0	0	0	0	0	0	0	7	0	0	0	0	0	0	0	21.9%	0.0%	0.0%	0.0%	0.0%	0.0%	0.0%	0.0%
tr|F1S4Q6|F1S4Q6	4	1	0	0	0	0	0	0	5	2	0	0	0	0	0	0	8.2%	1.0%	0.0%	0.0%	0.0%	0.0%	0.0%	0.0%
tr|F1S1V9|F1S1V9	4	0	0	0	0	0	0	0	7	0	0	0	0	0	0	0	10.4%	0.0%	0.0%	0.0%	0.0%	0.0%	0.0%	0.0%
tr|F1S1K0|F1S1K0	3	1	0	0	0	0	0	0	6	1	0	0	0	0	0	0	4.4%	1.6%	0.0%	0.0%	0.0%	0.0%	0.0%	0.0%
tr|F2Z5Q5|F2Z5Q5	0	2	0	0	0	0	0	0	0	7	0	0	0	0	0	0	0.0%	10.5%	0.0%	0.0%	0.0%	0.0%	0.0%	0.0%
tr|I3LNP9|I3LNP9	2	2	0	0	0	0	0	0	2	5	0	0	0	0	0	0	12.3%	12.6%	0.0%	0.0%	0.0%	0.0%	0.0%	0.0%
tr|F1SPT0|F1SPT0	2	1	0	0	0	0	0	0	2	5	0	0	0	0	0	0	3.3%	0.9%	0.0%	0.0%	0.0%	0.0%	0.0%	0.0%
tr|F1SCH1|F1SCH1	2	1	0	0	0	0	0	0	5	2	0	0	0	0	0	0	27.4%	11.3%	0.0%	0.0%	0.0%	0.0%	0.0%	0.0%
tr|F1S4D6|F1S4D6	2	0	0	0	0	0	0	0	7	0	0	0	0	0	0	0	2.9%	0.0%	0.0%	0.0%	0.0%	0.0%	0.0%	0.0%
tr|F1SEL1|F1SEL1	3	1	0	0	0	0	0	0	3	4	0	0	0	0	0	0	11.9%	3.0%	0.0%	0.0%	0.0%	0.0%	0.0%	0.0%
tr|F2Z5B8|F2Z5B8	1	2	0	0	0	0	0	0	4	3	0	0	0	0	0	0	22.6%	31.5%	0.0%	0.0%	0.0%	0.0%	0.0%	0.0%
tr|I3LGB7|I3LGB7	4	0	0	0	0	0	0	0	7	0	0	0	0	0	0	0	44.9%	0.0%	0.0%	0.0%	0.0%	0.0%	0.0%	0.0%
tr|I3LVF5|I3LVF5	2	2	0	0	0	0	0	0	5	2	0	0	0	0	0	0	18.0%	18.5%	0.0%	0.0%	0.0%	0.0%	0.0%	0.0%
tr|F1S0Q9|F1S0Q9	2	2	0	0	0	0	0	0	3	4	0	0	0	0	0	0	4.8%	2.7%	0.0%	0.0%	0.0%	0.0%	0.0%	0.0%
tr|K7GR29|K7GR29	4	2	0	0	0	0	0	0	5	2	0	0	0	0	0	0	7.0%	4.6%	0.0%	0.0%	0.0%	0.0%	0.0%	0.0%
tr|F1RU27|F1RU27	1	2	0	0	0	0	0	0	1	6	0	0	0	0	0	0	4.4%	6.8%	0.0%	0.0%	0.0%	0.0%	0.0%	0.0%
tr|F1RTI8|F1RTI8	4	2	0	0	0	0	0	0	5	2	0	0	0	0	0	0	6.9%	4.6%	0.0%	0.0%	0.0%	0.0%	0.0%	0.0%
tr|F1RU72|F1RU72	3	2	1	0	0	0	0	0	3	4	1	0	0	0	0	0	4.0%	1.6%	1.1%	0.0%	0.0%	0.0%	0.0%	0.0%
tr|F1RU28|F1RU28	2	2	1	0	0	0	0	0	2	5	1	0	0	0	0	0	5.9%	5.9%	12.2%	0.0%	0.0%	0.0%	0.0%	0.0%
tr|I3LKR1|I3LKR1	4	0	1	0	0	0	0	0	7	0	1	0	0	0	0	0	7.9%	0.0%	3.8%	0.0%	0.0%	0.0%	0.0%	0.0%
tr|I3LQV2|I3LQV2	4	1	1	0	0	0	0	0	5	2	2	0	0	0	0	0	24.6%	3.6%	4.8%	0.0%	0.0%	0.0%	0.0%	0.0%
tr|F1RHZ8|F1RHZ8	4	0	1	0	0	0	0	0	7	0	2	0	0	0	0	0	26.9%	0.0%	5.2%	0.0%	0.0%	0.0%	0.0%	0.0%
tr|F1RM40|F1RM40	1	2	1	0	0	0	0	0	2	5	2	0	0	0	0	0	2.2%	2.4%	2.0%	0.0%	0.0%	0.0%	0.0%	0.0%
tr|F1SSS9|F1SSS9	2	1	2	0	0	0	0	0	3	4	3	0	0	0	0	0	21.4%	11.9%	29.8%	0.0%	0.0%	0.0%	0.0%	0.0%
P79273	3	0	2	0	0	0	0	0	7	0	3	0	0	0	0	0	12.8%	0.0%	11.6%	0.0%	0.0%	0.0%	0.0%	0.0%
tr|F1S8B0|F1S8B0	4	0	2	0	0	0	0	0	7	0	4	0	0	0	0	0	16.0%	0.0%	10.6%	0.0%	0.0%	0.0%	0.0%	0.0%
tr|F1S2N0|F1S2N0	4	0	2	0	0	0	0	0	7	0	4	0	0	0	0	0	31.0%	0.0%	16.7%	0.0%	0.0%	0.0%	0.0%	0.0%
tr|F1RWJ0|F1RWJ0	4	0	1	0	0	0	0	0	7	0	4	0	0	0	0	0	21.8%	0.0%	9.2%	0.0%	0.0%	0.0%	0.0%	0.0%
tr|K7GR50|K7GR50	4	0	2	0	0	0	0	0	7	0	4	0	0	0	0	0	36.4%	0.0%	19.6%	0.0%	0.0%	0.0%	0.0%	0.0%
tr|I3LRG0|I3LRG0	2	1	1	0	0	0	0	0	6	1	4	0	0	0	0	0	15.0%	9.0%	5.2%	0.0%	0.0%	0.0%	0.0%	0.0%
tr|F1SIT4|F1SIT4	4	1	1	0	0	0	0	0	6	1	4	0	0	0	0	0	3.7%	0.9%	1.3%	0.0%	0.0%	0.0%	0.0%	0.0%
tr|K7GQV5|K7GQV5	4	0	2	0	0	0	0	0	7	0	4	0	0	0	0	0	30.9%	0.0%	16.6%	0.0%	0.0%	0.0%	0.0%	0.0%
tr|F1S8S6|F1S8S6	5	0	1	0	0	0	0	0	7	0	5	0	0	0	0	0	12.0%	0.0%	3.3%	0.0%	0.0%	0.0%	0.0%	0.0%
tr|F1S001|F1S001	3	0	1	0	0	0	0	0	7	0	6	0	0	0	0	0	10.7%	0.0%	11.4%	0.0%	0.0%	0.0%	0.0%	0.0%
tr|I3LU08|I3LU08	5	0	2	0	0	0	0	0	7	0	6	0	0	0	0	0	19.9%	0.0%	13.8%	0.0%	0.0%	0.0%	0.0%	0.0%
tr|I3LHK5|I3LHK5	5	1	2	0	0	0	0	0	5	2	6	0	0	0	0	0	20.6%	5.3%	10.6%	0.0%	0.0%	0.0%	0.0%	0.0%
tr|I3L9H6|I3L9H6	3	0	2	0	0	0	0	0	7	0	8	0	0	0	0	0	27.0%	0.0%	13.8%	0.0%	0.0%	0.0%	0.0%	0.0%
tr|K7GKW7|K7GKW7	3	0	4	0	0	0	0	0	7	0	8	0	0	0	0	0	20.9%	0.0%	13.2%	0.0%	0.0%	0.0%	0.0%	0.0%
tr|F1RFD4|F1RFD4	2	1	1	0	0	0	0	0	2	5	8	0	0	0	0	0	27.6%	19.0%	27.6%	0.0%	0.0%	0.0%	0.0%	0.0%
tr|Q32YV9|Q32YV9	5	1	4	0	0	0	0	0	6	1	9	0	0	0	0	0	21.8%	2.1%	11.4%	0.0%	0.0%	0.0%	0.0%	0.0%
tr|F1RXI6|F1RXI6	4	1	2	0	0	0	0	0	6	1	10	0	0	0	0	0	21.7%	4.0%	11.9%	0.0%	0.0%	0.0%	0.0%	0.0%
tr|F1SQ16|F1SQ16	6	1	5	0	0	0	0	0	6	1	11	0	0	0	0	0	2.4%	0.3%	2.2%	0.0%	0.0%	0.0%	0.0%	0.0%
Q5D144	1	1	2	0	0	0	0	0	1	6	12	0	0	0	0	0	3.7%	3.3%	11.0%	0.0%	0.0%	0.0%	0.0%	0.0%
tr|Q2EN80|Q2EN80	2	1	2	0	0	0	0	0	6	1	13	0	0	0	0	0	22.7%	8.6%	21.9%	0.0%	0.0%	0.0%	0.0%	0.0%
tr|F1S4N2|F1S4N2	3	2	3	0	0	0	0	0	5	2	14	0	0	0	0	0	15.1%	9.8%	12.5%	0.0%	0.0%	0.0%	0.0%	0.0%
tr|F1S1A8|F1S1A8	3	1	1	0	0	0	0	0	5	2	14	0	0	0	0	0	5.0%	2.4%	2.6%	0.0%	0.0%	0.0%	0.0%	0.0%
tr|I3LHY0|I3LHY0	5	0	6	0	0	0	0	0	7	0	17	0	0	0	0	0	17.3%	0.0%	16.6%	0.0%	0.0%	0.0%	0.0%	0.0%
tr|F1RNS8|F1RNS8	5	1	3	0	0	0	0	0	5	2	17	0	0	0	0	0	28.0%	6.0%	25.3%	0.0%	0.0%	0.0%	0.0%	0.0%
tr|F1RHA0|F1RHA0	4	1	2	0	0	0	0	0	6	1	17	0	0	0	0	0	13.1%	2.4%	4.1%	0.0%	0.0%	0.0%	0.0%	0.0%
tr|I3LNR4|I3LNR4	4	1	2	0	0	0	0	0	6	1	17	0	0	0	0	0	12.1%	2.2%	3.8%	0.0%	0.0%	0.0%	0.0%	0.0%
tr|F1SLD2|F1SLD2	1	1	3	0	0	0	0	0	4	3	18	0	0	0	0	0	15.9%	5.8%	26.1%	0.0%	0.0%	0.0%	0.0%	0.0%
tr|F1RQS5|F1RQS5	5	0	2	0	0	0	0	0	7	0	19	0	0	0	0	0	11.3%	0.0%	4.6%	0.0%	0.0%	0.0%	0.0%	0.0%
tr|I3LGT5|I3LGT5	4	1	1	0	0	0	0	0	6	1	20	0	0	0	0	0	21.7%	6.8%	8.7%	0.0%	0.0%	0.0%	0.0%	0.0%
Q95283	3	0	3	0	0	0	0	0	7	0	26	0	0	0	0	0	24.7%	0.0%	37.1%	0.0%	0.0%	0.0%	0.0%	0.0%
tr|F1RQ90|F1RQ90	2	2	6	0	0	0	0	0	2	5	44	0	0	0	0	0	4.1%	7.7%	19.5%	0.0%	0.0%	0.0%	0.0%	0.0%
tr|K7GRX3|K7GRX3	2	2	6	0	0	0	0	0	2	5	44	0	0	0	0	0	5.2%	9.8%	24.8%	0.0%	0.0%	0.0%	0.0%	0.0%
tr|I3LGC2|I3LGC2	6	0	10	0	0	0	0	0	7	0	47	0	0	0	0	0	7.0%	0.0%	8.8%	0.0%	0.0%	0.0%	0.0%	0.0%
tr|F1SNM7|F1SNM7	3	0	2	0	0	0	1	4	6	0	6	0	0	0	9	43	16.4%	0.0%	8.0%	0.0%	0.0%	0.0%	7.5%	18.7%
tr|F1S5P2|F1S5P2	2	0	2	0	0	0	2	5	6	0	2	0	0	0	7	14	1.7%	0.0%	2.5%	0.0%	0.0%	0.0%	2.8%	11.4%
tr|F1S340|F1S340	4	1	0	0	0	0	1	2	5	1	0	0	0	0	1	12	21.1%	5.0%	0.0%	0.0%	0.0%	0.0%	11.1%	10.1%
tr|F1RXZ6|F1RXZ6	3	1	0	0	0	0	1	1	4	2	0	0	0	0	6	5	15.1%	5.1%	0.0%	0.0%	0.0%	0.0%	5.1%	5.1%
tr|F1S445|F1S445	4	0	4	0	0	0	1	4	6	0	23	0	0	0	27	26	11.6%	0.0%	14.8%	0.0%	0.0%	0.0%	5.9%	13.9%
tr|K7GKJ1|K7GKJ1	2	1	1	0	0	0	1	3	3	3	1	0	0	0	4	7	15.9%	4.7%	6.1%	0.0%	0.0%	0.0%	6.1%	17.9%
P05207	2	0	3	0	0	0	2	0	6	0	21	0	0	0	24	0	18.7%	0.0%	18.7%	0.0%	0.0%	0.0%	18.7%	0.0%

	Peptide Count								Spectral Count								Sequence Coverage							
	Whole Cell	Whole Cell	Whole Cell	Cilia Fraction	Cilia Fraction	Cilia Fraction	Cilia Fraction	Cilia Fraction	Whole Cell	Whole Cell	Whole Cell	Cilia Fraction	Cilia Fraction	Cilia Fraction	Cilia Fraction	Cilia Fraction	Whole Cell	Whole Cell	Whole Cell	Cilia Fraction	Cilia Fraction	Cilia Fraction	Cilia Fraction	Cilia Fraction
Sample#	1	2	6	4	3	5	7	8	1	2	6	4	3	5	7	8	1	2	6	4	3	5	7	8
**Accession**																								
tr|I3LSJ4|I3LSJ4	4	2	1	0	0	0	1	2	4	2	2	0	0	0	3	4	12.7%	6.5%	2.9%	0.0%	0.0%	0.0%	8.6%	14.4%
Q2HYU2	0	3	0	0	0	0	0	1	0	6	0	0	0	0	0	5	0.0%	3.7%	0.0%	0.0%	0.0%	0.0%	0.0%	1.2%
tr|Q19QT0|Q19QT0	3	0	1	0	0	0	0	2	6	0	1	0	0	0	0	4	10.8%	0.0%	9.5%	0.0%	0.0%	0.0%	0.0%	7.2%
tr|F1SMW4|F1SMW4	2	1	3	0	0	0	2	3	2	4	12	0	0	0	5	4	7.6%	3.3%	10.6%	0.0%	0.0%	0.0%	7.0%	7.9%
tr|I3LAV6|I3LAV6	4	0	0	0	0	0	0	2	6	0	0	0	0	0	0	3	36.4%	0.0%	0.0%	0.0%	0.0%	0.0%	0.0%	31.4%
tr|I3LPD5|I3LPD5	1	2	0	0	0	0	1	1	1	5	0	0	0	0	1	2	4.9%	11.1%	0.0%	0.0%	0.0%	0.0%	4.9%	10.8%
tr|F1RWP0|F1RWP0	3	1	0	0	0	0	0	1	5	1	0	0	0	0	0	3	7.8%	4.4%	0.0%	0.0%	0.0%	0.0%	0.0%	3.6%
tr|F1S8M9|F1S8M9	5	0	6	0	0	0	1	3	6	0	21	0	0	0	1	12	9.3%	0.0%	24.8%	0.0%	0.0%	0.0%	2.3%	10.1%
tr|F2Z4Y0|F2Z4Y0	3	1	4	0	0	0	0	2	5	1	25	0	0	0	0	14	24.6%	7.9%	31.7%	0.0%	0.0%	0.0%	0.0%	16.7%
tr|F1RWS8|F1RWS8	6	0	1	0	0	0	1	1	6	0	1	0	0	0	2	1	36.5%	0.0%	4.3%	0.0%	0.0%	0.0%	13.7%	13.7%
tr|F1S3T0|F1S3T0	3	1	1	0	0	0	0	1	5	1	3	0	0	0	0	3	18.1%	4.0%	10.5%	0.0%	0.0%	0.0%	0.0%	10.9%
tr|F1RPL9|F1RPL9	1	1	0	0	0	0	1	1	2	4	0	0	0	0	1	1	5.5%	5.5%	0.0%	0.0%	0.0%	0.0%	5.5%	5.5%
Q2QLE0	3	2	0	0	0	0	1	0	4	2	0	0	0	0	2	0	4.0%	1.5%	0.0%	0.0%	0.0%	0.0%	1.5%	0.0%
tr|F1S306|F1S306	6	0	4	0	0	0	0	2	6	0	47	0	0	0	0	15	22.7%	0.0%	13.0%	0.0%	0.0%	0.0%	0.0%	8.8%
tr|F1RKZ6|F1RKZ6	2	3	5	0	0	0	1	2	2	4	14	0	0	0	2	3	3.8%	4.0%	6.4%	0.0%	0.0%	0.0%	2.9%	4.7%
tr|F2Z5J5|F2Z5J5	3	1	2	0	0	0	0	1	5	1	10	0	0	0	0	4	38.5%	23.1%	30.8%	0.0%	0.0%	0.0%	0.0%	7.7%
tr|F1SB44|F1SB44	2	1	2	0	0	0	2	0	3	3	17	0	0	0	5	0	30.4%	11.6%	30.4%	0.0%	0.0%	0.0%	37.5%	0.0%
tr|K7GSD8|K7GSD8	5	0	5	0	0	0	2	1	6	0	14	0	0	0	3	1	29.0%	0.0%	19.4%	0.0%	0.0%	0.0%	12.5%	7.2%
tr|I3LRG2|I3LRG2	5	0	5	0	0	0	2	1	6	0	14	0	0	0	3	1	24.0%	0.0%	16.0%	0.0%	0.0%	0.0%	10.4%	5.9%
tr|F1SFQ0|F1SFQ0	5	1	2	0	0	0	0	1	5	1	4	0	0	0	0	2	18.2%	3.2%	5.1%	0.0%	0.0%	0.0%	0.0%	5.1%
tr|I3LCY8|I3LCY8	1	0	0	0	0	0	0	1	6	0	0	0	0	0	0	1	3.2%	0.0%	0.0%	0.0%	0.0%	0.0%	0.0%	3.2%
tr|K7N7E5|K7N7E5	2	2	0	0	0	0	0	1	4	2	0	0	0	0	0	1	10.7%	5.7%	0.0%	0.0%	0.0%	0.0%	0.0%	5.0%
tr|F1RVA1|F1RVA1	1	1	0	0	0	0	0	1	4	2	0	0	0	0	0	1	14.4%	5.7%	0.0%	0.0%	0.0%	0.0%	0.0%	14.4%
tr|I3LAB3|I3LAB3	2	1	0	0	0	0	0	1	5	1	0	0	0	0	0	1	11.2%	6.9%	0.0%	0.0%	0.0%	0.0%	0.0%	6.9%
tr|I3L829|I3L829	5	0	0	0	0	0	0	1	6	0	0	0	0	0	0	1	13.5%	0.0%	0.0%	0.0%	0.0%	0.0%	0.0%	6.5%
tr|F1SKQ3|F1SKQ3	5	0	0	0	0	0	0	1	6	0	0	0	0	0	0	1	30.5%	0.0%	0.0%	0.0%	0.0%	0.0%	0.0%	6.5%
tr|F1SSV2|F1SSV2	5	0	5	0	0	0	0	2	6	0	15	0	0	0	0	3	31.0%	0.0%	24.4%	0.0%	0.0%	0.0%	0.0%	8.6%
tr|I3LTM8|I3LTM8	5	0	5	0	0	0	0	2	6	0	15	0	0	0	0	3	31.0%	0.0%	24.4%	0.0%	0.0%	0.0%	0.0%	8.6%
tr|I3LN65|I3LN65	2	1	1	0	0	0	0	1	5	1	2	0	0	0	0	1	19.7%	8.0%	5.9%	0.0%	0.0%	0.0%	0.0%	13.8%
tr|F1RF91|F1RF91	2	1	1	0	0	0	0	1	3	3	3	0	0	0	0	1	8.6%	3.4%	5.2%	0.0%	0.0%	0.0%	0.0%	14.5%
tr|F1SUP2|F1SUP2	2	2	5	0	0	0	0	2	3	3	16	0	0	0	0	2	11.9%	11.9%	24.8%	0.0%	0.0%	0.0%	0.0%	11.9%
tr|I3LRR4|I3LRR4	6	0	5	0	0	0	1	1	6	0	16	0	0	0	1	1	19.2%	0.0%	16.1%	0.0%	0.0%	0.0%	3.6%	3.4%
tr|I3L9R9|I3L9R9	4	1	4	0	0	0	0	1	5	1	5	0	0	0	0	1	6.2%	1.4%	4.8%	0.0%	0.0%	0.0%	0.0%	1.2%
tr|F1SJR4|F1SJR4	3	2	1	0	0	0	0	1	3	3	5	0	0	0	0	1	11.1%	4.8%	2.9%	0.0%	0.0%	0.0%	0.0%	4.8%
tr|F1S419|F1S419	2	2	2	0	0	0	0	1	2	4	7	0	0	0	0	1	13.2%	8.5%	10.3%	0.0%	0.0%	0.0%	0.0%	8.2%
tr|F1SAN6|F1SAN6	2	2	4	0	0	0	0	2	4	2	66	0	0	0	0	5	8.5%	5.4%	8.5%	0.0%	0.0%	0.0%	0.0%	8.3%
tr|F1SQK1|F1SQK1	2	1	4	0	0	0	0	1	4	2	13	0	0	0	0	1	4.8%	1.1%	12.1%	0.0%	0.0%	0.0%	0.0%	3.3%
tr|I3L9K5|I3L9K5	1	1	7	0	0	0	0	1	1	5	22	0	0	0	0	1	8.2%	4.5%	11.5%	0.0%	0.0%	0.0%	0.0%	4.5%
Q19QT7	3	0	0	0	0	0	0	0	6	0	0	0	0	0	0	0	14.3%	0.0%	0.0%	0.0%	0.0%	0.0%	0.0%	0.0%
tr|K7GRS5|K7GRS5	2	1	0	0	0	0	0	0	2	4	0	0	0	0	0	0	10.8%	3.7%	0.0%	0.0%	0.0%	0.0%	0.0%	0.0%
tr|I3L6Z8|I3L6Z8	1	2	0	0	0	0	0	0	1	5	0	0	0	0	0	0	10.4%	22.9%	0.0%	0.0%	0.0%	0.0%	0.0%	0.0%
tr|F1RJD0|F1RJD0	1	1	0	0	0	0	0	0	2	4	0	0	0	0	0	0	14.8%	6.7%	0.0%	0.0%	0.0%	0.0%	0.0%	0.0%
tr|F1RWV6|F1RWV6	1	1	0	0	0	0	0	0	1	5	0	0	0	0	0	0	2.4%	1.0%	0.0%	0.0%	0.0%	0.0%	0.0%	0.0%
tr|K7GRA6|K7GRA6	3	0	0	0	0	0	0	0	6	0	0	0	0	0	0	0	21.2%	0.0%	0.0%	0.0%	0.0%	0.0%	0.0%	0.0%
tr|F1RQU0|F1RQU0	0	1	0	0	0	0	0	0	0	6	0	0	0	0	0	0	0.0%	2.9%	0.0%	0.0%	0.0%	0.0%	0.0%	0.0%
tr|F1RGP5|F1RGP5	2	1	0	0	0	0	0	0	4	2	0	0	0	0	0	0	8.0%	1.7%	0.0%	0.0%	0.0%	0.0%	0.0%	0.0%
tr|F1SQH4|F1SQH4	2	2	0	0	0	0	0	0	3	3	0	0	0	0	0	0	6.9%	5.1%	0.0%	0.0%	0.0%	0.0%	0.0%	0.0%
tr|K7GLG2|K7GLG2	1	1	0	0	0	0	0	0	1	5	0	0	0	0	0	0	2.9%	1.2%	0.0%	0.0%	0.0%	0.0%	0.0%	0.0%
tr|F1SMG1|F1SMG1	3	0	0	0	0	0	0	0	6	0	0	0	0	0	0	0	14.7%	0.0%	0.0%	0.0%	0.0%	0.0%	0.0%	0.0%
tr|F1RIJ5|F1RIJ5	0	2	0	0	0	0	0	0	0	6	0	0	0	0	0	0	0.0%	8.2%	0.0%	0.0%	0.0%	0.0%	0.0%	0.0%
tr|F1SQY2|F1SQY2	3	1	0	0	0	0	0	0	5	1	0	0	0	0	0	0	18.6%	3.1%	0.0%	0.0%	0.0%	0.0%	0.0%	0.0%
tr|I3L6S9|I3L6S9	1	0	0	0	0	0	0	0	6	0	0	0	0	0	0	0	3.3%	0.0%	0.0%	0.0%	0.0%	0.0%	0.0%	0.0%
tr|F1RH59|F1RH59	4	1	0	0	0	0	0	0	5	1	0	0	0	0	0	0	14.8%	3.6%	0.0%	0.0%	0.0%	0.0%	0.0%	0.0%
tr|K7GLV4|K7GLV4	0	1	0	0	0	0	0	0	0	6	0	0	0	0	0	0	0.0%	3.3%	0.0%	0.0%	0.0%	0.0%	0.0%	0.0%
tr|F1RRP8|F1RRP8	2	0	0	0	0	0	0	0	6	0	0	0	0	0	0	0	18.3%	0.0%	0.0%	0.0%	0.0%	0.0%	0.0%	0.0%
tr|F1SLI2|F1SLI2	0	1	0	0	0	0	0	0	0	6	0	0	0	0	0	0	0.0%	6.2%	0.0%	0.0%	0.0%	0.0%	0.0%	0.0%
tr|F1SNF8|F1SNF8	4	1	0	0	0	0	0	0	5	1	0	0	0	0	0	0	3.9%	0.7%	0.0%	0.0%	0.0%	0.0%	0.0%	0.0%
tr|I3L7J7|I3L7J7	3	1	0	0	0	0	0	0	4	2	0	0	0	0	0	0	10.1%	3.8%	0.0%	0.0%	0.0%	0.0%	0.0%	0.0%
tr|I3LHS7|I3LHS7	3	1	0	0	0	0	0	0	5	1	0	0	0	0	0	0	20.4%	9.4%	0.0%	0.0%	0.0%	0.0%	0.0%	0.0%
tr|K7GPY8|K7GPY8	2	1	0	0	0	0	0	0	5	1	0	0	0	0	0	0	21.1%	8.7%	0.0%	0.0%	0.0%	0.0%	0.0%	0.0%
tr|I3LQE0|I3LQE0	3	0	0	0	0	0	0	0	6	0	0	0	0	0	0	0	12.0%	0.0%	0.0%	0.0%	0.0%	0.0%	0.0%	0.0%
tr|F1RN46|F1RN46	5	0	0	0	0	0	0	0	6	0	0	0	0	0	0	0	10.2%	0.0%	0.0%	0.0%	0.0%	0.0%	0.0%	0.0%
tr|I3LS10|I3LS10	6	0	0	0	0	0	0	0	6	0	0	0	0	0	0	0	20.6%	0.0%	0.0%	0.0%	0.0%	0.0%	0.0%	0.0%
tr|F1SCX5|F1SCX5	2	0	0	0	0	0	0	0	6	0	0	0	0	0	0	0	16.0%	0.0%	0.0%	0.0%	0.0%	0.0%	0.0%	0.0%
tr|I3LVR2|I3LVR2	1	1	0	0	0	0	0	0	5	1	0	0	0	0	0	0	23.9%	11.4%	0.0%	0.0%	0.0%	0.0%	0.0%	0.0%
tr|I3LK74|I3LK74	3	1	0	0	0	0	0	0	4	2	0	0	0	0	0	0	2.7%	0.9%	0.0%	0.0%	0.0%	0.0%	0.0%	0.0%
tr|A5GFZ4|A5GFZ4	4	1	0	0	0	0	0	0	4	2	0	0	0	0	0	0	24.2%	8.1%	0.0%	0.0%	0.0%	0.0%	0.0%	0.0%
tr|K7GL42|K7GL42	2	1	0	0	0	0	0	0	4	2	0	0	0	0	0	0	6.5%	1.4%	0.0%	0.0%	0.0%	0.0%	0.0%	0.0%
Q5BLZ2	5	0	0	0	0	0	0	0	6	0	0	0	0	0	0	0	28.0%	0.0%	0.0%	0.0%	0.0%	0.0%	0.0%	0.0%
tr|K7GP94|K7GP94	2	1	0	0	0	0	0	0	2	4	0	0	0	0	0	0	6.1%	3.1%	0.0%	0.0%	0.0%	0.0%	0.0%	0.0%
tr|F1SMM3|F1SMM3	1	1	0	0	0	0	0	0	1	5	0	0	0	0	0	0	0.6%	0.6%	0.0%	0.0%	0.0%	0.0%	0.0%	0.0%
tr|F1RX24|F1RX24	3	0	0	0	0	0	0	0	6	0	0	0	0	0	0	0	6.3%	0.0%	0.0%	0.0%	0.0%	0.0%	0.0%	0.0%
tr|F1SDW3|F1SDW3	3	0	0	0	0	0	0	0	6	0	0	0	0	0	0	0	30.1%	0.0%	0.0%	0.0%	0.0%	0.0%	0.0%	0.0%
tr|I3LAY1|I3LAY1	3	0	0	0	0	0	0	0	6	0	0	0	0	0	0	0	12.0%	0.0%	0.0%	0.0%	0.0%	0.0%	0.0%	0.0%
tr|I3LR44|I3LR44	3	0	0	0	0	0	0	0	6	0	0	0	0	0	0	0	19.4%	0.0%	0.0%	0.0%	0.0%	0.0%	0.0%	0.0%
tr|F1S9C9|F1S9C9	0	3	0	0	0	0	0	0	0	6	0	0	0	0	0	0	0.0%	17.1%	0.0%	0.0%	0.0%	0.0%	0.0%	0.0%
tr|I3LBV6|I3LBV6	4	1	0	0	0	0	0	0	5	1	0	0	0	0	0	0	6.8%	1.4%	0.0%	0.0%	0.0%	0.0%	0.0%	0.0%
tr|K7GQB6|K7GQB6	2	1	0	0	0	0	0	0	5	1	0	0	0	0	0	0	16.7%	6.9%	0.0%	0.0%	0.0%	0.0%	0.0%	0.0%
tr|F1SQL0|F1SQL0	1	1	0	0	0	0	0	0	1	5	0	0	0	0	0	0	6.8%	4.8%	0.0%	0.0%	0.0%	0.0%	0.0%	0.0%
tr|F1S069|F1S069	4	1	0	0	0	0	0	0	4	2	0	0	0	0	0	0	16.7%	5.5%	0.0%	0.0%	0.0%	0.0%	0.0%	0.0%
tr|K7GKJ7|K7GKJ7	2	1	0	0	0	0	0	0	4	2	0	0	0	0	0	0	8.3%	7.0%	0.0%	0.0%	0.0%	0.0%	0.0%	0.0%

	Peptide Count								Spectral Count								Sequence Coverage							
	Whole Cell	Whole Cell	Whole Cell	Cilia Fraction	Cilia Fraction	Cilia Fraction	Cilia Fraction	Cilia Fraction	Whole Cell	Whole Cell	Whole Cell	Cilia Fraction	Cilia Fraction	Cilia Fraction	Cilia Fraction	Cilia Fraction	Whole Cell	Whole Cell	Whole Cell	Cilia Fraction	Cilia Fraction	Cilia Fraction	Cilia Fraction	Cilia Fraction
Sample#	1	2	6	4	3	5	7	8	1	2	6	4	3	5	7	8	1	2	6	4	3	5	7	8
**Accession**																								
tr|I3LBT7|I3LBT7	0	1	0	0	0	0	0	0	0	6	0	0	0	0	0	0	0.0%	1.2%	0.0%	0.0%	0.0%	0.0%	0.0%	0.0%
tr|F1RWY7|F1RWY7	3	1	0	0	0	0	0	0	5	1	0	0	0	0	0	0	15.1%	3.4%	0.0%	0.0%	0.0%	0.0%	0.0%	0.0%
tr|K7GPJ6|K7GPJ6	3	0	0	0	0	0	0	0	6	0	0	0	0	0	0	0	7.2%	0.0%	0.0%	0.0%	0.0%	0.0%	0.0%	0.0%
tr|I3L590|I3L590	4	0	0	0	0	0	0	0	6	0	0	0	0	0	0	0	8.0%	0.0%	0.0%	0.0%	0.0%	0.0%	0.0%	0.0%
tr|F1RLL1|F1RLL1	3	0	0	0	0	0	0	0	6	0	0	0	0	0	0	0	8.3%	0.0%	0.0%	0.0%	0.0%	0.0%	0.0%	0.0%
tr|I3LLK1|I3LLK1	6	0	0	0	0	0	0	0	6	0	0	0	0	0	0	0	10.2%	0.0%	0.0%	0.0%	0.0%	0.0%	0.0%	0.0%
tr|C9K506|C9K506	1	1	0	0	0	0	0	0	2	4	0	0	0	0	0	0	8.0%	3.7%	0.0%	0.0%	0.0%	0.0%	0.0%	0.0%
tr|F1RMQ4|F1RMQ4	5	0	0	0	0	0	0	0	6	0	0	0	0	0	0	0	23.7%	0.0%	0.0%	0.0%	0.0%	0.0%	0.0%	0.0%
tr|I3LNI1|I3LNI1	1	1	0	0	0	0	0	0	3	3	0	0	0	0	0	0	3.2%	1.4%	0.0%	0.0%	0.0%	0.0%	0.0%	0.0%
Q2MJV9	4	0	0	0	0	0	0	0	6	0	0	0	0	0	0	0	9.8%	0.0%	0.0%	0.0%	0.0%	0.0%	0.0%	0.0%
tr|I3L8B1|I3L8B1	3	1	0	0	0	0	0	0	4	2	0	0	0	0	0	0	22.3%	4.5%	0.0%	0.0%	0.0%	0.0%	0.0%	0.0%
tr|F1RW16|F1RW16	2	1	0	0	0	0	0	0	2	4	0	0	0	0	0	0	5.6%	2.8%	0.0%	0.0%	0.0%	0.0%	0.0%	0.0%
tr|F1RLM4|F1RLM4	6	0	0	0	0	0	0	0	6	0	0	0	0	0	0	0	19.3%	0.0%	0.0%	0.0%	0.0%	0.0%	0.0%	0.0%
tr|I3LR07|I3LR07	5	0	0	0	0	0	0	0	6	0	0	0	0	0	0	0	7.4%	0.0%	0.0%	0.0%	0.0%	0.0%	0.0%	0.0%
tr|F1SJL5|F1SJL5	5	1	0	0	0	0	0	0	5	1	0	0	0	0	0	0	9.9%	1.9%	0.0%	0.0%	0.0%	0.0%	0.0%	0.0%
tr|I3LQG8|I3LQG8	2	1	1	0	0	0	0	0	3	3	1	0	0	0	0	0	13.5%	5.5%	9.0%	0.0%	0.0%	0.0%	0.0%	0.0%
tr|F1RS43|F1RS43	3	1	1	0	0	0	0	0	5	1	1	0	0	0	0	0	22.0%	8.1%	8.1%	0.0%	0.0%	0.0%	0.0%	0.0%
tr|F1SQG5|F1SQG5	3	2	1	0	0	0	0	0	4	2	2	0	0	0	0	0	14.0%	5.7%	2.6%	0.0%	0.0%	0.0%	0.0%	0.0%
tr|F1S399|F1S399	4	1	1	0	0	0	0	0	5	1	2	0	0	0	0	0	10.6%	3.4%	4.0%	0.0%	0.0%	0.0%	0.0%	0.0%
tr|F1RY63|F1RY63	3	2	1	0	0	0	0	0	3	3	2	0	0	0	0	0	7.3%	2.8%	1.9%	0.0%	0.0%	0.0%	0.0%	0.0%
tr|F1SH64|F1SH64	3	1	1	0	0	0	0	0	3	3	2	0	0	0	0	0	5.1%	3.3%	3.4%	0.0%	0.0%	0.0%	0.0%	0.0%
tr|I3LCL0|I3LCL0	3	0	1	0	0	0	0	0	6	0	2	0	0	0	0	0	8.7%	0.0%	2.3%	0.0%	0.0%	0.0%	0.0%	0.0%
tr|I3LJ79|I3LJ79	6	0	1	0	0	0	0	0	6	0	2	0	0	0	0	0	16.5%	0.0%	5.6%	0.0%	0.0%	0.0%	0.0%	0.0%
tr|F1S5Q3|F1S5Q3	2	1	1	0	0	0	0	0	2	4	2	0	0	0	0	0	8.0%	3.1%	2.1%	0.0%	0.0%	0.0%	0.0%	0.0%
tr|F1S5V0|F1S5V0	4	0	1	0	0	0	0	0	6	0	3	0	0	0	0	0	15.2%	0.0%	4.9%	0.0%	0.0%	0.0%	0.0%	0.0%
P80895	2	1	1	0	0	0	0	0	4	2	3	0	0	0	0	0	23.3%	7.5%	4.8%	0.0%	0.0%	0.0%	0.0%	0.0%
tr|K7GN85|K7GN85	3	0	1	0	0	0	0	0	6	0	3	0	0	0	0	0	29.5%	0.0%	13.1%	0.0%	0.0%	0.0%	0.0%	0.0%
tr|K7GSN3|K7GSN3	3	0	1	0	0	0	0	0	6	0	3	0	0	0	0	0	28.3%	0.0%	12.5%	0.0%	0.0%	0.0%	0.0%	0.0%
tr|F1RG04|F1RG04	4	1	1	0	0	0	0	0	5	1	3	0	0	0	0	0	9.4%	1.9%	3.1%	0.0%	0.0%	0.0%	0.0%	0.0%
tr|F1RZC1|F1RZC1	3	2	1	0	0	0	0	0	3	3	3	0	0	0	0	0	1.8%	1.3%	0.9%	0.0%	0.0%	0.0%	0.0%	0.0%
tr|J9JIK8|J9JIK8	2	1	1	0	0	0	0	0	4	2	3	0	0	0	0	0	25.5%	8.2%	5.3%	0.0%	0.0%	0.0%	0.0%	0.0%
tr|F1RRN2|F1RRN2	0	1	2	0	0	0	0	0	0	6	3	0	0	0	0	0	0.0%	2.9%	12.4%	0.0%	0.0%	0.0%	0.0%	0.0%
tr|F1SI83|F1SI83	4	1	2	0	0	0	0	0	5	1	4	0	0	0	0	0	53.8%	13.6%	19.7%	0.0%	0.0%	0.0%	0.0%	0.0%
tr|F1SHU0|F1SHU0	3	0	1	0	0	0	0	0	6	0	4	0	0	0	0	0	21.1%	0.0%	7.4%	0.0%	0.0%	0.0%	0.0%	0.0%
tr|K7GRT4|K7GRT4	1	1	3	0	0	0	0	0	5	1	6	0	0	0	0	0	14.9%	11.9%	11.3%	0.0%	0.0%	0.0%	0.0%	0.0%
Q007T0	2	2	2	0	0	0	0	0	3	3	6	0	0	0	0	0	16.4%	8.6%	9.6%	0.0%	0.0%	0.0%	0.0%	0.0%
tr|I3LDC1|I3LDC1	2	2	2	0	0	0	0	0	3	3	6	0	0	0	0	0	16.4%	8.6%	9.6%	0.0%	0.0%	0.0%	0.0%	0.0%
tr|F1RSL4|F1RSL4	2	2	1	0	0	0	0	0	2	4	6	0	0	0	0	0	3.2%	2.0%	1.4%	0.0%	0.0%	0.0%	0.0%	0.0%
tr|I3LCB9|I3LCB9	2	1	3	0	0	0	0	0	5	1	6	0	0	0	0	0	17.1%	17.1%	17.1%	0.0%	0.0%	0.0%	0.0%	0.0%
tr|K7GMG1|K7GMG1	1	1	3	0	0	0	0	0	5	1	6	0	0	0	0	0	11.9%	9.5%	9.0%	0.0%	0.0%	0.0%	0.0%	0.0%
tr|F1S4P0|F1S4P0	2	0	2	0	0	0	0	0	6	0	6	0	0	0	0	0	8.1%	0.0%	4.1%	0.0%	0.0%	0.0%	0.0%	0.0%
tr|F1RQI1|F1RQI1	4	1	2	0	0	0	0	0	4	2	6	0	0	0	0	0	30.1%	28.9%	27.7%	0.0%	0.0%	0.0%	0.0%	0.0%
Q9MYY8	2	3	2	0	0	0	0	0	2	4	7	0	0	0	0	0	6.4%	6.0%	6.0%	0.0%	0.0%	0.0%	0.0%	0.0%
tr|K7GR33|K7GR33	1	2	2	0	0	0	0	0	3	3	8	0	0	0	0	0	7.2%	6.0%	13.2%	0.0%	0.0%	0.0%	0.0%	0.0%
tr|I3LJY1|I3LJY1	3	1	3	0	0	0	0	0	4	2	10	0	0	0	0	0	28.2%	8.5%	28.2%	0.0%	0.0%	0.0%	0.0%	0.0%
tr|F1RZ24|F1RZ24	5	0	4	0	0	0	0	0	6	0	10	0	0	0	0	0	13.2%	0.0%	10.7%	0.0%	0.0%	0.0%	0.0%	0.0%
tr|F1RU12|F1RU12	3	0	3	0	0	0	0	0	6	0	14	0	0	0	0	0	14.6%	0.0%	14.2%	0.0%	0.0%	0.0%	0.0%	0.0%
tr|I3LQ34|I3LQ34	3	0	6	0	0	0	0	0	6	0	22	0	0	0	0	0	30.8%	0.0%	32.7%	0.0%	0.0%	0.0%	0.0%	0.0%
tr|I3LLA8|I3LLA8	2	1	2	0	0	0	0	0	2	4	23	0	0	0	0	0	5.1%	1.8%	3.4%	0.0%	0.0%	0.0%	0.0%	0.0%
tr|F1SK41|F1SK41	1	2	2	0	0	0	3	2	3	2	17	0	0	0	78	26	3.6%	9.5%	6.9%	0.0%	0.0%	0.0%	9.5%	9.5%
tr|F1RYM8|F1RYM8	3	0	0	0	0	0	0	6	5	0	0	0	0	0	0	21	12.3%	0.0%	0.0%	0.0%	0.0%	0.0%	0.0%	33.6%
tr|I3L674|I3L674	1	0	0	0	0	0	1	1	5	0	0	0	0	0	14	2	18.9%	0.0%	0.0%	0.0%	0.0%	0.0%	18.9%	18.9%
tr|Q9N0Y9|Q9N0Y9	4	0	2	0	0	0	2	2	5	0	3	0	0	0	13	12	24.1%	0.0%	5.1%	0.0%	0.0%	0.0%	12.2%	11.9%
tr|F1RS33|F1RS33	1	0	0	0	0	0	0	1	5	0	0	0	0	0	0	14	53.8%	0.0%	0.0%	0.0%	0.0%	0.0%	0.0%	53.8%
tr|F1SV26|F1SV26	0	1	1	0	0	0	2	1	0	5	3	0	0	0	15	3	0.0%	2.3%	3.5%	0.0%	0.0%	0.0%	8.8%	3.5%
tr|F1RW78|F1RW78	2	1	0	0	0	0	1	1	4	1	0	0	0	0	8	2	8.1%	2.8%	0.0%	0.0%	0.0%	0.0%	5.3%	5.3%
tr|I3LIP0|I3LIP0	2	0	1	0	0	0	1	2	5	0	6	0	0	0	6	14	52.2%	0.0%	14.5%	0.0%	0.0%	0.0%	14.5%	30.4%
tr|F1SJR5|F1SJR5	1	0	6	0	0	0	1	3	5	0	37	0	0	0	33	42	4.7%	0.0%	17.5%	0.0%	0.0%	0.0%	7.0%	14.0%
Q5ISC6	3	1	3	0	0	0	4	2	4	1	15	0	0	0	26	7	29.1%	7.9%	21.2%	0.0%	0.0%	0.0%	38.1%	20.6%
tr|K7GNH6|K7GNH6	1	1	4	0	0	0	1	1	4	1	43	0	0	0	24	42	32.1%	32.1%	32.1%	0.0%	0.0%	0.0%	32.1%	32.1%
tr|A5GHK8|A5GHK8	2	1	1	0	0	0	0	2	2	3	3	0	0	0	0	10	5.4%	2.2%	3.2%	0.0%	0.0%	0.0%	0.0%	7.5%
P61958	3	0	3	0	0	0	2	2	5	0	18	0	0	0	11	13	27.4%	0.0%	27.4%	0.0%	0.0%	0.0%	50.5%	50.5%
tr|F1S6H6|F1S6H6	2	1	1	0	0	0	0	3	2	3	3	0	0	0	0	8	4.3%	2.1%	3.9%	0.0%	0.0%	0.0%	0.0%	6.2%
tr|I3LCA1|I3LCA1	0	2	0	0	0	0	0	1	0	5	0	0	0	0	0	5	0.0%	3.3%	0.0%	0.0%	0.0%	0.0%	0.0%	1.5%
tr|F1S7L6|F1S7L6	3	0	1	0	0	0	0	2	5	0	5	0	0	0	0	9	14.5%	0.0%	4.3%	0.0%	0.0%	0.0%	0.0%	12.2%
tr|I3LF54|I3LF54	2	0	0	0	0	0	0	2	5	0	0	0	0	0	0	4	9.8%	0.0%	0.0%	0.0%	0.0%	0.0%	0.0%	10.5%
tr|F1SC70|F1SC70	3	1	0	0	0	0	0	2	3	2	0	0	0	0	0	4	6.1%	2.2%	0.0%	0.0%	0.0%	0.0%	0.0%	3.8%
tr|I3LRP5|I3LRP5	1	1	0	0	0	0	0	1	4	1	0	0	0	0	0	4	24.8%	24.8%	0.0%	0.0%	0.0%	0.0%	0.0%	24.8%
tr|F1SDR3|F1SDR3	1	0	0	0	0	0	1	1	5	0	0	0	0	0	1	3	6.6%	0.0%	0.0%	0.0%	0.0%	0.0%	6.6%	6.6%
Q0MVN8	4	0	2	0	0	0	0	2	5	0	9	0	0	0	0	10	24.3%	0.0%	5.8%	0.0%	0.0%	0.0%	0.0%	11.6%
tr|Q19QT8|Q19QT8	4	0	2	0	0	0	0	2	5	0	9	0	0	0	0	10	24.2%	0.0%	5.8%	0.0%	0.0%	0.0%	0.0%	11.5%
tr|F1RIP6|F1RIP6	4	0	5	0	0	0	0	7	5	0	17	0	0	0	0	15	14.0%	0.0%	7.9%	0.0%	0.0%	0.0%	0.0%	18.8%
P13618	3	0	8	0	0	0	1	1	5	0	89	0	0	0	14	35	63.2%	0.0%	63.2%	0.0%	0.0%	0.0%	31.6%	31.6%
tr|I3L6G6|I3L6G6	3	0	8	0	0	0	1	1	5	0	89	0	0	0	14	35	44.4%	0.0%	44.4%	0.0%	0.0%	0.0%	22.2%	22.2%
tr|K7GMR9|K7GMR9	2	1	0	0	0	0	0	1	3	2	0	0	0	0	0	2	5.0%	2.5%	0.0%	0.0%	0.0%	0.0%	0.0%	2.1%
Q4LE85	1	2	0	0	0	0	1	1	1	4	0	0	0	0	1	1	5.9%	17.6%	0.0%	0.0%	0.0%	0.0%	4.5%	5.9%
tr|F1RPE0|F1RPE0	2	0	0	0	0	0	1	1	5	0	0	0	0	0	1	1	2.6%	0.0%	0.0%	0.0%	0.0%	0.0%	1.7%	1.7%
tr|F1SFR1|F1SFR1	4	0	0	0	0	0	1	0	5	0	0	0	0	0	2	0	17.7%	0.0%	0.0%	0.0%	0.0%	0.0%	5.5%	0.0%
tr|K7GMZ7|K7GMZ7	1	1	0	0	0	0	0	1	4	1	0	0	0	0	0	2	27.0%	9.0%	0.0%	0.0%	0.0%	0.0%	0.0%	27.0%
tr|I3LLW0|I3LLW0	3	0	0	0	0	0	0	1	5	0	0	0	0	0	0	2	4.1%	0.0%	0.0%	0.0%	0.0%	0.0%	0.0%	1.5%
tr|I3LBP7|I3LBP7	4	0	2	0	0	0	0	1	5	0	4	0	0	0	0	3	24.9%	0.0%	6.7%	0.0%	0.0%	0.0%	0.0%	11.5%

	Peptide Count								Spectral Count								Sequence Coverage							
	Whole Cell	Whole Cell	Whole Cell	Cilia Fraction	Cilia Fraction	Cilia Fraction	Cilia Fraction	Cilia Fraction	Whole Cell	Whole Cell	Whole Cell	Cilia Fraction	Cilia Fraction	Cilia Fraction	Cilia Fraction	Cilia Fraction	Whole Cell	Whole Cell	Whole Cell	Cilia Fraction	Cilia Fraction	Cilia Fraction	Cilia Fraction	Cilia Fraction
Sample#	1	2	6	4	3	5	7	8	1	2	6	4	3	5	7	8	1	2	6	4	3	5	7	8
**Accession**																								
tr|F1RY67|F1RY67	2	1	3	0	0	0	0	2	3	2	8	0	0	0	0	4	7.8%	2.6%	8.8%	0.0%	0.0%	0.0%	0.0%	8.8%
O97763	2	0	1	0	0	0	0	1	5	0	34	0	0	0	0	11	17.4%	0.0%	13.4%	0.0%	0.0%	0.0%	0.0%	10.7%
tr|F1SAA5|F1SAA5	4	0	1	0	0	0	0	1	5	0	6	0	0	0	0	3	18.3%	0.0%	9.5%	0.0%	0.0%	0.0%	0.0%	9.5%
Q06AT3	4	0	2	0	0	0	0	1	5	0	8	0	0	0	0	3	14.0%	0.0%	12.2%	0.0%	0.0%	0.0%	0.0%	7.3%
tr|F1SAA9|F1SAA9	4	0	2	0	0	0	0	1	5	0	8	0	0	0	0	3	14.7%	0.0%	12.8%	0.0%	0.0%	0.0%	0.0%	7.7%
tr|I3LAD5|I3LAD5	3	0	1	0	0	0	0	1	5	0	4	0	0	0	0	2	12.3%	0.0%	3.0%	0.0%	0.0%	0.0%	0.0%	4.5%
tr|F1RWA6|F1RWA6	3	0	1	0	0	0	0	1	5	0	4	0	0	0	0	2	14.2%	0.0%	3.5%	0.0%	0.0%	0.0%	0.0%	5.2%
tr|F1S596|F1S596	4	0	9	0	0	0	1	2	5	0	67	0	0	0	4	11	15.8%	0.0%	27.4%	0.0%	0.0%	0.0%	7.1%	7.1%
tr|F1SH94|F1SH94	1	1	0	0	0	0	0	1	1	4	0	0	0	0	0	1	3.0%	1.4%	0.0%	0.0%	0.0%	0.0%	0.0%	1.0%
tr|F1S7K5|F1S7K5	1	2	0	0	0	0	0	1	2	3	0	0	0	0	0	1	1.2%	2.8%	0.0%	0.0%	0.0%	0.0%	0.0%	1.2%
tr|F1RN87|F1RN87	3	1	0	0	0	0	0	1	4	1	0	0	0	0	0	1	8.8%	2.0%	0.0%	0.0%	0.0%	0.0%	0.0%	3.3%
tr|K7GS62|K7GS62	2	0	0	0	0	0	1	0	5	0	0	0	0	0	1	0	22.4%	0.0%	0.0%	0.0%	0.0%	0.0%	10.9%	0.0%
tr|I3LA44|I3LA44	4	0	0	0	0	0	0	1	5	0	0	0	0	0	0	1	9.3%	0.0%	0.0%	0.0%	0.0%	0.0%	0.0%	3.1%
tr|F1S021|F1S021	1	1	0	0	0	0	0	1	1	4	0	0	0	0	0	1	1.2%	0.6%	0.0%	0.0%	0.0%	0.0%	0.0%	1.3%
tr|F1RZT2|F1RZT2	3	0	0	0	0	0	0	1	5	0	0	0	0	0	0	1	30.2%	0.0%	0.0%	0.0%	0.0%	0.0%	0.0%	8.5%
tr|F1RFH9|F1RFH9	2	3	0	0	0	0	1	0	2	3	0	0	0	0	1	0	3.1%	3.2%	0.0%	0.0%	0.0%	0.0%	1.5%	0.0%
tr|F1ST54|F1ST54	1	1	0	0	0	0	0	1	2	3	0	0	0	0	0	1	1.7%	1.7%	0.0%	0.0%	0.0%	0.0%	0.0%	1.7%
Q9TDR1	3	1	0	0	0	0	0	1	4	1	0	0	0	0	0	1	5.1%	2.0%	0.0%	0.0%	0.0%	0.0%	0.0%	2.0%
tr|F1STF7|F1STF7	2	2	0	0	0	0	0	1	2	3	0	0	0	0	0	1	18.3%	11.2%	0.0%	0.0%	0.0%	0.0%	0.0%	10.0%
tr|I3L6I7|I3L6I7	1	1	0	0	0	0	0	1	2	3	0	0	0	0	0	1	14.4%	6.4%	0.0%	0.0%	0.0%	0.0%	0.0%	14.4%
tr|I3LK62|I3LK62	1	1	0	0	0	0	0	1	2	3	0	0	0	0	0	1	3.6%	3.6%	0.0%	0.0%	0.0%	0.0%	0.0%	3.6%
tr|F1SF51|F1SF51	0	1	0	0	0	0	1	0	0	5	0	0	0	0	1	0	0.0%	2.8%	0.0%	0.0%	0.0%	0.0%	3.3%	0.0%
tr|Q197W4|Q197W4	2	0	1	0	0	0	0	1	5	0	7	0	0	0	0	2	11.0%	0.0%	6.2%	0.0%	0.0%	0.0%	0.0%	7.5%
tr|F1SP27|F1SP27	3	1	1	0	0	0	0	1	3	2	1	0	0	0	0	1	5.0%	0.8%	1.5%	0.0%	0.0%	0.0%	0.0%	3.3%
tr|F1RMD6|F1RMD6	1	2	1	0	0	0	0	1	1	4	1	0	0	0	0	1	3.9%	6.2%	3.9%	0.0%	0.0%	0.0%	0.0%	2.6%
tr|F1SKN6|F1SKN6	3	0	2	0	0	0	0	1	5	0	4	0	0	0	0	1	5.7%	0.0%	7.9%	0.0%	0.0%	0.0%	0.0%	1.7%
tr|F1SR21|F1SR21	2	0	3	0	0	0	1	1	5	0	14	0	0	0	1	1	4.1%	0.0%	5.9%	0.0%	0.0%	0.0%	3.3%	3.3%
tr|F1S4R5|F1S4R5	4	0	4	0	0	0	0	3	5	0	60	0	0	0	0	6	11.9%	0.0%	14.2%	0.0%	0.0%	0.0%	0.0%	11.9%
tr|F1SMK2|F1SMK2	3	0	2	0	0	0	0	2	5	0	17	0	0	0	0	2	13.3%	0.0%	5.7%	0.0%	0.0%	0.0%	0.0%	9.1%
A9YUB1	4	0	3	0	0	0	0	1	5	0	12	0	0	0	0	1	16.9%	0.0%	17.2%	0.0%	0.0%	0.0%	0.0%	4.8%
tr|F1SBQ7|F1SBQ7	4	1	5	0	0	0	0	1	4	1	14	0	0	0	0	1	4.7%	0.6%	4.1%	0.0%	0.0%	0.0%	0.0%	1.3%
tr|F1SMR3|F1SMR3	2	1	1	0	0	0	0	1	3	2	18	0	0	0	0	1	6.3%	3.0%	4.5%	0.0%	0.0%	0.0%	0.0%	2.4%
tr|I3LPW0|I3LPW0	2	1	4	0	0	0	0	1	3	2	37	0	0	0	0	1	25.6%	10.1%	32.6%	0.0%	0.0%	0.0%	0.0%	10.1%
A2TLM1	4	0	0	0	0	0	0	0	5	0	0	0	0	0	0	0	12.1%	0.0%	0.0%	0.0%	0.0%	0.0%	0.0%	0.0%
tr|D7RA29|D7RA29	3	0	0	0	0	0	0	0	5	0	0	0	0	0	0	0	21.6%	0.0%	0.0%	0.0%	0.0%	0.0%	0.0%	0.0%
tr|K7GPH1|K7GPH1	1	0	0	0	0	0	0	0	5	0	0	0	0	0	0	0	18.2%	0.0%	0.0%	0.0%	0.0%	0.0%	0.0%	0.0%
tr|I3LKG9|I3LKG9	4	1	0	0	0	0	0	0	4	1	0	0	0	0	0	0	7.8%	1.2%	0.0%	0.0%	0.0%	0.0%	0.0%	0.0%
tr|F1RK56|F1RK56	1	1	0	0	0	0	0	0	1	4	0	0	0	0	0	0	5.4%	5.4%	0.0%	0.0%	0.0%	0.0%	0.0%	0.0%
tr|F1SRT1|F1SRT1	2	1	0	0	0	0	0	0	2	3	0	0	0	0	0	0	23.5%	8.7%	0.0%	0.0%	0.0%	0.0%	0.0%	0.0%
tr|F1SBB8|F1SBB8	0	1	0	0	0	0	0	0	0	5	0	0	0	0	0	0	0.0%	9.0%	0.0%	0.0%	0.0%	0.0%	0.0%	0.0%
Q95JC8	3	1	0	0	0	0	0	0	4	1	0	0	0	0	0	0	16.5%	5.3%	0.0%	0.0%	0.0%	0.0%	0.0%	0.0%
tr|F1S663|F1S663	4	0	0	0	0	0	0	0	5	0	0	0	0	0	0	0	4.8%	0.0%	0.0%	0.0%	0.0%	0.0%	0.0%	0.0%
tr|F1SV16|F1SV16	1	2	0	0	0	0	0	0	1	4	0	0	0	0	0	0	4.7%	9.0%	0.0%	0.0%	0.0%	0.0%	0.0%	0.0%
tr|K7GQI5|K7GQI5	2	0	0	0	0	0	0	0	5	0	0	0	0	0	0	0	14.8%	0.0%	0.0%	0.0%	0.0%	0.0%	0.0%	0.0%
tr|F1RT00|F1RT00	2	1	0	0	0	0	0	0	3	2	0	0	0	0	0	0	18.5%	5.6%	0.0%	0.0%	0.0%	0.0%	0.0%	0.0%
tr|F1SHF6|F1SHF6	0	2	0	0	0	0	0	0	0	5	0	0	0	0	0	0	0.0%	0.7%	0.0%	0.0%	0.0%	0.0%	0.0%	0.0%
tr|F1SQ06|F1SQ06	1	1	0	0	0	0	0	0	4	1	0	0	0	0	0	0	11.1%	11.1%	0.0%	0.0%	0.0%	0.0%	0.0%	0.0%
tr|F1RNI5|F1RNI5	1	0	0	0	0	0	0	0	5	0	0	0	0	0	0	0	41.7%	0.0%	0.0%	0.0%	0.0%	0.0%	0.0%	0.0%
tr|F1S3V9|F1S3V9	5	0	0	0	0	0	0	0	5	0	0	0	0	0	0	0	5.3%	0.0%	0.0%	0.0%	0.0%	0.0%	0.0%	0.0%
tr|K7GSZ3|K7GSZ3	2	0	0	0	0	0	0	0	5	0	0	0	0	0	0	0	5.0%	0.0%	0.0%	0.0%	0.0%	0.0%	0.0%	0.0%
tr|F1S842|F1S842	5	0	0	0	0	0	0	0	5	0	0	0	0	0	0	0	5.3%	0.0%	0.0%	0.0%	0.0%	0.0%	0.0%	0.0%
tr|F1SUQ0|F1SUQ0	0	1	0	0	0	0	0	0	0	5	0	0	0	0	0	0	0.0%	3.0%	0.0%	0.0%	0.0%	0.0%	0.0%	0.0%
P41566	2	1	0	0	0	0	0	0	4	1	0	0	0	0	0	0	31.1%	12.3%	0.0%	0.0%	0.0%	0.0%	0.0%	0.0%
tr|I3LA81|I3LA81	1	2	0	0	0	0	0	0	2	3	0	0	0	0	0	0	9.8%	18.9%	0.0%	0.0%	0.0%	0.0%	0.0%	0.0%
tr|F1SDB8|F1SDB8	1	2	0	0	0	0	0	0	1	4	0	0	0	0	0	0	1.5%	2.3%	0.0%	0.0%	0.0%	0.0%	0.0%	0.0%
tr|F1SBP5|F1SBP5	3	0	0	0	0	0	0	0	5	0	0	0	0	0	0	0	6.7%	0.0%	0.0%	0.0%	0.0%	0.0%	0.0%	0.0%
tr|I3LVJ9|I3LVJ9	1	2	0	0	0	0	0	0	1	4	0	0	0	0	0	0	12.8%	18.0%	0.0%	0.0%	0.0%	0.0%	0.0%	0.0%
tr|F1SD78|F1SD78	2	0	0	0	0	0	0	0	5	0	0	0	0	0	0	0	14.8%	0.0%	0.0%	0.0%	0.0%	0.0%	0.0%	0.0%
tr|I3L812|I3L812	1	1	0	0	0	0	0	0	4	1	0	0	0	0	0	0	2.4%	2.4%	0.0%	0.0%	0.0%	0.0%	0.0%	0.0%
tr|I3L997|I3L997	3	0	0	0	0	0	0	0	5	0	0	0	0	0	0	0	18.8%	0.0%	0.0%	0.0%	0.0%	0.0%	0.0%	0.0%
tr|I3LKA7|I3LKA7	2	0	0	0	0	0	0	0	5	0	0	0	0	0	0	0	9.0%	0.0%	0.0%	0.0%	0.0%	0.0%	0.0%	0.0%
tr|F1RPI9|F1RPI9	0	1	0	0	0	0	0	0	0	5	0	0	0	0	0	0	0.0%	1.9%	0.0%	0.0%	0.0%	0.0%	0.0%	0.0%
tr|Q0KKC2|Q0KKC2	1	1	0	0	0	0	0	0	2	3	0	0	0	0	0	0	3.8%	1.8%	0.0%	0.0%	0.0%	0.0%	0.0%	0.0%
O79875	0	3	0	0	0	0	0	0	0	5	0	0	0	0	0	0	0.0%	6.6%	0.0%	0.0%	0.0%	0.0%	0.0%	0.0%
tr|I3LLB7|I3LLB7	2	0	0	0	0	0	0	0	5	0	0	0	0	0	0	0	3.3%	0.0%	0.0%	0.0%	0.0%	0.0%	0.0%	0.0%
tr|I3L9Y2|I3L9Y2	4	0	0	0	0	0	0	0	5	0	0	0	0	0	0	0	6.4%	0.0%	0.0%	0.0%	0.0%	0.0%	0.0%	0.0%
tr|F1RYL5|F1RYL5	4	1	0	0	0	0	0	0	4	1	0	0	0	0	0	0	7.2%	2.1%	0.0%	0.0%	0.0%	0.0%	0.0%	0.0%
tr|F1RK95|F1RK95	0	1	0	0	0	0	0	0	0	5	0	0	0	0	0	0	0.0%	0.9%	0.0%	0.0%	0.0%	0.0%	0.0%	0.0%
tr|F1SEH4|F1SEH4	2	0	0	0	0	0	0	0	5	0	0	0	0	0	0	0	8.6%	0.0%	0.0%	0.0%	0.0%	0.0%	0.0%	0.0%
tr|F1S8T6|F1S8T6	4	0	0	0	0	0	0	0	5	0	0	0	0	0	0	0	8.7%	0.0%	0.0%	0.0%	0.0%	0.0%	0.0%	0.0%
tr|F1S843|F1S843	5	0	0	0	0	0	0	0	5	0	0	0	0	0	0	0	5.3%	0.0%	0.0%	0.0%	0.0%	0.0%	0.0%	0.0%
tr|I3LC31|I3LC31	2	2	0	0	0	0	0	0	2	3	0	0	0	0	0	0	6.6%	5.2%	0.0%	0.0%	0.0%	0.0%	0.0%	0.0%
tr|F1SKR9|F1SKR9	2	0	0	0	0	0	0	0	5	0	0	0	0	0	0	0	4.4%	0.0%	0.0%	0.0%	0.0%	0.0%	0.0%	0.0%
tr|K7GT04|K7GT04	1	1	0	0	0	0	0	0	1	4	0	0	0	0	0	0	3.4%	3.4%	0.0%	0.0%	0.0%	0.0%	0.0%	0.0%
tr|F1RKK5|F1RKK5	4	0	0	0	0	0	0	0	5	0	0	0	0	0	0	0	11.9%	0.0%	0.0%	0.0%	0.0%	0.0%	0.0%	0.0%
tr|F1RMW4|F1RMW4	4	1	0	0	0	0	0	0	4	1	0	0	0	0	0	0	10.6%	1.5%	0.0%	0.0%	0.0%	0.0%	0.0%	0.0%
tr|K7GME0|K7GME0	1	0	0	0	0	0	0	0	5	0	0	0	0	0	0	0	35.2%	0.0%	0.0%	0.0%	0.0%	0.0%	0.0%	0.0%
tr|F1RJE7|F1RJE7	0	2	0	0	0	0	0	0	0	5	0	0	0	0	0	0	0.0%	12.7%	0.0%	0.0%	0.0%	0.0%	0.0%	0.0%
tr|I3LAA7|I3LAA7	3	0	0	0	0	0	0	0	5	0	0	0	0	0	0	0	14.2%	0.0%	0.0%	0.0%	0.0%	0.0%	0.0%	0.0%
tr|I3LCA9|I3LCA9	4	0	0	0	0	0	0	0	5	0	0	0	0	0	0	0	20.4%	0.0%	0.0%	0.0%	0.0%	0.0%	0.0%	0.0%
tr|F1SSF8|F1SSF8	3	0	0	0	0	0	0	0	5	0	0	0	0	0	0	0	11.9%	0.0%	0.0%	0.0%	0.0%	0.0%	0.0%	0.0%

	Peptide Count								Spectral Count								Sequence Coverage							
	Whole Cell	Whole Cell	Whole Cell	Cilia Fraction	Cilia Fraction	Cilia Fraction	Cilia Fraction	Cilia Fraction	Whole Cell	Whole Cell	Whole Cell	Cilia Fraction	Cilia Fraction	Cilia Fraction	Cilia Fraction	Cilia Fraction	Whole Cell	Whole Cell	Whole Cell	Cilia Fraction	Cilia Fraction	Cilia Fraction	Cilia Fraction	Cilia Fraction
Sample#	1	2	6	4	3	5	7	8	1	2	6	4	3	5	7	8	1	2	6	4	3	5	7	8
**Accession**																								
D3K5L7	2	1	0	0	0	0	0	0	4	1	0	0	0	0	0	0	6.3%	3.7%	0.0%	0.0%	0.0%	0.0%	0.0%	0.0%
tr|I3LQ20|I3LQ20	2	1	0	0	0	0	0	0	2	3	0	0	0	0	0	0	31.1%	9.7%	0.0%	0.0%	0.0%	0.0%	0.0%	0.0%
tr|I3L8G2|I3L8G2	0	2	0	0	0	0	0	0	0	5	0	0	0	0	0	0	0.0%	5.0%	0.0%	0.0%	0.0%	0.0%	0.0%	0.0%
tr|F1RYV4|F1RYV4	0	1	0	0	0	0	0	0	0	5	0	0	0	0	0	0	0.0%	9.0%	0.0%	0.0%	0.0%	0.0%	0.0%	0.0%
tr|F1RIT6|F1RIT6	3	1	0	0	0	0	0	0	4	1	0	0	0	0	0	0	6.0%	2.1%	0.0%	0.0%	0.0%	0.0%	0.0%	0.0%
tr|F1SJA3|F1SJA3	2	0	0	0	0	0	0	0	5	0	0	0	0	0	0	0	8.2%	0.0%	0.0%	0.0%	0.0%	0.0%	0.0%	0.0%
D0VWV4	2	1	0	0	0	0	0	0	4	1	0	0	0	0	0	0	11.8%	9.5%	0.0%	0.0%	0.0%	0.0%	0.0%	0.0%
tr|I3LJT9|I3LJT9	2	0	0	0	0	0	0	0	5	0	0	0	0	0	0	0	9.9%	0.0%	0.0%	0.0%	0.0%	0.0%	0.0%	0.0%
tr|I3LFQ0|I3LFQ0	0	1	0	0	0	0	0	0	0	5	0	0	0	0	0	0	0.0%	4.6%	0.0%	0.0%	0.0%	0.0%	0.0%	0.0%
tr|F1SQT5|F1SQT5	1	2	0	0	0	0	0	0	1	4	0	0	0	0	0	0	1.4%	2.6%	0.0%	0.0%	0.0%	0.0%	0.0%	0.0%
tr|F1RME2|F1RME2	4	0	0	0	0	0	0	0	5	0	0	0	0	0	0	0	22.5%	0.0%	0.0%	0.0%	0.0%	0.0%	0.0%	0.0%
tr|F1SIA1|F1SIA1	1	1	0	0	0	0	0	0	1	4	0	0	0	0	0	0	1.3%	1.3%	0.0%	0.0%	0.0%	0.0%	0.0%	0.0%
tr|I3L5B8|I3L5B8	2	1	0	0	0	0	0	0	3	2	0	0	0	0	0	0	6.3%	4.0%	0.0%	0.0%	0.0%	0.0%	0.0%	0.0%
Q29223	0	1	0	0	0	0	0	0	0	5	0	0	0	0	0	0	0.0%	8.5%	0.0%	0.0%	0.0%	0.0%	0.0%	0.0%
Q29244	2	0	0	0	0	0	0	0	5	0	0	0	0	0	0	0	12.7%	0.0%	0.0%	0.0%	0.0%	0.0%	0.0%	0.0%
tr|K7GQE9|K7GQE9	2	0	0	0	0	0	0	0	5	0	0	0	0	0	0	0	14.6%	0.0%	0.0%	0.0%	0.0%	0.0%	0.0%	0.0%
tr|F1RMN3|F1RMN3	0	2	0	0	0	0	0	0	0	5	0	0	0	0	0	0	0.0%	2.4%	0.0%	0.0%	0.0%	0.0%	0.0%	0.0%
tr|F1RTF0|F1RTF0	3	1	0	0	0	0	0	0	4	1	0	0	0	0	0	0	1.9%	0.6%	0.0%	0.0%	0.0%	0.0%	0.0%	0.0%
tr|K7GQ35|K7GQ35	3	0	0	0	0	0	0	0	5	0	0	0	0	0	0	0	15.0%	0.0%	0.0%	0.0%	0.0%	0.0%	0.0%	0.0%
tr|F2Z5B9|F2Z5B9	0	1	0	0	0	0	0	0	0	5	0	0	0	0	0	0	0.0%	8.5%	0.0%	0.0%	0.0%	0.0%	0.0%	0.0%
tr|F1SLQ3|F1SLQ3	2	2	0	0	0	0	0	0	3	2	0	0	0	0	0	0	28.4%	28.4%	0.0%	0.0%	0.0%	0.0%	0.0%	0.0%
tr|F1STC1|F1STC1	4	0	0	0	0	0	0	0	5	0	0	0	0	0	0	0	12.1%	0.0%	0.0%	0.0%	0.0%	0.0%	0.0%	0.0%
tr|I3L9Y6|I3L9Y6	4	0	0	0	0	0	0	0	5	0	0	0	0	0	0	0	35.2%	0.0%	0.0%	0.0%	0.0%	0.0%	0.0%	0.0%
tr|F1SA96|F1SA96	2	1	0	0	0	0	0	0	3	2	0	0	0	0	0	0	6.0%	3.2%	0.0%	0.0%	0.0%	0.0%	0.0%	0.0%
tr|K7GL09|K7GL09	2	0	0	0	0	0	0	0	5	0	0	0	0	0	0	0	11.1%	0.0%	0.0%	0.0%	0.0%	0.0%	0.0%	0.0%
tr|K7GMM8|K7GMM8	2	0	0	0	0	0	0	0	5	0	0	0	0	0	0	0	13.9%	0.0%	0.0%	0.0%	0.0%	0.0%	0.0%	0.0%
tr|I3LJX7|I3LJX7	3	1	0	0	0	0	0	0	4	1	0	0	0	0	0	0	6.1%	1.5%	0.0%	0.0%	0.0%	0.0%	0.0%	0.0%
tr|F1SJK6|F1SJK6	2	1	0	0	0	0	0	0	3	2	0	0	0	0	0	0	11.8%	3.0%	0.0%	0.0%	0.0%	0.0%	0.0%	0.0%
tr|H9KVH1|H9KVH1	0	3	0	0	0	0	0	0	0	5	0	0	0	0	0	0	0.0%	6.6%	0.0%	0.0%	0.0%	0.0%	0.0%	0.0%
tr|I3LPM1|I3LPM1	3	1	0	0	0	0	0	0	4	1	0	0	0	0	0	0	10.8%	5.6%	0.0%	0.0%	0.0%	0.0%	0.0%	0.0%
tr|F1SGR1|F1SGR1	0	1	0	0	0	0	0	0	0	5	0	0	0	0	0	0	0.0%	0.6%	0.0%	0.0%	0.0%	0.0%	0.0%	0.0%
tr|F1STU7|F1STU7	3	0	0	0	0	0	0	0	5	0	0	0	0	0	0	0	19.5%	0.0%	0.0%	0.0%	0.0%	0.0%	0.0%	0.0%
tr|I3LLQ7|I3LLQ7	2	1	0	0	0	0	0	0	2	3	0	0	0	0	0	0	12.7%	4.9%	0.0%	0.0%	0.0%	0.0%	0.0%	0.0%
tr|F1SMH5|F1SMH5	0	1	0	0	0	0	0	0	0	5	0	0	0	0	0	0	0.0%	2.8%	0.0%	0.0%	0.0%	0.0%	0.0%	0.0%
tr|F1RXY7|F1RXY7	3	0	0	0	0	0	0	0	5	0	0	0	0	0	0	0	18.0%	0.0%	0.0%	0.0%	0.0%	0.0%	0.0%	0.0%
tr|I3LCM4|I3LCM4	2	1	0	0	0	0	0	0	4	1	0	0	0	0	0	0	8.0%	3.2%	0.0%	0.0%	0.0%	0.0%	0.0%	0.0%
tr|F1SR70|F1SR70	2	1	0	0	0	0	0	0	3	2	0	0	0	0	0	0	15.7%	3.1%	0.0%	0.0%	0.0%	0.0%	0.0%	0.0%
tr|F1RGQ5|F1RGQ5	1	0	0	0	0	0	0	0	5	0	0	0	0	0	0	0	11.2%	0.0%	0.0%	0.0%	0.0%	0.0%	0.0%	0.0%
tr|F1S8N5|F1S8N5	1	3	0	0	0	0	0	0	1	4	0	0	0	0	0	0	0.4%	1.4%	0.0%	0.0%	0.0%	0.0%	0.0%	0.0%
tr|F1RWH6|F1RWH6	3	1	0	0	0	0	0	0	4	1	0	0	0	0	0	0	7.0%	6.1%	0.0%	0.0%	0.0%	0.0%	0.0%	0.0%
tr|I3L9M0|I3L9M0	4	0	0	0	0	0	0	0	5	0	0	0	0	0	0	0	41.8%	0.0%	0.0%	0.0%	0.0%	0.0%	0.0%	0.0%
tr|I3LEC4|I3LEC4	0	1	0	0	0	0	0	0	0	5	0	0	0	0	0	0	0.0%	0.7%	0.0%	0.0%	0.0%	0.0%	0.0%	0.0%
tr|F1SDW4|F1SDW4	0	1	0	0	0	0	0	0	0	5	0	0	0	0	0	0	0.0%	1.2%	0.0%	0.0%	0.0%	0.0%	0.0%	0.0%
tr|I3L5Y7|I3L5Y7	2	1	0	0	0	0	0	0	3	2	0	0	0	0	0	0	11.9%	3.0%	0.0%	0.0%	0.0%	0.0%	0.0%	0.0%
tr|I3L635|I3L635	5	0	0	0	0	0	0	0	5	0	0	0	0	0	0	0	15.9%	0.0%	0.0%	0.0%	0.0%	0.0%	0.0%	0.0%
tr|F1SKG9|F1SKG9	1	2	0	0	0	0	0	0	1	4	0	0	0	0	0	0	1.0%	2.6%	0.0%	0.0%	0.0%	0.0%	0.0%	0.0%
tr|E7CXS1|E7CXS1	4	1	0	0	0	0	0	0	4	1	0	0	0	0	0	0	7.7%	2.2%	0.0%	0.0%	0.0%	0.0%	0.0%	0.0%
tr|F1RUR7|F1RUR7	4	1	0	0	0	0	0	0	4	1	0	0	0	0	0	0	12.4%	1.8%	0.0%	0.0%	0.0%	0.0%	0.0%	0.0%
tr|F1RTK6|F1RTK6	3	0	0	0	0	0	0	0	5	0	0	0	0	0	0	0	14.9%	0.0%	0.0%	0.0%	0.0%	0.0%	0.0%	0.0%
tr|I3L781|I3L781	2	1	0	0	0	0	0	0	4	1	0	0	0	0	0	0	3.0%	1.4%	0.0%	0.0%	0.0%	0.0%	0.0%	0.0%
tr|F1SVD4|F1SVD4	1	1	0	0	0	0	0	0	1	4	0	0	0	0	0	0	3.8%	4.4%	0.0%	0.0%	0.0%	0.0%	0.0%	0.0%
tr|I3L6N5|I3L6N5	0	2	0	0	0	0	0	0	0	5	0	0	0	0	0	0	0.0%	5.0%	0.0%	0.0%	0.0%	0.0%	0.0%	0.0%
tr|F1RNV8|F1RNV8	3	1	0	0	0	0	0	0	3	2	0	0	0	0	0	0	7.0%	3.0%	0.0%	0.0%	0.0%	0.0%	0.0%	0.0%
tr|F1SV55|F1SV55	0	3	0	0	0	0	0	0	0	5	0	0	0	0	0	0	0.0%	10.5%	0.0%	0.0%	0.0%	0.0%	0.0%	0.0%
tr|F1SEZ4|F1SEZ4	3	0	0	0	0	0	0	0	5	0	0	0	0	0	0	0	8.2%	0.0%	0.0%	0.0%	0.0%	0.0%	0.0%	0.0%
tr|F1S0V6|F1S0V6	3	1	0	0	0	0	0	0	4	1	0	0	0	0	0	0	50.8%	7.3%	0.0%	0.0%	0.0%	0.0%	0.0%	0.0%
tr|F1S569|F1S569	1	2	0	0	0	0	0	0	1	4	0	0	0	0	0	0	7.0%	9.5%	0.0%	0.0%	0.0%	0.0%	0.0%	0.0%
tr|F2Z5G7|F2Z5G7	2	1	0	0	0	0	0	0	2	3	0	0	0	0	0	0	15.5%	4.6%	0.0%	0.0%	0.0%	0.0%	0.0%	0.0%
tr|I3L5Z6|I3L5Z6	3	1	0	0	0	0	0	0	3	2	0	0	0	0	0	0	19.5%	4.2%	0.0%	0.0%	0.0%	0.0%	0.0%	0.0%
tr|F1RQY4|F1RQY4	5	0	1	0	0	0	0	0	5	0	1	0	0	0	0	0	8.7%	0.0%	2.8%	0.0%	0.0%	0.0%	0.0%	0.0%
tr|K7GSF7|K7GSF7	0	1	1	0	0	0	0	0	0	5	1	0	0	0	0	0	0.0%	0.8%	0.8%	0.0%	0.0%	0.0%	0.0%	0.0%
tr|F1RGR6|F1RGR6	1	2	1	0	0	0	0	0	1	4	1	0	0	0	0	0	26.6%	26.6%	12.8%	0.0%	0.0%	0.0%	0.0%	0.0%
tr|F1S1L9|F1S1L9	2	0	1	0	0	0	0	0	5	0	1	0	0	0	0	0	8.2%	0.0%	2.7%	0.0%	0.0%	0.0%	0.0%	0.0%
tr|F1S7B0|F1S7B0	0	2	1	0	0	0	0	0	0	5	1	0	0	0	0	0	0.0%	3.1%	1.8%	0.0%	0.0%	0.0%	0.0%	0.0%
tr|A5GHK7|A5GHK7	1	1	1	0	0	0	0	0	1	4	1	0	0	0	0	0	1.1%	0.8%	1.0%	0.0%	0.0%	0.0%	0.0%	0.0%
tr|K7GQE7|K7GQE7	2	0	1	0	0	0	0	0	5	0	1	0	0	0	0	0	8.1%	0.0%	2.7%	0.0%	0.0%	0.0%	0.0%	0.0%
Q29307	1	0	1	0	0	0	0	0	5	0	1	0	0	0	0	0	10.2%	0.0%	14.8%	0.0%	0.0%	0.0%	0.0%	0.0%
tr|I3LRS8|I3LRS8	3	0	1	0	0	0	0	0	5	0	1	0	0	0	0	0	17.4%	0.0%	4.4%	0.0%	0.0%	0.0%	0.0%	0.0%
tr|K7GS58|K7GS58	2	0	1	0	0	0	0	0	5	0	1	0	0	0	0	0	7.9%	0.0%	2.6%	0.0%	0.0%	0.0%	0.0%	0.0%
tr|I3LE23|I3LE23	2	0	1	0	0	0	0	0	5	0	1	0	0	0	0	0	7.6%	0.0%	2.5%	0.0%	0.0%	0.0%	0.0%	0.0%
tr|F1SEM6|F1SEM6	2	0	1	0	0	0	0	0	5	0	2	0	0	0	0	0	6.8%	0.0%	5.9%	0.0%	0.0%	0.0%	0.0%	0.0%
tr|F1RU94|F1RU94	3	1	2	0	0	0	0	0	3	2	2	0	0	0	0	0	4.2%	1.2%	5.2%	0.0%	0.0%	0.0%	0.0%	0.0%
tr|J9JIK2|J9JIK2	3	1	2	0	0	0	0	0	3	2	2	0	0	0	0	0	4.4%	1.2%	5.5%	0.0%	0.0%	0.0%	0.0%	0.0%
tr|F1STG2|F1STG2	1	1	1	0	0	0	0	0	1	4	2	0	0	0	0	0	3.5%	2.1%	3.9%	0.0%	0.0%	0.0%	0.0%	0.0%
tr|A5D9P0|A5D9P0	5	0	1	0	0	0	0	0	5	0	2	0	0	0	0	0	15.3%	0.0%	3.4%	0.0%	0.0%	0.0%	0.0%	0.0%
tr|F1SRN6|F1SRN6	4	1	2	0	0	0	0	0	4	1	3	0	0	0	0	0	9.5%	2.1%	2.0%	0.0%	0.0%	0.0%	0.0%	0.0%
tr|I3L6P8|I3L6P8	2	1	1	0	0	0	0	0	2	3	3	0	0	0	0	0	5.2%	1.7%	3.3%	0.0%	0.0%	0.0%	0.0%	0.0%
tr|K7GT02|K7GT02	4	0	2	0	0	0	0	0	5	0	3	0	0	0	0	0	12.1%	0.0%	3.9%	0.0%	0.0%	0.0%	0.0%	0.0%
tr|F1RW40|F1RW40	4	0	2	0	0	0	0	0	5	0	3	0	0	0	0	0	11.5%	0.0%	3.7%	0.0%	0.0%	0.0%	0.0%	0.0%
tr|F1SJ05|F1SJ05	1	1	1	0	0	0	0	0	1	4	3	0	0	0	0	0	4.4%	6.3%	10.3%	0.0%	0.0%	0.0%	0.0%	0.0%
tr|I3L5V4|I3L5V4	3	0	1	0	0	0	0	0	5	0	3	0	0	0	0	0	4.7%	0.0%	1.7%	0.0%	0.0%	0.0%	0.0%	0.0%

	Peptide Count								Spectral Count								Sequence Coverage							
	Whole Cell	Whole Cell	Whole Cell	Cilia Fraction	Cilia Fraction	Cilia Fraction	Cilia Fraction	Cilia Fraction	Whole Cell	Whole Cell	Whole Cell	Cilia Fraction	Cilia Fraction	Cilia Fraction	Cilia Fraction	Cilia Fraction	Whole Cell	Whole Cell	Whole Cell	Cilia Fraction	Cilia Fraction	Cilia Fraction	Cilia Fraction	Cilia Fraction
Sample#	1	2	6	4	3	5	7	8	1	2	6	4	3	5	7	8	1	2	6	4	3	5	7	8
**Accession**																								
tr|F1SSB0|F1SSB0	3	0	1	0	0	0	0	0	5	0	3	0	0	0	0	0	4.7%	0.0%	1.7%	0.0%	0.0%	0.0%	0.0%	0.0%
tr|I3LH08|I3LH08	2	1	1	0	0	0	0	0	2	3	3	0	0	0	0	0	5.2%	1.7%	3.2%	0.0%	0.0%	0.0%	0.0%	0.0%
Q02038	4	1	2	0	0	0	0	0	4	1	3	0	0	0	0	0	10.1%	2.3%	2.1%	0.0%	0.0%	0.0%	0.0%	0.0%
tr|I3LC77|I3LC77	1	2	1	0	0	0	0	0	1	4	3	0	0	0	0	0	4.2%	3.8%	7.6%	0.0%	0.0%	0.0%	0.0%	0.0%
tr|F1S6C1|F1S6C1	1	2	1	0	0	0	0	0	1	4	3	0	0	0	0	0	4.4%	4.0%	7.9%	0.0%	0.0%	0.0%	0.0%	0.0%
tr|J9JIN0|J9JIN0	4	1	2	0	0	0	0	0	4	1	3	0	0	0	0	0	10.3%	2.3%	2.2%	0.0%	0.0%	0.0%	0.0%	0.0%
tr|K7GM50|K7GM50	2	0	2	0	0	0	0	0	5	0	4	0	0	0	0	0	17.4%	0.0%	18.5%	0.0%	0.0%	0.0%	0.0%	0.0%
tr|I3LGB3|I3LGB3	3	0	1	0	0	0	0	0	5	0	4	0	0	0	0	0	52.8%	0.0%	26.4%	0.0%	0.0%	0.0%	0.0%	0.0%
Q7YS99	3	1	1	0	0	0	0	0	4	1	4	0	0	0	0	0	7.3%	2.3%	1.7%	0.0%	0.0%	0.0%	0.0%	0.0%
tr|F1S620|F1S620	2	1	1	0	0	0	0	0	3	2	4	0	0	0	0	0	2.9%	1.2%	2.4%	0.0%	0.0%	0.0%	0.0%	0.0%
tr|F1RH75|F1RH75	4	0	3	0	0	0	0	0	5	0	4	0	0	0	0	0	3.1%	0.0%	2.6%	0.0%	0.0%	0.0%	0.0%	0.0%
tr|F1S286|F1S286	4	1	1	0	0	0	0	0	4	1	4	0	0	0	0	0	16.3%	5.6%	8.4%	0.0%	0.0%	0.0%	0.0%	0.0%
tr|F1S619|F1S619	2	1	1	0	0	0	0	0	3	2	4	0	0	0	0	0	2.9%	1.2%	2.3%	0.0%	0.0%	0.0%	0.0%	0.0%
tr|K7GQA9|K7GQA9	2	0	2	0	0	0	0	0	5	0	4	0	0	0	0	0	20.1%	0.0%	21.3%	0.0%	0.0%	0.0%	0.0%	0.0%
tr|F1S775|F1S775	3	0	1	0	0	0	0	0	5	0	5	0	0	0	0	0	7.0%	0.0%	1.1%	0.0%	0.0%	0.0%	0.0%	0.0%
tr|I6L631|I6L631	4	0	2	0	0	0	0	0	5	0	5	0	0	0	0	0	29.8%	0.0%	17.3%	0.0%	0.0%	0.0%	0.0%	0.0%
tr|F1SMG6|F1SMG6	2	1	1	0	0	0	0	0	4	1	5	0	0	0	0	0	10.8%	2.5%	4.2%	0.0%	0.0%	0.0%	0.0%	0.0%
tr|F1SUB1|F1SUB1	5	0	2	0	0	0	0	0	5	0	5	0	0	0	0	0	50.0%	0.0%	13.5%	0.0%	0.0%	0.0%	0.0%	0.0%
tr|F1SU46|F1SU46	5	0	1	0	0	0	0	0	5	0	5	0	0	0	0	0	8.7%	0.0%	3.3%	0.0%	0.0%	0.0%	0.0%	0.0%
tr|I3LEX9|I3LEX9	3	0	1	0	0	0	0	0	5	0	5	0	0	0	0	0	19.9%	0.0%	11.2%	0.0%	0.0%	0.0%	0.0%	0.0%
tr|K7GN24|K7GN24	1	0	3	0	0	0	0	0	5	0	6	0	0	0	0	0	10.0%	0.0%	7.6%	0.0%	0.0%	0.0%	0.0%	0.0%
tr|F2Z5P9|F2Z5P9	1	1	2	0	0	0	0	0	1	4	6	0	0	0	0	0	16.7%	19.8%	16.7%	0.0%	0.0%	0.0%	0.0%	0.0%
tr|I3LAK4|I3LAK4	2	1	2	0	0	0	0	0	3	2	7	0	0	0	0	0	3.4%	2.0%	6.5%	0.0%	0.0%	0.0%	0.0%	0.0%
Q35914	1	0	1	0	0	0	0	0	5	0	7	0	0	0	0	0	14.9%	0.0%	11.9%	0.0%	0.0%	0.0%	0.0%	0.0%
tr|F1SFG7|F1SFG7	4	0	3	0	0	0	0	0	5	0	7	0	0	0	0	0	10.2%	0.0%	7.0%	0.0%	0.0%	0.0%	0.0%	0.0%
tr|F1S0D4|F1S0D4	4	0	2	0	0	0	0	0	5	0	8	0	0	0	0	0	5.5%	0.0%	3.3%	0.0%	0.0%	0.0%	0.0%	0.0%
tr|F1RIQ4|F1RIQ4	2	1	3	0	0	0	0	0	2	3	8	0	0	0	0	0	13.5%	6.8%	18.2%	0.0%	0.0%	0.0%	0.0%	0.0%
tr|I3LM25|I3LM25	2	0	2	0	0	0	0	0	5	0	9	0	0	0	0	0	53.8%	0.0%	42.9%	0.0%	0.0%	0.0%	0.0%	0.0%
tr|F1STR1|F1STR1	3	1	1	0	0	0	0	0	4	1	9	0	0	0	0	0	11.4%	4.8%	3.0%	0.0%	0.0%	0.0%	0.0%	0.0%
tr|F1RIE0|F1RIE0	1	1	2	0	0	0	0	0	1	4	10	0	0	0	0	0	8.6%	6.5%	8.6%	0.0%	0.0%	0.0%	0.0%	0.0%
tr|I3LM66|I3LM66	1	1	3	0	0	0	0	0	3	2	11	0	0	0	0	0	14.5%	14.5%	51.8%	0.0%	0.0%	0.0%	0.0%	0.0%
tr|F1SEN4|F1SEN4	4	0	2	0	0	0	0	0	5	0	15	0	0	0	0	0	30.3%	0.0%	30.3%	0.0%	0.0%	0.0%	0.0%	0.0%
tr|F1S7L8|F1S7L8	3	1	5	0	0	0	0	0	4	1	16	0	0	0	0	0	9.2%	3.0%	14.6%	0.0%	0.0%	0.0%	0.0%	0.0%
tr|F1S3J0|F1S3J0	1	2	3	0	0	0	0	0	2	3	17	0	0	0	0	0	6.7%	5.7%	7.6%	0.0%	0.0%	0.0%	0.0%	0.0%
tr|F1RJM8|F1RJM8	4	0	3	0	0	0	0	0	5	0	19	0	0	0	0	0	18.6%	0.0%	13.5%	0.0%	0.0%	0.0%	0.0%	0.0%
tr|I3L679|I3L679	1	1	1	0	0	0	0	0	1	4	19	0	0	0	0	0	8.1%	11.1%	8.1%	0.0%	0.0%	0.0%	0.0%	0.0%
tr|F1SKI0|F1SKI0	1	1	2	0	0	0	0	0	1	4	20	0	0	0	0	0	1.2%	1.2%	3.1%	0.0%	0.0%	0.0%	0.0%	0.0%
tr|F1RZ16|F1RZ16	4	0	4	0	0	0	0	0	5	0	22	0	0	0	0	0	31.4%	0.0%	24.5%	0.0%	0.0%	0.0%	0.0%	0.0%
tr|F1REW5|F1REW5	3	1	4	0	0	0	0	0	4	1	26	0	0	0	0	0	4.4%	0.9%	6.3%	0.0%	0.0%	0.0%	0.0%	0.0%
tr|I3LRK1|I3LRK1	5	0	5	0	0	0	0	0	5	0	30	0	0	0	0	0	16.4%	0.0%	13.8%	0.0%	0.0%	0.0%	0.0%	0.0%
tr|F1SGX4|F1SGX4	1	1	1	0	0	0	0	0	2	3	136	0	0	0	0	0	6.1%	6.1%	6.1%	0.0%	0.0%	0.0%	0.0%	0.0%
tr|I3LSA3|I3LSA3	3	0	0	0	0	0	5	6	4	0	0	0	0	0	20	58	12.6%	0.0%	0.0%	0.0%	0.0%	0.0%	12.8%	21.9%
tr|F1RI88|F1RI88	2	1	0	0	0	0	3	3	2	2	0	0	0	0	12	33	8.3%	3.1%	0.0%	0.0%	0.0%	0.0%	14.9%	15.2%
tr|I3LRX8|I3LRX8	3	0	2	0	0	0	0	5	4	0	3	0	0	0	0	73	14.7%	0.0%	7.6%	0.0%	0.0%	0.0%	0.0%	12.7%
tr|F2Z5M5|F2Z5M5	2	0	1	0	0	0	2	7	4	0	1	0	0	0	10	38	8.1%	0.0%	9.6%	0.0%	0.0%	0.0%	19.3%	34.5%
tr|F1SRY1|F1SRY1	4	0	1	0	0	0	1	6	4	0	2	0	0	0	6	29	6.4%	0.0%	2.8%	0.0%	0.0%	0.0%	1.8%	12.0%
tr|I3LEV4|I3LEV4	1	3	0	0	0	0	0	3	1	3	0	0	0	0	0	23	2.1%	9.0%	0.0%	0.0%	0.0%	0.0%	0.0%	8.5%
tr|F1S336|F1S336	2	1	0	0	0	0	1	5	2	2	0	0	0	0	3	20	8.6%	3.1%	0.0%	0.0%	0.0%	0.0%	3.1%	14.5%
tr|F1RTW7|F1RTW7	3	0	0	0	0	0	0	5	4	0	0	0	0	0	0	19	19.0%	0.0%	0.0%	0.0%	0.0%	0.0%	0.0%	13.8%
tr|I3L5C4|I3L5C4	2	1	1	0	0	0	1	1	3	1	2	0	0	0	6	15	1.7%	1.7%	1.3%	0.0%	0.0%	0.0%	1.7%	1.7%
tr|I3LG18|I3LG18	3	0	0	0	0	0	1	2	4	0	0	0	0	0	11	3	2.8%	0.0%	0.0%	0.0%	0.0%	0.0%	1.3%	3.1%
tr|F1RQB2|F1RQB2	2	0	0	0	0	0	1	6	4	0	0	0	0	0	1	12	0.4%	0.0%	0.0%	0.0%	0.0%	0.0%	0.7%	3.7%
tr|I3LMS7|I3LMS7	1	1	0	0	0	0	0	2	2	2	0	0	0	0	0	12	3.0%	3.0%	0.0%	0.0%	0.0%	0.0%	0.0%	3.0%
tr|F2Z503|F2Z503	3	0	0	0	0	0	1	2	4	0	0	0	0	0	4	7	39.5%	0.0%	0.0%	0.0%	0.0%	0.0%	24.2%	39.5%
tr|I3LB00|I3LB00	1	1	1	0	0	0	1	1	1	3	3	0	0	0	13	5	4.2%	4.2%	4.2%	0.0%	0.0%	0.0%	4.2%	4.2%
tr|F1SRL9|F1SRL9	3	0	25	0	0	0	5	10	4	0	115	0	0	0	133	154	9.6%	0.0%	91.2%	0.0%	0.0%	0.0%	56.6%	66.7%
P61220	2	1	2	0	0	0	1	1	3	1	6	0	0	0	17	7	16.8%	16.8%	35.4%	0.0%	0.0%	0.0%	21.2%	21.2%
tr|I3LQ54|I3LQ54	3	0	0	0	0	0	1	1	4	0	0	0	0	0	2	7	21.1%	0.0%	0.0%	0.0%	0.0%	0.0%	14.1%	14.1%
tr|I3LSH8|I3LSH8	1	0	2	0	0	0	3	6	4	0	16	0	0	0	6	37	3.2%	0.0%	4.8%	0.0%	0.0%	0.0%	11.8%	19.5%
tr|F1SIC5|F1SIC5	1	0	2	0	0	0	3	6	4	0	16	0	0	0	6	37	3.3%	0.0%	4.9%	0.0%	0.0%	0.0%	12.0%	19.8%
tr|F1S415|F1S415	3	0	1	0	0	0	1	2	4	0	9	0	0	0	11	11	6.8%	0.0%	3.1%	0.0%	0.0%	0.0%	5.4%	9.4%
tr|I3L8F2|I3L8F2	3	0	0	0	0	0	1	1	4	0	0	0	0	0	3	3	11.0%	0.0%	0.0%	0.0%	0.0%	0.0%	2.7%	7.7%
tr|K7GMF6|K7GMF6	3	1	0	0	0	0	1	3	3	1	0	0	0	0	1	5	5.5%	2.4%	0.0%	0.0%	0.0%	0.0%	2.4%	5.9%
tr|I3LS61|I3LS61	0	1	0	0	0	0	1	1	0	4	0	0	0	0	4	2	0.0%	1.8%	0.0%	0.0%	0.0%	0.0%	1.8%	1.8%
tr|F1SD42|F1SD42	3	1	0	0	0	0	1	3	3	1	0	0	0	0	1	5	4.0%	1.7%	0.0%	0.0%	0.0%	0.0%	1.7%	4.3%
tr|I3LM14|I3LM14	1	0	1	0	0	0	1	3	4	0	7	0	0	0	9	7	7.2%	0.0%	4.0%	0.0%	0.0%	0.0%	6.9%	14.1%
Q29380	1	0	2	0	0	0	1	3	4	0	8	0	0	0	9	7	7.1%	0.0%	4.9%	0.0%	0.0%	0.0%	6.7%	13.8%
tr|K7GR25|K7GR25	2	2	1	0	0	0	1	1	2	2	5	0	0	0	2	10	19.1%	19.1%	7.3%	0.0%	0.0%	0.0%	11.8%	11.8%
tr|F1SAH0|F1SAH0	2	1	1	0	0	0	1	1	3	1	1	0	0	0	4	2	2.1%	1.8%	2.1%	0.0%	0.0%	0.0%	1.6%	1.6%
tr|F1SJY1|F1SJY1	3	1	3	0	0	0	0	3	3	1	9	0	0	0	0	14	16.9%	8.3%	8.3%	0.0%	0.0%	0.0%	0.0%	12.1%
Q767M0	1	0	0	0	0	0	1	2	4	0	0	0	0	0	1	3	2.1%	0.0%	0.0%	0.0%	0.0%	0.0%	3.9%	9.5%
tr|F1RR77|F1RR77	0	1	0	0	0	0	0	1	0	4	0	0	0	0	0	4	0.0%	2.6%	0.0%	0.0%	0.0%	0.0%	0.0%	6.2%
tr|F1SE27|F1SE27	1	0	0	0	0	0	0	2	4	0	0	0	0	0	0	4	15.5%	0.0%	0.0%	0.0%	0.0%	0.0%	0.0%	15.5%
tr|F1SE26|F1SE26	1	0	3	0	0	0	1	3	4	0	15	0	0	0	9	7	7.1%	0.0%	8.8%	0.0%	0.0%	0.0%	6.7%	13.8%
tr|F1SH25|F1SH25	2	1	2	0	0	0	2	1	2	2	2	0	0	0	3	2	13.1%	5.4%	13.1%	0.0%	0.0%	0.0%	11.7%	6.3%
tr|B2DCZ7|B2DCZ7	2	0	0	0	0	0	0	1	4	0	0	0	0	0	0	3	9.6%	0.0%	0.0%	0.0%	0.0%	0.0%	0.0%	3.0%
tr|B6V6V4|B6V6V4	4	0	0	0	0	0	0	3	4	0	0	0	0	0	0	3	11.2%	0.0%	0.0%	0.0%	0.0%	0.0%	0.0%	8.2%
tr|F1S8H4|F1S8H4	2	1	0	0	0	0	1	1	2	2	0	0	0	0	2	1	10.1%	4.7%	0.0%	0.0%	0.0%	0.0%	4.7%	8.3%
tr|F1SVA6|F1SVA6	3	0	0	0	0	0	0	1	4	0	0	0	0	0	0	3	10.3%	0.0%	0.0%	0.0%	0.0%	0.0%	0.0%	4.5%
tr|F1SSG1|F1SSG1	3	1	0	0	0	0	0	1	3	1	0	0	0	0	0	3	17.7%	6.6%	0.0%	0.0%	0.0%	0.0%	0.0%	6.6%
tr|F1S9W6|F1S9W6	2	0	0	0	0	0	0	2	4	0	0	0	0	0	0	3	2.2%	0.0%	0.0%	0.0%	0.0%	0.0%	0.0%	11.0%

	Peptide Count								Spectral Count								Sequence Coverage							
	Whole Cell	Whole Cell	Whole Cell	Cilia Fraction	Cilia Fraction	Cilia Fraction	Cilia Fraction	Cilia Fraction	Whole Cell	Whole Cell	Whole Cell	Cilia Fraction	Cilia Fraction	Cilia Fraction	Cilia Fraction	Cilia Fraction	Whole Cell	Whole Cell	Whole Cell	Cilia Fraction	Cilia Fraction	Cilia Fraction	Cilia Fraction	Cilia Fraction
Sample#	1	2	6	4	3	5	7	8	1	2	6	4	3	5	7	8	1	2	6	4	3	5	7	8
**Accession**																								
tr|I3LH70|I3LH70	2	0	1	0	0	0	0	1	4	0	1	0	0	0	0	3	4.0%	0.0%	6.4%	0.0%	0.0%	0.0%	0.0%	9.7%
tr|F1S100|F1S100	3	0	1	0	0	0	0	1	4	0	1	0	0	0	0	3	13.8%	0.0%	3.6%	0.0%	0.0%	0.0%	0.0%	7.8%
tr|F1SQG6|F1SQG6	2	0	1	0	0	0	0	1	4	0	3	0	0	0	0	4	9.6%	0.0%	5.9%	0.0%	0.0%	0.0%	0.0%	5.9%
A5GFN6	3	1	4	0	0	0	0	1	3	1	12	0	0	0	0	9	17.9%	4.6%	10.9%	0.0%	0.0%	0.0%	0.0%	5.4%
tr|F1SFD6|F1SFD6	2	0	3	0	0	0	0	1	4	0	8	0	0	0	0	6	24.1%	0.0%	18.4%	0.0%	0.0%	0.0%	0.0%	16.3%
tr|K7GLG5|K7GLG5	1	1	0	0	0	0	0	1	2	2	0	0	0	0	0	2	5.0%	6.1%	0.0%	0.0%	0.0%	0.0%	0.0%	5.0%
tr|K7GLD3|K7GLD3	1	1	0	0	0	0	0	1	2	2	0	0	0	0	0	2	3.8%	4.6%	0.0%	0.0%	0.0%	0.0%	0.0%	3.8%
I3LUP1	3	0	0	0	0	0	1	0	4	0	0	0	0	0	2	0	7.4%	0.0%	0.0%	0.0%	0.0%	0.0%	5.7%	0.0%
tr|K7GLR6|K7GLR6	1	1	0	0	0	0	0	1	2	2	0	0	0	0	0	2	3.4%	4.2%	0.0%	0.0%	0.0%	0.0%	0.0%	3.4%
tr|F1S268|F1S268	2	0	0	0	0	0	0	1	4	0	0	0	0	0	0	2	7.7%	0.0%	0.0%	0.0%	0.0%	0.0%	0.0%	9.5%
tr|F1SPN1|F1SPN1	3	1	3	0	0	0	0	1	3	1	31	0	0	0	0	15	17.9%	5.5%	17.9%	0.0%	0.0%	0.0%	0.0%	9.0%
tr|F1RW31|F1RW31	4	0	1	0	0	0	0	1	4	0	1	0	0	0	0	2	9.7%	0.0%	2.9%	0.0%	0.0%	0.0%	0.0%	3.7%
tr|I3LPR8|I3LPR8	1	1	4	0	0	0	0	1	3	1	21	0	0	0	0	8	17.5%	17.5%	17.5%	0.0%	0.0%	0.0%	0.0%	17.5%
P07802	2	0	3	0	0	0	0	2	4	0	8	0	0	0	0	3	4.7%	0.0%	11.1%	0.0%	0.0%	0.0%	0.0%	6.8%
tr|F1RV23|F1RV23	2	0	3	0	0	0	0	2	4	0	8	0	0	0	0	3	4.7%	0.0%	11.1%	0.0%	0.0%	0.0%	0.0%	6.8%
tr|F1S8T4|F1S8T4	0	1	0	0	0	0	0	1	0	4	0	0	0	0	0	1	0.0%	1.9%	0.0%	0.0%	0.0%	0.0%	0.0%	2.0%
tr|F1SG40|F1SG40	4	0	0	0	0	0	0	1	4	0	0	0	0	0	0	1	5.5%	0.0%	0.0%	0.0%	0.0%	0.0%	0.0%	2.8%
Q29594	1	1	0	0	0	0	0	1	2	2	0	0	0	0	0	1	15.7%	15.7%	0.0%	0.0%	0.0%	0.0%	0.0%	15.7%
tr|F1RR62|F1RR62	3	0	0	0	0	0	0	1	4	0	0	0	0	0	0	1	12.3%	0.0%	0.0%	0.0%	0.0%	0.0%	0.0%	5.0%
tr|F1RK77|F1RK77	2	1	0	0	0	0	0	1	3	1	0	0	0	0	0	1	10.5%	4.5%	0.0%	0.0%	0.0%	0.0%	0.0%	9.8%
tr|I3LLS0|I3LLS0	4	0	2	0	0	0	0	1	4	0	5	0	0	0	0	2	21.7%	0.0%	10.4%	0.0%	0.0%	0.0%	0.0%	6.8%
tr|F1S3E3|F1S3E3	3	0	1	0	0	0	0	1	4	0	6	0	0	0	0	2	28.8%	0.0%	12.4%	0.0%	0.0%	0.0%	0.0%	12.4%
tr|D4P3C2|D4P3C2	3	0	2	0	0	0	0	2	4	0	7	0	0	0	0	2	7.7%	0.0%	5.4%	0.0%	0.0%	0.0%	0.0%	5.0%
tr|I3LFR2|I3LFR2	3	0	1	0	0	0	0	1	4	0	3	0	0	0	0	1	4.1%	0.0%	1.8%	0.0%	0.0%	0.0%	0.0%	2.8%
tr|I3L7W1|I3L7W1	3	0	4	0	0	0	0	2	4	0	11	0	0	0	0	2	29.8%	0.0%	15.6%	0.0%	0.0%	0.0%	0.0%	14.2%
tr|I3LDE1|I3LDE1	3	0	2	0	0	0	0	1	4	0	12	0	0	0	0	2	54.0%	0.0%	17.5%	0.0%	0.0%	0.0%	0.0%	21.2%
tr|F1RNW4|F1RNW4	2	0	1	0	0	0	0	1	4	0	4	0	0	0	0	1	16.3%	0.0%	3.1%	0.0%	0.0%	0.0%	0.0%	7.8%
tr|F1SBT2|F1SBT2	2	0	2	0	0	0	0	1	4	0	17	0	0	0	0	2	9.5%	0.0%	12.5%	0.0%	0.0%	0.0%	0.0%	12.5%
tr|F1SU34|F1SU34	4	0	3	0	0	0	0	1	4	0	18	0	0	0	0	2	17.5%	0.0%	11.5%	0.0%	0.0%	0.0%	0.0%	5.5%
tr|F1SMQ4|F1SMQ4	2	2	2	0	0	0	0	1	2	2	7	0	0	0	0	1	2.5%	4.2%	4.2%	0.0%	0.0%	0.0%	0.0%	2.3%
tr|I3LCJ2|I3LCJ2	3	0	2	0	0	0	1	1	4	0	25	0	0	0	1	1	6.4%	0.0%	4.2%	0.0%	0.0%	0.0%	4.7%	4.7%
tr|F1SFU0|F1SFU0	3	0	2	0	0	0	1	1	4	0	25	0	0	0	1	1	6.3%	0.0%	4.1%	0.0%	0.0%	0.0%	4.7%	4.7%
tr|I3LCX3|I3LCX3	1	1	1	0	0	0	0	1	3	1	25	0	0	0	0	2	6.5%	6.5%	9.7%	0.0%	0.0%	0.0%	0.0%	7.0%
tr|Q06AA0|Q06AA0	2	1	3	0	0	0	1	0	3	1	17	0	0	0	1	0	6.7%	2.6%	10.1%	0.0%	0.0%	0.0%	4.1%	0.0%
tr|I3LV99|I3LV99	2	2	4	0	0	0	0	1	2	2	25	0	0	0	0	1	1.7%	1.4%	3.3%	0.0%	0.0%	0.0%	0.0%	0.8%
tr|I3LIE3|I3LIE3	2	2	4	0	0	0	0	1	2	2	25	0	0	0	0	1	1.7%	1.4%	3.3%	0.0%	0.0%	0.0%	0.0%	0.8%
tr|K7GQ92|K7GQ92	3	1	2	0	0	0	0	2	3	1	206	0	0	0	0	7	20.3%	7.4%	12.8%	0.0%	0.0%	0.0%	0.0%	12.8%
tr|I3LCY3|I3LCY3	2	1	0	0	0	0	0	0	3	1	0	0	0	0	0	0	3.7%	1.0%	0.0%	0.0%	0.0%	0.0%	0.0%	0.0%
tr|B3VQ66|B3VQ66	0	1	0	0	0	0	0	0	0	4	0	0	0	0	0	0	0.0%	1.7%	0.0%	0.0%	0.0%	0.0%	0.0%	0.0%
tr|I3LBR7|I3LBR7	2	2	0	0	0	0	0	0	2	2	0	0	0	0	0	0	2.0%	1.8%	0.0%	0.0%	0.0%	0.0%	0.0%	0.0%
tr|F1S8U4|F1S8U4	1	1	0	0	0	0	0	0	3	1	0	0	0	0	0	0	7.5%	7.5%	0.0%	0.0%	0.0%	0.0%	0.0%	0.0%
tr|I3LJ13|I3LJ13	3	0	0	0	0	0	0	0	4	0	0	0	0	0	0	0	13.9%	0.0%	0.0%	0.0%	0.0%	0.0%	0.0%	0.0%
tr|I3LUB3|I3LUB3	2	1	0	0	0	0	0	0	3	1	0	0	0	0	0	0	6.8%	6.1%	0.0%	0.0%	0.0%	0.0%	0.0%	0.0%
tr|F1S475|F1S475	3	0	0	0	0	0	0	0	4	0	0	0	0	0	0	0	3.9%	0.0%	0.0%	0.0%	0.0%	0.0%	0.0%	0.0%
Reverse_tr|K7GL83|K7GL83	0	1	0	0	0	0	0	0	0	4	0	0	0	0	0	0	0.0%	1.0%	0.0%	0.0%	0.0%	0.0%	0.0%	0.0%
tr|F1SG35|F1SG35	0	1	0	0	0	0	0	0	0	4	0	0	0	0	0	0	0.0%	3.2%	0.0%	0.0%	0.0%	0.0%	0.0%	0.0%
tr|I3LUJ0|I3LUJ0	2	0	0	0	0	0	0	0	4	0	0	0	0	0	0	0	9.1%	0.0%	0.0%	0.0%	0.0%	0.0%	0.0%	0.0%
tr|F1SMT1|F1SMT1	1	1	0	0	0	0	0	0	1	3	0	0	0	0	0	0	8.6%	6.9%	0.0%	0.0%	0.0%	0.0%	0.0%	0.0%
tr|I3LFU2|I3LFU2	0	1	0	0	0	0	0	0	0	4	0	0	0	0	0	0	0.0%	1.7%	0.0%	0.0%	0.0%	0.0%	0.0%	0.0%
tr|K7GSL9|K7GSL9	2	1	0	0	0	0	0	0	3	1	0	0	0	0	0	0	19.2%	10.2%	0.0%	0.0%	0.0%	0.0%	0.0%	0.0%
tr|I3LA10|I3LA10	2	1	0	0	0	0	0	0	2	2	0	0	0	0	0	0	14.2%	3.4%	0.0%	0.0%	0.0%	0.0%	0.0%	0.0%
tr|K7GPY4|K7GPY4	2	2	0	0	0	0	0	0	2	2	0	0	0	0	0	0	4.9%	2.7%	0.0%	0.0%	0.0%	0.0%	0.0%	0.0%
tr|F1S0W4|F1S0W4	2	1	0	0	0	0	0	0	3	1	0	0	0	0	0	0	0.9%	0.5%	0.0%	0.0%	0.0%	0.0%	0.0%	0.0%
tr|I3L678|I3L678	1	1	0	0	0	0	0	0	2	2	0	0	0	0	0	0	1.6%	0.8%	0.0%	0.0%	0.0%	0.0%	0.0%	0.0%
tr|F1SJP6|F1SJP6	2	0	0	0	0	0	0	0	4	0	0	0	0	0	0	0	12.3%	0.0%	0.0%	0.0%	0.0%	0.0%	0.0%	0.0%
tr|I3LMK7|I3LMK7	0	3	0	0	0	0	0	0	0	4	0	0	0	0	0	0	0.0%	2.6%	0.0%	0.0%	0.0%	0.0%	0.0%	0.0%
tr|Q2XQ99|Q2XQ99	3	1	0	0	0	0	0	0	3	1	0	0	0	0	0	0	6.3%	1.9%	0.0%	0.0%	0.0%	0.0%	0.0%	0.0%
tr|F1RU48|F1RU48	0	1	0	0	0	0	0	0	0	4	0	0	0	0	0	0	0.0%	3.5%	0.0%	0.0%	0.0%	0.0%	0.0%	0.0%
tr|F1SHR6|F1SHR6	2	0	0	0	0	0	0	0	4	0	0	0	0	0	0	0	10.6%	0.0%	0.0%	0.0%	0.0%	0.0%	0.0%	0.0%
tr|F1S324|F1S324	0	2	0	0	0	0	0	0	0	4	0	0	0	0	0	0	0.0%	0.7%	0.0%	0.0%	0.0%	0.0%	0.0%	0.0%
tr|F1RVK1|F1RVK1	3	0	0	0	0	0	0	0	4	0	0	0	0	0	0	0	7.0%	0.0%	0.0%	0.0%	0.0%	0.0%	0.0%	0.0%
tr|F1RU54|F1RU54	1	1	0	0	0	0	0	0	1	3	0	0	0	0	0	0	6.8%	6.2%	0.0%	0.0%	0.0%	0.0%	0.0%	0.0%
tr|I3LJ29|I3LJ29	4	0	0	0	0	0	0	0	4	0	0	0	0	0	0	0	12.8%	0.0%	0.0%	0.0%	0.0%	0.0%	0.0%	0.0%
tr|F1RSF6|F1RSF6	2	1	0	0	0	0	0	0	2	2	0	0	0	0	0	0	10.3%	9.2%	0.0%	0.0%	0.0%	0.0%	0.0%	0.0%
tr|I3LAC4|I3LAC4	2	1	0	0	0	0	0	0	3	1	0	0	0	0	0	0	10.6%	10.6%	0.0%	0.0%	0.0%	0.0%	0.0%	0.0%
tr|I3LH65|I3LH65	2	0	0	0	0	0	0	0	4	0	0	0	0	0	0	0	15.4%	0.0%	0.0%	0.0%	0.0%	0.0%	0.0%	0.0%
tr|I3LM85|I3LM85	0	1	0	0	0	0	0	0	0	4	0	0	0	0	0	0	0.0%	1.7%	0.0%	0.0%	0.0%	0.0%	0.0%	0.0%
O46420	2	1	0	0	0	0	0	0	2	2	0	0	0	0	0	0	4.2%	2.4%	0.0%	0.0%	0.0%	0.0%	0.0%	0.0%
tr|F1SPQ8|F1SPQ8	2	0	0	0	0	0	0	0	4	0	0	0	0	0	0	0	4.4%	0.0%	0.0%	0.0%	0.0%	0.0%	0.0%	0.0%
tr|F1RLG4|F1RLG4	3	0	0	0	0	0	0	0	4	0	0	0	0	0	0	0	27.1%	0.0%	0.0%	0.0%	0.0%	0.0%	0.0%	0.0%
tr|F1RW14|F1RW14	0	1	0	0	0	0	0	0	0	4	0	0	0	0	0	0	0.0%	0.4%	0.0%	0.0%	0.0%	0.0%	0.0%	0.0%
tr|I3LFC2|I3LFC2	1	1	0	0	0	0	0	0	1	3	0	0	0	0	0	0	23.0%	16.2%	0.0%	0.0%	0.0%	0.0%	0.0%	0.0%
tr|K7GLH9|K7GLH9	2	0	0	0	0	0	0	0	4	0	0	0	0	0	0	0	21.8%	0.0%	0.0%	0.0%	0.0%	0.0%	0.0%	0.0%
tr|F1SKE6|F1SKE6	3	0	0	0	0	0	0	0	4	0	0	0	0	0	0	0	6.4%	0.0%	0.0%	0.0%	0.0%	0.0%	0.0%	0.0%
tr|F1S870|F1S870	2	0	0	0	0	0	0	0	4	0	0	0	0	0	0	0	12.6%	0.0%	0.0%	0.0%	0.0%	0.0%	0.0%	0.0%
tr|F1S2I3|F1S2I3	2	1	0	0	0	0	0	0	3	1	0	0	0	0	0	0	8.0%	3.0%	0.0%	0.0%	0.0%	0.0%	0.0%	0.0%
tr|F1S7S7|F1S7S7	2	0	0	0	0	0	0	0	4	0	0	0	0	0	0	0	25.6%	0.0%	0.0%	0.0%	0.0%	0.0%	0.0%	0.0%
tr|F1RM09|F1RM09	2	0	0	0	0	0	0	0	4	0	0	0	0	0	0	0	7.3%	0.0%	0.0%	0.0%	0.0%	0.0%	0.0%	0.0%
tr|I3L7C4|I3L7C4	2	1	0	0	0	0	0	0	2	2	0	0	0	0	0	0	1.6%	1.2%	0.0%	0.0%	0.0%	0.0%	0.0%	0.0%
Reverse_tr|K7GM70|K7GM70	0	1	0	0	0	0	0	0	0	4	0	0	0	0	0	0	0.0%	1.0%	0.0%	0.0%	0.0%	0.0%	0.0%	0.0%
tr|I3LG35|I3LG35	3	0	0	0	0	0	0	0	4	0	0	0	0	0	0	0	5.5%	0.0%	0.0%	0.0%	0.0%	0.0%	0.0%	0.0%

	Peptide Count								Spectral Count								Sequence Coverage							
	Whole Cell	Whole Cell	Whole Cell	Cilia Fraction	Cilia Fraction	Cilia Fraction	Cilia Fraction	Cilia Fraction	Whole Cell	Whole Cell	Whole Cell	Cilia Fraction	Cilia Fraction	Cilia Fraction	Cilia Fraction	Cilia Fraction	Whole Cell	Whole Cell	Whole Cell	Cilia Fraction	Cilia Fraction	Cilia Fraction	Cilia Fraction	Cilia Fraction
Sample#	1	2	6	4	3	5	7	8	1	2	6	4	3	5	7	8	1	2	6	4	3	5	7	8
**Accession**																								
tr|I3L6C0|I3L6C0	2	1	0	0	0	0	0	0	2	2	0	0	0	0	0	0	8.5%	2.5%	0.0%	0.0%	0.0%	0.0%	0.0%	0.0%
tr|I3LTI2|I3LTI2	4	0	0	0	0	0	0	0	4	0	0	0	0	0	0	0	10.7%	0.0%	0.0%	0.0%	0.0%	0.0%	0.0%	0.0%
tr|I3LJ62|I3LJ62	2	1	0	0	0	0	0	0	3	1	0	0	0	0	0	0	16.9%	4.4%	0.0%	0.0%	0.0%	0.0%	0.0%	0.0%
tr|F1SB47|F1SB47	1	2	0	0	0	0	0	0	1	3	0	0	0	0	0	0	5.8%	5.8%	0.0%	0.0%	0.0%	0.0%	0.0%	0.0%
tr|F1S6Y8|F1S6Y8	1	1	0	0	0	0	0	0	2	2	0	0	0	0	0	0	1.6%	0.8%	0.0%	0.0%	0.0%	0.0%	0.0%	0.0%
P79307	3	1	0	0	0	0	0	0	3	1	0	0	0	0	0	0	6.1%	1.8%	0.0%	0.0%	0.0%	0.0%	0.0%	0.0%
tr|F1RUT9|F1RUT9	1	2	0	0	0	0	0	0	1	3	0	0	0	0	0	0	1.5%	1.9%	0.0%	0.0%	0.0%	0.0%	0.0%	0.0%
tr|F1S563|F1S563	2	1	0	0	0	0	0	0	2	2	0	0	0	0	0	0	4.4%	1.9%	0.0%	0.0%	0.0%	0.0%	0.0%	0.0%
tr|I3LPU5|I3LPU5	0	1	0	0	0	0	0	0	0	4	0	0	0	0	0	0	0.0%	19.1%	0.0%	0.0%	0.0%	0.0%	0.0%	0.0%
tr|F1RTU6|F1RTU6	1	1	0	0	0	0	0	0	3	1	0	0	0	0	0	0	15.7%	15.7%	0.0%	0.0%	0.0%	0.0%	0.0%	0.0%
tr|J9JIM8|J9JIM8	2	2	0	0	0	0	0	0	2	2	0	0	0	0	0	0	4.9%	2.6%	0.0%	0.0%	0.0%	0.0%	0.0%	0.0%
tr|Q2XQA0|Q2XQA0	3	1	0	0	0	0	0	0	3	1	0	0	0	0	0	0	6.8%	2.0%	0.0%	0.0%	0.0%	0.0%	0.0%	0.0%
tr|I3LPQ4|I3LPQ4	0	1	0	0	0	0	0	0	0	4	0	0	0	0	0	0	0.0%	4.9%	0.0%	0.0%	0.0%	0.0%	0.0%	0.0%
tr|F1S554|F1S554	3	0	0	0	0	0	0	0	4	0	0	0	0	0	0	0	5.1%	0.0%	0.0%	0.0%	0.0%	0.0%	0.0%	0.0%
tr|F1RV94|F1RV94	2	1	0	0	0	0	0	0	2	2	0	0	0	0	0	0	40.4%	14.9%	0.0%	0.0%	0.0%	0.0%	0.0%	0.0%
tr|I3LGX5|I3LGX5	2	1	0	0	0	0	0	0	3	1	0	0	0	0	0	0	8.4%	3.2%	0.0%	0.0%	0.0%	0.0%	0.0%	0.0%
tr|F1RFR4|F1RFR4	3	0	0	0	0	0	0	0	4	0	0	0	0	0	0	0	11.6%	0.0%	0.0%	0.0%	0.0%	0.0%	0.0%	0.0%
tr|I3L577|I3L577	2	1	0	0	0	0	0	0	2	2	0	0	0	0	0	0	4.5%	2.6%	0.0%	0.0%	0.0%	0.0%	0.0%	0.0%
tr|I3LEK6|I3LEK6	2	1	0	0	0	0	0	0	2	2	0	0	0	0	0	0	26.7%	9.6%	0.0%	0.0%	0.0%	0.0%	0.0%	0.0%
tr|F1RIW5|F1RIW5	1	1	0	0	0	0	0	0	1	3	0	0	0	0	0	0	12.3%	7.4%	0.0%	0.0%	0.0%	0.0%	0.0%	0.0%
tr|F1S459|F1S459	0	1	0	0	0	0	0	0	0	4	0	0	0	0	0	0	0.0%	3.5%	0.0%	0.0%	0.0%	0.0%	0.0%	0.0%
tr|F1S781|F1S781	3	1	0	0	0	0	0	0	3	1	0	0	0	0	0	0	9.3%	2.8%	0.0%	0.0%	0.0%	0.0%	0.0%	0.0%
Reverse_tr|K7GP99|K7GP99	0	1	0	0	0	0	0	0	0	4	0	0	0	0	0	0	0.0%	0.9%	0.0%	0.0%	0.0%	0.0%	0.0%	0.0%
tr|F1RKV6|F1RKV6	2	1	0	0	0	0	0	0	2	2	0	0	0	0	0	0	4.5%	1.6%	0.0%	0.0%	0.0%	0.0%	0.0%	0.0%
tr|F1RXT9|F1RXT9	2	1	0	0	0	0	0	0	3	1	0	0	0	0	0	0	2.6%	1.3%	0.0%	0.0%	0.0%	0.0%	0.0%	0.0%
tr|F1RRK0|F1RRK0	4	0	0	0	0	0	0	0	4	0	0	0	0	0	0	0	6.3%	0.0%	0.0%	0.0%	0.0%	0.0%	0.0%	0.0%
tr|F1S9S6|F1S9S6	2	1	0	0	0	0	0	0	2	2	0	0	0	0	0	0	8.6%	2.5%	0.0%	0.0%	0.0%	0.0%	0.0%	0.0%
tr|F1SBT3|F1SBT3	3	0	0	0	0	0	0	0	4	0	0	0	0	0	0	0	31.7%	0.0%	0.0%	0.0%	0.0%	0.0%	0.0%	0.0%
tr|F1RXC5|F1RXC5	3	0	0	0	0	0	0	0	4	0	0	0	0	0	0	0	9.8%	0.0%	0.0%	0.0%	0.0%	0.0%	0.0%	0.0%
tr|F1S6R5|F1S6R5	2	1	0	0	0	0	0	0	2	2	0	0	0	0	0	0	6.9%	3.9%	0.0%	0.0%	0.0%	0.0%	0.0%	0.0%
tr|F1RVX2|F1RVX2	2	0	0	0	0	0	0	0	4	0	0	0	0	0	0	0	27.3%	0.0%	0.0%	0.0%	0.0%	0.0%	0.0%	0.0%
tr|I3LSR2|I3LSR2	1	1	0	0	0	0	0	0	1	3	0	0	0	0	0	0	2.2%	2.2%	0.0%	0.0%	0.0%	0.0%	0.0%	0.0%
tr|F1SUG8|F1SUG8	3	0	0	0	0	0	0	0	4	0	0	0	0	0	0	0	7.6%	0.0%	0.0%	0.0%	0.0%	0.0%	0.0%	0.0%
tr|F1SA22|F1SA22	0	1	0	0	0	0	0	0	0	4	0	0	0	0	0	0	0.0%	0.5%	0.0%	0.0%	0.0%	0.0%	0.0%	0.0%
tr|F1SCM0|F1SCM0	2	0	0	0	0	0	0	0	4	0	0	0	0	0	0	0	25.0%	0.0%	0.0%	0.0%	0.0%	0.0%	0.0%	0.0%
tr|F1RPX2|F1RPX2	1	1	0	0	0	0	0	0	2	2	0	0	0	0	0	0	2.7%	1.4%	0.0%	0.0%	0.0%	0.0%	0.0%	0.0%
tr|I3LPS7|I3LPS7	0	1	0	0	0	0	0	0	0	4	0	0	0	0	0	0	0.0%	9.8%	0.0%	0.0%	0.0%	0.0%	0.0%	0.0%
tr|F1SE91|F1SE91	1	1	0	0	0	0	0	0	1	3	0	0	0	0	0	0	2.9%	1.0%	0.0%	0.0%	0.0%	0.0%	0.0%	0.0%
tr|I3LP59|I3LP59	0	1	0	0	0	0	0	0	0	4	0	0	0	0	0	0	0.0%	11.1%	0.0%	0.0%	0.0%	0.0%	0.0%	0.0%
tr|I3LM96|I3LM96	2	0	0	0	0	0	0	0	4	0	0	0	0	0	0	0	13.7%	0.0%	0.0%	0.0%	0.0%	0.0%	0.0%	0.0%
tr|I3L5Z9|I3L5Z9	2	2	0	0	0	0	0	0	2	2	0	0	0	0	0	0	2.0%	1.8%	0.0%	0.0%	0.0%	0.0%	0.0%	0.0%
tr|I3L5U1|I3L5U1	0	1	0	0	0	0	0	0	0	4	0	0	0	0	0	0	0.0%	4.0%	0.0%	0.0%	0.0%	0.0%	0.0%	0.0%
tr|K7GL95|K7GL95	2	0	0	0	0	0	0	0	4	0	0	0	0	0	0	0	16.7%	0.0%	0.0%	0.0%	0.0%	0.0%	0.0%	0.0%
tr|I3LVG2|I3LVG2	2	1	0	0	0	0	0	0	3	1	0	0	0	0	0	0	9.5%	1.7%	0.0%	0.0%	0.0%	0.0%	0.0%	0.0%
tr|I3LFK7|I3LFK7	2	1	0	0	0	0	0	0	2	2	0	0	0	0	0	0	4.5%	1.8%	0.0%	0.0%	0.0%	0.0%	0.0%	0.0%
Reverse_tr|F1S585|F1S585	0	1	0	0	0	0	0	0	0	4	0	0	0	0	0	0	0.0%	1.0%	0.0%	0.0%	0.0%	0.0%	0.0%	0.0%
tr|I3LJV8|I3LJV8	4	0	0	0	0	0	0	0	4	0	0	0	0	0	0	0	27.5%	0.0%	0.0%	0.0%	0.0%	0.0%	0.0%	0.0%
tr|F1RKM4|F1RKM4	1	1	0	0	0	0	0	0	1	3	0	0	0	0	0	0	2.8%	2.8%	0.0%	0.0%	0.0%	0.0%	0.0%	0.0%
tr|F1RUJ4|F1RUJ4	2	0	0	0	0	0	0	0	4	0	0	0	0	0	0	0	3.2%	0.0%	0.0%	0.0%	0.0%	0.0%	0.0%	0.0%
A1Z623	2	0	0	0	0	0	0	0	4	0	0	0	0	0	0	0	15.4%	0.0%	0.0%	0.0%	0.0%	0.0%	0.0%	0.0%
tr|I3LMQ8|I3LMQ8	4	0	0	0	0	0	0	0	4	0	0	0	0	0	0	0	19.1%	0.0%	0.0%	0.0%	0.0%	0.0%	0.0%	0.0%
tr|F1S8Y4|F1S8Y4	1	1	0	0	0	0	0	0	3	1	0	0	0	0	0	0	7.1%	7.1%	0.0%	0.0%	0.0%	0.0%	0.0%	0.0%
tr|F1SUQ2|F1SUQ2	1	2	0	0	0	0	0	0	1	3	0	0	0	0	0	0	10.0%	8.3%	0.0%	0.0%	0.0%	0.0%	0.0%	0.0%
tr|I3LVA4|I3LVA4	0	1	0	0	0	0	0	0	0	4	0	0	0	0	0	0	0.0%	4.4%	0.0%	0.0%	0.0%	0.0%	0.0%	0.0%
tr|K7GMC4|K7GMC4	2	0	0	0	0	0	0	0	4	0	0	0	0	0	0	0	9.1%	0.0%	0.0%	0.0%	0.0%	0.0%	0.0%	0.0%
tr|F1S346|F1S346	2	1	0	0	0	0	0	0	3	1	0	0	0	0	0	0	2.1%	1.4%	0.0%	0.0%	0.0%	0.0%	0.0%	0.0%
tr|F1SBH3|F1SBH3	3	0	0	0	0	0	0	0	4	0	0	0	0	0	0	0	15.6%	0.0%	0.0%	0.0%	0.0%	0.0%	0.0%	0.0%
Q8WN98	4	0	0	0	0	0	0	0	4	0	0	0	0	0	0	0	21.6%	0.0%	0.0%	0.0%	0.0%	0.0%	0.0%	0.0%
tr|I3LU87|I3LU87	0	1	0	0	0	0	0	0	0	4	0	0	0	0	0	0	0.0%	1.7%	0.0%	0.0%	0.0%	0.0%	0.0%	0.0%
tr|I3LIY8|I3LIY8	2	1	0	0	0	0	0	0	3	1	0	0	0	0	0	0	3.2%	2.0%	0.0%	0.0%	0.0%	0.0%	0.0%	0.0%
tr|F1S4P7|F1S4P7	4	0	0	0	0	0	0	0	4	0	0	0	0	0	0	0	17.9%	0.0%	0.0%	0.0%	0.0%	0.0%	0.0%	0.0%
tr|F1RVY4|F1RVY4	3	0	0	0	0	0	0	0	4	0	0	0	0	0	0	0	4.9%	0.0%	0.0%	0.0%	0.0%	0.0%	0.0%	0.0%
Reverse_tr|K7GQX0|K7GQX0	0	1	0	0	0	0	0	0	0	4	0	0	0	0	0	0	0.0%	2.5%	0.0%	0.0%	0.0%	0.0%	0.0%	0.0%
tr|F1RHS7|F1RHS7	2	1	0	0	0	0	0	0	3	1	0	0	0	0	0	0	11.1%	3.3%	0.0%	0.0%	0.0%	0.0%	0.0%	0.0%
tr|F1SBP3|F1SBP3	2	0	0	0	0	0	0	0	4	0	0	0	0	0	0	0	2.5%	0.0%	0.0%	0.0%	0.0%	0.0%	0.0%	0.0%
A5GFQ5	2	0	0	0	0	0	0	0	4	0	0	0	0	0	0	0	12.7%	0.0%	0.0%	0.0%	0.0%	0.0%	0.0%	0.0%
tr|F1S6G0|F1S6G0	3	1	0	0	0	0	0	0	3	1	0	0	0	0	0	0	6.7%	1.4%	0.0%	0.0%	0.0%	0.0%	0.0%	0.0%
tr|F1RR48|F1RR48	2	1	0	0	0	0	0	0	3	1	0	0	0	0	0	0	6.9%	6.1%	0.0%	0.0%	0.0%	0.0%	0.0%	0.0%
tr|I3LPF3|I3LPF3	2	2	0	0	0	0	0	0	2	2	0	0	0	0	0	0	2.0%	1.8%	0.0%	0.0%	0.0%	0.0%	0.0%	0.0%
Q8HY46	0	1	0	0	0	0	0	0	0	4	0	0	0	0	0	0	0.0%	1.3%	0.0%	0.0%	0.0%	0.0%	0.0%	0.0%
tr|F1SDM6|F1SDM6	3	0	0	0	0	0	0	0	4	0	0	0	0	0	0	0	12.3%	0.0%	0.0%	0.0%	0.0%	0.0%	0.0%	0.0%
tr|F1RLD6|F1RLD6	0	1	0	0	0	0	0	0	0	4	0	0	0	0	0	0	0.0%	4.2%	0.0%	0.0%	0.0%	0.0%	0.0%	0.0%
tr|F1SEX1|F1SEX1	2	1	0	0	0	0	0	0	3	1	0	0	0	0	0	0	12.6%	4.5%	0.0%	0.0%	0.0%	0.0%	0.0%	0.0%
tr|F1S3N2|F1S3N2	2	1	0	0	0	0	0	0	3	1	0	0	0	0	0	0	18.4%	9.8%	0.0%	0.0%	0.0%	0.0%	0.0%	0.0%
tr|I3LVT3|I3LVT3	0	1	0	0	0	0	0	0	0	4	0	0	0	0	0	0	0.0%	6.0%	0.0%	0.0%	0.0%	0.0%	0.0%	0.0%
tr|F1SL59|F1SL59	3	0	0	0	0	0	0	0	4	0	0	0	0	0	0	0	3.1%	0.0%	0.0%	0.0%	0.0%	0.0%	0.0%	0.0%
tr|I3L6Y9|I3L6Y9	2	1	0	0	0	0	0	0	2	2	0	0	0	0	0	0	2.3%	1.3%	0.0%	0.0%	0.0%	0.0%	0.0%	0.0%
tr|F1RRU6|F1RRU6	0	1	0	0	0	0	0	0	0	4	0	0	0	0	0	0	0.0%	1.5%	0.0%	0.0%	0.0%	0.0%	0.0%	0.0%
tr|F1SDX8|F1SDX8	3	0	0	0	0	0	0	0	4	0	0	0	0	0	0	0	18.9%	0.0%	0.0%	0.0%	0.0%	0.0%	0.0%	0.0%
tr|F1S484|F1S484	0	1	0	0	0	0	0	0	0	4	0	0	0	0	0	0	0.0%	2.6%	0.0%	0.0%	0.0%	0.0%	0.0%	0.0%
tr|F1SQJ7|F1SQJ7	2	2	0	0	0	0	0	0	2	2	0	0	0	0	0	0	2.9%	2.1%	0.0%	0.0%	0.0%	0.0%	0.0%	0.0%

	Peptide Count								Spectral Count								Sequence Coverage							
	Whole Cell	Whole Cell	Whole Cell	Cilia Fraction	Cilia Fraction	Cilia Fraction	Cilia Fraction	Cilia Fraction	Whole Cell	Whole Cell	Whole Cell	Cilia Fraction	Cilia Fraction	Cilia Fraction	Cilia Fraction	Cilia Fraction	Whole Cell	Whole Cell	Whole Cell	Cilia Fraction	Cilia Fraction	Cilia Fraction	Cilia Fraction	Cilia Fraction
Sample#	1	2	6	4	3	5	7	8	1	2	6	4	3	5	7	8	1	2	6	4	3	5	7	8
**Accession**																								
tr|I3LEL0|I3LEL0	3	1	0	0	0	0	0	0	3	1	0	0	0	0	0	0	4.5%	1.6%	0.0%	0.0%	0.0%	0.0%	0.0%	0.0%
tr|F1RI89|F1RI89	2	1	0	0	0	0	0	0	2	2	0	0	0	0	0	0	4.4%	4.0%	0.0%	0.0%	0.0%	0.0%	0.0%	0.0%
tr|F1SBM2|F1SBM2	2	1	0	0	0	0	0	0	2	2	0	0	0	0	0	0	8.0%	1.8%	0.0%	0.0%	0.0%	0.0%	0.0%	0.0%
tr|F1S4N5|F1S4N5	1	1	0	0	0	0	0	0	1	3	0	0	0	0	0	0	6.1%	6.1%	0.0%	0.0%	0.0%	0.0%	0.0%	0.0%
tr|I3LKD2|I3LKD2	2	1	0	0	0	0	0	0	2	2	0	0	0	0	0	0	18.1%	5.2%	0.0%	0.0%	0.0%	0.0%	0.0%	0.0%
tr|F1SKJ2|F1SKJ2	3	1	0	0	0	0	0	0	3	1	0	0	0	0	0	0	22.3%	12.7%	0.0%	0.0%	0.0%	0.0%	0.0%	0.0%
tr|I3LII0|I3LII0	2	0	0	0	0	0	0	0	4	0	0	0	0	0	0	0	12.3%	0.0%	0.0%	0.0%	0.0%	0.0%	0.0%	0.0%
tr|I3LS97|I3LS97	2	0	0	0	0	0	0	0	4	0	0	0	0	0	0	0	19.8%	0.0%	0.0%	0.0%	0.0%	0.0%	0.0%	0.0%
tr|F1RTZ5|F1RTZ5	4	0	1	0	0	0	0	0	4	0	1	0	0	0	0	0	9.4%	0.0%	2.0%	0.0%	0.0%	0.0%	0.0%	0.0%
tr|F1S4Z2|F1S4Z2	2	1	1	0	0	0	0	0	2	2	1	0	0	0	0	0	12.9%	7.1%	4.5%	0.0%	0.0%	0.0%	0.0%	0.0%
tr|I3L5J0|I3L5J0	2	0	1	0	0	0	0	0	4	0	1	0	0	0	0	0	19.1%	0.0%	6.7%	0.0%	0.0%	0.0%	0.0%	0.0%
tr|F1SSZ9|F1SSZ9	2	1	1	0	0	0	0	0	2	2	1	0	0	0	0	0	2.6%	1.3%	2.2%	0.0%	0.0%	0.0%	0.0%	0.0%
tr|F1SEE2|F1SEE2	1	1	1	0	0	0	0	0	1	3	1	0	0	0	0	0	3.9%	3.9%	7.6%	0.0%	0.0%	0.0%	0.0%	0.0%
tr|F1S4P1|F1S4P1	3	1	1	0	0	0	0	0	3	1	1	0	0	0	0	0	2.6%	0.8%	0.7%	0.0%	0.0%	0.0%	0.0%	0.0%
tr|F1SUK2|F1SUK2	3	1	1	0	0	0	0	0	3	1	1	0	0	0	0	0	8.3%	2.9%	1.7%	0.0%	0.0%	0.0%	0.0%	0.0%
Q29551	2	1	1	0	0	0	0	0	3	1	1	0	0	0	0	0	7.9%	5.4%	5.2%	0.0%	0.0%	0.0%	0.0%	0.0%
tr|F1SQJ5|F1SQJ5	3	0	1	0	0	0	0	0	4	0	1	0	0	0	0	0	12.8%	0.0%	2.7%	0.0%	0.0%	0.0%	0.0%	0.0%
tr|I3L6C1|I3L6C1	1	2	1	0	0	0	0	0	2	2	1	0	0	0	0	0	0.9%	2.3%	1.2%	0.0%	0.0%	0.0%	0.0%	0.0%
tr|F1RZV7|F1RZV7	2	1	1	0	0	0	0	0	2	2	1	0	0	0	0	0	5.6%	1.7%	3.1%	0.0%	0.0%	0.0%	0.0%	0.0%
tr|F1SCR9|F1SCR9	1	1	1	0	0	0	0	0	1	3	1	0	0	0	0	0	2.5%	1.9%	1.8%	0.0%	0.0%	0.0%	0.0%	0.0%
tr|F1S386|F1S386	3	0	1	0	0	0	0	0	4	0	2	0	0	0	0	0	8.8%	0.0%	7.4%	0.0%	0.0%	0.0%	0.0%	0.0%
tr|F1RT57|F1RT57	4	0	2	0	0	0	0	0	4	0	2	0	0	0	0	0	7.9%	0.0%	2.8%	0.0%	0.0%	0.0%	0.0%	0.0%
tr|I3LDM7|I3LDM7	2	1	1	0	0	0	0	0	2	2	2	0	0	0	0	0	14.7%	9.8%	14.7%	0.0%	0.0%	0.0%	0.0%	0.0%
tr|F1S145|F1S145	1	0	1	0	0	0	0	0	4	0	2	0	0	0	0	0	11.4%	0.0%	11.4%	0.0%	0.0%	0.0%	0.0%	0.0%
tr|F1RMY9|F1RMY9	3	0	1	0	0	0	0	0	4	0	2	0	0	0	0	0	8.8%	0.0%	3.5%	0.0%	0.0%	0.0%	0.0%	0.0%
tr|I3LUF8|I3LUF8	2	2	1	0	0	0	0	0	2	2	2	0	0	0	0	0	9.7%	4.8%	5.4%	0.0%	0.0%	0.0%	0.0%	0.0%
tr|F1RT06|F1RT06	0	3	1	0	0	0	0	0	0	4	2	0	0	0	0	0	0.0%	12.5%	6.5%	0.0%	0.0%	0.0%	0.0%	0.0%
tr|F1RNM4|F1RNM4	3	0	1	0	0	0	0	0	4	0	2	0	0	0	0	0	9.1%	0.0%	3.6%	0.0%	0.0%	0.0%	0.0%	0.0%
tr|F1SIP9|F1SIP9	3	0	1	0	0	0	0	0	4	0	3	0	0	0	0	0	4.7%	0.0%	1.7%	0.0%	0.0%	0.0%	0.0%	0.0%
tr|F1S8W2|F1S8W2	0	2	1	0	0	0	0	0	0	4	3	0	0	0	0	0	0.0%	3.8%	4.0%	0.0%	0.0%	0.0%	0.0%	0.0%
tr|I3LNZ2|I3LNZ2	1	2	1	0	0	0	0	0	2	2	3	0	0	0	0	0	6.7%	11.3%	9.8%	0.0%	0.0%	0.0%	0.0%	0.0%
tr|F1SQ44|F1SQ44	2	0	1	0	0	0	0	0	4	0	3	0	0	0	0	0	10.1%	0.0%	11.5%	0.0%	0.0%	0.0%	0.0%	0.0%
tr|I3LSY2|I3LSY2	3	0	1	0	0	0	0	0	4	0	3	0	0	0	0	0	4.8%	0.0%	1.8%	0.0%	0.0%	0.0%	0.0%	0.0%
tr|F1RJ88|F1RJ88	1	2	1	0	0	0	0	0	1	3	3	0	0	0	0	0	1.3%	2.2%	1.3%	0.0%	0.0%	0.0%	0.0%	0.0%
tr|I3LUZ8|I3LUZ8	2	0	2	0	0	0	0	0	4	0	3	0	0	0	0	0	31.9%	0.0%	24.5%	0.0%	0.0%	0.0%	0.0%	0.0%
tr|F1SIE7|F1SIE7	2	1	2	0	0	0	0	0	3	1	3	0	0	0	0	0	6.1%	2.8%	3.9%	0.0%	0.0%	0.0%	0.0%	0.0%
tr|F2Z5T8|F2Z5T8	3	0	1	0	0	0	0	0	4	0	3	0	0	0	0	0	31.7%	0.0%	9.7%	0.0%	0.0%	0.0%	0.0%	0.0%
tr|F1S766|F1S766	2	0	1	0	0	0	0	0	4	0	3	0	0	0	0	0	10.3%	0.0%	11.6%	0.0%	0.0%	0.0%	0.0%	0.0%
tr|F1RUE3|F1RUE3	3	0	1	0	0	0	0	0	4	0	3	0	0	0	0	0	7.4%	0.0%	3.5%	0.0%	0.0%	0.0%	0.0%	0.0%
tr|F1RS16|F1RS16	1	1	1	0	0	0	0	0	1	3	3	0	0	0	0	0	1.4%	1.7%	3.2%	0.0%	0.0%	0.0%	0.0%	0.0%
tr|I3L9X2|I3L9X2	1	1	1	0	0	0	0	0	2	2	3	0	0	0	0	0	1.7%	1.7%	2.3%	0.0%	0.0%	0.0%	0.0%	0.0%
tr|F1SIR4|F1SIR4	3	0	1	0	0	0	0	0	4	0	3	0	0	0	0	0	2.9%	0.0%	1.1%	0.0%	0.0%	0.0%	0.0%	0.0%
tr|I3LKU9|I3LKU9	2	1	2	0	0	0	0	0	3	1	3	0	0	0	0	0	13.5%	4.7%	8.1%	0.0%	0.0%	0.0%	0.0%	0.0%
tr|I3L9C3|I3L9C3	1	1	1	0	0	0	0	0	1	3	4	0	0	0	0	0	14.4%	3.1%	7.7%	0.0%	0.0%	0.0%	0.0%	0.0%
tr|F1SNX8|F1SNX8	4	0	1	0	0	0	0	0	4	0	4	0	0	0	0	0	10.9%	0.0%	3.7%	0.0%	0.0%	0.0%	0.0%	0.0%
tr|F1RTV6|F1RTV6	3	1	2	0	0	0	0	0	3	1	4	0	0	0	0	0	12.7%	8.1%	4.9%	0.0%	0.0%	0.0%	0.0%	0.0%
tr|K7GP19|K7GP19	4	0	1	0	0	0	0	0	4	0	4	0	0	0	0	0	18.8%	0.0%	9.7%	0.0%	0.0%	0.0%	0.0%	0.0%
tr|F1RF76|F1RF76	3	0	1	0	0	0	0	0	4	0	4	0	0	0	0	0	6.5%	0.0%	2.6%	0.0%	0.0%	0.0%	0.0%	0.0%
tr|I3LQA8|I3LQA8	4	0	1	0	0	0	0	0	4	0	4	0	0	0	0	0	14.9%	0.0%	6.4%	0.0%	0.0%	0.0%	0.0%	0.0%
tr|I3L6J1|I3L6J1	3	0	2	0	0	0	0	0	4	0	4	0	0	0	0	0	4.4%	0.0%	5.8%	0.0%	0.0%	0.0%	0.0%	0.0%
tr|F1SJ69|F1SJ69	0	1	1	0	0	0	0	0	0	4	5	0	0	0	0	0	0.0%	0.6%	1.5%	0.0%	0.0%	0.0%	0.0%	0.0%
tr|F1RYH8|F1RYH8	4	0	2	0	0	0	0	0	4	0	5	0	0	0	0	0	21.6%	0.0%	7.1%	0.0%	0.0%	0.0%	0.0%	0.0%
tr|F1S9W3|F1S9W3	1	1	2	0	0	0	0	0	1	3	5	0	0	0	0	0	1.5%	1.6%	2.9%	0.0%	0.0%	0.0%	0.0%	0.0%
tr|I3LCI4|I3LCI4	1	1	2	0	0	0	0	0	1	3	5	0	0	0	0	0	3.4%	2.5%	12.9%	0.0%	0.0%	0.0%	0.0%	0.0%
tr|F1S8A5|F1S8A5	3	0	1	0	0	0	0	0	4	0	5	0	0	0	0	0	19.0%	0.0%	8.8%	0.0%	0.0%	0.0%	0.0%	0.0%
tr|F1RM10|F1RM10	3	0	3	0	0	0	0	0	4	0	6	0	0	0	0	0	4.8%	0.0%	3.8%	0.0%	0.0%	0.0%	0.0%	0.0%
tr|F1RWL7|F1RWL7	1	0	2	0	0	0	0	0	4	0	6	0	0	0	0	0	8.6%	0.0%	8.6%	0.0%	0.0%	0.0%	0.0%	0.0%
tr|Q06A95|Q06A95	4	0	3	0	0	0	0	0	4	0	6	0	0	0	0	0	14.5%	0.0%	5.5%	0.0%	0.0%	0.0%	0.0%	0.0%
tr|F1RXF7|F1RXF7	3	1	1	0	0	0	0	0	3	1	6	0	0	0	0	0	24.0%	3.1%	7.2%	0.0%	0.0%	0.0%	0.0%	0.0%
tr|F1S2L2|F1S2L2	3	0	1	0	0	0	0	0	4	0	6	0	0	0	0	0	8.5%	0.0%	3.3%	0.0%	0.0%	0.0%	0.0%	0.0%
tr|F1S6Q1|F1S6Q1	4	0	1	0	0	0	0	0	4	0	6	0	0	0	0	0	35.4%	0.0%	6.9%	0.0%	0.0%	0.0%	0.0%	0.0%
tr|F1RS21|F1RS21	3	1	2	0	0	0	0	0	3	1	7	0	0	0	0	0	15.0%	2.4%	8.1%	0.0%	0.0%	0.0%	0.0%	0.0%
tr|F1RVH7|F1RVH7	3	0	2	0	0	0	0	0	4	0	7	0	0	0	0	0	18.8%	0.0%	5.7%	0.0%	0.0%	0.0%	0.0%	0.0%
tr|F1SBY5|F1SBY5	3	1	3	0	0	0	0	0	3	1	7	0	0	0	0	0	2.3%	1.0%	2.7%	0.0%	0.0%	0.0%	0.0%	0.0%
tr|I3LET0|I3LET0	1	0	2	0	0	0	0	0	4	0	9	0	0	0	0	0	17.3%	0.0%	33.3%	0.0%	0.0%	0.0%	0.0%	0.0%
tr|I3LAE7|I3LAE7	2	0	2	0	0	0	0	0	4	0	9	0	0	0	0	0	21.7%	0.0%	18.7%	0.0%	0.0%	0.0%	0.0%	0.0%
tr|F1RJE4|F1RJE4	3	1	2	0	0	0	0	0	3	1	9	0	0	0	0	0	7.6%	1.7%	5.1%	0.0%	0.0%	0.0%	0.0%	0.0%
tr|F1S7W3|F1S7W3	2	1	1	0	0	0	0	0	2	2	9	0	0	0	0	0	12.9%	12.1%	19.7%	0.0%	0.0%	0.0%	0.0%	0.0%
tr|F1SQS3|F1SQS3	2	0	3	0	0	0	0	0	4	0	10	0	0	0	0	0	8.0%	0.0%	8.2%	0.0%	0.0%	0.0%	0.0%	0.0%
tr|F1S3E8|F1S3E8	2	1	1	0	0	0	0	0	3	1	10	0	0	0	0	0	4.3%	4.3%	4.9%	0.0%	0.0%	0.0%	0.0%	0.0%
tr|F1S9D1|F1S9D1	3	1	3	0	0	0	0	0	3	1	10	0	0	0	0	0	2.1%	0.8%	3.6%	0.0%	0.0%	0.0%	0.0%	0.0%
tr|I3LRF8|I3LRF8	2	0	2	0	0	0	0	0	4	0	11	0	0	0	0	0	8.7%	0.0%	8.7%	0.0%	0.0%	0.0%	0.0%	0.0%
tr|I3LLG3|I3LLG3	2	0	2	0	0	0	0	0	4	0	11	0	0	0	0	0	11.5%	0.0%	24.0%	0.0%	0.0%	0.0%	0.0%	0.0%
tr|I3LP06|I3LP06	1	1	1	0	0	0	0	0	2	2	11	0	0	0	0	0	8.9%	8.9%	8.9%	0.0%	0.0%	0.0%	0.0%	0.0%
P56471	1	0	1	0	0	0	0	0	4	0	11	0	0	0	0	0	22.2%	0.0%	22.2%	0.0%	0.0%	0.0%	0.0%	0.0%
tr|I3LLH8|I3LLH8	2	1	1	0	0	0	0	0	3	1	13	0	0	0	0	0	16.3%	11.0%	5.8%	0.0%	0.0%	0.0%	0.0%	0.0%
tr|F1SPJ5|F1SPJ5	3	1	2	0	0	0	0	0	3	1	14	0	0	0	0	0	16.7%	4.8%	11.9%	0.0%	0.0%	0.0%	0.0%	0.0%
tr|I3LS80|I3LS80	1	1	2	0	0	0	0	0	2	2	15	0	0	0	0	0	22.2%	11.1%	14.8%	0.0%	0.0%	0.0%	0.0%	0.0%
tr|F1RP04|F1RP04	2	0	4	0	0	0	0	0	4	0	16	0	0	0	0	0	8.4%	0.0%	11.9%	0.0%	0.0%	0.0%	0.0%	0.0%
tr|F1SD70|F1SD70	3	0	4	0	0	0	0	0	4	0	20	0	0	0	0	0	7.8%	0.0%	10.4%	0.0%	0.0%	0.0%	0.0%	0.0%
tr|F1RQH2|F1RQH2	4	0	6	0	0	0	0	0	4	0	39	0	0	0	0	0	6.4%	0.0%	7.4%	0.0%	0.0%	0.0%	0.0%	0.0%

	Peptide Count								Spectral Count								Sequence Coverage							
	Whole Cell	Whole Cell	Whole Cell	Cilia Fraction	Cilia Fraction	Cilia Fraction	Cilia Fraction	Cilia Fraction	Whole Cell	Whole Cell	Whole Cell	Cilia Fraction	Cilia Fraction	Cilia Fraction	Cilia Fraction	Cilia Fraction	Whole Cell	Whole Cell	Whole Cell	Cilia Fraction	Cilia Fraction	Cilia Fraction	Cilia Fraction	Cilia Fraction
Sample#	1	2	6	4	3	5	7	8	1	2	6	4	3	5	7	8	1	2	6	4	3	5	7	8
**Accession**																								
tr|I3LGQ4|I3LGQ4	3	0	3	0	0	0	0	0	4	0	115	0	0	0	0	0	37.5%	0.0%	37.5%	0.0%	0.0%	0.0%	0.0%	0.0%
tr|F1RV70|F1RV70	2	1	2	0	0	0	0	0	3	1	136	0	0	0	0	0	10.2%	3.3%	7.7%	0.0%	0.0%	0.0%	0.0%	0.0%
tr|K7GMG4|K7GMG4	0	1	0	0	0	0	1	1	0	3	0	0	0	0	29	13	0.0%	19.1%	0.0%	0.0%	0.0%	0.0%	19.1%	19.1%
tr|F1RZB0|F1RZB0	3	0	1	0	0	0	0	10	3	0	1	0	0	0	0	31	5.6%	0.0%	3.4%	0.0%	0.0%	0.0%	0.0%	16.6%
tr|F1RVP8|F1RVP8	2	0	0	0	0	0	2	4	3	0	0	0	0	0	7	16	4.9%	0.0%	0.0%	0.0%	0.0%	0.0%	5.7%	10.5%
Q29268	3	0	1	0	0	0	1	6	3	0	2	0	0	0	5	33	39.0%	0.0%	14.4%	0.0%	0.0%	0.0%	15.3%	39.0%
tr|Q31072|Q31072	3	0	1	0	0	0	2	4	3	0	2	0	0	0	9	19	10.2%	0.0%	4.1%	0.0%	0.0%	0.0%	8.6%	14.3%
tr|F1RQ23|F1RQ23	3	0	0	0	0	0	1	3	3	0	0	0	0	0	2	12	11.4%	0.0%	0.0%	0.0%	0.0%	0.0%	5.1%	11.4%
tr|I3LDU9|I3LDU9	3	0	1	0	0	0	1	6	3	0	4	0	0	0	1	27	7.4%	0.0%	3.0%	0.0%	0.0%	0.0%	1.9%	16.7%
tr|I3LI91|I3LI91	1	1	0	0	0	0	1	3	1	2	0	0	0	0	3	9	5.1%	3.3%	0.0%	0.0%	0.0%	0.0%	3.3%	11.4%
tr|I3LSQ2|I3LSQ2	1	1	0	0	0	0	1	3	1	2	0	0	0	0	3	9	5.1%	3.3%	0.0%	0.0%	0.0%	0.0%	3.3%	11.5%
tr|F1RWH1|F1RWH1	1	1	2	0	0	0	2	1	1	2	6	0	0	0	27	1	8.6%	8.6%	9.7%	0.0%	0.0%	0.0%	24.3%	8.6%
tr|F1S4V8|F1S4V8	2	0	0	0	0	0	2	1	3	0	0	0	0	0	8	1	7.6%	0.0%	0.0%	0.0%	0.0%	0.0%	7.6%	3.7%
tr|I3LMD6|I3LMD6	2	0	0	0	0	0	0	4	3	0	0	0	0	0	0	8	3.3%	0.0%	0.0%	0.0%	0.0%	0.0%	0.0%	5.8%
tr|F1STQ3|F1STQ3	2	0	1	0	0	0	0	5	3	0	5	0	0	0	0	21	2.0%	0.0%	3.5%	0.0%	0.0%	0.0%	0.0%	7.9%
P48762	2	0	1	0	0	0	0	5	3	0	5	0	0	0	0	21	2.0%	0.0%	3.5%	0.0%	0.0%	0.0%	0.0%	7.9%
tr|M9MMN8|M9MMN8	2	0	0	0	0	0	0	3	3	0	0	0	0	0	0	7	3.8%	0.0%	0.0%	0.0%	0.0%	0.0%	0.0%	5.1%
tr|F1RZ68|F1RZ68	2	0	0	0	0	0	0	2	3	0	0	0	0	0	0	7	4.1%	0.0%	0.0%	0.0%	0.0%	0.0%	0.0%	4.1%
tr|F1RZ67|F1RZ67	2	0	0	0	0	0	0	2	3	0	0	0	0	0	0	7	4.2%	0.0%	0.0%	0.0%	0.0%	0.0%	0.0%	4.2%
tr|F1RFH5|F1RFH5	2	0	0	0	0	0	1	2	3	0	0	0	0	0	3	2	9.7%	0.0%	0.0%	0.0%	0.0%	0.0%	4.2%	8.1%
tr|F2Z5F9|F2Z5F9	1	0	1	0	0	0	0	2	3	0	3	0	0	0	0	8	3.3%	0.0%	4.6%	0.0%	0.0%	0.0%	0.0%	10.5%
tr|F1RQC2|F1RQC2	2	1	0	0	0	0	0	2	2	1	0	0	0	0	0	4	3.8%	2.7%	0.0%	0.0%	0.0%	0.0%	0.0%	7.5%
tr|I3LUW0|I3LUW0	1	0	0	0	0	0	2	0	3	0	0	0	0	0	4	0	11.8%	0.0%	0.0%	0.0%	0.0%	0.0%	11.8%	0.0%
tr|I3LVQ2|I3LVQ2	2	0	1	0	0	0	0	3	3	0	4	0	0	0	0	8	7.2%	0.0%	5.0%	0.0%	0.0%	0.0%	0.0%	8.7%
tr|I3LBF5|I3LBF5	2	0	0	0	0	0	1	0	3	0	0	0	0	0	3	0	7.8%	0.0%	0.0%	0.0%	0.0%	0.0%	6.6%	0.0%
tr|I3LS87|I3LS87	3	0	0	0	0	0	0	2	3	0	0	0	0	0	0	3	7.4%	0.0%	0.0%	0.0%	0.0%	0.0%	0.0%	9.6%
tr|I3LHF2|I3LHF2	1	0	0	0	0	0	1	0	3	0	0	0	0	0	3	0	15.1%	0.0%	0.0%	0.0%	0.0%	0.0%	15.1%	0.0%
tr|I3LE14|I3LE14	1	0	0	0	0	0	0	1	3	0	0	0	0	0	0	3	11.8%	0.0%	0.0%	0.0%	0.0%	0.0%	0.0%	11.8%
tr|I3LJG1|I3LJG1	3	0	1	0	0	0	0	2	3	0	3	0	0	0	0	5	26.9%	0.0%	10.8%	0.0%	0.0%	0.0%	0.0%	11.3%
tr|F1S9T0|F1S9T0	2	0	1	0	0	0	0	1	3	0	1	0	0	0	0	3	9.6%	0.0%	5.2%	0.0%	0.0%	0.0%	0.0%	4.4%
tr|F1RGU8|F1RGU8	3	0	0	0	0	0	0	2	3	0	0	0	0	0	0	2	26.5%	0.0%	0.0%	0.0%	0.0%	0.0%	0.0%	19.6%
tr|I3L5P2|I3L5P2	2	0	0	0	0	0	1	0	3	0	0	0	0	0	2	0	4.3%	0.0%	0.0%	0.0%	0.0%	0.0%	5.7%	0.0%
tr|I3LNF8|I3LNF8	3	0	0	0	0	0	0	1	3	0	0	0	0	0	0	2	4.1%	0.0%	0.0%	0.0%	0.0%	0.0%	0.0%	1.8%
tr|F1RRB6|F1RRB6	1	1	0	0	0	0	0	1	1	2	0	0	0	0	0	2	13.9%	5.0%	0.0%	0.0%	0.0%	0.0%	0.0%	13.9%
tr|F2Z5C0|F2Z5C0	1	1	0	0	0	0	1	1	2	1	0	0	0	0	1	1	8.1%	8.1%	0.0%	0.0%	0.0%	0.0%	8.1%	8.1%
tr|I3LJ76|I3LJ76	1	0	0	0	0	0	0	1	3	0	0	0	0	0	0	2	19.8%	0.0%	0.0%	0.0%	0.0%	0.0%	0.0%	19.8%
P20460	2	0	0	0	0	0	1	0	3	0	0	0	0	0	2	0	25.7%	0.0%	0.0%	0.0%	0.0%	0.0%	18.6%	0.0%
tr|F1SP28|F1SP28	2	0	0	0	0	0	0	1	3	0	0	0	0	0	0	2	8.8%	0.0%	0.0%	0.0%	0.0%	0.0%	0.0%	8.8%
tr|F1S3V2|F1S3V2	0	1	0	0	0	0	1	1	0	3	0	0	0	0	1	1	0.0%	2.1%	0.0%	0.0%	0.0%	0.0%	4.1%	4.1%
tr|F1S0X9|F1S0X9	1	1	0	0	0	0	0	1	2	1	0	0	0	0	0	2	4.6%	4.6%	0.0%	0.0%	0.0%	0.0%	0.0%	4.6%
tr|F1SJE2|F1SJE2	2	1	2	0	0	0	0	4	2	1	7	0	0	0	0	6	2.9%	1.0%	2.6%	0.0%	0.0%	0.0%	0.0%	6.4%
tr|I7KJP5|I7KJP5	3	0	2	0	0	0	1	0	3	0	19	0	0	0	12	0	22.3%	0.0%	9.6%	0.0%	0.0%	0.0%	9.6%	0.0%
tr|F1S5M9|F1S5M9	1	1	3	0	0	0	0	5	1	2	12	0	0	0	0	8	3.4%	2.5%	11.5%	0.0%	0.0%	0.0%	0.0%	11.2%
tr|I3L8Y2|I3L8Y2	2	0	1	0	0	0	2	0	3	0	9	0	0	0	6	0	26.4%	0.0%	18.9%	0.0%	0.0%	0.0%	34.0%	0.0%
tr|F1SKI8|F1SKI8	2	1	1	0	0	0	1	1	2	1	3	0	0	0	2	1	6.3%	1.5%	1.9%	0.0%	0.0%	0.0%	1.5%	2.9%
tr|F1RJH3|F1RJH3	1	1	1	0	0	0	0	1	1	2	1	0	0	0	0	2	3.6%	5.6%	7.9%	0.0%	0.0%	0.0%	0.0%	5.6%
tr|F1RFS8|F1RFS8	3	0	1	0	0	0	0	1	3	0	3	0	0	0	0	2	13.7%	0.0%	4.1%	0.0%	0.0%	0.0%	0.0%	4.1%
tr|I3LVK7|I3LVK7	1	1	0	0	0	0	0	1	2	1	0	0	0	0	0	1	5.0%	5.0%	0.0%	0.0%	0.0%	0.0%	0.0%	14.2%
tr|F1S0A3|F1S0A3	3	0	0	0	0	0	0	1	3	0	0	0	0	0	0	1	10.4%	0.0%	0.0%	0.0%	0.0%	0.0%	0.0%	3.0%
tr|I3LKK5|I3LKK5	2	0	0	0	0	0	0	1	3	0	0	0	0	0	0	1	6.2%	0.0%	0.0%	0.0%	0.0%	0.0%	0.0%	5.2%
tr|F1RKS8|F1RKS8	1	2	0	0	0	0	0	1	1	2	0	0	0	0	0	1	5.7%	6.5%	0.0%	0.0%	0.0%	0.0%	0.0%	5.7%
tr|F1SC49|F1SC49	2	0	0	0	0	0	0	1	3	0	0	0	0	0	0	1	3.1%	0.0%	0.0%	0.0%	0.0%	0.0%	0.0%	1.5%
tr|F1SQY7|F1SQY7	2	1	0	0	0	0	0	1	2	1	0	0	0	0	0	1	4.6%	2.3%	0.0%	0.0%	0.0%	0.0%	0.0%	2.3%
tr|F1RHJ3|F1RHJ3	2	0	0	0	0	0	0	1	3	0	0	0	0	0	0	1	4.7%	0.0%	0.0%	0.0%	0.0%	0.0%	0.0%	4.7%
tr|F1RNN0|F1RNN0	1	1	1	0	0	0	0	2	2	1	10	0	0	0	0	4	5.4%	7.0%	5.4%	0.0%	0.0%	0.0%	0.0%	12.4%
tr|I3LRK6|I3LRK6	2	0	2	0	0	0	0	1	3	0	2	0	0	0	0	1	14.1%	0.0%	14.5%	0.0%	0.0%	0.0%	0.0%	6.6%
tr|I3LC07|I3LC07	3	0	1	0	0	0	0	1	3	0	2	0	0	0	0	1	19.4%	0.0%	7.3%	0.0%	0.0%	0.0%	0.0%	9.2%
tr|F1SUU4|F1SUU4	2	0	3	0	0	0	1	1	3	0	8	0	0	0	1	1	4.8%	0.0%	11.1%	0.0%	0.0%	0.0%	4.0%	4.8%
tr|F1S7J6|F1S7J6	2	1	7	0	0	0	1	1	2	1	47	0	0	0	8	1	3.8%	1.3%	8.3%	0.0%	0.0%	0.0%	3.5%	3.5%
tr|F1SJ27|F1SJ27	2	0	1	0	0	0	0	1	3	0	3	0	0	0	0	1	5.6%	0.0%	3.1%	0.0%	0.0%	0.0%	0.0%	2.0%
tr|F1RK58|F1RK58	1	0	1	0	0	0	0	1	3	0	4	0	0	0	0	1	6.0%	0.0%	5.4%	0.0%	0.0%	0.0%	0.0%	8.2%
tr|K7GSV4|K7GSV4	3	0	2	0	0	0	0	1	3	0	5	0	0	0	0	1	15.0%	0.0%	8.0%	0.0%	0.0%	0.0%	0.0%	5.2%
tr|K7GSS4|K7GSS4	3	0	2	0	0	0	0	1	3	0	5	0	0	0	0	1	14.5%	0.0%	7.7%	0.0%	0.0%	0.0%	0.0%	5.0%
tr|F1RT12|F1RT12	2	1	3	0	0	0	0	1	2	1	14	0	0	0	0	2	2.8%	0.8%	7.2%	0.0%	0.0%	0.0%	0.0%	2.6%
tr|F1SPD2|F1SPD2	3	0	4	0	0	0	1	1	3	0	15	0	0	0	1	1	12.4%	0.0%	12.7%	0.0%	0.0%	0.0%	6.1%	4.3%
tr|F1S2F7|F1S2F7	3	0	1	0	0	0	0	1	3	0	6	0	0	0	0	1	12.6%	0.0%	3.8%	0.0%	0.0%	0.0%	0.0%	3.8%
O77735	3	0	1	0	0	0	0	1	3	0	6	0	0	0	0	1	10.9%	0.0%	3.3%	0.0%	0.0%	0.0%	0.0%	3.3%
tr|I3LIL1|I3LIL1	1	1	2	0	0	0	1	0	1	2	6	0	0	0	1	0	3.5%	1.9%	7.2%	0.0%	0.0%	0.0%	2.9%	0.0%
tr|I3LGU8|I3LGU8	3	0	2	0	0	0	0	1	3	0	7	0	0	0	0	1	4.9%	0.0%	2.1%	0.0%	0.0%	0.0%	0.0%	1.9%
tr|F1RLL9|F1RLL9	3	0	1	0	0	0	0	1	3	0	9	0	0	0	0	1	3.9%	0.0%	1.0%	0.0%	0.0%	0.0%	0.0%	1.2%
tr|I3LJS5|I3LJS5	0	1	2	0	0	0	0	1	0	3	10	0	0	0	0	1	0.0%	3.0%	13.0%	0.0%	0.0%	0.0%	0.0%	7.8%
tr|F1SIB9|F1SIB9	1	1	3	0	0	0	1	0	1	2	11	0	0	0	1	0	3.5%	1.9%	17.3%	0.0%	0.0%	0.0%	2.9%	0.0%
tr|F1RT25|F1RT25	1	1	2	0	0	0	0	1	2	1	13	0	0	0	0	1	8.7%	8.7%	16.5%	0.0%	0.0%	0.0%	0.0%	11.3%
tr|F1S192|F1S192	2	1	2	0	0	0	0	1	2	1	16	0	0	0	0	1	16.9%	7.8%	22.7%	0.0%	0.0%	0.0%	0.0%	12.3%
tr|I3LR69|I3LR69	2	0	5	0	0	0	0	1	3	0	29	0	0	0	0	1	23.2%	0.0%	26.0%	0.0%	0.0%	0.0%	0.0%	5.5%
tr|F1SPH5|F1SPH5	1	2	1	0	0	0	1	0	1	2	105	0	0	0	1	0	12.9%	10.3%	12.9%	0.0%	0.0%	0.0%	12.9%	0.0%
P49171	1	2	1	0	0	0	1	0	1	2	105	0	0	0	1	0	13.0%	10.4%	13.0%	0.0%	0.0%	0.0%	13.0%	0.0%
tr|F1RY43|F1RY43	1	1	0	0	0	0	0	0	1	2	0	0	0	0	0	0	1.7%	1.7%	0.0%	0.0%	0.0%	0.0%	0.0%	0.0%
tr|K7GL84|K7GL84	2	0	0	0	0	0	0	0	3	0	0	0	0	0	0	0	8.8%	0.0%	0.0%	0.0%	0.0%	0.0%	0.0%	0.0%
tr|I3LPY0|I3LPY0	3	0	0	0	0	0	0	0	3	0	0	0	0	0	0	0	22.6%	0.0%	0.0%	0.0%	0.0%	0.0%	0.0%	0.0%
tr|K7GRC8|K7GRC8	0	1	0	0	0	0	0	0	0	3	0	0	0	0	0	0	0.0%	3.2%	0.0%	0.0%	0.0%	0.0%	0.0%	0.0%

	Peptide Count								Spectral Count								Sequence Coverage							
	Whole Cell	Whole Cell	Whole Cell	Cilia Fraction	Cilia Fraction	Cilia Fraction	Cilia Fraction	Cilia Fraction	Whole Cell	Whole Cell	Whole Cell	Cilia Fraction	Cilia Fraction	Cilia Fraction	Cilia Fraction	Cilia Fraction	Whole Cell	Whole Cell	Whole Cell	Cilia Fraction	Cilia Fraction	Cilia Fraction	Cilia Fraction	Cilia Fraction
Sample#	1	2	6	4	3	5	7	8	1	2	6	4	3	5	7	8	1	2	6	4	3	5	7	8
**Accession**																								
tr|F1RKJ4|F1RKJ4	2	0	0	0	0	0	0	0	3	0	0	0	0	0	0	0	16.3%	0.0%	0.0%	0.0%	0.0%	0.0%	0.0%	0.0%
tr|I3LU33|I3LU33	1	1	0	0	0	0	0	0	2	1	0	0	0	0	0	0	26.7%	26.7%	0.0%	0.0%	0.0%	0.0%	0.0%	0.0%
tr|F1RT90|F1RT90	2	1	0	0	0	0	0	0	2	1	0	0	0	0	0	0	3.2%	1.6%	0.0%	0.0%	0.0%	0.0%	0.0%	0.0%
tr|F1RG46|F1RG46	1	1	0	0	0	0	0	0	1	2	0	0	0	0	0	0	3.0%	3.2%	0.0%	0.0%	0.0%	0.0%	0.0%	0.0%
tr|F1RLQ5|F1RLQ5	1	1	0	0	0	0	0	0	1	2	0	0	0	0	0	0	1.8%	2.4%	0.0%	0.0%	0.0%	0.0%	0.0%	0.0%
tr|F1SAK5|F1SAK5	2	0	0	0	0	0	0	0	3	0	0	0	0	0	0	0	5.6%	0.0%	0.0%	0.0%	0.0%	0.0%	0.0%	0.0%
tr|F1S8I4|F1S8I4	0	1	0	0	0	0	0	0	0	3	0	0	0	0	0	0	0.0%	5.1%	0.0%	0.0%	0.0%	0.0%	0.0%	0.0%
tr|F1RP01|F1RP01	1	0	0	0	0	0	0	0	3	0	0	0	0	0	0	0	1.8%	0.0%	0.0%	0.0%	0.0%	0.0%	0.0%	0.0%
tr|F1RJM5|F1RJM5	1	1	0	0	0	0	0	0	1	2	0	0	0	0	0	0	0.5%	0.5%	0.0%	0.0%	0.0%	0.0%	0.0%	0.0%
tr|I3LLQ8|I3LLQ8	3	0	0	0	0	0	0	0	3	0	0	0	0	0	0	0	7.4%	0.0%	0.0%	0.0%	0.0%	0.0%	0.0%	0.0%
tr|I3LBH9|I3LBH9	2	0	0	0	0	0	0	0	3	0	0	0	0	0	0	0	0.8%	0.0%	0.0%	0.0%	0.0%	0.0%	0.0%	0.0%
Reverse_tr|F1SUV6|F1SUV6	0	1	0	0	0	0	0	0	0	3	0	0	0	0	0	0	0.0%	5.2%	0.0%	0.0%	0.0%	0.0%	0.0%	0.0%
tr|I3L8M9|I3L8M9	0	1	0	0	0	0	0	0	0	3	0	0	0	0	0	0	0.0%	1.0%	0.0%	0.0%	0.0%	0.0%	0.0%	0.0%
tr|F1RU83|F1RU83	1	2	0	0	0	0	0	0	1	2	0	0	0	0	0	0	1.1%	2.1%	0.0%	0.0%	0.0%	0.0%	0.0%	0.0%
tr|I3LP21|I3LP21	2	1	0	0	0	0	0	0	2	1	0	0	0	0	0	0	11.4%	4.5%	0.0%	0.0%	0.0%	0.0%	0.0%	0.0%
tr|F1RKS0|F1RKS0	2	0	0	0	0	0	0	0	3	0	0	0	0	0	0	0	9.1%	0.0%	0.0%	0.0%	0.0%	0.0%	0.0%	0.0%
tr|I3LFZ6|I3LFZ6	1	1	0	0	0	0	0	0	1	2	0	0	0	0	0	0	1.8%	2.4%	0.0%	0.0%	0.0%	0.0%	0.0%	0.0%
tr|F1SFQ3|F1SFQ3	2	0	0	0	0	0	0	0	3	0	0	0	0	0	0	0	8.0%	0.0%	0.0%	0.0%	0.0%	0.0%	0.0%	0.0%
tr|F1SPQ9|F1SPQ9	3	0	0	0	0	0	0	0	3	0	0	0	0	0	0	0	3.8%	0.0%	0.0%	0.0%	0.0%	0.0%	0.0%	0.0%
tr|K7GNX2|K7GNX2	2	0	0	0	0	0	0	0	3	0	0	0	0	0	0	0	4.7%	0.0%	0.0%	0.0%	0.0%	0.0%	0.0%	0.0%
tr|F1RX18|F1RX18	0	1	0	0	0	0	0	0	0	3	0	0	0	0	0	0	0.0%	0.7%	0.0%	0.0%	0.0%	0.0%	0.0%	0.0%
tr|F1RR60|F1RR60	1	1	0	0	0	0	0	0	2	1	0	0	0	0	0	0	5.8%	5.8%	0.0%	0.0%	0.0%	0.0%	0.0%	0.0%
tr|K7GS08|K7GS08	1	1	0	0	0	0	0	0	1	2	0	0	0	0	0	0	9.0%	3.4%	0.0%	0.0%	0.0%	0.0%	0.0%	0.0%
tr|F6Q525|F6Q525	2	0	0	0	0	0	0	0	3	0	0	0	0	0	0	0	23.7%	0.0%	0.0%	0.0%	0.0%	0.0%	0.0%	0.0%
tr|K7GP12|K7GP12	1	0	0	0	0	0	0	0	3	0	0	0	0	0	0	0	15.2%	0.0%	0.0%	0.0%	0.0%	0.0%	0.0%	0.0%
tr|K7GPS6|K7GPS6	2	0	0	0	0	0	0	0	3	0	0	0	0	0	0	0	14.5%	0.0%	0.0%	0.0%	0.0%	0.0%	0.0%	0.0%
tr|F1S098|F1S098	0	3	0	0	0	0	0	0	0	3	0	0	0	0	0	0	0.0%	0.8%	0.0%	0.0%	0.0%	0.0%	0.0%	0.0%
tr|F1SAQ4|F1SAQ4	3	0	0	0	0	0	0	0	3	0	0	0	0	0	0	0	4.4%	0.0%	0.0%	0.0%	0.0%	0.0%	0.0%	0.0%
tr|F1RYG9|F1RYG9	2	0	0	0	0	0	0	0	3	0	0	0	0	0	0	0	15.3%	0.0%	0.0%	0.0%	0.0%	0.0%	0.0%	0.0%
tr|F1RR17|F1RR17	1	0	0	0	0	0	0	0	3	0	0	0	0	0	0	0	13.0%	0.0%	0.0%	0.0%	0.0%	0.0%	0.0%	0.0%
tr|F1RHR9|F1RHR9	2	1	0	0	0	0	0	0	2	1	0	0	0	0	0	0	4.2%	1.6%	0.0%	0.0%	0.0%	0.0%	0.0%	0.0%
tr|F1RZV4|F1RZV4	1	1	0	0	0	0	0	0	2	1	0	0	0	0	0	0	2.8%	2.8%	0.0%	0.0%	0.0%	0.0%	0.0%	0.0%
A2TF48	1	1	0	0	0	0	0	0	1	2	0	0	0	0	0	0	4.1%	2.7%	0.0%	0.0%	0.0%	0.0%	0.0%	0.0%
tr|I3LMS8|I3LMS8	1	1	0	0	0	0	0	0	1	2	0	0	0	0	0	0	2.4%	2.4%	0.0%	0.0%	0.0%	0.0%	0.0%	0.0%
tr|F1STY3|F1STY3	0	1	0	0	0	0	0	0	0	3	0	0	0	0	0	0	0.0%	3.8%	0.0%	0.0%	0.0%	0.0%	0.0%	0.0%
tr|F1RHH2|F1RHH2	1	1	0	0	0	0	0	0	2	1	0	0	0	0	0	0	5.4%	2.5%	0.0%	0.0%	0.0%	0.0%	0.0%	0.0%
tr|I3LHS9|I3LHS9	1	1	0	0	0	0	0	0	1	2	0	0	0	0	0	0	2.5%	1.7%	0.0%	0.0%	0.0%	0.0%	0.0%	0.0%
tr|K7GQX1|K7GQX1	0	1	0	0	0	0	0	0	0	3	0	0	0	0	0	0	0.0%	3.0%	0.0%	0.0%	0.0%	0.0%	0.0%	0.0%
tr|F1SBT5|F1SBT5	3	0	0	0	0	0	0	0	3	0	0	0	0	0	0	0	6.4%	0.0%	0.0%	0.0%	0.0%	0.0%	0.0%	0.0%
tr|F1SHP1|F1SHP1	1	1	0	0	0	0	0	0	1	2	0	0	0	0	0	0	2.0%	1.2%	0.0%	0.0%	0.0%	0.0%	0.0%	0.0%
tr|F1SAQ6|F1SAQ6	2	1	0	0	0	0	0	0	2	1	0	0	0	0	0	0	9.9%	7.0%	0.0%	0.0%	0.0%	0.0%	0.0%	0.0%
Q9TUI8	2	1	0	0	0	0	0	0	2	1	0	0	0	0	0	0	10.2%	3.1%	0.0%	0.0%	0.0%	0.0%	0.0%	0.0%
tr|F1S1J5|F1S1J5	1	1	0	0	0	0	0	0	2	1	0	0	0	0	0	0	4.3%	4.3%	0.0%	0.0%	0.0%	0.0%	0.0%	0.0%
tr|I3L6V6|I3L6V6	2	0	0	0	0	0	0	0	3	0	0	0	0	0	0	0	2.6%	0.0%	0.0%	0.0%	0.0%	0.0%	0.0%	0.0%
tr|F1RX08|F1RX08	2	0	0	0	0	0	0	0	3	0	0	0	0	0	0	0	5.0%	0.0%	0.0%	0.0%	0.0%	0.0%	0.0%	0.0%
tr|I3LC49|I3LC49	1	1	0	0	0	0	0	0	1	2	0	0	0	0	0	0	5.8%	1.7%	0.0%	0.0%	0.0%	0.0%	0.0%	0.0%
tr|I3LEM8|I3LEM8	1	1	0	0	0	0	0	0	2	1	0	0	0	0	0	0	10.5%	15.8%	0.0%	0.0%	0.0%	0.0%	0.0%	0.0%
tr|I3LS90|I3LS90	1	1	0	0	0	0	0	0	1	2	0	0	0	0	0	0	0.7%	0.7%	0.0%	0.0%	0.0%	0.0%	0.0%	0.0%
O79874	1	0	0	0	0	0	0	0	3	0	0	0	0	0	0	0	6.0%	0.0%	0.0%	0.0%	0.0%	0.0%	0.0%	0.0%
tr|F1RRM1|F1RRM1	1	1	0	0	0	0	0	0	1	2	0	0	0	0	0	0	9.3%	4.9%	0.0%	0.0%	0.0%	0.0%	0.0%	0.0%
tr|F1SDE2|F1SDE2	1	1	0	0	0	0	0	0	1	2	0	0	0	0	0	0	3.9%	3.9%	0.0%	0.0%	0.0%	0.0%	0.0%	0.0%
tr|F1RPV8|F1RPV8	1	1	0	0	0	0	0	0	1	2	0	0	0	0	0	0	7.2%	6.1%	0.0%	0.0%	0.0%	0.0%	0.0%	0.0%
tr|F1SC89|F1SC89	3	0	0	0	0	0	0	0	3	0	0	0	0	0	0	0	3.2%	0.0%	0.0%	0.0%	0.0%	0.0%	0.0%	0.0%
tr|F1SPY1|F1SPY1	2	0	0	0	0	0	0	0	3	0	0	0	0	0	0	0	4.3%	0.0%	0.0%	0.0%	0.0%	0.0%	0.0%	0.0%
tr|I3L6B0|I3L6B0	2	0	0	0	0	0	0	0	3	0	0	0	0	0	0	0	2.8%	0.0%	0.0%	0.0%	0.0%	0.0%	0.0%	0.0%
tr|F1SPR0|F1SPR0	1	0	0	0	0	0	0	0	3	0	0	0	0	0	0	0	1.6%	0.0%	0.0%	0.0%	0.0%	0.0%	0.0%	0.0%
tr|F1SJ23|F1SJ23	1	1	0	0	0	0	0	0	2	1	0	0	0	0	0	0	3.6%	4.6%	0.0%	0.0%	0.0%	0.0%	0.0%	0.0%
tr|I3LIQ2|I3LIQ2	1	0	0	0	0	0	0	0	3	0	0	0	0	0	0	0	17.1%	0.0%	0.0%	0.0%	0.0%	0.0%	0.0%	0.0%
tr|I3LV21|I3LV21	1	1	0	0	0	0	0	0	2	1	0	0	0	0	0	0	8.3%	8.3%	0.0%	0.0%	0.0%	0.0%	0.0%	0.0%
tr|I3LUY9|I3LUY9	0	1	0	0	0	0	0	0	0	3	0	0	0	0	0	0	0.0%	1.2%	0.0%	0.0%	0.0%	0.0%	0.0%	0.0%
tr|I3LVH3|I3LVH3	1	1	0	0	0	0	0	0	1	2	0	0	0	0	0	0	6.8%	2.6%	0.0%	0.0%	0.0%	0.0%	0.0%	0.0%
tr|F1SN46|F1SN46	3	0	0	0	0	0	0	0	3	0	0	0	0	0	0	0	42.2%	0.0%	0.0%	0.0%	0.0%	0.0%	0.0%	0.0%
tr|F2Z4Z2|F2Z4Z2	1	1	0	0	0	0	0	0	1	2	0	0	0	0	0	0	14.7%	32.1%	0.0%	0.0%	0.0%	0.0%	0.0%	0.0%
tr|F1RL80|F1RL80	3	0	0	0	0	0	0	0	3	0	0	0	0	0	0	0	8.9%	0.0%	0.0%	0.0%	0.0%	0.0%	0.0%	0.0%
tr|F1RX56|F1RX56	2	0	0	0	0	0	0	0	3	0	0	0	0	0	0	0	6.0%	0.0%	0.0%	0.0%	0.0%	0.0%	0.0%	0.0%
tr|F1SJM7|F1SJM7	1	1	0	0	0	0	0	0	1	2	0	0	0	0	0	0	6.8%	2.0%	0.0%	0.0%	0.0%	0.0%	0.0%	0.0%
tr|F1S029|F1S029	3	0	0	0	0	0	0	0	3	0	0	0	0	0	0	0	8.1%	0.0%	0.0%	0.0%	0.0%	0.0%	0.0%	0.0%
tr|F1RYT5|F1RYT5	1	1	0	0	0	0	0	0	1	2	0	0	0	0	0	0	1.6%	2.7%	0.0%	0.0%	0.0%	0.0%	0.0%	0.0%
tr|K7GSF8|K7GSF8	1	1	0	0	0	0	0	0	1	2	0	0	0	0	0	0	2.4%	2.4%	0.0%	0.0%	0.0%	0.0%	0.0%	0.0%
tr|F1S2T2|F1S2T2	1	0	0	0	0	0	0	0	3	0	0	0	0	0	0	0	0.9%	0.0%	0.0%	0.0%	0.0%	0.0%	0.0%	0.0%
tr|I3L872|I3L872	2	0	0	0	0	0	0	0	3	0	0	0	0	0	0	0	15.9%	0.0%	0.0%	0.0%	0.0%	0.0%	0.0%	0.0%
tr|I3LJI6|I3LJI6	1	0	0	0	0	0	0	0	3	0	0	0	0	0	0	0	1.9%	0.0%	0.0%	0.0%	0.0%	0.0%	0.0%	0.0%
tr|I3LJR4|I3LJR4	2	0	0	0	0	0	0	0	3	0	0	0	0	0	0	0	4.7%	0.0%	0.0%	0.0%	0.0%	0.0%	0.0%	0.0%
tr|F1RQV0|F1RQV0	3	0	0	0	0	0	0	0	3	0	0	0	0	0	0	0	18.6%	0.0%	0.0%	0.0%	0.0%	0.0%	0.0%	0.0%
tr|F1S3W2|F1S3W2	2	1	0	0	0	0	0	0	2	1	0	0	0	0	0	0	10.2%	3.1%	0.0%	0.0%	0.0%	0.0%	0.0%	0.0%
tr|F1RRF0|F1RRF0	1	0	0	0	0	0	0	0	3	0	0	0	0	0	0	0	5.0%	0.0%	0.0%	0.0%	0.0%	0.0%	0.0%	0.0%
tr|F1SHI5|F1SHI5	3	0	0	0	0	0	0	0	3	0	0	0	0	0	0	0	2.8%	0.0%	0.0%	0.0%	0.0%	0.0%	0.0%	0.0%
tr|K7N7E6|K7N7E6	0	2	0	0	0	0	0	0	0	3	0	0	0	0	0	0	0.0%	11.7%	0.0%	0.0%	0.0%	0.0%	0.0%	0.0%
tr|F1SHC9|F1SHC9	3	0	0	0	0	0	0	0	3	0	0	0	0	0	0	0	7.4%	0.0%	0.0%	0.0%	0.0%	0.0%	0.0%	0.0%
tr|F1RZ70|F1RZ70	2	1	0	0	0	0	0	0	2	1	0	0	0	0	0	0	4.4%	1.4%	0.0%	0.0%	0.0%	0.0%	0.0%	0.0%
tr|F1SV35|F1SV35	1	1	0	0	0	0	0	0	1	2	0	0	0	0	0	0	5.6%	3.9%	0.0%	0.0%	0.0%	0.0%	0.0%	0.0%

	Peptide Count								Spectral Count								Sequence Coverage							
	Whole Cell	Whole Cell	Whole Cell	Cilia Fraction	Cilia Fraction	Cilia Fraction	Cilia Fraction	Cilia Fraction	Whole Cell	Whole Cell	Whole Cell	Cilia Fraction	Cilia Fraction	Cilia Fraction	Cilia Fraction	Cilia Fraction	Whole Cell	Whole Cell	Whole Cell	Cilia Fraction	Cilia Fraction	Cilia Fraction	Cilia Fraction	Cilia Fraction
Sample#	1	2	6	4	3	5	7	8	1	2	6	4	3	5	7	8	1	2	6	4	3	5	7	8
**Accession**																								
tr|I3LA23|I3LA23	1	1	0	0	0	0	0	0	2	1	0	0	0	0	0	0	5.5%	2.5%	0.0%	0.0%	0.0%	0.0%	0.0%	0.0%
tr|I3LE05|I3LE05	2	1	0	0	0	0	0	0	2	1	0	0	0	0	0	0	4.4%	1.5%	0.0%	0.0%	0.0%	0.0%	0.0%	0.0%
tr|F2Z506|F2Z506	1	1	0	0	0	0	0	0	1	2	0	0	0	0	0	0	2.4%	2.4%	0.0%	0.0%	0.0%	0.0%	0.0%	0.0%
tr|F1SKM5|F1SKM5	3	0	0	0	0	0	0	0	3	0	0	0	0	0	0	0	15.1%	0.0%	0.0%	0.0%	0.0%	0.0%	0.0%	0.0%
tr|I3LLW6|I3LLW6	1	0	0	0	0	0	0	0	3	0	0	0	0	0	0	0	5.4%	0.0%	0.0%	0.0%	0.0%	0.0%	0.0%	0.0%
tr|I3LGM5|I3LGM5	3	0	0	0	0	0	0	0	3	0	0	0	0	0	0	0	55.8%	0.0%	0.0%	0.0%	0.0%	0.0%	0.0%	0.0%
tr|I3L5F4|I3L5F4	2	0	0	0	0	0	0	0	3	0	0	0	0	0	0	0	10.9%	0.0%	0.0%	0.0%	0.0%	0.0%	0.0%	0.0%
tr|F1SP03|F1SP03	3	0	0	0	0	0	0	0	3	0	0	0	0	0	0	0	7.0%	0.0%	0.0%	0.0%	0.0%	0.0%	0.0%	0.0%
tr|F1S771|F1S771	1	2	0	0	0	0	0	0	1	2	0	0	0	0	0	0	2.8%	4.4%	0.0%	0.0%	0.0%	0.0%	0.0%	0.0%
tr|I3LCT6|I3LCT6	0	1	0	0	0	0	0	0	0	3	0	0	0	0	0	0	0.0%	3.7%	0.0%	0.0%	0.0%	0.0%	0.0%	0.0%
tr|I3LRX5|I3LRX5	1	0	0	0	0	0	0	0	3	0	0	0	0	0	0	0	5.9%	0.0%	0.0%	0.0%	0.0%	0.0%	0.0%	0.0%
tr|F1RV39|F1RV39	1	1	0	0	0	0	0	0	1	2	0	0	0	0	0	0	14.6%	3.5%	0.0%	0.0%	0.0%	0.0%	0.0%	0.0%
tr|F1RJQ5|F1RJQ5	2	0	0	0	0	0	0	0	3	0	0	0	0	0	0	0	9.2%	0.0%	0.0%	0.0%	0.0%	0.0%	0.0%	0.0%
tr|F1RHT9|F1RHT9	1	1	0	0	0	0	0	0	2	1	0	0	0	0	0	0	2.4%	1.3%	0.0%	0.0%	0.0%	0.0%	0.0%	0.0%
tr|F1RFG5|F1RFG5	2	1	0	0	0	0	0	0	2	1	0	0	0	0	0	0	3.9%	1.0%	0.0%	0.0%	0.0%	0.0%	0.0%	0.0%
tr|I3LJ83|I3LJ83	1	1	0	0	0	0	0	0	1	2	0	0	0	0	0	0	4.1%	4.1%	0.0%	0.0%	0.0%	0.0%	0.0%	0.0%
tr|F2Z5I3|F2Z5I3	1	1	0	0	0	0	0	0	1	2	0	0	0	0	0	0	21.5%	25.3%	0.0%	0.0%	0.0%	0.0%	0.0%	0.0%
tr|I3L928|I3L928	3	0	0	0	0	0	0	0	3	0	0	0	0	0	0	0	9.1%	0.0%	0.0%	0.0%	0.0%	0.0%	0.0%	0.0%
tr|I3LJ02|I3LJ02	1	0	0	0	0	0	0	0	3	0	0	0	0	0	0	0	1.9%	0.0%	0.0%	0.0%	0.0%	0.0%	0.0%	0.0%
tr|F1RUF7|F1RUF7	1	1	0	0	0	0	0	0	2	1	0	0	0	0	0	0	7.5%	7.5%	0.0%	0.0%	0.0%	0.0%	0.0%	0.0%
tr|I3LI55|I3LI55	0	2	0	0	0	0	0	0	0	3	0	0	0	0	0	0	0.0%	1.7%	0.0%	0.0%	0.0%	0.0%	0.0%	0.0%
tr|F1SM93|F1SM93	0	1	0	0	0	0	0	0	0	3	0	0	0	0	0	0	0.0%	16.4%	0.0%	0.0%	0.0%	0.0%	0.0%	0.0%
tr|F1RQ47|F1RQ47	1	2	0	0	0	0	0	0	1	2	0	0	0	0	0	0	2.6%	5.8%	0.0%	0.0%	0.0%	0.0%	0.0%	0.0%
tr|F1SNU2|F1SNU2	0	1	0	0	0	0	0	0	0	3	0	0	0	0	0	0	0.0%	3.4%	0.0%	0.0%	0.0%	0.0%	0.0%	0.0%
Q767M3	1	1	0	0	0	0	0	0	1	2	0	0	0	0	0	0	0.7%	1.5%	0.0%	0.0%	0.0%	0.0%	0.0%	0.0%
tr|I3LCX2|I3LCX2	1	1	0	0	0	0	0	0	2	1	0	0	0	0	0	0	3.9%	1.6%	0.0%	0.0%	0.0%	0.0%	0.0%	0.0%
tr|I3LDY4|I3LDY4	2	0	0	0	0	0	0	0	3	0	0	0	0	0	0	0	23.2%	0.0%	0.0%	0.0%	0.0%	0.0%	0.0%	0.0%
tr|F2Z5C8|F2Z5C8	0	1	0	0	0	0	0	0	0	3	0	0	0	0	0	0	0.0%	7.2%	0.0%	0.0%	0.0%	0.0%	0.0%	0.0%
Q767K9	1	1	0	0	0	0	0	0	1	2	0	0	0	0	0	0	3.6%	1.2%	0.0%	0.0%	0.0%	0.0%	0.0%	0.0%
Q767K6	1	2	0	0	0	0	0	0	1	2	0	0	0	0	0	0	1.1%	2.1%	0.0%	0.0%	0.0%	0.0%	0.0%	0.0%
tr|F1SPI3|F1SPI3	2	0	0	0	0	0	0	0	3	0	0	0	0	0	0	0	2.7%	0.0%	0.0%	0.0%	0.0%	0.0%	0.0%	0.0%
tr|F1SAC0|F1SAC0	1	0	0	0	0	0	0	0	3	0	0	0	0	0	0	0	10.2%	0.0%	0.0%	0.0%	0.0%	0.0%	0.0%	0.0%
tr|F1S0G2|F1S0G2	1	1	0	0	0	0	0	0	2	1	0	0	0	0	0	0	3.8%	2.5%	0.0%	0.0%	0.0%	0.0%	0.0%	0.0%
tr|I3LF61|I3LF61	1	1	0	0	0	0	0	0	1	2	0	0	0	0	0	0	4.2%	2.9%	0.0%	0.0%	0.0%	0.0%	0.0%	0.0%
tr|I3LMH3|I3LMH3	0	2	0	0	0	0	0	0	0	3	0	0	0	0	0	0	0.0%	2.6%	0.0%	0.0%	0.0%	0.0%	0.0%	0.0%
tr|I3LNP0|I3LNP0	1	0	0	0	0	0	0	0	3	0	0	0	0	0	0	0	3.8%	0.0%	0.0%	0.0%	0.0%	0.0%	0.0%	0.0%
tr|F1SQI2|F1SQI2	1	1	0	0	0	0	0	0	1	2	0	0	0	0	0	0	7.4%	7.4%	0.0%	0.0%	0.0%	0.0%	0.0%	0.0%
tr|F1SQA3|F1SQA3	0	2	0	0	0	0	0	0	0	3	0	0	0	0	0	0	0.0%	10.1%	0.0%	0.0%	0.0%	0.0%	0.0%	0.0%
tr|F1RSW6|F1RSW6	1	0	0	0	0	0	0	0	3	0	0	0	0	0	0	0	16.2%	0.0%	0.0%	0.0%	0.0%	0.0%	0.0%	0.0%
tr|F1RFJ5|F1RFJ5	1	0	0	0	0	0	0	0	3	0	0	0	0	0	0	0	7.4%	0.0%	0.0%	0.0%	0.0%	0.0%	0.0%	0.0%
tr|I3LIM8|I3LIM8	2	1	0	0	0	0	0	0	2	1	0	0	0	0	0	0	3.0%	3.5%	0.0%	0.0%	0.0%	0.0%	0.0%	0.0%
tr|I3LJQ2|I3LJQ2	2	0	0	0	0	0	0	0	3	0	0	0	0	0	0	0	4.4%	0.0%	0.0%	0.0%	0.0%	0.0%	0.0%	0.0%
tr|I3LRC4|I3LRC4	3	0	0	0	0	0	0	0	3	0	0	0	0	0	0	0	26.9%	0.0%	0.0%	0.0%	0.0%	0.0%	0.0%	0.0%
tr|F1SC66|F1SC66	2	0	0	0	0	0	0	0	3	0	0	0	0	0	0	0	4.3%	0.0%	0.0%	0.0%	0.0%	0.0%	0.0%	0.0%
tr|I3LSY9|I3LSY9	1	1	0	0	0	0	0	0	1	2	0	0	0	0	0	0	5.9%	4.9%	0.0%	0.0%	0.0%	0.0%	0.0%	0.0%
tr|F1RTW8|F1RTW8	0	2	0	0	0	0	0	0	0	3	0	0	0	0	0	0	0.0%	4.6%	0.0%	0.0%	0.0%	0.0%	0.0%	0.0%
tr|F1RYU4|F1RYU4	2	0	0	0	0	0	0	0	3	0	0	0	0	0	0	0	5.6%	0.0%	0.0%	0.0%	0.0%	0.0%	0.0%	0.0%
tr|F1RY61|F1RY61	1	1	0	0	0	0	0	0	1	2	0	0	0	0	0	0	3.1%	2.9%	0.0%	0.0%	0.0%	0.0%	0.0%	0.0%
tr|F1RG43|F1RG43	3	0	0	0	0	0	0	0	3	0	0	0	0	0	0	0	3.4%	0.0%	0.0%	0.0%	0.0%	0.0%	0.0%	0.0%
tr|F1S6A9|F1S6A9	3	0	0	0	0	0	0	0	3	0	0	0	0	0	0	0	20.5%	0.0%	0.0%	0.0%	0.0%	0.0%	0.0%	0.0%
tr|F1SSD4|F1SSD4	1	1	0	0	0	0	0	0	1	2	0	0	0	0	0	0	6.3%	6.3%	0.0%	0.0%	0.0%	0.0%	0.0%	0.0%
tr|F1SIL5|F1SIL5	1	2	0	0	0	0	0	0	1	2	0	0	0	0	0	0	0.6%	0.9%	0.0%	0.0%	0.0%	0.0%	0.0%	0.0%
tr|I3LL57|I3LL57	1	1	0	0	0	0	0	0	1	2	0	0	0	0	0	0	3.6%	2.2%	0.0%	0.0%	0.0%	0.0%	0.0%	0.0%
tr|F1RHF2|F1RHF2	2	0	0	0	0	0	0	0	3	0	0	0	0	0	0	0	4.8%	0.0%	0.0%	0.0%	0.0%	0.0%	0.0%	0.0%
tr|F1RUE9|F1RUE9	1	1	0	0	0	0	0	0	1	2	0	0	0	0	0	0	3.5%	1.4%	0.0%	0.0%	0.0%	0.0%	0.0%	0.0%
tr|I3LFH4|I3LFH4	0	1	0	0	0	0	0	0	0	3	0	0	0	0	0	0	0.0%	3.3%	0.0%	0.0%	0.0%	0.0%	0.0%	0.0%
tr|I3LU36|I3LU36	0	1	0	0	0	0	0	0	0	3	0	0	0	0	0	0	0.0%	0.9%	0.0%	0.0%	0.0%	0.0%	0.0%	0.0%
tr|I3LEG3|I3LEG3	2	0	0	0	0	0	0	0	3	0	0	0	0	0	0	0	6.8%	0.0%	0.0%	0.0%	0.0%	0.0%	0.0%	0.0%
tr|K7GQX8|K7GQX8	0	1	0	0	0	0	0	0	0	3	0	0	0	0	0	0	0.0%	37.5%	0.0%	0.0%	0.0%	0.0%	0.0%	0.0%
tr|F1SPQ3|F1SPQ3	0	2	0	0	0	0	0	0	0	3	0	0	0	0	0	0	0.0%	36.2%	0.0%	0.0%	0.0%	0.0%	0.0%	0.0%
tr|F1SP54|F1SP54	2	0	0	0	0	0	0	0	3	0	0	0	0	0	0	0	5.2%	0.0%	0.0%	0.0%	0.0%	0.0%	0.0%	0.0%
tr|F1SJG9|F1SJG9	0	2	0	0	0	0	0	0	0	3	0	0	0	0	0	0	0.0%	1.5%	0.0%	0.0%	0.0%	0.0%	0.0%	0.0%
tr|K7GM95|K7GM95	2	0	0	0	0	0	0	0	3	0	0	0	0	0	0	0	4.5%	0.0%	0.0%	0.0%	0.0%	0.0%	0.0%	0.0%
tr|F1SC76|F1SC76	0	2	0	0	0	0	0	0	0	3	0	0	0	0	0	0	0.0%	11.0%	0.0%	0.0%	0.0%	0.0%	0.0%	0.0%
Q06AA4	1	0	0	0	0	0	0	0	3	0	0	0	0	0	0	0	4.6%	0.0%	0.0%	0.0%	0.0%	0.0%	0.0%	0.0%
tr|E1B2C7|E1B2C7	1	1	0	0	0	0	0	0	1	2	0	0	0	0	0	0	2.6%	2.6%	0.0%	0.0%	0.0%	0.0%	0.0%	0.0%
tr|F1SSD0|F1SSD0	2	1	0	0	0	0	0	0	2	1	0	0	0	0	0	0	2.4%	1.7%	0.0%	0.0%	0.0%	0.0%	0.0%	0.0%
tr|F1RR46|F1RR46	1	1	0	0	0	0	0	0	1	2	0	0	0	0	0	0	5.5%	7.1%	0.0%	0.0%	0.0%	0.0%	0.0%	0.0%
tr|F1SJQ4|F1SJQ4	2	0	0	0	0	0	0	0	3	0	0	0	0	0	0	0	3.8%	0.0%	0.0%	0.0%	0.0%	0.0%	0.0%	0.0%
tr|F1RQS0|F1RQS0	0	2	0	0	0	0	0	0	0	3	0	0	0	0	0	0	0.0%	7.3%	0.0%	0.0%	0.0%	0.0%	0.0%	0.0%
tr|K7GSW0|K7GSW0	1	0	0	0	0	0	0	0	3	0	0	0	0	0	0	0	15.0%	0.0%	0.0%	0.0%	0.0%	0.0%	0.0%	0.0%
tr|I3LNA6|I3LNA6	0	1	0	0	0	0	0	0	0	3	0	0	0	0	0	0	0.0%	14.4%	0.0%	0.0%	0.0%	0.0%	0.0%	0.0%
tr|F1RJ94|F1RJ94	0	1	0	0	0	0	0	0	0	3	0	0	0	0	0	0	0.0%	1.5%	0.0%	0.0%	0.0%	0.0%	0.0%	0.0%
tr|F1SGX9|F1SGX9	1	1	0	0	0	0	0	0	1	2	0	0	0	0	0	0	0.8%	0.8%	0.0%	0.0%	0.0%	0.0%	0.0%	0.0%
tr|F1RU99|F1RU99	1	1	0	0	0	0	0	0	2	1	0	0	0	0	0	0	26.3%	13.2%	0.0%	0.0%	0.0%	0.0%	0.0%	0.0%
tr|F1RZV6|F1RZV6	1	1	0	0	0	0	0	0	1	2	0	0	0	0	0	0	2.2%	3.6%	0.0%	0.0%	0.0%	0.0%	0.0%	0.0%
Q5W9D5	2	0	0	0	0	0	0	0	3	0	0	0	0	0	0	0	9.7%	0.0%	0.0%	0.0%	0.0%	0.0%	0.0%	0.0%
tr|F1S9N9|F1S9N9	2	0	0	0	0	0	0	0	3	0	0	0	0	0	0	0	8.5%	0.0%	0.0%	0.0%	0.0%	0.0%	0.0%	0.0%
tr|F1S580|F1S580	1	1	0	0	0	0	0	0	1	2	0	0	0	0	0	0	3.5%	3.1%	0.0%	0.0%	0.0%	0.0%	0.0%	0.0%
tr|F1SI90|F1SI90	2	0	0	0	0	0	0	0	3	0	0	0	0	0	0	0	13.8%	0.0%	0.0%	0.0%	0.0%	0.0%	0.0%	0.0%
tr|K7GLH0|K7GLH0	1	0	0	0	0	0	0	0	3	0	0	0	0	0	0	0	9.6%	0.0%	0.0%	0.0%	0.0%	0.0%	0.0%	0.0%

	Peptide Count								Spectral Count								Sequence Coverage							
	Whole Cell	Whole Cell	Whole Cell	Cilia Fraction	Cilia Fraction	Cilia Fraction	Cilia Fraction	Cilia Fraction	Whole Cell	Whole Cell	Whole Cell	Cilia Fraction	Cilia Fraction	Cilia Fraction	Cilia Fraction	Cilia Fraction	Whole Cell	Whole Cell	Whole Cell	Cilia Fraction	Cilia Fraction	Cilia Fraction	Cilia Fraction	Cilia Fraction
Sample#	1	2	6	4	3	5	7	8	1	2	6	4	3	5	7	8	1	2	6	4	3	5	7	8
**Accession**																								
tr|Q0GA84|Q0GA84	1	1	0	0	0	0	0	0	1	2	0	0	0	0	0	0	4.6%	4.3%	0.0%	0.0%	0.0%	0.0%	0.0%	0.0%
tr|I3L7U1|I3L7U1	1	0	0	0	0	0	0	0	3	0	0	0	0	0	0	0	5.4%	0.0%	0.0%	0.0%	0.0%	0.0%	0.0%	0.0%
tr|F1SBF6|F1SBF6	3	0	0	0	0	0	0	0	3	0	0	0	0	0	0	0	10.8%	0.0%	0.0%	0.0%	0.0%	0.0%	0.0%	0.0%
tr|F1RXV1|F1RXV1	0	1	0	0	0	0	0	0	0	3	0	0	0	0	0	0	0.0%	3.2%	0.0%	0.0%	0.0%	0.0%	0.0%	0.0%
Reverse_tr|F2Z5K6|F2Z5K6	1	0	0	0	0	0	0	0	3	0	0	0	0	0	0	0	16.2%	0.0%	0.0%	0.0%	0.0%	0.0%	0.0%	0.0%
tr|I3LQ80|I3LQ80	0	1	0	0	0	0	0	0	0	3	0	0	0	0	0	0	0.0%	4.8%	0.0%	0.0%	0.0%	0.0%	0.0%	0.0%
tr|Q8WNW8|Q8WNW8	1	1	0	0	0	0	0	0	1	2	0	0	0	0	0	0	4.5%	2.5%	0.0%	0.0%	0.0%	0.0%	0.0%	0.0%
tr|K7GMR8|K7GMR8	1	1	0	0	0	0	0	0	1	2	0	0	0	0	0	0	3.8%	2.1%	0.0%	0.0%	0.0%	0.0%	0.0%	0.0%
tr|F1SBU5|F1SBU5	2	0	0	0	0	0	0	0	3	0	0	0	0	0	0	0	4.8%	0.0%	0.0%	0.0%	0.0%	0.0%	0.0%	0.0%
tr|F1RZ13|F1RZ13	3	0	0	0	0	0	0	0	3	0	0	0	0	0	0	0	7.5%	0.0%	0.0%	0.0%	0.0%	0.0%	0.0%	0.0%
tr|I3L5S1|I3L5S1	2	0	0	0	0	0	0	0	3	0	0	0	0	0	0	0	7.2%	0.0%	0.0%	0.0%	0.0%	0.0%	0.0%	0.0%
tr|K7GPA5|K7GPA5	1	0	0	0	0	0	0	0	3	0	0	0	0	0	0	0	1.6%	0.0%	0.0%	0.0%	0.0%	0.0%	0.0%	0.0%
tr|K7GQW8|K7GQW8	2	0	0	0	0	0	0	0	3	0	0	0	0	0	0	0	10.2%	0.0%	0.0%	0.0%	0.0%	0.0%	0.0%	0.0%
tr|F1SK46|F1SK46	0	2	0	0	0	0	0	0	0	3	0	0	0	0	0	0	0.0%	8.6%	0.0%	0.0%	0.0%	0.0%	0.0%	0.0%
tr|F1SF34|F1SF34	1	0	0	0	0	0	0	0	3	0	0	0	0	0	0	0	5.2%	0.0%	0.0%	0.0%	0.0%	0.0%	0.0%	0.0%
tr|I3L9L4|I3L9L4	1	0	0	0	0	0	0	0	3	0	0	0	0	0	0	0	7.7%	0.0%	0.0%	0.0%	0.0%	0.0%	0.0%	0.0%
tr|F1SB99|F1SB99	1	0	0	0	0	0	0	0	3	0	0	0	0	0	0	0	2.6%	0.0%	0.0%	0.0%	0.0%	0.0%	0.0%	0.0%
A5GFY4	2	0	0	0	0	0	0	0	3	0	0	0	0	0	0	0	6.8%	0.0%	0.0%	0.0%	0.0%	0.0%	0.0%	0.0%
tr|F1RXX0|F1RXX0	1	0	0	0	0	0	0	0	3	0	0	0	0	0	0	0	14.4%	0.0%	0.0%	0.0%	0.0%	0.0%	0.0%	0.0%
tr|I3LBQ4|I3LBQ4	3	0	0	0	0	0	0	0	3	0	0	0	0	0	0	0	3.8%	0.0%	0.0%	0.0%	0.0%	0.0%	0.0%	0.0%
tr|I3LRD0|I3LRD0	1	0	0	0	0	0	0	0	3	0	0	0	0	0	0	0	15.1%	0.0%	0.0%	0.0%	0.0%	0.0%	0.0%	0.0%
tr|I3L908|I3L908	1	1	0	0	0	0	0	0	1	2	0	0	0	0	0	0	1.2%	1.0%	0.0%	0.0%	0.0%	0.0%	0.0%	0.0%
tr|I3LCJ3|I3LCJ3	1	1	0	0	0	0	0	0	1	2	0	0	0	0	0	0	4.8%	3.9%	0.0%	0.0%	0.0%	0.0%	0.0%	0.0%
tr|I3LT95|I3LT95	2	1	0	0	0	0	0	0	2	1	0	0	0	0	0	0	11.8%	4.8%	0.0%	0.0%	0.0%	0.0%	0.0%	0.0%
tr|F1SLQ7|F1SLQ7	2	0	0	0	0	0	0	0	3	0	0	0	0	0	0	0	12.6%	0.0%	0.0%	0.0%	0.0%	0.0%	0.0%	0.0%
tr|I3L5P9|I3L5P9	1	1	0	0	0	0	0	0	2	1	0	0	0	0	0	0	13.5%	13.5%	0.0%	0.0%	0.0%	0.0%	0.0%	0.0%
tr|F1S6V3|F1S6V3	1	1	0	0	0	0	0	0	2	1	0	0	0	0	0	0	0.8%	1.3%	0.0%	0.0%	0.0%	0.0%	0.0%	0.0%
A3KCL7	1	1	0	0	0	0	0	0	1	2	0	0	0	0	0	0	11.4%	5.6%	0.0%	0.0%	0.0%	0.0%	0.0%	0.0%
tr|F1RK59|F1RK59	3	0	0	0	0	0	0	0	3	0	0	0	0	0	0	0	21.4%	0.0%	0.0%	0.0%	0.0%	0.0%	0.0%	0.0%
tr|F1RU69|F1RU69	1	1	0	0	0	0	0	0	1	2	0	0	0	0	0	0	0.7%	1.5%	0.0%	0.0%	0.0%	0.0%	0.0%	0.0%
tr|F1SPG1|F1SPG1	2	1	0	0	0	0	0	0	2	1	0	0	0	0	0	0	10.7%	5.6%	0.0%	0.0%	0.0%	0.0%	0.0%	0.0%
tr|F1S9Z9|F1S9Z9	1	1	0	0	0	0	0	0	1	2	0	0	0	0	0	0	2.4%	1.7%	0.0%	0.0%	0.0%	0.0%	0.0%	0.0%
tr|I3LDE9|I3LDE9	1	1	0	0	0	0	0	0	1	2	0	0	0	0	0	0	5.5%	3.7%	0.0%	0.0%	0.0%	0.0%	0.0%	0.0%
tr|K7GRL4|K7GRL4	0	1	0	0	0	0	0	0	0	3	0	0	0	0	0	0	0.0%	0.7%	0.0%	0.0%	0.0%	0.0%	0.0%	0.0%
tr|I3LHN2|I3LHN2	1	1	0	0	0	0	0	0	1	2	0	0	0	0	0	0	0.7%	0.7%	0.0%	0.0%	0.0%	0.0%	0.0%	0.0%
P80171	2	0	0	0	0	0	0	0	3	0	0	0	0	0	0	0	33.3%	0.0%	0.0%	0.0%	0.0%	0.0%	0.0%	0.0%
tr|F1SNI1|F1SNI1	1	2	0	0	0	0	0	0	1	2	0	0	0	0	0	0	2.0%	4.9%	0.0%	0.0%	0.0%	0.0%	0.0%	0.0%
tr|F6Q023|F6Q023	1	1	0	0	0	0	0	0	1	2	0	0	0	0	0	0	3.4%	3.4%	0.0%	0.0%	0.0%	0.0%	0.0%	0.0%
tr|I3LIU1|I3LIU1	2	0	0	0	0	0	0	0	3	0	0	0	0	0	0	0	21.7%	0.0%	0.0%	0.0%	0.0%	0.0%	0.0%	0.0%
tr|I3L6R1|I3L6R1	2	0	0	0	0	0	0	0	3	0	0	0	0	0	0	0	34.9%	0.0%	0.0%	0.0%	0.0%	0.0%	0.0%	0.0%
tr|F1RJJ3|F1RJJ3	2	0	0	0	0	0	0	0	3	0	0	0	0	0	0	0	7.6%	0.0%	0.0%	0.0%	0.0%	0.0%	0.0%	0.0%
tr|I3LCC6|I3LCC6	1	0	0	0	0	0	0	0	3	0	0	0	0	0	0	0	22.4%	0.0%	0.0%	0.0%	0.0%	0.0%	0.0%	0.0%
tr|K7GMZ0|K7GMZ0	1	1	0	0	0	0	0	0	1	2	0	0	0	0	0	0	9.2%	3.5%	0.0%	0.0%	0.0%	0.0%	0.0%	0.0%
tr|I3LDC9|I3LDC9	0	1	0	0	0	0	0	0	0	3	0	0	0	0	0	0	0.0%	5.2%	0.0%	0.0%	0.0%	0.0%	0.0%	0.0%
tr|F1SND5|F1SND5	0	1	0	0	0	0	0	0	0	3	0	0	0	0	0	0	0.0%	3.6%	0.0%	0.0%	0.0%	0.0%	0.0%	0.0%
tr|F1SKG2|F1SKG2	0	2	0	0	0	0	0	0	0	3	0	0	0	0	0	0	0.0%	0.6%	0.0%	0.0%	0.0%	0.0%	0.0%	0.0%
tr|F1SB92|F1SB92	0	1	0	0	0	0	0	0	0	3	0	0	0	0	0	0	0.0%	8.8%	0.0%	0.0%	0.0%	0.0%	0.0%	0.0%
tr|F1SKY0|F1SKY0	0	1	0	0	0	0	0	0	0	3	0	0	0	0	0	0	0.0%	3.7%	0.0%	0.0%	0.0%	0.0%	0.0%	0.0%
O19175	0	1	0	0	0	0	0	0	0	3	0	0	0	0	0	0	0.0%	8.0%	0.0%	0.0%	0.0%	0.0%	0.0%	0.0%
tr|K7GQV1|K7GQV1	1	0	0	0	0	0	0	0	3	0	0	0	0	0	0	0	10.7%	0.0%	0.0%	0.0%	0.0%	0.0%	0.0%	0.0%
tr|F1S5K0|F1S5K0	2	0	0	0	0	0	0	0	3	0	0	0	0	0	0	0	7.7%	0.0%	0.0%	0.0%	0.0%	0.0%	0.0%	0.0%
tr|I3LJ64|I3LJ64	2	0	0	0	0	0	0	0	3	0	0	0	0	0	0	0	47.8%	0.0%	0.0%	0.0%	0.0%	0.0%	0.0%	0.0%
tr|I3LFL3|I3LFL3	1	1	0	0	0	0	0	0	1	2	0	0	0	0	0	0	8.6%	5.9%	0.0%	0.0%	0.0%	0.0%	0.0%	0.0%
tr|F1RK50|F1RK50	2	0	0	0	0	0	0	0	3	0	0	0	0	0	0	0	29.8%	0.0%	0.0%	0.0%	0.0%	0.0%	0.0%	0.0%
tr|I3L8N0|I3L8N0	0	1	0	0	0	0	0	0	0	3	0	0	0	0	0	0	0.0%	3.1%	0.0%	0.0%	0.0%	0.0%	0.0%	0.0%
tr|I3LK72|I3LK72	2	0	0	0	0	0	0	0	3	0	0	0	0	0	0	0	7.9%	0.0%	0.0%	0.0%	0.0%	0.0%	0.0%	0.0%
tr|F1RVA6|F1RVA6	0	2	0	0	0	0	0	0	0	3	0	0	0	0	0	0	0.0%	3.5%	0.0%	0.0%	0.0%	0.0%	0.0%	0.0%
tr|I3LB43|I3LB43	1	0	0	0	0	0	0	0	3	0	0	0	0	0	0	0	21.7%	0.0%	0.0%	0.0%	0.0%	0.0%	0.0%	0.0%
tr|F1SJK5|F1SJK5	1	1	0	0	0	0	0	0	1	2	0	0	0	0	0	0	3.9%	2.3%	0.0%	0.0%	0.0%	0.0%	0.0%	0.0%
tr|F1SK34|F1SK34	1	0	0	0	0	0	0	0	3	0	0	0	0	0	0	0	9.3%	0.0%	0.0%	0.0%	0.0%	0.0%	0.0%	0.0%
tr|F1RWE3|F1RWE3	2	1	0	0	0	0	0	0	2	1	0	0	0	0	0	0	9.1%	2.1%	0.0%	0.0%	0.0%	0.0%	0.0%	0.0%
tr|F1S0L4|F1S0L4	2	1	1	0	0	0	0	0	2	1	1	0	0	0	0	0	8.1%	2.5%	5.2%	0.0%	0.0%	0.0%	0.0%	0.0%
tr|F1SDI3|F1SDI3	2	0	1	0	0	0	0	0	3	0	1	0	0	0	0	0	10.9%	0.0%	4.7%	0.0%	0.0%	0.0%	0.0%	0.0%
tr|F1SU57|F1SU57	2	1	1	0	0	0	0	0	2	1	1	0	0	0	0	0	6.7%	4.8%	2.3%	0.0%	0.0%	0.0%	0.0%	0.0%
tr|F1S187|F1S187	1	2	1	0	0	0	0	0	1	2	1	0	0	0	0	0	4.2%	4.7%	4.4%	0.0%	0.0%	0.0%	0.0%	0.0%
tr|F1S540|F1S540	3	0	1	0	0	0	0	0	3	0	1	0	0	0	0	0	4.2%	0.0%	1.4%	0.0%	0.0%	0.0%	0.0%	0.0%
tr|I3LT90|I3LT90	2	1	1	0	0	0	0	0	2	1	1	0	0	0	0	0	3.6%	1.1%	1.5%	0.0%	0.0%	0.0%	0.0%	0.0%
tr|F1S3N6|F1S3N6	3	0	1	0	0	0	0	0	3	0	1	0	0	0	0	0	8.9%	0.0%	4.2%	0.0%	0.0%	0.0%	0.0%	0.0%
tr|F1SJE9|F1SJE9	2	1	1	0	0	0	0	0	2	1	1	0	0	0	0	0	1.8%	0.9%	0.9%	0.0%	0.0%	0.0%	0.0%	0.0%
tr|K7GPL8|K7GPL8	2	1	1	0	0	0	0	0	2	1	1	0	0	0	0	0	7.0%	5.0%	2.4%	0.0%	0.0%	0.0%	0.0%	0.0%
tr|I3LR88|I3LR88	0	2	1	0	0	0	0	0	0	3	1	0	0	0	0	0	0.0%	12.7%	4.7%	0.0%	0.0%	0.0%	0.0%	0.0%
tr|F1RX83|F1RX83	2	0	1	0	0	0	0	0	3	0	1	0	0	0	0	0	10.3%	0.0%	5.9%	0.0%	0.0%	0.0%	0.0%	0.0%
tr|I3LTD7|I3LTD7	3	0	1	0	0	0	0	0	3	0	1	0	0	0	0	0	5.7%	0.0%	3.5%	0.0%	0.0%	0.0%	0.0%	0.0%
tr|I3LJ55|I3LJ55	3	0	1	0	0	0	0	0	3	0	1	0	0	0	0	0	5.5%	0.0%	3.4%	0.0%	0.0%	0.0%	0.0%	0.0%
tr|K7GSF1|K7GSF1	0	2	1	0	0	0	0	0	0	3	1	0	0	0	0	0	0.0%	23.0%	9.1%	0.0%	0.0%	0.0%	0.0%	0.0%
tr|K7GMY6|K7GMY6	2	1	1	0	0	0	0	0	2	1	1	0	0	0	0	0	6.6%	4.7%	2.2%	0.0%	0.0%	0.0%	0.0%	0.0%
tr|F1S9B6|F1S9B6	2	0	1	0	0	0	0	0	3	0	1	0	0	0	0	0	4.2%	0.0%	2.2%	0.0%	0.0%	0.0%	0.0%	0.0%
tr|F1RVH4|F1RVH4	2	0	1	0	0	0	0	0	3	0	1	0	0	0	0	0	19.0%	0.0%	11.3%	0.0%	0.0%	0.0%	0.0%	0.0%
tr|F1S8I6|F1S8I6	2	1	1	0	0	0	0	0	2	1	1	0	0	0	0	0	16.2%	10.0%	6.2%	0.0%	0.0%	0.0%	0.0%	0.0%
tr|I3L8N4|I3L8N4	2	0	1	0	0	0	0	0	3	0	1	0	0	0	0	0	9.0%	0.0%	3.8%	0.0%	0.0%	0.0%	0.0%	0.0%
tr|F1RNH0|F1RNH0	2	1	1	0	0	0	0	0	2	1	1	0	0	0	0	0	8.2%	2.7%	3.6%	0.0%	0.0%	0.0%	0.0%	0.0%

	Peptide Count								Spectral Count								Sequence Coverage							
	Whole Cell	Whole Cell	Whole Cell	Cilia Fraction	Cilia Fraction	Cilia Fraction	Cilia Fraction	Cilia Fraction	Whole Cell	Whole Cell	Whole Cell	Cilia Fraction	Cilia Fraction	Cilia Fraction	Cilia Fraction	Cilia Fraction	Whole Cell	Whole Cell	Whole Cell	Cilia Fraction	Cilia Fraction	Cilia Fraction	Cilia Fraction	Cilia Fraction
Sample#	1	2	6	4	3	5	7	8	1	2	6	4	3	5	7	8	1	2	6	4	3	5	7	8
**Accession**																								
tr|K7GNY8|K7GNY8	2	1	1	0	0	0	0	0	2	1	1	0	0	0	0	0	6.8%	4.9%	2.3%	0.0%	0.0%	0.0%	0.0%	0.0%
tr|F1RFT3|F1RFT3	1	0	1	0	0	0	0	0	3	0	1	0	0	0	0	0	2.1%	0.0%	7.9%	0.0%	0.0%	0.0%	0.0%	0.0%
tr|I3LQL5|I3LQL5	1	0	1	0	0	0	0	0	3	0	1	0	0	0	0	0	6.2%	0.0%	5.5%	0.0%	0.0%	0.0%	0.0%	0.0%
tr|I3LEU9|I3LEU9	1	1	1	0	0	0	0	0	1	2	1	0	0	0	0	0	3.9%	1.5%	4.1%	0.0%	0.0%	0.0%	0.0%	0.0%
tr|F1SH89|F1SH89	2	1	1	0	0	0	0	0	2	1	1	0	0	0	0	0	5.4%	1.7%	3.2%	0.0%	0.0%	0.0%	0.0%	0.0%
tr|I3LSK0|I3LSK0	3	0	1	0	0	0	0	0	3	0	1	0	0	0	0	0	2.6%	0.0%	0.6%	0.0%	0.0%	0.0%	0.0%	0.0%
tr|F1RFW9|F1RFW9	3	0	1	0	0	0	0	0	3	0	1	0	0	0	0	0	7.3%	0.0%	2.5%	0.0%	0.0%	0.0%	0.0%	0.0%
tr|F1SGP8|F1SGP8	3	0	1	0	0	0	0	0	3	0	1	0	0	0	0	0	16.3%	0.0%	4.2%	0.0%	0.0%	0.0%	0.0%	0.0%
tr|K7GS05|K7GS05	2	1	1	0	0	0	0	0	2	1	1	0	0	0	0	0	6.7%	4.8%	2.3%	0.0%	0.0%	0.0%	0.0%	0.0%
tr|F1S0A8|F1S0A8	2	0	1	0	0	0	0	0	3	0	2	0	0	0	0	0	6.0%	0.0%	5.6%	0.0%	0.0%	0.0%	0.0%	0.0%
tr|I3LHV8|I3LHV8	3	0	1	0	0	0	0	0	3	0	2	0	0	0	0	0	11.9%	0.0%	3.0%	0.0%	0.0%	0.0%	0.0%	0.0%
tr|F1SV23|F1SV23	2	0	1	0	0	0	0	0	3	0	2	0	0	0	0	0	29.2%	0.0%	12.3%	0.0%	0.0%	0.0%	0.0%	0.0%
tr|F1SCS0|F1SCS0	2	0	1	0	0	0	0	0	3	0	2	0	0	0	0	0	7.0%	0.0%	4.4%	0.0%	0.0%	0.0%	0.0%	0.0%
tr|I3L5E4|I3L5E4	1	1	1	0	0	0	0	0	2	1	2	0	0	0	0	0	3.8%	3.8%	4.7%	0.0%	0.0%	0.0%	0.0%	0.0%
tr|F1S8W1|F1S8W1	2	0	1	0	0	0	0	0	3	0	2	0	0	0	0	0	8.0%	0.0%	3.4%	0.0%	0.0%	0.0%	0.0%	0.0%
tr|I3LGM4|I3LGM4	1	2	2	0	0	0	0	0	1	2	2	0	0	0	0	0	24.1%	30.5%	19.9%	0.0%	0.0%	0.0%	0.0%	0.0%
tr|I3LGT7|I3LGT7	2	0	1	0	0	0	0	0	3	0	2	0	0	0	0	0	14.0%	0.0%	7.0%	0.0%	0.0%	0.0%	0.0%	0.0%
tr|F1SUZ4|F1SUZ4	1	1	2	0	0	0	0	0	1	2	3	0	0	0	0	0	1.8%	2.3%	5.1%	0.0%	0.0%	0.0%	0.0%	0.0%
Q09136	3	0	1	0	0	0	0	0	3	0	3	0	0	0	0	0	14.3%	0.0%	2.6%	0.0%	0.0%	0.0%	0.0%	0.0%
tr|F1RWY9|F1RWY9	3	0	2	0	0	0	0	0	3	0	3	0	0	0	0	0	33.3%	0.0%	32.5%	0.0%	0.0%	0.0%	0.0%	0.0%
tr|I3LNP5|I3LNP5	1	0	1	0	0	0	0	0	3	0	3	0	0	0	0	0	8.1%	0.0%	13.3%	0.0%	0.0%	0.0%	0.0%	0.0%
tr|F1RUW0|F1RUW0	1	1	2	0	0	0	0	0	2	1	3	0	0	0	0	0	2.5%	2.5%	5.1%	0.0%	0.0%	0.0%	0.0%	0.0%
tr|I3LKE2|I3LKE2	2	1	1	0	0	0	0	0	2	1	3	0	0	0	0	0	9.9%	7.1%	3.5%	0.0%	0.0%	0.0%	0.0%	0.0%
tr|F1SJU9|F1SJU9	2	1	1	0	0	0	0	0	2	1	3	0	0	0	0	0	9.0%	6.4%	3.2%	0.0%	0.0%	0.0%	0.0%	0.0%
tr|F1RYV6|F1RYV6	2	0	1	0	0	0	0	0	3	0	3	0	0	0	0	0	5.6%	0.0%	3.1%	0.0%	0.0%	0.0%	0.0%	0.0%
tr|F1SFI3|F1SFI3	1	2	1	0	0	0	0	0	1	2	4	0	0	0	0	0	3.3%	6.6%	5.5%	0.0%	0.0%	0.0%	0.0%	0.0%
tr|I3L752|I3L752	2	0	1	0	0	0	0	0	3	0	4	0	0	0	0	0	5.8%	0.0%	2.7%	0.0%	0.0%	0.0%	0.0%	0.0%
tr|F1S2R7|F1S2R7	1	1	1	0	0	0	0	0	2	1	4	0	0	0	0	0	5.1%	3.7%	5.1%	0.0%	0.0%	0.0%	0.0%	0.0%
tr|F1RXX6|F1RXX6	2	1	1	0	0	0	0	0	2	1	4	0	0	0	0	0	13.3%	10.4%	7.6%	0.0%	0.0%	0.0%	0.0%	0.0%
tr|F1SV67|F1SV67	3	0	2	0	0	0	0	0	3	0	4	0	0	0	0	0	17.4%	0.0%	8.7%	0.0%	0.0%	0.0%	0.0%	0.0%
tr|I3L7I5|I3L7I5	3	0	2	0	0	0	0	0	3	0	4	0	0	0	0	0	8.2%	0.0%	5.8%	0.0%	0.0%	0.0%	0.0%	0.0%
Q29259	1	1	1	0	0	0	0	0	2	1	4	0	0	0	0	0	8.6%	8.6%	7.8%	0.0%	0.0%	0.0%	0.0%	0.0%
tr|I3LL98|I3LL98	3	0	1	0	0	0	0	0	3	0	5	0	0	0	0	0	15.9%	0.0%	5.3%	0.0%	0.0%	0.0%	0.0%	0.0%
tr|F1S8V3|F1S8V3	2	0	1	0	0	0	0	0	3	0	5	0	0	0	0	0	3.3%	0.0%	3.7%	0.0%	0.0%	0.0%	0.0%	0.0%
tr|D2WL17|D2WL17	3	0	1	0	0	0	0	0	3	0	5	0	0	0	0	0	12.5%	0.0%	3.2%	0.0%	0.0%	0.0%	0.0%	0.0%
tr|F1RJQ9|F1RJQ9	1	1	1	0	0	0	0	0	2	1	5	0	0	0	0	0	14.0%	14.0%	14.0%	0.0%	0.0%	0.0%	0.0%	0.0%
tr|I3L6K0|I3L6K0	2	0	1	0	0	0	0	0	3	0	5	0	0	0	0	0	8.9%	0.0%	6.4%	0.0%	0.0%	0.0%	0.0%	0.0%
tr|I3LDM2|I3LDM2	2	0	1	0	0	0	0	0	3	0	5	0	0	0	0	0	9.2%	0.0%	6.6%	0.0%	0.0%	0.0%	0.0%	0.0%
tr|F1RPK5|F1RPK5	3	0	2	0	0	0	0	0	3	0	5	0	0	0	0	0	23.7%	0.0%	17.1%	0.0%	0.0%	0.0%	0.0%	0.0%
tr|I3LIC9|I3LIC9	2	0	1	0	0	0	0	0	3	0	5	0	0	0	0	0	9.0%	0.0%	6.5%	0.0%	0.0%	0.0%	0.0%	0.0%
tr|F2Z5J6|F2Z5J6	1	0	2	0	0	0	0	0	3	0	6	0	0	0	0	0	6.6%	0.0%	5.1%	0.0%	0.0%	0.0%	0.0%	0.0%
tr|I3LB10|I3LB10	1	1	2	0	0	0	0	0	2	1	6	0	0	0	0	0	2.0%	1.1%	3.6%	0.0%	0.0%	0.0%	0.0%	0.0%
tr|I3LDL8|I3LDL8	1	0	2	0	0	0	0	0	3	0	6	0	0	0	0	0	4.6%	0.0%	5.0%	0.0%	0.0%	0.0%	0.0%	0.0%
tr|F1S2Z9|F1S2Z9	2	0	2	0	0	0	0	0	3	0	6	0	0	0	0	0	8.4%	0.0%	7.9%	0.0%	0.0%	0.0%	0.0%	0.0%
tr|K7GQ75|K7GQ75	1	0	1	0	0	0	0	0	3	0	6	0	0	0	0	0	24.8%	0.0%	5.6%	0.0%	0.0%	0.0%	0.0%	0.0%
tr|I3LP62|I3LP62	2	0	1	0	0	0	0	0	3	0	6	0	0	0	0	0	11.5%	0.0%	13.9%	0.0%	0.0%	0.0%	0.0%	0.0%
tr|F1SM98|F1SM98	3	0	2	0	0	0	0	0	3	0	6	0	0	0	0	0	10.4%	0.0%	8.0%	0.0%	0.0%	0.0%	0.0%	0.0%
tr|K7GMV9|K7GMV9	2	0	1	0	0	0	0	0	3	0	7	0	0	0	0	0	18.6%	0.0%	5.1%	0.0%	0.0%	0.0%	0.0%	0.0%
tr|F1S1V5|F1S1V5	2	0	2	0	0	0	0	0	3	0	7	0	0	0	0	0	1.6%	0.0%	2.4%	0.0%	0.0%	0.0%	0.0%	0.0%
tr|F1SL16|F1SL16	1	2	2	0	0	0	0	0	1	2	7	0	0	0	0	0	8.4%	10.7%	17.4%	0.0%	0.0%	0.0%	0.0%	0.0%
tr|F1S9H9|F1S9H9	1	1	3	0	0	0	0	0	2	1	7	0	0	0	0	0	14.9%	8.8%	23.6%	0.0%	0.0%	0.0%	0.0%	0.0%
tr|F1RRY6|F1RRY6	2	1	2	0	0	0	0	0	2	1	7	0	0	0	0	0	12.1%	2.4%	8.7%	0.0%	0.0%	0.0%	0.0%	0.0%
tr|I3LG77|I3LG77	2	0	1	0	0	0	0	0	3	0	7	0	0	0	0	0	17.9%	0.0%	4.9%	0.0%	0.0%	0.0%	0.0%	0.0%
tr|K7GM22|K7GM22	2	1	4	0	0	0	0	0	2	1	8	0	0	0	0	0	10.4%	9.9%	14.4%	0.0%	0.0%	0.0%	0.0%	0.0%
tr|F1SLY2|F1SLY2	1	0	1	0	0	0	0	0	3	0	8	0	0	0	0	0	22.4%	0.0%	13.8%	0.0%	0.0%	0.0%	0.0%	0.0%
tr|F1SGT3|F1SGT3	1	1	2	0	0	0	0	0	1	2	8	0	0	0	0	0	2.8%	3.0%	4.8%	0.0%	0.0%	0.0%	0.0%	0.0%
P56472	1	0	1	0	0	0	0	0	3	0	8	0	0	0	0	0	19.4%	0.0%	18.4%	0.0%	0.0%	0.0%	0.0%	0.0%
tr|F1RW55|F1RW55	2	0	1	0	0	0	0	0	3	0	9	0	0	0	0	0	10.7%	0.0%	10.1%	0.0%	0.0%	0.0%	0.0%	0.0%
tr|F2Z5G2|F2Z5G2	3	0	3	0	0	0	0	0	3	0	9	0	0	0	0	0	3.9%	0.0%	4.9%	0.0%	0.0%	0.0%	0.0%	0.0%
tr|K7GRQ4|K7GRQ4	2	0	1	0	0	0	0	0	3	0	9	0	0	0	0	0	14.2%	0.0%	13.4%	0.0%	0.0%	0.0%	0.0%	0.0%
tr|F1SAU5|F1SAU5	2	1	2	0	0	0	0	0	2	1	10	0	0	0	0	0	3.4%	1.4%	3.7%	0.0%	0.0%	0.0%	0.0%	0.0%
tr|F1STI9|F1STI9	3	0	2	0	0	0	0	0	3	0	11	0	0	0	0	0	7.2%	0.0%	4.4%	0.0%	0.0%	0.0%	0.0%	0.0%
tr|F1SKU5|F1SKU5	2	1	2	0	0	0	0	0	2	1	11	0	0	0	0	0	6.4%	1.6%	3.4%	0.0%	0.0%	0.0%	0.0%	0.0%
tr|F1S8V6|F1S8V6	2	0	2	0	0	0	0	0	3	0	12	0	0	0	0	0	3.0%	0.0%	6.5%	0.0%	0.0%	0.0%	0.0%	0.0%
tr|F1STP1|F1STP1	2	0	3	0	0	0	0	0	3	0	12	0	0	0	0	0	15.0%	0.0%	21.4%	0.0%	0.0%	0.0%	0.0%	0.0%
tr|F1SGH9|F1SGH9	0	1	2	0	0	0	0	0	0	3	12	0	0	0	0	0	0.0%	1.5%	5.9%	0.0%	0.0%	0.0%	0.0%	0.0%
tr|F1RGP6|F1RGP6	2	0	3	0	0	0	0	0	3	0	12	0	0	0	0	0	4.9%	0.0%	8.3%	0.0%	0.0%	0.0%	0.0%	0.0%
tr|K7GPA0|K7GPA0	1	1	2	0	0	0	0	0	1	2	13	0	0	0	0	0	16.8%	8.8%	27.7%	0.0%	0.0%	0.0%	0.0%	0.0%
tr|F1SFJ8|F1SFJ8	2	0	2	0	0	0	0	0	3	0	15	0	0	0	0	0	9.2%	0.0%	8.4%	0.0%	0.0%	0.0%	0.0%	0.0%
tr|F1S8X9|F1S8X9	2	0	2	0	0	0	0	0	3	0	17	0	0	0	0	0	16.0%	0.0%	10.4%	0.0%	0.0%	0.0%	0.0%	0.0%
tr|F2Z525|F2Z525	3	0	6	0	0	0	0	0	3	0	18	0	0	0	0	0	38.9%	0.0%	70.1%	0.0%	0.0%	0.0%	0.0%	0.0%
tr|J9JIL9|J9JIL9	3	0	1	0	0	0	0	0	3	0	19	0	0	0	0	0	8.8%	0.0%	5.2%	0.0%	0.0%	0.0%	0.0%	0.0%
P80147	3	0	1	0	0	0	0	0	3	0	19	0	0	0	0	0	8.4%	0.0%	5.0%	0.0%	0.0%	0.0%	0.0%	0.0%
tr|F1RPW8|F1RPW8	3	0	1	0	0	0	0	0	3	0	21	0	0	0	0	0	7.4%	0.0%	6.1%	0.0%	0.0%	0.0%	0.0%	0.0%
tr|F1RJF7|F1RJF7	2	0	5	0	0	0	0	0	3	0	23	0	0	0	0	0	6.9%	0.0%	14.1%	0.0%	0.0%	0.0%	0.0%	0.0%
tr|I3LIA4|I3LIA4	2	1	2	0	0	0	0	0	2	1	28	0	0	0	0	0	13.1%	5.9%	6.4%	0.0%	0.0%	0.0%	0.0%	0.0%
tr|I3LN38|I3LN38	2	1	2	0	0	0	0	0	2	1	36	0	0	0	0	0	5.4%	3.0%	5.4%	0.0%	0.0%	0.0%	0.0%	0.0%
tr|I3LR40|I3LR40	2	0	2	0	0	0	0	0	3	0	40	0	0	0	0	0	8.0%	0.0%	6.8%	0.0%	0.0%	0.0%	0.0%	0.0%
tr|I3LF95|I3LF95	1	0	8	0	0	0	14	34	2	0	43	0	0	0	524	524	8.0%	0.0%	45.3%	0.0%	0.0%	0.0%	61.3%	70.5%
tr|K7GRZ6|K7GRZ6	0	1	0	0	0	0	3	2	0	2	0	0	0	0	25	14	0.0%	16.9%	0.0%	0.0%	0.0%	0.0%	30.5%	16.9%
tr|I3LF22|I3LF22	2	0	0	0	0	0	1	7	2	0	0	0	0	0	4	26	17.1%	0.0%	0.0%	0.0%	0.0%	0.0%	12.2%	39.2%

	Peptide Count								Spectral Count								Sequence Coverage							
	Whole Cell	Whole Cell	Whole Cell	Cilia Fraction	Cilia Fraction	Cilia Fraction	Cilia Fraction	Cilia Fraction	Whole Cell	Whole Cell	Whole Cell	Cilia Fraction	Cilia Fraction	Cilia Fraction	Cilia Fraction	Cilia Fraction	Whole Cell	Whole Cell	Whole Cell	Cilia Fraction	Cilia Fraction	Cilia Fraction	Cilia Fraction	Cilia Fraction
Sample#	1	2	6	4	3	5	7	8	1	2	6	4	3	5	7	8	1	2	6	4	3	5	7	8
**Accession**																								
tr|F1SIC6|F1SIC6	1	1	0	0	0	0	2	4	1	1	0	0	0	0	7	14	3.4%	8.2%	0.0%	0.0%	0.0%	0.0%	8.2%	30.9%
O02713	2	0	2	0	0	0	2	7	2	0	10	0	0	0	40	76	1.8%	0.0%	3.2%	0.0%	0.0%	0.0%	7.6%	15.0%
tr|F1SB79|F1SB79	2	0	2	0	0	0	2	7	2	0	10	0	0	0	40	76	1.8%	0.0%	3.2%	0.0%	0.0%	0.0%	7.6%	15.0%
tr|F1RFB4|F1RFB4	2	0	1	0	0	0	2	3	2	0	1	0	0	0	14	15	9.9%	0.0%	5.4%	0.0%	0.0%	0.0%	9.6%	14.3%
tr|I3LGD9|I3LGD9	1	1	1	0	0	0	2	6	1	1	1	0	0	0	7	19	0.6%	0.6%	0.9%	0.0%	0.0%	0.0%	1.1%	3.7%
tr|I3LIZ3|I3LIZ3	1	1	0	0	0	0	2	2	1	1	0	0	0	0	7	9	7.3%	17.5%	0.0%	0.0%	0.0%	0.0%	17.5%	24.8%
Q29000	2	0	1	0	0	0	1	4	2	0	2	0	0	0	6	23	10.9%	0.0%	8.9%	0.0%	0.0%	0.0%	5.6%	31.6%
tr|F1SSM0|F1SSM0	2	0	0	0	0	0	0	4	2	0	0	0	0	0	0	13	6.6%	0.0%	0.0%	0.0%	0.0%	0.0%	0.0%	15.3%
tr|F1RLC9|F1RLC9	2	0	0	0	0	0	1	1	2	0	0	0	0	0	9	1	10.6%	0.0%	0.0%	0.0%	0.0%	0.0%	10.6%	10.6%
tr|I3LQ16|I3LQ16	2	0	1	0	0	0	1	1	2	0	1	0	0	0	7	5	36.1%	0.0%	16.7%	0.0%	0.0%	0.0%	16.7%	16.7%
tr|I3LTB8|I3LTB8	1	0	0	0	0	0	1	1	2	0	0	0	0	0	3	5	4.6%	0.0%	0.0%	0.0%	0.0%	0.0%	4.6%	4.6%
tr|F1SV97|F1SV97	2	0	0	0	0	0	0	3	2	0	0	0	0	0	0	8	25.7%	0.0%	0.0%	0.0%	0.0%	0.0%	0.0%	25.7%
tr|F1RRH2|F1RRH2	2	0	0	0	0	0	0	3	2	0	0	0	0	0	0	7	3.8%	0.0%	0.0%	0.0%	0.0%	0.0%	0.0%	5.6%
tr|I3LHE6|I3LHE6	2	0	0	0	0	0	0	2	2	0	0	0	0	0	0	6	3.9%	0.0%	0.0%	0.0%	0.0%	0.0%	0.0%	3.3%
tr|F2Z5H6|F2Z5H6	2	0	2	0	0	0	1	3	2	0	9	0	0	0	2	30	10.3%	0.0%	5.1%	0.0%	0.0%	0.0%	5.1%	20.8%
tr|F1SPU7|F1SPU7	1	0	0	0	0	0	0	3	2	0	0	0	0	0	0	5	2.1%	0.0%	0.0%	0.0%	0.0%	0.0%	0.0%	7.8%
Q5GN48	1	0	0	0	0	0	0	3	2	0	0	0	0	0	0	5	0.4%	0.0%	0.0%	0.0%	0.0%	0.0%	0.0%	1.4%
tr|F1RPY5|F1RPY5	1	1	0	0	0	0	1	0	1	1	0	0	0	0	5	0	5.7%	2.7%	0.0%	0.0%	0.0%	0.0%	2.7%	0.0%
tr|F1RQ24|F1RQ24	2	0	0	0	0	0	0	2	2	0	0	0	0	0	0	5	10.4%	0.0%	0.0%	0.0%	0.0%	0.0%	0.0%	10.4%
tr|I3LGE5|I3LGE5	2	0	5	0	0	0	1	10	2	0	20	0	0	0	10	41	3.0%	0.0%	10.7%	0.0%	0.0%	0.0%	1.6%	16.4%
tr|F1RSA2|F1RSA2	1	1	0	0	0	0	0	1	1	1	0	0	0	0	0	4	1.1%	1.2%	0.0%	0.0%	0.0%	0.0%	0.0%	3.4%
tr|F1SF12|F1SF12	0	2	0	0	0	0	1	0	0	2	0	0	0	0	4	0	0.0%	14.3%	0.0%	0.0%	0.0%	0.0%	10.9%	0.0%
tr|F1S3T8|F1S3T8	1	0	0	0	0	0	0	1	2	0	0	0	0	0	0	4	14.8%	0.0%	0.0%	0.0%	0.0%	0.0%	0.0%	14.8%
tr|F1S614|F1S614	1	1	0	0	0	0	1	1	1	1	0	0	0	0	1	3	2.5%	2.0%	0.0%	0.0%	0.0%	0.0%	2.0%	2.5%
tr|I3L804|I3L804	2	0	1	0	0	0	0	9	2	0	14	0	0	0	0	30	2.3%	0.0%	1.7%	0.0%	0.0%	0.0%	0.0%	10.0%
tr|F1S6Z8|F1S6Z8	1	1	1	0	0	0	1	1	1	1	3	0	0	0	8	1	9.8%	9.8%	4.1%	0.0%	0.0%	0.0%	9.8%	9.8%
tr|F1SQ70|F1SQ70	2	0	2	0	0	0	1	1	2	0	12	0	0	0	20	5	35.5%	0.0%	20.6%	0.0%	0.0%	0.0%	20.6%	20.6%
tr|I3LQP2|I3LQP2	2	0	4	0	0	0	0	4	2	0	12	0	0	0	0	24	5.8%	0.0%	13.9%	0.0%	0.0%	0.0%	0.0%	14.2%
tr|F1SK02|F1SK02	0	2	1	0	0	0	0	1	0	2	1	0	0	0	0	5	0.0%	9.7%	5.8%	0.0%	0.0%	0.0%	0.0%	11.9%
tr|I3LU94|I3LU94	1	0	3	0	0	0	1	1	2	0	6	0	0	0	10	3	14.1%	0.0%	14.7%	0.0%	0.0%	0.0%	12.5%	12.5%
tr|F1RM44|F1RM44	2	0	0	0	0	0	0	2	2	0	0	0	0	0	0	3	15.6%	0.0%	0.0%	0.0%	0.0%	0.0%	0.0%	15.6%
tr|I3LPH8|I3LPH8	1	0	0	0	0	0	0	2	2	0	0	0	0	0	0	3	2.9%	0.0%	0.0%	0.0%	0.0%	0.0%	0.0%	7.0%
tr|I3LIN8|I3LIN8	2	0	0	0	0	0	0	2	2	0	0	0	0	0	0	3	11.1%	0.0%	0.0%	0.0%	0.0%	0.0%	0.0%	9.0%
tr|F1RHC5|F1RHC5	2	0	0	0	0	0	0	2	2	0	0	0	0	0	0	3	11.1%	0.0%	0.0%	0.0%	0.0%	0.0%	0.0%	8.9%
tr|K7GSN9|K7GSN9	1	0	0	0	0	0	0	1	2	0	0	0	0	0	0	3	9.4%	0.0%	0.0%	0.0%	0.0%	0.0%	0.0%	9.4%
tr|F1SER9|F1SER9	1	0	0	0	0	0	0	1	2	0	0	0	0	0	0	3	0.8%	0.0%	0.0%	0.0%	0.0%	0.0%	0.0%	0.4%
tr|F1RJT3|F1RJT3	1	1	1	0	0	0	1	1	1	1	6	0	0	0	6	5	3.3%	3.3%	3.3%	0.0%	0.0%	0.0%	4.1%	4.1%
tr|F2Z5I7|F2Z5I7	2	0	4	0	0	0	1	3	2	0	15	0	0	0	13	8	7.8%	0.0%	12.1%	0.0%	0.0%	0.0%	4.7%	9.2%
tr|I3LCG1|I3LCG1	2	0	1	0	0	0	0	2	2	0	3	0	0	0	0	6	4.0%	0.0%	3.6%	0.0%	0.0%	0.0%	0.0%	3.4%
tr|I3LT56|I3LT56	1	1	2	0	0	0	0	4	1	1	7	0	0	0	0	10	4.3%	4.3%	13.0%	0.0%	0.0%	0.0%	0.0%	21.5%
tr|F1S9C5|F1S9C5	2	0	1	0	0	0	0	1	2	0	2	0	0	0	0	4	18.1%	0.0%	5.3%	0.0%	0.0%	0.0%	0.0%	5.3%
tr|I3LJZ9|I3LJZ9	2	0	1	0	0	0	1	0	2	0	1	0	0	0	3	0	24.4%	0.0%	11.0%	0.0%	0.0%	0.0%	12.2%	0.0%
tr|I3LSM5|I3LSM5	1	0	0	0	0	0	0	1	2	0	0	0	0	0	0	2	4.6%	0.0%	0.0%	0.0%	0.0%	0.0%	0.0%	4.6%
tr|I3LCF3|I3LCF3	1	1	0	0	0	0	0	2	1	1	0	0	0	0	0	2	4.4%	1.7%	0.0%	0.0%	0.0%	0.0%	0.0%	8.4%
tr|F1SCG1|F1SCG1	2	0	0	0	0	0	0	1	2	0	0	0	0	0	0	2	4.0%	0.0%	0.0%	0.0%	0.0%	0.0%	0.0%	3.5%
tr|F1SJC6|F1SJC6	1	0	0	0	0	0	1	0	2	0	0	0	0	0	2	0	2.9%	0.0%	0.0%	0.0%	0.0%	0.0%	3.8%	0.0%
P15983	1	0	0	0	0	0	0	1	2	0	0	0	0	0	0	2	8.9%	0.0%	0.0%	0.0%	0.0%	0.0%	0.0%	8.9%
P15982	1	0	0	0	0	0	0	1	2	0	0	0	0	0	0	2	8.8%	0.0%	0.0%	0.0%	0.0%	0.0%	0.0%	8.8%
tr|F1S9P4|F1S9P4	1	1	0	0	0	0	0	1	1	1	0	0	0	0	0	2	3.0%	2.5%	0.0%	0.0%	0.0%	0.0%	0.0%	3.5%
tr|I3LL45|I3LL45	2	0	0	0	0	0	0	2	2	0	0	0	0	0	0	2	26.1%	0.0%	0.0%	0.0%	0.0%	0.0%	0.0%	10.6%
tr|Q8MGP9|Q8MGP9	1	0	0	0	0	0	0	1	2	0	0	0	0	0	0	2	8.8%	0.0%	0.0%	0.0%	0.0%	0.0%	0.0%	8.8%
tr|F1RY74|F1RY74	1	0	0	0	0	0	0	1	2	0	0	0	0	0	0	2	4.6%	0.0%	0.0%	0.0%	0.0%	0.0%	0.0%	4.6%
tr|F1SUW7|F1SUW7	2	0	0	0	0	0	0	1	2	0	0	0	0	0	0	2	2.5%	0.0%	0.0%	0.0%	0.0%	0.0%	0.0%	1.4%
tr|F1S5M0|F1S5M0	1	0	3	0	0	0	0	2	2	0	22	0	0	0	0	23	8.8%	0.0%	8.8%	0.0%	0.0%	0.0%	0.0%	8.8%
Q29056	2	0	2	0	0	0	1	0	2	0	4	0	0	0	5	0	10.7%	0.0%	10.7%	0.0%	0.0%	0.0%	20.3%	0.0%
tr|I3LP69|I3LP69	1	0	1	0	0	0	0	1	2	0	2	0	0	0	0	3	8.5%	0.0%	8.8%	0.0%	0.0%	0.0%	0.0%	8.5%
tr|F1RR73|F1RR73	0	1	1	0	0	0	0	1	0	2	4	0	0	0	0	4	0.0%	5.7%	16.6%	0.0%	0.0%	0.0%	0.0%	5.7%
tr|F1SLT1|F1SLT1	2	0	1	0	0	0	0	2	2	0	1	0	0	0	0	2	17.4%	0.0%	7.3%	0.0%	0.0%	0.0%	0.0%	18.2%
tr|F1S031|F1S031	2	0	2	0	0	0	0	1	2	0	11	0	0	0	0	8	12.2%	0.0%	20.3%	0.0%	0.0%	0.0%	0.0%	12.2%
tr|I3L8U8|I3L8U8	1	0	2	0	0	0	0	1	2	0	9	0	0	0	0	6	18.3%	0.0%	8.5%	0.0%	0.0%	0.0%	0.0%	18.3%
P31006	1	0	0	0	0	0	0	1	2	0	0	0	0	0	0	1	11.1%	0.0%	0.0%	0.0%	0.0%	0.0%	0.0%	11.1%
tr|F1RPR6|F1RPR6	1	0	0	0	0	0	0	1	2	0	0	0	0	0	0	1	9.9%	0.0%	0.0%	0.0%	0.0%	0.0%	0.0%	4.0%
tr|F1S2Q4|F1S2Q4	0	1	0	0	0	0	0	1	0	2	0	0	0	0	0	1	0.0%	1.3%	0.0%	0.0%	0.0%	0.0%	0.0%	3.0%
tr|F1SFY5|F1SFY5	2	0	0	0	0	0	0	1	2	0	0	0	0	0	0	1	4.6%	0.0%	0.0%	0.0%	0.0%	0.0%	0.0%	2.9%
tr|I3LJ78|I3LJ78	2	0	0	0	0	0	0	1	2	0	0	0	0	0	0	1	10.0%	0.0%	0.0%	0.0%	0.0%	0.0%	0.0%	3.6%
P58780	2	0	0	0	0	0	0	1	2	0	0	0	0	0	0	1	7.1%	0.0%	0.0%	0.0%	0.0%	0.0%	0.0%	5.3%
tr|F1S5R9|F1S5R9	1	0	0	0	0	0	0	1	2	0	0	0	0	0	0	1	11.1%	0.0%	0.0%	0.0%	0.0%	0.0%	0.0%	11.1%
tr|F1RKH4|F1RKH4	2	0	0	0	0	0	1	0	2	0	0	0	0	0	1	0	21.4%	0.0%	0.0%	0.0%	0.0%	0.0%	13.3%	0.0%
tr|I3LBJ3|I3LBJ3	2	0	0	0	0	0	0	1	2	0	0	0	0	0	0	1	7.9%	0.0%	0.0%	0.0%	0.0%	0.0%	0.0%	4.3%
tr|F1SLF4|F1SLF4	2	0	0	0	0	0	0	1	2	0	0	0	0	0	0	1	11.8%	0.0%	0.0%	0.0%	0.0%	0.0%	0.0%	6.2%
tr|K7GPA4|K7GPA4	1	0	0	0	0	0	1	0	2	0	0	0	0	0	1	0	14.5%	0.0%	0.0%	0.0%	0.0%	0.0%	14.5%	0.0%
tr|F1SHG3|F1SHG3	2	0	0	0	0	0	0	1	2	0	0	0	0	0	0	1	16.2%	0.0%	0.0%	0.0%	0.0%	0.0%	0.0%	12.6%
tr|Q00P49|Q00P49	1	1	0	0	0	0	0	1	1	1	0	0	0	0	0	1	4.1%	2.8%	0.0%	0.0%	0.0%	0.0%	0.0%	4.1%
tr|K7GMY1|K7GMY1	1	0	0	0	0	0	1	0	2	0	0	0	0	0	1	0	25.7%	0.0%	0.0%	0.0%	0.0%	0.0%	25.7%	0.0%
tr|F1S6Q7|F1S6Q7	1	1	2	0	0	0	1	0	1	1	8	0	0	0	4	0	8.3%	8.3%	28.6%	0.0%	0.0%	0.0%	20.2%	0.0%
tr|I3LK21|I3LK21	0	1	2	0	0	0	1	1	0	2	10	0	0	0	3	1	0.0%	3.3%	7.0%	0.0%	0.0%	0.0%	3.3%	3.3%
tr|I3LS73|I3LS73	2	0	1	0	0	0	1	0	2	0	1	0	0	0	1	0	15.8%	0.0%	15.8%	0.0%	0.0%	0.0%	15.8%	0.0%
tr|I3LAS2|I3LAS2	2	0	1	0	0	0	0	1	2	0	1	0	0	0	0	1	3.8%	0.0%	1.9%	0.0%	0.0%	0.0%	0.0%	3.6%
tr|I3LJJ4|I3LJJ4	1	1	1	0	0	0	1	0	1	1	1	0	0	0	1	0	9.1%	7.0%	9.1%	0.0%	0.0%	0.0%	7.0%	0.0%
tr|I3LMN1|I3LMN1	2	0	1	0	0	0	1	0	2	0	6	0	0	0	2	0	9.5%	0.0%	9.9%	0.0%	0.0%	0.0%	9.5%	0.0%
tr|F1RGL0|F1RGL0	2	0	1	0	0	0	1	0	2	0	6	0	0	0	2	0	8.7%	0.0%	9.1%	0.0%	0.0%	0.0%	8.7%	0.0%

	Peptide Count								Spectral Count								Sequence Coverage							
	Whole Cell	Whole Cell	Whole Cell	Cilia Fraction	Cilia Fraction	Cilia Fraction	Cilia Fraction	Cilia Fraction	Whole Cell	Whole Cell	Whole Cell	Cilia Fraction	Cilia Fraction	Cilia Fraction	Cilia Fraction	Cilia Fraction	Whole Cell	Whole Cell	Whole Cell	Cilia Fraction	Cilia Fraction	Cilia Fraction	Cilia Fraction	Cilia Fraction
Sample#	1	2	6	4	3	5	7	8	1	2	6	4	3	5	7	8	1	2	6	4	3	5	7	8
**Accession**																								
tr|F1SD90|F1SD90	2	0	1	0	0	0	1	0	2	0	2	0	0	0	1	0	5.1%	0.0%	1.6%	0.0%	0.0%	0.0%	2.3%	0.0%
tr|I3LRM5|I3LRM5	1	0	1	0	0	0	1	0	2	0	7	0	0	0	2	0	1.5%	0.0%	1.5%	0.0%	0.0%	0.0%	1.5%	0.0%
tr|I3LCL4|I3LCL4	1	0	1	0	0	0	1	0	2	0	7	0	0	0	2	0	0.7%	0.0%	0.7%	0.0%	0.0%	0.0%	0.7%	0.0%
tr|F1RJW8|F1RJW8	1	0	1	0	0	0	0	1	2	0	3	0	0	0	0	1	3.5%	0.0%	2.9%	0.0%	0.0%	0.0%	0.0%	3.5%
tr|A7YX24|A7YX24	2	0	2	0	0	0	1	1	2	0	15	0	0	0	1	1	30.2%	0.0%	20.6%	0.0%	0.0%	0.0%	12.7%	12.7%
tr|I3LS60|I3LS60	2	0	6	0	0	0	0	1	2	0	26	0	0	0	0	3	11.0%	0.0%	21.3%	0.0%	0.0%	0.0%	0.0%	5.5%
tr|F1SCM9|F1SCM9	2	0	2	0	0	0	0	1	2	0	8	0	0	0	0	1	3.7%	0.0%	4.0%	0.0%	0.0%	0.0%	0.0%	3.5%
tr|K7GPA8|K7GPA8	1	0	1	0	0	0	0	1	2	0	8	0	0	0	0	1	14.0%	0.0%	4.5%	0.0%	0.0%	0.0%	0.0%	4.5%
tr|K7GQ44|K7GQ44	2	0	3	0	0	0	0	1	2	0	9	0	0	0	0	1	5.8%	0.0%	9.6%	0.0%	0.0%	0.0%	0.0%	3.7%
tr|K7GR37|K7GR37	2	0	3	0	0	0	0	1	2	0	9	0	0	0	0	1	5.9%	0.0%	9.6%	0.0%	0.0%	0.0%	0.0%	3.7%
tr|K7GKL7|K7GKL7	2	0	3	0	0	0	0	1	2	0	9	0	0	0	0	1	5.8%	0.0%	9.5%	0.0%	0.0%	0.0%	0.0%	3.7%
tr|F1RUA1|F1RUA1	2	0	3	0	0	0	0	1	2	0	9	0	0	0	0	1	5.8%	0.0%	9.6%	0.0%	0.0%	0.0%	0.0%	3.7%
tr|B0LXP3|B0LXP3	2	0	1	0	0	0	0	1	2	0	9	0	0	0	0	1	5.1%	0.0%	2.5%	0.0%	0.0%	0.0%	0.0%	2.5%
tr|I3LRH9|I3LRH9	1	1	2	0	0	0	1	0	1	1	14	0	0	0	1	0	9.6%	7.3%	24.3%	0.0%	0.0%	0.0%	7.3%	0.0%
tr|I3LKQ2|I3LKQ2	0	1	4	0	0	0	0	1	0	2	14	0	0	0	0	1	0.0%	6.9%	22.4%	0.0%	0.0%	0.0%	0.0%	10.3%
P20192	1	0	1	0	0	0	0	1	2	0	14	0	0	0	0	1	2.3%	0.0%	2.6%	0.0%	0.0%	0.0%	0.0%	2.3%
tr|F1RVD2|F1RVD2	1	1	4	0	0	0	1	0	1	1	18	0	0	0	1	0	8.4%	10.3%	10.7%	0.0%	0.0%	0.0%	5.0%	0.0%
tr|K7GLT1|K7GLT1	2	0	2	0	0	0	0	2	2	0	206	0	0	0	0	7	23.8%	0.0%	23.8%	0.0%	0.0%	0.0%	0.0%	23.8%
tr|F1S8V2|F1S8V2	2	0	0	0	0	0	0	0	2	0	0	0	0	0	0	0	6.3%	0.0%	0.0%	0.0%	0.0%	0.0%	0.0%	0.0%
tr|F1RP77|F1RP77	1	1	0	0	0	0	0	0	1	1	0	0	0	0	0	0	0.2%	0.5%	0.0%	0.0%	0.0%	0.0%	0.0%	0.0%
tr|F1SUU8|F1SUU8	1	1	0	0	0	0	0	0	1	1	0	0	0	0	0	0	2.7%	3.3%	0.0%	0.0%	0.0%	0.0%	0.0%	0.0%
tr|F1RW97|F1RW97	1	0	0	0	0	0	0	0	2	0	0	0	0	0	0	0	6.6%	0.0%	0.0%	0.0%	0.0%	0.0%	0.0%	0.0%
tr|F1RS13|F1RS13	2	0	0	0	0	0	0	0	2	0	0	0	0	0	0	0	13.1%	0.0%	0.0%	0.0%	0.0%	0.0%	0.0%	0.0%
tr|K7GM04|K7GM04	2	0	0	0	0	0	0	0	2	0	0	0	0	0	0	0	7.9%	0.0%	0.0%	0.0%	0.0%	0.0%	0.0%	0.0%
tr|I3LQP7|I3LQP7	1	1	0	0	0	0	0	0	1	1	0	0	0	0	0	0	3.9%	3.9%	0.0%	0.0%	0.0%	0.0%	0.0%	0.0%
tr|I3L6H8|I3L6H8	2	0	0	0	0	0	0	0	2	0	0	0	0	0	0	0	9.0%	0.0%	0.0%	0.0%	0.0%	0.0%	0.0%	0.0%
tr|I3L710|I3L710	2	0	0	0	0	0	0	0	2	0	0	0	0	0	0	0	6.8%	0.0%	0.0%	0.0%	0.0%	0.0%	0.0%	0.0%
tr|I3LJ45|I3LJ45	2	0	0	0	0	0	0	0	2	0	0	0	0	0	0	0	7.4%	0.0%	0.0%	0.0%	0.0%	0.0%	0.0%	0.0%
tr|F2Z5W5|F2Z5W5	1	1	0	0	0	0	0	0	1	1	0	0	0	0	0	0	3.1%	2.6%	0.0%	0.0%	0.0%	0.0%	0.0%	0.0%
tr|F1S2X6|F1S2X6	0	1	0	0	0	0	0	0	0	2	0	0	0	0	0	0	0.0%	3.0%	0.0%	0.0%	0.0%	0.0%	0.0%	0.0%
tr|I3LGV1|I3LGV1	0	2	0	0	0	0	0	0	0	2	0	0	0	0	0	0	0.0%	3.6%	0.0%	0.0%	0.0%	0.0%	0.0%	0.0%
tr|F1S120|F1S120	1	0	0	0	0	0	0	0	2	0	0	0	0	0	0	0	17.8%	0.0%	0.0%	0.0%	0.0%	0.0%	0.0%	0.0%
tr|K7GML9|K7GML9	2	0	0	0	0	0	0	0	2	0	0	0	0	0	0	0	8.0%	0.0%	0.0%	0.0%	0.0%	0.0%	0.0%	0.0%
tr|I3L7W6|I3L7W6	1	1	0	0	0	0	0	0	1	1	0	0	0	0	0	0	2.6%	2.4%	0.0%	0.0%	0.0%	0.0%	0.0%	0.0%
tr|F1SJI2|F1SJI2	0	1	0	0	0	0	0	0	0	2	0	0	0	0	0	0	0.0%	3.4%	0.0%	0.0%	0.0%	0.0%	0.0%	0.0%
tr|I3LVS3|I3LVS3	1	0	0	0	0	0	0	0	2	0	0	0	0	0	0	0	20.2%	0.0%	0.0%	0.0%	0.0%	0.0%	0.0%	0.0%
tr|F1S0Z4|F1S0Z4	0	1	0	0	0	0	0	0	0	2	0	0	0	0	0	0	0.0%	8.0%	0.0%	0.0%	0.0%	0.0%	0.0%	0.0%
tr|I3LB23|I3LB23	2	0	0	0	0	0	0	0	2	0	0	0	0	0	0	0	10.0%	0.0%	0.0%	0.0%	0.0%	0.0%	0.0%	0.0%
Reverse_tr|F1SGT7|F1SGT7	1	0	0	0	0	0	0	0	2	0	0	0	0	0	0	0	0.8%	0.0%	0.0%	0.0%	0.0%	0.0%	0.0%	0.0%
tr|F1RP47|F1RP47	0	1	0	0	0	0	0	0	0	2	0	0	0	0	0	0	0.0%	2.1%	0.0%	0.0%	0.0%	0.0%	0.0%	0.0%
tr|K7GSV8|K7GSV8	1	1	0	0	0	0	0	0	1	1	0	0	0	0	0	0	6.2%	6.2%	0.0%	0.0%	0.0%	0.0%	0.0%	0.0%
tr|F1RW61|F1RW61	1	0	0	0	0	0	0	0	2	0	0	0	0	0	0	0	14.0%	0.0%	0.0%	0.0%	0.0%	0.0%	0.0%	0.0%
tr|I3LS35|I3LS35	1	1	0	0	0	0	0	0	1	1	0	0	0	0	0	0	4.9%	4.0%	0.0%	0.0%	0.0%	0.0%	0.0%	0.0%
tr|F1S902|F1S902	0	1	0	0	0	0	0	0	0	2	0	0	0	0	0	0	0.0%	0.9%	0.0%	0.0%	0.0%	0.0%	0.0%	0.0%
tr|I3L9I8|I3L9I8	1	0	0	0	0	0	0	0	2	0	0	0	0	0	0	0	8.6%	0.0%	0.0%	0.0%	0.0%	0.0%	0.0%	0.0%
tr|D0G6R6|D0G6R6	1	1	0	0	0	0	0	0	1	1	0	0	0	0	0	0	6.1%	5.2%	0.0%	0.0%	0.0%	0.0%	0.0%	0.0%
tr|F1SUI9|F1SUI9	2	0	0	0	0	0	0	0	2	0	0	0	0	0	0	0	6.1%	0.0%	0.0%	0.0%	0.0%	0.0%	0.0%	0.0%
tr|I3LEB1|I3LEB1	1	0	0	0	0	0	0	0	2	0	0	0	0	0	0	0	4.7%	0.0%	0.0%	0.0%	0.0%	0.0%	0.0%	0.0%
tr|A7L861|A7L861	1	1	0	0	0	0	0	0	1	1	0	0	0	0	0	0	2.6%	1.9%	0.0%	0.0%	0.0%	0.0%	0.0%	0.0%
tr|F1S5I0|F1S5I0	1	1	0	0	0	0	0	0	1	1	0	0	0	0	0	0	4.1%	1.4%	0.0%	0.0%	0.0%	0.0%	0.0%	0.0%
tr|F1SQ87|F1SQ87	1	1	0	0	0	0	0	0	1	1	0	0	0	0	0	0	2.3%	2.5%	0.0%	0.0%	0.0%	0.0%	0.0%	0.0%
tr|F1S7S8|F1S7S8	2	0	0	0	0	0	0	0	2	0	0	0	0	0	0	0	4.0%	0.0%	0.0%	0.0%	0.0%	0.0%	0.0%	0.0%
tr|F1SUE6|F1SUE6	0	1	0	0	0	0	0	0	0	2	0	0	0	0	0	0	0.0%	1.6%	0.0%	0.0%	0.0%	0.0%	0.0%	0.0%
tr|F1SN51|F1SN51	2	0	0	0	0	0	0	0	2	0	0	0	0	0	0	0	13.4%	0.0%	0.0%	0.0%	0.0%	0.0%	0.0%	0.0%
tr|I3LH48|I3LH48	1	1	0	0	0	0	0	0	1	1	0	0	0	0	0	0	10.3%	7.7%	0.0%	0.0%	0.0%	0.0%	0.0%	0.0%
tr|F1S6C2|F1S6C2	2	0	0	0	0	0	0	0	2	0	0	0	0	0	0	0	9.2%	0.0%	0.0%	0.0%	0.0%	0.0%	0.0%	0.0%
tr|F1SRE7|F1SRE7	2	0	0	0	0	0	0	0	2	0	0	0	0	0	0	0	32.3%	0.0%	0.0%	0.0%	0.0%	0.0%	0.0%	0.0%
Q4KRV1	2	0	0	0	0	0	0	0	2	0	0	0	0	0	0	0	5.8%	0.0%	0.0%	0.0%	0.0%	0.0%	0.0%	0.0%
tr|F2Z5R2|F2Z5R2	1	1	0	0	0	0	0	0	1	1	0	0	0	0	0	0	6.5%	6.5%	0.0%	0.0%	0.0%	0.0%	0.0%	0.0%
tr|F1SQC5|F1SQC5	0	2	0	0	0	0	0	0	0	2	0	0	0	0	0	0	0.0%	2.1%	0.0%	0.0%	0.0%	0.0%	0.0%	0.0%
tr|I3LFF9|I3LFF9	1	0	0	0	0	0	0	0	2	0	0	0	0	0	0	0	16.5%	0.0%	0.0%	0.0%	0.0%	0.0%	0.0%	0.0%
tr|F1SM56|F1SM56	1	0	0	0	0	0	0	0	2	0	0	0	0	0	0	0	10.9%	0.0%	0.0%	0.0%	0.0%	0.0%	0.0%	0.0%
tr|F1RJV2|F1RJV2	2	0	0	0	0	0	0	0	2	0	0	0	0	0	0	0	2.7%	0.0%	0.0%	0.0%	0.0%	0.0%	0.0%	0.0%
tr|K7GPW5|K7GPW5	2	0	0	0	0	0	0	0	2	0	0	0	0	0	0	0	5.8%	0.0%	0.0%	0.0%	0.0%	0.0%	0.0%	0.0%
tr|F1RSS7|F1RSS7	0	1	0	0	0	0	0	0	0	2	0	0	0	0	0	0	0.0%	5.4%	0.0%	0.0%	0.0%	0.0%	0.0%	0.0%
tr|F1SQW8|F1SQW8	2	0	0	0	0	0	0	0	2	0	0	0	0	0	0	0	23.0%	0.0%	0.0%	0.0%	0.0%	0.0%	0.0%	0.0%
tr|I3LFB9|I3LFB9	0	1	0	0	0	0	0	0	0	2	0	0	0	0	0	0	0.0%	3.4%	0.0%	0.0%	0.0%	0.0%	0.0%	0.0%
tr|F1RZ75|F1RZ75	0	1	0	0	0	0	0	0	0	2	0	0	0	0	0	0	0.0%	2.4%	0.0%	0.0%	0.0%	0.0%	0.0%	0.0%
tr|F2Z5H3|F2Z5H3	1	0	0	0	0	0	0	0	2	0	0	0	0	0	0	0	4.9%	0.0%	0.0%	0.0%	0.0%	0.0%	0.0%	0.0%
tr|F1RYL9|F1RYL9	1	1	0	0	0	0	0	0	1	1	0	0	0	0	0	0	2.1%	1.6%	0.0%	0.0%	0.0%	0.0%	0.0%	0.0%
tr|I3L902|I3L902	1	1	0	0	0	0	0	0	1	1	0	0	0	0	0	0	5.5%	7.5%	0.0%	0.0%	0.0%	0.0%	0.0%	0.0%
tr|F1SD94|F1SD94	0	1	0	0	0	0	0	0	0	2	0	0	0	0	0	0	0.0%	4.5%	0.0%	0.0%	0.0%	0.0%	0.0%	0.0%
tr|F1S8P8|F1S8P8	1	1	0	0	0	0	0	0	1	1	0	0	0	0	0	0	2.5%	1.4%	0.0%	0.0%	0.0%	0.0%	0.0%	0.0%
tr|F1S3N9|F1S3N9	1	0	0	0	0	0	0	0	2	0	0	0	0	0	0	0	1.6%	0.0%	0.0%	0.0%	0.0%	0.0%	0.0%	0.0%
tr|F1SKV6|F1SKV6	0	1	0	0	0	0	0	0	0	2	0	0	0	0	0	0	0.0%	7.2%	0.0%	0.0%	0.0%	0.0%	0.0%	0.0%
tr|I3LIY6|I3LIY6	1	0	0	0	0	0	0	0	2	0	0	0	0	0	0	0	3.0%	0.0%	0.0%	0.0%	0.0%	0.0%	0.0%	0.0%
tr|F1RP50|F1RP50	0	1	0	0	0	0	0	0	0	2	0	0	0	0	0	0	0.0%	0.8%	0.0%	0.0%	0.0%	0.0%	0.0%	0.0%
tr|F1RS14|F1RS14	2	0	0	0	0	0	0	0	2	0	0	0	0	0	0	0	13.7%	0.0%	0.0%	0.0%	0.0%	0.0%	0.0%	0.0%
tr|K7GRE0|K7GRE0	2	0	0	0	0	0	0	0	2	0	0	0	0	0	0	0	27.0%	0.0%	0.0%	0.0%	0.0%	0.0%	0.0%	0.0%
tr|I3LR33|I3LR33	0	1	0	0	0	0	0	0	0	2	0	0	0	0	0	0	0.0%	11.6%	0.0%	0.0%	0.0%	0.0%	0.0%	0.0%
tr|F6Q7M8|F6Q7M8	2	0	0	0	0	0	0	0	2	0	0	0	0	0	0	0	12.1%	0.0%	0.0%	0.0%	0.0%	0.0%	0.0%	0.0%

	Peptide Count								Spectral Count								Sequence Coverage							
	Whole Cell	Whole Cell	Whole Cell	Cilia Fraction	Cilia Fraction	Cilia Fraction	Cilia Fraction	Cilia Fraction	Whole Cell	Whole Cell	Whole Cell	Cilia Fraction	Cilia Fraction	Cilia Fraction	Cilia Fraction	Cilia Fraction	Whole Cell	Whole Cell	Whole Cell	Cilia Fraction	Cilia Fraction	Cilia Fraction	Cilia Fraction	Cilia Fraction
Sample#	1	2	6	4	3	5	7	8	1	2	6	4	3	5	7	8	1	2	6	4	3	5	7	8
**Accession**																								
tr|F1SHM3|F1SHM3	1	0	0	0	0	0	0	0	2	0	0	0	0	0	0	0	1.1%	0.0%	0.0%	0.0%	0.0%	0.0%	0.0%	0.0%
tr|I3LQ32|I3LQ32	1	0	0	0	0	0	0	0	2	0	0	0	0	0	0	0	20.4%	0.0%	0.0%	0.0%	0.0%	0.0%	0.0%	0.0%
Q66RM2	1	1	0	0	0	0	0	0	1	1	0	0	0	0	0	0	3.8%	3.8%	0.0%	0.0%	0.0%	0.0%	0.0%	0.0%
tr|F1SBH1|F1SBH1	1	1	0	0	0	0	0	0	1	1	0	0	0	0	0	0	12.9%	10.5%	0.0%	0.0%	0.0%	0.0%	0.0%	0.0%
tr|F1RKU5|F1RKU5	2	0	0	0	0	0	0	0	2	0	0	0	0	0	0	0	5.4%	0.0%	0.0%	0.0%	0.0%	0.0%	0.0%	0.0%
tr|F1S1L1|F1S1L1	2	0	0	0	0	0	0	0	2	0	0	0	0	0	0	0	19.6%	0.0%	0.0%	0.0%	0.0%	0.0%	0.0%	0.0%
tr|F1RGS0|F1RGS0	0	1	0	0	0	0	0	0	0	2	0	0	0	0	0	0	0.0%	0.7%	0.0%	0.0%	0.0%	0.0%	0.0%	0.0%
tr|F1RM18|F1RM18	0	1	0	0	0	0	0	0	0	2	0	0	0	0	0	0	0.0%	3.0%	0.0%	0.0%	0.0%	0.0%	0.0%	0.0%
tr|K7GSP2|K7GSP2	0	1	0	0	0	0	0	0	0	2	0	0	0	0	0	0	0.0%	7.1%	0.0%	0.0%	0.0%	0.0%	0.0%	0.0%
tr|F1SIF5|F1SIF5	1	1	0	0	0	0	0	0	1	1	0	0	0	0	0	0	6.5%	6.5%	0.0%	0.0%	0.0%	0.0%	0.0%	0.0%
Reverse_tr|I3LHP2|I3LHP2	0	1	0	0	0	0	0	0	0	2	0	0	0	0	0	0	0.0%	3.9%	0.0%	0.0%	0.0%	0.0%	0.0%	0.0%
tr|F1SGR7|F1SGR7	0	1	0	0	0	0	0	0	0	2	0	0	0	0	0	0	0.0%	1.8%	0.0%	0.0%	0.0%	0.0%	0.0%	0.0%
tr|F1RZD0|F1RZD0	0	1	0	0	0	0	0	0	0	2	0	0	0	0	0	0	0.0%	5.0%	0.0%	0.0%	0.0%	0.0%	0.0%	0.0%
tr|F1RQE0|F1RQE0	2	0	0	0	0	0	0	0	2	0	0	0	0	0	0	0	4.2%	0.0%	0.0%	0.0%	0.0%	0.0%	0.0%	0.0%
tr|F1RZ35|F1RZ35	1	0	0	0	0	0	0	0	2	0	0	0	0	0	0	0	7.3%	0.0%	0.0%	0.0%	0.0%	0.0%	0.0%	0.0%
tr|K7GRK9|K7GRK9	2	0	0	0	0	0	0	0	2	0	0	0	0	0	0	0	21.1%	0.0%	0.0%	0.0%	0.0%	0.0%	0.0%	0.0%
tr|F1RF28|F1RF28	0	1	0	0	0	0	0	0	0	2	0	0	0	0	0	0	0.0%	0.7%	0.0%	0.0%	0.0%	0.0%	0.0%	0.0%
tr|K7GLQ7|K7GLQ7	0	1	0	0	0	0	0	0	0	2	0	0	0	0	0	0	0.0%	5.3%	0.0%	0.0%	0.0%	0.0%	0.0%	0.0%
tr|I3LHP0|I3LHP0	0	1	0	0	0	0	0	0	0	2	0	0	0	0	0	0	0.0%	4.8%	0.0%	0.0%	0.0%	0.0%	0.0%	0.0%
tr|F1SDD7|F1SDD7	1	1	0	0	0	0	0	0	1	1	0	0	0	0	0	0	3.9%	2.8%	0.0%	0.0%	0.0%	0.0%	0.0%	0.0%
tr|F1RNB1|F1RNB1	1	0	0	0	0	0	0	0	2	0	0	0	0	0	0	0	6.6%	0.0%	0.0%	0.0%	0.0%	0.0%	0.0%	0.0%
tr|F1SMC1|F1SMC1	0	1	0	0	0	0	0	0	0	2	0	0	0	0	0	0	0.0%	4.2%	0.0%	0.0%	0.0%	0.0%	0.0%	0.0%
A1Y2K1	2	0	0	0	0	0	0	0	2	0	0	0	0	0	0	0	2.8%	0.0%	0.0%	0.0%	0.0%	0.0%	0.0%	0.0%
tr|I3LUH6|I3LUH6	0	1	0	0	0	0	0	0	0	2	0	0	0	0	0	0	0.0%	9.6%	0.0%	0.0%	0.0%	0.0%	0.0%	0.0%
tr|I3LF14|I3LF14	1	0	0	0	0	0	0	0	2	0	0	0	0	0	0	0	30.5%	0.0%	0.0%	0.0%	0.0%	0.0%	0.0%	0.0%
tr|I3LKT2|I3LKT2	1	1	0	0	0	0	0	0	1	1	0	0	0	0	0	0	4.1%	4.1%	0.0%	0.0%	0.0%	0.0%	0.0%	0.0%
tr|F1RS71|F1RS71	2	0	0	0	0	0	0	0	2	0	0	0	0	0	0	0	5.5%	0.0%	0.0%	0.0%	0.0%	0.0%	0.0%	0.0%
tr|I3LRE5|I3LRE5	2	0	0	0	0	0	0	0	2	0	0	0	0	0	0	0	1.9%	0.0%	0.0%	0.0%	0.0%	0.0%	0.0%	0.0%
tr|F1RWX5|F1RWX5	0	1	0	0	0	0	0	0	0	2	0	0	0	0	0	0	0.0%	2.3%	0.0%	0.0%	0.0%	0.0%	0.0%	0.0%
tr|F1SA56|F1SA56	1	0	0	0	0	0	0	0	2	0	0	0	0	0	0	0	8.9%	0.0%	0.0%	0.0%	0.0%	0.0%	0.0%	0.0%
tr|F1RQV4|F1RQV4	2	0	0	0	0	0	0	0	2	0	0	0	0	0	0	0	5.2%	0.0%	0.0%	0.0%	0.0%	0.0%	0.0%	0.0%
tr|Q1EG61|Q1EG61	0	1	0	0	0	0	0	0	0	2	0	0	0	0	0	0	0.0%	5.7%	0.0%	0.0%	0.0%	0.0%	0.0%	0.0%
tr|F1SAJ9|F1SAJ9	2	0	0	0	0	0	0	0	2	0	0	0	0	0	0	0	10.5%	0.0%	0.0%	0.0%	0.0%	0.0%	0.0%	0.0%
tr|F1SAS1|F1SAS1	1	0	0	0	0	0	0	0	2	0	0	0	0	0	0	0	10.1%	0.0%	0.0%	0.0%	0.0%	0.0%	0.0%	0.0%
tr|F1S7P3|F1S7P3	1	0	0	0	0	0	0	0	2	0	0	0	0	0	0	0	2.0%	0.0%	0.0%	0.0%	0.0%	0.0%	0.0%	0.0%
tr|F1RMF9|F1RMF9	2	0	0	0	0	0	0	0	2	0	0	0	0	0	0	0	5.1%	0.0%	0.0%	0.0%	0.0%	0.0%	0.0%	0.0%
tr|I3LVM1|I3LVM1	0	1	0	0	0	0	0	0	0	2	0	0	0	0	0	0	0.0%	6.2%	0.0%	0.0%	0.0%	0.0%	0.0%	0.0%
Q8SQC1	0	1	0	0	0	0	0	0	0	2	0	0	0	0	0	0	0.0%	2.6%	0.0%	0.0%	0.0%	0.0%	0.0%	0.0%
tr|I3LD21|I3LD21	2	0	0	0	0	0	0	0	2	0	0	0	0	0	0	0	2.3%	0.0%	0.0%	0.0%	0.0%	0.0%	0.0%	0.0%
tr|A5GFY5|A5GFY5	1	0	0	0	0	0	0	0	2	0	0	0	0	0	0	0	8.9%	0.0%	0.0%	0.0%	0.0%	0.0%	0.0%	0.0%
tr|I3LHA8|I3LHA8	1	0	0	0	0	0	0	0	2	0	0	0	0	0	0	0	29.8%	0.0%	0.0%	0.0%	0.0%	0.0%	0.0%	0.0%
tr|F1S9S2|F1S9S2	2	0	0	0	0	0	0	0	2	0	0	0	0	0	0	0	1.8%	0.0%	0.0%	0.0%	0.0%	0.0%	0.0%	0.0%
tr|K7GKG2|K7GKG2	2	0	0	0	0	0	0	0	2	0	0	0	0	0	0	0	6.4%	0.0%	0.0%	0.0%	0.0%	0.0%	0.0%	0.0%
tr|I3LKD8|I3LKD8	1	0	0	0	0	0	0	0	2	0	0	0	0	0	0	0	17.1%	0.0%	0.0%	0.0%	0.0%	0.0%	0.0%	0.0%
Q9TUZ1	1	1	0	0	0	0	0	0	1	1	0	0	0	0	0	0	2.3%	1.3%	0.0%	0.0%	0.0%	0.0%	0.0%	0.0%
tr|F1S433|F1S433	2	0	0	0	0	0	0	0	2	0	0	0	0	0	0	0	5.8%	0.0%	0.0%	0.0%	0.0%	0.0%	0.0%	0.0%
tr|F1SFY7|F1SFY7	1	1	0	0	0	0	0	0	1	1	0	0	0	0	0	0	8.9%	8.9%	0.0%	0.0%	0.0%	0.0%	0.0%	0.0%
tr|F1SQL1|F1SQL1	0	2	0	0	0	0	0	0	0	2	0	0	0	0	0	0	0.0%	4.6%	0.0%	0.0%	0.0%	0.0%	0.0%	0.0%
tr|F1S5A7|F1S5A7	1	0	0	0	0	0	0	0	2	0	0	0	0	0	0	0	8.7%	0.0%	0.0%	0.0%	0.0%	0.0%	0.0%	0.0%
tr|I3LIT6|I3LIT6	1	1	0	0	0	0	0	0	1	1	0	0	0	0	0	0	4.4%	4.7%	0.0%	0.0%	0.0%	0.0%	0.0%	0.0%
tr|K7GM51|K7GM51	0	1	0	0	0	0	0	0	0	2	0	0	0	0	0	0	0.0%	5.2%	0.0%	0.0%	0.0%	0.0%	0.0%	0.0%
tr|K7GN04|K7GN04	2	0	0	0	0	0	0	0	2	0	0	0	0	0	0	0	22.3%	0.0%	0.0%	0.0%	0.0%	0.0%	0.0%	0.0%
tr|I3LJU1|I3LJU1	1	0	0	0	0	0	0	0	2	0	0	0	0	0	0	0	4.9%	0.0%	0.0%	0.0%	0.0%	0.0%	0.0%	0.0%
tr|F1SIF4|F1SIF4	0	1	0	0	0	0	0	0	0	2	0	0	0	0	0	0	0.0%	2.1%	0.0%	0.0%	0.0%	0.0%	0.0%	0.0%
tr|F1SNG4|F1SNG4	0	1	0	0	0	0	0	0	0	2	0	0	0	0	0	0	0.0%	3.1%	0.0%	0.0%	0.0%	0.0%	0.0%	0.0%
tr|I3LTK2|I3LTK2	2	0	0	0	0	0	0	0	2	0	0	0	0	0	0	0	10.9%	0.0%	0.0%	0.0%	0.0%	0.0%	0.0%	0.0%
tr|I3LPI1|I3LPI1	0	1	0	0	0	0	0	0	0	2	0	0	0	0	0	0	0.0%	2.6%	0.0%	0.0%	0.0%	0.0%	0.0%	0.0%
tr|F1S9C2|F1S9C2	2	0	0	0	0	0	0	0	2	0	0	0	0	0	0	0	7.3%	0.0%	0.0%	0.0%	0.0%	0.0%	0.0%	0.0%
Reverse_tr|F1S322|F1S322	1	1	0	0	0	0	0	0	1	1	0	0	0	0	0	0	4.6%	4.6%	0.0%	0.0%	0.0%	0.0%	0.0%	0.0%
P81140	0	1	0	0	0	0	0	0	0	2	0	0	0	0	0	0	0.0%	4.2%	0.0%	0.0%	0.0%	0.0%	0.0%	0.0%
tr|F1S8A3|F1S8A3	2	0	0	0	0	0	0	0	2	0	0	0	0	0	0	0	5.2%	0.0%	0.0%	0.0%	0.0%	0.0%	0.0%	0.0%
tr|I3LMC8|I3LMC8	2	0	0	0	0	0	0	0	2	0	0	0	0	0	0	0	13.8%	0.0%	0.0%	0.0%	0.0%	0.0%	0.0%	0.0%
tr|F1SN06|F1SN06	1	0	0	0	0	0	0	0	2	0	0	0	0	0	0	0	4.9%	0.0%	0.0%	0.0%	0.0%	0.0%	0.0%	0.0%
tr|F1RVH5|F1RVH5	1	1	0	0	0	0	0	0	1	1	0	0	0	0	0	0	6.2%	15.6%	0.0%	0.0%	0.0%	0.0%	0.0%	0.0%
tr|F1S3B0|F1S3B0	2	0	0	0	0	0	0	0	2	0	0	0	0	0	0	0	6.0%	0.0%	0.0%	0.0%	0.0%	0.0%	0.0%	0.0%
tr|I3LQD0|I3LQD0	2	0	0	0	0	0	0	0	2	0	0	0	0	0	0	0	24.2%	0.0%	0.0%	0.0%	0.0%	0.0%	0.0%	0.0%
tr|F1SBW4|F1SBW4	1	0	0	0	0	0	0	0	2	0	0	0	0	0	0	0	5.1%	0.0%	0.0%	0.0%	0.0%	0.0%	0.0%	0.0%
tr|K7GLP9|K7GLP9	2	0	0	0	0	0	0	0	2	0	0	0	0	0	0	0	19.5%	0.0%	0.0%	0.0%	0.0%	0.0%	0.0%	0.0%
tr|F1SEX3|F1SEX3	0	1	0	0	0	0	0	0	0	2	0	0	0	0	0	0	0.0%	3.9%	0.0%	0.0%	0.0%	0.0%	0.0%	0.0%
tr|I3LBV8|I3LBV8	2	0	0	0	0	0	0	0	2	0	0	0	0	0	0	0	1.8%	0.0%	0.0%	0.0%	0.0%	0.0%	0.0%	0.0%
tr|F1RQH8|F1RQH8	1	1	0	0	0	0	0	0	1	1	0	0	0	0	0	0	0.9%	1.0%	0.0%	0.0%	0.0%	0.0%	0.0%	0.0%
tr|F1SJS0|F1SJS0	1	1	0	0	0	0	0	0	1	1	0	0	0	0	0	0	5.2%	3.6%	0.0%	0.0%	0.0%	0.0%	0.0%	0.0%
B2LYG4	1	0	0	0	0	0	0	0	2	0	0	0	0	0	0	0	6.5%	0.0%	0.0%	0.0%	0.0%	0.0%	0.0%	0.0%
tr|F1S6E8|F1S6E8	2	0	0	0	0	0	0	0	2	0	0	0	0	0	0	0	4.4%	0.0%	0.0%	0.0%	0.0%	0.0%	0.0%	0.0%
tr|F1RH91|F1RH91	2	0	0	0	0	0	0	0	2	0	0	0	0	0	0	0	3.2%	0.0%	0.0%	0.0%	0.0%	0.0%	0.0%	0.0%
tr|F1RQ08|F1RQ08	2	0	0	0	0	0	0	0	2	0	0	0	0	0	0	0	2.9%	0.0%	0.0%	0.0%	0.0%	0.0%	0.0%	0.0%
tr|F1S3Z9|F1S3Z9	2	0	0	0	0	0	0	0	2	0	0	0	0	0	0	0	1.9%	0.0%	0.0%	0.0%	0.0%	0.0%	0.0%	0.0%
Reverse_tr|F1RK35|F1RK35	1	0	0	0	0	0	0	0	2	0	0	0	0	0	0	0	5.5%	0.0%	0.0%	0.0%	0.0%	0.0%	0.0%	0.0%
tr|F1RQP1|F1RQP1	2	0	0	0	0	0	0	0	2	0	0	0	0	0	0	0	21.6%	0.0%	0.0%	0.0%	0.0%	0.0%	0.0%	0.0%
tr|I3L5I4|I3L5I4	2	0	0	0	0	0	0	0	2	0	0	0	0	0	0	0	4.1%	0.0%	0.0%	0.0%	0.0%	0.0%	0.0%	0.0%
tr|A9QT41|A9QT41	1	1	0	0	0	0	0	0	1	1	0	0	0	0	0	0	4.8%	4.8%	0.0%	0.0%	0.0%	0.0%	0.0%	0.0%

	Peptide Count								Spectral Count								Sequence Coverage							
	Whole Cell	Whole Cell	Whole Cell	Cilia Fraction	Cilia Fraction	Cilia Fraction	Cilia Fraction	Cilia Fraction	Whole Cell	Whole Cell	Whole Cell	Cilia Fraction	Cilia Fraction	Cilia Fraction	Cilia Fraction	Cilia Fraction	Whole Cell	Whole Cell	Whole Cell	Cilia Fraction	Cilia Fraction	Cilia Fraction	Cilia Fraction	Cilia Fraction
Sample#	1	2	6	4	3	5	7	8	1	2	6	4	3	5	7	8	1	2	6	4	3	5	7	8
**Accession**																								
tr|F1RSR4|F1RSR4	2	0	0	0	0	0	0	0	2	0	0	0	0	0	0	0	9.6%	0.0%	0.0%	0.0%	0.0%	0.0%	0.0%	0.0%
tr|F1RNU7|F1RNU7	0	1	0	0	0	0	0	0	0	2	0	0	0	0	0	0	0.0%	4.1%	0.0%	0.0%	0.0%	0.0%	0.0%	0.0%
tr|F1STL2|F1STL2	1	1	0	0	0	0	0	0	1	1	0	0	0	0	0	0	3.1%	1.9%	0.0%	0.0%	0.0%	0.0%	0.0%	0.0%
tr|I3LD31|I3LD31	2	0	0	0	0	0	0	0	2	0	0	0	0	0	0	0	9.5%	0.0%	0.0%	0.0%	0.0%	0.0%	0.0%	0.0%
tr|F1SSL1|F1SSL1	1	0	0	0	0	0	0	0	2	0	0	0	0	0	0	0	8.7%	0.0%	0.0%	0.0%	0.0%	0.0%	0.0%	0.0%
tr|I3LR45|I3LR45	1	0	0	0	0	0	0	0	2	0	0	0	0	0	0	0	4.5%	0.0%	0.0%	0.0%	0.0%	0.0%	0.0%	0.0%
tr|F1RS78|F1RS78	1	0	0	0	0	0	0	0	2	0	0	0	0	0	0	0	5.8%	0.0%	0.0%	0.0%	0.0%	0.0%	0.0%	0.0%
tr|I3LIH8|I3LIH8	2	0	0	0	0	0	0	0	2	0	0	0	0	0	0	0	2.0%	0.0%	0.0%	0.0%	0.0%	0.0%	0.0%	0.0%
tr|I3LHX6|I3LHX6	1	0	0	0	0	0	0	0	2	0	0	0	0	0	0	0	5.8%	0.0%	0.0%	0.0%	0.0%	0.0%	0.0%	0.0%
tr|I3LQG9|I3LQG9	2	0	0	0	0	0	0	0	2	0	0	0	0	0	0	0	18.4%	0.0%	0.0%	0.0%	0.0%	0.0%	0.0%	0.0%
tr|F1SHX8|F1SHX8	0	1	0	0	0	0	0	0	0	2	0	0	0	0	0	0	0.0%	5.5%	0.0%	0.0%	0.0%	0.0%	0.0%	0.0%
tr|K7GLI2|K7GLI2	1	0	0	0	0	0	0	0	2	0	0	0	0	0	0	0	27.6%	0.0%	0.0%	0.0%	0.0%	0.0%	0.0%	0.0%
tr|F1RZR5|F1RZR5	0	1	0	0	0	0	0	0	0	2	0	0	0	0	0	0	0.0%	0.7%	0.0%	0.0%	0.0%	0.0%	0.0%	0.0%
tr|I3LDY1|I3LDY1	1	1	0	0	0	0	0	0	1	1	0	0	0	0	0	0	1.5%	1.5%	0.0%	0.0%	0.0%	0.0%	0.0%	0.0%
tr|K7GQ10|K7GQ10	0	1	0	0	0	0	0	0	0	2	0	0	0	0	0	0	0.0%	6.4%	0.0%	0.0%	0.0%	0.0%	0.0%	0.0%
tr|F1RG41|F1RG41	1	0	0	0	0	0	0	0	2	0	0	0	0	0	0	0	3.5%	0.0%	0.0%	0.0%	0.0%	0.0%	0.0%	0.0%
tr|F1S698|F1S698	2	0	0	0	0	0	0	0	2	0	0	0	0	0	0	0	10.4%	0.0%	0.0%	0.0%	0.0%	0.0%	0.0%	0.0%
tr|F1SK83|F1SK83	2	0	0	0	0	0	0	0	2	0	0	0	0	0	0	0	7.0%	0.0%	0.0%	0.0%	0.0%	0.0%	0.0%	0.0%
tr|I3L786|I3L786	0	1	0	0	0	0	0	0	0	2	0	0	0	0	0	0	0.0%	8.9%	0.0%	0.0%	0.0%	0.0%	0.0%	0.0%
tr|F1SJ00|F1SJ00	2	0	0	0	0	0	0	0	2	0	0	0	0	0	0	0	2.6%	0.0%	0.0%	0.0%	0.0%	0.0%	0.0%	0.0%
tr|F1RK31|F1RK31	1	1	0	0	0	0	0	0	1	1	0	0	0	0	0	0	1.0%	0.8%	0.0%	0.0%	0.0%	0.0%	0.0%	0.0%
tr|F1SAB5|F1SAB5	2	0	0	0	0	0	0	0	2	0	0	0	0	0	0	0	8.0%	0.0%	0.0%	0.0%	0.0%	0.0%	0.0%	0.0%
tr|F2Z5H0|F2Z5H0	2	0	0	0	0	0	0	0	2	0	0	0	0	0	0	0	9.0%	0.0%	0.0%	0.0%	0.0%	0.0%	0.0%	0.0%
tr|F1SAY2|F1SAY2	0	2	0	0	0	0	0	0	0	2	0	0	0	0	0	0	0.0%	2.2%	0.0%	0.0%	0.0%	0.0%	0.0%	0.0%
tr|F2Z559|F2Z559	0	1	0	0	0	0	0	0	0	2	0	0	0	0	0	0	0.0%	14.5%	0.0%	0.0%	0.0%	0.0%	0.0%	0.0%
tr|I3LRA4|I3LRA4	1	0	0	0	0	0	0	0	2	0	0	0	0	0	0	0	9.6%	0.0%	0.0%	0.0%	0.0%	0.0%	0.0%	0.0%
tr|F1RXS1|F1RXS1	0	1	0	0	0	0	0	0	0	2	0	0	0	0	0	0	0.0%	0.7%	0.0%	0.0%	0.0%	0.0%	0.0%	0.0%
tr|F2Z513|F2Z513	0	2	0	0	0	0	0	0	0	2	0	0	0	0	0	0	0.0%	14.5%	0.0%	0.0%	0.0%	0.0%	0.0%	0.0%
tr|I3LDG1|I3LDG1	0	1	0	0	0	0	0	0	0	2	0	0	0	0	0	0	0.0%	2.5%	0.0%	0.0%	0.0%	0.0%	0.0%	0.0%
tr|I3LHP1|I3LHP1	1	0	0	0	0	0	0	0	2	0	0	0	0	0	0	0	13.1%	0.0%	0.0%	0.0%	0.0%	0.0%	0.0%	0.0%
tr|F1SE16|F1SE16	1	0	0	0	0	0	0	0	2	0	0	0	0	0	0	0	5.9%	0.0%	0.0%	0.0%	0.0%	0.0%	0.0%	0.0%
tr|F1S8S7|F1S8S7	1	1	0	0	0	0	0	0	1	1	0	0	0	0	0	0	3.0%	1.5%	0.0%	0.0%	0.0%	0.0%	0.0%	0.0%
tr|F1RTZ8|F1RTZ8	2	0	0	0	0	0	0	0	2	0	0	0	0	0	0	0	12.7%	0.0%	0.0%	0.0%	0.0%	0.0%	0.0%	0.0%
tr|K7GRY3|K7GRY3	0	1	0	0	0	0	0	0	0	2	0	0	0	0	0	0	0.0%	1.2%	0.0%	0.0%	0.0%	0.0%	0.0%	0.0%
tr|F1S8J5|F1S8J5	0	1	0	0	0	0	0	0	0	2	0	0	0	0	0	0	0.0%	0.3%	0.0%	0.0%	0.0%	0.0%	0.0%	0.0%
tr|I3LFB6|I3LFB6	1	0	0	0	0	0	0	0	2	0	0	0	0	0	0	0	2.2%	0.0%	0.0%	0.0%	0.0%	0.0%	0.0%	0.0%
tr|F1SSX0|F1SSX0	2	0	0	0	0	0	0	0	2	0	0	0	0	0	0	0	18.8%	0.0%	0.0%	0.0%	0.0%	0.0%	0.0%	0.0%
tr|F1SJH3|F1SJH3	2	0	0	0	0	0	0	0	2	0	0	0	0	0	0	0	29.8%	0.0%	0.0%	0.0%	0.0%	0.0%	0.0%	0.0%
tr|I3LPR6|I3LPR6	2	0	0	0	0	0	0	0	2	0	0	0	0	0	0	0	2.5%	0.0%	0.0%	0.0%	0.0%	0.0%	0.0%	0.0%
tr|I3L9F2|I3L9F2	2	0	0	0	0	0	0	0	2	0	0	0	0	0	0	0	2.7%	0.0%	0.0%	0.0%	0.0%	0.0%	0.0%	0.0%
tr|F1S4F7|F1S4F7	0	1	0	0	0	0	0	0	0	2	0	0	0	0	0	0	0.0%	2.2%	0.0%	0.0%	0.0%	0.0%	0.0%	0.0%
tr|F1SSZ8|F1SSZ8	1	1	0	0	0	0	0	0	1	1	0	0	0	0	0	0	2.1%	1.1%	0.0%	0.0%	0.0%	0.0%	0.0%	0.0%
tr|I3LDG5|I3LDG5	2	0	0	0	0	0	0	0	2	0	0	0	0	0	0	0	3.7%	0.0%	0.0%	0.0%	0.0%	0.0%	0.0%	0.0%
tr|F1RF27|F1RF27	0	1	0	0	0	0	0	0	0	2	0	0	0	0	0	0	0.0%	1.8%	0.0%	0.0%	0.0%	0.0%	0.0%	0.0%
tr|F1SII1|F1SII1	1	1	0	0	0	0	0	0	1	1	0	0	0	0	0	0	7.7%	10.5%	0.0%	0.0%	0.0%	0.0%	0.0%	0.0%
tr|F1RZN7|F1RZN7	1	0	0	0	0	0	0	0	2	0	0	0	0	0	0	0	4.5%	0.0%	0.0%	0.0%	0.0%	0.0%	0.0%	0.0%
tr|F1RPS7|F1RPS7	1	0	0	0	0	0	0	0	2	0	0	0	0	0	0	0	5.0%	0.0%	0.0%	0.0%	0.0%	0.0%	0.0%	0.0%
tr|F1S2V9|F1S2V9	1	0	0	0	0	0	0	0	2	0	0	0	0	0	0	0	17.2%	0.0%	0.0%	0.0%	0.0%	0.0%	0.0%	0.0%
tr|I3L5Q3|I3L5Q3	2	0	0	0	0	0	0	0	2	0	0	0	0	0	0	0	12.3%	0.0%	0.0%	0.0%	0.0%	0.0%	0.0%	0.0%
tr|F1SCA0|F1SCA0	2	0	0	0	0	0	0	0	2	0	0	0	0	0	0	0	2.3%	0.0%	0.0%	0.0%	0.0%	0.0%	0.0%	0.0%
tr|I3L592|I3L592	0	2	0	0	0	0	0	0	0	2	0	0	0	0	0	0	0.0%	4.6%	0.0%	0.0%	0.0%	0.0%	0.0%	0.0%
tr|F1SLS5|F1SLS5	1	0	0	0	0	0	0	0	2	0	0	0	0	0	0	0	4.2%	0.0%	0.0%	0.0%	0.0%	0.0%	0.0%	0.0%
tr|F1SDP1|F1SDP1	0	1	0	0	0	0	0	0	0	2	0	0	0	0	0	0	0.0%	5.1%	0.0%	0.0%	0.0%	0.0%	0.0%	0.0%
tr|I3LMR6|I3LMR6	1	0	0	0	0	0	0	0	2	0	0	0	0	0	0	0	34.6%	0.0%	0.0%	0.0%	0.0%	0.0%	0.0%	0.0%
tr|F1SCU6|F1SCU6	2	0	0	0	0	0	0	0	2	0	0	0	0	0	0	0	2.9%	0.0%	0.0%	0.0%	0.0%	0.0%	0.0%	0.0%
tr|F1S4M8|F1S4M8	0	1	0	0	0	0	0	0	0	2	0	0	0	0	0	0	0.0%	2.5%	0.0%	0.0%	0.0%	0.0%	0.0%	0.0%
tr|F1SEA8|F1SEA8	1	0	0	0	0	0	0	0	2	0	0	0	0	0	0	0	3.5%	0.0%	0.0%	0.0%	0.0%	0.0%	0.0%	0.0%
tr|F2Z5T0|F2Z5T0	2	0	0	0	0	0	0	0	2	0	0	0	0	0	0	0	9.4%	0.0%	0.0%	0.0%	0.0%	0.0%	0.0%	0.0%
tr|I3L991|I3L991	1	1	0	0	0	0	0	0	1	1	0	0	0	0	0	0	11.8%	7.5%	0.0%	0.0%	0.0%	0.0%	0.0%	0.0%
tr|I3LCM3|I3LCM3	0	1	0	0	0	0	0	0	0	2	0	0	0	0	0	0	0.0%	5.9%	0.0%	0.0%	0.0%	0.0%	0.0%	0.0%
tr|I3LCG8|I3LCG8	0	1	0	0	0	0	0	0	0	2	0	0	0	0	0	0	0.0%	9.1%	0.0%	0.0%	0.0%	0.0%	0.0%	0.0%
tr|I3L7V5|I3L7V5	0	2	0	0	0	0	0	0	0	2	0	0	0	0	0	0	0.0%	2.3%	0.0%	0.0%	0.0%	0.0%	0.0%	0.0%
tr|F1RRM7|F1RRM7	0	1	0	0	0	0	0	0	0	2	0	0	0	0	0	0	0.0%	5.5%	0.0%	0.0%	0.0%	0.0%	0.0%	0.0%
tr|F1SSP1|F1SSP1	0	1	0	0	0	0	0	0	0	2	0	0	0	0	0	0	0.0%	2.0%	0.0%	0.0%	0.0%	0.0%	0.0%	0.0%
tr|K7GQV6|K7GQV6	1	1	0	0	0	0	0	0	1	1	0	0	0	0	0	0	5.4%	5.4%	0.0%	0.0%	0.0%	0.0%	0.0%	0.0%
tr|F1S8X4|F1S8X4	0	2	0	0	0	0	0	0	0	2	0	0	0	0	0	0	0.0%	6.3%	0.0%	0.0%	0.0%	0.0%	0.0%	0.0%
tr|I3LIJ0|I3LIJ0	0	1	0	0	0	0	0	0	0	2	0	0	0	0	0	0	0.0%	4.1%	0.0%	0.0%	0.0%	0.0%	0.0%	0.0%
tr|F1SEB1|F1SEB1	1	0	0	0	0	0	0	0	2	0	0	0	0	0	0	0	4.7%	0.0%	0.0%	0.0%	0.0%	0.0%	0.0%	0.0%
tr|I3L6Y7|I3L6Y7	1	1	0	0	0	0	0	0	1	1	0	0	0	0	0	0	0.9%	1.0%	0.0%	0.0%	0.0%	0.0%	0.0%	0.0%
tr|F1RVK3|F1RVK3	2	0	0	0	0	0	0	0	2	0	0	0	0	0	0	0	7.8%	0.0%	0.0%	0.0%	0.0%	0.0%	0.0%	0.0%
tr|F1SV46|F1SV46	2	0	0	0	0	0	0	0	2	0	0	0	0	0	0	0	3.3%	0.0%	0.0%	0.0%	0.0%	0.0%	0.0%	0.0%
tr|K7GPU5|K7GPU5	2	0	0	0	0	0	0	0	2	0	0	0	0	0	0	0	3.9%	0.0%	0.0%	0.0%	0.0%	0.0%	0.0%	0.0%
tr|I3LEP6|I3LEP6	0	1	0	0	0	0	0	0	0	2	0	0	0	0	0	0	0.0%	4.2%	0.0%	0.0%	0.0%	0.0%	0.0%	0.0%
tr|I3LE17|I3LE17	2	0	0	0	0	0	0	0	2	0	0	0	0	0	0	0	24.6%	0.0%	0.0%	0.0%	0.0%	0.0%	0.0%	0.0%
tr|I3LNZ3|I3LNZ3	1	1	0	0	0	0	0	0	1	1	0	0	0	0	0	0	3.5%	2.5%	0.0%	0.0%	0.0%	0.0%	0.0%	0.0%
tr|F1STD0|F1STD0	2	0	0	0	0	0	0	0	2	0	0	0	0	0	0	0	23.9%	0.0%	0.0%	0.0%	0.0%	0.0%	0.0%	0.0%
Q29121	1	0	0	0	0	0	0	0	2	0	0	0	0	0	0	0	3.8%	0.0%	0.0%	0.0%	0.0%	0.0%	0.0%	0.0%
tr|F1RX57|F1RX57	2	0	0	0	0	0	0	0	2	0	0	0	0	0	0	0	1.6%	0.0%	0.0%	0.0%	0.0%	0.0%	0.0%	0.0%
tr|I3L774|I3L774	0	1	0	0	0	0	0	0	0	2	0	0	0	0	0	0	0.0%	1.5%	0.0%	0.0%	0.0%	0.0%	0.0%	0.0%
tr|I3LLD8|I3LLD8	0	1	0	0	0	0	0	0	0	2	0	0	0	0	0	0	0.0%	2.3%	0.0%	0.0%	0.0%	0.0%	0.0%	0.0%
tr|I3LT67|I3LT67	1	0	0	0	0	0	0	0	2	0	0	0	0	0	0	0	5.1%	0.0%	0.0%	0.0%	0.0%	0.0%	0.0%	0.0%

	Peptide Count								Spectral Count								Sequence Coverage							
	Whole Cell	Whole Cell	Whole Cell	Cilia Fraction	Cilia Fraction	Cilia Fraction	Cilia Fraction	Cilia Fraction	Whole Cell	Whole Cell	Whole Cell	Cilia Fraction	Cilia Fraction	Cilia Fraction	Cilia Fraction	Cilia Fraction	Whole Cell	Whole Cell	Whole Cell	Cilia Fraction	Cilia Fraction	Cilia Fraction	Cilia Fraction	Cilia Fraction
Sample#	1	2	6	4	3	5	7	8	1	2	6	4	3	5	7	8	1	2	6	4	3	5	7	8
**Accession**																								
tr|F1RR16|F1RR16	2	0	0	0	0	0	0	0	2	0	0	0	0	0	0	0	1.7%	0.0%	0.0%	0.0%	0.0%	0.0%	0.0%	0.0%
tr|F1RYR2|F1RYR2	2	0	0	0	0	0	0	0	2	0	0	0	0	0	0	0	11.0%	0.0%	0.0%	0.0%	0.0%	0.0%	0.0%	0.0%
tr|F1RJC9|F1RJC9	1	0	0	0	0	0	0	0	2	0	0	0	0	0	0	0	7.7%	0.0%	0.0%	0.0%	0.0%	0.0%	0.0%	0.0%
tr|F1SB54|F1SB54	1	0	0	0	0	0	0	0	2	0	0	0	0	0	0	0	5.3%	0.0%	0.0%	0.0%	0.0%	0.0%	0.0%	0.0%
tr|F1S776|F1S776	0	1	0	0	0	0	0	0	0	2	0	0	0	0	0	0	0.0%	4.1%	0.0%	0.0%	0.0%	0.0%	0.0%	0.0%
tr|J9JIM1|J9JIM1	0	1	0	0	0	0	0	0	0	2	0	0	0	0	0	0	0.0%	2.8%	0.0%	0.0%	0.0%	0.0%	0.0%	0.0%
tr|K7GL51|K7GL51	2	0	0	0	0	0	0	0	2	0	0	0	0	0	0	0	6.0%	0.0%	0.0%	0.0%	0.0%	0.0%	0.0%	0.0%
tr|I3L8K1|I3L8K1	1	1	0	0	0	0	0	0	1	1	0	0	0	0	0	0	3.0%	1.4%	0.0%	0.0%	0.0%	0.0%	0.0%	0.0%
tr|I3LR79|I3LR79	2	0	0	0	0	0	0	0	2	0	0	0	0	0	0	0	21.5%	0.0%	0.0%	0.0%	0.0%	0.0%	0.0%	0.0%
tr|F1SCN0|F1SCN0	2	0	0	0	0	0	0	0	2	0	0	0	0	0	0	0	4.6%	0.0%	0.0%	0.0%	0.0%	0.0%	0.0%	0.0%
tr|F1SPX9|F1SPX9	2	0	0	0	0	0	0	0	2	0	0	0	0	0	0	0	4.9%	0.0%	0.0%	0.0%	0.0%	0.0%	0.0%	0.0%
tr|F1SUN6|F1SUN6	0	1	0	0	0	0	0	0	0	2	0	0	0	0	0	0	0.0%	3.4%	0.0%	0.0%	0.0%	0.0%	0.0%	0.0%
tr|F1S448|F1S448	1	0	0	0	0	0	0	0	2	0	0	0	0	0	0	0	2.2%	0.0%	0.0%	0.0%	0.0%	0.0%	0.0%	0.0%
tr|F1SQ52|F1SQ52	1	0	0	0	0	0	0	0	2	0	0	0	0	0	0	0	4.3%	0.0%	0.0%	0.0%	0.0%	0.0%	0.0%	0.0%
tr|F1SI92|F1SI92	2	0	0	0	0	0	0	0	2	0	0	0	0	0	0	0	10.5%	0.0%	0.0%	0.0%	0.0%	0.0%	0.0%	0.0%
tr|I3LS23|I3LS23	1	1	0	0	0	0	0	0	1	1	0	0	0	0	0	0	6.1%	6.1%	0.0%	0.0%	0.0%	0.0%	0.0%	0.0%
tr|F1SRB8|F1SRB8	1	0	0	0	0	0	0	0	2	0	0	0	0	0	0	0	2.9%	0.0%	0.0%	0.0%	0.0%	0.0%	0.0%	0.0%
tr|K7GPF8|K7GPF8	2	0	0	0	0	0	0	0	2	0	0	0	0	0	0	0	13.0%	0.0%	0.0%	0.0%	0.0%	0.0%	0.0%	0.0%
tr|I3LAZ7|I3LAZ7	2	0	0	0	0	0	0	0	2	0	0	0	0	0	0	0	22.7%	0.0%	0.0%	0.0%	0.0%	0.0%	0.0%	0.0%
tr|F1S7U4|F1S7U4	1	1	0	0	0	0	0	0	1	1	0	0	0	0	0	0	1.0%	0.9%	0.0%	0.0%	0.0%	0.0%	0.0%	0.0%
tr|I3LK31|I3LK31	0	1	0	0	0	0	0	0	0	2	0	0	0	0	0	0	0.0%	4.6%	0.0%	0.0%	0.0%	0.0%	0.0%	0.0%
tr|I3LFH5|I3LFH5	0	1	0	0	0	0	0	0	0	2	0	0	0	0	0	0	0.0%	9.2%	0.0%	0.0%	0.0%	0.0%	0.0%	0.0%
tr|F1RIS6|F1RIS6	1	0	0	0	0	0	0	0	2	0	0	0	0	0	0	0	7.3%	0.0%	0.0%	0.0%	0.0%	0.0%	0.0%	0.0%
tr|F1SJI5|F1SJI5	0	1	0	0	0	0	0	0	0	2	0	0	0	0	0	0	0.0%	2.9%	0.0%	0.0%	0.0%	0.0%	0.0%	0.0%
tr|K7GMN4|K7GMN4	1	0	0	0	0	0	0	0	2	0	0	0	0	0	0	0	3.7%	0.0%	0.0%	0.0%	0.0%	0.0%	0.0%	0.0%
tr|F2Z5V7|F2Z5V7	0	1	0	0	0	0	0	0	0	2	0	0	0	0	0	0	0.0%	13.3%	0.0%	0.0%	0.0%	0.0%	0.0%	0.0%
tr|F1SMD1|F1SMD1	0	1	0	0	0	0	0	0	0	2	0	0	0	0	0	0	0.0%	0.8%	0.0%	0.0%	0.0%	0.0%	0.0%	0.0%
tr|I3LUL1|I3LUL1	1	0	0	0	0	0	0	0	2	0	0	0	0	0	0	0	3.4%	0.0%	0.0%	0.0%	0.0%	0.0%	0.0%	0.0%
tr|F1SHE8|F1SHE8	2	0	0	0	0	0	0	0	2	0	0	0	0	0	0	0	13.7%	0.0%	0.0%	0.0%	0.0%	0.0%	0.0%	0.0%
tr|F1RI83|F1RI83	1	1	0	0	0	0	0	0	1	1	0	0	0	0	0	0	3.5%	2.5%	0.0%	0.0%	0.0%	0.0%	0.0%	0.0%
tr|F1SI54|F1SI54	2	0	0	0	0	0	0	0	2	0	0	0	0	0	0	0	20.5%	0.0%	0.0%	0.0%	0.0%	0.0%	0.0%	0.0%
tr|I3LLY6|I3LLY6	1	1	0	0	0	0	0	0	1	1	0	0	0	0	0	0	7.6%	3.2%	0.0%	0.0%	0.0%	0.0%	0.0%	0.0%
tr|I3LFU8|I3LFU8	2	0	0	0	0	0	0	0	2	0	0	0	0	0	0	0	9.2%	0.0%	0.0%	0.0%	0.0%	0.0%	0.0%	0.0%
tr|F1RJU1|F1RJU1	0	1	0	0	0	0	0	0	0	2	0	0	0	0	0	0	0.0%	2.8%	0.0%	0.0%	0.0%	0.0%	0.0%	0.0%
tr|F1SMH6|F1SMH6	1	0	0	0	0	0	0	0	2	0	0	0	0	0	0	0	3.8%	0.0%	0.0%	0.0%	0.0%	0.0%	0.0%	0.0%
tr|I3LBQ6|I3LBQ6	2	0	0	0	0	0	0	0	2	0	0	0	0	0	0	0	7.9%	0.0%	0.0%	0.0%	0.0%	0.0%	0.0%	0.0%
tr|I3L9E4|I3L9E4	2	0	0	0	0	0	0	0	2	0	0	0	0	0	0	0	8.2%	0.0%	0.0%	0.0%	0.0%	0.0%	0.0%	0.0%
tr|F1SCH4|F1SCH4	2	0	0	0	0	0	0	0	2	0	0	0	0	0	0	0	3.1%	0.0%	0.0%	0.0%	0.0%	0.0%	0.0%	0.0%
tr|F1SJJ4|F1SJJ4	1	1	0	0	0	0	0	0	1	1	0	0	0	0	0	0	2.6%	3.2%	0.0%	0.0%	0.0%	0.0%	0.0%	0.0%
tr|K7GKE1|K7GKE1	2	0	0	0	0	0	0	0	2	0	0	0	0	0	0	0	15.7%	0.0%	0.0%	0.0%	0.0%	0.0%	0.0%	0.0%
tr|F1RTH6|F1RTH6	2	0	0	0	0	0	0	0	2	0	0	0	0	0	0	0	1.7%	0.0%	0.0%	0.0%	0.0%	0.0%	0.0%	0.0%
tr|F1RUJ0|F1RUJ0	0	1	0	0	0	0	0	0	0	2	0	0	0	0	0	0	0.0%	2.1%	0.0%	0.0%	0.0%	0.0%	0.0%	0.0%
tr|B0LXP7|B0LXP7	1	1	0	0	0	0	0	0	1	1	0	0	0	0	0	0	4.9%	4.9%	0.0%	0.0%	0.0%	0.0%	0.0%	0.0%
tr|F1RLF2|F1RLF2	0	1	0	0	0	0	0	0	0	2	0	0	0	0	0	0	0.0%	0.6%	0.0%	0.0%	0.0%	0.0%	0.0%	0.0%
tr|F1SRD9|F1SRD9	1	0	0	0	0	0	0	0	2	0	0	0	0	0	0	0	14.9%	0.0%	0.0%	0.0%	0.0%	0.0%	0.0%	0.0%
tr|F1RNN8|F1RNN8	0	1	0	0	0	0	0	0	0	2	0	0	0	0	0	0	0.0%	18.2%	0.0%	0.0%	0.0%	0.0%	0.0%	0.0%
tr|F1RFD0|F1RFD0	1	0	0	0	0	0	0	0	2	0	0	0	0	0	0	0	5.2%	0.0%	0.0%	0.0%	0.0%	0.0%	0.0%	0.0%
tr|F1S224|F1S224	2	0	0	0	0	0	0	0	2	0	0	0	0	0	0	0	3.4%	0.0%	0.0%	0.0%	0.0%	0.0%	0.0%	0.0%
tr|K7GKW0|K7GKW0	1	0	0	0	0	0	0	0	2	0	0	0	0	0	0	0	11.5%	0.0%	0.0%	0.0%	0.0%	0.0%	0.0%	0.0%
B3VKQ2	2	0	0	0	0	0	0	0	2	0	0	0	0	0	0	0	5.4%	0.0%	0.0%	0.0%	0.0%	0.0%	0.0%	0.0%
tr|I3L9E6|I3L9E6	2	0	0	0	0	0	0	0	2	0	0	0	0	0	0	0	2.7%	0.0%	0.0%	0.0%	0.0%	0.0%	0.0%	0.0%
tr|I3LNR5|I3LNR5	0	1	0	0	0	0	0	0	0	2	0	0	0	0	0	0	0.0%	3.2%	0.0%	0.0%	0.0%	0.0%	0.0%	0.0%
tr|F1S438|F1S438	0	1	0	0	0	0	0	0	0	2	0	0	0	0	0	0	0.0%	7.4%	0.0%	0.0%	0.0%	0.0%	0.0%	0.0%
tr|I3LFX2|I3LFX2	2	0	0	0	0	0	0	0	2	0	0	0	0	0	0	0	9.2%	0.0%	0.0%	0.0%	0.0%	0.0%	0.0%	0.0%
tr|I3L7B8|I3L7B8	2	0	0	0	0	0	0	0	2	0	0	0	0	0	0	0	15.5%	0.0%	0.0%	0.0%	0.0%	0.0%	0.0%	0.0%
tr|F1S727|F1S727	1	1	0	0	0	0	0	0	1	1	0	0	0	0	0	0	2.1%	0.8%	0.0%	0.0%	0.0%	0.0%	0.0%	0.0%
tr|I3LAF9|I3LAF9	0	2	0	0	0	0	0	0	0	2	0	0	0	0	0	0	0.0%	3.0%	0.0%	0.0%	0.0%	0.0%	0.0%	0.0%
tr|I3LPS8|I3LPS8	0	1	0	0	0	0	0	0	0	2	0	0	0	0	0	0	0.0%	4.9%	0.0%	0.0%	0.0%	0.0%	0.0%	0.0%
tr|B8XSJ9|B8XSJ9	0	1	0	0	0	0	0	0	0	2	0	0	0	0	0	0	0.0%	4.3%	0.0%	0.0%	0.0%	0.0%	0.0%	0.0%
tr|I3LD06|I3LD06	1	1	0	0	0	0	0	0	1	1	0	0	0	0	0	0	2.1%	1.1%	0.0%	0.0%	0.0%	0.0%	0.0%	0.0%
tr|F1S9U2|F1S9U2	1	1	0	0	0	0	0	0	1	1	0	0	0	0	0	0	3.6%	2.9%	0.0%	0.0%	0.0%	0.0%	0.0%	0.0%
Reverse_sp|P51779|CFAD	0	1	0	0	0	0	0	0	0	2	0	0	0	0	0	0	0.0%	7.7%	0.0%	0.0%	0.0%	0.0%	0.0%	0.0%
tr|I3LQK9|I3LQK9	0	1	0	0	0	0	0	0	0	2	0	0	0	0	0	0	0.0%	2.5%	0.0%	0.0%	0.0%	0.0%	0.0%	0.0%
tr|I3LGA2|I3LGA2	0	2	0	0	0	0	0	0	0	2	0	0	0	0	0	0	0.0%	7.5%	0.0%	0.0%	0.0%	0.0%	0.0%	0.0%
tr|F1S4F8|F1S4F8	0	1	0	0	0	0	0	0	0	2	0	0	0	0	0	0	0.0%	2.2%	0.0%	0.0%	0.0%	0.0%	0.0%	0.0%
tr|F1SGN5|F1SGN5	2	0	0	0	0	0	0	0	2	0	0	0	0	0	0	0	4.0%	0.0%	0.0%	0.0%	0.0%	0.0%	0.0%	0.0%
tr|K7GPP3|K7GPP3	1	0	0	0	0	0	0	0	2	0	0	0	0	0	0	0	15.8%	0.0%	0.0%	0.0%	0.0%	0.0%	0.0%	0.0%
tr|F1RJL6|F1RJL6	2	0	0	0	0	0	0	0	2	0	0	0	0	0	0	0	3.6%	0.0%	0.0%	0.0%	0.0%	0.0%	0.0%	0.0%
tr|F1RZM8|F1RZM8	2	0	0	0	0	0	0	0	2	0	0	0	0	0	0	0	2.8%	0.0%	0.0%	0.0%	0.0%	0.0%	0.0%	0.0%
tr|F1RFP4|F1RFP4	2	0	0	0	0	0	0	0	2	0	0	0	0	0	0	0	8.1%	0.0%	0.0%	0.0%	0.0%	0.0%	0.0%	0.0%
tr|I3L992|I3L992	2	0	0	0	0	0	0	0	2	0	0	0	0	0	0	0	3.6%	0.0%	0.0%	0.0%	0.0%	0.0%	0.0%	0.0%
tr|I3LMN7|I3LMN7	2	0	0	0	0	0	0	0	2	0	0	0	0	0	0	0	47.1%	0.0%	0.0%	0.0%	0.0%	0.0%	0.0%	0.0%
tr|F1RIY4|F1RIY4	1	1	0	0	0	0	0	0	1	1	0	0	0	0	0	0	5.5%	4.0%	0.0%	0.0%	0.0%	0.0%	0.0%	0.0%
tr|I3LJZ6|I3LJZ6	0	1	0	0	0	0	0	0	0	2	0	0	0	0	0	0	0.0%	4.7%	0.0%	0.0%	0.0%	0.0%	0.0%	0.0%
tr|I3LJX6|I3LJX6	0	1	0	0	0	0	0	0	0	2	0	0	0	0	0	0	0.0%	6.6%	0.0%	0.0%	0.0%	0.0%	0.0%	0.0%
tr|I3LNW2|I3LNW2	2	0	0	0	0	0	0	0	2	0	0	0	0	0	0	0	9.5%	0.0%	0.0%	0.0%	0.0%	0.0%	0.0%	0.0%
tr|I3LDG0|I3LDG0	0	1	0	0	0	0	0	0	0	2	0	0	0	0	0	0	0.0%	1.9%	0.0%	0.0%	0.0%	0.0%	0.0%	0.0%
tr|F1RM07|F1RM07	1	1	0	0	0	0	0	0	1	1	0	0	0	0	0	0	1.7%	0.7%	0.0%	0.0%	0.0%	0.0%	0.0%	0.0%
tr|F1RMI9|F1RMI9	0	1	0	0	0	0	0	0	0	2	0	0	0	0	0	0	0.0%	1.2%	0.0%	0.0%	0.0%	0.0%	0.0%	0.0%
tr|F1S7Z0|F1S7Z0	1	1	0	0	0	0	0	0	1	1	0	0	0	0	0	0	4.6%	2.6%	0.0%	0.0%	0.0%	0.0%	0.0%	0.0%
tr|I3LIU0|I3LIU0	2	0	0	0	0	0	0	0	2	0	0	0	0	0	0	0	3.5%	0.0%	0.0%	0.0%	0.0%	0.0%	0.0%	0.0%

	Peptide Count								Spectral Count								Sequence Coverage							
	Whole Cell	Whole Cell	Whole Cell	Cilia Fraction	Cilia Fraction	Cilia Fraction	Cilia Fraction	Cilia Fraction	Whole Cell	Whole Cell	Whole Cell	Cilia Fraction	Cilia Fraction	Cilia Fraction	Cilia Fraction	Cilia Fraction	Whole Cell	Whole Cell	Whole Cell	Cilia Fraction	Cilia Fraction	Cilia Fraction	Cilia Fraction	Cilia Fraction
Sample#	1	2	6	4	3	5	7	8	1	2	6	4	3	5	7	8	1	2	6	4	3	5	7	8
**Accession**																								
tr|I3LTD5|I3LTD5	1	1	0	0	0	0	0	0	1	1	0	0	0	0	0	0	0.8%	0.9%	0.0%	0.0%	0.0%	0.0%	0.0%	0.0%
tr|I3L9H4|I3L9H4	2	0	0	0	0	0	0	0	2	0	0	0	0	0	0	0	14.6%	0.0%	0.0%	0.0%	0.0%	0.0%	0.0%	0.0%
tr|I3L8P7|I3L8P7	1	0	0	0	0	0	0	0	2	0	0	0	0	0	0	0	20.0%	0.0%	0.0%	0.0%	0.0%	0.0%	0.0%	0.0%
tr|F1SAI6|F1SAI6	1	0	0	0	0	0	0	0	2	0	0	0	0	0	0	0	3.8%	0.0%	0.0%	0.0%	0.0%	0.0%	0.0%	0.0%
tr|I3L660|I3L660	1	1	0	0	0	0	0	0	1	1	0	0	0	0	0	0	8.7%	5.0%	0.0%	0.0%	0.0%	0.0%	0.0%	0.0%
tr|F1RT88|F1RT88	0	1	0	0	0	0	0	0	0	2	0	0	0	0	0	0	0.0%	0.3%	0.0%	0.0%	0.0%	0.0%	0.0%	0.0%
tr|K7GPA9|K7GPA9	0	1	0	0	0	0	0	0	0	2	0	0	0	0	0	0	0.0%	11.2%	0.0%	0.0%	0.0%	0.0%	0.0%	0.0%
tr|I3LKI4|I3LKI4	1	1	0	0	0	0	0	0	1	1	0	0	0	0	0	0	3.9%	3.3%	0.0%	0.0%	0.0%	0.0%	0.0%	0.0%
tr|I3LHT9|I3LHT9	2	0	0	0	0	0	0	0	2	0	0	0	0	0	0	0	15.6%	0.0%	0.0%	0.0%	0.0%	0.0%	0.0%	0.0%
tr|I3L6X7|I3L6X7	2	0	0	0	0	0	0	0	2	0	0	0	0	0	0	0	20.2%	0.0%	0.0%	0.0%	0.0%	0.0%	0.0%	0.0%
P57770	0	1	0	0	0	0	0	0	0	2	0	0	0	0	0	0	0.0%	2.2%	0.0%	0.0%	0.0%	0.0%	0.0%	0.0%
tr|F1RFD8|F1RFD8	1	1	0	0	0	0	0	0	1	1	0	0	0	0	0	0	0.8%	0.5%	0.0%	0.0%	0.0%	0.0%	0.0%	0.0%
tr|I3LKY3|I3LKY3	2	0	0	0	0	0	0	0	2	0	0	0	0	0	0	0	9.4%	0.0%	0.0%	0.0%	0.0%	0.0%	0.0%	0.0%
Q8HXL3	0	1	0	0	0	0	0	0	0	2	0	0	0	0	0	0	0.0%	0.6%	0.0%	0.0%	0.0%	0.0%	0.0%	0.0%
tr|I3LRJ6|I3LRJ6	2	0	0	0	0	0	0	0	2	0	0	0	0	0	0	0	2.7%	0.0%	0.0%	0.0%	0.0%	0.0%	0.0%	0.0%
tr|F1SSN0|F1SSN0	0	1	0	0	0	0	0	0	0	2	0	0	0	0	0	0	0.0%	2.5%	0.0%	0.0%	0.0%	0.0%	0.0%	0.0%
tr|K7GKX0|K7GKX0	0	1	0	0	0	0	0	0	0	2	0	0	0	0	0	0	0.0%	1.2%	0.0%	0.0%	0.0%	0.0%	0.0%	0.0%
tr|F1SAH7|F1SAH7	2	0	0	0	0	0	0	0	2	0	0	0	0	0	0	0	4.4%	0.0%	0.0%	0.0%	0.0%	0.0%	0.0%	0.0%
tr|K7GS44|K7GS44	0	1	0	0	0	0	0	0	0	2	0	0	0	0	0	0	0.0%	2.7%	0.0%	0.0%	0.0%	0.0%	0.0%	0.0%
tr|F1ST00|F1ST00	2	0	0	0	0	0	0	0	2	0	0	0	0	0	0	0	3.9%	0.0%	0.0%	0.0%	0.0%	0.0%	0.0%	0.0%
tr|F1RMA8|F1RMA8	2	0	0	0	0	0	0	0	2	0	0	0	0	0	0	0	6.9%	0.0%	0.0%	0.0%	0.0%	0.0%	0.0%	0.0%
tr|K7GPF0|K7GPF0	1	0	0	0	0	0	0	0	2	0	0	0	0	0	0	0	11.3%	0.0%	0.0%	0.0%	0.0%	0.0%	0.0%	0.0%
tr|I3L6K4|I3L6K4	0	1	0	0	0	0	0	0	0	2	0	0	0	0	0	0	0.0%	6.0%	0.0%	0.0%	0.0%	0.0%	0.0%	0.0%
tr|K7GP33|K7GP33	2	0	0	0	0	0	0	0	2	0	0	0	0	0	0	0	19.0%	0.0%	0.0%	0.0%	0.0%	0.0%	0.0%	0.0%
tr|F1SUU9|F1SUU9	1	1	0	0	0	0	0	0	1	1	0	0	0	0	0	0	3.1%	3.8%	0.0%	0.0%	0.0%	0.0%	0.0%	0.0%
tr|F1SE47|F1SE47	2	0	0	0	0	0	0	0	2	0	0	0	0	0	0	0	4.2%	0.0%	0.0%	0.0%	0.0%	0.0%	0.0%	0.0%
Reverse_tr|F1SQA3|F1SQA3	1	0	0	0	0	0	0	0	2	0	0	0	0	0	0	0	4.5%	0.0%	0.0%	0.0%	0.0%	0.0%	0.0%	0.0%
tr|F1S5K6|F1S5K6	2	0	0	0	0	0	0	0	2	0	0	0	0	0	0	0	4.7%	0.0%	0.0%	0.0%	0.0%	0.0%	0.0%	0.0%
tr|F1SJG5|F1SJG5	2	0	0	0	0	0	0	0	2	0	0	0	0	0	0	0	2.1%	0.0%	0.0%	0.0%	0.0%	0.0%	0.0%	0.0%
tr|I3LUF5|I3LUF5	0	1	0	0	0	0	0	0	0	2	0	0	0	0	0	0	0.0%	3.2%	0.0%	0.0%	0.0%	0.0%	0.0%	0.0%
tr|F1RGB8|F1RGB8	2	0	0	0	0	0	0	0	2	0	0	0	0	0	0	0	6.6%	0.0%	0.0%	0.0%	0.0%	0.0%	0.0%	0.0%
tr|A1Y2K2|A1Y2K2	2	0	0	0	0	0	0	0	2	0	0	0	0	0	0	0	3.8%	0.0%	0.0%	0.0%	0.0%	0.0%	0.0%	0.0%
tr|F1SBZ9|F1SBZ9	0	1	0	0	0	0	0	0	0	2	0	0	0	0	0	0	0.0%	0.6%	0.0%	0.0%	0.0%	0.0%	0.0%	0.0%
tr|I3LLT8|I3LLT8	2	0	0	0	0	0	0	0	2	0	0	0	0	0	0	0	9.7%	0.0%	0.0%	0.0%	0.0%	0.0%	0.0%	0.0%
tr|I3L6H4|I3L6H4	0	1	0	0	0	0	0	0	0	2	0	0	0	0	0	0	0.0%	12.6%	0.0%	0.0%	0.0%	0.0%	0.0%	0.0%
tr|F1SHX3|F1SHX3	2	0	0	0	0	0	0	0	2	0	0	0	0	0	0	0	1.6%	0.0%	0.0%	0.0%	0.0%	0.0%	0.0%	0.0%
tr|I3LJD7|I3LJD7	1	0	0	0	0	0	0	0	2	0	0	0	0	0	0	0	28.2%	0.0%	0.0%	0.0%	0.0%	0.0%	0.0%	0.0%
tr|I3L8A1|I3L8A1	2	0	0	0	0	0	0	0	2	0	0	0	0	0	0	0	4.9%	0.0%	0.0%	0.0%	0.0%	0.0%	0.0%	0.0%
tr|F1SDB6|F1SDB6	1	0	0	0	0	0	0	0	2	0	0	0	0	0	0	0	11.0%	0.0%	0.0%	0.0%	0.0%	0.0%	0.0%	0.0%
tr|I3LBG0|I3LBG0	2	0	0	0	0	0	0	0	2	0	0	0	0	0	0	0	6.8%	0.0%	0.0%	0.0%	0.0%	0.0%	0.0%	0.0%
tr|F1SSN9|F1SSN9	2	0	0	0	0	0	0	0	2	0	0	0	0	0	0	0	4.8%	0.0%	0.0%	0.0%	0.0%	0.0%	0.0%	0.0%
tr|I3L778|I3L778	1	0	0	0	0	0	0	0	2	0	0	0	0	0	0	0	11.9%	0.0%	0.0%	0.0%	0.0%	0.0%	0.0%	0.0%
tr|F1RFB9|F1RFB9	2	0	0	0	0	0	0	0	2	0	0	0	0	0	0	0	11.2%	0.0%	0.0%	0.0%	0.0%	0.0%	0.0%	0.0%
tr|F1RS72|F1RS72	2	0	0	0	0	0	0	0	2	0	0	0	0	0	0	0	4.9%	0.0%	0.0%	0.0%	0.0%	0.0%	0.0%	0.0%
tr|I3LSW0|I3LSW0	1	1	0	0	0	0	0	0	1	1	0	0	0	0	0	0	16.7%	9.3%	0.0%	0.0%	0.0%	0.0%	0.0%	0.0%
tr|F1SEK2|F1SEK2	0	1	0	0	0	0	0	0	0	2	0	0	0	0	0	0	0.0%	1.1%	0.0%	0.0%	0.0%	0.0%	0.0%	0.0%
tr|F1SUG1|F1SUG1	2	0	0	0	0	0	0	0	2	0	0	0	0	0	0	0	5.3%	0.0%	0.0%	0.0%	0.0%	0.0%	0.0%	0.0%
tr|K7GN44|K7GN44	2	0	0	0	0	0	0	0	2	0	0	0	0	0	0	0	5.3%	0.0%	0.0%	0.0%	0.0%	0.0%	0.0%	0.0%
tr|F1SDD4|F1SDD4	2	0	0	0	0	0	0	0	2	0	0	0	0	0	0	0	6.0%	0.0%	0.0%	0.0%	0.0%	0.0%	0.0%	0.0%
tr|F1S3S7|F1S3S7	1	0	0	0	0	0	0	0	2	0	0	0	0	0	0	0	6.6%	0.0%	0.0%	0.0%	0.0%	0.0%	0.0%	0.0%
tr|I3L9V8|I3L9V8	1	0	0	0	0	0	0	0	2	0	0	0	0	0	0	0	14.8%	0.0%	0.0%	0.0%	0.0%	0.0%	0.0%	0.0%
tr|F1S2V3|F1S2V3	1	0	0	0	0	0	0	0	2	0	0	0	0	0	0	0	8.3%	0.0%	0.0%	0.0%	0.0%	0.0%	0.0%	0.0%
tr|I3LP05|I3LP05	2	0	0	0	0	0	0	0	2	0	0	0	0	0	0	0	20.2%	0.0%	0.0%	0.0%	0.0%	0.0%	0.0%	0.0%
tr|F1S6P9|F1S6P9	2	0	0	0	0	0	0	0	2	0	0	0	0	0	0	0	4.4%	0.0%	0.0%	0.0%	0.0%	0.0%	0.0%	0.0%
tr|I3LJK4|I3LJK4	1	0	0	0	0	0	0	0	2	0	0	0	0	0	0	0	9.2%	0.0%	0.0%	0.0%	0.0%	0.0%	0.0%	0.0%
tr|F1S9V0|F1S9V0	0	1	0	0	0	0	0	0	0	2	0	0	0	0	0	0	0.0%	1.2%	0.0%	0.0%	0.0%	0.0%	0.0%	0.0%
tr|I3LF57|I3LF57	2	0	0	0	0	0	0	0	2	0	0	0	0	0	0	0	29.7%	0.0%	0.0%	0.0%	0.0%	0.0%	0.0%	0.0%
tr|I3LTQ1|I3LTQ1	2	0	0	0	0	0	0	0	2	0	0	0	0	0	0	0	2.3%	0.0%	0.0%	0.0%	0.0%	0.0%	0.0%	0.0%
Reverse_tr|F1SID7|F1SID7	1	0	0	0	0	0	0	0	2	0	0	0	0	0	0	0	2.0%	0.0%	0.0%	0.0%	0.0%	0.0%	0.0%	0.0%
tr|F1SFU9|F1SFU9	2	0	0	0	0	0	0	0	2	0	0	0	0	0	0	0	6.5%	0.0%	0.0%	0.0%	0.0%	0.0%	0.0%	0.0%
tr|I3LPU4|I3LPU4	1	0	0	0	0	0	0	0	2	0	0	0	0	0	0	0	7.2%	0.0%	0.0%	0.0%	0.0%	0.0%	0.0%	0.0%
tr|I3LJT3|I3LJT3	0	1	0	0	0	0	0	0	0	2	0	0	0	0	0	0	0.0%	2.0%	0.0%	0.0%	0.0%	0.0%	0.0%	0.0%
tr|F1RMK7|F1RMK7	2	0	0	0	0	0	0	0	2	0	0	0	0	0	0	0	6.3%	0.0%	0.0%	0.0%	0.0%	0.0%	0.0%	0.0%
tr|F1SDE3|F1SDE3	2	0	0	0	0	0	0	0	2	0	0	0	0	0	0	0	17.5%	0.0%	0.0%	0.0%	0.0%	0.0%	0.0%	0.0%
tr|F1SUA2|F1SUA2	2	0	0	0	0	0	0	0	2	0	0	0	0	0	0	0	1.9%	0.0%	0.0%	0.0%	0.0%	0.0%	0.0%	0.0%
tr|I3LRP7|I3LRP7	2	0	1	0	0	0	0	0	2	0	1	0	0	0	0	0	17.2%	0.0%	8.6%	0.0%	0.0%	0.0%	0.0%	0.0%
tr|F1S939|F1S939	2	0	1	0	0	0	0	0	2	0	1	0	0	0	0	0	1.1%	0.0%	1.8%	0.0%	0.0%	0.0%	0.0%	0.0%
Q712U6	1	1	1	0	0	0	0	0	1	1	1	0	0	0	0	0	23.2%	7.1%	9.8%	0.0%	0.0%	0.0%	0.0%	0.0%
tr|F1SQ61|F1SQ61	1	0	1	0	0	0	0	0	2	0	1	0	0	0	0	0	2.6%	0.0%	2.5%	0.0%	0.0%	0.0%	0.0%	0.0%
tr|K7GNU1|K7GNU1	1	1	1	0	0	0	0	0	1	1	1	0	0	0	0	0	2.2%	1.1%	2.2%	0.0%	0.0%	0.0%	0.0%	0.0%
P79384	2	0	1	0	0	0	0	0	2	0	1	0	0	0	0	0	7.8%	0.0%	3.9%	0.0%	0.0%	0.0%	0.0%	0.0%
tr|F1S8P6|F1S8P6	0	2	1	0	0	0	0	0	0	2	1	0	0	0	0	0	0.0%	5.7%	2.9%	0.0%	0.0%	0.0%	0.0%	0.0%
tr|K7GLS3|K7GLS3	1	1	1	0	0	0	0	0	1	1	1	0	0	0	0	0	3.1%	3.1%	1.9%	0.0%	0.0%	0.0%	0.0%	0.0%
tr|I3LNC2|I3LNC2	2	0	1	0	0	0	0	0	2	0	1	0	0	0	0	0	9.4%	0.0%	3.5%	0.0%	0.0%	0.0%	0.0%	0.0%
tr|F6Q1K6|F6Q1K6	1	1	1	0	0	0	0	0	1	1	1	0	0	0	0	0	2.1%	2.1%	2.6%	0.0%	0.0%	0.0%	0.0%	0.0%
tr|F1RG31|F1RG31	1	0	1	0	0	0	0	0	2	0	1	0	0	0	0	0	42.4%	0.0%	27.1%	0.0%	0.0%	0.0%	0.0%	0.0%
tr|M9MMN4|M9MMN4	1	1	1	0	0	0	0	0	1	1	1	0	0	0	0	0	5.5%	6.7%	6.5%	0.0%	0.0%	0.0%	0.0%	0.0%
tr|F1RRX1|F1RRX1	0	1	1	0	0	0	0	0	0	2	1	0	0	0	0	0	0.0%	7.5%	7.5%	0.0%	0.0%	0.0%	0.0%	0.0%
tr|I3L777|I3L777	2	0	1	0	0	0	0	0	2	0	1	0	0	0	0	0	2.2%	0.0%	4.2%	0.0%	0.0%	0.0%	0.0%	0.0%
tr|I3LUV8|I3LUV8	2	0	1	0	0	0	0	0	2	0	1	0	0	0	0	0	33.6%	0.0%	15.5%	0.0%	0.0%	0.0%	0.0%	0.0%
tr|F1S6R1|F1S6R1	0	1	1	0	0	0	0	0	0	2	1	0	0	0	0	0	0.0%	12.2%	6.4%	0.0%	0.0%	0.0%	0.0%	0.0%

	Peptide Count								Spectral Count								Sequence Coverage							
	Whole Cell	Whole Cell	Whole Cell	Cilia Fraction	Cilia Fraction	Cilia Fraction	Cilia Fraction	Cilia Fraction	Whole Cell	Whole Cell	Whole Cell	Cilia Fraction	Cilia Fraction	Cilia Fraction	Cilia Fraction	Cilia Fraction	Whole Cell	Whole Cell	Whole Cell	Cilia Fraction	Cilia Fraction	Cilia Fraction	Cilia Fraction	Cilia Fraction
Sample#	1	2	6	4	3	5	7	8	1	2	6	4	3	5	7	8	1	2	6	4	3	5	7	8
**Accession**																								
tr|I3LFM8|I3LFM8	0	1	1	0	0	0	0	0	0	2	1	0	0	0	0	0	0.0%	9.3%	19.8%	0.0%	0.0%	0.0%	0.0%	0.0%
tr|F1SRQ7|F1SRQ7	1	0	1	0	0	0	0	0	2	0	1	0	0	0	0	0	10.1%	0.0%	9.5%	0.0%	0.0%	0.0%	0.0%	0.0%
tr|F1RSN8|F1RSN8	1	1	1	0	0	0	0	0	1	1	1	0	0	0	0	0	1.5%	0.7%	1.5%	0.0%	0.0%	0.0%	0.0%	0.0%
tr|K7GPI8|K7GPI8	0	2	1	0	0	0	0	0	0	2	1	0	0	0	0	0	0.0%	1.3%	1.8%	0.0%	0.0%	0.0%	0.0%	0.0%
tr|I3LGM1|I3LGM1	0	2	1	0	0	0	0	0	0	2	1	0	0	0	0	0	0.0%	2.1%	2.9%	0.0%	0.0%	0.0%	0.0%	0.0%
tr|I3LBW0|I3LBW0	2	0	1	0	0	0	0	0	2	0	1	0	0	0	0	0	9.4%	0.0%	3.5%	0.0%	0.0%	0.0%	0.0%	0.0%
tr|B6UV57|B6UV57	2	0	1	0	0	0	0	0	2	0	1	0	0	0	0	0	5.7%	0.0%	3.1%	0.0%	0.0%	0.0%	0.0%	0.0%
tr|F1SQU8|F1SQU8	1	1	1	0	0	0	0	0	1	1	1	0	0	0	0	0	2.9%	2.9%	1.7%	0.0%	0.0%	0.0%	0.0%	0.0%
tr|F1SEA5|F1SEA5	1	0	1	0	0	0	0	0	2	0	1	0	0	0	0	0	2.1%	0.0%	1.7%	0.0%	0.0%	0.0%	0.0%	0.0%
tr|F1SD86|F1SD86	2	0	1	0	0	0	0	0	2	0	1	0	0	0	0	0	2.4%	0.0%	0.7%	0.0%	0.0%	0.0%	0.0%	0.0%
tr|F1RZX9|F1RZX9	2	0	1	0	0	0	0	0	2	0	1	0	0	0	0	0	16.2%	0.0%	8.1%	0.0%	0.0%	0.0%	0.0%	0.0%
tr|I3LAX9|I3LAX9	2	0	1	0	0	0	0	0	2	0	2	0	0	0	0	0	8.9%	0.0%	5.4%	0.0%	0.0%	0.0%	0.0%	0.0%
tr|F1RUP2|F1RUP2	2	0	1	0	0	0	0	0	2	0	2	0	0	0	0	0	9.4%	0.0%	4.0%	0.0%	0.0%	0.0%	0.0%	0.0%
tr|F1RGE8|F1RGE8	2	0	2	0	0	0	0	0	2	0	2	0	0	0	0	0	1.3%	0.0%	1.7%	0.0%	0.0%	0.0%	0.0%	0.0%
tr|I3LB49|I3LB49	2	0	1	0	0	0	0	0	2	0	2	0	0	0	0	0	5.2%	0.0%	2.7%	0.0%	0.0%	0.0%	0.0%	0.0%
tr|I3LQU8|I3LQU8	0	1	1	0	0	0	0	0	0	2	2	0	0	0	0	0	0.0%	16.1%	15.3%	0.0%	0.0%	0.0%	0.0%	0.0%
tr|F1S650|F1S650	2	0	1	0	0	0	0	0	2	0	2	0	0	0	0	0	9.0%	0.0%	5.4%	0.0%	0.0%	0.0%	0.0%	0.0%
tr|I3LAQ6|I3LAQ6	2	0	1	0	0	0	0	0	2	0	2	0	0	0	0	0	22.1%	0.0%	10.5%	0.0%	0.0%	0.0%	0.0%	0.0%
tr|I3LKA3|I3LKA3	1	1	1	0	0	0	0	0	1	1	2	0	0	0	0	0	3.2%	1.7%	1.5%	0.0%	0.0%	0.0%	0.0%	0.0%
tr|F1RYY7|F1RYY7	2	0	1	0	0	0	0	0	2	0	2	0	0	0	0	0	15.1%	0.0%	8.6%	0.0%	0.0%	0.0%	0.0%	0.0%
tr|F1STL4|F1STL4	1	1	1	0	0	0	0	0	1	1	2	0	0	0	0	0	4.3%	3.5%	4.0%	0.0%	0.0%	0.0%	0.0%	0.0%
tr|F1RQT4|F1RQT4	2	0	1	0	0	0	0	0	2	0	2	0	0	0	0	0	8.0%	0.0%	5.8%	0.0%	0.0%	0.0%	0.0%	0.0%
tr|F1RR38|F1RR38	2	0	1	0	0	0	0	0	2	0	2	0	0	0	0	0	4.5%	0.0%	12.0%	0.0%	0.0%	0.0%	0.0%	0.0%
tr|F1S594|F1S594	2	0	1	0	0	0	0	0	2	0	2	0	0	0	0	0	3.5%	0.0%	1.5%	0.0%	0.0%	0.0%	0.0%	0.0%
tr|F1SQI5|F1SQI5	1	1	1	0	0	0	0	0	1	1	2	0	0	0	0	0	2.2%	2.7%	2.4%	0.0%	0.0%	0.0%	0.0%	0.0%
tr|I3LT10|I3LT10	1	0	1	0	0	0	0	0	2	0	2	0	0	0	0	0	7.1%	0.0%	5.9%	0.0%	0.0%	0.0%	0.0%	0.0%
tr|F1SD63|F1SD63	1	1	1	0	0	0	0	0	1	1	2	0	0	0	0	0	2.0%	1.2%	3.3%	0.0%	0.0%	0.0%	0.0%	0.0%
tr|F1SPI2|F1SPI2	1	0	1	0	0	0	0	0	2	0	3	0	0	0	0	0	1.0%	0.0%	2.0%	0.0%	0.0%	0.0%	0.0%	0.0%
tr|F2Z5D1|F2Z5D1	1	0	2	0	0	0	0	0	2	0	3	0	0	0	0	0	3.4%	0.0%	8.6%	0.0%	0.0%	0.0%	0.0%	0.0%
tr|F1SB61|F1SB61	0	1	1	0	0	0	0	0	0	2	3	0	0	0	0	0	0.0%	3.0%	3.5%	0.0%	0.0%	0.0%	0.0%	0.0%
tr|F1S1D0|F1S1D0	2	0	1	0	0	0	0	0	2	0	3	0	0	0	0	0	4.4%	0.0%	2.3%	0.0%	0.0%	0.0%	0.0%	0.0%
tr|F1S108|F1S108	1	0	1	0	0	0	0	0	2	0	3	0	0	0	0	0	10.4%	0.0%	11.1%	0.0%	0.0%	0.0%	0.0%	0.0%
tr|I3LQU7|I3LQU7	0	1	1	0	0	0	0	0	0	2	3	0	0	0	0	0	0.0%	0.9%	2.0%	0.0%	0.0%	0.0%	0.0%	0.0%
tr|F1RY22|F1RY22	2	0	1	0	0	0	0	0	2	0	3	0	0	0	0	0	0.9%	0.0%	0.8%	0.0%	0.0%	0.0%	0.0%	0.0%
tr|F1RMF3|F1RMF3	2	0	3	0	0	0	0	0	2	0	3	0	0	0	0	0	13.9%	0.0%	16.3%	0.0%	0.0%	0.0%	0.0%	0.0%
tr|K7GQW7|K7GQW7	1	1	1	0	0	0	0	0	1	1	4	0	0	0	0	0	7.4%	3.7%	4.5%	0.0%	0.0%	0.0%	0.0%	0.0%
tr|F1SD98|F1SD98	1	1	1	0	0	0	0	0	1	1	4	0	0	0	0	0	2.8%	2.8%	3.5%	0.0%	0.0%	0.0%	0.0%	0.0%
tr|F1RG77|F1RG77	2	0	1	0	0	0	0	0	2	0	4	0	0	0	0	0	2.7%	0.0%	2.4%	0.0%	0.0%	0.0%	0.0%	0.0%
tr|I3LIR0|I3LIR0	2	0	2	0	0	0	0	0	2	0	4	0	0	0	0	0	11.7%	0.0%	11.4%	0.0%	0.0%	0.0%	0.0%	0.0%
tr|I3LR72|I3LR72	2	0	3	0	0	0	0	0	2	0	4	0	0	0	0	0	7.4%	0.0%	15.8%	0.0%	0.0%	0.0%	0.0%	0.0%
tr|I3LNN1|I3LNN1	2	0	1	0	0	0	0	0	2	0	4	0	0	0	0	0	2.7%	0.0%	2.7%	0.0%	0.0%	0.0%	0.0%	0.0%
tr|K7GT64|K7GT64	2	0	2	0	0	0	0	0	2	0	4	0	0	0	0	0	3.1%	0.0%	3.1%	0.0%	0.0%	0.0%	0.0%	0.0%
tr|I3LNJ0|I3LNJ0	1	1	1	0	0	0	0	0	1	1	4	0	0	0	0	0	10.3%	4.1%	10.3%	0.0%	0.0%	0.0%	0.0%	0.0%
tr|I3LNI6|I3LNI6	1	1	2	0	0	0	0	0	1	1	4	0	0	0	0	0	3.7%	4.0%	6.3%	0.0%	0.0%	0.0%	0.0%	0.0%
tr|I3LIN5|I3LIN5	1	0	2	0	0	0	0	0	2	0	4	0	0	0	0	0	6.0%	0.0%	15.8%	0.0%	0.0%	0.0%	0.0%	0.0%
tr|F1RVC4|F1RVC4	1	0	1	0	0	0	0	0	2	0	4	0	0	0	0	0	10.2%	0.0%	7.1%	0.0%	0.0%	0.0%	0.0%	0.0%
tr|F1RI80|F1RI80	1	1	2	0	0	0	0	0	1	1	4	0	0	0	0	0	1.5%	1.7%	5.3%	0.0%	0.0%	0.0%	0.0%	0.0%
tr|K7GSW3|K7GSW3	2	0	1	0	0	0	0	0	2	0	4	0	0	0	0	0	11.6%	0.0%	10.2%	0.0%	0.0%	0.0%	0.0%	0.0%
tr|F2Z5S4|F2Z5S4	2	0	1	0	0	0	0	0	2	0	4	0	0	0	0	0	45.1%	0.0%	11.8%	0.0%	0.0%	0.0%	0.0%	0.0%
tr|I3LNH8|I3LNH8	1	0	1	0	0	0	0	0	2	0	4	0	0	0	0	0	14.8%	0.0%	14.8%	0.0%	0.0%	0.0%	0.0%	0.0%
tr|F1RL85|F1RL85	2	0	2	0	0	0	0	0	2	0	4	0	0	0	0	0	9.7%	0.0%	11.0%	0.0%	0.0%	0.0%	0.0%	0.0%
tr|I3L6F0|I3L6F0	2	0	1	0	0	0	0	0	2	0	5	0	0	0	0	0	3.5%	0.0%	2.9%	0.0%	0.0%	0.0%	0.0%	0.0%
tr|F1SM44|F1SM44	1	1	1	0	0	0	0	0	1	1	5	0	0	0	0	0	1.7%	2.6%	3.1%	0.0%	0.0%	0.0%	0.0%	0.0%
tr|F1SV45|F1SV45	1	0	2	0	0	0	0	0	2	0	5	0	0	0	0	0	8.3%	0.0%	8.3%	0.0%	0.0%	0.0%	0.0%	0.0%
P81405	1	0	1	0	0	0	0	0	2	0	5	0	0	0	0	0	25.0%	0.0%	12.5%	0.0%	0.0%	0.0%	0.0%	0.0%
tr|K7GPE7|K7GPE7	2	0	1	0	0	0	0	0	2	0	5	0	0	0	0	0	16.2%	0.0%	6.6%	0.0%	0.0%	0.0%	0.0%	0.0%
tr|F1SAQ7|F1SAQ7	2	0	1	0	0	0	0	0	2	0	5	0	0	0	0	0	3.8%	0.0%	3.0%	0.0%	0.0%	0.0%	0.0%	0.0%
tr|F1S7X9|F1S7X9	2	0	1	0	0	0	0	0	2	0	5	0	0	0	0	0	4.1%	0.0%	4.6%	0.0%	0.0%	0.0%	0.0%	0.0%
tr|I3LJE7|I3LJE7	1	0	2	0	0	0	0	0	2	0	5	0	0	0	0	0	10.4%	0.0%	8.7%	0.0%	0.0%	0.0%	0.0%	0.0%
tr|F1RF24|F1RF24	1	0	2	0	0	0	0	0	2	0	6	0	0	0	0	0	0.7%	0.0%	3.0%	0.0%	0.0%	0.0%	0.0%	0.0%
tr|I3LSA6|I3LSA6	2	0	1	0	0	0	0	0	2	0	6	0	0	0	0	0	5.1%	0.0%	2.3%	0.0%	0.0%	0.0%	0.0%	0.0%
tr|I3LEA3|I3LEA3	1	1	1	0	0	0	0	0	1	1	6	0	0	0	0	0	8.8%	8.8%	8.8%	0.0%	0.0%	0.0%	0.0%	0.0%
tr|I3LQY4|I3LQY4	0	2	1	0	0	0	0	0	0	2	6	0	0	0	0	0	0.0%	2.8%	2.4%	0.0%	0.0%	0.0%	0.0%	0.0%
tr|F1RTZ1|F1RTZ1	2	0	2	0	0	0	0	0	2	0	6	0	0	0	0	0	8.9%	0.0%	6.6%	0.0%	0.0%	0.0%	0.0%	0.0%
tr|F1RLZ7|F1RLZ7	1	1	1	0	0	0	0	0	1	1	6	0	0	0	0	0	3.4%	3.4%	3.4%	0.0%	0.0%	0.0%	0.0%	0.0%
tr|I3L7D4|I3L7D4	2	0	2	0	0	0	0	0	2	0	6	0	0	0	0	0	10.5%	0.0%	24.5%	0.0%	0.0%	0.0%	0.0%	0.0%
tr|F1S0M3|F1S0M3	0	2	1	0	0	0	0	0	0	2	6	0	0	0	0	0	0.0%	2.9%	2.6%	0.0%	0.0%	0.0%	0.0%	0.0%
tr|K7GMB9|K7GMB9	2	0	2	0	0	0	0	0	2	0	6	0	0	0	0	0	10.0%	0.0%	7.4%	0.0%	0.0%	0.0%	0.0%	0.0%
tr|I3LGR7|I3LGR7	1	0	2	0	0	0	0	0	2	0	6	0	0	0	0	0	0.7%	0.0%	3.0%	0.0%	0.0%	0.0%	0.0%	0.0%
tr|I3LS27|I3LS27	1	0	2	0	0	0	0	0	2	0	6	0	0	0	0	0	0.7%	0.0%	3.0%	0.0%	0.0%	0.0%	0.0%	0.0%
B0FWK4	2	0	2	0	0	0	0	0	2	0	6	0	0	0	0	0	31.4%	0.0%	14.7%	0.0%	0.0%	0.0%	0.0%	0.0%
tr|I3L587|I3L587	1	0	2	0	0	0	0	0	2	0	6	0	0	0	0	0	0.7%	0.0%	3.1%	0.0%	0.0%	0.0%	0.0%	0.0%
tr|K7GN40|K7GN40	2	0	2	0	0	0	0	0	2	0	6	0	0	0	0	0	9.0%	0.0%	6.6%	0.0%	0.0%	0.0%	0.0%	0.0%
tr|F1RYS1|F1RYS1	1	1	1	0	0	0	0	0	1	1	6	0	0	0	0	0	9.0%	8.6%	7.2%	0.0%	0.0%	0.0%	0.0%	0.0%
tr|F1S4B2|F1S4B2	1	1	1	0	0	0	0	0	1	1	6	0	0	0	0	0	3.4%	3.4%	3.4%	0.0%	0.0%	0.0%	0.0%	0.0%
tr|I3LTC0|I3LTC0	2	0	1	0	0	0	0	0	2	0	6	0	0	0	0	0	18.7%	0.0%	12.0%	0.0%	0.0%	0.0%	0.0%	0.0%
tr|F1S537|F1S537	2	0	1	0	0	0	0	0	2	0	6	0	0	0	0	0	2.5%	0.0%	3.9%	0.0%	0.0%	0.0%	0.0%	0.0%
tr|F1SAB6|F1SAB6	1	0	3	0	0	0	0	0	2	0	7	0	0	0	0	0	11.5%	0.0%	21.2%	0.0%	0.0%	0.0%	0.0%	0.0%
tr|F1S5L7|F1S5L7	1	1	1	0	0	0	0	0	1	1	7	0	0	0	0	0	4.9%	2.3%	4.5%	0.0%	0.0%	0.0%	0.0%	0.0%
tr|K7GNG0|K7GNG0	2	0	1	0	0	0	0	0	2	0	7	0	0	0	0	0	18.0%	0.0%	7.9%	0.0%	0.0%	0.0%	0.0%	0.0%
tr|I3LDS4|I3LDS4	1	0	1	0	0	0	0	0	2	0	7	0	0	0	0	0	21.9%	0.0%	13.1%	0.0%	0.0%	0.0%	0.0%	0.0%

	Peptide Count								Spectral Count								Sequence Coverage							
	Whole Cell	Whole Cell	Whole Cell	Cilia Fraction	Cilia Fraction	Cilia Fraction	Cilia Fraction	Cilia Fraction	Whole Cell	Whole Cell	Whole Cell	Cilia Fraction	Cilia Fraction	Cilia Fraction	Cilia Fraction	Cilia Fraction	Whole Cell	Whole Cell	Whole Cell	Cilia Fraction	Cilia Fraction	Cilia Fraction	Cilia Fraction	Cilia Fraction
Sample#	1	2	6	4	3	5	7	8	1	2	6	4	3	5	7	8	1	2	6	4	3	5	7	8
**Accession**																								
tr|I3LG34|I3LG34	1	0	4	0	0	0	0	0	2	0	7	0	0	0	0	0	1.9%	0.0%	3.3%	0.0%	0.0%	0.0%	0.0%	0.0%
tr|F2Z5D8|F2Z5D8	1	1	2	0	0	0	0	0	1	1	7	0	0	0	0	0	1.9%	1.9%	5.8%	0.0%	0.0%	0.0%	0.0%	0.0%
tr|F1RY70|F1RY70	2	0	1	0	0	0	0	0	2	0	7	0	0	0	0	0	17.2%	0.0%	10.0%	0.0%	0.0%	0.0%	0.0%	0.0%
tr|F1S9I6|F1S9I6	1	0	1	0	0	0	0	0	2	0	8	0	0	0	0	0	7.6%	0.0%	7.6%	0.0%	0.0%	0.0%	0.0%	0.0%
tr|F1S1R4|F1S1R4	0	1	2	0	0	0	0	0	0	2	8	0	0	0	0	0	0.0%	12.1%	16.2%	0.0%	0.0%	0.0%	0.0%	0.0%
tr|F1RGX3|F1RGX3	2	0	2	0	0	0	0	0	2	0	8	0	0	0	0	0	7.0%	0.0%	4.9%	0.0%	0.0%	0.0%	0.0%	0.0%
tr|F1S413|F1S413	2	0	1	0	0	0	0	0	2	0	9	0	0	0	0	0	7.6%	0.0%	3.0%	0.0%	0.0%	0.0%	0.0%	0.0%
tr|F1RMA6|F1RMA6	1	1	3	0	0	0	0	0	1	1	9	0	0	0	0	0	1.5%	1.4%	5.5%	0.0%	0.0%	0.0%	0.0%	0.0%
tr|F1REW9|F1REW9	1	0	3	0	0	0	0	0	2	0	9	0	0	0	0	0	10.9%	0.0%	24.8%	0.0%	0.0%	0.0%	0.0%	0.0%
tr|F1S2A8|F1S2A8	2	0	4	0	0	0	0	0	2	0	9	0	0	0	0	0	11.8%	0.0%	16.3%	0.0%	0.0%	0.0%	0.0%	0.0%
tr|F1RMR5|F1RMR5	1	0	2	0	0	0	0	0	2	0	10	0	0	0	0	0	10.5%	0.0%	19.5%	0.0%	0.0%	0.0%	0.0%	0.0%
tr|Q06A99|Q06A99	1	1	2	0	0	0	0	0	1	1	10	0	0	0	0	0	1.8%	1.8%	4.3%	0.0%	0.0%	0.0%	0.0%	0.0%
tr|I3LSZ1|I3LSZ1	2	0	2	0	0	0	0	0	2	0	10	0	0	0	0	0	27.8%	0.0%	8.2%	0.0%	0.0%	0.0%	0.0%	0.0%
tr|K7GKR9|K7GKR9	0	1	2	0	0	0	0	0	0	2	11	0	0	0	0	0	0.0%	7.1%	11.7%	0.0%	0.0%	0.0%	0.0%	0.0%
tr|F1SQN5|F1SQN5	2	0	3	0	0	0	0	0	2	0	11	0	0	0	0	0	17.4%	0.0%	22.9%	0.0%	0.0%	0.0%	0.0%	0.0%
tr|K7GNR2|K7GNR2	0	1	2	0	0	0	0	0	0	2	11	0	0	0	0	0	0.0%	10.9%	17.8%	0.0%	0.0%	0.0%	0.0%	0.0%
tr|K7GQ39|K7GQ39	0	1	2	0	0	0	0	0	0	2	11	0	0	0	0	0	0.0%	13.9%	22.8%	0.0%	0.0%	0.0%	0.0%	0.0%
tr|I3LAQ2|I3LAQ2	2	0	3	0	0	0	0	0	2	0	11	0	0	0	0	0	17.4%	0.0%	22.9%	0.0%	0.0%	0.0%	0.0%	0.0%
tr|K7GL60|K7GL60	2	0	3	0	0	0	0	0	2	0	11	0	0	0	0	0	21.6%	0.0%	28.4%	0.0%	0.0%	0.0%	0.0%	0.0%
tr|K7GMF5|K7GMF5	0	1	2	0	0	0	0	0	0	2	11	0	0	0	0	0	0.0%	17.5%	28.8%	0.0%	0.0%	0.0%	0.0%	0.0%
tr|F1SNF6|F1SNF6	1	0	2	0	0	0	0	0	2	0	12	0	0	0	0	0	8.8%	0.0%	11.9%	0.0%	0.0%	0.0%	0.0%	0.0%
tr|F1RK10|F1RK10	1	1	2	0	0	0	0	0	1	1	16	0	0	0	0	0	5.6%	2.4%	7.8%	0.0%	0.0%	0.0%	0.0%	0.0%
tr|F1SM81|F1SM81	1	0	3	0	0	0	0	0	2	0	16	0	0	0	0	0	4.1%	0.0%	13.9%	0.0%	0.0%	0.0%	0.0%	0.0%
O97580	1	1	2	0	0	0	0	0	1	1	16	0	0	0	0	0	6.1%	2.6%	8.5%	0.0%	0.0%	0.0%	0.0%	0.0%
tr|F1RUT1|F1RUT1	2	0	3	0	0	0	0	0	2	0	18	0	0	0	0	0	11.2%	0.0%	10.7%	0.0%	0.0%	0.0%	0.0%	0.0%
tr|A6N8P5|A6N8P5	2	0	2	0	0	0	0	0	2	0	21	0	0	0	0	0	14.9%	0.0%	10.1%	0.0%	0.0%	0.0%	0.0%	0.0%
tr|I3LEL7|I3LEL7	1	0	2	0	0	0	0	0	2	0	23	0	0	0	0	0	14.3%	0.0%	25.5%	0.0%	0.0%	0.0%	0.0%	0.0%
tr|F1SLC2|F1SLC2	1	0	2	0	0	0	0	0	2	0	59	0	0	0	0	0	7.7%	0.0%	16.2%	0.0%	0.0%	0.0%	0.0%	0.0%
tr|F1RT53|F1RT53	1	0	0	0	0	0	2	10	1	0	0	0	0	0	6	71	3.9%	0.0%	0.0%	0.0%	0.0%	0.0%	8.6%	23.8%
tr|F1S2B2|F1S2B2	1	0	0	0	0	0	2	3	1	0	0	0	0	0	8	50	1.1%	0.0%	0.0%	0.0%	0.0%	0.0%	1.7%	4.1%
tr|I3LP99|I3LP99	1	0	0	0	0	0	1	1	1	0	0	0	0	0	35	19	14.5%	0.0%	0.0%	0.0%	0.0%	0.0%	14.5%	14.5%
tr|K7GR94|K7GR94	1	0	0	0	0	0	1	9	1	0	0	0	0	0	1	45	5.7%	0.0%	0.0%	0.0%	0.0%	0.0%	4.8%	37.2%
tr|F1RMT1|F1RMT1	1	0	0	0	0	0	1	9	1	0	0	0	0	0	1	45	4.9%	0.0%	0.0%	0.0%	0.0%	0.0%	4.1%	32.1%
tr|F1S4K9|F1S4K9	1	0	0	0	0	0	3	4	1	0	0	0	0	0	11	31	4.4%	0.0%	0.0%	0.0%	0.0%	0.0%	26.8%	34.9%
tr|I3LSX3|I3LSX3	1	0	0	0	0	0	0	6	1	0	0	0	0	0	0	38	4.5%	0.0%	0.0%	0.0%	0.0%	0.0%	0.0%	38.2%
tr|F1RIR2|F1RIR2	1	0	0	0	0	0	1	4	1	0	0	0	0	0	5	29	3.7%	0.0%	0.0%	0.0%	0.0%	0.0%	10.1%	19.2%
tr|F1SB08|F1SB08	1	0	0	0	0	0	2	5	1	0	0	0	0	0	5	29	0.3%	0.0%	0.0%	0.0%	0.0%	0.0%	2.8%	6.3%
tr|F1RMX5|F1RMX5	1	0	0	0	0	0	2	6	1	0	0	0	0	0	2	30	1.6%	0.0%	0.0%	0.0%	0.0%	0.0%	3.1%	12.2%
tr|I3LHI3|I3LHI3	1	0	0	0	0	0	1	1	1	0	0	0	0	0	5	25	6.7%	0.0%	0.0%	0.0%	0.0%	0.0%	6.7%	6.7%
tr|F1SIT8|F1SIT8	1	0	0	0	0	0	6	1	1	0	0	0	0	0	23	4	2.6%	0.0%	0.0%	0.0%	0.0%	0.0%	10.2%	1.7%
O02839	1	0	0	0	0	0	0	4	1	0	0	0	0	0	0	26	3.6%	0.0%	0.0%	0.0%	0.0%	0.0%	0.0%	16.3%
Q6PQZ1	1	0	1	0	0	0	2	6	1	0	1	0	0	0	14	32	12.2%	0.0%	7.4%	0.0%	0.0%	0.0%	8.1%	18.1%
tr|D3K5K6|D3K5K6	1	0	0	0	0	0	2	2	1	0	0	0	0	0	2	20	1.7%	0.0%	0.0%	0.0%	0.0%	0.0%	3.2%	2.7%
tr|I3L7R2|I3L7R2	1	0	0	0	0	0	1	2	1	0	0	0	0	0	1	20	5.1%	0.0%	0.0%	0.0%	0.0%	0.0%	5.1%	8.3%
tr|B6EUU6|B6EUU6	1	0	0	0	0	0	1	1	1	0	0	0	0	0	9	9	4.5%	0.0%	0.0%	0.0%	0.0%	0.0%	8.5%	4.5%
P23220	1	0	3	0	0	0	6	12	1	0	5	0	0	0	21	86	0.9%	0.0%	5.7%	0.0%	0.0%	0.0%	7.2%	16.9%
tr|F1SPZ4|F1SPZ4	1	0	3	0	0	0	6	11	1	0	5	0	0	0	21	82	1.0%	0.0%	6.4%	0.0%	0.0%	0.0%	8.0%	16.6%
tr|K7GSX5|K7GSX5	1	0	1	0	0	0	0	8	1	0	3	0	0	0	0	60	3.8%	0.0%	2.4%	0.0%	0.0%	0.0%	0.0%	18.9%
tr|I3LU77|I3LU77	1	0	0	0	0	0	0	5	1	0	0	0	0	0	0	13	2.1%	0.0%	0.0%	0.0%	0.0%	0.0%	0.0%	13.9%
tr|F1SIB1|F1SIB1	1	0	0	0	0	0	3	2	1	0	0	0	0	0	6	6	1.4%	0.0%	0.0%	0.0%	0.0%	0.0%	5.0%	2.6%
Q19AZ8	1	0	0	0	0	0	3	2	1	0	0	0	0	0	6	6	1.4%	0.0%	0.0%	0.0%	0.0%	0.0%	5.0%	2.6%
tr|F1RVJ7|F1RVJ7	0	1	0	0	0	0	0	4	0	1	0	0	0	0	0	10	0.0%	3.0%	0.0%	0.0%	0.0%	0.0%	0.0%	13.8%
tr|I3LD20|I3LD20	1	0	2	0	0	0	0	5	1	0	4	0	0	0	0	43	1.7%	0.0%	5.4%	0.0%	0.0%	0.0%	0.0%	9.0%
tr|F1RTL4|F1RTL4	1	0	1	0	0	0	1	4	1	0	1	0	0	0	1	16	3.8%	0.0%	3.3%	0.0%	0.0%	0.0%	2.5%	16.5%
tr|I3LM05|I3LM05	0	1	1	0	0	0	2	5	0	1	1	0	0	0	2	14	0.0%	5.1%	4.8%	0.0%	0.0%	0.0%	11.3%	27.5%
tr|F1STU6|F1STU6	1	0	0	0	0	0	1	1	1	0	0	0	0	0	1	7	10.2%	0.0%	0.0%	0.0%	0.0%	0.0%	5.1%	5.1%
tr|F1STL1|F1STL1	1	0	0	0	0	0	1	1	1	0	0	0	0	0	3	5	5.6%	0.0%	0.0%	0.0%	0.0%	0.0%	5.2%	5.2%
tr|I3LSL7|I3LSL7	1	0	0	0	0	0	0	1	1	0	0	0	0	0	0	7	12.4%	0.0%	0.0%	0.0%	0.0%	0.0%	0.0%	12.4%
tr|I3L9X0|I3L9X0	1	0	0	0	0	0	0	1	1	0	0	0	0	0	0	6	2.7%	0.0%	0.0%	0.0%	0.0%	0.0%	0.0%	2.7%
tr|F1RL45|F1RL45	1	0	0	0	0	0	0	1	1	0	0	0	0	0	0	6	7.0%	0.0%	0.0%	0.0%	0.0%	0.0%	0.0%	11.1%
tr|I3LQY3|I3LQY3	1	0	0	0	0	0	0	1	1	0	0	0	0	0	0	6	2.5%	0.0%	0.0%	0.0%	0.0%	0.0%	0.0%	2.5%
tr|F1SE25|F1SE25	1	0	0	0	0	0	0	2	1	0	0	0	0	0	0	6	7.6%	0.0%	0.0%	0.0%	0.0%	0.0%	0.0%	10.6%
tr|F1RMA9|F1RMA9	1	0	0	0	0	0	0	1	1	0	0	0	0	0	0	6	5.1%	0.0%	0.0%	0.0%	0.0%	0.0%	0.0%	5.1%
tr|F1RN75|F1RN75	1	0	0	0	0	0	0	1	1	0	0	0	0	0	0	6	4.6%	0.0%	0.0%	0.0%	0.0%	0.0%	0.0%	4.6%
tr|I3LHE4|I3LHE4	1	0	0	0	0	0	0	1	1	0	0	0	0	0	0	6	2.5%	0.0%	0.0%	0.0%	0.0%	0.0%	0.0%	2.5%
P80387	1	0	0	0	0	0	0	1	1	0	0	0	0	0	0	6	15.6%	0.0%	0.0%	0.0%	0.0%	0.0%	0.0%	24.6%
tr|K7GK81|K7GK81	1	0	0	0	0	0	0	1	1	0	0	0	0	0	0	6	5.1%	0.0%	0.0%	0.0%	0.0%	0.0%	0.0%	5.1%
tr|F1SJ46|F1SJ46	1	0	1	0	0	0	1	1	1	0	3	0	0	0	8	13	6.1%	0.0%	3.7%	0.0%	0.0%	0.0%	3.7%	3.7%
tr|I3L919|I3L919	1	0	0	0	0	0	0	2	1	0	0	0	0	0	0	5	16.9%	0.0%	0.0%	0.0%	0.0%	0.0%	0.0%	16.9%
tr|F1SF00|F1SF00	1	0	0	0	0	0	1	2	1	0	0	0	0	0	2	3	1.6%	0.0%	0.0%	0.0%	0.0%	0.0%	1.3%	4.3%
tr|F1SEC0|F1SEC0	1	0	0	0	0	0	1	1	1	0	0	0	0	0	4	1	8.6%	0.0%	0.0%	0.0%	0.0%	0.0%	8.6%	10.8%
tr|F1S8V1|F1S8V1	1	0	0	0	0	0	1	1	1	0	0	0	0	0	1	4	14.7%	0.0%	0.0%	0.0%	0.0%	0.0%	14.7%	14.7%
tr|I3LI68|I3LI68	1	0	0	0	0	0	0	1	1	0	0	0	0	0	0	5	3.2%	0.0%	0.0%	0.0%	0.0%	0.0%	0.0%	3.2%
tr|I3L7Z9|I3L7Z9	0	1	0	0	0	0	1	0	0	1	0	0	0	0	5	0	0.0%	3.7%	0.0%	0.0%	0.0%	0.0%	3.7%	0.0%
tr|F1SH47|F1SH47	1	0	0	0	0	0	0	4	1	0	0	0	0	0	0	5	1.5%	0.0%	0.0%	0.0%	0.0%	0.0%	0.0%	3.1%
tr|F1RU24|F1RU24	1	0	0	0	0	0	1	1	1	0	0	0	0	0	3	1	3.1%	0.0%	0.0%	0.0%	0.0%	0.0%	3.4%	3.1%
tr|K7GKZ4|K7GKZ4	1	0	0	0	0	0	0	2	1	0	0	0	0	0	0	4	1.2%	0.0%	0.0%	0.0%	0.0%	0.0%	0.0%	2.1%
tr|F2Z5L9|F2Z5L9	1	0	0	0	0	0	0	1	1	0	0	0	0	0	0	4	15.2%	0.0%	0.0%	0.0%	0.0%	0.0%	0.0%	15.2%
tr|F1SKS3|F1SKS3	1	0	0	0	0	0	0	2	1	0	0	0	0	0	0	4	1.2%	0.0%	0.0%	0.0%	0.0%	0.0%	0.0%	2.1%
tr|F2Z5V2|F2Z5V2	1	0	0	0	0	0	1	1	1	0	0	0	0	0	3	1	17.1%	0.0%	0.0%	0.0%	0.0%	0.0%	17.1%	9.2%
tr|I3LHE8|I3LHE8	1	0	0	0	0	0	1	1	1	0	0	0	0	0	3	1	8.4%	0.0%	0.0%	0.0%	0.0%	0.0%	8.4%	8.4%

	Peptide Count								Spectral Count								Sequence Coverage							
	Whole Cell	Whole Cell	Whole Cell	Cilia Fraction	Cilia Fraction	Cilia Fraction	Cilia Fraction	Cilia Fraction	Whole Cell	Whole Cell	Whole Cell	Cilia Fraction	Cilia Fraction	Cilia Fraction	Cilia Fraction	Cilia Fraction	Whole Cell	Whole Cell	Whole Cell	Cilia Fraction	Cilia Fraction	Cilia Fraction	Cilia Fraction	Cilia Fraction
Sample#	1	2	6	4	3	5	7	8	1	2	6	4	3	5	7	8	1	2	6	4	3	5	7	8
**Accession**																								
tr|I3L6A7|I3L6A7	1	0	1	0	0	0	0	2	1	0	1	0	0	0	0	6	7.8%	0.0%	7.0%	0.0%	0.0%	0.0%	0.0%	18.4%
Q35916	1	0	0	0	0	0	0	1	1	0	0	0	0	0	0	3	5.4%	0.0%	0.0%	0.0%	0.0%	0.0%	0.0%	5.4%
tr|F1RIA5|F1RIA5	1	0	0	0	0	0	0	1	1	0	0	0	0	0	0	3	5.3%	0.0%	0.0%	0.0%	0.0%	0.0%	0.0%	5.3%
tr|F1RG61|F1RG61	1	0	0	0	0	0	0	1	1	0	0	0	0	0	0	3	2.3%	0.0%	0.0%	0.0%	0.0%	0.0%	0.0%	3.4%
tr|I3LEN0|I3LEN0	1	0	0	0	0	0	1	1	1	0	0	0	0	0	2	1	35.1%	0.0%	0.0%	0.0%	0.0%	0.0%	35.1%	35.1%
tr|I3LP32|I3LP32	1	0	0	0	0	0	0	1	1	0	0	0	0	0	0	3	21.6%	0.0%	0.0%	0.0%	0.0%	0.0%	0.0%	16.5%
tr|K7GSJ4|K7GSJ4	1	0	1	0	0	0	0	2	1	0	3	0	0	0	0	10	33.6%	0.0%	19.3%	0.0%	0.0%	0.0%	0.0%	33.6%
tr|K7GLY9|K7GLY9	1	0	1	0	0	0	0	3	1	0	5	0	0	0	0	14	14.0%	0.0%	8.4%	0.0%	0.0%	0.0%	0.0%	36.5%
tr|F1SR85|F1SR85	1	0	1	0	0	0	0	4	1	0	2	0	0	0	0	7	2.6%	0.0%	3.1%	0.0%	0.0%	0.0%	0.0%	5.0%
tr|K7GM94|K7GM94	1	0	1	0	0	0	0	3	1	0	4	0	0	0	0	10	9.1%	0.0%	11.0%	0.0%	0.0%	0.0%	0.0%	14.0%
tr|F1SQU2|F1SQU2	1	0	1	0	0	0	1	1	1	0	1	0	0	0	1	3	4.0%	0.0%	4.0%	0.0%	0.0%	0.0%	5.5%	5.5%
tr|F1SKS4|F1SKS4	1	0	0	0	0	0	0	2	1	0	0	0	0	0	0	2	5.5%	0.0%	0.0%	0.0%	0.0%	0.0%	0.0%	16.7%
tr|F1SRN4|F1SRN4	1	0	0	0	0	0	0	1	1	0	0	0	0	0	0	2	2.2%	0.0%	0.0%	0.0%	0.0%	0.0%	0.0%	1.8%
tr|I3L8J2|I3L8J2	1	0	0	0	0	0	0	1	1	0	0	0	0	0	0	2	8.1%	0.0%	0.0%	0.0%	0.0%	0.0%	0.0%	12.8%
tr|I3LIV0|I3LIV0	1	0	0	0	0	0	0	1	1	0	0	0	0	0	0	2	8.1%	0.0%	0.0%	0.0%	0.0%	0.0%	0.0%	12.8%
tr|I3LG98|I3LG98	1	0	0	0	0	0	0	1	1	0	0	0	0	0	0	2	6.6%	0.0%	0.0%	0.0%	0.0%	0.0%	0.0%	9.8%
tr|F1RPP6|F1RPP6	1	0	0	0	0	0	0	1	1	0	0	0	0	0	0	2	17.4%	0.0%	0.0%	0.0%	0.0%	0.0%	0.0%	17.4%
tr|F2Z4Z5|F2Z4Z5	1	0	0	0	0	0	0	1	1	0	0	0	0	0	0	2	5.6%	0.0%	0.0%	0.0%	0.0%	0.0%	0.0%	8.5%
tr|F1RKE8|F1RKE8	1	0	0	0	0	0	0	2	1	0	0	0	0	0	0	2	5.6%	0.0%	0.0%	0.0%	0.0%	0.0%	0.0%	11.7%
tr|F1SMV1|F1SMV1	1	0	0	0	0	0	0	1	1	0	0	0	0	0	0	2	5.6%	0.0%	0.0%	0.0%	0.0%	0.0%	0.0%	5.6%
tr|F1S2V7|F1S2V7	0	1	0	0	0	0	0	1	0	1	0	0	0	0	0	2	0.0%	3.3%	0.0%	0.0%	0.0%	0.0%	0.0%	3.1%
tr|I3L5H9|I3L5H9	1	0	2	0	0	0	1	2	1	0	2	0	0	0	3	2	1.8%	0.0%	2.5%	0.0%	0.0%	0.0%	2.4%	5.1%
tr|F1SA61|F1SA61	1	0	2	0	0	0	1	2	1	0	2	0	0	0	3	2	2.0%	0.0%	2.7%	0.0%	0.0%	0.0%	2.6%	5.6%
tr|F1RMA7|F1RMA7	1	0	1	0	0	0	1	1	1	0	1	0	0	0	1	2	5.8%	0.0%	2.8%	0.0%	0.0%	0.0%	5.8%	5.8%
tr|K7GMT0|K7GMT0	0	1	1	0	0	0	0	1	0	1	1	0	0	0	0	3	0.0%	2.4%	1.9%	0.0%	0.0%	0.0%	0.0%	4.5%
tr|Q9GJV4|Q9GJV4	0	1	1	0	0	0	1	1	0	1	2	0	0	0	3	1	0.0%	3.4%	5.1%	0.0%	0.0%	0.0%	8.8%	4.4%
tr|F1SGD2|F1SGD2	1	0	1	0	0	0	1	1	1	0	2	0	0	0	2	2	1.7%	0.0%	2.8%	0.0%	0.0%	0.0%	1.7%	1.7%
tr|K7GS06|K7GS06	0	1	1	0	0	0	1	1	0	1	2	0	0	0	3	1	0.0%	3.6%	5.4%	0.0%	0.0%	0.0%	9.3%	4.6%
tr|F1SA12|F1SA12	1	0	1	0	0	0	1	1	1	0	8	0	0	0	2	8	9.0%	0.0%	9.0%	0.0%	0.0%	0.0%	9.0%	9.0%
A7WLI0	1	0	2	0	0	0	1	1	1	0	10	0	0	0	9	3	14.7%	0.0%	14.7%	0.0%	0.0%	0.0%	14.7%	14.7%
tr|I3L5X4|I3L5X4	1	0	1	0	0	0	0	1	1	0	3	0	0	0	0	4	34.5%	0.0%	41.4%	0.0%	0.0%	0.0%	0.0%	17.2%
Q66X52	1	0	1	0	0	0	0	1	1	0	3	0	0	0	0	4	22.0%	0.0%	26.4%	0.0%	0.0%	0.0%	0.0%	11.0%
tr|F1S622|F1S622	1	0	1	0	0	0	0	1	1	0	3	0	0	0	0	4	22.5%	0.0%	27.0%	0.0%	0.0%	0.0%	0.0%	11.2%
tr|I3LK99|I3LK99	1	0	1	0	0	0	0	1	1	0	1	0	0	0	0	2	11.0%	0.0%	9.9%	0.0%	0.0%	0.0%	0.0%	11.0%
tr|F1S611|F1S611	1	0	1	0	0	0	0	1	1	0	1	0	0	0	0	2	4.5%	0.0%	4.1%	0.0%	0.0%	0.0%	0.0%	4.5%
tr|I3L629|I3L629	1	0	0	0	0	0	0	1	1	0	0	0	0	0	0	1	2.0%	0.0%	0.0%	0.0%	0.0%	0.0%	0.0%	2.0%
tr|F1SFK9|F1SFK9	1	0	0	0	0	0	0	1	1	0	0	0	0	0	0	1	2.6%	0.0%	0.0%	0.0%	0.0%	0.0%	0.0%	3.2%
tr|K7GSH5|K7GSH5	1	0	0	0	0	0	0	1	1	0	0	0	0	0	0	1	3.9%	0.0%	0.0%	0.0%	0.0%	0.0%	0.0%	7.0%
tr|I3LBN9|I3LBN9	0	1	0	0	0	0	1	0	0	1	0	0	0	0	1	0	0.0%	3.2%	0.0%	0.0%	0.0%	0.0%	5.7%	0.0%
tr|F1S7Q6|F1S7Q6	1	0	0	0	0	0	0	1	1	0	0	0	0	0	0	1	3.3%	0.0%	0.0%	0.0%	0.0%	0.0%	0.0%	2.3%
tr|F1SH88|F1SH88	1	0	0	0	0	0	1	0	1	0	0	0	0	0	1	0	12.5%	0.0%	0.0%	0.0%	0.0%	0.0%	15.1%	0.0%
tr|I3LDB1|I3LDB1	1	0	0	0	0	0	0	1	1	0	0	0	0	0	0	1	0.7%	0.0%	0.0%	0.0%	0.0%	0.0%	0.0%	1.1%
tr|I3LMX1|I3LMX1	1	0	0	0	0	0	0	1	1	0	0	0	0	0	0	1	5.5%	0.0%	0.0%	0.0%	0.0%	0.0%	0.0%	7.0%
Q95313	1	0	0	0	0	0	0	1	1	0	0	0	0	0	0	1	9.8%	0.0%	0.0%	0.0%	0.0%	0.0%	0.0%	9.8%
tr|I3LSR1|I3LSR1	1	0	0	0	0	0	1	0	1	0	0	0	0	0	1	0	7.4%	0.0%	0.0%	0.0%	0.0%	0.0%	10.7%	0.0%
tr|F1RS74|F1RS74	0	1	0	0	0	0	0	1	0	1	0	0	0	0	0	1	0.0%	1.5%	0.0%	0.0%	0.0%	0.0%	0.0%	4.1%
Q5PXT2	1	0	0	0	0	0	0	1	1	0	0	0	0	0	0	1	2.5%	0.0%	0.0%	0.0%	0.0%	0.0%	0.0%	3.0%
tr|K7GPB9|K7GPB9	1	0	0	0	0	0	0	1	1	0	0	0	0	0	0	1	4.0%	0.0%	0.0%	0.0%	0.0%	0.0%	0.0%	7.1%
tr|F1S4D4|F1S4D4	1	0	0	0	0	0	0	1	1	0	0	0	0	0	0	1	2.7%	0.0%	0.0%	0.0%	0.0%	0.0%	0.0%	2.2%
tr|F1SCN1|F1SCN1	1	0	0	0	0	0	0	1	1	0	0	0	0	0	0	1	6.6%	0.0%	0.0%	0.0%	0.0%	0.0%	0.0%	6.6%
tr|F1S0E8|F1S0E8	1	0	0	0	0	0	1	0	1	0	0	0	0	0	1	0	2.1%	0.0%	0.0%	0.0%	0.0%	0.0%	2.8%	0.0%
Reverse_tr|F1S4W1|F1S4W1	1	0	0	0	0	0	1	0	1	0	0	0	0	0	1	0	1.0%	0.0%	0.0%	0.0%	0.0%	0.0%	1.0%	0.0%
tr|I3LV53|I3LV53	1	0	0	0	0	0	0	1	1	0	0	0	0	0	0	1	2.8%	0.0%	0.0%	0.0%	0.0%	0.0%	0.0%	2.8%
tr|A5GFW3|A5GFW3	1	0	0	0	0	0	0	1	1	0	0	0	0	0	0	1	5.6%	0.0%	0.0%	0.0%	0.0%	0.0%	0.0%	2.8%
tr|F1RGI2|F1RGI2	0	1	0	0	0	0	0	1	0	1	0	0	0	0	0	1	0.0%	3.9%	0.0%	0.0%	0.0%	0.0%	0.0%	3.9%
tr|I3L9F0|I3L9F0	1	0	0	0	0	0	0	1	1	0	0	0	0	0	0	1	2.8%	0.0%	0.0%	0.0%	0.0%	0.0%	0.0%	2.8%
tr|K7GMU8|K7GMU8	1	0	0	0	0	0	0	1	1	0	0	0	0	0	0	1	3.4%	0.0%	0.0%	0.0%	0.0%	0.0%	0.0%	6.1%
tr|F1SLF9|F1SLF9	1	0	0	0	0	0	0	1	1	0	0	0	0	0	0	1	2.8%	0.0%	0.0%	0.0%	0.0%	0.0%	0.0%	4.7%
tr|F1S1Z3|F1S1Z3	0	1	0	0	0	0	1	0	0	1	0	0	0	0	1	0	0.0%	6.2%	0.0%	0.0%	0.0%	0.0%	6.2%	0.0%
tr|I3LPH6|I3LPH6	1	0	0	0	0	0	0	1	1	0	0	0	0	0	0	1	2.7%	0.0%	0.0%	0.0%	0.0%	0.0%	0.0%	2.7%
tr|F1SH49|F1SH49	0	1	0	0	0	0	0	1	0	1	0	0	0	0	0	1	0.0%	4.4%	0.0%	0.0%	0.0%	0.0%	0.0%	4.4%
tr|F1RKG7|F1RKG7	1	0	0	0	0	0	0	1	1	0	0	0	0	0	0	1	5.6%	0.0%	0.0%	0.0%	0.0%	0.0%	0.0%	5.6%
tr|I3LKX9|I3LKX9	1	0	0	0	0	0	0	1	1	0	0	0	0	0	0	1	1.5%	0.0%	0.0%	0.0%	0.0%	0.0%	0.0%	2.2%
tr|F1SU28|F1SU28	1	0	0	0	0	0	1	0	1	0	0	0	0	0	1	0	3.1%	0.0%	0.0%	0.0%	0.0%	0.0%	4.6%	0.0%
tr|F1RS65|F1RS65	0	1	0	0	0	0	0	1	0	1	0	0	0	0	0	1	0.0%	1.5%	0.0%	0.0%	0.0%	0.0%	0.0%	4.2%
tr|F1S405|F1S405	1	0	0	0	0	0	0	1	1	0	0	0	0	0	0	1	1.0%	0.0%	0.0%	0.0%	0.0%	0.0%	0.0%	1.0%
tr|I3LS76|I3LS76	1	0	0	0	0	0	0	1	1	0	0	0	0	0	0	1	12.8%	0.0%	0.0%	0.0%	0.0%	0.0%	0.0%	25.6%
tr|I3LEG2|I3LEG2	1	0	0	0	0	0	0	1	1	0	0	0	0	0	0	1	4.6%	0.0%	0.0%	0.0%	0.0%	0.0%	0.0%	4.2%
tr|F1SFH0|F1SFH0	1	0	0	0	0	0	1	0	1	0	0	0	0	0	1	0	2.8%	0.0%	0.0%	0.0%	0.0%	0.0%	3.7%	0.0%
tr|F1SKI4|F1SKI4	1	0	0	0	0	0	0	1	1	0	0	0	0	0	0	1	4.0%	0.0%	0.0%	0.0%	0.0%	0.0%	0.0%	4.0%
tr|F1RP13|F1RP13	1	0	0	0	0	0	0	1	1	0	0	0	0	0	0	1	10.0%	0.0%	0.0%	0.0%	0.0%	0.0%	0.0%	6.0%
tr|F1RKE4|F1RKE4	1	0	0	0	0	0	0	1	1	0	0	0	0	0	0	1	15.0%	0.0%	0.0%	0.0%	0.0%	0.0%	0.0%	30.0%
tr|I3LA12|I3LA12	1	0	0	0	0	0	0	1	1	0	0	0	0	0	0	1	2.8%	0.0%	0.0%	0.0%	0.0%	0.0%	0.0%	2.8%
tr|F1RP42|F1RP42	0	1	2	0	0	0	1	2	0	1	11	0	0	0	4	6	0.0%	5.6%	5.0%	0.0%	0.0%	0.0%	4.4%	5.0%
tr|F1S2I8|F1S2I8	1	0	1	0	0	0	0	1	1	0	4	0	0	0	0	4	14.0%	0.0%	14.0%	0.0%	0.0%	0.0%	0.0%	14.0%
tr|F1SV88|F1SV88	1	0	6	0	0	0	1	3	1	0	52	0	0	0	6	36	7.5%	0.0%	53.3%	0.0%	0.0%	0.0%	20.1%	22.1%
tr|K7GS63|K7GS63	1	0	1	0	0	0	0	1	1	0	2	0	0	0	0	2	9.0%	0.0%	6.2%	0.0%	0.0%	0.0%	0.0%	9.0%
tr|I3LM99|I3LM99	1	0	1	0	0	0	0	1	1	0	2	0	0	0	0	2	8.8%	0.0%	6.0%	0.0%	0.0%	0.0%	0.0%	8.8%
tr|F1RQG9|F1RQG9	1	0	1	0	0	0	0	1	1	0	2	0	0	0	0	2	5.4%	0.0%	5.2%	0.0%	0.0%	0.0%	0.0%	5.2%
tr|F1S416|F1S416	1	0	2	0	0	0	0	1	1	0	2	0	0	0	0	2	6.6%	0.0%	18.2%	0.0%	0.0%	0.0%	0.0%	6.6%
tr|F2Z516|F2Z516	1	0	1	0	0	0	0	2	1	0	7	0	0	0	0	4	13.7%	0.0%	13.7%	0.0%	0.0%	0.0%	0.0%	39.5%

	Peptide Count								Spectral Count								Sequence Coverage							
	Whole Cell	Whole Cell	Whole Cell	Cilia Fraction	Cilia Fraction	Cilia Fraction	Cilia Fraction	Cilia Fraction	Whole Cell	Whole Cell	Whole Cell	Cilia Fraction	Cilia Fraction	Cilia Fraction	Cilia Fraction	Cilia Fraction	Whole Cell	Whole Cell	Whole Cell	Cilia Fraction	Cilia Fraction	Cilia Fraction	Cilia Fraction	Cilia Fraction
Sample#	1	2	6	4	3	5	7	8	1	2	6	4	3	5	7	8	1	2	6	4	3	5	7	8
**Accession**																								
tr|I3LEH0|I3LEH0	1	0	1	0	0	0	0	2	1	0	7	0	0	0	0	4	13.6%	0.0%	13.6%	0.0%	0.0%	0.0%	0.0%	39.2%
tr|I3LTZ1|I3LTZ1	1	0	1	0	0	0	0	1	1	0	1	0	0	0	0	1	9.7%	0.0%	9.7%	0.0%	0.0%	0.0%	0.0%	9.7%
tr|F1S9R6|F1S9R6	0	1	1	0	0	0	0	1	0	1	1	0	0	0	0	1	0.0%	1.0%	2.2%	0.0%	0.0%	0.0%	0.0%	1.7%
tr|I3LK37|I3LK37	1	0	1	0	0	0	0	1	1	0	4	0	0	0	0	2	3.2%	0.0%	3.6%	0.0%	0.0%	0.0%	0.0%	2.0%
tr|F1RIZ9|F1RIZ9	1	0	1	0	0	0	0	1	1	0	4	0	0	0	0	2	2.9%	0.0%	3.3%	0.0%	0.0%	0.0%	0.0%	1.8%
tr|F1S0R4|F1S0R4	1	0	1	0	0	0	0	1	1	0	5	0	0	0	0	2	6.8%	0.0%	4.7%	0.0%	0.0%	0.0%	0.0%	8.6%
tr|I3LT98|I3LT98	1	0	1	0	0	0	0	1	1	0	2	0	0	0	0	1	30.1%	0.0%	11.8%	0.0%	0.0%	0.0%	0.0%	30.1%
tr|F1RJA8|F1RJA8	1	0	1	0	0	0	0	1	1	0	2	0	0	0	0	1	7.2%	0.0%	8.7%	0.0%	0.0%	0.0%	0.0%	15.5%
tr|I3L8E5|I3L8E5	1	0	3	0	0	0	0	1	1	0	11	0	0	0	0	3	8.2%	0.0%	11.9%	0.0%	0.0%	0.0%	0.0%	6.0%
tr|I3LCS2|I3LCS2	1	0	3	0	0	0	0	1	1	0	11	0	0	0	0	3	8.1%	0.0%	11.8%	0.0%	0.0%	0.0%	0.0%	5.9%
tr|I3LH45|I3LH45	1	0	3	0	0	0	0	1	1	0	11	0	0	0	0	3	8.2%	0.0%	11.9%	0.0%	0.0%	0.0%	0.0%	6.0%
tr|F1RI18|F1RI18	1	0	1	0	0	0	0	1	1	0	3	0	0	0	0	1	15.9%	0.0%	15.9%	0.0%	0.0%	0.0%	0.0%	15.9%
tr|I3LC25|I3LC25	1	0	2	0	0	0	0	1	1	0	3	0	0	0	0	1	1.6%	0.0%	4.0%	0.0%	0.0%	0.0%	0.0%	4.0%
tr|F1RX12|F1RX12	1	0	4	0	0	0	0	1	1	0	8	0	0	0	0	2	3.1%	0.0%	14.9%	0.0%	0.0%	0.0%	0.0%	5.7%
tr|F1STT2|F1STT2	1	0	6	0	0	0	0	3	1	0	23	0	0	0	0	5	2.3%	0.0%	8.3%	0.0%	0.0%	0.0%	0.0%	6.1%
tr|F1S226|F1S226	1	0	1	0	0	0	0	1	1	0	9	0	0	0	0	2	7.4%	0.0%	7.4%	0.0%	0.0%	0.0%	0.0%	7.4%
tr|I3LPY1|I3LPY1	1	0	2	0	0	0	0	1	1	0	10	0	0	0	0	2	5.6%	0.0%	6.9%	0.0%	0.0%	0.0%	0.0%	5.6%
tr|I3LHA6|I3LHA6	1	0	1	0	0	0	1	0	1	0	10	0	0	0	2	0	9.6%	0.0%	11.2%	0.0%	0.0%	0.0%	14.4%	0.0%
tr|F1SFF3|F1SFF3	1	0	6	0	0	0	0	2	1	0	17	0	0	0	0	3	1.5%	0.0%	6.5%	0.0%	0.0%	0.0%	0.0%	3.1%
tr|I3LDJ8|I3LDJ8	1	0	1	0	0	0	0	1	1	0	12	0	0	0	0	2	3.0%	0.0%	3.0%	0.0%	0.0%	0.0%	0.0%	3.0%
tr|I3L811|I3L811	1	0	1	0	0	0	0	1	1	0	12	0	0	0	0	2	10.9%	0.0%	12.2%	0.0%	0.0%	0.0%	0.0%	12.2%
tr|F1S2F9|F1S2F9	1	0	1	0	0	0	0	1	1	0	6	0	0	0	0	1	17.1%	0.0%	15.7%	0.0%	0.0%	0.0%	0.0%	15.7%
tr|F2Z4X6|F2Z4X6	1	0	1	0	0	0	0	1	1	0	6	0	0	0	0	1	13.7%	0.0%	8.2%	0.0%	0.0%	0.0%	0.0%	8.2%
tr|F1RSR5|F1RSR5	1	0	2	0	0	0	0	1	1	0	7	0	0	0	0	1	1.1%	0.0%	3.3%	0.0%	0.0%	0.0%	0.0%	1.1%
tr|I3L5P8|I3L5P8	1	0	1	0	0	0	0	1	1	0	7	0	0	0	0	1	9.2%	0.0%	9.2%	0.0%	0.0%	0.0%	0.0%	9.2%
tr|I3LHA4|I3LHA4	1	0	1	0	0	0	0	1	1	0	7	0	0	0	0	1	17.5%	0.0%	17.5%	0.0%	0.0%	0.0%	0.0%	17.5%
tr|I3LDA0|I3LDA0	1	0	2	0	0	0	1	0	1	0	13	0	0	0	1	0	20.0%	0.0%	20.0%	0.0%	0.0%	0.0%	20.0%	0.0%
tr|A5GHK3|A5GHK3	1	0	1	0	0	0	1	1	1	0	29	0	0	0	1	1	9.0%	0.0%	11.2%	0.0%	0.0%	0.0%	11.2%	8.2%
tr|F1S4S1|F1S4S1	1	0	5	0	0	0	0	1	1	0	23	0	0	0	0	1	3.2%	0.0%	13.9%	0.0%	0.0%	0.0%	0.0%	4.6%
tr|I3L5L2|I3L5L2	1	0	1	0	0	0	1	0	1	0	105	0	0	0	1	0	19.7%	0.0%	19.7%	0.0%	0.0%	0.0%	19.7%	0.0%
tr|A5A8X7|A5A8X7	1	0	0	0	0	0	0	0	1	0	0	0	0	0	0	0	3.0%	0.0%	0.0%	0.0%	0.0%	0.0%	0.0%	0.0%
tr|K7GNQ9|K7GNQ9	1	0	0	0	0	0	0	0	1	0	0	0	0	0	0	0	14.2%	0.0%	0.0%	0.0%	0.0%	0.0%	0.0%	0.0%
tr|F1RTC7|F1RTC7	1	0	0	0	0	0	0	0	1	0	0	0	0	0	0	0	12.4%	0.0%	0.0%	0.0%	0.0%	0.0%	0.0%	0.0%
tr|F2Z5K0|F2Z5K0	1	0	0	0	0	0	0	0	1	0	0	0	0	0	0	0	7.3%	0.0%	0.0%	0.0%	0.0%	0.0%	0.0%	0.0%
tr|I3L7Q4|I3L7Q4	1	0	0	0	0	0	0	0	1	0	0	0	0	0	0	0	1.8%	0.0%	0.0%	0.0%	0.0%	0.0%	0.0%	0.0%
tr|F1S1M1|F1S1M1	1	0	0	0	0	0	0	0	1	0	0	0	0	0	0	0	3.2%	0.0%	0.0%	0.0%	0.0%	0.0%	0.0%	0.0%
tr|F1SQ75|F1SQ75	1	0	0	0	0	0	0	0	1	0	0	0	0	0	0	0	3.5%	0.0%	0.0%	0.0%	0.0%	0.0%	0.0%	0.0%
tr|I3LH51|I3LH51	0	1	0	0	0	0	0	0	0	1	0	0	0	0	0	0	0.0%	2.3%	0.0%	0.0%	0.0%	0.0%	0.0%	0.0%
tr|K7N7E1|K7N7E1	1	0	0	0	0	0	0	0	1	0	0	0	0	0	0	0	4.7%	0.0%	0.0%	0.0%	0.0%	0.0%	0.0%	0.0%
tr|F1SK43|F1SK43	0	1	0	0	0	0	0	0	0	1	0	0	0	0	0	0	0.0%	3.7%	0.0%	0.0%	0.0%	0.0%	0.0%	0.0%
tr|I3L7C3|I3L7C3	0	1	0	0	0	0	0	0	0	1	0	0	0	0	0	0	0.0%	4.9%	0.0%	0.0%	0.0%	0.0%	0.0%	0.0%
P28220	0	1	0	0	0	0	0	0	0	1	0	0	0	0	0	0	0.0%	5.7%	0.0%	0.0%	0.0%	0.0%	0.0%	0.0%
tr|I3LCE2|I3LCE2	0	1	0	0	0	0	0	0	0	1	0	0	0	0	0	0	0.0%	3.8%	0.0%	0.0%	0.0%	0.0%	0.0%	0.0%
tr|F1S0X6|F1S0X6	1	0	0	0	0	0	0	0	1	0	0	0	0	0	0	0	9.0%	0.0%	0.0%	0.0%	0.0%	0.0%	0.0%	0.0%
tr|F1SJ72|F1SJ72	1	0	0	0	0	0	0	0	1	0	0	0	0	0	0	0	8.0%	0.0%	0.0%	0.0%	0.0%	0.0%	0.0%	0.0%
tr|K7GKU5|K7GKU5	1	0	0	0	0	0	0	0	1	0	0	0	0	0	0	0	13.1%	0.0%	0.0%	0.0%	0.0%	0.0%	0.0%	0.0%
tr|K7GPA6|K7GPA6	0	1	0	0	0	0	0	0	0	1	0	0	0	0	0	0	0.0%	3.3%	0.0%	0.0%	0.0%	0.0%	0.0%	0.0%
tr|F1S2Y4|F1S2Y4	1	0	0	0	0	0	0	0	1	0	0	0	0	0	0	0	1.9%	0.0%	0.0%	0.0%	0.0%	0.0%	0.0%	0.0%
tr|F1SUV0|F1SUV0	0	1	0	0	0	0	0	0	0	1	0	0	0	0	0	0	0.0%	1.8%	0.0%	0.0%	0.0%	0.0%	0.0%	0.0%
tr|I3L5K3|I3L5K3	0	1	0	0	0	0	0	0	0	1	0	0	0	0	0	0	0.0%	4.5%	0.0%	0.0%	0.0%	0.0%	0.0%	0.0%
tr|I3LKY7|I3LKY7	1	0	0	0	0	0	0	0	1	0	0	0	0	0	0	0	1.8%	0.0%	0.0%	0.0%	0.0%	0.0%	0.0%	0.0%
tr|F1RM25|F1RM25	1	0	0	0	0	0	0	0	1	0	0	0	0	0	0	0	2.2%	0.0%	0.0%	0.0%	0.0%	0.0%	0.0%	0.0%
tr|F1SCY3|F1SCY3	1	0	0	0	0	0	0	0	1	0	0	0	0	0	0	0	2.4%	0.0%	0.0%	0.0%	0.0%	0.0%	0.0%	0.0%
tr|F1RME0|F1RME0	0	1	0	0	0	0	0	0	0	1	0	0	0	0	0	0	0.0%	5.3%	0.0%	0.0%	0.0%	0.0%	0.0%	0.0%
tr|F1SL67|F1SL67	1	0	0	0	0	0	0	0	1	0	0	0	0	0	0	0	4.8%	0.0%	0.0%	0.0%	0.0%	0.0%	0.0%	0.0%
tr|F1SFD4|F1SFD4	1	0	0	0	0	0	0	0	1	0	0	0	0	0	0	0	2.3%	0.0%	0.0%	0.0%	0.0%	0.0%	0.0%	0.0%
tr|F6Q8T7|F6Q8T7	1	0	0	0	0	0	0	0	1	0	0	0	0	0	0	0	10.9%	0.0%	0.0%	0.0%	0.0%	0.0%	0.0%	0.0%
tr|A5GHK9|A5GHK9	0	1	0	0	0	0	0	0	0	1	0	0	0	0	0	0	0.0%	2.6%	0.0%	0.0%	0.0%	0.0%	0.0%	0.0%
tr|F1SET4|F1SET4	1	0	0	0	0	0	0	0	1	0	0	0	0	0	0	0	9.3%	0.0%	0.0%	0.0%	0.0%	0.0%	0.0%	0.0%
tr|K7GQ72|K7GQ72	0	1	0	0	0	0	0	0	0	1	0	0	0	0	0	0	0.0%	2.5%	0.0%	0.0%	0.0%	0.0%	0.0%	0.0%
tr|F1RNC7|F1RNC7	0	1	0	0	0	0	0	0	0	1	0	0	0	0	0	0	0.0%	0.6%	0.0%	0.0%	0.0%	0.0%	0.0%	0.0%
Reverse_tr|F1SVD4|F1SVD4	1	0	0	0	0	0	0	0	1	0	0	0	0	0	0	0	6.7%	0.0%	0.0%	0.0%	0.0%	0.0%	0.0%	0.0%
tr|I3LPS1|I3LPS1	1	0	0	0	0	0	0	0	1	0	0	0	0	0	0	0	5.7%	0.0%	0.0%	0.0%	0.0%	0.0%	0.0%	0.0%
tr|F1SNX7|F1SNX7	0	1	0	0	0	0	0	0	0	1	0	0	0	0	0	0	0.0%	1.3%	0.0%	0.0%	0.0%	0.0%	0.0%	0.0%
tr|F1RQZ4|F1RQZ4	1	0	0	0	0	0	0	0	1	0	0	0	0	0	0	0	4.0%	0.0%	0.0%	0.0%	0.0%	0.0%	0.0%	0.0%
tr|I3LAX0|I3LAX0	1	0	0	0	0	0	0	0	1	0	0	0	0	0	0	0	6.5%	0.0%	0.0%	0.0%	0.0%	0.0%	0.0%	0.0%
tr|I3LAQ3|I3LAQ3	0	1	0	0	0	0	0	0	0	1	0	0	0	0	0	0	0.0%	3.8%	0.0%	0.0%	0.0%	0.0%	0.0%	0.0%
tr|I3LMC2|I3LMC2	1	0	0	0	0	0	0	0	1	0	0	0	0	0	0	0	2.2%	0.0%	0.0%	0.0%	0.0%	0.0%	0.0%	0.0%
tr|I3LK73|I3LK73	1	0	0	0	0	0	0	0	1	0	0	0	0	0	0	0	0.4%	0.0%	0.0%	0.0%	0.0%	0.0%	0.0%	0.0%
tr|F1SSF4|F1SSF4	1	0	0	0	0	0	0	0	1	0	0	0	0	0	0	0	10.4%	0.0%	0.0%	0.0%	0.0%	0.0%	0.0%	0.0%
Reverse_tr|F1RYX0|F1RYX0	0	1	0	0	0	0	0	0	0	1	0	0	0	0	0	0	0.0%	8.7%	0.0%	0.0%	0.0%	0.0%	0.0%	0.0%
Q0GFF6	1	0	0	0	0	0	0	0	1	0	0	0	0	0	0	0	1.9%	0.0%	0.0%	0.0%	0.0%	0.0%	0.0%	0.0%
tr|I3LUF1|I3LUF1	1	0	0	0	0	0	0	0	1	0	0	0	0	0	0	0	3.3%	0.0%	0.0%	0.0%	0.0%	0.0%	0.0%	0.0%
tr|K7GT56|K7GT56	1	0	0	0	0	0	0	0	1	0	0	0	0	0	0	0	69.8%	0.0%	0.0%	0.0%	0.0%	0.0%	0.0%	0.0%
tr|I3LN16|I3LN16	0	1	0	0	0	0	0	0	0	1	0	0	0	0	0	0	0.0%	7.6%	0.0%	0.0%	0.0%	0.0%	0.0%	0.0%
tr|F1RSN0|F1RSN0	1	0	0	0	0	0	0	0	1	0	0	0	0	0	0	0	4.0%	0.0%	0.0%	0.0%	0.0%	0.0%	0.0%	0.0%
Reverse_tr|I3LJS9|I3LJS9	1	0	0	0	0	0	0	0	1	0	0	0	0	0	0	0	1.8%	0.0%	0.0%	0.0%	0.0%	0.0%	0.0%	0.0%
tr|F1RN67|F1RN67	1	0	0	0	0	0	0	0	1	0	0	0	0	0	0	0	9.2%	0.0%	0.0%	0.0%	0.0%	0.0%	0.0%	0.0%
tr|I3LL66|I3LL66	1	0	0	0	0	0	0	0	1	0	0	0	0	0	0	0	25.2%	0.0%	0.0%	0.0%	0.0%	0.0%	0.0%	0.0%
tr|F1RWZ2|F1RWZ2	0	1	0	0	0	0	0	0	0	1	0	0	0	0	0	0	0.0%	4.3%	0.0%	0.0%	0.0%	0.0%	0.0%	0.0%
Reverse_tr|F2Z5S8|F2Z5S8	0	1	0	0	0	0	0	0	0	1	0	0	0	0	0	0	0.0%	2.9%	0.0%	0.0%	0.0%	0.0%	0.0%	0.0%

	Peptide Count								Spectral Count								Sequence Coverage							
	Whole Cell	Whole Cell	Whole Cell	Cilia Fraction	Cilia Fraction	Cilia Fraction	Cilia Fraction	Cilia Fraction	Whole Cell	Whole Cell	Whole Cell	Cilia Fraction	Cilia Fraction	Cilia Fraction	Cilia Fraction	Cilia Fraction	Whole Cell	Whole Cell	Whole Cell	Cilia Fraction	Cilia Fraction	Cilia Fraction	Cilia Fraction	Cilia Fraction
Sample#	1	2	6	4	3	5	7	8	1	2	6	4	3	5	7	8	1	2	6	4	3	5	7	8
**Accession**																								
tr|F1RVS1|F1RVS1	1	0	0	0	0	0	0	0	1	0	0	0	0	0	0	0	4.5%	0.0%	0.0%	0.0%	0.0%	0.0%	0.0%	0.0%
tr|I3LU60|I3LU60	1	0	0	0	0	0	0	0	1	0	0	0	0	0	0	0	24.7%	0.0%	0.0%	0.0%	0.0%	0.0%	0.0%	0.0%
tr|I3LPU3|I3LPU3	0	1	0	0	0	0	0	0	0	1	0	0	0	0	0	0	0.0%	1.2%	0.0%	0.0%	0.0%	0.0%	0.0%	0.0%
tr|F1S6B5|F1S6B5	1	0	0	0	0	0	0	0	1	0	0	0	0	0	0	0	7.7%	0.0%	0.0%	0.0%	0.0%	0.0%	0.0%	0.0%
tr|F1RN09|F1RN09	0	1	0	0	0	0	0	0	0	1	0	0	0	0	0	0	0.0%	5.6%	0.0%	0.0%	0.0%	0.0%	0.0%	0.0%
tr|I3LIQ3|I3LIQ3	1	0	0	0	0	0	0	0	1	0	0	0	0	0	0	0	5.4%	0.0%	0.0%	0.0%	0.0%	0.0%	0.0%	0.0%
tr|F1SAQ9|F1SAQ9	1	0	0	0	0	0	0	0	1	0	0	0	0	0	0	0	23.7%	0.0%	0.0%	0.0%	0.0%	0.0%	0.0%	0.0%
tr|I3LGW8|I3LGW8	1	0	0	0	0	0	0	0	1	0	0	0	0	0	0	0	3.8%	0.0%	0.0%	0.0%	0.0%	0.0%	0.0%	0.0%
tr|K7GMT3|K7GMT3	1	0	0	0	0	0	0	0	1	0	0	0	0	0	0	0	4.1%	0.0%	0.0%	0.0%	0.0%	0.0%	0.0%	0.0%
tr|I3LHX0|I3LHX0	1	0	0	0	0	0	0	0	1	0	0	0	0	0	0	0	5.9%	0.0%	0.0%	0.0%	0.0%	0.0%	0.0%	0.0%
tr|K7GNP2|K7GNP2	0	1	0	0	0	0	0	0	0	1	0	0	0	0	0	0	0.0%	0.8%	0.0%	0.0%	0.0%	0.0%	0.0%	0.0%
tr|I3LA14|I3LA14	0	1	0	0	0	0	0	0	0	1	0	0	0	0	0	0	0.0%	9.5%	0.0%	0.0%	0.0%	0.0%	0.0%	0.0%
tr|F1SUG6|F1SUG6	0	1	0	0	0	0	0	0	0	1	0	0	0	0	0	0	0.0%	1.4%	0.0%	0.0%	0.0%	0.0%	0.0%	0.0%
tr|F1RK85|F1RK85	1	0	0	0	0	0	0	0	1	0	0	0	0	0	0	0	1.4%	0.0%	0.0%	0.0%	0.0%	0.0%	0.0%	0.0%
tr|I3L873|I3L873	1	0	0	0	0	0	0	0	1	0	0	0	0	0	0	0	3.4%	0.0%	0.0%	0.0%	0.0%	0.0%	0.0%	0.0%
tr|F1SEK6|F1SEK6	1	0	0	0	0	0	0	0	1	0	0	0	0	0	0	0	8.9%	0.0%	0.0%	0.0%	0.0%	0.0%	0.0%	0.0%
Reverse_tr|F1RXT5|F1RXT5	0	1	0	0	0	0	0	0	0	1	0	0	0	0	0	0	0.0%	0.8%	0.0%	0.0%	0.0%	0.0%	0.0%	0.0%
tr|F1RG90|F1RG90	0	1	0	0	0	0	0	0	0	1	0	0	0	0	0	0	0.0%	0.8%	0.0%	0.0%	0.0%	0.0%	0.0%	0.0%
tr|F1S3J1|F1S3J1	1	0	0	0	0	0	0	0	1	0	0	0	0	0	0	0	10.5%	0.0%	0.0%	0.0%	0.0%	0.0%	0.0%	0.0%
tr|F1SL73|F1SL73	0	1	0	0	0	0	0	0	0	1	0	0	0	0	0	0	0.0%	5.9%	0.0%	0.0%	0.0%	0.0%	0.0%	0.0%
tr|I3L5Y8|I3L5Y8	0	1	0	0	0	0	0	0	0	1	0	0	0	0	0	0	0.0%	6.2%	0.0%	0.0%	0.0%	0.0%	0.0%	0.0%
tr|K7GLS0|K7GLS0	1	0	0	0	0	0	0	0	1	0	0	0	0	0	0	0	1.8%	0.0%	0.0%	0.0%	0.0%	0.0%	0.0%	0.0%
tr|F1RG63|F1RG63	1	0	0	0	0	0	0	0	1	0	0	0	0	0	0	0	6.2%	0.0%	0.0%	0.0%	0.0%	0.0%	0.0%	0.0%
tr|A5GFW0|A5GFW0	0	1	0	0	0	0	0	0	0	1	0	0	0	0	0	0	0.0%	6.8%	0.0%	0.0%	0.0%	0.0%	0.0%	0.0%
tr|F1S5W6|F1S5W6	0	1	0	0	0	0	0	0	0	1	0	0	0	0	0	0	0.0%	8.4%	0.0%	0.0%	0.0%	0.0%	0.0%	0.0%
tr|F1SRD7|F1SRD7	1	0	0	0	0	0	0	0	1	0	0	0	0	0	0	0	17.6%	0.0%	0.0%	0.0%	0.0%	0.0%	0.0%	0.0%
tr|F1RFN3|F1RFN3	1	0	0	0	0	0	0	0	1	0	0	0	0	0	0	0	1.2%	0.0%	0.0%	0.0%	0.0%	0.0%	0.0%	0.0%
tr|I3LIY2|I3LIY2	0	1	0	0	0	0	0	0	0	1	0	0	0	0	0	0	0.0%	6.7%	0.0%	0.0%	0.0%	0.0%	0.0%	0.0%
tr|I3LAK8|I3LAK8	1	0	0	0	0	0	0	0	1	0	0	0	0	0	0	0	20.0%	0.0%	0.0%	0.0%	0.0%	0.0%	0.0%	0.0%
tr|F1RIG0|F1RIG0	1	0	0	0	0	0	0	0	1	0	0	0	0	0	0	0	7.4%	0.0%	0.0%	0.0%	0.0%	0.0%	0.0%	0.0%
Reverse_tr|F1SR80|F1SR80	0	1	0	0	0	0	0	0	0	1	0	0	0	0	0	0	0.0%	2.9%	0.0%	0.0%	0.0%	0.0%	0.0%	0.0%
tr|I3LR25|I3LR25	0	1	0	0	0	0	0	0	0	1	0	0	0	0	0	0	0.0%	3.1%	0.0%	0.0%	0.0%	0.0%	0.0%	0.0%
tr|I3LN06|I3LN06	1	0	0	0	0	0	0	0	1	0	0	0	0	0	0	0	1.3%	0.0%	0.0%	0.0%	0.0%	0.0%	0.0%	0.0%
tr|I3LH84|I3LH84	0	1	0	0	0	0	0	0	0	1	0	0	0	0	0	0	0.0%	4.4%	0.0%	0.0%	0.0%	0.0%	0.0%	0.0%
tr|I3LFM6|I3LFM6	1	0	0	0	0	0	0	0	1	0	0	0	0	0	0	0	4.6%	0.0%	0.0%	0.0%	0.0%	0.0%	0.0%	0.0%
tr|I3LF21|I3LF21	1	0	0	0	0	0	0	0	1	0	0	0	0	0	0	0	7.1%	0.0%	0.0%	0.0%	0.0%	0.0%	0.0%	0.0%
tr|I3LBX9|I3LBX9	0	1	0	0	0	0	0	0	0	1	0	0	0	0	0	0	0.0%	17.5%	0.0%	0.0%	0.0%	0.0%	0.0%	0.0%
tr|F1RW48|F1RW48	1	0	0	0	0	0	0	0	1	0	0	0	0	0	0	0	5.6%	0.0%	0.0%	0.0%	0.0%	0.0%	0.0%	0.0%
tr|K7GLT6|K7GLT6	1	0	0	0	0	0	0	0	1	0	0	0	0	0	0	0	4.2%	0.0%	0.0%	0.0%	0.0%	0.0%	0.0%	0.0%
tr|I3LTI9|I3LTI9	1	0	0	0	0	0	0	0	1	0	0	0	0	0	0	0	6.9%	0.0%	0.0%	0.0%	0.0%	0.0%	0.0%	0.0%
tr|I3L655|I3L655	1	0	0	0	0	0	0	0	1	0	0	0	0	0	0	0	2.0%	0.0%	0.0%	0.0%	0.0%	0.0%	0.0%	0.0%
tr|K7N7E4|K7N7E4	0	1	0	0	0	0	0	0	0	1	0	0	0	0	0	0	0.0%	9.8%	0.0%	0.0%	0.0%	0.0%	0.0%	0.0%
tr|I3L8Q5|I3L8Q5	1	0	0	0	0	0	0	0	1	0	0	0	0	0	0	0	8.0%	0.0%	0.0%	0.0%	0.0%	0.0%	0.0%	0.0%
tr|F1SF72|F1SF72	1	0	0	0	0	0	0	0	1	0	0	0	0	0	0	0	3.6%	0.0%	0.0%	0.0%	0.0%	0.0%	0.0%	0.0%
tr|F1SNE8|F1SNE8	1	0	0	0	0	0	0	0	1	0	0	0	0	0	0	0	3.2%	0.0%	0.0%	0.0%	0.0%	0.0%	0.0%	0.0%
tr|I3L7I7|I3L7I7	1	0	0	0	0	0	0	0	1	0	0	0	0	0	0	0	9.4%	0.0%	0.0%	0.0%	0.0%	0.0%	0.0%	0.0%
tr|I3LBC4|I3LBC4	1	0	0	0	0	0	0	0	1	0	0	0	0	0	0	0	7.4%	0.0%	0.0%	0.0%	0.0%	0.0%	0.0%	0.0%
tr|F1SRD1|F1SRD1	1	0	0	0	0	0	0	0	1	0	0	0	0	0	0	0	9.1%	0.0%	0.0%	0.0%	0.0%	0.0%	0.0%	0.0%
tr|F1RV56|F1RV56	1	0	0	0	0	0	0	0	1	0	0	0	0	0	0	0	13.2%	0.0%	0.0%	0.0%	0.0%	0.0%	0.0%	0.0%
tr|I3LPC8|I3LPC8	1	0	0	0	0	0	0	0	1	0	0	0	0	0	0	0	27.1%	0.0%	0.0%	0.0%	0.0%	0.0%	0.0%	0.0%
tr|F1S576|F1S576	1	0	0	0	0	0	0	0	1	0	0	0	0	0	0	0	2.0%	0.0%	0.0%	0.0%	0.0%	0.0%	0.0%	0.0%
Reverse_tr|F1SJS9|F1SJS9	1	0	0	0	0	0	0	0	1	0	0	0	0	0	0	0	1.1%	0.0%	0.0%	0.0%	0.0%	0.0%	0.0%	0.0%
tr|I3LNF0|I3LNF0	0	1	0	0	0	0	0	0	0	1	0	0	0	0	0	0	0.0%	0.4%	0.0%	0.0%	0.0%	0.0%	0.0%	0.0%
tr|I3LB63|I3LB63	1	0	0	0	0	0	0	0	1	0	0	0	0	0	0	0	5.2%	0.0%	0.0%	0.0%	0.0%	0.0%	0.0%	0.0%
tr|I3LIC5|I3LIC5	0	1	0	0	0	0	0	0	0	1	0	0	0	0	0	0	0.0%	1.5%	0.0%	0.0%	0.0%	0.0%	0.0%	0.0%
tr|F1SG23|F1SG23	0	1	0	0	0	0	0	0	0	1	0	0	0	0	0	0	0.0%	5.2%	0.0%	0.0%	0.0%	0.0%	0.0%	0.0%
tr|I3L866|I3L866	1	0	0	0	0	0	0	0	1	0	0	0	0	0	0	0	2.2%	0.0%	0.0%	0.0%	0.0%	0.0%	0.0%	0.0%
Reverse_tr|F1RKA9|F1RKA9	1	0	0	0	0	0	0	0	1	0	0	0	0	0	0	0	3.1%	0.0%	0.0%	0.0%	0.0%	0.0%	0.0%	0.0%
tr|F1RYI5|F1RYI5	1	0	0	0	0	0	0	0	1	0	0	0	0	0	0	0	2.5%	0.0%	0.0%	0.0%	0.0%	0.0%	0.0%	0.0%
tr|F1RFK3|F1RFK3	1	0	0	0	0	0	0	0	1	0	0	0	0	0	0	0	0.9%	0.0%	0.0%	0.0%	0.0%	0.0%	0.0%	0.0%
tr|F1SRK9|F1SRK9	0	1	0	0	0	0	0	0	0	1	0	0	0	0	0	0	0.0%	3.7%	0.0%	0.0%	0.0%	0.0%	0.0%	0.0%
tr|I3L5U5|I3L5U5	1	0	0	0	0	0	0	0	1	0	0	0	0	0	0	0	5.1%	0.0%	0.0%	0.0%	0.0%	0.0%	0.0%	0.0%
tr|I3LA45|I3LA45	1	0	0	0	0	0	0	0	1	0	0	0	0	0	0	0	6.7%	0.0%	0.0%	0.0%	0.0%	0.0%	0.0%	0.0%
tr|F1S0U6|F1S0U6	0	1	0	0	0	0	0	0	0	1	0	0	0	0	0	0	0.0%	0.4%	0.0%	0.0%	0.0%	0.0%	0.0%	0.0%
tr|F1SMI6|F1SMI6	1	0	0	0	0	0	0	0	1	0	0	0	0	0	0	0	18.2%	0.0%	0.0%	0.0%	0.0%	0.0%	0.0%	0.0%
tr|F1RQH7|F1RQH7	1	0	0	0	0	0	0	0	1	0	0	0	0	0	0	0	2.9%	0.0%	0.0%	0.0%	0.0%	0.0%	0.0%	0.0%
tr|F1SUL0|F1SUL0	1	0	0	0	0	0	0	0	1	0	0	0	0	0	0	0	3.6%	0.0%	0.0%	0.0%	0.0%	0.0%	0.0%	0.0%
tr|F1RV32|F1RV32	1	0	0	0	0	0	0	0	1	0	0	0	0	0	0	0	1.4%	0.0%	0.0%	0.0%	0.0%	0.0%	0.0%	0.0%
tr|I3LHW7|I3LHW7	1	0	0	0	0	0	0	0	1	0	0	0	0	0	0	0	19.4%	0.0%	0.0%	0.0%	0.0%	0.0%	0.0%	0.0%
tr|I3LJ77|I3LJ77	0	1	0	0	0	0	0	0	0	1	0	0	0	0	0	0	0.0%	2.7%	0.0%	0.0%	0.0%	0.0%	0.0%	0.0%
tr|F1SIH0|F1SIH0	1	0	0	0	0	0	0	0	1	0	0	0	0	0	0	0	21.6%	0.0%	0.0%	0.0%	0.0%	0.0%	0.0%	0.0%
Reverse_tr|F1SK00|F1SK00	1	0	0	0	0	0	0	0	1	0	0	0	0	0	0	0	4.8%	0.0%	0.0%	0.0%	0.0%	0.0%	0.0%	0.0%
tr|F2Z5D9|F2Z5D9	1	0	0	0	0	0	0	0	1	0	0	0	0	0	0	0	19.1%	0.0%	0.0%	0.0%	0.0%	0.0%	0.0%	0.0%
tr|F1RVV9|F1RVV9	0	1	0	0	0	0	0	0	0	1	0	0	0	0	0	0	0.0%	1.8%	0.0%	0.0%	0.0%	0.0%	0.0%	0.0%
tr|K7GMF0|K7GMF0	1	0	0	0	0	0	0	0	1	0	0	0	0	0	0	0	2.9%	0.0%	0.0%	0.0%	0.0%	0.0%	0.0%	0.0%
tr|K7GSX6|K7GSX6	0	1	0	0	0	0	0	0	0	1	0	0	0	0	0	0	0.0%	5.1%	0.0%	0.0%	0.0%	0.0%	0.0%	0.0%
tr|I3LHH0|I3LHH0	0	1	0	0	0	0	0	0	0	1	0	0	0	0	0	0	0.0%	1.2%	0.0%	0.0%	0.0%	0.0%	0.0%	0.0%
tr|F1SHQ4|F1SHQ4	1	0	0	0	0	0	0	0	1	0	0	0	0	0	0	0	2.0%	0.0%	0.0%	0.0%	0.0%	0.0%	0.0%	0.0%
tr|F1RFE3|F1RFE3	1	0	0	0	0	0	0	0	1	0	0	0	0	0	0	0	6.6%	0.0%	0.0%	0.0%	0.0%	0.0%	0.0%	0.0%
tr|K7GR19|K7GR19	0	1	0	0	0	0	0	0	0	1	0	0	0	0	0	0	0.0%	3.3%	0.0%	0.0%	0.0%	0.0%	0.0%	0.0%
tr|I3LU42|I3LU42	0	1	0	0	0	0	0	0	0	1	0	0	0	0	0	0	0.0%	6.2%	0.0%	0.0%	0.0%	0.0%	0.0%	0.0%

	Peptide Count								Spectral Count								Sequence Coverage							
	Whole Cell	Whole Cell	Whole Cell	Cilia Fraction	Cilia Fraction	Cilia Fraction	Cilia Fraction	Cilia Fraction	Whole Cell	Whole Cell	Whole Cell	Cilia Fraction	Cilia Fraction	Cilia Fraction	Cilia Fraction	Cilia Fraction	Whole Cell	Whole Cell	Whole Cell	Cilia Fraction	Cilia Fraction	Cilia Fraction	Cilia Fraction	Cilia Fraction
Sample#	1	2	6	4	3	5	7	8	1	2	6	4	3	5	7	8	1	2	6	4	3	5	7	8
**Accession**																								
tr|F1RY40|F1RY40	1	0	0	0	0	0	0	0	1	0	0	0	0	0	0	0	1.4%	0.0%	0.0%	0.0%	0.0%	0.0%	0.0%	0.0%
tr|F1SDS2|F1SDS2	0	1	0	0	0	0	0	0	0	1	0	0	0	0	0	0	0.0%	3.5%	0.0%	0.0%	0.0%	0.0%	0.0%	0.0%
tr|F1ST27|F1ST27	1	0	0	0	0	0	0	0	1	0	0	0	0	0	0	0	13.4%	0.0%	0.0%	0.0%	0.0%	0.0%	0.0%	0.0%
tr|F1RYQ6|F1RYQ6	0	1	0	0	0	0	0	0	0	1	0	0	0	0	0	0	0.0%	1.3%	0.0%	0.0%	0.0%	0.0%	0.0%	0.0%
tr|I3LCR2|I3LCR2	0	1	0	0	0	0	0	0	0	1	0	0	0	0	0	0	0.0%	42.6%	0.0%	0.0%	0.0%	0.0%	0.0%	0.0%
tr|F1RVT1|F1RVT1	1	0	0	0	0	0	0	0	1	0	0	0	0	0	0	0	1.5%	0.0%	0.0%	0.0%	0.0%	0.0%	0.0%	0.0%
tr|F1RRT2|F1RRT2	1	0	0	0	0	0	0	0	1	0	0	0	0	0	0	0	7.9%	0.0%	0.0%	0.0%	0.0%	0.0%	0.0%	0.0%
tr|I3L7G7|I3L7G7	1	0	0	0	0	0	0	0	1	0	0	0	0	0	0	0	6.5%	0.0%	0.0%	0.0%	0.0%	0.0%	0.0%	0.0%
tr|F1S150|F1S150	1	0	0	0	0	0	0	0	1	0	0	0	0	0	0	0	2.2%	0.0%	0.0%	0.0%	0.0%	0.0%	0.0%	0.0%
tr|F1SNB1|F1SNB1	1	0	0	0	0	0	0	0	1	0	0	0	0	0	0	0	4.6%	0.0%	0.0%	0.0%	0.0%	0.0%	0.0%	0.0%
Q28944	0	1	0	0	0	0	0	0	0	1	0	0	0	0	0	0	0.0%	6.9%	0.0%	0.0%	0.0%	0.0%	0.0%	0.0%
Q28948	1	0	0	0	0	0	0	0	1	0	0	0	0	0	0	0	3.1%	0.0%	0.0%	0.0%	0.0%	0.0%	0.0%	0.0%
Q28955	1	0	0	0	0	0	0	0	1	0	0	0	0	0	0	0	6.8%	0.0%	0.0%	0.0%	0.0%	0.0%	0.0%	0.0%
Q28970	0	1	0	0	0	0	0	0	0	1	0	0	0	0	0	0	0.0%	2.5%	0.0%	0.0%	0.0%	0.0%	0.0%	0.0%
tr|I3LEF7|I3LEF7	1	0	0	0	0	0	0	0	1	0	0	0	0	0	0	0	3.9%	0.0%	0.0%	0.0%	0.0%	0.0%	0.0%	0.0%
tr|I3LLZ7|I3LLZ7	0	1	0	0	0	0	0	0	0	1	0	0	0	0	0	0	0.0%	6.5%	0.0%	0.0%	0.0%	0.0%	0.0%	0.0%
tr|I3L7K3|I3L7K3	1	0	0	0	0	0	0	0	1	0	0	0	0	0	0	0	3.6%	0.0%	0.0%	0.0%	0.0%	0.0%	0.0%	0.0%
tr|I3LE56|I3LE56	0	1	0	0	0	0	0	0	0	1	0	0	0	0	0	0	0.0%	2.2%	0.0%	0.0%	0.0%	0.0%	0.0%	0.0%
tr|F1SGA9|F1SGA9	1	0	0	0	0	0	0	0	1	0	0	0	0	0	0	0	12.1%	0.0%	0.0%	0.0%	0.0%	0.0%	0.0%	0.0%
tr|F1SQL8|F1SQL8	1	0	0	0	0	0	0	0	1	0	0	0	0	0	0	0	5.3%	0.0%	0.0%	0.0%	0.0%	0.0%	0.0%	0.0%
tr|I3LDA1|I3LDA1	1	0	0	0	0	0	0	0	1	0	0	0	0	0	0	0	1.2%	0.0%	0.0%	0.0%	0.0%	0.0%	0.0%	0.0%
tr|F1RGM7|F1RGM7	1	0	0	0	0	0	0	0	1	0	0	0	0	0	0	0	7.3%	0.0%	0.0%	0.0%	0.0%	0.0%	0.0%	0.0%
tr|F1RMF6|F1RMF6	1	0	0	0	0	0	0	0	1	0	0	0	0	0	0	0	5.1%	0.0%	0.0%	0.0%	0.0%	0.0%	0.0%	0.0%
tr|I3LLE0|I3LLE0	1	0	0	0	0	0	0	0	1	0	0	0	0	0	0	0	4.5%	0.0%	0.0%	0.0%	0.0%	0.0%	0.0%	0.0%
tr|I3L8T3|I3L8T3	0	1	0	0	0	0	0	0	0	1	0	0	0	0	0	0	0.0%	1.8%	0.0%	0.0%	0.0%	0.0%	0.0%	0.0%
tr|F1SDQ3|F1SDQ3	1	0	0	0	0	0	0	0	1	0	0	0	0	0	0	0	3.8%	0.0%	0.0%	0.0%	0.0%	0.0%	0.0%	0.0%
tr|F1SRR2|F1SRR2	1	0	0	0	0	0	0	0	1	0	0	0	0	0	0	0	17.9%	0.0%	0.0%	0.0%	0.0%	0.0%	0.0%	0.0%
tr|I3L6I5|I3L6I5	1	0	0	0	0	0	0	0	1	0	0	0	0	0	0	0	5.6%	0.0%	0.0%	0.0%	0.0%	0.0%	0.0%	0.0%
tr|F1RL37|F1RL37	0	1	0	0	0	0	0	0	0	1	0	0	0	0	0	0	0.0%	13.8%	0.0%	0.0%	0.0%	0.0%	0.0%	0.0%
tr|F1RKR7|F1RKR7	0	1	0	0	0	0	0	0	0	1	0	0	0	0	0	0	0.0%	4.6%	0.0%	0.0%	0.0%	0.0%	0.0%	0.0%
tr|F1SQK6|F1SQK6	1	0	0	0	0	0	0	0	1	0	0	0	0	0	0	0	1.7%	0.0%	0.0%	0.0%	0.0%	0.0%	0.0%	0.0%
tr|I3L694|I3L694	0	1	0	0	0	0	0	0	0	1	0	0	0	0	0	0	0.0%	4.9%	0.0%	0.0%	0.0%	0.0%	0.0%	0.0%
tr|F1RRV9|F1RRV9	1	0	0	0	0	0	0	0	1	0	0	0	0	0	0	0	2.0%	0.0%	0.0%	0.0%	0.0%	0.0%	0.0%	0.0%
tr|F1RQH3|F1RQH3	1	0	0	0	0	0	0	0	1	0	0	0	0	0	0	0	1.2%	0.0%	0.0%	0.0%	0.0%	0.0%	0.0%	0.0%
tr|A0ZVR0|A0ZVR0	1	0	0	0	0	0	0	0	1	0	0	0	0	0	0	0	5.7%	0.0%	0.0%	0.0%	0.0%	0.0%	0.0%	0.0%
tr|F1S5S8|F1S5S8	1	0	0	0	0	0	0	0	1	0	0	0	0	0	0	0	2.7%	0.0%	0.0%	0.0%	0.0%	0.0%	0.0%	0.0%
tr|F1S6Z9|F1S6Z9	1	0	0	0	0	0	0	0	1	0	0	0	0	0	0	0	2.4%	0.0%	0.0%	0.0%	0.0%	0.0%	0.0%	0.0%
tr|F1RVI6|F1RVI6	0	1	0	0	0	0	0	0	0	1	0	0	0	0	0	0	0.0%	2.7%	0.0%	0.0%	0.0%	0.0%	0.0%	0.0%
tr|F1RQ27|F1RQ27	1	0	0	0	0	0	0	0	1	0	0	0	0	0	0	0	2.8%	0.0%	0.0%	0.0%	0.0%	0.0%	0.0%	0.0%
tr|K7GQ81|K7GQ81	0	1	0	0	0	0	0	0	0	1	0	0	0	0	0	0	0.0%	4.6%	0.0%	0.0%	0.0%	0.0%	0.0%	0.0%
Reverse_tr|I3L5N6|I3L5N6	1	0	0	0	0	0	0	0	1	0	0	0	0	0	0	0	16.5%	0.0%	0.0%	0.0%	0.0%	0.0%	0.0%	0.0%
tr|F1RFU0|F1RFU0	0	1	0	0	0	0	0	0	0	1	0	0	0	0	0	0	0.0%	2.7%	0.0%	0.0%	0.0%	0.0%	0.0%	0.0%
tr|F1SLH6|F1SLH6	0	1	0	0	0	0	0	0	0	1	0	0	0	0	0	0	0.0%	2.7%	0.0%	0.0%	0.0%	0.0%	0.0%	0.0%
tr|F1RG33|F1RG33	1	0	0	0	0	0	0	0	1	0	0	0	0	0	0	0	2.0%	0.0%	0.0%	0.0%	0.0%	0.0%	0.0%	0.0%
tr|F1RIC1|F1RIC1	1	0	0	0	0	0	0	0	1	0	0	0	0	0	0	0	3.0%	0.0%	0.0%	0.0%	0.0%	0.0%	0.0%	0.0%
tr|F1SGN7|F1SGN7	1	0	0	0	0	0	0	0	1	0	0	0	0	0	0	0	11.0%	0.0%	0.0%	0.0%	0.0%	0.0%	0.0%	0.0%
tr|I3LSR0|I3LSR0	1	0	0	0	0	0	0	0	1	0	0	0	0	0	0	0	14.3%	0.0%	0.0%	0.0%	0.0%	0.0%	0.0%	0.0%
tr|F1SUS4|F1SUS4	1	0	0	0	0	0	0	0	1	0	0	0	0	0	0	0	13.5%	0.0%	0.0%	0.0%	0.0%	0.0%	0.0%	0.0%
tr|I3L5R8|I3L5R8	1	0	0	0	0	0	0	0	1	0	0	0	0	0	0	0	2.2%	0.0%	0.0%	0.0%	0.0%	0.0%	0.0%	0.0%
tr|F1RQU4|F1RQU4	1	0	0	0	0	0	0	0	1	0	0	0	0	0	0	0	3.1%	0.0%	0.0%	0.0%	0.0%	0.0%	0.0%	0.0%
tr|I3LGC9|I3LGC9	1	0	0	0	0	0	0	0	1	0	0	0	0	0	0	0	9.7%	0.0%	0.0%	0.0%	0.0%	0.0%	0.0%	0.0%
tr|F1S3L2|F1S3L2	1	0	0	0	0	0	0	0	1	0	0	0	0	0	0	0	1.4%	0.0%	0.0%	0.0%	0.0%	0.0%	0.0%	0.0%
tr|F1SHV6|F1SHV6	0	1	0	0	0	0	0	0	0	1	0	0	0	0	0	0	0.0%	2.9%	0.0%	0.0%	0.0%	0.0%	0.0%	0.0%
tr|F1SI76|F1SI76	0	1	0	0	0	0	0	0	0	1	0	0	0	0	0	0	0.0%	1.2%	0.0%	0.0%	0.0%	0.0%	0.0%	0.0%
tr|I3LGI1|I3LGI1	0	1	0	0	0	0	0	0	0	1	0	0	0	0	0	0	0.0%	7.6%	0.0%	0.0%	0.0%	0.0%	0.0%	0.0%
tr|F1RGT2|F1RGT2	1	0	0	0	0	0	0	0	1	0	0	0	0	0	0	0	6.8%	0.0%	0.0%	0.0%	0.0%	0.0%	0.0%	0.0%
tr|I3LS78|I3LS78	1	0	0	0	0	0	0	0	1	0	0	0	0	0	0	0	5.8%	0.0%	0.0%	0.0%	0.0%	0.0%	0.0%	0.0%
tr|F1S1S9|F1S1S9	0	1	0	0	0	0	0	0	0	1	0	0	0	0	0	0	0.0%	10.2%	0.0%	0.0%	0.0%	0.0%	0.0%	0.0%
tr|F2Z5H7|F2Z5H7	1	0	0	0	0	0	0	0	1	0	0	0	0	0	0	0	3.5%	0.0%	0.0%	0.0%	0.0%	0.0%	0.0%	0.0%
tr|F1S2B1|F1S2B1	0	1	0	0	0	0	0	0	0	1	0	0	0	0	0	0	0.0%	4.8%	0.0%	0.0%	0.0%	0.0%	0.0%	0.0%
tr|F1SIH4|F1SIH4	1	0	0	0	0	0	0	0	1	0	0	0	0	0	0	0	0.9%	0.0%	0.0%	0.0%	0.0%	0.0%	0.0%	0.0%
tr|F6PWG7|F6PWG7	1	0	0	0	0	0	0	0	1	0	0	0	0	0	0	0	9.3%	0.0%	0.0%	0.0%	0.0%	0.0%	0.0%	0.0%
Reverse_tr|F1SGX3|F1SGX3	1	0	0	0	0	0	0	0	1	0	0	0	0	0	0	0	0.8%	0.0%	0.0%	0.0%	0.0%	0.0%	0.0%	0.0%
tr|F1RL14|F1RL14	1	0	0	0	0	0	0	0	1	0	0	0	0	0	0	0	5.5%	0.0%	0.0%	0.0%	0.0%	0.0%	0.0%	0.0%
tr|I3LJK1|I3LJK1	0	1	0	0	0	0	0	0	0	1	0	0	0	0	0	0	0.0%	2.2%	0.0%	0.0%	0.0%	0.0%	0.0%	0.0%
tr|I3L673|I3L673	0	1	0	0	0	0	0	0	0	1	0	0	0	0	0	0	0.0%	6.2%	0.0%	0.0%	0.0%	0.0%	0.0%	0.0%
tr|F1S680|F1S680	0	1	0	0	0	0	0	0	0	1	0	0	0	0	0	0	0.0%	1.1%	0.0%	0.0%	0.0%	0.0%	0.0%	0.0%
tr|I3LCS9|I3LCS9	1	0	0	0	0	0	0	0	1	0	0	0	0	0	0	0	4.6%	0.0%	0.0%	0.0%	0.0%	0.0%	0.0%	0.0%
tr|F1RLR4|F1RLR4	0	1	0	0	0	0	0	0	0	1	0	0	0	0	0	0	0.0%	2.4%	0.0%	0.0%	0.0%	0.0%	0.0%	0.0%
tr|F1RJU6|F1RJU6	1	0	0	0	0	0	0	0	1	0	0	0	0	0	0	0	1.3%	0.0%	0.0%	0.0%	0.0%	0.0%	0.0%	0.0%
tr|F1RFH0|F1RFH0	1	0	0	0	0	0	0	0	1	0	0	0	0	0	0	0	2.5%	0.0%	0.0%	0.0%	0.0%	0.0%	0.0%	0.0%
Reverse_tr|I3LTJ6|I3LTJ6	1	0	0	0	0	0	0	0	1	0	0	0	0	0	0	0	0.7%	0.0%	0.0%	0.0%	0.0%	0.0%	0.0%	0.0%
tr|I3LSS6|I3LSS6	0	1	0	0	0	0	0	0	0	1	0	0	0	0	0	0	0.0%	1.9%	0.0%	0.0%	0.0%	0.0%	0.0%	0.0%
Q5EE05	1	0	0	0	0	0	0	0	1	0	0	0	0	0	0	0	8.9%	0.0%	0.0%	0.0%	0.0%	0.0%	0.0%	0.0%
tr|F1RJQ0|F1RJQ0	1	0	0	0	0	0	0	0	1	0	0	0	0	0	0	0	5.3%	0.0%	0.0%	0.0%	0.0%	0.0%	0.0%	0.0%
tr|K7GMC9|K7GMC9	1	0	0	0	0	0	0	0	1	0	0	0	0	0	0	0	30.3%	0.0%	0.0%	0.0%	0.0%	0.0%	0.0%	0.0%
tr|I3LS02|I3LS02	1	0	0	0	0	0	0	0	1	0	0	0	0	0	0	0	8.5%	0.0%	0.0%	0.0%	0.0%	0.0%	0.0%	0.0%
tr|F1RMM1|F1RMM1	0	1	0	0	0	0	0	0	0	1	0	0	0	0	0	0	0.0%	1.8%	0.0%	0.0%	0.0%	0.0%	0.0%	0.0%
tr|K7GRZ5|K7GRZ5	0	1	0	0	0	0	0	0	0	1	0	0	0	0	0	0	0.0%	15.0%	0.0%	0.0%	0.0%	0.0%	0.0%	0.0%
tr|I3LV73|I3LV73	1	0	0	0	0	0	0	0	1	0	0	0	0	0	0	0	10.8%	0.0%	0.0%	0.0%	0.0%	0.0%	0.0%	0.0%
tr|F1S022|F1S022	1	0	0	0	0	0	0	0	1	0	0	0	0	0	0	0	4.2%	0.0%	0.0%	0.0%	0.0%	0.0%	0.0%	0.0%

	Peptide Count								Spectral Count								Sequence Coverage							
	Whole Cell	Whole Cell	Whole Cell	Cilia Fraction	Cilia Fraction	Cilia Fraction	Cilia Fraction	Cilia Fraction	Whole Cell	Whole Cell	Whole Cell	Cilia Fraction	Cilia Fraction	Cilia Fraction	Cilia Fraction	Cilia Fraction	Whole Cell	Whole Cell	Whole Cell	Cilia Fraction	Cilia Fraction	Cilia Fraction	Cilia Fraction	Cilia Fraction
Sample#	1	2	6	4	3	5	7	8	1	2	6	4	3	5	7	8	1	2	6	4	3	5	7	8
**Accession**																								
F1SRI0	1	0	0	0	0	0	0	0	1	0	0	0	0	0	0	0	19.3%	0.0%	0.0%	0.0%	0.0%	0.0%	0.0%	0.0%
tr|F1SN14|F1SN14	1	0	0	0	0	0	0	0	1	0	0	0	0	0	0	0	1.2%	0.0%	0.0%	0.0%	0.0%	0.0%	0.0%	0.0%
tr|I3LNN4|I3LNN4	1	0	0	0	0	0	0	0	1	0	0	0	0	0	0	0	9.7%	0.0%	0.0%	0.0%	0.0%	0.0%	0.0%	0.0%
tr|F1S3W1|F1S3W1	0	1	0	0	0	0	0	0	0	1	0	0	0	0	0	0	0.0%	5.7%	0.0%	0.0%	0.0%	0.0%	0.0%	0.0%
tr|F1RS81|F1RS81	1	0	0	0	0	0	0	0	1	0	0	0	0	0	0	0	1.4%	0.0%	0.0%	0.0%	0.0%	0.0%	0.0%	0.0%
tr|I3LD94|I3LD94	1	0	0	0	0	0	0	0	1	0	0	0	0	0	0	0	14.4%	0.0%	0.0%	0.0%	0.0%	0.0%	0.0%	0.0%
tr|F1S8D2|F1S8D2	0	1	0	0	0	0	0	0	0	1	0	0	0	0	0	0	0.0%	4.0%	0.0%	0.0%	0.0%	0.0%	0.0%	0.0%
tr|F1RF38|F1RF38	1	0	0	0	0	0	0	0	1	0	0	0	0	0	0	0	6.0%	0.0%	0.0%	0.0%	0.0%	0.0%	0.0%	0.0%
A1XQR6	1	0	0	0	0	0	0	0	1	0	0	0	0	0	0	0	21.5%	0.0%	0.0%	0.0%	0.0%	0.0%	0.0%	0.0%
tr|I3LB77|I3LB77	0	1	0	0	0	0	0	0	0	1	0	0	0	0	0	0	0.0%	2.1%	0.0%	0.0%	0.0%	0.0%	0.0%	0.0%
tr|I3L729|I3L729	1	0	0	0	0	0	0	0	1	0	0	0	0	0	0	0	4.2%	0.0%	0.0%	0.0%	0.0%	0.0%	0.0%	0.0%
tr|I3LVE4|I3LVE4	1	0	0	0	0	0	0	0	1	0	0	0	0	0	0	0	1.5%	0.0%	0.0%	0.0%	0.0%	0.0%	0.0%	0.0%
tr|K7GS71|K7GS71	0	1	0	0	0	0	0	0	0	1	0	0	0	0	0	0	0.0%	0.8%	0.0%	0.0%	0.0%	0.0%	0.0%	0.0%
tr|F1RSP2|F1RSP2	0	1	0	0	0	0	0	0	0	1	0	0	0	0	0	0	0.0%	2.5%	0.0%	0.0%	0.0%	0.0%	0.0%	0.0%
tr|F1S4P2|F1S4P2	1	0	0	0	0	0	0	0	1	0	0	0	0	0	0	0	4.3%	0.0%	0.0%	0.0%	0.0%	0.0%	0.0%	0.0%
tr|F1SQR9|F1SQR9	1	0	0	0	0	0	0	0	1	0	0	0	0	0	0	0	6.2%	0.0%	0.0%	0.0%	0.0%	0.0%	0.0%	0.0%
tr|F1RUN0|F1RUN0	0	1	0	0	0	0	0	0	0	1	0	0	0	0	0	0	0.0%	4.7%	0.0%	0.0%	0.0%	0.0%	0.0%	0.0%
tr|K7GQ01|K7GQ01	0	1	0	0	0	0	0	0	0	1	0	0	0	0	0	0	0.0%	4.0%	0.0%	0.0%	0.0%	0.0%	0.0%	0.0%
tr|F1RMG0|F1RMG0	0	1	0	0	0	0	0	0	0	1	0	0	0	0	0	0	0.0%	1.8%	0.0%	0.0%	0.0%	0.0%	0.0%	0.0%
tr|F1ST67|F1ST67	1	0	0	0	0	0	0	0	1	0	0	0	0	0	0	0	5.8%	0.0%	0.0%	0.0%	0.0%	0.0%	0.0%	0.0%
tr|K7GLY3|K7GLY3	1	0	0	0	0	0	0	0	1	0	0	0	0	0	0	0	13.7%	0.0%	0.0%	0.0%	0.0%	0.0%	0.0%	0.0%
tr|F1SB02|F1SB02	1	0	0	0	0	0	0	0	1	0	0	0	0	0	0	0	3.4%	0.0%	0.0%	0.0%	0.0%	0.0%	0.0%	0.0%
Q719N1	1	0	0	0	0	0	0	0	1	0	0	0	0	0	0	0	3.4%	0.0%	0.0%	0.0%	0.0%	0.0%	0.0%	0.0%
tr|F1S156|F1S156	1	0	0	0	0	0	0	0	1	0	0	0	0	0	0	0	3.2%	0.0%	0.0%	0.0%	0.0%	0.0%	0.0%	0.0%
tr|K7GKK9|K7GKK9	1	0	0	0	0	0	0	0	1	0	0	0	0	0	0	0	4.7%	0.0%	0.0%	0.0%	0.0%	0.0%	0.0%	0.0%
tr|I3LQ40|I3LQ40	1	0	0	0	0	0	0	0	1	0	0	0	0	0	0	0	9.0%	0.0%	0.0%	0.0%	0.0%	0.0%	0.0%	0.0%
Reverse_tr|I3L8T2|I3L8T2	0	1	0	0	0	0	0	0	0	1	0	0	0	0	0	0	0.0%	2.8%	0.0%	0.0%	0.0%	0.0%	0.0%	0.0%
tr|F1S130|F1S130	1	0	0	0	0	0	0	0	1	0	0	0	0	0	0	0	7.8%	0.0%	0.0%	0.0%	0.0%	0.0%	0.0%	0.0%
tr|I3LUN6|I3LUN6	1	0	0	0	0	0	0	0	1	0	0	0	0	0	0	0	4.5%	0.0%	0.0%	0.0%	0.0%	0.0%	0.0%	0.0%
tr|F1SGJ7|F1SGJ7	1	0	0	0	0	0	0	0	1	0	0	0	0	0	0	0	3.4%	0.0%	0.0%	0.0%	0.0%	0.0%	0.0%	0.0%
tr|I3LFX1|I3LFX1	0	1	0	0	0	0	0	0	0	1	0	0	0	0	0	0	0.0%	0.9%	0.0%	0.0%	0.0%	0.0%	0.0%	0.0%
tr|I3LGA1|I3LGA1	1	0	0	0	0	0	0	0	1	0	0	0	0	0	0	0	4.3%	0.0%	0.0%	0.0%	0.0%	0.0%	0.0%	0.0%
tr|F1SFX2|F1SFX2	0	1	0	0	0	0	0	0	0	1	0	0	0	0	0	0	0.0%	2.5%	0.0%	0.0%	0.0%	0.0%	0.0%	0.0%
tr|I3L7Z3|I3L7Z3	1	0	0	0	0	0	0	0	1	0	0	0	0	0	0	0	7.4%	0.0%	0.0%	0.0%	0.0%	0.0%	0.0%	0.0%
tr|F1SG44|F1SG44	1	0	0	0	0	0	0	0	1	0	0	0	0	0	0	0	6.9%	0.0%	0.0%	0.0%	0.0%	0.0%	0.0%	0.0%
tr|B8Y649|B8Y649	1	0	0	0	0	0	0	0	1	0	0	0	0	0	0	0	12.6%	0.0%	0.0%	0.0%	0.0%	0.0%	0.0%	0.0%
tr|F1SH36|F1SH36	0	1	0	0	0	0	0	0	0	1	0	0	0	0	0	0	0.0%	2.1%	0.0%	0.0%	0.0%	0.0%	0.0%	0.0%
tr|F1SH07|F1SH07	0	1	0	0	0	0	0	0	0	1	0	0	0	0	0	0	0.0%	1.6%	0.0%	0.0%	0.0%	0.0%	0.0%	0.0%
Q9TTB4	1	0	0	0	0	0	0	0	1	0	0	0	0	0	0	0	19.7%	0.0%	0.0%	0.0%	0.0%	0.0%	0.0%	0.0%
tr|I3LAD3|I3LAD3	1	0	0	0	0	0	0	0	1	0	0	0	0	0	0	0	2.5%	0.0%	0.0%	0.0%	0.0%	0.0%	0.0%	0.0%
tr|I3LNJ2|I3LNJ2	1	0	0	0	0	0	0	0	1	0	0	0	0	0	0	0	1.3%	0.0%	0.0%	0.0%	0.0%	0.0%	0.0%	0.0%
tr|F1RQW9|F1RQW9	0	1	0	0	0	0	0	0	0	1	0	0	0	0	0	0	0.0%	0.9%	0.0%	0.0%	0.0%	0.0%	0.0%	0.0%
tr|I3LFL8|I3LFL8	1	0	0	0	0	0	0	0	1	0	0	0	0	0	0	0	2.2%	0.0%	0.0%	0.0%	0.0%	0.0%	0.0%	0.0%
tr|F1RN10|F1RN10	0	1	0	0	0	0	0	0	0	1	0	0	0	0	0	0	0.0%	4.1%	0.0%	0.0%	0.0%	0.0%	0.0%	0.0%
tr|F1S2X8|F1S2X8	1	0	0	0	0	0	0	0	1	0	0	0	0	0	0	0	3.3%	0.0%	0.0%	0.0%	0.0%	0.0%	0.0%	0.0%
tr|F1S834|F1S834	0	1	0	0	0	0	0	0	0	1	0	0	0	0	0	0	0.0%	4.9%	0.0%	0.0%	0.0%	0.0%	0.0%	0.0%
tr|K7GNB9|K7GNB9	1	0	0	0	0	0	0	0	1	0	0	0	0	0	0	0	12.6%	0.0%	0.0%	0.0%	0.0%	0.0%	0.0%	0.0%
tr|F1S5K3|F1S5K3	0	1	0	0	0	0	0	0	0	1	0	0	0	0	0	0	0.0%	2.5%	0.0%	0.0%	0.0%	0.0%	0.0%	0.0%
tr|F1RNU0|F1RNU0	1	0	0	0	0	0	0	0	1	0	0	0	0	0	0	0	6.1%	0.0%	0.0%	0.0%	0.0%	0.0%	0.0%	0.0%
tr|F1SR54|F1SR54	1	0	0	0	0	0	0	0	1	0	0	0	0	0	0	0	3.1%	0.0%	0.0%	0.0%	0.0%	0.0%	0.0%	0.0%
tr|F1SRV4|F1SRV4	0	1	0	0	0	0	0	0	0	1	0	0	0	0	0	0	0.0%	8.4%	0.0%	0.0%	0.0%	0.0%	0.0%	0.0%
tr|F1S4G4|F1S4G4	1	0	0	0	0	0	0	0	1	0	0	0	0	0	0	0	3.3%	0.0%	0.0%	0.0%	0.0%	0.0%	0.0%	0.0%
tr|F1RMQ6|F1RMQ6	0	1	0	0	0	0	0	0	0	1	0	0	0	0	0	0	0.0%	0.6%	0.0%	0.0%	0.0%	0.0%	0.0%	0.0%
tr|I3LDY5|I3LDY5	1	0	0	0	0	0	0	0	1	0	0	0	0	0	0	0	6.8%	0.0%	0.0%	0.0%	0.0%	0.0%	0.0%	0.0%
tr|F1RK57|F1RK57	1	0	0	0	0	0	0	0	1	0	0	0	0	0	0	0	6.1%	0.0%	0.0%	0.0%	0.0%	0.0%	0.0%	0.0%
tr|F1S1G9|F1S1G9	1	0	0	0	0	0	0	0	1	0	0	0	0	0	0	0	1.7%	0.0%	0.0%	0.0%	0.0%	0.0%	0.0%	0.0%
Reverse_tr|F1S9D1|F1S9D1	1	0	0	0	0	0	0	0	1	0	0	0	0	0	0	0	1.3%	0.0%	0.0%	0.0%	0.0%	0.0%	0.0%	0.0%
tr|K7GMC1|K7GMC1	1	0	0	0	0	0	0	0	1	0	0	0	0	0	0	0	25.5%	0.0%	0.0%	0.0%	0.0%	0.0%	0.0%	0.0%
tr|I3LTC2|I3LTC2	1	0	0	0	0	0	0	0	1	0	0	0	0	0	0	0	2.9%	0.0%	0.0%	0.0%	0.0%	0.0%	0.0%	0.0%
Reverse_tr|I3LP16|I3LP16	0	1	0	0	0	0	0	0	0	1	0	0	0	0	0	0	0.0%	6.4%	0.0%	0.0%	0.0%	0.0%	0.0%	0.0%
P79432	1	0	0	0	0	0	0	0	1	0	0	0	0	0	0	0	3.0%	0.0%	0.0%	0.0%	0.0%	0.0%	0.0%	0.0%
tr|F1STS2|F1STS2	1	0	0	0	0	0	0	0	1	0	0	0	0	0	0	0	1.6%	0.0%	0.0%	0.0%	0.0%	0.0%	0.0%	0.0%
tr|F1SHZ5|F1SHZ5	0	1	0	0	0	0	0	0	0	1	0	0	0	0	0	0	0.0%	3.4%	0.0%	0.0%	0.0%	0.0%	0.0%	0.0%
tr|I3LAX1|I3LAX1	1	0	0	0	0	0	0	0	1	0	0	0	0	0	0	0	1.2%	0.0%	0.0%	0.0%	0.0%	0.0%	0.0%	0.0%
tr|I3L9G6|I3L9G6	0	1	0	0	0	0	0	0	0	1	0	0	0	0	0	0	0.0%	10.3%	0.0%	0.0%	0.0%	0.0%	0.0%	0.0%
tr|I3LM60|I3LM60	1	0	0	0	0	0	0	0	1	0	0	0	0	0	0	0	3.2%	0.0%	0.0%	0.0%	0.0%	0.0%	0.0%	0.0%
tr|F1SD67|F1SD67	1	0	0	0	0	0	0	0	1	0	0	0	0	0	0	0	1.4%	0.0%	0.0%	0.0%	0.0%	0.0%	0.0%	0.0%
tr|F1SMU6|F1SMU6	0	1	0	0	0	0	0	0	0	1	0	0	0	0	0	0	0.0%	1.3%	0.0%	0.0%	0.0%	0.0%	0.0%	0.0%
tr|I3LKY5|I3LKY5	0	1	0	0	0	0	0	0	0	1	0	0	0	0	0	0	0.0%	2.9%	0.0%	0.0%	0.0%	0.0%	0.0%	0.0%
tr|K7GKA2|K7GKA2	0	1	0	0	0	0	0	0	0	1	0	0	0	0	0	0	0.0%	1.2%	0.0%	0.0%	0.0%	0.0%	0.0%	0.0%
Reverse_tr|F1S360|F1S360	1	0	0	0	0	0	0	0	1	0	0	0	0	0	0	0	5.3%	0.0%	0.0%	0.0%	0.0%	0.0%	0.0%	0.0%
tr|I3L7J5|I3L7J5	1	0	0	0	0	0	0	0	1	0	0	0	0	0	0	0	15.7%	0.0%	0.0%	0.0%	0.0%	0.0%	0.0%	0.0%
tr|I3L6N3|I3L6N3	1	0	0	0	0	0	0	0	1	0	0	0	0	0	0	0	15.0%	0.0%	0.0%	0.0%	0.0%	0.0%	0.0%	0.0%
tr|F1S9F0|F1S9F0	0	1	0	0	0	0	0	0	0	1	0	0	0	0	0	0	0.0%	6.2%	0.0%	0.0%	0.0%	0.0%	0.0%	0.0%
tr|F1S096|F1S096	1	0	0	0	0	0	0	0	1	0	0	0	0	0	0	0	5.8%	0.0%	0.0%	0.0%	0.0%	0.0%	0.0%	0.0%
tr|F1RF13|F1RF13	1	0	0	0	0	0	0	0	1	0	0	0	0	0	0	0	1.9%	0.0%	0.0%	0.0%	0.0%	0.0%	0.0%	0.0%
tr|I3L8F7|I3L8F7	1	0	0	0	0	0	0	0	1	0	0	0	0	0	0	0	0.9%	0.0%	0.0%	0.0%	0.0%	0.0%	0.0%	0.0%
tr|I3LF96|I3LF96	0	1	0	0	0	0	0	0	0	1	0	0	0	0	0	0	0.0%	41.8%	0.0%	0.0%	0.0%	0.0%	0.0%	0.0%
tr|F1S3W7|F1S3W7	1	0	0	0	0	0	0	0	1	0	0	0	0	0	0	0	5.0%	0.0%	0.0%	0.0%	0.0%	0.0%	0.0%	0.0%
tr|I3LET5|I3LET5	1	0	0	0	0	0	0	0	1	0	0	0	0	0	0	0	2.0%	0.0%	0.0%	0.0%	0.0%	0.0%	0.0%	0.0%
tr|F1RJQ1|F1RJQ1	1	0	0	0	0	0	0	0	1	0	0	0	0	0	0	0	5.2%	0.0%	0.0%	0.0%	0.0%	0.0%	0.0%	0.0%

	Peptide Count								Spectral Count								Sequence Coverage							
	Whole Cell	Whole Cell	Whole Cell	Cilia Fraction	Cilia Fraction	Cilia Fraction	Cilia Fraction	Cilia Fraction	Whole Cell	Whole Cell	Whole Cell	Cilia Fraction	Cilia Fraction	Cilia Fraction	Cilia Fraction	Cilia Fraction	Whole Cell	Whole Cell	Whole Cell	Cilia Fraction	Cilia Fraction	Cilia Fraction	Cilia Fraction	Cilia Fraction
Sample#	1	2	6	4	3	5	7	8	1	2	6	4	3	5	7	8	1	2	6	4	3	5	7	8
**Accession**																								
tr|F1SHU7|F1SHU7	1	0	0	0	0	0	0	0	1	0	0	0	0	0	0	0	0.8%	0.0%	0.0%	0.0%	0.0%	0.0%	0.0%	0.0%
tr|I3LFD4|I3LFD4	1	0	0	0	0	0	0	0	1	0	0	0	0	0	0	0	2.9%	0.0%	0.0%	0.0%	0.0%	0.0%	0.0%	0.0%
tr|F1S847|F1S847	1	0	0	0	0	0	0	0	1	0	0	0	0	0	0	0	2.4%	0.0%	0.0%	0.0%	0.0%	0.0%	0.0%	0.0%
Reverse_sp|Q29126|WAP3	0	1	0	0	0	0	0	0	0	1	0	0	0	0	0	0	0.0%	8.3%	0.0%	0.0%	0.0%	0.0%	0.0%	0.0%
tr|D2IE28|D2IE28	0	1	0	0	0	0	0	0	0	1	0	0	0	0	0	0	0.0%	2.5%	0.0%	0.0%	0.0%	0.0%	0.0%	0.0%
tr|F1RIY7|F1RIY7	1	0	0	0	0	0	0	0	1	0	0	0	0	0	0	0	2.1%	0.0%	0.0%	0.0%	0.0%	0.0%	0.0%	0.0%
tr|K7GMJ9|K7GMJ9	1	0	0	0	0	0	0	0	1	0	0	0	0	0	0	0	13.3%	0.0%	0.0%	0.0%	0.0%	0.0%	0.0%	0.0%
tr|F1RH71|F1RH71	1	0	0	0	0	0	0	0	1	0	0	0	0	0	0	0	11.8%	0.0%	0.0%	0.0%	0.0%	0.0%	0.0%	0.0%
tr|I3L924|I3L924	1	0	0	0	0	0	0	0	1	0	0	0	0	0	0	0	8.6%	0.0%	0.0%	0.0%	0.0%	0.0%	0.0%	0.0%
tr|F1ST74|F1ST74	1	0	0	0	0	0	0	0	1	0	0	0	0	0	0	0	2.1%	0.0%	0.0%	0.0%	0.0%	0.0%	0.0%	0.0%
tr|F1RZC3|F1RZC3	0	1	0	0	0	0	0	0	0	1	0	0	0	0	0	0	0.0%	2.0%	0.0%	0.0%	0.0%	0.0%	0.0%	0.0%
tr|I3LPG3|I3LPG3	1	0	0	0	0	0	0	0	1	0	0	0	0	0	0	0	4.6%	0.0%	0.0%	0.0%	0.0%	0.0%	0.0%	0.0%
tr|F1SJV0|F1SJV0	1	0	0	0	0	0	0	0	1	0	0	0	0	0	0	0	2.1%	0.0%	0.0%	0.0%	0.0%	0.0%	0.0%	0.0%
tr|I3LTT0|I3LTT0	1	0	0	0	0	0	0	0	1	0	0	0	0	0	0	0	4.9%	0.0%	0.0%	0.0%	0.0%	0.0%	0.0%	0.0%
tr|F1RHK0|F1RHK0	1	0	0	0	0	0	0	0	1	0	0	0	0	0	0	0	5.2%	0.0%	0.0%	0.0%	0.0%	0.0%	0.0%	0.0%
tr|F1RIH7|F1RIH7	1	0	0	0	0	0	0	0	1	0	0	0	0	0	0	0	4.2%	0.0%	0.0%	0.0%	0.0%	0.0%	0.0%	0.0%
tr|K7GRM4|K7GRM4	1	0	0	0	0	0	0	0	1	0	0	0	0	0	0	0	6.7%	0.0%	0.0%	0.0%	0.0%	0.0%	0.0%	0.0%
tr|I3LVG1|I3LVG1	1	0	0	0	0	0	0	0	1	0	0	0	0	0	0	0	1.3%	0.0%	0.0%	0.0%	0.0%	0.0%	0.0%	0.0%
tr|I3LA74|I3LA74	1	0	0	0	0	0	0	0	1	0	0	0	0	0	0	0	3.3%	0.0%	0.0%	0.0%	0.0%	0.0%	0.0%	0.0%
tr|K7GNR9|K7GNR9	1	0	0	0	0	0	0	0	1	0	0	0	0	0	0	0	6.0%	0.0%	0.0%	0.0%	0.0%	0.0%	0.0%	0.0%
tr|F1S203|F1S203	1	0	0	0	0	0	0	0	1	0	0	0	0	0	0	0	1.4%	0.0%	0.0%	0.0%	0.0%	0.0%	0.0%	0.0%
tr|F1RKW4|F1RKW4	1	0	0	0	0	0	0	0	1	0	0	0	0	0	0	0	1.5%	0.0%	0.0%	0.0%	0.0%	0.0%	0.0%	0.0%
tr|F1SN94|F1SN94	1	0	0	0	0	0	0	0	1	0	0	0	0	0	0	0	3.6%	0.0%	0.0%	0.0%	0.0%	0.0%	0.0%	0.0%
tr|I3LSP2|I3LSP2	1	0	0	0	0	0	0	0	1	0	0	0	0	0	0	0	1.5%	0.0%	0.0%	0.0%	0.0%	0.0%	0.0%	0.0%
tr|I3LAD4|I3LAD4	1	0	0	0	0	0	0	0	1	0	0	0	0	0	0	0	6.2%	0.0%	0.0%	0.0%	0.0%	0.0%	0.0%	0.0%
tr|F1SDH3|F1SDH3	0	1	0	0	0	0	0	0	0	1	0	0	0	0	0	0	0.0%	1.2%	0.0%	0.0%	0.0%	0.0%	0.0%	0.0%
tr|F1RMY6|F1RMY6	1	0	0	0	0	0	0	0	1	0	0	0	0	0	0	0	2.8%	0.0%	0.0%	0.0%	0.0%	0.0%	0.0%	0.0%
tr|I3LF08|I3LF08	0	1	0	0	0	0	0	0	0	1	0	0	0	0	0	0	0.0%	7.6%	0.0%	0.0%	0.0%	0.0%	0.0%	0.0%
tr|I3L8G5|I3L8G5	1	0	0	0	0	0	0	0	1	0	0	0	0	0	0	0	8.3%	0.0%	0.0%	0.0%	0.0%	0.0%	0.0%	0.0%
tr|F1RMF1|F1RMF1	1	0	0	0	0	0	0	0	1	0	0	0	0	0	0	0	4.3%	0.0%	0.0%	0.0%	0.0%	0.0%	0.0%	0.0%
Reverse_tr|F1RIP8|F1RIP8	1	0	0	0	0	0	0	0	1	0	0	0	0	0	0	0	3.4%	0.0%	0.0%	0.0%	0.0%	0.0%	0.0%	0.0%
tr|F1S4G1|F1S4G1	1	0	0	0	0	0	0	0	1	0	0	0	0	0	0	0	3.7%	0.0%	0.0%	0.0%	0.0%	0.0%	0.0%	0.0%
tr|F1RLY9|F1RLY9	1	0	0	0	0	0	0	0	1	0	0	0	0	0	0	0	1.0%	0.0%	0.0%	0.0%	0.0%	0.0%	0.0%	0.0%
tr|I3LTE4|I3LTE4	1	0	0	0	0	0	0	0	1	0	0	0	0	0	0	0	36.4%	0.0%	0.0%	0.0%	0.0%	0.0%	0.0%	0.0%
tr|I3LKS0|I3LKS0	0	1	0	0	0	0	0	0	0	1	0	0	0	0	0	0	0.0%	3.0%	0.0%	0.0%	0.0%	0.0%	0.0%	0.0%
tr|I3LL40|I3LL40	1	0	0	0	0	0	0	0	1	0	0	0	0	0	0	0	8.6%	0.0%	0.0%	0.0%	0.0%	0.0%	0.0%	0.0%
tr|F1RUR6|F1RUR6	1	0	0	0	0	0	0	0	1	0	0	0	0	0	0	0	7.5%	0.0%	0.0%	0.0%	0.0%	0.0%	0.0%	0.0%
tr|F1S6R6|F1S6R6	1	0	0	0	0	0	0	0	1	0	0	0	0	0	0	0	4.9%	0.0%	0.0%	0.0%	0.0%	0.0%	0.0%	0.0%
tr|I3L7P0|I3L7P0	0	1	0	0	0	0	0	0	0	1	0	0	0	0	0	0	0.0%	4.6%	0.0%	0.0%	0.0%	0.0%	0.0%	0.0%
tr|F1SHV5|F1SHV5	0	1	0	0	0	0	0	0	0	1	0	0	0	0	0	0	0.0%	3.1%	0.0%	0.0%	0.0%	0.0%	0.0%	0.0%
tr|F1S0S8|F1S0S8	1	0	0	0	0	0	0	0	1	0	0	0	0	0	0	0	2.3%	0.0%	0.0%	0.0%	0.0%	0.0%	0.0%	0.0%
Reverse_tr|F1SPB1|F1SPB1	1	0	0	0	0	0	0	0	1	0	0	0	0	0	0	0	8.4%	0.0%	0.0%	0.0%	0.0%	0.0%	0.0%	0.0%
tr|I3L8P1|I3L8P1	1	0	0	0	0	0	0	0	1	0	0	0	0	0	0	0	4.1%	0.0%	0.0%	0.0%	0.0%	0.0%	0.0%	0.0%
tr|I3LS94|I3LS94	0	1	0	0	0	0	0	0	0	1	0	0	0	0	0	0	0.0%	2.8%	0.0%	0.0%	0.0%	0.0%	0.0%	0.0%
tr|I3LMZ3|I3LMZ3	1	0	0	0	0	0	0	0	1	0	0	0	0	0	0	0	6.4%	0.0%	0.0%	0.0%	0.0%	0.0%	0.0%	0.0%
tr|F1S7H5|F1S7H5	1	0	0	0	0	0	0	0	1	0	0	0	0	0	0	0	15.4%	0.0%	0.0%	0.0%	0.0%	0.0%	0.0%	0.0%
tr|F1RP70|F1RP70	1	0	0	0	0	0	0	0	1	0	0	0	0	0	0	0	1.4%	0.0%	0.0%	0.0%	0.0%	0.0%	0.0%	0.0%
tr|F1SIT5|F1SIT5	1	0	0	0	0	0	0	0	1	0	0	0	0	0	0	0	3.8%	0.0%	0.0%	0.0%	0.0%	0.0%	0.0%	0.0%
tr|F1SL48|F1SL48	1	0	0	0	0	0	0	0	1	0	0	0	0	0	0	0	4.2%	0.0%	0.0%	0.0%	0.0%	0.0%	0.0%	0.0%
tr|I3LKD1|I3LKD1	1	0	0	0	0	0	0	0	1	0	0	0	0	0	0	0	4.5%	0.0%	0.0%	0.0%	0.0%	0.0%	0.0%	0.0%
tr|F1SRA8|F1SRA8	1	0	0	0	0	0	0	0	1	0	0	0	0	0	0	0	0.8%	0.0%	0.0%	0.0%	0.0%	0.0%	0.0%	0.0%
tr|I3LGV6|I3LGV6	0	1	0	0	0	0	0	0	0	1	0	0	0	0	0	0	0.0%	1.3%	0.0%	0.0%	0.0%	0.0%	0.0%	0.0%
tr|I3LEI3|I3LEI3	1	0	0	0	0	0	0	0	1	0	0	0	0	0	0	0	7.6%	0.0%	0.0%	0.0%	0.0%	0.0%	0.0%	0.0%
tr|F1RIX8|F1RIX8	1	0	0	0	0	0	0	0	1	0	0	0	0	0	0	0	9.2%	0.0%	0.0%	0.0%	0.0%	0.0%	0.0%	0.0%
tr|F1RYM2|F1RYM2	1	0	0	0	0	0	0	0	1	0	0	0	0	0	0	0	1.0%	0.0%	0.0%	0.0%	0.0%	0.0%	0.0%	0.0%
Reverse_tr|F1SQ11|F1SQ11	1	0	0	0	0	0	0	0	1	0	0	0	0	0	0	0	1.3%	0.0%	0.0%	0.0%	0.0%	0.0%	0.0%	0.0%
tr|I3LAN7|I3LAN7	1	0	0	0	0	0	0	0	1	0	0	0	0	0	0	0	4.0%	0.0%	0.0%	0.0%	0.0%	0.0%	0.0%	0.0%
tr|F1RIA1|F1RIA1	1	0	0	0	0	0	0	0	1	0	0	0	0	0	0	0	12.7%	0.0%	0.0%	0.0%	0.0%	0.0%	0.0%	0.0%
tr|I3LQF3|I3LQF3	0	1	0	0	0	0	0	0	0	1	0	0	0	0	0	0	0.0%	2.3%	0.0%	0.0%	0.0%	0.0%	0.0%	0.0%
tr|F1RXT5|F1RXT5	1	0	0	0	0	0	0	0	1	0	0	0	0	0	0	0	1.1%	0.0%	0.0%	0.0%	0.0%	0.0%	0.0%	0.0%
tr|I3LG75|I3LG75	0	1	0	0	0	0	0	0	0	1	0	0	0	0	0	0	0.0%	4.5%	0.0%	0.0%	0.0%	0.0%	0.0%	0.0%
Reverse_tr|F1RP36|F1RP36	0	1	0	0	0	0	0	0	0	1	0	0	0	0	0	0	0.0%	3.6%	0.0%	0.0%	0.0%	0.0%	0.0%	0.0%
Reverse_tr|I3LRH0|I3LRH0	0	1	0	0	0	0	0	0	0	1	0	0	0	0	0	0	0.0%	0.9%	0.0%	0.0%	0.0%	0.0%	0.0%	0.0%
tr|F1S449|F1S449	1	0	0	0	0	0	0	0	1	0	0	0	0	0	0	0	3.0%	0.0%	0.0%	0.0%	0.0%	0.0%	0.0%	0.0%
tr|I3LFD9|I3LFD9	0	1	0	0	0	0	0	0	0	1	0	0	0	0	0	0	0.0%	1.1%	0.0%	0.0%	0.0%	0.0%	0.0%	0.0%
tr|F1SE54|F1SE54	1	0	0	0	0	0	0	0	1	0	0	0	0	0	0	0	2.9%	0.0%	0.0%	0.0%	0.0%	0.0%	0.0%	0.0%
tr|I3LQI7|I3LQI7	1	0	0	0	0	0	0	0	1	0	0	0	0	0	0	0	3.6%	0.0%	0.0%	0.0%	0.0%	0.0%	0.0%	0.0%
tr|F1RW11|F1RW11	1	0	0	0	0	0	0	0	1	0	0	0	0	0	0	0	4.6%	0.0%	0.0%	0.0%	0.0%	0.0%	0.0%	0.0%
tr|F1RX25|F1RX25	1	0	0	0	0	0	0	0	1	0	0	0	0	0	0	0	2.4%	0.0%	0.0%	0.0%	0.0%	0.0%	0.0%	0.0%
tr|I3LSS5|I3LSS5	1	0	0	0	0	0	0	0	1	0	0	0	0	0	0	0	15.3%	0.0%	0.0%	0.0%	0.0%	0.0%	0.0%	0.0%
tr|F1STJ9|F1STJ9	1	0	0	0	0	0	0	0	1	0	0	0	0	0	0	0	4.3%	0.0%	0.0%	0.0%	0.0%	0.0%	0.0%	0.0%
tr|I3LI39|I3LI39	1	0	0	0	0	0	0	0	1	0	0	0	0	0	0	0	13.1%	0.0%	0.0%	0.0%	0.0%	0.0%	0.0%	0.0%
tr|F1S759|F1S759	0	1	0	0	0	0	0	0	0	1	0	0	0	0	0	0	0.0%	3.8%	0.0%	0.0%	0.0%	0.0%	0.0%	0.0%
tr|F1S548|F1S548	1	0	0	0	0	0	0	0	1	0	0	0	0	0	0	0	10.6%	0.0%	0.0%	0.0%	0.0%	0.0%	0.0%	0.0%
tr|F1RT48|F1RT48	1	0	0	0	0	0	0	0	1	0	0	0	0	0	0	0	1.4%	0.0%	0.0%	0.0%	0.0%	0.0%	0.0%	0.0%
tr|F1RGX1|F1RGX1	0	1	0	0	0	0	0	0	0	1	0	0	0	0	0	0	0.0%	11.8%	0.0%	0.0%	0.0%	0.0%	0.0%	0.0%
tr|K7GLF1|K7GLF1	1	0	0	0	0	0	0	0	1	0	0	0	0	0	0	0	45.8%	0.0%	0.0%	0.0%	0.0%	0.0%	0.0%	0.0%
P50584	1	0	0	0	0	0	0	0	1	0	0	0	0	0	0	0	9.5%	0.0%	0.0%	0.0%	0.0%	0.0%	0.0%	0.0%
tr|F1SQU7|F1SQU7	1	0	0	0	0	0	0	0	1	0	0	0	0	0	0	0	11.0%	0.0%	0.0%	0.0%	0.0%	0.0%	0.0%	0.0%
tr|F1RXB9|F1RXB9	0	1	0	0	0	0	0	0	0	1	0	0	0	0	0	0	0.0%	10.0%	0.0%	0.0%	0.0%	0.0%	0.0%	0.0%
tr|I3LBZ1|I3LBZ1	1	0	0	0	0	0	0	0	1	0	0	0	0	0	0	0	14.7%	0.0%	0.0%	0.0%	0.0%	0.0%	0.0%	0.0%

	Peptide Count								Spectral Count								Sequence Coverage							
	Whole Cell	Whole Cell	Whole Cell	Cilia Fraction	Cilia Fraction	Cilia Fraction	Cilia Fraction	Cilia Fraction	Whole Cell	Whole Cell	Whole Cell	Cilia Fraction	Cilia Fraction	Cilia Fraction	Cilia Fraction	Cilia Fraction	Whole Cell	Whole Cell	Whole Cell	Cilia Fraction	Cilia Fraction	Cilia Fraction	Cilia Fraction	Cilia Fraction
Sample#	1	2	6	4	3	5	7	8	1	2	6	4	3	5	7	8	1	2	6	4	3	5	7	8
**Accession**																								
tr|K7GN18|K7GN18	1	0	0	0	0	0	0	0	1	0	0	0	0	0	0	0	9.3%	0.0%	0.0%	0.0%	0.0%	0.0%	0.0%	0.0%
tr|I3LNX7|I3LNX7	1	0	0	0	0	0	0	0	1	0	0	0	0	0	0	0	6.4%	0.0%	0.0%	0.0%	0.0%	0.0%	0.0%	0.0%
tr|I3LAX4|I3LAX4	1	0	0	0	0	0	0	0	1	0	0	0	0	0	0	0	5.4%	0.0%	0.0%	0.0%	0.0%	0.0%	0.0%	0.0%
tr|F1SJG3|F1SJG3	0	1	0	0	0	0	0	0	0	1	0	0	0	0	0	0	0.0%	8.7%	0.0%	0.0%	0.0%	0.0%	0.0%	0.0%
tr|I3LMK3|I3LMK3	0	1	0	0	0	0	0	0	0	1	0	0	0	0	0	0	0.0%	6.8%	0.0%	0.0%	0.0%	0.0%	0.0%	0.0%
Reverse_tr|F1S8Y2|F1S8Y2	0	1	0	0	0	0	0	0	0	1	0	0	0	0	0	0	0.0%	1.8%	0.0%	0.0%	0.0%	0.0%	0.0%	0.0%
tr|F1S5B2|F1S5B2	1	0	0	0	0	0	0	0	1	0	0	0	0	0	0	0	7.6%	0.0%	0.0%	0.0%	0.0%	0.0%	0.0%	0.0%
tr|I3LB11|I3LB11	1	0	0	0	0	0	0	0	1	0	0	0	0	0	0	0	1.6%	0.0%	0.0%	0.0%	0.0%	0.0%	0.0%	0.0%
tr|K7GN09|K7GN09	1	0	0	0	0	0	0	0	1	0	0	0	0	0	0	0	2.1%	0.0%	0.0%	0.0%	0.0%	0.0%	0.0%	0.0%
tr|F1SBX4|F1SBX4	1	0	0	0	0	0	0	0	1	0	0	0	0	0	0	0	6.0%	0.0%	0.0%	0.0%	0.0%	0.0%	0.0%	0.0%
tr|F1RIY6|F1RIY6	1	0	0	0	0	0	0	0	1	0	0	0	0	0	0	0	6.6%	0.0%	0.0%	0.0%	0.0%	0.0%	0.0%	0.0%
tr|I3LFZ4|I3LFZ4	1	0	0	0	0	0	0	0	1	0	0	0	0	0	0	0	3.3%	0.0%	0.0%	0.0%	0.0%	0.0%	0.0%	0.0%
tr|F1S451|F1S451	1	0	0	0	0	0	0	0	1	0	0	0	0	0	0	0	7.2%	0.0%	0.0%	0.0%	0.0%	0.0%	0.0%	0.0%
tr|F1SD77|F1SD77	1	0	0	0	0	0	0	0	1	0	0	0	0	0	0	0	10.2%	0.0%	0.0%	0.0%	0.0%	0.0%	0.0%	0.0%
tr|F1SCS4|F1SCS4	0	1	0	0	0	0	0	0	0	1	0	0	0	0	0	0	0.0%	1.3%	0.0%	0.0%	0.0%	0.0%	0.0%	0.0%
tr|I3LTZ9|I3LTZ9	1	0	0	0	0	0	0	0	1	0	0	0	0	0	0	0	21.1%	0.0%	0.0%	0.0%	0.0%	0.0%	0.0%	0.0%
tr|F1SC67|F1SC67	1	0	0	0	0	0	0	0	1	0	0	0	0	0	0	0	1.5%	0.0%	0.0%	0.0%	0.0%	0.0%	0.0%	0.0%
tr|I3L7K4|I3L7K4	0	1	0	0	0	0	0	0	0	1	0	0	0	0	0	0	0.0%	3.0%	0.0%	0.0%	0.0%	0.0%	0.0%	0.0%
tr|F1S0Y7|F1S0Y7	0	1	0	0	0	0	0	0	0	1	0	0	0	0	0	0	0.0%	4.8%	0.0%	0.0%	0.0%	0.0%	0.0%	0.0%
tr|F1SN83|F1SN83	1	0	0	0	0	0	0	0	1	0	0	0	0	0	0	0	2.7%	0.0%	0.0%	0.0%	0.0%	0.0%	0.0%	0.0%
tr|K7GP56|K7GP56	1	0	0	0	0	0	0	0	1	0	0	0	0	0	0	0	29.4%	0.0%	0.0%	0.0%	0.0%	0.0%	0.0%	0.0%
tr|B6CVL4|B6CVL4	1	0	0	0	0	0	0	0	1	0	0	0	0	0	0	0	1.3%	0.0%	0.0%	0.0%	0.0%	0.0%	0.0%	0.0%
tr|I3LE25|I3LE25	1	0	0	0	0	0	0	0	1	0	0	0	0	0	0	0	2.4%	0.0%	0.0%	0.0%	0.0%	0.0%	0.0%	0.0%
tr|F1SH87|F1SH87	1	0	0	0	0	0	0	0	1	0	0	0	0	0	0	0	3.9%	0.0%	0.0%	0.0%	0.0%	0.0%	0.0%	0.0%
tr|F1S283|F1S283	1	0	0	0	0	0	0	0	1	0	0	0	0	0	0	0	7.7%	0.0%	0.0%	0.0%	0.0%	0.0%	0.0%	0.0%
tr|I3LGA8|I3LGA8	1	0	0	0	0	0	0	0	1	0	0	0	0	0	0	0	4.6%	0.0%	0.0%	0.0%	0.0%	0.0%	0.0%	0.0%
tr|F1S213|F1S213	0	1	0	0	0	0	0	0	0	1	0	0	0	0	0	0	0.0%	4.8%	0.0%	0.0%	0.0%	0.0%	0.0%	0.0%
tr|F1SI25|F1SI25	1	0	0	0	0	0	0	0	1	0	0	0	0	0	0	0	5.4%	0.0%	0.0%	0.0%	0.0%	0.0%	0.0%	0.0%
tr|F1S7T6|F1S7T6	1	0	0	0	0	0	0	0	1	0	0	0	0	0	0	0	6.8%	0.0%	0.0%	0.0%	0.0%	0.0%	0.0%	0.0%
tr|I3L751|I3L751	1	0	0	0	0	0	0	0	1	0	0	0	0	0	0	0	5.2%	0.0%	0.0%	0.0%	0.0%	0.0%	0.0%	0.0%
tr|F1RNH1|F1RNH1	0	1	0	0	0	0	0	0	0	1	0	0	0	0	0	0	0.0%	3.9%	0.0%	0.0%	0.0%	0.0%	0.0%	0.0%
tr|F1S5P5|F1S5P5	1	0	0	0	0	0	0	0	1	0	0	0	0	0	0	0	1.9%	0.0%	0.0%	0.0%	0.0%	0.0%	0.0%	0.0%
tr|F1RTP5|F1RTP5	1	0	0	0	0	0	0	0	1	0	0	0	0	0	0	0	1.6%	0.0%	0.0%	0.0%	0.0%	0.0%	0.0%	0.0%
tr|F1S478|F1S478	0	1	0	0	0	0	0	0	0	1	0	0	0	0	0	0	0.0%	4.2%	0.0%	0.0%	0.0%	0.0%	0.0%	0.0%
tr|K7GMW4|K7GMW4	1	0	0	0	0	0	0	0	1	0	0	0	0	0	0	0	2.3%	0.0%	0.0%	0.0%	0.0%	0.0%	0.0%	0.0%
tr|I3LS01|I3LS01	0	1	0	0	0	0	0	0	0	1	0	0	0	0	0	0	0.0%	0.9%	0.0%	0.0%	0.0%	0.0%	0.0%	0.0%
tr|I3LI96|I3LI96	1	0	0	0	0	0	0	0	1	0	0	0	0	0	0	0	5.3%	0.0%	0.0%	0.0%	0.0%	0.0%	0.0%	0.0%
tr|K7GKY4|K7GKY4	1	0	0	0	0	0	0	0	1	0	0	0	0	0	0	0	7.0%	0.0%	0.0%	0.0%	0.0%	0.0%	0.0%	0.0%
tr|F1RMH4|F1RMH4	1	0	0	0	0	0	0	0	1	0	0	0	0	0	0	0	1.5%	0.0%	0.0%	0.0%	0.0%	0.0%	0.0%	0.0%
tr|F1S1C7|F1S1C7	1	0	0	0	0	0	0	0	1	0	0	0	0	0	0	0	8.2%	0.0%	0.0%	0.0%	0.0%	0.0%	0.0%	0.0%
tr|F1RGP9|F1RGP9	1	0	0	0	0	0	0	0	1	0	0	0	0	0	0	0	9.9%	0.0%	0.0%	0.0%	0.0%	0.0%	0.0%	0.0%
tr|F1RJY2|F1RJY2	1	0	0	0	0	0	0	0	1	0	0	0	0	0	0	0	10.5%	0.0%	0.0%	0.0%	0.0%	0.0%	0.0%	0.0%
tr|F1RU17|F1RU17	1	0	0	0	0	0	0	0	1	0	0	0	0	0	0	0	3.7%	0.0%	0.0%	0.0%	0.0%	0.0%	0.0%	0.0%
tr|F1RN84|F1RN84	1	0	0	0	0	0	0	0	1	0	0	0	0	0	0	0	0.7%	0.0%	0.0%	0.0%	0.0%	0.0%	0.0%	0.0%
tr|F1RUU2|F1RUU2	1	0	0	0	0	0	0	0	1	0	0	0	0	0	0	0	10.4%	0.0%	0.0%	0.0%	0.0%	0.0%	0.0%	0.0%
tr|F1SD84|F1SD84	1	0	0	0	0	0	0	0	1	0	0	0	0	0	0	0	6.6%	0.0%	0.0%	0.0%	0.0%	0.0%	0.0%	0.0%
tr|F1SA24|F1SA24	1	0	0	0	0	0	0	0	1	0	0	0	0	0	0	0	3.4%	0.0%	0.0%	0.0%	0.0%	0.0%	0.0%	0.0%
Q9MZU4	1	0	0	0	0	0	0	0	1	0	0	0	0	0	0	0	7.7%	0.0%	0.0%	0.0%	0.0%	0.0%	0.0%	0.0%
tr|K7GSM8|K7GSM8	1	0	0	0	0	0	0	0	1	0	0	0	0	0	0	0	9.8%	0.0%	0.0%	0.0%	0.0%	0.0%	0.0%	0.0%
tr|I3LSI7|I3LSI7	1	0	0	0	0	0	0	0	1	0	0	0	0	0	0	0	4.8%	0.0%	0.0%	0.0%	0.0%	0.0%	0.0%	0.0%
Reverse_tr|F1RG65|F1RG65	1	0	0	0	0	0	0	0	1	0	0	0	0	0	0	0	1.4%	0.0%	0.0%	0.0%	0.0%	0.0%	0.0%	0.0%
tr|F1SDX6|F1SDX6	1	0	0	0	0	0	0	0	1	0	0	0	0	0	0	0	1.7%	0.0%	0.0%	0.0%	0.0%	0.0%	0.0%	0.0%
tr|F1SA85|F1SA85	1	0	0	0	0	0	0	0	1	0	0	0	0	0	0	0	2.1%	0.0%	0.0%	0.0%	0.0%	0.0%	0.0%	0.0%
tr|F1RKX2|F1RKX2	1	0	0	0	0	0	0	0	1	0	0	0	0	0	0	0	5.1%	0.0%	0.0%	0.0%	0.0%	0.0%	0.0%	0.0%
tr|F1S6G5|F1S6G5	0	1	0	0	0	0	0	0	0	1	0	0	0	0	0	0	0.0%	9.8%	0.0%	0.0%	0.0%	0.0%	0.0%	0.0%
tr|F1SGJ4|F1SGJ4	1	0	0	0	0	0	0	0	1	0	0	0	0	0	0	0	5.0%	0.0%	0.0%	0.0%	0.0%	0.0%	0.0%	0.0%
tr|I3LGP5|I3LGP5	1	0	0	0	0	0	0	0	1	0	0	0	0	0	0	0	3.7%	0.0%	0.0%	0.0%	0.0%	0.0%	0.0%	0.0%
tr|I3LLQ1|I3LLQ1	0	1	0	0	0	0	0	0	0	1	0	0	0	0	0	0	0.0%	19.3%	0.0%	0.0%	0.0%	0.0%	0.0%	0.0%
tr|I3LGT4|I3LGT4	1	0	0	0	0	0	0	0	1	0	0	0	0	0	0	0	10.2%	0.0%	0.0%	0.0%	0.0%	0.0%	0.0%	0.0%
tr|F1SGJ1|F1SGJ1	0	1	0	0	0	0	0	0	0	1	0	0	0	0	0	0	0.0%	12.9%	0.0%	0.0%	0.0%	0.0%	0.0%	0.0%
tr|F1RZN9|F1RZN9	0	1	0	0	0	0	0	0	0	1	0	0	0	0	0	0	0.0%	5.9%	0.0%	0.0%	0.0%	0.0%	0.0%	0.0%
tr|I3L9K3|I3L9K3	1	0	0	0	0	0	0	0	1	0	0	0	0	0	0	0	1.2%	0.0%	0.0%	0.0%	0.0%	0.0%	0.0%	0.0%
tr|K7GRM1|K7GRM1	1	0	0	0	0	0	0	0	1	0	0	0	0	0	0	0	5.3%	0.0%	0.0%	0.0%	0.0%	0.0%	0.0%	0.0%
Reverse_tr|F1S4P1|F1S4P1	0	1	0	0	0	0	0	0	0	1	0	0	0	0	0	0	0.0%	0.3%	0.0%	0.0%	0.0%	0.0%	0.0%	0.0%
A5GZW8	1	0	0	0	0	0	0	0	1	0	0	0	0	0	0	0	6.3%	0.0%	0.0%	0.0%	0.0%	0.0%	0.0%	0.0%
tr|F1RSB9|F1RSB9	0	1	0	0	0	0	0	0	0	1	0	0	0	0	0	0	0.0%	4.6%	0.0%	0.0%	0.0%	0.0%	0.0%	0.0%
tr|F1RFR1|F1RFR1	1	0	0	0	0	0	0	0	1	0	0	0	0	0	0	0	2.0%	0.0%	0.0%	0.0%	0.0%	0.0%	0.0%	0.0%
tr|F1RL05|F1RL05	1	0	0	0	0	0	0	0	1	0	0	0	0	0	0	0	3.1%	0.0%	0.0%	0.0%	0.0%	0.0%	0.0%	0.0%
tr|F1S1J8|F1S1J8	1	0	0	0	0	0	0	0	1	0	0	0	0	0	0	0	4.8%	0.0%	0.0%	0.0%	0.0%	0.0%	0.0%	0.0%
tr|I3L910|I3L910	1	0	0	0	0	0	0	0	1	0	0	0	0	0	0	0	26.2%	0.0%	0.0%	0.0%	0.0%	0.0%	0.0%	0.0%
tr|F1SR51|F1SR51	0	1	0	0	0	0	0	0	0	1	0	0	0	0	0	0	0.0%	4.1%	0.0%	0.0%	0.0%	0.0%	0.0%	0.0%
tr|F1RFB2|F1RFB2	0	1	0	0	0	0	0	0	0	1	0	0	0	0	0	0	0.0%	0.6%	0.0%	0.0%	0.0%	0.0%	0.0%	0.0%
tr|I3LSG8|I3LSG8	1	0	0	0	0	0	0	0	1	0	0	0	0	0	0	0	1.7%	0.0%	0.0%	0.0%	0.0%	0.0%	0.0%	0.0%
tr|K7GRT0|K7GRT0	1	0	0	0	0	0	0	0	1	0	0	0	0	0	0	0	10.7%	0.0%	0.0%	0.0%	0.0%	0.0%	0.0%	0.0%
tr|I3LVA1|I3LVA1	1	0	0	0	0	0	0	0	1	0	0	0	0	0	0	0	4.3%	0.0%	0.0%	0.0%	0.0%	0.0%	0.0%	0.0%
tr|F1SKJ0|F1SKJ0	1	0	0	0	0	0	0	0	1	0	0	0	0	0	0	0	3.9%	0.0%	0.0%	0.0%	0.0%	0.0%	0.0%	0.0%
tr|F1SGL8|F1SGL8	1	0	0	0	0	0	0	0	1	0	0	0	0	0	0	0	3.5%	0.0%	0.0%	0.0%	0.0%	0.0%	0.0%	0.0%
tr|K7GT13|K7GT13	0	1	0	0	0	0	0	0	0	1	0	0	0	0	0	0	0.0%	5.7%	0.0%	0.0%	0.0%	0.0%	0.0%	0.0%
tr|F1RYM5|F1RYM5	0	1	0	0	0	0	0	0	0	1	0	0	0	0	0	0	0.0%	2.6%	0.0%	0.0%	0.0%	0.0%	0.0%	0.0%
tr|F6Q0Q4|F6Q0Q4	0	1	0	0	0	0	0	0	0	1	0	0	0	0	0	0	0.0%	3.4%	0.0%	0.0%	0.0%	0.0%	0.0%	0.0%
Reverse_tr|I3LPD6|I3LPD6	1	0	0	0	0	0	0	0	1	0	0	0	0	0	0	0	0.5%	0.0%	0.0%	0.0%	0.0%	0.0%	0.0%	0.0%

	Peptide Count								Spectral Count								Sequence Coverage							
	Whole Cell	Whole Cell	Whole Cell	Cilia Fraction	Cilia Fraction	Cilia Fraction	Cilia Fraction	Cilia Fraction	Whole Cell	Whole Cell	Whole Cell	Cilia Fraction	Cilia Fraction	Cilia Fraction	Cilia Fraction	Cilia Fraction	Whole Cell	Whole Cell	Whole Cell	Cilia Fraction	Cilia Fraction	Cilia Fraction	Cilia Fraction	Cilia Fraction
Sample#	1	2	6	4	3	5	7	8	1	2	6	4	3	5	7	8	1	2	6	4	3	5	7	8
**Accession**																								
tr|F1STR8|F1STR8	1	0	0	0	0	0	0	0	1	0	0	0	0	0	0	0	2.9%	0.0%	0.0%	0.0%	0.0%	0.0%	0.0%	0.0%
Reverse_tr|F1S5P5|F1S5P5	1	0	0	0	0	0	0	0	1	0	0	0	0	0	0	0	2.7%	0.0%	0.0%	0.0%	0.0%	0.0%	0.0%	0.0%
tr|F1SVC8|F1SVC8	1	0	0	0	0	0	0	0	1	0	0	0	0	0	0	0	1.9%	0.0%	0.0%	0.0%	0.0%	0.0%	0.0%	0.0%
tr|F1SR84|F1SR84	1	0	0	0	0	0	0	0	1	0	0	0	0	0	0	0	1.1%	0.0%	0.0%	0.0%	0.0%	0.0%	0.0%	0.0%
tr|F1SLG6|F1SLG6	0	1	0	0	0	0	0	0	0	1	0	0	0	0	0	0	0.0%	4.7%	0.0%	0.0%	0.0%	0.0%	0.0%	0.0%
Reverse_tr|F1RM51|F1RM51	0	1	0	0	0	0	0	0	0	1	0	0	0	0	0	0	0.0%	3.8%	0.0%	0.0%	0.0%	0.0%	0.0%	0.0%
tr|F1S7C3|F1S7C3	1	0	0	0	0	0	0	0	1	0	0	0	0	0	0	0	2.4%	0.0%	0.0%	0.0%	0.0%	0.0%	0.0%	0.0%
tr|F1RJI2|F1RJI2	1	0	0	0	0	0	0	0	1	0	0	0	0	0	0	0	5.5%	0.0%	0.0%	0.0%	0.0%	0.0%	0.0%	0.0%
tr|I3LE86|I3LE86	0	1	0	0	0	0	0	0	0	1	0	0	0	0	0	0	0.0%	1.6%	0.0%	0.0%	0.0%	0.0%	0.0%	0.0%
tr|F1S455|F1S455	1	0	0	0	0	0	0	0	1	0	0	0	0	0	0	0	4.6%	0.0%	0.0%	0.0%	0.0%	0.0%	0.0%	0.0%
tr|I3LGG8|I3LGG8	1	0	0	0	0	0	0	0	1	0	0	0	0	0	0	0	2.0%	0.0%	0.0%	0.0%	0.0%	0.0%	0.0%	0.0%
tr|F1RGB5|F1RGB5	1	0	0	0	0	0	0	0	1	0	0	0	0	0	0	0	0.6%	0.0%	0.0%	0.0%	0.0%	0.0%	0.0%	0.0%
tr|F1SH38|F1SH38	1	0	0	0	0	0	0	0	1	0	0	0	0	0	0	0	1.0%	0.0%	0.0%	0.0%	0.0%	0.0%	0.0%	0.0%
tr|I3LLC0|I3LLC0	0	1	0	0	0	0	0	0	0	1	0	0	0	0	0	0	0.0%	1.7%	0.0%	0.0%	0.0%	0.0%	0.0%	0.0%
tr|F1S6K3|F1S6K3	0	1	0	0	0	0	0	0	0	1	0	0	0	0	0	0	0.0%	2.7%	0.0%	0.0%	0.0%	0.0%	0.0%	0.0%
tr|F1SJJ3|F1SJJ3	1	0	0	0	0	0	0	0	1	0	0	0	0	0	0	0	3.0%	0.0%	0.0%	0.0%	0.0%	0.0%	0.0%	0.0%
tr|F2Z5J3|F2Z5J3	1	0	0	0	0	0	0	0	1	0	0	0	0	0	0	0	9.3%	0.0%	0.0%	0.0%	0.0%	0.0%	0.0%	0.0%
tr|F1RLE8|F1RLE8	1	0	0	0	0	0	0	0	1	0	0	0	0	0	0	0	3.1%	0.0%	0.0%	0.0%	0.0%	0.0%	0.0%	0.0%
Reverse_tr|I3LPJ0|I3LPJ0	1	0	0	0	0	0	0	0	1	0	0	0	0	0	0	0	6.1%	0.0%	0.0%	0.0%	0.0%	0.0%	0.0%	0.0%
tr|F1RFL7|F1RFL7	1	0	0	0	0	0	0	0	1	0	0	0	0	0	0	0	7.8%	0.0%	0.0%	0.0%	0.0%	0.0%	0.0%	0.0%
tr|F1S5F4|F1S5F4	1	0	0	0	0	0	0	0	1	0	0	0	0	0	0	0	8.7%	0.0%	0.0%	0.0%	0.0%	0.0%	0.0%	0.0%
tr|I3LJH2|I3LJH2	0	1	0	0	0	0	0	0	0	1	0	0	0	0	0	0	0.0%	16.5%	0.0%	0.0%	0.0%	0.0%	0.0%	0.0%
tr|I3LLY2|I3LLY2	1	0	0	0	0	0	0	0	1	0	0	0	0	0	0	0	3.6%	0.0%	0.0%	0.0%	0.0%	0.0%	0.0%	0.0%
tr|I3L971|I3L971	0	1	0	0	0	0	0	0	0	1	0	0	0	0	0	0	0.0%	8.0%	0.0%	0.0%	0.0%	0.0%	0.0%	0.0%
tr|F1RXC7|F1RXC7	1	0	0	0	0	0	0	0	1	0	0	0	0	0	0	0	1.8%	0.0%	0.0%	0.0%	0.0%	0.0%	0.0%	0.0%
tr|I3LQI1|I3LQI1	0	1	0	0	0	0	0	0	0	1	0	0	0	0	0	0	0.0%	2.0%	0.0%	0.0%	0.0%	0.0%	0.0%	0.0%
O19071	0	1	0	0	0	0	0	0	0	1	0	0	0	0	0	0	0.0%	4.5%	0.0%	0.0%	0.0%	0.0%	0.0%	0.0%
tr|F1RQG8|F1RQG8	1	0	0	0	0	0	0	0	1	0	0	0	0	0	0	0	2.0%	0.0%	0.0%	0.0%	0.0%	0.0%	0.0%	0.0%
tr|I3LLX2|I3LLX2	0	1	0	0	0	0	0	0	0	1	0	0	0	0	0	0	0.0%	0.7%	0.0%	0.0%	0.0%	0.0%	0.0%	0.0%
tr|F1SFG2|F1SFG2	1	0	0	0	0	0	0	0	1	0	0	0	0	0	0	0	2.3%	0.0%	0.0%	0.0%	0.0%	0.0%	0.0%	0.0%
tr|I3L9Z3|I3L9Z3	1	0	0	0	0	0	0	0	1	0	0	0	0	0	0	0	2.6%	0.0%	0.0%	0.0%	0.0%	0.0%	0.0%	0.0%
Q5H7C0	0	1	0	0	0	0	0	0	0	1	0	0	0	0	0	0	0.0%	3.2%	0.0%	0.0%	0.0%	0.0%	0.0%	0.0%
Reverse_tr|I3L9T1|I3L9T1	1	0	0	0	0	0	0	0	1	0	0	0	0	0	0	0	0.5%	0.0%	0.0%	0.0%	0.0%	0.0%	0.0%	0.0%
tr|I3L8H1|I3L8H1	1	0	0	0	0	0	0	0	1	0	0	0	0	0	0	0	18.9%	0.0%	0.0%	0.0%	0.0%	0.0%	0.0%	0.0%
tr|I3LUQ0|I3LUQ0	0	1	0	0	0	0	0	0	0	1	0	0	0	0	0	0	0.0%	9.9%	0.0%	0.0%	0.0%	0.0%	0.0%	0.0%
tr|F1RK19|F1RK19	1	0	0	0	0	0	0	0	1	0	0	0	0	0	0	0	2.3%	0.0%	0.0%	0.0%	0.0%	0.0%	0.0%	0.0%
tr|F1SHQ3|F1SHQ3	1	0	0	0	0	0	0	0	1	0	0	0	0	0	0	0	0.8%	0.0%	0.0%	0.0%	0.0%	0.0%	0.0%	0.0%
Reverse_tr|F1SHM1|F1SHM1	1	0	0	0	0	0	0	0	1	0	0	0	0	0	0	0	3.7%	0.0%	0.0%	0.0%	0.0%	0.0%	0.0%	0.0%
tr|I3LQX0|I3LQX0	1	0	0	0	0	0	0	0	1	0	0	0	0	0	0	0	3.9%	0.0%	0.0%	0.0%	0.0%	0.0%	0.0%	0.0%
tr|K7GN41|K7GN41	1	0	0	0	0	0	0	0	1	0	0	0	0	0	0	0	10.9%	0.0%	0.0%	0.0%	0.0%	0.0%	0.0%	0.0%
tr|I3LIE2|I3LIE2	1	0	0	0	0	0	0	0	1	0	0	0	0	0	0	0	62.0%	0.0%	0.0%	0.0%	0.0%	0.0%	0.0%	0.0%
tr|K7GRS0|K7GRS0	1	0	0	0	0	0	0	0	1	0	0	0	0	0	0	0	5.2%	0.0%	0.0%	0.0%	0.0%	0.0%	0.0%	0.0%
tr|F1RHU1|F1RHU1	1	0	0	0	0	0	0	0	1	0	0	0	0	0	0	0	3.6%	0.0%	0.0%	0.0%	0.0%	0.0%	0.0%	0.0%
tr|F1RQT6|F1RQT6	1	0	0	0	0	0	0	0	1	0	0	0	0	0	0	0	1.7%	0.0%	0.0%	0.0%	0.0%	0.0%	0.0%	0.0%
tr|A5D9K9|A5D9K9	1	0	0	0	0	0	0	0	1	0	0	0	0	0	0	0	8.0%	0.0%	0.0%	0.0%	0.0%	0.0%	0.0%	0.0%
tr|F1SA03|F1SA03	1	0	0	0	0	0	0	0	1	0	0	0	0	0	0	0	2.2%	0.0%	0.0%	0.0%	0.0%	0.0%	0.0%	0.0%
tr|K7GS73|K7GS73	1	0	0	0	0	0	0	0	1	0	0	0	0	0	0	0	1.3%	0.0%	0.0%	0.0%	0.0%	0.0%	0.0%	0.0%
tr|F1RR03|F1RR03	0	1	0	0	0	0	0	0	0	1	0	0	0	0	0	0	0.0%	1.8%	0.0%	0.0%	0.0%	0.0%	0.0%	0.0%
tr|F1SE30|F1SE30	0	1	0	0	0	0	0	0	0	1	0	0	0	0	0	0	0.0%	0.6%	0.0%	0.0%	0.0%	0.0%	0.0%	0.0%
Q767L9	1	0	0	0	0	0	0	0	1	0	0	0	0	0	0	0	7.6%	0.0%	0.0%	0.0%	0.0%	0.0%	0.0%	0.0%
tr|I3L614|I3L614	1	0	0	0	0	0	0	0	1	0	0	0	0	0	0	0	8.3%	0.0%	0.0%	0.0%	0.0%	0.0%	0.0%	0.0%
tr|F1SJX2|F1SJX2	0	1	0	0	0	0	0	0	0	1	0	0	0	0	0	0	0.0%	5.1%	0.0%	0.0%	0.0%	0.0%	0.0%	0.0%
tr|F1RUP6|F1RUP6	1	0	0	0	0	0	0	0	1	0	0	0	0	0	0	0	7.8%	0.0%	0.0%	0.0%	0.0%	0.0%	0.0%	0.0%
tr|I3L9T3|I3L9T3	1	0	0	0	0	0	0	0	1	0	0	0	0	0	0	0	9.7%	0.0%	0.0%	0.0%	0.0%	0.0%	0.0%	0.0%
tr|F1SLA1|F1SLA1	1	0	0	0	0	0	0	0	1	0	0	0	0	0	0	0	9.7%	0.0%	0.0%	0.0%	0.0%	0.0%	0.0%	0.0%
tr|I3L750|I3L750	1	0	0	0	0	0	0	0	1	0	0	0	0	0	0	0	18.5%	0.0%	0.0%	0.0%	0.0%	0.0%	0.0%	0.0%
tr|F1SLG3|F1SLG3	1	0	0	0	0	0	0	0	1	0	0	0	0	0	0	0	5.3%	0.0%	0.0%	0.0%	0.0%	0.0%	0.0%	0.0%
P32394	1	0	0	0	0	0	0	0	1	0	0	0	0	0	0	0	4.5%	0.0%	0.0%	0.0%	0.0%	0.0%	0.0%	0.0%
tr|F1RZC4|F1RZC4	0	1	0	0	0	0	0	0	0	1	0	0	0	0	0	0	0.0%	10.2%	0.0%	0.0%	0.0%	0.0%	0.0%	0.0%
tr|I3LNK4|I3LNK4	1	0	0	0	0	0	0	0	1	0	0	0	0	0	0	0	15.6%	0.0%	0.0%	0.0%	0.0%	0.0%	0.0%	0.0%
tr|F1RPM5|F1RPM5	1	0	0	0	0	0	0	0	1	0	0	0	0	0	0	0	3.3%	0.0%	0.0%	0.0%	0.0%	0.0%	0.0%	0.0%
tr|I3LC39|I3LC39	1	0	0	0	0	0	0	0	1	0	0	0	0	0	0	0	2.0%	0.0%	0.0%	0.0%	0.0%	0.0%	0.0%	0.0%
tr|K7GPP2|K7GPP2	1	0	0	0	0	0	0	0	1	0	0	0	0	0	0	0	9.9%	0.0%	0.0%	0.0%	0.0%	0.0%	0.0%	0.0%
A1YIZ1	1	0	0	0	0	0	0	0	1	0	0	0	0	0	0	0	5.5%	0.0%	0.0%	0.0%	0.0%	0.0%	0.0%	0.0%
tr|F1RTB9|F1RTB9	0	1	0	0	0	0	0	0	0	1	0	0	0	0	0	0	0.0%	1.8%	0.0%	0.0%	0.0%	0.0%	0.0%	0.0%
tr|F1SSM9|F1SSM9	1	0	0	0	0	0	0	0	1	0	0	0	0	0	0	0	1.6%	0.0%	0.0%	0.0%	0.0%	0.0%	0.0%	0.0%
tr|K7GQR2|K7GQR2	1	0	0	0	0	0	0	0	1	0	0	0	0	0	0	0	15.8%	0.0%	0.0%	0.0%	0.0%	0.0%	0.0%	0.0%
tr|F1RKC3|F1RKC3	0	1	0	0	0	0	0	0	0	1	0	0	0	0	0	0	0.0%	2.1%	0.0%	0.0%	0.0%	0.0%	0.0%	0.0%
Reverse_tr|F1RMY0|F1RMY0	0	1	0	0	0	0	0	0	0	1	0	0	0	0	0	0	0.0%	1.7%	0.0%	0.0%	0.0%	0.0%	0.0%	0.0%
tr|F1RG79|F1RG79	0	1	0	0	0	0	0	0	0	1	0	0	0	0	0	0	0.0%	1.1%	0.0%	0.0%	0.0%	0.0%	0.0%	0.0%
Reverse_tr|F1S086|F1S086	0	1	0	0	0	0	0	0	0	1	0	0	0	0	0	0	0.0%	2.9%	0.0%	0.0%	0.0%	0.0%	0.0%	0.0%
tr|F1SIT6|F1SIT6	1	0	0	0	0	0	0	0	1	0	0	0	0	0	0	0	3.3%	0.0%	0.0%	0.0%	0.0%	0.0%	0.0%	0.0%
tr|I3LNZ6|I3LNZ6	1	0	0	0	0	0	0	0	1	0	0	0	0	0	0	0	6.9%	0.0%	0.0%	0.0%	0.0%	0.0%	0.0%	0.0%
tr|F1S1L0|F1S1L0	1	0	0	0	0	0	0	0	1	0	0	0	0	0	0	0	4.1%	0.0%	0.0%	0.0%	0.0%	0.0%	0.0%	0.0%
tr|F1SDF8|F1SDF8	1	0	0	0	0	0	0	0	1	0	0	0	0	0	0	0	10.9%	0.0%	0.0%	0.0%	0.0%	0.0%	0.0%	0.0%
tr|I3LNK9|I3LNK9	1	0	0	0	0	0	0	0	1	0	0	0	0	0	0	0	2.2%	0.0%	0.0%	0.0%	0.0%	0.0%	0.0%	0.0%
tr|F1SST1|F1SST1	1	0	0	0	0	0	0	0	1	0	0	0	0	0	0	0	6.9%	0.0%	0.0%	0.0%	0.0%	0.0%	0.0%	0.0%
tr|F1RLI4|F1RLI4	0	1	0	0	0	0	0	0	0	1	0	0	0	0	0	0	0.0%	1.6%	0.0%	0.0%	0.0%	0.0%	0.0%	0.0%
tr|I3L9Y7|I3L9Y7	1	0	0	0	0	0	0	0	1	0	0	0	0	0	0	0	25.0%	0.0%	0.0%	0.0%	0.0%	0.0%	0.0%	0.0%
tr|I3LJL2|I3LJL2	1	0	0	0	0	0	0	0	1	0	0	0	0	0	0	0	5.7%	0.0%	0.0%	0.0%	0.0%	0.0%	0.0%	0.0%
tr|F1SDI9|F1SDI9	1	0	0	0	0	0	0	0	1	0	0	0	0	0	0	0	2.3%	0.0%	0.0%	0.0%	0.0%	0.0%	0.0%	0.0%

	Peptide Count								Spectral Count								Sequence Coverage							
	Whole Cell	Whole Cell	Whole Cell	Cilia Fraction	Cilia Fraction	Cilia Fraction	Cilia Fraction	Cilia Fraction	Whole Cell	Whole Cell	Whole Cell	Cilia Fraction	Cilia Fraction	Cilia Fraction	Cilia Fraction	Cilia Fraction	Whole Cell	Whole Cell	Whole Cell	Cilia Fraction	Cilia Fraction	Cilia Fraction	Cilia Fraction	Cilia Fraction
Sample#	1	2	6	4	3	5	7	8	1	2	6	4	3	5	7	8	1	2	6	4	3	5	7	8
**Accession**																								
tr|F1SIW3|F1SIW3	1	0	0	0	0	0	0	0	1	0	0	0	0	0	0	0	1.6%	0.0%	0.0%	0.0%	0.0%	0.0%	0.0%	0.0%
tr|I3LIB0|I3LIB0	1	0	0	0	0	0	0	0	1	0	0	0	0	0	0	0	30.9%	0.0%	0.0%	0.0%	0.0%	0.0%	0.0%	0.0%
tr|I3LFI1|I3LFI1	0	1	0	0	0	0	0	0	0	1	0	0	0	0	0	0	0.0%	0.4%	0.0%	0.0%	0.0%	0.0%	0.0%	0.0%
tr|I3LJK0|I3LJK0	1	0	0	0	0	0	0	0	1	0	0	0	0	0	0	0	22.6%	0.0%	0.0%	0.0%	0.0%	0.0%	0.0%	0.0%
tr|F2Z5N4|F2Z5N4	1	0	0	0	0	0	0	0	1	0	0	0	0	0	0	0	2.6%	0.0%	0.0%	0.0%	0.0%	0.0%	0.0%	0.0%
Q29333	0	1	0	0	0	0	0	0	0	1	0	0	0	0	0	0	0.0%	16.7%	0.0%	0.0%	0.0%	0.0%	0.0%	0.0%
tr|F1RLG2|F1RLG2	1	0	0	0	0	0	0	0	1	0	0	0	0	0	0	0	6.8%	0.0%	0.0%	0.0%	0.0%	0.0%	0.0%	0.0%
tr|I3LNL4|I3LNL4	1	0	0	0	0	0	0	0	1	0	0	0	0	0	0	0	24.7%	0.0%	0.0%	0.0%	0.0%	0.0%	0.0%	0.0%
Reverse_tr|I3LI00|I3LI00	0	1	0	0	0	0	0	0	0	1	0	0	0	0	0	0	0.0%	2.7%	0.0%	0.0%	0.0%	0.0%	0.0%	0.0%
tr|F1RQN5|F1RQN5	1	0	0	0	0	0	0	0	1	0	0	0	0	0	0	0	3.7%	0.0%	0.0%	0.0%	0.0%	0.0%	0.0%	0.0%
tr|F1RFL9|F1RFL9	1	0	0	0	0	0	0	0	1	0	0	0	0	0	0	0	1.5%	0.0%	0.0%	0.0%	0.0%	0.0%	0.0%	0.0%
tr|I3LBY5|I3LBY5	1	0	0	0	0	0	0	0	1	0	0	0	0	0	0	0	6.4%	0.0%	0.0%	0.0%	0.0%	0.0%	0.0%	0.0%
tr|F1RRH9|F1RRH9	1	0	0	0	0	0	0	0	1	0	0	0	0	0	0	0	4.0%	0.0%	0.0%	0.0%	0.0%	0.0%	0.0%	0.0%
Reverse_sp|O02696|PI3R5	1	0	0	0	0	0	0	0	1	0	0	0	0	0	0	0	2.4%	0.0%	0.0%	0.0%	0.0%	0.0%	0.0%	0.0%
tr|F1S444|F1S444	0	1	0	0	0	0	0	0	0	1	0	0	0	0	0	0	0.0%	3.5%	0.0%	0.0%	0.0%	0.0%	0.0%	0.0%
tr|F1SE04|F1SE04	0	1	0	0	0	0	0	0	0	1	0	0	0	0	0	0	0.0%	2.4%	0.0%	0.0%	0.0%	0.0%	0.0%	0.0%
tr|I3LVP6|I3LVP6	1	0	0	0	0	0	0	0	1	0	0	0	0	0	0	0	1.7%	0.0%	0.0%	0.0%	0.0%	0.0%	0.0%	0.0%
tr|I3LGM0|I3LGM0	1	0	0	0	0	0	0	0	1	0	0	0	0	0	0	0	13.3%	0.0%	0.0%	0.0%	0.0%	0.0%	0.0%	0.0%
tr|F1S513|F1S513	0	1	0	0	0	0	0	0	0	1	0	0	0	0	0	0	0.0%	1.5%	0.0%	0.0%	0.0%	0.0%	0.0%	0.0%
tr|I3LFC0|I3LFC0	1	0	0	0	0	0	0	0	1	0	0	0	0	0	0	0	23.1%	0.0%	0.0%	0.0%	0.0%	0.0%	0.0%	0.0%
tr|F1S604|F1S604	0	1	0	0	0	0	0	0	0	1	0	0	0	0	0	0	0.0%	8.0%	0.0%	0.0%	0.0%	0.0%	0.0%	0.0%
tr|I3LR60|I3LR60	1	0	0	0	0	0	0	0	1	0	0	0	0	0	0	0	6.7%	0.0%	0.0%	0.0%	0.0%	0.0%	0.0%	0.0%
tr|K7GMK7|K7GMK7	1	0	0	0	0	0	0	0	1	0	0	0	0	0	0	0	8.6%	0.0%	0.0%	0.0%	0.0%	0.0%	0.0%	0.0%
tr|F1SAI0|F1SAI0	1	0	0	0	0	0	0	0	1	0	0	0	0	0	0	0	1.7%	0.0%	0.0%	0.0%	0.0%	0.0%	0.0%	0.0%
tr|F1RMF7|F1RMF7	1	0	0	0	0	0	0	0	1	0	0	0	0	0	0	0	5.1%	0.0%	0.0%	0.0%	0.0%	0.0%	0.0%	0.0%
tr|I3LQR4|I3LQR4	1	0	0	0	0	0	0	0	1	0	0	0	0	0	0	0	6.2%	0.0%	0.0%	0.0%	0.0%	0.0%	0.0%	0.0%
tr|F1S0U0|F1S0U0	0	1	0	0	0	0	0	0	0	1	0	0	0	0	0	0	0.0%	1.4%	0.0%	0.0%	0.0%	0.0%	0.0%	0.0%
Reverse_tr|I3LB00|I3LB00	0	1	0	0	0	0	0	0	0	1	0	0	0	0	0	0	0.0%	9.5%	0.0%	0.0%	0.0%	0.0%	0.0%	0.0%
tr|F1SMQ7|F1SMQ7	0	1	0	0	0	0	0	0	0	1	0	0	0	0	0	0	0.0%	2.1%	0.0%	0.0%	0.0%	0.0%	0.0%	0.0%
tr|F1RJH9|F1RJH9	1	0	0	0	0	0	0	0	1	0	0	0	0	0	0	0	4.6%	0.0%	0.0%	0.0%	0.0%	0.0%	0.0%	0.0%
tr|I3LJA0|I3LJA0	0	1	0	0	0	0	0	0	0	1	0	0	0	0	0	0	0.0%	2.6%	0.0%	0.0%	0.0%	0.0%	0.0%	0.0%
tr|F1SRI4|F1SRI4	0	1	0	0	0	0	0	0	0	1	0	0	0	0	0	0	0.0%	0.6%	0.0%	0.0%	0.0%	0.0%	0.0%	0.0%
tr|F1S141|F1S141	0	1	0	0	0	0	0	0	0	1	0	0	0	0	0	0	0.0%	4.9%	0.0%	0.0%	0.0%	0.0%	0.0%	0.0%
tr|F1RX78|F1RX78	1	0	0	0	0	0	0	0	1	0	0	0	0	0	0	0	6.4%	0.0%	0.0%	0.0%	0.0%	0.0%	0.0%	0.0%
tr|I3L734|I3L734	0	1	0	0	0	0	0	0	0	1	0	0	0	0	0	0	0.0%	5.0%	0.0%	0.0%	0.0%	0.0%	0.0%	0.0%
tr|F1RH83|F1RH83	1	0	0	0	0	0	0	0	1	0	0	0	0	0	0	0	4.5%	0.0%	0.0%	0.0%	0.0%	0.0%	0.0%	0.0%
tr|F1SB84|F1SB84	1	0	0	0	0	0	0	0	1	0	0	0	0	0	0	0	5.7%	0.0%	0.0%	0.0%	0.0%	0.0%	0.0%	0.0%
Reverse_tr|F1SV12|F1SV12	1	0	0	0	0	0	0	0	1	0	0	0	0	0	0	0	2.0%	0.0%	0.0%	0.0%	0.0%	0.0%	0.0%	0.0%
tr|F1SGQ1|F1SGQ1	1	0	0	0	0	0	0	0	1	0	0	0	0	0	0	0	2.4%	0.0%	0.0%	0.0%	0.0%	0.0%	0.0%	0.0%
Reverse_tr|F1RWG7|F1RWG7	1	0	0	0	0	0	0	0	1	0	0	0	0	0	0	0	9.1%	0.0%	0.0%	0.0%	0.0%	0.0%	0.0%	0.0%
tr|I3LKQ5|I3LKQ5	1	0	0	0	0	0	0	0	1	0	0	0	0	0	0	0	7.2%	0.0%	0.0%	0.0%	0.0%	0.0%	0.0%	0.0%
tr|F1RJ87|F1RJ87	1	0	0	0	0	0	0	0	1	0	0	0	0	0	0	0	3.4%	0.0%	0.0%	0.0%	0.0%	0.0%	0.0%	0.0%
Reverse_tr|F1RYR0|F1RYR0	1	0	0	0	0	0	0	0	1	0	0	0	0	0	0	0	2.9%	0.0%	0.0%	0.0%	0.0%	0.0%	0.0%	0.0%
tr|I3L9D4|I3L9D4	1	0	0	0	0	0	0	0	1	0	0	0	0	0	0	0	13.1%	0.0%	0.0%	0.0%	0.0%	0.0%	0.0%	0.0%
tr|F1S4G2|F1S4G2	1	0	0	0	0	0	0	0	1	0	0	0	0	0	0	0	6.3%	0.0%	0.0%	0.0%	0.0%	0.0%	0.0%	0.0%
tr|I3L5P7|I3L5P7	1	0	0	0	0	0	0	0	1	0	0	0	0	0	0	0	12.5%	0.0%	0.0%	0.0%	0.0%	0.0%	0.0%	0.0%
tr|F1SPF7|F1SPF7	1	0	0	0	0	0	0	0	1	0	0	0	0	0	0	0	4.3%	0.0%	0.0%	0.0%	0.0%	0.0%	0.0%	0.0%
Reverse_tr|I3LKJ5|I3LKJ5	1	0	0	0	0	0	0	0	1	0	0	0	0	0	0	0	2.5%	0.0%	0.0%	0.0%	0.0%	0.0%	0.0%	0.0%
tr|F1RJZ4|F1RJZ4	1	0	0	0	0	0	0	0	1	0	0	0	0	0	0	0	3.2%	0.0%	0.0%	0.0%	0.0%	0.0%	0.0%	0.0%
tr|I3LQK1|I3LQK1	1	0	0	0	0	0	0	0	1	0	0	0	0	0	0	0	15.6%	0.0%	0.0%	0.0%	0.0%	0.0%	0.0%	0.0%
tr|Q767M2|Q767M2	1	0	0	0	0	0	0	0	1	0	0	0	0	0	0	0	4.5%	0.0%	0.0%	0.0%	0.0%	0.0%	0.0%	0.0%
tr|F1SMR7|F1SMR7	1	0	0	0	0	0	0	0	1	0	0	0	0	0	0	0	8.7%	0.0%	0.0%	0.0%	0.0%	0.0%	0.0%	0.0%
tr|F1RNY8|F1RNY8	1	0	0	0	0	0	0	0	1	0	0	0	0	0	0	0	5.0%	0.0%	0.0%	0.0%	0.0%	0.0%	0.0%	0.0%
Reverse_tr|K7GSB6|K7GSB6	1	0	0	0	0	0	0	0	1	0	0	0	0	0	0	0	4.4%	0.0%	0.0%	0.0%	0.0%	0.0%	0.0%	0.0%
tr|I3LSW2|I3LSW2	1	0	0	0	0	0	0	0	1	0	0	0	0	0	0	0	7.9%	0.0%	0.0%	0.0%	0.0%	0.0%	0.0%	0.0%
tr|K7GKQ5|K7GKQ5	1	0	0	0	0	0	0	0	1	0	0	0	0	0	0	0	3.5%	0.0%	0.0%	0.0%	0.0%	0.0%	0.0%	0.0%
Q29026	1	0	0	0	0	0	0	0	1	0	0	0	0	0	0	0	12.9%	0.0%	0.0%	0.0%	0.0%	0.0%	0.0%	0.0%
Q29036	1	0	0	0	0	0	0	0	1	0	0	0	0	0	0	0	10.6%	0.0%	0.0%	0.0%	0.0%	0.0%	0.0%	0.0%
tr|I3LNF6|I3LNF6	1	0	0	0	0	0	0	0	1	0	0	0	0	0	0	0	1.5%	0.0%	0.0%	0.0%	0.0%	0.0%	0.0%	0.0%
tr|F1SMA9|F1SMA9	1	0	0	0	0	0	0	0	1	0	0	0	0	0	0	0	6.3%	0.0%	0.0%	0.0%	0.0%	0.0%	0.0%	0.0%
tr|K7GRJ6|K7GRJ6	1	0	0	0	0	0	0	0	1	0	0	0	0	0	0	0	14.4%	0.0%	0.0%	0.0%	0.0%	0.0%	0.0%	0.0%
Q29081	1	0	0	0	0	0	0	0	1	0	0	0	0	0	0	0	14.6%	0.0%	0.0%	0.0%	0.0%	0.0%	0.0%	0.0%
tr|I3LPZ9|I3LPZ9	0	1	0	0	0	0	0	0	0	1	0	0	0	0	0	0	0.0%	1.5%	0.0%	0.0%	0.0%	0.0%	0.0%	0.0%
tr|I3LJR5|I3LJR5	1	0	0	0	0	0	0	0	1	0	0	0	0	0	0	0	5.0%	0.0%	0.0%	0.0%	0.0%	0.0%	0.0%	0.0%
tr|F1RVI4|F1RVI4	0	1	0	0	0	0	0	0	0	1	0	0	0	0	0	0	0.0%	2.8%	0.0%	0.0%	0.0%	0.0%	0.0%	0.0%
tr|I3LPL2|I3LPL2	1	0	0	0	0	0	0	0	1	0	0	0	0	0	0	0	4.0%	0.0%	0.0%	0.0%	0.0%	0.0%	0.0%	0.0%
tr|F1SHU8|F1SHU8	1	0	0	0	0	0	0	0	1	0	0	0	0	0	0	0	0.8%	0.0%	0.0%	0.0%	0.0%	0.0%	0.0%	0.0%
tr|F1RZA1|F1RZA1	1	0	0	0	0	0	0	0	1	0	0	0	0	0	0	0	4.3%	0.0%	0.0%	0.0%	0.0%	0.0%	0.0%	0.0%
tr|K7GQK7|K7GQK7	1	0	0	0	0	0	0	0	1	0	0	0	0	0	0	0	25.0%	0.0%	0.0%	0.0%	0.0%	0.0%	0.0%	0.0%
tr|F1RUS8|F1RUS8	1	0	0	0	0	0	0	0	1	0	0	0	0	0	0	0	6.4%	0.0%	0.0%	0.0%	0.0%	0.0%	0.0%	0.0%
tr|F1SDD2|F1SDD2	1	0	0	0	0	0	0	0	1	0	0	0	0	0	0	0	9.5%	0.0%	0.0%	0.0%	0.0%	0.0%	0.0%	0.0%
tr|F1S369|F1S369	1	0	0	0	0	0	0	0	1	0	0	0	0	0	0	0	3.7%	0.0%	0.0%	0.0%	0.0%	0.0%	0.0%	0.0%
tr|I3LUI3|I3LUI3	0	1	0	0	0	0	0	0	0	1	0	0	0	0	0	0	0.0%	2.0%	0.0%	0.0%	0.0%	0.0%	0.0%	0.0%
tr|F1SGK8|F1SGK8	1	0	0	0	0	0	0	0	1	0	0	0	0	0	0	0	3.3%	0.0%	0.0%	0.0%	0.0%	0.0%	0.0%	0.0%
tr|I3L7M1|I3L7M1	0	1	0	0	0	0	0	0	0	1	0	0	0	0	0	0	0.0%	12.8%	0.0%	0.0%	0.0%	0.0%	0.0%	0.0%
Q5XLD3	1	0	0	0	0	0	0	0	1	0	0	0	0	0	0	0	8.4%	0.0%	0.0%	0.0%	0.0%	0.0%	0.0%	0.0%
C5HGF3	1	0	0	0	0	0	0	0	1	0	0	0	0	0	0	0	5.9%	0.0%	0.0%	0.0%	0.0%	0.0%	0.0%	0.0%
tr|F1S9L5|F1S9L5	1	0	0	0	0	0	0	0	1	0	0	0	0	0	0	0	2.6%	0.0%	0.0%	0.0%	0.0%	0.0%	0.0%	0.0%
tr|F1SQG4|F1SQG4	0	1	0	0	0	0	0	0	0	1	0	0	0	0	0	0	0.0%	1.9%	0.0%	0.0%	0.0%	0.0%	0.0%	0.0%
tr|F1SK11|F1SK11	0	1	0	0	0	0	0	0	0	1	0	0	0	0	0	0	0.0%	5.1%	0.0%	0.0%	0.0%	0.0%	0.0%	0.0%
tr|F1S2A1|F1S2A1	1	0	0	0	0	0	0	0	1	0	0	0	0	0	0	0	2.6%	0.0%	0.0%	0.0%	0.0%	0.0%	0.0%	0.0%

	Peptide Count								Spectral Count								Sequence Coverage							
	Whole Cell	Whole Cell	Whole Cell	Cilia Fraction	Cilia Fraction	Cilia Fraction	Cilia Fraction	Cilia Fraction	Whole Cell	Whole Cell	Whole Cell	Cilia Fraction	Cilia Fraction	Cilia Fraction	Cilia Fraction	Cilia Fraction	Whole Cell	Whole Cell	Whole Cell	Cilia Fraction	Cilia Fraction	Cilia Fraction	Cilia Fraction	Cilia Fraction
Sample#	1	2	6	4	3	5	7	8	1	2	6	4	3	5	7	8	1	2	6	4	3	5	7	8
**Accession**																								
tr|I3LMP8|I3LMP8	1	0	0	0	0	0	0	0	1	0	0	0	0	0	0	0	6.2%	0.0%	0.0%	0.0%	0.0%	0.0%	0.0%	0.0%
Reverse_tr|F1S146|F1S146	1	0	0	0	0	0	0	0	1	0	0	0	0	0	0	0	0.4%	0.0%	0.0%	0.0%	0.0%	0.0%	0.0%	0.0%
tr|I3LDD4|I3LDD4	0	1	0	0	0	0	0	0	0	1	0	0	0	0	0	0	0.0%	3.4%	0.0%	0.0%	0.0%	0.0%	0.0%	0.0%
tr|F1SK82|F1SK82	0	1	0	0	0	0	0	0	0	1	0	0	0	0	0	0	0.0%	6.2%	0.0%	0.0%	0.0%	0.0%	0.0%	0.0%
tr|I3LK49|I3LK49	0	1	0	0	0	0	0	0	0	1	0	0	0	0	0	0	0.0%	6.4%	0.0%	0.0%	0.0%	0.0%	0.0%	0.0%
tr|I3LN46|I3LN46	1	0	0	0	0	0	0	0	1	0	0	0	0	0	0	0	21.7%	0.0%	0.0%	0.0%	0.0%	0.0%	0.0%	0.0%
tr|I3LMS6|I3LMS6	1	0	0	0	0	0	0	0	1	0	0	0	0	0	0	0	11.2%	0.0%	0.0%	0.0%	0.0%	0.0%	0.0%	0.0%
tr|F1SGE5|F1SGE5	0	1	0	0	0	0	0	0	0	1	0	0	0	0	0	0	0.0%	2.8%	0.0%	0.0%	0.0%	0.0%	0.0%	0.0%
tr|I3LVP2|I3LVP2	0	1	0	0	0	0	0	0	0	1	0	0	0	0	0	0	0.0%	5.0%	0.0%	0.0%	0.0%	0.0%	0.0%	0.0%
tr|F1SAL9|F1SAL9	1	0	0	0	0	0	0	0	1	0	0	0	0	0	0	0	4.6%	0.0%	0.0%	0.0%	0.0%	0.0%	0.0%	0.0%
tr|F1RPC2|F1RPC2	1	0	0	0	0	0	0	0	1	0	0	0	0	0	0	0	3.7%	0.0%	0.0%	0.0%	0.0%	0.0%	0.0%	0.0%
tr|I3LES5|I3LES5	0	1	0	0	0	0	0	0	0	1	0	0	0	0	0	0	0.0%	2.0%	0.0%	0.0%	0.0%	0.0%	0.0%	0.0%
tr|I3LIP1|I3LIP1	1	0	0	0	0	0	0	0	1	0	0	0	0	0	0	0	12.2%	0.0%	0.0%	0.0%	0.0%	0.0%	0.0%	0.0%
tr|I3LIN2|I3LIN2	1	0	0	0	0	0	0	0	1	0	0	0	0	0	0	0	11.8%	0.0%	0.0%	0.0%	0.0%	0.0%	0.0%	0.0%
Reverse_tr|I3LSX2|I3LSX2	0	1	0	0	0	0	0	0	0	1	0	0	0	0	0	0	0.0%	1.6%	0.0%	0.0%	0.0%	0.0%	0.0%	0.0%
tr|F1S0W2|F1S0W2	1	0	0	0	0	0	0	0	1	0	0	0	0	0	0	0	9.6%	0.0%	0.0%	0.0%	0.0%	0.0%	0.0%	0.0%
tr|I3LCE6|I3LCE6	1	0	0	0	0	0	0	0	1	0	0	0	0	0	0	0	1.4%	0.0%	0.0%	0.0%	0.0%	0.0%	0.0%	0.0%
tr|K7GP00|K7GP00	1	0	0	0	0	0	0	0	1	0	0	0	0	0	0	0	5.0%	0.0%	0.0%	0.0%	0.0%	0.0%	0.0%	0.0%
tr|F1RU31|F1RU31	1	0	0	0	0	0	0	0	1	0	0	0	0	0	0	0	2.5%	0.0%	0.0%	0.0%	0.0%	0.0%	0.0%	0.0%
tr|F1RQF4|F1RQF4	1	0	0	0	0	0	0	0	1	0	0	0	0	0	0	0	9.2%	0.0%	0.0%	0.0%	0.0%	0.0%	0.0%	0.0%
tr|F1SKT8|F1SKT8	0	1	0	0	0	0	0	0	0	1	0	0	0	0	0	0	0.0%	1.9%	0.0%	0.0%	0.0%	0.0%	0.0%	0.0%
tr|F1RPT0|F1RPT0	1	0	0	0	0	0	0	0	1	0	0	0	0	0	0	0	7.2%	0.0%	0.0%	0.0%	0.0%	0.0%	0.0%	0.0%
tr|I3LGD7|I3LGD7	1	0	0	0	0	0	0	0	1	0	0	0	0	0	0	0	7.8%	0.0%	0.0%	0.0%	0.0%	0.0%	0.0%	0.0%
tr|I3L904|I3L904	1	0	0	0	0	0	0	0	1	0	0	0	0	0	0	0	10.2%	0.0%	0.0%	0.0%	0.0%	0.0%	0.0%	0.0%
tr|K7GRZ3|K7GRZ3	1	0	0	0	0	0	0	0	1	0	0	0	0	0	0	0	13.1%	0.0%	0.0%	0.0%	0.0%	0.0%	0.0%	0.0%
tr|K7GKH1|K7GKH1	0	1	0	0	0	0	0	0	0	1	0	0	0	0	0	0	0.0%	2.4%	0.0%	0.0%	0.0%	0.0%	0.0%	0.0%
tr|F1SAB0|F1SAB0	1	0	0	0	0	0	0	0	1	0	0	0	0	0	0	0	3.4%	0.0%	0.0%	0.0%	0.0%	0.0%	0.0%	0.0%
tr|F1RX38|F1RX38	1	0	0	0	0	0	0	0	1	0	0	0	0	0	0	0	4.5%	0.0%	0.0%	0.0%	0.0%	0.0%	0.0%	0.0%
tr|I3LKX6|I3LKX6	1	0	0	0	0	0	0	0	1	0	0	0	0	0	0	0	1.6%	0.0%	0.0%	0.0%	0.0%	0.0%	0.0%	0.0%
tr|I3L9V1|I3L9V1	1	0	0	0	0	0	0	0	1	0	0	0	0	0	0	0	1.7%	0.0%	0.0%	0.0%	0.0%	0.0%	0.0%	0.0%
tr|F1RSX7|F1RSX7	1	0	0	0	0	0	0	0	1	0	0	0	0	0	0	0	4.0%	0.0%	0.0%	0.0%	0.0%	0.0%	0.0%	0.0%
tr|F1RVV7|F1RVV7	1	0	0	0	0	0	0	0	1	0	0	0	0	0	0	0	4.1%	0.0%	0.0%	0.0%	0.0%	0.0%	0.0%	0.0%
Q1A730	0	1	0	0	0	0	0	0	0	1	0	0	0	0	0	0	0.0%	5.1%	0.0%	0.0%	0.0%	0.0%	0.0%	0.0%
tr|G5EA75|G5EA75	1	0	0	0	0	0	0	0	1	0	0	0	0	0	0	0	18.8%	0.0%	0.0%	0.0%	0.0%	0.0%	0.0%	0.0%
tr|I3LUY0|I3LUY0	1	0	0	0	0	0	0	0	1	0	0	0	0	0	0	0	4.9%	0.0%	0.0%	0.0%	0.0%	0.0%	0.0%	0.0%
tr|I3L5E8|I3L5E8	0	1	0	0	0	0	0	0	0	1	0	0	0	0	0	0	0.0%	2.7%	0.0%	0.0%	0.0%	0.0%	0.0%	0.0%
Q06AU3	0	1	0	0	0	0	0	0	0	1	0	0	0	0	0	0	0.0%	8.6%	0.0%	0.0%	0.0%	0.0%	0.0%	0.0%
tr|F1SU69|F1SU69	0	1	0	0	0	0	0	0	0	1	0	0	0	0	0	0	0.0%	1.6%	0.0%	0.0%	0.0%	0.0%	0.0%	0.0%
tr|F1STU9|F1STU9	1	0	0	0	0	0	0	0	1	0	0	0	0	0	0	0	0.8%	0.0%	0.0%	0.0%	0.0%	0.0%	0.0%	0.0%
tr|F1SV36|F1SV36	0	1	0	0	0	0	0	0	0	1	0	0	0	0	0	0	0.0%	3.7%	0.0%	0.0%	0.0%	0.0%	0.0%	0.0%
tr|I3LFE4|I3LFE4	0	1	0	0	0	0	0	0	0	1	0	0	0	0	0	0	0.0%	11.8%	0.0%	0.0%	0.0%	0.0%	0.0%	0.0%
tr|F1S4V3|F1S4V3	0	1	0	0	0	0	0	0	0	1	0	0	0	0	0	0	0.0%	2.4%	0.0%	0.0%	0.0%	0.0%	0.0%	0.0%
tr|I3L727|I3L727	0	1	0	0	0	0	0	0	0	1	0	0	0	0	0	0	0.0%	1.5%	0.0%	0.0%	0.0%	0.0%	0.0%	0.0%
tr|F1SJV7|F1SJV7	1	0	0	0	0	0	0	0	1	0	0	0	0	0	0	0	4.2%	0.0%	0.0%	0.0%	0.0%	0.0%	0.0%	0.0%
tr|F1RKW6|F1RKW6	1	0	0	0	0	0	0	0	1	0	0	0	0	0	0	0	5.6%	0.0%	0.0%	0.0%	0.0%	0.0%	0.0%	0.0%
tr|F2Z596|F2Z596	1	0	0	0	0	0	0	0	1	0	0	0	0	0	0	0	20.4%	0.0%	0.0%	0.0%	0.0%	0.0%	0.0%	0.0%
tr|I3LBD6|I3LBD6	1	0	0	0	0	0	0	0	1	0	0	0	0	0	0	0	5.3%	0.0%	0.0%	0.0%	0.0%	0.0%	0.0%	0.0%
Reverse_tr|F1RFK3|F1RFK3	1	0	0	0	0	0	0	0	1	0	0	0	0	0	0	0	0.7%	0.0%	0.0%	0.0%	0.0%	0.0%	0.0%	0.0%
tr|F1S536|F1S536	1	0	0	0	0	0	0	0	1	0	0	0	0	0	0	0	2.4%	0.0%	0.0%	0.0%	0.0%	0.0%	0.0%	0.0%
tr|I3LDJ9|I3LDJ9	1	0	0	0	0	0	0	0	1	0	0	0	0	0	0	0	4.2%	0.0%	0.0%	0.0%	0.0%	0.0%	0.0%	0.0%
tr|K7GLY1|K7GLY1	1	0	0	0	0	0	0	0	1	0	0	0	0	0	0	0	6.2%	0.0%	0.0%	0.0%	0.0%	0.0%	0.0%	0.0%
tr|I3LIA5|I3LIA5	1	0	0	0	0	0	0	0	1	0	0	0	0	0	0	0	7.5%	0.0%	0.0%	0.0%	0.0%	0.0%	0.0%	0.0%
tr|I3LAV9|I3LAV9	1	0	0	0	0	0	0	0	1	0	0	0	0	0	0	0	2.1%	0.0%	0.0%	0.0%	0.0%	0.0%	0.0%	0.0%
tr|K7GRC5|K7GRC5	0	1	0	0	0	0	0	0	0	1	0	0	0	0	0	0	0.0%	1.9%	0.0%	0.0%	0.0%	0.0%	0.0%	0.0%
tr|F2Z5I0|F2Z5I0	0	1	0	0	0	0	0	0	0	1	0	0	0	0	0	0	0.0%	14.5%	0.0%	0.0%	0.0%	0.0%	0.0%	0.0%
tr|F1SA76|F1SA76	0	1	0	0	0	0	0	0	0	1	0	0	0	0	0	0	0.0%	3.7%	0.0%	0.0%	0.0%	0.0%	0.0%	0.0%
tr|F1S4S9|F1S4S9	1	0	0	0	0	0	0	0	1	0	0	0	0	0	0	0	1.7%	0.0%	0.0%	0.0%	0.0%	0.0%	0.0%	0.0%
tr|F1RT49|F1RT49	1	0	0	0	0	0	0	0	1	0	0	0	0	0	0	0	0.8%	0.0%	0.0%	0.0%	0.0%	0.0%	0.0%	0.0%
tr|F1RK61|F1RK61	1	0	0	0	0	0	0	0	1	0	0	0	0	0	0	0	4.9%	0.0%	0.0%	0.0%	0.0%	0.0%	0.0%	0.0%
tr|F1RUA4|F1RUA4	1	0	0	0	0	0	0	0	1	0	0	0	0	0	0	0	3.8%	0.0%	0.0%	0.0%	0.0%	0.0%	0.0%	0.0%
tr|I3LFY4|I3LFY4	0	1	0	0	0	0	0	0	0	1	0	0	0	0	0	0	0.0%	0.8%	0.0%	0.0%	0.0%	0.0%	0.0%	0.0%
tr|F1SNV6|F1SNV6	0	1	0	0	0	0	0	0	0	1	0	0	0	0	0	0	0.0%	6.5%	0.0%	0.0%	0.0%	0.0%	0.0%	0.0%
tr|I3LDB5|I3LDB5	1	0	0	0	0	0	0	0	1	0	0	0	0	0	0	0	12.4%	0.0%	0.0%	0.0%	0.0%	0.0%	0.0%	0.0%
tr|I3LJF4|I3LJF4	0	1	0	0	0	0	0	0	0	1	0	0	0	0	0	0	0.0%	2.7%	0.0%	0.0%	0.0%	0.0%	0.0%	0.0%
tr|I3LPE0|I3LPE0	1	0	0	0	0	0	0	0	1	0	0	0	0	0	0	0	1.3%	0.0%	0.0%	0.0%	0.0%	0.0%	0.0%	0.0%
tr|F1RVF1|F1RVF1	0	1	0	0	0	0	0	0	0	1	0	0	0	0	0	0	0.0%	3.8%	0.0%	0.0%	0.0%	0.0%	0.0%	0.0%
tr|F1SJW8|F1SJW8	1	0	0	0	0	0	0	0	1	0	0	0	0	0	0	0	5.7%	0.0%	0.0%	0.0%	0.0%	0.0%	0.0%	0.0%
tr|K7GPI6|K7GPI6	1	0	0	0	0	0	0	0	1	0	0	0	0	0	0	0	11.5%	0.0%	0.0%	0.0%	0.0%	0.0%	0.0%	0.0%
tr|F1SBJ3|F1SBJ3	1	0	0	0	0	0	0	0	1	0	0	0	0	0	0	0	6.7%	0.0%	0.0%	0.0%	0.0%	0.0%	0.0%	0.0%
tr|F1SPR6|F1SPR6	0	1	0	0	0	0	0	0	0	1	0	0	0	0	0	0	0.0%	1.1%	0.0%	0.0%	0.0%	0.0%	0.0%	0.0%
tr|I3LB82|I3LB82	1	0	0	0	0	0	0	0	1	0	0	0	0	0	0	0	5.6%	0.0%	0.0%	0.0%	0.0%	0.0%	0.0%	0.0%
tr|B8XSJ1|B8XSJ1	0	1	0	0	0	0	0	0	0	1	0	0	0	0	0	0	0.0%	5.5%	0.0%	0.0%	0.0%	0.0%	0.0%	0.0%
tr|F1SUT6|F1SUT6	1	0	0	0	0	0	0	0	1	0	0	0	0	0	0	0	1.0%	0.0%	0.0%	0.0%	0.0%	0.0%	0.0%	0.0%
tr|K7GNA8|K7GNA8	0	1	0	0	0	0	0	0	0	1	0	0	0	0	0	0	0.0%	6.3%	0.0%	0.0%	0.0%	0.0%	0.0%	0.0%
tr|I3LE00|I3LE00	1	0	0	0	0	0	0	0	1	0	0	0	0	0	0	0	7.4%	0.0%	0.0%	0.0%	0.0%	0.0%	0.0%	0.0%
P0C8B5	1	0	0	0	0	0	0	0	1	0	0	0	0	0	0	0	20.0%	0.0%	0.0%	0.0%	0.0%	0.0%	0.0%	0.0%
tr|F1RY31|F1RY31	1	0	0	0	0	0	0	0	1	0	0	0	0	0	0	0	4.3%	0.0%	0.0%	0.0%	0.0%	0.0%	0.0%	0.0%
tr|F1SJZ3|F1SJZ3	1	0	0	0	0	0	0	0	1	0	0	0	0	0	0	0	5.9%	0.0%	0.0%	0.0%	0.0%	0.0%	0.0%	0.0%
Reverse_tr|I3L7N5|I3L7N5	1	0	0	0	0	0	0	0	1	0	0	0	0	0	0	0	2.2%	0.0%	0.0%	0.0%	0.0%	0.0%	0.0%	0.0%
tr|I3LTQ6|I3LTQ6	1	0	0	0	0	0	0	0	1	0	0	0	0	0	0	0	9.0%	0.0%	0.0%	0.0%	0.0%	0.0%	0.0%	0.0%
tr|F1RI43|F1RI43	1	0	0	0	0	0	0	0	1	0	0	0	0	0	0	0	0.5%	0.0%	0.0%	0.0%	0.0%	0.0%	0.0%	0.0%

	Peptide Count								Spectral Count								Sequence Coverage							
	Whole Cell	Whole Cell	Whole Cell	Cilia Fraction	Cilia Fraction	Cilia Fraction	Cilia Fraction	Cilia Fraction	Whole Cell	Whole Cell	Whole Cell	Cilia Fraction	Cilia Fraction	Cilia Fraction	Cilia Fraction	Cilia Fraction	Whole Cell	Whole Cell	Whole Cell	Cilia Fraction	Cilia Fraction	Cilia Fraction	Cilia Fraction	Cilia Fraction
Sample#	1	2	6	4	3	5	7	8	1	2	6	4	3	5	7	8	1	2	6	4	3	5	7	8
**Accession**																								
tr|I3LN04|I3LN04	0	1	0	0	0	0	0	0	0	1	0	0	0	0	0	0	0.0%	1.4%	0.0%	0.0%	0.0%	0.0%	0.0%	0.0%
tr|F1RSS6|F1RSS6	0	1	0	0	0	0	0	0	0	1	0	0	0	0	0	0	0.0%	2.2%	0.0%	0.0%	0.0%	0.0%	0.0%	0.0%
tr|F1RT46|F1RT46	0	1	0	0	0	0	0	0	0	1	0	0	0	0	0	0	0.0%	1.9%	0.0%	0.0%	0.0%	0.0%	0.0%	0.0%
tr|F1SKT6|F1SKT6	0	1	0	0	0	0	0	0	0	1	0	0	0	0	0	0	0.0%	2.5%	0.0%	0.0%	0.0%	0.0%	0.0%	0.0%
tr|F1RU84|F1RU84	0	1	0	0	0	0	0	0	0	1	0	0	0	0	0	0	0.0%	3.5%	0.0%	0.0%	0.0%	0.0%	0.0%	0.0%
tr|F1SUV2|F1SUV2	0	1	0	0	0	0	0	0	0	1	0	0	0	0	0	0	0.0%	0.9%	0.0%	0.0%	0.0%	0.0%	0.0%	0.0%
tr|I3LML2|I3LML2	1	0	0	0	0	0	0	0	1	0	0	0	0	0	0	0	8.4%	0.0%	0.0%	0.0%	0.0%	0.0%	0.0%	0.0%
tr|I3L702|I3L702	0	1	0	0	0	0	0	0	0	1	0	0	0	0	0	0	0.0%	5.0%	0.0%	0.0%	0.0%	0.0%	0.0%	0.0%
tr|I3LVB6|I3LVB6	1	0	0	0	0	0	0	0	1	0	0	0	0	0	0	0	10.9%	0.0%	0.0%	0.0%	0.0%	0.0%	0.0%	0.0%
tr|F1RS30|F1RS30	1	0	0	0	0	0	0	0	1	0	0	0	0	0	0	0	3.6%	0.0%	0.0%	0.0%	0.0%	0.0%	0.0%	0.0%
tr|F1RRF3|F1RRF3	1	0	0	0	0	0	0	0	1	0	0	0	0	0	0	0	2.8%	0.0%	0.0%	0.0%	0.0%	0.0%	0.0%	0.0%
tr|F1RSW2|F1RSW2	0	1	0	0	0	0	0	0	0	1	0	0	0	0	0	0	0.0%	1.1%	0.0%	0.0%	0.0%	0.0%	0.0%	0.0%
tr|F1S2U6|F1S2U6	0	1	0	0	0	0	0	0	0	1	0	0	0	0	0	0	0.0%	2.1%	0.0%	0.0%	0.0%	0.0%	0.0%	0.0%
tr|F1RM20|F1RM20	0	1	0	0	0	0	0	0	0	1	0	0	0	0	0	0	0.0%	2.6%	0.0%	0.0%	0.0%	0.0%	0.0%	0.0%
tr|K7GQF0|K7GQF0	0	1	0	0	0	0	0	0	0	1	0	0	0	0	0	0	0.0%	10.0%	0.0%	0.0%	0.0%	0.0%	0.0%	0.0%
tr|F1RSV8|F1RSV8	0	1	0	0	0	0	0	0	0	1	0	0	0	0	0	0	0.0%	0.8%	0.0%	0.0%	0.0%	0.0%	0.0%	0.0%
tr|F1SLH8|F1SLH8	1	0	0	0	0	0	0	0	1	0	0	0	0	0	0	0	17.9%	0.0%	0.0%	0.0%	0.0%	0.0%	0.0%	0.0%
tr|I3LGD1|I3LGD1	1	0	0	0	0	0	0	0	1	0	0	0	0	0	0	0	3.8%	0.0%	0.0%	0.0%	0.0%	0.0%	0.0%	0.0%
tr|F1SB14|F1SB14	1	0	0	0	0	0	0	0	1	0	0	0	0	0	0	0	15.9%	0.0%	0.0%	0.0%	0.0%	0.0%	0.0%	0.0%
tr|I3LLI6|I3LLI6	0	1	0	0	0	0	0	0	0	1	0	0	0	0	0	0	0.0%	5.0%	0.0%	0.0%	0.0%	0.0%	0.0%	0.0%
tr|K7GKA0|K7GKA0	1	0	0	0	0	0	0	0	1	0	0	0	0	0	0	0	16.4%	0.0%	0.0%	0.0%	0.0%	0.0%	0.0%	0.0%
tr|F1SD83|F1SD83	1	0	0	0	0	0	0	0	1	0	0	0	0	0	0	0	3.6%	0.0%	0.0%	0.0%	0.0%	0.0%	0.0%	0.0%
tr|F1SQN7|F1SQN7	1	0	0	0	0	0	0	0	1	0	0	0	0	0	0	0	2.5%	0.0%	0.0%	0.0%	0.0%	0.0%	0.0%	0.0%
Q9N1U3	1	0	0	0	0	0	0	0	1	0	0	0	0	0	0	0	2.9%	0.0%	0.0%	0.0%	0.0%	0.0%	0.0%	0.0%
tr|K7GRB5|K7GRB5	0	1	0	0	0	0	0	0	0	1	0	0	0	0	0	0	0.0%	8.4%	0.0%	0.0%	0.0%	0.0%	0.0%	0.0%
tr|F1S6M0|F1S6M0	1	0	0	0	0	0	0	0	1	0	0	0	0	0	0	0	14.2%	0.0%	0.0%	0.0%	0.0%	0.0%	0.0%	0.0%
tr|F1SI07|F1SI07	1	0	0	0	0	0	0	0	1	0	0	0	0	0	0	0	1.8%	0.0%	0.0%	0.0%	0.0%	0.0%	0.0%	0.0%
tr|F1RXA0|F1RXA0	0	1	0	0	0	0	0	0	0	1	0	0	0	0	0	0	0.0%	1.3%	0.0%	0.0%	0.0%	0.0%	0.0%	0.0%
tr|I3LGI4|I3LGI4	1	0	0	0	0	0	0	0	1	0	0	0	0	0	0	0	5.6%	0.0%	0.0%	0.0%	0.0%	0.0%	0.0%	0.0%
tr|I3LPI5|I3LPI5	0	1	0	0	0	0	0	0	0	1	0	0	0	0	0	0	0.0%	1.5%	0.0%	0.0%	0.0%	0.0%	0.0%	0.0%
tr|I3LUE3|I3LUE3	0	1	0	0	0	0	0	0	0	1	0	0	0	0	0	0	0.0%	1.3%	0.0%	0.0%	0.0%	0.0%	0.0%	0.0%
tr|K7GKT9|K7GKT9	1	0	0	0	0	0	0	0	1	0	0	0	0	0	0	0	11.5%	0.0%	0.0%	0.0%	0.0%	0.0%	0.0%	0.0%
tr|K7GMV3|K7GMV3	1	0	0	0	0	0	0	0	1	0	0	0	0	0	0	0	5.9%	0.0%	0.0%	0.0%	0.0%	0.0%	0.0%	0.0%
tr|F1RS38|F1RS38	1	0	0	0	0	0	0	0	1	0	0	0	0	0	0	0	14.9%	0.0%	0.0%	0.0%	0.0%	0.0%	0.0%	0.0%
tr|I3LUT7|I3LUT7	1	0	0	0	0	0	0	0	1	0	0	0	0	0	0	0	6.9%	0.0%	0.0%	0.0%	0.0%	0.0%	0.0%	0.0%
tr|F2Z5R8|F2Z5R8	1	0	0	0	0	0	0	0	1	0	0	0	0	0	0	0	9.5%	0.0%	0.0%	0.0%	0.0%	0.0%	0.0%	0.0%
tr|F1SBA6|F1SBA6	1	0	0	0	0	0	0	0	1	0	0	0	0	0	0	0	4.3%	0.0%	0.0%	0.0%	0.0%	0.0%	0.0%	0.0%
tr|F1RKS6|F1RKS6	0	1	0	0	0	0	0	0	0	1	0	0	0	0	0	0	0.0%	3.1%	0.0%	0.0%	0.0%	0.0%	0.0%	0.0%
tr|K7GQ31|K7GQ31	1	0	0	0	0	0	0	0	1	0	0	0	0	0	0	0	5.5%	0.0%	0.0%	0.0%	0.0%	0.0%	0.0%	0.0%
tr|I3LCH8|I3LCH8	1	0	0	0	0	0	0	0	1	0	0	0	0	0	0	0	3.4%	0.0%	0.0%	0.0%	0.0%	0.0%	0.0%	0.0%
tr|F1RLM7|F1RLM7	1	0	0	0	0	0	0	0	1	0	0	0	0	0	0	0	3.6%	0.0%	0.0%	0.0%	0.0%	0.0%	0.0%	0.0%
tr|K7GNF9|K7GNF9	1	0	0	0	0	0	0	0	1	0	0	0	0	0	0	0	7.6%	0.0%	0.0%	0.0%	0.0%	0.0%	0.0%	0.0%
tr|F1RFQ9|F1RFQ9	1	0	0	0	0	0	0	0	1	0	0	0	0	0	0	0	1.9%	0.0%	0.0%	0.0%	0.0%	0.0%	0.0%	0.0%
Reverse_tr|F1RJZ3|F1RJZ3	1	0	0	0	0	0	0	0	1	0	0	0	0	0	0	0	5.5%	0.0%	0.0%	0.0%	0.0%	0.0%	0.0%	0.0%
tr|F1SDQ0|F1SDQ0	1	0	0	0	0	0	0	0	1	0	0	0	0	0	0	0	3.2%	0.0%	0.0%	0.0%	0.0%	0.0%	0.0%	0.0%
tr|F1S8I5|F1S8I5	0	1	0	0	0	0	0	0	0	1	0	0	0	0	0	0	0.0%	1.1%	0.0%	0.0%	0.0%	0.0%	0.0%	0.0%
tr|F1SAZ6|F1SAZ6	1	0	0	0	0	0	0	0	1	0	0	0	0	0	0	0	12.2%	0.0%	0.0%	0.0%	0.0%	0.0%	0.0%	0.0%
tr|F1SK65|F1SK65	1	0	0	0	0	0	0	0	1	0	0	0	0	0	0	0	4.4%	0.0%	0.0%	0.0%	0.0%	0.0%	0.0%	0.0%
tr|K7GPG5|K7GPG5	1	0	0	0	0	0	0	0	1	0	0	0	0	0	0	0	13.7%	0.0%	0.0%	0.0%	0.0%	0.0%	0.0%	0.0%
tr|I3L9V4|I3L9V4	1	0	0	0	0	0	0	0	1	0	0	0	0	0	0	0	17.5%	0.0%	0.0%	0.0%	0.0%	0.0%	0.0%	0.0%
tr|F1SU56|F1SU56	1	0	0	0	0	0	0	0	1	0	0	0	0	0	0	0	1.2%	0.0%	0.0%	0.0%	0.0%	0.0%	0.0%	0.0%
tr|F1RHF1|F1RHF1	1	0	0	0	0	0	0	0	1	0	0	0	0	0	0	0	4.1%	0.0%	0.0%	0.0%	0.0%	0.0%	0.0%	0.0%
tr|K7GM36|K7GM36	0	1	0	0	0	0	0	0	0	1	0	0	0	0	0	0	0.0%	6.9%	0.0%	0.0%	0.0%	0.0%	0.0%	0.0%
tr|I3LB03|I3LB03	1	0	0	0	0	0	0	0	1	0	0	0	0	0	0	0	2.9%	0.0%	0.0%	0.0%	0.0%	0.0%	0.0%	0.0%
tr|F1SGM5|F1SGM5	1	0	0	0	0	0	0	0	1	0	0	0	0	0	0	0	3.6%	0.0%	0.0%	0.0%	0.0%	0.0%	0.0%	0.0%
Reverse_tr|F1RMS4|F1RMS4	0	1	0	0	0	0	0	0	0	1	0	0	0	0	0	0	0.0%	5.6%	0.0%	0.0%	0.0%	0.0%	0.0%	0.0%
tr|I3LQ27|I3LQ27	1	0	0	0	0	0	0	0	1	0	0	0	0	0	0	0	6.1%	0.0%	0.0%	0.0%	0.0%	0.0%	0.0%	0.0%
tr|I3L5I6|I3L5I6	0	1	0	0	0	0	0	0	0	1	0	0	0	0	0	0	0.0%	1.2%	0.0%	0.0%	0.0%	0.0%	0.0%	0.0%
tr|C9E1C9|C9E1C9	1	0	0	0	0	0	0	0	1	0	0	0	0	0	0	0	4.3%	0.0%	0.0%	0.0%	0.0%	0.0%	0.0%	0.0%
tr|F1RSC9|F1RSC9	1	0	0	0	0	0	0	0	1	0	0	0	0	0	0	0	2.1%	0.0%	0.0%	0.0%	0.0%	0.0%	0.0%	0.0%
tr|F1S9L2|F1S9L2	1	0	0	0	0	0	0	0	1	0	0	0	0	0	0	0	7.7%	0.0%	0.0%	0.0%	0.0%	0.0%	0.0%	0.0%
tr|F1S1M2|F1S1M2	1	0	0	0	0	0	0	0	1	0	0	0	0	0	0	0	2.6%	0.0%	0.0%	0.0%	0.0%	0.0%	0.0%	0.0%
tr|F1SHI1|F1SHI1	0	1	0	0	0	0	0	0	0	1	0	0	0	0	0	0	0.0%	1.5%	0.0%	0.0%	0.0%	0.0%	0.0%	0.0%
tr|I3L5E9|I3L5E9	1	0	0	0	0	0	0	0	1	0	0	0	0	0	0	0	6.0%	0.0%	0.0%	0.0%	0.0%	0.0%	0.0%	0.0%
tr|I3LRR6|I3LRR6	1	0	0	0	0	0	0	0	1	0	0	0	0	0	0	0	1.9%	0.0%	0.0%	0.0%	0.0%	0.0%	0.0%	0.0%
tr|F1SRT0|F1SRT0	1	0	0	0	0	0	0	0	1	0	0	0	0	0	0	0	3.9%	0.0%	0.0%	0.0%	0.0%	0.0%	0.0%	0.0%
tr|F1SH04|F1SH04	1	0	0	0	0	0	0	0	1	0	0	0	0	0	0	0	2.5%	0.0%	0.0%	0.0%	0.0%	0.0%	0.0%	0.0%
tr|I3L913|I3L913	1	0	0	0	0	0	0	0	1	0	0	0	0	0	0	0	4.5%	0.0%	0.0%	0.0%	0.0%	0.0%	0.0%	0.0%
tr|F1SPI4|F1SPI4	1	0	0	0	0	0	0	0	1	0	0	0	0	0	0	0	1.9%	0.0%	0.0%	0.0%	0.0%	0.0%	0.0%	0.0%
tr|F1S0S6|F1S0S6	1	0	0	0	0	0	0	0	1	0	0	0	0	0	0	0	2.7%	0.0%	0.0%	0.0%	0.0%	0.0%	0.0%	0.0%
A5GFZ6	0	1	0	0	0	0	0	0	0	1	0	0	0	0	0	0	0.0%	5.7%	0.0%	0.0%	0.0%	0.0%	0.0%	0.0%
tr|I3LV81|I3LV81	1	0	0	0	0	0	0	0	1	0	0	0	0	0	0	0	7.8%	0.0%	0.0%	0.0%	0.0%	0.0%	0.0%	0.0%
Reverse_tr|F1RZ71|F1RZ71	0	1	0	0	0	0	0	0	0	1	0	0	0	0	0	0	0.0%	2.7%	0.0%	0.0%	0.0%	0.0%	0.0%	0.0%
tr|F1RMY1|F1RMY1	1	0	0	0	0	0	0	0	1	0	0	0	0	0	0	0	3.9%	0.0%	0.0%	0.0%	0.0%	0.0%	0.0%	0.0%
tr|I3LFA4|I3LFA4	1	0	0	0	0	0	0	0	1	0	0	0	0	0	0	0	12.5%	0.0%	0.0%	0.0%	0.0%	0.0%	0.0%	0.0%
tr|F1RX65|F1RX65	1	0	0	0	0	0	0	0	1	0	0	0	0	0	0	0	9.1%	0.0%	0.0%	0.0%	0.0%	0.0%	0.0%	0.0%
tr|F1SG77|F1SG77	1	0	0	0	0	0	0	0	1	0	0	0	0	0	0	0	2.3%	0.0%	0.0%	0.0%	0.0%	0.0%	0.0%	0.0%
tr|I3LR29|I3LR29	1	0	0	0	0	0	0	0	1	0	0	0	0	0	0	0	7.1%	0.0%	0.0%	0.0%	0.0%	0.0%	0.0%	0.0%
tr|F1SU33|F1SU33	1	0	0	0	0	0	0	0	1	0	0	0	0	0	0	0	3.2%	0.0%	0.0%	0.0%	0.0%	0.0%	0.0%	0.0%
tr|F1SST2|F1SST2	0	1	0	0	0	0	0	0	0	1	0	0	0	0	0	0	0.0%	3.2%	0.0%	0.0%	0.0%	0.0%	0.0%	0.0%
tr|F1S4B7|F1S4B7	1	0	0	0	0	0	0	0	1	0	0	0	0	0	0	0	3.1%	0.0%	0.0%	0.0%	0.0%	0.0%	0.0%	0.0%

	Peptide Count								Spectral Count								Sequence Coverage							
	Whole Cell	Whole Cell	Whole Cell	Cilia Fraction	Cilia Fraction	Cilia Fraction	Cilia Fraction	Cilia Fraction	Whole Cell	Whole Cell	Whole Cell	Cilia Fraction	Cilia Fraction	Cilia Fraction	Cilia Fraction	Cilia Fraction	Whole Cell	Whole Cell	Whole Cell	Cilia Fraction	Cilia Fraction	Cilia Fraction	Cilia Fraction	Cilia Fraction
Sample#	1	2	6	4	3	5	7	8	1	2	6	4	3	5	7	8	1	2	6	4	3	5	7	8
**Accession**																								
tr|F1S3B7|F1S3B7	1	0	0	0	0	0	0	0	1	0	0	0	0	0	0	0	1.3%	0.0%	0.0%	0.0%	0.0%	0.0%	0.0%	0.0%
tr|I3L7H6|I3L7H6	1	0	0	0	0	0	0	0	1	0	0	0	0	0	0	0	1.8%	0.0%	0.0%	0.0%	0.0%	0.0%	0.0%	0.0%
tr|I3LH24|I3LH24	1	0	0	0	0	0	0	0	1	0	0	0	0	0	0	0	14.6%	0.0%	0.0%	0.0%	0.0%	0.0%	0.0%	0.0%
A5GFW7	1	0	0	0	0	0	0	0	1	0	0	0	0	0	0	0	3.3%	0.0%	0.0%	0.0%	0.0%	0.0%	0.0%	0.0%
tr|I3LTK1|I3LTK1	1	0	0	0	0	0	0	0	1	0	0	0	0	0	0	0	7.9%	0.0%	0.0%	0.0%	0.0%	0.0%	0.0%	0.0%
tr|I3LAG1|I3LAG1	1	0	0	0	0	0	0	0	1	0	0	0	0	0	0	0	3.8%	0.0%	0.0%	0.0%	0.0%	0.0%	0.0%	0.0%
A5GFQ0	1	0	0	0	0	0	0	0	1	0	0	0	0	0	0	0	7.3%	0.0%	0.0%	0.0%	0.0%	0.0%	0.0%	0.0%
tr|F1S1B8|F1S1B8	1	0	0	0	0	0	0	0	1	0	0	0	0	0	0	0	5.3%	0.0%	0.0%	0.0%	0.0%	0.0%	0.0%	0.0%
tr|I3LMI1|I3LMI1	1	0	0	0	0	0	0	0	1	0	0	0	0	0	0	0	12.0%	0.0%	0.0%	0.0%	0.0%	0.0%	0.0%	0.0%
tr|F1SB76|F1SB76	0	1	0	0	0	0	0	0	0	1	0	0	0	0	0	0	0.0%	3.6%	0.0%	0.0%	0.0%	0.0%	0.0%	0.0%
tr|I3LQ17|I3LQ17	0	1	0	0	0	0	0	0	0	1	0	0	0	0	0	0	0.0%	0.5%	0.0%	0.0%	0.0%	0.0%	0.0%	0.0%
Reverse_sp|P33198|IDHP	1	0	0	0	0	0	0	0	1	0	0	0	0	0	0	0	4.8%	0.0%	0.0%	0.0%	0.0%	0.0%	0.0%	0.0%
tr|F1SHE5|F1SHE5	1	0	0	0	0	0	0	0	1	0	0	0	0	0	0	0	0.8%	0.0%	0.0%	0.0%	0.0%	0.0%	0.0%	0.0%
tr|F1RUG0|F1RUG0	1	0	0	0	0	0	0	0	1	0	0	0	0	0	0	0	4.5%	0.0%	0.0%	0.0%	0.0%	0.0%	0.0%	0.0%
tr|F1RM35|F1RM35	1	0	0	0	0	0	0	0	1	0	0	0	0	0	0	0	2.9%	0.0%	0.0%	0.0%	0.0%	0.0%	0.0%	0.0%
tr|I3LKH8|I3LKH8	1	0	0	0	0	0	0	0	1	0	0	0	0	0	0	0	19.2%	0.0%	0.0%	0.0%	0.0%	0.0%	0.0%	0.0%
tr|F1RYL1|F1RYL1	1	0	0	0	0	0	0	0	1	0	0	0	0	0	0	0	8.4%	0.0%	0.0%	0.0%	0.0%	0.0%	0.0%	0.0%
tr|F1RKZ7|F1RKZ7	1	0	0	0	0	0	0	0	1	0	0	0	0	0	0	0	10.0%	0.0%	0.0%	0.0%	0.0%	0.0%	0.0%	0.0%
tr|F2Z5T7|F2Z5T7	1	0	0	0	0	0	0	0	1	0	0	0	0	0	0	0	11.8%	0.0%	0.0%	0.0%	0.0%	0.0%	0.0%	0.0%
tr|F1RSH3|F1RSH3	1	0	0	0	0	0	0	0	1	0	0	0	0	0	0	0	6.5%	0.0%	0.0%	0.0%	0.0%	0.0%	0.0%	0.0%
tr|F1SRH9|F1SRH9	1	0	0	0	0	0	0	0	1	0	0	0	0	0	0	0	1.4%	0.0%	0.0%	0.0%	0.0%	0.0%	0.0%	0.0%
tr|F1S5D4|F1S5D4	0	1	0	0	0	0	0	0	0	1	0	0	0	0	0	0	0.0%	1.5%	0.0%	0.0%	0.0%	0.0%	0.0%	0.0%
tr|I3LFA8|I3LFA8	0	1	0	0	0	0	0	0	0	1	0	0	0	0	0	0	0.0%	7.4%	0.0%	0.0%	0.0%	0.0%	0.0%	0.0%
Q6TA49	1	0	0	0	0	0	0	0	1	0	0	0	0	0	0	0	8.7%	0.0%	0.0%	0.0%	0.0%	0.0%	0.0%	0.0%
tr|I3L5J9|I3L5J9	1	0	0	0	0	0	0	0	1	0	0	0	0	0	0	0	2.8%	0.0%	0.0%	0.0%	0.0%	0.0%	0.0%	0.0%
tr|I3LRZ8|I3LRZ8	1	0	0	0	0	0	0	0	1	0	0	0	0	0	0	0	5.2%	0.0%	0.0%	0.0%	0.0%	0.0%	0.0%	0.0%
tr|F1S9B7|F1S9B7	1	0	0	0	0	0	0	0	1	0	0	0	0	0	0	0	26.6%	0.0%	0.0%	0.0%	0.0%	0.0%	0.0%	0.0%
tr|I3L616|I3L616	1	0	0	0	0	0	0	0	1	0	0	0	0	0	0	0	2.5%	0.0%	0.0%	0.0%	0.0%	0.0%	0.0%	0.0%
tr|K7GR99|K7GR99	0	1	0	0	0	0	0	0	0	1	0	0	0	0	0	0	0.0%	0.9%	0.0%	0.0%	0.0%	0.0%	0.0%	0.0%
tr|I3L985|I3L985	1	0	0	0	0	0	0	0	1	0	0	0	0	0	0	0	10.8%	0.0%	0.0%	0.0%	0.0%	0.0%	0.0%	0.0%
tr|I3L6F6|I3L6F6	1	0	0	0	0	0	0	0	1	0	0	0	0	0	0	0	37.1%	0.0%	0.0%	0.0%	0.0%	0.0%	0.0%	0.0%
tr|I3LKQ8|I3LKQ8	1	0	0	0	0	0	0	0	1	0	0	0	0	0	0	0	1.2%	0.0%	0.0%	0.0%	0.0%	0.0%	0.0%	0.0%
tr|K7GL50|K7GL50	1	0	0	0	0	0	0	0	1	0	0	0	0	0	0	0	8.6%	0.0%	0.0%	0.0%	0.0%	0.0%	0.0%	0.0%
tr|F1RXF2|F1RXF2	0	1	0	0	0	0	0	0	0	1	0	0	0	0	0	0	0.0%	3.3%	0.0%	0.0%	0.0%	0.0%	0.0%	0.0%
tr|F1RYR3|F1RYR3	0	1	0	0	0	0	0	0	0	1	0	0	0	0	0	0	0.0%	2.3%	0.0%	0.0%	0.0%	0.0%	0.0%	0.0%
tr|F1SUQ7|F1SUQ7	1	0	0	0	0	0	0	0	1	0	0	0	0	0	0	0	2.3%	0.0%	0.0%	0.0%	0.0%	0.0%	0.0%	0.0%
tr|F1SGL6|F1SGL6	1	0	0	0	0	0	0	0	1	0	0	0	0	0	0	0	1.5%	0.0%	0.0%	0.0%	0.0%	0.0%	0.0%	0.0%
tr|I3LK36|I3LK36	1	0	0	0	0	0	0	0	1	0	0	0	0	0	0	0	7.4%	0.0%	0.0%	0.0%	0.0%	0.0%	0.0%	0.0%
tr|F1SE84|F1SE84	1	0	0	0	0	0	0	0	1	0	0	0	0	0	0	0	9.3%	0.0%	0.0%	0.0%	0.0%	0.0%	0.0%	0.0%
tr|F1RZ41|F1RZ41	1	0	0	0	0	0	0	0	1	0	0	0	0	0	0	0	4.1%	0.0%	0.0%	0.0%	0.0%	0.0%	0.0%	0.0%
Reverse_tr|F1S106|F1S106	0	1	0	0	0	0	0	0	0	1	0	0	0	0	0	0	0.0%	1.6%	0.0%	0.0%	0.0%	0.0%	0.0%	0.0%
tr|K7GSX2|K7GSX2	1	0	0	0	0	0	0	0	1	0	0	0	0	0	0	0	15.1%	0.0%	0.0%	0.0%	0.0%	0.0%	0.0%	0.0%
tr|F1SIE6|F1SIE6	1	0	0	0	0	0	0	0	1	0	0	0	0	0	0	0	3.3%	0.0%	0.0%	0.0%	0.0%	0.0%	0.0%	0.0%
tr|F1SF38|F1SF38	1	0	0	0	0	0	0	0	1	0	0	0	0	0	0	0	2.1%	0.0%	0.0%	0.0%	0.0%	0.0%	0.0%	0.0%
P16960	1	0	0	0	0	0	0	0	1	0	0	0	0	0	0	0	0.4%	0.0%	0.0%	0.0%	0.0%	0.0%	0.0%	0.0%
tr|F1SGX3|F1SGX3	0	1	0	0	0	0	0	0	0	1	0	0	0	0	0	0	0.0%	0.6%	0.0%	0.0%	0.0%	0.0%	0.0%	0.0%
tr|F1RTC4|F1RTC4	0	1	0	0	0	0	0	0	0	1	0	0	0	0	0	0	0.0%	0.8%	0.0%	0.0%	0.0%	0.0%	0.0%	0.0%
tr|K7GLM3|K7GLM3	0	1	0	0	0	0	0	0	0	1	0	0	0	0	0	0	0.0%	9.8%	0.0%	0.0%	0.0%	0.0%	0.0%	0.0%
tr|I3LR62|I3LR62	1	0	0	0	0	0	0	0	1	0	0	0	0	0	0	0	8.3%	0.0%	0.0%	0.0%	0.0%	0.0%	0.0%	0.0%
P08552	1	0	0	0	0	0	0	0	1	0	0	0	0	0	0	0	2.6%	0.0%	0.0%	0.0%	0.0%	0.0%	0.0%	0.0%
tr|I3LHX9|I3LHX9	1	0	0	0	0	0	0	0	1	0	0	0	0	0	0	0	2.0%	0.0%	0.0%	0.0%	0.0%	0.0%	0.0%	0.0%
tr|F1RMH8|F1RMH8	1	0	0	0	0	0	0	0	1	0	0	0	0	0	0	0	7.8%	0.0%	0.0%	0.0%	0.0%	0.0%	0.0%	0.0%
tr|I3LBD0|I3LBD0	1	0	0	0	0	0	0	0	1	0	0	0	0	0	0	0	5.8%	0.0%	0.0%	0.0%	0.0%	0.0%	0.0%	0.0%
tr|I3LD96|I3LD96	1	0	0	0	0	0	0	0	1	0	0	0	0	0	0	0	4.3%	0.0%	0.0%	0.0%	0.0%	0.0%	0.0%	0.0%
tr|I3LDE0|I3LDE0	0	1	0	0	0	0	0	0	0	1	0	0	0	0	0	0	0.0%	16.4%	0.0%	0.0%	0.0%	0.0%	0.0%	0.0%
tr|F1RK97|F1RK97	1	0	0	0	0	0	0	0	1	0	0	0	0	0	0	0	8.4%	0.0%	0.0%	0.0%	0.0%	0.0%	0.0%	0.0%
tr|F1RR28|F1RR28	0	1	0	0	0	0	0	0	0	1	0	0	0	0	0	0	0.0%	5.1%	0.0%	0.0%	0.0%	0.0%	0.0%	0.0%
tr|I3LKJ4|I3LKJ4	1	0	0	0	0	0	0	0	1	0	0	0	0	0	0	0	6.7%	0.0%	0.0%	0.0%	0.0%	0.0%	0.0%	0.0%
tr|K7GPE1|K7GPE1	1	0	0	0	0	0	0	0	1	0	0	0	0	0	0	0	3.0%	0.0%	0.0%	0.0%	0.0%	0.0%	0.0%	0.0%
tr|I3LFZ7|I3LFZ7	0	1	0	0	0	0	0	0	0	1	0	0	0	0	0	0	0.0%	2.0%	0.0%	0.0%	0.0%	0.0%	0.0%	0.0%
tr|F1S721|F1S721	1	0	0	0	0	0	0	0	1	0	0	0	0	0	0	0	10.2%	0.0%	0.0%	0.0%	0.0%	0.0%	0.0%	0.0%
tr|I3L9J7|I3L9J7	0	1	0	0	0	0	0	0	0	1	0	0	0	0	0	0	0.0%	6.1%	0.0%	0.0%	0.0%	0.0%	0.0%	0.0%
tr|Q9BEA4|Q9BEA4	1	0	0	0	0	0	0	0	1	0	0	0	0	0	0	0	8.1%	0.0%	0.0%	0.0%	0.0%	0.0%	0.0%	0.0%
Reverse_tr|F1S2L2|F1S2L2	1	0	0	0	0	0	0	0	1	0	0	0	0	0	0	0	5.9%	0.0%	0.0%	0.0%	0.0%	0.0%	0.0%	0.0%
tr|I3L739|I3L739	1	0	0	0	0	0	0	0	1	0	0	0	0	0	0	0	5.3%	0.0%	0.0%	0.0%	0.0%	0.0%	0.0%	0.0%
tr|F1S750|F1S750	1	0	0	0	0	0	0	0	1	0	0	0	0	0	0	0	11.1%	0.0%	0.0%	0.0%	0.0%	0.0%	0.0%	0.0%
tr|F1S640|F1S640	0	1	0	0	0	0	0	0	0	1	0	0	0	0	0	0	0.0%	0.5%	0.0%	0.0%	0.0%	0.0%	0.0%	0.0%
tr|F1S971|F1S971	1	0	0	0	0	0	0	0	1	0	0	0	0	0	0	0	1.5%	0.0%	0.0%	0.0%	0.0%	0.0%	0.0%	0.0%
tr|K7GL45|K7GL45	1	0	0	0	0	0	0	0	1	0	0	0	0	0	0	0	6.1%	0.0%	0.0%	0.0%	0.0%	0.0%	0.0%	0.0%
tr|I3LL78|I3LL78	1	0	0	0	0	0	0	0	1	0	0	0	0	0	0	0	1.4%	0.0%	0.0%	0.0%	0.0%	0.0%	0.0%	0.0%
tr|F1RM57|F1RM57	1	0	0	0	0	0	0	0	1	0	0	0	0	0	0	0	2.7%	0.0%	0.0%	0.0%	0.0%	0.0%	0.0%	0.0%
tr|I3LTJ6|I3LTJ6	1	0	0	0	0	0	0	0	1	0	0	0	0	0	0	0	0.9%	0.0%	0.0%	0.0%	0.0%	0.0%	0.0%	0.0%
tr|F1SEE0|F1SEE0	1	0	0	0	0	0	0	0	1	0	0	0	0	0	0	0	7.6%	0.0%	0.0%	0.0%	0.0%	0.0%	0.0%	0.0%
tr|I3LP66|I3LP66	0	1	0	0	0	0	0	0	0	1	0	0	0	0	0	0	0.0%	3.0%	0.0%	0.0%	0.0%	0.0%	0.0%	0.0%
tr|F1SSC5|F1SSC5	1	0	0	0	0	0	0	0	1	0	0	0	0	0	0	0	34.5%	0.0%	0.0%	0.0%	0.0%	0.0%	0.0%	0.0%
tr|F1RXR0|F1RXR0	0	1	0	0	0	0	0	0	0	1	0	0	0	0	0	0	0.0%	1.7%	0.0%	0.0%	0.0%	0.0%	0.0%	0.0%
tr|I3LHN9|I3LHN9	1	0	0	0	0	0	0	0	1	0	0	0	0	0	0	0	22.6%	0.0%	0.0%	0.0%	0.0%	0.0%	0.0%	0.0%
tr|K7GM42|K7GM42	1	0	0	0	0	0	0	0	1	0	0	0	0	0	0	0	2.7%	0.0%	0.0%	0.0%	0.0%	0.0%	0.0%	0.0%
tr|F1S6I2|F1S6I2	0	1	0	0	0	0	0	0	0	1	0	0	0	0	0	0	0.0%	1.2%	0.0%	0.0%	0.0%	0.0%	0.0%	0.0%
Reverse_tr|F1RJN0|F1RJN0	0	1	0	0	0	0	0	0	0	1	0	0	0	0	0	0	0.0%	6.4%	0.0%	0.0%	0.0%	0.0%	0.0%	0.0%
tr|F1S819|F1S819	0	1	0	0	0	0	0	0	0	1	0	0	0	0	0	0	0.0%	1.7%	0.0%	0.0%	0.0%	0.0%	0.0%	0.0%

	Peptide Count								Spectral Count								Sequence Coverage							
	Whole Cell	Whole Cell	Whole Cell	Cilia Fraction	Cilia Fraction	Cilia Fraction	Cilia Fraction	Cilia Fraction	Whole Cell	Whole Cell	Whole Cell	Cilia Fraction	Cilia Fraction	Cilia Fraction	Cilia Fraction	Cilia Fraction	Whole Cell	Whole Cell	Whole Cell	Cilia Fraction	Cilia Fraction	Cilia Fraction	Cilia Fraction	Cilia Fraction
Sample#	1	2	6	4	3	5	7	8	1	2	6	4	3	5	7	8	1	2	6	4	3	5	7	8
**Accession**																								
tr|F1RSQ0|F1RSQ0	1	0	0	0	0	0	0	0	1	0	0	0	0	0	0	0	7.8%	0.0%	0.0%	0.0%	0.0%	0.0%	0.0%	0.0%
tr|I3LBW6|I3LBW6	1	0	0	0	0	0	0	0	1	0	0	0	0	0	0	0	3.9%	0.0%	0.0%	0.0%	0.0%	0.0%	0.0%	0.0%
tr|F1RM12|F1RM12	1	0	0	0	0	0	0	0	1	0	0	0	0	0	0	0	5.1%	0.0%	0.0%	0.0%	0.0%	0.0%	0.0%	0.0%
tr|I3LP45|I3LP45	1	0	0	0	0	0	0	0	1	0	0	0	0	0	0	0	3.4%	0.0%	0.0%	0.0%	0.0%	0.0%	0.0%	0.0%
tr|I3LB84|I3LB84	1	0	0	0	0	0	0	0	1	0	0	0	0	0	0	0	2.5%	0.0%	0.0%	0.0%	0.0%	0.0%	0.0%	0.0%
tr|F1SAH1|F1SAH1	1	0	0	0	0	0	0	0	1	0	0	0	0	0	0	0	2.4%	0.0%	0.0%	0.0%	0.0%	0.0%	0.0%	0.0%
tr|F1SKW1|F1SKW1	0	1	0	0	0	0	0	0	0	1	0	0	0	0	0	0	0.0%	0.8%	0.0%	0.0%	0.0%	0.0%	0.0%	0.0%
tr|F1RIV4|F1RIV4	1	0	0	0	0	0	0	0	1	0	0	0	0	0	0	0	0.6%	0.0%	0.0%	0.0%	0.0%	0.0%	0.0%	0.0%
tr|K7GMB2|K7GMB2	1	0	0	0	0	0	0	0	1	0	0	0	0	0	0	0	3.9%	0.0%	0.0%	0.0%	0.0%	0.0%	0.0%	0.0%
tr|I3LFU3|I3LFU3	1	0	0	0	0	0	0	0	1	0	0	0	0	0	0	0	3.1%	0.0%	0.0%	0.0%	0.0%	0.0%	0.0%	0.0%
tr|K7GP27|K7GP27	0	1	0	0	0	0	0	0	0	1	0	0	0	0	0	0	0.0%	14.4%	0.0%	0.0%	0.0%	0.0%	0.0%	0.0%
tr|I3L6W1|I3L6W1	1	0	0	0	0	0	0	0	1	0	0	0	0	0	0	0	2.8%	0.0%	0.0%	0.0%	0.0%	0.0%	0.0%	0.0%
tr|F1SE03|F1SE03	0	1	0	0	0	0	0	0	0	1	0	0	0	0	0	0	0.0%	8.0%	0.0%	0.0%	0.0%	0.0%	0.0%	0.0%
tr|I3LU43|I3LU43	0	1	0	0	0	0	0	0	0	1	0	0	0	0	0	0	0.0%	15.3%	0.0%	0.0%	0.0%	0.0%	0.0%	0.0%
tr|F1S3H4|F1S3H4	1	0	0	0	0	0	0	0	1	0	0	0	0	0	0	0	4.8%	0.0%	0.0%	0.0%	0.0%	0.0%	0.0%	0.0%
tr|F1SJJ1|F1SJJ1	0	1	0	0	0	0	0	0	0	1	0	0	0	0	0	0	0.0%	5.1%	0.0%	0.0%	0.0%	0.0%	0.0%	0.0%
tr|F1SAF2|F1SAF2	1	0	0	0	0	0	0	0	1	0	0	0	0	0	0	0	6.4%	0.0%	0.0%	0.0%	0.0%	0.0%	0.0%	0.0%
Reverse_tr|I3LH63|I3LH63	1	0	0	0	0	0	0	0	1	0	0	0	0	0	0	0	1.1%	0.0%	0.0%	0.0%	0.0%	0.0%	0.0%	0.0%
Reverse_tr|F1SJC7|F1SJC7	0	1	0	0	0	0	0	0	0	1	0	0	0	0	0	0	0.0%	0.9%	0.0%	0.0%	0.0%	0.0%	0.0%	0.0%
tr|F1S7Y1|F1S7Y1	1	0	0	0	0	0	0	0	1	0	0	0	0	0	0	0	2.2%	0.0%	0.0%	0.0%	0.0%	0.0%	0.0%	0.0%
tr|F1RW49|F1RW49	1	0	0	0	0	0	0	0	1	0	0	0	0	0	0	0	1.6%	0.0%	0.0%	0.0%	0.0%	0.0%	0.0%	0.0%
tr|F1STI7|F1STI7	0	1	0	0	0	0	0	0	0	1	0	0	0	0	0	0	0.0%	3.2%	0.0%	0.0%	0.0%	0.0%	0.0%	0.0%
tr|I3LBI4|I3LBI4	1	0	0	0	0	0	0	0	1	0	0	0	0	0	0	0	4.9%	0.0%	0.0%	0.0%	0.0%	0.0%	0.0%	0.0%
tr|F1RGW6|F1RGW6	1	0	0	0	0	0	0	0	1	0	0	0	0	0	0	0	1.5%	0.0%	0.0%	0.0%	0.0%	0.0%	0.0%	0.0%
tr|I3LD55|I3LD55	1	0	0	0	0	0	0	0	1	0	0	0	0	0	0	0	2.4%	0.0%	0.0%	0.0%	0.0%	0.0%	0.0%	0.0%
tr|F1SS60|F1SS60	0	1	0	0	0	0	0	0	0	1	0	0	0	0	0	0	0.0%	5.6%	0.0%	0.0%	0.0%	0.0%	0.0%	0.0%
tr|F1RG00|F1RG00	0	1	0	0	0	0	0	0	0	1	0	0	0	0	0	0	0.0%	6.0%	0.0%	0.0%	0.0%	0.0%	0.0%	0.0%
tr|I3LN07|I3LN07	1	0	0	0	0	0	0	0	1	0	0	0	0	0	0	0	1.1%	0.0%	0.0%	0.0%	0.0%	0.0%	0.0%	0.0%
tr|F1SHF7|F1SHF7	0	1	0	0	0	0	0	0	0	1	0	0	0	0	0	0	0.0%	0.6%	0.0%	0.0%	0.0%	0.0%	0.0%	0.0%
tr|F1RFN6|F1RFN6	1	0	0	0	0	0	0	0	1	0	0	0	0	0	0	0	1.6%	0.0%	0.0%	0.0%	0.0%	0.0%	0.0%	0.0%
tr|I3LJA9|I3LJA9	1	0	0	0	0	0	0	0	1	0	0	0	0	0	0	0	20.5%	0.0%	0.0%	0.0%	0.0%	0.0%	0.0%	0.0%
tr|I3LKA2|I3LKA2	1	0	0	0	0	0	0	0	1	0	0	0	0	0	0	0	5.6%	0.0%	0.0%	0.0%	0.0%	0.0%	0.0%	0.0%
Q08353	0	1	0	0	0	0	0	0	0	1	0	0	0	0	0	0	0.0%	6.4%	0.0%	0.0%	0.0%	0.0%	0.0%	0.0%
tr|F1S3Z2|F1S3Z2	1	0	0	0	0	0	0	0	1	0	0	0	0	0	0	0	4.4%	0.0%	0.0%	0.0%	0.0%	0.0%	0.0%	0.0%
tr|F1S2T4|F1S2T4	0	1	0	0	0	0	0	0	0	1	0	0	0	0	0	0	0.0%	1.3%	0.0%	0.0%	0.0%	0.0%	0.0%	0.0%
tr|I3LU03|I3LU03	1	0	0	0	0	0	0	0	1	0	0	0	0	0	0	0	7.9%	0.0%	0.0%	0.0%	0.0%	0.0%	0.0%	0.0%
tr|I3LJC8|I3LJC8	1	0	0	0	0	0	0	0	1	0	0	0	0	0	0	0	5.6%	0.0%	0.0%	0.0%	0.0%	0.0%	0.0%	0.0%
tr|F1S1W5|F1S1W5	1	0	0	0	0	0	0	0	1	0	0	0	0	0	0	0	8.9%	0.0%	0.0%	0.0%	0.0%	0.0%	0.0%	0.0%
tr|I3LJI4|I3LJI4	1	0	0	0	0	0	0	0	1	0	0	0	0	0	0	0	2.1%	0.0%	0.0%	0.0%	0.0%	0.0%	0.0%	0.0%
tr|F1RQX0|F1RQX0	0	1	0	0	0	0	0	0	0	1	0	0	0	0	0	0	0.0%	0.9%	0.0%	0.0%	0.0%	0.0%	0.0%	0.0%
tr|I3LIU7|I3LIU7	1	0	0	0	0	0	0	0	1	0	0	0	0	0	0	0	4.8%	0.0%	0.0%	0.0%	0.0%	0.0%	0.0%	0.0%
O13010	0	1	0	0	0	0	0	0	0	1	0	0	0	0	0	0	0.0%	2.5%	0.0%	0.0%	0.0%	0.0%	0.0%	0.0%
Reverse_tr|F1S8Y0|F1S8Y0	1	0	0	0	0	0	0	0	1	0	0	0	0	0	0	0	5.4%	0.0%	0.0%	0.0%	0.0%	0.0%	0.0%	0.0%
tr|F1S786|F1S786	1	0	0	0	0	0	0	0	1	0	0	0	0	0	0	0	3.6%	0.0%	0.0%	0.0%	0.0%	0.0%	0.0%	0.0%
tr|I3L7R7|I3L7R7	1	0	0	0	0	0	0	0	1	0	0	0	0	0	0	0	3.7%	0.0%	0.0%	0.0%	0.0%	0.0%	0.0%	0.0%
tr|I3L837|I3L837	1	0	0	0	0	0	0	0	1	0	0	0	0	0	0	0	2.8%	0.0%	0.0%	0.0%	0.0%	0.0%	0.0%	0.0%
tr|I3LLV0|I3LLV0	0	1	0	0	0	0	0	0	0	1	0	0	0	0	0	0	0.0%	6.0%	0.0%	0.0%	0.0%	0.0%	0.0%	0.0%
tr|I3LA22|I3LA22	1	0	0	0	0	0	0	0	1	0	0	0	0	0	0	0	6.5%	0.0%	0.0%	0.0%	0.0%	0.0%	0.0%	0.0%
tr|F1RSZ7|F1RSZ7	1	0	0	0	0	0	0	0	1	0	0	0	0	0	0	0	5.2%	0.0%	0.0%	0.0%	0.0%	0.0%	0.0%	0.0%
tr|K7GNT3|K7GNT3	1	0	0	0	0	0	0	0	1	0	0	0	0	0	0	0	3.4%	0.0%	0.0%	0.0%	0.0%	0.0%	0.0%	0.0%
tr|F1SGV5|F1SGV5	1	0	0	0	0	0	0	0	1	0	0	0	0	0	0	0	1.7%	0.0%	0.0%	0.0%	0.0%	0.0%	0.0%	0.0%
tr|F1S9A8|F1S9A8	1	0	0	0	0	0	0	0	1	0	0	0	0	0	0	0	0.4%	0.0%	0.0%	0.0%	0.0%	0.0%	0.0%	0.0%
tr|I3L7Q8|I3L7Q8	0	1	0	0	0	0	0	0	0	1	0	0	0	0	0	0	0.0%	1.9%	0.0%	0.0%	0.0%	0.0%	0.0%	0.0%
tr|K7GNB1|K7GNB1	1	0	0	0	0	0	0	0	1	0	0	0	0	0	0	0	2.4%	0.0%	0.0%	0.0%	0.0%	0.0%	0.0%	0.0%
tr|K7GNR1|K7GNR1	1	0	0	0	0	0	0	0	1	0	0	0	0	0	0	0	2.4%	0.0%	0.0%	0.0%	0.0%	0.0%	0.0%	0.0%
Q864V5	1	0	0	0	0	0	0	0	1	0	0	0	0	0	0	0	6.9%	0.0%	0.0%	0.0%	0.0%	0.0%	0.0%	0.0%
tr|F1SAW9|F1SAW9	1	0	0	0	0	0	0	0	1	0	0	0	0	0	0	0	10.4%	0.0%	0.0%	0.0%	0.0%	0.0%	0.0%	0.0%
tr|I3LUZ2|I3LUZ2	1	0	0	0	0	0	0	0	1	0	0	0	0	0	0	0	10.2%	0.0%	0.0%	0.0%	0.0%	0.0%	0.0%	0.0%
tr|I3LRL0|I3LRL0	0	1	0	0	0	0	0	0	0	1	0	0	0	0	0	0	0.0%	5.9%	0.0%	0.0%	0.0%	0.0%	0.0%	0.0%
tr|I3LF91|I3LF91	1	0	0	0	0	0	0	0	1	0	0	0	0	0	0	0	5.9%	0.0%	0.0%	0.0%	0.0%	0.0%	0.0%	0.0%
tr|I3LBS4|I3LBS4	1	0	0	0	0	0	0	0	1	0	0	0	0	0	0	0	21.4%	0.0%	0.0%	0.0%	0.0%	0.0%	0.0%	0.0%
tr|F2Z4Z6|F2Z4Z6	0	1	0	0	0	0	0	0	0	1	0	0	0	0	0	0	0.0%	2.1%	0.0%	0.0%	0.0%	0.0%	0.0%	0.0%
tr|F1SRV6|F1SRV6	1	0	0	0	0	0	0	0	1	0	0	0	0	0	0	0	6.6%	0.0%	0.0%	0.0%	0.0%	0.0%	0.0%	0.0%
Reverse_tr|F1SG07|F1SG07	1	0	0	0	0	0	0	0	1	0	0	0	0	0	0	0	1.5%	0.0%	0.0%	0.0%	0.0%	0.0%	0.0%	0.0%
tr|F1RSK9|F1RSK9	1	0	0	0	0	0	0	0	1	0	0	0	0	0	0	0	2.2%	0.0%	0.0%	0.0%	0.0%	0.0%	0.0%	0.0%
tr|I3LJ09|I3LJ09	1	0	0	0	0	0	0	0	1	0	0	0	0	0	0	0	13.2%	0.0%	0.0%	0.0%	0.0%	0.0%	0.0%	0.0%
tr|I3LIU4|I3LIU4	0	1	0	0	0	0	0	0	0	1	0	0	0	0	0	0	0.0%	13.9%	0.0%	0.0%	0.0%	0.0%	0.0%	0.0%
tr|F1SQP9|F1SQP9	0	1	0	0	0	0	0	0	0	1	0	0	0	0	0	0	0.0%	1.9%	0.0%	0.0%	0.0%	0.0%	0.0%	0.0%
tr|I3LPR5|I3LPR5	1	0	0	0	0	0	0	0	1	0	0	0	0	0	0	0	4.5%	0.0%	0.0%	0.0%	0.0%	0.0%	0.0%	0.0%
tr|K7GQD8|K7GQD8	0	1	0	0	0	0	0	0	0	1	0	0	0	0	0	0	0.0%	1.6%	0.0%	0.0%	0.0%	0.0%	0.0%	0.0%
tr|K7GSH9|K7GSH9	1	0	0	0	0	0	0	0	1	0	0	0	0	0	0	0	12.8%	0.0%	0.0%	0.0%	0.0%	0.0%	0.0%	0.0%
Reverse_tr|F1RRU6|F1RRU6	0	1	0	0	0	0	0	0	0	1	0	0	0	0	0	0	0.0%	2.3%	0.0%	0.0%	0.0%	0.0%	0.0%	0.0%
Reverse_tr|I3LGB6|I3LGB6	0	1	0	0	0	0	0	0	0	1	0	0	0	0	0	0	0.0%	1.5%	0.0%	0.0%	0.0%	0.0%	0.0%	0.0%
tr|F1RVE4|F1RVE4	1	0	0	0	0	0	0	0	1	0	0	0	0	0	0	0	6.9%	0.0%	0.0%	0.0%	0.0%	0.0%	0.0%	0.0%
tr|I3LBL7|I3LBL7	1	0	0	0	0	0	0	0	1	0	0	0	0	0	0	0	4.0%	0.0%	0.0%	0.0%	0.0%	0.0%	0.0%	0.0%
tr|F1SLU9|F1SLU9	1	0	0	0	0	0	0	0	1	0	0	0	0	0	0	0	2.7%	0.0%	0.0%	0.0%	0.0%	0.0%	0.0%	0.0%
tr|I3LBJ8|I3LBJ8	1	0	0	0	0	0	0	0	1	0	0	0	0	0	0	0	8.4%	0.0%	0.0%	0.0%	0.0%	0.0%	0.0%	0.0%
tr|F1S0E1|F1S0E1	1	0	0	0	0	0	0	0	1	0	0	0	0	0	0	0	3.2%	0.0%	0.0%	0.0%	0.0%	0.0%	0.0%	0.0%
tr|F1RKV2|F1RKV2	1	0	0	0	0	0	0	0	1	0	0	0	0	0	0	0	0.6%	0.0%	0.0%	0.0%	0.0%	0.0%	0.0%	0.0%
tr|K7GQD5|K7GQD5	1	0	0	0	0	0	0	0	1	0	0	0	0	0	0	0	10.6%	0.0%	0.0%	0.0%	0.0%	0.0%	0.0%	0.0%
tr|F1RHU3|F1RHU3	0	1	0	0	0	0	0	0	0	1	0	0	0	0	0	0	0.0%	2.0%	0.0%	0.0%	0.0%	0.0%	0.0%	0.0%

	Peptide Count								Spectral Count								Sequence Coverage							
	Whole Cell	Whole Cell	Whole Cell	Cilia Fraction	Cilia Fraction	Cilia Fraction	Cilia Fraction	Cilia Fraction	Whole Cell	Whole Cell	Whole Cell	Cilia Fraction	Cilia Fraction	Cilia Fraction	Cilia Fraction	Cilia Fraction	Whole Cell	Whole Cell	Whole Cell	Cilia Fraction	Cilia Fraction	Cilia Fraction	Cilia Fraction	Cilia Fraction
Sample#	1	2	6	4	3	5	7	8	1	2	6	4	3	5	7	8	1	2	6	4	3	5	7	8
**Accession**																								
tr|I3L626|I3L626	1	0	0	0	0	0	0	0	1	0	0	0	0	0	0	0	5.2%	0.0%	0.0%	0.0%	0.0%	0.0%	0.0%	0.0%
tr|I3LN95|I3LN95	0	1	0	0	0	0	0	0	0	1	0	0	0	0	0	0	0.0%	1.9%	0.0%	0.0%	0.0%	0.0%	0.0%	0.0%
tr|F1SS98|F1SS98	1	0	0	0	0	0	0	0	1	0	0	0	0	0	0	0	8.1%	0.0%	0.0%	0.0%	0.0%	0.0%	0.0%	0.0%
tr|F1RQE2|F1RQE2	1	0	0	0	0	0	0	0	1	0	0	0	0	0	0	0	3.1%	0.0%	0.0%	0.0%	0.0%	0.0%	0.0%	0.0%
tr|K7GQ49|K7GQ49	0	1	0	0	0	0	0	0	0	1	0	0	0	0	0	0	0.0%	10.6%	0.0%	0.0%	0.0%	0.0%	0.0%	0.0%
tr|K7GN81|K7GN81	0	1	0	0	0	0	0	0	0	1	0	0	0	0	0	0	0.0%	4.6%	0.0%	0.0%	0.0%	0.0%	0.0%	0.0%
tr|I3LNY6|I3LNY6	1	0	0	0	0	0	0	0	1	0	0	0	0	0	0	0	0.9%	0.0%	0.0%	0.0%	0.0%	0.0%	0.0%	0.0%
tr|I3LM88|I3LM88	1	0	0	0	0	0	0	0	1	0	0	0	0	0	0	0	2.0%	0.0%	0.0%	0.0%	0.0%	0.0%	0.0%	0.0%
tr|F1SLT9|F1SLT9	0	1	0	0	0	0	0	0	0	1	0	0	0	0	0	0	0.0%	13.2%	0.0%	0.0%	0.0%	0.0%	0.0%	0.0%
tr|I3LCU8|I3LCU8	1	0	0	0	0	0	0	0	1	0	0	0	0	0	0	0	12.0%	0.0%	0.0%	0.0%	0.0%	0.0%	0.0%	0.0%
tr|F1RG19|F1RG19	1	0	0	0	0	0	0	0	1	0	0	0	0	0	0	0	6.4%	0.0%	0.0%	0.0%	0.0%	0.0%	0.0%	0.0%
tr|F1S074|F1S074	0	1	0	0	0	0	0	0	0	1	0	0	0	0	0	0	0.0%	1.2%	0.0%	0.0%	0.0%	0.0%	0.0%	0.0%
tr|I3LPK4|I3LPK4	1	0	0	0	0	0	0	0	1	0	0	0	0	0	0	0	4.1%	0.0%	0.0%	0.0%	0.0%	0.0%	0.0%	0.0%
tr|I3L7M6|I3L7M6	1	0	0	0	0	0	0	0	1	0	0	0	0	0	0	0	2.6%	0.0%	0.0%	0.0%	0.0%	0.0%	0.0%	0.0%
tr|I3LN69|I3LN69	1	0	0	0	0	0	0	0	1	0	0	0	0	0	0	0	9.1%	0.0%	0.0%	0.0%	0.0%	0.0%	0.0%	0.0%
tr|F1RVC1|F1RVC1	0	1	0	0	0	0	0	0	0	1	0	0	0	0	0	0	0.0%	2.9%	0.0%	0.0%	0.0%	0.0%	0.0%	0.0%
tr|I3LUZ6|I3LUZ6	1	0	0	0	0	0	0	0	1	0	0	0	0	0	0	0	3.8%	0.0%	0.0%	0.0%	0.0%	0.0%	0.0%	0.0%
tr|F1SUB2|F1SUB2	1	0	0	0	0	0	0	0	1	0	0	0	0	0	0	0	3.5%	0.0%	0.0%	0.0%	0.0%	0.0%	0.0%	0.0%
tr|F1SDJ6|F1SDJ6	1	0	1	0	0	0	0	0	1	0	1	0	0	0	0	0	1.3%	0.0%	1.0%	0.0%	0.0%	0.0%	0.0%	0.0%
tr|F1RXA9|F1RXA9	1	0	1	0	0	0	0	0	1	0	1	0	0	0	0	0	1.6%	0.0%	1.5%	0.0%	0.0%	0.0%	0.0%	0.0%
tr|I3LQC2|I3LQC2	1	0	1	0	0	0	0	0	1	0	1	0	0	0	0	0	23.3%	0.0%	23.3%	0.0%	0.0%	0.0%	0.0%	0.0%
tr|F1SGD0|F1SGD0	0	1	1	0	0	0	0	0	0	1	1	0	0	0	0	0	0.0%	5.4%	2.9%	0.0%	0.0%	0.0%	0.0%	0.0%
tr|F1S7T5|F1S7T5	1	0	1	0	0	0	0	0	1	0	1	0	0	0	0	0	3.9%	0.0%	4.5%	0.0%	0.0%	0.0%	0.0%	0.0%
tr|F1SA47|F1SA47	1	0	1	0	0	0	0	0	1	0	1	0	0	0	0	0	1.5%	0.0%	1.5%	0.0%	0.0%	0.0%	0.0%	0.0%
tr|F1RFY3|F1RFY3	1	0	1	0	0	0	0	0	1	0	1	0	0	0	0	0	12.8%	0.0%	5.7%	0.0%	0.0%	0.0%	0.0%	0.0%
tr|K7GKB9|K7GKB9	1	0	1	0	0	0	0	0	1	0	1	0	0	0	0	0	9.3%	0.0%	6.5%	0.0%	0.0%	0.0%	0.0%	0.0%
tr|I3LB48|I3LB48	1	0	1	0	0	0	0	0	1	0	1	0	0	0	0	0	12.9%	0.0%	12.9%	0.0%	0.0%	0.0%	0.0%	0.0%
tr|Q2EN79|Q2EN79	1	0	1	0	0	0	0	0	1	0	1	0	0	0	0	0	26.6%	0.0%	26.6%	0.0%	0.0%	0.0%	0.0%	0.0%
tr|I3LUL3|I3LUL3	1	0	1	0	0	0	0	0	1	0	1	0	0	0	0	0	14.8%	0.0%	46.3%	0.0%	0.0%	0.0%	0.0%	0.0%
tr|F1RHC6|F1RHC6	1	0	1	0	0	0	0	0	1	0	1	0	0	0	0	0	10.4%	0.0%	12.8%	0.0%	0.0%	0.0%	0.0%	0.0%
tr|F1STE6|F1STE6	1	0	1	0	0	0	0	0	1	0	1	0	0	0	0	0	10.2%	0.0%	9.7%	0.0%	0.0%	0.0%	0.0%	0.0%
tr|F1SI71|F1SI71	1	0	1	0	0	0	0	0	1	0	1	0	0	0	0	0	4.9%	0.0%	4.9%	0.0%	0.0%	0.0%	0.0%	0.0%
tr|F1S4J0|F1S4J0	1	0	1	0	0	0	0	0	1	0	1	0	0	0	0	0	3.6%	0.0%	3.6%	0.0%	0.0%	0.0%	0.0%	0.0%
tr|K7GLQ5|K7GLQ5	1	0	1	0	0	0	0	0	1	0	1	0	0	0	0	0	4.3%	0.0%	6.7%	0.0%	0.0%	0.0%	0.0%	0.0%
tr|K7GL12|K7GL12	0	1	1	0	0	0	0	0	0	1	1	0	0	0	0	0	0.0%	2.4%	1.9%	0.0%	0.0%	0.0%	0.0%	0.0%
tr|F1SD01|F1SD01	1	0	1	0	0	0	0	0	1	0	1	0	0	0	0	0	4.9%	0.0%	2.4%	0.0%	0.0%	0.0%	0.0%	0.0%
tr|F1RKC7|F1RKC7	0	1	1	0	0	0	0	0	0	1	1	0	0	0	0	0	0.0%	2.1%	3.3%	0.0%	0.0%	0.0%	0.0%	0.0%
tr|F1RRG7|F1RRG7	0	1	1	0	0	0	0	0	0	1	1	0	0	0	0	0	0.0%	2.2%	1.7%	0.0%	0.0%	0.0%	0.0%	0.0%
tr|I3LKW6|I3LKW6	1	0	1	0	0	0	0	0	1	0	1	0	0	0	0	0	7.9%	0.0%	13.3%	0.0%	0.0%	0.0%	0.0%	0.0%
tr|F1RN66|F1RN66	1	0	1	0	0	0	0	0	1	0	1	0	0	0	0	0	0.6%	0.0%	1.5%	0.0%	0.0%	0.0%	0.0%	0.0%
tr|F1SEZ6|F1SEZ6	1	0	1	0	0	0	0	0	1	0	1	0	0	0	0	0	3.6%	0.0%	3.2%	0.0%	0.0%	0.0%	0.0%	0.0%
tr|F1SA28|F1SA28	1	0	1	0	0	0	0	0	1	0	1	0	0	0	0	0	3.2%	0.0%	2.1%	0.0%	0.0%	0.0%	0.0%	0.0%
tr|F1RFL4|F1RFL4	1	0	1	0	0	0	0	0	1	0	1	0	0	0	0	0	5.7%	0.0%	3.0%	0.0%	0.0%	0.0%	0.0%	0.0%
tr|I3LQ99|I3LQ99	1	0	1	0	0	0	0	0	1	0	1	0	0	0	0	0	11.8%	0.0%	11.8%	0.0%	0.0%	0.0%	0.0%	0.0%
Reverse_tr|F1SNR3|F1SNR3	0	1	1	0	0	0	0	0	0	1	1	0	0	0	0	0	0.0%	0.7%	1.1%	0.0%	0.0%	0.0%	0.0%	0.0%
tr|F1S8J8|F1S8J8	1	0	1	0	0	0	0	0	1	0	1	0	0	0	0	0	2.7%	0.0%	3.3%	0.0%	0.0%	0.0%	0.0%	0.0%
tr|I3LA04|I3LA04	1	0	1	0	0	0	0	0	1	0	1	0	0	0	0	0	11.9%	0.0%	11.3%	0.0%	0.0%	0.0%	0.0%	0.0%
tr|F1S4R1|F1S4R1	1	0	1	0	0	0	0	0	1	0	1	0	0	0	0	0	5.4%	0.0%	2.4%	0.0%	0.0%	0.0%	0.0%	0.0%
tr|F1SID1|F1SID1	1	0	1	0	0	0	0	0	1	0	1	0	0	0	0	0	2.3%	0.0%	1.1%	0.0%	0.0%	0.0%	0.0%	0.0%
tr|A5GFR8|A5GFR8	1	0	1	0	0	0	0	0	1	0	1	0	0	0	0	0	4.8%	0.0%	5.5%	0.0%	0.0%	0.0%	0.0%	0.0%
tr|F1S713|F1S713	1	0	1	0	0	0	0	0	1	0	1	0	0	0	0	0	15.2%	0.0%	10.5%	0.0%	0.0%	0.0%	0.0%	0.0%
tr|I3L6N4|I3L6N4	1	0	1	0	0	0	0	0	1	0	1	0	0	0	0	0	0.6%	0.0%	1.5%	0.0%	0.0%	0.0%	0.0%	0.0%
tr|K7GMT1|K7GMT1	0	1	1	0	0	0	0	0	0	1	1	0	0	0	0	0	0.0%	2.5%	2.0%	0.0%	0.0%	0.0%	0.0%	0.0%
tr|E9LK28|E9LK28	1	0	1	0	0	0	0	0	1	0	1	0	0	0	0	0	3.4%	0.0%	5.4%	0.0%	0.0%	0.0%	0.0%	0.0%
tr|I3LCW7|I3LCW7	1	0	1	0	0	0	0	0	1	0	1	0	0	0	0	0	10.7%	0.0%	20.0%	0.0%	0.0%	0.0%	0.0%	0.0%
tr|I3LDB2|I3LDB2	1	0	1	0	0	0	0	0	1	0	1	0	0	0	0	0	3.5%	0.0%	7.6%	0.0%	0.0%	0.0%	0.0%	0.0%
tr|I3LIS2|I3LIS2	1	0	1	0	0	0	0	0	1	0	1	0	0	0	0	0	0.6%	0.0%	1.5%	0.0%	0.0%	0.0%	0.0%	0.0%
tr|F1RXB3|F1RXB3	1	0	1	0	0	0	0	0	1	0	1	0	0	0	0	0	1.6%	0.0%	1.5%	0.0%	0.0%	0.0%	0.0%	0.0%
tr|F1S549|F1S549	1	0	1	0	0	0	0	0	1	0	1	0	0	0	0	0	6.7%	0.0%	6.5%	0.0%	0.0%	0.0%	0.0%	0.0%
tr|J9JIK5|J9JIK5	1	0	1	0	0	0	0	0	1	0	1	0	0	0	0	0	18.1%	0.0%	6.2%	0.0%	0.0%	0.0%	0.0%	0.0%
tr|F1SPI0|F1SPI0	1	0	1	0	0	0	0	0	1	0	1	0	0	0	0	0	16.7%	0.0%	13.0%	0.0%	0.0%	0.0%	0.0%	0.0%
tr|A5GFQ7|A5GFQ7	0	1	1	0	0	0	0	0	0	1	1	0	0	0	0	0	0.0%	1.5%	2.7%	0.0%	0.0%	0.0%	0.0%	0.0%
tr|I3LDE4|I3LDE4	1	0	1	0	0	0	0	0	1	0	1	0	0	0	0	0	6.6%	0.0%	6.3%	0.0%	0.0%	0.0%	0.0%	0.0%
tr|F1RG29|F1RG29	1	0	1	0	0	0	0	0	1	0	1	0	0	0	0	0	6.5%	0.0%	6.2%	0.0%	0.0%	0.0%	0.0%	0.0%
tr|I3L670|I3L670	1	0	1	0	0	0	0	0	1	0	1	0	0	0	0	0	11.7%	0.0%	17.1%	0.0%	0.0%	0.0%	0.0%	0.0%
tr|F1RFY6|F1RFY6	1	0	1	0	0	0	0	0	1	0	1	0	0	0	0	0	1.3%	0.0%	2.0%	0.0%	0.0%	0.0%	0.0%	0.0%
tr|F1S3C2|F1S3C2	0	1	1	0	0	0	0	0	0	1	1	0	0	0	0	0	0.0%	8.4%	7.4%	0.0%	0.0%	0.0%	0.0%	0.0%
tr|F1RNH3|F1RNH3	1	0	1	0	0	0	0	0	1	0	1	0	0	0	0	0	3.3%	0.0%	2.6%	0.0%	0.0%	0.0%	0.0%	0.0%
tr|F1SNZ1|F1SNZ1	0	1	1	0	0	0	0	0	0	1	1	0	0	0	0	0	0.0%	6.0%	6.7%	0.0%	0.0%	0.0%	0.0%	0.0%
tr|I3LRG9|I3LRG9	1	0	1	0	0	0	0	0	1	0	1	0	0	0	0	0	6.2%	0.0%	10.6%	0.0%	0.0%	0.0%	0.0%	0.0%
P24540	1	0	1	0	0	0	0	0	1	0	1	0	0	0	0	0	25.7%	0.0%	8.9%	0.0%	0.0%	0.0%	0.0%	0.0%
tr|F1STJ3|F1STJ3	1	0	1	0	0	0	0	0	1	0	1	0	0	0	0	0	10.4%	0.0%	9.5%	0.0%	0.0%	0.0%	0.0%	0.0%
tr|F1SJX4|F1SJX4	1	0	1	0	0	0	0	0	1	0	1	0	0	0	0	0	5.5%	0.0%	3.6%	0.0%	0.0%	0.0%	0.0%	0.0%
tr|I3LP39|I3LP39	1	0	1	0	0	0	0	0	1	0	1	0	0	0	0	0	6.7%	0.0%	6.4%	0.0%	0.0%	0.0%	0.0%	0.0%
tr|F1S4R2|F1S4R2	1	0	1	0	0	0	0	0	1	0	1	0	0	0	0	0	3.4%	0.0%	1.8%	0.0%	0.0%	0.0%	0.0%	0.0%
tr|F1S765|F1S765	1	0	1	0	0	0	0	0	1	0	2	0	0	0	0	0	10.6%	0.0%	10.6%	0.0%	0.0%	0.0%	0.0%	0.0%
tr|F1SU22|F1SU22	1	0	1	0	0	0	0	0	1	0	2	0	0	0	0	0	4.3%	0.0%	4.3%	0.0%	0.0%	0.0%	0.0%	0.0%
tr|F1S2Z5|F1S2Z5	0	1	1	0	0	0	0	0	0	1	2	0	0	0	0	0	0.0%	6.2%	12.0%	0.0%	0.0%	0.0%	0.0%	0.0%
tr|F1S1F3|F1S1F3	1	0	1	0	0	0	0	0	1	0	2	0	0	0	0	0	8.5%	0.0%	11.3%	0.0%	0.0%	0.0%	0.0%	0.0%
tr|F1SQY0|F1SQY0	1	0	1	0	0	0	0	0	1	0	2	0	0	0	0	0	7.9%	0.0%	7.9%	0.0%	0.0%	0.0%	0.0%	0.0%
Q9GKX6	1	0	1	0	0	0	0	0	1	0	2	0	0	0	0	0	5.0%	0.0%	4.1%	0.0%	0.0%	0.0%	0.0%	0.0%

	Peptide Count								Spectral Count								Sequence Coverage							
	Whole Cell	Whole Cell	Whole Cell	Cilia Fraction	Cilia Fraction	Cilia Fraction	Cilia Fraction	Cilia Fraction	Whole Cell	Whole Cell	Whole Cell	Cilia Fraction	Cilia Fraction	Cilia Fraction	Cilia Fraction	Cilia Fraction	Whole Cell	Whole Cell	Whole Cell	Cilia Fraction	Cilia Fraction	Cilia Fraction	Cilia Fraction	Cilia Fraction
Sample#	1	2	6	4	3	5	7	8	1	2	6	4	3	5	7	8	1	2	6	4	3	5	7	8
**Accession**																								
tr|I3LV12|I3LV12	1	0	1	0	0	0	0	0	1	0	2	0	0	0	0	0	7.5%	0.0%	7.5%	0.0%	0.0%	0.0%	0.0%	0.0%
tr|A5D9P5|A5D9P5	1	0	2	0	0	0	0	0	1	0	2	0	0	0	0	0	2.2%	0.0%	5.3%	0.0%	0.0%	0.0%	0.0%	0.0%
Q8MI68	1	0	1	0	0	0	0	0	1	0	2	0	0	0	0	0	2.9%	0.0%	3.2%	0.0%	0.0%	0.0%	0.0%	0.0%
tr|F1RZT5|F1RZT5	0	1	1	0	0	0	0	0	0	1	2	0	0	0	0	0	0.0%	1.5%	2.8%	0.0%	0.0%	0.0%	0.0%	0.0%
tr|I3LIU9|I3LIU9	1	0	1	0	0	0	0	0	1	0	2	0	0	0	0	0	16.2%	0.0%	11.5%	0.0%	0.0%	0.0%	0.0%	0.0%
tr|F1RSM4|F1RSM4	1	0	1	0	0	0	0	0	1	0	2	0	0	0	0	0	5.7%	0.0%	3.4%	0.0%	0.0%	0.0%	0.0%	0.0%
tr|I3LN18|I3LN18	1	0	1	0	0	0	0	0	1	0	2	0	0	0	0	0	3.0%	0.0%	2.4%	0.0%	0.0%	0.0%	0.0%	0.0%
tr|F1RUT8|F1RUT8	1	0	1	0	0	0	0	0	1	0	2	0	0	0	0	0	3.3%	0.0%	2.8%	0.0%	0.0%	0.0%	0.0%	0.0%
tr|I3LIW1|I3LIW1	1	0	1	0	0	0	0	0	1	0	2	0	0	0	0	0	2.2%	0.0%	4.4%	0.0%	0.0%	0.0%	0.0%	0.0%
tr|F2Z5Q9|F2Z5Q9	1	0	1	0	0	0	0	0	1	0	2	0	0	0	0	0	15.4%	0.0%	10.9%	0.0%	0.0%	0.0%	0.0%	0.0%
tr|F1RTN1|F1RTN1	1	0	1	0	0	0	0	0	1	0	2	0	0	0	0	0	2.1%	0.0%	2.2%	0.0%	0.0%	0.0%	0.0%	0.0%
tr|F1RUE0|F1RUE0	0	1	1	0	0	0	0	0	0	1	2	0	0	0	0	0	0.0%	15.8%	12.3%	0.0%	0.0%	0.0%	0.0%	0.0%
tr|F2Z530|F2Z530	1	0	1	0	0	0	0	0	1	0	2	0	0	0	0	0	4.8%	0.0%	6.0%	0.0%	0.0%	0.0%	0.0%	0.0%
tr|F1ST39|F1ST39	0	1	1	0	0	0	0	0	0	1	2	0	0	0	0	0	0.0%	1.8%	4.2%	0.0%	0.0%	0.0%	0.0%	0.0%
tr|A5D9P3|A5D9P3	1	0	2	0	0	0	0	0	1	0	2	0	0	0	0	0	1.7%	0.0%	4.1%	0.0%	0.0%	0.0%	0.0%	0.0%
tr|F1S3R5|F1S3R5	1	0	1	0	0	0	0	0	1	0	2	0	0	0	0	0	2.6%	0.0%	5.2%	0.0%	0.0%	0.0%	0.0%	0.0%
tr|F1SMW5|F1SMW5	0	1	1	0	0	0	0	0	0	1	2	0	0	0	0	0	0.0%	4.2%	4.8%	0.0%	0.0%	0.0%	0.0%	0.0%
tr|F1SSB8|F1SSB8	0	1	1	0	0	0	0	0	0	1	3	0	0	0	0	0	0.0%	1.4%	2.6%	0.0%	0.0%	0.0%	0.0%	0.0%
tr|F1RRJ1|F1RRJ1	0	1	1	0	0	0	0	0	0	1	3	0	0	0	0	0	0.0%	2.3%	3.1%	0.0%	0.0%	0.0%	0.0%	0.0%
tr|F1RG56|F1RG56	1	0	1	0	0	0	0	0	1	0	3	0	0	0	0	0	2.4%	0.0%	5.8%	0.0%	0.0%	0.0%	0.0%	0.0%
tr|I3LLG4|I3LLG4	0	1	1	0	0	0	0	0	0	1	3	0	0	0	0	0	0.0%	7.4%	3.4%	0.0%	0.0%	0.0%	0.0%	0.0%
tr|K7GN28|K7GN28	1	0	1	0	0	0	0	0	1	0	3	0	0	0	0	0	6.0%	0.0%	10.9%	0.0%	0.0%	0.0%	0.0%	0.0%
tr|F1S3K4|F1S3K4	1	0	1	0	0	0	0	0	1	0	3	0	0	0	0	0	5.9%	0.0%	10.8%	0.0%	0.0%	0.0%	0.0%	0.0%
tr|I3LLA0|I3LLA0	1	0	1	0	0	0	0	0	1	0	3	0	0	0	0	0	3.3%	0.0%	3.5%	0.0%	0.0%	0.0%	0.0%	0.0%
tr|I3L825|I3L825	1	0	1	0	0	0	0	0	1	0	3	0	0	0	0	0	4.2%	0.0%	7.3%	0.0%	0.0%	0.0%	0.0%	0.0%
tr|F1SCJ2|F1SCJ2	0	1	1	0	0	0	0	0	0	1	3	0	0	0	0	0	0.0%	1.2%	1.5%	0.0%	0.0%	0.0%	0.0%	0.0%
tr|F1SUU7|F1SUU7	1	0	1	0	0	0	0	0	1	0	3	0	0	0	0	0	3.5%	0.0%	7.0%	0.0%	0.0%	0.0%	0.0%	0.0%
tr|F1RRL4|F1RRL4	0	1	1	0	0	0	0	0	0	1	3	0	0	0	0	0	0.0%	2.4%	3.2%	0.0%	0.0%	0.0%	0.0%	0.0%
tr|I3LIP6|I3LIP6	1	0	1	0	0	0	0	0	1	0	3	0	0	0	0	0	2.8%	0.0%	3.7%	0.0%	0.0%	0.0%	0.0%	0.0%
tr|F1RJC5|F1RJC5	1	0	1	0	0	0	0	0	1	0	3	0	0	0	0	0	1.7%	0.0%	2.2%	0.0%	0.0%	0.0%	0.0%	0.0%
tr|F1S4A6|F1S4A6	1	0	1	0	0	0	0	0	1	0	3	0	0	0	0	0	8.3%	0.0%	8.3%	0.0%	0.0%	0.0%	0.0%	0.0%
tr|F1S852|F1S852	1	0	1	0	0	0	0	0	1	0	3	0	0	0	0	0	6.2%	0.0%	6.2%	0.0%	0.0%	0.0%	0.0%	0.0%
tr|K7GKV9|K7GKV9	1	0	1	0	0	0	0	0	1	0	3	0	0	0	0	0	5.3%	0.0%	5.3%	0.0%	0.0%	0.0%	0.0%	0.0%
tr|F1S1E0|F1S1E0	0	1	1	0	0	0	0	0	0	1	3	0	0	0	0	0	0.0%	2.6%	3.2%	0.0%	0.0%	0.0%	0.0%	0.0%
tr|F1RQI0|F1RQI0	1	0	1	0	0	0	0	0	1	0	3	0	0	0	0	0	2.5%	0.0%	1.9%	0.0%	0.0%	0.0%	0.0%	0.0%
tr|F1SEG4|F1SEG4	0	1	1	0	0	0	0	0	0	1	3	0	0	0	0	0	0.0%	2.5%	5.2%	0.0%	0.0%	0.0%	0.0%	0.0%
tr|I3LPR4|I3LPR4	1	0	1	0	0	0	0	0	1	0	3	0	0	0	0	0	5.2%	0.0%	5.2%	0.0%	0.0%	0.0%	0.0%	0.0%
Q56P27	1	0	1	0	0	0	0	0	1	0	3	0	0	0	0	0	11.1%	0.0%	5.6%	0.0%	0.0%	0.0%	0.0%	0.0%
tr|K7GQ41|K7GQ41	1	0	3	0	0	0	0	0	1	0	3	0	0	0	0	0	7.5%	0.0%	13.0%	0.0%	0.0%	0.0%	0.0%	0.0%
tr|I3LGV4|I3LGV4	1	0	1	0	0	0	0	0	1	0	3	0	0	0	0	0	3.1%	0.0%	3.1%	0.0%	0.0%	0.0%	0.0%	0.0%
Q864R5	1	0	1	0	0	0	0	0	1	0	3	0	0	0	0	0	2.4%	0.0%	5.8%	0.0%	0.0%	0.0%	0.0%	0.0%
tr|F1RGX0|F1RGX0	1	0	1	0	0	0	0	0	1	0	4	0	0	0	0	0	3.3%	0.0%	3.7%	0.0%	0.0%	0.0%	0.0%	0.0%
tr|I3LEW6|I3LEW6	1	0	2	0	0	0	0	0	1	0	4	0	0	0	0	0	7.2%	0.0%	11.6%	0.0%	0.0%	0.0%	0.0%	0.0%
tr|F1SS61|F1SS61	1	0	1	0	0	0	0	0	1	0	4	0	0	0	0	0	0.5%	0.0%	0.5%	0.0%	0.0%	0.0%	0.0%	0.0%
tr|F1S9D6|F1S9D6	1	0	1	0	0	0	0	0	1	0	4	0	0	0	0	0	0.5%	0.0%	0.5%	0.0%	0.0%	0.0%	0.0%	0.0%
tr|F1RGH8|F1RGH8	1	0	2	0	0	0	0	0	1	0	4	0	0	0	0	0	1.8%	0.0%	7.1%	0.0%	0.0%	0.0%	0.0%	0.0%
tr|F1SS65|F1SS65	1	0	1	0	0	0	0	0	1	0	4	0	0	0	0	0	0.6%	0.0%	0.6%	0.0%	0.0%	0.0%	0.0%	0.0%
tr|F1RRD7|F1RRD7	1	0	1	0	0	0	0	0	1	0	4	0	0	0	0	0	2.3%	0.0%	2.3%	0.0%	0.0%	0.0%	0.0%	0.0%
Q9TV61	1	0	1	0	0	0	0	0	1	0	4	0	0	0	0	0	0.5%	0.0%	0.5%	0.0%	0.0%	0.0%	0.0%	0.0%
Q9TV63	1	0	1	0	0	0	0	0	1	0	4	0	0	0	0	0	0.5%	0.0%	0.5%	0.0%	0.0%	0.0%	0.0%	0.0%
Q9TV62	1	0	1	0	0	0	0	0	1	0	4	0	0	0	0	0	0.5%	0.0%	0.5%	0.0%	0.0%	0.0%	0.0%	0.0%
tr|F1SS62|F1SS62	1	0	1	0	0	0	0	0	1	0	4	0	0	0	0	0	0.6%	0.0%	0.6%	0.0%	0.0%	0.0%	0.0%	0.0%
P81271	1	0	1	0	0	0	0	0	1	0	4	0	0	0	0	0	13.8%	0.0%	13.8%	0.0%	0.0%	0.0%	0.0%	0.0%
tr|F1SQA6|F1SQA6	1	0	2	0	0	0	0	0	1	0	4	0	0	0	0	0	3.4%	0.0%	7.8%	0.0%	0.0%	0.0%	0.0%	0.0%
P79293	1	0	1	0	0	0	0	0	1	0	4	0	0	0	0	0	0.5%	0.0%	0.5%	0.0%	0.0%	0.0%	0.0%	0.0%
tr|F1RG17|F1RG17	1	0	1	0	0	0	0	0	1	0	4	0	0	0	0	0	11.2%	0.0%	10.0%	0.0%	0.0%	0.0%	0.0%	0.0%
Q27HK4	1	0	2	0	0	0	0	0	1	0	4	0	0	0	0	0	4.4%	0.0%	9.5%	0.0%	0.0%	0.0%	0.0%	0.0%
tr|F1SF13|F1SF13	1	0	1	0	0	0	0	0	1	0	4	0	0	0	0	0	7.4%	0.0%	5.2%	0.0%	0.0%	0.0%	0.0%	0.0%
tr|F1SS66|F1SS66	1	0	1	0	0	0	0	0	1	0	4	0	0	0	0	0	0.5%	0.0%	0.5%	0.0%	0.0%	0.0%	0.0%	0.0%
tr|I3LJH0|I3LJH0	1	0	1	0	0	0	0	0	1	0	4	0	0	0	0	0	9.5%	0.0%	9.5%	0.0%	0.0%	0.0%	0.0%	0.0%
tr|F1SN89|F1SN89	1	0	1	0	0	0	0	0	1	0	4	0	0	0	0	0	1.0%	0.0%	2.1%	0.0%	0.0%	0.0%	0.0%	0.0%
tr|F1SNY4|F1SNY4	1	0	2	0	0	0	0	0	1	0	4	0	0	0	0	0	4.4%	0.0%	6.7%	0.0%	0.0%	0.0%	0.0%	0.0%
tr|I3L897|I3L897	1	0	1	0	0	0	0	0	1	0	4	0	0	0	0	0	4.7%	0.0%	4.7%	0.0%	0.0%	0.0%	0.0%	0.0%
tr|F1SLR6|F1SLR6	1	0	2	0	0	0	0	0	1	0	4	0	0	0	0	0	1.8%	0.0%	3.7%	0.0%	0.0%	0.0%	0.0%	0.0%
tr|K7GLB8|K7GLB8	1	0	2	0	0	0	0	0	1	0	4	0	0	0	0	0	4.4%	0.0%	6.7%	0.0%	0.0%	0.0%	0.0%	0.0%
tr|F1SS64|F1SS64	1	0	1	0	0	0	0	0	1	0	4	0	0	0	0	0	0.5%	0.0%	0.5%	0.0%	0.0%	0.0%	0.0%	0.0%
tr|F1S4X7|F1S4X7	1	0	1	0	0	0	0	0	1	0	4	0	0	0	0	0	0.5%	0.0%	0.5%	0.0%	0.0%	0.0%	0.0%	0.0%
Q06AT9	1	0	2	0	0	0	0	0	1	0	4	0	0	0	0	0	3.3%	0.0%	5.8%	0.0%	0.0%	0.0%	0.0%	0.0%
tr|I3LTH3|I3LTH3	1	0	1	0	0	0	0	0	1	0	4	0	0	0	0	0	3.4%	0.0%	3.4%	0.0%	0.0%	0.0%	0.0%	0.0%
tr|F1SJT9|F1SJT9	1	0	2	0	0	0	0	0	1	0	4	0	0	0	0	0	2.7%	0.0%	6.4%	0.0%	0.0%	0.0%	0.0%	0.0%
tr|F1SH40|F1SH40	1	0	2	0	0	0	0	0	1	0	5	0	0	0	0	0	5.2%	0.0%	5.5%	0.0%	0.0%	0.0%	0.0%	0.0%
tr|F1SPJ0|F1SPJ0	1	0	2	0	0	0	0	0	1	0	5	0	0	0	0	0	6.4%	0.0%	16.2%	0.0%	0.0%	0.0%	0.0%	0.0%
tr|K7GMD9|K7GMD9	1	0	2	0	0	0	0	0	1	0	5	0	0	0	0	0	10.2%	0.0%	14.9%	0.0%	0.0%	0.0%	0.0%	0.0%
P42174	1	0	1	0	0	0	0	0	1	0	5	0	0	0	0	0	45.5%	0.0%	45.5%	0.0%	0.0%	0.0%	0.0%	0.0%
O02858	1	0	2	0	0	0	0	0	1	0	5	0	0	0	0	0	9.3%	0.0%	7.5%	0.0%	0.0%	0.0%	0.0%	0.0%
tr|F1SEH0|F1SEH0	1	0	1	0	0	0	0	0	1	0	5	0	0	0	0	0	1.9%	0.0%	2.6%	0.0%	0.0%	0.0%	0.0%	0.0%
tr|F1SKU1|F1SKU1	1	0	1	0	0	0	0	0	1	0	5	0	0	0	0	0	10.9%	0.0%	4.8%	0.0%	0.0%	0.0%	0.0%	0.0%
tr|F1S3I0|F1S3I0	1	0	1	0	0	0	0	0	1	0	5	0	0	0	0	0	3.9%	0.0%	6.3%	0.0%	0.0%	0.0%	0.0%	0.0%
tr|F1SFF5|F1SFF5	1	0	2	0	0	0	0	0	1	0	5	0	0	0	0	0	2.1%	0.0%	4.8%	0.0%	0.0%	0.0%	0.0%	0.0%
tr|I3LIK0|I3LIK0	1	0	1	0	0	0	0	0	1	0	5	0	0	0	0	0	9.1%	0.0%	9.1%	0.0%	0.0%	0.0%	0.0%	0.0%
tr|Q6RWA7|Q6RWA7	1	0	2	0	0	0	0	0	1	0	5	0	0	0	0	0	8.6%	0.0%	7.0%	0.0%	0.0%	0.0%	0.0%	0.0%

	Peptide Count								Spectral Count								Sequence Coverage							
	Whole Cell	Whole Cell	Whole Cell	Cilia Fraction	Cilia Fraction	Cilia Fraction	Cilia Fraction	Cilia Fraction	Whole Cell	Whole Cell	Whole Cell	Cilia Fraction	Cilia Fraction	Cilia Fraction	Cilia Fraction	Cilia Fraction	Whole Cell	Whole Cell	Whole Cell	Cilia Fraction	Cilia Fraction	Cilia Fraction	Cilia Fraction	Cilia Fraction
Sample#	1	2	6	4	3	5	7	8	1	2	6	4	3	5	7	8	1	2	6	4	3	5	7	8
**Accession**																								
tr|I3LTB0|I3LTB0	1	0	1	0	0	0	0	0	1	0	5	0	0	0	0	0	4.4%	0.0%	6.1%	0.0%	0.0%	0.0%	0.0%	0.0%
tr|F1ST04|F1ST04	1	0	2	0	0	0	0	0	1	0	6	0	0	0	0	0	1.8%	0.0%	1.8%	0.0%	0.0%	0.0%	0.0%	0.0%
tr|F1SRQ3|F1SRQ3	1	0	1	0	0	0	0	0	1	0	6	0	0	0	0	0	3.1%	0.0%	3.1%	0.0%	0.0%	0.0%	0.0%	0.0%
tr|I3LR95|I3LR95	1	0	1	0	0	0	0	0	1	0	6	0	0	0	0	0	19.2%	0.0%	19.2%	0.0%	0.0%	0.0%	0.0%	0.0%
tr|I3L723|I3L723	1	0	2	0	0	0	0	0	1	0	6	0	0	0	0	0	10.5%	0.0%	22.1%	0.0%	0.0%	0.0%	0.0%	0.0%
tr|F1RRJ4|F1RRJ4	1	0	1	0	0	0	0	0	1	0	6	0	0	0	0	0	6.0%	0.0%	6.0%	0.0%	0.0%	0.0%	0.0%	0.0%
tr|I3LCN6|I3LCN6	1	0	1	0	0	0	0	0	1	0	6	0	0	0	0	0	7.1%	0.0%	9.0%	0.0%	0.0%	0.0%	0.0%	0.0%
tr|F1S994|F1S994	1	0	2	0	0	0	0	0	1	0	6	0	0	0	0	0	12.7%	0.0%	12.7%	0.0%	0.0%	0.0%	0.0%	0.0%
tr|I3LRZ7|I3LRZ7	1	0	1	0	0	0	0	0	1	0	6	0	0	0	0	0	8.2%	0.0%	8.2%	0.0%	0.0%	0.0%	0.0%	0.0%
O77591	1	0	3	0	0	0	0	0	1	0	6	0	0	0	0	0	3.6%	0.0%	13.7%	0.0%	0.0%	0.0%	0.0%	0.0%
tr|I3LLA2|I3LLA2	0	1	1	0	0	0	0	0	0	1	6	0	0	0	0	0	0.0%	4.4%	11.8%	0.0%	0.0%	0.0%	0.0%	0.0%
tr|F1REW6|F1REW6	1	0	1	0	0	0	0	0	1	0	6	0	0	0	0	0	1.8%	0.0%	2.8%	0.0%	0.0%	0.0%	0.0%	0.0%
tr|F1RIU0|F1RIU0	1	0	1	0	0	0	0	0	1	0	6	0	0	0	0	0	22.3%	0.0%	22.3%	0.0%	0.0%	0.0%	0.0%	0.0%
tr|F1SG95|F1SG95	1	0	3	0	0	0	0	0	1	0	7	0	0	0	0	0	8.3%	0.0%	10.8%	0.0%	0.0%	0.0%	0.0%	0.0%
tr|F1SR88|F1SR88	1	0	1	0	0	0	0	0	1	0	7	0	0	0	0	0	2.4%	0.0%	4.6%	0.0%	0.0%	0.0%	0.0%	0.0%
tr|F1SED4|F1SED4	1	0	2	0	0	0	0	0	1	0	7	0	0	0	0	0	6.5%	0.0%	6.7%	0.0%	0.0%	0.0%	0.0%	0.0%
tr|F1SEJ0|F1SEJ0	1	0	1	0	0	0	0	0	1	0	7	0	0	0	0	0	4.7%	0.0%	4.7%	0.0%	0.0%	0.0%	0.0%	0.0%
tr|I3LJQ7|I3LJQ7	1	0	3	0	0	0	0	0	1	0	7	0	0	0	0	0	1.6%	0.0%	7.0%	0.0%	0.0%	0.0%	0.0%	0.0%
P80230	1	0	1	0	0	0	0	0	1	0	7	0	0	0	0	0	46.9%	0.0%	46.9%	0.0%	0.0%	0.0%	0.0%	0.0%
tr|F1REX8|F1REX8	1	0	2	0	0	0	0	0	1	0	8	0	0	0	0	0	2.3%	0.0%	2.2%	0.0%	0.0%	0.0%	0.0%	0.0%
tr|F1SA62|F1SA62	1	0	3	0	0	0	0	0	1	0	8	0	0	0	0	0	15.7%	0.0%	15.7%	0.0%	0.0%	0.0%	0.0%	0.0%
tr|K7GSK4|K7GSK4	1	0	1	0	0	0	0	0	1	0	8	0	0	0	0	0	10.8%	0.0%	10.8%	0.0%	0.0%	0.0%	0.0%	0.0%
tr|F1RWZ9|F1RWZ9	1	0	1	0	0	0	0	0	1	0	8	0	0	0	0	0	11.0%	0.0%	11.0%	0.0%	0.0%	0.0%	0.0%	0.0%
tr|I3L949|I3L949	1	0	2	0	0	0	0	0	1	0	8	0	0	0	0	0	4.3%	0.0%	8.4%	0.0%	0.0%	0.0%	0.0%	0.0%
tr|I3LHC7|I3LHC7	0	1	1	0	0	0	0	0	0	1	8	0	0	0	0	0	0.0%	2.5%	5.1%	0.0%	0.0%	0.0%	0.0%	0.0%
tr|F1SED8|F1SED8	1	0	3	0	0	0	0	0	1	0	9	0	0	0	0	0	1.9%	0.0%	5.3%	0.0%	0.0%	0.0%	0.0%	0.0%
tr|F1S7D6|F1S7D6	1	0	2	0	0	0	0	0	1	0	9	0	0	0	0	0	2.8%	0.0%	5.8%	0.0%	0.0%	0.0%	0.0%	0.0%
tr|I3LPF8|I3LPF8	1	0	3	0	0	0	0	0	1	0	9	0	0	0	0	0	2.3%	0.0%	4.2%	0.0%	0.0%	0.0%	0.0%	0.0%
tr|I3LMV8|I3LMV8	0	1	1	0	0	0	0	0	0	1	9	0	0	0	0	0	0.0%	1.1%	1.8%	0.0%	0.0%	0.0%	0.0%	0.0%
tr|F1SPH2|F1SPH2	1	0	2	0	0	0	0	0	1	0	10	0	0	0	0	0	6.2%	0.0%	3.1%	0.0%	0.0%	0.0%	0.0%	0.0%
Q767K8	1	0	1	0	0	0	0	0	1	0	10	0	0	0	0	0	3.9%	0.0%	13.6%	0.0%	0.0%	0.0%	0.0%	0.0%
tr|I3LCX9|I3LCX9	1	0	1	0	0	0	0	0	1	0	11	0	0	0	0	0	5.5%	0.0%	2.7%	0.0%	0.0%	0.0%	0.0%	0.0%
tr|F1RPE5|F1RPE5	1	0	1	0	0	0	0	0	1	0	11	0	0	0	0	0	5.4%	0.0%	2.7%	0.0%	0.0%	0.0%	0.0%	0.0%
tr|I3L6I8|I3L6I8	1	0	4	0	0	0	0	0	1	0	11	0	0	0	0	0	5.8%	0.0%	17.0%	0.0%	0.0%	0.0%	0.0%	0.0%
tr|F1SGC6|F1SGC6	1	0	3	0	0	0	0	0	1	0	11	0	0	0	0	0	7.9%	0.0%	12.2%	0.0%	0.0%	0.0%	0.0%	0.0%
tr|I3LKX8|I3LKX8	1	0	2	0	0	0	0	0	1	0	13	0	0	0	0	0	8.1%	0.0%	11.9%	0.0%	0.0%	0.0%	0.0%	0.0%
tr|F1SBU8|F1SBU8	0	1	3	0	0	0	0	0	0	1	13	0	0	0	0	0	0.0%	18.6%	39.8%	0.0%	0.0%	0.0%	0.0%	0.0%
tr|F1RXX7|F1RXX7	1	0	2	0	0	0	0	0	1	0	13	0	0	0	0	0	7.1%	0.0%	10.5%	0.0%	0.0%	0.0%	0.0%	0.0%
tr|I3LAS7|I3LAS7	1	0	2	0	0	0	0	0	1	0	13	0	0	0	0	0	8.4%	0.0%	12.4%	0.0%	0.0%	0.0%	0.0%	0.0%
tr|I3LDD0|I3LDD0	1	0	2	0	0	0	0	0	1	0	13	0	0	0	0	0	8.2%	0.0%	12.1%	0.0%	0.0%	0.0%	0.0%	0.0%
tr|F1SNF7|F1SNF7	1	0	1	0	0	0	0	0	1	0	13	0	0	0	0	0	3.4%	0.0%	4.4%	0.0%	0.0%	0.0%	0.0%	0.0%
tr|F1SSK4|F1SSK4	1	0	2	0	0	0	0	0	1	0	14	0	0	0	0	0	18.0%	0.0%	34.8%	0.0%	0.0%	0.0%	0.0%	0.0%
tr|I3LLT5|I3LLT5	1	0	4	0	0	0	0	0	1	0	14	0	0	0	0	0	5.5%	0.0%	9.2%	0.0%	0.0%	0.0%	0.0%	0.0%
tr|I3LIW3|I3LIW3	1	0	5	0	0	0	0	0	1	0	15	0	0	0	0	0	11.4%	0.0%	41.0%	0.0%	0.0%	0.0%	0.0%	0.0%
tr|I3LR30|I3LR30	1	0	1	0	0	0	0	0	1	0	16	0	0	0	0	0	19.2%	0.0%	19.2%	0.0%	0.0%	0.0%	0.0%	0.0%
tr|F1SF11|F1SF11	1	0	2	0	0	0	0	0	1	0	16	0	0	0	0	0	2.3%	0.0%	4.5%	0.0%	0.0%	0.0%	0.0%	0.0%
tr|F1SMF9|F1SMF9	1	0	2	0	0	0	0	0	1	0	17	0	0	0	0	0	5.6%	0.0%	11.9%	0.0%	0.0%	0.0%	0.0%	0.0%
Q29228	0	1	3	0	0	0	0	0	0	1	21	0	0	0	0	0	0.0%	13.0%	23.2%	0.0%	0.0%	0.0%	0.0%	0.0%
tr|F1S3H1|F1S3H1	1	0	3	0	0	0	0	0	1	0	25	0	0	0	0	0	1.7%	0.0%	10.3%	0.0%	0.0%	0.0%	0.0%	0.0%
tr|I3LU44|I3LU44	1	0	1	0	0	0	0	0	1	0	31	0	0	0	0	0	52.1%	0.0%	52.1%	0.0%	0.0%	0.0%	0.0%	0.0%
tr|F1S4V5|F1S4V5	1	0	2	0	0	0	0	0	1	0	33	0	0	0	0	0	2.3%	0.0%	3.4%	0.0%	0.0%	0.0%	0.0%	0.0%
Reverse_tr|F1RZ03|F1RZ03	1	0	1	0	0	0	0	0	1	0	41	0	0	0	0	0	1.2%	0.0%	1.2%	0.0%	0.0%	0.0%	0.0%	0.0%
Reverse_tr|I3LQZ3|I3LQZ3	1	0	1	0	0	0	0	0	1	0	41	0	0	0	0	0	1.0%	0.0%	1.0%	0.0%	0.0%	0.0%	0.0%	0.0%
tr|F1S7V1|F1S7V1	0	0	1	0	0	0	0	1	0	0	1	0	0	0	0	17	0.0%	0.0%	4.7%	0.0%	0.0%	0.0%	0.0%	5.8%
tr|F1RZV9|F1RZV9	0	0	1	0	0	0	1	8	0	0	2	0	0	0	2	29	0.0%	0.0%	4.5%	0.0%	0.0%	0.0%	6.8%	30.5%
tr|F1SJX9|F1SJX9	0	0	1	0	0	0	2	5	0	0	1	0	0	0	2	12	0.0%	0.0%	2.2%	0.0%	0.0%	0.0%	4.9%	10.1%
tr|F1SD57|F1SD57	0	0	1	0	0	0	1	4	0	0	2	0	0	0	4	21	0.0%	0.0%	4.7%	0.0%	0.0%	0.0%	4.7%	14.0%
tr|F1SD58|F1SD58	0	0	2	0	0	0	1	5	0	0	3	0	0	0	4	22	0.0%	0.0%	7.1%	0.0%	0.0%	0.0%	4.1%	15.4%
tr|F1RPA3|F1RPA3	0	0	1	0	0	0	2	2	0	0	4	0	0	0	12	17	0.0%	0.0%	3.5%	0.0%	0.0%	0.0%	6.6%	3.5%
tr|I3L5Z1|I3L5Z1	0	0	1	0	0	0	1	2	0	0	2	0	0	0	7	7	0.0%	0.0%	6.0%	0.0%	0.0%	0.0%	6.0%	16.4%
tr|I3L7W9|I3L7W9	0	0	2	0	0	0	0	2	0	0	4	0	0	0	0	27	0.0%	0.0%	6.5%	0.0%	0.0%	0.0%	0.0%	6.5%
tr|I3L5R2|I3L5R2	0	0	2	0	0	0	0	2	0	0	4	0	0	0	0	27	0.0%	0.0%	7.9%	0.0%	0.0%	0.0%	0.0%	7.9%
tr|F1S2F5|F1S2F5	0	0	1	0	0	0	0	2	0	0	2	0	0	0	0	13	0.0%	0.0%	11.0%	0.0%	0.0%	0.0%	0.0%	14.9%
Q2MJV8	0	0	2	0	0	0	4	7	0	0	9	0	0	0	24	32	0.0%	0.0%	14.2%	0.0%	0.0%	0.0%	26.4%	48.1%
tr|F1RWM5|F1RWM5	0	0	1	0	0	0	0	8	0	0	4	0	0	0	0	24	0.0%	0.0%	1.7%	0.0%	0.0%	0.0%	0.0%	13.4%
tr|K7GS43|K7GS43	0	0	1	0	0	0	0	8	0	0	4	0	0	0	0	24	0.0%	0.0%	1.7%	0.0%	0.0%	0.0%	0.0%	13.4%
tr|F1RZJ0|F1RZJ0	0	0	2	0	0	0	0	4	0	0	3	0	0	0	0	17	0.0%	0.0%	5.4%	0.0%	0.0%	0.0%	0.0%	11.8%
tr|F1S6R4|F1S6R4	0	0	2	0	0	0	2	2	0	0	9	0	0	0	22	23	0.0%	0.0%	23.6%	0.0%	0.0%	0.0%	16.7%	23.6%
tr|F1S0C8|F1S0C8	0	0	1	0	0	0	1	1	0	0	3	0	0	0	12	3	0.0%	0.0%	3.5%	0.0%	0.0%	0.0%	3.5%	3.5%
tr|K7GNZ2|K7GNZ2	0	0	2	0	0	0	0	2	0	0	2	0	0	0	0	10	0.0%	0.0%	12.1%	0.0%	0.0%	0.0%	0.0%	6.9%
tr|F1RMV8|F1RMV8	0	0	1	0	0	0	0	3	0	0	2	0	0	0	0	10	0.0%	0.0%	5.3%	0.0%	0.0%	0.0%	0.0%	11.4%
tr|K7GPM8|K7GPM8	0	0	1	0	0	0	0	7	0	0	4	0	0	0	0	19	0.0%	0.0%	2.0%	0.0%	0.0%	0.0%	0.0%	14.5%
tr|K7GPE2|K7GPE2	0	0	2	0	0	0	1	1	0	0	6	0	0	0	17	7	0.0%	0.0%	37.4%	0.0%	0.0%	0.0%	22.4%	22.4%
tr|F1RNP4|F1RNP4	0	0	1	0	0	0	1	1	0	0	1	0	0	0	2	2	0.0%	0.0%	5.9%	0.0%	0.0%	0.0%	5.9%	5.9%
tr|F1RZH3|F1RZH3	0	0	1	0	0	0	0	2	0	0	2	0	0	0	0	7	0.0%	0.0%	4.2%	0.0%	0.0%	0.0%	0.0%	5.8%
tr|F6Q1A4|F6Q1A4	0	0	1	0	0	0	0	2	0	0	4	0	0	0	0	12	0.0%	0.0%	3.8%	0.0%	0.0%	0.0%	0.0%	18.1%
tr|F1SB81|F1SB81	0	0	2	0	0	0	1	0	0	0	3	0	0	0	9	0	0.0%	0.0%	2.0%	0.0%	0.0%	0.0%	2.0%	0.0%
P06867	0	0	2	0	0	0	1	0	0	0	3	0	0	0	9	0	0.0%	0.0%	2.0%	0.0%	0.0%	0.0%	2.0%	0.0%
tr|F1SUT8|F1SUT8	0	0	1	0	0	0	1	1	0	0	1	0	0	0	1	2	0.0%	0.0%	1.6%	0.0%	0.0%	0.0%	3.4%	2.5%
tr|I3LSZ7|I3LSZ7	0	0	1	0	0	0	0	1	0	0	1	0	0	0	0	3	0.0%	0.0%	3.3%	0.0%	0.0%	0.0%	0.0%	3.0%
tr|I3LR80|I3LR80	0	0	1	0	0	0	0	1	0	0	1	0	0	0	0	3	0.0%	0.0%	4.2%	0.0%	0.0%	0.0%	0.0%	4.2%

	Peptide Count								Spectral Count								Sequence Coverage							
	Whole Cell	Whole Cell	Whole Cell	Cilia Fraction	Cilia Fraction	Cilia Fraction	Cilia Fraction	Cilia Fraction	Whole Cell	Whole Cell	Whole Cell	Cilia Fraction	Cilia Fraction	Cilia Fraction	Cilia Fraction	Cilia Fraction	Whole Cell	Whole Cell	Whole Cell	Cilia Fraction	Cilia Fraction	Cilia Fraction	Cilia Fraction	Cilia Fraction
Sample#	1	2	6	4	3	5	7	8	1	2	6	4	3	5	7	8	1	2	6	4	3	5	7	8
**Accession**																								
tr|F1RIP1|F1RIP1	0	0	1	0	0	0	0	2	0	0	2	0	0	0	0	5	0.0%	0.0%	4.1%	0.0%	0.0%	0.0%	0.0%	5.3%
tr|F1RF14|F1RF14	0	0	2	0	0	0	1	1	0	0	4	0	0	0	7	2	0.0%	0.0%	7.9%	0.0%	0.0%	0.0%	4.1%	4.1%
Reverse_tr|F1SER9|F1SER9	0	0	1	0	0	0	1	1	0	0	5	0	0	0	4	7	0.0%	0.0%	0.6%	0.0%	0.0%	0.0%	0.6%	0.6%
tr|I3LSF9|I3LSF9	0	0	1	0	0	0	1	1	0	0	5	0	0	0	8	2	0.0%	0.0%	16.2%	0.0%	0.0%	0.0%	16.2%	16.2%
tr|F1S1W0|F1S1W0	0	0	2	0	0	0	0	1	0	0	3	0	0	0	0	6	0.0%	0.0%	15.9%	0.0%	0.0%	0.0%	0.0%	4.7%
tr|F1SDG3|F1SDG3	0	0	1	0	0	0	0	1	0	0	1	0	0	0	0	2	0.0%	0.0%	2.3%	0.0%	0.0%	0.0%	0.0%	3.3%
tr|F1S0Q2|F1S0Q2	0	0	2	0	0	0	0	7	0	0	11	0	0	0	0	21	0.0%	0.0%	6.1%	0.0%	0.0%	0.0%	0.0%	18.9%
tr|F1SV11|F1SV11	0	0	1	0	0	0	1	2	0	0	4	0	0	0	3	4	0.0%	0.0%	2.0%	0.0%	0.0%	0.0%	2.0%	2.0%
F1RT67	0	0	2	0	0	0	0	1	0	0	7	0	0	0	0	8	0.0%	0.0%	8.6%	0.0%	0.0%	0.0%	0.0%	8.9%
tr|I3LA61|I3LA61	0	0	1	0	0	0	0	1	0	0	2	0	0	0	0	2	0.0%	0.0%	2.3%	0.0%	0.0%	0.0%	0.0%	4.6%
tr|F1RN91|F1RN91	0	0	1	0	0	0	1	1	0	0	2	0	0	0	1	1	0.0%	0.0%	1.5%	0.0%	0.0%	0.0%	1.5%	1.6%
tr|F1SB91|F1SB91	0	0	1	0	0	0	0	1	0	0	2	0	0	0	0	2	0.0%	0.0%	2.2%	0.0%	0.0%	0.0%	0.0%	4.5%
tr|I3L7J4|I3L7J4	0	0	1	0	0	0	0	1	0	0	1	0	0	0	0	1	0.0%	0.0%	1.9%	0.0%	0.0%	0.0%	0.0%	3.1%
Reverse_tr|I3LGH8|I3LGH8	0	0	1	0	0	0	0	1	0	0	1	0	0	0	0	1	0.0%	0.0%	8.6%	0.0%	0.0%	0.0%	0.0%	8.6%
tr|F1SJE5|F1SJE5	0	0	1	0	0	0	0	1	0	0	1	0	0	0	0	1	0.0%	0.0%	13.7%	0.0%	0.0%	0.0%	0.0%	13.7%
tr|F1S0L5|F1S0L5	0	0	1	0	0	0	0	1	0	0	1	0	0	0	0	1	0.0%	0.0%	7.3%	0.0%	0.0%	0.0%	0.0%	6.3%
tr|F1SFE2|F1SFE2	0	0	1	0	0	0	0	1	0	0	1	0	0	0	0	1	0.0%	0.0%	4.7%	0.0%	0.0%	0.0%	0.0%	6.8%
tr|F1RJH8|F1RJH8	0	0	1	0	0	0	1	0	0	0	1	0	0	0	1	0	0.0%	0.0%	3.2%	0.0%	0.0%	0.0%	4.7%	0.0%
tr|I3LPD2|I3LPD2	0	0	2	0	0	0	1	2	0	0	11	0	0	0	4	6	0.0%	0.0%	4.2%	0.0%	0.0%	0.0%	3.7%	4.2%
tr|F1SIN8|F1SIN8	0	0	2	0	0	0	1	2	0	0	11	0	0	0	4	6	0.0%	0.0%	4.0%	0.0%	0.0%	0.0%	3.5%	4.0%
tr|F1RNR7|F1RNR7	0	0	1	0	0	0	0	1	0	0	4	0	0	0	0	3	0.0%	0.0%	9.4%	0.0%	0.0%	0.0%	0.0%	4.9%
tr|F1RN89|F1RN89	0	0	1	0	0	0	0	1	0	0	3	0	0	0	0	2	0.0%	0.0%	3.2%	0.0%	0.0%	0.0%	0.0%	4.7%
tr|F1SMS9|F1SMS9	0	0	1	0	0	0	0	1	0	0	3	0	0	0	0	2	0.0%	0.0%	3.3%	0.0%	0.0%	0.0%	0.0%	3.3%
tr|I3LKC9|I3LKC9	0	0	2	0	0	0	1	1	0	0	8	0	0	0	3	2	0.0%	0.0%	9.9%	0.0%	0.0%	0.0%	9.9%	9.9%
tr|F1S4P4|F1S4P4	0	0	1	0	0	0	2	0	0	0	8	0	0	0	5	0	0.0%	0.0%	7.8%	0.0%	0.0%	0.0%	14.2%	0.0%
tr|F1SU35|F1SU35	0	0	1	0	0	0	0	1	0	0	4	0	0	0	0	2	0.0%	0.0%	5.6%	0.0%	0.0%	0.0%	0.0%	11.0%
tr|K7GMI0|K7GMI0	0	0	1	0	0	0	0	1	0	0	4	0	0	0	0	2	0.0%	0.0%	8.6%	0.0%	0.0%	0.0%	0.0%	6.5%
tr|K7GNV9|K7GNV9	0	0	1	0	0	0	0	1	0	0	4	0	0	0	0	2	0.0%	0.0%	12.0%	0.0%	0.0%	0.0%	0.0%	9.0%
tr|F1REY0|F1REY0	0	0	1	0	0	0	1	0	0	0	2	0	0	0	1	0	0.0%	0.0%	6.3%	0.0%	0.0%	0.0%	8.7%	0.0%
tr|I3LNH7|I3LNH7	0	0	1	0	0	0	0	1	0	0	2	0	0	0	0	1	0.0%	0.0%	5.8%	0.0%	0.0%	0.0%	0.0%	9.7%
tr|I3L810|I3L810	0	0	1	0	0	0	0	1	0	0	2	0	0	0	0	1	0.0%	0.0%	5.8%	0.0%	0.0%	0.0%	0.0%	6.2%
tr|F1RL41|F1RL41	0	0	2	0	0	0	0	1	0	0	2	0	0	0	0	1	0.0%	0.0%	7.3%	0.0%	0.0%	0.0%	0.0%	6.8%
tr|I3LKZ2|I3LKZ2	0	0	1	0	0	0	0	1	0	0	2	0	0	0	0	1	0.0%	0.0%	3.3%	0.0%	0.0%	0.0%	0.0%	2.9%
tr|I3LM16|I3LM16	0	0	3	0	0	0	1	0	0	0	10	0	0	0	4	0	0.0%	0.0%	17.1%	0.0%	0.0%	0.0%	7.1%	0.0%
tr|F1RWM4|F1RWM4	0	0	1	0	0	0	0	1	0	0	5	0	0	0	0	2	0.0%	0.0%	16.9%	0.0%	0.0%	0.0%	0.0%	16.9%
tr|I3LM89|I3LM89	0	0	1	0	0	0	1	0	0	0	3	0	0	0	1	0	0.0%	0.0%	30.0%	0.0%	0.0%	0.0%	30.0%	0.0%
tr|F1RTT0|F1RTT0	0	0	1	0	0	0	0	1	0	0	3	0	0	0	0	1	0.0%	0.0%	3.4%	0.0%	0.0%	0.0%	0.0%	2.8%
tr|F1RG09|F1RG09	0	0	1	0	0	0	0	1	0	0	3	0	0	0	0	1	0.0%	0.0%	3.2%	0.0%	0.0%	0.0%	0.0%	3.2%
tr|F2Z5S6|F2Z5S6	0	0	2	0	0	0	1	1	0	0	7	0	0	0	1	1	0.0%	0.0%	22.7%	0.0%	0.0%	0.0%	10.7%	10.7%
tr|F2Z563|F2Z563	0	0	1	0	0	0	0	1	0	0	8	0	0	0	0	2	0.0%	0.0%	7.0%	0.0%	0.0%	0.0%	0.0%	19.2%
tr|K7GSE5|K7GSE5	0	0	2	0	0	0	1	1	0	0	20	0	0	0	1	3	0.0%	0.0%	22.3%	0.0%	0.0%	0.0%	15.6%	15.6%
tr|F1RRP9|F1RRP9	0	0	2	0	0	0	1	1	0	0	20	0	0	0	1	3	0.0%	0.0%	22.2%	0.0%	0.0%	0.0%	15.6%	15.6%
tr|F1SUF3|F1SUF3	0	0	1	0	0	0	0	1	0	0	10	0	0	0	0	2	0.0%	0.0%	1.3%	0.0%	0.0%	0.0%	0.0%	2.6%
tr|F1S4I1|F1S4I1	0	0	2	0	0	0	1	0	0	0	5	0	0	0	1	0	0.0%	0.0%	10.3%	0.0%	0.0%	0.0%	2.9%	0.0%
Q6J1I8	0	0	1	0	0	0	0	1	0	0	5	0	0	0	0	1	0.0%	0.0%	7.9%	0.0%	0.0%	0.0%	0.0%	11.0%
tr|F2Z5W4|F2Z5W4	0	0	3	0	0	0	1	2	0	0	170	0	0	0	12	14	0.0%	0.0%	37.6%	0.0%	0.0%	0.0%	7.5%	23.3%
tr|F1RR65|F1RR65	0	0	1	0	0	0	0	1	0	0	7	0	0	0	0	1	0.0%	0.0%	5.0%	0.0%	0.0%	0.0%	0.0%	2.8%
tr|F1SCL3|F1SCL3	0	0	1	0	0	0	0	1	0	0	8	0	0	0	0	1	0.0%	0.0%	3.7%	0.0%	0.0%	0.0%	0.0%	5.1%
tr|F1SCM6|F1SCM6	0	0	1	0	0	0	0	1	0	0	8	0	0	0	0	1	0.0%	0.0%	3.7%	0.0%	0.0%	0.0%	0.0%	5.1%
tr|F1S829|F1S829	0	0	4	0	0	0	0	1	0	0	11	0	0	0	0	1	0.0%	0.0%	8.8%	0.0%	0.0%	0.0%	0.0%	4.0%
tr|F1SHW1|F1SHW1	0	0	2	0	0	0	1	0	0	0	15	0	0	0	1	0	0.0%	0.0%	4.1%	0.0%	0.0%	0.0%	2.2%	0.0%
P00172	0	0	6	0	0	0	0	1	0	0	41	0	0	0	0	1	0.0%	0.0%	34.3%	0.0%	0.0%	0.0%	0.0%	9.7%
Reverse_tr|F1RL39|F1RL39	0	0	1	0	0	0	0	0	0	0	1	0	0	0	0	0	0.0%	0.0%	0.8%	0.0%	0.0%	0.0%	0.0%	0.0%
Reverse_tr|K7GN95|K7GN95	0	0	1	0	0	0	0	0	0	0	1	0	0	0	0	0	0.0%	0.0%	2.1%	0.0%	0.0%	0.0%	0.0%	0.0%
tr|F1SGC0|F1SGC0	0	0	1	0	0	0	0	0	0	0	1	0	0	0	0	0	0.0%	0.0%	3.0%	0.0%	0.0%	0.0%	0.0%	0.0%
Q19QU3	0	0	1	0	0	0	0	0	0	0	1	0	0	0	0	0	0.0%	0.0%	3.7%	0.0%	0.0%	0.0%	0.0%	0.0%
tr|F1RWL3|F1RWL3	0	0	1	0	0	0	0	0	0	0	1	0	0	0	0	0	0.0%	0.0%	0.8%	0.0%	0.0%	0.0%	0.0%	0.0%
tr|F1SR48|F1SR48	0	0	1	0	0	0	0	0	0	0	1	0	0	0	0	0	0.0%	0.0%	65.7%	0.0%	0.0%	0.0%	0.0%	0.0%
tr|F1RUA7|F1RUA7	0	0	1	0	0	0	0	0	0	0	1	0	0	0	0	0	0.0%	0.0%	3.8%	0.0%	0.0%	0.0%	0.0%	0.0%
tr|I3LIL8|I3LIL8	0	0	1	0	0	0	0	0	0	0	1	0	0	0	0	0	0.0%	0.0%	9.0%	0.0%	0.0%	0.0%	0.0%	0.0%
tr|F1RXM3|F1RXM3	0	0	1	0	0	0	0	0	0	0	1	0	0	0	0	0	0.0%	0.0%	4.8%	0.0%	0.0%	0.0%	0.0%	0.0%
tr|I3LPV8|I3LPV8	0	0	1	0	0	0	0	0	0	0	1	0	0	0	0	0	0.0%	0.0%	2.8%	0.0%	0.0%	0.0%	0.0%	0.0%
tr|K7GMJ5|K7GMJ5	0	0	1	0	0	0	0	0	0	0	1	0	0	0	0	0	0.0%	0.0%	7.5%	0.0%	0.0%	0.0%	0.0%	0.0%
tr|I3L5M9|I3L5M9	0	0	1	0	0	0	0	0	0	0	1	0	0	0	0	0	0.0%	0.0%	9.4%	0.0%	0.0%	0.0%	0.0%	0.0%
Reverse_tr|F1S7I5|F1S7I5	0	0	1	0	0	0	0	0	0	0	1	0	0	0	0	0	0.0%	0.0%	22.3%	0.0%	0.0%	0.0%	0.0%	0.0%
tr|F1SQG1|F1SQG1	0	0	1	0	0	0	0	0	0	0	1	0	0	0	0	0	0.0%	0.0%	1.0%	0.0%	0.0%	0.0%	0.0%	0.0%
Q28943	0	0	1	0	0	0	0	0	0	0	1	0	0	0	0	0	0.0%	0.0%	2.5%	0.0%	0.0%	0.0%	0.0%	0.0%
Reverse_tr|F1RJU0|F1RJU0	0	0	1	0	0	0	0	0	0	0	1	0	0	0	0	0	0.0%	0.0%	2.1%	0.0%	0.0%	0.0%	0.0%	0.0%
tr|F1SKX8|F1SKX8	0	0	1	0	0	0	0	0	0	0	1	0	0	0	0	0	0.0%	0.0%	3.0%	0.0%	0.0%	0.0%	0.0%	0.0%
tr|I3L9Q4|I3L9Q4	0	0	1	0	0	0	0	0	0	0	1	0	0	0	0	0	0.0%	0.0%	3.3%	0.0%	0.0%	0.0%	0.0%	0.0%
tr|K7GPT4|K7GPT4	0	0	1	0	0	0	0	0	0	0	1	0	0	0	0	0	0.0%	0.0%	6.1%	0.0%	0.0%	0.0%	0.0%	0.0%
tr|F1RHK3|F1RHK3	0	0	1	0	0	0	0	0	0	0	1	0	0	0	0	0	0.0%	0.0%	7.5%	0.0%	0.0%	0.0%	0.0%	0.0%
tr|F1SCL6|F1SCL6	0	0	1	0	0	0	0	0	0	0	1	0	0	0	0	0	0.0%	0.0%	1.5%	0.0%	0.0%	0.0%	0.0%	0.0%
tr|F1RQL3|F1RQL3	0	0	1	0	0	0	0	0	0	0	1	0	0	0	0	0	0.0%	0.0%	7.0%	0.0%	0.0%	0.0%	0.0%	0.0%
tr|F1RHN0|F1RHN0	0	0	1	0	0	0	0	0	0	0	1	0	0	0	0	0	0.0%	0.0%	5.2%	0.0%	0.0%	0.0%	0.0%	0.0%
tr|F1RZQ8|F1RZQ8	0	0	1	0	0	0	0	0	0	0	1	0	0	0	0	0	0.0%	0.0%	6.7%	0.0%	0.0%	0.0%	0.0%	0.0%
tr|I3LTX1|I3LTX1	0	0	1	0	0	0	0	0	0	0	1	0	0	0	0	0	0.0%	0.0%	1.7%	0.0%	0.0%	0.0%	0.0%	0.0%
tr|F1SRW6|F1SRW6	0	0	1	0	0	0	0	0	0	0	1	0	0	0	0	0	0.0%	0.0%	4.4%	0.0%	0.0%	0.0%	0.0%	0.0%
Reverse_tr|Q19QT0|Q19QT0	0	0	1	0	0	0	0	0	0	0	1	0	0	0	0	0	0.0%	0.0%	1.4%	0.0%	0.0%	0.0%	0.0%	0.0%
tr|F1RW22|F1RW22	0	0	1	0	0	0	0	0	0	0	1	0	0	0	0	0	0.0%	0.0%	3.8%	0.0%	0.0%	0.0%	0.0%	0.0%
tr|I3LFW1|I3LFW1	0	0	1	0	0	0	0	0	0	0	1	0	0	0	0	0	0.0%	0.0%	4.4%	0.0%	0.0%	0.0%	0.0%	0.0%

	Peptide Count								Spectral Count								Sequence Coverage							
	Whole Cell	Whole Cell	Whole Cell	Cilia Fraction	Cilia Fraction	Cilia Fraction	Cilia Fraction	Cilia Fraction	Whole Cell	Whole Cell	Whole Cell	Cilia Fraction	Cilia Fraction	Cilia Fraction	Cilia Fraction	Cilia Fraction	Whole Cell	Whole Cell	Whole Cell	Cilia Fraction	Cilia Fraction	Cilia Fraction	Cilia Fraction	Cilia Fraction
Sample#	1	2	6	4	3	5	7	8	1	2	6	4	3	5	7	8	1	2	6	4	3	5	7	8
**Accession**																								
tr|F1SMY9|F1SMY9	0	0	1	0	0	0	0	0	0	0	1	0	0	0	0	0	0.0%	0.0%	3.9%	0.0%	0.0%	0.0%	0.0%	0.0%
tr|I3L5M8|I3L5M8	0	0	1	0	0	0	0	0	0	0	1	0	0	0	0	0	0.0%	0.0%	5.2%	0.0%	0.0%	0.0%	0.0%	0.0%
tr|F1RIB4|F1RIB4	0	0	1	0	0	0	0	0	0	0	1	0	0	0	0	0	0.0%	0.0%	9.7%	0.0%	0.0%	0.0%	0.0%	0.0%
tr|F1RJX9|F1RJX9	0	0	1	0	0	0	0	0	0	0	1	0	0	0	0	0	0.0%	0.0%	4.7%	0.0%	0.0%	0.0%	0.0%	0.0%
tr|F1SSM5|F1SSM5	0	0	1	0	0	0	0	0	0	0	1	0	0	0	0	0	0.0%	0.0%	7.1%	0.0%	0.0%	0.0%	0.0%	0.0%
tr|F1S1D3|F1S1D3	0	0	1	0	0	0	0	0	0	0	1	0	0	0	0	0	0.0%	0.0%	3.7%	0.0%	0.0%	0.0%	0.0%	0.0%
tr|I3LFE2|I3LFE2	0	0	1	0	0	0	0	0	0	0	1	0	0	0	0	0	0.0%	0.0%	10.6%	0.0%	0.0%	0.0%	0.0%	0.0%
tr|K7GLI8|K7GLI8	0	0	1	0	0	0	0	0	0	0	1	0	0	0	0	0	0.0%	0.0%	1.9%	0.0%	0.0%	0.0%	0.0%	0.0%
Reverse_tr|K7GRX6|K7GRX6	0	0	1	0	0	0	0	0	0	0	1	0	0	0	0	0	0.0%	0.0%	2.5%	0.0%	0.0%	0.0%	0.0%	0.0%
tr|I3LM95|I3LM95	0	0	1	0	0	0	0	0	0	0	1	0	0	0	0	0	0.0%	0.0%	4.7%	0.0%	0.0%	0.0%	0.0%	0.0%
tr|F1S577|F1S577	0	0	1	0	0	0	0	0	0	0	1	0	0	0	0	0	0.0%	0.0%	5.3%	0.0%	0.0%	0.0%	0.0%	0.0%
tr|F1RXR2|F1RXR2	0	0	1	0	0	0	0	0	0	0	1	0	0	0	0	0	0.0%	0.0%	10.8%	0.0%	0.0%	0.0%	0.0%	0.0%
tr|I3LUD4|I3LUD4	0	0	1	0	0	0	0	0	0	0	1	0	0	0	0	0	0.0%	0.0%	3.4%	0.0%	0.0%	0.0%	0.0%	0.0%
Reverse_tr|F1RMP2|F1RMP2	0	0	1	0	0	0	0	0	0	0	1	0	0	0	0	0	0.0%	0.0%	0.4%	0.0%	0.0%	0.0%	0.0%	0.0%
tr|I3LCV9|I3LCV9	0	0	1	0	0	0	0	0	0	0	1	0	0	0	0	0	0.0%	0.0%	1.4%	0.0%	0.0%	0.0%	0.0%	0.0%
tr|F1SUA4|F1SUA4	0	0	1	0	0	0	0	0	0	0	1	0	0	0	0	0	0.0%	0.0%	15.7%	0.0%	0.0%	0.0%	0.0%	0.0%
tr|I3LTH5|I3LTH5	0	0	1	0	0	0	0	0	0	0	1	0	0	0	0	0	0.0%	0.0%	1.1%	0.0%	0.0%	0.0%	0.0%	0.0%
Reverse_tr|F1SPX1|F1SPX1	0	0	1	0	0	0	0	0	0	0	1	0	0	0	0	0	0.0%	0.0%	1.0%	0.0%	0.0%	0.0%	0.0%	0.0%
tr|I3LA87|I3LA87	0	0	1	0	0	0	0	0	0	0	1	0	0	0	0	0	0.0%	0.0%	7.0%	0.0%	0.0%	0.0%	0.0%	0.0%
Reverse_tr|F1SD92|F1SD92	0	0	1	0	0	0	0	0	0	0	1	0	0	0	0	0	0.0%	0.0%	1.7%	0.0%	0.0%	0.0%	0.0%	0.0%
tr|I3L692|I3L692	0	0	1	0	0	0	0	0	0	0	1	0	0	0	0	0	0.0%	0.0%	8.8%	0.0%	0.0%	0.0%	0.0%	0.0%
tr|F1SD95|F1SD95	0	0	1	0	0	0	0	0	0	0	1	0	0	0	0	0	0.0%	0.0%	5.0%	0.0%	0.0%	0.0%	0.0%	0.0%
tr|I3L6A4|I3L6A4	0	0	1	0	0	0	0	0	0	0	1	0	0	0	0	0	0.0%	0.0%	14.5%	0.0%	0.0%	0.0%	0.0%	0.0%
tr|K7GQX7|K7GQX7	0	0	1	0	0	0	0	0	0	0	1	0	0	0	0	0	0.0%	0.0%	9.5%	0.0%	0.0%	0.0%	0.0%	0.0%
tr|F1RRQ0|F1RRQ0	0	0	1	0	0	0	0	0	0	0	1	0	0	0	0	0	0.0%	0.0%	4.1%	0.0%	0.0%	0.0%	0.0%	0.0%
tr|F1SPE8|F1SPE8	0	0	1	0	0	0	0	0	0	0	1	0	0	0	0	0	0.0%	0.0%	3.7%	0.0%	0.0%	0.0%	0.0%	0.0%
Reverse_tr|F1S9Y8|F1S9Y8	0	0	1	0	0	0	0	0	0	0	1	0	0	0	0	0	0.0%	0.0%	0.4%	0.0%	0.0%	0.0%	0.0%	0.0%
Q8MIR4	0	0	1	0	0	0	0	0	0	0	1	0	0	0	0	0	0.0%	0.0%	3.2%	0.0%	0.0%	0.0%	0.0%	0.0%
tr|F1SHS4|F1SHS4	0	0	1	0	0	0	0	0	0	0	1	0	0	0	0	0	0.0%	0.0%	2.0%	0.0%	0.0%	0.0%	0.0%	0.0%
tr|I3LR64|I3LR64	0	0	1	0	0	0	0	0	0	0	1	0	0	0	0	0	0.0%	0.0%	7.1%	0.0%	0.0%	0.0%	0.0%	0.0%
tr|I3LNY4|I3LNY4	0	0	1	0	0	0	0	0	0	0	1	0	0	0	0	0	0.0%	0.0%	5.1%	0.0%	0.0%	0.0%	0.0%	0.0%
tr|F1RVR8|F1RVR8	0	0	1	0	0	0	0	0	0	0	1	0	0	0	0	0	0.0%	0.0%	2.4%	0.0%	0.0%	0.0%	0.0%	0.0%
tr|F1S592|F1S592	0	0	1	0	0	0	0	0	0	0	1	0	0	0	0	0	0.0%	0.0%	7.5%	0.0%	0.0%	0.0%	0.0%	0.0%
Reverse_tr|F1SN99|F1SN99	0	0	1	0	0	0	0	0	0	0	1	0	0	0	0	0	0.0%	0.0%	0.8%	0.0%	0.0%	0.0%	0.0%	0.0%
Q8SPR7	0	0	1	0	0	0	0	0	0	0	1	0	0	0	0	0	0.0%	0.0%	1.4%	0.0%	0.0%	0.0%	0.0%	0.0%
tr|F1RX33|F1RX33	0	0	1	0	0	0	0	0	0	0	1	0	0	0	0	0	0.0%	0.0%	3.9%	0.0%	0.0%	0.0%	0.0%	0.0%
tr|F1RMW0|F1RMW0	0	0	1	0	0	0	0	0	0	0	1	0	0	0	0	0	0.0%	0.0%	8.9%	0.0%	0.0%	0.0%	0.0%	0.0%
tr|F1SA25|F1SA25	0	0	1	0	0	0	0	0	0	0	1	0	0	0	0	0	0.0%	0.0%	10.8%	0.0%	0.0%	0.0%	0.0%	0.0%
tr|F1S4Y0|F1S4Y0	0	0	1	0	0	0	0	0	0	0	1	0	0	0	0	0	0.0%	0.0%	3.9%	0.0%	0.0%	0.0%	0.0%	0.0%
tr|F1SBR3|F1SBR3	0	0	1	0	0	0	0	0	0	0	1	0	0	0	0	0	0.0%	0.0%	6.5%	0.0%	0.0%	0.0%	0.0%	0.0%
tr|F1SNZ3|F1SNZ3	0	0	1	0	0	0	0	0	0	0	1	0	0	0	0	0	0.0%	0.0%	2.7%	0.0%	0.0%	0.0%	0.0%	0.0%
tr|I3LKJ3|I3LKJ3	0	0	1	0	0	0	0	0	0	0	1	0	0	0	0	0	0.0%	0.0%	8.9%	0.0%	0.0%	0.0%	0.0%	0.0%
tr|I3LT16|I3LT16	0	0	1	0	0	0	0	0	0	0	1	0	0	0	0	0	0.0%	0.0%	19.5%	0.0%	0.0%	0.0%	0.0%	0.0%
tr|F1S2Z2|F1S2Z2	0	0	1	0	0	0	0	0	0	0	1	0	0	0	0	0	0.0%	0.0%	1.7%	0.0%	0.0%	0.0%	0.0%	0.0%
tr|I3LHD4|I3LHD4	0	0	1	0	0	0	0	0	0	0	1	0	0	0	0	0	0.0%	0.0%	2.7%	0.0%	0.0%	0.0%	0.0%	0.0%
tr|F1REZ9|F1REZ9	0	0	1	0	0	0	0	0	0	0	1	0	0	0	0	0	0.0%	0.0%	11.0%	0.0%	0.0%	0.0%	0.0%	0.0%
Q29243	0	0	1	0	0	0	0	0	0	0	1	0	0	0	0	0	0.0%	0.0%	14.1%	0.0%	0.0%	0.0%	0.0%	0.0%
tr|I3L944|I3L944	0	0	1	0	0	0	0	0	0	0	1	0	0	0	0	0	0.0%	0.0%	2.9%	0.0%	0.0%	0.0%	0.0%	0.0%
tr|F1RZQ3|F1RZQ3	0	0	1	0	0	0	0	0	0	0	1	0	0	0	0	0	0.0%	0.0%	3.6%	0.0%	0.0%	0.0%	0.0%	0.0%
tr|F1SRB7|F1SRB7	0	0	1	0	0	0	0	0	0	0	1	0	0	0	0	0	0.0%	0.0%	7.3%	0.0%	0.0%	0.0%	0.0%	0.0%
Reverse_tr|K7GLU0|K7GLU0	0	0	1	0	0	0	0	0	0	0	1	0	0	0	0	0	0.0%	0.0%	2.5%	0.0%	0.0%	0.0%	0.0%	0.0%
Reverse_tr|F1SQQ8|F1SQQ8	0	0	1	0	0	0	0	0	0	0	1	0	0	0	0	0	0.0%	0.0%	2.8%	0.0%	0.0%	0.0%	0.0%	0.0%
tr|F1S642|F1S642	0	0	1	0	0	0	0	0	0	0	1	0	0	0	0	0	0.0%	0.0%	1.4%	0.0%	0.0%	0.0%	0.0%	0.0%
tr|I3LQQ8|I3LQQ8	0	0	1	0	0	0	0	0	0	0	1	0	0	0	0	0	0.0%	0.0%	1.7%	0.0%	0.0%	0.0%	0.0%	0.0%
Reverse_tr|F1RM80|F1RM80	0	0	1	0	0	0	0	0	0	0	1	0	0	0	0	0	0.0%	0.0%	15.2%	0.0%	0.0%	0.0%	0.0%	0.0%
tr|F1SHN7|F1SHN7	0	0	1	0	0	0	0	0	0	0	1	0	0	0	0	0	0.0%	0.0%	0.6%	0.0%	0.0%	0.0%	0.0%	0.0%
tr|I3LGT6|I3LGT6	0	0	1	0	0	0	0	0	0	0	1	0	0	0	0	0	0.0%	0.0%	4.5%	0.0%	0.0%	0.0%	0.0%	0.0%
Reverse_tr|I3LDZ4|I3LDZ4	0	0	1	0	0	0	0	0	0	0	1	0	0	0	0	0	0.0%	0.0%	3.6%	0.0%	0.0%	0.0%	0.0%	0.0%
tr|F1S8G6|F1S8G6	0	0	1	0	0	0	0	0	0	0	1	0	0	0	0	0	0.0%	0.0%	4.2%	0.0%	0.0%	0.0%	0.0%	0.0%
tr|I3LIF4|I3LIF4	0	0	1	0	0	0	0	0	0	0	1	0	0	0	0	0	0.0%	0.0%	8.5%	0.0%	0.0%	0.0%	0.0%	0.0%
tr|F1RZ77|F1RZ77	0	0	1	0	0	0	0	0	0	0	1	0	0	0	0	0	0.0%	0.0%	6.1%	0.0%	0.0%	0.0%	0.0%	0.0%
tr|F1SUH7|F1SUH7	0	0	1	0	0	0	0	0	0	0	1	0	0	0	0	0	0.0%	0.0%	4.6%	0.0%	0.0%	0.0%	0.0%	0.0%
tr|F1SUA1|F1SUA1	0	0	1	0	0	0	0	0	0	0	1	0	0	0	0	0	0.0%	0.0%	5.5%	0.0%	0.0%	0.0%	0.0%	0.0%
tr|I3LUS9|I3LUS9	0	0	1	0	0	0	0	0	0	0	1	0	0	0	0	0	0.0%	0.0%	0.6%	0.0%	0.0%	0.0%	0.0%	0.0%
tr|F1SH66|F1SH66	0	0	1	0	0	0	0	0	0	0	1	0	0	0	0	0	0.0%	0.0%	7.3%	0.0%	0.0%	0.0%	0.0%	0.0%
tr|F1S4R6|F1S4R6	0	0	1	0	0	0	0	0	0	0	1	0	0	0	0	0	0.0%	0.0%	2.0%	0.0%	0.0%	0.0%	0.0%	0.0%
Reverse_tr|F1S2U8|F1S2U8	0	0	1	0	0	0	0	0	0	0	1	0	0	0	0	0	0.0%	0.0%	9.8%	0.0%	0.0%	0.0%	0.0%	0.0%
Reverse_tr|I3LUR7|I3LUR7	0	0	1	0	0	0	0	0	0	0	1	0	0	0	0	0	0.0%	0.0%	0.7%	0.0%	0.0%	0.0%	0.0%	0.0%
tr|I3LUC6|I3LUC6	0	0	1	0	0	0	0	0	0	0	1	0	0	0	0	0	0.0%	0.0%	14.6%	0.0%	0.0%	0.0%	0.0%	0.0%
tr|C3UP60|C3UP60	0	0	1	0	0	0	0	0	0	0	1	0	0	0	0	0	0.0%	0.0%	6.2%	0.0%	0.0%	0.0%	0.0%	0.0%
Reverse_tr|F1RVP4|F1RVP4	0	0	1	0	0	0	0	0	0	0	1	0	0	0	0	0	0.0%	0.0%	2.5%	0.0%	0.0%	0.0%	0.0%	0.0%
tr|I3L7P4|I3L7P4	0	0	1	0	0	0	0	0	0	0	1	0	0	0	0	0	0.0%	0.0%	6.1%	0.0%	0.0%	0.0%	0.0%	0.0%
tr|I3LNX4|I3LNX4	0	0	1	0	0	0	0	0	0	0	1	0	0	0	0	0	0.0%	0.0%	2.9%	0.0%	0.0%	0.0%	0.0%	0.0%
tr|F1SFL6|F1SFL6	0	0	1	0	0	0	0	0	0	0	1	0	0	0	0	0	0.0%	0.0%	5.9%	0.0%	0.0%	0.0%	0.0%	0.0%
Reverse_tr|F1SDV6|F1SDV6	0	0	1	0	0	0	0	0	0	0	1	0	0	0	0	0	0.0%	0.0%	0.9%	0.0%	0.0%	0.0%	0.0%	0.0%
tr|F1SBW6|F1SBW6	0	0	1	0	0	0	0	0	0	0	1	0	0	0	0	0	0.0%	0.0%	1.9%	0.0%	0.0%	0.0%	0.0%	0.0%
tr|F1S550|F1S550	0	0	1	0	0	0	0	0	0	0	1	0	0	0	0	0	0.0%	0.0%	2.7%	0.0%	0.0%	0.0%	0.0%	0.0%
tr|F1RL82|F1RL82	0	0	1	0	0	0	0	0	0	0	1	0	0	0	0	0	0.0%	0.0%	2.8%	0.0%	0.0%	0.0%	0.0%	0.0%
tr|I3LVP5|I3LVP5	0	0	1	0	0	0	0	0	0	0	1	0	0	0	0	0	0.0%	0.0%	3.6%	0.0%	0.0%	0.0%	0.0%	0.0%
tr|I3LD26|I3LD26	0	0	1	0	0	0	0	0	0	0	1	0	0	0	0	0	0.0%	0.0%	4.8%	0.0%	0.0%	0.0%	0.0%	0.0%
Reverse_tr|I3LQS3|I3LQS3	0	0	1	0	0	0	0	0	0	0	1	0	0	0	0	0	0.0%	0.0%	3.3%	0.0%	0.0%	0.0%	0.0%	0.0%

	Peptide Count								Spectral Count								Sequence Coverage							
	Whole Cell	Whole Cell	Whole Cell	Cilia Fraction	Cilia Fraction	Cilia Fraction	Cilia Fraction	Cilia Fraction	Whole Cell	Whole Cell	Whole Cell	Cilia Fraction	Cilia Fraction	Cilia Fraction	Cilia Fraction	Cilia Fraction	Whole Cell	Whole Cell	Whole Cell	Cilia Fraction	Cilia Fraction	Cilia Fraction	Cilia Fraction	Cilia Fraction
Sample#	1	2	6	4	3	5	7	8	1	2	6	4	3	5	7	8	1	2	6	4	3	5	7	8
**Accession**																								
tr|F1RRC2|F1RRC2	0	0	1	0	0	0	0	0	0	0	1	0	0	0	0	0	0.0%	0.0%	0.6%	0.0%	0.0%	0.0%	0.0%	0.0%
tr|F1S8W0|F1S8W0	0	0	1	0	0	0	0	0	0	0	1	0	0	0	0	0	0.0%	0.0%	8.4%	0.0%	0.0%	0.0%	0.0%	0.0%
tr|F1SQC6|F1SQC6	0	0	1	0	0	0	0	0	0	0	1	0	0	0	0	0	0.0%	0.0%	6.9%	0.0%	0.0%	0.0%	0.0%	0.0%
tr|I3LVL7|I3LVL7	0	0	1	0	0	0	0	0	0	0	1	0	0	0	0	0	0.0%	0.0%	5.9%	0.0%	0.0%	0.0%	0.0%	0.0%
tr|I3L7V8|I3L7V8	0	0	1	0	0	0	0	0	0	0	1	0	0	0	0	0	0.0%	0.0%	19.4%	0.0%	0.0%	0.0%	0.0%	0.0%
tr|F1S7I3|F1S7I3	0	0	1	0	0	0	0	0	0	0	1	0	0	0	0	0	0.0%	0.0%	14.0%	0.0%	0.0%	0.0%	0.0%	0.0%
Reverse_tr|I3LGJ5|I3LGJ5	0	0	1	0	0	0	0	0	0	0	1	0	0	0	0	0	0.0%	0.0%	0.6%	0.0%	0.0%	0.0%	0.0%	0.0%
Reverse_tr|I3LDF6|I3LDF6	0	0	1	0	0	0	0	0	0	0	1	0	0	0	0	0	0.0%	0.0%	1.7%	0.0%	0.0%	0.0%	0.0%	0.0%
tr|F1S4I0|F1S4I0	0	0	1	0	0	0	0	0	0	0	1	0	0	0	0	0	0.0%	0.0%	1.9%	0.0%	0.0%	0.0%	0.0%	0.0%
tr|F1RJT5|F1RJT5	0	0	1	0	0	0	0	0	0	0	1	0	0	0	0	0	0.0%	0.0%	10.0%	0.0%	0.0%	0.0%	0.0%	0.0%
tr|F1SSG3|F1SSG3	0	0	1	0	0	0	0	0	0	0	1	0	0	0	0	0	0.0%	0.0%	3.4%	0.0%	0.0%	0.0%	0.0%	0.0%
tr|F1RZS1|F1RZS1	0	0	1	0	0	0	0	0	0	0	1	0	0	0	0	0	0.0%	0.0%	3.8%	0.0%	0.0%	0.0%	0.0%	0.0%
A0P8Z5	0	0	1	0	0	0	0	0	0	0	1	0	0	0	0	0	0.0%	0.0%	2.8%	0.0%	0.0%	0.0%	0.0%	0.0%
tr|F1RW76|F1RW76	0	0	1	0	0	0	0	0	0	0	1	0	0	0	0	0	0.0%	0.0%	2.3%	0.0%	0.0%	0.0%	0.0%	0.0%
tr|I3LJJ3|I3LJJ3	0	0	1	0	0	0	0	0	0	0	1	0	0	0	0	0	0.0%	0.0%	11.8%	0.0%	0.0%	0.0%	0.0%	0.0%
P80272	0	0	1	0	0	0	0	0	0	0	1	0	0	0	0	0	0.0%	0.0%	11.1%	0.0%	0.0%	0.0%	0.0%	0.0%
tr|I3LRV1|I3LRV1	0	0	1	0	0	0	0	0	0	0	1	0	0	0	0	0	0.0%	0.0%	3.3%	0.0%	0.0%	0.0%	0.0%	0.0%
tr|I3LJ57|I3LJ57	0	0	1	0	0	0	0	0	0	0	1	0	0	0	0	0	0.0%	0.0%	11.2%	0.0%	0.0%	0.0%	0.0%	0.0%
tr|D2D0E5|D2D0E5	0	0	1	0	0	0	0	0	0	0	1	0	0	0	0	0	0.0%	0.0%	6.8%	0.0%	0.0%	0.0%	0.0%	0.0%
tr|F1S4T2|F1S4T2	0	0	1	0	0	0	0	0	0	0	1	0	0	0	0	0	0.0%	0.0%	19.5%	0.0%	0.0%	0.0%	0.0%	0.0%
Reverse_tr|I3LJQ7|I3LJQ7	0	0	1	0	0	0	0	0	0	0	1	0	0	0	0	0	0.0%	0.0%	3.1%	0.0%	0.0%	0.0%	0.0%	0.0%
tr|F1S1X8|F1S1X8	0	0	1	0	0	0	0	0	0	0	1	0	0	0	0	0	0.0%	0.0%	2.1%	0.0%	0.0%	0.0%	0.0%	0.0%
tr|F1RSR2|F1RSR2	0	0	1	0	0	0	0	0	0	0	1	0	0	0	0	0	0.0%	0.0%	5.9%	0.0%	0.0%	0.0%	0.0%	0.0%
tr|F1S1E1|F1S1E1	0	0	1	0	0	0	0	0	0	0	1	0	0	0	0	0	0.0%	0.0%	2.9%	0.0%	0.0%	0.0%	0.0%	0.0%
tr|F1ST80|F1ST80	0	0	1	0	0	0	0	0	0	0	1	0	0	0	0	0	0.0%	0.0%	3.6%	0.0%	0.0%	0.0%	0.0%	0.0%
tr|F1SL15|F1SL15	0	0	1	0	0	0	0	0	0	0	1	0	0	0	0	0	0.0%	0.0%	5.8%	0.0%	0.0%	0.0%	0.0%	0.0%
tr|I3LLT6|I3LLT6	0	0	1	0	0	0	0	0	0	0	2	0	0	0	0	0	0.0%	0.0%	2.3%	0.0%	0.0%	0.0%	0.0%	0.0%
tr|I3LSU9|I3LSU9	0	0	1	0	0	0	0	0	0	0	2	0	0	0	0	0	0.0%	0.0%	7.0%	0.0%	0.0%	0.0%	0.0%	0.0%
tr|F1RLV6|F1RLV6	0	0	1	0	0	0	0	0	0	0	2	0	0	0	0	0	0.0%	0.0%	4.0%	0.0%	0.0%	0.0%	0.0%	0.0%
tr|F1SA97|F1SA97	0	0	1	0	0	0	0	0	0	0	2	0	0	0	0	0	0.0%	0.0%	6.3%	0.0%	0.0%	0.0%	0.0%	0.0%
tr|F1S1B7|F1S1B7	0	0	1	0	0	0	0	0	0	0	2	0	0	0	0	0	0.0%	0.0%	2.7%	0.0%	0.0%	0.0%	0.0%	0.0%
tr|I3LD78|I3LD78	0	0	1	0	0	0	0	0	0	0	2	0	0	0	0	0	0.0%	0.0%	1.9%	0.0%	0.0%	0.0%	0.0%	0.0%
Q03472	0	0	1	0	0	0	0	0	0	0	2	0	0	0	0	0	0.0%	0.0%	6.4%	0.0%	0.0%	0.0%	0.0%	0.0%
tr|I3LHJ6|I3LHJ6	0	0	1	0	0	0	0	0	0	0	2	0	0	0	0	0	0.0%	0.0%	3.4%	0.0%	0.0%	0.0%	0.0%	0.0%
tr|G8ENM4|G8ENM4	0	0	1	0	0	0	0	0	0	0	2	0	0	0	0	0	0.0%	0.0%	3.7%	0.0%	0.0%	0.0%	0.0%	0.0%
tr|I3LU34|I3LU34	0	0	1	0	0	0	0	0	0	0	2	0	0	0	0	0	0.0%	0.0%	13.0%	0.0%	0.0%	0.0%	0.0%	0.0%
tr|F1RQ10|F1RQ10	0	0	1	0	0	0	0	0	0	0	2	0	0	0	0	0	0.0%	0.0%	4.6%	0.0%	0.0%	0.0%	0.0%	0.0%
tr|I3LNQ4|I3LNQ4	0	0	1	0	0	0	0	0	0	0	2	0	0	0	0	0	0.0%	0.0%	4.2%	0.0%	0.0%	0.0%	0.0%	0.0%
Reverse_tr|F1RJ15|F1RJ15	0	0	1	0	0	0	0	0	0	0	2	0	0	0	0	0	0.0%	0.0%	1.6%	0.0%	0.0%	0.0%	0.0%	0.0%
tr|I3LND2|I3LND2	0	0	1	0	0	0	0	0	0	0	2	0	0	0	0	0	0.0%	0.0%	4.1%	0.0%	0.0%	0.0%	0.0%	0.0%
tr|K7GPM5|K7GPM5	0	0	2	0	0	0	0	0	0	0	2	0	0	0	0	0	0.0%	0.0%	13.4%	0.0%	0.0%	0.0%	0.0%	0.0%
tr|F1RH84|F1RH84	0	0	1	0	0	0	0	0	0	0	2	0	0	0	0	0	0.0%	0.0%	22.0%	0.0%	0.0%	0.0%	0.0%	0.0%
tr|K7GP21|K7GP21	0	0	2	0	0	0	0	0	0	0	2	0	0	0	0	0	0.0%	0.0%	6.5%	0.0%	0.0%	0.0%	0.0%	0.0%
tr|F1RPM0|F1RPM0	0	0	1	0	0	0	0	0	0	0	2	0	0	0	0	0	0.0%	0.0%	2.7%	0.0%	0.0%	0.0%	0.0%	0.0%
tr|F1SH78|F1SH78	0	0	1	0	0	0	0	0	0	0	2	0	0	0	0	0	0.0%	0.0%	3.3%	0.0%	0.0%	0.0%	0.0%	0.0%
tr|K7GKC8|K7GKC8	0	0	1	0	0	0	0	0	0	0	2	0	0	0	0	0	0.0%	0.0%	3.8%	0.0%	0.0%	0.0%	0.0%	0.0%
tr|F1SUE0|F1SUE0	0	0	1	0	0	0	0	0	0	0	2	0	0	0	0	0	0.0%	0.0%	1.8%	0.0%	0.0%	0.0%	0.0%	0.0%
tr|F1SA60|F1SA60	0	0	1	0	0	0	0	0	0	0	2	0	0	0	0	0	0.0%	0.0%	0.4%	0.0%	0.0%	0.0%	0.0%	0.0%
Reverse_tr|F1SSB4|F1SSB4	0	0	1	0	0	0	0	0	0	0	2	0	0	0	0	0	0.0%	0.0%	10.4%	0.0%	0.0%	0.0%	0.0%	0.0%
tr|A5GHL0|A5GHL0	0	0	1	0	0	0	0	0	0	0	2	0	0	0	0	0	0.0%	0.0%	10.8%	0.0%	0.0%	0.0%	0.0%	0.0%
tr|I3L8R9|I3L8R9	0	0	1	0	0	0	0	0	0	0	2	0	0	0	0	0	0.0%	0.0%	2.8%	0.0%	0.0%	0.0%	0.0%	0.0%
tr|F1RIZ5|F1RIZ5	0	0	1	0	0	0	0	0	0	0	2	0	0	0	0	0	0.0%	0.0%	4.2%	0.0%	0.0%	0.0%	0.0%	0.0%
tr|Q0GFF5|Q0GFF5	0	0	2	0	0	0	0	0	0	0	2	0	0	0	0	0	0.0%	0.0%	4.5%	0.0%	0.0%	0.0%	0.0%	0.0%
tr|F1RR85|F1RR85	0	0	1	0	0	0	0	0	0	0	2	0	0	0	0	0	0.0%	0.0%	3.4%	0.0%	0.0%	0.0%	0.0%	0.0%
tr|F1SC53|F1SC53	0	0	1	0	0	0	0	0	0	0	2	0	0	0	0	0	0.0%	0.0%	5.8%	0.0%	0.0%	0.0%	0.0%	0.0%
tr|I3LTY6|I3LTY6	0	0	1	0	0	0	0	0	0	0	2	0	0	0	0	0	0.0%	0.0%	4.2%	0.0%	0.0%	0.0%	0.0%	0.0%
tr|F1S9V5|F1S9V5	0	0	1	0	0	0	0	0	0	0	2	0	0	0	0	0	0.0%	0.0%	5.4%	0.0%	0.0%	0.0%	0.0%	0.0%
tr|I3LA16|I3LA16	0	0	1	0	0	0	0	0	0	0	2	0	0	0	0	0	0.0%	0.0%	4.7%	0.0%	0.0%	0.0%	0.0%	0.0%
tr|J9JIL6|J9JIL6	0	0	2	0	0	0	0	0	0	0	2	0	0	0	0	0	0.0%	0.0%	19.7%	0.0%	0.0%	0.0%	0.0%	0.0%
tr|I3LNU3|I3LNU3	0	0	1	0	0	0	0	0	0	0	2	0	0	0	0	0	0.0%	0.0%	2.4%	0.0%	0.0%	0.0%	0.0%	0.0%
P0C2C3	0	0	1	0	0	0	0	0	0	0	2	0	0	0	0	0	0.0%	0.0%	40.0%	0.0%	0.0%	0.0%	0.0%	0.0%
tr|F1RSJ2|F1RSJ2	0	0	1	0	0	0	0	0	0	0	2	0	0	0	0	0	0.0%	0.0%	2.4%	0.0%	0.0%	0.0%	0.0%	0.0%
Reverse_tr|K7GKC1|K7GKC1	0	0	1	0	0	0	0	0	0	0	2	0	0	0	0	0	0.0%	0.0%	2.0%	0.0%	0.0%	0.0%	0.0%	0.0%
Q29076	0	0	1	0	0	0	0	0	0	0	2	0	0	0	0	0	0.0%	0.0%	2.8%	0.0%	0.0%	0.0%	0.0%	0.0%
tr|F1S7M1|F1S7M1	0	0	1	0	0	0	0	0	0	0	2	0	0	0	0	0	0.0%	0.0%	4.0%	0.0%	0.0%	0.0%	0.0%	0.0%
tr|F2Z5L8|F2Z5L8	0	0	2	0	0	0	0	0	0	0	2	0	0	0	0	0	0.0%	0.0%	6.5%	0.0%	0.0%	0.0%	0.0%	0.0%
tr|D7RA31|D7RA31	0	0	1	0	0	0	0	0	0	0	2	0	0	0	0	0	0.0%	0.0%	4.8%	0.0%	0.0%	0.0%	0.0%	0.0%
tr|F1S265|F1S265	0	0	1	0	0	0	0	0	0	0	2	0	0	0	0	0	0.0%	0.0%	2.9%	0.0%	0.0%	0.0%	0.0%	0.0%
Q8HZJ6	0	0	2	0	0	0	0	0	0	0	2	0	0	0	0	0	0.0%	0.0%	14.9%	0.0%	0.0%	0.0%	0.0%	0.0%
tr|F1SF84|F1SF84	0	0	1	0	0	0	0	0	0	0	2	0	0	0	0	0	0.0%	0.0%	5.6%	0.0%	0.0%	0.0%	0.0%	0.0%
Q06AK6	0	0	1	0	0	0	0	0	0	0	2	0	0	0	0	0	0.0%	0.0%	2.8%	0.0%	0.0%	0.0%	0.0%	0.0%
tr|K7GNA4|K7GNA4	0	0	1	0	0	0	0	0	0	0	2	0	0	0	0	0	0.0%	0.0%	1.7%	0.0%	0.0%	0.0%	0.0%	0.0%
Reverse_tr|F1RQW6|F1RQW6	0	0	1	0	0	0	0	0	0	0	2	0	0	0	0	0	0.0%	0.0%	2.1%	0.0%	0.0%	0.0%	0.0%	0.0%
tr|I3LGP7|I3LGP7	0	0	1	0	0	0	0	0	0	0	2	0	0	0	0	0	0.0%	0.0%	2.3%	0.0%	0.0%	0.0%	0.0%	0.0%
tr|F1S9C1|F1S9C1	0	0	1	0	0	0	0	0	0	0	2	0	0	0	0	0	0.0%	0.0%	1.7%	0.0%	0.0%	0.0%	0.0%	0.0%
tr|K7GQM0|K7GQM0	0	0	2	0	0	0	0	0	0	0	2	0	0	0	0	0	0.0%	0.0%	5.7%	0.0%	0.0%	0.0%	0.0%	0.0%
tr|I3L5H4|I3L5H4	0	0	1	0	0	0	0	0	0	0	2	0	0	0	0	0	0.0%	0.0%	7.5%	0.0%	0.0%	0.0%	0.0%	0.0%
tr|I3LF49|I3LF49	0	0	1	0	0	0	0	0	0	0	2	0	0	0	0	0	0.0%	0.0%	3.0%	0.0%	0.0%	0.0%	0.0%	0.0%
tr|I3LJ14|I3LJ14	0	0	1	0	0	0	0	0	0	0	2	0	0	0	0	0	0.0%	0.0%	25.4%	0.0%	0.0%	0.0%	0.0%	0.0%
tr|I3LNQ1|I3LNQ1	0	0	1	0	0	0	0	0	0	0	2	0	0	0	0	0	0.0%	0.0%	11.4%	0.0%	0.0%	0.0%	0.0%	0.0%
tr|I3LR86|I3LR86	0	0	1	0	0	0	0	0	0	0	2	0	0	0	0	0	0.0%	0.0%	3.0%	0.0%	0.0%	0.0%	0.0%	0.0%

	Peptide Count								Spectral Count								Sequence Coverage							
	Whole Cell	Whole Cell	Whole Cell	Cilia Fraction	Cilia Fraction	Cilia Fraction	Cilia Fraction	Cilia Fraction	Whole Cell	Whole Cell	Whole Cell	Cilia Fraction	Cilia Fraction	Cilia Fraction	Cilia Fraction	Cilia Fraction	Whole Cell	Whole Cell	Whole Cell	Cilia Fraction	Cilia Fraction	Cilia Fraction	Cilia Fraction	Cilia Fraction
Sample#	1	2	6	4	3	5	7	8	1	2	6	4	3	5	7	8	1	2	6	4	3	5	7	8
**Accession**																								
tr|F1SDE8|F1SDE8	0	0	1	0	0	0	0	0	0	0	2	0	0	0	0	0	0.0%	0.0%	3.1%	0.0%	0.0%	0.0%	0.0%	0.0%
tr|F1RVZ0|F1RVZ0	0	0	1	0	0	0	0	0	0	0	2	0	0	0	0	0	0.0%	0.0%	1.7%	0.0%	0.0%	0.0%	0.0%	0.0%
tr|F1RSJ3|F1RSJ3	0	0	1	0	0	0	0	0	0	0	2	0	0	0	0	0	0.0%	0.0%	2.4%	0.0%	0.0%	0.0%	0.0%	0.0%
tr|I3LRE7|I3LRE7	0	0	1	0	0	0	0	0	0	0	2	0	0	0	0	0	0.0%	0.0%	3.1%	0.0%	0.0%	0.0%	0.0%	0.0%
tr|F1STI4|F1STI4	0	0	1	0	0	0	0	0	0	0	2	0	0	0	0	0	0.0%	0.0%	3.9%	0.0%	0.0%	0.0%	0.0%	0.0%
tr|I3LCJ7|I3LCJ7	0	0	1	0	0	0	0	0	0	0	2	0	0	0	0	0	0.0%	0.0%	6.7%	0.0%	0.0%	0.0%	0.0%	0.0%
tr|K7GQJ8|K7GQJ8	0	0	1	0	0	0	0	0	0	0	2	0	0	0	0	0	0.0%	0.0%	5.1%	0.0%	0.0%	0.0%	0.0%	0.0%
tr|F1SK49|F1SK49	0	0	1	0	0	0	0	0	0	0	2	0	0	0	0	0	0.0%	0.0%	10.2%	0.0%	0.0%	0.0%	0.0%	0.0%
tr|I3LQS3|I3LQS3	0	0	1	0	0	0	0	0	0	0	2	0	0	0	0	0	0.0%	0.0%	3.0%	0.0%	0.0%	0.0%	0.0%	0.0%
tr|F1RG71|F1RG71	0	0	1	0	0	0	0	0	0	0	2	0	0	0	0	0	0.0%	0.0%	2.8%	0.0%	0.0%	0.0%	0.0%	0.0%
tr|I3LCX6|I3LCX6	0	0	1	0	0	0	0	0	0	0	2	0	0	0	0	0	0.0%	0.0%	19.1%	0.0%	0.0%	0.0%	0.0%	0.0%
tr|F1RU55|F1RU55	0	0	1	0	0	0	0	0	0	0	2	0	0	0	0	0	0.0%	0.0%	2.7%	0.0%	0.0%	0.0%	0.0%	0.0%
tr|F1SIY7|F1SIY7	0	0	1	0	0	0	0	0	0	0	2	0	0	0	0	0	0.0%	0.0%	2.4%	0.0%	0.0%	0.0%	0.0%	0.0%
tr|I3LBS8|I3LBS8	0	0	1	0	0	0	0	0	0	0	2	0	0	0	0	0	0.0%	0.0%	1.7%	0.0%	0.0%	0.0%	0.0%	0.0%
tr|F1S0D8|F1S0D8	0	0	1	0	0	0	0	0	0	0	2	0	0	0	0	0	0.0%	0.0%	4.4%	0.0%	0.0%	0.0%	0.0%	0.0%
tr|F1RN72|F1RN72	0	0	1	0	0	0	0	0	0	0	2	0	0	0	0	0	0.0%	0.0%	3.2%	0.0%	0.0%	0.0%	0.0%	0.0%
tr|F1SGJ6|F1SGJ6	0	0	1	0	0	0	0	0	0	0	2	0	0	0	0	0	0.0%	0.0%	5.9%	0.0%	0.0%	0.0%	0.0%	0.0%
tr|K7GSY0|K7GSY0	0	0	1	0	0	0	0	0	0	0	2	0	0	0	0	0	0.0%	0.0%	25.0%	0.0%	0.0%	0.0%	0.0%	0.0%
tr|I3LVF3|I3LVF3	0	0	1	0	0	0	0	0	0	0	2	0	0	0	0	0	0.0%	0.0%	3.0%	0.0%	0.0%	0.0%	0.0%	0.0%
tr|I3LBT0|I3LBT0	0	0	1	0	0	0	0	0	0	0	2	0	0	0	0	0	0.0%	0.0%	1.9%	0.0%	0.0%	0.0%	0.0%	0.0%
tr|F1SCK5|F1SCK5	0	0	1	0	0	0	0	0	0	0	3	0	0	0	0	0	0.0%	0.0%	2.2%	0.0%	0.0%	0.0%	0.0%	0.0%
tr|F1RLG0|F1RLG0	0	0	1	0	0	0	0	0	0	0	3	0	0	0	0	0	0.0%	0.0%	1.7%	0.0%	0.0%	0.0%	0.0%	0.0%
tr|B6ICU5|B6ICU5	0	0	1	0	0	0	0	0	0	0	3	0	0	0	0	0	0.0%	0.0%	12.7%	0.0%	0.0%	0.0%	0.0%	0.0%
tr|F1S4T3|F1S4T3	0	0	1	0	0	0	0	0	0	0	3	0	0	0	0	0	0.0%	0.0%	3.7%	0.0%	0.0%	0.0%	0.0%	0.0%
tr|I3LR73|I3LR73	0	0	1	0	0	0	0	0	0	0	3	0	0	0	0	0	0.0%	0.0%	3.9%	0.0%	0.0%	0.0%	0.0%	0.0%
tr|F1RP07|F1RP07	0	0	1	0	0	0	0	0	0	0	3	0	0	0	0	0	0.0%	0.0%	1.1%	0.0%	0.0%	0.0%	0.0%	0.0%
tr|F1SUS9|F1SUS9	0	0	1	0	0	0	0	0	0	0	3	0	0	0	0	0	0.0%	0.0%	5.2%	0.0%	0.0%	0.0%	0.0%	0.0%
tr|F1SI50|F1SI50	0	0	1	0	0	0	0	0	0	0	3	0	0	0	0	0	0.0%	0.0%	12.2%	0.0%	0.0%	0.0%	0.0%	0.0%
tr|F1SQE3|F1SQE3	0	0	1	0	0	0	0	0	0	0	3	0	0	0	0	0	0.0%	0.0%	4.5%	0.0%	0.0%	0.0%	0.0%	0.0%
tr|F1S1E2|F1S1E2	0	0	1	0	0	0	0	0	0	0	3	0	0	0	0	0	0.0%	0.0%	8.5%	0.0%	0.0%	0.0%	0.0%	0.0%
tr|F1SD18|F1SD18	0	0	2	0	0	0	0	0	0	0	3	0	0	0	0	0	0.0%	0.0%	14.3%	0.0%	0.0%	0.0%	0.0%	0.0%
tr|I3LEZ1|I3LEZ1	0	0	1	0	0	0	0	0	0	0	3	0	0	0	0	0	0.0%	0.0%	8.5%	0.0%	0.0%	0.0%	0.0%	0.0%
tr|F1SG92|F1SG92	0	0	1	0	0	0	0	0	0	0	3	0	0	0	0	0	0.0%	0.0%	7.1%	0.0%	0.0%	0.0%	0.0%	0.0%
tr|F1RRW7|F1RRW7	0	0	2	0	0	0	0	0	0	0	3	0	0	0	0	0	0.0%	0.0%	10.9%	0.0%	0.0%	0.0%	0.0%	0.0%
tr|F1S1E6|F1S1E6	0	0	1	0	0	0	0	0	0	0	3	0	0	0	0	0	0.0%	0.0%	7.7%	0.0%	0.0%	0.0%	0.0%	0.0%
tr|F1SNE5|F1SNE5	0	0	1	0	0	0	0	0	0	0	3	0	0	0	0	0	0.0%	0.0%	3.6%	0.0%	0.0%	0.0%	0.0%	0.0%
tr|K7GLH4|K7GLH4	0	0	2	0	0	0	0	0	0	0	3	0	0	0	0	0	0.0%	0.0%	14.3%	0.0%	0.0%	0.0%	0.0%	0.0%
tr|I3LIY5|I3LIY5	0	0	1	0	0	0	0	0	0	0	3	0	0	0	0	0	0.0%	0.0%	2.4%	0.0%	0.0%	0.0%	0.0%	0.0%
tr|K7GRW8|K7GRW8	0	0	2	0	0	0	0	0	0	0	3	0	0	0	0	0	0.0%	0.0%	21.1%	0.0%	0.0%	0.0%	0.0%	0.0%
tr|F1S5M7|F1S5M7	0	0	2	0	0	0	0	0	0	0	3	0	0	0	0	0	0.0%	0.0%	13.9%	0.0%	0.0%	0.0%	0.0%	0.0%
tr|F1RMH0|F1RMH0	0	0	1	0	0	0	0	0	0	0	3	0	0	0	0	0	0.0%	0.0%	2.3%	0.0%	0.0%	0.0%	0.0%	0.0%
tr|I3LU92|I3LU92	0	0	2	0	0	0	0	0	0	0	3	0	0	0	0	0	0.0%	0.0%	15.8%	0.0%	0.0%	0.0%	0.0%	0.0%
tr|I3L6B8|I3L6B8	0	0	2	0	0	0	0	0	0	0	3	0	0	0	0	0	0.0%	0.0%	8.4%	0.0%	0.0%	0.0%	0.0%	0.0%
tr|F1RPB1|F1RPB1	0	0	1	0	0	0	0	0	0	0	3	0	0	0	0	0	0.0%	0.0%	3.1%	0.0%	0.0%	0.0%	0.0%	0.0%
tr|F1RHS6|F1RHS6	0	0	1	0	0	0	0	0	0	0	3	0	0	0	0	0	0.0%	0.0%	3.2%	0.0%	0.0%	0.0%	0.0%	0.0%
tr|K7GRP2|K7GRP2	0	0	1	0	0	0	0	0	0	0	3	0	0	0	0	0	0.0%	0.0%	41.7%	0.0%	0.0%	0.0%	0.0%	0.0%
tr|F1S0B4|F1S0B4	0	0	1	0	0	0	0	0	0	0	3	0	0	0	0	0	0.0%	0.0%	5.2%	0.0%	0.0%	0.0%	0.0%	0.0%
tr|B6CVL5|B6CVL5	0	0	1	0	0	0	0	0	0	0	3	0	0	0	0	0	0.0%	0.0%	19.8%	0.0%	0.0%	0.0%	0.0%	0.0%
tr|I3LJ88|I3LJ88	0	0	1	0	0	0	0	0	0	0	3	0	0	0	0	0	0.0%	0.0%	5.8%	0.0%	0.0%	0.0%	0.0%	0.0%
tr|F1SJ93|F1SJ93	0	0	2	0	0	0	0	0	0	0	3	0	0	0	0	0	0.0%	0.0%	10.1%	0.0%	0.0%	0.0%	0.0%	0.0%
tr|J9PBT8|J9PBT8	0	0	1	0	0	0	0	0	0	0	3	0	0	0	0	0	0.0%	0.0%	18.4%	0.0%	0.0%	0.0%	0.0%	0.0%
tr|F1S8G3|F1S8G3	0	0	1	0	0	0	0	0	0	0	3	0	0	0	0	0	0.0%	0.0%	11.3%	0.0%	0.0%	0.0%	0.0%	0.0%
tr|I3LCC0|I3LCC0	0	0	1	0	0	0	0	0	0	0	3	0	0	0	0	0	0.0%	0.0%	1.2%	0.0%	0.0%	0.0%	0.0%	0.0%
tr|F1RSQ6|F1RSQ6	0	0	1	0	0	0	0	0	0	0	3	0	0	0	0	0	0.0%	0.0%	25.7%	0.0%	0.0%	0.0%	0.0%	0.0%
tr|F1RT10|F1RT10	0	0	2	0	0	0	0	0	0	0	3	0	0	0	0	0	0.0%	0.0%	8.2%	0.0%	0.0%	0.0%	0.0%	0.0%
tr|I3LJN6|I3LJN6	0	0	1	0	0	0	0	0	0	0	3	0	0	0	0	0	0.0%	0.0%	1.2%	0.0%	0.0%	0.0%	0.0%	0.0%
tr|F1RPU8|F1RPU8	0	0	1	0	0	0	0	0	0	0	3	0	0	0	0	0	0.0%	0.0%	9.4%	0.0%	0.0%	0.0%	0.0%	0.0%
tr|F1RQR1|F1RQR1	0	0	1	0	0	0	0	0	0	0	3	0	0	0	0	0	0.0%	0.0%	2.2%	0.0%	0.0%	0.0%	0.0%	0.0%
A5A779	0	0	1	0	0	0	0	0	0	0	3	0	0	0	0	0	0.0%	0.0%	4.1%	0.0%	0.0%	0.0%	0.0%	0.0%
tr|F1S435|F1S435	0	0	1	0	0	0	0	0	0	0	3	0	0	0	0	0	0.0%	0.0%	5.9%	0.0%	0.0%	0.0%	0.0%	0.0%
tr|K7GR75|K7GR75	0	0	1	0	0	0	0	0	0	0	3	0	0	0	0	0	0.0%	0.0%	16.3%	0.0%	0.0%	0.0%	0.0%	0.0%
tr|I3LIW5|I3LIW5	0	0	1	0	0	0	0	0	0	0	3	0	0	0	0	0	0.0%	0.0%	13.3%	0.0%	0.0%	0.0%	0.0%	0.0%
tr|F1RVB1|F1RVB1	0	0	1	0	0	0	0	0	0	0	3	0	0	0	0	0	0.0%	0.0%	4.1%	0.0%	0.0%	0.0%	0.0%	0.0%
tr|K7N7E9|K7N7E9	0	0	1	0	0	0	0	0	0	0	3	0	0	0	0	0	0.0%	0.0%	15.1%	0.0%	0.0%	0.0%	0.0%	0.0%
tr|I3LQ81|I3LQ81	0	0	1	0	0	0	0	0	0	0	4	0	0	0	0	0	0.0%	0.0%	1.5%	0.0%	0.0%	0.0%	0.0%	0.0%
tr|F1SH10|F1SH10	0	0	1	0	0	0	0	0	0	0	4	0	0	0	0	0	0.0%	0.0%	7.5%	0.0%	0.0%	0.0%	0.0%	0.0%
tr|K7GSG2|K7GSG2	0	0	1	0	0	0	0	0	0	0	4	0	0	0	0	0	0.0%	0.0%	19.6%	0.0%	0.0%	0.0%	0.0%	0.0%
tr|F1SJN6|F1SJN6	0	0	2	0	0	0	0	0	0	0	4	0	0	0	0	0	0.0%	0.0%	2.4%	0.0%	0.0%	0.0%	0.0%	0.0%
tr|I3LQ55|I3LQ55	0	0	1	0	0	0	0	0	0	0	4	0	0	0	0	0	0.0%	0.0%	3.3%	0.0%	0.0%	0.0%	0.0%	0.0%
Reverse_tr|F1RGW2|F1RGW2	0	0	1	0	0	0	0	0	0	0	4	0	0	0	0	0	0.0%	0.0%	6.2%	0.0%	0.0%	0.0%	0.0%	0.0%
tr|I3LM20|I3LM20	0	0	1	0	0	0	0	0	0	0	4	0	0	0	0	0	0.0%	0.0%	14.3%	0.0%	0.0%	0.0%	0.0%	0.0%
Q9MZS9	0	0	1	0	0	0	0	0	0	0	4	0	0	0	0	0	0.0%	0.0%	3.2%	0.0%	0.0%	0.0%	0.0%	0.0%
tr|I3LH54|I3LH54	0	0	1	0	0	0	0	0	0	0	4	0	0	0	0	0	0.0%	0.0%	10.4%	0.0%	0.0%	0.0%	0.0%	0.0%
tr|I3LCC7|I3LCC7	0	0	2	0	0	0	0	0	0	0	4	0	0	0	0	0	0.0%	0.0%	18.1%	0.0%	0.0%	0.0%	0.0%	0.0%
tr|I3LBP0|I3LBP0	0	0	1	0	0	0	0	0	0	0	4	0	0	0	0	0	0.0%	0.0%	7.1%	0.0%	0.0%	0.0%	0.0%	0.0%
tr|F1SAA1|F1SAA1	0	0	1	0	0	0	0	0	0	0	4	0	0	0	0	0	0.0%	0.0%	4.5%	0.0%	0.0%	0.0%	0.0%	0.0%
tr|I3L598|I3L598	0	0	1	0	0	0	0	0	0	0	4	0	0	0	0	0	0.0%	0.0%	12.2%	0.0%	0.0%	0.0%	0.0%	0.0%
tr|I3LF59|I3LF59	0	0	2	0	0	0	0	0	0	0	4	0	0	0	0	0	0.0%	0.0%	4.2%	0.0%	0.0%	0.0%	0.0%	0.0%
tr|I3LCI8|I3LCI8	0	0	1	0	0	0	0	0	0	0	4	0	0	0	0	0	0.0%	0.0%	12.4%	0.0%	0.0%	0.0%	0.0%	0.0%
tr|K7GMG7|K7GMG7	0	0	2	0	0	0	0	0	0	0	4	0	0	0	0	0	0.0%	0.0%	31.7%	0.0%	0.0%	0.0%	0.0%	0.0%
tr|F1RZR2|F1RZR2	0	0	1	0	0	0	0	0	0	0	4	0	0	0	0	0	0.0%	0.0%	16.0%	0.0%	0.0%	0.0%	0.0%	0.0%

	Peptide Count								Spectral Count								Sequence Coverage							
	Whole Cell	Whole Cell	Whole Cell	Cilia Fraction	Cilia Fraction	Cilia Fraction	Cilia Fraction	Cilia Fraction	Whole Cell	Whole Cell	Whole Cell	Cilia Fraction	Cilia Fraction	Cilia Fraction	Cilia Fraction	Cilia Fraction	Whole Cell	Whole Cell	Whole Cell	Cilia Fraction	Cilia Fraction	Cilia Fraction	Cilia Fraction	Cilia Fraction
Sample#	1	2	6	4	3	5	7	8	1	2	6	4	3	5	7	8	1	2	6	4	3	5	7	8
**Accession**																								
tr|F1SNQ9|F1SNQ9	0	0	1	0	0	0	0	0	0	0	4	0	0	0	0	0	0.0%	0.0%	4.7%	0.0%	0.0%	0.0%	0.0%	0.0%
tr|I3LIZ1|I3LIZ1	0	0	1	0	0	0	0	0	0	0	4	0	0	0	0	0	0.0%	0.0%	7.3%	0.0%	0.0%	0.0%	0.0%	0.0%
tr|I3LLM9|I3LLM9	0	0	2	0	0	0	0	0	0	0	4	0	0	0	0	0	0.0%	0.0%	13.6%	0.0%	0.0%	0.0%	0.0%	0.0%
tr|K7GQ09|K7GQ09	0	0	1	0	0	0	0	0	0	0	4	0	0	0	0	0	0.0%	0.0%	23.1%	0.0%	0.0%	0.0%	0.0%	0.0%
tr|I3LJW3|I3LJW3	0	0	1	0	0	0	0	0	0	0	4	0	0	0	0	0	0.0%	0.0%	5.1%	0.0%	0.0%	0.0%	0.0%	0.0%
tr|F1SKZ0|F1SKZ0	0	0	1	0	0	0	0	0	0	0	4	0	0	0	0	0	0.0%	0.0%	10.8%	0.0%	0.0%	0.0%	0.0%	0.0%
tr|F1SDK3|F1SDK3	0	0	1	0	0	0	0	0	0	0	4	0	0	0	0	0	0.0%	0.0%	5.8%	0.0%	0.0%	0.0%	0.0%	0.0%
tr|I3LAL5|I3LAL5	0	0	1	0	0	0	0	0	0	0	4	0	0	0	0	0	0.0%	0.0%	3.1%	0.0%	0.0%	0.0%	0.0%	0.0%
tr|F1RU32|F1RU32	0	0	1	0	0	0	0	0	0	0	4	0	0	0	0	0	0.0%	0.0%	7.2%	0.0%	0.0%	0.0%	0.0%	0.0%
tr|F1S4M5|F1S4M5	0	0	2	0	0	0	0	0	0	0	4	0	0	0	0	0	0.0%	0.0%	7.4%	0.0%	0.0%	0.0%	0.0%	0.0%
tr|F1RFG1|F1RFG1	0	0	1	0	0	0	0	0	0	0	5	0	0	0	0	0	0.0%	0.0%	1.1%	0.0%	0.0%	0.0%	0.0%	0.0%
tr|F1S0H4|F1S0H4	0	0	1	0	0	0	0	0	0	0	5	0	0	0	0	0	0.0%	0.0%	6.5%	0.0%	0.0%	0.0%	0.0%	0.0%
tr|I3LFD1|I3LFD1	0	0	1	0	0	0	0	0	0	0	5	0	0	0	0	0	0.0%	0.0%	22.4%	0.0%	0.0%	0.0%	0.0%	0.0%
tr|F1S712|F1S712	0	0	1	0	0	0	0	0	0	0	5	0	0	0	0	0	0.0%	0.0%	12.8%	0.0%	0.0%	0.0%	0.0%	0.0%
tr|F1SQK9|F1SQK9	0	0	2	0	0	0	0	0	0	0	5	0	0	0	0	0	0.0%	0.0%	9.9%	0.0%	0.0%	0.0%	0.0%	0.0%
tr|I3LPA3|I3LPA3	0	0	1	0	0	0	0	0	0	0	5	0	0	0	0	0	0.0%	0.0%	4.2%	0.0%	0.0%	0.0%	0.0%	0.0%
Q2L969	0	0	2	0	0	0	0	0	0	0	5	0	0	0	0	0	0.0%	0.0%	6.4%	0.0%	0.0%	0.0%	0.0%	0.0%
tr|F1RH41|F1RH41	0	0	2	0	0	0	0	0	0	0	5	0	0	0	0	0	0.0%	0.0%	8.4%	0.0%	0.0%	0.0%	0.0%	0.0%
tr|F1SA10|F1SA10	0	0	1	0	0	0	0	0	0	0	5	0	0	0	0	0	0.0%	0.0%	5.6%	0.0%	0.0%	0.0%	0.0%	0.0%
tr|I3LKV6|I3LKV6	0	0	2	0	0	0	0	0	0	0	5	0	0	0	0	0	0.0%	0.0%	38.4%	0.0%	0.0%	0.0%	0.0%	0.0%
tr|K7GKT7|K7GKT7	0	0	2	0	0	0	0	0	0	0	5	0	0	0	0	0	0.0%	0.0%	7.0%	0.0%	0.0%	0.0%	0.0%	0.0%
tr|K7GML0|K7GML0	0	0	1	0	0	0	0	0	0	0	5	0	0	0	0	0	0.0%	0.0%	14.8%	0.0%	0.0%	0.0%	0.0%	0.0%
tr|F1S7A5|F1S7A5	0	0	1	0	0	0	0	0	0	0	5	0	0	0	0	0	0.0%	0.0%	2.7%	0.0%	0.0%	0.0%	0.0%	0.0%
tr|F1SHC5|F1SHC5	0	0	2	0	0	0	0	0	0	0	5	0	0	0	0	0	0.0%	0.0%	14.0%	0.0%	0.0%	0.0%	0.0%	0.0%
tr|F1RTW4|F1RTW4	0	0	1	0	0	0	0	0	0	0	5	0	0	0	0	0	0.0%	0.0%	9.8%	0.0%	0.0%	0.0%	0.0%	0.0%
tr|I3LV41|I3LV41	0	0	1	0	0	0	0	0	0	0	5	0	0	0	0	0	0.0%	0.0%	2.7%	0.0%	0.0%	0.0%	0.0%	0.0%
tr|I3L5T1|I3L5T1	0	0	1	0	0	0	0	0	0	0	6	0	0	0	0	0	0.0%	0.0%	8.4%	0.0%	0.0%	0.0%	0.0%	0.0%
tr|F1SN45|F1SN45	0	0	1	0	0	0	0	0	0	0	6	0	0	0	0	0	0.0%	0.0%	2.8%	0.0%	0.0%	0.0%	0.0%	0.0%
tr|I3LVQ6|I3LVQ6	0	0	1	0	0	0	0	0	0	0	6	0	0	0	0	0	0.0%	0.0%	12.9%	0.0%	0.0%	0.0%	0.0%	0.0%
tr|F1RTZ0|F1RTZ0	0	0	1	0	0	0	0	0	0	0	6	0	0	0	0	0	0.0%	0.0%	4.6%	0.0%	0.0%	0.0%	0.0%	0.0%
tr|F1SBY8|F1SBY8	0	0	2	0	0	0	0	0	0	0	6	0	0	0	0	0	0.0%	0.0%	5.4%	0.0%	0.0%	0.0%	0.0%	0.0%
tr|F1SQP4|F1SQP4	0	0	3	0	0	0	0	0	0	0	6	0	0	0	0	0	0.0%	0.0%	19.0%	0.0%	0.0%	0.0%	0.0%	0.0%
Q7PCJ9	0	0	2	0	0	0	0	0	0	0	6	0	0	0	0	0	0.0%	0.0%	13.5%	0.0%	0.0%	0.0%	0.0%	0.0%
tr|F1SEB9|F1SEB9	0	0	1	0	0	0	0	0	0	0	6	0	0	0	0	0	0.0%	0.0%	14.1%	0.0%	0.0%	0.0%	0.0%	0.0%
tr|F1ST40|F1ST40	0	0	2	0	0	0	0	0	0	0	6	0	0	0	0	0	0.0%	0.0%	13.5%	0.0%	0.0%	0.0%	0.0%	0.0%
tr|I3LSW7|I3LSW7	0	0	1	0	0	0	0	0	0	0	6	0	0	0	0	0	0.0%	0.0%	11.4%	0.0%	0.0%	0.0%	0.0%	0.0%
tr|K7GPZ8|K7GPZ8	0	0	3	0	0	0	0	0	0	0	6	0	0	0	0	0	0.0%	0.0%	19.2%	0.0%	0.0%	0.0%	0.0%	0.0%
tr|F1S4R9|F1S4R9	0	0	1	0	0	0	0	0	0	0	6	0	0	0	0	0	0.0%	0.0%	4.5%	0.0%	0.0%	0.0%	0.0%	0.0%
tr|F1S5H2|F1S5H2	0	0	2	0	0	0	0	0	0	0	6	0	0	0	0	0	0.0%	0.0%	4.7%	0.0%	0.0%	0.0%	0.0%	0.0%
tr|I3LMS2|I3LMS2	0	0	1	0	0	0	0	0	0	0	6	0	0	0	0	0	0.0%	0.0%	2.5%	0.0%	0.0%	0.0%	0.0%	0.0%
tr|I3LJ21|I3LJ21	0	0	2	0	0	0	0	0	0	0	6	0	0	0	0	0	0.0%	0.0%	13.3%	0.0%	0.0%	0.0%	0.0%	0.0%
tr|F1S5U8|F1S5U8	0	0	1	0	0	0	0	0	0	0	6	0	0	0	0	0	0.0%	0.0%	4.4%	0.0%	0.0%	0.0%	0.0%	0.0%
tr|I3LDP1|I3LDP1	0	0	1	0	0	0	0	0	0	0	6	0	0	0	0	0	0.0%	0.0%	3.6%	0.0%	0.0%	0.0%	0.0%	0.0%
tr|I3LGA3|I3LGA3	0	0	1	0	0	0	0	0	0	0	6	0	0	0	0	0	0.0%	0.0%	10.5%	0.0%	0.0%	0.0%	0.0%	0.0%
tr|F1SJP9|F1SJP9	0	0	1	0	0	0	0	0	0	0	6	0	0	0	0	0	0.0%	0.0%	2.7%	0.0%	0.0%	0.0%	0.0%	0.0%
tr|I3LTC6|I3LTC6	0	0	3	0	0	0	0	0	0	0	6	0	0	0	0	0	0.0%	0.0%	13.8%	0.0%	0.0%	0.0%	0.0%	0.0%
tr|I3L7T0|I3L7T0	0	0	1	0	0	0	0	0	0	0	6	0	0	0	0	0	0.0%	0.0%	7.3%	0.0%	0.0%	0.0%	0.0%	0.0%
tr|K7GN72|K7GN72	0	0	1	0	0	0	0	0	0	0	7	0	0	0	0	0	0.0%	0.0%	3.3%	0.0%	0.0%	0.0%	0.0%	0.0%
tr|I3LN63|I3LN63	0	0	2	0	0	0	0	0	0	0	7	0	0	0	0	0	0.0%	0.0%	13.4%	0.0%	0.0%	0.0%	0.0%	0.0%
tr|F1SU66|F1SU66	0	0	2	0	0	0	0	0	0	0	7	0	0	0	0	0	0.0%	0.0%	6.7%	0.0%	0.0%	0.0%	0.0%	0.0%
tr|I3L8U2|I3L8U2	0	0	1	0	0	0	0	0	0	0	7	0	0	0	0	0	0.0%	0.0%	4.8%	0.0%	0.0%	0.0%	0.0%	0.0%
tr|F1S3Z4|F1S3Z4	0	0	1	0	0	0	0	0	0	0	7	0	0	0	0	0	0.0%	0.0%	11.9%	0.0%	0.0%	0.0%	0.0%	0.0%
tr|I3LFW8|I3LFW8	0	0	1	0	0	0	0	0	0	0	7	0	0	0	0	0	0.0%	0.0%	21.1%	0.0%	0.0%	0.0%	0.0%	0.0%
tr|F1SIE4|F1SIE4	0	0	1	0	0	0	0	0	0	0	7	0	0	0	0	0	0.0%	0.0%	4.7%	0.0%	0.0%	0.0%	0.0%	0.0%
tr|F1RI72|F1RI72	0	0	2	0	0	0	0	0	0	0	7	0	0	0	0	0	0.0%	0.0%	2.3%	0.0%	0.0%	0.0%	0.0%	0.0%
tr|F1RLR7|F1RLR7	0	0	1	0	0	0	0	0	0	0	7	0	0	0	0	0	0.0%	0.0%	4.9%	0.0%	0.0%	0.0%	0.0%	0.0%
tr|Q8MKG1|Q8MKG1	0	0	2	0	0	0	0	0	0	0	7	0	0	0	0	0	0.0%	0.0%	9.2%	0.0%	0.0%	0.0%	0.0%	0.0%
tr|F1RJV5|F1RJV5	0	0	2	0	0	0	0	0	0	0	7	0	0	0	0	0	0.0%	0.0%	8.7%	0.0%	0.0%	0.0%	0.0%	0.0%
A7WLH8	0	0	1	0	0	0	0	0	0	0	7	0	0	0	0	0	0.0%	0.0%	11.9%	0.0%	0.0%	0.0%	0.0%	0.0%
tr|I3LHR8|I3LHR8	0	0	1	0	0	0	0	0	0	0	7	0	0	0	0	0	0.0%	0.0%	4.9%	0.0%	0.0%	0.0%	0.0%	0.0%
tr|F1RJM4|F1RJM4	0	0	1	0	0	0	0	0	0	0	7	0	0	0	0	0	0.0%	0.0%	3.3%	0.0%	0.0%	0.0%	0.0%	0.0%
tr|I3LFB4|I3LFB4	0	0	1	0	0	0	0	0	0	0	7	0	0	0	0	0	0.0%	0.0%	6.5%	0.0%	0.0%	0.0%	0.0%	0.0%
tr|F1SGV6|F1SGV6	0	0	1	0	0	0	0	0	0	0	7	0	0	0	0	0	0.0%	0.0%	6.4%	0.0%	0.0%	0.0%	0.0%	0.0%
tr|F1S0B5|F1S0B5	0	0	1	0	0	0	0	0	0	0	7	0	0	0	0	0	0.0%	0.0%	11.9%	0.0%	0.0%	0.0%	0.0%	0.0%
tr|I3LL51|I3LL51	0	0	1	0	0	0	0	0	0	0	7	0	0	0	0	0	0.0%	0.0%	4.7%	0.0%	0.0%	0.0%	0.0%	0.0%
tr|I3L7H3|I3L7H3	0	0	1	0	0	0	0	0	0	0	8	0	0	0	0	0	0.0%	0.0%	8.0%	0.0%	0.0%	0.0%	0.0%	0.0%
tr|K7GP26|K7GP26	0	0	1	0	0	0	0	0	0	0	8	0	0	0	0	0	0.0%	0.0%	8.0%	0.0%	0.0%	0.0%	0.0%	0.0%
tr|K7GR44|K7GR44	0	0	1	0	0	0	0	0	0	0	8	0	0	0	0	0	0.0%	0.0%	7.6%	0.0%	0.0%	0.0%	0.0%	0.0%
tr|I3LNE1|I3LNE1	0	0	3	0	0	0	0	0	0	0	8	0	0	0	0	0	0.0%	0.0%	3.7%	0.0%	0.0%	0.0%	0.0%	0.0%
tr|I3LEP9|I3LEP9	0	0	3	0	0	0	0	0	0	0	8	0	0	0	0	0	0.0%	0.0%	3.7%	0.0%	0.0%	0.0%	0.0%	0.0%
tr|I3LJ28|I3LJ28	0	0	2	0	0	0	0	0	0	0	8	0	0	0	0	0	0.0%	0.0%	12.2%	0.0%	0.0%	0.0%	0.0%	0.0%
tr|K7GSZ0|K7GSZ0	0	0	1	0	0	0	0	0	0	0	8	0	0	0	0	0	0.0%	0.0%	8.3%	0.0%	0.0%	0.0%	0.0%	0.0%
tr|I3LFS7|I3LFS7	0	0	1	0	0	0	0	0	0	0	8	0	0	0	0	0	0.0%	0.0%	4.4%	0.0%	0.0%	0.0%	0.0%	0.0%
tr|B8XTR3|B8XTR3	0	0	2	0	0	0	0	0	0	0	8	0	0	0	0	0	0.0%	0.0%	8.5%	0.0%	0.0%	0.0%	0.0%	0.0%
tr|F1SEQ5|F1SEQ5	0	0	1	0	0	0	0	0	0	0	8	0	0	0	0	0	0.0%	0.0%	2.8%	0.0%	0.0%	0.0%	0.0%	0.0%
tr|I3LU76|I3LU76	0	0	2	0	0	0	0	0	0	0	9	0	0	0	0	0	0.0%	0.0%	2.0%	0.0%	0.0%	0.0%	0.0%	0.0%
tr|F1SND9|F1SND9	0	0	1	0	0	0	0	0	0	0	9	0	0	0	0	0	0.0%	0.0%	4.9%	0.0%	0.0%	0.0%	0.0%	0.0%
tr|K7GSY4|K7GSY4	0	0	1	0	0	0	0	0	0	0	9	0	0	0	0	0	0.0%	0.0%	35.1%	0.0%	0.0%	0.0%	0.0%	0.0%
tr|I3L8P6|I3L8P6	0	0	1	0	0	0	0	0	0	0	9	0	0	0	0	0	0.0%	0.0%	7.9%	0.0%	0.0%	0.0%	0.0%	0.0%
tr|I3LS49|I3LS49	0	0	2	0	0	0	0	0	0	0	9	0	0	0	0	0	0.0%	0.0%	21.6%	0.0%	0.0%	0.0%	0.0%	0.0%
tr|F1RSJ8|F1RSJ8	0	0	2	0	0	0	0	0	0	0	9	0	0	0	0	0	0.0%	0.0%	4.7%	0.0%	0.0%	0.0%	0.0%	0.0%

	Peptide Count								Spectral Count								Sequence Coverage							
	Whole Cell	Whole Cell	Whole Cell	Cilia Fraction	Cilia Fraction	Cilia Fraction	Cilia Fraction	Cilia Fraction	Whole Cell	Whole Cell	Whole Cell	Cilia Fraction	Cilia Fraction	Cilia Fraction	Cilia Fraction	Cilia Fraction	Whole Cell	Whole Cell	Whole Cell	Cilia Fraction	Cilia Fraction	Cilia Fraction	Cilia Fraction	Cilia Fraction
Sample#	1	2	6	4	3	5	7	8	1	2	6	4	3	5	7	8	1	2	6	4	3	5	7	8
**Accession**																								
tr|I3LC46|I3LC46	0	0	2	0	0	0	0	0	0	0	9	0	0	0	0	0	0.0%	0.0%	2.8%	0.0%	0.0%	0.0%	0.0%	0.0%
tr|F1SBJ2|F1SBJ2	0	0	1	0	0	0	0	0	0	0	9	0	0	0	0	0	0.0%	0.0%	3.1%	0.0%	0.0%	0.0%	0.0%	0.0%
tr|F1SAR5|F1SAR5	0	0	2	0	0	0	0	0	0	0	9	0	0	0	0	0	0.0%	0.0%	24.0%	0.0%	0.0%	0.0%	0.0%	0.0%
tr|F1SQ89|F1SQ89	0	0	2	0	0	0	0	0	0	0	9	0	0	0	0	0	0.0%	0.0%	2.1%	0.0%	0.0%	0.0%	0.0%	0.0%
tr|F1RKY8|F1RKY8	0	0	1	0	0	0	0	0	0	0	9	0	0	0	0	0	0.0%	0.0%	11.2%	0.0%	0.0%	0.0%	0.0%	0.0%
tr|F1SDP0|F1SDP0	0	0	2	0	0	0	0	0	0	0	9	0	0	0	0	0	0.0%	0.0%	7.5%	0.0%	0.0%	0.0%	0.0%	0.0%
tr|F1RU86|F1RU86	0	0	1	0	0	0	0	0	0	0	10	0	0	0	0	0	0.0%	0.0%	29.4%	0.0%	0.0%	0.0%	0.0%	0.0%
tr|F1SD73|F1SD73	0	0	1	0	0	0	0	0	0	0	10	0	0	0	0	0	0.0%	0.0%	19.0%	0.0%	0.0%	0.0%	0.0%	0.0%
tr|I3LH04|I3LH04	0	0	1	0	0	0	0	0	0	0	10	0	0	0	0	0	0.0%	0.0%	24.0%	0.0%	0.0%	0.0%	0.0%	0.0%
tr|F1SVB4|F1SVB4	0	0	1	0	0	0	0	0	0	0	10	0	0	0	0	0	0.0%	0.0%	5.8%	0.0%	0.0%	0.0%	0.0%	0.0%
tr|I3LFS1|I3LFS1	0	0	1	0	0	0	0	0	0	0	10	0	0	0	0	0	0.0%	0.0%	4.4%	0.0%	0.0%	0.0%	0.0%	0.0%
tr|I3LQ38|I3LQ38	0	0	1	0	0	0	0	0	0	0	10	0	0	0	0	0	0.0%	0.0%	20.8%	0.0%	0.0%	0.0%	0.0%	0.0%
tr|I3LI59|I3LI59	0	0	2	0	0	0	0	0	0	0	10	0	0	0	0	0	0.0%	0.0%	10.7%	0.0%	0.0%	0.0%	0.0%	0.0%
tr|I3LN60|I3LN60	0	0	1	0	0	0	0	0	0	0	10	0	0	0	0	0	0.0%	0.0%	29.6%	0.0%	0.0%	0.0%	0.0%	0.0%
tr|I3LHS5|I3LHS5	0	0	2	0	0	0	0	0	0	0	10	0	0	0	0	0	0.0%	0.0%	14.0%	0.0%	0.0%	0.0%	0.0%	0.0%
tr|I3LMR9|I3LMR9	0	0	2	0	0	0	0	0	0	0	11	0	0	0	0	0	0.0%	0.0%	16.8%	0.0%	0.0%	0.0%	0.0%	0.0%
tr|F1S995|F1S995	0	0	2	0	0	0	0	0	0	0	11	0	0	0	0	0	0.0%	0.0%	4.5%	0.0%	0.0%	0.0%	0.0%	0.0%
P36968	0	0	5	0	0	0	0	0	0	0	11	0	0	0	0	0	0.0%	0.0%	22.3%	0.0%	0.0%	0.0%	0.0%	0.0%
tr|I3LMK0|I3LMK0	0	0	2	0	0	0	0	0	0	0	11	0	0	0	0	0	0.0%	0.0%	16.5%	0.0%	0.0%	0.0%	0.0%	0.0%
tr|F1RPB0|F1RPB0	0	0	4	0	0	0	0	0	0	0	12	0	0	0	0	0	0.0%	0.0%	8.3%	0.0%	0.0%	0.0%	0.0%	0.0%
P19130	0	0	2	0	0	0	0	0	0	0	12	0	0	0	0	0	0.0%	0.0%	12.7%	0.0%	0.0%	0.0%	0.0%	0.0%
tr|F1RT63|F1RT63	0	0	2	0	0	0	0	0	0	0	12	0	0	0	0	0	0.0%	0.0%	36.5%	0.0%	0.0%	0.0%	0.0%	0.0%
tr|K7GQ47|K7GQ47	0	0	2	0	0	0	0	0	0	0	12	0	0	0	0	0	0.0%	0.0%	10.2%	0.0%	0.0%	0.0%	0.0%	0.0%
tr|F1RQW5|F1RQW5	0	0	2	0	0	0	0	0	0	0	12	0	0	0	0	0	0.0%	0.0%	10.1%	0.0%	0.0%	0.0%	0.0%	0.0%
tr|I3LI90|I3LI90	0	0	3	0	0	0	0	0	0	0	13	0	0	0	0	0	0.0%	0.0%	22.3%	0.0%	0.0%	0.0%	0.0%	0.0%
tr|F2Z542|F2Z542	0	0	3	0	0	0	0	0	0	0	13	0	0	0	0	0	0.0%	0.0%	20.5%	0.0%	0.0%	0.0%	0.0%	0.0%
A1XQR7	0	0	3	0	0	0	0	0	0	0	13	0	0	0	0	0	0.0%	0.0%	39.8%	0.0%	0.0%	0.0%	0.0%	0.0%
tr|F1SQR4|F1SQR4	0	0	1	0	0	0	0	0	0	0	13	0	0	0	0	0	0.0%	0.0%	15.1%	0.0%	0.0%	0.0%	0.0%	0.0%
tr|D3GGC9|D3GGC9	0	0	2	0	0	0	0	0	0	0	13	0	0	0	0	0	0.0%	0.0%	6.5%	0.0%	0.0%	0.0%	0.0%	0.0%
Q6QGC0	0	0	2	0	0	0	0	0	0	0	13	0	0	0	0	0	0.0%	0.0%	4.9%	0.0%	0.0%	0.0%	0.0%	0.0%
P82125	0	0	1	0	0	0	0	0	0	0	14	0	0	0	0	0	0.0%	0.0%	3.0%	0.0%	0.0%	0.0%	0.0%	0.0%
tr|I3LRN9|I3LRN9	0	0	1	0	0	0	0	0	0	0	14	0	0	0	0	0	0.0%	0.0%	3.1%	0.0%	0.0%	0.0%	0.0%	0.0%
tr|I3LL26|I3LL26	0	0	1	0	0	0	0	0	0	0	14	0	0	0	0	0	0.0%	0.0%	7.9%	0.0%	0.0%	0.0%	0.0%	0.0%
tr|I3LIC1|I3LIC1	0	0	1	0	0	0	0	0	0	0	15	0	0	0	0	0	0.0%	0.0%	26.4%	0.0%	0.0%	0.0%	0.0%	0.0%
tr|I3LJM0|I3LJM0	0	0	3	0	0	0	0	0	0	0	15	0	0	0	0	0	0.0%	0.0%	60.6%	0.0%	0.0%	0.0%	0.0%	0.0%
tr|K7GM69|K7GM69	0	0	2	0	0	0	0	0	0	0	17	0	0	0	0	0	0.0%	0.0%	12.9%	0.0%	0.0%	0.0%	0.0%	0.0%
tr|K7GT34|K7GT34	0	0	2	0	0	0	0	0	0	0	17	0	0	0	0	0	0.0%	0.0%	20.0%	0.0%	0.0%	0.0%	0.0%	0.0%
tr|I3L9B6|I3L9B6	0	0	3	0	0	0	0	0	0	0	18	0	0	0	0	0	0.0%	0.0%	11.4%	0.0%	0.0%	0.0%	0.0%	0.0%
tr|F1SQX8|F1SQX8	0	0	2	0	0	0	0	0	0	0	19	0	0	0	0	0	0.0%	0.0%	27.4%	0.0%	0.0%	0.0%	0.0%	0.0%
tr|I3L8N1|I3L8N1	0	0	2	0	0	0	0	0	0	0	19	0	0	0	0	0	0.0%	0.0%	20.0%	0.0%	0.0%	0.0%	0.0%	0.0%
tr|F1RWV4|F1RWV4	0	0	1	0	0	0	0	0	0	0	26	0	0	0	0	0	0.0%	0.0%	17.5%	0.0%	0.0%	0.0%	0.0%	0.0%
tr|F1RVL9|F1RVL9	0	0	2	0	0	0	0	0	0	0	29	0	0	0	0	0	0.0%	0.0%	12.3%	0.0%	0.0%	0.0%	0.0%	0.0%
tr|I3L9C9|I3L9C9	0	0	4	0	0	0	0	0	0	0	29	0	0	0	0	0	0.0%	0.0%	4.0%	0.0%	0.0%	0.0%	0.0%	0.0%
tr|A5GFP5|A5GFP5	0	0	6	0	0	0	0	0	0	0	31	0	0	0	0	0	0.0%	0.0%	25.7%	0.0%	0.0%	0.0%	0.0%	0.0%
tr|A5A8Y7|A5A8Y7	0	0	1	0	0	0	0	0	0	0	48	0	0	0	0	0	0.0%	0.0%	7.3%	0.0%	0.0%	0.0%	0.0%	0.0%
tr|A5A8Y6|A5A8Y6	0	0	1	0	0	0	0	0	0	0	48	0	0	0	0	0	0.0%	0.0%	8.3%	0.0%	0.0%	0.0%	0.0%	0.0%
tr|I3LAH9|I3LAH9	0	0	2	0	0	0	0	0	0	0	86	0	0	0	0	0	0.0%	0.0%	20.5%	0.0%	0.0%	0.0%	0.0%	0.0%
tr|F1RQT1|F1RQT1	0	0	0	0	0	0	9	14	0	0	0	0	0	0	57	77	0.0%	0.0%	0.0%	0.0%	0.0%	0.0%	21.4%	25.1%
P22412	0	0	0	0	0	0	5	9	0	0	0	0	0	0	42	53	0.0%	0.0%	0.0%	0.0%	0.0%	0.0%	26.2%	25.7%
tr|I3L719|I3L719	0	0	0	0	0	0	4	7	0	0	0	0	0	0	25	41	0.0%	0.0%	0.0%	0.0%	0.0%	0.0%	21.3%	20.8%
tr|F1S3P2|F1S3P2	0	0	0	0	0	0	2	2	0	0	0	0	0	0	26	36	0.0%	0.0%	0.0%	0.0%	0.0%	0.0%	10.1%	10.1%
tr|K7GMT2|K7GMT2	0	0	0	0	0	0	1	1	0	0	0	0	0	0	8	54	0.0%	0.0%	0.0%	0.0%	0.0%	0.0%	32.9%	32.9%
tr|F1S785|F1S785	0	0	0	0	0	0	2	2	0	0	0	0	0	0	18	17	0.0%	0.0%	0.0%	0.0%	0.0%	0.0%	11.6%	11.6%
tr|I3LH49|I3LH49	0	0	0	0	0	0	1	2	0	0	0	0	0	0	28	3	0.0%	0.0%	0.0%	0.0%	0.0%	0.0%	6.5%	12.3%
tr|F1SCU4|F1SCU4	0	0	0	0	0	0	1	1	0	0	0	0	0	0	16	15	0.0%	0.0%	0.0%	0.0%	0.0%	0.0%	4.5%	4.5%
tr|F1S4Q8|F1S4Q8	0	0	0	0	0	0	1	1	0	0	0	0	0	0	28	2	0.0%	0.0%	0.0%	0.0%	0.0%	0.0%	5.5%	6.3%
tr|F1SIE2|F1SIE2	0	0	0	0	0	0	1	6	0	0	0	0	0	0	3	27	0.0%	0.0%	0.0%	0.0%	0.0%	0.0%	3.1%	6.8%
tr|F1SDL5|F1SDL5	0	0	0	0	0	0	1	3	0	0	0	0	0	0	8	20	0.0%	0.0%	0.0%	0.0%	0.0%	0.0%	4.4%	11.0%
tr|I3L6D7|I3L6D7	0	0	0	0	0	0	2	3	0	0	0	0	0	0	14	14	0.0%	0.0%	0.0%	0.0%	0.0%	0.0%	4.5%	5.4%
Q684M4	0	0	0	0	0	0	3	3	0	0	0	0	0	0	14	14	0.0%	0.0%	0.0%	0.0%	0.0%	0.0%	12.8%	13.0%
tr|I3LIM4|I3LIM4	0	0	0	0	0	0	2	3	0	0	0	0	0	0	14	14	0.0%	0.0%	0.0%	0.0%	0.0%	0.0%	4.4%	5.4%
tr|F1S3Q9|F1S3Q9	0	0	0	0	0	0	3	3	0	0	0	0	0	0	11	16	0.0%	0.0%	0.0%	0.0%	0.0%	0.0%	7.7%	11.1%
tr|I3LBL4|I3LBL4	0	0	0	0	0	0	0	4	0	0	0	0	0	0	0	25	0.0%	0.0%	0.0%	0.0%	0.0%	0.0%	0.0%	16.3%
tr|F1SEB5|F1SEB5	0	0	0	0	0	0	1	4	0	0	0	0	0	0	3	21	0.0%	0.0%	0.0%	0.0%	0.0%	0.0%	5.6%	14.8%
tr|K7GNP9|K7GNP9	0	0	0	0	0	0	0	6	0	0	0	0	0	0	0	24	0.0%	0.0%	0.0%	0.0%	0.0%	0.0%	0.0%	29.9%
tr|F1RLV1|F1RLV1	0	0	0	0	0	0	3	3	0	0	0	0	0	0	19	4	0.0%	0.0%	0.0%	0.0%	0.0%	0.0%	7.3%	6.2%
P26429	0	0	0	0	0	0	3	3	0	0	0	0	0	0	19	4	0.0%	0.0%	0.0%	0.0%	0.0%	0.0%	7.9%	6.8%
tr|F1RRN0|F1RRN0	0	0	0	0	0	0	0	8	0	0	0	0	0	0	0	22	0.0%	0.0%	0.0%	0.0%	0.0%	0.0%	0.0%	12.5%
tr|I3LJ42|I3LJ42	0	0	0	0	0	0	0	2	0	0	0	0	0	0	0	21	0.0%	0.0%	0.0%	0.0%	0.0%	0.0%	0.0%	33.0%
tr|F1SMP2|F1SMP2	0	0	0	0	0	0	2	3	0	0	0	0	0	0	16	5	0.0%	0.0%	0.0%	0.0%	0.0%	0.0%	9.2%	17.8%
tr|F1SAX6|F1SAX6	0	0	0	0	0	0	0	1	0	0	0	0	0	0	0	20	0.0%	0.0%	0.0%	0.0%	0.0%	0.0%	0.0%	24.6%
tr|I3LIR5|I3LIR5	0	0	0	0	0	0	1	3	0	0	0	0	0	0	6	14	0.0%	0.0%	0.0%	0.0%	0.0%	0.0%	3.6%	12.5%
tr|F1SK96|F1SK96	0	0	0	0	0	0	0	4	0	0	0	0	0	0	0	20	0.0%	0.0%	0.0%	0.0%	0.0%	0.0%	0.0%	36.7%
tr|F1RFS7|F1RFS7	0	0	0	0	0	0	0	5	0	0	0	0	0	0	0	20	0.0%	0.0%	0.0%	0.0%	0.0%	0.0%	0.0%	10.1%
tr|I3LMU5|I3LMU5	0	0	0	0	0	0	2	2	0	0	0	0	0	0	7	13	0.0%	0.0%	0.0%	0.0%	0.0%	0.0%	1.3%	1.3%
tr|F1RGX2|F1RGX2	0	0	0	0	0	0	1	3	0	0	0	0	0	0	5	15	0.0%	0.0%	0.0%	0.0%	0.0%	0.0%	2.4%	5.3%
tr|I3LA41|I3LA41	0	0	0	0	0	0	0	1	0	0	0	0	0	0	0	19	0.0%	0.0%	0.0%	0.0%	0.0%	0.0%	0.0%	5.8%
tr|I3LUE2|I3LUE2	0	0	0	0	0	0	2	2	0	0	0	0	0	0	16	3	0.0%	0.0%	0.0%	0.0%	0.0%	0.0%	2.8%	2.8%
tr|F1RSN6|F1RSN6	0	0	0	0	0	0	0	3	0	0	0	0	0	0	0	19	0.0%	0.0%	0.0%	0.0%	0.0%	0.0%	0.0%	13.7%
tr|F1RXA1|F1RXA1	0	0	0	0	0	0	0	4	0	0	0	0	0	0	0	19	0.0%	0.0%	0.0%	0.0%	0.0%	0.0%	0.0%	10.8%
tr|I3LGE9|I3LGE9	0	0	0	0	0	0	2	2	0	0	0	0	0	0	16	3	0.0%	0.0%	0.0%	0.0%	0.0%	0.0%	1.8%	1.8%

	Peptide Count								Spectral Count								Sequence Coverage							
	Whole Cell	Whole Cell	Whole Cell	Cilia Fraction	Cilia Fraction	Cilia Fraction	Cilia Fraction	Cilia Fraction	Whole Cell	Whole Cell	Whole Cell	Cilia Fraction	Cilia Fraction	Cilia Fraction	Cilia Fraction	Cilia Fraction	Whole Cell	Whole Cell	Whole Cell	Cilia Fraction	Cilia Fraction	Cilia Fraction	Cilia Fraction	Cilia Fraction
Sample#	1	2	6	4	3	5	7	8	1	2	6	4	3	5	7	8	1	2	6	4	3	5	7	8
**Accession**																								
tr|I3LCS3|I3LCS3	0	0	0	0	0	0	1	1	0	0	0	0	0	0	15	4	0.0%	0.0%	0.0%	0.0%	0.0%	0.0%	3.0%	3.0%
tr|K7GNY7|K7GNY7	0	0	0	0	0	0	1	3	0	0	0	0	0	0	3	16	0.0%	0.0%	0.0%	0.0%	0.0%	0.0%	19.4%	21.3%
tr|I3L6Z1|I3L6Z1	0	0	0	0	0	0	1	2	0	0	0	0	0	0	3	16	0.0%	0.0%	0.0%	0.0%	0.0%	0.0%	2.9%	2.8%
Q863Y7	0	0	0	0	0	0	0	3	0	0	0	0	0	0	0	19	0.0%	0.0%	0.0%	0.0%	0.0%	0.0%	0.0%	13.7%
tr|F1SA41|F1SA41	0	0	0	0	0	0	0	8	0	0	0	0	0	0	0	18	0.0%	0.0%	0.0%	0.0%	0.0%	0.0%	0.0%	15.5%
tr|I3LTJ1|I3LTJ1	0	0	0	0	0	0	1	4	0	0	0	0	0	0	5	13	0.0%	0.0%	0.0%	0.0%	0.0%	0.0%	2.6%	6.9%
tr|Q85ZW4|Q85ZW4	0	0	0	0	0	0	0	1	0	0	0	0	0	0	0	17	0.0%	0.0%	0.0%	0.0%	0.0%	0.0%	0.0%	9.1%
tr|F1S7V3|F1S7V3	0	0	0	0	0	0	0	1	0	0	0	0	0	0	0	17	0.0%	0.0%	0.0%	0.0%	0.0%	0.0%	0.0%	4.5%
tr|I3LE42|I3LE42	0	0	0	0	0	0	1	4	0	0	0	0	0	0	1	14	0.0%	0.0%	0.0%	0.0%	0.0%	0.0%	4.9%	12.3%
tr|I3LRJ7|I3LRJ7	0	0	0	0	0	0	1	4	0	0	0	0	0	0	1	14	0.0%	0.0%	0.0%	0.0%	0.0%	0.0%	4.5%	8.9%
tr|I3LGQ1|I3LGQ1	0	0	0	0	0	0	1	1	0	0	0	0	0	0	4	11	0.0%	0.0%	0.0%	0.0%	0.0%	0.0%	4.0%	4.0%
tr|F1RJW5|F1RJW5	0	0	0	0	0	0	1	6	0	0	0	0	0	0	1	13	0.0%	0.0%	0.0%	0.0%	0.0%	0.0%	2.1%	17.5%
P20303	0	0	0	0	0	0	1	2	0	0	0	0	0	0	9	5	0.0%	0.0%	0.0%	0.0%	0.0%	0.0%	5.3%	7.3%
tr|I3LTE1|I3LTE1	0	0	0	0	0	0	0	4	0	0	0	0	0	0	0	14	0.0%	0.0%	0.0%	0.0%	0.0%	0.0%	0.0%	17.5%
tr|F1RRI2|F1RRI2	0	0	0	0	0	0	0	6	0	0	0	0	0	0	0	13	0.0%	0.0%	0.0%	0.0%	0.0%	0.0%	0.0%	21.5%
tr|F1RYX3|F1RYX3	0	0	0	0	0	0	1	2	0	0	0	0	0	0	9	3	0.0%	0.0%	0.0%	0.0%	0.0%	0.0%	2.9%	2.9%
tr|F1SH61|F1SH61	0	0	0	0	0	0	0	3	0	0	0	0	0	0	0	12	0.0%	0.0%	0.0%	0.0%	0.0%	0.0%	0.0%	32.3%
tr|F1SMP3|F1SMP3	0	0	0	0	0	0	0	6	0	0	0	0	0	0	0	12	0.0%	0.0%	0.0%	0.0%	0.0%	0.0%	0.0%	8.4%
tr|I3LG33|I3LG33	0	0	0	0	0	0	0	1	0	0	0	0	0	0	0	12	0.0%	0.0%	0.0%	0.0%	0.0%	0.0%	0.0%	3.3%
tr|B3TFD9|B3TFD9	0	0	0	0	0	0	0	6	0	0	0	0	0	0	0	12	0.0%	0.0%	0.0%	0.0%	0.0%	0.0%	0.0%	17.1%
tr|I3LIE6|I3LIE6	0	0	0	0	0	0	1	3	0	0	0	0	0	0	4	8	0.0%	0.0%	0.0%	0.0%	0.0%	0.0%	5.2%	12.1%
tr|I3L5Z2|I3L5Z2	0	0	0	0	0	0	1	1	0	0	0	0	0	0	9	3	0.0%	0.0%	0.0%	0.0%	0.0%	0.0%	18.6%	18.6%
tr|K7GRW6|K7GRW6	0	0	0	0	0	0	0	3	0	0	0	0	0	0	0	11	0.0%	0.0%	0.0%	0.0%	0.0%	0.0%	0.0%	8.3%
tr|F1S8Y1|F1S8Y1	0	0	0	0	0	0	0	3	0	0	0	0	0	0	0	11	0.0%	0.0%	0.0%	0.0%	0.0%	0.0%	0.0%	26.3%
tr|I3L9P2|I3L9P2	0	0	0	0	0	0	0	3	0	0	0	0	0	0	0	11	0.0%	0.0%	0.0%	0.0%	0.0%	0.0%	0.0%	27.3%
Q2QDF0	0	0	0	0	0	0	3	2	0	0	0	0	0	0	8	3	0.0%	0.0%	0.0%	0.0%	0.0%	0.0%	17.5%	16.2%
tr|I3LHT8|I3LHT8	0	0	0	0	0	0	1	1	0	0	0	0	0	0	1	10	0.0%	0.0%	0.0%	0.0%	0.0%	0.0%	4.3%	4.3%
tr|I3LN42|I3LN42	0	0	0	0	0	0	2	1	0	0	0	0	0	0	10	1	0.0%	0.0%	0.0%	0.0%	0.0%	0.0%	8.2%	4.8%
Q06AA5	0	0	0	0	0	0	0	1	0	0	0	0	0	0	0	11	0.0%	0.0%	0.0%	0.0%	0.0%	0.0%	0.0%	13.8%
tr|F1RGZ3|F1RGZ3	0	0	0	0	0	0	0	3	0	0	0	0	0	0	0	11	0.0%	0.0%	0.0%	0.0%	0.0%	0.0%	0.0%	7.4%
tr|F1SQ58|F1SQ58	0	0	0	0	0	0	1	1	0	0	0	0	0	0	6	4	0.0%	0.0%	0.0%	0.0%	0.0%	0.0%	5.0%	10.0%
tr|F1SSS5|F1SSS5	0	0	0	0	0	0	2	1	0	0	0	0	0	0	4	6	0.0%	0.0%	0.0%	0.0%	0.0%	0.0%	20.0%	11.1%
tr|I3LN54|I3LN54	0	0	0	0	0	0	3	1	0	0	0	0	0	0	9	1	0.0%	0.0%	0.0%	0.0%	0.0%	0.0%	18.7%	13.8%
tr|F1SSS3|F1SSS3	0	0	0	0	0	0	2	1	0	0	0	0	0	0	4	6	0.0%	0.0%	0.0%	0.0%	0.0%	0.0%	20.3%	11.2%
tr|K7GK77|K7GK77	0	0	0	0	0	0	0	3	0	0	0	0	0	0	0	10	0.0%	0.0%	0.0%	0.0%	0.0%	0.0%	0.0%	69.0%
tr|F1SBZ3|F1SBZ3	0	0	0	0	0	0	0	5	0	0	0	0	0	0	0	10	0.0%	0.0%	0.0%	0.0%	0.0%	0.0%	0.0%	26.0%
tr|K7GQE8|K7GQE8	0	0	0	0	0	0	2	3	0	0	0	0	0	0	5	5	0.0%	0.0%	0.0%	0.0%	0.0%	0.0%	11.5%	17.8%
tr|F1RV21|F1RV21	0	0	0	0	0	0	0	1	0	0	0	0	0	0	0	10	0.0%	0.0%	0.0%	0.0%	0.0%	0.0%	0.0%	2.5%
tr|I3L887|I3L887	0	0	0	0	0	0	1	1	0	0	0	0	0	0	4	6	0.0%	0.0%	0.0%	0.0%	0.0%	0.0%	14.4%	14.4%
tr|I3LU23|I3LU23	0	0	0	0	0	0	1	2	0	0	0	0	0	0	1	9	0.0%	0.0%	0.0%	0.0%	0.0%	0.0%	6.6%	12.5%
tr|F1RKA7|F1RKA7	0	0	0	0	0	0	0	3	0	0	0	0	0	0	0	10	0.0%	0.0%	0.0%	0.0%	0.0%	0.0%	0.0%	5.2%
tr|I3LSM1|I3LSM1	0	0	0	0	0	0	0	2	0	0	0	0	0	0	0	9	0.0%	0.0%	0.0%	0.0%	0.0%	0.0%	0.0%	9.1%
tr|I3LTI5|I3LTI5	0	0	0	0	0	0	2	1	0	0	0	0	0	0	7	2	0.0%	0.0%	0.0%	0.0%	0.0%	0.0%	11.8%	10.4%
tr|K7GRH1|K7GRH1	0	0	0	0	0	0	2	0	0	0	0	0	0	0	9	0	0.0%	0.0%	0.0%	0.0%	0.0%	0.0%	18.0%	0.0%
tr|K7GLY4|K7GLY4	0	0	0	0	0	0	2	2	0	0	0	0	0	0	5	4	0.0%	0.0%	0.0%	0.0%	0.0%	0.0%	12.6%	15.2%
tr|K7GPH3|K7GPH3	0	0	0	0	0	0	2	2	0	0	0	0	0	0	5	4	0.0%	0.0%	0.0%	0.0%	0.0%	0.0%	13.6%	16.4%
tr|F1RFQ6|F1RFQ6	0	0	0	0	0	0	1	1	0	0	0	0	0	0	5	4	0.0%	0.0%	0.0%	0.0%	0.0%	0.0%	10.6%	4.8%
tr|K7GMA7|K7GMA7	0	0	0	0	0	0	1	1	0	0	0	0	0	0	5	4	0.0%	0.0%	0.0%	0.0%	0.0%	0.0%	13.8%	6.2%
tr|A2TEQ2|A2TEQ2	0	0	0	0	0	0	0	3	0	0	0	0	0	0	0	8	0.0%	0.0%	0.0%	0.0%	0.0%	0.0%	0.0%	9.6%
tr|K7GLA5|K7GLA5	0	0	0	0	0	0	0	4	0	0	0	0	0	0	0	8	0.0%	0.0%	0.0%	0.0%	0.0%	0.0%	0.0%	19.8%
tr|F1SCK8|F1SCK8	0	0	0	0	0	0	1	4	0	0	0	0	0	0	2	6	0.0%	0.0%	0.0%	0.0%	0.0%	0.0%	4.1%	12.9%
tr|I3LVA8|I3LVA8	0	0	0	0	0	0	1	2	0	0	0	0	0	0	2	6	0.0%	0.0%	0.0%	0.0%	0.0%	0.0%	11.8%	11.8%
tr|I3L6R5|I3L6R5	0	0	0	0	0	0	0	4	0	0	0	0	0	0	0	8	0.0%	0.0%	0.0%	0.0%	0.0%	0.0%	0.0%	23.7%
tr|I3LBA8|I3LBA8	0	0	0	0	0	0	1	1	0	0	0	0	0	0	7	1	0.0%	0.0%	0.0%	0.0%	0.0%	0.0%	11.0%	11.0%
tr|F1SR79|F1SR79	0	0	0	0	0	0	0	2	0	0	0	0	0	0	0	7	0.0%	0.0%	0.0%	0.0%	0.0%	0.0%	0.0%	7.7%
tr|F1RMJ6|F1RMJ6	0	0	0	0	0	0	0	4	0	0	0	0	0	0	0	7	0.0%	0.0%	0.0%	0.0%	0.0%	0.0%	0.0%	9.2%
tr|K7GMY7|K7GMY7	0	0	0	0	0	0	0	3	0	0	0	0	0	0	0	7	0.0%	0.0%	0.0%	0.0%	0.0%	0.0%	0.0%	6.7%
tr|F1S092|F1S092	0	0	0	0	0	0	0	1	0	0	0	0	0	0	0	7	0.0%	0.0%	0.0%	0.0%	0.0%	0.0%	0.0%	9.9%
tr|F1S025|F1S025	0	0	0	0	0	0	0	1	0	0	0	0	0	0	0	7	0.0%	0.0%	0.0%	0.0%	0.0%	0.0%	0.0%	1.4%
tr|F1SUC8|F1SUC8	0	0	0	0	0	0	1	1	0	0	0	0	0	0	6	1	0.0%	0.0%	0.0%	0.0%	0.0%	0.0%	25.8%	25.8%
tr|I3LAB9|I3LAB9	0	0	0	0	0	0	0	2	0	0	0	0	0	0	0	7	0.0%	0.0%	0.0%	0.0%	0.0%	0.0%	0.0%	3.3%
tr|F1RI93|F1RI93	0	0	0	0	0	0	0	4	0	0	0	0	0	0	0	7	0.0%	0.0%	0.0%	0.0%	0.0%	0.0%	0.0%	9.2%
tr|F1RRT7|F1RRT7	0	0	0	0	0	0	1	2	0	0	0	0	0	0	1	6	0.0%	0.0%	0.0%	0.0%	0.0%	0.0%	6.9%	3.7%
Q1RPR6	0	0	0	0	0	0	0	2	0	0	0	0	0	0	0	7	0.0%	0.0%	0.0%	0.0%	0.0%	0.0%	0.0%	5.1%
tr|F1SFC0|F1SFC0	0	0	0	0	0	0	0	2	0	0	0	0	0	0	0	7	0.0%	0.0%	0.0%	0.0%	0.0%	0.0%	0.0%	33.9%
tr|I3LRI6|I3LRI6	0	0	0	0	0	0	0	2	0	0	0	0	0	0	0	7	0.0%	0.0%	0.0%	0.0%	0.0%	0.0%	0.0%	29.5%
tr|I3LTJ5|I3LTJ5	0	0	0	0	0	0	0	1	0	0	0	0	0	0	0	6	0.0%	0.0%	0.0%	0.0%	0.0%	0.0%	0.0%	4.3%
tr|F1S6Q8|F1S6Q8	0	0	0	0	0	0	0	1	0	0	0	0	0	0	0	6	0.0%	0.0%	0.0%	0.0%	0.0%	0.0%	0.0%	2.5%
tr|I3LGN6|I3LGN6	0	0	0	0	0	0	2	0	0	0	0	0	0	0	6	0	0.0%	0.0%	0.0%	0.0%	0.0%	0.0%	21.4%	0.0%
tr|K7GML7|K7GML7	0	0	0	0	0	0	0	2	0	0	0	0	0	0	0	6	0.0%	0.0%	0.0%	0.0%	0.0%	0.0%	0.0%	6.0%
tr|I3L5J8|I3L5J8	0	0	0	0	0	0	0	1	0	0	0	0	0	0	0	6	0.0%	0.0%	0.0%	0.0%	0.0%	0.0%	0.0%	2.3%
tr|K7GNE0|K7GNE0	0	0	0	0	0	0	0	1	0	0	0	0	0	0	0	6	0.0%	0.0%	0.0%	0.0%	0.0%	0.0%	0.0%	2.0%
tr|I3LSY4|I3LSY4	0	0	0	0	0	0	0	1	0	0	0	0	0	0	0	6	0.0%	0.0%	0.0%	0.0%	0.0%	0.0%	0.0%	8.3%
tr|K7GQ78|K7GQ78	0	0	0	0	0	0	0	1	0	0	0	0	0	0	0	6	0.0%	0.0%	0.0%	0.0%	0.0%	0.0%	0.0%	3.8%
tr|F1SII2|F1SII2	0	0	0	0	0	0	0	1	0	0	0	0	0	0	0	6	0.0%	0.0%	0.0%	0.0%	0.0%	0.0%	0.0%	3.8%
tr|K7GKL8|K7GKL8	0	0	0	0	0	0	0	1	0	0	0	0	0	0	0	6	0.0%	0.0%	0.0%	0.0%	0.0%	0.0%	0.0%	4.4%
tr|I3LQM7|I3LQM7	0	0	0	0	0	0	0	1	0	0	0	0	0	0	0	6	0.0%	0.0%	0.0%	0.0%	0.0%	0.0%	0.0%	2.8%
tr|F1SSJ3|F1SSJ3	0	0	0	0	0	0	1	3	0	0	0	0	0	0	1	5	0.0%	0.0%	0.0%	0.0%	0.0%	0.0%	1.8%	5.9%
tr|K7GNQ6|K7GNQ6	0	0	0	0	0	0	1	2	0	0	0	0	0	0	3	3	0.0%	0.0%	0.0%	0.0%	0.0%	0.0%	8.9%	23.0%
P32307	0	0	0	0	0	0	0	2	0	0	0	0	0	0	0	6	0.0%	0.0%	0.0%	0.0%	0.0%	0.0%	0.0%	9.2%
tr|I3L9U2|I3L9U2	0	0	0	0	0	0	1	0	0	0	0	0	0	0	6	0	0.0%	0.0%	0.0%	0.0%	0.0%	0.0%	14.2%	0.0%

	Peptide Count								Spectral Count								Sequence Coverage							
	Whole Cell	Whole Cell	Whole Cell	Cilia Fraction	Cilia Fraction	Cilia Fraction	Cilia Fraction	Cilia Fraction	Whole Cell	Whole Cell	Whole Cell	Cilia Fraction	Cilia Fraction	Cilia Fraction	Cilia Fraction	Cilia Fraction	Whole Cell	Whole Cell	Whole Cell	Cilia Fraction	Cilia Fraction	Cilia Fraction	Cilia Fraction	Cilia Fraction
Sample#	1	2	6	4	3	5	7	8	1	2	6	4	3	5	7	8	1	2	6	4	3	5	7	8
**Accession**																								
tr|F1S8B6|F1S8B6	0	0	0	0	0	0	0	1	0	0	0	0	0	0	0	6	0.0%	0.0%	0.0%	0.0%	0.0%	0.0%	0.0%	1.8%
tr|I3LPC6|I3LPC6	0	0	0	0	0	0	1	3	0	0	0	0	0	0	1	5	0.0%	0.0%	0.0%	0.0%	0.0%	0.0%	2.0%	4.3%
tr|I3LP31|I3LP31	0	0	0	0	0	0	0	2	0	0	0	0	0	0	0	6	0.0%	0.0%	0.0%	0.0%	0.0%	0.0%	0.0%	25.0%
tr|F1SM80|F1SM80	0	0	0	0	0	0	0	1	0	0	0	0	0	0	0	6	0.0%	0.0%	0.0%	0.0%	0.0%	0.0%	0.0%	8.2%
tr|F1SFS5|F1SFS5	0	0	0	0	0	0	0	3	0	0	0	0	0	0	0	5	0.0%	0.0%	0.0%	0.0%	0.0%	0.0%	0.0%	7.0%
tr|F1RHJ5|F1RHJ5	0	0	0	0	0	0	1	0	0	0	0	0	0	0	5	0	0.0%	0.0%	0.0%	0.0%	0.0%	0.0%	1.5%	0.0%
Q28969	0	0	0	0	0	0	1	2	0	0	0	0	0	0	1	4	0.0%	0.0%	0.0%	0.0%	0.0%	0.0%	1.7%	3.6%
tr|I3L7H8|I3L7H8	0	0	0	0	0	0	0	2	0	0	0	0	0	0	0	5	0.0%	0.0%	0.0%	0.0%	0.0%	0.0%	0.0%	7.3%
tr|F1S2V8|F1S2V8	0	0	0	0	0	0	1	1	0	0	0	0	0	0	2	3	0.0%	0.0%	0.0%	0.0%	0.0%	0.0%	5.7%	5.7%
tr|K7GKP9|K7GKP9	0	0	0	0	0	0	0	2	0	0	0	0	0	0	0	5	0.0%	0.0%	0.0%	0.0%	0.0%	0.0%	0.0%	4.4%
tr|I3LBD5|I3LBD5	0	0	0	0	0	0	0	1	0	0	0	0	0	0	0	5	0.0%	0.0%	0.0%	0.0%	0.0%	0.0%	0.0%	3.8%
tr|I3LLG6|I3LLG6	0	0	0	0	0	0	0	1	0	0	0	0	0	0	0	5	0.0%	0.0%	0.0%	0.0%	0.0%	0.0%	0.0%	2.5%
tr|F1RLP4|F1RLP4	0	0	0	0	0	0	0	1	0	0	0	0	0	0	0	5	0.0%	0.0%	0.0%	0.0%	0.0%	0.0%	0.0%	8.1%
tr|F1S3P0|F1S3P0	0	0	0	0	0	0	0	1	0	0	0	0	0	0	0	5	0.0%	0.0%	0.0%	0.0%	0.0%	0.0%	0.0%	8.3%
tr|I3LH68|I3LH68	0	0	0	0	0	0	1	1	0	0	0	0	0	0	2	3	0.0%	0.0%	0.0%	0.0%	0.0%	0.0%	10.1%	10.1%
Q8WNR0	0	0	0	0	0	0	1	1	0	0	0	0	0	0	2	3	0.0%	0.0%	0.0%	0.0%	0.0%	0.0%	10.1%	10.1%
tr|F1SI16|F1SI16	0	0	0	0	0	0	0	2	0	0	0	0	0	0	0	5	0.0%	0.0%	0.0%	0.0%	0.0%	0.0%	0.0%	4.3%
tr|A3KDL8|A3KDL8	0	0	0	0	0	0	0	1	0	0	0	0	0	0	0	5	0.0%	0.0%	0.0%	0.0%	0.0%	0.0%	0.0%	18.1%
tr|F1RZV2|F1RZV2	0	0	0	0	0	0	1	1	0	0	0	0	0	0	2	3	0.0%	0.0%	0.0%	0.0%	0.0%	0.0%	7.2%	5.9%
tr|F1SCI1|F1SCI1	0	0	0	0	0	0	0	2	0	0	0	0	0	0	0	5	0.0%	0.0%	0.0%	0.0%	0.0%	0.0%	0.0%	4.2%
tr|F1SFW7|F1SFW7	0	0	0	0	0	0	0	2	0	0	0	0	0	0	0	5	0.0%	0.0%	0.0%	0.0%	0.0%	0.0%	0.0%	7.3%
tr|I3LHZ5|I3LHZ5	0	0	0	0	0	0	0	2	0	0	0	0	0	0	0	5	0.0%	0.0%	0.0%	0.0%	0.0%	0.0%	0.0%	27.2%
tr|I3LR16|I3LR16	0	0	0	0	0	0	1	1	0	0	0	0	0	0	2	3	0.0%	0.0%	0.0%	0.0%	0.0%	0.0%	12.5%	12.5%
tr|I3LAB8|I3LAB8	0	0	0	0	0	0	0	2	0	0	0	0	0	0	0	5	0.0%	0.0%	0.0%	0.0%	0.0%	0.0%	0.0%	7.0%
tr|F1S972|F1S972	0	0	0	0	0	0	0	1	0	0	0	0	0	0	0	4	0.0%	0.0%	0.0%	0.0%	0.0%	0.0%	0.0%	19.9%
Q2LE37	0	0	0	0	0	0	1	0	0	0	0	0	0	0	4	0	0.0%	0.0%	0.0%	0.0%	0.0%	0.0%	8.0%	0.0%
tr|I3LSS2|I3LSS2	0	0	0	0	0	0	1	0	0	0	0	0	0	0	4	0	0.0%	0.0%	0.0%	0.0%	0.0%	0.0%	10.8%	0.0%
tr|I3LGT1|I3LGT1	0	0	0	0	0	0	0	2	0	0	0	0	0	0	0	4	0.0%	0.0%	0.0%	0.0%	0.0%	0.0%	0.0%	45.0%
tr|F1S3P3|F1S3P3	0	0	0	0	0	0	1	2	0	0	0	0	0	0	2	2	0.0%	0.0%	0.0%	0.0%	0.0%	0.0%	15.2%	15.2%
tr|I3LRQ5|I3LRQ5	0	0	0	0	0	0	0	2	0	0	0	0	0	0	0	4	0.0%	0.0%	0.0%	0.0%	0.0%	0.0%	0.0%	5.8%
tr|F1SPZ5|F1SPZ5	0	0	0	0	0	0	0	1	0	0	0	0	0	0	0	4	0.0%	0.0%	0.0%	0.0%	0.0%	0.0%	0.0%	6.3%
tr|F6Q402|F6Q402	0	0	0	0	0	0	1	0	0	0	0	0	0	0	4	0	0.0%	0.0%	0.0%	0.0%	0.0%	0.0%	2.2%	0.0%
tr|I3LBQ1|I3LBQ1	0	0	0	0	0	0	0	1	0	0	0	0	0	0	0	4	0.0%	0.0%	0.0%	0.0%	0.0%	0.0%	0.0%	40.3%
tr|K7GQV4|K7GQV4	0	0	0	0	0	0	0	2	0	0	0	0	0	0	0	4	0.0%	0.0%	0.0%	0.0%	0.0%	0.0%	0.0%	12.3%
tr|K7GPH5|K7GPH5	0	0	0	0	0	0	1	1	0	0	0	0	0	0	3	1	0.0%	0.0%	0.0%	0.0%	0.0%	0.0%	8.0%	4.0%
tr|K7GR16|K7GR16	0	0	0	0	0	0	0	3	0	0	0	0	0	0	0	4	0.0%	0.0%	0.0%	0.0%	0.0%	0.0%	0.0%	13.1%
tr|F1STQ5|F1STQ5	0	0	0	0	0	0	0	2	0	0	0	0	0	0	0	4	0.0%	0.0%	0.0%	0.0%	0.0%	0.0%	0.0%	13.6%
tr|F1SEI8|F1SEI8	0	0	0	0	0	0	0	1	0	0	0	0	0	0	0	4	0.0%	0.0%	0.0%	0.0%	0.0%	0.0%	0.0%	6.7%
tr|I3LNV1|I3LNV1	0	0	0	0	0	0	1	1	0	0	0	0	0	0	1	3	0.0%	0.0%	0.0%	0.0%	0.0%	0.0%	33.0%	33.0%
tr|F1SR87|F1SR87	0	0	0	0	0	0	0	1	0	0	0	0	0	0	0	4	0.0%	0.0%	0.0%	0.0%	0.0%	0.0%	0.0%	13.8%
tr|K7GKW2|K7GKW2	0	0	0	0	0	0	0	2	0	0	0	0	0	0	0	4	0.0%	0.0%	0.0%	0.0%	0.0%	0.0%	0.0%	18.1%
tr|K7GSC1|K7GSC1	0	0	0	0	0	0	0	2	0	0	0	0	0	0	0	4	0.0%	0.0%	0.0%	0.0%	0.0%	0.0%	0.0%	7.1%
P49927	0	0	0	0	0	0	0	2	0	0	0	0	0	0	0	4	0.0%	0.0%	0.0%	0.0%	0.0%	0.0%	0.0%	4.7%
tr|F1SIQ5|F1SIQ5	0	0	0	0	0	0	0	1	0	0	0	0	0	0	0	4	0.0%	0.0%	0.0%	0.0%	0.0%	0.0%	0.0%	5.6%
tr|F1SL39|F1SL39	0	0	0	0	0	0	0	1	0	0	0	0	0	0	0	4	0.0%	0.0%	0.0%	0.0%	0.0%	0.0%	0.0%	6.1%
tr|I3LUT9|I3LUT9	0	0	0	0	0	0	0	1	0	0	0	0	0	0	0	4	0.0%	0.0%	0.0%	0.0%	0.0%	0.0%	0.0%	5.3%
tr|I3LJX1|I3LJX1	0	0	0	0	0	0	0	3	0	0	0	0	0	0	0	4	0.0%	0.0%	0.0%	0.0%	0.0%	0.0%	0.0%	11.1%
tr|I3LJX4|I3LJX4	0	0	0	0	0	0	0	2	0	0	0	0	0	0	0	4	0.0%	0.0%	0.0%	0.0%	0.0%	0.0%	0.0%	6.5%
tr|F1SDR9|F1SDR9	0	0	0	0	0	0	1	1	0	0	0	0	0	0	3	1	0.0%	0.0%	0.0%	0.0%	0.0%	0.0%	6.6%	6.6%
tr|I3LPS9|I3LPS9	0	0	0	0	0	0	0	1	0	0	0	0	0	0	0	4	0.0%	0.0%	0.0%	0.0%	0.0%	0.0%	0.0%	11.5%
tr|K7GME2|K7GME2	0	0	0	0	0	0	0	2	0	0	0	0	0	0	0	4	0.0%	0.0%	0.0%	0.0%	0.0%	0.0%	0.0%	9.6%
tr|K7GSR0|K7GSR0	0	0	0	0	0	0	1	0	0	0	0	0	0	0	4	0	0.0%	0.0%	0.0%	0.0%	0.0%	0.0%	17.7%	0.0%
tr|I3LI95|I3LI95	0	0	0	0	0	0	1	1	0	0	0	0	0	0	2	2	0.0%	0.0%	0.0%	0.0%	0.0%	0.0%	1.1%	1.4%
Reverse_tr|F1SIL5|F1SIL5	0	0	0	0	0	0	1	0	0	0	0	0	0	0	4	0	0.0%	0.0%	0.0%	0.0%	0.0%	0.0%	0.5%	0.0%
tr|K7GRN0|K7GRN0	0	0	0	0	0	0	0	1	0	0	0	0	0	0	0	4	0.0%	0.0%	0.0%	0.0%	0.0%	0.0%	0.0%	12.5%
tr|K7GKZ5|K7GKZ5	0	0	0	0	0	0	1	0	0	0	0	0	0	0	4	0	0.0%	0.0%	0.0%	0.0%	0.0%	0.0%	16.8%	0.0%
tr|K7GQH7|K7GQH7	0	0	0	0	0	0	0	2	0	0	0	0	0	0	0	4	0.0%	0.0%	0.0%	0.0%	0.0%	0.0%	0.0%	10.2%
tr|F1RFD6|F1RFD6	0	0	0	0	0	0	1	3	0	0	0	0	0	0	1	3	0.0%	0.0%	0.0%	0.0%	0.0%	0.0%	4.1%	12.2%
tr|F1SCU2|F1SCU2	0	0	0	0	0	0	1	1	0	0	0	0	0	0	2	2	0.0%	0.0%	0.0%	0.0%	0.0%	0.0%	1.1%	1.4%
A2BD05	0	0	0	0	0	0	2	0	0	0	0	0	0	0	4	0	0.0%	0.0%	0.0%	0.0%	0.0%	0.0%	7.7%	0.0%
tr|F1S1M4|F1S1M4	0	0	0	0	0	0	0	2	0	0	0	0	0	0	0	4	0.0%	0.0%	0.0%	0.0%	0.0%	0.0%	0.0%	6.9%
tr|A5D9L6|A5D9L6	0	0	0	0	0	0	1	0	0	0	0	0	0	0	4	0	0.0%	0.0%	0.0%	0.0%	0.0%	0.0%	12.9%	0.0%
tr|K7GQA6|K7GQA6	0	0	0	0	0	0	1	0	0	0	0	0	0	0	4	0	0.0%	0.0%	0.0%	0.0%	0.0%	0.0%	17.9%	0.0%
tr|F1S3P5|F1S3P5	0	0	0	0	0	0	0	1	0	0	0	0	0	0	0	4	0.0%	0.0%	0.0%	0.0%	0.0%	0.0%	0.0%	2.8%
tr|I3LT73|I3LT73	0	0	0	0	0	0	0	2	0	0	0	0	0	0	0	4	0.0%	0.0%	0.0%	0.0%	0.0%	0.0%	0.0%	6.9%
tr|I3LRD7|I3LRD7	0	0	0	0	0	0	1	1	0	0	0	0	0	0	1	3	0.0%	0.0%	0.0%	0.0%	0.0%	0.0%	31.4%	31.4%
tr|I3L969|I3L969	0	0	0	0	0	0	0	2	0	0	0	0	0	0	0	3	0.0%	0.0%	0.0%	0.0%	0.0%	0.0%	0.0%	8.3%
tr|F1SDT5|F1SDT5	0	0	0	0	0	0	1	1	0	0	0	0	0	0	2	1	0.0%	0.0%	0.0%	0.0%	0.0%	0.0%	4.5%	5.0%
tr|F1SCY7|F1SCY7	0	0	0	0	0	0	0	1	0	0	0	0	0	0	0	3	0.0%	0.0%	0.0%	0.0%	0.0%	0.0%	0.0%	4.2%
tr|F1SB78|F1SB78	0	0	0	0	0	0	0	1	0	0	0	0	0	0	0	3	0.0%	0.0%	0.0%	0.0%	0.0%	0.0%	0.0%	2.2%
tr|K7GRY2|K7GRY2	0	0	0	0	0	0	0	1	0	0	0	0	0	0	0	3	0.0%	0.0%	0.0%	0.0%	0.0%	0.0%	0.0%	16.2%
tr|F1RVX0|F1RVX0	0	0	0	0	0	0	0	1	0	0	0	0	0	0	0	3	0.0%	0.0%	0.0%	0.0%	0.0%	0.0%	0.0%	1.7%
tr|I3LD75|I3LD75	0	0	0	0	0	0	0	1	0	0	0	0	0	0	0	3	0.0%	0.0%	0.0%	0.0%	0.0%	0.0%	0.0%	5.4%
tr|F1SA40|F1SA40	0	0	0	0	0	0	0	2	0	0	0	0	0	0	0	3	0.0%	0.0%	0.0%	0.0%	0.0%	0.0%	0.0%	15.8%
tr|K7GKL1|K7GKL1	0	0	0	0	0	0	0	2	0	0	0	0	0	0	0	3	0.0%	0.0%	0.0%	0.0%	0.0%	0.0%	0.0%	51.3%
tr|F1RGV1|F1RGV1	0	0	0	0	0	0	0	1	0	0	0	0	0	0	0	3	0.0%	0.0%	0.0%	0.0%	0.0%	0.0%	0.0%	5.7%
tr|K7GSE1|K7GSE1	0	0	0	0	0	0	0	1	0	0	0	0	0	0	0	3	0.0%	0.0%	0.0%	0.0%	0.0%	0.0%	0.0%	6.9%
tr|F1RPR7|F1RPR7	0	0	0	0	0	0	0	1	0	0	0	0	0	0	0	3	0.0%	0.0%	0.0%	0.0%	0.0%	0.0%	0.0%	8.2%
tr|F1RRI4|F1RRI4	0	0	0	0	0	0	0	2	0	0	0	0	0	0	0	3	0.0%	0.0%	0.0%	0.0%	0.0%	0.0%	0.0%	15.6%
tr|I3LSE4|I3LSE4	0	0	0	0	0	0	0	2	0	0	0	0	0	0	0	3	0.0%	0.0%	0.0%	0.0%	0.0%	0.0%	0.0%	15.0%
tr|I3LVP7|I3LVP7	0	0	0	0	0	0	1	2	0	0	0	0	0	0	1	2	0.0%	0.0%	0.0%	0.0%	0.0%	0.0%	4.6%	8.7%

	Peptide Count								Spectral Count								Sequence Coverage							
	Whole Cell	Whole Cell	Whole Cell	Cilia Fraction	Cilia Fraction	Cilia Fraction	Cilia Fraction	Cilia Fraction	Whole Cell	Whole Cell	Whole Cell	Cilia Fraction	Cilia Fraction	Cilia Fraction	Cilia Fraction	Cilia Fraction	Whole Cell	Whole Cell	Whole Cell	Cilia Fraction	Cilia Fraction	Cilia Fraction	Cilia Fraction	Cilia Fraction
Sample#	1	2	6	4	3	5	7	8	1	2	6	4	3	5	7	8	1	2	6	4	3	5	7	8
**Accession**																								
tr|I3LN05|I3LN05	0	0	0	0	0	0	1	1	0	0	0	0	0	0	2	1	0.0%	0.0%	0.0%	0.0%	0.0%	0.0%	4.6%	5.0%
tr|F1SNX3|F1SNX3	0	0	0	0	0	0	1	1	0	0	0	0	0	0	1	2	0.0%	0.0%	0.0%	0.0%	0.0%	0.0%	4.8%	4.8%
tr|I3L7V7|I3L7V7	0	0	0	0	0	0	0	1	0	0	0	0	0	0	0	3	0.0%	0.0%	0.0%	0.0%	0.0%	0.0%	0.0%	5.1%
tr|I3LFD2|I3LFD2	0	0	0	0	0	0	0	1	0	0	0	0	0	0	0	3	0.0%	0.0%	0.0%	0.0%	0.0%	0.0%	0.0%	5.4%
tr|I3LAG4|I3LAG4	0	0	0	0	0	0	0	1	0	0	0	0	0	0	0	3	0.0%	0.0%	0.0%	0.0%	0.0%	0.0%	0.0%	4.7%
tr|F1SSI1|F1SSI1	0	0	0	0	0	0	1	1	0	0	0	0	0	0	1	2	0.0%	0.0%	0.0%	0.0%	0.0%	0.0%	4.7%	6.3%
O77736	0	0	0	0	0	0	0	1	0	0	0	0	0	0	0	3	0.0%	0.0%	0.0%	0.0%	0.0%	0.0%	0.0%	4.2%
tr|F1S5A6|F1S5A6	0	0	0	0	0	0	0	2	0	0	0	0	0	0	0	3	0.0%	0.0%	0.0%	0.0%	0.0%	0.0%	0.0%	4.3%
tr|I3LAI6|I3LAI6	0	0	0	0	0	0	0	2	0	0	0	0	0	0	0	3	0.0%	0.0%	0.0%	0.0%	0.0%	0.0%	0.0%	15.5%
tr|F1RLT1|F1RLT1	0	0	0	0	0	0	0	1	0	0	0	0	0	0	0	3	0.0%	0.0%	0.0%	0.0%	0.0%	0.0%	0.0%	2.3%
Q06AU5	0	0	0	0	0	0	0	1	0	0	0	0	0	0	0	3	0.0%	0.0%	0.0%	0.0%	0.0%	0.0%	0.0%	9.3%
Reverse_tr|F1SJZ5|F1SJZ5	0	0	0	0	0	0	1	1	0	0	0	0	0	0	2	1	0.0%	0.0%	0.0%	0.0%	0.0%	0.0%	2.8%	2.8%
tr|F1SQA7|F1SQA7	0	0	0	0	0	0	0	1	0	0	0	0	0	0	0	3	0.0%	0.0%	0.0%	0.0%	0.0%	0.0%	0.0%	4.8%
tr|F1SBY9|F1SBY9	0	0	0	0	0	0	0	2	0	0	0	0	0	0	0	3	0.0%	0.0%	0.0%	0.0%	0.0%	0.0%	0.0%	2.4%
tr|F1ST61|F1ST61	0	0	0	0	0	0	1	1	0	0	0	0	0	0	2	1	0.0%	0.0%	0.0%	0.0%	0.0%	0.0%	4.8%	4.8%
tr|F1RNK7|F1RNK7	0	0	0	0	0	0	0	1	0	0	0	0	0	0	0	3	0.0%	0.0%	0.0%	0.0%	0.0%	0.0%	0.0%	4.9%
tr|F1RSF8|F1RSF8	0	0	0	0	0	0	0	1	0	0	0	0	0	0	0	3	0.0%	0.0%	0.0%	0.0%	0.0%	0.0%	0.0%	7.0%
tr|F1S747|F1S747	0	0	0	0	0	0	0	1	0	0	0	0	0	0	0	3	0.0%	0.0%	0.0%	0.0%	0.0%	0.0%	0.0%	9.3%
tr|F1RKI1|F1RKI1	0	0	0	0	0	0	0	2	0	0	0	0	0	0	0	3	0.0%	0.0%	0.0%	0.0%	0.0%	0.0%	0.0%	44.0%
tr|F1RSP4|F1RSP4	0	0	0	0	0	0	0	1	0	0	0	0	0	0	0	3	0.0%	0.0%	0.0%	0.0%	0.0%	0.0%	0.0%	4.6%
tr|I3LG67|I3LG67	0	0	0	0	0	0	0	2	0	0	0	0	0	0	0	3	0.0%	0.0%	0.0%	0.0%	0.0%	0.0%	0.0%	39.8%
tr|K7GMM7|K7GMM7	0	0	0	0	0	0	0	2	0	0	0	0	0	0	0	3	0.0%	0.0%	0.0%	0.0%	0.0%	0.0%	0.0%	2.6%
tr|F1SUH8|F1SUH8	0	0	0	0	0	0	0	1	0	0	0	0	0	0	0	3	0.0%	0.0%	0.0%	0.0%	0.0%	0.0%	0.0%	11.6%
tr|F1SBZ0|F1SBZ0	0	0	0	0	0	0	0	2	0	0	0	0	0	0	0	3	0.0%	0.0%	0.0%	0.0%	0.0%	0.0%	0.0%	2.9%
tr|F1RSK5|F1RSK5	0	0	0	0	0	0	0	2	0	0	0	0	0	0	0	3	0.0%	0.0%	0.0%	0.0%	0.0%	0.0%	0.0%	2.8%
tr|I3LTY7|I3LTY7	0	0	0	0	0	0	0	1	0	0	0	0	0	0	0	3	0.0%	0.0%	0.0%	0.0%	0.0%	0.0%	0.0%	2.2%
tr|F1SG89|F1SG89	0	0	0	0	0	0	1	0	0	0	0	0	0	0	3	0	0.0%	0.0%	0.0%	0.0%	0.0%	0.0%	1.1%	0.0%
Q863T6	0	0	0	0	0	0	0	1	0	0	0	0	0	0	0	3	0.0%	0.0%	0.0%	0.0%	0.0%	0.0%	0.0%	2.2%
tr|F1RMY4|F1RMY4	0	0	0	0	0	0	0	1	0	0	0	0	0	0	0	3	0.0%	0.0%	0.0%	0.0%	0.0%	0.0%	0.0%	3.7%
tr|I3LG19|I3LG19	0	0	0	0	0	0	0	1	0	0	0	0	0	0	0	2	0.0%	0.0%	0.0%	0.0%	0.0%	0.0%	0.0%	14.9%
tr|I3LPX1|I3LPX1	0	0	0	0	0	0	0	1	0	0	0	0	0	0	0	2	0.0%	0.0%	0.0%	0.0%	0.0%	0.0%	0.0%	9.5%
tr|F1SDA0|F1SDA0	0	0	0	0	0	0	0	1	0	0	0	0	0	0	0	2	0.0%	0.0%	0.0%	0.0%	0.0%	0.0%	0.0%	2.6%
tr|F1SDH5|F1SDH5	0	0	0	0	0	0	0	1	0	0	0	0	0	0	0	2	0.0%	0.0%	0.0%	0.0%	0.0%	0.0%	0.0%	2.2%
tr|F1SDA4|F1SDA4	0	0	0	0	0	0	0	2	0	0	0	0	0	0	0	2	0.0%	0.0%	0.0%	0.0%	0.0%	0.0%	0.0%	7.0%
tr|I3LMQ5|I3LMQ5	0	0	0	0	0	0	0	1	0	0	0	0	0	0	0	2	0.0%	0.0%	0.0%	0.0%	0.0%	0.0%	0.0%	4.1%
tr|K7GMV5|K7GMV5	0	0	0	0	0	0	0	1	0	0	0	0	0	0	0	2	0.0%	0.0%	0.0%	0.0%	0.0%	0.0%	0.0%	20.8%
tr|I3LLG5|I3LLG5	0	0	0	0	0	0	0	1	0	0	0	0	0	0	0	2	0.0%	0.0%	0.0%	0.0%	0.0%	0.0%	0.0%	13.8%
tr|F1S2G7|F1S2G7	0	0	0	0	0	0	0	1	0	0	0	0	0	0	0	2	0.0%	0.0%	0.0%	0.0%	0.0%	0.0%	0.0%	1.5%
tr|I3LBU6|I3LBU6	0	0	0	0	0	0	1	0	0	0	0	0	0	0	2	0	0.0%	0.0%	0.0%	0.0%	0.0%	0.0%	1.9%	0.0%
tr|F1S8E6|F1S8E6	0	0	0	0	0	0	1	1	0	0	0	0	0	0	1	1	0.0%	0.0%	0.0%	0.0%	0.0%	0.0%	7.6%	7.6%
tr|K7GQ24|K7GQ24	0	0	0	0	0	0	1	0	0	0	0	0	0	0	2	0	0.0%	0.0%	0.0%	0.0%	0.0%	0.0%	2.1%	0.0%
tr|I3LNL1|I3LNL1	0	0	0	0	0	0	1	0	0	0	0	0	0	0	2	0	0.0%	0.0%	0.0%	0.0%	0.0%	0.0%	31.3%	0.0%
tr|K7GN71|K7GN71	0	0	0	0	0	0	0	1	0	0	0	0	0	0	0	2	0.0%	0.0%	0.0%	0.0%	0.0%	0.0%	0.0%	8.7%
tr|I3L801|I3L801	0	0	0	0	0	0	0	1	0	0	0	0	0	0	0	2	0.0%	0.0%	0.0%	0.0%	0.0%	0.0%	0.0%	2.9%
tr|I3L6P9|I3L6P9	0	0	0	0	0	0	0	1	0	0	0	0	0	0	0	2	0.0%	0.0%	0.0%	0.0%	0.0%	0.0%	0.0%	4.0%
tr|F1SFI8|F1SFI8	0	0	0	0	0	0	0	2	0	0	0	0	0	0	0	2	0.0%	0.0%	0.0%	0.0%	0.0%	0.0%	0.0%	7.7%
tr|C3VMW5|C3VMW5	0	0	0	0	0	0	0	1	0	0	0	0	0	0	0	2	0.0%	0.0%	0.0%	0.0%	0.0%	0.0%	0.0%	7.6%
tr|Q06AT7|Q06AT7	0	0	0	0	0	0	0	2	0	0	0	0	0	0	0	2	0.0%	0.0%	0.0%	0.0%	0.0%	0.0%	0.0%	15.6%
tr|F1SD44|F1SD44	0	0	0	0	0	0	0	1	0	0	0	0	0	0	0	2	0.0%	0.0%	0.0%	0.0%	0.0%	0.0%	0.0%	1.0%
tr|F1RMJ5|F1RMJ5	0	0	0	0	0	0	0	1	0	0	0	0	0	0	0	2	0.0%	0.0%	0.0%	0.0%	0.0%	0.0%	0.0%	1.8%
tr|I3LSE5|I3LSE5	0	0	0	0	0	0	0	1	0	0	0	0	0	0	0	2	0.0%	0.0%	0.0%	0.0%	0.0%	0.0%	0.0%	11.3%
tr|F1RPM9|F1RPM9	0	0	0	0	0	0	0	2	0	0	0	0	0	0	0	2	0.0%	0.0%	0.0%	0.0%	0.0%	0.0%	0.0%	3.3%
tr|F1S212|F1S212	0	0	0	0	0	0	0	1	0	0	0	0	0	0	0	2	0.0%	0.0%	0.0%	0.0%	0.0%	0.0%	0.0%	1.5%
tr|I3LR11|I3LR11	0	0	0	0	0	0	0	1	0	0	0	0	0	0	0	2	0.0%	0.0%	0.0%	0.0%	0.0%	0.0%	0.0%	6.7%
tr|F1RQM0|F1RQM0	0	0	0	0	0	0	0	2	0	0	0	0	0	0	0	2	0.0%	0.0%	0.0%	0.0%	0.0%	0.0%	0.0%	3.8%
tr|F1SM29|F1SM29	0	0	0	0	0	0	0	1	0	0	0	0	0	0	0	2	0.0%	0.0%	0.0%	0.0%	0.0%	0.0%	0.0%	2.4%
tr|F1RVB0|F1RVB0	0	0	0	0	0	0	0	1	0	0	0	0	0	0	0	2	0.0%	0.0%	0.0%	0.0%	0.0%	0.0%	0.0%	4.8%
Reverse_tr|F1S587|F1S587	0	0	0	0	0	0	1	1	0	0	0	0	0	0	1	1	0.0%	0.0%	0.0%	0.0%	0.0%	0.0%	7.6%	7.6%
tr|F1RYI2|F1RYI2	0	0	0	0	0	0	0	1	0	0	0	0	0	0	0	2	0.0%	0.0%	0.0%	0.0%	0.0%	0.0%	0.0%	4.3%
tr|F1SRL4|F1SRL4	0	0	0	0	0	0	1	0	0	0	0	0	0	0	2	0	0.0%	0.0%	0.0%	0.0%	0.0%	0.0%	3.7%	0.0%
tr|F1RHK9|F1RHK9	0	0	0	0	0	0	0	1	0	0	0	0	0	0	0	2	0.0%	0.0%	0.0%	0.0%	0.0%	0.0%	0.0%	0.9%
tr|F1SJG2|F1SJG2	0	0	0	0	0	0	0	1	0	0	0	0	0	0	0	2	0.0%	0.0%	0.0%	0.0%	0.0%	0.0%	0.0%	4.0%
tr|I3LF29|I3LF29	0	0	0	0	0	0	0	1	0	0	0	0	0	0	0	2	0.0%	0.0%	0.0%	0.0%	0.0%	0.0%	0.0%	9.0%
tr|I3LET6|I3LET6	0	0	0	0	0	0	1	1	0	0	0	0	0	0	1	1	0.0%	0.0%	0.0%	0.0%	0.0%	0.0%	8.9%	8.9%
tr|K7GQ65|K7GQ65	0	0	0	0	0	0	0	1	0	0	0	0	0	0	0	2	0.0%	0.0%	0.0%	0.0%	0.0%	0.0%	0.0%	14.8%
tr|I3LL54|I3LL54	0	0	0	0	0	0	0	1	0	0	0	0	0	0	0	2	0.0%	0.0%	0.0%	0.0%	0.0%	0.0%	0.0%	2.7%
Q29095	0	0	0	0	0	0	0	2	0	0	0	0	0	0	0	2	0.0%	0.0%	0.0%	0.0%	0.0%	0.0%	0.0%	9.5%
Reverse_tr|F1SC38|F1SC38	0	0	0	0	0	0	1	0	0	0	0	0	0	0	2	0	0.0%	0.0%	0.0%	0.0%	0.0%	0.0%	7.0%	0.0%
tr|F1RF36|F1RF36	0	0	0	0	0	0	0	2	0	0	0	0	0	0	0	2	0.0%	0.0%	0.0%	0.0%	0.0%	0.0%	0.0%	4.0%
tr|K7GMZ3|K7GMZ3	0	0	0	0	0	0	0	1	0	0	0	0	0	0	0	2	0.0%	0.0%	0.0%	0.0%	0.0%	0.0%	0.0%	2.2%
tr|F1S483|F1S483	0	0	0	0	0	0	0	1	0	0	0	0	0	0	0	2	0.0%	0.0%	0.0%	0.0%	0.0%	0.0%	0.0%	5.9%
Q8WMQ3	0	0	0	0	0	0	0	1	0	0	0	0	0	0	0	2	0.0%	0.0%	0.0%	0.0%	0.0%	0.0%	0.0%	7.1%
tr|F1SAK9|F1SAK9	0	0	0	0	0	0	0	1	0	0	0	0	0	0	0	2	0.0%	0.0%	0.0%	0.0%	0.0%	0.0%	0.0%	10.2%
tr|F1RHD4|F1RHD4	0	0	0	0	0	0	0	1	0	0	0	0	0	0	0	2	0.0%	0.0%	0.0%	0.0%	0.0%	0.0%	0.0%	5.2%
tr|F1S5D3|F1S5D3	0	0	0	0	0	0	1	0	0	0	0	0	0	0	2	0	0.0%	0.0%	0.0%	0.0%	0.0%	0.0%	2.8%	0.0%
tr|I3LGB8|I3LGB8	0	0	0	0	0	0	0	1	0	0	0	0	0	0	0	2	0.0%	0.0%	0.0%	0.0%	0.0%	0.0%	0.0%	5.8%
tr|F1RKN8|F1RKN8	0	0	0	0	0	0	0	1	0	0	0	0	0	0	0	2	0.0%	0.0%	0.0%	0.0%	0.0%	0.0%	0.0%	2.0%
Reverse_tr|F1RN91|F1RN91	0	0	0	0	0	0	1	0	0	0	0	0	0	0	2	0	0.0%	0.0%	0.0%	0.0%	0.0%	0.0%	1.9%	0.0%
tr|F1RPC8|F1RPC8	0	0	0	0	0	0	1	0	0	0	0	0	0	0	2	0	0.0%	0.0%	0.0%	0.0%	0.0%	0.0%	6.7%	0.0%
tr|I3L7J8|I3L7J8	0	0	0	0	0	0	0	1	0	0	0	0	0	0	0	2	0.0%	0.0%	0.0%	0.0%	0.0%	0.0%	0.0%	5.4%
tr|I3LK93|I3LK93	0	0	0	0	0	0	0	1	0	0	0	0	0	0	0	2	0.0%	0.0%	0.0%	0.0%	0.0%	0.0%	0.0%	12.0%

	Peptide Count								Spectral Count								Sequence Coverage							
	Whole Cell	Whole Cell	Whole Cell	Cilia Fraction	Cilia Fraction	Cilia Fraction	Cilia Fraction	Cilia Fraction	Whole Cell	Whole Cell	Whole Cell	Cilia Fraction	Cilia Fraction	Cilia Fraction	Cilia Fraction	Cilia Fraction	Whole Cell	Whole Cell	Whole Cell	Cilia Fraction	Cilia Fraction	Cilia Fraction	Cilia Fraction	Cilia Fraction
Sample#	1	2	6	4	3	5	7	8	1	2	6	4	3	5	7	8	1	2	6	4	3	5	7	8
**Accession**																								
tr|K7GNW4|K7GNW4	0	0	0	0	0	0	0	1	0	0	0	0	0	0	0	2	0.0%	0.0%	0.0%	0.0%	0.0%	0.0%	0.0%	5.7%
tr|A5GFS1|A5GFS1	0	0	0	0	0	0	1	0	0	0	0	0	0	0	2	0	0.0%	0.0%	0.0%	0.0%	0.0%	0.0%	2.0%	0.0%
tr|K7GQ80|K7GQ80	0	0	0	0	0	0	1	0	0	0	0	0	0	0	2	0	0.0%	0.0%	0.0%	0.0%	0.0%	0.0%	2.4%	0.0%
tr|F1RNN4|F1RNN4	0	0	0	0	0	0	0	1	0	0	0	0	0	0	0	2	0.0%	0.0%	0.0%	0.0%	0.0%	0.0%	0.0%	1.8%
tr|F1SS31|F1SS31	0	0	0	0	0	0	0	1	0	0	0	0	0	0	0	2	0.0%	0.0%	0.0%	0.0%	0.0%	0.0%	0.0%	7.5%
tr|K7GLV5|K7GLV5	0	0	0	0	0	0	0	2	0	0	0	0	0	0	0	2	0.0%	0.0%	0.0%	0.0%	0.0%	0.0%	0.0%	15.2%
Reverse_tr|I3LMK7|I3LMK7	0	0	0	0	0	0	1	0	0	0	0	0	0	0	2	0	0.0%	0.0%	0.0%	0.0%	0.0%	0.0%	1.5%	0.0%
tr|I3LMY2|I3LMY2	0	0	0	0	0	0	0	1	0	0	0	0	0	0	0	2	0.0%	0.0%	0.0%	0.0%	0.0%	0.0%	0.0%	3.6%
tr|A5GFU8|A5GFU8	0	0	0	0	0	0	0	1	0	0	0	0	0	0	0	2	0.0%	0.0%	0.0%	0.0%	0.0%	0.0%	0.0%	21.3%
tr|I3LE68|I3LE68	0	0	0	0	0	0	0	1	0	0	0	0	0	0	0	2	0.0%	0.0%	0.0%	0.0%	0.0%	0.0%	0.0%	13.0%
tr|G9BWQ2|G9BWQ2	0	0	0	0	0	0	0	1	0	0	0	0	0	0	0	2	0.0%	0.0%	0.0%	0.0%	0.0%	0.0%	0.0%	5.2%
tr|I3LJL0|I3LJL0	0	0	0	0	0	0	0	1	0	0	0	0	0	0	0	1	0.0%	0.0%	0.0%	0.0%	0.0%	0.0%	0.0%	4.0%
Reverse_tr|F1SFE3|F1SFE3	0	0	0	0	0	0	1	0	0	0	0	0	0	0	1	0	0.0%	0.0%	0.0%	0.0%	0.0%	0.0%	4.2%	0.0%
tr|I3LVB5|I3LVB5	0	0	0	0	0	0	1	0	0	0	0	0	0	0	1	0	0.0%	0.0%	0.0%	0.0%	0.0%	0.0%	34.9%	0.0%
Reverse_tr|F1S9I3|F1S9I3	0	0	0	0	0	0	0	1	0	0	0	0	0	0	0	1	0.0%	0.0%	0.0%	0.0%	0.0%	0.0%	0.0%	5.3%
tr|I3LCD5|I3LCD5	0	0	0	0	0	0	0	1	0	0	0	0	0	0	0	1	0.0%	0.0%	0.0%	0.0%	0.0%	0.0%	0.0%	7.3%
tr|F1RQZ7|F1RQZ7	0	0	0	0	0	0	0	1	0	0	0	0	0	0	0	1	0.0%	0.0%	0.0%	0.0%	0.0%	0.0%	0.0%	4.0%
tr|F1RQY9|F1RQY9	0	0	0	0	0	0	0	1	0	0	0	0	0	0	0	1	0.0%	0.0%	0.0%	0.0%	0.0%	0.0%	0.0%	2.7%
tr|F1RFL2|F1RFL2	0	0	0	0	0	0	0	1	0	0	0	0	0	0	0	1	0.0%	0.0%	0.0%	0.0%	0.0%	0.0%	0.0%	1.1%
tr|F1SFK6|F1SFK6	0	0	0	0	0	0	0	1	0	0	0	0	0	0	0	1	0.0%	0.0%	0.0%	0.0%	0.0%	0.0%	0.0%	3.8%
tr|I3LEL8|I3LEL8	0	0	0	0	0	0	1	0	0	0	0	0	0	0	1	0	0.0%	0.0%	0.0%	0.0%	0.0%	0.0%	3.5%	0.0%
Reverse_tr|F1RQ00|F1RQ00	0	0	0	0	0	0	0	1	0	0	0	0	0	0	0	1	0.0%	0.0%	0.0%	0.0%	0.0%	0.0%	0.0%	0.9%
tr|K7GME9|K7GME9	0	0	0	0	0	0	0	1	0	0	0	0	0	0	0	1	0.0%	0.0%	0.0%	0.0%	0.0%	0.0%	0.0%	2.7%
tr|F1SF35|F1SF35	0	0	0	0	0	0	0	1	0	0	0	0	0	0	0	1	0.0%	0.0%	0.0%	0.0%	0.0%	0.0%	0.0%	12.7%
tr|A5A4L7|A5A4L7	0	0	0	0	0	0	0	1	0	0	0	0	0	0	0	1	0.0%	0.0%	0.0%	0.0%	0.0%	0.0%	0.0%	1.9%
tr|A7XP20|A7XP20	0	0	0	0	0	0	0	1	0	0	0	0	0	0	0	1	0.0%	0.0%	0.0%	0.0%	0.0%	0.0%	0.0%	14.2%
tr|F1SPN8|F1SPN8	0	0	0	0	0	0	0	1	0	0	0	0	0	0	0	1	0.0%	0.0%	0.0%	0.0%	0.0%	0.0%	0.0%	4.4%
tr|I3L9A3|I3L9A3	0	0	0	0	0	0	1	0	0	0	0	0	0	0	1	0	0.0%	0.0%	0.0%	0.0%	0.0%	0.0%	6.0%	0.0%
Reverse_tr|F1SV22|F1SV22	0	0	0	0	0	0	1	0	0	0	0	0	0	0	1	0	0.0%	0.0%	0.0%	0.0%	0.0%	0.0%	0.5%	0.0%
tr|I3L8D8|I3L8D8	0	0	0	0	0	0	0	1	0	0	0	0	0	0	0	1	0.0%	0.0%	0.0%	0.0%	0.0%	0.0%	0.0%	10.1%
tr|I3LTT7|I3LTT7	0	0	0	0	0	0	0	1	0	0	0	0	0	0	0	1	0.0%	0.0%	0.0%	0.0%	0.0%	0.0%	0.0%	5.3%
tr|K7GKN8|K7GKN8	0	0	0	0	0	0	0	1	0	0	0	0	0	0	0	1	0.0%	0.0%	0.0%	0.0%	0.0%	0.0%	0.0%	4.7%
tr|F1S3J6|F1S3J6	0	0	0	0	0	0	0	1	0	0	0	0	0	0	0	1	0.0%	0.0%	0.0%	0.0%	0.0%	0.0%	0.0%	4.2%
tr|F1RHA2|F1RHA2	0	0	0	0	0	0	1	0	0	0	0	0	0	0	1	0	0.0%	0.0%	0.0%	0.0%	0.0%	0.0%	3.7%	0.0%
tr|I3LR87|I3LR87	0	0	0	0	0	0	1	0	0	0	0	0	0	0	1	0	0.0%	0.0%	0.0%	0.0%	0.0%	0.0%	8.8%	0.0%
tr|F1RK47|F1RK47	0	0	0	0	0	0	0	1	0	0	0	0	0	0	0	1	0.0%	0.0%	0.0%	0.0%	0.0%	0.0%	0.0%	6.1%
tr|I3L9M4|I3L9M4	0	0	0	0	0	0	0	1	0	0	0	0	0	0	0	1	0.0%	0.0%	0.0%	0.0%	0.0%	0.0%	0.0%	24.3%
tr|I3LUW9|I3LUW9	0	0	0	0	0	0	0	1	0	0	0	0	0	0	0	1	0.0%	0.0%	0.0%	0.0%	0.0%	0.0%	0.0%	17.8%
tr|F1SDK6|F1SDK6	0	0	0	0	0	0	0	1	0	0	0	0	0	0	0	1	0.0%	0.0%	0.0%	0.0%	0.0%	0.0%	0.0%	3.5%
Reverse_tr|K7GQ01|K7GQ01	0	0	0	0	0	0	0	1	0	0	0	0	0	0	0	1	0.0%	0.0%	0.0%	0.0%	0.0%	0.0%	0.0%	5.1%
tr|F1S3A2|F1S3A2	0	0	0	0	0	0	1	0	0	0	0	0	0	0	1	0	0.0%	0.0%	0.0%	0.0%	0.0%	0.0%	3.9%	0.0%
tr|F1RK24|F1RK24	0	0	0	0	0	0	0	1	0	0	0	0	0	0	0	1	0.0%	0.0%	0.0%	0.0%	0.0%	0.0%	0.0%	8.7%
tr|F1RUR3|F1RUR3	0	0	0	0	0	0	0	1	0	0	0	0	0	0	0	1	0.0%	0.0%	0.0%	0.0%	0.0%	0.0%	0.0%	5.2%
tr|F1RL24|F1RL24	0	0	0	0	0	0	0	1	0	0	0	0	0	0	0	1	0.0%	0.0%	0.0%	0.0%	0.0%	0.0%	0.0%	3.6%
tr|I3LAC2|I3LAC2	0	0	0	0	0	0	0	1	0	0	0	0	0	0	0	1	0.0%	0.0%	0.0%	0.0%	0.0%	0.0%	0.0%	18.3%
tr|I3LFC8|I3LFC8	0	0	0	0	0	0	1	0	0	0	0	0	0	0	1	0	0.0%	0.0%	0.0%	0.0%	0.0%	0.0%	10.0%	0.0%
tr|I3LRV8|I3LRV8	0	0	0	0	0	0	1	0	0	0	0	0	0	0	1	0	0.0%	0.0%	0.0%	0.0%	0.0%	0.0%	9.1%	0.0%
tr|K7GN08|K7GN08	0	0	0	0	0	0	0	1	0	0	0	0	0	0	0	1	0.0%	0.0%	0.0%	0.0%	0.0%	0.0%	0.0%	1.9%
Reverse_tr|F1SJL5|F1SJL5	0	0	0	0	0	0	0	1	0	0	0	0	0	0	0	1	0.0%	0.0%	0.0%	0.0%	0.0%	0.0%	0.0%	6.5%
tr|I3LAE4|I3LAE4	0	0	0	0	0	0	0	1	0	0	0	0	0	0	0	1	0.0%	0.0%	0.0%	0.0%	0.0%	0.0%	0.0%	6.2%
tr|F1RZK6|F1RZK6	0	0	0	0	0	0	0	1	0	0	0	0	0	0	0	1	0.0%	0.0%	0.0%	0.0%	0.0%	0.0%	0.0%	2.7%
tr|F1SD56|F1SD56	0	0	0	0	0	0	0	1	0	0	0	0	0	0	0	1	0.0%	0.0%	0.0%	0.0%	0.0%	0.0%	0.0%	3.4%
Reverse_tr|F1SS82|F1SS82	0	0	0	0	0	0	0	1	0	0	0	0	0	0	0	1	0.0%	0.0%	0.0%	0.0%	0.0%	0.0%	0.0%	5.1%
tr|I3LLX3|I3LLX3	0	0	0	0	0	0	0	1	0	0	0	0	0	0	0	1	0.0%	0.0%	0.0%	0.0%	0.0%	0.0%	0.0%	2.2%
tr|F1RIS0|F1RIS0	0	0	0	0	0	0	0	1	0	0	0	0	0	0	0	1	0.0%	0.0%	0.0%	0.0%	0.0%	0.0%	0.0%	3.9%
Reverse_tr|I3LET7|I3LET7	0	0	0	0	0	0	1	0	0	0	0	0	0	0	1	0	0.0%	0.0%	0.0%	0.0%	0.0%	0.0%	2.1%	0.0%
tr|K7GQF4|K7GQF4	0	0	0	0	0	0	0	1	0	0	0	0	0	0	0	1	0.0%	0.0%	0.0%	0.0%	0.0%	0.0%	0.0%	2.2%
Reverse_sp|E2IUK4|HORM1	0	0	0	0	0	0	0	1	0	0	0	0	0	0	0	1	0.0%	0.0%	0.0%	0.0%	0.0%	0.0%	0.0%	5.1%
tr|I3LHP7|I3LHP7	0	0	0	0	0	0	1	0	0	0	0	0	0	0	1	0	0.0%	0.0%	0.0%	0.0%	0.0%	0.0%	8.8%	0.0%
tr|F1SRQ0|F1SRQ0	0	0	0	0	0	0	0	1	0	0	0	0	0	0	0	1	0.0%	0.0%	0.0%	0.0%	0.0%	0.0%	0.0%	30.6%
tr|F1SM35|F1SM35	0	0	0	0	0	0	1	0	0	0	0	0	0	0	1	0	0.0%	0.0%	0.0%	0.0%	0.0%	0.0%	1.1%	0.0%
tr|F1SK93|F1SK93	0	0	0	0	0	0	0	1	0	0	0	0	0	0	0	1	0.0%	0.0%	0.0%	0.0%	0.0%	0.0%	0.0%	8.8%
tr|F1SU08|F1SU08	0	0	0	0	0	0	1	0	0	0	0	0	0	0	1	0	0.0%	0.0%	0.0%	0.0%	0.0%	0.0%	4.7%	0.0%
tr|K7GQC7|K7GQC7	0	0	0	0	0	0	0	1	0	0	0	0	0	0	0	1	0.0%	0.0%	0.0%	0.0%	0.0%	0.0%	0.0%	6.7%
tr|F1S293|F1S293	0	0	0	0	0	0	0	1	0	0	0	0	0	0	0	1	0.0%	0.0%	0.0%	0.0%	0.0%	0.0%	0.0%	1.9%
tr|F1RNW3|F1RNW3	0	0	0	0	0	0	0	1	0	0	0	0	0	0	0	1	0.0%	0.0%	0.0%	0.0%	0.0%	0.0%	0.0%	14.2%
tr|F1RPB5|F1RPB5	0	0	0	0	0	0	0	1	0	0	0	0	0	0	0	1	0.0%	0.0%	0.0%	0.0%	0.0%	0.0%	0.0%	7.6%
tr|F1SFU2|F1SFU2	0	0	0	0	0	0	1	0	0	0	0	0	0	0	1	0	0.0%	0.0%	0.0%	0.0%	0.0%	0.0%	9.7%	0.0%
O79876	0	0	0	0	0	0	0	1	0	0	0	0	0	0	0	1	0.0%	0.0%	0.0%	0.0%	0.0%	0.0%	0.0%	6.6%
tr|F1RGE3|F1RGE3	0	0	0	0	0	0	0	1	0	0	0	0	0	0	0	1	0.0%	0.0%	0.0%	0.0%	0.0%	0.0%	0.0%	11.1%
tr|F1S0R5|F1S0R5	0	0	0	0	0	0	0	1	0	0	0	0	0	0	0	1	0.0%	0.0%	0.0%	0.0%	0.0%	0.0%	0.0%	1.7%
tr|K7GST0|K7GST0	0	0	0	0	0	0	0	1	0	0	0	0	0	0	0	1	0.0%	0.0%	0.0%	0.0%	0.0%	0.0%	0.0%	14.3%
tr|I3LBF6|I3LBF6	0	0	0	0	0	0	0	1	0	0	0	0	0	0	0	1	0.0%	0.0%	0.0%	0.0%	0.0%	0.0%	0.0%	3.2%
Reverse_tr|I3LJD3|I3LJD3	0	0	0	0	0	0	0	1	0	0	0	0	0	0	0	1	0.0%	0.0%	0.0%	0.0%	0.0%	0.0%	0.0%	5.5%
tr|F1SV32|F1SV32	0	0	0	0	0	0	0	1	0	0	0	0	0	0	0	1	0.0%	0.0%	0.0%	0.0%	0.0%	0.0%	0.0%	10.9%
tr|F1SAM6|F1SAM6	0	0	0	0	0	0	0	1	0	0	0	0	0	0	0	1	0.0%	0.0%	0.0%	0.0%	0.0%	0.0%	0.0%	13.8%
tr|F1SMT7|F1SMT7	0	0	0	0	0	0	1	0	0	0	0	0	0	0	1	0	0.0%	0.0%	0.0%	0.0%	0.0%	0.0%	4.9%	0.0%
tr|F1SKA2|F1SKA2	0	0	0	0	0	0	0	1	0	0	0	0	0	0	0	1	0.0%	0.0%	0.0%	0.0%	0.0%	0.0%	0.0%	6.2%
tr|F1S0A1|F1S0A1	0	0	0	0	0	0	0	1	0	0	0	0	0	0	0	1	0.0%	0.0%	0.0%	0.0%	0.0%	0.0%	0.0%	3.9%
Reverse_tr|F1S3P8|F1S3P8	0	0	0	0	0	0	1	0	0	0	0	0	0	0	1	0	0.0%	0.0%	0.0%	0.0%	0.0%	0.0%	5.0%	0.0%
tr|F1RQR5|F1RQR5	0	0	0	0	0	0	0	1	0	0	0	0	0	0	0	1	0.0%	0.0%	0.0%	0.0%	0.0%	0.0%	0.0%	2.4%

	Peptide Count								Spectral Count								Sequence Coverage							
	Whole Cell	Whole Cell	Whole Cell	Cilia Fraction	Cilia Fraction	Cilia Fraction	Cilia Fraction	Cilia Fraction	Whole Cell	Whole Cell	Whole Cell	Cilia Fraction	Cilia Fraction	Cilia Fraction	Cilia Fraction	Cilia Fraction	Whole Cell	Whole Cell	Whole Cell	Cilia Fraction	Cilia Fraction	Cilia Fraction	Cilia Fraction	Cilia Fraction
Sample#	1	2	6	4	3	5	7	8	1	2	6	4	3	5	7	8	1	2	6	4	3	5	7	8
**Accession**																								
tr|F1SGW7|F1SGW7	0	0	0	0	0	0	0	1	0	0	0	0	0	0	0	1	0.0%	0.0%	0.0%	0.0%	0.0%	0.0%	0.0%	3.3%
Reverse_tr|F1SE65|F1SE65	0	0	0	0	0	0	0	1	0	0	0	0	0	0	0	1	0.0%	0.0%	0.0%	0.0%	0.0%	0.0%	0.0%	3.3%
Reverse_tr|F1RZT3|F1RZT3	0	0	0	0	0	0	0	1	0	0	0	0	0	0	0	1	0.0%	0.0%	0.0%	0.0%	0.0%	0.0%	0.0%	2.6%
tr|F1RIK4|F1RIK4	0	0	0	0	0	0	0	1	0	0	0	0	0	0	0	1	0.0%	0.0%	0.0%	0.0%	0.0%	0.0%	0.0%	3.3%
Reverse_tr|F1RMM1|F1RMM1	0	0	0	0	0	0	0	1	0	0	0	0	0	0	0	1	0.0%	0.0%	0.0%	0.0%	0.0%	0.0%	0.0%	2.2%
Reverse_tr|I3LP05|I3LP05	0	0	0	0	0	0	1	0	0	0	0	0	0	0	1	0	0.0%	0.0%	0.0%	0.0%	0.0%	0.0%	21.0%	0.0%
tr|K7GLQ8|K7GLQ8	0	0	0	0	0	0	0	1	0	0	0	0	0	0	0	1	0.0%	0.0%	0.0%	0.0%	0.0%	0.0%	0.0%	7.8%
tr|F1STU8|F1STU8	0	0	0	0	0	0	0	1	0	0	0	0	0	0	0	1	0.0%	0.0%	0.0%	0.0%	0.0%	0.0%	0.0%	3.4%
tr|F1SLB3|F1SLB3	0	0	0	0	0	0	1	0	0	0	0	0	0	0	1	0	0.0%	0.0%	0.0%	0.0%	0.0%	0.0%	5.3%	0.0%
tr|K7GKV2|K7GKV2	0	0	0	0	0	0	0	1	0	0	0	0	0	0	0	1	0.0%	0.0%	0.0%	0.0%	0.0%	0.0%	0.0%	3.0%
tr|F1SPK8|F1SPK8	0	0	0	0	0	0	0	1	0	0	0	0	0	0	0	1	0.0%	0.0%	0.0%	0.0%	0.0%	0.0%	0.0%	7.6%
Reverse_tr|F1SC09|F1SC09	0	0	0	0	0	0	0	1	0	0	0	0	0	0	0	1	0.0%	0.0%	0.0%	0.0%	0.0%	0.0%	0.0%	1.3%
tr|K7GL06|K7GL06	0	0	0	0	0	0	0	1	0	0	0	0	0	0	0	1	0.0%	0.0%	0.0%	0.0%	0.0%	0.0%	0.0%	20.6%
tr|K7GLY5|K7GLY5	0	0	0	0	0	0	0	1	0	0	0	0	0	0	0	1	0.0%	0.0%	0.0%	0.0%	0.0%	0.0%	0.0%	8.4%
tr|I3L7X0|I3L7X0	0	0	0	0	0	0	0	1	0	0	0	0	0	0	0	1	0.0%	0.0%	0.0%	0.0%	0.0%	0.0%	0.0%	9.8%
tr|F1RU39|F1RU39	0	0	0	0	0	0	0	1	0	0	0	0	0	0	0	1	0.0%	0.0%	0.0%	0.0%	0.0%	0.0%	0.0%	2.7%
tr|F1RJ53|F1RJ53	0	0	0	0	0	0	0	1	0	0	0	0	0	0	0	1	0.0%	0.0%	0.0%	0.0%	0.0%	0.0%	0.0%	3.9%
Reverse_tr|K7GNQ3|K7GNQ3	0	0	0	0	0	0	0	1	0	0	0	0	0	0	0	1	0.0%	0.0%	0.0%	0.0%	0.0%	0.0%	0.0%	5.5%
Reverse_tr|F1ST01|F1ST01	0	0	0	0	0	0	0	1	0	0	0	0	0	0	0	1	0.0%	0.0%	0.0%	0.0%	0.0%	0.0%	0.0%	2.8%
tr|F1SDC4|F1SDC4	0	0	0	0	0	0	0	1	0	0	0	0	0	0	0	1	0.0%	0.0%	0.0%	0.0%	0.0%	0.0%	0.0%	2.5%
tr|F1SLV6|F1SLV6	0	0	0	0	0	0	1	0	0	0	0	0	0	0	1	0	0.0%	0.0%	0.0%	0.0%	0.0%	0.0%	2.1%	0.0%
B2ZHY2	0	0	0	0	0	0	0	1	0	0	0	0	0	0	0	1	0.0%	0.0%	0.0%	0.0%	0.0%	0.0%	0.0%	2.0%
tr|F1RZR9|F1RZR9	0	0	0	0	0	0	1	0	0	0	0	0	0	0	1	0	0.0%	0.0%	0.0%	0.0%	0.0%	0.0%	3.2%	0.0%
tr|I3LD02|I3LD02	0	0	0	0	0	0	0	1	0	0	0	0	0	0	0	1	0.0%	0.0%	0.0%	0.0%	0.0%	0.0%	0.0%	16.3%
Reverse_tr|F1RLI4|F1RLI4	0	0	0	0	0	0	1	0	0	0	0	0	0	0	1	0	0.0%	0.0%	0.0%	0.0%	0.0%	0.0%	3.9%	0.0%
tr|F1RQB9|F1RQB9	0	0	0	0	0	0	0	1	0	0	0	0	0	0	0	1	0.0%	0.0%	0.0%	0.0%	0.0%	0.0%	0.0%	2.5%
tr|K7GN37|K7GN37	0	0	0	0	0	0	0	1	0	0	0	0	0	0	0	1	0.0%	0.0%	0.0%	0.0%	0.0%	0.0%	0.0%	2.2%
Reverse_tr|F1SV29|F1SV29	0	0	0	0	0	0	0	1	0	0	0	0	0	0	0	1	0.0%	0.0%	0.0%	0.0%	0.0%	0.0%	0.0%	5.5%
tr|I3L783|I3L783	0	0	0	0	0	0	0	1	0	0	0	0	0	0	0	1	0.0%	0.0%	0.0%	0.0%	0.0%	0.0%	0.0%	5.0%
Reverse_tr|I3LAI2|I3LAI2	0	0	0	0	0	0	0	1	0	0	0	0	0	0	0	1	0.0%	0.0%	0.0%	0.0%	0.0%	0.0%	0.0%	1.1%
tr|F1SNB8|F1SNB8	0	0	0	0	0	0	0	1	0	0	0	0	0	0	0	1	0.0%	0.0%	0.0%	0.0%	0.0%	0.0%	0.0%	4.3%
tr|F1RWJ1|F1RWJ1	0	0	0	0	0	0	0	1	0	0	0	0	0	0	0	1	0.0%	0.0%	0.0%	0.0%	0.0%	0.0%	0.0%	1.1%
tr|F1RNZ3|F1RNZ3	0	0	0	0	0	0	0	1	0	0	0	0	0	0	0	1	0.0%	0.0%	0.0%	0.0%	0.0%	0.0%	0.0%	4.6%
tr|F1S7C7|F1S7C7	0	0	0	0	0	0	0	1	0	0	0	0	0	0	0	1	0.0%	0.0%	0.0%	0.0%	0.0%	0.0%	0.0%	4.4%
Reverse_tr|I3LNU2|I3LNU2	0	0	0	0	0	0	0	1	0	0	0	0	0	0	0	1	0.0%	0.0%	0.0%	0.0%	0.0%	0.0%	0.0%	0.5%
tr|F1S731|F1S731	0	0	0	0	0	0	1	0	0	0	0	0	0	0	1	0	0.0%	0.0%	0.0%	0.0%	0.0%	0.0%	9.8%	0.0%
Reverse_tr|I3L7A3|I3L7A3	0	0	0	0	0	0	0	1	0	0	0	0	0	0	0	1	0.0%	0.0%	0.0%	0.0%	0.0%	0.0%	0.0%	1.3%
tr|A2SW51|A2SW51	0	0	0	0	0	0	0	1	0	0	0	0	0	0	0	1	0.0%	0.0%	0.0%	0.0%	0.0%	0.0%	0.0%	8.3%
tr|F1SUV9|F1SUV9	0	0	0	0	0	0	1	0	0	0	0	0	0	0	1	0	0.0%	0.0%	0.0%	0.0%	0.0%	0.0%	5.7%	0.0%
tr|F1S5J5|F1S5J5	0	0	0	0	0	0	0	1	0	0	0	0	0	0	0	1	0.0%	0.0%	0.0%	0.0%	0.0%	0.0%	0.0%	2.4%
tr|I3LH46|I3LH46	0	0	0	0	0	0	0	1	0	0	0	0	0	0	0	1	0.0%	0.0%	0.0%	0.0%	0.0%	0.0%	0.0%	10.2%
tr|K7GSN6|K7GSN6	0	0	0	0	0	0	0	1	0	0	0	0	0	0	0	1	0.0%	0.0%	0.0%	0.0%	0.0%	0.0%	0.0%	15.5%
tr|F1SM42|F1SM42	0	0	0	0	0	0	0	1	0	0	0	0	0	0	0	1	0.0%	0.0%	0.0%	0.0%	0.0%	0.0%	0.0%	4.4%
tr|I3LAH3|I3LAH3	0	0	0	0	0	0	0	1	0	0	0	0	0	0	0	1	0.0%	0.0%	0.0%	0.0%	0.0%	0.0%	0.0%	2.5%
tr|F1RT97|F1RT97	0	0	0	0	0	0	0	1	0	0	0	0	0	0	0	1	0.0%	0.0%	0.0%	0.0%	0.0%	0.0%	0.0%	2.8%
tr|F1SNR2|F1SNR2	0	0	0	0	0	0	0	1	0	0	0	0	0	0	0	1	0.0%	0.0%	0.0%	0.0%	0.0%	0.0%	0.0%	3.7%
tr|K7GLE8|K7GLE8	0	0	0	0	0	0	0	1	0	0	0	0	0	0	0	1	0.0%	0.0%	0.0%	0.0%	0.0%	0.0%	0.0%	5.2%
tr|K7GLZ4|K7GLZ4	0	0	0	0	0	0	0	1	0	0	0	0	0	0	0	1	0.0%	0.0%	0.0%	0.0%	0.0%	0.0%	0.0%	8.4%
tr|F1SD43|F1SD43	0	0	0	0	0	0	0	1	0	0	0	0	0	0	0	1	0.0%	0.0%	0.0%	0.0%	0.0%	0.0%	0.0%	13.0%
tr|I3LIF8|I3LIF8	0	0	0	0	0	0	0	1	0	0	0	0	0	0	0	1	0.0%	0.0%	0.0%	0.0%	0.0%	0.0%	0.0%	11.8%
Reverse_tr|F1SU61|F1SU61	0	0	0	0	0	0	1	0	0	0	0	0	0	0	1	0	0.0%	0.0%	0.0%	0.0%	0.0%	0.0%	2.5%	0.0%
tr|F1SQF8|F1SQF8	0	0	0	0	0	0	0	1	0	0	0	0	0	0	0	1	0.0%	0.0%	0.0%	0.0%	0.0%	0.0%	0.0%	8.1%
tr|K7GRF9|K7GRF9	0	0	0	0	0	0	0	1	0	0	0	0	0	0	0	1	0.0%	0.0%	0.0%	0.0%	0.0%	0.0%	0.0%	13.2%
Reverse_tr|F1RGJ6|F1RGJ6	0	0	0	0	0	0	0	1	0	0	0	0	0	0	0	1	0.0%	0.0%	0.0%	0.0%	0.0%	0.0%	0.0%	1.9%
Reverse_tr|F1SR46|F1SR46	0	0	0	0	0	0	0	1	0	0	0	0	0	0	0	1	0.0%	0.0%	0.0%	0.0%	0.0%	0.0%	0.0%	3.0%
tr|I3LV30|I3LV30	0	0	0	0	0	0	0	1	0	0	0	0	0	0	0	1	0.0%	0.0%	0.0%	0.0%	0.0%	0.0%	0.0%	13.8%
Reverse_tr|F1RYN0|F1RYN0	0	0	0	0	0	0	0	1	0	0	0	0	0	0	0	1	0.0%	0.0%	0.0%	0.0%	0.0%	0.0%	0.0%	11.4%
tr|F1SJF2|F1SJF2	0	0	0	0	0	0	1	0	0	0	0	0	0	0	1	0	0.0%	0.0%	0.0%	0.0%	0.0%	0.0%	1.3%	0.0%
tr|I3LA82|I3LA82	0	0	0	0	0	0	0	1	0	0	0	0	0	0	0	1	0.0%	0.0%	0.0%	0.0%	0.0%	0.0%	0.0%	11.3%
tr|I3LQY2|I3LQY2	0	0	0	0	0	0	1	0	0	0	0	0	0	0	1	0	0.0%	0.0%	0.0%	0.0%	0.0%	0.0%	0.9%	0.0%
tr|I3LK03|I3LK03	0	0	0	0	0	0	0	1	0	0	0	0	0	0	0	1	0.0%	0.0%	0.0%	0.0%	0.0%	0.0%	0.0%	12.0%
Reverse_tr|F1RYP1|F1RYP1	0	0	0	0	0	0	0	1	0	0	0	0	0	0	0	1	0.0%	0.0%	0.0%	0.0%	0.0%	0.0%	0.0%	2.8%
tr|I3L9P7|I3L9P7	0	0	0	0	0	0	0	1	0	0	0	0	0	0	0	1	0.0%	0.0%	0.0%	0.0%	0.0%	0.0%	0.0%	8.6%
Q6JTA8	0	0	0	0	0	0	0	1	0	0	0	0	0	0	0	1	0.0%	0.0%	0.0%	0.0%	0.0%	0.0%	0.0%	3.0%
tr|F1STN3|F1STN3	0	0	0	0	0	0	0	1	0	0	0	0	0	0	0	1	0.0%	0.0%	0.0%	0.0%	0.0%	0.0%	0.0%	0.7%
tr|F1RIZ3|F1RIZ3	0	0	0	0	0	0	0	1	0	0	0	0	0	0	0	1	0.0%	0.0%	0.0%	0.0%	0.0%	0.0%	0.0%	2.2%
tr|I3LFS0|I3LFS0	0	0	0	0	0	0	1	0	0	0	0	0	0	0	1	0	0.0%	0.0%	0.0%	0.0%	0.0%	0.0%	9.0%	0.0%
tr|F1RN30|F1RN30	0	0	0	0	0	0	0	1	0	0	0	0	0	0	0	1	0.0%	0.0%	0.0%	0.0%	0.0%	0.0%	0.0%	3.2%
tr|F1SN78|F1SN78	0	0	0	0	0	0	0	1	0	0	0	0	0	0	0	1	0.0%	0.0%	0.0%	0.0%	0.0%	0.0%	0.0%	3.4%
tr|F1RJE5|F1RJE5	0	0	0	0	0	0	0	1	0	0	0	0	0	0	0	1	0.0%	0.0%	0.0%	0.0%	0.0%	0.0%	0.0%	2.1%
Reverse_tr|F1S736|F1S736	0	0	0	0	0	0	0	1	0	0	0	0	0	0	0	1	0.0%	0.0%	0.0%	0.0%	0.0%	0.0%	0.0%	0.6%
tr|I3LI79|I3LI79	0	0	0	0	0	0	1	0	0	0	0	0	0	0	1	0	0.0%	0.0%	0.0%	0.0%	0.0%	0.0%	2.1%	0.0%
tr|F1RY26|F1RY26	0	0	0	0	0	0	0	1	0	0	0	0	0	0	0	1	0.0%	0.0%	0.0%	0.0%	0.0%	0.0%	0.0%	3.0%
tr|I3LNW9|I3LNW9	0	0	0	0	0	0	0	1	0	0	0	0	0	0	0	1	0.0%	0.0%	0.0%	0.0%	0.0%	0.0%	0.0%	4.8%
tr|K7GP48|K7GP48	0	0	0	0	0	0	0	1	0	0	0	0	0	0	0	1	0.0%	0.0%	0.0%	0.0%	0.0%	0.0%	0.0%	13.5%
tr|F1SL34|F1SL34	0	0	0	0	0	0	0	1	0	0	0	0	0	0	0	1	0.0%	0.0%	0.0%	0.0%	0.0%	0.0%	0.0%	5.3%
tr|K7GQN5|K7GQN5	0	0	0	0	0	0	0	1	0	0	0	0	0	0	0	1	0.0%	0.0%	0.0%	0.0%	0.0%	0.0%	0.0%	8.4%
tr|F1SHN8|F1SHN8	0	0	0	0	0	0	0	1	0	0	0	0	0	0	0	1	0.0%	0.0%	0.0%	0.0%	0.0%	0.0%	0.0%	2.2%
tr|F1SLY8|F1SLY8	0	0	0	0	0	0	1	0	0	0	0	0	0	0	1	0	0.0%	0.0%	0.0%	0.0%	0.0%	0.0%	1.7%	0.0%
tr|F1RH86|F1RH86	0	0	0	0	0	0	0	1	0	0	0	0	0	0	0	1	0.0%	0.0%	0.0%	0.0%	0.0%	0.0%	0.0%	12.0%
tr|I3L799|I3L799	0	0	0	0	0	0	0	1	0	0	0	0	0	0	0	1	0.0%	0.0%	0.0%	0.0%	0.0%	0.0%	0.0%	17.9%

	Peptide Count								Spectral Count								Sequence Coverage							
	Whole Cell	Whole Cell	Whole Cell	Cilia Fraction	Cilia Fraction	Cilia Fraction	Cilia Fraction	Cilia Fraction	Whole Cell	Whole Cell	Whole Cell	Cilia Fraction	Cilia Fraction	Cilia Fraction	Cilia Fraction	Cilia Fraction	Whole Cell	Whole Cell	Whole Cell	Cilia Fraction	Cilia Fraction	Cilia Fraction	Cilia Fraction	Cilia Fraction
Sample#	1	2	6	4	3	5	7	8	1	2	6	4	3	5	7	8	1	2	6	4	3	5	7	8
**Accession**																								
tr|F1RWF7|F1RWF7	0	0	0	0	0	0	0	1	0	0	0	0	0	0	0	1	0.0%	0.0%	0.0%	0.0%	0.0%	0.0%	0.0%	5.6%
tr|F1RRU2|F1RRU2	0	0	0	0	0	0	0	1	0	0	0	0	0	0	0	1	0.0%	0.0%	0.0%	0.0%	0.0%	0.0%	0.0%	2.5%
Reverse_tr|F1RYS0|F1RYS0	0	0	0	0	0	0	0	1	0	0	0	0	0	0	0	1	0.0%	0.0%	0.0%	0.0%	0.0%	0.0%	0.0%	0.5%
A5A769	0	0	0	0	0	0	0	1	0	0	0	0	0	0	0	1	0.0%	0.0%	0.0%	0.0%	0.0%	0.0%	0.0%	8.0%
tr|I3LPG0|I3LPG0	0	0	0	0	0	0	0	1	0	0	0	0	0	0	0	1	0.0%	0.0%	0.0%	0.0%	0.0%	0.0%	0.0%	3.6%
tr|I3LHI8|I3LHI8	0	0	0	0	0	0	1	0	0	0	0	0	0	0	1	0	0.0%	0.0%	0.0%	0.0%	0.0%	0.0%	12.8%	0.0%
tr|I3LD45|I3LD45	0	0	0	0	0	0	0	1	0	0	0	0	0	0	0	1	0.0%	0.0%	0.0%	0.0%	0.0%	0.0%	0.0%	11.2%
tr|F1SH98|F1SH98	0	0	0	0	0	0	0	1	0	0	0	0	0	0	0	1	0.0%	0.0%	0.0%	0.0%	0.0%	0.0%	0.0%	4.3%
tr|I3LBF9|I3LBF9	0	0	0	0	0	0	0	1	0	0	0	0	0	0	0	1	0.0%	0.0%	0.0%	0.0%	0.0%	0.0%	0.0%	2.7%
tr|I3LS16|I3LS16	0	0	0	0	0	0	0	1	0	0	0	0	0	0	0	1	0.0%	0.0%	0.0%	0.0%	0.0%	0.0%	0.0%	11.2%
tr|K7GMC3|K7GMC3	0	0	0	0	0	0	0	1	0	0	0	0	0	0	0	1	0.0%	0.0%	0.0%	0.0%	0.0%	0.0%	0.0%	8.2%
tr|F1STC6|F1STC6	0	0	0	0	0	0	0	1	0	0	0	0	0	0	0	1	0.0%	0.0%	0.0%	0.0%	0.0%	0.0%	0.0%	8.5%
tr|F1RL78|F1RL78	0	0	0	0	0	0	0	1	0	0	0	0	0	0	0	1	0.0%	0.0%	0.0%	0.0%	0.0%	0.0%	0.0%	2.5%
tr|K7GQ17|K7GQ17	0	0	0	0	0	0	0	1	0	0	0	0	0	0	0	1	0.0%	0.0%	0.0%	0.0%	0.0%	0.0%	0.0%	46.9%
tr|I3LSJ0|I3LSJ0	0	0	0	0	0	0	0	1	0	0	0	0	0	0	0	1	0.0%	0.0%	0.0%	0.0%	0.0%	0.0%	0.0%	4.5%
Q75QW1	0	0	4	0	0	0	3	10	0	0	11	0	0	0	161	260	0.0%	0.0%	12.1%	0.0%	0.0%	0.0%	16.6%	27.4%
tr|I3LE55|I3LE55	0	0	1	0	0	0	0	4	0	0	1	0	0	0	0	28	0.0%	0.0%	6.3%	0.0%	0.0%	0.0%	0.0%	18.5%
tr|F1S0M1|F1S0M1	0	0	8	0	0	0	16	27	0	0	40	0	0	0	526	348	0.0%	0.0%	45.0%	0.0%	0.0%	0.0%	54.4%	63.1%
