# Supplementary material for: Proteomic Identification Reveals the Role of Ciliary Extracellular‐Like Vesicle in Cardiovascular Function
Source: Adv Sci (Weinh). 2020 Jun 16;7(16):1903140. doi: 10.1002/advs.201903140 (PMC7435257; doi:10.1002/advs.201903140)
Supplement: Supplementary file 6 — Supplemental Table 2 [file ADVS-7-1903140-s006.pdf]

Supplementary Table 2

Accession#	Description	Sequence Coverage		Spectral Count			Classification	Reference
		Whole Cells	Cilia	Whole Cells	Cilia	Ratio		
tr|F1RWN4|F1RWN4	14 kDa phosphohistidine phosphatase	0.00%	11.90%	0.00	3.00	HIGH	New	N/A
tr|F1S367|F1S367	4-hydroxyphenylpyruvate dioxygenase	0.00%	2.17%	0.00	0.33	HIGH	New	N/A
P80387	5'-AMP-activated protein kinase subunit beta-1	0.00%	11.10%	0.00	3.00	HIGH	New Ɨ	N/A
tr|F1RL45|F1RL45	5'-AMP-activated protein kinase subunit beta-1	0.00%	24.60%	0.00	3.00	HIGH	New Ɨ	N/A
Q09138	5'-AMP-activated protein kinase subunit gamma-1	0.00%	6.10%	0.00	1.00	HIGH	New	N/A
P49666	60S ribosomal protein L21	0.00%	17.70%	0.00	2.50	HIGH	Known	1
Q29187	60S ribosomal protein L4 (Fragment)	0.00%	10.90%	0.00	0.50	HIGH	Known	2
Q2HYU2	6-phosphofructokinase, muscle type	0.00%	1.50%	0.00	2.50	HIGH	Known Ɨ	3
tr|I3LCA1|I3LCA1	6-phosphofructokinase, muscle type	0.00%	1.20%	0.00	2.50	HIGH	Known Ɨ	3
tr|F1SKQ9|F1SKQ9	Actin-related protein 2/3 complex subunit 5	0.00%	36.60%	0.00	3.00	HIGH	Known	4
P58780	Adenosine deaminase CECR1	0.00%	5.30%	0.00	0.50	HIGH	New	N/A
tr|I3LN54|I3LN54	ADP-ribosylation factor-like protein 6-interacting protein 1	0.00%	9.35%	0.00	5.00	HIGH	New	N/A
tr|I6L6E1|I6L6E1	Aldehyde dehydrogenase	0.00%	4.00%	0.00	36.00	HIGH	New Ɨ	N/A
tr|V9GZ89|V9GZ89	Aldehyde dehydrogenase	0.00%	2.40%	0.00	31.50	HIGH	New Ɨ	N/A

tr|F1SDC4|F1SDC4	Aldehyde dehydrogenase (Fragment)	0.00%	2.50%	0.00	0.50	HIGH	New Ɨ	N/A
tr|F1SMT7|F1SMT7	Alkaline phosphatase	0.00%	4.90%	0.00	0.50	HIGH	New Ɨ	5
tr|I3LB86|I3LB86	Alkaline phosphatase	0.00%	6.45%	0.00	79.50	HIGH	NewƗ	5
P50447	Alpha-1-antitrypsin	0.00%	3.10%	0.00	12.00	HIGH	New Ɨ	N/A
tr|F1SCF0|F1SCF0	Alpha-1-antitrypsin	0.00%	3.10%	0.00	12.00	HIGH	New Ɨ	N/A
Q8MJ76	Alpha-fetoprotein	0.00%	1.30%	0.00	13.00	HIGH	New	N/A
P15145	Aminopeptidase N	8.25%	15.77%	8.50	62.00	7.29	New Ɨ,*	6
tr|F1SK03|F1SK03	Aminopeptidase N	8.25%	16.80%	8.50	62.67	7.37	New Ɨ	N/A
tr|F1SGY1|F1SGY1	Anoctamin	0.45%	0.60%	3.00	6.33	2.11	Known	7,8
F1SPM8	AP2-associated protein kinase 1	0.00%	1.05%	0.00	5.50	HIGH	Known Ɨ	4
tr|I3L548|I3L548	AP2-associated protein kinase 1	0.00%	1.05%	0.00	5.50	HIGH	Known Ɨ	4
Q2LE37	Apolipoprotein M	0.00%	4.00%	0.00	2.00	HIGH	New Ɨ	N/A
tr|A5D9L6|A5D9L6	Apolipoprotein M	0.00%	6.45%	0.00	2.00	HIGH	New Ɨ	N/A
tr|B6DZ39|B6DZ39	APPL2	0.00%	1.30%	0.00	0.33	HIGH	New	N/A
Q35915	ATP synthase subunit a	0.00%	6.20%	0.00	1.00	HIGH	Known	9
tr|A5GFS1|A5GFS1	ATPase, Class II, type 9A	0.00%	2.00%	0.00	1.00	HIGH	New	N/A
tr|K7GT44|K7GT44	ATPase, Cu++ transporting, alpha polypeptide	0.00%	0.57%	0.00	0.33	HIGH	New	N/A

Q8MIB3	ATP-binding cassette sub-family G member 2	0.00%	1.83%	0.00	3.00	HIGH	New Ɨ	10
tr|F1RW52|F1RW52	ATP-binding cassette sub-family G member 2	0.00%	1.83%	0.00	3.00	HIGH	New Ɨ	10
tr|K7GLY4|K7GLY4	ATP-binding cassette sub-family G member 2	0.00%	6.30%	0.00	4.50	HIGH	New Ɨ	10
tr|K7GPH3|K7GPH3	ATP-binding cassette sub-family G member 2	0.00%	6.80%	0.00	4.50	HIGH	New Ɨ	10
tr|K7GQE8|K7GQE8	ATP-binding cassette sub-family G member 2	0.00%	5.75%	0.00	5.00	HIGH	New Ɨ	10
tr|F1SI55|F1SI55	Basic leucine zipper and W2 domain-containing protein 1	0.00%	6.30%	0.00	2.00	HIGH	New	N/A
tr|A7XP20|A7XP20	B-cell CLL/lymphoma 10	0.00%	14.20%	0.00	0.50	HIGH	New	N/A
A5A769	Biogenesis of lysosome-related organelles complex 1 subunit 3	0.00%	8.00%	0.00	0.50	HIGH	New	N/A
tr|F1SI16|F1SI16	Bone morphogenetic protein type II receptor (BMPR2)	0.00%	4.30%	0.00	2.50	HIGH	Known	11
tr|Q7JFN4|Q7JFN4	Calcium/calmodulin-dependent protein kinase II isoform gamma-B	1.85%	2.50%	0.50	2.67	5.33	New	N/A
tr|Q7JFN3|Q7JFN3	Calcium/calmodulin-dependent protein kinase II isoform gamma-C protein kinase II	1.90%	2.63%	0.50	2.67	5.33	New	N/A
tr|Q7JFN2|Q7JFN2	Calcium/calmodulin-dependent protein kinase II isoform gamma-G	1.75%	2.43%	0.50	2.67	5.33	New	N/A
tr|F1SUV9|F1SUV9	Caspase	0.00%	5.70%	0.00	0.50	HIGH	Known Ɨ	12
tr|I3L9A3|I3L9A3	Caspase	0.00%	6.00%	0.00	0.50	HIGH	Known Ɨ	12
Q29288	C-C motif chemokine 5	0.00%	8.00%	0.00	1.33	HIGH	Known Ɨ	13
tr|K7GLL8|K7GLL8	C-C motif chemokine 5	0.00%	4.87%	0.00	1.33	HIGH	Known Ɨ	13
tr|Q2EN89|Q2EN89	C-C motif chemokine 5	0.00%	4.40%	0.00	1.33	HIGH	Known Ɨ	13

Q8WMQ3	CD9 antigen	0.00%	7.10%	0.00	1.00	HIGH	Known Ɨ,*	2,14
tr|I3LPR3|I3LPR3	CD9 antigen	0.00%	18.60%	0.00	18.50	HIGH	Known Ɨ	2
tr|K7GMI9|K7GMI9	CD9 antigen	0.00%	30.40%	0.00	18.50	HIGH	Known Ɨ	2
tr|K7GLH2|K7GLH2	Cell division control protein 42 homolog	24.40%	32.57%	2.00	5.00	2.50	Known Ɨ	15
tr|K7GMZ6|K7GMZ6	Cell division control protein 42 homolog	18.60%	24.83%	2.00	5.00	2.50	Known Ɨ	15
tr|K7GR85|K7GR85	Cell division control protein 42 homolog	26.80%	35.77%	2.00	5.00	2.50	Known Ɨ	15
F1S584	Choline transporter-like protein 2	0.00%	1.50%	0.00	9.00	HIGH	New	N/A
tr|I3L6H2|I3L6H2	Citrate synthase, mitochondrial	0.00%	10.70%	0.00	3.00	HIGH	New	N/A
tr|F2Z540|F2Z540	C-Jun activation domain binding protein-1	0.00%	2.85%	0.00	2.00	HIGH	New	N/A
tr|C3VML0|C3VML0	Claudin	0.00%	1.90%	0.00	0.33	HIGH	Known Ɨ,*	16,17
tr|C3VMW5|C3VMW5	Claudin	0.00%	7.60%	0.00	1.00	HIGH	Known Ɨ	16
tr|I3LHE9|I3LHE9	Claudin	5.45%	17.53%	4.00	9.67	2.42	Known Ɨ	16
tr|A5GFW3|A5GFW3	Cleavage stimulation factor, 3' pre-RNA, subunit 1, 50kDa	0.00%	2.80%	0.00	0.50	HIGH	New	N/A
Q9GLP1	Coagulation factor V	0.00%	0.30%	0.00	2.50	HIGH	New Ɨ	N/A
tr|F1RPW2|F1RPW2	Coagulation factor V	0.00%	0.30%	0.00	2.50	HIGH	New Ɨ	N/A
P01025	Complement C3	0.00%	1.65%	0.00	17.50	HIGH	New	N/A
tr|F1SME1|F1SME1	Complement C5a anaphylatoxin	0.00%	0.27%	0.00	2.00	HIGH	New	N/A

Q9TUQ3	Complement component C7	0.00%	1.95%	0.00	42.50	HIGH	New Ɨ	N/A
tr|F1SMJ1|F1SMJ1	Complement component C7	0.00%	1.17%	0.00	3.00	HIGH	New Ɨ	N/A
tr|I3L9X0|I3L9X0	Coronin	0.00%	2.70%	0.00	3.00	HIGH	Known Ɨ	18
tr|I3LHE4|I3LHE4	Coronin	0.00%	2.50%	0.00	3.00	HIGH	Known Ɨ	18
tr|I3LQY3|I3LQY3	Coronin	0.00%	2.50%	0.00	3.00	HIGH	Known Ɨ	18
Q29594	Creatine kinase B-type (Fragment)	0.00%	15.70%	0.00	0.50	HIGH	New	N/A
O79876	Cytochrome c oxidase subunit 1	0.00%	6.60%	0.00	0.50	HIGH	New	N/A
Q35916	Cytochrome c oxidase subunit 3	0.00%	5.40%	0.00	1.50	HIGH	New	N/A
tr|F1RJI7|F1RJI7	Cytochrome c oxidase subunit 6A, mitochondrial	0.00%	9.65%	0.00	4.50	HIGH	New	N/A
tr|F1SIE9|F1SIE9	Cytochrome P450	0.00%	0.70%	0.00	0.33	HIGH	New	N/A
P00371	D-amino-acid oxidase	0.00%	2.47%	0.00	1.00	HIGH	New	N/A
tr|F1RPB5|F1RPB5	DCN1-like protein	0.00%	7.60%	0.00	0.50	HIGH	New	N/A
tr|F1S8E6|F1S8E6	Deoxyhypusine hydroxylase	0.00%	7.60%	0.00	1.00	HIGH	New	N/A
tr|I3LTI5|I3LTI5	Deoxyribonuclease (Fragment)	0.00%	5.90%	0.00	4.50	HIGH	New	N/A
Q2QDF0	Deoxyribonuclease-1-like 1	0.00%	8.75%	0.00	5.50	HIGH	New	N/A
tr|I3L719|I3L719	Dipeptidase	0.00%	10.65%	0.00	33.00	HIGH	Known	19
P22412	Dipeptidase 1	0.00%	13.10%	0.00	47.50	HIGH	Known	19

P22411	Dipeptidyl peptidase 4	4.50%	7.10%	3.50	21.67	6.19	New	N/A
tr|F1RZH4|F1RZH4	Disintegrin and metalloproteinase domain-containing protein 10	0.00%	11.30%	0.00	1.50	HIGH	Known*	4,20
A5GFZ5	Dolichol-phosphate mannosyltransferase subunit 1	0.00%	4.60%	0.00	1.00	HIGH	New	N/A
tr|D9U8D1|D9U8D1	Dynein, light chain, LC8-type 1	0.00%	21.30%	0.00	14.00	HIGH	New	21
Q5GN48	Dystrophin	0.00%	7.80%	0.00	2.50	HIGH	Known Ɨ	22
tr|I3LPH8|I3LPH8	Dystrophin	0.00%	7.00%	0.00	1.50	HIGH	Known Ɨ	22
tr|F1SPU7|F1SPU7	Dystrophin (Fragment)	0.00%	1.40%	0.00	2.50	HIGH	Known Ɨ	22
tr|F1SM37|F1SM37	E3 ubiquitin-protein ligase	0.00%	1.95%	0.00	6.00	HIGH	New	N/A
tr|I3LV30|I3LV30	E3 ubiquitin-protein ligase RNF114 (Fragment)	0.00%	13.80%	0.00	0.50	HIGH	New	N/A
tr|F1RLP4|F1RLP4	EFNB2	0.00%	8.10%	0.00	2.50	HIGH	New	N/A
P37176	Endoglin	0.00%	1.63%	0.00	0.33	HIGH	New Ɨ	N/A
tr|F1RS03|F1RS03	Endoglin	0.00%	1.63%	0.00	0.33	HIGH	New Ɨ	N/A
tr|K7GQF2|K7GQF2	Endoglin	0.00%	1.73%	0.00	0.33	HIGH	New Ɨ	N/A
P35463	Endothelin B receptor	0.00%	3.40%	0.00	0.50	HIGH	New	23
tr|D3K5K6|D3K5K6	EPH receptor B3	0.00%	1.60%	0.00	11.00	HIGH	New	N/A
P20460	Eukaryotic translation initiation factor 2 subunit 1 (Fragment)	0.00%	18.60%	0.00	1.00	HIGH	New	N/A
tr|F1SGR0|F1SGR0	Eukaryotic translation initiation factor 3 subunit M	0.00%	13.10%	0.00	3.00	HIGH	New	N/A

tr|B2ZI35|B2ZI35	F11 receptor	0.00%	4.20%	0.00	24.00	HIGH	New	N/A
Q767L6	Flotillin-1	3.75%	3.80%	1.00	7.00	7.00	Known Ɨ,*	24,25
tr|K7GPJ9|K7GPJ9	Flotillin-1	5.25%	5.33%	1.00	7.00	7.00	Known Ɨ	24
tr|F8S319|F8S319	Gag protein	0.00%	1.15%	0.00	8.00	HIGH	New Ɨ	N/A
tr|F8S326|F8S326	Gag protein	0.00%	3.80%	0.00	15.00	HIGH	New Ɨ	N/A
Q29043	Galactoside 2-alpha-L-fucosyltransferase 1	0.00%	1.57%	0.00	0.33	HIGH	New Ɨ	N/A
tr|F1RIQ8|F1RIQ8	Galactoside 2-alpha-L-fucosyltransferase 1	0.00%	1.57%	0.00	0.33	HIGH	New Ɨ	N/A
tr|I3L8J2|I3L8J2	Galectin	0.00%	12.80%	0.00	1.00	HIGH	New Ɨ	N/A
tr|I3LIV0|I3LIV0	Galectin (Fragment)	0.00%	12.80%	0.00	1.00	HIGH	New Ɨ	26
tr|K7GKZ5|K7GKZ5	Gamma-glutamyltranspeptidase 1	0.00%	8.40%	0.00	2.00	HIGH	Known Ɨ	27
tr|K7GQA6|K7GQA6	Gamma-glutamyltranspeptidase 1	0.00%	8.95%	0.00	2.00	HIGH	Known Ɨ	27
tr|K7GQU9|K7GQU9	Gamma-glutamyltranspeptidase 1	0.00%	9.40%	0.00	3.00	HIGH	Known Ɨ	27
tr|K7GRH1|K7GRH1	Gamma-glutamyltranspeptidase 1	0.00%	9.00%	0.00	4.50	HIGH	Known Ɨ	27
tr|K7GSR0|K7GSR0	Gamma-glutamyltranspeptidase 1	0.00%	8.85%	0.00	2.00	HIGH	Known Ɨ	27
tr|F1S138|F1S138	Glutamyl aminopeptidase	5.40%	8.43%	4.00	9.33	2.33	New Ɨ	N/A
tr|K7GMS4|K7GMS4	Glutamyl aminopeptidase	0.00%	16.40%	0.00	25.50	HIGH	New Ɨ	N/A
tr|K7GQE4|K7GQE4	Glutamyl aminopeptidase	15.60%	20.80%	1.50	4.00	2.67	New Ɨ	N/A

tr|K7GT33|K7GT33	Glutamyl aminopeptidase	4.95%	8.83%	1.00	4.33	4.33	New Ⱡ	N/A
Q8MJ14	Glutathione peroxidase 1	0.00%	5.35%	0.00	7.00	HIGH	New	N/A
P51781	Glutathione S-transferase alpha M14	0.00%	4.07%	0.00	0.33	HIGH	New	N/A
tr|A3KDL8|A3KDL8	Glypican 6 (Fragment)	0.00%	18.10%	0.00	2.50	HIGH	New#	28
tr|A5GFU8|A5GFU8	GNAS complex locus	0.00%	21.30%	0.00	1.00	HIGH	New	N/A
B2ZHY2	G-protein coupled receptor 39	0.00%	2.00%	0.00	0.50	HIGH	Known Ⱡ	29
tr|F1RY26|F1RY26	G-protein-coupled receptor 39	0.00%	3.00%	0.00	0.50	HIGH	Known Ⱡ	29
Q52NJ3	GTP-binding protein SAR1a	0.00%	8.60%	0.00	2.50	HIGH	New	N/A
tr|I7GF95|I7GF95	Guanine nucleotide binding protein-like 1	0.00%	3.00%	0.00	0.50	HIGH	New	N/A
tr|A4L2Z4|A4L2Z4	Guanine nucleotide-binding protein alpha 14	0.00%	4.80%	0.00	31.00	HIGH	New	N/A
tr|J9JIL1|J9JIL1	Guanine nucleotide-binding protein G(q) subunit alpha	0.00%	9.95%	0.00	11.00	HIGH	New	N/A
tr|I3LJ42|I3LJ42	Guanine nucleotide-binding protein subunit gamma	0.00%	33.00%	0.00	10.50	HIGH	New	N/A
P31006	Guanylate kinase	0.00%	11.10%	0.00	0.50	HIGH	New	N/A
tr|A5A4L7|A5A4L7	H+ coupled di/tri-peptide cotransporter 2	0.00%	1.90%	0.00	0.50	HIGH	New	N/A
P01965	Hemoglobin subunit alpha	7.80%	8.27%	3.00	14.00	4.67	New	N/A
P02067	Hemoglobin subunit beta	0.00%	7.15%	0.00	545.00	HIGH	New Ⱡ	N/A
tr|F1RII7|F1RII7	Hemoglobin subunit beta	0.00%	7.15%	0.00	545.00	HIGH	New Ⱡ	N/A

P02101	Hemoglobin subunit epsilon	0.00%	3.05%	0.00	5.00	HIGH	New Ɨ	N/A
tr|F1RII6|F1RII6	Hemoglobin subunit epsilon	0.00%	3.05%	0.00	5.00	HIGH	New Ɨ	N/A
P04246	Hemoglobin subunit theta	0.00%	3.10%	0.00	5.00	HIGH	New	N/A
Q2QLE0	Hepatocyte growth factor receptor	0.00%	1.50%	0.00	1.00	HIGH	Known	30
Q8WNR0	High affinity copper uptake protein 1	0.00%	5.05%	0.00	2.50	HIGH	New Ɨ	N/A
tr|I3LH68|I3LH68	High affinity copper uptake protein 1	0.00%	5.05%	0.00	2.50	HIGH	New Ɨ	N/A
tr|F1RHC5|F1RHC5	Histone H2A	0.00%	8.90%	0.00	1.50	HIGH	Known Ɨ	31
tr|F1RTQ7|F1RTQ7	Histone H2A	0.00%	7.25%	0.00	16.50	HIGH	Known Ɨ	31
tr|F2Z587|F2Z587	Histone H2A	0.00%	7.30%	0.00	6.50	HIGH	Known Ɨ	31
tr|F2Z5L6|F2Z5L6	Histone H2A	0.00%	7.30%	0.00	16.50	HIGH	Known Ɨ	31
tr|I3L7T6|I3L7T6	Histone H2A	0.00%	6.65%	0.00	16.50	HIGH	Known Ɨ	31
tr|I3LIN8|I3LIN8	Histone H2A	0.00%	9.00%	0.00	1.50	HIGH	Known Ɨ	31
tr|F2Z5P1|F2Z5P1	Histone H2A (Fragment)	0.00%	22.80%	0.00	4.50	HIGH	Known Ɨ	31
Reverse_sp|E2IUK4|HORM1	HORMA domain-containing protein 1	0.00%	5.10%	0.00	0.50	HIGH	New Ɨ	N/A
Reverse_tr|F1SS82|F1SS82	HORMA domain-containing protein 1	0.00%	5.10%	0.00	0.50	HIGH	New Ɨ	N/A
tr|F1SPN8|F1SPN8	Hyaluronidase	0.00%	4.40%	0.00	0.50	HIGH	New	32
tr|A9QW81|A9QW81	IFITM1	10.50%	22.60%	1.00	2.33	2.33	New	33

tr|F1SV93|F1SV93	Importin subunit alpha	0.00%	3.25%	0.00	7.50	HIGH	New Ɨ	N/A
tr|I3L9F7|I3L9F7	Importin subunit alpha	0.00%	3.50%	0.00	5.00	HIGH	New Ɨ	N/A
tr|F1RTU5|F1RTU5	Importin subunit alpha (Fragment)	0.00%	1.30%	0.00	4.50	HIGH	New Ɨ	N/A
P24853	Insulin-like growth factor-binding protein 2	0.00%	6.65%	0.00	8.50	HIGH	Known	34
Q52N47	Integral membrane protein 2B	0.00%	27.10%	0.00	5.00	HIGH	New Ɨ	N/A
tr|F1RK12|F1RK12	Integral membrane protein 2B	0.00%	27.10%	0.00	5.00	HIGH	New Ɨ	N/A
tr|F1RVY6|F1RVY6	Integrin beta	0.70%	1.10%	1.00	5.67	5.67	New Ɨ	N/A
tr|I3LF22|I3LF22	Integrin beta	0.00%	6.10%	0.00	15.00	HIGH	New Ɨ	N/A
tr|K7GP66|K7GP66	Integrin beta	0.70%	1.13%	1.00	5.67	5.67	New Ɨ	N/A
tr|K7GPD4|K7GPD4	Integrin beta	0.70%	1.13%	1.00	5.67	5.67	New Ɨ	N/A
tr|F1RPR7|F1RPR7	Integrin beta (Fragment)	0.00%	8.20%	0.00	1.50	HIGH	New	N/A
tr|K7GNY7|K7GNY7	Integrin beta-1	0.00%	9.70%	0.00	9.50	HIGH	Known	35
Q1RPR6	Integrin beta-6	0.00%	5.10%	0.00	3.50	HIGH	New	N/A
Q684M4	Kelch-like ECH-associated protein 1	0.00%	6.40%	0.00	14.00	HIGH	New	N/A
Reverse_sp|Q9MZS9|KMO	Kynurenine 3-monooxygenase	0.00%	1.27%	0.00	0.33	HIGH	New	N/A
P79385	Lactadherin	1.85%	8.47%	3.00	8.00	2.67	Known Ɨ	36
P79385	Lactadherin	0.00%	14.20%	0.00	74.50	HIGH	Known Ɨ	36

tr|B2CZF8|B2CZF8	Lactadherin	1.75%	8.03%	3.00	8.00	2.67	Known Ɨ	36
tr|B2CZF8|B2CZF8	Lactadherin	0.00%	13.45%	0.00	74.50	HIGH	Known Ɨ	36
A5D9M6	Large proline-rich protein BAG6	0.00%	0.80%	0.00	1.00	HIGH	New Ɨ	N/A
tr|F1RQX8|F1RQX8	Large proline-rich protein BAG6	0.00%	0.80%	0.00	1.00	HIGH	New Ɨ	N/A
tr|K7GLD3|K7GLD3	Large proline-rich protein BAG6	0.00%	3.80%	0.00	1.00	HIGH	New Ɨ	N/A
tr|K7GLG5|K7GLG5	Large proline-rich protein BAG6	0.00%	5.00%	0.00	1.00	HIGH	New Ɨ	N/A
tr|K7GLR6|K7GLR6	Large proline-rich protein BAG6	0.00%	3.40%	0.00	1.00	HIGH	New Ɨ	N/A
tr|K7GLU1|K7GLU1	Large proline-rich protein BAG6	0.00%	1.20%	0.00	1.00	HIGH	New Ɨ	N/A
tr|K7GMR9|K7GMR9	Large proline-rich protein BAG6	0.00%	2.10%	0.00	1.00	HIGH	New Ɨ	N/A
Q9GKE8	Leukocyte surface antigen CD47	0.00%	2.30%	0.00	43.50	HIGH	Known Ɨ	37
tr|F1SL37|F1SL37	Leukocyte surface antigen CD47	0.00%	2.45%	0.00	43.50	HIGH	Known Ɨ	37
Q5PXT2	LIM and cysteine-rich domains protein 1	0.00%	3.00%	0.00	0.50	HIGH	New Ɨ	N/A
tr|F1SFK9|F1SFK9	LIM and cysteine-rich domains protein 1	0.00%	3.20%	0.00	0.50	HIGH	New Ɨ	N/A
tr|F1SCY4|F1SCY4	Lipase	0.00%	6.30%	0.00	0.50	HIGH	know	38
P12067	Lysozyme C-1	0.00%	3.13%	0.00	1.00	HIGH	New	N/A
P12068	Lysozyme C-2	0.00%	2.73%	0.00	1.00	HIGH	New	N/A
P12069	Lysozyme C-3	0.00%	2.70%	0.00	1.00	HIGH	New	N/A

P49927	Major prion protein	0.00%	4.70%	0.00	2.00	HIGH	New	N/A
Q95313	Male-enhanced antigen 1	0.00%	9.80%	0.00	0.50	HIGH	New	N/A
I3LUP1	Mannose-1-phosphate guanyltransferase alpha	0.00%	5.70%	0.00	1.00	HIGH	New	N/A
P0C5I2	Mannose-1-phosphate guanyltransferase beta	0.00%	5.15%	0.00	4.00	HIGH	New Ɨ	N/A
tr|F1SPR4|F1SPR4	Mannose-1-phosphate guanyltransferase beta	0.00%	5.15%	0.00	4.00	HIGH	New Ɨ	N/A
O02839	Membrane cofactor protein	0.00%	16.30%	0.00	13.00	HIGH	New	N/A
tr|Q8MHU0|Q8MHU0	MHC class I antigen	0.00%	2.33%	0.00	1.00	HIGH	New*	39
tr|Q85ZW4|Q85ZW4	MHC class II antigen	0.00%	9.10%	0.00	8.50	HIGH	Known Ɨ,*	39,40
tr|Q8MGP9|Q8MGP9	MHC class II antigen	0.00%	8.80%	0.00	1.00	HIGH	Known Ɨ	40
tr|A2SW51|A2SW51	Monocyte differentiation antigen CD14	0.00%	8.30%	0.00	0.50	HIGH	New	N/A
P84024	Mothers against decapentaplegic homolog 3	0.00%	6.40%	0.00	0.50	HIGH	New	N/A
tr|F1SNW4|F1SNW4	MYL3	0.00%	2.20%	0.00	0.33	HIGH	New	41
tr|F1RGE3|F1RGE3	NADH dehydrogenase [ubiquinone] 1 alpha subcomplex subunit 2	0.00%	11.10%	0.00	0.50	HIGH	New Ɨ	N/A
tr|I3LS16|I3LS16	NADH dehydrogenase [ubiquinone] 1 alpha subcomplex subunit 2	0.00%	11.20%	0.00	0.50	HIGH	New Ɨ	N/A
tr|F1SRG2|F1SRG2	NADH dehydrogenase [ubiquinone] 1 alpha subcomplex subunit 6	0.00%	11.70%	0.00	0.50	HIGH	New	N/A
Q9TDR1	NADH-ubiquinone oxidoreductase chain 5	0.00%	2.00%	0.00	0.50	HIGH	New	N/A
Reverse_tr|F1RYP1|F1RYP1	Neurogenic differentiation factor	0.00%	2.80%	0.00	0.50	HIGH	New Ɨ	N/A

Reverse_tr|F1SR46|F1SR46	Neurogenic differentiation factor	0.00%	3.00%	0.00	0.50	HIGH	New Ɨ	N/A
tr|F1SSJ3|F1SSJ3	Nitric oxide synthase	0.00%	0.90%	0.00	3.00	HIGH	Known	42
Q28969	Nitric oxide synthase, endothelial	0.00%	0.85%	0.00	2.50	HIGH	Known	42
tr|I3LL54|I3LL54	Nuclear factor 1	0.00%	2.70%	0.00	1.00	HIGH	New Ɨ	N/A
tr|F1SDA0|F1SDA0	Nuclear factor 1 (Fragment)	0.00%	2.60%	0.00	1.00	HIGH	New Ɨ	N/A
tr|A5GFR1|A5GFR1	OTTSUSP00000000835	0.00%	0.57%	0.00	0.33	HIGH	New	N/A
tr|F1RMJ9|F1RMJ9	Oxysterol-binding protein	0.00%	2.00%	0.00	1.00	HIGH	New Ɨ	N/A
tr|F1RPE0|F1RPE0	Oxysterol-binding protein	0.00%	1.70%	0.00	1.00	HIGH	New Ɨ	N/A
tr|I3LAG4|I3LAG4	Palmitoyltransferase (Fragment)	0.00%	4.70%	0.00	1.50	HIGH	New	N/A
Q6QA76	PDZ domain-containing protein 11	0.00%	18.60%	0.00	0.50	HIGH	New Ɨ	N/A
tr|K7GNX1|K7GNX1	PDZ domain-containing protein 11	0.00%	19.10%	0.00	0.50	HIGH	New Ɨ	N/A
tr|K7GRD4|K7GRD4	PDZ domain-containing protein 11	0.00%	21.50%	0.00	0.50	HIGH	New Ɨ	N/A
tr|K7GSP4|K7GSP4	PDZ domain-containing protein 11	0.00%	24.30%	0.00	0.50	HIGH	New Ɨ	N/A
tr|F1SF95|F1SF95	Peptidyl-prolyl cis-trans isomerase	0.00%	15.30%	0.00	0.50	HIGH	New Ɨ	N/A
tr|I3L6I7|I3L6I7	Peptidyl-prolyl cis-trans isomerase	0.00%	14.40%	0.00	0.50	HIGH	New Ɨ	N/A
tr|I3LRQ5|I3LRQ5	Phosphatase and actin regulator	0.00%	5.80%	0.00	2.00	HIGH	New	N/A
Q767M0	Phostensin	0.00%	1.95%	0.00	2.00	HIGH	Known	4

tr|A2TEQ2|A2TEQ2	Poliovirus receptor related 2	0.00%	9.60%	0.00	4.00	HIGH	New	N/A
tr|B3TFD9|B3TFD9	Poliovirus receptor-related 2 transcript variant delta	0.00%	17.10%	0.00	6.00	HIGH	New	N/A
tr|F1S8H4|F1S8H4	Probable tRNA N6-adenosine threonylcarbamoyltransferase	0.00%	8.30%	0.00	1.50	HIGH	New	N/A
tr|A3RKG5|A3RKG5	Progesterone receptor membrane component 2 (PGRMC2)	0.00%	8.50%	0.00	0.50	HIGH	New Ɨ	N/A
tr|A9LM01|A9LM01	Progesterone receptor membrane component 2 (PGRMC2)	0.00%	15.2%	0.00	1.00	HIGH	New Ɨ	N/A
tr|F1RNX2|F1RNX2	Programmed cell death protein 5	0.00%	8.80%	0.00	2.00	HIGH	New#	14
Q6JTA8	Prolactin receptor	0.00%	3.00%	0.00	0.50	HIGH	New	N/A
tr|K7GKV2|K7GKV2	Prolactin receptor (Fragment)	0.00%	3.00%	0.00	0.50	HIGH	New	N/A
Q29095	Prostaglandin-H2 D-isomerase	0.00%	9.50%	0.00	1.00	HIGH	New	N/A
tr|A5D9J0|A5D9J0	Proteasome (Prosome, macropain) subunit, beta type, 9 (Large multifunctional protease 2)	0.00%	44.00%	0.00	2.50	HIGH	New	N/A
tr|F1RFV5|F1RFV5	Proteasome subunit beta type	0.00%	16.20%	0.00	2.00	HIGH	New Ɨ	N/A
tr|Q2PYM7|Q2PYM7	Proteasome subunit beta type	0.00%	20.10%	0.00	2.50	HIGH	New Ɨ	N/A
tr|I3LR85|I3LR85	Protein kinase C delta type	0.00%	1.10%	0.00	2.50	HIGH	New	43
tr|Q00P49|Q00P49	Protein O-fucosyltransferase 1	0.00%	4.10%	0.00	0.50	HIGH	New	44
tr|A5GFQ9|A5GFQ9	Protein phosphatase 2, regulatory subunit B (B56), delta isoform	0.00%	2.00%	0.00	0.50	HIGH	New	N/A
tr|F1RI78|F1RI78	Protein tweety homolog	0.00%	4.20%	0.00	21.50	HIGH	New Ɨ	N/A
tr|F1RV50|F1RV50	Protein tweety homolog	0.00%	2.73%	0.00	2.67	HIGH	New Ɨ	N/A

Q19AZ8	Prothrombin	0.00%	2.50%	0.00	6.00	HIGH	New Ɨ	N/A
tr|F1SIB1|F1SIB1	Prothrombin	0.00%	2.50%	0.00	6.00	HIGH	New Ɨ	N/A
Q4LE85	Ras-related protein Rab-27A	0.00%	5.90%	0.00	1.00	HIGH	New	45
Q06AU5	Ras-related protein Rab-32	0.00%	9.30%	0.00	1.50	HIGH	New Ɨ	45
tr|F1S747|F1S747	Ras-related protein Rab-32	0.00%	9.30%	0.00	1.50	HIGH	New Ɨ	45
Q06AU4	Ras-related protein Rab-34	0.00%	3.85%	0.00	2.50	HIGH	New	N/A
Q06AU2	Ras-related protein Rap-2a	0.00%	7.10%	0.00	16.00	HIGH	New Ɨ	N/A
tr|F2Z519|F2Z519	Ras-related protein Rap-2a	0.00%	19.50%	0.00	11.50	HIGH	New Ɨ	N/A
tr|F1S8A4|F1S8A4	Receptor-type tyrosine-protein phosphatase-A	0.00%	0.93%	0.00	3.00	HIGH	New	46
tr|F1SDK4|F1SDK4	Receptor-type tyrosine-protein phosphatase-E	0.00%	1.53%	0.00	2.67	HIGH	Known	46
tr|3LGE9|I3LGE9	Receptor-type tyrosine-protein phosphatase-S (PTPRS)	0.00%	3.30%	0.00	6.00	HIGH	Known	46
tr|Q06AT8|Q06AT8	RHOB	0.00%	12.75%	0.00	11.50	HIGH	New	47
tr|Q06AT7|Q06AT7	RHOF	0.00%	15.60%	0.00	1.00	HIGH	New	48
A2BD05	Serine/threonine-protein kinase Nek6	0.00%	3.85%	0.00	2.00	HIGH	New	N/A
tr|F1RRB6|F1RRB6	Serine/threonine-protein kinase OSR1	0.00%	13.90%	0.00	1.00	HIGH	Known	49
tr|I3LCY8|I3LCY8	Serine/threonine-protein phosphatase 2A 55 kDa regulatory subunit B	0.00%	3.20%	0.00	0.50	HIGH	New	N/A
Q6PQD5	Serine-protein kinase ATM	0.25%	0.33%	0.50	1.00	2.00	New Ɨ	N/A

tr|I3L5F1|I3L5F1	Serine-protein kinase ATM	0.35%	0.40%	0.50	1.00	2.00	New Ɨ	N/A
tr|B3CL06|B3CL06	Serotransferrin	0.00%	2.50%	0.00	0.50	HIGH	New Ɨ	N/A
P09571	Serotransferrin	0.00%	2.60%	0.00	0.50	HIGH	New Ɨ	N/A
P15982	SLA class II histocompatibility antigen, DQ haplotype C beta chain	0.00%	8.80%	0.00	1.00	HIGH	New	N/A
P15983	SLA class II histocompatibility antigen, DQ haplotype D beta chain	0.00%	8.90%	0.00	1.00	HIGH	New	N/A
A1XQR9	Small nuclear ribonucleoprotein E	0.00%	17.40%	0.00	1.50	HIGH	New Ɨ	N/A
tr|I3LDW9|I3LDW9	Small nuclear ribonucleoprotein E	0.00%	17.40%	0.00	1.50	HIGH	New Ɨ	N/A
P26429	Sodium/glucose cotransporter 1	0.00%	3.95%	0.00	11.50	HIGH	New Ɨ	N/A
tr|F1RLV1|F1RLV1	Sodium/glucose cotransporter 1	0.00%	3.65%	0.00	11.50	HIGH	New Ɨ	N/A
tr|F1SB19|F1SB19	Sodium/glucose cotransporter 5	0.00%	4.60%	0.00	1.50	HIGH	New	N/A
tr|F1STH4|F1STH4	Sodium/hydrogen exchanger	0.00%	0.70%	0.00	0.33	HIGH	New	N/A
tr|F1RVX0|F1RVX0	Solute carrier family 16, member 5 (Monocarboxylic acid transporter 6)	0.00%	1.70%	0.00	1.50	HIGH	New	N/A
P20303	Solute carrier family 2, facilitated glucose transporter member 1	0.00%	2.65%	0.00	7.00	HIGH	New Ɨ	N/A
tr|I3LGB8|I3LGB8	Solute carrier family 2, facilitated glucose transporter member 1	0.00%	5.80%	0.00	1.00	HIGH	New Ɨ	N/A
tr|I3LNT8|I3LNT8	Solute carrier family 2, facilitated glucose transporter member 2	0.00%	2.40%	0.00	9.00	HIGH	New	N/A
Q863T6	Solute carrier family 22 member 1	0.00%	2.20%	0.00	1.50	HIGH	New Ɨ	N/A
tr|F1SB78|F1SB78	Solute carrier family 22 member 1	0.00%	2.20%	0.00	1.50	HIGH	New Ɨ	N/A

tr|B6V6V4|B6V6V4	Solute carrier family 30 member 7	0.00%	8.20%	0.00	1.50	HIGH	New	N/A
tr|F1SAI9|F1SAI9	Solute carrier family 37 (Glucose-6-phosphate transporter), member 4	0.00%	3.60%	0.00	3.00	HIGH	New	N/A
Q863Y7	Solute carrier family 52, riboflavin transporter, member 2	0.00%	13.70%	0.00	9.50	HIGH	New Ɨ	N/A
tr|F1RSN6|F1RSN6	Solute carrier family 52, riboflavin transporter, member 2	0.00%	13.70%	0.00	9.50	HIGH	New Ɨ	N/A
tr|K7GT57|K7GT57	Spliceosome RNA helicase DDX39B	0.00%	3.85%	0.00	15.50	HIGH	New	N/A
tr|F1CNZ4|F1CNZ4	STEAP family member 4	0.00%	2.80%	0.00	1.00	HIGH	New	N/A
tr|B2DCZ7|B2DCZ7	Stromal membrane-associated protein 1-like	0.00%	3.00%	0.00	1.50	HIGH	New	N/A
tr|F1SH47|F1SH47	Sucrase-isomaltase, intestinal	0.00%	3.10%	0.00	2.50	HIGH	New	N/A
tr|I3LUD1|I3LUD1	Superoxide dismutase [Cu-Zn]	0.00%	14.80%	0.00	2.50	HIGH	New	50
tr|F1STL1|F1STL1	Syndecan	0.00%	2.60%	0.00	4.00	HIGH	New Ɨ,*	51
tr|F1SCU4|F1SCU4	Syndecan (Fragment)	0.00%	2.25%	0.00	15.50	HIGH	New Ɨ	N/A
tr|A5GFS9|A5GFS9	Syntaxin 16	0.00%	5.90%	0.00	1.50	HIGH	New Ɨ	N/A
tr|A5GFT0|A5GFT0	Syntaxin 16	0.00%	5.80%	0.00	1.50	HIGH	New Ɨ	N/A
tr|A5GFT1|A5GFT1	Syntaxin 16	0.00%	5.50%	0.00	1.50	HIGH	New Ɨ	N/A
tr|A5GFT2|A5GFT2	Syntaxin 16	0.00%	5.60%	0.00	1.50	HIGH	New Ɨ	N/A
tr|F1SMI5|F1SMI5	Tenascin	0.00%	0.60%	0.00	0.33	HIGH	Known Ɨ	52
tr|I3LDR6|I3LDR6	Tenascin	0.00%	0.67%	0.00	0.33	HIGH	Known Ɨ	52

tr|I3LJU9|I3LJU9	Tenascin SV=1	0.00%	0.70%	0.00	0.33	HIGH	New	N/A
Q06AA5	Tetraspanin-9	0.00%	13.80%	0.00	5.50	HIGH	New#	53
tr|B6EUU6|B6EUU6	Toll-like receptor 3 short-type	0.00%	4.25%	0.00	9.00	HIGH	Known	54
tr|D7RK08|D7RK08	Transferrin receptor protein	0.00%	2.70%	0.00	2.00	HIGH	New	N/A
Q8HZV3	Transferrin receptor protein 1 (TfR1)	0.00%	2.70%	0.00	2.00	HIGH	New	N/A
tr|F1S025|F1S025	Transporter	0.00%	1.40%	0.00	3.50	HIGH	New	N/A
P50390	Transthyretin	0.00%	15.30%	0.00	3.00	HIGH	New	55
tr|I3LBJ3|I3LBJ3	tRNA (guanine-N(7)-)-methyltransferase non-catalytic subunit WDR4	0.00%	4.30%	0.00	0.50	HIGH	New	N/A
tr|F2Z562|F2Z562	Tubulin gamma chain	0.00%	22.20%	0.00	3.50	HIGH	Known	56
Q8SQ34	Tumor necrosis factor receptor superfamily member 5	0.00%	4.85%	0.00	33.50	HIGH	New Ɨ	N/A
tr|K7GKQ2|K7GKQ2	Tumor necrosis factor receptor superfamily member 5	0.00%	5.15%	0.00	33.50	HIGH	New Ɨ	N/A
tr|K7GMT2|K7GMT2	Tumor necrosis factor receptor superfamily member 5	0.00%	16.45%	0.00	31.00	HIGH	New Ɨ	N/A
tr|K7GQ17|K7GQ17	Tumor necrosis factor receptor superfamily member 5 (Fragment)	0.00%	46.90%	0.00	0.50	HIGH	New Ɨ	N/A
O77736	Tumor necrosis factor receptor superfamily member 6	0.00%	4.20%	0.00	1.50	HIGH	New Ɨ	N/A
tr|F1SCY7|F1SCY7	Tumor necrosis factor receptor superfamily member 6	0.00%	4.20%	0.00	1.50	HIGH	New Ɨ	N/A
tr|J9JIK9|J9JIK9	Tyrosine-protein kinase	0.00%	0.50%	0.00	3.50	HIGH	New Ɨ	N/A
tr|K7GMY7|K7GMY7	Tyrosine-protein kinase	0.00%	5.80%	0.00	3.50	HIGH	New Ɨ	N/A

O19064	Tyrosine-protein kinase JAK2	0.00%	0.90%	0.00	6.00	HIGH	New Ɨ	N/A
tr|K7GML7|K7GML7	Tyrosine-protein kinase JAK2	0.00%	6.00%	0.00	3.00	HIGH	New Ɨ	N/A
tr|K7GRY2|K7GRY2	Tyrosine-protein kinase JAK2 (Fragment)	0.00%	16.20%	0.00	1.50	HIGH	New Ɨ	N/A
tr|F1RHJ5|F1RHJ5	Tyrosine-protein kinase receptor	0.00%	0.75%	0.00	2.50	HIGH	New	N/A
Reverse_tr|I3LGC6|I3LGC6	Ubiquitin carboxyl-terminal hydrolase (Fragment)	0.00%	0.20%	0.00	0.33	HIGH	New	N/A
Q06AA9	Ubiquitin-conjugating enzyme E2 D2	0.00%	8.15%	0.00	5.00	HIGH	New Ɨ	N/A
tr|F1S105|F1S105	Ubiquitin-conjugating enzyme E2 D2	0.00%	8.15%	0.00	5.00	HIGH	New Ɨ	N/A
tr|I3LUK9|I3LUK9	Ubiquitin-conjugating enzyme E2 D2	0.00%	10.45%	0.00	5.00	HIGH	New Ɨ	N/A
P32307	Vasopressin V2 receptor	0.00%	9.20%	0.00	3.00	HIGH	Known	57
Q29261	Villin-1	0.00%	1.20%	0.00	1.00	HIGH	New Ɨ	N/A
tr|F1SRY3|F1SRY3	Villin-1	0.00%	1.20%	0.00	1.00	HIGH	New Ɨ	N/A
P48819	Vitronectin	0.00%	1.70%	0.00	5.00	HIGH	New Ɨ	N/A
tr|I3L638|I3L638	Vitronectin	0.00%	2.10%	0.00	5.00	HIGH	New Ɨ	N/A
tr|I3L998|I3L998	Vitronectin	0.00%	1.70%	0.00	5.00	HIGH	New Ɨ	N/A
tr|I3L9F8|I3L9F8	Vitronectin	0.00%	1.70%	0.00	5.00	HIGH	New Ɨ	N/A
tr|I3LP50|I3LP50	Vitronectin	0.00%	2.10%	0.00	5.00	HIGH	New Ɨ	N/A
tr|F1SMN6|F1SMN6	V-type proton ATPase subunit F	0.00%	7.15%	0.00	4.50	HIGH	New	N/A

Q9TVC1	V-type proton ATPase subunit H	0.00%	2.40%	0.00	7.00	HIGH	New Ɨ	N/A
tr|F1RSG6|F1RSG6	V-type proton ATPase subunit H	0.00%	2.40%	0.00	7.00	HIGH	New Ɨ	N/A
tr|I3LQB3|I3LQB3	V-type proton ATPase subunit H	0.00%	2.45%	0.00	7.00	HIGH	New Ɨ	N/A

Note:

Note:

Ɨ, same proteins with different accession numbers in the Uniport database.
*, protein known as an exosomal biomarker.
#, known family protein as an exosomal biomarker.
N/A, not available at the time of data analysis.
Total proteins on the table are 324 (= 211 proteins + 113 repeated proteins with different accession numbers). Out of the 211 proteins, there are 172 newly identified proteins and 39 previously confirmed in primary cilia. Out of the 172 newly identified proteins, there are 8 proteins known as exosomal biomarkers and 3 belong to a known exosomal biomarker family.

Reference

1 Ishikawa, H., Thompson, J., Yates, J. R., 3rd & Marshall, W. F. Proteomic analysis of mammalian primary cilia. *Curr Biol* **22**, 414-419, doi:10.1016/j.cub.2012.01.031 (2012).

2 Martin-Alonso, J. M., Hernando, N., Ghosh, S. & Coca-Prados, M. Molecular cloning of the bovine CD9 antigen from ocular ciliary epithelial cells. *J Biochem* **112**, 63-67 (1992).

3 https://proteomescout.wustl.edu/proteins/145284/expression.

4 Kohli, P. *et al.* The ciliary membrane-associated proteome reveals actin-binding proteins as key components of cilia. *EMBO Rep* **18**, 1521-1535, doi:10.15252/embr.201643846 (2017).

5 Klumpp, S. & Schultz, J. E. Alkaline phosphatase from Paramecium cilia and cell bodies: purification and characterization. *Biochim Biophys Acta* **1037**, 233-239 (1990).

6 Moon, P. G. *et al.* Proteomic analysis of urinary exosomes from patients of early IgA nephropathy and thin basement membrane nephropathy. *Proteomics* **11**, 2459-2475, doi:10.1002/pmic.201000443 (2011).

7 Forschbach, V. *et al.* Anoctamin 6 is localized in the primary cilium of renal tubular cells and is involved in apoptosis-dependent cyst lumen formation. *Cell Death Dis* **6**, e1899, doi:10.1038/cddis.2015.273 (2015).

8 Henkel, B. *et al.* Co-expression of anoctamins in cilia of olfactory sensory neurons. *Chem Senses* **40**, 73-87, doi:10.1093/chemse/bju061 (2015).

9 Hu, J. & Barr, M. M. ATP-2 interacts with the PLAT domain of LOV-1 and is involved in Caenorhabditis elegans polycystin signaling. *Mol Biol Cell* **16**, 458-469, doi:10.1091/mbc.E04-09-0851 (2005).

10 Yano, J. *et al.* Proteomic analysis of the cilia membrane of Paramecium tetraurelia. *J Proteomics* **78**, 113-122, doi:10.1016/j.jprot.2012.09.040 (2013).

11 Xie, Y. F. *et al.* Pulsed electromagnetic fields stimulate osteogenic differentiation and maturation of osteoblasts by upregulating the expression of BMPRII localized at the base of primary cilium. *Bone* **93**, 22-32, doi:10.1016/j.bone.2016.09.008 (2016).

12 Wang, S., Wei, Q., Dong, G. & Dong, Z. ERK-mediated suppression of cilia in cisplatin-induced tubular cell apoptosis and acute kidney injury. *Biochim Biophys Acta* **1832**, 1582-1590, doi:10.1016/j.bbadis.2013.05.023 (2013).

13 Trinh, L. *et al.* Th1- and Th2-related chemokine and chemokine receptor expression on the ocular surface in endotoxin-induced uveitis. *Mol Vis* **14**, 2428-2434 (2008).

14 Sandfeld-Paulsen, B. *et al.* Exosomal proteins as prognostic biomarkers in non-small cell lung cancer. *Mol Oncol* **10**, 1595-1602, doi:10.1016/j.molonc.2016.10.003 (2016).

15 Zuo, X., Fogelgren, B. & Lipschutz, J. H. The small GTPase Cdc42 is necessary for primary ciliogenesis in renal tubular epithelial cells. *J Biol Chem* **286**, 22469-22477, doi:10.1074/jbc.M111.238469 (2011).

16 Nishiyama, K., Sakaguchi, H., Hu, J. G., Bok, D. & Hollyfield, J. G. Claudin localization in cilia of the retinal pigment epithelium. *Anat Rec* **267**, 196-203, doi:10.1002/ar.10102 (2002).

17 Li, J. *et al.* Claudin-containing exosomes in the peripheral circulation of women with ovarian cancer. *BMC Cancer* **9**, 244, doi:10.1186/1471-2407-9-244 (2009).

18 Liu, Q. *et al.* The proteome of the mouse photoreceptor sensory cilium complex. *Mol Cell Proteomics* **6**, 1299-1317, doi:10.1074/mcp.M700054-MCP200 (2007).
19 Ikeda, H., Ueda, M., Kobayashi, H. & Honda, Y. Localization and activity of membrane dipeptidase in bovine ciliary epithelium. *Invest Ophthalmol Vis Sci* **44**, 37-43 (2003).
20 Keller, S. *et al.* Systemic presence and tumor-growth promoting effect of ovarian carcinoma released exosomes. *Cancer Lett* **278**, 73-81, doi:10.1016/j.canlet.2008.12.028 (2009).
21 Goggolidou, P. *et al.* ATMIN is a transcriptional regulator of both lung morphogenesis and ciliogenesis. *Development* **141**, 3966-3977, doi:10.1242/dev.107755 (2014).
22 Perrone-Capano, C. *et al.* Dystrophin localization and gene expression in the developing nervous system of the chick. *J Neurosci Res* **51**, 109-118, doi:10.1002/(SICI)1097-4547(19980101)51:1<109::AID-JNR12>3.0.CO;2-6 (1998).
23 Wang, J., Ma, H. Y., Krishnamoorthy, R. R., Yorio, T. & He, S. A feed-forward regulation of endothelin receptors by c-Jun in human non-pigmented ciliary epithelial cells and retinal ganglion cells. *PLoS One* **12**, e0185390, doi:10.1371/journal.pone.0185390 (2017).
24 Jerman, S. *et al.* OFD1 and flotillins are integral components of a ciliary signaling protein complex organized by polycystins in renal epithelia and odontoblasts. *PLoS One* **9**, e106330, doi:10.1371/journal.pone.0106330 (2014).
25 Otto, G. P. & Nichols, B. J. The roles of flotillin microdomains--endocytosis and beyond. *J Cell Sci* **124**, 3933-3940, doi:10.1242/jcs.092015 (2011).
26 Rondanino, C. *et al.* Galectin-7 modulates the length of the primary cilia and wound repair in polarized kidney epithelial cells. *Am J Physiol Renal Physiol* **301**, F622-633, doi:10.1152/ajprenal.00134.2011 (2011).
27 Shichi, H., Mahalak, S. M., Sakamoto, S., Lin, W. L. & Essner, E. S. Immunocytochemical localization of gamma-glutamyl transpeptidase in porcine ciliary epithelium. *Exp Eye Res* **53**, 39-46 (1991).
28 Melo, S. A. *et al.* Glypican-1 identifies cancer exosomes and detects early pancreatic cancer. *Nature* **523**, 177-182, doi:10.1038/nature14581 (2015).
29 Schou, K. B., Pedersen, L. B. & Christensen, S. T. Ins and outs of GPCR signaling in primary cilia. *EMBO Rep* **16**, 1099-1113, doi:10.15252/embr.201540530 (2015).
30 Sakamoto, O. *et al.* Role of macrophage-stimulating protein and its receptor, RON tyrosine kinase, in ciliary motility. *J Clin Invest* **99**, 701-709, doi:10.1172/JCI119214 (1997).
31 Pazour, G. J., Agrin, N., Leszyk, J. & Witman, G. B. Proteomic analysis of a eukaryotic cilium. *J Cell Biol* **170**, 103-113, doi:10.1083/jcb.200504008 (2005).
32 Feldman, C., Cockeran, R., Jedrzejas, M. J., Mitchell, T. J. & Anderson, R. Hyaluronidase augments pneumolysin-mediated injury to human ciliated epithelium. *Int J Infect Dis* **11**, 11-15, doi:10.1016/j.ijid.2005.09.002 (2007).
33 Bailey, C. C., Zhong, G., Huang, I. C. & Farzan, M. IFITM-Family Proteins: The Cell's First Line of Antiviral Defense. *Annu Rev Virol* **1**, 261-283, doi:10.1146/annurev-virology-031413-085537 (2014).
34 Burren, C. P., Berka, J. L. & Batch, J. A. Localization studies of IGFBP-2 and IGFBP-5 in the anterior compartment of the eye. *Curr Eye Res* **16**, 256-262 (1997).
35 Praetorius, H. A., Praetorius, J., Nielsen, S., Frokiaer, J. & Spring, K. R. Beta1-integrins in the primary cilium of MDCK cells potentiate fibronectin-induced Ca2+ signaling. *Am J Physiol Renal Physiol* **287**, F969-978, doi:10.1152/ajprenal.00096.2004 (2004).
36 https://proteomescout.wustl.edu/proteins/118118/expression.
37 Goel, R. *et al.* Characterizing the normal proteome of human ciliary body. *Clin Proteomics* **10**, 9, doi:10.1186/1559-0275-10-9 (2013).

38 Casaroli-Marano, R. P. *et al.* Lipoprotein lipase in highly vascularized structures of the eye. *J Lipid Res* **37**, 1037-1044 (1996).

39 Nakayama, M. Antigen Presentation by MHC-Dressed Cells. *Front Immunol* **5**, 672, doi:10.3389/fimmu.2014.00672 (2014).

40 Helbig, H. *et al.* Induction of MHC class II antigen in cultured bovine ciliary epithelial cells. *Graefes Arch Clin Exp Ophthalmol* **228**, 556-561 (1990).

41 Assis, L. H. *et al.* The molecular motor Myosin Va interacts with the cilia-centrosomal protein RPGRIP1L. *Sci Rep* **7**, 43692, doi:10.1038/srep43692 (2017).

42 Jackson, C. L. *et al.* Neuronal NOS localises to human airway cilia. *Nitric Oxide* **44**, 3-7, doi:10.1016/j.niox.2014.11.003 (2015).

43 Wyatt, T. A. *et al.* Co-exposure to cigarette smoke and alcohol decreases airway epithelial cell cilia beating in a protein kinase Cepsilon-dependent manner. *Am J Pathol* **181**, 431-440, doi:10.1016/j.ajpath.2012.04.022 (2012).

44 Tsao, P. N. *et al.* Notch signaling controls the balance of ciliated and secretory cell fates in developing airways. *Development* **136**, 2297-2307, doi:10.1242/dev.034884 (2009).

45 Bolasco, G. *et al.* Loss of Rab27 function results in abnormal lung epithelium structure in mice. *Am J Physiol Cell Physiol* **300**, C466-476, doi:10.1152/ajpcell.00446.2010 (2011).

46 Boucher, C. A. *et al.* Receptor protein tyrosine phosphatases are novel components of a polycystin complex. *Biochim Biophys Acta* **1812**, 1225-1238, doi:10.1016/j.bbadis.2010.11.006 (2011).

47 Pan, J., You, Y., Huang, T. & Brody, S. L. RhoA-mediated apical actin enrichment is required for ciliogenesis and promoted by Foxj1. *J Cell Sci* **120**, 1868-1876, doi:10.1242/jcs.005306 (2007).

48 Del Debbio, C. B., Santos, M. F., Yan, C. Y., Ahmad, I. & Hamassaki, D. E. Rho GTPases control ciliary epithelium cells proliferation and progenitor profile induction in vivo. *Invest Ophthalmol Vis Sci* **55**, 2631-2641, doi:10.1167/iovs.13-13162 (2014).

49 Edelbusch, C. *et al.* Mutation of serine/threonine protein kinase 36 (STK36) causes primary ciliary dyskinesia with a central pair defect. *Hum Mutat* **38**, 964-969, doi:10.1002/humu.23261 (2017).

50 Sakai, M. *et al.* Immunocytochemical localization of copper-zinc superoxide dismutase in mouse olfactory mucosa. *Auris Nasus Larynx* **20**, 113-116 (1993).

51 Baietti, M. F. *et al.* Syndecan-syntenin-ALIX regulates the biogenesis of exosomes. *Nat Cell Biol* **14**, 677-685, doi:10.1038/ncb2502 (2012).

52 Dias da Silva, M. R., Tiffin, N., Mima, T., Mikawa, T. & Hyer, J. FGF-mediated induction of ciliary body tissue in the chick eye. *Dev Biol* **304**, 272-285, doi:10.1016/j.ydbio.2006.12.033 (2007).

53 Nazarenko, I. *et al.* Cell surface tetraspanin Tspan8 contributes to molecular pathways of exosome-induced endothelial cell activation. *Cancer Res* **70**, 1668-1678, doi:10.1158/0008-5472.CAN-09-2470 (2010).

54 Brito, B. E. *et al.* Toll-like receptor 4 and CD14 expression in human ciliary body and TLR-4 in human iris endothelial cells. *Exp Eye Res* **79**, 203-208, doi:10.1016/j.exer.2004.03.012 (2004).

55 Dwork, A. J. *et al.* Distribution of transthyretin in the rat eye. *Invest Ophthalmol Vis Sci* **31**, 489-496 (1990).

56 Muresan, V., Joshi, H. C. & Besharse, J. C. Gamma-tubulin in differentiated cell types: localization in the vicinity of basal bodies in retinal photoreceptors and ciliated epithelia. *J Cell Sci* **104 (Pt 4)**, 1229-1237 (1993).

57 Raychowdhury, M. K. *et al.* Vasopressin receptor-mediated functional signaling pathway in primary cilia of renal epithelial cells. *Am J Physiol Renal Physiol* **296**, F87-97, doi:10.1152/ajprenal.90509.2008 (2009).
