# Supplementary material for: Proteomic Identification Reveals the Role of Ciliary Extracellular‐Like Vesicle in Cardiovascular Function
Source: Adv Sci (Weinh). 2020 Jun 16;7(16):1903140. doi: 10.1002/advs.201903140 (PMC7435257; doi:10.1002/advs.201903140)
Supplement: Supplementary file 7 — Supplemental Table 3 [file ADVS-7-1903140-s007.pdf]

Table 3. Working heart parameters

	Scrambled control			*PGRMC2*			*F11*		
	**control**	**adrenalin**	**verapamil**	**control**	**adrenalin**	**verapamil**	**control**	**adrenalin**	**verapamil**
**HR (beat/min)**	140±12	209±19	90±9	143±12	207±19	91±9	140±12	211±20	95±9
**ESPVR (mmHg/µL)**	4.2±0.2	11.9±0.7	1.4±0.1	4.2±0.3	14.1±1.4	1.9±0.1	4.7±0.2	15.4±1.4	1.9±0.1
**EDPVR (mmHg/µL)**	0.12±0.01	0.13±0.01	0.13±0.01	0.17±0.02	0.20±0.03	0.17±0.01	0.20±0.02	0.10±0.03	0.16±0.01
**dP/dtmax (mmHg/s)**	5,667±88	17,056±265	3,048±911	8,288±343	17,500±310	4,347±1223	79,187±131	17,478±258	4,426±1425
**dP/dtmin (mmHg/s)**	-1,574±146	-3,065±797	-1,441±36	-1,886±185	-4,284±216	-1,575±101	-2,178±225	-2,196±179	-1,465±138
**LV Pmax (mmHg)**	52.5±0.8	85.3±1.3	33.9±1.0	76.7±3.2	87.5±1.6	48.3±1.4	73.31±1.21	87.39±1.3	49.2±1.6
**LV ESP (mmHg)**	39.3±0.6	64.0±1.0	25.4±0.8	57.6±2.4	65.6±1.2	36.2±1.0	54.9±0.9	65.5±0.97	36.88±1.19
**LV EDP (mmHg)**	4.9±0.4	5.1±0.3	5.3±0.7	5.8±0.6	7.1±1.1	5.8±0.2	6.7±0.7	3.7±0.9	5.4±0.3
**LV ESV (µL)**	12.6±0.3	7.3±0.5	25.3±1.0	18.6±0.9	6.4±0.7	24.8±0.5	15.8±0.9	5.8±0.5	24.8±0.6
**LV EDV (µL)**	39.5±0.5	40.0±0.1	40.2±0.3	34.9±0.8	35.7±0.5	33.7±0.5	33.2±0.6	34.7±0.6	33.7±0.5
**SV (µL)**	26.9±0.7	32.7±0.5	15.0±1.2	16.4±0.5	29.3±0.8	8.9±0.7	16.9±0.8	28.9±0.9	8.9±0.9
**SW (mmHg•µL)**	1,281±45	2,618±35	427±37	1,167±74	2,348±60	379±28	1,161±103	2,421±109	392±45
**EF (%)**	68.1±1.1	81.8±1.2	37.1±2.8	47.0±1.8	82.0±2.0	26.5±1.8	50.3±3.0	83.2±1.6	26.5±2.4
**CO (µL/min)**	3,736±113	5,874±80	1,346±20	2,349±62	6,062±149	806.1±60	2,428±154	6,096±183	806±80

HR, heart rate; ESPVR and EDPVR, end-systolic and end-diastolic pressure volume relation, respectively; dP/dtmax and dP/dtmin, maximum rate of left ventricle (LV) pressure rise and fall, respectively; Pmax, systolic pressure; ESP, end-systolic pressure; EDP, end-diastolic pressure; ESV, end-systolic volume; EDV, end-diastolic volume; SV, stroke volume; SW, stroke work; EF, ejection fraction; CO, cardiac output.
